# Identification of Quercetin as a Natural MMP1 Inhibitor for Overcoming Cisplatin Resistance in Epithelial Ovarian Cancer

**DOI:** 10.7150/jca.110517

**Published:** 2025-05-31

**Authors:** Shubo Wang, Ziming Zhao, Lili Yu, Jue Wang, Yue Ouyang, Hua Zhou, Weixing Shen, Qibiao Wu

**Affiliations:** 1 Faculty of Chinese Medicine and State Key Laboratory of Quality Research in Chinese Medicine, Macau University of Science and Technology, 999078 Macao, China.; 2 Chinese Medicine Guangdong Laboratory (Hengqin Laboratory), Guangdong-Macao ln-Depth Cooperation Zone in Hengqin, 519000, China; 3 The First Clinical Medical College, Nanjing University of Chinese Medicine, Nanjing, 210023, Jiangsu, China; Collaborative Innovation Center of Traditional Chinese Medicine Prevention and Treatment of Tumor, Nanjing, 210023, Jiangsu, China.

**Keywords:** Cisplatin resistance, Epithelial ovarian cancer, MMP1, Natural products, Molecular docking, *In vitro* validation.

## Abstract

**Background:** It is challenging to find a therapeutic Chinese medicine monomer (CHMs) for platinum-resistant patients, who currently have few treatment options, due to the complex mechanisms and the large number of CHMs available. This study aimed to identify CHM to overcome cisplatin resistance in EOC through bioinformatics analysis with *in vitro* experiment.

**Methods:** This study used a strategy opposite to conventional network pharmacology. RNA expression of cisplatin sensitive and resistant EOC cell lines was obtained from the GEO database. Through differential expression gene (DEG) analysis, PPI network analysis, and survival analysis, we identified hub genes related to platinum resistance. CHMs targeting these hub genes were identified from the HIT 2.0 and molecular docking, molecular dynamics (MD) simulations, and SPR assay were used to validate their binding ability. Then, the anti-cancer effects of CHM in cisplatin-resistant cell lines were verified via *in vitro* experiment.

**Results:** 16 hub genes were selected through DEG and PPI network analysis. Following validation via survival analysis in the TCGA-OV cohort, the investigation ultimately focused on MMP1. Western blotting results demonstrated that MAPK signaling pathway activation induced MMP1 expression in cisplatin-resistant EOC cells. 22 CHMs targeting MMP1 were found in HIT 2.0 and quercetin was demonstrated the strong affinity of quercetin for MMP1 via molecular docking, MD simulations and SPR assay. Quercetin also exhibited strong binding affinity to other hub genes, including EGR1, STAT1, and PRKCA. *In vitro* experiments demonstrated that quercetin effectively inhibited the proliferation, apoptosis resistance, invasion, and migration of cisplatin-resistant EOC cells. The combination of cisplatin and quercetin had a strong synergistic effect, as indicated by the ZIP synergy score (≥10).

**Conclusion:** Our study identified quercetin, which functions by targeting multiple cisplatin resistance-related proteins, including MMP1, as a potential therapeutic CHM for cisplatin resistant EOC. This study also explored quercetin's mechanisms against platinum-resistant ovarian cancer. By identifying hub platinum-resistance associated genes and screening potential CHMs through bioinformatics, computer simulation, and *in vitro* experiments, this integrated analytical approach also offers a reference for discovering CHMs for other diseases.

## Background

Epithelial ovarian cancer (EOC) remains one of the leading causes of cancer-related mortality in women worldwide. The standard treatment for EOC typically involves cytoreductive surgery followed by chemotherapy using a platinum-taxane combination 1. Despite advancements in treatment, such as PARP inhibitors, antibody-drug conjugates, and multitargeted small-molecule tyrosine kinase inhibitors, platinum-based chemotherapy remains the cornerstone of systemic therapy for EOC 2, 3. However, resistance to platinum compounds, which generally develops within five years, severely limits the long-term efficacy of these treatments 4. Patients with platinum-resistant EOC often require alternative therapeutic strategies, yet their prognosis remains poor 4. Addressing platinum resistance is, therefore, a critical priority in the management of EOC. The mechanisms of platinum resistance are multifactorial, involving processes such as DNA repair, metabolic reprogramming, multidrug resistance, altered cell cycle regulation, apoptosis resistance, oxidative stress, cancer stem cell activity, and autophagy dysfunction 4, 5. At the molecular level, platinum resistance is driven by genetic and epigenetic alterations that affect various genes and proteins 5.

Currently, limited effective drugs are available to combat platinum resistance in EOC, and further research is necessary to better understand their efficacy and safety 6, 7. Natural products derived from organisms or their metabolites have long been utilized for their therapeutic properties. Traditional Chinese medicine (TCM), with its rich history of using plant-based natural products, offers a promising source of therapeutic agents for various diseases 8. Modern research has focused on evaluating Chinese herbal monomers (CHMs) extracted from Chinese herbs (CHs) for their pharmacological potential, using pharmacokinetic, pharmacological, and clinical studies 9-14. These active compounds have diverse medicinal properties, which make them valuable candidates for new drug development. CHMs have attracted significant attention in cancer therapy due to their ability to target multiple signaling pathways, offering distinct advantages over conventional small-molecule inhibitors, especially in the context of platinum-resistant EOC, which involves multiple dysregulated pathways 13, 15. Numerous studies have shown that CHMs hold great potential in overcoming platinum resistance in EOC 16-20. However, much of this research is based on evidence from other cancers, and the underlying molecular mechanisms are still not fully understood.

**Figure 1:**
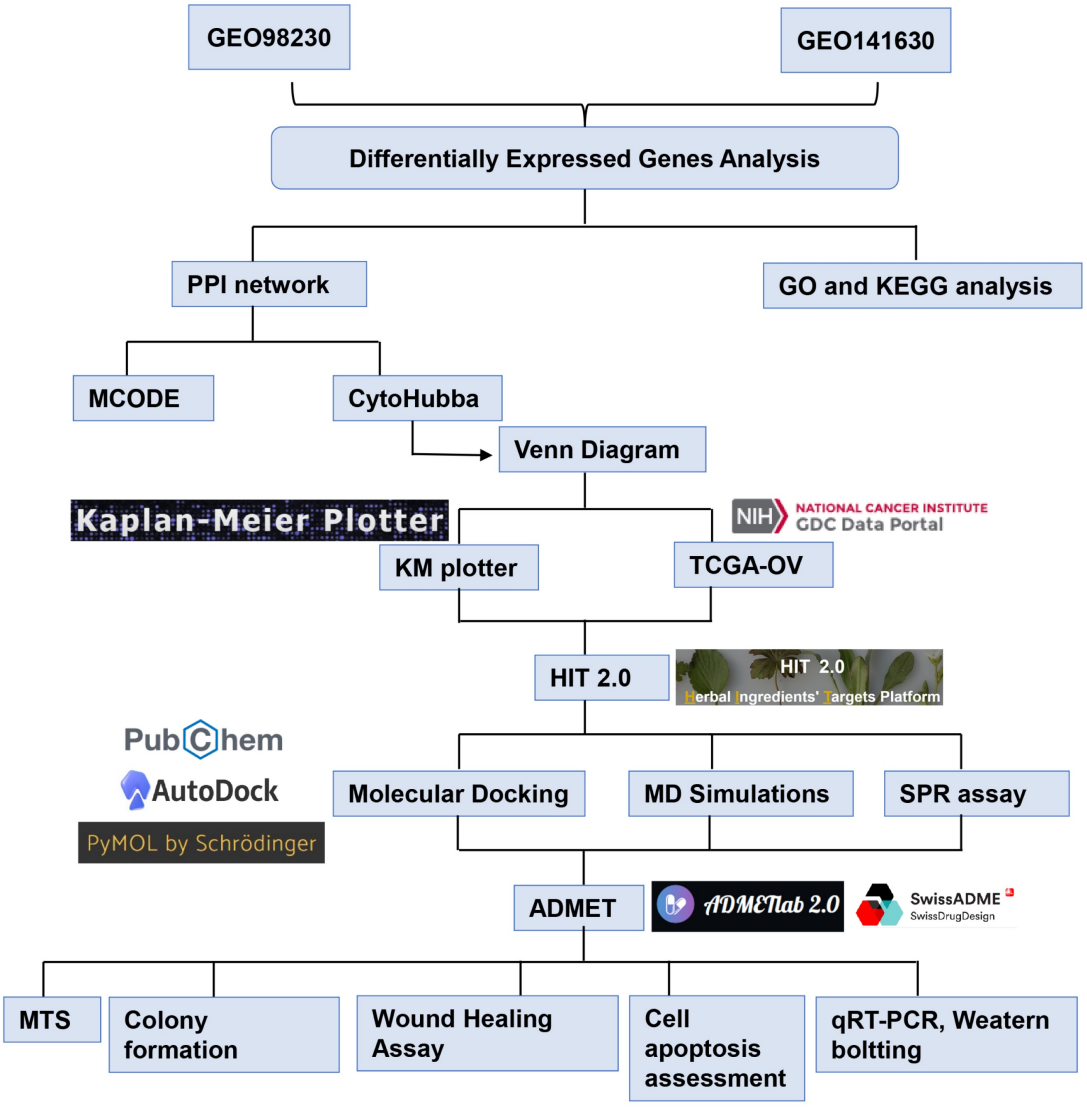
The flowchart of the study.

Recent advances in bioinformatics techniques have revolutionized the identification of potential natural product candidates that target key proteins. Network pharmacology, which integrates protein-protein interaction (PPI) networks and TCM databases, has emerged as a powerful tool for exploring the mechanisms of CHMs, individual herbs, and TCM formulations 21-23. Additionally, molecular docking studies have been employed to predict the interactions between CHMs and their targets, providing valuable insights into their therapeutic potential and side effects 24, 25. This study employed bioinformatics and computer-aided drug design to investigate the molecular networks and hub genes associated with platinum-resistant EOC. Then, we identified a promising CHM with potential therapeutic effects and validated its efficacy through *in vitro* experiments. The integration of bioinformatics and experimental methods provides a novel strategy for identifying effective therapies against platinum-resistant EOC, as outlined in **Figure 1**.

## Materials and methods

### Datasets

RNA-Seq data for A2780/A2780DDP and Ovcar4/Ovcar4DDP were obtained from the GSE98230 and GSE141630 using the GEOquery package in R (version 4.4.1). Data preprocessing, including cleaning, normalization, and annotation, was performed using the dplyr and edgeR R packages. In short, samples with zero expression for all probes were removed. Annotation file from GPL18573 and GPL20301 were used for annotation of GSE98230 and GSE141630. RNA-Seq and clinical data from the TCGA-OC cohort (N=489) were downloaded from cBioPortal (https://www.cbioportal.org/). We extracted patients with newly diagnosed ovarian serous adenocarcinoma from the cohort, who had not received any prior treatment including chemotherapy or radiotherapy, had a serous histological type, were undergoing surgical resection, and were included regardless of their staging or histological grade, and whose platinum-free interval was less than six months, for further analysis (N=90).

### Identification of Differentially Expressed Genes (DEGs) and Functional Enrichment

Differentially expressed genes (DEGs) were identified using the limma package in R, with a log2 (fold change) threshold of > 0.5 and a P value < 0.05. Visualizations of DEGs were generated with volcano plots (ggplot2) and heatmaps (Complex-Heatmap). Venn diagrams were created using the Venn diagram package. Gene Ontology (GO) and Kyoto Encyclopedia of Genes and Genomes (KEGG) pathway analyses were conducted using the OmicShare tool (https://www.omicshare.com/tools/home/report/koenrich.html), considering P values and Q values < 0.05 as significant.

### Establishment of Protein-Protein Interaction (PPI) Network and Target-Compound Network

STRING (https://cn.string-db.org) were used to construct a PPI network. Visualization of the PPI network was carried out using Cytoscape (version 3.9.1). Key modules within the PPI network were identified using the MCODE plugin with a node score threshold of 0.2 and a K-core value of 2. The top 20 hub genes were selected based on four topological analysis methods (MNC, MCC, DEGREE, and EPC) using the cytoHubba plugin in Cytoscape. Finally, the target-compound network was constructed by linking hub genes to potential therapeutic compounds.

### Survival Analysis

Survival analysis of platinum-resistant EOC patients (N=90) from the TCGA-OC cohort was performed using the survival package in R. Forest plot was generated using the forest later package. Kaplan-Meier survival curves were constructed to evaluate the association between gene expression and overall survival in EOC patients using the Kaplan-Meier Plotter website (https://kmplot.com/analysis/index.php?p=background).

### Cell Culture and Cisplatin-Resistant Cell Line Construction

A2780 (Jinjin, Shanghai, China), A2780DDP (Jinjin, Shanghai, China), and Ovcar4 cells (Biofeng, Shanghai, China) were cultured in RPMI 1640 (Sigma‒Aldrich, United States) and DMEM (Sigma‒Aldrich, United States) supplemented with 10% FBS (Thermo Fisher Scientific, MA, United States), respectively. The cisplatin-resistant Ovcar4 cell line (Ovcar4DDP) was established using a stepwise gradient approach with escalating cisplatin concentrations (0.5-3 μM), as described by Amaral et al 26. Ovcar4 cells were exposed to increasing cisplatin concentrations until stable cisplatin resistance was developed (maintained at 1 μM cisplatin for 2 months).

### Western bolting

RIPA Lysis Buffer (Sigma-aldrich, USA) were used to extract soluble proteins from A2780DDP cells or Ovcar4DDP cells which were treated with or without cisplatin (5μM for 72 hours), quercetin (25μM or 50μM for 72 hours), or C16-PAF (5μM for 12 hours) (MCE, USA). The protein concentration was calculated via Micro BCA Protein Assay Kit (Thermo Fisher Scientific, USA). After separating the proteins in SDS-PAGE gel by electrophoresis, proteins were transferred into PVDF membrane. After blocking with blocking buffer (Beyotime, China) for 6 hours, incubate overnight with the primary antibodies. Primary antibodies included MMP1(Beyotime, China; AF0231;1:1000), ERK (Beyotime, China; AF0144, 1:1000), p-ERK (Beyotime, China; AF5818, 1:500), JNK (Beyotime, China; AF1048, 1:1000), p-JNK (Beyotime, China; AF1048, 1:500), p38 (Beyotime, China; AF1111, 1:1000), p-p38 (Beyotime, China; AF7668, 1:500), and β-actin (Beyotime, China; AF2811, 1:2000). Then, after washing by blocking buffer for 30min, PVDF membrane were incubated with secondary antibodies (anti-rabbit, Beyotime, China, A0208, 1:2000; anti-mouse, Beyotime, China, A0216, 1:2000) for 1 hour. Then PVDF membrane were washed for 30min by TBST buffer and detected by ChemiDoc XRS+ Imaging system (Bio-Rad, USA).

### Hub Gene-Related Chinese Herbal Monomer (CHM) Detection and Molecular Docking

The Herbal Ingredients' Targets Platform 2.0 (HIT 2.0) was utilized to identify potential Chinese herbal monomers (CHMs) associated with the hub genes. The 3D structures of proteins (in PDB format) were obtained from the Protein Data Bank (https://www.rcsb.org/) and preprocessed using PyMOL (version 1.7.0). The 3D structures of CHMs were acquired from the PubChem database (https://pubchem.ncbi.nlm.nih.gov/) and converted into the required format using Open Babel (version 2.4.1). Proteins were uploaded, add hydrogen bond, computed gasteiger Charge and then assigned AD4 type in AutoDock Vina (version 1.1.2). Ligands were also uploaded, detected root, showed root expansion and saved as PDBQT format for further analysis. Grid Box were set to cover the entire proteins. Molecular docking was performed with Genetic Algorithm (Number of GA runs=50, other parameters set as default values), and the binding affinity between the ligands (CHMs) and target proteins was calculated in kcal/mol. PyMOL was used to visualize the 3D protein-ligand complexes obtained from molecular docking analysis.

### Molecular Dynamics (MD) Simulations

MD simulations were conducted using GROMACS 2022, with the GAFF force field for small molecules and the AMBER14SB force field for proteins. The simulation system was constructed by merging the ligand and protein files, followed by energy minimization. Periodic boundary conditions were applied, and the LINCS algorithm was used to constrain hydrogen bonds with a 2-fs integration time step. Electrostatic interactions were calculated using the particle-mesh Ewald (PME) method with a threshold of 1.2 nm. Nonbonded interactions had a cutoff of 10 Å, updated every 10 steps. The simulation temperature was maintained at 298 K using the V-rescale coupling method, and pressure was maintained at 1 bar using the Berendsen approach. After a 100 ps NPT and NVT equilibration, a 100 ns MD simulation was carried out. The simulation data were saved every 10 ps for further analysis with PyMOL and VMD. The protein-ligand binding free energy was calculated using the MMPBSA method with the g_mmpbsa tool.

### Surface Plasmon Resonance (SPR) assay

We used the Biacore T200 system (GE Healthcare Life Sciences, Uppsala, Sweden) to quantitatively measure the interaction between quercetin and MMP1. Following the manufacturer's instructions, purified MMP1 protein was immobilized on CM5 chips, and quercetin at various concentrations was used as the analyte for multi-cycle kinetic analysis. All results were fitted to a 1:1 binding model for kinetic and affinity analysis. Biacore T200 Evaluation Software (version 2.0) were used when analyzing the data.

### Prediction of Absorption, Distribution, Metabolism, Excretion, and Toxicity (ADMET) of CHMs

SwissADME (http://www.swissadme.ch) and ADMETLAB2.0 (https://admetmesh.scbdd.com) are robust tools which are capable of access ADMET prediction models such as Bioavailability Radar, BOILED-Egg etc. ADMET properties of quercetin were predicted using the SwissADME and ADMETLAB2.0 using the default parameters provided by each platform. We compiled parameters provided by them (mainly by ADMETLAB2.0).

### Cytotoxicity Assay and Synergy Determination with SynergyFinder

A2780, A2780DDP, Ovcar4, and Ovcar4DDP cells were seeded in 96-well plates at 5 × 10^3^ cells per well and incubated overnight at 37°C with 5% CO_2_. Cisplatin (0-50 μM) or quercetin (0-500 μM) was added for single-drug treatments. For the combination treatment, cisplatin (0-10 μM) and quercetin (0-100 μM) were used in a 6×6 dose matrix, resulting in 36 combinations. After 72 h, cell viability was assessed using the MTT assay. IC50 values were calculated using GraphPad Prism (version 8.0). The synergy between cisplatin and quercetin was evaluated using the Zero Interaction Potency (ZIP) model via SynergyFinder (https://synergyfinder.fimm.fi). A ZIP score > 10 indicated strong synergy.

### Colony Formation Assay

A2780DDP and Ovcar4DDP cells were treated with 50 μM quercetin for 72 h. After treatment, cells were trypsinized, counted, and seeded in Petri dishes (300 cells/dish). After 14 days of incubation, colonies were fixed with paraformaldehyde and stained with crystal violet. Colonies containing > 50 cells were counted.

### Wound Healing Assay

A2780DDP and Ovcar4DDP cells were seeded into 6-well plates at a 5 × 10^5^ cells/well density. A scratch was made in the cell monolayer using a sterile micropipette tip, and cells were treated with 0 or 50 μM quercetin. Images were captured at 0, 24, 48, and 72 h. Cell migration was quantified using ImageJ2 software (2.15.0).

### Transwell assay

A2780DDP and Ovcar4DDP cells were treated with 0 or 50 μM quercetin for 72 h. Cells (1 × 10^4^) were seeded in the upper chamber of a Transwell plate with or without Matrigel in an FBS-free medium, and the bottom chamber contained a complete medium. After 48 h, cells that migrated or invaded through the membrane were stained with crystal violet and counted using ImageJ2 software.

### Cell apoptosis assay

Annexin V-FITC apoptosis detection was performed using the Annexin V-FITC kit (Sigma‒Aldrich, United States). A2780DDP and Ovcar4DDP cells were treated with cisplatin (1.25 μM) or quercetin (50 μM) alone or in combination for 72 h. Apoptosis was analyzed by flow cytometry after staining with Annexin V-FITC and propidium iodide (Sigma‒Aldrich, United States).

### Reverse Transcription-Quantitative Polymerase Chain Reaction (RT-qPCR)

Total RNA was extracted from the cells using Trizol reagent (Thermo Fisher Scientific, MA, USA), followed by cDNA synthesis with the Maxima First Strand cDNA Synthesis Kit (Thermo Fisher Scientific, MA, USA). Quantitative RT-PCR (qRT-PCR) was conducted on a Bio-Rad CFX96 Real-Time Thermocycler (Bio-Rad Laboratories, CA, USA) using the SYBR Premix Ex Taq kit (Takara Bio, Japan) according to the manufacturer's instructions. The relative gene expression was quantified using the 2^-∆∆CT^ method, normalizing to GAPDH as the housekeeping gene. Primer sequences used for qRT-PCR are provided in **Table S1**.

### Statistical analysis

Each experiment was performed in triplicate, and data are presented as means ± standard deviations (SD) unless otherwise indicated. Statistical analyses were performed using GraphPad Prism 5 software (GraphPad Software, CA, USA). Comparisons between two groups were made using a two-tailed Student's t-test, while a one-way analysis of variance (ANOVA) was employed to evaluate differences among multiple groups. Tukey's post hoc test was applied for pairwise comparisons following ANOVA. A P value < 0.05 was considered statistically significant.

## Results

### Cisplatin-resistant (CR) Gene Identification in Ovarian Cancer

**Figure 2:**
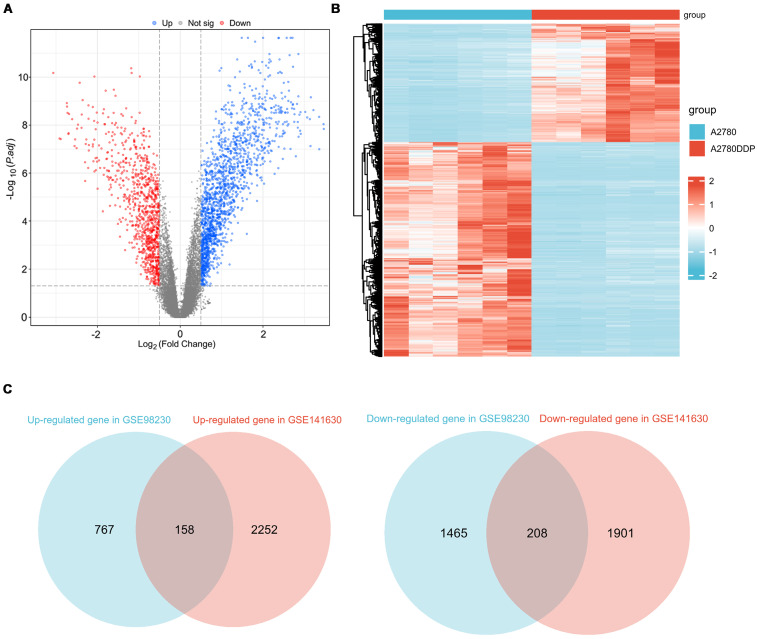
** Identification of CR genes. (A)** The volcano plot of DEGs between A2780 and A2780DDP cells. **(B)** The heatmap of DEGs between A2780 and A2780DDP cells. **(C)** Identification of CR genes based on upregulated or downregulated DEGs.

Analysis of the GSE98230 dataset identified 2598 differentially expressed genes (DEGs) between the A2780 and A2780DDP cell lines (**Figure 2A, Table S2**). The heatmap (**Figure 2B**) demonstrates a distinct separation in RNA expression profiles between these cell lines. Similarly, the GSE141630 dataset, comparing Ovcar4 and Ovcar4DDP cells, revealed 4519 DEGs (**Figure S1, Table 3**). Integrating data from both datasets, 366 cisplatin-resistant (CR) genes were identified, comprising 158 upregulated and 208 downregulated genes (**Figure 2C, Table S4**). These CR genes represent key molecular alterations associated with cisplatin resistance in EOC.

### Gene Ontology (GO) and KEGG Pathway Analyses

**Figure 3:**
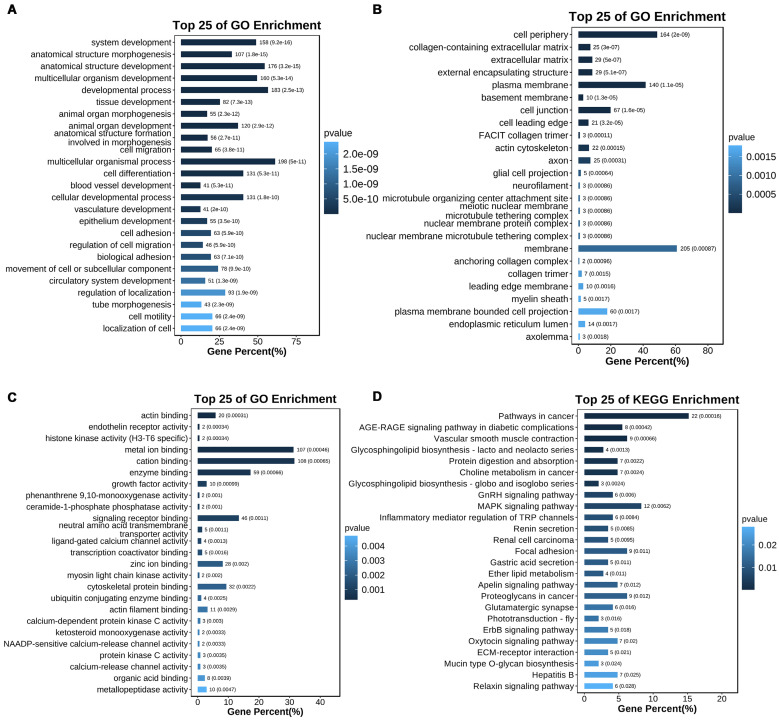
** Enrichment analysis of CR genes. (A-C)** GO enrichment analysis of CR genes (BP, CC, and MF). **(D)** KEGG enrichment analysis of CR genes. Top 25 of terms of GO enrichment and top 25 pathways of KEGG enrichment were shown.

Functional enrichment analyses of the 366 CR genes were conducted using Gene Ontology (GO) and Kyoto Encyclopedia of Genes and Genomes (KEGG) pathway tools (**Table S5, S6**). The top 25 GO terms for Biological Processes (BP), Cellular Components (CC), and Molecular Functions (MF) are presented in **Figures 3A, 3B, and 3C**, respectively. The BP terms identified include key processes such as cell differentiation, developmental processes, and system development (**Figure 3A**). For CC, the most enriched terms were plasma membrane, cell periphery, and cell junction (**Figure 3B**). MF analysis highlighted functional categories like enzyme binding, metal ion binding, and receptor binding (**Figure 3C**). KEGG pathway enrichment analysis (**Figure 3D**) revealed pathways such as cancer signaling, MAPK signaling, and focal adhesion, all known to be involved in platinum resistance mechanisms in EOC.

### Protein-Protein Interaction (PPI) Network Construction

A PPI network was constructed using STRING and visualized in Cytoscape (**Figure S2A**). Module analysis via the MCODE plugin identified a core submodule of 17 genes, including MMP1, IFITM1, TGFB2, TIMP3, APOE, and CSF1, which form a highly interconnected network (**Figure S2B**). This submodule represents critical molecular nodes associated with platinum resistance in EOC.

### Hub Gene Identification and Validation

**Figure 4:**
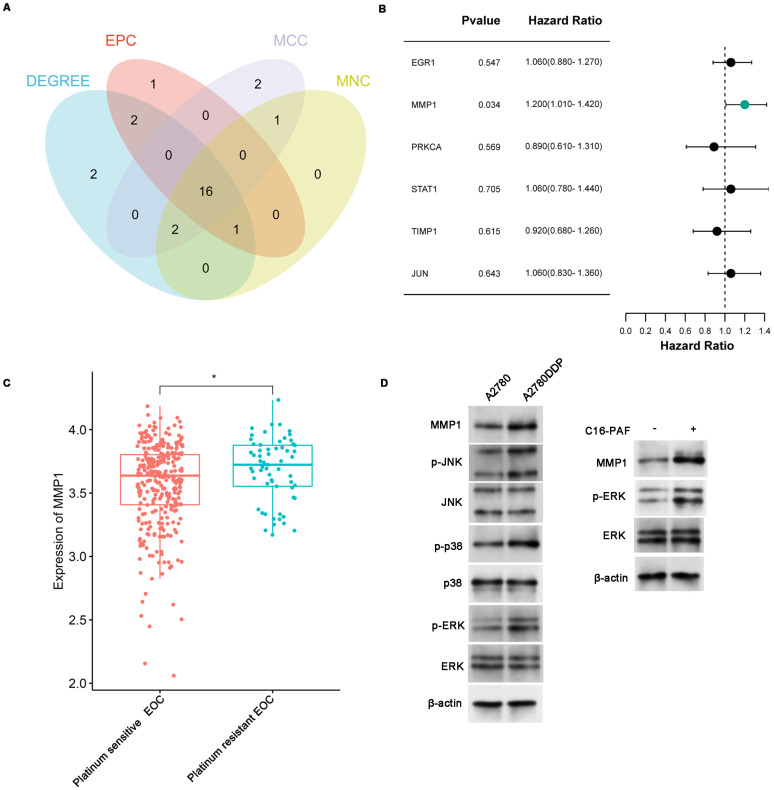
** Identification and validation of hub genes. (A)** Hub genes collection based on four topological analysis methods (MCC, MNC, DEGREE, and EPC). **(B)** Univariate COX regression analysis of genes in platinum-resistant patients from the TCGA-OC cohort (N=90). **(C)** The MMP1 expression between Platinum-sensitive or -resistant EOC. **(D)** Western blotting revealed the mechanism of MMP1 overexpression. The expression of MMP1, p-ERK, ERK, p-JNK, JNK, p-p38, and p38 in A2780 and A2780DDP cells tested by western blotting (left). The expression of MMP1, ERK, and p-ERK in A2780 cells after treated with 5μM C16-PAF for 12 hours, a MAPK agonist that primarily activates the MEK/ERK signaling pathway (right). *p<0.05.

Topological analysis of the PPI network was conducted using four algorithms from the CytoHubba plugin (MCC, MNC, DEGREE, and EPC) to identify the most influential hub genes. The intersection of these methods yielded 16 hub genes, including MMP1, STAT1, EGR1, and PRKCA (**Figure 4A, Table S7**). Considering that most CHMs and anticancer drugs function by binding to or inhibiting protein functions, genes with high expression in cisplatin-resistant cells, including MMP1, STAT1, TIMP1, EGR1, JUN, and PRKCA, were selected for further validation via the clinical data of EOC patients. Kaplan-Meier survival analysis of the OC cohort (N=1657) showed that high expression of MMP1 and EGR1 was associated with poor overall survival (OS) (MMP1: HR=1.15, 95% CI: 1.01-1.31, p=0.04; EGR1: HR=1.15, 95% CI: 1.01-1.31, p=0.037) (**Figure S3**). We included 90 platinum-resistant EOC patients from the TCGA-OC cohort for further analysis, and their demographic characteristics can be found in **Table S8 and S9**. Univariate and multivariate Cox regression analyses confirmed that MMP1 overexpression was significantly associated with poorer OS (HR=1.20, 95% CI: 1.01-1.42, p=0.034; adjusted HR=1.2015, 95% CI=1.0067-1.434, p=0.042) (**Figure 4B, Table 1**). Correlation analysis revealed that MMP1 was strongly associated with other hub genes, including ACTA2, APOE, and TIMP3, further confirming its central role in the CR gene network (**Figure S4**). Notably, MMP1 expression was significantly elevated in platinum-resistant patients (**Figure 4C**), solidifying its role as a key therapeutic target for overcoming cisplatin resistance.

### The Mechanism of MMP1 Overexpression in Cisplatin-Resistant EOC

As indicated by KEGG enrichment analysis presented above, CR genes were enriched in MAPK signaling pathway. Subsequently, we investigated whether the activation of thfe MAPK signaling pathway resulted in the overexpression of MMP1. The western blotting revealed that the phosphorylation level of JNK, p38, and ERK was higher in A2780DDP cells compared to A2780 cells (**Figure 4D**). Furthermore, treating A2780 cells with C16-PAF (a MAPK agonist) resulted in higher expression of MMP1 (**Figure 4D**). These findings demonstrated that the activation of MAPK signaling pathway was associated with the overexpression of MMP1 in Platinum-Resistant EOC.

### Screening of Potential Chinese Herbal Monomers (CHMs)

**Figure 5:**
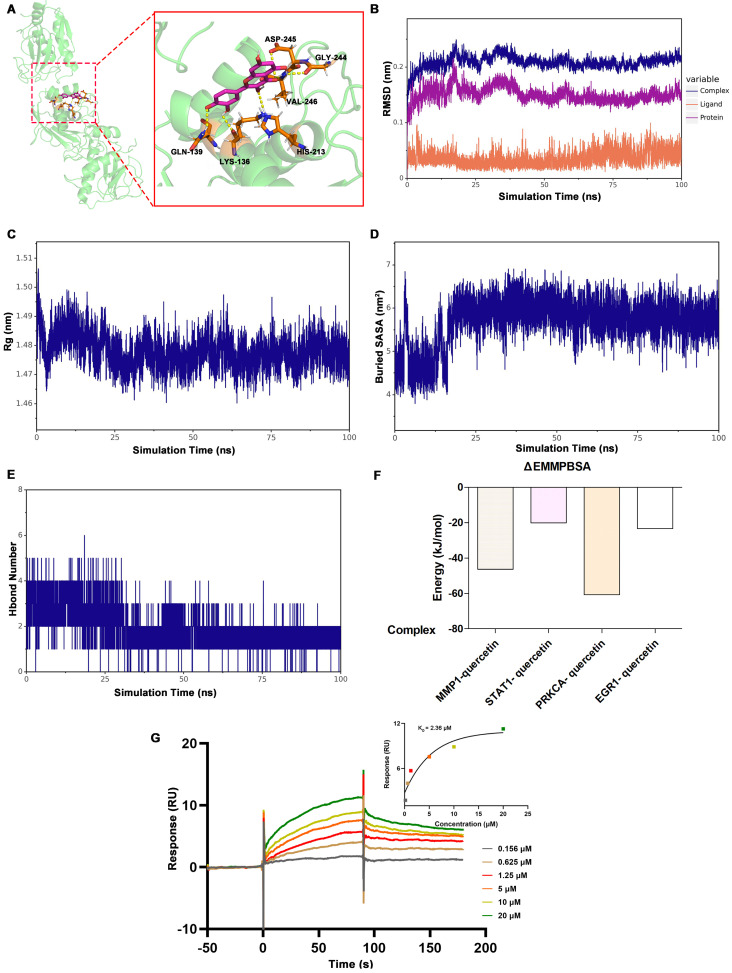
** The binding between quercetin and MMP1. (A)** Molecular docking conformation of quercetin interacting with MMP1. **(B-E)** showed the MD Simulations of MMP1-quercetin complexes. **(B)** RMSD values of the complexes of MMP1-quercetin. **(C)** Rg values of the complex. **(D)** SASA values of the complex. (E) illustrated the variation in the number of hydrogen bonds within the comple during the molecular simulation. **(F)** The result of ΔEMMPBSA using the MM/GBSA method. **(G)** The SPR assay results of the quercetin and MMP1.

22 CHMs with potential interactions with MMP1 were identified via the HIT 2.0 platform (**Table S10**). Molecular docking analysis identified quercetin as a top candidate, showing the strongest affinity for MMP1 with a binding energy of -5.46 kJ/mol (**Table 2**). The docking modes between quercetin and MMP1 were shown in **Figure 5A**, illustrating stable interactions at key residues. Additionally, target-compound network analysis indicated that quercetin interacts with other hub genes such as STAT1, PRKCA, EGR1, JUN, and APOE (**Figure S5A**). Binding affinities of quercetin for STAT1, EGR1, and PARCK were strong (binding energies < -5 kJ/mol), while interactions with APOE and JUN were weaker (binding energies > -5 kJ/mol) (**Table 3**). The docking modes between quercetin and these proteins were shown in **Figure S5B, S5C, S5D, S5E, S5F**. These findings suggest that quercetin may modulate multiple targets involved in cisplatin resistance, making it a promising therapeutic agent.

### Molecular Dynamics (MD) Simulations of Quercetin and Protein Complexes

To validate the stability of the quercetin-protein complexes, MD simulations were performed. The RMSD of the MMP1-quercetin, EGR1-quercetin, STAT1-quercetin, and PRKCA-quercetin complexes gradually stabilized as the simulation progresses, without undergoing significant fluctuations (fluctuations within 1 nm), indicating the stability of the complexes in a physiological environment (**Figure 5B and Figure S6**). The diagram of radius of gyration (Rg) over time of these complexes gradually stabilized after 20ns (**Figure 5C and Figure S7**). The value of solvent-accessible surface area (SASA) of these complexes gradually stabilized which indicated that contact area between the quercetin and the proteins remained stable (**Figure 5D and Figure S8**). As for the change of hydrogen bond during simulation, the number of hydrogen bonds between quercetin and MMP1 primarily fluctuated between 1 and 2 after stabilization (**Figure 5E**). The number of hydrogen bonds between quercetin and STAT1 primarily fluctuated within a range of 1 to 4 (**Figure S9A**). And the number of hydrogen bonds between quercetin and PRKCA primarily fluctuated within a range of 3 to 5 (**Figure S9B**). As for quercetin and EGR1, the number of hydrogen bonds primarily fluctuated within a range of 2 to 5 after 65ns (**Figure S9C**). The binding free energies calculated using the MM/GBSA method were as follows: MMP1-quercetin (-46.39 ± 4.16 kJ/mol), EGR1-quercetin (-20.04 ± 1.26 kJ/mol), STAT1-quercetin (-60.70 ± 2.22 kJ/mol), and PRKCA-quercetin (-23.35 ± 2.28 kJ/mol) (**Figure 5F and Table 4**). These results corroborate the molecular docking findings and suggest that quercetin forms stable complexes with these proteins.

### The SPR assay of the quercetin and MMP1

To further investigate the direct interaction between quercetin and MMP1, SPR assay was conducted to measure the binding affinity of quercetin and MMP1. The results showed that quercetin binds to MMP1 in a concentration-dependent manner. The K_D_ value determined by the SPR experiment was 2.36 μM (**Figure 5G**).

### ADMET of Quercetin

**Figure 6:**
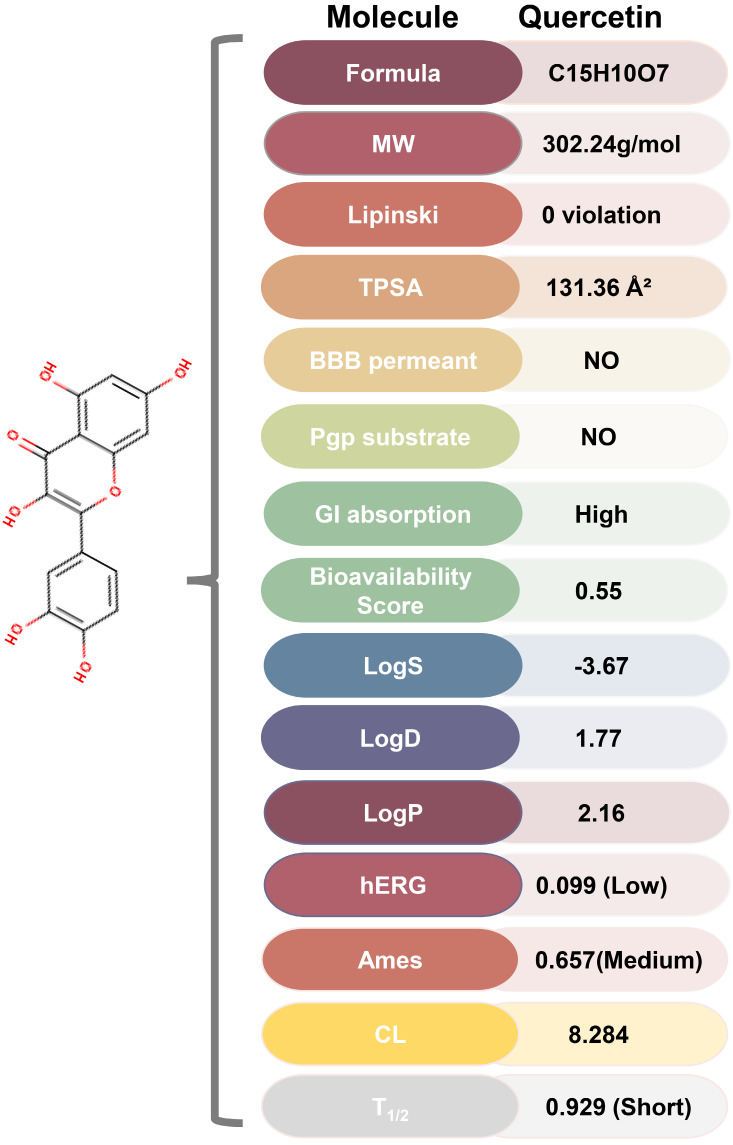
** ADMET of quercetin.** MW, Molecular Weight; Lipinski, Lipinski rule of five; TPSA, Topological Polar Surface Area, Optimal:0~140; BBB Penetration, Blood-Brain Barrier Penetration; Pgp substrate, P Glycoprotein; GI absorption, Gastrointestinal absorption; LogS, Log of the aqueous solubility, Optimal: -4~0.5 log mol/L; LogD, logP at physiological pH 7.4. Optimal: 1~3; LogP, Log of the octanol/water partition coefficient. Optimal: 0~3; hERG, human Ether-a-go-go Related Gene; Ames, the bacterial strains and mutagenicity test procedure developed by Bruce Ames; CL, Clearance, High: >15 mL/min/kg, moderate: 5-15 mL/min/kg, low: <5 mL/min/kg; T_1/2_: terminal elimination half-life, long half-life: >3h; short half-life: <3h.

ADMET analysis using ADMETlab 2.0 and SwissADME revealed that quercetin possesses favorable physicochemical properties, including optimal TPSA, logS, logD, and logP (**Figure 6, Figure S10**). Furthermore, quercetin's predicted absorption, distribution, metabolism, excretion, and toxicity profiles fall within ideal ranges for therapeutic compounds, suggesting its potential as a drug-like molecule.

### *In Vitro* Validation

**Figure 7:**
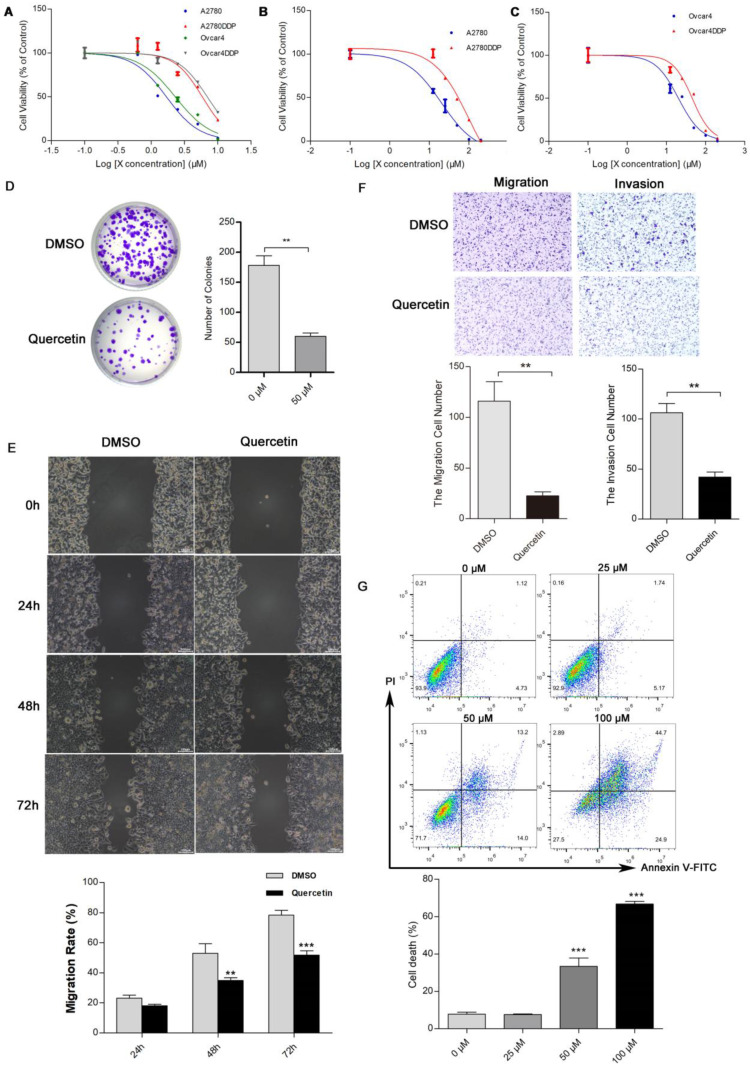
** Effects of quercetin on the proliferation, apoptosis, migration, and invasion of cisplatin-resistant EOC cells. (A), (B),** and **(C)** A2780, Ovcar4, A2780DDP, and Ovcar4DDP cells were treated with escalating concentrations of cisplatin or quercetin for 72 hours, MTT were used to determine the cell viability. **(D)** Colony formation assay of A2780DDP cells after treated with 0 (DMSO) or 50 μM of quercetin for 72 hours. **(E)** Wound-healing scratch assay in A2780DDP cells exposed to DMSO or quercetin (50 μM). **(F)** Transwell migration and invasion assay of A2780DDP cells treated with DMSO or 50 μM of quercetin for 72 hours. **(G)** Effect of quercetin on apoptosis in A2780DDP cells. Annexin V-PI double staining assay by flow cytometry were performed in cells after the treatment of quercetin for 72 hours. *p<0.05; **p<0.01; ***p<0.001.

**Figure 8:**
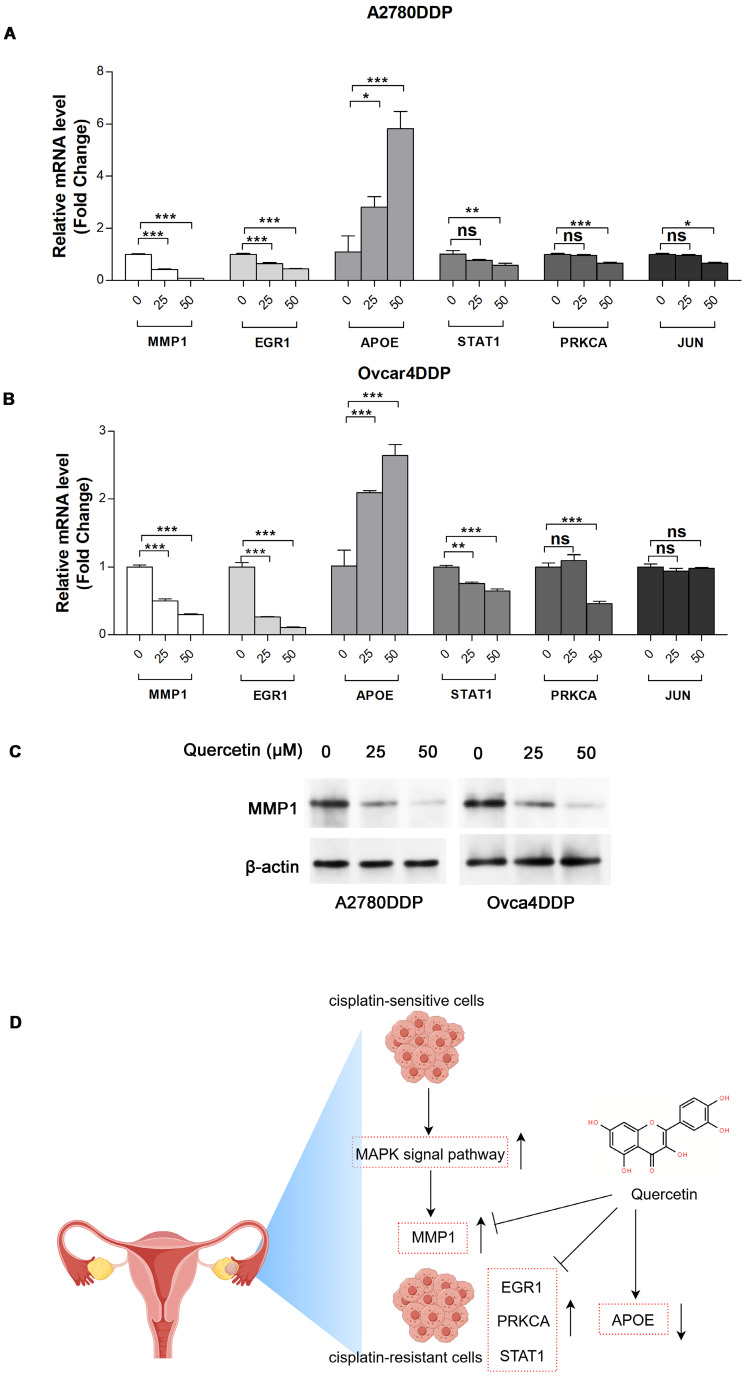
** Expression of MMP1, EGR1, APOE, STAT1, PRKCA, and JUN in A2780DDP and Ovcar4DDP treated with quercetin. (A)** and **(B)** A2780DDP and Ovcar4DDP cells were treated with 0, 25, 50μM of quercetin for 72 hours, and the mRNA expression levels of MMP1, EGR1, PRKCA, STAT1 and APOE were measured by qRT-PCR. **(C)** The protein expression of MMP1 in A2780DDP and Ovcar4DDP cells treated with 0, 25, 50μM quercetin measured by western blotting. **(D)** This picture displays the working model of quercetin. During the transition of wild-type EOC cells into cisplatin-resistant EOC cells, the MAPK signaling pathway is abnormally activated compared with wild-type EOC cells, leading to elevated expression of MMP1. Quercetin can induce apoptosis in cisplatin-resistant cells by regulating MMP1. Additionally, quercetin also influences other genes associated with cisplatin resistance. *p<0.05; **p<0.01; ***p<0.001.

The MTT assay indicated that cisplatin resistance in A2780DDP and Ovcar4DDP cells resulted in increased IC50 values (5.83 µM and 6.94 µM, respectively) compared to their parental counterparts (1.69 µM and 2.29 µM) (**Figure 7A, Table S11**). Quercetin treatment suppressed cell proliferation in both cisplatin-sensitive and -resistant cell lines, with IC50 values of 16.82 µM and 44.09 µM for A2780DDP and Ovcar4DDP cells, respectively (**Figures 7B, 7C**). Colony formation assays demonstrated that quercetin significantly reduced cell proliferation in A2780DDP and Ovcar4DDP cells (**Figure 7D, Figure S11A**). Quercetin also significantly impaired cell migration and invasion, as shown by the scratch and transwell assays (**Figures 7E, 7F, Figures S11B, S11C**). Apoptosis analysis via Annexin V/PI staining revealed that quercetin induced dose-dependent apoptosis in A2780DDP and Ovcar4DDP cells (**Figure 7G, Figure S11D**). qRT-PCR analysis confirmed that quercetin significantly downregulated MMP1, EGR1, STAT1, and PRKCA expression while upregulating APOE expression in these resistant cell lines (**Figures 8A, 8B**). Western blotting also demonstrated that the expression of MMP1 protein were decreased after the treatment of 25μM and 50μM quercetin for 72 hours (**Figure 8C**). Our finding indicated that quercetin could regulate the expression of MMP1, which was activated by MAPK signal pathway, as well as other hub CR genes to achieved tumor clearance (**Figure 8D**).

### Quercetin Enhances Cisplatin Toxicity in A2780DDP and Ovcar4DDP Cells

**Figure 9:**
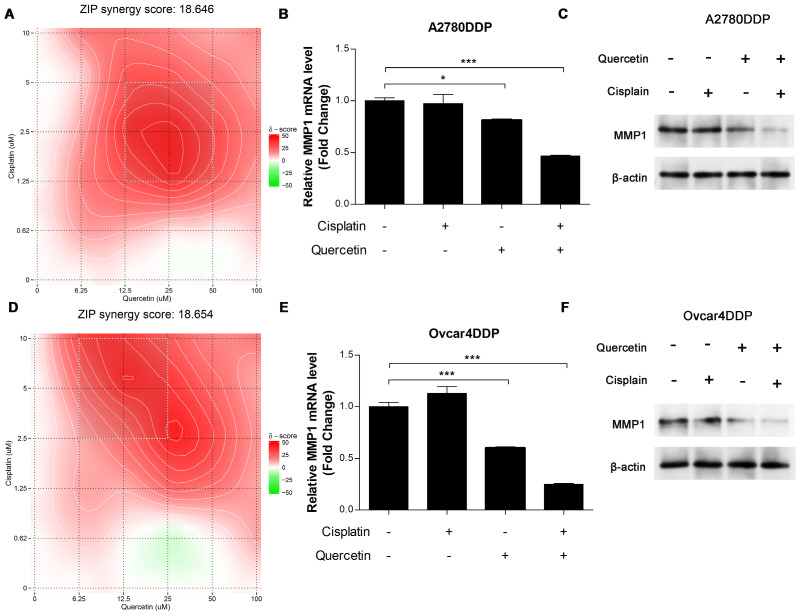
** The synergistic effect of cisplatin and quercetin. (A)** Heatmaps of drug combination responses. Cisplatin and quercetin act synergistically on A2780DDP. Cells were treated with both of cisplatin and quercetin of elevated concentrations for 72 hours. Then, MTT assay were performed to detect the cell viability. Synergyfinder were used to calculated the ZIP synergy scores and used for visualization. ZIP synergy score greater than 10 indicates a synergistic effect of drug combination. The gradient red areas represent the intensity of synergy at specific concentrations, with the white boxed region indicating the area with the strongest synergistic effect. **(B)** A2780DDP cells were treated with 0 μM cisplatin or quercetin, 1.25 μM cisplatin, 12.5 μM quercetin, the combination of 1.25 μM cisplatin and 12.5 μM quercetin respectively for 72h, and the mRNA expression of MMP1 was measured by qRT-PCR. **(C)** Heatmaps of drug combination responses. Cisplatin and quercetin act synergistically on Ovcar4DDP. **(D)** Ovcar4DDP cells were treated with 0 μM cisplatin or quercetin, 2.5 μM cisplatin, 6.25 μM quercetin, the combination of 2.5 μM cisplatin and 6.25 μM quercetin respectively, and the mRNA expression of MMP1 was measured by qRT-PCR. *p<0.05; **p<0.01; ***p<0.001.

Synergistic effects between quercetin and cisplatin were evaluated using the ZIP score via SynergyFinder. The combination of quercetin and cisplatin exhibited strong synergy, with ZIP scores greater than 10 in A2780DDP cells (**Figure 9A**). qRT-PCR and western blotting analysis revealed that either alone or combined with cisplatin, quercetin significantly downregulated MMP1 RNA and protein expression in A2780DDP (**Figures 9B, 9C**). Similar results were observed in the Ovcar4DDP cells (**Figure 9D, 9E, 9F**). These results highlight quercetin's potential to enhance the effectiveness of cisplatin in platinum-resistant EOC by modulating MMP1.

## Discussion

This study identified MMP1 as a central hub gene in cisplatin resistance in epithelial ovarian cancer (EOC). Through bioinformatics analysis of differentially expressed genes (DEGs) between cisplatin-sensitive and cisplatin-resistant EOC cell lines, followed by the construction of a protein-protein interaction (PPI) network, MMP1 emerged as a key regulator in the resistance phenotype. Our findings indicate that MMP1 is upregulated in platinum-resistant EOC and is associated with poor patient prognosis.

As a collagenase, MMP1 can cleave stromal collagen and various extracellular matrix (ECM) and non-ECM substrates 27. It plays an essential role in tissue remodeling, wound healing, and maintaining ECM integrity 27. However, no studies have yet explored the mechanism underlying the high expression of MMP1 in cisplatin-resistant EOC cells. We hypothesized the aberrant activation of multiple oncogenic signaling pathways likely drives MMP1's overexpression in cisplatin-resistant EOC cells comparing with wild-type EOC cells. Studies also showed that pathways such as PI3K/AKT, MAPK, and NF-kappaB, frequently activated in tumors, can increase MMP1 expression 27, 28. We were the first to find that activating MAPK pathway, which was suggest by KEGG enrichment analysis, in platinum-resistant EOC cells leads to the upregulation of MMP1.The upregulation of MMP1 is suggest to promote cancer cell proliferation, invasion, and metastasis in cancers 27. In EOC cells, MMP1 can be secreted into the tumor microenvironment via vesicles, enhancing the invasive capacity of the tumor cells 29. Additionally, intracellular MMP1 can increase the invasion and anti-apoptotic ability of EOC cells through a cascade reaction mediated by the MMP-1-PAR1 axis 30. Although MMP1 may not be the sole driver of cisplatin resistance in EOC, it likely acts as a crucial mediator of the resistant phenotype. Therefore, targeting MMP1 presents a promising approach to counteract cisplatin resistance in EOC.

While quercetin has been applied to treat platinum-resistant EOC, its potential as a therapeutic compound and mechanisms for this application were unclear. This study screened for CHMs candidate associated with MMP1. Using bioinformatics and molecular docking approaches, we found that quercetin interacts strongly with MMP1. The results of MD simulations further confirmed the stability of the protein-quercetin complexes, suggesting a strong potential for quercetin to modulate MMP1. Our *in vitro* experiments validated these findings, showing that quercetin effectively reduced cell proliferation, invasion, and migration in cisplatin-resistant EOC cells.

Additionally, although the underlying mechanisms involved were different, quercetin has been demonstrated to enhance the sensitivity of tumor cells to chemotherapy in various cancers, including triple-negative breast cancer and bladder cancer 31, 32. Our finding demonstrated that quercetin combined with cisplatin exhibited synergistic effect via zero interaction potency model, which may provide favorable support for conducting subsequent *in vivo* experiments or clinical studies. As for the potential mechanism, quercetin and cisplatin significantly downregulated MMP1 expression, the effect not observed with cisplatin treatment alone. These findings suggest that quercetin may be an effective adjuvant to overcome cisplatin resistance in EOC.

The advantage of Chinese herbal medicines (CHMs), such as quercetin, lies in their ability to target multiple molecular pathways simultaneously. Through Hit 2.0 and compound-targets network, quercetin was found to act on other hub genes involved in cisplatin resistance, including APOE, EGR1, STAT1, JUN, and PRKCA. Using molecular docking, we found that quercetin interacts strongly with STAT1, EGR1, and PRKCA. Our findings also showed that quercetin decreased the RNA expression of EGR1, STAT1, and PRKCA while increasing APOE expression in cisplatin-resistant EOC cells. Reduced APOE expression activates the FAK/ERK/MMP pathway, enhancing cell invasiveness and tumor progression 33. EGR1 and STAT1 are critical regulators of cell proliferation and tumorigenesis in EOC 34, 35, and quercetin's ability to inhibit these proteins may contribute to its efficacy. Although quercetin has been reported to inhibit JUN expression in other contexts, our study did not observe a significant effect on JUN expression in EOC cells. This discrepancy may reflect differences in cellular models or experimental conditions. Nonetheless, the multitarget activity of quercetin underscores its potential as a therapeutic strategy for overcoming cisplatin resistance in EOC.

In addition to its promising therapeutic effects, quercetin has favorable ADMET (Absorption, Distribution, Metabolism, Excretion, and Toxicity) profiles, which support its potential use in clinical settings. Our ADMET analysis using ADMETlab 2.0 and SwissADME indicated that quercetin possesses optimal physicochemical properties, including favorable values for TPSA, logS, logD, and logP. Furthermore, clinical studies have demonstrated the safety of quercetin at intravenous dosages of 945 mg/m² and oral dosages of ≥1000 mg/day, with good tolerability in cancer patients 36. These findings align with our predictions and suggest that quercetin has an acceptable safety profile, making it a promising candidate for subsequent clinical trials in cisplatin resistant EOC.

Screening appropriate CHMs for diseases mostly relies on clinical experience and ancient medical texts. However, this approach is inefficient and makes it hard to judge if the selected candidates are the most suitable CHMs. Several researches integrated bioinformatics, computer simulation, and *in vitro* and *in vivo* experiments to quickly find suitable CHMs. For example, Yilin Chen et al. used computer simulation and *in vitro* experiments to identify three hub genes for atherosclerosis and selected genistein as a therapeutic agent 25. Ziruo Talihati et al. used a similar method to screen six potential CHMs for cervical cancer patients 37. In these studies, bioinformatics finds hub genes in diseases, and computer simulation like molecular docking helps target these genes with CHMs. This approach reduces costs, makes the screening more targeted and rational, and allows further experimental verification. Our findings validate the feasibility of this strategy for platinum-resistant ovarian cancer.

While this study provides valuable insights into the potential of quercetin as a therapeutic agent for cisplatin-resistant EOC, several limitations should be addressed in future research. First, the interactions between quercetin and hub genes were validated based on bioinformatics predictions and *in vitro* assays, with no *in vivo* confirmation. Future studies should focus on animal models to validate these findings and assess the therapeutic efficacy of quercetin *in vivo*. Second, this study did not compare quercetin and other non-platinum-based chemotherapeutic agents, such as capecitabine or ifosfamide. Evaluating quercetin's relative efficacy in combination with these drugs would be beneficial to determine its potential advantages. And further research is needed to fully elucidate the molecular mechanisms by which quercetin overcomes cisplatin resistance, particularly the pathways through which it modulates MMP1 and other resistance-related genes. Understanding these mechanisms in greater detail will provide deeper insights into how quercetin can be optimized for clinical use. In addition, we found favorable ADMET profile of quercetin, however, the long-term safety of quercetin, particularly its potential toxicity and side effects during continuous therapeutic high-dose usage, there is currently a lack of sufficient data. This may be a challenge for future clinical trial.

## Conclusion

Combining bioinformatics analysis, molecular docking, molecular dynamics simulations, and *in vitro* validation, we identified MMP1 as a key target associated with cisplatin resistance in EOC and demonstrates the potential of quercetin, as therapeutic agents to overcome cisplatin resistance in EOC. This study also clarified the underlying mechanism by which quercetin affects the MMP1 to treat platinum-resistant EOC, justifying its potential as a therapeutic CHM. Moreover, the integrated analytical approach in this research offers a powerful strategy for rationalizing therapeutic natural products with improved efficacy and specificity. Expanding this approach to screen other natural products and their interactions with hub genes may lead to new treatments for other diseases.

## Supplementary Material

Supplementary figures and tables.

## Figures and Tables

**Table 1:** Multivariate Cox regression analysis of MMP1.

Characteristics	Total (N)	Hazard Ratio (95% CI)	P value
Age	90		
<60 years old	43	Reference	/
≥ 60 years old	47	2.0321(1.2248-3.372)	0.00605
Stage	90		
IIC	1	Reference	/
IIIB	1	7.9622 (0.4744-133.624)	0.14936
IIIC	75	1.0107 (0.1354-7.542)	0.99175
IV	13	0.9084(0.1083- 7.618)	0.92947
Grade	89		
G2	8	Reference	/
G3	81	1.1116 (0.4798-2.576)	0.80500
MMP1	90	1.2015 (1.0067-1.434)	0.04190

**Table 2:** Molecular docking results of Compounds with MMP1 (PDB ID: 3shi).

Compound Name	Pubchem ID	Formula	binding energy (kcal/mol)
Delphinidin	CID:128853	C15H10O7	-3.24
Eckol	CID:145937	C18H12O9	-1.04
Emodin	CID:3220	C15H10O5	-4.05
Esculetin	CID:5281416	C9H6O4	-3.73
Kaempferol	CID:5280863	C15H10O6	-3.17
Luteolin	CID:5280445	C15H10O6	-2.54
Phorbol Myristate Acetate	CID:27924	C36H56O8	-1.73
Quercetin	CID:5280343	C15H10O7	-5.49
Ursolic Acid	CID:64945	C30H48O3	-4.37
Wogonin	CID:5281703	C16H12O5	-2.54
(+/-)Nicotine	CID:942	C10H14N2	-3.18
(1E,6E)-1,7-Bis(4-Hydroxy-3-Methoxyphenyl)Hepta-1,6-Diene-3,5-Dione	CID:969516	C21H20O6	-2.86
Bornyl Acetate	CID:6950274	C12H20O2	-2.88
Apigenin	CID:5280443	C15H10O5	-2.78
Astragaloside Iv	CID:124761776	C41H68O14	-1.16
hypericin	CID:5281051	C30H16O8	-4.04
lutein	CID:6433159	C40H56O2	-2.1
Berberine	CID:2353	C20H18NO4	-3.06
Beta Carotene	CID:5280489	C40H56	-2.76
Capsaicin	CID:1548943	C18H27NO3	-2.12
Celastrol	CID:122724	C29H38O4	-1.98
Colchicine	CID:6167	C22H25NO6	-2.47

**Table 3:** Molecular docking results of quercetin with target proteins.

Target	PDB ID	Compound	binding energy (kcal/mol)
MMP1	3shi	Quercetin	-5.49
APOE	8cdy	Quercetin	-2.98
STAT1	1yvl	Quercetin	-5.61
PRKCA	8uak	Quercetin	-5.05
EGR1	4x9j	Quercetin	-5.35
JUN	5t01	Quercetin	-4.31

**Table 4:** The binding free energies calculated using the MM/GBSA method (kJ/mol).

Complex	ΔE_vdw_	ΔE_ele_	ΔE_pol_	ΔE_nonpol_	ΔE_MMPBSA_^a^	-TΔS	ΔG_bind_^b^
MMP1-quercetin	-137.708 ±1.764	-44.512 ±0.582	151.128 ±3.962	-15.301 ±0.06	-46.393 ±4.163	20.644 ±1.289	-25.749 ±5.344
STAT1- quercetin	-158.613 ±0.997	-61.116 ±1.646	217.153 ±0.588	-17.461 ±0.091	-20.038 ±1.261	19.893 ±6.069	-0.145 ±7.041
PRKCA- quercetin	-122.754 ±1.065	-113.837 ±2.842	192.533 ±6.049	-16.643 ±0.087	-60.703 ±2.22	17.744 ±2.314	-42.958 ±0.89
EGR1- quercetin	-70.677 ±5.267	-90.313 ±0.917	157.435 ±5.06	-13.794 ±0.535	-23.349 ±2.277	22.332 ±2.815	-1.017 ±4.421

ΔE_vdw_, Van der Waals energy; ΔE_ele_, Electrostatic energy; ΔE_pol_, Polar solvation energy; ΔE_nonpol_, non-Polar solvation energy; TΔS, solvation free energy change. ^a^ΔE_MMPBSA_=ΔE_vdw_+ΔE_ele_+ΔE_pol_+ΔE_nonpol;_^b^ΔG_bind_=ΔE_vdw_+ΔE_ele_+ΔE_pol_+ΔE_nonpol_-TΔS.

## References

[1] Vergote I, Gonzalez-Martin A, Lorusso D, Gourley C, Mirza MR, Kurtz JE (2022). Clinical research in ovarian cancer: consensus recommendations from the Gynecologic Cancer InterGroup. The Lancet Oncology.

[2] Herzog TJ, Pothuri B (2006). Ovarian cancer: a focus on management of recurrent disease. Nature Clinical Practice Oncology.

[3] Corrado G, Salutari V, Palluzzi E, Distefano MG, Scambia G, Ferrandina G (2017). Optimizing treatment in recurrent epithelial ovarian cancer. Expert review of anticancer therapy.

[4] Ortiz M, Wabel E, Mitchell K, Horibata S (2022). Mechanisms of chemotherapy resistance in ovarian cancer. Cancer Drug Resist.

[5] Yang L, Xie HJ, Li YY, Wang X, Liu XX, Mai J (2022). Molecular mechanisms of platinum-based chemotherapy resistance in ovarian cancer (Review). Oncology reports.

[6] Leung SOA, Konstantinopoulos PA (2021). Advances in the treatment of platinum resistant epithelial ovarian cancer: an update on standard and experimental therapies. Expert Opin Investig Drugs.

[7] Wu XH, Zhu JQ, Yin RT, Yang JX, Liu JH, Wang J (2021). Niraparib maintenance therapy in patients with platinum-sensitive recurrent ovarian cancer using an individualized starting dose (NORA): a randomized, double-blind, placebo-controlled phase III trial(☆). Annals of oncology: official journal of the European Society for Medical Oncology.

[8] Cheung F (2011). TCM: Made in China. Nature.

[9] Xie YJ, Gao WN, Wu QB, Yao XJ, Jiang ZB, Wang YW (2020). Chelidonine selectively inhibits the growth of gefitinib-resistant non-small cell lung cancer cells through the EGFR-AMPK pathway. Pharmacol Res.

[10] Zhai B, Wu Q, Wang W, Zhang M, Han X, Li Q (2020). Preparation, characterization, pharmacokinetics and anticancer effects of PEGylated β-elemene liposomes. Cancer biology & medicine.

[11] Zhang FY, Li RZ, Xu C, Fan XX, Li JX, Meng WY (2022). Emodin induces apoptosis and suppresses non-small-cell lung cancer growth via downregulation of sPLA2-IIa. Phytomedicine.

[12] Ma P, Yuan L, Jia S, Zhou Z, Xu D, Huang S (2024). Lonicerae Japonicae Flos with the homology of medicine and food: a review of active ingredients, anticancer mechanisms, pharmacokinetics, quality control, toxicity and applications. Frontiers in oncology.

[13] Zhang X, Wei X, Shi L, Jiang H, Ma F, Li Y (2024). The latest research progress: Active components of Traditional Chinese medicine as promising candidates for ovarian cancer therapy. J Ethnopharmacol.

[14] Wu J, Tang G, Cheng CS, Yeerken R, Chan YT, Fu Z (2024). Traditional Chinese medicine for the treatment of cancers of hepatobiliary system: from clinical evidence to drug discovery. Molecular cancer.

[15] He Q, Wan S, Jiang M, Li W, Zhang Y, Zhang L (2024). Exploring the therapeutic potential of tonic Chinese herbal medicine for gynecological disorders: An updated review. J Ethnopharmacol.

[16] Chen L, Liang L, Yan X, Liu N, Gong L, Pan S (2013). Survivin status affects prognosis and chemosensitivity in epithelial ovarian cancer. Int J Gynecol Cancer.

[17] Farrand L, Byun S, Kim JY, Im-Aram A, Lee J, Lim S (2013). Piceatannol enhances cisplatin sensitivity in ovarian cancer via modulation of p53, X-linked inhibitor of apoptosis protein (XIAP), and mitochondrial fission. J Biol Chem.

[18] Kasaian J, Mosaffa F, Behravan J, Masullo M, Piacente S, Iranshahi M (2016). Modulation of Multidrug Resistance Protein 2 Efflux in the Cisplatin Resistance Human Ovarian Carcinoma Cells A2780/RCIS by Sesquiterpene Coumarins. Phytother Res.

[19] Rauf A, Imran M, Butt MS, Nadeem M, Peters DG, Mubarak MS (2018). Resveratrol as an anti-cancer agent: A review. Crit Rev Food Sci Nutr.

[20] Sarkhosh-Inanlou R, Molaparast M, Mohammadzadeh A, Shafiei-Irannejad V (2020). Sanguinarine enhances cisplatin sensitivity via glutathione depletion in cisplatin-resistant ovarian cancer (A2780) cells. Chem Biol Drug Des.

[21] Nogales C, Mamdouh ZM, List M, Kiel C, Casas AI, Schmidt H (2022). Network pharmacology: curing causal mechanisms instead of treating symptoms. Trends Pharmacol Sci.

[22] Li X, Liu Z, Liao J, Chen Q, Lu X, Fan X (2023). Network pharmacology approaches for research of Traditional Chinese Medicines. Chin J Nat Med.

[23] Zhao L, Zhang H, Li N, Chen J, Xu H, Wang Y (2023). Network pharmacology, a promising approach to reveal the pharmacology mechanism of Chinese medicine formula. J Ethnopharmacol.

[24] Yin X, Li J, Hao Z, Ding R, Qiao Y (2022). A systematic study of traditional Chinese medicine treating hepatitis B virus-related hepatocellular carcinoma based on target-driven reverse network pharmacology. Front Cell Infect Microbiol.

[25] Chen Y, Zhang F, Sun J, Zhang L (2023). Identifying the natural products in the treatment of atherosclerosis by increasing HDL-C level based on bioinformatics analysis, molecular docking, and *in vitro* experiment. J Transl Med.

[26] Amaral MVS, AJ DESP, EL DAS, L DEOS, JH DASM, MEA DEM (2019). Establishment of Drug-resistant Cell Lines as a Model in Experimental Oncology: A Review. Anticancer Res.

[27] Cabral-Pacheco GA, Garza-Veloz I, Castruita-De la Rosa C, Ramirez-Acuña JM, Perez-Romero BA, Guerrero-Rodriguez JF (2020). The Roles of Matrix Metalloproteinases and Their Inhibitors in Human Diseases. International journal of molecular sciences.

[28] Wu T, Jiao Z, Li Y, Su X, Yao F, Peng J (2022). HPRT1 Promotes Chemoresistance in Oral Squamous Cell Carcinoma via Activating MMP1/PI3K/Akt Signaling Pathway. Cancers.

[29] Yokoi A, Yoshioka Y, Yamamoto Y, Ishikawa M, Ikeda SI, Kato T (2017). Malignant extracellular vesicles carrying MMP1 mRNA facilitate peritoneal dissemination in ovarian cancer. Nature communications.

[30] Wang FQ, Fisher J, Fishman DA (2011). MMP-1-PAR1 axis mediates LPA-induced epithelial ovarian cancer (EOC) invasion. Gynecologic oncology.

[31] Meng S, Cao Y, Lu L, Li X, Sun S, Jiang F (2024). Quercetin Promote the Chemosensitivity in Organoids Derived from Patients with Breast Cancer. Breast Cancer (Dove Med Press).

[32] Cho CJ, Yu CP, Wu CL, Ho JY, Yang CW, Yu DS (2021). Decreased drug resistance of bladder cancer using phytochemicals treatment. Kaohsiung J Med Sci.

[33] Lai H, Zhao X, Qin Y, Ding Y, Chen R, Li G (2018). FAK-ERK activation in cell/matrix adhesion induced by the loss of apolipoprotein E stimulates the malignant progression of ovarian cancer. J Exp Clin Cancer Res.

[34] Geng T, Sun Q, He J, Chen Y, Cheng W, Shen J (2024). CXXC5 drove inflammation and ovarian cancer proliferation via transcriptional activation of ZNF143 and EGR1. Cell Signal.

[35] Gaire B, Padmanabhan S, Zou Y, Uddin MM, Reddy SU, Vancurova I (2023). IFNγ induces Bcl3 expression by JAK1/STAT1/p65 signaling, resulting in increased IL-8 expression in ovarian cancer cells. FEBS Open Bio.

[36] Ferry DR, Smith A, Malkhandi J, Fyfe DW, deTakats PG, Anderson D (1996). Phase I clinical trial of the flavonoid quercetin: pharmacokinetics and evidence for *in vivo* tyrosine kinase inhibition. Clin Cancer Res.

[37] Talihati Z, Abudurousuli K, Hailati S, Han M, Nuer M, Khan N (2025). Screening of Hub Genes and Therapeutic Drugs in Cervical Cancer Using Integrated Bioinformatics Analysis. J Cancer.

