# Supplementary material for: Identification of Quercetin as a Natural MMP1 Inhibitor for Overcoming Cisplatin Resistance in Epithelial Ovarian Cancer
Source: J Cancer. 2025 May 31;16(8):2578–94. doi: 10.7150/jca.110517 (PMC12171002; doi:10.7150/jca.110517)
Supplement: Supplementary file 1 — Supplementary figures and tables. [file jcav16p2578s1.pdf]

Supplementary Figure

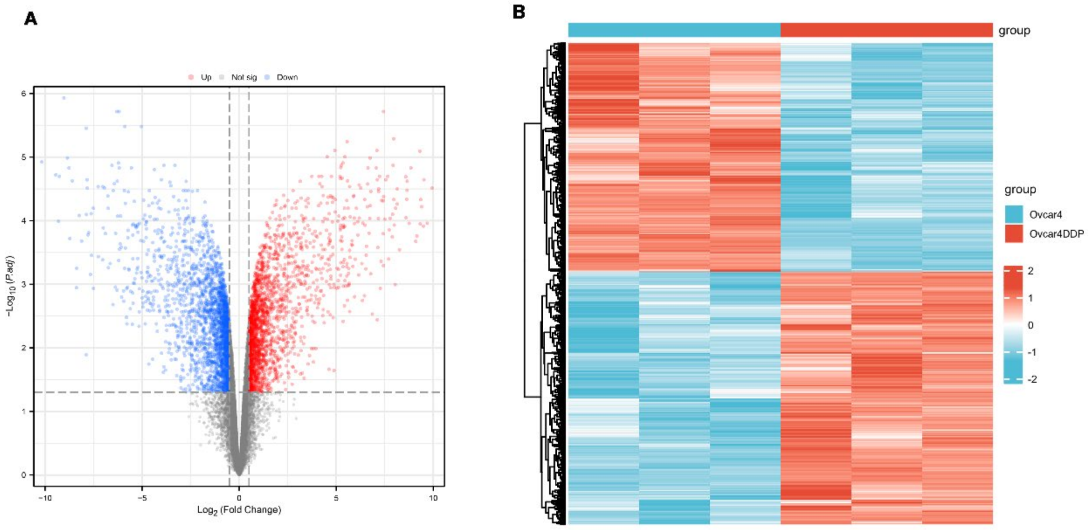

Supplementary Figure 1. Identification of differential expressed genes (DEGs) between Ovcar4 and Ovcar4DDP cells. (A) The volcano plot of DEGs between Ovcar4 and Ovcar4DDP cells. DEGs between Ovcar4 and Ovcar4DDP cells were identified using the limma package in R. Genes with a log2(fold change) threshold of >0.5 and a P value < 0.05 were marked in blue (downregulated genes) or red (upregulated genes). (B) The heatmap of DEGs between Ovcar4 and Ovcar4DDP cells.

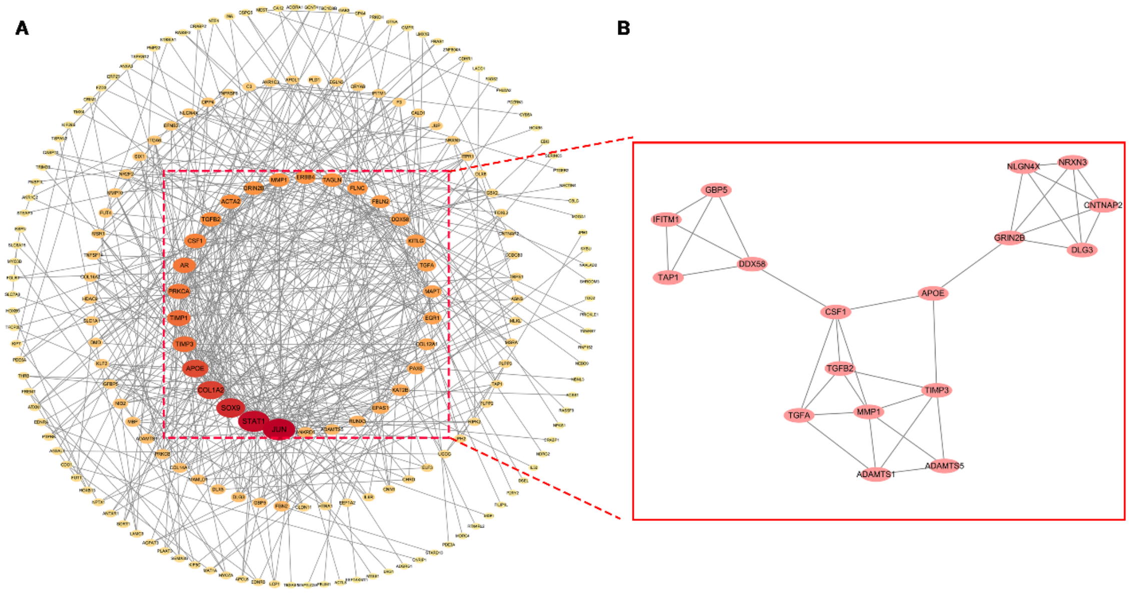

Supplementary Figure 2. PPI network construction of cisplatin-resistance-related (CR) genes. (A) The PPI network of DEGs was visualized using the STRING and

Cytoscape software. The color intensity and size of genes in the PPI network are determined by their DEGREE values in the PPI network. (B) MCODE plugin in Cytoscape software were performed to identified the core submodule of the PPI network. 17 genes including MMP1, IFITM1, TGFB2, TIMP3, APOE, and CSF1 were identified in the core submodule.

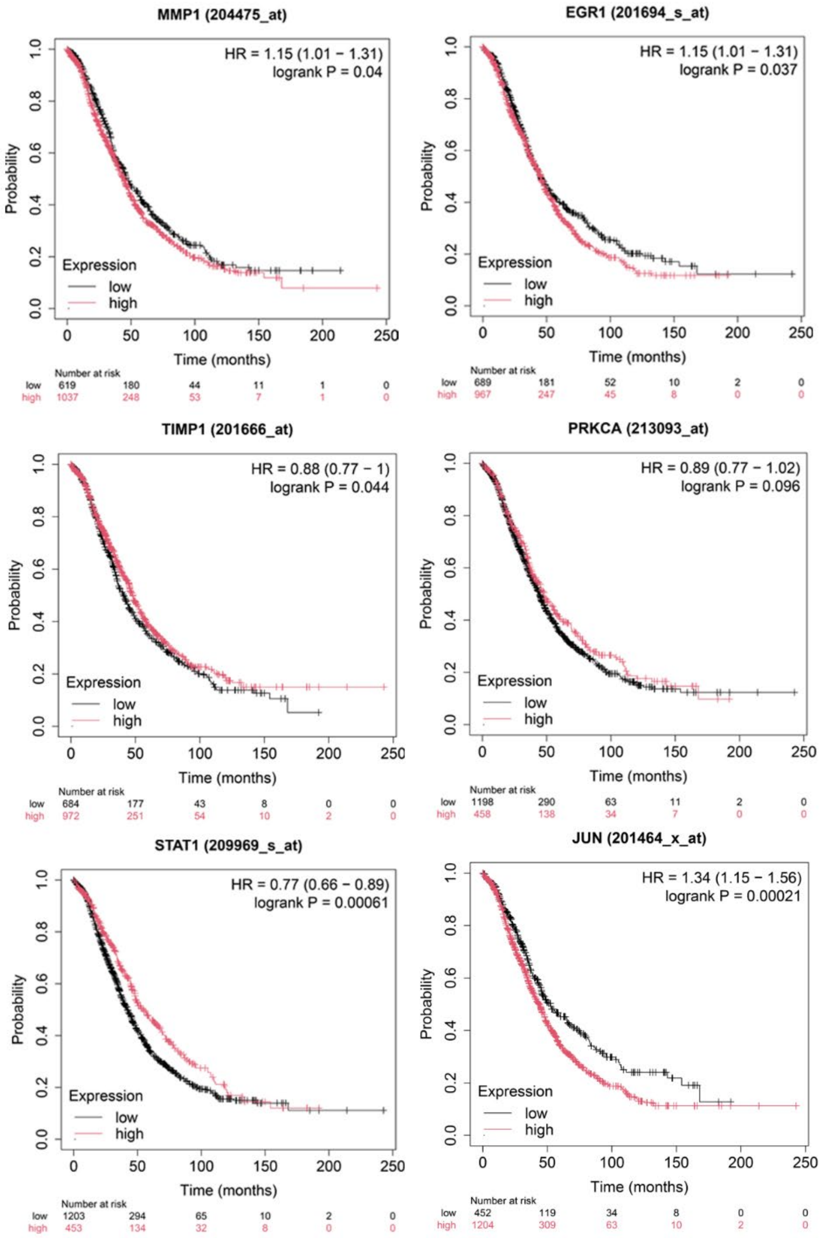

Supplementary Figure 3. Survival curves of OC patients in different high- and low-expressed RNA groups analyzed by the Kaplan-Meier plotter. The expression of MMP1,

EGR1, TIMP1, STAT1, and JUN is associated with overall survival (OS) in EOC patients ($P < 0.05$). And High expression of MMP1 and EGR1 was found to be associated with poor OS in EOC.

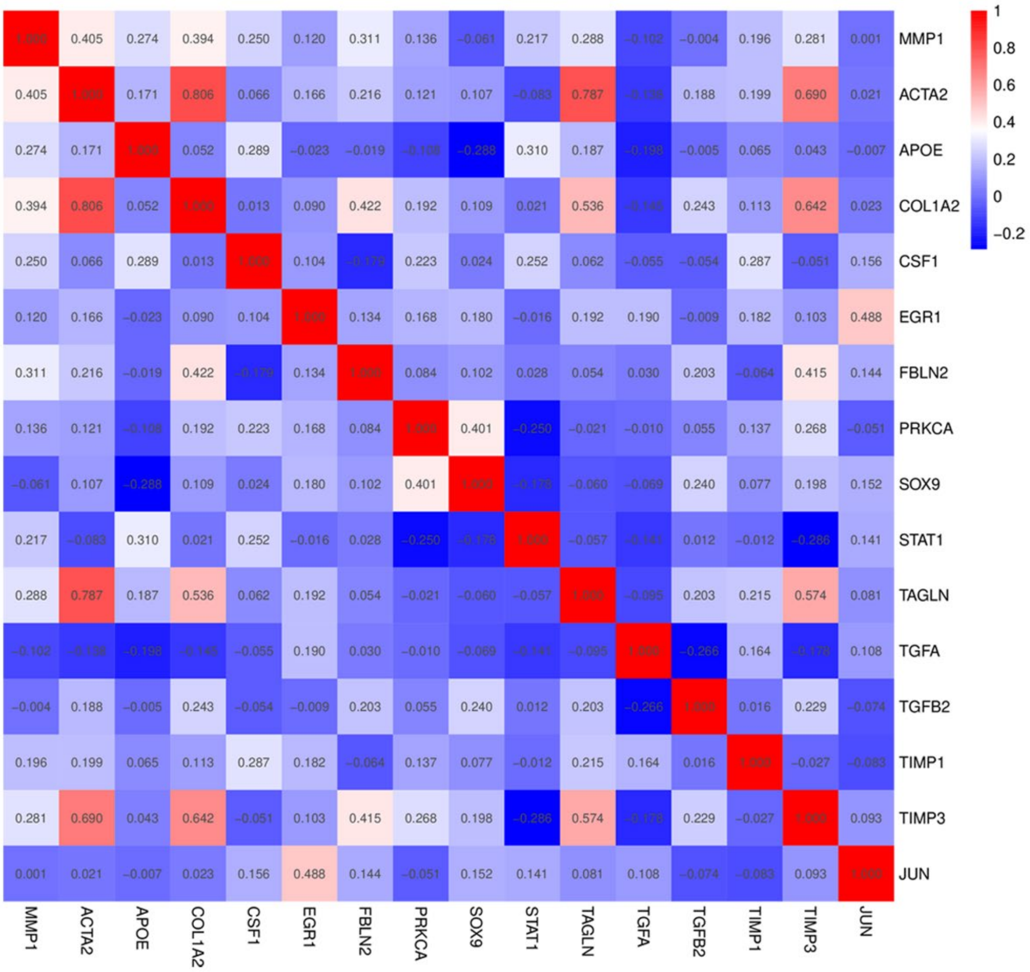

Supplementary Figure 4. Correlation matrix of hub genes. Spearman's method was used to analyze the correlations among hub genes, and the correlation matrix was plotted based on the Spearman correlation coefficient (rs). A coefficient of 0.9 < rs < 1 indicates a high correlation; 0.7 < rs < 0.9 indicates a strong correlation; 0.4 < rs < 0.7 indicates a moderate correlation; 0.2 < rs < 0.4 indicates a weak correlation; and 0 < rs < 0.2 indicates a very weak correlation or no correlation.

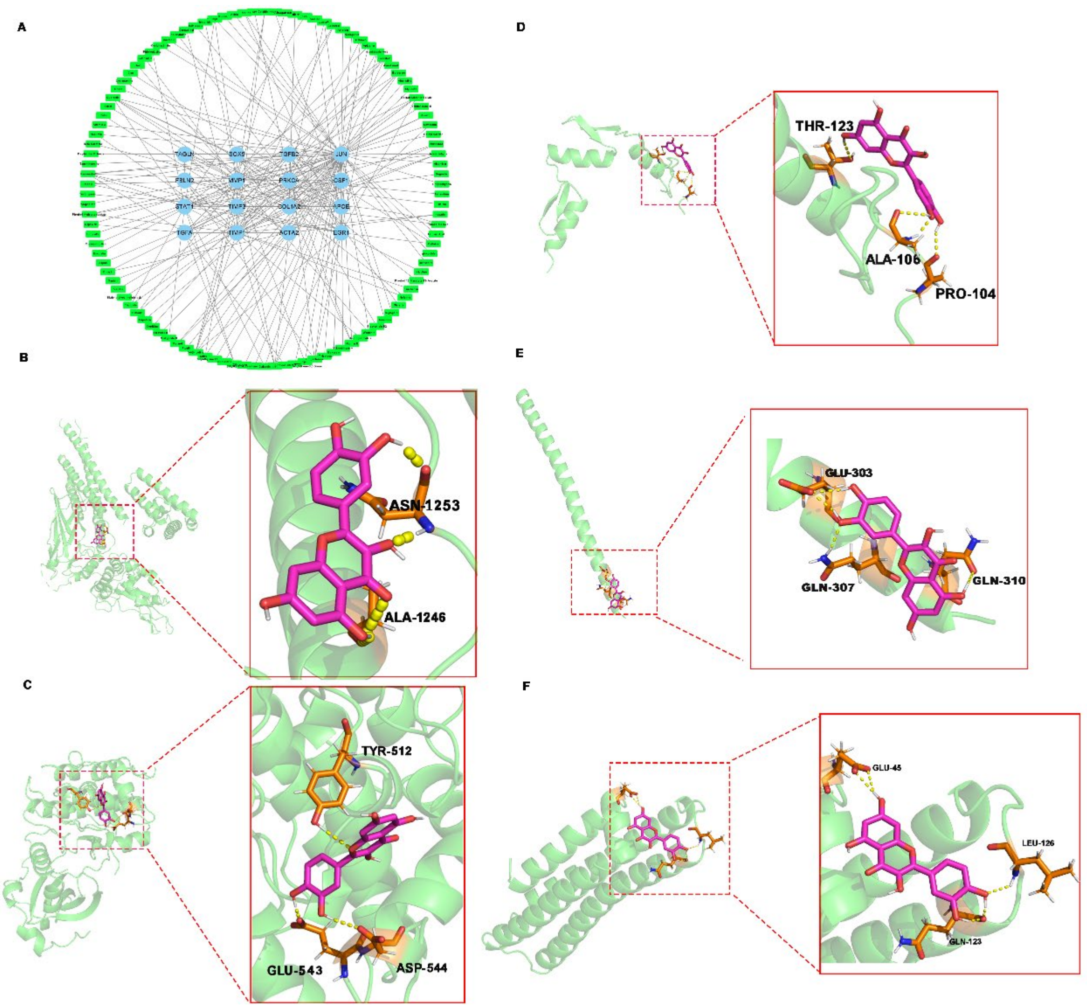

Supplementary Figure 5. CHMs and targets. (A) The target-compound network of CHMs and hub genes. Green dots represent hub genes, blue dots represent CHMs, and connected edges indicate a correlation between them. Molecular docking conformation of Quercetin interacting with proteins (B-F). Green, pink, and orange, and white represent protein receptors, small drug ligands, amino acid residues, and hydrogen bonds, and amino acid residues. (B) Molecular docking conformation of Quercetin interacting with STAT1. (C) Molecular docking conformation of Quercetin interacting with PRKCA. (D) Molecular docking conformation of Quercetin interacting with EGR1. (E) Molecular docking conformation of Quercetin interacting with c-Jun. (F) Molecular docking conformation of Quercetin interacting with APOE.

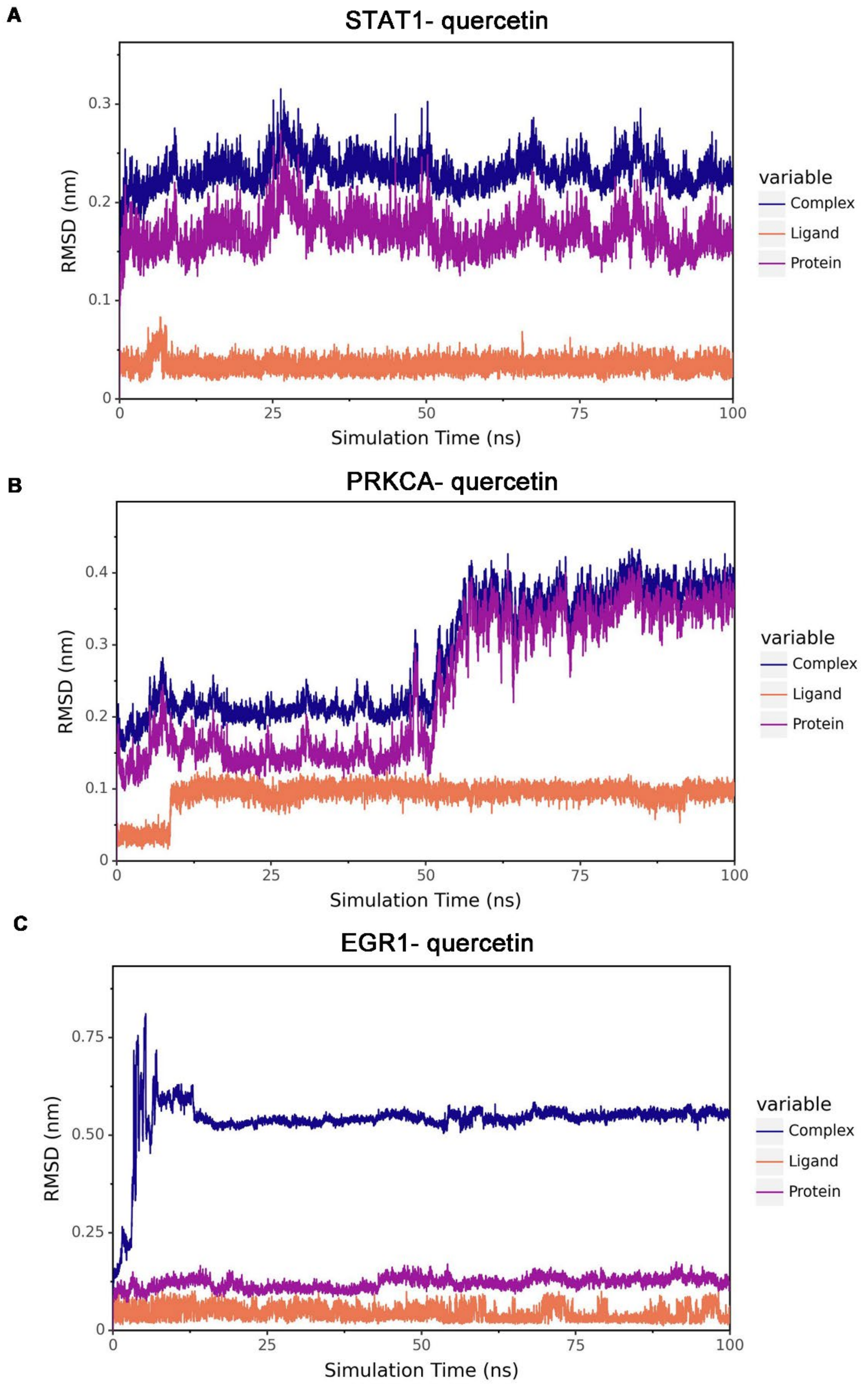

Supplementary Figure 6. RMSD values in MD Simulations of protein–ligand complexes：STAT1- quercetin, PRKCA-quercetin, and EGR1-quercetin. (A) RMSD values of the complexes of STAT1- quercetin. (B) RMSD values of the complexes of PRKCA-quercetin. (C) RMSD values of the complexes of EGR1-quercetin.

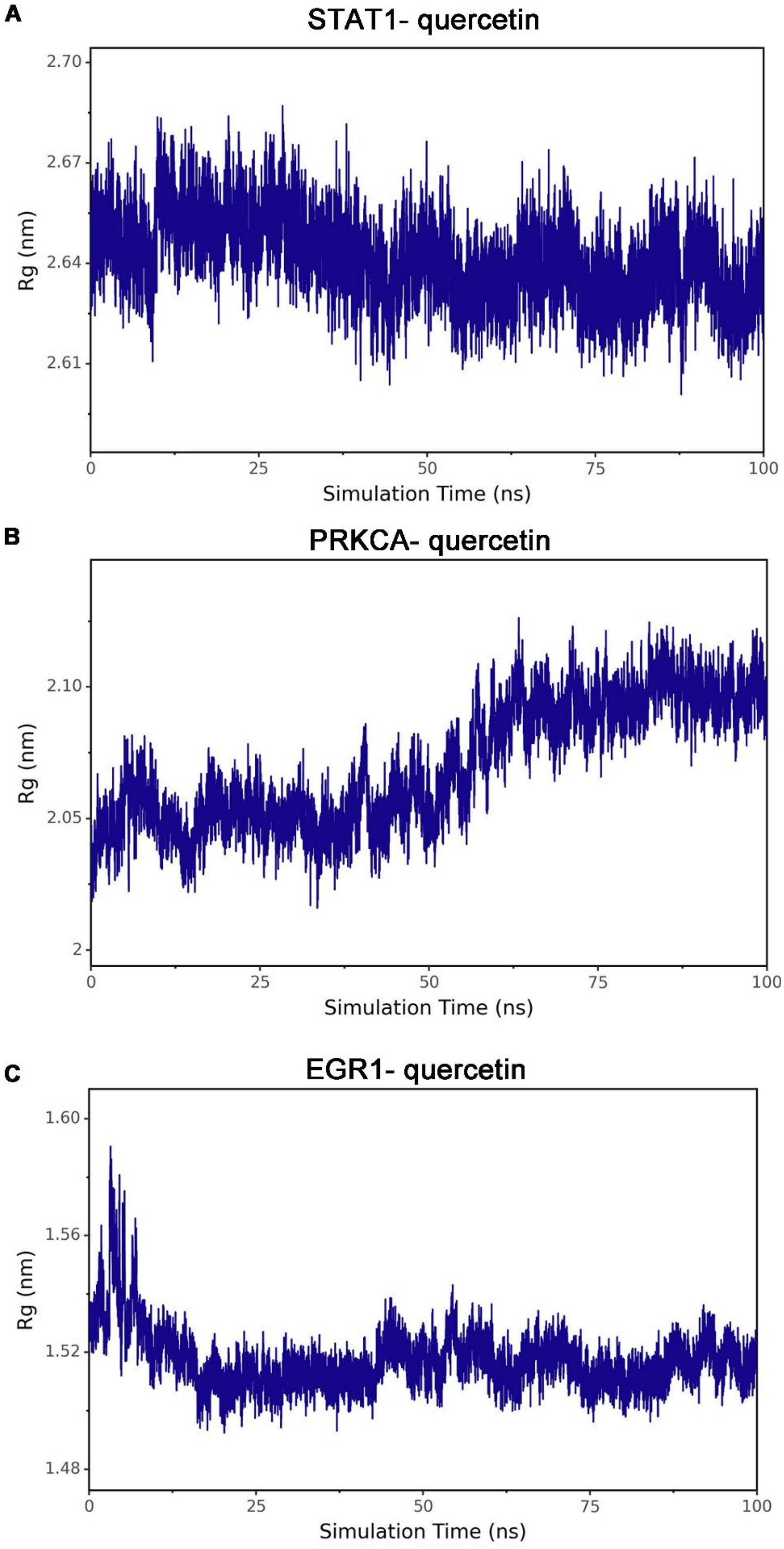

Supplementary Figure 7. The diagram of Rg over time of protein–ligand complexes.

(A) The diagram of Rg of STAT1- quercetin. (B) The diagram of Rg of PRKCA-quercetin.

(C) The diagram of Rg of EGR1-quercetin.

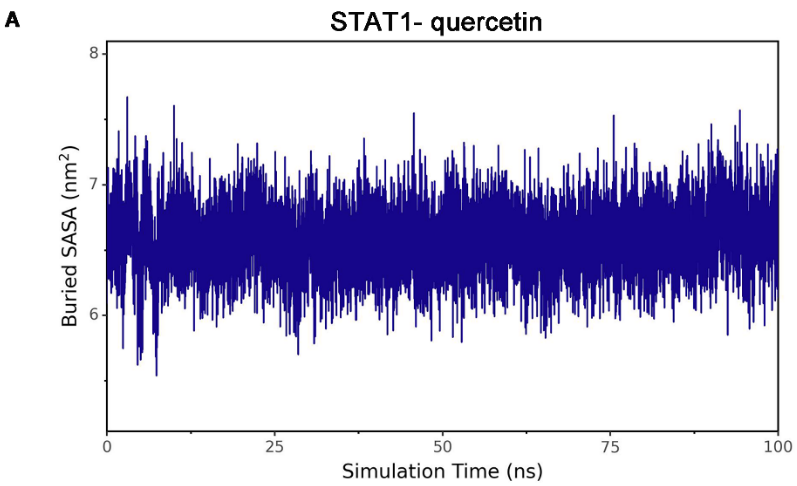

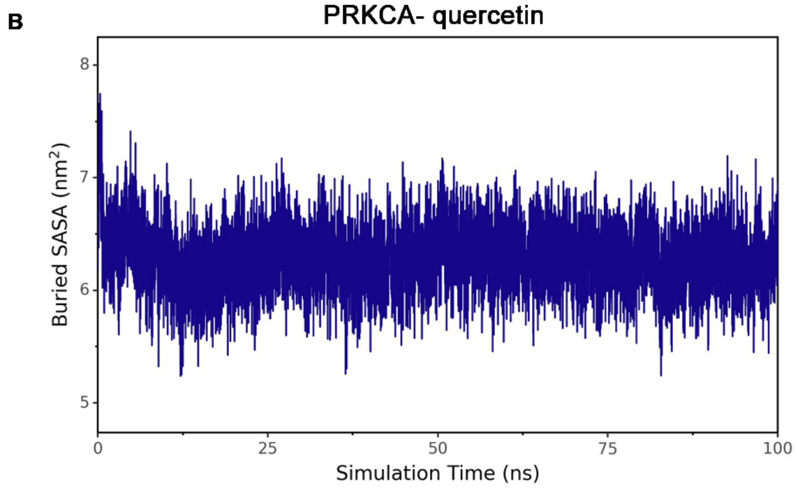

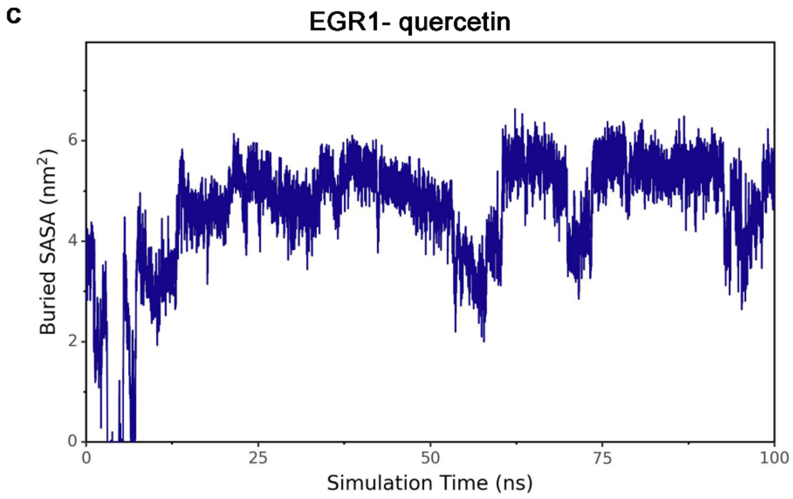

Supplementary Figure 8. SASA values in MD Simulations of protein–ligand

complexes：STAT1- quercetin, PRKCA-quercetin, and EGR1-quercetin. (A) SASA values of STAT1- quercetin complex. (B) SASA values of PRKCA-quercetin complex. (C) SASA values of EGR1-quercetin complex.

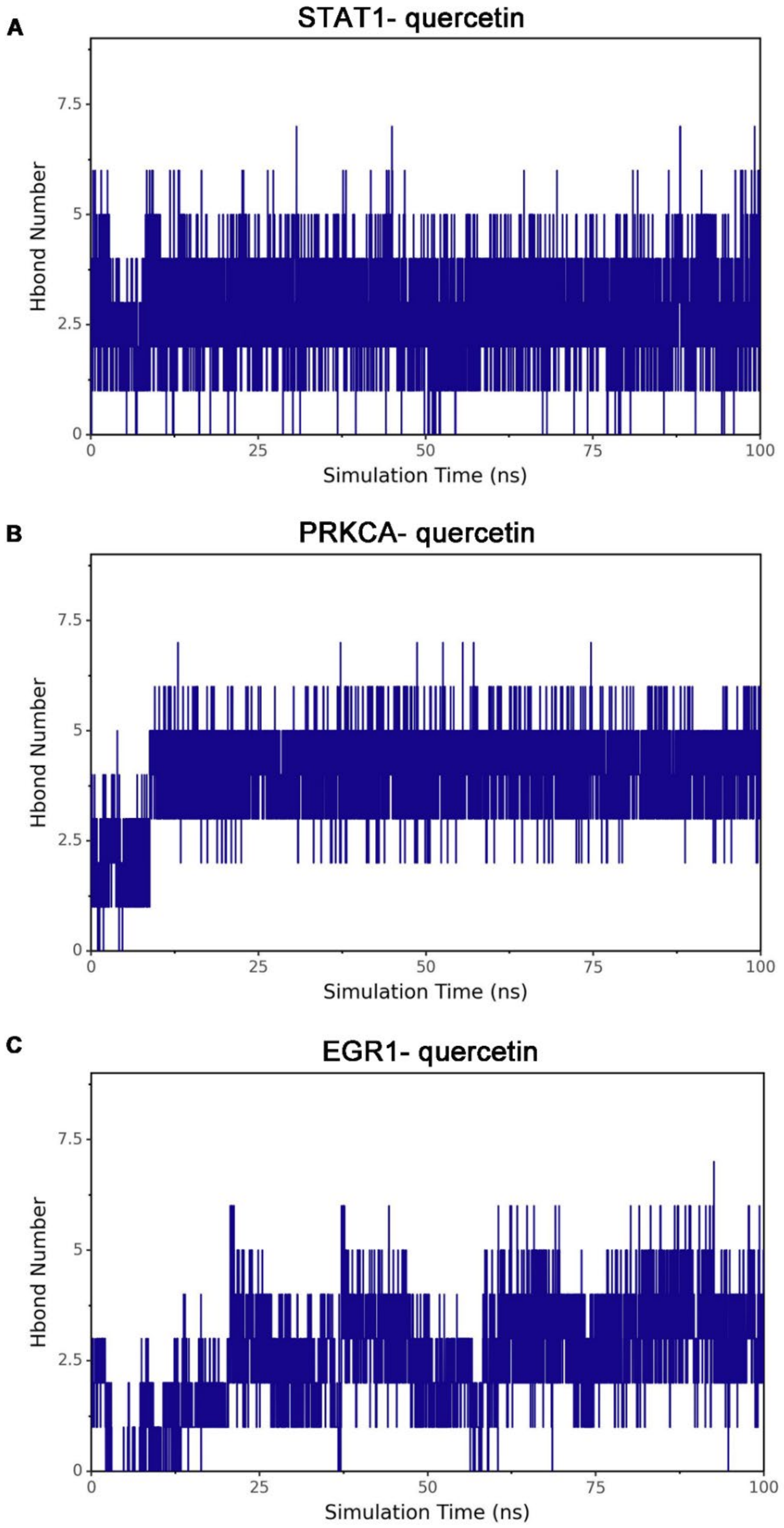

Supplementary Figure 9. Variation in the number of hydrogen bonds within the complexes during the molecular simulation. (A) The variation in the number of hydrogen bonds within STAT1- quercetin complex. (B) The variation in the number of hydrogen bonds within PRKCA- quercetin complex. (C) The variation in the number of hydrogen bonds within EGR1- quercetin complex.

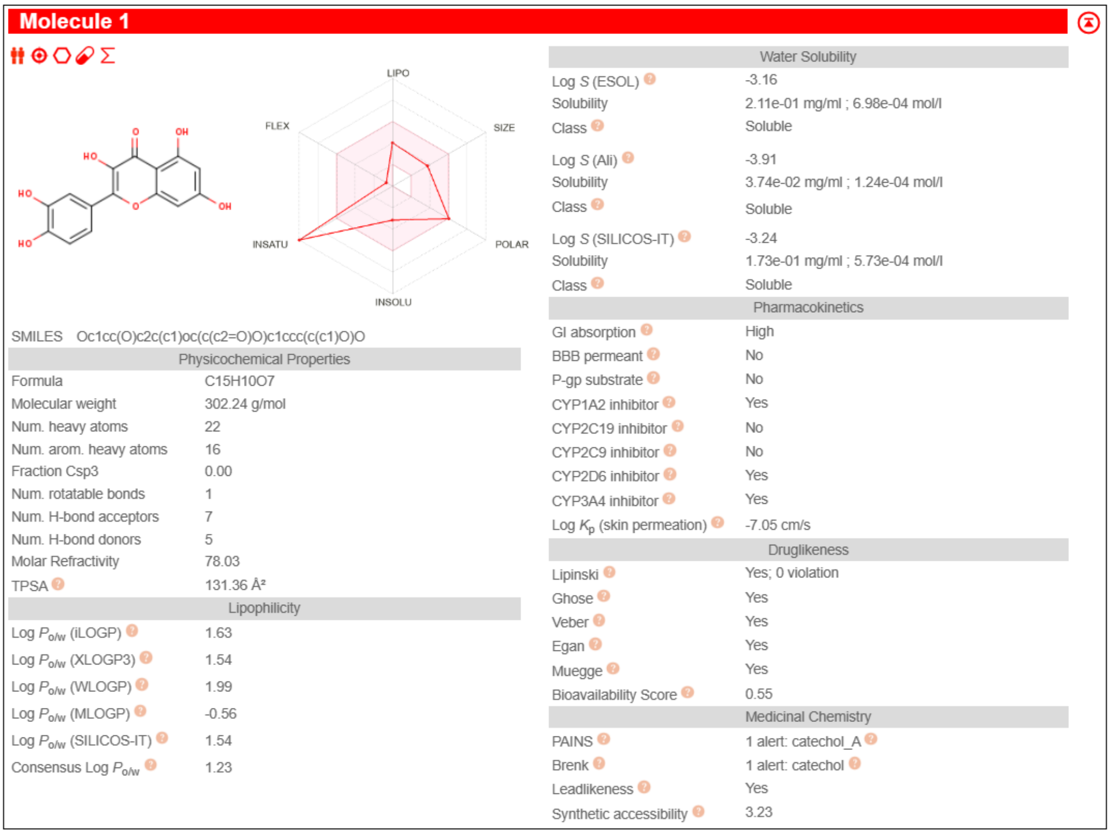

SMILES Oc1cc(O)c2c(c1)oc(c(c2=O)O)c1ccc(c(c1)O)O

Physicochemical Properties	
Formula	C15H10O7
Molecular weight	302.24 g/mol
Num. heavy atoms	22
Num. arom. heavy atoms	16
Fraction Csp3	0.00
Num. rotatable bonds	1
Num. H-bond acceptors	7
Num. H-bond donors	5
Molar Refractivity	78.03
TPSA	131.36 Å²

Lipophilicity	
Log $P_{o/w}$ (iLOGP)	1.63
Log $P_{o/w}$ (XLOGP3)	1.54
Log $P_{o/w}$ (WLOGP)	1.99
Log $P_{o/w}$ (MLOGP)	-0.56
Log $P_{o/w}$ (SILICOS-IT)	1.54
Consensus Log $P_{o/w}$	1.23

Water Solubility	
Log *S* (ESOL)	-3.16
Solubility	2.11e-01 mg/ml ; 6.98e-04 mol/l
Class	Soluble
Log *S* (Ali)	-3.91
Solubility	3.74e-02 mg/ml ; 1.24e-04 mol/l
Class	Soluble
Log *S* (SILICOS-IT)	-3.24
Solubility	1.73e-01 mg/ml ; 5.73e-04 mol/l
Class	Soluble

Pharmacokinetics	
GI absorption	High
BBB permeant	No
P-gp substrate	No
CYP1A2 inhibitor	Yes
CYP2C19 inhibitor	No
CYP2C9 inhibitor	No
CYP2D6 inhibitor	Yes
CYP3A4 inhibitor	Yes
Log K_p (skin permeation)	-7.05 cm/s

Druglikeness	
Lipinski	Yes; 0 violation
Ghose	Yes
Veber	Yes
Egan	Yes
Muegge	Yes
Bioavailability Score	0.55

Medicinal Chemistry	
PAINS	1 alert: catechol_A
Brenk	1 alert: catechol
Leadlikeness	Yes
Synthetic accessibility	3.23

Supplementary Figure 10. The report of quercetin from SwissADME. The chemical structure of quercetin was uploaded to the SwissADME to obtain its ADME properties.

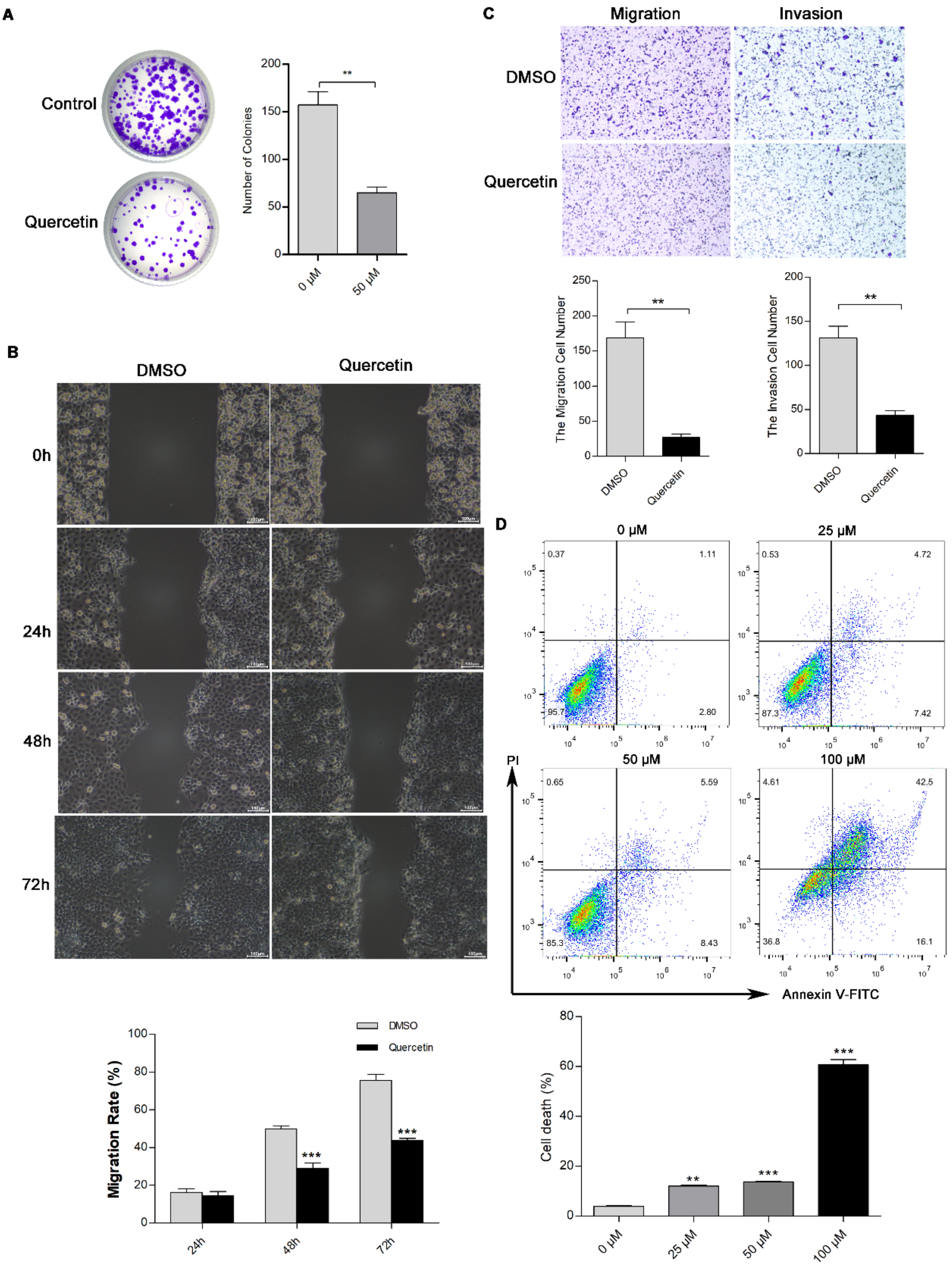

Supplementary Figure 11. Effects of quercetin on the proliferation, apoptosis, migration, and invasion of Ovcar4DDP cells. (A) Colony formation assay of Ovcar4DDP cells after treated with 0 or 50 μM of quercetin for 72 hours. (B) Wound-healing scratch assay in Ovcar4DDP cells exposed to DMSO or quercetin (50 μM) for 72 hours. The Scratch of cells treated with DMSO or quercetin were photographed at 0h, 24h, 48h, and 72h. (C) Transwell migration and invasion assay of Ovcar4DDP cells

treated with 0 or 50 μM of quercetin for 72 hours. Cells that migrated or invaded through the membrane were counted using ImageJ2 software. (D) Effect of quercetin on apoptosis in Ovcar4DDP cells. Annexin V-PI double staining assay by flow cytometry were performed in cells after the treatment of 0μM, 25μM, 50μM, and 100μM quercetin for 72 hours. $*p < 0.05$; $**p < 0.01$; $***p < 0.001$.

Supplementary Table 1: qRT-PCR Primers

Gene	Sequence (5' -> 3')	
MMP1	Forward	TTTCATTTCTGTTTTCTGGCCA
	Reverse	CATCTCTGTCGGCAAATTCGT
STAT1	Forward	CCATCCTTTGGTACAACATGC
	Reverse	TGCACATGGTGGAGTCAGG
EGR1	Forward	GCCTGCGACATCTGTGGAA
	Reverse	CGCAAGTGGATCTTGGTATGC
APOE	Forward	TGCTCAGCTCCCAGGTCAC
	Reverse	GCCTTCAACTCCTTCATGGTCT
PRKCA	Forward	ATGGCTGATGTCTTCCAGCA
	Reverse	AAGCCCCATATAAAGTCGGT
JUN	Forward	GAGCTGGAGCGCCTGATAAT
	Reverse	CCCTCCTGCTCATCTGTCAC
GAPDH	Forward	GCAAAGTGGAGATTGTTGCCAT
	Reverse	CCTTGACTGTGCCGTTGAATTT

Supplementary Table 2: DEGs in A2780/A2780DDP

gene	logFC	AveExpr	t	P.Value	adj.P.Val	B
INHBA	-3.0661	1.5832	-53.018	5.68E-14	6.65E-11	22.7814
CD36	-2.9146	1.65333	-22.857	3.26E-10	3.63E-08	13.828
TBR1	-2.8862	1.78963	-22.72	3.46E-10	3.81E-08	13.7628
KHDRBS3	-2.7387	1.92803	-36.459	2.71E-12	1.21E-09	18.8807
PTGER3	-2.7332	1.59663	-34.602	4.64E-12	1.69E-09	18.322
SLC4A4	-2.7206	1.8817	-31.328	1.29E-11	3.09E-09	17.2528
PERP	-2.7007	1.68926	-24.188	1.83E-10	2.33E-08	14.4451
GALNT14	-2.6964	1.59906	-24.422	1.66E-10	2.19E-08	14.5499
BACE2	-2.6849	1.49294	-30.913	1.48E-11	3.44E-09	17.1089
ST3GAL1	-2.6096	1.43451	-29.028	2.82E-11	5.64E-09	16.4281
TDO2	-2.5523	1.52702	-20.84	8.33E-10	7.42E-08	12.8207
RUNX3	-2.5375	1.39846	-22.629	3.61E-10	3.94E-08	13.7187
PTPN3	-2.5027	1.63191	-20.332	1.07E-09	9.09E-08	12.5519
PDLIM1	-2.492	1.37569	-25.823	9.37E-11	1.40E-08	15.1574
FILIP1	-2.469	2.09818	-26.99	5.96E-11	9.62E-09	15.6379
FAM9C	-2.4289	2.69696	-47.065	1.95E-13	1.72E-10	21.5648
LRRTM3	-2.3838	1.75529	-26.058	8.54E-11	1.29E-08	15.2557
DNAJC6	-2.379	1.34001	-30.645	1.62E-11	3.61E-09	17.0148
CHST15	-2.3572	1.22875	-37.823	1.86E-12	9.11E-10	19.2719
CAMK4	-2.299	1.4165	-17.227	5.71E-09	3.56E-07	10.7499
CACNA1H	-2.2963	1.632	-13.87	4.96E-08	2.13E-06	8.41304
SHTN1	-2.282	1.34167	-21.374	6.44E-10	6.10E-08	13.097
GABRA5	-2.2676	1.3932	-22.603	3.65E-10	3.96E-08	13.7066
LINC0088	-2.2222	1.487	-16.135	1.10E-08	6.08E-07	10.0402
RALYL	-2.2163	1.15833	-35.222	3.87E-12	1.51E-09	18.5121
MYO6	-2.2147	1.96061	-29.174	2.68E-11	5.44E-09	16.4823
NFATC1	-2.2074	1.25419	-19.734	1.45E-09	1.14E-07	12.2267
SLC4A10	-2.2052	1.31181	-21.072	7.44E-10	6.81E-08	12.9419
SOX8	-2.1125	1.66215	-20.712	8.87E-10	7.83E-08	12.7538
JAKMIP1	-2.1114	1.0557	-34.355	5.00E-12	1.77E-09	18.2451
SOST	-2.1079	1.28401	-21.258	6.81E-10	6.35E-08	13.0374
GALNT18	-2.0961	1.22789	-17.441	5.04E-09	3.23E-07	10.8833
COL12A1	-2.0785	3.13126	-28.47	3.45E-11	6.68E-09	16.2176
HSPA12A	-2.0749	1.16714	-22.539	3.76E-10	4.05E-08	13.6753
MMP1	-2.0731	2.97492	-50.392	9.60E-14	9.43E-11	22.266
CEROX1	-2.072	1.41186	-15.47	1.68E-08	8.61E-07	9.586
PPIC	-2.0459	1.52	-18.085	3.50E-09	2.41E-07	11.2774
HTRA1	-2.0407	1.12069	-25.589	1.03E-10	1.50E-08	15.0582
GRID1	-2.0364	1.14787	-15.746	1.41E-08	7.54E-07	9.77635
GNAI1	-2.0155	2.49683	-31.609	1.18E-11	2.99E-09	17.3491
LMX1B	-2.0118	1.13557	-18.181	3.32E-09	2.31E-07	11.3349
FAM83H	-2.0095	1.3143	-17.377	5.23E-09	3.33E-07	10.8436
ARHGAP	-1.9971	1.04872	-27.835	4.34E-11	7.84E-09	15.9729
NTN1	-1.9947	1.57393	-12.672	1.21E-07	4.52E-06	7.44994
SFMBT2	-1.9625	1.16113	-20.978	7.79E-10	7.02E-08	12.893
LRATD2	-1.9619	1.39082	-17.614	4.57E-09	2.98E-07	10.9905
CD55	-1.9335	1.37663	-16.128	1.11E-08	6.09E-07	10.0358
LRRK1	-1.9023	1.48986	-20.221	1.13E-09	9.47E-08	12.4924
PLPP2	-1.8687	1.3366	-9.7653	1.48E-06	3.97E-05	4.73339
LINC0248	-1.8645	0.9824	-31.12	1.38E-11	3.25E-09	17.181
SYN2	-1.8481	1.45641	-14.893	2.45E-08	1.18E-06	9.1761
TAFA4	-1.8432	0.97179	-33.082	7.37E-12	2.19E-09	17.8398
DLX3	-1.8327	1.04604	-16.13	1.10E-08	6.09E-07	10.0373
TLR8-AS1	-1.8287	1.01469	-27.187	5.53E-11	9.15E-09	15.717
ZC3H12C	-1.8183	1.59754	-27.124	5.66E-11	9.26E-09	15.692
PDGFC	-1.7999	2.3988	-42.717	5.29E-13	3.65E-10	20.5565

PPARG	-1.793	0.99685	-24.205	1.81E-10	2.33E-08	14.4529
SYT17	-1.7833	1.03749	-17.903	3.87E-09	2.59E-07	11.1674
GALNT17	-1.7679	0.88395	-17.991	3.69E-09	2.51E-07	11.2205
HOXB13	-1.7668	0.93358	-15.686	1.46E-08	7.77E-07	9.73573
TRIM58	-1.7609	0.93062	-19.309	1.81E-09	1.39E-07	11.9897
SSTR5-A	-1.7602	0.88701	-14.605	2.97E-08	1.39E-06	8.96663
ALDH1A3	-1.7456	1.46258	-19.371	1.75E-09	1.35E-07	12.0249
FGFR2	-1.7367	0.91852	-29.748	2.20E-11	4.64E-09	16.6935
NMU	-1.7315	1.42619	-14.458	3.29E-08	1.52E-06	8.85785
MOCOS	-1.7272	1.89736	-15.639	1.50E-08	7.96E-07	9.70322
PAPPA	-1.7268	2.2967	-24.786	1.42E-10	1.94E-08	14.7112
CFAP47	-1.7231	1.15793	-14.069	4.31E-08	1.89E-06	8.56529
TMEFF2	-1.7204	1.30706	-15.514	1.63E-08	8.46E-07	9.61633
PRDM16	-1.7113	1.16523	-12.568	1.31E-07	4.84E-06	7.3626
PAPPA-A	-1.7072	1.79902	-21.75	5.40E-10	5.29E-08	13.2868
BARX1	-1.697	1.17887	-10.428	7.96E-07	2.32E-05	5.4079
ECEL1	-1.6855	1.0918	-14.359	3.52E-08	1.60E-06	8.78431
TTC39C	-1.6819	1.31204	-13.291	7.56E-08	3.06E-06	7.95718
GLRB	-1.678	1.19873	-14.455	3.30E-08	1.52E-06	8.85516
NPTX2	-1.673	1.37052	-12.902	1.01E-07	3.93E-06	7.64105
MIRLET7	-1.6547	1.04703	-11.836	2.35E-07	8.10E-06	6.72905
EGR2	-1.6504	1.5534	-18.54	2.72E-09	1.97E-07	11.5478
ALDH1A3	-1.6501	1.31446	-19.814	1.39E-09	1.11E-07	12.2708
ADCY8	-1.6499	0.87514	-23.565	2.39E-10	2.87E-08	14.1608
LSAMP	-1.6441	1.54801	-27.563	4.80E-11	8.30E-09	15.8663
PRKCB	-1.6409	1.20954	-14.021	4.46E-08	1.95E-06	8.52876
CA13	-1.6397	1.85761	-17.367	5.26E-09	3.33E-07	10.8373
ADAMTS1	-1.635	0.86765	-16.555	8.51E-09	4.94E-07	10.3181
TSLP	-1.6305	0.86541	-24.744	1.45E-10	1.97E-08	14.6927
SPATA13	-1.623	1.58406	-22.389	4.02E-10	4.28E-08	13.6025
KIF26B	-1.621	1.98533	-14.015	4.48E-08	1.96E-06	8.52421
EOMES	-1.6076	2.39242	-31.381	1.27E-11	3.07E-09	17.2712
MMP3	-1.6036	3.8271	-43.342	4.55E-13	3.35E-10	20.7086
MAP3K21	-1.5846	0.84249	-24.052	1.94E-10	2.44E-08	14.3835
OLFM1	-1.5789	0.83964	-26.917	6.12E-11	9.77E-09	15.6087
SGCG	-1.5676	1.28082	-10.489	7.53E-07	2.21E-05	5.46842
DPP4	-1.565	0.83269	-21.149	7.18E-10	6.60E-08	12.9814
VEGFC	-1.5624	2.06104	-40.173	9.97E-13	6.00E-10	19.9105
STK32B	-1.561	1.06925	-13.72	5.52E-08	2.34E-06	8.29645
CFAP46	-1.5591	0.95941	-14.729	2.73E-08	1.29E-06	9.05725
FOXO1	-1.5576	1.16786	-17.101	6.15E-09	3.76E-07	10.6697
JAK1	-1.5513	3.06616	-27.671	4.62E-11	8.12E-09	15.9088
MLKL	-1.5269	1.69087	-14.122	4.15E-08	1.84E-06	8.60572
EPHA6	-1.5219	1.79241	-15.629	1.51E-08	8.00E-07	9.69587
COL11A1	-1.5135	3.5145	-26.198	8.08E-11	1.26E-08	15.3141
HMX1	-1.5045	0.80243	-8.4068	5.98E-06	0.00013	3.22706
DMBX1	-1.5016	0.87013	-11.38	3.44E-07	1.12E-05	6.31686
KANK1	-1.4915	2.00808	-19.165	1.95E-09	1.48E-07	11.9085
ADGRL3	-1.4887	0.92421	-16.937	6.77E-09	4.11E-07	10.5654
ANKRD33	-1.4878	1.21161	-15.646	1.50E-08	7.93E-07	9.70793
PCED1B-	-1.4749	1.8991	-16.638	8.09E-09	4.74E-07	10.3727
COL14A1	-1.4669	1.20205	-13.281	7.61E-08	3.08E-06	7.94966
TWIST2	-1.4633	1.14917	-8.8706	3.65E-06	8.64E-05	3.76136
RTN4RL2	-1.4547	1.94446	-7.1971	2.41E-05	0.00044	1.72372
SSH3	-1.4512	1.62936	-10.325	8.75E-07	2.51E-05	5.30567
DPYSL3	-1.4421	1.78974	-16.279	1.01E-08	5.66E-07	10.1366
MNX1	-1.4401	0.72005	-14.291	3.69E-08	1.67E-06	8.73274
GRIN2B	-1.44	1.73712	-17.174	5.89E-09	3.65E-07	10.7161

PPP2R2C	-1.4351	1.91141	-12.821	1.08E-07	4.13E-06	7.57465
HECA	-1.4342	1.88365	-24.414	1.66E-10	2.19E-08	14.5465
NKX2-5	-1.4295	1.29426	-8.6182	4.76E-06	0.00011	3.47336
FGF10	-1.4209	2.46239	-30.843	1.52E-11	3.49E-09	17.0843
SEMA3C	-1.4199	2.4768	-26.121	8.33E-11	1.28E-08	15.2824
LINC0120	-1.416	0.80835	-15.101	2.13E-08	1.05E-06	9.32531
EFCAB5	-1.4036	1.54424	-24.073	1.92E-10	2.43E-08	14.3932
PLPP3	-1.3992	0.74976	-21.617	5.74E-10	5.57E-08	13.22
MSX2	-1.3935	0.77629	-15.355	1.81E-08	9.19E-07	9.50532
FGF1	-1.3867	1.46595	-13.936	4.73E-08	2.05E-06	8.46372
HSD11B2	-1.3858	1.09313	-9.0362	3.07E-06	7.45E-05	3.94693
LRRC7	-1.3835	2.23133	-30.161	1.91E-11	4.10E-09	16.8425
OPN5	-1.3797	0.7902	-18.798	2.37E-09	1.75E-07	11.6978
KLHL29	-1.3774	1.6287	-21.043	7.55E-10	6.87E-08	12.9265
NRG1	-1.3756	1.20098	-17.324	5.39E-09	3.40E-07	10.8106
CCDC149	-1.3736	1.32209	-13.929	4.76E-08	2.06E-06	8.45788
TLR7	-1.3708	0.73559	-17.56	4.71E-09	3.07E-07	10.9577
DNAJC12	-1.3692	2.23672	-16.299	9.95E-09	5.62E-07	10.15
AGPAT3	-1.3678	1.85121	-13.162	8.32E-08	3.31E-06	7.85334
FGF10-AS	-1.362	1.43243	-11.561	2.95E-07	9.88E-06	6.48215
HPSE	-1.3535	1.46884	-11.055	4.55E-07	1.42E-05	6.0139
PCDHB17	-1.3514	0.77915	-15.674	1.47E-08	7.81E-07	9.72734
TNFSF14	-1.3497	0.87556	-11.876	2.27E-07	7.85E-06	6.76432
MMP12	-1.3463	1.76483	-25.02	1.29E-10	1.79E-08	14.8133
KAZN	-1.346	1.77639	-20.046	1.24E-09	1.00E-07	12.3976
FAM225A	-1.3369	1.28849	-11.857	2.31E-07	7.96E-06	6.74774
OSGIN1	-1.331	1.26937	-6.0249	0.00011	0.00161	0.09656
CCPG1	-1.3278	2.99945	-25.788	9.50E-11	1.41E-08	15.1424
FCF1P3	-1.3246	0.66232	-21.872	5.10E-10	5.08E-08	13.3479
PDGFA	-1.3202	1.7001	-17.121	6.07E-09	3.73E-07	10.6824
ICOSLG	-1.319	1.71344	-11.6	2.86E-07	9.60E-06	6.5173
SLC1A1	-1.3174	1.02696	-10.022	1.16E-06	3.21E-05	4.99874
ALPL	-1.3166	2.0541	-8.8589	3.69E-06	8.74E-05	3.74822
CPEB2	-1.3133	0.93686	-17.932	3.81E-09	2.57E-07	11.1853
CD226	-1.3126	1.21204	-18.043	3.58E-09	2.45E-07	11.2519
CACNB4	-1.3042	1.85762	-33.687	6.12E-12	1.99E-09	18.0344
WLS	-1.3039	2.08069	-22.295	4.19E-10	4.40E-08	13.5569
SLC24A4	-1.3031	1.90084	-18.141	3.39E-09	2.35E-07	11.3108
MAMLD1	-1.2979	1.24724	-14.407	3.41E-08	1.56E-06	8.81979
PTPRE	-1.296	1.0371	-11.311	3.65E-07	1.18E-05	6.25307
ARFGEF3	-1.2954	0.6799	-17.142	6.00E-09	3.71E-07	10.6959
MICAL2	-1.2853	1.86392	-15.531	1.61E-08	8.38E-07	9.62828
LAMC3	-1.2817	2.03292	-7.8058	1.17E-05	0.00024	2.5009
RNF144A	-1.2737	1.38361	-8.3207	6.57E-06	0.00014	3.12544
TOX2	-1.2736	0.63681	-12.561	1.32E-07	4.86E-06	7.35705
GABRQ	-1.27	0.73534	-11.37	3.47E-07	1.12E-05	6.30721
ZNF365	-1.2627	1.11875	-10.295	9.00E-07	2.58E-05	5.2751
TBC1D30	-1.2473	2.42762	-23.033	3.01E-10	3.42E-08	13.912
GS1-600G	-1.2473	0.75333	-14.138	4.10E-08	1.82E-06	8.61788
FAM225B	-1.2397	1.23778	-10.853	5.43E-07	1.65E-05	5.82236
KRT18	-1.2371	1.93454	-11.044	4.59E-07	1.43E-05	6.00395
ARRDC4	-1.231	1.03303	-11.153	4.18E-07	1.31E-05	6.10632
PIERCE2	-1.2301	1.56193	-17.122	6.07E-09	3.73E-07	10.6834
ARNT2	-1.2294	1.49118	-11.582	2.90E-07	9.73E-06	6.50074
KCNH3	-1.2262	1.74058	-6.648	4.80E-05	0.00079	0.98376
FRRS1L	-1.2178	0.78875	-11.299	3.68E-07	1.18E-05	6.24242
GLYATL2	-1.2172	1.67574	-16.677	7.91E-09	4.67E-07	10.3981
MNX1-AS	-1.2171	0.60853	-11.764	2.49E-07	8.50E-06	6.66457

ACSS1	-1.217	1.58322	-10.612	6.73E-07	2.00E-05	5.58932
KNDC1	-1.2143	0.65731	-9.5194	1.89E-06	4.87E-05	4.47351
DNAAF4-	-1.2108	2.98462	-23.971	2.00E-10	2.50E-08	14.3471
ATXN3L	-1.2096	0.65499	-19.945	1.30E-09	1.05E-07	12.3429
DEF6	-1.2091	1.86807	-8.6669	4.52E-06	0.0001	3.52941
ADGRE2	-1.206	1.60899	-10.063	1.12E-06	3.11E-05	5.04089
LPXN	-1.1942	2.25133	-20.259	1.11E-09	9.36E-08	12.5129
LURAP1L	-1.1895	2.55059	-17.958	3.76E-09	2.55E-07	11.2008
FOXF2	-1.183	0.5915	-56.382	3.01E-14	4.26E-11	23.3985
HSPA12A	-1.1732	0.58659	-52.966	5.74E-14	6.65E-11	22.7716
KITLG	-1.1721	3.03822	-29.721	2.22E-11	4.64E-09	16.6837
LDAF1	-1.1704	1.39329	-12.268	1.66E-07	6.00E-06	7.10674
TFAP2E	-1.1683	1.35524	-9.2526	2.46E-06	6.11E-05	4.18548
COMP	-1.1681	0.84342	-6.642	4.84E-05	0.00079	0.97543
CHD5	-1.1636	2.10132	-8.2271	7.28E-06	0.00016	3.01407
VGLL3	-1.1635	2.86778	-24.425	1.65E-10	2.19E-08	14.5515
OTUD1	-1.1587	0.57937	-14.479	3.24E-08	1.50E-06	8.87333
MAPT	-1.1579	1.55716	-8.6696	4.51E-06	0.0001	3.53254
CAMK2N1	-1.1495	0.90513	-11.63	2.79E-07	9.38E-06	6.54413
NOTUM	-1.1492	1.15118	-6.2263	8.35E-05	0.00127	0.38897
ROR1	-1.1485	0.67459	-15.297	1.88E-08	9.47E-07	9.46466
EPHB2	-1.1421	1.58433	-8.2796	6.87E-06	0.00015	3.07667
SATB2	-1.1382	1.06968	-10.911	5.16E-07	1.58E-05	5.87716
TFCP2L1	-1.1376	1.54284	-15.845	1.32E-08	7.14E-07	9.84404
SH3RF3	-1.1353	1.48539	-14.384	3.46E-08	1.58E-06	8.80299
ACE	-1.1352	0.87718	-8.2459	7.13E-06	0.00015	3.03658
CCDC3	-1.1347	1.61954	-13.743	5.43E-08	2.31E-06	8.3145
IQANK1	-1.1333	0.80022	-11.215	3.96E-07	1.25E-05	6.16403
SOX7	-1.1253	1.87092	-14.492	3.21E-08	1.49E-06	8.88304
RHCG	-1.124	0.66233	-13.328	7.35E-08	3.00E-06	7.98696
STOX2	-1.1218	2.12163	-16.52	8.69E-09	5.03E-07	10.2956
MIR9-3HG	-1.1197	1.23062	-10.232	9.54E-07	2.71E-05	5.21179
COBL	-1.1174	0.84745	-9.92	1.28E-06	3.50E-05	4.89415
SFXN3	-1.1172	1.86701	-11.939	2.16E-07	7.52E-06	6.82069
DENND11	-1.1148	1.83655	-22.222	4.34E-10	4.51E-08	13.5209
CACHD1	-1.111	2.25321	-22.535	3.76E-10	4.05E-08	13.6736
SLIT1	-1.1095	1.02243	-7.2095	2.37E-05	0.00044	1.74
PMEPA1	-1.1094	0.55471	-21.841	5.17E-10	5.14E-08	13.3327
PKIB	-1.1085	1.18097	-21.041	7.56E-10	6.87E-08	12.9258
NECTIN1	-1.1072	0.98431	-12.328	1.58E-07	5.77E-06	7.15882
VDR	-1.1067	2.07133	-12.794	1.10E-07	4.19E-06	7.55147
PTCHD4	-1.1063	1.42797	-22.117	4.55E-10	4.67E-08	13.4692
MAFA	-1.1032	1.0325	-6.9314	3.35E-05	0.00058	1.37036
LINC0236	-1.1031	0.60175	-6.2483	8.11E-05	0.00124	0.42061
NAV1	-1.0988	2.45395	-13.601	6.02E-08	2.53E-06	8.20334
ZNF804A	-1.0941	1.33839	-16.699	7.80E-09	4.61E-07	10.4123
AKR1C2	-1.0914	1.56459	-17.242	5.66E-09	3.54E-07	10.7588
ARHGAP2	-1.0895	1.46436	-10.577	6.95E-07	2.06E-05	5.55457
FNDC4	-1.0881	1.29842	-11.401	3.38E-07	1.10E-05	6.33603
PSD2	-1.0875	0.54376	-10.712	6.16E-07	1.85E-05	5.68612
DEPTOR	-1.0845	0.82245	-10.468	7.67E-07	2.25E-05	5.44761
LINC0273	-1.082	0.541	-27.601	4.74E-11	8.29E-09	15.8811
KCNQ3	-1.0791	0.63987	-11.943	2.15E-07	7.51E-06	6.82384
RIN3	-1.079	1.2361	-7.0899	2.75E-05	0.00049	1.58216
TSHZ3	-1.0783	0.5893	-20.582	9.46E-10	8.20E-08	12.685
TNFRSF1	-1.0775	3.22797	-20.485	9.92E-10	8.55E-08	12.6335
ST3GAL1	-1.0773	0.639	-14.866	2.49E-08	1.20E-06	9.15652
HOPX	-1.0758	0.71778	-11.327	3.60E-07	1.16E-05	6.26782

CDHR1	-1.0753	1.00626	-15.092	2.15E-08	1.06E-06	9.31942
LGI3	-1.0751	0.58772	-7.8614	1.10E-05	0.00022	2.56985
ITGA6-AS	-1.0751	0.76758	-8.2776	6.89E-06	0.00015	3.07428
GPR176	-1.074	1.96863	-19.002	2.12E-09	1.58E-07	11.8154
C1QL4	-1.073	1.64951	-8.2454	7.14E-06	0.00015	3.03593
TMEM26	-1.0713	2.01497	-28.499	3.41E-11	6.64E-09	16.2286
CYP4F2	-1.0709	0.76547	-7.2142	2.36E-05	0.00043	1.74612
HNRNPA	-1.0654	0.62402	-14.611	2.96E-08	1.39E-06	8.9705
ZFYVE28	-1.0654	1.05704	-7.3187	2.08E-05	0.00039	1.88242
EPIC1	-1.0619	0.53096	-15.803	1.36E-08	7.31E-07	9.81565
GPR27	-1.0616	0.58098	-20.097	1.20E-09	9.93E-08	12.4252
EML6	-1.061	2.04176	-20.639	9.19E-10	8.04E-08	12.7154
MAPK15	-1.0598	1.41715	-5.0566	0.00044	0.00538	-1.3865
ASIP	-1.0577	1.03443	-8.3474	6.38E-06	0.00014	3.15704
PRDM6	-1.0543	0.75718	-9.1569	2.71E-06	6.67E-05	4.08056
TESC	-1.0508	1.32236	-7.4536	1.77E-05	0.00034	2.0566
SDK2	-1.05	1.57894	-5.346	0.00029	0.00372	-0.9298
ABCA15F	-1.0496	0.5248	-19.03	2.09E-09	1.57E-07	11.8313
PRPH	-1.0467	0.77376	-6.5442	5.49E-05	0.00088	0.83952
PARD3B	-1.0442	0.75212	-8.659	4.56E-06	0.0001	3.52035
TMCO4	-1.0436	1.55528	-8.9891	3.22E-06	7.75E-05	3.8945
LRRCC1	-1.0425	2.27381	-13.384	7.05E-08	2.89E-06	8.03186
KBTBD11	-1.0422	0.88082	-12.067	1.95E-07	6.89E-06	6.933
TBXAS1	-1.0409	0.52044	-13.274	7.65E-08	3.09E-06	7.94404
HLX	-1.0388	0.64911	-6.9726	3.18E-05	0.00056	1.42576
MYORG	-1.0312	2.01535	-13.946	4.70E-08	2.04E-06	8.47143
EEF1A2	-1.0295	2.66004	-5.5437	0.00022	0.00292	-0.6244
TRIB2	-1.0292	0.81097	-7.6913	1.34E-05	0.00027	2.35801
MYLIP	-1.0287	1.50364	-18.081	3.51E-09	2.41E-07	11.275
SMAGP	-1.0264	1.72916	-23.666	2.28E-10	2.78E-08	14.2075
SYCP2	-1.0263	2.27749	-14.977	2.32E-08	1.12E-06	9.23695
PLAC4	-1.0262	0.5131	-11.294	3.70E-07	1.19E-05	6.23731
ANKRD34	-1.0223	2.03711	-18.5	2.78E-09	2.00E-07	11.5238
CFAP20D	-1.0211	0.79075	-12.914	1.00E-07	3.90E-06	7.65114
RNU7-15	-1.021	0.59003	-11.889	2.25E-07	7.78E-06	6.77621
FGD4	-1.0188	0.6581	-8.9119	3.49E-06	8.31E-05	3.80787
HYLS1	-1.0184	1.60878	-14.719	2.75E-08	1.30E-06	9.0499
PRDM16-	-1.0177	0.50884	-8.2796	6.87E-06	0.00015	3.07661
AR	-1.0151	1.34337	-12.129	1.85E-07	6.61E-06	6.98651
MIR9-1H(	-1.012	0.97138	-8.0995	8.40E-06	0.00018	2.86078
PALM2AK	-1.0057	2.38077	-13.818	5.15E-08	2.20E-06	8.37223
CALN1	-1.0051	0.50255	-7.4788	1.72E-05	0.00033	2.0888
FOXE1	-1.0016	0.99021	-9.9507	1.24E-06	3.41E-05	4.92582
TRABD2E	-1.0014	0.70141	-10.467	7.68E-07	2.25E-05	5.44644
ARC	-1.0004	1.22817	-8.2361	7.21E-06	0.00016	3.02483
CITED2	-0.9996	2.5272	-9.7815	1.46E-06	3.92E-05	4.75028
CPT1A	-0.9985	2.54161	-14.998	2.28E-08	1.11E-06	9.25185
C3	-0.9974	1.52845	-10.056	1.12E-06	3.13E-05	5.03371
FAM81A	-0.9965	1.29816	-6.1164	9.69E-05	0.00145	0.23009
C1orf105	-0.9941	0.87758	-7.2068	2.38E-05	0.00044	1.73645
LRRC7-A	-0.9923	0.69682	-9.2674	2.43E-06	6.03E-05	4.20163
NPTX1	-0.9909	1.07201	-10.049	1.13E-06	3.15E-05	5.02683
ELAPOR2	-0.9888	0.61374	-13.275	7.65E-08	3.09E-06	7.94414
TMC3-AS	-0.9885	1.08401	-8.7618	4.09E-06	9.52E-05	3.63801
LINC0294	-0.9843	1.27677	-5.8914	0.00013	0.00189	-0.1003
EFCC1	-0.9842	0.89996	-5.8607	0.00014	0.00196	-0.1458
LMCD1	-0.9827	1.53636	-10.308	8.89E-07	2.55E-05	5.28853
PPIC-AS1	-0.98	0.49002	-6.965	3.21E-05	0.00056	1.41551

GUCY1A1	-0.9796	2.78766	-31.442	1.24E-11	3.05E-09	17.292
GNAT3	-0.9755	0.48775	-50.329	9.73E-14	9.43E-11	22.253
SYNE3	-0.9744	1.45602	-13.437	6.78E-08	2.81E-06	8.07396
MCOLN3	-0.9743	1.69758	-18.687	2.51E-09	1.84E-07	11.6334
LAMP3	-0.9723	1.3221	-10.823	5.57E-07	1.69E-05	5.79367
CYP4F33	-0.9719	1.93998	-11.702	2.63E-07	8.90E-06	6.60894
ENPP7P6	-0.9717	0.59982	-9.4602	2.00E-06	5.12E-05	4.41023
WTAPP1	-0.9697	2.53024	-17.503	4.86E-09	3.15E-07	10.9221
FERMT1	-0.9694	2.00857	-14.348	3.55E-08	1.61E-06	8.77584
TATDN2F	-0.968	0.61369	-8.7617	4.09E-06	9.52E-05	3.63788
JUN	-0.9656	1.03356	-8.4747	5.55E-06	0.00012	3.30671
AK4	-0.9654	2.65487	-20.339	1.07E-09	9.09E-08	12.556
DERL3	-0.9648	2.34797	-7.7176	1.30E-05	0.00026	2.391
CD70	-0.963	2.30552	-7.1652	2.51E-05	0.00046	1.68169
TAFA1	-0.961	0.4805	-9.1753	2.66E-06	6.57E-05	4.10083
LINC0063	-0.9548	0.62793	-10.371	8.39E-07	2.43E-05	5.35112
SLC22A3	-0.952	1.53041	-4.2002	0.00169	0.01635	-2.8019
GPD1	-0.9515	0.8968	-5.882	0.00013	0.00191	-0.1143
C6orf141	-0.9504	0.4752	-12.268	1.66E-07	6.00E-06	7.10673
C7orf31	-0.9492	1.83319	-15.3	1.87E-08	9.46E-07	9.46666
DOC2B	-0.9467	1.80745	-15.772	1.38E-08	7.43E-07	9.79478
TNFRSF2	-0.9449	2.21408	-15.08	2.16E-08	1.06E-06	9.31056
CYP2S1	-0.9433	1.36719	-4.8461	0.00061	0.00706	-1.726
CALB1	-0.9432	1.29982	-18.22	3.25E-09	2.27E-07	11.3584
IGFBP7-A	-0.9426	0.47132	-16.587	8.35E-09	4.86E-07	10.339
TMEM178	-0.9414	0.85977	-12.047	1.98E-07	6.97E-06	6.91543
IL1R1	-0.9377	1.81276	-24.401	1.67E-10	2.20E-08	14.5408
SGK3	-0.9358	1.79254	-12.919	9.99E-08	3.89E-06	7.65527
MCC	-0.9335	1.9658	-12.179	1.78E-07	6.38E-06	7.0303
LINC0300	-0.9326	0.63002	-12.766	1.12E-07	4.26E-06	7.52828
RNASE4	-0.9307	0.6452	-9.1217	2.81E-06	6.88E-05	4.04171
SNX18P3	-0.9264	1.61053	-18.184	3.31E-09	2.31E-07	11.3369
PLCG2	-0.9257	1.34989	-8.0974	8.42E-06	0.00018	2.85816
NKAIN1	-0.9254	1.58752	-6.7789	4.06E-05	0.00068	1.16362
ENTPD2	-0.9246	0.74251	-5.8367	0.00014	0.00202	-0.1816
RASSF2	-0.9239	2.13926	-12.234	1.70E-07	6.14E-06	7.07821
TSPAN9	-0.9236	2.60092	-8.9326	3.42E-06	8.16E-05	3.83123
RMDN2	-0.9222	2.51635	-21.18	7.07E-10	6.54E-08	12.9977
PODXL	-0.922	2.14551	-14.145	4.09E-08	1.82E-06	8.62289
KCNIP3	-0.9205	1.96754	-7.1969	2.41E-05	0.00044	1.72335
GPRC5A	-0.9192	0.81932	-7.0314	2.96E-05	0.00052	1.50438
ATP8B3	-0.9188	1.54549	-5.385	0.00027	0.00354	-0.8692
TTLL7	-0.9175	2.00445	-14.505	3.18E-08	1.48E-06	8.89239
TMEM51-	-0.9163	0.60865	-9.8632	1.35E-06	3.68E-05	4.83532
IMPG1	-0.916	0.63788	-7.3581	1.98E-05	0.00038	1.93355
SMPDL3A	-0.9154	0.9926	-6.3843	6.77E-05	0.00106	0.61452
BARX1-D	-0.9131	0.60706	-7.5909	1.50E-05	0.00029	2.23156
TPSG1	-0.9131	0.50671	-8.2909	6.79E-06	0.00015	3.09006
USP43	-0.9128	0.55676	-9.7343	1.53E-06	4.06E-05	4.70091
AHNAK	-0.9117	3.01138	-14.993	2.29E-08	1.11E-06	9.24861
GAS7	-0.9093	0.75101	-5.9582	0.00012	0.00174	-0.0015
SSH2	-0.9088	3.15164	-14.324	3.61E-08	1.63E-06	8.75809
SGMS2	-0.9079	2.13292	-17.887	3.91E-09	2.61E-07	11.1577
NEK10	-0.9043	1.42584	-15.674	1.47E-08	7.81E-07	9.72691
COL3A1	-0.9043	4.00698	-10.36	8.48E-07	2.45E-05	5.33998
LGI4	-0.9041	0.68206	-5.0833	0.00042	0.0052	-1.3439
BMAL2	-0.9032	2.45338	-23.734	2.22E-10	2.72E-08	14.2387
TRIM7	-0.9005	1.60571	-7.9109	1.04E-05	0.00021	2.63084

TSPAN13	-0.8965	2.72478	-17.507	4.85E-09	3.15E-07	10.9243
CA7	-0.8953	0.69852	-5.7848	0.00015	0.00216	-0.2592
SLC9A2	-0.8952	0.80734	-9.6764	1.62E-06	4.26E-05	4.64005
CCNO	-0.8946	0.49749	-6.5451	5.48E-05	0.00088	0.84073
GBX2	-0.8919	0.49611	-8.9278	3.44E-06	8.20E-05	3.82581
PHLDA2	-0.8914	1.08566	-4.7167	0.00074	0.00833	-1.9373
CD9	-0.8906	1.92592	-10.429	7.95E-07	2.32E-05	5.40873
FGD3	-0.8898	1.18182	-7.2927	2.15E-05	0.0004	1.84863
ECHDC2	-0.8897	2.19046	-11.991	2.07E-07	7.27E-06	6.86567
HOXC12	-0.8884	0.63095	-9.0158	3.14E-06	7.58E-05	3.92427
CPSF1P1	-0.8884	0.98173	-6.1086	9.79E-05	0.00146	0.21872
RPS3AP2	-0.8863	0.44315	-13.323	7.38E-08	3.00E-06	7.98292
SEMA3D	-0.8851	2.93072	-25.686	9.89E-11	1.45E-08	15.0997
CORO6	-0.8842	2.28293	-6.0608	0.0001	0.00154	0.14912
P2RX5	-0.8818	1.98882	-8.8232	3.83E-06	9.02E-05	3.70773
MMP16	-0.8813	2.31503	-25.805	9.43E-11	1.40E-08	15.1497
ITGA6	-0.8812	2.85134	-14.314	3.63E-08	1.64E-06	8.75055
LINC0093	-0.8804	0.9019	-7.6334	1.43E-05	0.00028	2.28526
ACSL1	-0.8801	2.90945	-24.79	1.42E-10	1.94E-08	14.7131
ITPR3	-0.8791	2.7204	-6.1217	9.62E-05	0.00144	0.23788
FDX1	-0.8784	2.93836	-20.456	1.01E-09	8.65E-08	12.6181
ITGB3	-0.8777	0.6689	-6.9881	3.12E-05	0.00055	1.44646
ENPP7P1	-0.8767	0.58283	-9.4982	1.93E-06	4.96E-05	4.45086
CBLN2	-0.8754	3.82899	-23.711	2.24E-10	2.74E-08	14.2282
CHMP4C	-0.8733	2.5876	-18.058	3.55E-09	2.44E-07	11.2609
PAG1	-0.8727	2.17001	-22.339	4.11E-10	4.36E-08	13.578
STAMBPL	-0.8722	1.66654	-20.227	1.13E-09	9.47E-08	12.4957
TMEM151	-0.8717	0.72461	-5.1192	0.0004	0.00496	-1.2868
OSTF1	-0.8711	1.7824	-11.206	3.99E-07	1.26E-05	6.15572
ALOX12B	-0.8691	0.53487	-8.4561	5.67E-06	0.00013	3.28497
CSRP1	-0.869	1.11527	-11.811	2.40E-07	8.22E-06	6.70695
NDRG1	-0.8664	0.74277	-7.7215	1.29E-05	0.00026	2.39585
FGD5	-0.8641	0.90065	-5.8583	0.00014	0.00197	-0.1494
ARHGAP2	-0.8626	1.74459	-13.217	7.98E-08	3.20E-06	7.89761
PRPH2	-0.8612	1.96314	-10.046	1.14E-06	3.15E-05	5.02338
LNMICC	-0.8606	2.54971	-7.5288	1.62E-05	0.00032	2.15264
OGFRL1	-0.8595	1.81317	-17.557	4.72E-09	3.07E-07	10.9555
FOXA3	-0.8582	1.19758	-4.5308	0.00099	0.01061	-2.2449
MFSD2A	-0.8573	2.13807	-11.665	2.71E-07	9.15E-06	6.5759
NPPC	-0.8552	0.93786	-6.5293	5.60E-05	0.0009	0.81866
MEOX1	-0.8546	2.07731	-8.0352	9.02E-06	0.00019	2.78282
CDH23	-0.8514	1.24345	-5.981	0.00012	0.0017	0.03206
ITPR2	-0.8496	2.25759	-13.151	8.39E-08	3.34E-06	7.84426
ULK4P1	-0.8492	0.71796	-14.558	3.07E-08	1.44E-06	8.93154
KCNH4	-0.8471	0.74924	-5.3439	0.00029	0.00373	-0.9332
ANG	-0.8466	0.60316	-9.2972	2.36E-06	5.88E-05	4.23409
ADORA1	-0.8454	1.62097	-7.5486	1.58E-05	0.00031	2.17785
MCOLN2	-0.8441	2.20179	-18.725	2.46E-09	1.81E-07	11.6553
H19	-0.8439	1.93063	-4.9715	0.0005	0.00599	-1.523
FABP5	-0.8439	3.50596	-15.537	1.61E-08	8.37E-07	9.63247
TLR8	-0.8436	0.47196	-7.7948	1.19E-05	0.00024	2.48727
NPAS1	-0.8421	2.04655	-4.7431	0.00071	0.00807	-1.894
TNFRSF1	-0.8408	0.4204	-12.988	9.48E-08	3.72E-06	7.71199
FABP5P7	-0.8402	3.05905	-16.167	1.08E-08	5.99E-07	10.062
USH1G	-0.8394	0.9593	-4.3606	0.0013	0.01324	-2.5301
IRAK4	-0.8382	1.74748	-10.989	4.82E-07	1.48E-05	5.9518
AKR1C1	-0.836	1.20851	-14.616	2.95E-08	1.39E-06	8.9742
ZYG11A	-0.8351	2.24206	-11.299	3.68E-07	1.18E-05	6.24231

P4HA2	-0.8349	2.684	-8.2033	7.48E-06	0.00016	2.98567
MYZAP	-0.8337	0.89305	-10.104	1.07E-06	3.01E-05	5.08276
DAZL	-0.8332	0.49611	-9.6409	1.68E-06	4.39E-05	4.60264
CTSZ	-0.8324	2.57801	-9.743	1.52E-06	4.03E-05	4.71008
HSD3B7	-0.8315	1.86129	-4.5229	0.00101	0.01072	-2.2579
HCAR3	-0.8286	0.48208	-7.3317	2.05E-05	0.00039	1.89928
SLC9A7	-0.828	2.27962	-10.248	9.39E-07	2.68E-05	5.22849
EPB41L4	-0.8278	2.19326	-16.198	1.06E-08	5.91E-07	10.0827
FRMPD1	-0.8276	0.59366	-7.8267	1.14E-05	0.00023	2.52686
RNF212	-0.8266	2.18857	-9.7133	1.56E-06	4.14E-05	4.67888
ANKRD29	-0.8259	0.56349	-8.6344	4.68E-06	0.00011	3.49206
OR2W3	-0.8242	0.41212	-13.637	5.87E-08	2.47E-06	8.23132
PTPN6	-0.8239	1.5793	-4.8756	0.00058	0.0068	-1.6779
FAM201A	-0.8239	0.94687	-5.2998	0.00031	0.00396	-1.002
KLHDC7B	-0.8237	0.59169	-5.4294	0.00025	0.00336	-0.8003
TMEM26-	-0.8229	0.97836	-5.5491	0.00021	0.0029	-0.6161
HTR1F	-0.8221	2.14857	-23.475	2.48E-10	2.94E-08	14.119
PLD1	-0.8213	1.07528	-6.8532	3.69E-05	0.00063	1.26475
RET	-0.82	1.4207	-6.3742	6.86E-05	0.00107	0.60024
SYPL2	-0.8198	1.63349	-14.042	4.39E-08	1.92E-06	8.54445
FABP5P1	-0.8183	1.91157	-11.483	3.15E-07	1.04E-05	6.41144
PFKP	-0.8167	2.79792	-6.7369	4.28E-05	0.00072	1.10604
VSTM4	-0.8164	1.10297	-5.0249	0.00046	0.0056	-1.4374
PRRT4	-0.8157	1.15047	-3.8707	0.0029	0.02555	-3.3685
CHRM4	-0.8148	0.60343	-5.7415	0.00016	0.00228	-0.3242
SLC27A2	-0.8146	2.08992	-16.635	8.11E-09	4.75E-07	10.3705
CDH15	-0.8142	0.78763	-5.4213	0.00026	0.00339	-0.8128
SNX16	-0.8137	3.1374	-18.167	3.34E-09	2.32E-07	11.3267
RTTN	-0.8122	3.22196	-15.674	1.47E-08	7.81E-07	9.72745
SNX29P1	-0.8117	0.42483	-6.6772	4.62E-05	0.00076	1.02406
ABCC8	-0.811	1.26558	-7.02	3.00E-05	0.00053	1.48917
NOG	-0.8088	0.50476	-10.446	7.83E-07	2.29E-05	5.4255
CHRFAM	-0.8088	0.93013	-9.4287	2.06E-06	5.25E-05	4.37636
LINC0188	-0.8087	0.88665	-13.223	7.95E-08	3.19E-06	7.90265
SGCA	-0.8081	1.326	-4.3114	0.00141	0.01415	-2.6131
GLYATL1	-0.8077	1.72412	-17.805	4.09E-09	2.72E-07	11.1081
EPHA10	-0.8068	1.4192	-6.6182	4.99E-05	0.00081	0.94249
SACS-AS	-0.8066	1.0016	-7.1599	2.52E-05	0.00046	1.6748
AK4P1	-0.8052	1.57815	-11.489	3.14E-07	1.04E-05	6.41613
INKA1	-0.805	1.04891	-4.0351	0.00221	0.02043	-3.0845
HPCAL4	-0.8046	1.77647	-11.37	3.47E-07	1.12E-05	6.30756
KIF5C	-0.8031	1.60615	-11.486	3.14E-07	1.04E-05	6.41391
TMEM64	-0.8022	2.86661	-17.369	5.26E-09	3.33E-07	10.8385
SLC4A11	-0.8021	2.41487	-6.4899	5.89E-05	0.00094	0.76355
AGTR1	-0.8017	1.29181	-8.3573	6.31E-06	0.00014	3.16877
LINC0202	-0.8005	0.40023	-17.127	6.05E-09	3.73E-07	10.6864
LARGE2	-0.8001	1.97995	-4.0032	0.00233	0.02136	-3.1394
ADGRD2	-0.7997	0.47937	-6.3111	7.46E-05	0.00115	0.51051
FABP5P1	-0.7992	1.25413	-13.323	7.38E-08	3.00E-06	7.98288
APOBEC3	-0.7991	1.9748	-18.514	2.76E-09	1.99E-07	11.5321
MAP3K5	-0.7965	1.91233	-17.915	3.85E-09	2.59E-07	11.1746
RAPGEF4	-0.7958	1.40572	-10.95	4.98E-07	1.53E-05	5.91486
SYT7	-0.795	1.4896	-7.8936	1.06E-05	0.00022	2.6095
CLDN11	-0.7939	1.56729	-7.2303	2.32E-05	0.00043	1.76714
SORBS1	-0.7931	1.14756	-6.089	0.0001	0.00149	0.1902
LINC0139	-0.7916	1.19583	-6.9924	3.10E-05	0.00055	1.45229
CA12	-0.7909	2.78349	-9.625	1.70E-06	4.45E-05	4.58578
P4HA2-AS	-0.7907	1.67386	-7.9457	9.99E-06	0.00021	2.67348

ALDH3B1	-0.7899	1.30781	-5.2134	0.00035	0.0044	-1.1377
FBN1	-0.7897	2.75819	-13.587	6.08E-08	2.56E-06	8.19233
CNTF	-0.7887	2.0972	-15.946	1.24E-08	6.77E-07	9.9131
GLYATL1	-0.7887	0.44454	-9.7015	1.58E-06	4.17E-05	4.66646
SNAI1	-0.7873	1.19279	-4.5218	0.00101	0.01073	-2.2598
ARHGEF3	-0.7859	1.76286	-9.7895	1.45E-06	3.90E-05	4.75867
LINC0101	-0.7856	0.47234	-6.7685	4.11E-05	0.00069	1.14942
SNHG26	-0.7834	1.90496	-15.172	2.04E-08	1.01E-06	9.37579
SHB	-0.7832	1.75861	-8.7847	3.99E-06	9.34E-05	3.66404
LGI2	-0.7828	0.98379	-5.0416	0.00045	0.00549	-1.4107
KCNK6	-0.7816	1.74799	-9.0676	2.97E-06	7.24E-05	3.98183
PMP22	-0.7809	1.86931	-11.308	3.66E-07	1.18E-05	6.25024
INHBA-AS	-0.7795	0.38974	-13.322	7.38E-08	3.00E-06	7.98214
DAPK2	-0.7786	0.8899	-5.4133	0.00026	0.00342	-0.8253
KDELR3	-0.7777	2.58828	-14.147	4.08E-08	1.82E-06	8.62428
GLYATL1	-0.7772	0.48896	-8.241	7.17E-06	0.00015	3.03066
DLX4	-0.7763	1.04425	-6.9046	3.46E-05	0.0006	1.33426
DOCK5	-0.7742	2.37294	-13.335	7.31E-08	2.99E-06	7.9925
LINC0064	-0.7741	0.48738	-7.7223	1.29E-05	0.00026	2.39687
AK4P3	-0.7739	1.38184	-11.556	2.96E-07	9.91E-06	6.47725
PDGFA-D	-0.7734	0.43686	-5.4835	0.00024	0.00315	-0.7168
CFTR	-0.7729	0.68281	-6.1686	9.03E-05	0.00136	0.30579
SMOC1	-0.772	0.64537	-6.5088	5.75E-05	0.00092	0.79001
ZNF703	-0.7715	1.0753	-4.656	0.00082	0.00902	-2.0372
ATP2A3	-0.7714	1.56011	-3.8478	0.00301	0.02636	-3.4082
KIAA0319	-0.7708	1.15036	-6.1896	8.78E-05	0.00133	0.33606
RCN3	-0.7698	1.62536	-4.3828	0.00126	0.01284	-2.4927
ZDHHC23	-0.7691	2.86582	-17.321	5.40E-09	3.40E-07	10.8089
DOK6	-0.7689	1.72223	-8.3918	6.08E-06	0.00013	3.20946
FABP5P2	-0.7686	1.69676	-13.307	7.47E-08	3.03E-06	7.97006
GATA2	-0.7676	2.78646	-7.242	2.28E-05	0.00042	1.7825
SH2D3A	-0.7675	1.05252	-4.5237	0.001	0.01071	-2.2566
LINC0208	-0.7674	1.12785	-8.6398	4.65E-06	0.00011	3.49827
APOBEC3	-0.7662	2.66244	-11.72	2.59E-07	8.78E-06	6.62536
OTOP2	-0.7658	0.43305	-6.7854	4.02E-05	0.00068	1.17248
TRABD2A	-0.7655	1.13715	-8.3819	6.14E-06	0.00014	3.19774
PTGES	-0.7655	1.96009	-5.6091	0.0002	0.00268	-0.5245
CAMK2B	-0.7652	1.17192	-6.1013	9.89E-05	0.00147	0.2082
MSRA	-0.7648	1.83102	-7.9436	1.00E-05	0.00021	2.67093
ANKRD18	-0.7643	2.03731	-11.956	2.13E-07	7.45E-06	6.83516
LAMA3	-0.7631	0.51126	-7.0316	2.96E-05	0.00052	1.50464
C1QTNF1	-0.7608	0.48075	-6.9978	3.08E-05	0.00054	1.45947
IGFBP4	-0.7602	2.17363	-6.2441	8.16E-05	0.00125	0.41451
DKK2	-0.759	2.49627	-20.607	9.34E-10	8.13E-08	12.6985
APOBEC3	-0.7576	1.8944	-14.329	3.59E-08	1.63E-06	8.76179
ZNF704	-0.7571	2.40365	-15.973	1.22E-08	6.67E-07	9.93094
NFIB	-0.7568	3.3074	-17.114	6.10E-09	3.74E-07	10.6781
NKX3-1	-0.7559	0.99971	-6.6319	4.90E-05	0.0008	0.96149
JAM2	-0.7552	1.25486	-7.2808	2.18E-05	0.00041	1.8332
CMPK2	-0.7549	0.8942	-7.0886	2.75E-05	0.00049	1.5805
GDPD5	-0.7546	0.99483	-5.2757	0.00032	0.00408	-1.0397
CYP26B1	-0.7546	1.48825	-6.5022	5.80E-05	0.00092	0.78079
IFNLR1	-0.7527	1.56687	-6.0524	0.00011	0.00156	0.13688
FDX1P1	-0.7522	1.49889	-14.255	3.78E-08	1.70E-06	8.70598
TMX4	-0.7521	2.75601	-20.662	9.09E-10	7.99E-08	12.7276
DENND1B	-0.752	1.84495	-25.008	1.30E-10	1.79E-08	14.8084
SYT12	-0.7495	1.25225	-5.6071	0.0002	0.00269	-0.5277
LURAP1L	-0.7479	0.94085	-5.48	0.00024	0.00316	-0.7222

TRNP1	-0.7478	1.59633	-13.704	5.59E-08	2.36E-06	8.28376
ANTXR1	-0.7472	2.52372	-10.389	8.25E-07	2.39E-05	5.36954
ULK4P2	-0.747	0.73239	-9.1033	2.87E-06	7.00E-05	4.0214
AHR	-0.7462	0.42327	-8.9679	3.30E-06	7.91E-05	3.87072
CTSH	-0.7457	1.88228	-12.257	1.67E-07	6.05E-06	7.09733
NKX2-3	-0.7449	0.37247	-6.9867	3.13E-05	0.00055	1.44455
SYTL2	-0.7444	2.06427	-16.368	9.53E-09	5.42E-07	10.1958
DENND5I	-0.7426	1.41726	-14.083	4.27E-08	1.88E-06	8.57614
WIF1	-0.7425	2.82292	-11.768	2.49E-07	8.48E-06	6.66815
PRKAG2-	-0.7417	0.65108	-6.2792	7.78E-05	0.0012	0.46485
C1QTNF9	-0.7402	0.58783	-12.529	1.35E-07	4.98E-06	7.32978
EPHA8	-0.7394	1.68232	-4.3854	0.00125	0.01279	-2.4882
KLF2	-0.7391	1.29676	-9.2852	2.38E-06	5.94E-05	4.22105
BATF3	-0.7388	1.14549	-5.4482	0.00025	0.00329	-0.7713
TPBGL	-0.7378	0.54878	-7.8009	1.18E-05	0.00024	2.49486
TMC3	-0.7352	0.894	-6.7245	4.35E-05	0.00073	1.08911
HTRA4	-0.7351	0.57676	-6.654	4.76E-05	0.00078	0.99201
ARL4A	-0.7348	1.99766	-23.41	2.55E-10	2.98E-08	14.089
STAT1	-0.7324	3.66481	15.3294	1.84E-08	9.31E-07	9.48733
FXYD5	-0.7321	2.32678	-5.9763	0.00012	0.00171	0.02524
GMPR	-0.7317	0.46621	-6.7999	3.95E-05	0.00067	1.19229
GNG12-A	-0.7315	0.67532	-5.7042	0.00017	0.00238	-0.3805
CTH	-0.731	2.17655	-10.41	8.09E-07	2.35E-05	5.39032
PHOSPH	-0.7301	0.91521	-3.5476	0.005	0.04023	-3.9326
SLC16A1	-0.7298	0.86192	-4.8927	0.00057	0.00666	-1.6502
CHRNA7	-0.7295	1.0508	-9.709	1.57E-06	4.15E-05	4.67433
ADGRG6	-0.7281	2.01745	-11.636	2.77E-07	9.35E-06	6.54976
TMEM98	-0.7263	1.86358	-12.869	1.04E-07	4.01E-06	7.61384
GSTM3P1	-0.7245	0.91299	-4.9603	0.00051	0.00608	-1.5411
NPAS2	-0.7244	1.93606	-8.7777	4.02E-06	9.39E-05	3.65607
TAGLN2	-0.7242	2.75605	-7.7436	1.26E-05	0.00025	2.42354
TMEM30A	-0.7235	1.07096	-10.252	9.36E-07	2.67E-05	5.23248
FBXL16	-0.7209	1.21947	-4.2268	0.00161	0.0158	-2.7566
GCAWKR	-0.7209	0.79555	-4.3664	0.00129	0.01313	-2.5203
FOXA1	-0.7207	1.23211	-7.1046	2.70E-05	0.00049	1.60161
PLAAT3	-0.7206	2.06987	-7.5537	1.57E-05	0.00031	2.18434
ULK4P3	-0.7189	0.60002	-13.855	5.01E-08	2.15E-06	8.40149
GALNT16	-0.7185	2.11312	-6.1881	8.79E-05	0.00133	0.33402
GSTM5P1	-0.7183	0.35916	-8.7919	3.96E-06	9.28E-05	3.67222
TMEM51	-0.7178	2.04689	-8.8013	3.92E-06	9.20E-05	3.68299
ATP8B4	-0.7169	0.91684	-5.2144	0.00035	0.00439	-1.1362
EGR1	-0.7167	2.41806	-7.1352	2.60E-05	0.00047	1.6421
TNFRSF8	-0.7162	0.52477	-4.5731	0.00093	0.01007	-2.1745
FHIP1A	-0.7157	1.04123	-6.6116	5.03E-05	0.00082	0.93332
CNIH3-AS	-0.7132	0.45692	-6.3127	7.44E-05	0.00115	0.51274
TUBB2A	-0.7127	2.28479	-5.5849	0.0002	0.00277	-0.5615
CLMN	-0.7125	1.78699	-7.8771	1.08E-05	0.00022	2.58916
SHISA4	-0.7123	1.79434	-5.5187	0.00022	0.00301	-0.6628
SNORA2(	-0.7117	0.80568	-4.8752	0.00058	0.0068	-1.6787
KRT8	-0.7117	1.49273	-6.0504	0.00011	0.00156	0.13395
COL16A1	-0.709	0.77202	-3.6827	0.00397	0.03322	-3.6959
HYMAI	-0.708	0.6342	-6.9175	3.41E-05	0.00059	1.35166
TUBBP5	-0.7069	1.84324	-5.6173	0.00019	0.00265	-0.5121
IGSF9B	-0.7057	0.9881	-4.7037	0.00076	0.00847	-1.9587
GJA3	-0.7053	2.05211	-9.1827	2.64E-06	6.53E-05	4.1089
SNORD93	-0.7039	0.79118	-5.9942	0.00011	0.00167	0.05148
PRPS2	-0.7037	3.3145	-15.036	2.23E-08	1.09E-06	9.27881
DUSP9	-0.7025	1.8881	-5.0973	0.00041	0.00511	-1.3216

GASK1B	-0.7019	1.86982	-14.583	3.02E-08	1.41E-06	8.95004
SMCO2	-0.7001	0.50058	-6.687	4.56E-05	0.00076	1.03753
ZHX2	-0.6995	0.34977	-14.08	4.28E-08	1.88E-06	8.57335
DCLK2	-0.699	1.60447	-8.0252	9.13E-06	0.00019	2.77068
BHLHA15	-0.6974	0.77475	-3.9104	0.00271	0.02416	-3.2998
DTX4	-0.6972	2.74849	-11.524	3.04E-07	1.01E-05	6.44861
SLC30A3	-0.6969	0.94939	-5.148	0.00038	0.00479	-1.2412
LINC0061	-0.6966	0.3483	-12.781	1.11E-07	4.22E-06	7.54075
RAPGEF3	-0.6952	1.42815	-5.4623	0.00024	0.00323	-0.7496
TMEM86A	-0.695	0.67786	-11.623	2.80E-07	9.43E-06	6.53786
TMSB4XP	-0.6947	1.10947	-8.5532	5.10E-06	0.00012	3.39815
TP73	-0.6929	1.85418	-5.3681	0.00028	0.00361	-0.8954
LINC0264	-0.6919	0.34596	-8.9836	3.24E-06	7.79E-05	3.88831
UNC93B1	-0.6918	1.72358	-3.7903	0.00332	0.02854	-3.5081
C1R	-0.6911	1.61595	-7.6026	1.48E-05	0.00029	2.24626
IL6R	-0.6907	1.72245	-11.457	3.22E-07	1.06E-05	6.3877
SPATA3-A	-0.6904	1.20571	-7.6914	1.34E-05	0.00027	2.35819
ANKRD13	-0.6899	2.70827	-4.7117	0.00075	0.00839	-1.9455
PMAIP1	-0.6896	1.7456	-12.27	1.65E-07	6.00E-06	7.10915
DNAAF11	-0.6894	0.7414	-9.5686	1.80E-06	4.66E-05	4.52603
HERC5	-0.6894	1.87833	-8.8714	3.64E-06	8.64E-05	3.76231
TMEM38A	-0.6893	2.1632	-9.7997	1.44E-06	3.87E-05	4.76935
POU6F1	-0.689	1.89753	-7.2861	2.16E-05	0.0004	1.84012
CSPG5	-0.6887	2.04535	-6.7912	4.00E-05	0.00068	1.18035
LINC0099	-0.6881	0.34403	-7.0719	2.81E-05	0.0005	1.55834
HIPK2	-0.6865	1.97637	-8.8204	3.84E-06	9.04E-05	3.70459
CARMIL1	-0.6864	2.2061	-10.302	8.93E-07	2.56E-05	5.28291
SIRT1	-0.686	3.30713	-14.832	2.55E-08	1.22E-06	9.13187
BMP7	-0.6856	2.38579	-6.6191	4.98E-05	0.00081	0.94374
BMP2	-0.6854	0.44306	-6.1584	9.15E-05	0.00138	0.29101
EGR3	-0.6848	1.43499	-6.9129	3.43E-05	0.00059	1.34544
NPFFR2	-0.6839	0.85979	-4.4061	0.00121	0.01244	-2.4535
CHST6	-0.6832	2.32989	-9.61	1.73E-06	4.51E-05	4.56991
SLC35D3	-0.6804	0.39039	-10.592	6.86E-07	2.03E-05	5.56956
TIMP1	-0.6795	3.41015	-7.7147	1.30E-05	0.00026	2.38738
MRAP2	-0.6787	1.91403	-8.9235	3.45E-06	8.22E-05	3.821
ABHD15	-0.6786	2.79187	-8.5128	5.33E-06	0.00012	3.3511
C1QTNF9	-0.6782	1.53038	-11.25	3.84E-07	1.22E-05	6.19672
MBOAT1	-0.6776	1.44647	-7.6431	1.42E-05	0.00028	2.29742
TXNIP	-0.6771	1.99456	-12.193	1.76E-07	6.33E-06	7.04266
AHCY	-0.676	3.88202	-6.8625	3.65E-05	0.00063	1.2773
CHRD	-0.6758	0.56792	-4.2079	0.00167	0.01619	-2.7888
FABP5P9	-0.6749	0.76935	-11.999	2.06E-07	7.23E-06	6.87346
SRRM3	-0.6743	1.37995	-6.1716	8.99E-05	0.00136	0.31014
FOXD2	-0.6705	0.8455	-4.4375	0.00115	0.01197	-2.4008
KIF7	-0.6699	2.53046	-4.8214	0.00063	0.00727	-1.766
RIPK2	-0.6697	3.14626	-16.193	1.06E-08	5.92E-07	10.0789
PCDHB10	-0.6678	0.69853	-5.1599	0.00038	0.00471	-1.2223
MAMSTR	-0.6678	1.64933	-5.7359	0.00016	0.00229	-0.3326
ANO1	-0.6672	0.33362	-12.438	1.45E-07	5.32E-06	7.25236
MIR124-1	-0.6672	0.33362	-12.438	1.45E-07	5.32E-06	7.25236
MIR6826	-0.6672	0.50029	-7.7649	1.23E-05	0.00025	2.45005
ABLIM1	-0.665	2.76954	-9.6498	1.66E-06	4.36E-05	4.61197
ARHGAP3	-0.6649	1.3263	-6.6525	4.77E-05	0.00078	0.98996
ESAM	-0.6645	1.14033	-5.8884	0.00013	0.0019	-0.1047
UBE2Q2P	-0.6632	0.48497	-8.3908	6.08E-06	0.00013	3.20829
A4GALT	-0.6631	1.04543	-3.685	0.00396	0.03312	-3.6919
PPP1R16	-0.6627	0.79438	-5.6678	0.00018	0.00249	-0.4354

NFATC2	-0.6621	1.81532	-6.946	3.29E-05	0.00057	1.39002
BMAL2-A	-0.6619	0.87349	-9.8314	1.39E-06	3.77E-05	4.80236
PCOTH	-0.6618	1.63465	-10.285	9.08E-07	2.60E-05	5.26536
STEAP3-/	-0.6609	1.75611	-4.4787	0.00108	0.01136	-2.3318
AKR1B10	-0.6608	0.33038	-5.4013	0.00026	0.00347	-0.8438
GABBR2	-0.6601	1.10115	-7.8425	1.12E-05	0.00023	2.54643
PLIN5	-0.6599	1.45098	-5.1589	0.00038	0.00472	-1.2238
TMEM163	-0.6596	0.48032	-7.9947	9.45E-06	0.0002	2.73341
FRMD8	-0.6569	2.53346	-5.5039	0.00023	0.00307	-0.6855
MIR1291	-0.6566	0.6795	-4.797	0.00066	0.0075	-1.8059
RHOV	-0.6565	1.59499	-5.7716	0.00016	0.00219	-0.279
NIPAL2	-0.6547	1.598	-12.146	1.83E-07	6.53E-06	7.00178
LINC0035	-0.654	0.60719	-5.5837	0.0002	0.00277	-0.5633
ITPR1	-0.6536	2.51589	-8.7748	4.03E-06	9.41E-05	3.65278
NR2F2	-0.6536	1.94259	-6.4529	6.18E-05	0.00098	0.71144
STEAP3	-0.6534	2.66842	-6.2322	8.29E-05	0.00126	0.39744
KAT2B	-0.653	2.05411	-13.907	4.83E-08	2.08E-06	8.44085
FABP5P1	-0.6527	0.82974	-9.3579	2.22E-06	5.58E-05	4.29997
OR7E14F	-0.6518	0.50931	-4.5911	0.0009	0.00983	-2.1446
KRT18P4	-0.6511	0.32555	-8.1785	7.69E-06	0.00016	2.95594
ADAMTS:	-0.651	1.11687	-5.2169	0.00035	0.00438	-1.1321
PCDH10	-0.6501	3.35569	-12.016	2.03E-07	7.14E-06	6.88843
LRAT	-0.6498	0.37507	-5.9356	0.00012	0.00179	-0.0348
MMP13	-0.6493	0.92296	-4.6591	0.00081	0.00899	-2.0322
IGFBP6	-0.6493	1.8219	-4.7399	0.00072	0.0081	-1.8993
ERFL	-0.6492	0.79046	-3.9359	0.0026	0.02333	-3.2555
CFHR1	-0.6489	2.79221	-14.382	3.46E-08	1.58E-06	8.80101
NOS2	-0.6484	1.52283	-4.282	0.00148	0.0147	-2.6629
MOCS1	-0.6482	1.79534	-7.7921	1.19E-05	0.00024	2.48389
DOP1B	-0.6474	1.81675	-7.7927	1.19E-05	0.00024	2.48465
LINC0190	-0.6448	0.86847	-6.2747	7.83E-05	0.0012	0.45842
SMKR1	-0.6445	1.68393	-10.252	9.36E-07	2.67E-05	5.23248
TANK	-0.6437	2.94117	-16.844	7.15E-09	4.30E-07	10.5061
TYRP1	-0.6431	3.30166	-19.055	2.06E-09	1.56E-07	11.8456
CYBRD1	-0.6426	2.96513	-13.984	4.58E-08	1.99E-06	8.50014
CNRIP1	-0.6423	1.58985	-11.157	4.16E-07	1.31E-05	6.10966
ASIC5	-0.6414	0.32071	-6.3135	7.43E-05	0.00115	0.51391
ITPKB	-0.6393	2.33303	-4.7142	0.00074	0.00836	-1.9415
GAB3	-0.6383	0.92971	-5.512	0.00023	0.00304	-0.673
SH3RF3-/	-0.6379	0.36912	-3.8862	0.00283	0.02496	-3.3416
KIFC2	-0.6379	2.35791	-3.6614	0.00412	0.03419	-3.7333
SPINT2	-0.6374	1.05226	-3.5433	0.00503	0.04046	-3.9401
SRGAP1	-0.6373	2.1008	-9.1371	2.77E-06	6.79E-05	4.05872
MAN1C1	-0.637	1.33729	-6.2429	8.17E-05	0.00125	0.41278
CHST11	-0.6357	2.08915	-8.9836	3.24E-06	7.79E-05	3.88829
VIP	-0.6353	0.31763	-6.8974	3.49E-05	0.0006	1.32456
TRIM46	-0.6352	1.38306	-3.5608	0.00489	0.03955	-3.9095
LRRC73	-0.6328	1.42739	-4.7407	0.00071	0.00809	-1.898
SOWAHA	-0.6321	1.19042	-6.842	3.75E-05	0.00064	1.24946
SMYD3	-0.6315	2.68728	-11.911	2.21E-07	7.67E-06	6.79525
DAAM2	-0.6311	0.66137	-4.1175	0.00193	0.01825	-2.943
CABYR	-0.6303	1.96991	-8.0925	8.46E-06	0.00018	2.85224
COL23A1	-0.6288	1.89861	-3.7106	0.00379	0.03193	-3.6471
CCDC69	-0.6274	1.52048	-6.787	4.02E-05	0.00068	1.1746
NMRK1	-0.627	1.48116	-11.2	4.01E-07	1.26E-05	6.14988
LMX1B-D	-0.6267	0.36354	-10.388	8.26E-07	2.39E-05	5.36798
SEMA7A	-0.6249	1.52675	-4.3054	0.00142	0.01426	-2.6233
GPR137E	-0.6224	2.26308	-10.417	8.04E-07	2.34E-05	5.39723

ARRDC2	-0.6224	2.16789	-5.6756	0.00018	0.00246	-0.4236
MYO5A	-0.6221	2.65552	-13.461	6.67E-08	2.77E-06	8.09304
RHAG	-0.6221	0.31103	-8.0772	8.61E-06	0.00018	2.83377
ATOH8	-0.6217	0.31087	-6.8884	3.53E-05	0.00061	1.31236
DBNDD2	-0.6212	2.59728	-7.7861	1.20E-05	0.00024	2.47652
CELSR1F	-0.6209	1.21854	-5.7054	0.00017	0.00238	-0.3786
SLC41A2	-0.6204	1.88191	-10.993	4.80E-07	1.48E-05	5.95495
LIPA	-0.6187	4.03847	-16.654	8.02E-09	4.71E-07	10.383
C2orf88	-0.6186	1.88278	-10.228	9.57E-07	2.72E-05	5.20861
AKR1C3	-0.6176	1.73432	-10.886	5.27E-07	1.61E-05	5.8537
DLGAP2	-0.6171	0.40888	-6.9317	3.35E-05	0.00058	1.37082
BARX2	-0.6163	0.40665	-6.1048	9.84E-05	0.00146	0.21326
DCAF4	-0.6154	2.11735	-8.2359	7.21E-06	0.00016	3.0246
HS6ST2	-0.6153	2.5298	-9.8373	1.38E-06	3.75E-05	4.80849
KCTD12	-0.6143	0.50784	-6.4317	6.36E-05	0.001	0.68159
MEGF11	-0.6143	1.66363	-6.6762	4.63E-05	0.00076	1.02268
CASP10	-0.614	1.74771	-6.9481	3.28E-05	0.00057	1.39279
LINC0131	-0.6137	0.79627	-5.9672	0.00012	0.00172	0.01187
PRKG1-A	-0.6137	1.83491	-15.355	1.81E-08	9.19E-07	9.505
LINGO1	-0.6132	1.63338	-4.4417	0.00114	0.01191	-2.3937
MATK	-0.6131	2.26774	-3.558	0.00491	0.0397	-3.9144
ADAMTSI	-0.6126	2.04062	-3.8238	0.00314	0.02727	-3.4499
DENND2(	-0.6088	1.40854	-8.9012	3.53E-06	8.38E-05	3.79586
SEMA3G	-0.6079	1.5763	-3.507	0.00535	0.04251	-4.0041
PSLNR	-0.607	0.55526	-5.3898	0.00027	0.00352	-0.8617
SPINK1	-0.6059	0.30296	-12.64	1.24E-07	4.61E-06	7.42297
DUBR	-0.6058	2.23164	-11.726	2.57E-07	8.74E-06	6.63072
GASK1B-	-0.6055	1.23203	-6.1148	9.71E-05	0.00145	0.22778
SUGCT	-0.6053	2.00727	-9.3429	2.25E-06	5.66E-05	4.28369
FAM110A	-0.6033	1.45473	-4.6959	0.00077	0.00856	-1.9715
PLEKHA7	-0.6033	1.45519	-3.9902	0.00238	0.02174	-3.1619
GPRC5B	-0.6028	2.33182	-8.5687	5.02E-06	0.00011	3.41606
SH3BP5	-0.6027	2.33213	-12.411	1.48E-07	5.43E-06	7.22937
RAB27A	-0.6024	2.65309	-11.55	2.98E-07	9.93E-06	6.47249
DMKN	-0.602	2.57447	-6.9147	3.42E-05	0.00059	1.3478
STXBP2	-0.6003	2.09584	-4.0065	0.00232	0.02128	-3.1338
HTR7	-0.5985	0.5906	-5.4586	0.00024	0.00325	-0.7552
SYS1-DB	-0.5984	2.59801	-7.046	2.90E-05	0.00052	1.52382
ALDH1A1	-0.598	1.92715	-11.24	3.87E-07	1.23E-05	6.18761
MCIDAS	-0.5974	0.29869	-7.3415	2.02E-05	0.00038	1.91199
ADAMTSI	-0.5971	0.96581	-3.7101	0.0038	0.03195	-3.6481
PRNP	-0.5971	3.01283	-14.795	2.62E-08	1.25E-06	9.10511
SPATS2L	-0.597	1.35055	-8.7476	4.15E-06	9.65E-05	3.62186
VILL	-0.5969	0.78786	-3.4249	0.00616	0.04763	-4.1485
MCUB	-0.5961	2.51701	-11.817	2.39E-07	8.20E-06	6.71208
MAP3K7(	-0.5954	0.67823	-4.6937	0.00077	0.00857	-1.9752
MMP10	-0.5944	4.04201	-16	1.20E-08	6.57E-07	9.94943
SUGT1P1	-0.594	1.06536	-6.3089	7.48E-05	0.00116	0.50739
CTNNA3	-0.5938	1.51204	-13.711	5.56E-08	2.36E-06	8.28967
TMSB4XF	-0.5936	1.9148	-11.812	2.40E-07	8.22E-06	6.7077
TMSB4X	-0.593	4.3583	-12.912	1.00E-07	3.90E-06	7.6492
FAM111A	-0.5926	3.37713	-13.277	7.64E-08	3.09E-06	7.94594
CDC42EF	-0.5921	2.09913	-12.562	1.31E-07	4.86E-06	7.35764
YPEL2	-0.5906	1.54607	-6.6178	4.99E-05	0.00081	0.94192
PNPLA5	-0.5886	0.39465	-5.2184	0.00035	0.00437	-1.1299
METRNL	-0.588	1.42174	-3.8082	0.00322	0.02784	-3.4771
MIR27B	-0.5877	0.64392	-4.1937	0.0017	0.01648	-2.8129
ZCCHC12	-0.5877	0.42355	-4.6026	0.00089	0.00968	-2.1256

MYO7A	-0.5871	1.55145	-3.5994	0.00458	0.03739	-3.8418
C1D	-0.5859	2.83069	-11.69	2.65E-07	8.98E-06	6.59835
TTPA	-0.5857	1.51488	-9.0083	3.16E-06	7.62E-05	3.9159
ADAMTSI	-0.5853	1.56212	-7.0604	2.85E-05	0.00051	1.54295
EDA	-0.5845	1.94709	-7.2344	2.30E-05	0.00043	1.77261
FUT4	-0.5843	1.39733	-6.7417	4.26E-05	0.00071	1.11262
NEURL1	-0.5834	0.72241	-4.6664	0.0008	0.0089	-2.0201
MAP9	-0.5828	3.29538	-13.854	5.02E-08	2.15E-06	8.40011
TLE1	-0.5817	2.03796	-5.2187	0.00035	0.00437	-1.1293
ELL2	-0.5816	1.37408	-10.656	6.47E-07	1.94E-05	5.63181
SERINC5	-0.5807	2.49826	-11.513	3.07E-07	1.02E-05	6.43847
TP53INP2	-0.5802	2.3566	-7.2969	2.14E-05	0.0004	1.85412
EEPD1	-0.58	1.94062	-5.1717	0.00037	0.00465	-1.2035
SLC35E4	-0.5799	1.90815	-5.5029	0.00023	0.00307	-0.687
UBASH3E	-0.5788	2.21605	-9.6494	1.66E-06	4.36E-05	4.61158
PLXND1	-0.5777	2.39886	-4.2773	0.00149	0.01478	-2.6709
SEPTIN3	-0.5775	2.50252	-7.2062	2.38E-05	0.00044	1.73565
FAM222A	-0.5774	1.56391	-4.4183	0.00119	0.01223	-2.4331
FXYD6-A	-0.5772	0.3681	-4.6085	0.00088	0.0096	-2.1157
MEOX2	-0.5753	3.4465	-9.7538	1.50E-06	4.00E-05	4.72136
CNNM2	-0.5743	3.47255	-12.951	9.75E-08	3.81E-06	7.68108
C10orf10	-0.5734	0.38704	-5.069	0.00043	0.0053	-1.3668
ATP10A	-0.5732	1.86704	-7.2075	2.38E-05	0.00044	1.73726
LINC0135	-0.5731	0.33672	-8.0847	8.54E-06	0.00018	2.84283
CYP2U1-	-0.5728	0.95866	-4.7852	0.00067	0.00763	-1.8252
SPATA20	-0.5728	2.28982	-4.1551	0.00181	0.01738	-2.8788
MAP9-AS	-0.5724	2.43735	-12.673	1.21E-07	4.51E-06	7.45137
TUBB2BF	-0.5718	0.95629	-5.4392	0.00025	0.00332	-0.7852
IL27RA	-0.5708	2.57761	-6.2895	7.68E-05	0.00118	0.47964
TUBB8B	-0.5706	1.45224	-4.8999	0.00056	0.0066	-1.6386
ZFP91-CN	-0.5706	3.23379	-11.375	3.45E-07	1.12E-05	6.31227
HNRNPA	-0.5704	1.10478	-7.2959	2.14E-05	0.0004	1.85282
ZBTB41	-0.5691	2.91405	-13.413	6.91E-08	2.85E-06	8.05443
PROC	-0.5689	0.41416	-4.3026	0.00143	0.0143	-2.628
PUS7L	-0.5677	2.15221	-8.337	6.45E-06	0.00014	3.14474
ANKH	-0.5676	2.21419	-7.513	1.65E-05	0.00032	2.13248
ZFP91	-0.5675	3.75954	-12.833	1.07E-07	4.10E-06	7.58443
SLC22A1	-0.5669	0.52964	-4.2348	0.00159	0.01564	-2.7431
TNNC2	-0.5667	0.60054	-3.5299	0.00515	0.04124	-3.9638
PCDHB7	-0.5661	0.46774	-4.4551	0.00112	0.01172	-2.3713
PDK1-AS	-0.5657	1.29593	-9.6521	1.66E-06	4.36E-05	4.61447
PSD4	-0.5648	0.85398	-4.2528	0.00155	0.01528	-2.7125
CCDC113	-0.5643	2.33459	-7.9899	9.50E-06	0.0002	2.7276
PATE2	-0.5643	0.33231	-6.2362	8.24E-05	0.00126	0.40318
PXDC1	-0.564	1.55655	-7.3249	2.06E-05	0.00039	1.8905
LINC0219	-0.5638	0.83306	-7.627	1.44E-05	0.00028	2.27711
PRKCA	-0.5633	2.08607	-7.6345	1.43E-05	0.00028	2.28664
FAM78B	-0.5626	0.58235	-4.8489	0.0006	0.00704	-1.7213
ZNF654	-0.5615	2.8421	-18.651	2.56E-09	1.87E-07	11.6122
SH3RF1	-0.5608	1.88998	-7.1689	2.50E-05	0.00045	1.68658
KCNC4	-0.5607	1.82618	-7.8504	1.11E-05	0.00023	2.55623
DECR1	-0.5597	2.83732	-12.589	1.29E-07	4.77E-06	7.3805
CCDC148	-0.5596	1.3274	-8.9556	3.34E-06	7.99E-05	3.85696
SCN5A	-0.5595	1.30094	-4.4815	0.00107	0.01133	-2.3272
RPL23AP	-0.5594	0.85514	-6.7154	4.40E-05	0.00073	1.07663
FSTL3	-0.5592	2.2458	-4.4021	0.00122	0.01251	-2.4602
HSF2	-0.5589	3.03349	-12.649	1.23E-07	4.59E-06	7.43064
NT5C2	-0.5577	3.7124	-14.339	3.57E-08	1.62E-06	8.76883

ABCG4	-0.5568	1.30935	-4.5622	0.00094	0.01021	-2.1926
NIBAN1	-0.5564	2.32705	-9.4574	2.01E-06	5.13E-05	4.40717
UBE2Q2	-0.5563	2.64712	-9.2602	2.44E-06	6.07E-05	4.19382
ENPP7P1	-0.5561	0.42858	-4.7291	0.00073	0.00821	-1.917
B3GNT7	-0.5561	0.69648	-4.7742	0.00068	0.00773	-1.8432
IGSF11	-0.5557	0.32804	-9.6589	1.65E-06	4.33E-05	4.62165
RN7SL68	-0.5557	0.27787	-8.0227	9.15E-06	0.00019	2.76754
CLDN23	-0.5531	0.8455	-6.3099	7.47E-05	0.00116	0.50879
TMEM144	-0.5494	1.76331	-6.5269	5.61E-05	0.0009	0.81531
CADM2	-0.5489	2.08015	-8.5415	5.17E-06	0.00012	3.38448
DYNLT3	-0.5473	1.90555	-10.25	9.38E-07	2.68E-05	5.2302
PDE6A	-0.5471	0.56388	-5.1848	0.00036	0.00457	-1.1829
DUSP2	-0.5462	2.20755	-5.247	0.00033	0.00422	-1.0849
AOPEP	-0.5455	3.16621	-10.224	9.61E-07	2.73E-05	5.20383
PAQR5	-0.5452	1.75177	-5.4196	0.00026	0.0034	-0.8155
OSGIN2	-0.5451	3.31082	-12.675	1.20E-07	4.51E-06	7.45252
TMEM117	-0.5451	2.17384	-10.529	7.26E-07	2.14E-05	5.508
ENPP7P4	-0.5448	0.60277	-4.2742	0.0015	0.01484	-2.6761
TMSB4XP	-0.5444	2.98817	-13.185	8.17E-08	3.27E-06	7.87228
LPAL2	-0.5425	0.32144	-6.2979	7.59E-05	0.00117	0.49157
ADGRF2	-0.542	0.50103	-6.5363	5.54E-05	0.00089	0.82841
MTMR11	-0.5419	2.60127	-7.0422	2.92E-05	0.00052	1.51873
ANGPT1	-0.5419	2.24573	-9.7663	1.48E-06	3.96E-05	4.73449
TMEM30A	-0.5403	3.45276	-12.324	1.59E-07	5.79E-06	7.15516
SLC19A3	-0.5396	0.2698	-8.3678	6.24E-06	0.00014	3.18121
TENM2-A	-0.5396	0.31997	-4.6229	0.00086	0.00944	-2.092
ALCAM	-0.5394	3.33319	-12.968	9.62E-08	3.77E-06	7.69568
IL17RD	-0.5392	2.11422	-7.8894	1.06E-05	0.00022	2.6044
RIPPLY3	-0.538	1.50422	-5.7166	0.00017	0.00234	-0.3616
DLK2	-0.538	1.81564	-3.6082	0.00451	0.03695	-3.8264
EPHX2	-0.5376	1.38901	-5.2327	0.00034	0.0043	-1.1073
SERPINB	-0.5376	0.31895	-4.0956	0.002	0.01879	-2.9805
RELL1	-0.5368	1.56919	-5.476	0.00024	0.00318	-0.7285
ZMYND10	-0.5365	1.10363	-3.8261	0.00312	0.02717	-3.4459
LYSMD2	-0.5362	2.13344	-13.453	6.70E-08	2.79E-06	8.08686
SLC66A3	-0.536	2.34019	-11.979	2.09E-07	7.32E-06	6.85544
RGS20	-0.5359	1.88639	-12.614	1.26E-07	4.69E-06	7.40116
CREG2	-0.5349	0.31763	-5.2027	0.00035	0.00446	-1.1545
PAX1	-0.5349	0.3678	-5.2524	0.00033	0.0042	-1.0763
SLITRK5	-0.5349	0.51832	-7.483	1.71E-05	0.00033	2.09421
MB21D2	-0.534	0.3967	-4.5672	0.00094	0.01015	-2.1843
LEMD3	-0.5337	3.18165	-13.855	5.01E-08	2.15E-06	8.40149
FOXH1	-0.5334	2.06459	-3.7846	0.00335	0.02873	-3.5182
TLE2	-0.5328	2.18632	-3.4579	0.00582	0.04545	-4.0903
HSPA4L	-0.5326	3.18683	-10.374	8.37E-07	2.42E-05	5.35392
FAS	-0.5325	2.51493	-14.905	2.43E-08	1.17E-06	9.18531
CDO1	-0.5322	1.95558	-7.9611	9.81E-06	0.0002	2.69238
MTND5P1	-0.5311	0.26553	-10.748	5.96E-07	1.80E-05	5.72132
MTSS1	-0.531	1.84872	-7.9451	9.99E-06	0.00021	2.67281
MORC4	-0.5305	2.51454	-7.9034	1.05E-05	0.00022	2.62156
FAT4	-0.5299	1.61955	-8.5852	4.93E-06	0.00011	3.43523
NPVF	-0.5299	0.26496	-7.272	2.20E-05	0.00041	1.82176
RHBDL3	-0.5298	1.44345	-11.831	2.36E-07	8.12E-06	6.72482
PIWIL4-A	-0.5297	0.76192	-5.448	0.00025	0.00329	-0.7716
CDC25B	-0.5293	2.87533	-4.2238	0.00162	0.01586	-2.7616
FAM20C	-0.5287	2.07671	-4.2222	0.00163	0.0159	-2.7645
ABTB2	-0.5282	1.91802	-5.8552	0.00014	0.00198	-0.154
IGDCC4	-0.5277	2.15773	-6.3039	7.53E-05	0.00116	0.50018

KLF4	-0.5264	0.2632	-6.52	5.66E-05	0.00091	0.80567
RPRML	-0.5264	0.41371	-4.0107	0.0023	0.02115	-3.1264
OCA2	-0.5259	1.79856	-5.9973	0.00011	0.00167	0.05617
NDRG2	-0.5254	2.03104	-6.6518	4.77E-05	0.00078	0.98902
FBXO32	-0.5253	0.41314	-3.8931	0.00279	0.02472	-3.3296
TRAPPC6	-0.5235	2.89442	-10.69	6.28E-07	1.88E-05	5.66515
LINC0261	-0.5232	1.17497	-6.3622	6.97E-05	0.00109	0.58317
FXYD6	-0.5223	0.65783	-4.3911	0.00124	0.0127	-2.4788
MYLK2	-0.5222	0.74848	-3.9067	0.00273	0.02428	-3.3061
GLYATL1	-0.5213	1.62624	-5.6871	0.00018	0.00243	-0.4062
PCDHGB	-0.5201	0.5696	-4.6141	0.00087	0.00954	-2.1065
TMEM225	-0.5199	0.79398	-4.9373	0.00053	0.00627	-1.5782
SLC39A4	-0.5199	1.91482	-3.7328	0.00365	0.03093	-3.6085
TMSB4XP	-0.5196	1.33668	-8.9111	3.50E-06	8.31E-05	3.80702
CBFA2T3	-0.5188	1.22655	-4.0816	0.00205	0.01917	-3.0046
CNIH3	-0.5187	1.12999	-4.1628	0.00179	0.01721	-2.8656
SAT1	-0.5186	2.18305	-8.6389	4.66E-06	0.00011	3.49721
SNX29P2	-0.5181	0.30739	-5.4812	0.00024	0.00316	-0.7203
CSPG4P1	-0.5173	0.78132	-4.5942	0.0009	0.00979	-2.1395
EGILA	-0.5167	0.35871	-5.0557	0.00044	0.00538	-1.388
RNF157	-0.5161	2.33368	-4.9963	0.00048	0.00581	-1.4832
SNORA44	-0.5149	1.39527	-8.5275	5.25E-06	0.00012	3.36828
FXYD1	-0.5148	0.92966	-3.7632	0.00347	0.02961	-3.5555
ARL4AP5	-0.5138	0.68091	-9.6883	1.60E-06	4.22E-05	4.65266
PLAGL1	-0.5138	2.51511	-7.3676	1.96E-05	0.00037	1.94579
EFNA2	-0.5137	0.45752	-3.6695	0.00406	0.03383	-3.719
TMEM87B	-0.5136	2.67255	-9.7503	1.51E-06	4.01E-05	4.71768
PLIN1	-0.5132	0.30677	-3.8468	0.00302	0.02639	-3.41
SCD5	-0.5131	2.49265	-5.5026	0.00023	0.00307	-0.6875
ZFAND4	-0.5128	2.23553	-10.192	9.90E-07	2.80E-05	5.17207
NAAA	-0.5127	2.7839	-10.194	9.88E-07	2.80E-05	5.1738
SNORD1(	-0.5121	1.17225	-6.1745	8.96E-05	0.00135	0.31436
F11R	-0.5114	2.15107	-4.886	0.00057	0.00672	-1.6611
STOM	-0.5109	2.96631	-10.348	8.57E-07	2.47E-05	5.32795
RFTN1	-0.5109	1.90987	-8.7181	4.28E-06	9.93E-05	3.58807
LINC0222	-0.5102	0.30529	-9.172	2.67E-06	6.58E-05	4.0972
TFR2	-0.5099	2.33558	-4.1725	0.00176	0.01698	-2.8491
C1DP1	-0.5099	1.49302	-8.3066	6.67E-06	0.00015	3.10871
TCF15	-0.5093	1.3788	-8.2031	7.48E-06	0.00016	2.98543
EMID1	-0.509	1.53408	-3.4635	0.00577	0.0451	-4.0806
PRSS23	-0.5081	1.9795	-9.853	1.36E-06	3.70E-05	4.82478
KLF13	-0.5079	2.68293	-7.6701	1.37E-05	0.00027	2.33146
HEBP2	-0.5071	2.26229	-8.766	4.07E-06	9.48E-05	3.64283
IQCK	-0.5068	2.1878	-10.451	7.79E-07	2.28E-05	5.43107
NHSL1	-0.5058	1.50377	-5.4131	0.00026	0.00342	-0.8256
REEP2	-0.5057	2.51447	-3.4136	0.00628	0.04845	-4.1683
WNT7B	-0.5056	0.25279	-4.5865	0.00091	0.00989	-2.1523
KCNT2	-0.5053	1.89661	-10.581	6.93E-07	2.05E-05	5.55852
LARGE1	-0.5051	0.862	-4.2477	0.00156	0.01538	-2.7211
PHB1P19	-0.5039	0.57691	-3.5734	0.00478	0.03888	-3.8875
LRRC37A	-0.5036	2.07122	-8.283	6.85E-06	0.00015	3.08076
VSTM2L	-0.5032	0.85251	-3.4957	0.00546	0.04311	-4.0239
SEPTIN4	-0.503	0.86004	-4.0806	0.00205	0.01919	-3.0063
LIN7A	-0.5024	0.76028	-4.2979	0.00144	0.0144	-2.636
ABRACL	-0.5015	2.4707	-9.34	2.26E-06	5.67E-05	4.28057
NECTIN1	-0.5014	2.57696	-5.1262	0.0004	0.00492	-1.2757
ASZ1	-0.5	0.25	-4.3108	0.00141	0.01415	-2.6142
MEGF8	0.5003	2.19607	3.49846	0.00543	0.04296	-4.019

HMCN1	0.5009	0.77684	5.18151	0.00037	0.00459	-1.1881
STAT2	0.50093	2.61851	4.97607	0.0005	0.00596	-1.5157
HNRNPD	0.50172	0.25086	5.09313	0.00042	0.00513	-1.3283
SNORD1	0.5019	0.53116	4.13847	0.00186	0.01779	-2.9072
ANXA1	0.50204	2.26752	9.30496	2.34E-06	5.84E-05	4.24254
RALGPS2	0.50228	0.25114	7.29687	2.14E-05	0.0004	1.85409
BTBD8	0.5027	1.38971	5.63162	0.00019	0.0026	-0.4903
CNTNAP	0.50296	1.39843	8.30516	6.68E-06	0.00015	3.10703
EDARADI	0.50302	1.53823	4.45014	0.00113	0.01179	-2.3796
TRPV2	0.50314	0.85748	3.52937	0.00515	0.04126	-3.9647
CCDC144	0.50319	0.72859	6.60597	5.07E-05	0.00082	0.92548
USP12P1	0.50444	1.00133	6.69063	4.54E-05	0.00075	1.04252
SSC5D	0.50444	0.91184	3.76011	0.00349	0.02973	-3.5608
CACNA1I	0.50481	0.8111	5.45507	0.00024	0.00326	-0.7607
IFIH1	0.50482	1.80477	8.5915	4.90E-06	0.00011	3.44249
BBS10	0.50493	2.15433	9.42527	2.07E-06	5.26E-05	4.37267
CCDC18-	0.50526	1.97319	5.88295	0.00013	0.00191	-0.1128
CRYAB	0.50557	0.35313	6.43136	6.36E-05	0.001	0.68111
MYMX	0.50557	0.25279	4.58646	0.00091	0.00989	-2.1523
PPBP	0.50557	0.25279	6.35516	7.03E-05	0.0011	0.57321
VNN2	0.50557	0.25279	4.66867	0.0008	0.00888	-2.0164
DTX3L	0.50562	2.82506	8.59839	4.86E-06	0.00011	3.45046
SLC44A2	0.50635	2.3569	4.29184	0.00145	0.01451	-2.6462
TMED5	0.50708	2.90566	9.82606	1.40E-06	3.78E-05	4.79677
STC2	0.50717	2.83105	5.32533	0.0003	0.00383	-0.9621
LMNB1-D	0.50853	0.35461	6.68836	4.56E-05	0.00076	1.0394
LINC0044	0.50923	0.69386	4.45619	0.00112	0.0117	-2.3695
CPQ	0.50977	1.72376	7.44846	1.78E-05	0.00034	2.04996
BNIP3P2	0.51024	0.25512	10.6643	6.42E-07	1.92E-05	5.63984
CXCL6	0.51024	0.25512	6.76126	4.15E-05	0.0007	1.13946
LINC020	0.51024	0.25512	6.76126	4.15E-05	0.0007	1.13946
SLITRK1	0.51024	0.25512	4.8445	0.00061	0.00707	-1.7285
CCDC152	0.51108	1.5991	9.51318	1.90E-06	4.90E-05	4.46692
ABTB3	0.51136	0.58606	5.0637	0.00044	0.00533	-1.3753
SESN2	0.51164	2.64893	4.07896	0.00206	0.01924	-3.0091
EEF1AKN	0.51168	1.92661	16.6197	8.18E-09	4.77E-07	10.3606
LINC0111	0.51194	0.26917	4.29613	0.00144	0.01443	-2.639
FABP4	0.51206	0.59493	5.67671	0.00018	0.00246	-0.4219
TSPEAR-	0.5125	1.70717	4.025	0.00225	0.02072	-3.1019
REELD1	0.5132	0.30677	6.54216	5.50E-05	0.00089	0.83663
SHROOM	0.5132	0.2566	4.84331	0.00061	0.00707	-1.7304
TMEM17	0.5132	0.30677	5.24891	0.00033	0.00421	-1.0818
TNNT2	0.5132	0.2566	4.24241	0.00157	0.01548	-2.7301
SNN	0.51326	2.05357	6.0105	0.00011	0.00164	0.07548
NRN1	0.5141	0.25705	14.1622	4.04E-08	1.80E-06	8.63594
SIGLEC1	0.51447	0.37657	5.95996	0.00012	0.00174	0.00114
PPM1AP1	0.51469	0.5669	4.62713	0.00085	0.00938	-2.085
RHOU	0.51483	1.85701	9.70192	1.58E-06	4.17E-05	4.66693
MIR548XI	0.51566	0.27682	3.76527	0.00346	0.02951	-3.5518
LINC0297	0.51641	0.56449	4.25731	0.00154	0.01521	-2.7048
A2MP1	0.51673	0.40888	6.07087	0.0001	0.00152	0.16382
ARID3BP	0.51699	1.44837	3.84874	0.00301	0.02633	-3.4066
CLMP	0.51728	0.75569	5.30971	0.0003	0.0039	-0.9865
KIF13B	0.51769	0.98946	3.60153	0.00456	0.03726	-3.838
SERPINI1	0.51864	1.03543	5.08281	0.00042	0.0052	-1.3448
MYLK4	0.51877	0.38908	4.38996	0.00124	0.01272	-2.4806
HLA-C	0.51886	3.24144	4.64901	0.00082	0.0091	-2.0488
TMEM16	0.51887	1.56537	9.23934	2.50E-06	6.19E-05	4.171

BCHE	0.51914	1.4475	7.52649	1.62E-05	0.00032	2.14971
JDP2-AS1	0.51938	1.26526	16.6737	7.92E-09	4.67E-07	10.3958
PMCHL2	0.51968	0.31354	5.53943	0.00022	0.00293	-0.631
TLCD4-RW	0.52024	2.20374	11.2208	3.94E-07	1.25E-05	6.16936
SMARCA	0.52026	0.56116	5.29365	0.00031	0.00399	-1.0116
AGKP2	0.5214	0.3194	6.40366	6.60E-05	0.00104	0.64199
ANKRD17	0.5214	0.39039	4.69548	0.00077	0.00856	-1.9722
PTH2	0.5214	0.32702	5.26379	0.00032	0.00415	-1.0584
ARHGAP	0.52172	0.26086	8.49137	5.46E-06	0.00012	3.32616
CLTRN	0.52172	0.41138	4.09858	0.00199	0.01872	-2.9755
GALNTL6	0.52172	0.46155	4.35213	0.00132	0.01338	-2.5443
H3Y1	0.52172	0.26086	5.7345	0.00016	0.00229	-0.3347
GPD2	0.52174	2.92271	10.8671	5.36E-07	1.63E-05	5.83548
ZNF680	0.52252	2.24845	9.4164	2.09E-06	5.29E-05	4.36312
ARHGAP	0.52435	0.36252	5.46827	0.00024	0.00321	-0.7403
ST3GAL6	0.52454	0.91836	6.93204	3.35E-05	0.00058	1.37123
GREB1	0.52516	1.67822	4.66053	0.00081	0.00897	-2.0298
H2BC8	0.52552	0.90065	3.72468	0.0037	0.03129	-3.6226
CTTNBP2	0.52598	2.23148	8.5392	5.18E-06	0.00012	3.38186
LINC0245	0.52599	0.94286	10.2675	9.23E-07	2.64E-05	5.24787
LEKR1	0.52639	0.2632	9.43553	2.05E-06	5.23E-05	4.3837
LINC0109	0.52639	0.2632	9.43553	2.05E-06	5.23E-05	4.3837
USP26	0.52639	0.2632	4.73855	0.00072	0.00811	-1.9015
UBE2L6	0.52662	3.07211	8.45556	5.67E-06	0.00013	3.2843
SUGT1-D	0.52704	1.13257	5.68162	0.00018	0.00245	-0.4145
GEMIN8F	0.52719	0.97838	3.99986	0.00234	0.02146	-3.1452
SPATA17	0.5273	1.54659	6.22747	8.34E-05	0.00127	0.39068
ICAM4-AS	0.52735	1.03178	4.49613	0.00105	0.0111	-2.3027
VCL	0.52745	3.88936	10.522	7.31E-07	2.15E-05	5.50072
CNTNAP	0.5276	0.67485	5.71244	0.00017	0.00235	-0.368
ADGRE1	0.52808	1.11468	5.95451	0.00012	0.00175	-0.0069
CCDC144	0.52848	0.7457	6.21834	8.44E-05	0.00128	0.37754
GPC3	0.52957	2.51463	8.79661	3.94E-06	9.24E-05	3.67762
GPR149	0.52993	0.26496	7.27205	2.20E-05	0.00041	1.82176
MIR23C	0.52993	0.36531	6.06873	0.0001	0.00153	0.16069
TNS1	0.53007	0.77677	3.50554	0.00537	0.04258	-4.0065
MAPK8IP	0.53087	1.54366	3.43264	0.00608	0.04708	-4.1348
LINC0123	0.53103	2.02957	6.3745	6.86E-05	0.00107	0.60067
LINC0065	0.53105	0.74642	4.79969	0.00065	0.00748	-1.8015
LYG2	0.53215	0.64663	3.71429	0.00377	0.03179	-3.6407
OLFM3	0.53244	3.12159	14.7687	2.66E-08	1.27E-06	9.08615
ACVR2B	0.53251	2.4969	5.99823	0.00011	0.00167	0.05746
TWF2-DT	0.53262	0.76689	3.78609	0.00334	0.02868	-3.5155
OR7E7P	0.53268	0.73757	4.29377	0.00145	0.01447	-2.643
ANKRD7	0.53288	0.31661	5.68975	0.00018	0.00242	-0.4022
MTF2	0.53345	2.97579	10.644	6.54E-07	1.95E-05	5.62016
BCO1	0.53492	0.31763	7.43745	1.80E-05	0.00035	2.03583
STARD13	0.53551	0.49779	5.98541	0.00012	0.00169	0.03861
MGAT3-A	0.53625	0.46881	4.53249	0.00099	0.0106	-2.242
PELI1	0.53657	2.4115	9.84709	1.37E-06	3.72E-05	4.81865
CYB5AP5	0.53697	0.34801	5.32411	0.0003	0.00383	-0.964
PARP14	0.53727	3.21589	11.7105	2.61E-07	8.84E-06	6.617
RPL7AP2	0.53755	0.26878	6.11762	9.67E-05	0.00144	0.23188
RTN1	0.53755	0.41929	4.57047	0.00093	0.0101	-2.1789
SMTNL1	0.53866	1.08578	4.49564	0.00105	0.01111	-2.3035
FAM167B	0.53909	0.62927	4.24961	0.00156	0.01535	-2.7178
LINC0030	0.53959	0.2698	19.6722	1.50E-09	1.18E-07	12.1926
LINC0203	0.53959	0.2698	8.36785	6.24E-06	0.00014	3.18121

MT1E	0.53959	0.2698	8.36785	6.24E-06	0.00014	3.18121
TRIM64E	0.5398	0.2699	4.91361	0.00055	0.00648	-1.6164
LINC0096	0.54028	0.75103	3.43683	0.00604	0.04681	-4.1274
ADH6	0.54092	0.65101	5.44652	0.00025	0.0033	-0.7739
H1-2	0.54105	2.06903	5.51011	0.00023	0.00305	-0.6759
LRCH2	0.54222	0.27111	6.46704	6.07E-05	0.00096	0.73137
CYP4F22	0.54225	0.71037	5.36415	0.00028	0.00363	-0.9016
FAM167A	0.54241	1.16524	6.76758	4.12E-05	0.00069	1.1481
CAPN6	0.54255	0.27127	6.37596	6.84E-05	0.00107	0.60274
LINC0273	0.54255	0.27127	4.74695	0.00071	0.00803	-1.8877
PRDM1	0.54255	0.32144	3.74925	0.00355	0.0302	-3.5797
TMEM178	0.54255	0.37162	5.06287	0.00044	0.00534	-1.3766
SERPINE	0.54374	1.00748	5.98982	0.00012	0.00168	0.04511
ZNF596	0.54397	1.53362	5.84472	0.00014	0.002	-0.1697
DST	0.54425	2.86254	8.95544	3.34E-06	7.99E-05	3.8568
PPP1R9A	0.54435	0.88104	7.14354	2.57E-05	0.00047	1.65316
TCP10L	0.54445	0.58178	4.32047	0.00139	0.01397	-2.5978
DNAJC3-	0.5459	1.30977	7.39255	1.90E-05	0.00036	1.97804
RIN2	0.54608	0.32321	4.46957	0.00109	0.01151	-2.3471
UNC80	0.54608	0.37338	5.18162	0.00037	0.00459	-1.1879
ZNF880	0.54624	1.03221	6.05809	0.0001	0.00155	0.14516
TMSB15A	0.54638	2.03502	10.4979	7.47E-07	2.20E-05	5.47704
CPB2-AS	0.54722	0.27361	9.77373	1.47E-06	3.94E-05	4.74221
PRAMEF9	0.5474	0.2737	13.3524	7.22E-08	2.95E-06	8.00646
ENPP1	0.54741	0.36642	5.64753	0.00019	0.00255	-0.4662
WDR25	0.54791	1.74865	5.54046	0.00022	0.00293	-0.6294
HHIP-AS1	0.54812	0.27406	4.24868	0.00156	0.01536	-2.7194
MSRB2	0.54826	1.8873	7.34378	2.02E-05	0.00038	1.915
LINC0261	0.54872	0.50439	3.6213	0.00441	0.03626	-3.8034
DISP2	0.54892	0.88228	3.82798	0.00311	0.0271	-3.4427
PLEKHH2	0.54948	1.03412	5.36581	0.00028	0.00362	-0.899
ZNF528	0.54985	0.7227	10.9839	4.84E-07	1.49E-05	5.94677
FRG2	0.54995	0.36302	6.91825	3.40E-05	0.00059	1.35264
FGF7P4	0.55011	0.44647	10.1773	1.00E-06	2.83E-05	5.15705
FRG2C	0.55025	0.3253	4.71199	0.00075	0.00839	-1.9451
ADAMTS1	0.55056	1.89575	11.518	3.06E-07	1.02E-05	6.44302
C16orf96	0.55075	0.40507	4.35246	0.00132	0.01338	-2.5438
LINC0032	0.55075	0.45524	4.29865	0.00144	0.01439	-2.6347
H2AC11	0.55103	1.15352	4.75361	0.0007	0.00795	-1.8768
CSRNP3	0.55122	2.06401	13.55	6.25E-08	2.62E-06	8.16321
RPL37A-I	0.55128	1.1926	8.67309	4.49E-06	0.0001	3.53653
MAP3K4-	0.55131	0.91445	6.88264	3.56E-05	0.00061	1.30457
CCDC71L	0.55202	1.85494	6.42936	6.38E-05	0.001	0.67829
ATP5MC1	0.55208	0.59675	3.89215	0.0028	0.02474	-3.3313
RBKS	0.55302	1.39881	6.24897	8.10E-05	0.00124	0.42157
SALL2	0.55363	2.36541	4.79868	0.00065	0.00749	-1.8031
KCNK15-/	0.5537	0.37719	6.9413	3.31E-05	0.00057	1.38368
MRC1	0.5537	0.37719	6.9413	3.31E-05	0.00057	1.38368
SORT1	0.55389	2.37898	10.0371	1.14E-06	3.17E-05	5.01451
ZNF568	0.55426	0.35902	4.92426	0.00054	0.00638	-1.5992
ATXN1	0.55574	0.30722	6.65057	4.78E-05	0.00078	0.98725
GHET1	0.55574	0.55808	4.8598	0.00059	0.00694	-1.7036
HNMT	0.55574	0.27787	8.02266	9.15E-06	0.00019	2.76754
MAMDC2	0.55574	0.32804	8.08571	8.53E-06	0.00018	2.84406
RN7SKP4	0.55574	0.27787	8.02266	9.15E-06	0.00019	2.76754
TMCO5B	0.55647	0.28513	6.56751	5.32E-05	0.00086	0.872
UTRN	0.55664	2.22008	9.791	1.45E-06	3.89E-05	4.76025
KCNMB4	0.55737	0.4361	5.403	0.00026	0.00346	-0.8412

TRIM38	0.55756	1.49176	6.88521	3.55E-05	0.00061	1.30804
OLMALIN	0.55793	2.0795	4.87938	0.00058	0.00677	-1.6719
SLF2	0.55906	3.18431	9.68895	1.60E-06	4.22E-05	4.65328
KRT24	0.55927	0.27964	6.70547	4.46E-05	0.00074	1.06294
TMEM132	0.55927	0.37998	5.20583	0.00035	0.00444	-1.1497
ANKRD24	0.55931	0.7899	3.56248	0.00487	0.03946	-3.9065
C1orf162	0.5595	0.85985	3.76241	0.00348	0.02964	-3.5568
EGFEM1I	0.56041	0.28021	5.63327	0.00019	0.0026	-0.4878
NR5A2	0.56041	0.28021	8.77944	4.01E-06	9.39E-05	3.6581
IRF1-AS1	0.56105	1.62416	6.63775	4.86E-05	0.00079	0.96953
CSH2	0.56283	0.28141	6.6877	4.56E-05	0.00076	1.03849
APOL4	0.56337	0.38203	3.49628	0.00545	0.04309	-4.0228
HLA-DRA	0.56337	0.28168	9.22735	2.53E-06	6.25E-05	4.15789
LINC0060	0.56337	0.33186	6.77657	4.07E-05	0.00069	1.16039
ZNF30-AS	0.56337	0.41138	6.30582	7.51E-05	0.00116	0.50294
CRIM1	0.56366	1.13826	7.62566	1.44E-05	0.00028	2.27546
DDIT4	0.56481	2.8246	5.4744	0.00024	0.00319	-0.7309
ZNF204P	0.56565	1.41279	4.05222	0.00215	0.01996	-3.0551
C2orf15	0.566	0.6219	5.03082	0.00046	0.00556	-1.4278
NELL1	0.566	0.39187	6.53389	5.56E-05	0.00089	0.82509
ARL9	0.56632	0.41285	6.54643	5.47E-05	0.00088	0.8426
LINC0259	0.56632	0.3835	5.17399	0.00037	0.00463	-1.2
SCOC-AS	0.56634	1.00545	4.84512	0.00061	0.00707	-1.7275
ASXL3-DT	0.5669	0.28345	4.16691	0.00178	0.01711	-2.8586
CFAP58	0.5669	0.46331	4.81292	0.00064	0.00735	-1.7799
IMPG2	0.5669	0.41314	5.68242	0.00018	0.00245	-0.4133
LINC0255	0.5669	0.38379	6.89521	3.50E-05	0.0006	1.32156
XK	0.5669	0.64318	4.48821	0.00106	0.01122	-2.3159
ZEB2-AS1	0.5669	0.28345	7.39598	1.90E-05	0.00036	1.98247
ABCC6	0.56702	1.94739	5.77148	0.00016	0.00219	-0.2792
RTN2	0.56823	2.03213	4.60002	0.00089	0.00971	-2.1299
GRHL3	0.56843	0.6146	3.88425	0.00284	0.02503	-3.345
SNRPGP	0.56873	0.30336	4.62094	0.00086	0.00946	-2.0952
SALRNA2	0.56894	0.33464	6.63284	4.89E-05	0.0008	0.96273
NPL	0.5693	2.0931	10.1292	1.05E-06	2.95E-05	5.10832
ERBB4	0.57147	1.70621	14.1335	4.12E-08	1.82E-06	8.61419
TARS1-D	0.5716	0.77522	7.19669	2.41E-05	0.00044	1.72313
ARHGAP	0.57189	0.38629	4.86053	0.00059	0.00693	-1.7025
ASIC4	0.57189	0.46581	4.33559	0.00135	0.01368	-2.5723
IRX6	0.57461	0.2873	4.74117	0.00071	0.00809	-1.8972
TRIM6-TF	0.57461	1.96851	6.18601	8.82E-05	0.00133	0.33094
TMEM10	0.57497	1.23494	7.09918	2.72E-05	0.00049	1.59449
ZNF697	0.57515	1.82644	9.94215	1.25E-06	3.43E-05	4.917
LUNAR1	0.57651	0.75595	4.12053	0.00192	0.01819	-2.9379
KHDC1	0.57657	0.38863	4.21259	0.00165	0.0161	-2.7808
KHDC1P1	0.57657	0.28828	16.788	7.40E-09	4.42E-07	10.4698
FAM229B	0.57906	0.91397	4.52484	0.001	0.01069	-2.2548
CSGALN	0.57919	1.49409	7.66753	1.38E-05	0.00027	2.3282
DPYSL5	0.5794	0.35603	3.4416	0.00599	0.04649	-4.119
RXFP1	0.57952	0.33993	5.43196	0.00025	0.00335	-0.7964
MYO15A	0.57961	0.51517	3.49289	0.00548	0.04329	-4.0288
CP	0.57991	2.15884	10.6986	6.23E-07	1.87E-05	5.67318
CDKN1A	0.58078	2.53992	8.3093	6.65E-06	0.00015	3.11195
LRP1-AS	0.5809	0.61616	4.13739	0.00187	0.01781	-2.9091
HOXB-AS	0.58152	0.97769	3.39847	0.00645	0.0495	-4.195
RWDD3	0.58173	2.17935	11.4403	3.27E-07	1.07E-05	6.37201
CASP5	0.58305	0.3417	6.09579	9.96E-05	0.00148	0.20014
LINC0114	0.58305	0.44204	7.84288	1.12E-05	0.00023	2.54691

USP30-A!	0.58365	0.49251	4.61634	0.00087	0.00951	-2.1028
UGCG	0.58404	2.93899	17.5007	4.87E-09	3.15E-07	10.9207
ETS1	0.58419	0.37162	5.16689	0.00037	0.00467	-1.2112
ESPN	0.58508	0.71859	4.09399	0.00201	0.01883	-2.9834
DACH1	0.58509	0.29255	11.1636	4.14E-07	1.30E-05	6.11596
SLC5A5	0.58642	0.53177	4.48808	0.00106	0.01122	-2.3161
DNAI3	0.58661	1.26511	7.008	3.04E-05	0.00054	1.47312
NUDT10	0.5873	1.582	12.3	1.62E-07	5.89E-06	7.13468
KRTAP7-	0.58772	0.29386	4.0926	0.00201	0.01887	-2.9858
LINC0234	0.58772	0.34403	6.4569	6.15E-05	0.00098	0.7171
UNC5C-A	0.58805	0.29402	12.0746	1.93E-07	6.86E-06	6.93934
STXBP5L	0.58945	1.38153	8.30697	6.67E-06	0.00015	3.10919
VLDLR	0.59033	2.57735	9.76061	1.49E-06	3.98E-05	4.72848
CHAC1	0.59034	2.17647	4.47902	0.00108	0.01136	-2.3313
KCNK12	0.59059	0.75536	5.78164	0.00015	0.00216	-0.2639
FAM230I	0.59088	0.49613	5.72325	0.00017	0.00232	-0.3517
RNASE1	0.59125	0.39597	5.89914	0.00013	0.00187	-0.0888
LINC0088	0.59258	0.42598	4.49168	0.00106	0.01117	-2.3101
STX18-A!	0.59259	1.5401	6.02876	0.00011	0.0016	0.10225
CD101-A!	0.59272	0.3967	6.93835	3.32E-05	0.00058	1.37972
PMP2	0.59272	0.32571	10.232	9.54E-07	2.71E-05	5.21226
POU2AF1	0.59272	0.29636	8.38608	6.12E-06	0.00014	3.20271
PYROXD	0.59272	0.47622	5.39079	0.00027	0.00352	-0.8602
RIT2	0.59272	0.29636	8.38608	6.12E-06	0.00014	3.20271
MRPL23-/	0.59348	0.37626	5.12131	0.0004	0.00495	-1.2835
WARS1	0.59462	3.6534	8.99344	3.21E-06	7.72E-05	3.8993
GRIP1	0.59594	0.73518	3.44761	0.00593	0.0461	-4.1084
HOXB-AS	0.59659	1.86812	7.0025	3.06E-05	0.00054	1.46576
ZNF626	0.59674	2.13889	15.6896	1.46E-08	7.77E-07	9.73793
HLA-K	0.59696	0.63035	4.28328	0.00147	0.01467	-2.6607
SELENBF	0.59739	0.29869	4.74515	0.00071	0.00805	-1.8907
HLA-DPB	0.59808	0.31803	6.68312	4.59E-05	0.00076	1.03218
C8orf88	0.59818	0.65882	4.55654	0.00095	0.01029	-2.202
LINC0228	0.59829	0.29914	10.1215	1.06E-06	2.97E-05	5.10047
MISP	0.59829	0.29914	10.1215	1.06E-06	2.97E-05	5.10047
DGKI	0.59862	0.37883	3.78853	0.00333	0.02859	-3.5113
RNASEL	0.59953	1.80483	7.46153	1.75E-05	0.00034	2.06672
TMEM37	0.60015	1.93889	5.38775	0.00027	0.00353	-0.8649
CLPTM1L	0.60034	0.30017	6.1151	9.70E-05	0.00145	0.22823
ELAVL3	0.60069	0.88544	6.1665	9.05E-05	0.00136	0.30274
DLGAP1-	0.60119	1.60497	11.2293	3.91E-07	1.24E-05	6.17726
C5	0.60136	2.2962	12.9284	9.92E-08	3.87E-06	7.66289
AMOT	0.60178	1.85857	7.52172	1.63E-05	0.00032	2.14363
RNU6-35	0.60257	0.72734	4.93792	0.00053	0.00627	-1.5772
DNAI7	0.60387	0.35211	8.62011	4.75E-06	0.00011	3.47554
STX18	0.60398	2.9567	11.2365	3.89E-07	1.23E-05	6.18394
MLIP	0.60429	0.45266	4.80744	0.00064	0.0074	-1.7888
MESTP4	0.60435	0.33152	8.54671	5.14E-06	0.00012	3.39058
PPP1R3C	0.60562	1.60019	8.18717	7.61E-06	0.00016	2.96629
ERP27	0.60591	0.30296	12.6395	1.24E-07	4.61E-06	7.42297
MANCR	0.60591	0.30296	6.90676	3.45E-05	0.0006	1.33715
OR5G3	0.60591	0.30296	8.82454	3.83E-06	9.02E-05	3.7093
NRXN3	0.60623	0.41726	9.01495	3.14E-06	7.58E-05	3.9233
FLT3LG	0.60626	1.24003	5.3204	0.0003	0.00385	-0.9698
SEMA3E	0.6066	1.10047	5.0109	0.00047	0.0057	-1.4598
GRM8	0.60741	0.50439	5.14681	0.00039	0.00479	-1.243
UBTFL1	0.60785	0.30392	6.59746	5.12E-05	0.00083	0.91365
PDZRN3	0.60804	1.02996	4.58516	0.00091	0.0099	-2.1545

PAN3-AS	0.60818	0.95051	4.79875	0.00065	0.00749	-1.803
STEAP2	0.60821	1.55706	11.1987	4.02E-07	1.27E-05	6.14875
RBM17P1	0.60823	0.3231	6.22013	8.42E-05	0.00128	0.38012
DNAH6	0.60855	0.48414	5.41495	0.00026	0.00342	-0.8227
EBI3	0.60887	0.43413	4.52947	0.00099	0.01063	-2.2471
GJA5	0.60887	0.35461	9.3917	2.14E-06	5.41E-05	4.33649
GRM1	0.60945	0.30472	6.90348	3.47E-05	0.0006	1.33272
ZFPM2	0.61043	0.80693	9.43178	2.06E-06	5.24E-05	4.37967
MAGEA1	0.61059	0.30529	15.8513	1.31E-08	7.13E-07	9.84867
SLC2A12	0.61077	0.86876	7.15029	2.55E-05	0.00046	1.66206
HEXA-AS	0.61137	0.58589	5.68165	0.00018	0.00245	-0.4145
P2RX3	0.61148	0.6731	4.69838	0.00076	0.00853	-1.9675
TRIM51E	0.61198	0.30599	7.04739	2.90E-05	0.00052	1.52568
EDNRB	0.61208	0.30604	7.21918	2.35E-05	0.00043	1.75264
ERVMER	0.6128	1.62267	9.7067	1.57E-06	4.16E-05	4.67196
AGAP2	0.61425	1.79621	4.31562	0.0014	0.01406	-2.606
B2M	0.61495	3.86927	12.1903	1.76E-07	6.34E-06	7.04
LINC0183	0.61513	1.2725	10.8895	5.26E-07	1.60E-05	5.85691
USP18	0.61534	2.09543	8.58707	4.92E-06	0.00011	3.43736
ADGRL2	0.61552	1.73778	9.5235	1.88E-06	4.86E-05	4.47794
ACTL8	0.6158	1.01496	5.77801	0.00015	0.00217	-0.2694
LINC0152	0.61605	1.03944	3.55661	0.00492	0.03976	-3.9168
STARD8	0.61663	0.83004	4.85546	0.0006	0.00697	-1.7107
ANXA10	0.61707	0.35871	8.47295	5.57E-06	0.00012	3.30465
BCAN-AS	0.61707	0.40888	5.00557	0.00048	0.00574	-1.4683
DLGAP1-	0.61836	1.88513	8.96027	3.32E-06	7.96E-05	3.86221
ZBTB8B	0.61861	1.15779	10.688	6.29E-07	1.88E-05	5.66292
FAM226B	0.61895	0.49786	5.44368	0.00025	0.0033	-0.7783
ADAMTS	0.61911	0.4099	5.42487	0.00026	0.00338	-0.8074
LINC0039	0.61911	0.30956	11.4195	3.33E-07	1.09E-05	6.35294
RGS22	0.61911	0.42605	6.65758	4.74E-05	0.00078	0.99694
RINL	0.62042	0.4399	4.68025	0.00079	0.00873	-1.9973
EDNRA	0.62044	0.85834	4.04251	0.00218	0.02023	-3.0718
MPP4	0.62049	0.41748	7.92003	1.03E-05	0.00021	2.64203
PRSS8	0.6206	0.46082	4.55284	0.00096	0.01034	-2.2082
ZBED9	0.62143	1.56749	6.99652	3.09E-05	0.00054	1.45777
ASNS	0.62169	3.5385	14.8369	2.54E-08	1.21E-06	9.13573
FAT3	0.62207	0.31103	6.98837	3.12E-05	0.00055	1.44686
RPS17P1	0.62207	0.31103	12.3784	1.52E-07	5.56E-06	7.20186
NGFR	0.62217	0.8592	5.96025	0.00012	0.00174	0.00157
SEMA6A-	0.62263	1.44302	6.96535	3.21E-05	0.00056	1.41599
FZD3	0.6228	2.53029	15.4723	1.67E-08	8.61E-07	9.58743
S1PR3	0.62326	1.66362	10.1198	1.06E-06	2.97E-05	5.09879
STK24-AS	0.62399	0.69254	6.18473	8.83E-05	0.00134	0.32909
TRANK1	0.624	1.25446	7.11348	2.67E-05	0.00048	1.61342
NTF4	0.62418	1.37833	5.68897	0.00018	0.00243	-0.4034
ANKRD31	0.62422	1.11241	7.22484	2.33E-05	0.00043	1.76006
C1GALT1	0.6249	0.4128	8.52497	5.26E-06	0.00012	3.36531
RTL9	0.6256	0.3128	9.23081	2.52E-06	6.23E-05	4.16167
CTNNA2-	0.62674	0.31337	15.2813	1.90E-08	9.53E-07	9.4535
GLT1D1	0.62674	0.31337	7.23484	2.30E-05	0.00043	1.77315
PCM1	0.62675	3.78587	14.8887	2.46E-08	1.18E-06	9.17322
SBK1	0.62813	1.61587	3.68263	0.00397	0.03322	-3.696
CPHL1P	0.62823	0.41446	6.89323	3.51E-05	0.0006	1.31887
NCCRP1	0.62823	0.41446	6.89323	3.51E-05	0.0006	1.31887
OR5AK1F	0.62823	0.31411	7.31413	2.09E-05	0.00039	1.87653
ZSCAN10	0.62866	0.56519	6.43868	6.30E-05	0.00099	0.69144
ARHGAP	0.62871	0.99979	7.63514	1.43E-05	0.00028	2.28742

PBX3	0.62873	3.11324	11.8256	2.37E-07	8.15E-06	6.71987
IRF1	0.62913	2.60359	8.50428	5.38E-06	0.00012	3.34121
CD200R1	0.62937	0.3942	6.63229	4.90E-05	0.0008	0.96197
PCBP3	0.62951	0.51544	4.46599	0.0011	0.01156	-2.3531
RSPO3	0.62969	0.31485	9.21902	2.55E-06	6.30E-05	4.14877
SP110	0.62999	1.88626	6.33611	7.21E-05	0.00112	0.54613
LINC0240	0.63027	0.31513	6.59201	5.16E-05	0.00084	0.90609
SCTR	0.63027	0.41548	5.44533	0.00025	0.0033	-0.7757
NEFHP1	0.63064	0.31532	7.49398	1.69E-05	0.00033	2.10824
BTN3A1	0.63192	2.26261	5.53402	0.00022	0.00295	-0.6393
NAB1	0.63193	2.51837	16.8194	7.26E-09	4.36E-07	10.49
KCNN3	0.63214	0.64645	5.37156	0.00028	0.0036	-0.8901
THSD4	0.63296	1.64369	5.47382	0.00024	0.00319	-0.7318
GRIN1	0.63322	0.4008	6.22954	8.32E-05	0.00127	0.39365
SLC7A2	0.63447	3.16532	13.9354	4.74E-08	2.05E-06	8.46305
ATP1A2	0.63526	0.31763	5.57486	0.00021	0.0028	-0.5768
DPYS	0.63526	0.54767	7.35408	1.99E-05	0.00038	1.92834
SYN3	0.63526	0.3678	5.66512	0.00018	0.0025	-0.4395
PKDREJ	0.636	0.76487	5.04762	0.00045	0.00544	-1.401
TRIM43B	0.63679	0.31839	6.19461	8.72E-05	0.00132	0.34335
CPNE8	0.63702	3.07212	14.2711	3.74E-08	1.69E-06	8.71806
DYNC1LI	0.63716	0.57167	5.02446	0.00046	0.0056	-1.438
CYP4F29	0.63717	0.35656	17.2222	5.72E-09	3.56E-07	10.7466
LINC0179	0.63719	0.90369	9.79979	1.44E-06	3.87E-05	4.76941
GUCY1A2	0.63789	0.41929	6.78834	4.01E-05	0.00068	1.17646
SERTAD4	0.63789	0.31895	4.82152	0.00063	0.00727	-1.7659
TM4SF4	0.63789	0.31895	12.8786	1.03E-07	3.98E-06	7.6219
LINC0037	0.63822	0.79533	4.8478	0.00061	0.00705	-1.7231
NECTIN4	0.63822	0.46962	5.78968	0.00015	0.00214	-0.2519
MBD3L5	0.63825	0.31912	15.3998	1.75E-08	8.97E-07	9.53677
NUGGC	0.6385	0.52846	4.34691	0.00133	0.01348	-2.5532
IFI35	0.63864	2.2509	6.71523	4.40E-05	0.00073	1.07636
LARP6	0.6399	2.30804	10.9394	5.03E-07	1.54E-05	5.90446
AIM2	0.63993	0.31997	6.2582	8.00E-05	0.00123	0.43481
RCAN1	0.6421	2.77898	14.2188	3.88E-08	1.74E-06	8.67871
CASC15	0.64289	0.37162	10.9842	4.84E-07	1.49E-05	5.947
CLPSL2	0.64289	0.32144	15.2385	1.95E-08	9.73E-07	9.42329
GDNF-AS	0.64289	0.32144	15.2385	1.95E-08	9.73E-07	9.42329
H2BC27P	0.64379	0.32189	5.05472	0.00044	0.00539	-1.3896
CA2	0.6438	1.83328	11.2789	3.75E-07	1.20E-05	6.2233
DLG1-AS	0.64416	1.08316	5.58435	0.0002	0.00277	-0.5623
TMEM126	0.64419	2.6221	12.1152	1.87E-07	6.68E-06	6.97477
MAGED2	0.64456	3.0225	8.25008	7.10E-06	0.00015	3.04152
H2AC6	0.64557	1.55824	11.153	4.18E-07	1.31E-05	6.10603
KCNE1	0.64571	0.62388	6.7631	4.14E-05	0.0007	1.14198
GAB2	0.64623	1.78171	5.93289	0.00012	0.0018	-0.0388
EXO5-DT	0.64756	0.32378	11.2463	3.85E-07	1.23E-05	6.19312
PKN2-AS	0.64756	0.32378	21.5978	5.80E-10	5.60E-08	13.2104
PURPL	0.64901	2.42188	11.7585	2.50E-07	8.53E-06	6.66
PANCR	0.64905	0.32453	5.82756	0.00014	0.00204	-0.1953
ZC2HC1C	0.64912	1.35153	4.93094	0.00053	0.00633	-1.5884
KDM4E	0.64938	0.32469	11.5231	3.05E-07	1.01E-05	6.4476
MYLK3	0.64942	0.99195	6.51626	5.69E-05	0.00091	0.80043
DMD	0.64944	3.54438	13.8991	4.86E-08	2.09E-06	8.43512
THBS4	0.64991	0.53417	3.62488	0.00438	0.03611	-3.7971
FBLIM1	0.65052	2.03448	8.85846	3.69E-06	8.74E-05	3.74768
SSC4D	0.65099	0.69375	6.58637	5.20E-05	0.00084	0.89824
RGS4	0.65109	0.40507	7.89235	1.06E-05	0.00022	2.60798

TRIM69	0.65112	2.42374	9.95413	1.24E-06	3.41E-05	4.92934
TSPY26P	0.65115	0.83468	6.27477	7.83E-05	0.0012	0.45855
RNF152	0.65119	0.79419	5.62266	0.00019	0.00264	-0.5039
PHF24	0.65141	0.37588	6.37772	6.83E-05	0.00107	0.60524
TIGD2	0.65233	2.59905	11.2429	3.87E-07	1.23E-05	6.18995
GPX3	0.6524	0.85259	5.18586	0.00036	0.00456	-1.1812
LCP1	0.65329	1.37556	13.4061	6.94E-08	2.86E-06	8.04929
LIN28B	0.65377	3.2598	16.4816	8.90E-09	5.13E-07	10.2703
CDH11	0.65405	0.32702	4.93961	0.00053	0.00626	-1.5744
AMPH	0.65412	0.85345	4.77009	0.00068	0.00777	-1.8498
DLG3	0.65421	2.44442	7.23708	2.30E-05	0.00042	1.77608
LINC0202	0.65462	0.75336	4.1828	0.00173	0.01674	-2.8315
FAM110D	0.65562	1.43287	5.20239	0.00035	0.00446	-1.1551
SH2D4A	0.6559	2.7921	13.2686	7.68E-08	3.09E-06	7.93936
SYNM-AS	0.65609	0.50791	4.69125	0.00077	0.0086	-1.9792
ZIC5	0.65609	0.32804	9.62186	1.71E-06	4.46E-05	4.58248
EPHA5	0.65622	1.13153	12.1365	1.84E-07	6.58E-06	6.9933
RHOT1P1	0.65633	0.51347	7.01161	3.03E-05	0.00054	1.47795
CACNG7	0.65718	2.12704	4.34343	0.00134	0.01354	-2.559
MOV10	0.65719	2.83544	5.68026	0.00018	0.00245	-0.4166
BTN3A2	0.65721	2.4787	6.55395	5.42E-05	0.00087	0.8531
SLIT2-IT1	0.65904	0.42986	7.92716	1.02E-05	0.00021	2.65079
CACNA1(	0.65943	1.29055	3.42111	0.0062	0.04789	-4.1551
SLC7A7	0.65955	1.45681	5.87175	0.00014	0.00194	-0.1294
P2RY1	0.66047	0.68996	6.04805	0.00011	0.00156	0.13048
H2BC12L	0.6607	0.88199	9.99651	1.19E-06	3.28E-05	4.9729
SNCA-AS	0.66076	0.33038	7.81394	1.16E-05	0.00024	2.51106
ABCA6	0.66271	0.44213	6.97117	3.19E-05	0.00056	1.4238
DLGAP1	0.66289	1.32653	7.88076	1.08E-05	0.00022	2.5937
IGF2BP1	0.66359	3.41492	7.37242	1.95E-05	0.00037	1.95205
NES	0.66383	3.51912	7.20048	2.40E-05	0.00044	1.72812
RFX5-AS	0.66415	1.11228	6.14878	9.27E-05	0.00139	0.2771
SLC16A4	0.66453	1.15502	5.28227	0.00032	0.00405	-1.0295
HAS2-AS	0.66461	0.33231	5.55093	0.00021	0.00289	-0.6133
CHN1	0.6659	1.89401	10.3267	8.74E-07	2.51E-05	5.30719
IDO2	0.66667	0.3967	6.16573	9.06E-05	0.00136	0.30163
PDGFB	0.66667	0.46303	6.80229	3.94E-05	0.00067	1.19548
BRWD1P	0.66724	0.33362	4.98326	0.00049	0.00591	-1.5041
LINC0161	0.66724	0.33362	8.41728	5.91E-06	0.00013	3.23941
DCBLD2	0.66778	2.71773	17.3643	5.27E-09	3.33E-07	10.8357
SGK1	0.66797	0.45738	4.70677	0.00075	0.00844	-1.9537
S100A1	0.66801	1.93309	7.12721	2.63E-05	0.00047	1.63159
DISP1	0.6685	1.48371	9.58596	1.77E-06	4.60E-05	4.54442
TSPAN2	0.66928	0.38481	7.40771	1.87E-05	0.00036	1.99758
TBX10	0.66951	0.64431	5.12336	0.0004	0.00493	-1.2802
COL6A5	0.6702	0.38527	7.57988	1.52E-05	0.0003	2.21756
RPL10L	0.67022	0.45484	5.83077	0.00014	0.00203	-0.1905
BAGE2	0.67043	1.02163	5.26308	0.00032	0.00415	-1.0596
SOX6	0.67048	1.29325	11.9432	2.15E-07	7.51E-06	6.82401
CEACAM	0.67151	1.25885	9.15869	2.71E-06	6.66E-05	4.08252
CARMAL	0.67191	0.33596	10.1645	1.02E-06	2.86E-05	5.1441
LINC0095	0.67224	0.33612	5.3707	0.00028	0.0036	-0.8914
PHOX2B-	0.67224	0.33612	9.95007	1.24E-06	3.41E-05	4.92516
UBAC2-A	0.67228	1.06971	8.67232	4.49E-06	0.0001	3.53565
SP140	0.67273	0.87424	6.68297	4.59E-05	0.00076	1.03197
ARL10	0.67287	2.59901	4.76195	0.00069	0.00787	-1.8632
FBXO27	0.67419	2.01721	6.03137	0.00011	0.0016	0.10608
H4C8	0.67498	1.20412	6.96521	3.21E-05	0.00056	1.41581

LRRTM1	0.67508	0.38771	5.96018	0.00012	0.00174	0.00147
ADCY10	0.67519	0.64715	4.08526	0.00203	0.01907	-2.9983
CPA1	0.67519	0.3376	8.02904	9.09E-06	0.00019	2.77529
STAU2-A	0.67691	0.38863	5.75253	0.00016	0.00224	-0.3076
FOXO1B	0.67771	0.58504	4.8758	0.00058	0.0068	-1.6777
WWC2-A	0.67806	0.88715	4.42477	0.00117	0.01212	-2.4221
CRMA	0.6784	0.3392	11.5081	3.09E-07	1.02E-05	6.43401
MIR130AI	0.67861	1.6625	8.82354	3.83E-06	9.02E-05	3.70817
BMP4	0.67914	0.43991	6.30675	7.50E-05	0.00116	0.50428
FERMT2	0.68053	3.70784	18.1131	3.44E-09	2.38E-07	11.2942
KHDRBS:	0.68076	0.34038	9.2698	2.42E-06	6.03E-05	4.20426
OR2B6	0.6834	0.39187	7.70015	1.32E-05	0.00026	2.36915
CAV1	0.6838	1.19753	9.55993	1.81E-06	4.70E-05	4.51675
DISC1FP	0.68429	0.34215	7.43209	1.82E-05	0.00035	2.02894
PTGER2	0.68429	0.44249	7.16456	2.51E-05	0.00046	1.68087
MIR17HG	0.68463	2.13203	12.1015	1.89E-07	6.73E-06	6.96281
TBC1D8E	0.68463	1.30788	7.8732	1.08E-05	0.00022	2.58437
MACIR	0.6849	2.04468	15.0079	2.27E-08	1.10E-06	9.259
HHLA2	0.68665	0.34332	13.948	4.69E-08	2.04E-06	8.4727
DDO	0.68693	0.34346	10.0116	1.17E-06	3.24E-05	4.98837
STYXL2	0.68693	0.34346	10.0116	1.17E-06	3.24E-05	4.98837
HHIP	0.68702	1.02983	9.5609	1.81E-06	4.70E-05	4.51779
SCIRT	0.68737	0.50273	5.28897	0.00031	0.00402	-1.019
LINC0185	0.68744	1.05646	10.605	6.78E-07	2.01E-05	5.58206
NR3C1	0.68807	0.42355	6.22776	8.34E-05	0.00127	0.39109
SHISA3	0.6884	1.98853	9.01887	3.13E-06	7.56E-05	3.92767
LINC0103	0.69011	0.4454	5.90327	0.00013	0.00186	-0.0827
GNG11	0.69029	0.34515	4.81698	0.00064	0.00731	-1.7733
HEPH	0.69062	0.49582	5.5969	0.0002	0.00272	-0.5431
SCN2A	0.69092	2.06549	16.3764	9.49E-09	5.40E-07	10.201
RBM20	0.69101	0.44585	5.60957	0.0002	0.00268	-0.5238
SPATA46	0.6916	0.3458	7.06971	2.82E-05	0.0005	1.55538
CALD1	0.69183	3.15582	13.2719	7.66E-08	3.09E-06	7.94195
GRIK2	0.69295	1.24112	7.88624	1.07E-05	0.00022	2.60046
ADD3-AS	0.69306	0.34653	7.38209	1.93E-05	0.00037	1.96455
FGF21	0.69325	0.57666	5.71041	0.00017	0.00236	-0.371
ZNF860	0.69325	0.39193	7.77476	1.21E-05	0.00024	2.46236
ERVV-1	0.69399	0.39717	6.46865	6.06E-05	0.00096	0.73364
HOXB6	0.6941	1.66961	6.96381	3.22E-05	0.00056	1.41393
HLA-DOA	0.69418	0.41046	6.15578	9.18E-05	0.00138	0.28724
PATL2	0.6951	0.87394	4.44991	0.00113	0.01179	-2.38
TEX19	0.69575	1.45439	5.03423	0.00046	0.00554	-1.4224
PDLIM3	0.69922	0.34961	4.97621	0.0005	0.00596	-1.5155
RPL12P7	0.69955	0.34977	14.0797	4.28E-08	1.88E-06	8.57335
HOXB4	0.70033	1.93549	4.58023	0.00092	0.00997	-2.1627
EPHA5-A	0.70126	0.45098	7.88437	1.07E-05	0.00022	2.59815
RNU6-27	0.70126	0.35063	7.93245	1.01E-05	0.00021	2.65728
LINC0127	0.70159	0.35079	5.4826	0.00024	0.00315	-0.7182
LAMA4	0.70422	0.35211	11.2735	3.77E-07	1.21E-05	6.21831
LINC0298	0.70422	0.35211	11.2735	3.77E-07	1.21E-05	6.21831
LRG1	0.70422	0.5528	5.93349	0.00012	0.0018	-0.0379
PAPPA2	0.70422	0.35211	7.34907	2.01E-05	0.00038	1.92185
ACHE	0.70508	0.99044	4.70414	0.00076	0.00847	-1.958
EDIL3-DT	0.70512	0.35256	9.0017	3.18E-06	7.67E-05	3.90852
H3P21	0.70626	0.35313	12.5048	1.37E-07	5.06E-06	7.30945
PNMA8B	0.70626	0.4033	7.6863	1.35E-05	0.00027	2.35178
GRAPLDI	0.70679	0.46819	7.2567	2.24E-05	0.00042	1.80173
LUM	0.70681	1.2711	10.8998	5.21E-07	1.59E-05	5.86678

PTBP2	0.70694	2.55952	15.1698	2.04E-08	1.01E-06	9.37458
CD207	0.70738	0.40386	7.34286	2.02E-05	0.00038	1.91381
SYNPO2	0.70802	1.37554	9.57588	1.79E-06	4.64E-05	4.53372
CNTNAP3	0.7093	1.29631	11.7958	2.43E-07	8.31E-06	6.6933
DSCAS	0.70979	0.35489	6.77832	4.06E-05	0.00069	1.16278
FGF14	0.70979	0.35489	9.51296	1.90E-06	4.90E-05	4.46669
NAV3	0.71044	1.76304	11.4845	3.15E-07	1.04E-05	6.41246
MIR34AH	0.71097	1.01737	4.01006	0.0023	0.02116	-3.1276
SPARCL1	0.71127	1.22946	9.05685	3.01E-06	7.31E-05	3.96992
STX1B	0.71225	1.22232	5.39177	0.00027	0.00351	-0.8587
H2BC11	0.71307	1.29737	8.58247	4.95E-06	0.00011	3.43204
EXPH5	0.71354	0.50729	8.20789	7.44E-06	0.00016	2.99112
RAB9B	0.71401	0.86258	11.3251	3.60E-07	1.17E-05	6.26613
IGSF1	0.71448	0.61663	5.90615	0.00013	0.00186	-0.0784
PRAMEF1	0.71478	0.35739	7.9123	1.04E-05	0.00021	2.63253
PLCH1	0.71539	1.99301	8.45479	5.68E-06	0.00013	3.2834
CNDP1	0.71593	0.83419	7.6394	1.42E-05	0.00028	2.29279
CACNG6	0.71611	0.40823	7.05096	2.89E-05	0.00051	1.53044
KCNC1	0.71628	0.61752	5.81353	0.00015	0.00208	-0.2162
FZD2	0.71656	2.19373	3.39311	0.00651	0.04985	-4.2044
PRTG	0.71738	3.04877	16.5945	8.31E-09	4.84E-07	10.3442
ADGRF1	0.71742	0.40888	11.0078	4.74E-07	1.46E-05	5.96937
CARMN	0.71742	0.45905	8.27116	6.94E-06	0.00015	3.06663
LINC0162	0.71742	0.35871	11.9254	2.18E-07	7.60E-06	6.80828
SYBU	0.71742	0.43823	6.75426	4.19E-05	0.0007	1.12989
CBR3	0.71774	1.16433	10.6322	6.61E-07	1.97E-05	5.60865
CFAP91	0.71774	0.35887	7.48039	1.71E-05	0.00033	2.09087
HLA-G	0.71843	1.25039	4.58675	0.00091	0.00989	-2.1519
RIGI	0.71882	2.22479	11.4923	3.13E-07	1.03E-05	6.41954
SLC25A2	0.71962	2.3779	15.489	1.66E-08	8.57E-07	9.59905
HOXB5	0.71978	1.69713	6.94303	3.30E-05	0.00057	1.38601
ZNF736	0.7213	0.41772	9.12209	2.81E-06	6.88E-05	4.04216
IRF9	0.72172	2.52231	7.66738	1.38E-05	0.00027	2.32801
USP41	0.72204	1.24456	6.77942	4.06E-05	0.00068	1.16428
BTG4	0.72241	0.41138	7.73504	1.27E-05	0.00025	2.41283
NFIA	0.72321	0.46195	6.95136	3.27E-05	0.00057	1.39721
GREB1L-	0.72577	0.8523	7.37058	1.95E-05	0.00037	1.94967
TRIM15	0.72594	0.36297	11.8796	2.27E-07	7.84E-06	6.76782
ADM	0.72861	1.76788	7.03835	2.93E-05	0.00052	1.51363
DNAJA1F	0.72919	3.03076	13.9279	4.76E-08	2.06E-06	8.45729
ASB9	0.72931	0.41483	8.02168	9.16E-06	0.00019	2.76634
FNBP1L	0.73055	2.49658	17.3214	5.40E-09	3.40E-07	10.8089
RIC3-DT	0.73061	0.36531	11.3863	3.42E-07	1.11E-05	6.3225
PHGDH	0.73181	3.29877	8.69108	4.41E-06	0.0001	3.55717
SCG2	0.73284	0.54628	8.05651	8.81E-06	0.00019	2.80867
TRIM51B	0.73321	0.36661	9.03498	3.08E-06	7.46E-05	3.94561
PTPRR	0.73357	0.41696	8.32783	6.52E-06	0.00014	3.13391
CCDC160	0.73369	1.40609	9.04219	3.05E-06	7.41E-05	3.95362
LINC0230	0.73443	0.84344	9.48824	1.95E-06	5.00E-05	4.44025
VLDLR-A	0.73451	1.73821	7.4544	1.77E-05	0.00034	2.05757
MYOF	0.73483	1.33608	5.84575	0.00014	0.002	-0.1681
SPTSSB	0.73506	0.36753	12.6282	1.25E-07	4.65E-06	7.41348
MTMR9LI	0.73518	1.51608	7.03585	2.94E-05	0.00052	1.5103
BAALC-A	0.73536	0.93458	7.30564	2.11E-05	0.0004	1.8655
BMF	0.73553	0.93803	3.54475	0.00502	0.04037	-3.9377
PPP1R3A	0.73561	0.3678	8.46246	5.63E-06	0.00013	3.29238
TNC	0.7371	1.30434	7.22032	2.34E-05	0.00043	1.75414
BMP5	0.73795	0.82905	7.77042	1.22E-05	0.00024	2.45695

FGG	0.73813	1.66787	16.7959	7.36E-09	4.40E-07	10.4749
RBM11	0.73824	0.46946	8.03889	8.99E-06	0.00019	2.78727
AK3P5	0.73904	0.52856	6.77706	4.07E-05	0.00069	1.16106
LINC0068	0.73914	0.41974	5.11474	0.0004	0.00499	-1.2939
SLC6A20	0.73967	0.42001	8.21419	7.39E-06	0.00016	2.99866
NTRK3	0.74119	0.3706	4.86636	0.00059	0.00689	-1.693
SOX2	0.74323	0.37162	11.6329	2.78E-07	9.37E-06	6.54713
ASB2	0.74472	0.45188	8.45659	5.67E-06	0.00013	3.28551
RUNX1	0.74547	0.83576	8.59548	4.88E-06	0.00011	3.44709
PKD1L2	0.74572	0.38606	7.25307	2.25E-05	0.00042	1.79699
FUT1	0.7466	1.00004	5.96604	0.00012	0.00173	0.0101
TGFB2-O	0.74676	0.37338	22.0037	4.80E-10	4.87E-08	13.4134
XKR5	0.7474	1.38617	7.31433	2.09E-05	0.00039	1.8768
MBD3L4	0.74757	0.37378	14.8787	2.47E-08	1.19E-06	9.16594
MRPL35P	0.74823	0.63509	6.72517	4.35E-05	0.00073	1.09
MIX23P2	0.7503	0.37515	10.2334	9.52E-07	2.71E-05	5.21365
ACOX2	0.75098	0.42566	10.5625	7.04E-07	2.08E-05	5.54045
NOVA2	0.75106	1.47728	6.41344	6.51E-05	0.00102	0.65581
MAML2	0.75123	0.65399	7.44551	1.79E-05	0.00034	2.04617
TSPAN6	0.75184	2.96946	15.6017	1.54E-08	8.10E-07	9.67727
FBXO15	0.75484	0.81667	7.68603	1.35E-05	0.00027	2.35144
RGS21	0.75529	0.37764	9.63552	1.68E-06	4.41E-05	4.59693
TRIM6	0.75551	2.06332	8.71209	4.31E-06	9.99E-05	3.58124
MEG3	0.75588	0.37794	6.82712	3.82E-05	0.00065	1.22928
PPP3R2	0.75643	0.37821	12.6063	1.27E-07	4.72E-06	7.39507
LINC0180	0.75747	1.04894	11.4294	3.30E-07	1.08E-05	6.36204
ITPRID2-I	0.75792	0.37896	10.041	1.14E-06	3.16E-05	5.0185
GDAP1	0.75925	2.84837	20.1733	1.16E-09	9.63E-08	12.4667
FABP9	0.75996	0.43015	8.90496	3.52E-06	8.36E-05	3.80011
HNRNPC	0.7609	1.08191	6.98209	3.14E-05	0.00055	1.43844
H3C6	0.76099	1.12463	9.68383	1.61E-06	4.24E-05	4.64789
DSG2-AS	0.76259	0.43147	11.3906	3.41E-07	1.11E-05	6.32647
RGS8	0.76292	0.38146	9.5932	1.76E-06	4.58E-05	4.55211
MBD3L3	0.76327	0.38164	12.8114	1.08E-07	4.15E-06	7.56628
SLC17A6	0.76345	0.69128	7.96954	9.72E-06	0.0002	2.70272
NLGN1	0.76366	0.98774	7.82555	1.15E-05	0.00023	2.52545
HMGN5	0.7641	2.40794	15.5708	1.57E-08	8.23E-07	9.65589
COBLL1	0.76448	2.12028	14.2345	3.84E-08	1.72E-06	8.69053
LRRC8D-	0.76575	0.53339	6.57299	5.29E-05	0.00086	0.87963
PRKN	0.76628	0.43331	9.90191	1.30E-06	3.55E-05	4.87545
SLC6A15	0.76628	0.38314	8.62741	4.71E-06	0.00011	3.48396
LRRK2	0.76715	1.09836	9.93876	1.26E-06	3.44E-05	4.9135
ADAM12	0.76791	0.38395	10.0219	1.16E-06	3.21E-05	4.9989
APOBEC3	0.76805	1.42731	8.28595	6.83E-06	0.00015	3.08423
CTSLP4	0.76929	0.38464	16.9361	6.77E-09	4.11E-07	10.565
INHBE	0.76963	2.12433	8.3567	6.32E-06	0.00014	3.16805
CACNA1E	0.77035	1.78141	7.23924	2.29E-05	0.00042	1.7789
GABARAI	0.77068	1.6459	10.187	9.95E-07	2.81E-05	5.16683
SERP2	0.77258	0.38629	11.192	4.04E-07	1.27E-05	6.14253
TNIK	0.77258	0.53681	7.26753	2.21E-05	0.00041	1.81587
LOXL4	0.77346	1.0038	7.68668	1.35E-05	0.00027	2.35225
TMPRSS1	0.77367	1.0212	8.06888	8.69E-06	0.00018	2.82367
SCN1A	0.77496	1.02021	14.5318	3.13E-08	1.46E-06	8.91239
MIR8089	0.77521	0.38761	15.3685	1.79E-08	9.14E-07	9.51482
KRTAP19	0.77611	0.38806	7.22027	2.34E-05	0.00043	1.75407
SLC38A5	0.7762	1.10552	8.77178	4.05E-06	9.43E-05	3.64938
COL6A6	0.77654	0.56813	5.86745	0.00014	0.00195	-0.1358
ZIC2	0.77665	0.51802	6.09542	9.97E-05	0.00148	0.1996

MDM1	0.77668	2.67964	12.1067	1.89E-07	6.71E-06	6.96737
CSF1	0.77728	1.89426	6.5664	5.33E-05	0.00086	0.87045
MGAT4A	0.77838	0.96871	9.4865	1.95E-06	5.01E-05	4.43839
KYAT3	0.77968	2.63038	15.4957	1.65E-08	8.54E-07	9.60373
SP100	0.77971	1.96525	13.3986	6.98E-08	2.87E-06	8.04332
OSBPL10	0.77979	1.26972	8.08056	8.58E-06	0.00018	2.83782
ANO5	0.78061	1.08337	10.4294	7.95E-07	2.32E-05	5.40931
FBXO39	0.78078	0.49074	5.73584	0.00016	0.00229	-0.3327
ZNF614	0.78083	0.39042	4.84094	0.00061	0.00709	-1.7343
PLOD2	0.78154	1.63162	15.0806	2.16E-08	1.06E-06	9.31102
TRIM43	0.78281	0.3914	19.3215	1.79E-09	1.38E-07	11.9968
EFNA5	0.78285	1.46433	9.84978	1.37E-06	3.71E-05	4.82144
THRB-AS	0.78301	0.39151	9.18389	2.64E-06	6.53E-05	4.11023
APLF	0.78372	0.87161	7.61995	1.45E-05	0.00029	2.26825
NPY1R	0.78374	0.54238	9.80538	1.43E-06	3.85E-05	4.77524
TRIM10	0.78415	0.39208	9.28685	2.38E-06	5.94E-05	4.22284
LRRN3	0.78455	1.71228	14.6195	2.94E-08	1.38E-06	8.97702
YPEL4	0.78493	1.81139	8.77888	4.02E-06	9.39E-05	3.65745
FSIP2	0.7863	0.72799	5.74639	0.00016	0.00226	-0.3168
XCR1	0.78668	0.44351	12.839	1.06E-07	4.08E-06	7.5892
MIR548A1	0.78711	0.39355	7.24348	2.28E-05	0.00042	1.78445
RASGRP	0.7878	1.1873	9.15869	2.71E-06	6.66E-05	4.08252
AKAP6	0.78996	1.03024	8.12904	8.12E-06	0.00017	2.8964
CERKL	0.79064	0.39532	7.42053	1.84E-05	0.00035	2.01408
ARID3A	0.79141	2.63076	5.10767	0.00041	0.00504	-1.3052
PANTR1	0.79194	0.39597	18.9361	2.20E-09	1.63E-07	11.7775
ERRFI1	0.79234	2.48185	14.0955	4.23E-08	1.86E-06	8.58541
BTN3A3	0.79237	1.96847	9.15645	2.72E-06	6.67E-05	4.08006
NWD2	0.79268	0.44651	8.02981	9.08E-06	0.00019	2.77623
TMEM170	0.79386	1.19536	13.4092	6.92E-08	2.86E-06	8.05173
RNVU1-4	0.7949	0.46377	9.81353	1.42E-06	3.82E-05	4.78374
NMI	0.79551	2.29545	11.2711	3.77E-07	1.21E-05	6.21615
LNC-LBC	0.7966	1.67299	6.14238	9.35E-05	0.0014	0.26782
LINC0204	0.79694	0.39847	10.7658	5.87E-07	1.77E-05	5.73818
FRAS1	0.79806	0.49937	7.24261	2.28E-05	0.00042	1.78331
ETV1	0.79904	2.14942	10.1262	1.05E-06	2.96E-05	5.10526
CSH1	0.79942	0.39971	9.11036	2.85E-06	6.95E-05	4.02921
RGS3	0.79954	2.64935	7.47875	1.72E-05	0.00033	2.08876
MGP	0.79987	2.32918	23.2543	2.73E-10	3.14E-08	14.016
CHRNA9	0.79989	0.39995	17.723	4.29E-09	2.83E-07	11.0577
GASK1A	0.8003	0.50049	8.91321	3.49E-06	8.30E-05	3.80939
POTEC	0.80033	0.40017	11.8072	2.41E-07	8.24E-06	6.70341
OLFML3	0.80353	0.65262	6.086	0.0001	0.0015	0.18589
ADPRHL1	0.80358	1.02576	4.67679	0.00079	0.00877	-2.003
CBLC	0.80387	0.40193	8.68556	4.43E-06	0.0001	3.55085
LINC0303	0.8044	0.53189	8.15568	7.89E-06	0.00017	2.92847
FABP3	0.80531	1.44034	9.85756	1.36E-06	3.69E-05	4.82951
NFE2	0.8054	1.39565	7.94879	9.95E-06	0.00021	2.67732
TNS3	0.80594	1.39495	6.82919	3.81E-05	0.00065	1.2321
H2BC12	0.8077	2.14511	6.53954	5.52E-05	0.00089	0.83298
CLYBL	0.80873	1.18048	9.85549	1.36E-06	3.70E-05	4.82736
CA14	0.80876	2.53605	10.6138	6.72E-07	2.00E-05	5.59066
ZNF516-A	0.8093	1.08498	7.46329	1.75E-05	0.00034	2.06898
KCNK2	0.80956	0.55529	7.40068	1.89E-05	0.00036	1.98852
GNG2	0.81013	0.40507	13.0492	9.05E-08	3.57E-06	7.76182
NFKBIZ	0.81137	2.23157	14.5431	3.10E-08	1.45E-06	8.92075
CXADRP3	0.81199	0.5045	7.86685	1.09E-05	0.00022	2.57653
EBF3	0.81423	0.40711	7.20839	2.38E-05	0.00044	1.73849

SERPING	0.81488	2.47005	7.79167	1.19E-05	0.00024	2.4834
SESN3	0.81499	0.57416	6.63934	4.85E-05	0.00079	0.97172
FLG-AS1	0.81502	0.68771	7.54753	1.58E-05	0.00031	2.17649
KIF25	0.81513	0.40756	5.39091	0.00027	0.00352	-0.86
HOXB7	0.81559	1.36219	7.29086	2.15E-05	0.0004	1.84628
RNF144B	0.81604	0.45819	10.8695	5.35E-07	1.63E-05	5.83777
TTYH1	0.81679	1.07538	5.34935	0.00029	0.0037	-0.9246
MYL7	0.81776	0.40888	25.2835	1.16E-10	1.64E-08	14.9276
MYOT	0.81869	0.7644	7.49875	1.68E-05	0.00033	2.11433
BCAN	0.81888	0.55996	5.90023	0.00013	0.00187	-0.0872
COLEC11	0.81907	0.78541	4.59951	0.00089	0.00972	-2.1307
SYT4	0.81952	0.97666	5.97182	0.00012	0.00172	0.01862
HLA-DRB	0.81991	0.56047	9.86424	1.35E-06	3.67E-05	4.83644
LINC0238	0.82071	0.41036	10.8838	5.28E-07	1.61E-05	5.8515
USP51	0.82095	2.05106	18.4069	2.93E-09	2.09E-07	11.4691
SLC16A4	0.82252	1.76536	10.0353	1.15E-06	3.17E-05	5.01265
CNTN6	0.82314	0.96731	11.9688	2.11E-07	7.38E-06	6.84653
COL9A1	0.82408	0.41204	11.3183	3.62E-07	1.17E-05	6.25984
LINC0177	0.82433	0.41216	11.4664	3.20E-07	1.05E-05	6.39591
ATCAY	0.82467	0.56285	6.50649	5.76E-05	0.00092	0.78675
GPR84	0.82527	0.85188	7.30772	2.11E-05	0.0004	1.8682
CLDN6	0.8255	0.66361	6.95641	3.25E-05	0.00057	1.404
PDCD4-A	0.82559	0.83885	6.11056	9.77E-05	0.00145	0.22163
IGF1R	0.82601	1.68305	9.49525	1.93E-06	4.97E-05	4.44776
TEX11	0.82855	0.51462	9.08386	2.92E-06	7.13E-05	3.99987
AGPAT4	0.8289	1.6934	7.23208	2.31E-05	0.00043	1.76954
SIPA1L2	0.82962	0.6742	6.92283	3.38E-05	0.00059	1.35881
SLC27A6	0.82986	1.08217	8.02988	9.08E-06	0.00019	2.77632
H4C9	0.8322	2.25196	16.6274	8.15E-09	4.76E-07	10.3657
ZSCAN4	0.83481	0.46758	7.27387	2.20E-05	0.00041	1.82413
ATF3	0.83531	1.73592	9.9109	1.29E-06	3.53E-05	4.88475
SPOCK2	0.83637	0.90293	6.67031	4.66E-05	0.00077	1.01451
TCN2	0.8364	0.64824	5.44664	0.00025	0.0033	-0.7737
PDE4C	0.83707	1.37048	7.82654	1.14E-05	0.00023	2.52668
BDNF-AS	0.83941	0.75304	6.56109	5.37E-05	0.00087	0.86305
LINC0220	0.83944	0.54941	7.27418	2.20E-05	0.00041	1.82455
LINC0260	0.84058	1.16148	11.6859	2.66E-07	9.00E-06	6.59485
BEND4	0.84307	2.2779	17.6293	4.52E-09	2.96E-07	11.0002
TBX15	0.84324	0.42162	14.6892	2.81E-08	1.33E-06	9.02812
CABP1	0.84413	0.99863	7.22551	2.33E-05	0.00043	1.76093
FCGR2A	0.8458	0.47307	10.393	8.22E-07	2.38E-05	5.37322
PCDH19	0.84597	1.75866	11.8963	2.24E-07	7.75E-06	6.78256
HMGB3P	0.84711	0.42355	12.184	1.77E-07	6.36E-06	7.03453
TSGA10	0.84732	1.22676	15.2458	1.94E-08	9.72E-07	9.42839
HOXD-AS	0.84754	0.42377	8.69252	4.40E-06	0.0001	3.55882
SH3BGRL	0.84877	2.01549	16.0285	1.18E-08	6.46E-07	9.96879
LINC0298	0.84982	0.65495	5.93176	0.00012	0.0018	-0.0405
FYN	0.85015	1.20456	6.98764	3.12E-05	0.00055	1.44588
MBD3L2E	0.85115	0.42557	14.2423	3.82E-08	1.72E-06	8.69642
SPP1	0.85116	1.29555	12.2939	1.62E-07	5.92E-06	7.12939
SCN3A	0.85139	2.22088	18.9403	2.19E-09	1.63E-07	11.7798
MSRB3-A	0.85237	1.46078	14.8869	2.46E-08	1.18E-06	9.17191
DCC	0.8538	0.47707	11.9168	2.20E-07	7.64E-06	6.80068
SYT16	0.85416	1.35857	11.4803	3.16E-07	1.04E-05	6.40866
ZBTB20	0.85456	0.86653	7.25082	2.26E-05	0.00042	1.79404
ZNF781	0.85504	1.23988	8.81738	3.86E-06	9.06E-05	3.70119
PAX9	0.85525	1.67188	8.73922	4.19E-06	9.72E-05	3.61225
HIF3A	0.85627	0.45748	7.13138	2.61E-05	0.00047	1.6371

DNAH7	0.8568	0.80428	7.35054	2.00E-05	0.00038	1.92375
DISC1	0.85731	1.37814	5.6387	0.00019	0.00258	-0.4796
HLA-DMB	0.85765	0.81586	6.53603	5.55E-05	0.00089	0.82808
IFIT3	0.85866	2.5206	29.1605	2.70E-11	5.44E-09	16.4774
ICA1	0.85894	0.70968	7.92162	1.03E-05	0.00021	2.64399
ETV7	0.85973	0.42986	16.9175	6.85E-09	4.14E-07	10.5531
ZDBF2	0.86069	0.71055	7.04893	2.89E-05	0.00052	1.52773
PIFO	0.86152	0.9997	11.9866	2.08E-07	7.29E-06	6.86225
RASGRP2	0.86204	2.15477	7.6008	1.49E-05	0.00029	2.24404
PLP1	0.86326	0.51115	7.38002	1.93E-05	0.00037	1.96187
LINC0226	0.86358	0.43179	10.8999	5.21E-07	1.59E-05	5.86684
MUC19	0.86496	1.20343	7.77463	1.21E-05	0.00024	2.4622
MBD3L2	0.8651	0.43255	32.1329	9.95E-12	2.72E-09	17.5263
ITGA2	0.86695	1.06021	12.7983	1.10E-07	4.18E-06	7.55542
SLITRK6	0.86905	0.43453	11.2679	3.78E-07	1.21E-05	6.21311
LINC0197	0.86933	0.64388	6.50966	5.74E-05	0.00092	0.79119
IL15	0.87267	0.43634	10.5024	7.44E-07	2.19E-05	5.48147
GCNT1	0.87405	0.53737	7.3554	1.99E-05	0.00038	1.93004
CNKSR2	0.87407	0.71724	11.2784	3.75E-07	1.20E-05	6.22289
RNU1-153	0.87437	0.43718	14.1027	4.21E-08	1.86E-06	8.59085
KCNMA1-	0.87515	0.48775	8.13778	8.04E-06	0.00017	2.90693
PIK3CG	0.87646	0.43823	12.6956	1.19E-07	4.45E-06	7.46997
ITPRID2	0.87732	2.57576	23.4141	2.55E-10	2.98E-08	14.0907
PROB1	0.87869	1.33399	5.31774	0.0003	0.00387	-0.974
GABRB2	0.87982	0.43991	17.8324	4.03E-09	2.68E-07	11.1245
ADGRG1	0.88021	0.72031	8.40547	5.99E-06	0.00013	3.22552
DACT1	0.88115	0.57027	12.1455	1.83E-07	6.53E-06	7.0011
PARM1	0.88268	0.74237	18.0278	3.61E-09	2.47E-07	11.243
KLHL32	0.88335	0.54202	9.39849	2.13E-06	5.37E-05	4.34383
JUP	0.88552	1.88124	4.71913	0.00074	0.00831	-1.9334
CCDC85A	0.88622	0.62297	9.87974	1.33E-06	3.63E-05	4.85252
CASP4LP	0.88631	0.44315	16.4751	8.93E-09	5.13E-07	10.266
MC5R	0.88724	0.49379	8.40624	5.98E-06	0.00013	3.22643
PAX6	0.8925	1.7091	12.8554	1.05E-07	4.04E-06	7.60269
RIOX1	0.89251	2.17453	9.0286	3.10E-06	7.49E-05	3.9385
LINC0030	0.8928	0.69726	6.68898	4.55E-05	0.00076	1.04025
GABRP	0.8928	0.5973	14.2652	3.76E-08	1.69E-06	8.71363
SAP30-DT	0.89324	0.7129	6.79146	3.99E-05	0.00068	1.18072
SLC38A1	0.8942	0.87315	8.59533	4.88E-06	0.00011	3.44693
XKR7	0.8942	0.54744	9.05104	3.03E-06	7.35E-05	3.96347
IGFBP5	0.89421	0.54745	9.77928	1.46E-06	3.92E-05	4.748
BMPR1B	0.89465	0.44732	15.2283	1.96E-08	9.78E-07	9.41605
LINC0099	0.89465	0.44732	12.8266	1.07E-07	4.11E-06	7.5789
PGGT1BP	0.89486	0.46642	9.35954	2.21E-06	5.57E-05	4.30174
INSM2	0.89494	0.62733	7.92371	1.02E-05	0.00021	2.64654
GSAP	0.89677	0.44839	10.4326	7.93E-07	2.31E-05	5.41245
HEATR4	0.89684	1.17346	13.5626	6.19E-08	2.60E-06	8.17311
ELAVL2	0.89728	0.44864	18.3063	3.09E-09	2.18E-07	11.4095
OR5G5P	0.89808	0.44904	12.0584	1.96E-07	6.93E-06	6.92517
PDE11A-A	0.89808	0.44904	10.8575	5.41E-07	1.64E-05	5.82632
EPAS1	0.8984	0.67924	8.68672	4.43E-06	0.0001	3.55217
CASZ1	0.89966	2.13022	6.94874	3.28E-05	0.00057	1.39369
PLEKHA5	0.89983	2.38577	16.7466	7.58E-09	4.51E-07	10.443
TOGARA	0.90005	0.55037	6.45062	6.20E-05	0.00098	0.70826
SUSD1	0.90112	0.86046	5.44608	0.00025	0.0033	-0.7746
HLA-U	0.90324	0.65231	5.00521	0.00048	0.00575	-1.4689
COL1A1	0.90389	1.51239	4.31714	0.0014	0.01403	-2.6034
F2RL1	0.90418	0.6026	9.40942	2.10E-06	5.32E-05	4.3556

GPM6A-D	0.9053	0.50282	12.8866	1.02E-07	3.97E-06	7.62849
CNN3-DT	0.90619	0.92932	9.033	3.08E-06	7.47E-05	3.9434
TLR3	0.90652	2.01454	14.0121	4.49E-08	1.96E-06	8.52179
GIPC3	0.90672	0.78374	6.39695	6.66E-05	0.00104	0.63249
KRT25	0.90825	0.53365	10.7761	5.81E-07	1.76E-05	5.74811
TAGLN	0.91054	2.74918	9.42685	2.07E-06	5.26E-05	4.37437
MX1	0.91217	1.26268	9.38973	2.15E-06	5.41E-05	4.33437
CD24	0.91336	0.89126	8.4777	5.54E-06	0.00012	3.3102
DNAH11	0.91343	0.55706	9.79342	1.44E-06	3.89E-05	4.76277
GCNT4	0.91529	1.70651	12.8897	1.02E-07	3.96E-06	7.63103
TRIM49B	0.91626	0.45813	17.715	4.31E-09	2.84E-07	11.0528
GAD2	0.91627	0.45813	15.4833	1.66E-08	8.59E-07	9.59507
RIPOR3	0.91913	0.78994	8.46011	5.64E-06	0.00013	3.28963
LINC0210	0.91972	0.51003	11.9504	2.14E-07	7.48E-06	6.83034
ZFP69	0.92003	1.62443	21.5729	5.86E-10	5.64E-08	13.1978
GLCCI1	0.92116	1.13642	7.36139	1.98E-05	0.00037	1.9378
TPTEP2	0.92218	1.40578	9.98492	1.20E-06	3.31E-05	4.961
HMGB1P	0.92237	0.46118	17.999	3.67E-09	2.51E-07	11.2255
FOLR1	0.92288	0.56179	11.1416	4.22E-07	1.32E-05	6.09539
USP44	0.92453	0.83814	9.16006	2.71E-06	6.66E-05	4.08403
LINC0169	0.92539	0.79307	9.12848	2.79E-06	6.84E-05	4.04922
FGA	0.92628	0.54266	6.71477	4.40E-05	0.00073	1.07572
NRROS	0.92802	1.02555	4.88298	0.00057	0.00675	-1.666
PPM1L	0.93193	1.5192	8.92572	3.44E-06	8.21E-05	3.82345
PBX3-DT	0.93528	0.61816	8.21539	7.38E-06	0.00016	3.00009
PALMD	0.93616	0.46808	23.2873	2.69E-10	3.11E-08	14.0315
PTPRD-A	0.93683	0.46841	15.3498	1.81E-08	9.21E-07	9.5017
RCBTB2	0.93693	2.07615	27.5669	4.80E-11	8.30E-09	15.8678
FGFBP3	0.93719	2.19011	14.3522	3.54E-08	1.61E-06	8.7789
TAF11L12	0.93741	0.46871	15.7837	1.37E-08	7.39E-07	9.8025
FST	0.93871	0.6862	6.86107	3.66E-05	0.00063	1.27536
DACT3	0.93912	2.17312	10.3361	8.66E-07	2.50E-05	5.31652
FADS2B	0.94015	0.47007	28.4433	3.48E-11	6.71E-09	16.2075
SKAP1	0.9403	0.47015	8.56057	5.06E-06	0.00011	3.40667
NUAK2	0.94191	1.23804	6.80375	3.93E-05	0.00067	1.19747
LRRC8C	0.94199	1.79328	17.0728	6.25E-09	3.82E-07	10.6521
HMGN1P	0.94285	0.47143	21.3231	6.60E-10	6.19E-08	13.0708
KIAA1217	0.94334	1.3201	10.6492	6.51E-07	1.94E-05	5.62516
ATP1B2	0.94337	1.17591	9.1785	2.66E-06	6.55E-05	4.10431
PRDM8-A	0.94338	0.47169	21.5775	5.85E-10	5.64E-08	13.2001
MAP7D2	0.94352	2.29157	15.2721	1.91E-08	9.58E-07	9.447
F3	0.94368	0.62235	8.41014	5.96E-06	0.00013	3.23102
GNA14	0.94468	0.47234	23.7854	2.17E-10	2.68E-08	14.2622
WFDC1	0.94468	0.52251	12.2813	1.64E-07	5.96E-06	7.11859
DCN	0.94551	1.638	13.4513	6.71E-08	2.79E-06	8.08518
SHISAL2B	0.94605	1.31174	8.13793	8.04E-06	0.00017	2.90711
TAMALIN	0.94667	1.3433	5.38701	0.00027	0.00353	-0.8661
ATP11B	0.94817	2.67639	21.0594	7.49E-10	6.84E-08	12.9352
SSPN	0.94835	0.72503	10.9164	5.13E-07	1.57E-05	5.88262
CD48	0.94857	0.47429	13.6396	5.85E-08	2.47E-06	8.23356
RNF217	0.95186	1.00233	8.08786	8.51E-06	0.00018	2.84665
SERPINB	0.95217	0.90213	9.41798	2.09E-06	5.29E-05	4.36482
GDNF	0.95244	0.47622	54.7908	4.04E-14	5.35E-11	23.1124
SERTM1	0.95377	0.47689	12.2842	1.64E-07	5.96E-06	7.12107
CRNDE	0.95439	0.77823	9.43239	2.06E-06	5.24E-05	4.38033
ONECUT	0.95504	1.77679	15.608	1.53E-08	8.07E-07	9.68165
LINC0123	0.95598	0.52816	11.0417	4.60E-07	1.43E-05	6.00146
EN1	0.9573	0.47865	9.10678	2.86E-06	6.98E-05	4.02524

SPAG6	0.95751	0.5791	13.0505	9.04E-08	3.57E-06	7.76288
DYNC1I1	0.95824	0.47912	10.0765	1.10E-06	3.08E-05	5.05469
PLAU	0.95843	1.7854	16.5714	8.43E-09	4.89E-07	10.3291
PDZD2	0.95865	1.42374	8.56425	5.04E-06	0.00011	3.41093
CDH19	0.95875	0.47938	19.8447	1.37E-09	1.10E-07	12.2877
LINGO2	0.95891	0.62997	11.505	3.09E-07	1.02E-05	6.43116
ACOT12	0.95921	0.4796	22.3964	4.01E-10	4.28E-08	13.6063
SLC8A2	0.95957	1.03053	7.95237	9.91E-06	0.00021	2.68171
ZNF516-D	0.96263	0.53148	9.13549	2.77E-06	6.80E-05	4.05695
PRKCH-A	0.96291	0.48145	13.5201	6.38E-08	2.67E-06	8.13963
CALCB	0.96514	0.53274	12.0941	1.90E-07	6.77E-06	6.95636
LINC0168	0.96551	0.48275	10.9493	4.99E-07	1.53E-05	5.91394
GPR158	0.96732	1.02621	9.41966	2.08E-06	5.28E-05	4.36663
CDH2	0.96735	2.34853	18.4623	2.84E-09	2.03E-07	11.5018
GABRA4	0.96742	0.53388	10.098	1.08E-06	3.02E-05	5.07658
TRIM64	0.96781	0.4839	12.7702	1.12E-07	4.25E-06	7.53213
ENOX1	0.9692	0.53477	13.3857	7.05E-08	2.89E-06	8.03303
DNM1	0.96988	2.32875	7.4571	1.76E-05	0.00034	2.06104
GRAPL	0.9704	0.65993	8.56421	5.04E-06	0.00011	3.41089
ANXA3	0.97082	0.48541	15.4804	1.67E-08	8.59E-07	9.59308
C19orf84	0.97105	0.63604	7.3705	1.95E-05	0.00037	1.94957
SLC16A1	0.97167	0.74522	6.77746	4.07E-05	0.00069	1.1616
EPHB6	0.97251	0.53643	7.84524	1.12E-05	0.00023	2.54982
TGFA	0.97266	0.66619	9.7981	1.44E-06	3.87E-05	4.76765
MAPK10	0.97356	0.64582	8.45674	5.66E-06	0.00013	3.28569
CYB5A	0.97426	0.48713	14.1922	3.95E-08	1.77E-06	8.65861
MT1F	0.97472	0.81144	7.89465	1.06E-05	0.00022	2.61081
VWA2	0.97611	0.48805	10.4549	7.77E-07	2.27E-05	5.43454
RGS16	0.9779	2.63391	25.465	1.08E-10	1.56E-08	15.0054
HGD	0.97899	0.80758	8.29524	6.76E-06	0.00015	3.09526
GABRA1	0.97903	0.53968	12.8016	1.09E-07	4.18E-06	7.55819
SIX3-AS1	0.97975	0.48988	12.6433	1.23E-07	4.61E-06	7.42615
IRX5	0.9801	0.61974	8.35994	6.29E-06	0.00014	3.17187
TSPAN12	0.98284	1.60605	15.472	1.67E-08	8.61E-07	9.58723
MEF2C-A	0.98304	1.01324	11.1179	4.31E-07	1.34E-05	6.07313
CBR1	0.98319	0.4916	24.4831	1.61E-10	2.15E-08	14.5772
OR5E1P	0.98345	0.5419	11.2474	3.85E-07	1.23E-05	6.19415
DNAJB13	0.98348	0.91779	11.5258	3.04E-07	1.01E-05	6.45006
SYCP2L	0.98349	0.49175	18.4758	2.82E-09	2.02E-07	11.5098
KERA	0.9839	0.84315	16.2833	1.00E-08	5.65E-07	10.1393
CETP	0.98532	0.54283	12.8099	1.09E-07	4.15E-06	7.56505
HCG27	0.98551	0.74361	8.39072	6.09E-06	0.00013	3.20817
MAGI3	0.98605	1.59901	10.3589	8.48E-07	2.45E-05	5.33927
LINC0200	0.98652	0.49326	20.5576	9.57E-10	8.28E-08	12.6723
GALNT13	0.98665	0.88036	6.55968	5.38E-05	0.00087	0.86108
ZIC4-AS1	0.98706	0.49353	9.78388	1.46E-06	3.91E-05	4.75281
RASGEF1	0.98745	0.62342	10.5271	7.27E-07	2.14E-05	5.50572
HLA-H	0.98782	1.52399	5.0171	0.00047	0.00566	-1.4498
LINC0232	0.98819	0.49409	16.2893	1.00E-08	5.64E-07	10.1433
FLNC-AS	0.98949	0.49475	13.7244	5.51E-08	2.34E-06	8.29983
EHHADH	0.98988	0.7335	8.03146	9.06E-06	0.00019	2.77825
RAB3IP	0.99218	1.6676	20.8372	8.34E-10	7.42E-08	12.8195
SAP30	0.99264	1.62263	6.45926	6.13E-05	0.00097	0.72043
TMTC2	0.99513	1.60538	15.2595	1.92E-08	9.64E-07	9.43811
RFPL4AL	0.99521	0.4976	56.6781	2.85E-14	4.19E-11	23.4506
CALCA	0.99561	0.49781	15.0663	2.18E-08	1.07E-06	9.30081
BEND3P1	0.99581	1.14382	9.57491	1.79E-06	4.65E-05	4.53268
FRZB	0.99665	0.59867	12.9837	9.51E-08	3.73E-06	7.70829

RGS2	0.99677	3.31662	24.8546	1.38E-10	1.90E-08	14.7413
KCNH1	0.99722	0.49861	18.308	3.09E-09	2.18E-07	11.4105
BACH2	0.99895	0.49948	27.2448	5.41E-11	9.14E-09	15.7402
RND3	0.99966	0.9599	9.83727	1.39E-06	3.75E-05	4.80844
SSU72P6	1.00085	0.50042	33.208	7.09E-12	2.18E-09	17.8805
GRB14	1.00134	1.44182	10.9893	4.82E-07	1.48E-05	5.95186
MGAT3	1.00182	1.90583	8.95506	3.34E-06	7.99E-05	3.85637
TRIM49C	1.00185	0.50092	11.7702	2.48E-07	8.47E-06	6.67041
GRIK1-AS	1.00272	0.50136	22.7691	3.39E-10	3.77E-08	13.7862
NR6A1	1.00289	2.72648	11.1761	4.09E-07	1.29E-05	6.12769
DACT3-A	1.00307	0.75888	5.95116	0.00012	0.00176	-0.0118
ABCA1	1.00362	2.02829	21.2092	6.97E-10	6.47E-08	13.0124
NPAS4	1.00383	0.68178	11.1911	4.04E-07	1.27E-05	6.14168
SOX5	1.00424	0.68198	9.62544	1.70E-06	4.45E-05	4.58626
MIR2052H	1.00487	0.63213	10.689	6.28E-07	1.88E-05	5.66389
RFPL4A	1.00873	0.50436	33.0699	7.40E-12	2.19E-09	17.8357
ICAM1	1.01003	1.20628	7.42001	1.84E-05	0.00035	2.01341
CCDC194	1.01104	0.53487	21.9481	4.92E-10	4.95E-08	13.3858
JAG1	1.01685	1.68063	12.1141	1.87E-07	6.68E-06	6.9738
BATF2	1.01797	1.75595	6.80286	3.94E-05	0.00067	1.19625
HOXD3	1.01816	0.50908	19.6597	1.50E-09	1.18E-07	12.1857
LINC0017	1.01922	0.91951	5.64079	0.00019	0.00258	-0.4764
SEMA6A-	1.01937	0.50968	27.2142	5.47E-11	9.14E-09	15.728
MESTP3	1.02019	0.5101	20.3838	1.04E-09	8.91E-08	12.5798
TRIM53C	1.02025	0.51012	8.68324	4.44E-06	0.0001	3.54818
KCND2	1.02067	0.51034	14.6397	2.90E-08	1.37E-06	8.99188
LAMA2	1.02099	0.61084	12.882	1.03E-07	3.98E-06	7.62465
ABCA9	1.02132	0.611	12.0419	1.99E-07	7.00E-06	6.9108
UBA7	1.02415	1.88701	7.51762	1.64E-05	0.00032	2.1384
PELO-AS	1.02424	0.51212	16.1687	1.08E-08	5.99E-07	10.0629
INPP5D	1.02459	0.98852	9.73415	1.53E-06	4.06E-05	4.70078
SAA2-SA	1.02555	0.59229	18.8659	2.28E-09	1.69E-07	11.7371
FBLN2	1.02717	0.56376	9.46173	2.00E-06	5.12E-05	4.41184
TRIM71	1.0288	2.62254	15.4123	1.74E-08	8.91E-07	9.54554
C10orf55	1.03038	0.89574	8.83768	3.77E-06	8.92E-05	3.72419
PARP9	1.0318	2.67083	17.0071	6.49E-09	3.96E-07	10.6103
PTH1R	1.03196	0.51598	14.7673	2.66E-08	1.27E-06	9.08514
PKD2L2-I	1.03423	0.91382	7.07445	2.80E-05	0.0005	1.56168
ADAM19	1.03707	2.0004	12.2505	1.68E-07	6.07E-06	7.09208
SAA2	1.03925	0.69949	16.2706	1.01E-08	5.68E-07	10.1309
DGKK	1.03941	0.59923	15.3326	1.83E-08	9.30E-07	9.48958
SP140L	1.04027	1.95081	14.11	4.19E-08	1.85E-06	8.5964
IQGAP2	1.04131	1.01271	8.25067	7.10E-06	0.00015	3.04223
SIX1	1.04241	2.746	13.8185	5.15E-08	2.20E-06	8.37292
FAM102B	1.04425	2.07239	18.7611	2.42E-09	1.78E-07	11.6764
GSG1	1.04549	1.23549	14.0667	4.32E-08	1.89E-06	8.56344
PRSS35	1.04615	0.67359	13.1649	8.30E-08	3.31E-06	7.85579
FAM163A	1.04678	0.52339	15.893	1.28E-08	6.98E-07	9.877
NLGN4X	1.04744	2.38069	26.1456	8.25E-11	1.28E-08	15.2925
HRC	1.04767	1.13887	6.32083	7.36E-05	0.00114	0.52435
LRFN5-D	1.04812	0.52406	32.8155	8.01E-12	2.31E-09	17.7527
ANGPTL1	1.04869	0.57452	13.8257	5.12E-08	2.19E-06	8.37849
GATM	1.04908	1.37002	16.5432	8.57E-09	4.96E-07	10.3107
SLC15A3	1.04994	1.62907	10.6514	6.50E-07	1.94E-05	5.62734
PURG	1.05133	0.70553	10.3317	8.70E-07	2.50E-05	5.31217
LINC0127	1.05155	0.52577	12.5216	1.36E-07	5.00E-06	7.32361
PCGF5	1.05182	1.24332	14.9936	2.29E-08	1.11E-06	9.24879
CPA4	1.05212	0.57623	13.4868	6.54E-08	2.73E-06	8.11328

ZNF493	1.05295	0.78272	11.552	2.97E-07	9.93E-06	6.4739
SLC44A5	1.05498	0.83705	9.50801	1.91E-06	4.91E-05	4.4614
INSIG2	1.05518	2.41921	17.4651	4.97E-09	3.20E-07	10.8986
PTPRK	1.05542	0.6574	10.827	5.56E-07	1.68E-05	5.79707
RMST	1.05595	0.69464	8.25586	7.06E-06	0.00015	3.04842
EGLN3	1.0565	0.70811	12.2793	1.64E-07	5.97E-06	7.1169
CD53	1.05831	0.57933	14.5095	3.17E-08	1.48E-06	8.89588
PIM1	1.05845	2.11995	12.7345	1.15E-07	4.35E-06	7.50241
CHST9	1.05858	0.52929	21.8263	5.21E-10	5.16E-08	13.3251
DHRS2	1.05917	1.22618	9.54862	1.83E-06	4.74E-05	4.50472
SHC4	1.06099	1.05689	10.7411	6.00E-07	1.81E-05	5.71429
DBX2	1.06131	0.53066	34.5574	4.71E-12	1.70E-09	18.3081
RFPL4B	1.06232	0.53116	16.2939	9.98E-09	5.63E-07	10.1464
CCR3	1.06248	0.53124	10.3946	8.21E-07	2.38E-05	5.37479
ST8SIA1	1.06255	0.81148	9.77962	1.46E-06	3.92E-05	4.74837
PDGFRB	1.06402	1.27178	6.7954	3.97E-05	0.00067	1.18609
SLC44A1	1.06647	2.88284	18.6295	2.59E-09	1.89E-07	11.5999
PLAAT4	1.06766	1.49743	22.5856	3.68E-10	3.98E-08	13.698
CHRDL1	1.06786	0.66362	10.4718	7.65E-07	2.25E-05	5.4513
SLITRK4	1.06862	0.664	11.4381	3.27E-07	1.07E-05	6.36999
AMOTL1	1.06878	1.44487	13.3647	7.16E-08	2.93E-06	8.01626
PNMA3	1.06897	0.92356	8.52885	5.24E-06	0.00012	3.36983
NELL2	1.0706	1.40546	11.7508	2.52E-07	8.58E-06	6.65308
TENM1	1.07066	0.53533	27.7952	4.41E-11	7.92E-09	15.9574
DHRS3	1.07139	1.14924	9.74507	1.51E-06	4.03E-05	4.71222
MLXP1	1.0717	0.58602	15.5397	1.60E-08	8.37E-07	9.63433
LMO3	1.07228	1.21954	18.4461	2.87E-09	2.05E-07	11.4923
IRX3	1.07315	0.6161	10.6114	6.74E-07	2.00E-05	5.58828
LINC0103	1.07413	0.53706	25.1959	1.20E-10	1.69E-08	14.8898
CPNE8-A	1.07547	0.96846	8.92664	3.44E-06	8.20E-05	3.82449
LINC0182	1.07766	0.84839	11.3025	3.67E-07	1.18E-05	6.24518
GABRG2	1.08051	0.59043	13.0684	8.92E-08	3.53E-06	7.77744
EPDR1	1.0807	0.54035	12.5957	1.28E-07	4.75E-06	7.38619
IZUMO3	1.08103	0.54051	20.1393	1.18E-09	9.77E-08	12.4483
GABRA2	1.08153	0.69128	8.9991	3.19E-06	7.69E-05	3.90561
FLRT2	1.0817	1.15144	14.1342	4.12E-08	1.82E-06	8.61477
KCTD16	1.08243	1.66002	17.7449	4.24E-09	2.81E-07	11.0711
MPZL2	1.08259	0.64164	13.0062	9.35E-08	3.68E-06	7.72667
BRINP3-D	1.08335	0.59184	13.3292	7.35E-08	3.00E-06	7.98795
FES	1.08586	0.79379	7.11197	2.68E-05	0.00048	1.61143
CAVIN2	1.0862	0.59327	13.3885	7.03E-08	2.89E-06	8.03527
SPX	1.08658	0.95614	8.63026	4.70E-06	0.00011	3.48725
ACTG2	1.08774	0.61735	13.8088	5.18E-08	2.21E-06	8.36541
P2RY2	1.08789	0.59412	9.42409	2.07E-06	5.27E-05	4.3714
TMTC1	1.08809	1.34098	20.9906	7.74E-10	6.99E-08	12.8995
GPHA2	1.08868	0.92022	8.03167	9.06E-06	0.00019	2.77849
TRIM49	1.08875	0.54437	22.2445	4.29E-10	4.48E-08	13.5321
SUCNR1	1.09037	0.98443	9.74741	1.51E-06	4.02E-05	4.71467
GRID2	1.09536	0.54768	23.0052	3.05E-10	3.44E-08	13.8987
TMEM35A	1.09549	0.69826	9.46419	1.99E-06	5.11E-05	4.41449
NKX2-1-A	1.09616	0.54808	13.1495	8.40E-08	3.34E-06	7.84328
QRFPR	1.09666	0.54833	35.4316	3.64E-12	1.46E-09	18.5756
SH3BP4	1.09746	2.14998	16.4507	9.07E-09	5.19E-07	10.25
DIO3OS	1.09781	0.5621	21.996	4.81E-10	4.87E-08	13.4095
SHC2	1.09845	0.94593	6.69312	4.53E-05	0.00075	1.04595
SOX9-AS	1.09874	0.54937	14.4342	3.34E-08	1.54E-06	8.84001
TM4SF1	1.10116	0.6301	12.5905	1.29E-07	4.77E-06	7.38178
LTF	1.10137	0.65103	11.9115	2.21E-07	7.67E-06	6.79606

THBS1-A	1.1015	0.68044	11.5891	2.88E-07	9.67E-06	6.50752
TRIM49D	1.1015	0.55075	31.4269	1.25E-11	3.05E-09	17.2868
CXXC4	1.10326	2.98989	18.5411	2.72E-09	1.97E-07	11.5481
HOATZ	1.10439	0.5522	14.2288	3.85E-08	1.73E-06	8.68623
ITGA4	1.10477	0.60256	16.8013	7.34E-09	4.40E-07	10.4784
TRIM49D	1.10504	0.55252	33.1534	7.21E-12	2.19E-09	17.8628
HOXB8	1.10604	1.23683	8.82926	3.81E-06	8.98E-05	3.71465
CDH10	1.10758	1.8855	18.5296	2.74E-09	1.98E-07	11.5414
ELOVL2	1.10925	0.68432	12.0893	1.91E-07	6.79E-06	6.95223
UTS2B	1.11034	1.20262	12.0689	1.94E-07	6.89E-06	6.93439
GRIN3A	1.11153	1.86876	15.5319	1.61E-08	8.38E-07	9.62888
KRT28	1.11333	0.55666	20.0028	1.26E-09	1.02E-07	12.3742
MEIS3	1.11442	2.34604	7.43993	1.80E-05	0.00034	2.03901
RTP4	1.11475	0.65772	15.7014	1.45E-08	7.72E-07	9.74602
LGR5	1.11585	1.42272	9.64287	1.67E-06	4.38E-05	4.6047
TMEM74	1.1161	0.70856	14.6653	2.85E-08	1.35E-06	9.01059
AQP9	1.11721	0.5586	16.1473	1.09E-08	6.05E-07	10.0486
FOXL2NE	1.11727	1.59902	11.2702	3.78E-07	1.21E-05	6.21523
ERVV-2	1.11843	0.65956	12.064	1.95E-07	6.90E-06	6.93009
RBM47	1.12377	1.22354	11.8116	2.40E-07	8.22E-06	6.70739
HLA-DPA	1.12387	0.72257	13.436	6.79E-08	2.81E-06	8.07302
HNF4G	1.12497	1.67324	16.9862	6.58E-09	4.01E-07	10.597
LRP2	1.12668	0.79337	13.1545	8.36E-08	3.33E-06	7.84731
SYT1	1.12827	0.77334	10.7241	6.09E-07	1.83E-05	5.69789
RNF180	1.1325	0.74611	12.2009	1.75E-07	6.30E-06	7.0492
DPYD-AS	1.13311	0.56655	20.1085	1.20E-09	9.89E-08	12.4316
S1PR1	1.13921	1.21898	11.9837	2.08E-07	7.30E-06	6.85966
ANKRD30	1.13934	0.59902	18.3474	3.03E-09	2.15E-07	11.434
SERPINA	1.13937	0.56969	9.75671	1.50E-06	3.99E-05	4.7244
NXPH1	1.13958	0.61996	12.3591	1.54E-07	5.64E-06	7.18536
MMRN2	1.1399	1.0497	6.6921	4.53E-05	0.00075	1.04454
LINC0287	1.1407	0.62052	9.37669	2.17E-06	5.48E-05	4.32029
LINC0279	1.14589	0.57984	30.8052	1.53E-11	3.50E-09	17.0711
DIO3	1.15072	0.70505	11.6574	2.72E-07	9.20E-06	6.56926
ROCR	1.15128	0.57564	20.68	9.01E-10	7.94E-08	12.737
FADS2	1.15138	2.9574	10.5524	7.11E-07	2.10E-05	5.53062
LIPK	1.1519	0.57595	20.08	1.21E-09	9.93E-08	12.4161
CCN1	1.15305	1.97948	17.5089	4.85E-09	3.15E-07	10.9258
TM4SF18	1.15333	0.57667	20.0809	1.21E-09	9.93E-08	12.4166
GRIA3	1.15557	0.94047	8.95203	3.35E-06	8.01E-05	3.85298
PABPC5	1.15686	0.57843	19.465	1.66E-09	1.29E-07	12.0773
OR5AK2	1.15695	0.57847	13.5046	6.46E-08	2.70E-06	8.12736
LINC0109	1.15784	0.57892	14.7347	2.72E-08	1.29E-06	9.06137
CDH6	1.15842	0.62938	13.9783	4.59E-08	2.00E-06	8.49597
LINC0115	1.15918	0.57959	17.4623	4.98E-09	3.20E-07	10.8969
LGALS3B	1.15984	2.28309	7.89283	1.06E-05	0.00022	2.60856
PDZRN4	1.16005	0.58002	36.6497	2.57E-12	1.16E-09	18.9365
INA	1.16303	2.48044	22.655	3.56E-10	3.90E-08	13.7314
GNG8	1.16655	0.58328	12.792	1.10E-07	4.19E-06	7.55025
APELA	1.16738	0.58369	45.6051	2.69E-13	2.23E-10	21.2387
NLRC5	1.1681	2.45553	12.9127	1.00E-07	3.90E-06	7.64997
IL32	1.16815	0.6636	11.7722	2.48E-07	8.47E-06	6.67221
CD69	1.16854	0.73478	13.2435	7.83E-08	3.14E-06	7.9192
SLC66A1	1.16974	0.63504	13.3812	7.07E-08	2.90E-06	8.02939
MAOA	1.17202	0.75268	9.88299	1.33E-06	3.62E-05	4.85588
MYOCOS	1.17437	1.10038	9.37038	2.19E-06	5.51E-05	4.31346
MAP1LC3	1.1757	0.66737	9.59067	1.76E-06	4.58E-05	4.54943
CYSLTR2	1.17723	1.36586	17.6656	4.43E-09	2.91E-07	11.0225

SFTA3	1.17841	0.5892	20.0612	1.23E-09	9.96E-08	12.4059
TENM3	1.17972	0.76972	10.8202	5.59E-07	1.69E-05	5.79054
LINC0046	1.18184	0.59092	25.4962	1.07E-10	1.55E-08	15.0188
TGFB2	1.18343	0.8379	9.42147	2.08E-06	5.28E-05	4.36858
CCDC80	1.18557	2.03365	15.5135	1.63E-08	8.46E-07	9.61608
RFPL1	1.1858	0.62225	18.0917	3.49E-09	2.40E-07	11.2814
NLGN4Y	1.18582	0.98198	15.4503	1.70E-08	8.71E-07	9.57209
NID2	1.18649	1.9457	15.2435	1.94E-08	9.72E-07	9.42681
PDLIM5	1.18738	2.22277	32.8916	7.82E-12	2.29E-09	17.7776
SLC16A3	1.18837	1.26922	8.60777	4.81E-06	0.00011	3.4613
TRIM22	1.18971	1.07108	16.4979	8.81E-09	5.09E-07	10.281
TP53INP1	1.19056	1.6321	16.2001	1.06E-08	5.91E-07	10.0839
GFY	1.19228	1.56664	11.389	3.41E-07	1.11E-05	6.32501
PDE11A	1.19323	0.89765	9.43583	2.05E-06	5.23E-05	4.38403
HLA-A	1.19338	1.2328	4.91913	0.00054	0.00643	-1.6075
ARX	1.19407	0.59703	9.45323	2.02E-06	5.15E-05	4.40273
HOXD10	1.19752	0.59876	15.1499	2.07E-08	1.02E-06	9.36046
HLA-B	1.19772	3.00235	12.2439	1.69E-07	6.10E-06	7.08632
SLC34A2	1.19928	0.59964	15.8773	1.29E-08	7.04E-07	9.86631
ARPP21	1.19943	0.59972	30.6278	1.63E-11	3.61E-09	17.0087
DSPP	1.20072	0.80105	15.8705	1.30E-08	7.05E-07	9.86169
KHDC1L	1.20102	0.7302	13.0104	9.32E-08	3.67E-06	7.73012
CCDC120	1.20233	1.25988	11.5105	3.08E-07	1.02E-05	6.43613
TANC1	1.20241	2.31885	17.2551	5.61E-09	3.51E-07	10.7673
CNTN1	1.20419	0.60209	45.135	3.00E-13	2.38E-10	21.1311
NKX3-2	1.20505	0.65269	10.6519	6.50E-07	1.94E-05	5.6278
FIRRE	1.20682	1.27565	15.1334	2.09E-08	1.03E-06	9.34867
CNTN4-A	1.20834	0.60417	43.0156	4.93E-13	3.49E-10	20.6294
GOLGA8S	1.21053	1.17109	17.9013	3.88E-09	2.59E-07	11.1664
LRRTM4	1.21143	2.14219	20.8536	8.28E-10	7.39E-08	12.8281
ZNF385D	1.21153	1.05617	9.92987	1.27E-06	3.47E-05	4.90434
CAPS2	1.21208	0.65621	21.3387	6.55E-10	6.15E-08	13.0788
INPP1	1.21266	0.60633	19.6092	1.54E-09	1.21E-07	12.1577
TNMD	1.21642	0.86759	12.5156	1.36E-07	5.02E-06	7.31853
KCNJ2	1.21732	1.50626	19.6049	1.55E-09	1.21E-07	12.1553
SH2D2A	1.21835	0.70952	10.2572	9.32E-07	2.66E-05	5.23755
FBN2	1.2195	2.7221	19.0431	2.08E-09	1.56E-07	11.8388
DGKB	1.22353	0.79853	10.8975	5.22E-07	1.59E-05	5.86458
RERGL	1.22393	1.04917	9.54349	1.84E-06	4.76E-05	4.49926
PTX3	1.22439	0.66237	19.2192	1.89E-09	1.45E-07	11.939
MSR1	1.22531	0.98001	16.4779	8.92E-09	5.13E-07	10.2679
PRKCH	1.22565	2.18142	16.9159	6.85E-09	4.14E-07	10.552
SOCS3-D	1.22755	0.66395	14.3486	3.55E-08	1.61E-06	8.77619
OSTN	1.22795	2.38552	31.4379	1.25E-11	3.05E-09	17.2906
FLG	1.22873	0.9154	8.52084	5.29E-06	0.00012	3.36051
DSEL	1.2302	2.01474	30.7447	1.57E-11	3.53E-09	17.0498
MYO3B	1.23147	0.61573	10.764	5.88E-07	1.77E-05	5.73645
CTTNBP2	1.23176	0.79574	13.444	6.75E-08	2.80E-06	8.07939
LINC0166	1.23377	1.18128	13.4396	6.77E-08	2.81E-06	8.07589
LINC0127	1.2338	0.6169	15.1514	2.06E-08	1.02E-06	9.36146
PLAG1	1.23382	1.57289	17.7227	4.29E-09	2.83E-07	11.0575
IL21R-AS	1.23524	1.08885	6.85463	3.69E-05	0.00063	1.26663
LGR4	1.23582	2.80342	29.7314	2.21E-11	4.64E-09	16.6874
UACA	1.23596	3.39315	27.725	4.52E-11	8.02E-09	15.9299
SULT2A1	1.23677	0.66856	21.8227	5.22E-10	5.16E-08	13.3233
TECRL	1.23701	0.92806	14.2193	3.88E-08	1.74E-06	8.67909
MBP	1.23891	0.79932	10.307	8.90E-07	2.55E-05	5.28753
BEX4	1.24055	0.62028	19.516	1.62E-09	1.26E-07	12.1058

CXXC4-A	1.24169	1.27693	10.0925	1.09E-06	3.04E-05	5.07104
MPPED2	1.24649	0.86018	12.7094	1.17E-07	4.42E-06	7.48149
CPXM1	1.24743	0.62371	16.2335	1.04E-08	5.80E-07	10.1062
MPZ	1.24821	0.72445	11.5614	2.95E-07	9.88E-06	6.48246
BMPR1B-	1.2484	0.6242	35.2586	3.83E-12	1.51E-09	18.5232
FOLH1	1.24855	1.07968	10.795	5.72E-07	1.73E-05	5.7663
OLFML1	1.24954	1.83069	15.2847	1.89E-08	9.52E-07	9.45585
ALX4	1.25052	1.08533	12.8574	1.05E-07	4.03E-06	7.60435
XAF1	1.2517	1.96964	14.1446	4.09E-08	1.82E-06	8.62267
LBX1-AS1	1.25301	0.6265	12.6943	1.19E-07	4.45E-06	7.46888
ACTA2	1.25547	2.32756	36.9188	2.38E-12	1.11E-09	19.0145
BCL6	1.25664	1.35357	7.48099	1.71E-05	0.00033	2.09163
ZDHHC2	1.25716	2.6513	30.8324	1.52E-11	3.49E-09	17.0806
VANGL2	1.25763	2.04017	9.77493	1.47E-06	3.94E-05	4.74346
NHSL2	1.26102	2.10021	16.3468	9.66E-09	5.48E-07	10.1814
PDE4DIP	1.26676	0.77707	14.4153	3.39E-08	1.56E-06	8.82598
ATP6V0D	1.26692	0.83415	11.9099	2.21E-07	7.67E-06	6.79464
LBX1	1.26864	0.63432	11.1364	4.24E-07	1.32E-05	6.09049
APOL1	1.27019	1.90175	13.6759	5.70E-08	2.41E-06	8.26199
GBP2	1.27193	0.73631	12.9692	9.62E-08	3.77E-06	7.69639
RPA4	1.27429	1.51829	15.8458	1.32E-08	7.14E-07	9.84487
RARB	1.27942	1.18783	18.2252	3.24E-09	2.27E-07	11.3613
ZFP42	1.28024	0.81999	13.497	6.49E-08	2.71E-06	8.12136
MIR222H(	1.28038	0.9204	15.9763	1.22E-08	6.66E-07	9.9335
ACTA2-A	1.28165	1.91157	20.2367	1.12E-09	9.45E-08	12.5008
TCIM	1.2826	0.6413	22.7536	3.41E-10	3.78E-08	13.7788
ABCD2	1.28285	0.79194	12.3614	1.54E-07	5.63E-06	7.18737
SALRNA1	1.28294	0.988	9.87563	1.33E-06	3.64E-05	4.84825
MARCKS	1.28384	0.74226	13.324	7.37E-08	3.00E-06	7.98376
VSNL1	1.284	0.74234	15.2005	2.00E-08	9.95E-07	9.39637
PRAL	1.28857	1.77139	11.1677	4.12E-07	1.30E-05	6.11979
ELF3	1.28882	0.82428	11.0721	4.48E-07	1.40E-05	6.03014
DDX60	1.28971	2.2388	23.5653	2.39E-10	2.87E-08	14.1609
LINC0191	1.29111	0.64555	23.5238	2.43E-10	2.91E-08	14.1417
ANKRD1	1.29228	0.90552	13.2004	8.08E-08	3.23E-06	7.88441
CACNA1(	1.2923	1.17608	10.0246	1.16E-06	3.21E-05	5.00164
LINC0037	1.29325	0.64662	35.1478	3.95E-12	1.52E-09	18.4895
DLC1	1.29374	0.84756	10.866	5.37E-07	1.63E-05	5.83441
LINC0038	1.29458	0.64729	21.9962	4.81E-10	4.87E-08	13.4096
CSF2RA	1.29614	0.70836	16.3263	9.78E-09	5.54E-07	10.1679
TMEM156	1.29783	0.82878	12.8609	1.04E-07	4.03E-06	7.60724
CARTPT	1.29847	0.64924	24.3652	1.70E-10	2.22E-08	14.5246
NUPR1	1.29864	1.80136	9.32086	2.30E-06	5.75E-05	4.25981
IKZF3	1.30024	1.07617	14.3873	3.45E-08	1.58E-06	8.80506
FGB	1.30066	0.86717	11.061	4.52E-07	1.41E-05	6.0196
OTOL1	1.30075	0.70055	20.0665	1.22E-09	9.96E-08	12.4088
QPCT	1.30404	0.80253	17.9789	3.71E-09	2.52E-07	11.2134
UBBP2	1.30539	1.10261	13.0032	9.37E-08	3.68E-06	7.72427
PSMB8	1.30704	1.24624	14.4309	3.35E-08	1.54E-06	8.83758
ACTG1P2	1.3091	0.98026	10.9418	5.02E-07	1.54E-05	5.90674
POF1B	1.31008	0.80556	16.8767	7.02E-09	4.22E-07	10.5269
SUN3	1.31206	1.18386	19.1969	1.92E-09	1.46E-07	11.9264
PINCR	1.31254	0.99517	15.4726	1.67E-08	8.61E-07	9.5876
IQCJ-SCH	1.31299	0.70667	18.3897	2.96E-09	2.10E-07	11.459
OSTN-AS	1.3157	1.15489	13.9932	4.55E-08	1.98E-06	8.50736
PRICKLE	1.31675	1.0181	21.2134	6.96E-10	6.47E-08	13.0146
SAMD10	1.32056	0.8108	10.2996	8.96E-07	2.57E-05	5.28006
CRISPLD	1.32142	2.08709	15.9189	1.26E-08	6.88E-07	9.89459

RGL1	1.32153	1.67676	21.7071	5.51E-10	5.38E-08	13.2654
EPS8	1.32303	1.3009	9.17276	2.67E-06	6.58E-05	4.098
TNIP3	1.32464	0.66232	49.8829	1.07E-13	1.01E-10	22.1622
FAM241A	1.3275	0.83249	16.3043	9.92E-09	5.61E-07	10.1532
GBP1P1	1.33087	0.76578	19.8413	1.37E-09	1.10E-07	12.2859
DTNA	1.33134	1.12574	20.2921	1.09E-09	9.25E-08	12.5306
RPS6KA6	1.3324	1.23514	21.9128	5.00E-10	5.02E-08	13.3682
ZNF66	1.33413	0.77284	25.9206	9.01E-11	1.36E-08	15.1984
B3GALT1	1.33542	0.66771	23.3065	2.67E-10	3.09E-08	14.0405
SGCE	1.33675	0.66837	23.0419	3.00E-10	3.42E-08	13.916
HOXD8	1.3371	0.66855	19.7537	1.43E-09	1.14E-07	12.2377
ENO2	1.3373	1.70403	13.2652	7.70E-08	3.10E-06	7.93659
KCNJ3	1.3422	0.75062	13.1118	8.64E-08	3.43E-06	7.8127
ITM2A	1.34437	1.28722	12.1689	1.79E-07	6.42E-06	7.02148
CCRL2	1.34679	0.72357	21.157	7.15E-10	6.60E-08	12.9856
CARD17F	1.34852	0.67426	25.6493	1.00E-10	1.47E-08	15.0839
CXCL10	1.35266	0.7265	19.2147	1.90E-09	1.45E-07	11.9364
C3orf70	1.35267	0.7265	21.3479	6.52E-10	6.15E-08	13.0835
HTATIP2	1.35378	0.67689	21.2611	6.80E-10	6.35E-08	13.0391
SLC7A8	1.35543	0.84438	11.5994	2.86E-07	9.60E-06	6.5169
SULF1	1.35601	0.77835	12.2768	1.65E-07	5.98E-06	7.11473
PDE3A	1.35713	1.38949	21.4218	6.30E-10	6.00E-08	13.1212
HFM1	1.35824	0.67912	21.0351	7.58E-10	6.88E-08	12.9225
TMCC3	1.36013	1.56151	17.8574	3.98E-09	2.65E-07	11.1397
IFITM1	1.36338	2.68484	11.7311	2.56E-07	8.71E-06	6.63549
KCNJ16	1.36456	0.73245	23.1814	2.82E-10	3.23E-08	13.9818
CDCA7L	1.36594	0.73314	19.1537	1.96E-09	1.49E-07	11.9018
IRF8	1.36847	0.73441	22.3945	4.01E-10	4.28E-08	13.6053
PCDH9	1.37007	2.3861	31.1359	1.38E-11	3.25E-09	17.1864
ABCA10	1.37098	0.81051	18.6671	2.54E-09	1.86E-07	11.6218
SCRG1	1.373	0.7003	22.3486	4.09E-10	4.35E-08	13.5829
SIX6	1.37651	0.68825	20.6451	9.17E-10	8.04E-08	12.7186
PSMB8-A	1.37783	2.14312	15.0342	2.23E-08	1.09E-06	9.27784
TRIM53B	1.37792	0.68896	65.0072	6.89E-15	1.82E-11	24.7904
CCR5	1.37857	0.68928	27.5863	4.76E-11	8.30E-09	15.8755
THSD7B	1.38013	0.69006	14.4116	3.39E-08	1.56E-06	8.8232
TFPI2	1.38073	0.79071	17.1262	6.05E-09	3.73E-07	10.686
GIMAP2	1.38091	1.29138	12.9454	9.79E-08	3.82E-06	7.67686
TFEC	1.38163	0.82051	13.0861	8.80E-08	3.48E-06	7.79182
LINC0300	1.38165	1.26272	25.4468	1.09E-10	1.56E-08	14.9976
NEDD9	1.38509	0.90939	16.2264	1.04E-08	5.82E-07	10.1014
GABRB1	1.38778	0.74406	20.1365	1.18E-09	9.77E-08	12.4468
LINC0138	1.38795	0.69397	23.4038	2.56E-10	2.98E-08	14.0859
IL26	1.38943	0.74488	26.1833	8.13E-11	1.27E-08	15.3081
NOS1AP	1.38994	0.84548	11.9256	2.18E-07	7.60E-06	6.80849
ACTN3	1.39016	2.21397	10.2023	9.80E-07	2.78E-05	5.18231
PPP1R3B	1.39251	1.44992	16.4433	9.11E-09	5.21E-07	10.2451
LINC0057	1.39288	0.69644	28.1418	3.88E-11	7.31E-09	16.0919
APOE	-1.3951	1.91431	8.51791	5.30E-06	0.00012	3.3571
OAS1	1.39529	0.74781	16.4808	8.90E-09	5.13E-07	10.2698
JPH1	1.39537	1.62381	16.9167	6.85E-09	4.14E-07	10.5526
CDH12	1.39555	1.22479	12.7963	1.10E-07	4.19E-06	7.55379
GBP7	1.39616	0.69808	17.9821	3.71E-09	2.52E-07	11.2154
ADAMTS5	1.39906	0.82922	12.7202	1.16E-07	4.39E-06	7.49047
FOXN4	1.39923	1.40253	10.2367	9.50E-07	2.70E-05	5.21698
MAP2	1.41216	1.72828	15.6217	1.52E-08	8.03E-07	9.69109
TRIM64B	1.41368	0.70684	23.4335	2.53E-10	2.97E-08	14.0997
HOXD4	1.41399	0.75717	19.0315	2.09E-09	1.57E-07	11.8322

SAMD9	1.4144	0.85772	17.453	5.01E-09	3.21E-07	10.891
ZNF516	1.4145	2.14718	12.7312	1.15E-07	4.35E-06	7.49966
PDE4DIP	1.42195	0.84067	13.7781	5.30E-08	2.26E-06	8.3416
B3GALNT	1.4241	0.71205	27.4696	4.97E-11	8.52E-09	15.8294
CTSF	1.42425	0.74147	22.2003	4.38E-10	4.53E-08	13.5104
CDH7	1.43194	0.81631	21.3421	6.54E-10	6.15E-08	13.0805
CRADD-A	1.43748	0.76891	24.5544	1.57E-10	2.11E-08	14.6089
DNAJC15	1.44028	0.72014	54.1278	4.58E-14	5.87E-11	22.9902
LRRTM4-	1.44245	0.72122	41.6442	6.88E-13	4.48E-10	20.2895
PIK3C2G	1.44256	0.82162	20.0797	1.21E-09	9.93E-08	12.416
PCLO	1.4447	1.01108	15.1735	2.03E-08	1.01E-06	9.37716
MIR100H	1.4504	0.87572	19.0943	2.02E-09	1.53E-07	11.868
LINC0171	1.45131	0.8329	17.1841	5.85E-09	3.63E-07	10.7226
POU4F1	1.45366	0.72683	27.8802	4.27E-11	7.75E-09	15.9905
CCN2	1.45435	1.18257	12.2463	1.69E-07	6.09E-06	7.08845
BEX1	1.45481	1.26528	23.788	2.17E-10	2.68E-08	14.2634
TAP1	1.45519	2.73568	14.1713	4.01E-08	1.79E-06	8.64286
NEO1	1.45529	2.21937	18.3128	3.08E-09	2.18E-07	11.4134
TRIM9	1.45751	0.90862	15.3156	1.85E-08	9.38E-07	9.47763
SORCS1	1.45794	0.72897	27.0631	5.79E-11	9.43E-09	15.6675
LINC0086	1.45857	1.47666	20.8712	8.21E-10	7.34E-08	12.8373
RBPMS	1.46307	2.4015	19.7797	1.42E-09	1.13E-07	12.252
GAREM1	1.46372	1.38591	17.1584	5.94E-09	3.68E-07	10.7064
JPH2	1.46427	0.78231	23.0108	3.04E-10	3.44E-08	13.9013
PALLD	1.46526	1.77162	13.2366	7.87E-08	3.16E-06	7.91359
THRB	1.46621	0.73311	22.9089	3.18E-10	3.56E-08	13.8529
ZNF835	1.46709	0.78372	18.5882	2.65E-09	1.93E-07	11.5757
CDH9	1.46805	0.7842	22.2741	4.24E-10	4.43E-08	13.5465
BDNF	1.47153	0.87235	14.7204	2.75E-08	1.30E-06	9.05092
APOL6	1.47656	2.42762	32.9615	7.66E-12	2.25E-09	17.8004
LZTS1	1.47925	1.25282	9.95322	1.24E-06	3.41E-05	4.92841
C1orf21	1.48539	1.07307	12.79	1.10E-07	4.19E-06	7.54857
ZFHX4-A	1.48732	0.74366	88.0112	2.99E-16	2.29E-12	27.5398
RGS18	1.48736	0.74368	28.0532	4.01E-11	7.44E-09	16.0577
TRPS1	1.49107	2.09901	26.9413	6.07E-11	9.72E-09	15.6184
HAND2-A	1.49156	0.74578	14.852	2.52E-08	1.20E-06	9.14664
DOCK10	1.49423	1.10684	16.436	9.15E-09	5.22E-07	10.2403
SLC1A3	1.49463	1.07769	12.7345	1.15E-07	4.35E-06	7.50237
CYP1B1	1.49571	2.88906	27.6672	4.62E-11	8.12E-09	15.9073
COLEC12	1.49791	1.18543	11.4011	3.38E-07	1.10E-05	6.33612
F2RL2	1.49878	1.0296	10.1574	1.02E-06	2.88E-05	5.13694
SCHIP1	1.49946	0.85007	20.3356	1.07E-09	9.09E-08	12.554
EPM2AIP	1.49986	0.76312	35.0362	4.08E-12	1.56E-09	18.4555
LINC0155	1.50108	0.9373	15.0855	2.16E-08	1.06E-06	9.31457
TRIM53A	1.50423	0.75211	33.9044	5.73E-12	1.93E-09	18.1035
LRP1B	1.50452	1.01164	16.3931	9.39E-09	5.35E-07	10.212
CCDC33	1.50492	0.75246	15.8371	1.33E-08	7.16E-07	9.83898
BMPER	1.5053	1.00351	14.8482	2.52E-08	1.21E-06	9.14392
HOXB9	1.5057	1.8692	21.6835	5.57E-10	5.42E-08	13.2535
ZNF439	1.50775	1.526	37.3431	2.12E-12	1.00E-09	19.1361
USH2A	1.5117	1.26405	14.9089	2.42E-08	1.17E-06	9.18782
LINC0085	1.51296	0.836	21.7826	5.32E-10	5.23E-08	13.3033
COL2A1	1.51506	0.96674	11.0216	4.68E-07	1.45E-05	5.98242
OVCH2	1.51587	0.83745	15.6179	1.52E-08	8.03E-07	9.68849
MLLT3	1.51587	0.9378	15.7412	1.41E-08	7.55E-07	9.77334
DEPDC7	1.51595	0.80815	23.7566	2.20E-10	2.70E-08	14.249
ADRB1	1.51632	0.80833	17.3001	5.47E-09	3.44E-07	10.7956
RIC3	1.52246	1.47978	21.7034	5.52E-10	5.38E-08	13.2636

NEFL	1.52276	1.76966	12.6146	1.26E-07	4.69E-06	7.40209
CHODL	1.52287	0.91195	17.7123	4.32E-09	2.84E-07	11.0512
LMO4	1.52721	2.42332	24.3805	1.69E-10	2.21E-08	14.5315
MDGA1	1.5302	1.58172	18.3339	3.05E-09	2.16E-07	11.4259
MESTP1	1.53035	0.76517	31.6167	1.17E-11	2.99E-09	17.3517
KCTD14	1.53137	0.95245	16.4549	9.04E-09	5.18E-07	10.2528
VCAN-AS	1.53399	0.91751	20.0701	1.22E-09	9.96E-08	12.4108
ADAMTS(	1.53465	1.65331	27.1552	5.60E-11	9.19E-09	15.7044
FCGRT	1.539	1.09988	15.3601	1.80E-08	9.18E-07	9.50895
DSG3	1.53923	0.76961	38.5546	1.52E-12	7.86E-10	19.4754
VEPH1	1.53987	0.82011	28.5638	3.33E-11	6.52E-09	16.2533
KRT222	1.54031	1.29655	13.1008	8.71E-08	3.45E-06	7.80378
MLH1	1.5407	0.97104	14.5267	3.14E-08	1.46E-06	8.90862
PHOX2B	1.54254	0.77127	19.7671	1.42E-09	1.13E-07	12.2451
TRHDE-A	1.54419	1.25684	13.7057	5.58E-08	2.36E-06	8.28524
AGT	1.54523	0.77262	36.0589	3.04E-12	1.30E-09	18.7631
BHLHE41	1.54733	0.92418	15.7592	1.39E-08	7.48E-07	9.78572
RERG	1.5485	1.325	19.0271	2.10E-09	1.57E-07	11.8297
FGF5	1.55142	1.40147	12.7916	1.10E-07	4.19E-06	7.54985
PLCB4	1.55172	0.77586	46.3688	2.27E-13	1.92E-10	21.4108
ADD3	1.55424	1.9396	24.2903	1.75E-10	2.27E-08	14.4911
MYH15	1.55524	1.47421	25.2006	1.20E-10	1.69E-08	14.8918
UCHL1	1.55644	2.45832	25.2987	1.16E-10	1.64E-08	14.9341
PRTFDC1	1.55885	0.77943	44.3731	3.57E-13	2.73E-10	20.9539
DSG2	1.55922	1.22123	23.0295	3.02E-10	3.42E-08	13.9102
ANTXR2	1.56181	1.04029	14.434	3.34E-08	1.54E-06	8.83991
POU3F4	1.56302	0.78151	22.0743	4.64E-10	4.75E-08	13.4483
HAS2	1.56466	0.99154	15.1954	2.00E-08	9.97E-07	9.39275
ZNF354C	1.5699	1.46978	19.575	1.57E-09	1.22E-07	12.1387
PGPEP1L	1.5778	0.7889	29.902	2.08E-11	4.45E-09	16.7493
HOXD9	1.57811	0.78905	22.0254	4.75E-10	4.84E-08	13.4241
RBPMS-A	1.58495	0.84265	19.0149	2.11E-09	1.57E-07	11.8226
UGT3A2	1.58805	1.22475	15.5894	1.55E-08	8.14E-07	9.66875
GBP3	1.59051	0.94577	22.7413	3.43E-10	3.79E-08	13.7729
FRRS1	1.59156	1.09681	13.0914	8.77E-08	3.47E-06	7.79617
PRL	1.59199	0.796	36.3656	2.78E-12	1.23E-09	18.8535
ABCA5	1.59389	1.72843	22.0257	4.75E-10	4.84E-08	13.4243
RADX	1.59856	0.89962	21.1543	7.16E-10	6.60E-08	12.9842
NEGR1	1.60071	0.80035	36.6875	2.54E-12	1.16E-09	18.9475
TMEM47	1.60172	1.87597	22.3076	4.17E-10	4.38E-08	13.5629
GAD1	1.60706	1.59063	17.2736	5.55E-09	3.48E-07	10.7789
GCNT2	1.609	1.14136	17.3713	5.25E-09	3.33E-07	10.8401
VMO1	1.60977	1.4686	7.458	1.76E-05	0.00034	2.06219
FOXL2	1.60998	0.95551	11.24	3.88E-07	1.23E-05	6.18727
RFPL1S	1.61123	0.96465	17.7085	4.32E-09	2.84E-07	11.0488
CYP4F35	1.613	0.91821	17.2934	5.49E-09	3.45E-07	10.7914
MDFI	1.6133	0.93634	13.5744	6.14E-08	2.58E-06	8.18242
EPB41L3	1.61416	1.1668	16.716	7.72E-09	4.57E-07	10.4232
OR5AO1P	1.62041	0.86038	26.2048	8.06E-11	1.26E-08	15.3171
SLIT2	1.62263	2.50238	16.7355	7.63E-09	4.53E-07	10.4359
QPRT	1.6258	2.00266	17.4942	4.89E-09	3.15E-07	10.9166
SCIN	1.62957	0.86496	22.308	4.17E-10	4.38E-08	13.5631
IFNG	1.63055	0.86545	25.482	1.07E-10	1.55E-08	15.0127
EFNB2	1.63102	0.99537	16.1412	1.10E-08	6.07E-07	10.0445
NIPAL4	1.63376	1.12643	20.4088	1.03E-09	8.82E-08	12.5931
TARID	1.63661	0.81831	92.19	1.85E-16	2.29E-12	27.9294
ANK3	1.63734	2.54049	27.2012	5.50E-11	9.14E-09	15.7228
IFI16	1.63758	0.94848	18.3292	3.06E-09	2.16E-07	11.4231

MGAT4C	1.63798	0.92623	23.9341	2.04E-10	2.53E-08	14.3301
DLK1	1.64105	0.82053	29.1699	2.69E-11	5.44E-09	16.4809
LMO1	1.6428	1.2049	12.8794	1.03E-07	3.98E-06	7.62252
SAA1	1.64292	0.9805	18.6911	2.51E-09	1.84E-07	11.6357
PCDH20	1.65135	1.32625	19.2969	1.82E-09	1.40E-07	11.9829
MMP8	1.6581	1.55146	21.3708	6.45E-10	6.10E-08	13.0952
SRPX2	1.65908	1.19689	12.6994	1.18E-07	4.45E-06	7.47311
LINC0259	1.65986	0.84892	27.7726	4.45E-11	7.95E-09	15.9486
TPH2	1.66373	0.83186	58.9988	1.88E-14	3.39E-11	23.8478
TRG-AS1	1.66754	0.93411	16.9497	6.72E-09	4.09E-07	10.5737
NR2E1	1.6678	0.8339	28.1248	3.91E-11	7.32E-09	16.0853
SHC3	1.6689	0.83445	31.0372	1.42E-11	3.32E-09	17.1521
SEMA6A	1.67352	2.20829	17.9475	3.78E-09	2.55E-07	11.1944
CCSER1	1.67483	0.85061	33.3531	6.78E-12	2.13E-09	17.9274
SHROOM	1.67592	1.62786	21.6349	5.70E-10	5.53E-08	13.2291
MAP2K6	1.68059	2.15246	45.2693	2.91E-13	2.36E-10	21.1619
PREX2	1.68075	0.89055	26.1151	8.35E-11	1.28E-08	15.2798
MAT1A	1.68199	1.09185	15.2889	1.89E-08	9.51E-07	9.45887
KIF25-AS	1.68296	0.84148	16.1757	1.07E-08	5.97E-07	10.0676
DDAH1	1.6853	1.6619	22.4434	3.92E-10	4.21E-08	13.6291
PYGM	1.68729	1.33306	15.0759	2.17E-08	1.06E-06	9.30773
HAND2	1.68765	0.84383	15.8752	1.30E-08	7.04E-07	9.8649
SNCG	1.69081	1.27145	12.0568	1.96E-07	6.93E-06	6.92377
NAALAD2	1.6944	2.39649	24.0504	1.94E-10	2.44E-08	14.383
NKX2-1	1.69911	0.84955	22.9783	3.08E-10	3.47E-08	13.8859
ALDH1A2	1.70145	1.21045	17.9034	3.87E-09	2.59E-07	11.1677
LINC0109	1.70231	1.55741	23.4929	2.46E-10	2.93E-08	14.1273
POU3F3	1.70386	0.85193	30.3524	1.79E-11	3.90E-09	16.911
TAC1	1.71385	0.85693	60.3728	1.48E-14	2.94E-11	24.0739
GLUL	1.7141	1.74095	33.9872	5.59E-12	1.90E-09	18.1296
BOC	1.719	1.81176	16.7345	7.64E-09	4.53E-07	10.4352
NRP1	1.72054	1.23615	17.4478	5.02E-09	3.22E-07	10.8878
PDGFRL	1.72081	1.62263	14.9842	2.30E-08	1.12E-06	9.242
RIPOR2	1.7247	1.04222	17.1937	5.82E-09	3.62E-07	10.7286
DCLK1	1.72543	0.86272	36.0722	3.03E-12	1.30E-09	18.767
NETO1	1.72657	1.09332	21.1304	7.24E-10	6.64E-08	12.9718
METTL7B	1.72893	1.44456	13.4761	6.59E-08	2.75E-06	8.10481
LINC0255	1.73097	0.86548	57.0693	2.65E-14	4.07E-11	23.5189
ARL4C	1.73168	0.91601	20.8741	8.20E-10	7.34E-08	12.8388
MME-AS1	1.73532	0.86766	58.3512	2.11E-14	3.64E-11	23.7389
TMEM200	1.73846	0.86923	43.0917	4.84E-13	3.49E-10	20.6479
PDGFRA	1.73908	2.51641	34.8941	4.26E-12	1.60E-09	18.412
ZNF737	1.74472	1.24554	24.5156	1.59E-10	2.14E-08	14.5917
OAS2	1.7476	1.56981	33.3326	6.82E-12	2.13E-09	17.9208
IKZF2	1.75488	0.92761	26.4917	7.21E-11	1.14E-08	15.4355
SLC2A10	1.76042	0.88021	32.4075	9.11E-12	2.53E-09	17.618
ANO3-AS	1.7632	0.8816	40.0825	1.02E-12	6.05E-10	19.8868
D21S208	1.76734	0.93384	32.4266	9.06E-12	2.53E-09	17.6243
SIX3	1.77456	0.93745	24.6314	1.52E-10	2.05E-08	14.643
RNLS	1.78385	0.99227	25.9001	9.09E-11	1.36E-08	15.1898
KLHDC8A	1.78751	0.94393	22.6533	3.57E-10	3.90E-08	13.7306
CDK6	1.78765	1.12386	19.0419	2.08E-09	1.56E-07	11.8381
BRINP3	1.7891	1.53744	18.2206	3.24E-09	2.27E-07	11.3585
ZNF432	1.79335	0.99702	24.205	1.81E-10	2.33E-08	14.4527
FGF18	1.79503	1.42686	20.9997	7.71E-10	6.98E-08	12.9042
CD40	1.79942	0.94988	24.1406	1.86E-10	2.37E-08	14.4237
FRMD7	1.80128	0.90064	63.1337	9.32E-15	2.20E-11	24.5089
CLDN17	1.80211	0.95123	28.343	3.61E-11	6.93E-09	16.1692

ZNF841	1.80536	0.9759	25.7753	9.55E-11	1.41E-08	15.1373
DCDC1	1.80556	1.34965	16.7664	7.49E-09	4.47E-07	10.4558
TBX18	1.80687	0.90344	40.2767	9.71E-13	5.96E-10	19.9378
THBS1	1.80863	1.81717	30.472	1.72E-11	3.77E-09	16.9535
KCNS1	1.80987	0.90493	80.5654	7.46E-16	3.29E-12	26.7725
CNTNAP2	1.8108	0.96247	31.9374	1.06E-11	2.82E-09	17.4605
FILIP1L	1.8176	2.08518	38.6813	1.47E-12	7.80E-10	19.5102
OTOAP1	1.82026	1.23937	21.9607	4.89E-10	4.93E-08	13.392
SHROOM	1.82377	0.98105	26.4075	7.45E-11	1.17E-08	15.4009
NBEA	1.8257	2.08157	25.141	1.23E-10	1.72E-08	14.866
ITGA8	1.82571	1.0132	23.5121	2.44E-10	2.92E-08	14.1362
PCYT1B	1.82864	1.37439	24.4926	1.61E-10	2.15E-08	14.5814
ANKRD30	1.82873	1.04405	28.0264	4.05E-11	7.48E-09	16.0473
GPR85	1.83107	0.91553	43.8852	4.01E-13	3.00E-10	20.8386
CER1	1.83825	0.9693	33.2628	6.97E-12	2.16E-09	17.8983
TLX3	1.83927	1.01998	15.5888	1.55E-08	8.14E-07	9.66835
CNN1	1.84117	1.49601	16.751	7.56E-09	4.50E-07	10.4459
RASSF9	1.84178	1.2011	17.9495	3.77E-09	2.55E-07	11.1956
LRRC55	1.8451	0.97272	23.6307	2.32E-10	2.82E-08	14.1911
CASP4	1.85107	1.34659	15.7375	1.41E-08	7.56E-07	9.77083
MBNL3	1.85259	1.10616	20.1799	1.16E-09	9.62E-08	12.4702
LACC1	1.85463	0.97749	30.6505	1.62E-11	3.61E-09	17.0167
C1orf226	1.855	1.07802	14.925	2.40E-08	1.16E-06	9.19941
ARMCX4	1.85534	0.92767	17.6257	4.53E-09	2.96E-07	10.9979
IL21R	1.85716	1.23814	11.021	4.68E-07	1.45E-05	5.98186
APOL3	1.8586	1.19631	13.1774	8.22E-08	3.28E-06	7.86589
LINC0238	1.86041	1.00972	22.163	4.46E-10	4.60E-08	13.492
CRABP2	1.86207	2.18279	16.6464	8.05E-09	4.73E-07	10.378
EDIL3	1.86806	1.16406	14.7637	2.67E-08	1.27E-06	9.08253
CCR5AS	1.86909	0.93454	31.6255	1.17E-11	2.99E-09	17.3547
COL4A6	1.87204	0.98619	22.6028	3.65E-10	3.96E-08	13.7063
FOXG1	1.87458	0.93729	30.26	1.84E-11	3.98E-09	16.878
ZNF85	1.87562	1.22934	23.2834	2.70E-10	3.11E-08	14.0297
BISPR	1.87907	1.16957	18.626	2.60E-09	1.89E-07	11.5978
PRDM8	1.88377	1.47531	18.183	3.31E-09	2.31E-07	11.3361
FREM1	1.88881	1.09492	22.3114	4.16E-10	4.38E-08	13.5648
PRR16	1.89218	1.26327	19.8279	1.38E-09	1.11E-07	12.2785
DSC2	1.89513	1.09808	23.4529	2.50E-10	2.96E-08	14.1088
LINC0165	1.89524	0.94762	55.2062	3.74E-14	5.12E-11	23.188
LINC0021	1.89722	0.94861	38.4229	1.58E-12	7.94E-10	19.4391
MME	1.89983	2.20929	35.9361	3.15E-12	1.32E-09	18.7266
CRABP1	1.89986	0.94993	26.1318	8.29E-11	1.28E-08	15.2867
ISL1-DT	1.90036	1.00035	31.8184	1.10E-11	2.90E-09	17.4203
PIP5K1B	1.90825	1.05447	28.9269	2.93E-11	5.79E-09	16.3903
MIR7-3H(	1.90954	1.00494	34.0922	5.41E-12	1.89E-09	18.1628
DPYD	1.91303	1.42511	41.0866	7.91E-13	4.99E-10	20.1477
ZEB2	1.91677	1.00856	24.0968	1.90E-10	2.41E-08	14.404
MEF2C-A	1.92206	0.96103	39.3172	1.25E-12	6.90E-10	19.6829
LRFN5	1.92305	1.17074	18.9475	2.19E-09	1.63E-07	11.784
LINC0111	1.92833	0.96417	34.748	4.45E-12	1.65E-09	18.367
BICC1	1.93378	1.44664	14.3793	3.47E-08	1.58E-06	8.7991
ARHGDIE	1.93624	1.01829	30.2831	1.83E-11	3.97E-09	16.8863
FGL1	1.937	0.9685	52.5702	6.20E-14	6.68E-11	22.6958
GPC6	1.94219	1.67459	16.6727	7.93E-09	4.67E-07	10.3951
PPP2R2E	1.94699	1.10318	21.7835	5.31E-10	5.23E-08	13.3037
GPC5	1.95887	1.1593	20.8792	8.17E-10	7.34E-08	12.8415
SOCS3	1.96362	1.6853	14.4452	3.32E-08	1.53E-06	8.84819
EXO5	1.96374	1.08221	19.6652	1.50E-09	1.18E-07	12.1887

LINC0062	1.96471	1.01171	34.6098	4.63E-12	1.69E-09	18.3243
IGFN1	1.96821	1.43394	13.9625	4.65E-08	2.02E-06	8.48382
CYB5R2	1.97406	1.24641	20.6207	9.28E-10	8.10E-08	12.7057
GRIK1	1.97578	1.73112	20.4326	1.02E-09	8.74E-08	12.6058
PITX2	1.97746	1.0389	25.4012	1.11E-10	1.58E-08	14.9781
USP53	1.97903	1.42876	23.9533	2.02E-10	2.51E-08	14.3389
MESTIT1	1.98249	0.99124	62.4202	1.05E-14	2.31E-11	24.3988
PPP1R1C	1.98266	1.0547	38.4383	1.57E-12	7.94E-10	19.4433
L1TD1	1.98269	1.16937	36.1847	2.93E-12	1.28E-09	18.8003
ISL1	1.98299	1.64758	18.1776	3.32E-09	2.31E-07	11.3328
SAMD9L	1.98474	1.04254	34.0495	5.48E-12	1.89E-09	18.1493
KANK4	1.98543	1.46131	17.8426	4.01E-09	2.67E-07	11.1307
RGS13	1.98825	1.0443	28.9707	2.88E-11	5.73E-09	16.4067
NRK	1.99416	2.29643	45.0096	3.08E-13	2.40E-10	21.1021
IFI27	1.99782	1.60745	16.9146	6.86E-09	4.14E-07	10.5512
PSMB9	2.00192	1.88321	19.809	1.39E-09	1.11E-07	12.2681
LINC0219	2.00232	1.00116	95.8967	1.23E-16	2.29E-12	28.2529
A1CF	2.00617	1.32027	18.1958	3.29E-09	2.30E-07	11.3438
GBP1	2.01008	2.25354	35.8325	3.24E-12	1.34E-09	18.6958
PTPRO	2.01012	1.05523	27.7587	4.47E-11	7.96E-09	15.9431
LINC0114	2.01161	1.10432	26.9789	5.98E-11	9.62E-09	15.6336
ROBO2	2.01248	1.55699	22.9496	3.12E-10	3.51E-08	13.8723
ZIC4	2.01291	1.05663	28.7304	3.14E-11	6.17E-09	16.3164
SLC7A3	2.02055	1.16079	25.317	1.15E-10	1.63E-08	14.942
PXMP4	2.0242	1.06546	26.9797	5.98E-11	9.62E-09	15.6339
CD248	2.02599	1.2932	13.4655	6.64E-08	2.77E-06	8.0964
KCNMA1	2.02931	1.14435	20.2203	1.13E-09	9.47E-08	12.492
FLRT3	2.03569	1.76814	33.5784	6.33E-12	2.04E-09	17.9997
PDE4DIP	2.03787	1.10425	27.9019	4.24E-11	7.72E-09	15.999
ANKRD20	2.03808	1.19423	20.2863	1.10E-09	9.26E-08	12.5275
CTNNA2	2.03922	1.29982	17.4165	5.11E-09	3.27E-07	10.8683
MUC15	2.03959	1.32935	19.204	1.91E-09	1.46E-07	11.9304
FRY	2.04546	1.0729	33.1016	7.33E-12	2.19E-09	17.846
EBF2	2.04661	1.0233	48.7546	1.35E-13	1.25E-10	21.9279
MMRN1	2.05064	1.46457	39.301	1.25E-12	6.90E-10	19.6786
DTX3	2.05334	1.12701	20.5004	9.84E-10	8.50E-08	12.6419
FMN1	2.06252	1.03126	37.6223	1.96E-12	9.50E-10	19.2154
ZNF521	2.06778	1.39362	32.774	8.12E-12	2.32E-09	17.739
RUNX2	2.06997	1.39471	21.4815	6.12E-10	5.85E-08	13.1515
TRPM3	2.07462	1.03731	59.431	1.74E-14	3.30E-11	23.9196
OR5AK4P	2.086	1.24369	29.3338	2.54E-11	5.22E-09	16.5416
PSD3	2.08749	1.71598	27.5317	4.86E-11	8.36E-09	15.8539
GPM6A	2.09087	2.2568	41.1838	7.72E-13	4.95E-10	20.1726
NTS	2.09938	2.98774	42.6226	5.41E-13	3.65E-10	20.5333
CACNA2D	2.1105	1.31463	19.6084	1.55E-09	1.21E-07	12.1572
NEFH	2.11217	2.2831	22.7343	3.44E-10	3.80E-08	13.7695
SCN7A	2.1126	1.40283	21.551	5.93E-10	5.69E-08	13.1867
TDRD1	2.12832	1.06416	57.0412	2.66E-14	4.07E-11	23.5141
HAPLN1	2.1296	1.81099	50.4599	9.47E-14	9.43E-11	22.2796
INPP4B	2.1351	1.48597	19.1889	1.92E-09	1.46E-07	11.9218
MIR137HG	2.13705	1.21904	20.804	8.48E-10	7.50E-08	12.8022
ZNF738	2.1435	1.20144	21.8714	5.10E-10	5.08E-08	13.3476
SCN1A-A	2.14446	1.48657	21.5361	5.97E-10	5.71E-08	13.1792
DSC1	2.14683	1.08031	61.667	1.19E-14	2.49E-11	24.2809
CNTN4	2.15401	1.17735	29.4717	2.42E-11	5.00E-09	16.5924
SOX9	2.15641	1.0782	27.2645	5.37E-11	9.12E-09	15.748
IDO1	2.15641	1.07821	70.3952	3.02E-15	1.09E-11	25.544
CLDN8	2.15806	1.07903	65.7668	6.11E-15	1.82E-11	24.9016

RSPO2	2.15933	1.33052	21.8809	5.08E-10	5.08E-08	13.3524
GBP5	2.16906	1.16405	26.1748	8.16E-11	1.27E-08	15.3046
PEG10	2.16978	1.26475	21.4131	6.33E-10	6.01E-08	13.1167
VCAN	2.1708	1.80577	51.2843	8.01E-14	8.37E-11	22.4446
FOLH1B	2.17263	1.16584	38.8253	1.42E-12	7.61E-10	19.5496
BAALC	2.17765	1.33501	17.4087	5.14E-09	3.28E-07	10.8635
PWWP3E	2.18802	1.40356	19.7457	1.44E-09	1.14E-07	12.2333
POSTN	2.18889	2.99337	63.0838	9.40E-15	2.20E-11	24.5013
FGF20	2.19496	1.14765	33.8663	5.79E-12	1.93E-09	18.0914
RORB	2.20097	1.28035	29.2721	2.59E-11	5.31E-09	16.5188
TRHDE	2.20538	1.20303	31.7355	1.13E-11	2.95E-09	17.3922
NDNF	2.21019	1.15527	33.8446	5.83E-12	1.93E-09	18.0845
STRA6	2.21067	1.25585	19.5913	1.56E-09	1.21E-07	12.1478
CEMIP2	2.22212	2.20273	38.3081	1.63E-12	8.09E-10	19.4073
PDE1A	2.2276	1.39401	22.2002	4.38E-10	4.53E-08	13.5103
ASS1	2.26444	1.13222	40.2593	9.75E-13	5.96E-10	19.9332
UNC5C	2.26837	1.18436	31.3392	1.29E-11	3.09E-09	17.2566
SHOX2	2.26995	2.4106	34.4146	4.91E-12	1.76E-09	18.2637
SLC30A8	2.28019	1.34078	22.119	4.55E-10	4.67E-08	13.4704
PDGFD	2.28122	1.47099	15.0448	2.21E-08	1.08E-06	9.28546
LINC0233	2.29998	1.21915	39.0971	1.32E-12	7.18E-10	19.6235
CASP1P2	2.30911	1.2549	32.6479	8.45E-12	2.38E-09	17.6975
MAML3	2.30977	1.21063	23.5666	2.38E-10	2.87E-08	14.1615
VAV3	2.31137	1.20586	35.2419	3.85E-12	1.51E-09	18.5181
POU3F2	2.3147	1.25769	27.9593	4.15E-11	7.60E-09	16.0213
L3MBTL4	2.31866	1.2095	31.5749	1.19E-11	2.99E-09	17.3375
RALGPS2	2.32267	1.78044	24.0319	1.95E-10	2.45E-08	14.3745
B3GALT1	2.33287	1.30656	25.9045	9.07E-11	1.36E-08	15.1917
PCDH15	2.3528	1.35626	27.3299	5.24E-11	8.94E-09	15.7741
NTNG1	2.36775	1.18388	52.8606	5.86E-14	6.65E-11	22.7514
NRXN1	2.37117	1.62279	16.0867	1.13E-08	6.24E-07	10.0079
CPA2	2.37391	1.2873	34.9001	4.25E-12	1.60E-09	18.4138
NOX4	2.37493	1.18747	84.146	4.76E-16	2.36E-12	27.154
SEMA6D	2.37564	1.28816	33.1164	7.30E-12	2.19E-09	17.8508
TLE4	2.37822	1.59434	20.6051	9.35E-10	8.13E-08	12.6975
CCDC178	2.37936	1.52006	28.2317	3.76E-11	7.14E-09	16.1265
XIRP2	2.38055	1.19027	89.0592	2.64E-16	2.29E-12	27.6401
TMEM30E	2.39255	1.34679	31.5317	1.21E-11	3.01E-09	17.3227
SLC5A12	2.39892	1.19946	65.2483	6.63E-15	1.82E-11	24.8259
ZIC1	2.40455	1.61217	23.4374	2.52E-10	2.97E-08	14.1016
CNNM1	2.40846	1.2544	32.1197	9.99E-12	2.72E-09	17.5219
ZNF826P	2.41521	1.26751	53.8295	4.85E-14	6.02E-11	22.9346
SDC2	2.42101	2.17411	34.0289	5.52E-12	1.89E-09	18.1428
SNX7	2.42724	1.31396	32.8236	7.99E-12	2.31E-09	17.7553
IL1RAPL2	2.44113	1.5216	31.2705	1.32E-11	3.13E-09	17.2329
KRT8P48	2.44488	1.27261	34.2206	5.21E-12	1.83E-09	18.2031
TIMP3	2.44583	2.08434	32.074	1.01E-11	2.74E-09	17.5066
CPED1	2.45018	1.88925	41.6632	6.85E-13	4.48E-10	20.2943
ME1	2.45175	1.22587	52.5489	6.23E-14	6.68E-11	22.6916
PTPRD	2.46249	1.51145	23.5005	2.45E-10	2.93E-08	14.1308
ALX1	2.46679	1.33374	32.1477	9.90E-12	2.72E-09	17.5313
GBP4	2.4688	1.28457	38.6356	1.49E-12	7.80E-10	19.4976
FLNC	2.47813	1.48879	17.9319	3.81E-09	2.57E-07	11.185
GRIA4	2.48065	1.67105	24.2143	1.81E-10	2.33E-08	14.457
ANO3	2.48197	1.86772	27.1707	5.56E-11	9.17E-09	15.7106
LAMA1	2.49218	1.60935	25.1213	1.24E-10	1.72E-08	14.8575
PI15	2.50284	3.30793	46.9179	2.01E-13	1.73E-10	21.5325
MFAP2	2.50417	1.30226	24.3592	1.70E-10	2.22E-08	14.522

MSX1	2.51642	1.76378	20.1865	1.15E-09	9.61E-08	12.4738
MARCHF	2.52448	1.31241	27.9641	4.14E-11	7.60E-09	16.0231
TWIST1	2.52937	1.57424	24.6405	1.51E-10	2.05E-08	14.647
PBX1	2.53539	1.2677	31.5256	1.21E-11	3.01E-09	17.3206
PRRX1	2.5363	1.31832	39.6644	1.14E-12	6.55E-10	19.776
CASP1	2.5407	1.43702	31.6984	1.14E-11	2.97E-09	17.3796
LAYN	2.57207	1.28604	85.497	4.03E-16	2.29E-12	27.2918
LHX8	2.57382	1.76313	28.2474	3.74E-11	7.14E-09	16.1325
GPRIN3	2.58333	2.26979	33.4122	6.66E-12	2.12E-09	17.9464
FIBIN	2.59261	1.92453	26.725	6.59E-11	1.05E-08	15.5308
ZFHX4	2.59627	1.44865	31.586	1.19E-11	2.99E-09	17.3412
MTUS1	2.61179	2.04862	47.5544	1.75E-13	1.58E-10	21.6716
CEP41	2.61274	1.35654	37.4747	2.04E-12	9.78E-10	19.1736
TRPC4	2.61585	1.68848	27.221	5.46E-11	9.14E-09	15.7307
KLHL14	2.62739	1.36386	42.6362	5.40E-13	3.65E-10	20.5366
EPHA3	2.64302	1.98522	69.1444	3.64E-15	1.20E-11	25.3761
ITGA1	2.66499	1.68256	20.0884	1.21E-09	9.93E-08	12.4207
ESRRG	2.66813	1.33406	86.2653	3.68E-16	2.29E-12	27.3687
MEF2C	2.6767	1.94445	36.8503	2.43E-12	1.12E-09	18.9947
FIGN	2.70219	1.65212	33.6933	6.11E-12	1.99E-09	18.0364
ALPK2	2.71258	1.35629	88.5537	2.80E-16	2.29E-12	27.592
UCP2	2.71796	1.35898	35.5324	3.53E-12	1.43E-09	18.6059
CCR1	2.72541	1.83893	35.81	3.26E-12	1.34E-09	18.6891
SCN9A	2.72625	2.36415	58.0318	2.23E-14	3.69E-11	23.6847
EPHA7	2.75326	1.8822	33.4057	6.67E-12	2.12E-09	17.9443
COL1A2	2.75575	1.42805	28.143	3.88E-11	7.31E-09	16.0924
OTOA	2.76119	1.65114	30.7672	1.55E-11	3.53E-09	17.0577
CARD16	2.79307	1.80939	18.499	2.78E-09	2.00E-07	11.5234
PAX2	2.82281	1.41141	36.0015	3.09E-12	1.30E-09	18.7461
EYA4	2.84035	1.62938	28.0821	3.97E-11	7.40E-09	16.0688
MAB21L2	2.84061	1.55	26.0942	8.42E-11	1.28E-08	15.271
DLX6	2.85458	1.47746	39.8514	1.08E-12	6.33E-10	19.8257
SNCA	2.85548	1.42774	70.6459	2.91E-15	1.09E-11	25.5772
FREM2	2.85992	1.74714	23.3435	2.63E-10	3.05E-08	14.0578
DLX5	2.88101	1.49068	30.564	1.66E-11	3.67E-09	16.9861
ASXL3	2.89764	1.66566	25.14	1.23E-10	1.72E-08	14.8656
HDAC9	2.90642	1.63545	31.8406	1.09E-11	2.89E-09	17.4278
RGS1	2.91223	1.87717	24.204	1.82E-10	2.33E-08	14.4523
ZPLD1	3.02114	1.76143	31.9455	1.06E-11	2.82E-09	17.4633
LIN28A	3.06842	2.00544	29.614	2.30E-11	4.79E-09	16.6446
COL6A3	3.13261	2.1402	29.0414	2.81E-11	5.64E-09	16.4331
BST2	3.14792	1.7908	20.8161	8.43E-10	7.48E-08	12.8085
TSIX	3.15647	2.18495	39.3488	1.24E-12	6.90E-10	19.6914
MAB21L1	3.21882	1.78927	32.6813	8.36E-12	2.37E-09	17.7086
ARHGAP	3.3571	1.97491	29.8408	2.13E-11	4.52E-09	16.7272
MEST	3.35992	3.12374	20.008	1.26E-09	1.02E-07	12.377
XIST	3.46291	2.2822	27.211	5.48E-11	9.14E-09	15.7267
DLX6-AS	3.46959	2.18167	25.6946	9.86E-11	1.45E-08	15.1032

Supplementary Table 3: DEGs in Ovsc4/OVsc4DDP

gene	logFC	AveExpr	t	P.Value	adj.P.Val	B
HOXD1	-10.173	-1.3863	-41.581	1.31E-08	1.19E-05	10.7679
TSPAN7	-9.4485	-2.3856	-34.431	4.03E-08	1.89E-05	9.78066
CALCR	-9.3055	-2.0875	-19.083	1.35E-06	0.0001	6.25245
HAGLR	-9.2626	-2.0709	-32.978	5.21E-08	2.01E-05	9.54311
ZNF385D	-9.0295	-1.4137	-98.318	7.55E-11	1.17E-06	13.8582
EN2	-8.8515	-1.9057	-43.016	1.07E-08	1.03E-05	10.9355
ANO3	-8.769	-2.1016	-36.255	2.96E-08	1.48E-05	10.0597
HAGLRO	-8.7275	-0.0828	-15.701	4.25E-06	0.00019	5.00276
PRKCB	-8.6017	-0.387	-30.842	7.77E-08	2.36E-05	9.16591
NOSTRIN	-8.3844	-1.502	-9.4934	7.81E-05	0.00113	1.74492
VNN1	-8.3745	-1.5396	-11.627	2.45E-05	0.00056	3.05438
HAVCR1	-8.2385	-2.6159	-20.596	8.57E-07	8.30E-05	6.73395
TDGF1	-8.1169	-2.2972	-15.345	4.86E-06	0.00021	4.85468
TSHR	-7.8788	-3.1941	-59.14	1.59E-09	3.51E-06	12.3338
SST	-7.8749	0.70746	-4.8073	0.00298	0.01286	-2.3811
NR1H4	-7.8689	-3.7827	-20.77	8.16E-07	8.01E-05	6.78687
TMEM252	-7.8328	-2.7456	-31.266	7.16E-08	2.27E-05	9.24365
HOXD8	-7.8258	-2.6653	-28.161	1.34E-07	3.07E-05	8.64006
TDGF1P3	-7.7825	-1.8779	-16.391	3.30E-06	0.00017	5.28006
PLCL1	-7.7619	-1.1879	-16.854	2.80E-06	0.00015	5.45928
HP	-7.6633	-1.2506	-10.914	3.52E-05	0.0007	2.64423
TNIK	-7.6267	-2.273	-20.953	7.74E-07	7.77E-05	6.84183
FEZ1	-7.6058	-0.7938	-13.252	1.15E-05	0.00035	3.90401
ANGPTL1	-7.512	-2.5922	-21.855	6.03E-07	6.38E-05	7.10486
CLEC4E	-7.5051	-2.843	-9.4337	8.10E-05	0.00116	1.70448
PAK3	-7.2682	-2.8121	-28.587	1.22E-07	2.92E-05	8.72787
CA4	-7.2551	-3.6281	-13.093	1.23E-05	0.00037	3.82568
FAM13C	-7.0368	-4.1126	-28.638	1.21E-07	2.92E-05	8.73817
GJA5	-7.0319	0.4473	-12.66	1.50E-05	0.00041	3.60724
TDRD10	-7.0054	-3.4487	-17.605	2.17E-06	0.00013	5.73879
HOXD-AS	-6.9379	-4.0247	-28.108	1.35E-07	3.07E-05	8.62887
RGS5	-6.8892	-0.7319	-16.609	3.05E-06	0.00016	5.36526
LINC0111	-6.7658	-3.4708	-25.612	2.35E-07	4.10E-05	8.0761
SPATA18	-6.7463	-3.8611	-21.846	6.05E-07	6.38E-05	7.10234
NRG2	-6.7385	-2.9444	-11.705	2.35E-05	0.00055	3.09793
A2ML1	-6.6442	-3.144	-12.236	1.82E-05	0.00047	3.38616
KCND2	-6.5555	-3.2722	-38.841	1.96E-08	1.35E-05	10.4219
SHE	-6.5055	-0.4622	-7.9995	0.0002	0.00209	0.65564
ZIC2	-6.4859	-2.7312	-26.943	1.74E-07	3.37E-05	8.37906
AADAC	-6.4299	-3.6388	-29.706	9.72E-08	2.63E-05	8.95082
CHGA	-6.3775	-0.2723	-9.5541	7.53E-05	0.00111	1.78586
BMX	-6.3683	-3.7747	-16.731	2.93E-06	0.00016	5.41235
TMEM252	-6.3022	-4.5397	-71.821	4.95E-10	1.92E-06	13.02
MMP1	-6.2907	-0.4557	-9.2762	8.91E-05	0.00123	1.59654
PTPRD	-6.2852	3.22814	-30.655	8.06E-08	2.40E-05	9.13138
C12orf75-	-6.2644	-1.8584	-20.382	9.12E-07	8.47E-05	6.66843
HBE1	-6.2213	-3.6259	-40.742	1.47E-08	1.24E-05	10.6658
TM4SF18	-6.2069	0.27011	-12.601	1.54E-05	0.00042	3.57686
GATA4	-6.1892	-4.7458	-72.596	4.65E-10	1.92E-06	13.0541
TWIST1	-6.1816	-2.7629	-36.363	2.91E-08	1.48E-05	10.0755
THBS2	-6.1611	-3.8599	-14.079	8.05E-06	0.00028	4.29686
CECR2	-6.1243	-1.2746	-14.907	5.76E-06	0.00023	4.66736
FOXR2	-6.107	-2.8135	-20.458	8.92E-07	8.44E-05	6.69175
HLF	-6.0734	-1.4792	-14.408	7.04E-06	0.00026	4.44676
MIR3142H	-6.0578	-1.4491	-8.6382	0.00013	0.00159	1.14169
SPAG17	-5.96	-1.556	-9.1967	9.35E-05	0.00127	1.54143

FLG	-5.9456	-3.983	-8.2914	0.00017	0.00184	0.88183
ZIC5	-5.9187	-3.8138	-27.029	1.71E-07	3.37E-05	8.39799
TNFAIP6	-5.9041	-2.2786	-11.613	2.46E-05	0.00056	3.04682
PTPRD-A	-5.892	-3.716	-61.319	1.28E-09	3.30E-06	12.4715
CHST1	-5.8675	-2.3072	-23.158	4.28E-07	5.35E-05	7.46298
SYTL5	-5.8613	-4.3661	-14.241	7.53E-06	0.00027	4.37117
GRAMD1	-5.8066	-0.6264	-7.0055	0.00042	0.00332	-0.1709
DPP4	-5.7923	0.15617	-9.403	8.25E-05	0.00117	1.68352
CLDN2	-5.7852	-3.7854	-6.5617	0.0006	0.00419	-0.5712
VNN2	-5.7515	-3.4184	-9.5383	7.61E-05	0.00111	1.77523
SPARC	-5.7332	4.17337	-15.838	4.04E-06	0.00019	5.05881
LINC0253	-5.727	-3.031	-17.21	2.48E-06	0.00014	5.59345
ZC4H2	-5.7066	-2.9583	-26.538	1.90E-07	3.51E-05	8.28883
POU6F2-	-5.6913	-2.3851	-11.549	2.54E-05	0.00057	3.01114
CALHM6	-5.6828	-2.9266	-11.395	2.75E-05	0.00059	2.92403
AADACP1	-5.6114	-3.1969	-7.6644	0.00026	0.00243	0.38707
CALHM5	-5.5956	-2.0542	-9.1527	9.61E-05	0.00129	1.51072
DGKG	-5.5527	-4.1079	-36.865	2.68E-08	1.48E-05	10.1484
RBM20	-5.5087	-2.3177	-18.234	1.76E-06	0.00012	5.96293
LINC0230	-5.5058	-0.7776	-6.5294	0.00062	0.00427	-0.6012
NAV3	-5.4883	-2.4282	-9.0168	0.0001	0.00137	1.41511
LINC0093	-5.4782	-3.7923	-12.789	1.41E-05	0.0004	3.6733
LINC0216	-5.4779	1.22737	-16.338	3.37E-06	0.00017	5.25907
PHACTR1	-5.4686	1.39632	-8.1172	0.00019	0.00198	0.7477
LINC0059	-5.4513	-2.6259	-9.9716	5.91E-05	0.00096	2.06097
SEPTIN4-	-5.4456	-1.9236	-18.428	1.66E-06	0.00011	6.03056
SLC8A3	-5.3972	-3.2131	-23.75	3.68E-07	4.97E-05	7.61779
RASGRF2	-5.3929	-2.1088	-9.9365	6.03E-05	0.00097	2.03826
C6orf141	-5.3763	-2.2971	-10.426	4.58E-05	0.00082	2.34845
TIE1	-5.3384	2.85083	-18.077	1.85E-06	0.00012	5.90779
NKX2-4	-5.3378	-3.8625	-17.875	1.98E-06	0.00012	5.83622
TBXA2R	-5.2996	-4.0087	-17.68	2.11E-06	0.00013	5.76591
LINC0185	-5.2869	-2.9626	-11.063	3.26E-05	0.00067	2.73228
CFP	-5.2576	-1.3424	-11.507	2.60E-05	0.00058	2.98707
AR	-5.2471	-3.3898	-17.931	1.95E-06	0.00012	5.85596
ACSM1	-5.2241	-4.1142	-12.841	1.38E-05	0.00039	3.69946
SHISA3	-5.2201	-1.0623	-18.365	1.69E-06	0.00011	6.00855
NKX2-1	-5.2186	-0.341	-11.784	2.26E-05	0.00054	3.14173
ELMO1	-5.2138	-1.2258	-16.942	2.72E-06	0.00015	5.49256
GJA4	-5.1603	1.89036	-6.3977	0.00069	0.00458	-0.7245
ST8SIA4	-5.1531	-1.8373	-7.4038	0.00031	0.00271	0.17132
ADAM33	-5.1467	-3.1394	-9.3833	8.35E-05	0.00118	1.67008
STAT4	-5.1407	-2.4444	-18.605	1.56E-06	0.00011	6.09136
C2CD4C	-5.0707	-4.0375	-15.443	4.69E-06	0.00021	4.89587
SRGN	-5.061	0.45058	-11.136	3.14E-05	0.00065	2.7749
CLEC18B	-5.0301	-3.1285	-62.944	1.09E-09	3.30E-06	12.5683
SHC2	-5.0073	-4.6676	-10.539	4.31E-05	0.00079	2.41782
LRRTM2	-4.9922	-3.1284	-8.5793	0.00014	0.00164	1.09826
SIRPB2	-4.9654	-3.3455	-7.7982	0.00024	0.00228	0.49547
FAM83A	-4.9619	-4.1714	-9.1654	9.53E-05	0.00128	1.51961
FXYD2	-4.9574	-3.9789	-8.1249	0.00019	0.00198	0.75366
MRAP2	-4.9302	-3.621	-19.57	1.16E-06	9.37E-05	6.41208
SLC25A1	-4.9297	-1.7288	-15.244	5.06E-06	0.00021	4.81218
JAKMIP3	-4.9252	-2.3068	-24.315	3.20E-07	4.77E-05	7.7614
PKHD1	-4.8639	-3.2029	-20.673	8.39E-07	8.18E-05	6.75732
MCOLN2	-4.8418	-3.5129	-6.5623	0.0006	0.00419	-0.5706
BTBD16	-4.8204	-4.0588	-8.7068	0.00013	0.00156	1.19206
ST8SIA5	-4.8149	-3.6875	-18.342	1.70E-06	0.00012	6.00049

ADAMTS1	-4.8054	-3.1842	-8.2475	0.00017	0.00188	0.84825
UNC80	-4.7961	-3.5451	-18.959	1.40E-06	0.0001	6.21094
TUBAL3	-4.796	-3.9413	-15.878	3.98E-06	0.00018	5.07502
AOAH	-4.78	-1.1313	-10.167	5.29E-05	0.00089	2.18629
STK32B	-4.7729	1.54743	-17.481	2.26E-06	0.00013	5.69337
LINC0233	-4.7626	-2.6502	-18.886	1.43E-06	0.0001	6.18667
INSYN2B	-4.7527	-3.3077	-13.42	1.07E-05	0.00034	3.98576
CHP2	-4.7464	-4.2223	-11.137	3.14E-05	0.00065	2.77528
DIRAS2	-4.7279	-3.9336	-9.0321	0.0001	0.00136	1.42593
SIX3-AS1	-4.7237	-1.9972	-7.5527	0.00028	0.00255	0.29534
CRYM	-4.7169	-3.7696	-11.213	3.02E-05	0.00064	2.81919
EFNA3	-4.7066	0.76417	-11.763	2.29E-05	0.00054	3.12984
AMOT	-4.6978	-0.3656	-10.585	4.20E-05	0.00078	2.44598
SELENOI	-4.6861	-1.9022	-8.5456	0.00014	0.00166	1.07324
TAL1	-4.6811	-3.6233	-8.4032	0.00016	0.00177	0.96663
SPARCL1	-4.6774	-4.1942	-9.1259	9.77E-05	0.00131	1.49196
ROBO3	-4.6697	-1.4843	-13.952	8.49E-06	0.00029	4.23846
TMEM10(	-4.658	0.3588	-4.3606	0.00477	0.01797	-2.9083
GLI3	-4.6556	1.21576	-22.641	4.89E-07	5.80E-05	7.3238
FFAR4	-4.6262	-4.0108	-26.97	1.73E-07	3.37E-05	8.38483
CCDC181	-4.6232	-2.1595	-10.854	3.64E-05	0.00071	2.60857
MAGEC1	-4.6037	-1.1624	-7.5021	0.00029	0.00261	0.25338
HOXA11	-4.5929	1.71326	-20.302	9.34E-07	8.61E-05	6.64367
CA3	-4.5835	-2.259	-8.0981	0.00019	0.00201	0.73284
MAOB	-4.5692	0.59191	-8.082	0.00019	0.00202	0.72024
GLIS1	-4.5637	-3.7531	-10.729	3.89E-05	0.00074	2.53341
ADGRE1	-4.5388	-4.3215	-9.0463	0.0001	0.00135	1.43597
ZNF804A	-4.5228	-3.3038	-10.181	5.25E-05	0.00089	2.19466
ZPLD1	-4.4829	-2.5913	-7.8382	0.00023	0.00223	0.5276
PPP4R4	-4.4705	-4.1642	-23.341	4.08E-07	5.30E-05	7.51131
MMP10	-4.4531	0.53539	-4.5898	0.00374	0.01511	-2.6342
PPP1R16	-4.4521	0.02614	-15.065	5.42E-06	0.00022	4.73567
PHYHIPL	-4.4433	-0.5697	-10.203	5.18E-05	0.00088	2.20862
SLC23A1	-4.4244	-2.0068	-8.3768	0.00016	0.00178	0.94671
LINC0150	-4.4239	-2.0965	-8.048	0.0002	0.00205	0.69368
LINC0196	-4.4175	-2.8037	-8.6588	0.00013	0.00159	1.15685
AOX1	-4.416	-1.0718	-7.548	0.00028	0.00255	0.29146
GALNT16	-4.3869	-3.934	-13.267	1.14E-05	0.00035	3.91156
LRRK2	-4.3779	-4.0313	-12.958	1.31E-05	0.00038	3.75837
SCN3A	-4.373	-3.5514	-19.415	1.22E-06	9.53E-05	6.36178
LINC0106	-4.3676	-2.8092	-35.719	3.24E-08	1.57E-05	9.97983
ADRA2A	-4.3639	1.50122	-9.192	9.38E-05	0.00127	1.53817
CDO1	-4.3577	-2.6612	-12.538	1.58E-05	0.00043	3.54437
COL4A6	-4.3422	-3.3692	-10.499	4.40E-05	0.00081	2.39372
LINC0136	-4.3383	-2.2432	-17.107	2.57E-06	0.00015	5.55488
RMST	-4.3289	-5.0243	-25.119	2.64E-07	4.31E-05	7.95875
KCNJ16	-4.301	-0.203	-13.865	8.81E-06	0.00029	4.19748
FAM133A	-4.2652	-2.0645	-6.5593	0.0006	0.00419	-0.5734
TDO2	-4.2414	-3.5012	-19.467	1.20E-06	9.43E-05	6.37877
CASC19	-4.2373	-2.797	-9.1482	9.63E-05	0.00129	1.50762
P2RX6	-4.2165	-3.1937	-23.3	4.12E-07	5.30E-05	7.50069
RBFOX3	-4.2164	-2.666	-6.094	0.00089	0.00551	-1.0163
PITX2	-4.1997	-2.3193	-11.397	2.75E-05	0.00059	2.92477
APBB1	-4.1972	0.02742	-16.617	3.05E-06	0.00016	5.36828
CECR7	-4.1678	-0.1493	-17.32	2.39E-06	0.00014	5.63425
ST8SIA2	-4.1587	-0.8468	-10.262	5.02E-05	0.00087	2.24589
SORBS2	-4.1497	2.9296	-10.953	3.45E-05	0.00069	2.66745
LINC0135	-4.1492	-4.1803	-13.88	8.75E-06	0.00029	4.20458

GEM	-4.1484	-1.0773	-7.8672	0.00022	0.00221	0.5508
GAB3	-4.1361	-2.0761	-21.907	5.95E-07	6.37E-05	7.11965
MAGEA3	-4.1244	2.61389	-23.992	3.47E-07	4.82E-05	7.67974
SLC22A1	-4.1236	-3.6019	-14.11	7.95E-06	0.00028	4.31111
MMRN2	-4.1047	-0.3653	-7.4356	0.00031	0.00269	0.19798
PKDCC	-4.095	-2.0116	-13.16	1.19E-05	0.00036	3.85899
F2	-4.0921	-2.9683	-7.5566	0.00028	0.00254	0.29859
RAD21L1	-4.073	-3.8613	-9.8386	6.38E-05	0.001	1.97449
LINC0144	-4.0691	-1.3314	-11.896	2.14E-05	0.00052	3.20311
BCAN-AS	-4.0361	-3.5595	-15.726	4.21E-06	0.00019	5.01308
PTX3	-4.0299	5.23821	-21.767	6.18E-07	6.47E-05	7.07963
CUBN	-4.023	-2.9736	-17.161	2.52E-06	0.00014	5.57491
PKIA	-4.0198	-4.2402	-13.153	1.20E-05	0.00036	3.85517
LNCRNA-	-4.0072	-3.6437	-12.998	1.28E-05	0.00037	3.77846
RTN4RL2	-4.0052	-3.0428	-10.398	4.65E-05	0.00083	2.33119
WSCD1	-4.003	0.64801	-31.254	7.18E-08	2.27E-05	9.24152
RASSF2	-3.9988	-0.2799	-4.8831	0.00276	0.01216	-2.2945
KCNMB4	-3.9888	-0.6326	-12.926	1.33E-05	0.00038	3.74211
SLC13A5	-3.9845	-2.5479	-15.929	3.91E-06	0.00018	5.09604
IGSF1	-3.9822	-3.2314	-16.845	2.81E-06	0.00015	5.45576
LINC0144	-3.976	-3.8306	-9.6306	7.20E-05	0.00108	1.83706
NINJ2	-3.9314	-3.7774	-17.749	2.07E-06	0.00013	5.79086
DIO2	-3.9203	-4.2517	-9.4048	8.24E-05	0.00117	1.6848
NAP1L2	-3.9072	-0.2414	-3.9955	0.00716	0.02436	-3.361
ABCA13	-3.8922	-2.6466	-15.146	5.25E-06	0.00022	4.77035
LINC0204	-3.8849	-3.0711	-13.218	1.16E-05	0.00035	3.88747
ADAM12	-3.8826	-2.3519	-4.2379	0.00546	0.01985	-3.0582
CXXC4	-3.8813	-2.8805	-38.5	2.07E-08	1.35E-05	10.3761
HHIP	-3.8739	-2.5917	-6.9932	0.00043	0.00334	-0.1818
CLDN14-/	-3.8417	-1.7304	-15.221	5.10E-06	0.00021	4.80216
CASC6	-3.8323	-4.349	-8.361	0.00016	0.00179	0.93474
PPM1E	-3.7978	-2.7578	-4.1008	0.00636	0.02234	-3.2284
HNF1A-A	-3.7932	-2.9166	-7.8155	0.00023	0.00226	0.50936
GALNT5	-3.7901	-3.2089	-8.6395	0.00013	0.00159	1.14267
IMPG2	-3.7851	-3.1693	-5.489	0.00153	0.00806	-1.631
ZNF233	-3.7572	-1.6766	-6.4545	0.00066	0.00444	-0.671
SHC3	-3.746	-3.1922	-7.4511	0.0003	0.00267	0.21096
CCDC198	-3.7288	0.42352	-6.1578	0.00084	0.00532	-0.9541
DLGAP1	-3.7282	1.31406	-11.478	2.64E-05	0.00058	2.97096
ATP8A1	-3.7218	3.03311	-11.976	2.06E-05	0.00051	3.24632
ANKRD18	-3.71	-3.9823	-7.9879	0.00021	0.0021	0.64648
FAM124A	-3.703	-4.4724	-10.055	5.63E-05	0.00093	2.11462
SIX3	-3.7001	-0.0536	-8.3759	0.00016	0.00178	0.94606
CLDN11	-3.6816	3.60225	-14.525	6.71E-06	0.00026	4.49944
MLPH	-3.6744	0.33158	-7.3642	0.00032	0.00275	0.13795
NEBL	-3.6643	2.97044	-7.408	0.00031	0.00271	0.17481
RTP4	-3.6596	-1.7963	-9.7034	6.90E-05	0.00105	1.88546
GCNT3	-3.6584	0.47419	-11.212	3.02E-05	0.00064	2.81862
ACTN2	-3.6574	-5.0678	-9.2767	8.90E-05	0.00123	1.59686
PLSCR4	-3.6445	-3.783	-12.667	1.49E-05	0.00041	3.61082
ELAPOR1	-3.6439	-3.7392	-7.1397	0.00038	0.0031	-0.0539
COL14A1	-3.6368	-3.776	-8.8337	0.00012	0.00148	1.28423
CA8	-3.6329	0.41492	-4.9178	0.00267	0.01186	-2.2552
DPYSL4	-3.6253	-2.4009	-5.4273	0.00162	0.0084	-1.6963
ENTREP2	-3.6244	1.3853	-21.258	7.11E-07	7.30E-05	6.93215
TSPEAR-	-3.6003	-4.4439	-7.9049	0.00022	0.00217	0.58079
LUCAT1	-3.5906	-4.6119	-5.0986	0.00223	0.01041	-2.0527
COL15A1	-3.5863	-3.2523	-6.4551	0.00066	0.00444	-0.6705

MYH3	-3.5741	-2.5324	-9.8334	6.40E-05	0.001	1.97112
BCO1	-3.5723	0.33754	-30.11	8.97E-08	2.48E-05	9.0286
KLF2	-3.5694	2.22079	-11.66	2.41E-05	0.00056	3.0732
WT1	-3.5589	-3.295	-5.3766	0.0017	0.00867	-1.7502
OAS1	-3.5316	-0.0261	-14.296	7.36E-06	0.00026	4.3962
C1orf127	-3.521	-0.8127	-29.047	1.11E-07	2.82E-05	8.82086
AKR1C3	-3.5167	1.049	-11.588	2.50E-05	0.00057	3.03265
MYO1B-A	-3.5159	-3.4493	-16.385	3.31E-06	0.00017	5.27763
JPH3	-3.5103	-1.4172	-12.031	2.01E-05	0.0005	3.27649
SOX6	-3.4986	2.2814	-4.5642	0.00384	0.0154	-2.6643
CNRIP1	-3.4898	-1.8839	-4.85	0.00286	0.01246	-2.3322
SPX	-3.4884	-2.1522	-12.685	1.48E-05	0.00041	3.62012
SH2D1B	-3.4802	-4.2033	-7.4422	0.0003	0.00268	0.20351
FAAHP1	-3.4749	-2.0277	-6.4646	0.00065	0.00442	-0.6616
GREB1	-3.4456	-0.8647	-17.639	2.14E-06	0.00013	5.75122
AMBP	-3.4421	-2.0646	-9.2177	9.23E-05	0.00126	1.55606
TLR6	-3.4369	-3.7116	-9.7698	6.64E-05	0.00103	1.92932
MAJIN	-3.4364	-3.7178	-16.809	2.85E-06	0.00016	5.44216
SLC22A1	-3.4352	-1.3638	-12.446	1.65E-05	0.00045	3.49664
EPDR1	-3.415	0.84577	-8.5065	0.00014	0.0017	1.04409
NKD2	-3.3963	-1.7378	-6.5216	0.00062	0.00428	-0.6083
STAC	-3.3808	1.19221	-25.392	2.47E-07	4.17E-05	8.0242
HSD17B1	-3.3795	0.51608	-6.8984	0.00046	0.00349	-0.2657
SRPX2	-3.376	-1.7898	-9.6077	7.30E-05	0.00109	1.82181
HSPA12B	-3.3647	-3.1669	-12.723	1.45E-05	0.00041	3.63975
PLCD4	-3.3365	-3.014	-13.03	1.26E-05	0.00037	3.79462
GNG4	-3.3286	-2.6654	-8.1262	0.00019	0.00198	0.75467
TRIM9	-3.3274	-1.7914	-13.673	9.55E-06	0.00031	4.10697
DTX1	-3.313	-3.6632	-38.438	2.09E-08	1.35E-05	10.3678
MMP19	-3.3123	-1.7398	-4.596	0.00371	0.01504	-2.6269
LINC0184	-3.3103	-2.3214	-6.4719	0.00065	0.0044	-0.6548
LDLRAD4	-3.3089	0.85611	-21.555	6.55E-07	6.80E-05	7.01857
DAPL1	-3.3027	-4.7908	-5.4349	0.00161	0.00837	-1.6882
LINC0183	-3.2997	0.31069	-10.012	5.77E-05	0.00095	2.08708
DUSP6	-3.2878	2.96797	-10.93	3.49E-05	0.0007	2.65368
KCNE3	-3.2595	-0.4579	-6.3598	0.00071	0.00469	-0.7603
HABP2	-3.2558	1.68372	-6.3953	0.00069	0.00459	-0.7267
MAN1C1	-3.2505	-2.5553	-8.4833	0.00015	0.00171	1.02679
LINC0270	-3.2499	-2.4355	-9.6689	7.04E-05	0.00107	1.86258
APLN	-3.2461	0.09926	-5.2792	0.00187	0.00923	-1.855
ADAMTS8	-3.2407	-1.3176	-4.5783	0.00378	0.01524	-2.6478
MORC4	-3.2373	2.44931	-14.482	6.83E-06	0.00026	4.47994
ENTPD1	-3.2371	1.26713	-11.864	2.18E-05	0.00053	3.18578
HLA-DPB	-3.2336	0.20371	-15.394	4.77E-06	0.00021	4.8751
PTGS2	-3.2293	-4.5246	-11.306	2.88E-05	0.00061	2.87294
PDZD4	-3.2247	-2.8087	-12.594	1.54E-05	0.00042	3.57326
LINC0064	-3.2203	-3.2794	-3.8579	0.00839	0.02734	-3.5365
TRIM6	-3.2178	0.01597	-12.128	1.92E-05	0.00049	3.32849
PCDH12	-3.209	-4.4816	-6.0944	0.00089	0.00551	-1.0159
LINC0048	-3.2021	-5.1861	-7.6265	0.00027	0.00246	0.35607
ACVRL1	-3.1948	1.22388	-13.058	1.25E-05	0.00037	3.80809
GRIK4	-3.191	-0.5421	-4.9825	0.0025	0.01128	-2.1821
FAM43A	-3.1874	2.5625	-9.0082	0.00011	0.00137	1.40904
FAM66C	-3.1765	-2.1805	-9.886	6.21E-05	0.00098	2.00545
TMEM101	-3.1721	3.05417	-5.7481	0.00121	0.00684	-1.3621
WNK4	-3.1699	-0.0416	-5.8505	0.0011	0.00641	-1.2582
ADAMTS3	-3.1579	0.344	-20.776	8.14E-07	8.01E-05	6.78858
TRPV2	-3.154	-2.5146	-10.491	4.42E-05	0.00081	2.38878

SAMD5	-3.1509	1.81215	-16.615	3.05E-06	0.00016	5.36732
OPRD1	-3.1429	-0.3021	-11.551	2.54E-05	0.00057	3.01176
SAMD14	-3.1427	-2.1104	-11.178	3.07E-05	0.00064	2.79933
LINC0051	-3.1356	-0.2293	-21.538	6.58E-07	6.80E-05	7.01379
AQP5	-3.1339	-1.231	-10.273	4.98E-05	0.00087	2.25302
RGS11	-3.1205	-1.0699	-5.1859	0.00205	0.00982	-1.9566
PLIN2	-3.117	3.36262	-5.872	0.00108	0.00632	-1.2366
CDH13	-3.1148	2.06008	-8.7598	0.00012	0.00152	1.23069
ROR2	-3.1126	0.40658	-10.179	5.25E-05	0.00089	2.19361
KLRG1	-3.108	-3.7197	-11.135	3.14E-05	0.00065	2.77406
ARL14EP	-3.1073	-4.7065	-5.2153	0.00199	0.00962	-1.9245
ARHGAP(	-3.1036	1.41536	-24.053	3.41E-07	4.82E-05	7.69541
CHST9	-3.0986	0.68694	-18.453	1.64E-06	0.00011	6.03912
IFI44L	-3.0965	1.05558	-6.0784	0.0009	0.00555	-1.0316
GPR173	-3.0913	-1.3257	-5.0687	0.00229	0.01061	-2.0858
SLC4A5	-3.0829	-2.4277	-10.781	3.78E-05	0.00073	2.56516
DOCK2	-3.079	0.46167	-13.95	8.50E-06	0.00029	4.23736
GPR55	-3.0749	-1.8401	-6.3362	0.00072	0.00476	-0.7827
METTL7A	-3.0748	1.91253	-19.809	1.08E-06	9.18E-05	6.48869
SAMD9L	-3.071	1.02685	-8.3889	0.00016	0.00178	0.95589
LINC0064	-3.0672	-2.1196	-8.1219	0.00019	0.00198	0.75134
ZNF610	-3.0602	-0.482	-17.689	2.11E-06	0.00013	5.76918
IRAK3	-3.0602	1.13032	-6.928	0.00045	0.00344	-0.2393
CFAP70	-3.0464	-2.8969	-10.054	5.64E-05	0.00093	2.11408
B4GALNT	-3.041	5.09731	-8.8343	0.00012	0.00148	1.28465
SESN3	-3.0398	2.76503	-9.3367	8.58E-05	0.0012	1.63818
IGFBP1	-3.0116	-3.2902	-3.7162	0.00991	0.03086	-3.7203
CD247	-3.0103	-2.7496	-3.8106	0.00887	0.02844	-3.5976
KIF19	-2.9994	-1.7242	-3.641	0.01083	0.03305	-3.819
FOSB	-2.9985	4.09134	-3.3643	0.01516	0.04261	-4.1887
RHBDL3	-2.9968	-4.7021	-6.7071	0.00053	0.00387	-0.4378
RIMKLA	-2.9884	-2.8224	-6.637	0.00057	0.00403	-0.5018
HLA-DPA	-2.9878	-2.4123	-7.9805	0.00021	0.0021	0.64064
DCHS1	-2.9857	-2.1318	-6.0452	0.00093	0.00566	-1.0642
TIGD4	-2.9823	-3.4719	-8.5019	0.00015	0.0017	1.04069
MIR646H(	-2.9746	-3.4609	-6.5138	0.00063	0.00429	-0.6156
DMRTA1	-2.971	-1.3173	-10.366	4.73E-05	0.00084	2.31118
OAS2	-2.9657	-0.0305	-7.4053	0.00031	0.00271	0.17257
ADAMTS(	-2.9605	-2.5805	-6.2335	0.00079	0.00506	-0.8809
B3GALT5	-2.9416	-1.748	-4.1268	0.00618	0.02186	-3.1959
ABCC2	-2.9368	-1.2096	-8.7464	0.00012	0.00153	1.22096
CHRNA6	-2.9364	-3.3949	-5.7695	0.00119	0.00675	-1.3403
LMO1	-2.9304	-4.3763	-15.193	5.16E-06	0.00021	4.79013
LINC0097	-2.9253	0.15409	-4.2988	0.00511	0.01888	-2.9835
GRAP	-2.9181	-2.4362	-11.968	2.07E-05	0.00051	3.24203
STEAP1	-2.9168	2.3765	-12.532	1.59E-05	0.00043	3.54121
MCC	-2.9066	3.05261	-9.2631	8.98E-05	0.00124	1.58749
SLC16A2	-2.903	-0.6059	-12.718	1.46E-05	0.00041	3.63686
EFHB	-2.9025	-3.0183	-10.958	3.44E-05	0.00069	2.67055
TG	-2.8967	-3.8602	-5.5238	0.00148	0.00791	-1.5944
PPM1L	-2.8913	-0.9391	-7.121	0.00039	0.00313	-0.0701
ECSCR	-2.8824	3.96573	-22.145	5.58E-07	6.36E-05	7.18676
DNAAF11	-2.8805	-2.3762	-5.8251	0.00113	0.00652	-1.2839
CFAP95	-2.876	-1.6945	-4.0823	0.00649	0.02271	-3.2515
PFN2	-2.8742	4.64291	-27.126	1.67E-07	3.37E-05	8.41922
AATK	-2.872	-3.7916	-15.367	4.82E-06	0.00021	4.86377
NPNT	-2.8686	-0.6837	-9.4659	7.94E-05	0.00115	1.72632
TRIM22	-2.8663	-1.8358	-3.2805	0.01683	0.04599	-4.3025

CDH2	-2.8609	4.6356	-8.2985	0.00017	0.00184	0.88722
NPTX1	-2.86	-3.1985	-3.76	0.00941	0.0297	-3.6632
ALDH1L1	-2.8573	-1.7003	-13.264	1.14E-05	0.00035	3.91003
SKAP1	-2.8558	-1.0465	-9.8216	6.44E-05	0.00101	1.96336
NALF2	-2.855	-4.1654	-7.3977	0.00031	0.00271	0.16619
FAR2	-2.8519	-0.4156	-4.9036	0.00271	0.01198	-2.2712
SLC44A5	-2.8431	-2.0447	-10.74	3.87E-05	0.00074	2.54004
RASGRP	-2.8343	-3.299	-15.339	4.88E-06	0.00021	4.85206
LMX1B	-2.8301	-5.0339	-8.8344	0.00012	0.00148	1.2847
SLC1A1	-2.8296	0.95968	-12.031	2.01E-05	0.0005	3.27625
ZNF429	-2.8282	-1.3859	-16.232	3.50E-06	0.00017	5.2173
SMILR	-2.8229	-3.3503	-9.926	6.06E-05	0.00097	2.03143
DNAI3	-2.8177	-3.8428	-11.159	3.10E-05	0.00065	2.78835
ZP1	-2.8176	-3.938	-5.1899	0.00204	0.0098	-1.9523
HGD	-2.8162	5.32908	-18.482	1.63E-06	0.00011	6.04892
ARL9	-2.8148	-2.0373	-7.9157	0.00022	0.00216	0.58936
TESC	-2.8064	-2.4745	-4.7627	0.00312	0.01327	-2.4324
SENCR	-2.8019	3.07548	-17.312	2.39E-06	0.00014	5.63113
PCBP3	-2.7916	-3.872	-5.8156	0.00114	0.00657	-1.2935
LANCL3	-2.7905	-3.0833	-16.652	3.01E-06	0.00016	5.38157
PLXNB3-/	-2.7839	-1.6238	-9.8244	6.43E-05	0.00101	1.96519
NHSL2	-2.7778	2.89797	-19.182	1.31E-06	0.0001	6.2854
CASC15	-2.7708	-1.5549	-14.54	6.67E-06	0.00025	4.50598
WNT10B	-2.7643	-0.2729	-7.4947	0.00029	0.00262	0.24729
LGI2	-2.7627	0.11538	-19.625	1.14E-06	9.31E-05	6.42984
SPEF2	-2.7581	-1.9454	-8.5697	0.00014	0.00164	1.0911
MAPK11	-2.7566	1.94981	-14.183	7.71E-06	0.00027	4.34468
TNFRSF8	-2.751	-3.9928	-3.328	0.01586	0.04408	-4.2379
HOXA10	-2.7474	1.45229	-27.336	1.60E-07	3.37E-05	8.46478
STEAP2	-2.7414	1.28983	-15.359	4.84E-06	0.00021	4.86072
SNPH	-2.738	0.45493	-24.01	3.45E-07	4.82E-05	7.68442
CXCR4	-2.7324	-1.8654	-3.2056	0.01848	0.04944	-4.4051
DNAH6	-2.7304	-2.8401	-30.361	8.54E-08	2.41E-05	9.07609
NKX3-2	-2.7189	-0.9918	-13.457	1.05E-05	0.00034	4.00361
ENG	-2.7188	3.33685	-10.707	3.93E-05	0.00074	2.52012
NEBL-AS	-2.7137	-1.3186	-5.0023	0.00245	0.01112	-2.16
GRK5	-2.7134	1.34374	-9.1472	9.64E-05	0.00129	1.50691
SDK2	-2.7121	-2.8352	-5.1321	0.00216	0.0102	-2.0157
KDM2B-D	-2.7104	-2.9908	-9.1735	9.48E-05	0.00128	1.52528
CCDC180	-2.7099	-2.8933	-6.4861	0.00064	0.00436	-0.6415
SLC47A2	-2.7091	-3.231	-7.9502	0.00021	0.00212	0.61675
LAMA2	-2.6978	1.12562	-19.853	1.07E-06	9.18E-05	6.5026
MTSS1	-2.6954	-0.0149	-6.7366	0.00052	0.00383	-0.411
LIPC	-2.6952	0.52943	-10.203	5.18E-05	0.00088	2.2086
AQP4	-2.6934	-3.3554	-6.9375	0.00045	0.00342	-0.2309
NLGN3	-2.6883	-2.8784	-6.2765	0.00076	0.00493	-0.8397
PODNL1	-2.6879	-3.3223	-9.3869	8.33E-05	0.00118	1.67256
MMP17	-2.6763	-2.1791	-5.4394	0.00161	0.00835	-1.6834
F2RL2	-2.6707	1.52281	-9.4988	7.79E-05	0.00113	1.74861
CDH5	-2.6667	3.79636	-21.983	5.83E-07	6.37E-05	7.14119
PLCH1	-2.6615	-2.8364	-5.5187	0.00149	0.00794	-1.5997
PINLYP	-2.6611	-1.7832	-5.9897	0.00098	0.00585	-1.119
EFEMP1	-2.654	3.31316	-12.334	1.74E-05	0.00046	3.43762
TTLL7	-2.6515	2.2482	-14.317	7.30E-06	0.00026	4.40581
EMILIN3	-2.6434	-2.3782	-3.9069	0.00793	0.0262	-3.4737
ID2-AS1	-2.6378	-2.0212	-16.994	2.67E-06	0.00015	5.5122
ST3GAL5	-2.6203	0.32363	-8.9298	0.00011	0.00142	1.35318
TNFRSF1	-2.6187	-0.2584	-27.152	1.66E-07	3.37E-05	8.42483

CPP	-2.6158	5.47949	-20.009	1.02E-06	9.07E-05	6.55219
HSPA12A	-2.6091	1.31315	-11.145	3.12E-05	0.00065	2.78011
SLC44A4	-2.6048	-2.5642	-7.201	0.00036	0.00299	-0.0011
CD22	-2.6007	-4.7186	-3.4495	0.01365	0.03954	-4.0737
THSD1	-2.5997	-2.0736	-7.3946	0.00031	0.00271	0.16356
B4GALT6	-2.5966	2.06116	-20.426	9.01E-07	8.46E-05	6.68195
RCAN2	-2.5897	-0.7124	-5.4182	0.00164	0.00845	-1.7059
STX18	-2.586	4.04216	-13.25	1.15E-05	0.00035	3.90298
FTCD	-2.577	-3.0493	-7.0554	0.00041	0.00323	-0.1272
IGF2	-2.5763	-2.6503	-9.7599	6.68E-05	0.00103	1.92283
SPAAR	-2.5739	-0.7046	-11.026	3.32E-05	0.00067	2.71063
CNTFR	-2.5687	-2.728	-10.991	3.39E-05	0.00068	2.68959
ADGRG4	-2.5626	-3.8895	-6.5688	0.0006	0.00417	-0.5646
LAMB4	-2.5564	-2.3283	-7.3523	0.00032	0.00276	0.12793
RAB3B	-2.5553	2.13636	-4.8572	0.00284	0.0124	-2.3239
FLI1	-2.55	3.94352	-14.304	7.34E-06	0.00026	4.40008
IGFN1	-2.5464	-3.0049	-3.2419	0.01766	0.04772	-4.3553
MEGF11	-2.5463	-2.2045	-18.605	1.56E-06	0.00011	6.09139
CD34	-2.5418	0.28065	-5.5181	0.00149	0.00794	-1.6004
CYTIP	-2.538	-3.6167	-5.7483	0.00121	0.00684	-1.3619
SCOC-AS	-2.5377	-2.8937	-5.1613	0.0021	0.00999	-1.9836
TNFSF14	-2.5371	-1.9207	-3.3157	0.0161	0.04464	-4.2545
FAM53A	-2.5307	-1.3954	-5.6838	0.00128	0.00715	-1.428
ADAMTS9	-2.53	1.37848	-8.0462	0.0002	0.00205	0.69227
SCN2A	-2.5245	-4.6576	-5.3777	0.0017	0.00866	-1.7491
ZNF345	-2.5216	-1.5015	-7.4856	0.00029	0.00263	0.23969
SLC7A10	-2.5151	-2.7213	-4.1143	0.00626	0.0221	-3.2115
HHEX	-2.5138	4.14047	-10.401	4.64E-05	0.00083	2.33274
FAM110D	-2.5076	-0.4387	-9.4194	8.17E-05	0.00116	1.69476
TAGLN3	-2.4972	-1.4979	-4.0557	0.00669	0.02322	-3.285
TPK1	-2.4916	4.73224	-14.02	8.25E-06	0.00029	4.26973
TMEM229	-2.4905	-2.7863	-7.0096	0.00042	0.00331	-0.1673
EFR3B	-2.4904	-0.0068	-4.3181	0.005	0.0186	-2.96
BAHCC1	-2.4829	-3.3288	-5.4066	0.00166	0.00851	-1.7183
RNASEL	-2.4813	-0.4795	-10.554	4.27E-05	0.00079	2.42727
SLC26A1	-2.4769	-0.555	-9.9288	6.05E-05	0.00097	2.03324
ITGB1-DT	-2.4754	-2.1596	-6.1914	0.00082	0.0052	-0.9216
MMP2	-2.4676	4.70552	-14.426	6.98E-06	0.00026	4.45481
CMKLR2	-2.4619	-2.4381	-14.12	7.92E-06	0.00028	4.3159
CYP27A1	-2.4611	-1.4993	-6.764	0.00051	0.00376	-0.3862
CUX2	-2.4561	-5.4739	-4.8098	0.00298	0.01284	-2.3783
FAM149A	-2.4521	-0.73	-9.7526	6.70E-05	0.00103	1.91799
HDX	-2.4502	-0.6421	-5.5008	0.00152	0.00801	-1.6185
NR2F2-AS	-2.4464	-2.134	-4.1916	0.00575	0.02064	-3.1154
SAMD13	-2.4443	-3.3822	-11.646	2.42E-05	0.00056	3.06525
TECTA	-2.4438	-1.5181	-17.3	2.40E-06	0.00014	5.62678
PRDM16	-2.4435	-3.7711	-4.7459	0.00318	0.01341	-2.4518
ARHGDIG	-2.4407	-3.5763	-4.6081	0.00367	0.0149	-2.6126
LINC0134	-2.4398	-3.3593	-8.3726	0.00016	0.00178	0.94355
HOXA11-	-2.4375	1.85741	-11.823	2.22E-05	0.00053	3.16317
TGM5	-2.4338	-1.3351	-8.1269	0.00019	0.00198	0.75525
CDH26	-2.4292	-2.7913	-7.4893	0.00029	0.00263	0.24275
PRKD1	-2.4284	-0.8163	-4.7762	0.00308	0.01315	-2.4169
ZFP92	-2.4276	-2.6026	-5.3635	0.00173	0.00873	-1.7643
TCAF2	-2.4216	-4.4741	-10.484	4.44E-05	0.00081	2.3841
SLC40A1	-2.4212	0.49893	-11.475	2.64E-05	0.00058	2.96908
CRHBP	-2.4208	-2.8382	-6.1138	0.00088	0.00545	-0.997
EFCAB6-	-2.4205	-2.6178	-5.4056	0.00166	0.00851	-1.7194

AQP5-AS	-2.4199	0.99211	-8.546	0.00014	0.00166	1.07352
EFCAB10	-2.4173	-1.0396	-8.8865	0.00011	0.00144	1.32219
SLC16A4	-2.4163	1.06999	-15.13	5.28E-06	0.00022	4.76343
CP	-2.4114	6.027	-26.792	1.80E-07	3.44E-05	8.34545
RELN	-2.4112	3.30711	-8.1857	0.00018	0.00193	0.80077
RASGRP	-2.4076	-4.3047	-13.949	8.50E-06	0.00029	4.23665
S1PR1	-2.4063	0.93588	-7.4872	0.00029	0.00263	0.24102
PPFIBP2	-2.4029	1.51446	-19.71	1.11E-06	9.23E-05	6.45701
EGF	-2.4024	-0.7113	-10.154	5.33E-05	0.0009	2.17792
MOV10L1	-2.4018	-0.0335	-4.5416	0.00393	0.01564	-2.6911
LONRF2	-2.4009	2.10434	-9.6686	7.04E-05	0.00107	1.8624
MAP3K15	-2.3992	-0.9796	-7.6424	0.00026	0.00244	0.36911
TTYH2	-2.3881	1.06274	-11.998	2.04E-05	0.00051	3.25827
CXCL5	-2.3854	4.49612	-8.4401	0.00015	0.00174	0.99442
COX7A1	-2.3807	-2.9549	-3.489	0.01301	0.03807	-4.0207
PARP11	-2.38	-1.3644	-6.7337	0.00052	0.00383	-0.4136
CTSS	-2.3743	1.81607	-19.075	1.35E-06	0.0001	6.24981
BNC1	-2.3667	-1.8053	-5.2362	0.00195	0.00949	-1.9018
SLC16A6	-2.3578	-1.0712	-8.8303	0.00012	0.00148	1.28173
VAT1	-2.3556	5.31481	-15.727	4.21E-06	0.00019	5.01336
CLMP	-2.3547	0.19621	-6.3222	0.00073	0.0048	-0.796
DPYD	-2.3394	4.65738	-12.169	1.88E-05	0.00048	3.3505
MAP2K6	-2.339	1.33506	-19.021	1.37E-06	0.0001	6.23171
MAGEA6	-2.3369	0.70658	-13.742	9.27E-06	0.00031	4.13999
GABRP	-2.3337	0.09742	-10.845	3.66E-05	0.00071	2.60302
OLFML3	-2.3274	2.62497	-11.519	2.58E-05	0.00058	2.99409
LCTL	-2.3274	0.59192	-9.9878	5.85E-05	0.00095	2.07143
GGT1	-2.3263	0.00472	-6.4629	0.00065	0.00442	-0.6632
ZSCAN18	-2.3226	2.39233	-11.048	3.29E-05	0.00067	2.7234
ITGAX	-2.3182	-4.1369	-4.5247	0.004	0.01584	-2.7113
LHFPL3-A	-2.3157	-1.0532	-4.0979	0.00638	0.02239	-3.232
MLLT11	-2.3134	4.06388	-19.945	1.04E-06	9.09E-05	6.53181
MICE	-2.3048	-2.9191	-5.8062	0.00115	0.00661	-1.3031
EPHX4	-2.3047	0.89541	-7.6466	0.00026	0.00244	0.3725
NLRP1	-2.3045	0.42573	-6.0646	0.00091	0.00559	-1.0451
FHOD3	-2.3008	1.41579	-7.4401	0.0003	0.00268	0.20175
ST6GAL1	-2.2996	0.72693	-6.3156	0.00074	0.00482	-0.8023
HACD4	-2.291	0.38123	-9.7658	6.65E-05	0.00103	1.92672
HHIP-AS1	-2.2873	-1.9373	-6.8091	0.00049	0.00368	-0.3455
ZNF19	-2.2859	-3.3321	-9.9379	6.02E-05	0.00097	2.03915
AHRR	-2.2809	-0.681	-6.6489	0.00056	0.00401	-0.4909
KLHDC7A	-2.28	1.07536	-5.0147	0.00242	0.01103	-2.1461
ADGRA2	-2.2748	0.29333	-11.622	2.45E-05	0.00056	3.05152
SMTNL2	-2.2745	-3.9009	-3.64	0.01085	0.03309	-3.8203
LINC0158	-2.2696	0.48441	-4.4315	0.00442	0.01701	-2.8226
EEF1A2	-2.2682	4.99345	-4.3693	0.00473	0.01788	-2.8978
FGF13	-2.2646	1.5278	-5.0858	0.00226	0.0105	-2.067
IL6-AS1	-2.2545	-1.0773	-10.061	5.61E-05	0.00093	2.1188
SLC26A7	-2.2545	-1.3343	-3.8039	0.00894	0.02861	-3.6062
PDLIM4	-2.2425	2.40277	-5.1	0.00223	0.0104	-2.0512
GALNT14	-2.2358	3.32545	-11.067	3.25E-05	0.00067	2.73465
ULBP2	-2.2346	-0.5449	-3.8922	0.00807	0.02652	-3.4925
LINC0303	-2.2327	-0.0395	-4.169	0.00589	0.02106	-3.1434
NEK10	-2.2297	-1.7665	-7.1251	0.00039	0.00312	-0.0666
ZNF790	-2.2184	-1.6322	-5.1791	0.00206	0.00986	-1.964
PDIA2	-2.2178	-3.7487	-4.0494	0.00674	0.0233	-3.2928
PLPP3	-2.2167	3.43551	-11.856	2.19E-05	0.00053	3.18088
CD79B	-2.2141	-3.422	-4.3324	0.00492	0.0184	-2.9426

CPEB1	-2.213	-1.6878	-11.642	2.43E-05	0.00056	3.06268
THBS4	-2.2099	-2.4596	-5.9879	0.00098	0.00585	-1.1208
LRRC17	-2.2026	0.35093	-13.447	1.05E-05	0.00034	3.99891
PDE2A	-2.2016	-0.3385	-6.413	0.00068	0.00454	-0.7101
SLC3A1	-2.2013	0.12433	-6.2146	0.0008	0.00512	-0.8991
MPZ	-2.2001	-3.3448	-4.1962	0.00572	0.02056	-3.1097
LINC0181	-2.1973	0.12035	-8.7647	0.00012	0.00152	1.23423
LY6G5C	-2.1969	-2.6047	-16.318	3.39E-06	0.00017	5.25128
LIMD2	-2.194	3.02908	-8.9743	0.00011	0.00139	1.38493
TSPAN18	-2.1922	0.81415	-7.4869	0.00029	0.00263	0.24076
JCAD	-2.1909	2.91869	-9.6948	6.93E-05	0.00106	1.87981
TBXAS1	-2.188	1.7924	-15.229	5.09E-06	0.00021	4.80554
APPBP2-	-2.1828	-2.635	-11.984	2.06E-05	0.00051	3.25067
LMO2	-2.1769	0.62487	-5.531	0.00147	0.00788	-1.5867
ARSL	-2.1727	2.06984	-13.995	8.34E-06	0.00029	4.25807
CXCL6	-2.1696	0.60822	-7.32	0.00033	0.0028	0.10053
SLC47A1	-2.1685	-0.2209	-5.0621	0.00231	0.01067	-2.0933
ACSM3	-2.1643	3.03519	-5.3677	0.00172	0.00871	-1.7598
GCAWKR	-2.1566	-1.9816	-5.5954	0.00139	0.00758	-1.5195
COCH	-2.1559	0.11995	-7.9308	0.00021	0.00214	0.60138
SLC2A5	-2.1519	-1.4622	-3.4136	0.01427	0.04077	-4.1221
ITIH5	-2.1515	1.5923	-4.7594	0.00313	0.0133	-2.4362
S100Z	-2.1472	-1.9346	-5.3545	0.00174	0.00877	-1.7739
RPA4	-2.1457	-1.0318	-3.6868	0.01026	0.03171	-3.7588
ASB9	-2.1454	0.42813	-6.7327	0.00052	0.00383	-0.4145
NMRAL2P	-2.1445	-1.0923	-4.0047	0.00709	0.02416	-3.3493
NRGN	-2.144	4.05455	-26.97	1.73E-07	3.37E-05	8.3848
LGR6	-2.1399	-2.0058	-3.3339	0.01574	0.04384	-4.2298
BHLHE41	-2.1383	1.46676	-10.461	4.49E-05	0.00081	2.36987
HORMAD	-2.1281	-1.3987	-4.4067	0.00454	0.01731	-2.8525
VAV3	-2.1274	-0.2371	-4.4575	0.0043	0.01667	-2.7915
ARHGAP	-2.1263	1.93199	-9.742	6.75E-05	0.00104	1.91099
COL24A1	-2.1236	-0.5409	-5.4317	0.00162	0.00838	-1.6916
DYNLT4	-2.1179	-1.4298	-11.491	2.62E-05	0.00058	2.97805
LINC0132	-2.1034	0.34662	-7.3398	0.00033	0.00277	0.11736
ARHGEF	-2.0999	-0.6044	-10.312	4.88E-05	0.00086	2.27762
SCARF2	-2.0983	3.68576	-7.5947	0.00027	0.0025	0.33
RASL10A	-2.096	-2.1406	-7.2632	0.00035	0.00289	0.05223
TP53AIP1	-2.0959	-1.3204	-6.605	0.00058	0.00409	-0.5312
RNLS	-2.0882	-1.064	-4.1462	0.00604	0.02149	-3.1717
B3GNT9	-2.0871	2.22254	-10.048	5.66E-05	0.00094	2.11034
CXCL12	-2.0788	0.73346	-3.7813	0.00918	0.02918	-3.6355
LAMA4	-2.077	-2.2129	-6.7434	0.00052	0.00381	-0.4048
MAPK12	-2.0749	2.82117	-15.122	5.30E-06	0.00022	4.76006
USHBP1	-2.071	-0.8943	-8.182	0.00018	0.00194	0.79785
PLAAT4	-2.0652	1.66512	-8.6597	0.00013	0.00159	1.15753
ITGA5	-2.0645	4.79576	-8.721	0.00013	0.00154	1.20242
OGFRL1	-2.0628	4.36662	-9.6685	7.04E-05	0.00107	1.86235
RAMP2	-2.0616	1.18438	-10.402	4.64E-05	0.00083	2.33345
ACHE	-2.0606	0.54781	-7.0997	0.00039	0.00315	-0.0886
PCDHB9	-2.0583	-1.3664	-4.9803	0.00251	0.0113	-2.1846
BTNL9	-2.0574	1.0202	-15.267	5.01E-06	0.00021	4.82164
SOX5	-2.0572	3.306	-3.7658	0.00934	0.02956	-3.6557
PDE4D	-2.0564	1.77794	-7.9979	0.0002	0.00209	0.6544
SH2B3	-2.0557	2.87021	-14.711	6.23E-06	0.00024	4.58173
ZNF382	-2.0493	0.63738	-12.308	1.76E-05	0.00046	3.42417
IFI44	-2.0463	0.78221	-7.781	0.00024	0.0023	0.4816
ABTB3	-2.046	1.21336	-7.1356	0.00038	0.00311	-0.0575

GDPD1	-2.0448	-0.8873	-3.3188	0.01604	0.04451	-4.2504
TOB1-AS	-2.0447	-2.9928	-16.393	3.30E-06	0.00017	5.28104
MSX1	-2.0441	3.49305	-10.172	5.27E-05	0.00089	2.18949
CCNB3	-2.0415	-2.784	-5.2388	0.00194	0.00948	-1.8989
TLR1	-2.0413	-2.6902	-5.757	0.0012	0.0068	-1.353
XKR4	-2.037	1.6676	-4.5769	0.00379	0.01525	-2.6494
CARD16	-2.0328	-1.343	-16.1	3.67E-06	0.00018	5.16475
CFAP69	-2.0175	-0.9501	-7.7077	0.00025	0.00238	0.42235
RRAGD	-2.0155	3.2429	-10.041	5.68E-05	0.00094	2.1059
PKIG	-2.0098	2.56998	-10.078	5.56E-05	0.00093	2.12934
CEACAM	-2.0034	1.61158	-5.4679	0.00156	0.00818	-1.6533
RARB	-1.9964	-0.6371	-7.5395	0.00028	0.00257	0.28444
DNM1	-1.9932	3.01745	-9.3796	8.36E-05	0.00118	1.66755
ECM1	-1.9921	0.48436	-8.3998	0.00016	0.00178	0.9641
ZNF280A	-1.9898	-0.2634	-7.0922	0.0004	0.00316	-0.0951
VEPH1	-1.9832	2.44178	-9.9017	6.15E-05	0.00098	2.01568
B3GALNT	-1.9825	4.18651	-16.905	2.75E-06	0.00015	5.47868
OASL	-1.9771	0.61592	-4.4107	0.00452	0.01727	-2.8477
TMEM154	-1.9751	0.36121	-6.5074	0.00063	0.0043	-0.6216
KIAA1549	-1.9747	-0.7038	-5.4225	0.00163	0.00842	-1.7014
MMD	-1.9735	3.0389	-12.689	1.48E-05	0.00041	3.62192
ESPNL	-1.9705	-3.0488	-11.304	2.88E-05	0.00061	2.87194
PHETA2	-1.9705	2.84797	-12.849	1.37E-05	0.00039	3.70376
CD7	-1.9673	-4.3353	-6.4634	0.00065	0.00442	-0.6627
SIPA1L2	-1.9649	4.09705	-12.014	2.03E-05	0.00051	3.26732
PIK3CD-A	-1.9638	-2.6332	-8.7145	0.00013	0.00155	1.1977
LINC0298	-1.963	-0.6175	-15.918	3.92E-06	0.00018	5.09152
ITGA10	-1.9619	-2.3333	-12.035	2.01E-05	0.0005	3.27832
DPY19L1	-1.954	-1.1104	-6.5674	0.0006	0.00418	-0.5659
ZNF547	-1.9532	-1.5966	-3.8374	0.00859	0.02781	-3.5629
LINC0141	-1.946	-2.0389	-3.5354	0.0123	0.03641	-3.9588
OTUB2	-1.9427	0.94161	-6.9765	0.00043	0.00336	-0.1965
SERPINA	-1.9422	2.04094	-3.6787	0.01036	0.03195	-3.7694
TBX1	-1.938	-1.4025	-3.5653	0.01186	0.03542	-3.919
GAS1	-1.9368	1.93774	-5.3241	0.00179	0.00895	-1.8066
RHOU	-1.9352	1.78404	-5.5292	0.00148	0.00789	-1.5887
PNPLA7	-1.9341	-2.7713	-8.1252	0.00019	0.00198	0.75387
TMEM37	-1.9313	2.18292	-8.1839	0.00018	0.00194	0.79933
PLEKHG4	-1.93	0.63505	-9.0845	0.0001	0.00133	1.46291
PRSS35	-1.9262	-2.7251	-3.5036	0.01278	0.03759	-4.0012
AGAP2-A	-1.9175	1.46006	-8.9461	0.00011	0.00141	1.36488
STARD8	-1.9129	2.01374	-9.2148	9.25E-05	0.00126	1.55403
DNASE1L	-1.9113	-1.5942	-8.9161	0.00011	0.00143	1.34343
ALDH6A1	-1.9087	4.94402	-15.411	4.74E-06	0.00021	4.88238
CGNL1	-1.9075	3.63934	-10.223	5.12E-05	0.00088	2.22167
HOGA1	-1.9063	-0.4016	-5.7843	0.00117	0.00668	-1.3252
SYT16	-1.9048	-1.7612	-5.0265	0.00239	0.01095	-2.1329
PAPLN	-1.891	-0.5472	-7.3826	0.00032	0.00273	0.15343
PLA2G4C	-1.8909	-4.2492	-4.0284	0.0069	0.02372	-3.3193
FTCDNL1	-1.8852	-2.2275	-4.4536	0.00432	0.01671	-2.7961
LINC0141	-1.885	0.73984	-7.0718	0.0004	0.0032	-0.1129
CHST10	-1.884	2.30973	-7.9263	0.00021	0.00215	0.59777
LBX2-AS1	-1.8811	-0.3676	-6.1494	0.00085	0.00534	-0.9623
TEKT4P2	-1.8778	-0.1951	-8.7463	0.00012	0.00153	1.22087
SCAT1	-1.8776	-1.8016	-3.2468	0.01755	0.04755	-4.3486
TTC28	-1.8761	5.65888	-15.852	4.02E-06	0.00018	5.0646
PLEKHG4	-1.8758	3.99122	-13.863	8.81E-06	0.00029	4.19685
PAXBP1-,	-1.8757	-2.1178	-4.3071	0.00506	0.01876	-2.9733

TBX2-AS	-1.8757	-2.0994	-6.1216	0.00087	0.00543	-0.9893
PIR	-1.8734	3.96055	-15.81	4.08E-06	0.00019	5.04744
CYP1B1	-1.8732	1.46456	-10.012	5.77E-05	0.00095	2.08727
RAB6B	-1.8686	1.61775	-18.985	1.39E-06	0.0001	6.21976
ENTPD8	-1.8658	-1.5707	-18.171	1.80E-06	0.00012	5.94086
ISM1	-1.8657	-1.8149	-3.3492	0.01545	0.04321	-4.2091
FAM102B	-1.8637	0.0699	-8.8833	0.00011	0.00145	1.31988
SYNE1	-1.8592	1.10052	-11.009	3.35E-05	0.00068	2.70047
LY96	-1.8582	-4.5551	-7.1475	0.00038	0.00309	-0.0472
PRAME	-1.8532	5.16877	-11.994	2.05E-05	0.00051	3.25618
PGBD5	-1.8492	0.29759	-8.8963	0.00011	0.00144	1.32922
TGFB1	-1.8448	4.973	-20.027	1.01E-06	9.07E-05	6.55791
EGFL7	-1.843	2.0706	-5.6709	0.0013	0.0072	-1.4413
SLC9A9	-1.8422	-5.1231	-5.1071	0.00221	0.01035	-2.0434
FHAD1	-1.8389	0.19294	-6.2136	0.0008	0.00512	-0.9001
KIF5C	-1.837	-3.4462	-4.6361	0.00356	0.0146	-2.5797
KITLG	-1.8336	-0.4931	-15.783	4.12E-06	0.00019	5.03661
NFKBIZ	-1.8302	3.6289	-6.2867	0.00076	0.0049	-0.8299
ZNF223	-1.8293	-2.0483	-6.0296	0.00094	0.00572	-1.0796
SULT4A1	-1.8292	-0.5651	-10.006	5.79E-05	0.00095	2.08335
ASRGL1	-1.8285	5.49519	-9.2214	9.21E-05	0.00126	1.55859
LINC0031	-1.8279	-0.4159	-3.4274	0.01403	0.04031	-4.1034
CFAP54	-1.8278	-0.1248	-5.4134	0.00165	0.00848	-1.711
EEF1A1P	-1.8257	-0.8933	-5.3682	0.00172	0.00871	-1.7592
OAS3	-1.8215	2.89493	-4.1762	0.00585	0.02093	-3.1345
GPR162	-1.8206	-1.2518	-7.7292	0.00025	0.00236	0.43977
FBXL9P	-1.8191	-2.1594	-4.7066	0.00331	0.01385	-2.4974
CFAP418	-1.819	1.24927	-13.013	1.27E-05	0.00037	3.78607
SYNE3	-1.8181	-0.2659	-10.239	5.08E-05	0.00088	2.23141
CASP1	-1.8174	-0.662	-5.4886	0.00153	0.00806	-1.6314
ANGPTL2	-1.8169	2.35076	-6.1771	0.00083	0.00525	-0.9354
IGFL2-AS	-1.8151	0.59081	-7.5574	0.00028	0.00254	0.29925
SERINC5	-1.8118	3.13322	-6.3034	0.00075	0.00485	-0.8139
PLAAT3	-1.8102	3.2587	-14.624	6.45E-06	0.00025	4.54314
LINC0127	-1.8096	-0.0416	-12.344	1.73E-05	0.00046	3.44312
MTCL1	-1.8071	4.02902	-13.364	1.09E-05	0.00034	3.95891
CCL5	-1.8064	-2.3467	-3.309	0.01624	0.04488	-4.2637
SPATA20	-1.8062	3.86595	-16.532	3.14E-06	0.00016	5.33517
TCN2	-1.8031	2.69906	-13.285	1.13E-05	0.00035	3.92019
FOS	-1.7996	5.81679	-3.4207	0.01414	0.04053	-4.1125
HOXA4	-1.7891	-0.318	-5.1809	0.00206	0.00985	-1.9621
RASSF8-/	-1.7873	0.62822	-18.424	1.66E-06	0.00011	6.02915
LINC0227	-1.7866	-0.8748	-5.647	0.00133	0.00732	-1.466
AMIGO2	-1.7851	6.35711	-7.5542	0.00028	0.00255	0.29662
BCHE	-1.7848	1.27607	-7.696	0.00025	0.00239	0.41281
TMCC1-D	-1.7842	0.66159	-7.3443	0.00033	0.00277	0.12114
MLLT3	-1.7837	0.79519	-7.643	0.00026	0.00244	0.36956
SULT1C2	-1.7836	0.65575	-4.4147	0.0045	0.01721	-2.8428
TMEM64	-1.7826	2.90301	-6.8903	0.00046	0.00351	-0.2729
AP1S2	-1.7809	4.58092	-6.9707	0.00043	0.00337	-0.2016
TNRC6C	-1.7807	1.50797	-7.4366	0.00031	0.00269	0.19875
SLC52A1	-1.7803	-2.8877	-6.014	0.00095	0.00578	-1.095
AKR1C2	-1.7789	-4.3804	-6.8468	0.00048	0.0036	-0.3117
FRMD4A	-1.7782	4.05401	-9.4497	8.02E-05	0.00115	1.71533
CFI	-1.7779	3.89923	-7.0753	0.0004	0.0032	-0.1098
RBM24	-1.7758	0.43762	-9.7258	6.81E-05	0.00104	1.9003
CLU	-1.7742	5.38319	-5.8653	0.00109	0.00635	-1.2434
MFAP3L	-1.7715	-0.0056	-6.6603	0.00056	0.00398	-0.4804

EVC	-1.7714	1.82031	-8.6922	0.00013	0.00156	1.18134
FOLH1	-1.7706	0.42306	-3.4341	0.01391	0.04007	-4.0944
KAT2B	-1.767	2.87757	-8.1976	0.00018	0.00192	0.80988
PLEKHD1	-1.7652	-1.2118	-4.2709	0.00526	0.01933	-3.0176
MAPT	-1.7641	-3.9705	-5.7117	0.00125	0.00703	-1.3994
IFI27L2	-1.7638	3.92904	-11.351	2.81E-05	0.0006	2.89857
GLRX	-1.7634	1.25759	-23.606	3.82E-07	5.10E-05	7.58058
LINC0066	-1.763	1.32414	-20.498	8.82E-07	8.39E-05	6.7042
LINC0092	-1.7629	-2.3862	-5.1641	0.00209	0.00997	-1.9805
SALL1	-1.7619	3.39962	-10.449	4.52E-05	0.00082	2.36263
TNFRSF1	-1.7584	-2.8952	-5.1554	0.00211	0.01003	-1.9901
SIGLEC9	-1.7491	-3.7688	-5.0644	0.0023	0.01065	-2.0907
SLC37A2	-1.748	-0.3731	-5.4155	0.00164	0.00846	-1.7088
TMEM91	-1.7453	-1.575	-4.865	0.00281	0.01235	-2.3151
ZNF589	-1.7377	2.13245	-18.662	1.54E-06	0.00011	6.11068
NCR3LG1	-1.7348	-1.504	-3.3016	0.01639	0.04516	-4.2737
ANXA2R	-1.7322	0.52002	-9.1551	9.59E-05	0.00129	1.51244
DAPK2	-1.731	-2.8587	-9.2423	9.09E-05	0.00125	1.5731
THNSL2	-1.7287	0.09788	-13.014	1.27E-05	0.00037	3.78663
THPO	-1.7249	-2.7259	-4.5281	0.00399	0.01582	-2.7072
PLAAT1	-1.7239	-1.0001	-6.9029	0.00046	0.00349	-0.2616
SMPD1	-1.7227	3.68189	-8.1307	0.00019	0.00198	0.75815
RPGRIP1	-1.7221	-2.0062	-3.992	0.00719	0.02444	-3.3654
MAMLD1	-1.7199	0.17196	-4.6408	0.00354	0.01454	-2.5742
RGL1	-1.7175	2.94889	-7.8696	0.00022	0.00221	0.55266
UAP1L1	-1.7163	3.63867	-17.956	1.93E-06	0.00012	5.8648
WNT3	-1.7129	0.61521	-10.244	5.07E-05	0.00088	2.23447
ITPR1	-1.7125	1.90991	-9.0905	9.98E-05	0.00133	1.46714
SCUBE1	-1.7124	-5.6886	-6.6868	0.00054	0.00392	-0.4563
STEAP1B	-1.7087	0.99773	-18.003	1.90E-06	0.00012	5.88147
FABP3	-1.7077	3.17926	-10.405	4.63E-05	0.00083	2.3353
CAPN5	-1.7077	2.01713	-7.5571	0.00028	0.00254	0.29895
LINC0092	-1.7074	-2.4565	-8.09	0.00019	0.00201	0.72649
ADAM11	-1.7073	0.29504	-10.87	3.61E-05	0.00071	2.6184
MGAT5B	-1.7025	2.79968	-10.531	4.33E-05	0.00079	2.41322
RAMP2-A	-1.7018	-0.4197	-6.699	0.00054	0.00389	-0.4451
RASL12	-1.6957	0.70855	-6.6036	0.00058	0.00409	-0.5325
SPATA7	-1.6953	-0.9384	-5.9119	0.00104	0.00616	-1.1966
SLC4A8	-1.694	-1.5329	-4.9876	0.00249	0.01125	-2.1765
KIAA1614	-1.6938	-1.7984	-3.3786	0.0149	0.04205	-4.1693
MVB12B	-1.6891	-0.4673	-3.4925	0.01296	0.03797	-4.0161
ZNF320	-1.6826	1.22314	-7.9064	0.00022	0.00217	0.58203
PLA1A	-1.6814	-1.0066	-6.9122	0.00045	0.00347	-0.2534
SH3PXD2	-1.6805	-0.1968	-10.626	4.11E-05	0.00077	2.47146
ZEB1	-1.6797	2.91436	-10.817	3.71E-05	0.00072	2.58649
HAP1	-1.6796	-2.9669	-8.3525	0.00016	0.0018	0.92827
MSRA	-1.6787	1.43467	-8.4546	0.00015	0.00173	1.00533
C8orf88	-1.6766	1.73554	-17.227	2.46E-06	0.00014	5.59958
AK9	-1.6766	-1.621	-9.4863	7.84E-05	0.00114	1.74016
GSG1L	-1.6765	-1.6338	-7.0493	0.00041	0.00325	-0.1326
ZNF423	-1.6744	1.32453	-4.4139	0.00451	0.01722	-2.8438
ZNF502	-1.6744	-2.7372	-5.3554	0.00174	0.00877	-1.773
LINC0085	-1.6688	-2.5354	-10.43	4.57E-05	0.00082	2.35119
NR2F2	-1.6683	4.6518	-11.685	2.38E-05	0.00055	3.08702
HOXA3	-1.668	2.34905	-9.5842	7.40E-05	0.0011	1.80607
FOXN3-A	-1.6635	1.55674	-7.4462	0.0003	0.00268	0.20686
MEIOC	-1.6607	0.82906	-3.4792	0.01317	0.03846	-4.0339
SSPOP	-1.659	0.06997	-5.3545	0.00174	0.00877	-1.7739

APBB1IP	-1.6582	-2.2227	-5.9733	0.00099	0.00591	-1.1353
RASGRF2	-1.6541	3.59641	-19.127	1.33E-06	0.0001	6.2669
CEP126	-1.6515	1.0169	-18.484	1.63E-06	0.00011	6.04987
LPCAT2	-1.6487	3.04616	-4.7068	0.00331	0.01385	-2.4972
SLC30A3	-1.6485	-5.0045	-4.4559	0.00431	0.01668	-2.7934
LRRC24	-1.6471	0.83188	-11.093	3.21E-05	0.00066	2.74948
PLA2G4A	-1.644	-0.0271	-8.0235	0.0002	0.00207	0.67453
MIAT	-1.6416	-2.1	-13.561	1.00E-05	0.00032	4.05383
ONECUT1	-1.639	-1.3584	-7.5792	0.00028	0.00252	0.31718
HCLS1	-1.6372	1.06995	-7.34	0.00033	0.00277	0.11749
GLT8D2	-1.6355	2.00716	-13.717	9.37E-06	0.00031	4.1282
CYP39A1	-1.6322	-1.4593	-7.3556	0.00032	0.00276	0.13067
ADORA1	-1.6312	4.04958	-7.4039	0.00031	0.00271	0.17134
FLRT3	-1.6284	2.01173	-11.472	2.64E-05	0.00058	2.96736
CXCL1	-1.6271	5.98818	-4.6144	0.00364	0.01483	-2.6052
BRPF3-A	-1.6271	-3.5159	-4.3244	0.00497	0.01851	-2.9523
SCNN1D	-1.6263	-0.9573	-7.531	0.00028	0.00257	0.27737
GAREM2	-1.6262	0.55949	-16.847	2.81E-06	0.00015	5.45647
BATF2	-1.6246	-2.2408	-3.5376	0.01227	0.03636	-3.956
TRABD2A	-1.6222	-0.4504	-5.6504	0.00132	0.00731	-1.4625
CACNA2D	-1.6212	0.1854	-8.252	0.00017	0.00188	0.85171
DND1	-1.621	-0.0191	-4.7371	0.00321	0.01351	-2.4621
TSHZ1	-1.62	0.94569	-4.8893	0.00274	0.01211	-2.2875
NXPE3	-1.6198	3.96407	-14.801	6.01E-06	0.00024	4.62117
DGCR6	-1.6197	2.12504	-12.15	1.90E-05	0.00048	3.34017
CAMK1G	-1.6193	-4.0062	-3.7581	0.00943	0.02975	-3.6657
ZBTB20	-1.6186	1.82992	-4.8849	0.00276	0.01214	-2.2925
TEX19	-1.6183	-1.0745	-3.9821	0.00727	0.02465	-3.3779
CFH	-1.6165	4.39899	-4.3581	0.00479	0.01799	-2.9114
SELENBP	-1.6162	2.72125	-13.892	8.70E-06	0.00029	4.21046
LBX2	-1.6105	-1.1024	-5.0764	0.00228	0.01057	-2.0773
MYO7A	-1.6085	-0.2405	-11.622	2.45E-05	0.00056	3.05155
SLC49A3	-1.6058	-4.5113	-4.9087	0.00269	0.01193	-2.2654
MERTK	-1.6057	1.25835	-16.232	3.50E-06	0.00017	5.2174
VWDE	-1.6038	-3.3586	-3.2657	0.01714	0.04666	-4.3228
MAP3K7C	-1.6036	-0.3159	-10.484	4.44E-05	0.00081	2.38419
CPM	-1.6036	5.05357	-7.9544	0.00021	0.00212	0.62007
RUNDC3A	-1.5911	-1.8755	-4.7121	0.00329	0.01379	-2.4911
OPRL1	-1.5894	-1.7145	-4.75	0.00316	0.01337	-2.4472
METTL27	-1.5862	-1.8627	-3.2675	0.0171	0.04659	-4.3202
FIBCD1	-1.5853	-4.8963	-3.6659	0.01052	0.03233	-3.7862
LINC0293	-1.5852	-1.2912	-4.3134	0.00503	0.01868	-2.9657
TOR4A	-1.582	3.56824	-15.929	3.91E-06	0.00018	5.09605
UCHL1	-1.5804	5.92525	-9.9716	5.91E-05	0.00096	2.06094
PRUNE2	-1.5764	2.90095	-8.0263	0.0002	0.00207	0.67669
PROCR	-1.575	4.08908	-10.808	3.73E-05	0.00072	2.58095
RIMKLB	-1.5748	4.5065	-3.3604	0.01524	0.04277	-4.1939
KIF26B	-1.5747	3.32574	-3.6631	0.01055	0.0324	-3.7899
MARCHF	-1.5714	-0.3544	-10.587	4.20E-05	0.00078	2.44725
PRKAR2E	-1.5681	2.88633	-12.238	1.82E-05	0.00047	3.387
PPM1F	-1.5679	4.73261	-11.367	2.79E-05	0.0006	2.90797
HOXB13	-1.5668	1.39389	-10.911	3.53E-05	0.0007	2.64277
PHLDA1	-1.5653	2.81333	-4.5202	0.00402	0.01591	-2.7166
RGS2	-1.5649	2.76584	-7.1652	0.00037	0.00306	-0.0319
FAM222A	-1.5598	1.22513	-4.5046	0.00409	0.01607	-2.7352
GPAT3	-1.5593	0.61869	-3.754	0.00948	0.02984	-3.671
HOXA-AS	-1.5483	1.68317	-14.104	7.97E-06	0.00028	4.30877
CACHD1	-1.5465	4.41918	-4.8837	0.00276	0.01216	-2.2938

MACC1-D	-1.5458	0.23635	-8.2004	0.00018	0.00192	0.81206
COL5A2	-1.5457	1.5484	-15.385	4.79E-06	0.00021	4.87143
FAM89A	-1.5449	0.43488	-7.5256	0.00029	0.00258	0.27294
TP53TG1	-1.5436	0.88769	-7.1016	0.00039	0.00315	-0.087
TMEM98	-1.5419	0.74745	-5.4109	0.00165	0.00849	-1.7137
MAP1B	-1.539	5.25654	-7.3814	0.00032	0.00273	0.15242
SNHG25	-1.5383	1.50117	-5.2233	0.00197	0.00956	-1.9158
UBAP1L	-1.5326	-2.6205	-6.3119	0.00074	0.00483	-0.8059
ARHGEF2	-1.5314	2.97519	-9.6501	7.12E-05	0.00107	1.85009
HOMER3	-1.5288	-1.8476	-5.3225	0.00179	0.00896	-1.8083
DAB2	-1.5283	1.16849	-11.139	3.13E-05	0.00065	2.77637
GNG2	-1.525	-0.4007	-7.2815	0.00034	0.00286	0.06782
SLC19A3	-1.5248	-1.023	-3.4624	0.01344	0.03909	-4.0564
TLR5	-1.5245	-1.158	-15.262	5.02E-06	0.00021	4.81983
ETFBKMT	-1.5228	-0.8257	-11.534	2.56E-05	0.00058	3.00222
SNX10	-1.5215	2.3694	-13.074	1.24E-05	0.00037	3.81639
NECAB2	-1.5205	-1.7069	-4.5922	0.00373	0.01509	-2.6313
PTK6	-1.5196	-2.4505	-5.6834	0.00128	0.00715	-1.4284
DYNC1I1	-1.5194	1.86672	-7.0241	0.00042	0.00329	-0.1546
RIPK2	-1.5193	5.0929	-14.322	7.29E-06	0.00026	4.40788
APOBEC3	-1.519	-2.2905	-3.7918	0.00906	0.02891	-3.622
LINC0051	-1.5175	1.70556	-9.9036	6.14E-05	0.00098	2.01688
PDK1-AS	-1.5132	-2.0868	-4.3596	0.00478	0.01798	-2.9095
RAB27B	-1.5131	-3.2025	-9.0473	0.0001	0.00135	1.4367
ICAM2	-1.513	-2.2594	-3.6634	0.01055	0.0324	-3.7895
SNHG26	-1.512	-1.72	-3.6453	0.01078	0.03294	-3.8134
RBPMS-A	-1.5105	-0.5652	-7.3646	0.00032	0.00275	0.13831
GABRE	-1.5101	3.44431	-6.1147	0.00087	0.00545	-0.9961
SPP1	-1.5052	7.14264	-6.0662	0.00091	0.00558	-1.0436
CFL1P1	-1.5038	-2.6148	-5.5697	0.00142	0.0077	-1.5463
ZNF667	-1.4998	0.69009	-8.6393	0.00013	0.00159	1.14257
MYLK	-1.4992	6.24114	-7.7166	0.00025	0.00237	0.42952
CLDN15	-1.4965	2.65596	-9.1759	9.47E-05	0.00128	1.52691
RFFL	-1.4964	1.90814	-9.646	7.14E-05	0.00107	1.84737
KCNAB2	-1.4962	3.29645	-8.8925	0.00011	0.00144	1.32649
SEMA6B	-1.4947	3.61795	-4.0685	0.00659	0.02298	-3.2688
SLC38A4	-1.4927	-2.0932	-3.316	0.0161	0.04463	-4.2542
PLB1	-1.4914	-1.5271	-3.8431	0.00854	0.02766	-3.5556
PNMA2	-1.4877	0.89218	-8.9617	0.00011	0.0014	1.37594
TMEM140	-1.4874	-0.8325	-6.0133	0.00096	0.00578	-1.0957
FMO4	-1.4869	-1.1747	-10.619	4.12E-05	0.00077	2.46727
STUM	-1.4867	-2.0252	-4.4582	0.0043	0.01666	-2.7906
EIF5A2	-1.4863	1.72625	-9.8614	6.29E-05	0.00099	1.98937
PREX1	-1.4849	4.47122	-3.8599	0.00837	0.0273	-3.534
DENND3-	-1.482	-0.6003	-6.6056	0.00058	0.00409	-0.5307
SARDH	-1.4812	-1.1342	-5.3907	0.00168	0.00859	-1.7353
CEP112	-1.4803	1.54623	-8.0555	0.0002	0.00204	0.69955
CACNA1D	-1.4761	-0.2311	-5.7006	0.00126	0.00708	-1.4108
KCNH3	-1.4741	1.46233	-4.8294	0.00292	0.01265	-2.3558
DNMBP	-1.4741	2.39904	-7.5794	0.00027	0.00252	0.31734
LAMA1	-1.473	4.71993	-4.6998	0.00333	0.01391	-2.5054
ANKRD6	-1.4691	3.38793	-7.543	0.00028	0.00256	0.28736
NID1	-1.4656	3.3137	-8.7727	0.00012	0.00151	1.24007
TAMALIN	-1.4652	2.49539	-7.5653	0.00028	0.00254	0.30578
CDK5R1	-1.4631	1.13535	-4.9141	0.00268	0.01189	-2.2593
CYP2D6	-1.4562	-1.7118	-4.8352	0.0029	0.0126	-2.3491
RESF1	-1.4559	5.29387	-8.2655	0.00017	0.00187	0.86206
RFX5-AS	-1.4553	0.17378	-10.317	4.86E-05	0.00086	2.28049

PCTP	-1.4551	2.71224	-6.8608	0.00047	0.00357	-0.2992
C1orf226	-1.455	1.4535	-11.94	2.10E-05	0.00051	3.22703
IFI35	-1.4543	2.49006	-12.746	1.44E-05	0.0004	3.65114
HOXA13	-1.4504	0.83679	-4.5172	0.00404	0.01595	-2.7201
NDUFA6-	-1.4482	-1.3439	-6.5298	0.00062	0.00427	-0.6008
LIPH	-1.4466	-2.0522	-4.3068	0.00506	0.01876	-2.9737
ADAM8	-1.4464	0.10104	-9.1056	9.89E-05	0.00132	1.47772
MPP1	-1.4456	3.13609	-9.295	8.80E-05	0.00123	1.60952
PRODH	-1.4455	0.88784	-3.3101	0.01622	0.04483	-4.2623
CAMK1	-1.445	1.27519	-13.298	1.12E-05	0.00035	3.9268
NWD1	-1.4449	-0.4104	-6.9706	0.00043	0.00337	-0.2016
FOXF2	-1.4439	1.42907	-6.2057	0.00081	0.00514	-0.9077
MCIDAS	-1.4438	-2.1152	-3.402	0.01447	0.04118	-4.1376
CYP2J2	-1.439	1.41588	-7.9654	0.00021	0.00212	0.62878
SCARB1	-1.4385	4.74674	-13.109	1.22E-05	0.00037	3.83353
TBC1D8	-1.4361	4.12393	-7.6198	0.00027	0.00247	0.35055
ZNF114	-1.4357	2.36777	-4.4843	0.00418	0.01634	-2.7594
GLI4	-1.4331	2.67108	-7.2038	0.00036	0.00298	0.00138
SDSL	-1.4327	1.95496	-8.3419	0.00016	0.00181	0.92023
SLC25A4	-1.4326	0.14138	-4.1894	0.00576	0.02068	-3.1181
DCBLD2	-1.4319	6.0452	-9.9394	6.02E-05	0.00097	2.0401
PTGIS	-1.4314	1.54275	-10.257	5.03E-05	0.00087	2.24301
CACNA1(	-1.43	-1.5057	-4.4848	0.00418	0.01634	-2.7588
SLC6A20	-1.43	-2.4571	-4.7043	0.00332	0.01387	-2.5001
APBA2	-1.4299	3.18967	-18.109	1.84E-06	0.00012	5.91902
CTHRC1	-1.4288	2.17529	-7.5345	0.00028	0.00257	0.28027
DUSP19	-1.4263	-0.4958	-9.6127	7.28E-05	0.00109	1.82514
ZBTB47	-1.4225	1.19126	-7.016	0.00042	0.0033	-0.1618
SNRPA1-	-1.422	-3.0138	-3.1948	0.01874	0.04987	-4.4199
SNRK	-1.4189	2.31385	-11.462	2.66E-05	0.00058	2.96194
PMP22	-1.4169	0.96478	-6.4553	0.00066	0.00444	-0.6703
KCNJ5-A!	-1.4159	1.16948	-8.083	0.00019	0.00202	0.72102
CAPS2	-1.4152	-1.2118	-11.401	2.74E-05	0.00059	2.92745
ZNF134	-1.415	0.44326	-14.297	7.36E-06	0.00026	4.39671
FGFR4	-1.4143	5.2216	-15.539	4.52E-06	0.0002	4.93576
ELOVL2	-1.4107	2.35879	-8.5786	0.00014	0.00164	1.09775
BTBD19	-1.4104	1.70249	-6.3734	0.0007	0.00465	-0.7474
AKAP1-D	-1.4082	-1.3672	-3.2965	0.01649	0.04536	-4.2807
BRSK2	-1.4076	-2.5842	-7.4942	0.00029	0.00262	0.24687
KIF26A	-1.4074	0.83568	-4.0251	0.00693	0.02379	-3.3235
RUNX3	-1.4072	1.67318	-12.78	1.42E-05	0.0004	3.6687
MAST4	-1.4072	2.23874	-8.5561	0.00014	0.00165	1.08104
PAPSS2	-1.4068	4.32726	-7.9616	0.00021	0.00212	0.62577
LINC0115	-1.4059	0.13023	-3.3441	0.01555	0.04341	-4.216
PSMG3-A	-1.4058	-1.0176	-3.806	0.00891	0.02855	-3.6035
SGMS1-A	-1.4043	-0.6179	-4.4423	0.00437	0.01685	-2.8097
TNKS2-D	-1.4013	-2.0024	-6.3263	0.00073	0.00479	-0.7921
C17orf58	-1.3974	3.44686	-10.094	5.51E-05	0.00092	2.13991
SEPTIN4	-1.3968	1.11289	-8.5265	0.00014	0.00168	1.059
DNAAF8	-1.3962	-2.9185	-3.2158	0.01825	0.04897	-4.391
ZNF667-A	-1.3931	0.66807	-11.446	2.68E-05	0.00059	2.95284
BNIP3	-1.3925	3.74144	-7.8258	0.00023	0.00225	0.51765
HOXA2	-1.3916	0.85424	-4.3476	0.00484	0.01814	-2.9241
ARHGAP	-1.3905	3.51277	-9.266	8.96E-05	0.00124	1.58947
LHFPL2	-1.3891	3.39489	-10.468	4.48E-05	0.00081	2.37424
SYTL4	-1.3888	0.63002	-17.037	2.63E-06	0.00015	5.5286
KCNC4	-1.3885	0.83164	-9.0397	0.0001	0.00135	1.43134
BMERB1	-1.3864	-1.2691	-6.2595	0.00077	0.00498	-0.8559

RHCE	-1.3819	-1.0121	-11.068	3.25E-05	0.00067	2.73511
ARHGAP:	-1.3811	-2.9158	-4.4805	0.0042	0.01638	-2.764
POMGNT	-1.3802	2.51669	-11.401	2.74E-05	0.00059	2.92695
SMIM6	-1.3778	-0.0596	-4.9406	0.00261	0.01164	-2.2293
PDGFRL	-1.3734	0.03696	-7.4007	0.00031	0.00271	0.16872
FLT4	-1.3733	-2.5436	-3.5792	0.01167	0.03503	-3.9007
CATSPEI	-1.3728	-1.3219	-5.3083	0.00182	0.00904	-1.8236
GRIN2B	-1.3721	-0.0304	-3.4291	0.014	0.04026	-4.1011
ANXA2R-	-1.3716	0.72962	-10.903	3.54E-05	0.0007	2.63781
SEMA3G	-1.3704	0.65214	-3.643	0.01081	0.03299	-3.8164
NLGN1	-1.3681	1.95408	-5.1788	0.00206	0.00986	-1.9644
APH1B	-1.3672	0.75683	-6.1225	0.00087	0.00543	-0.9884
TPBG	-1.3666	2.91574	-10.315	4.87E-05	0.00086	2.27913
ABCA1	-1.3663	1.96445	-3.4848	0.01308	0.03823	-4.0263
MGST3	-1.3662	4.68972	-12.623	1.52E-05	0.00042	3.58816
SCIRT	-1.3652	-1.2371	-6.7413	0.00052	0.00382	-0.4067
RAB31	-1.3645	3.25271	-9.3824	8.35E-05	0.00118	1.66946
XAF1	-1.3636	0.69141	-6.8583	0.00047	0.00357	-0.3014
THBS2-A	-1.3617	-2.4383	-3.4274	0.01403	0.04031	-4.1035
KDM4A-A	-1.3602	-1.9779	-4.735	0.00321	0.01353	-2.4645
PPP1R3C	-1.3584	-0.7386	-7.5465	0.00028	0.00256	0.29024
CMTM3	-1.3579	2.11372	-8.2309	0.00017	0.00189	0.83554
RPS6KA5	-1.3571	3.37557	-14.59	6.54E-06	0.00025	4.52835
CCDC116	-1.3541	-0.8978	-4.7043	0.00332	0.01387	-2.5001
LAMC3	-1.3527	1.61777	-6.7674	0.00051	0.00376	-0.3831
OSGIN1	-1.3517	1.75014	-15.062	5.43E-06	0.00022	4.7342
NPEPL1	-1.3515	0.09826	-7.6747	0.00026	0.00242	0.39547
CA12	-1.3472	5.3835	-6.5597	0.0006	0.00419	-0.573
WIPF1	-1.3467	3.74853	-17.005	2.66E-06	0.00015	5.51644
PTPRB	-1.3458	1.59508	-4.4131	0.00451	0.01723	-2.8448
PCDHB5	-1.3452	1.12758	-8.0953	0.00019	0.00201	0.73063
PYROXD:	-1.3426	0.47781	-9.2146	9.25E-05	0.00126	1.55387
SHBG	-1.3399	-2.5588	-8.8425	0.00012	0.00148	1.29052
LPAR3	-1.3392	1.75119	-9.9965	5.82E-05	0.00095	2.077
ABCA5	-1.3377	1.39349	-11.125	3.16E-05	0.00065	2.7682
FAM21EF	-1.3365	-2.5669	-3.6374	0.01088	0.03316	-3.8237
GPR153	-1.3321	1.68791	-11.791	2.26E-05	0.00054	3.14568
GPRIN3	-1.331	-0.0117	-4.46	0.00429	0.01664	-2.7885
HEYL	-1.3306	-0.445	-5.9232	0.00103	0.00611	-1.1852
SDCBP2-	-1.3286	-0.6724	-9.413	8.20E-05	0.00116	1.69036
OSBPL5	-1.3249	1.3271	-8.1488	0.00018	0.00196	0.77217
LINC0066	-1.3247	-1.6546	-8.0088	0.0002	0.00208	0.66293
SLC16A7	-1.3243	3.07593	-5.8599	0.00109	0.00637	-1.2488
GALNT1	-1.3214	3.89875	-7.0501	0.00041	0.00325	-0.1319
ARL4D	-1.3188	3.3542	-6.0132	0.00096	0.00578	-1.0958
SLC7A2	-1.3186	3.27327	-8.1191	0.00019	0.00198	0.74915
MAPK10	-1.3158	-0.5014	-5.9042	0.00105	0.00619	-1.2042
MCOLN3	-1.314	1.34648	-13.328	1.11E-05	0.00035	3.94143
TMEM44	-1.3138	2.4318	-13.805	9.03E-06	0.0003	4.16966
SLC9A5	-1.3136	-0.6194	-5.5869	0.0014	0.00761	-1.5284
HOXA5	-1.3118	1.7972	-6.5399	0.00061	0.00424	-0.5914
GTF2H2C	-1.311	-1.4484	-4.4164	0.00449	0.01719	-2.8408
ASB2	-1.3071	-3.4539	-3.3099	0.01622	0.04484	-4.2625
KIAA1614	-1.306	0.06185	-8.6645	0.00013	0.00158	1.16103
CROT	-1.3017	2.3037	-6.3303	0.00073	0.00478	-0.7883
TLR3	-1.3013	-0.0136	-7.2752	0.00034	0.00287	0.06244
ST3GAL4	-1.3001	3.67921	-6.574	0.0006	0.00417	-0.5598
ZNF284	-1.2984	0.17615	-6.9804	0.00043	0.00335	-0.1931

NKX2-8	-1.2972	0.54193	-7.2418	0.00035	0.00293	0.03399
TGFBI	-1.2968	4.41852	-3.2466	0.01756	0.04756	-4.3488
LRRC63	-1.2947	-2.4677	-4.0123	0.00703	0.02403	-3.3396
ENTPD3-	-1.2938	-1.7835	-5.8778	0.00108	0.0063	-1.2307
PCOLCE2	-1.292	2.86639	-6.4082	0.00068	0.00456	-0.7145
SH3TC1	-1.2903	1.00417	-6.075	0.00091	0.00556	-1.035
SEMA6C	-1.2888	2.0318	-9.2407	9.10E-05	0.00125	1.57195
NAGLU	-1.2875	2.88536	-11.126	3.15E-05	0.00065	2.76907
KANSL1-A	-1.2856	1.16642	-9.5797	7.42E-05	0.0011	1.80306
TMED2-D	-1.2854	0.47293	-6.7208	0.00053	0.00385	-0.4253
INPP1	-1.2831	2.68799	-12.378	1.70E-05	0.00045	3.46099
CFAP20D	-1.2784	0.9314	-6.8305	0.00048	0.00363	-0.3263
ZNF567-D	-1.2763	0.78789	-4.8174	0.00295	0.01279	-2.3695
PIK3R3	-1.2744	3.96186	-7.2757	0.00034	0.00287	0.06294
BCYRN1	-1.2712	2.64825	-6.4563	0.00066	0.00444	-0.6694
RBM11	-1.2678	-1.1862	-4.46	0.00429	0.01664	-2.7884
ISL1	-1.2677	0.08132	-3.8194	0.00878	0.02825	-3.5862
PDCD4-A	-1.2651	-0.0301	-4.8372	0.00289	0.01258	-2.3469
IL6R	-1.2647	2.87176	-11.378	2.77E-05	0.0006	2.91384
TRANK1	-1.2632	-0.2983	-6.7392	0.00052	0.00382	-0.4086
TTLL1	-1.2619	-0.5774	-5.0226	0.0024	0.01098	-2.1372
SARNP	-1.2613	-2.4967	-4.0865	0.00646	0.02263	-3.2462
PHLDA2	-1.2611	3.38714	-5.5603	0.00144	0.00774	-1.5562
GPX3	-1.2604	7.86441	-4.2288	0.00551	0.02	-3.0695
SAMD9	-1.2603	1.626	-4.484	0.00418	0.01634	-2.7597
RIN1	-1.26	2.04015	-7.6021	0.00027	0.0025	0.33601
ZBTB44-D	-1.2594	-2.5238	-5.5464	0.00145	0.0078	-1.5706
C9orf43	-1.2589	-1.7111	-3.7459	0.00957	0.03004	-3.6815
CCDC17	-1.2581	-0.8238	-6.2875	0.00076	0.00489	-0.8291
GDPD5	-1.2578	5.07643	-3.7692	0.00931	0.02947	-3.6513
CFAP74	-1.2559	-3.0885	-3.8357	0.00861	0.02785	-3.5652
SCN8A	-1.2555	-0.7607	-5.1136	0.0022	0.0103	-2.0362
FBLN1	-1.2552	6.04467	-8.6258	0.00013	0.0016	1.13263
GMPR	-1.2543	2.45125	-8.2549	0.00017	0.00188	0.85392
DACT3	-1.2538	0.61902	-11.701	2.36E-05	0.00055	3.09582
SYT3	-1.2537	-0.4259	-7.5666	0.00028	0.00254	0.30682
CCDC137	-1.2532	0.73624	-6.2602	0.00077	0.00498	-0.8553
HLA-F-AS	-1.252	-1.7349	-8.6436	0.00013	0.00159	1.14573
TMOD1	-1.2499	2.49393	-9.3798	8.36E-05	0.00118	1.66768
DNAJC27	-1.2487	-2.6354	-3.4967	0.01289	0.03781	-4.0105
COLQ	-1.2474	-2.5575	-5.851	0.0011	0.00641	-1.2577
OSGIN2	-1.2431	5.31222	-12.985	1.29E-05	0.00038	3.77182
ALDH2	-1.2422	4.39446	-8.4344	0.00015	0.00175	0.99015
HMGN5	-1.2412	2.01331	-8.0363	0.0002	0.00206	0.68453
OSBPL7	-1.2406	2.59208	-9.8859	6.21E-05	0.00098	2.00538
ASIC1	-1.2388	2.13242	-4.3925	0.00461	0.01751	-2.8697
GLYCTK	-1.2376	1.32486	-6.5422	0.00061	0.00424	-0.5892
PIK3R1	-1.2363	3.88681	-5.5465	0.00145	0.0078	-1.5705
PLEKHB1	-1.2359	0.33961	-8.2363	0.00017	0.00189	0.83963
STAT1	-1.2356	5.29868	-5.857	0.0011	0.00638	-1.2517
SLC12A5	-1.2351	-0.3004	-5.3538	0.00174	0.00877	-1.7747
GPSM1	-1.233	2.38459	-13.29	1.13E-05	0.00035	3.92259
LINC0261	-1.2328	0.39393	-6.527	0.00062	0.00427	-0.6034
CD44	-1.2325	4.8721	-5.1638	0.00209	0.00997	-1.9809
PDGFRB	-1.2321	-2.1217	-4.3966	0.00459	0.01746	-2.8647
BNIP3P1	-1.2307	5.01562	-7.1883	0.00037	0.00301	-0.0119
A4GALT	-1.2302	3.37956	-4.6084	0.00366	0.0149	-2.6122
PAXIP1-A	-1.2277	-1.8348	-3.7833	0.00916	0.02914	-3.633

ZNF234	-1.227	2.02391	-12.172	1.88E-05	0.00048	3.35205
MR1	-1.227	2.9465	-13.88	8.75E-06	0.00029	4.20458
MTA1-DT	-1.2242	1.67195	-3.5561	0.012	0.03573	-3.9313
LINC0108	-1.2242	1.77299	-5.7193	0.00124	0.00699	-1.3916
WASF1	-1.2212	2.95797	-11.985	2.05E-05	0.00051	3.25142
IKBIP	-1.2205	3.9705	-10.662	4.03E-05	0.00076	2.49295
DRAXIN	-1.2196	-1.303	-6.8726	0.00047	0.00354	-0.2887
CYCSP6	-1.2174	-1.4759	-3.282	0.0168	0.04595	-4.3006
DLK2	-1.2164	-0.6897	-5.3652	0.00172	0.00872	-1.7625
GNAL	-1.2145	-0.0271	-9.4064	8.23E-05	0.00117	1.68587
CASC8	-1.2115	1.76699	-5.2359	0.00195	0.00949	-1.9021
HMX1	-1.2065	3.49721	-8.299	0.00017	0.00184	0.8876
MOCS2-D	-1.2043	0.97006	-8.4171	0.00015	0.00176	0.9771
BTD	-1.2037	0.77985	-6.5	0.00063	0.00432	-0.6285
TNFSF10	-1.2027	2.66282	-3.223	0.01808	0.04862	-4.3812
GRAMD1	-1.2025	0.25673	-3.7844	0.00914	0.02911	-3.6315
VASH1	-1.2015	0.10889	-5.9496	0.00101	0.006	-1.1589
SPAG4	-1.1995	1.34695	-3.4467	0.0137	0.03963	-4.0775
DZIP1L	-1.1992	0.86942	-5.6044	0.00138	0.00755	-1.5101
CLDN16	-1.1989	3.84753	-4.7404	0.0032	0.01348	-2.4582
LRRC4B	-1.1985	0.25136	-3.3797	0.01488	0.04201	-4.1678
GBX2	-1.1977	-0.0519	-6.1864	0.00082	0.00521	-0.9263
SEPTIN5	-1.197	1.15784	-9.414	8.19E-05	0.00116	1.69103
SLC15A1	-1.1963	-2.5054	-3.3404	0.01562	0.04357	-4.2211
SRD5A3	-1.1955	2.84649	-7.5359	0.00028	0.00257	0.28146
CHRD	-1.1924	1.48278	-4.3653	0.00475	0.01791	-2.9026
TNFRSF1	-1.1903	-4.1054	-3.954	0.00751	0.02523	-3.4136
HOXC6	-1.1889	0.10391	-8.9629	0.00011	0.0014	1.37683
COA3	-1.1871	5.56553	-12.345	1.73E-05	0.00046	3.44358
TMEM108	-1.1864	-0.2016	-11.049	3.28E-05	0.00067	2.72419
C20orf20	-1.185	-0.6606	-3.2	0.01861	0.04966	-4.4127
GRK3	-1.1846	1.81734	-11.855	2.19E-05	0.00053	3.18045
CCDC15	-1.1823	0.40579	-5.3859	0.00169	0.00862	-1.7404
CAPN14	-1.1823	-2.3373	-5.344	0.00176	0.00884	-1.7852
HOXA6	-1.182	-0.6454	-4.1681	0.0059	0.02107	-3.1445
ZEB1-AS1	-1.1787	0.08543	-6.9872	0.00043	0.00334	-0.1871
IFTAP	-1.1781	-0.0391	-8.2909	0.00017	0.00184	0.88148
TGFB3	-1.1772	0.70971	-3.7709	0.00929	0.02942	-3.6491
IL11RA	-1.1761	0.65819	-7.3652	0.00032	0.00275	0.13879
PFKM	-1.1747	5.14579	-13.154	1.20E-05	0.00036	3.85585
SLC9A3-A	-1.1728	2.25273	-6.9169	0.00045	0.00347	-0.2492
SHC1P2	-1.1726	0.87456	-5.2253	0.00197	0.00955	-1.9135
LINC0094	-1.1721	1.36351	-5.9684	0.00099	0.00593	-1.1402
CEACAM	-1.172	-0.9099	-4.7577	0.00314	0.01332	-2.4382
AARSD1	-1.1709	-1.2872	-6.4843	0.00064	0.00436	-0.6432
TTN-AS1	-1.1692	0.72716	-11.437	2.69E-05	0.00059	2.9475
USP44	-1.1692	1.39567	-5.4239	0.00163	0.00841	-1.6999
GAL3ST4	-1.169	-2.3243	-5.0137	0.00242	0.01103	-2.1472
PLOD2	-1.1688	4.26195	-11.201	3.03E-05	0.00064	2.81263
KCNMA1	-1.1674	0.36423	-5.0783	0.00227	0.01056	-2.0753
LPP-AS2	-1.1673	1.84031	-5.0441	0.00235	0.01082	-2.1132
COL12A1	-1.1671	5.81158	-3.873	0.00825	0.02697	-3.5171
SRP54-AS	-1.1652	-2.5131	-4.1143	0.00626	0.0221	-3.2115
SNX22	-1.1648	-1.5177	-5.3385	0.00177	0.00887	-1.7912
ZNF155	-1.1635	1.03517	-6.96	0.00044	0.00339	-0.211
SULF1	-1.162	-1.6114	-3.6165	0.01116	0.03382	-3.8513
TFCP2L1	-1.1618	-0.4631	-5.7665	0.00119	0.00676	-1.3433
C2orf72	-1.1616	-0.0163	-5.6913	0.00127	0.00712	-1.4203

JUN	-1.1611	6.5585	-6.068	0.00091	0.00558	-1.0418
HOXB-AS	-1.1611	0.6366	-4.8602	0.00283	0.01238	-2.3206
SH3TC2	-1.1605	0.64477	-3.6947	0.01016	0.03145	-3.7484
UBE2L6	-1.1591	3.79901	-6.7838	0.0005	0.00372	-0.3683
ANKRD44	-1.1591	1.69842	-4.4517	0.00433	0.01674	-2.7984
COQ10A	-1.158	2.59809	-9.3686	8.42E-05	0.00118	1.66007
TMEM218	-1.1569	2.24785	-12.205	1.85E-05	0.00048	3.36945
ZNF542P	-1.1552	1.08509	-7.0773	0.0004	0.0032	-0.1081
DIPK1B	-1.152	3.98343	-7.4273	0.00031	0.00269	0.19098
SLC15A2	-1.1518	-2.1803	-8.3287	0.00016	0.00182	0.91025
SORD2P	-1.1484	-2.6279	-7.5892	0.00027	0.00251	0.32541
CHST2	-1.1441	3.83229	-3.9749	0.00733	0.02479	-3.3871
SP5	-1.1439	0.21315	-5.1694	0.00208	0.00993	-1.9747
PDE6A	-1.1435	-0.8512	-4.2059	0.00566	0.02038	-3.0977
CRYGS	-1.1413	-1.4057	-3.9245	0.00777	0.02583	-3.4512
LHX2	-1.1394	-0.4405	-8.0629	0.0002	0.00204	0.70535
PITPNM2	-1.1392	3.04914	-4.2518	0.00538	0.01965	-3.0412
IDH1-AS1	-1.1387	0.66933	-3.5369	0.01228	0.03636	-3.9569
MOXD1	-1.1385	-1.0202	-5.9087	0.00105	0.00617	-1.1997
HIVEP3	-1.138	2.18641	-5.2894	0.00185	0.00917	-1.844
ARSG	-1.1372	0.43856	-7.3695	0.00032	0.00274	0.14245
TIMP2	-1.1369	6.85846	-13.669	9.57E-06	0.00031	4.1053
ZNF702P	-1.1363	0.46558	-4.3284	0.00494	0.01845	-2.9474
ZNF569	-1.1358	1.2594	-11.271	2.93E-05	0.00062	2.85298
CCDC88B	-1.1352	0.15749	-5.3038	0.00183	0.00907	-1.8285
AQP11	-1.129	-0.7593	-5.4346	0.00161	0.00837	-1.6885
RSAD1	-1.1276	4.16055	-9.1364	9.70E-05	0.0013	1.49931
NUDT14	-1.1271	2.96279	-6.1999	0.00081	0.00517	-0.9133
ZNF235	-1.1268	0.43075	-8.1803	0.00018	0.00194	0.79656
SYNPO	-1.125	6.01483	-5.017	0.00242	0.01101	-2.1435
THAP7-A	-1.1247	0.23555	-8.5716	0.00014	0.00164	1.09256
ZNF420	-1.124	1.05634	-10.769	3.81E-05	0.00073	2.55768
STRADA	-1.1238	-0.7108	-3.2199	0.01816	0.04876	-4.3855
PDCD6IP	-1.1208	-0.7616	-3.6838	0.0103	0.03179	-3.7628
PRTG	-1.1206	1.44422	-6.736	0.00052	0.00383	-0.4115
KCNIP2	-1.1184	-1.2135	-4.0114	0.00704	0.02404	-3.3408
FMNL2	-1.1169	5.6408	-11.726	2.33E-05	0.00055	3.1098
MIR130AI	-1.1167	-0.4519	-5.5878	0.0014	0.00761	-1.5274
TNS2	-1.1149	3.99478	-4.4034	0.00456	0.01736	-2.8565
NOS1AP	-1.1137	-0.0143	-5.7865	0.00117	0.00667	-1.323
GFRA3	-1.1133	-1.6989	-6.2491	0.00078	0.00501	-0.8659
LINC0091	-1.1133	-0.3537	-4.0092	0.00705	0.02409	-3.3436
ACOT1	-1.1113	0.59092	-8.5385	0.00014	0.00167	1.06799
HYPK	-1.1102	-1.8759	-3.2005	0.0186	0.04965	-4.4121
RAI2	-1.1101	3.39919	-5.9661	0.001	0.00593	-1.1424
EGR1	-1.1099	6.54992	-3.2308	0.01791	0.04828	-4.3705
SULT1A1	-1.1096	2.85648	-7.8278	0.00023	0.00225	0.51923
TCF12	-1.1082	5.12411	-7.205	0.00036	0.00298	0.00242
TMEM121	-1.1061	-1.6326	-3.4892	0.01301	0.03807	-4.0205
SLC46A3	-1.1058	-1.7396	-4.0535	0.00671	0.02324	-3.2877
FBF1	-1.1041	0.6586	-11.65	2.42E-05	0.00056	3.06763
GDF11	-1.1024	4.45795	-4.6494	0.00351	0.01446	-2.5641
ADAP2	-1.1018	-0.3791	-3.4423	0.01377	0.03977	-4.0834
PRR7	-1.1006	3.57763	-14.186	7.70E-06	0.00027	4.34633
COG1	-1.1006	3.6095	-14.482	6.83E-06	0.00026	4.47993
ANXA4	-1.0991	6.53214	-11.307	2.88E-05	0.00061	2.87351
RPL39L	-1.0975	3.04282	-10.847	3.65E-05	0.00071	2.60466
PLEKHA7	-1.0941	2.36152	-7.9241	0.00022	0.00215	0.59608

BNC2	-1.0938	4.01659	-12.245	1.81E-05	0.00047	3.39055
RBPMS	-1.0933	3.91697	-4.0498	0.00674	0.0233	-3.2924
C3orf18	-1.0929	-0.1028	-6.6226	0.00057	0.00406	-0.5151
MBLAC1	-1.0919	2.32539	-4.4632	0.00427	0.01661	-2.7846
TRNP1	-1.0909	5.42367	-6.0463	0.00093	0.00566	-1.0632
TECPR1	-1.0905	3.29866	-9.4408	8.06E-05	0.00116	1.70927
CRLF1	-1.0888	1.0888	-4.8932	0.00273	0.01207	-2.2831
RNFT1	-1.0886	0.7374	-11.159	3.10E-05	0.00065	2.78794
FTSJ3	-1.0879	5.1328	-6.7692	0.00051	0.00375	-0.3814
SELENOI	-1.0871	3.85788	-7.7159	0.00025	0.00237	0.42896
CA2	-1.087	3.32927	-3.3927	0.01464	0.04151	-4.1502
GPR158	-1.0865	1.96184	-7.3169	0.00033	0.00281	0.09795
ADGRB2	-1.085	2.60545	-7.5944	0.00027	0.0025	0.32976
CCDC137	-1.0831	4.86565	-8.0047	0.0002	0.00208	0.65973
GIHCG	-1.083	2.45484	-5.7108	0.00125	0.00703	-1.4003
DYSF	-1.082	4.01698	-5.2858	0.00186	0.00918	-1.8479
GPER1	-1.0808	-1.1632	-4.6297	0.00358	0.01466	-2.5873
MGST1	-1.0805	4.92188	-9.2933	8.81E-05	0.00123	1.60833
TBX2	-1.0803	0.43875	-5.238	0.00195	0.00948	-1.8998
ATXN2-A	-1.0791	-0.1886	-4.5615	0.00385	0.01541	-2.6676
EDA	-1.0779	1.2824	-3.207	0.01845	0.04938	-4.4031
LRP4	-1.0765	0.01354	-4.8595	0.00283	0.01239	-2.3214
EIF1P5	-1.0764	1.46288	-3.9025	0.00797	0.02628	-3.4793
ABCB6	-1.0764	0.25835	-9.52	7.69E-05	0.00112	1.76288
MCRIP1	-1.075	3.83838	-6.1927	0.00082	0.0052	-0.9203
TUBA8	-1.0733	-0.3138	-3.9359	0.00767	0.02562	-3.4366
PHETA1	-1.0722	3.56843	-8.9267	0.00011	0.00142	1.35096
ZNF697	-1.0717	1.64801	-5.4006	0.00167	0.00853	-1.7247
IFIT1	-1.0674	1.84069	-3.4811	0.01314	0.03839	-4.0314
FBXO10	-1.0668	-0.1447	-3.6776	0.01037	0.03198	-3.7709
PASD1	-1.0667	3.05907	-6.0353	0.00094	0.0057	-1.074
GPR135	-1.0665	0.95973	-9.7024	6.90E-05	0.00105	1.8848
TRAF5	-1.0657	1.1091	-7.7095	0.00025	0.00238	0.42383
ANKHD1-	-1.0644	-2.2876	-5.579	0.00141	0.00765	-1.5366
HHAT	-1.0641	-0.0155	-3.6998	0.0101	0.03132	-3.7418
ZNF222	-1.0623	0.74102	-8.8145	0.00012	0.00149	1.27032
ITGA6	-1.0621	3.22131	-5.1248	0.00217	0.01024	-2.0237
B3GALT9	-1.0617	-0.54	-3.9333	0.00769	0.02567	-3.4399
IFI16	-1.0602	3.85355	-8.7497	0.00012	0.00153	1.22332
OSTM1	-1.057	3.66871	-8.9497	0.00011	0.0014	1.36739
ASIC3	-1.0564	-1.7191	-4.8778	0.00278	0.01221	-2.3005
ZNRF2	-1.0544	3.32405	-7.27	0.00035	0.00288	0.05809
MIR3677H	-1.0534	-1.5916	-13.66	9.60E-06	0.00031	4.10104
STXBP4	-1.0531	1.53095	-5.3775	0.0017	0.00866	-1.7493
MALINC1	-1.053	-0.5908	-3.2777	0.01688	0.0461	-4.3063
GPC4	-1.0509	5.45067	-3.2102	0.01838	0.04925	-4.3988
CBFA2T2	-1.0504	5.10728	-6.7486	0.00052	0.0038	-0.4001
DSE	-1.0492	0.9002	-5.0565	0.00232	0.01072	-2.0994
NMNAT3	-1.0485	0.13083	-7.5016	0.00029	0.00261	0.25304
GSAP	-1.0484	-1.0173	-9.1882	9.40E-05	0.00127	1.53554
FAM241A	-1.0477	1.61352	-6.4929	0.00064	0.00434	-0.6351
LRMDA	-1.047	3.42952	-6.6165	0.00058	0.00406	-0.5206
SLFN12	-1.0458	1.95866	-3.9326	0.0077	0.02568	-3.4409
PLEKHM1	-1.0443	2.55173	-7.2473	0.00035	0.00292	0.03869
TMEM63C	-1.0438	2.82259	-6.3336	0.00073	0.00477	-0.7852
CDKN1C	-1.0427	1.81297	-4.7961	0.00302	0.01297	-2.394
ZNF396	-1.0427	-2.7277	-3.3652	0.01515	0.04258	-4.1875
NT5C3B	-1.0395	3.20549	-7.5304	0.00028	0.00257	0.27688

ZNF227	-1.0394	2.50036	-6.6775	0.00055	0.00394	-0.4648
BLVRB	-1.0393	5.40539	-4.3103	0.00504	0.01872	-2.9695
ADORA2E	-1.0373	0.9254	-8.9978	0.00011	0.00138	1.40163
VCAN	-1.0372	6.43985	-5.787	0.00117	0.00667	-1.3225
CD177	-1.0364	-2.6655	-5.7056	0.00126	0.00706	-1.4056
SLC25A2	-1.0347	1.61119	-10.26	5.02E-05	0.00087	2.24487
SH3RF2	-1.0345	3.86489	-6.7305	0.00052	0.00383	-0.4165
SLC10A4	-1.0337	-0.4606	-4.3156	0.00501	0.01864	-2.963
HMOX2	-1.0333	4.2287	-6.2568	0.00077	0.00498	-0.8585
BRSK1	-1.0333	2.33616	-4.6815	0.0034	0.01409	-2.5267
JAZF1	-1.0332	4.24727	-13.638	9.70E-06	0.00032	4.09045
FAM104A	-1.0305	3.584	-13.36	1.09E-05	0.00034	3.95693
NRROS	-1.0303	-0.2797	-9.074	0.0001	0.00134	1.4555
INPP4B	-1.0292	3.61451	-5.4406	0.0016	0.00835	-1.6822
HIGD1A	-1.0229	4.47925	-7.9908	0.00021	0.0021	0.64881
CRADD	-1.0229	0.37258	-9.1136	9.84E-05	0.00131	1.48334
KLHL3	-1.0224	0.20468	-8.206	0.00018	0.00191	0.81634
OXLD1	-1.0221	4.83295	-4.8567	0.00284	0.01241	-2.3245
LINC0257	-1.022	1.52231	-7.3701	0.00032	0.00274	0.1429
COLEC12	-1.0215	0.54904	-3.4369	0.01387	0.03996	-4.0907
EXTL3-AS	-1.0202	-1.4692	-7.7714	0.00024	0.0023	0.47392
PDZK1IP1	-1.0194	1.71415	-3.2108	0.01836	0.04923	-4.3979
ARMCX1	-1.0191	4.04332	-7.0227	0.00042	0.00329	-0.1558
MDH1B	-1.019	-2.5748	-3.38	0.01487	0.042	-4.1674
TTC21B-A	-1.0189	-6.3443	-5.3283	0.00178	0.00893	-1.8021
ZNF45	-1.0186	2.34159	-6.1335	0.00086	0.00539	-0.9778
STYK1	-1.018	0.65531	-3.98	0.00729	0.02468	-3.3806
PPP1R9B	-1.0175	4.65396	-10.511	4.37E-05	0.0008	2.40093
INTS2	-1.0168	1.4708	-8.6683	0.00013	0.00158	1.16384
MHENCR	-1.0163	3.0813	-3.8385	0.00858	0.0278	-3.5615
CASP4	-1.016	2.8178	-9.1217	9.79E-05	0.00131	1.48907
AJM1	-1.0138	1.21028	-4.0258	0.00692	0.02377	-3.3226
HTRA1	-1.0134	2.42748	-5.0898	0.00225	0.01047	-2.0625
STK39	-1.0126	2.65199	-7.3415	0.00033	0.00277	0.11874
PRKCA	-1.012	3.5875	-10.06	5.62E-05	0.00093	2.1181
C2orf74-D	-1.0097	2.03077	-7.3139	0.00033	0.00281	0.09538
LINC0269	-1.0094	0.879	-3.4419	0.01378	0.03977	-4.084
PLCXD2	-1.0085	-1.4619	-5.4731	0.00156	0.00815	-1.6477
HMGCR	-1.0078	6.4851	-10.867	3.61E-05	0.00071	2.61619
ARSJ	-1.0077	1.69207	-3.5643	0.01188	0.03545	-3.9205
ARL4C	-1.0074	5.03225	-10.727	3.89E-05	0.00074	2.53237
DGCR6L	-1.0044	4.05489	-7.3306	0.00033	0.00279	0.10954
BICD1	-1.0034	4.06504	-7.9573	0.00021	0.00212	0.62232
ATP5MC1	-1.0031	1.34317	-7.2301	0.00036	0.00294	0.02398
SLC35E3	-1.0028	2.63939	-6.7118	0.00053	0.00386	-0.4335
NRP1	-1.0027	4.90125	-3.8941	0.00805	0.02648	-3.4901
ASPHD2	-1.0017	-0.3706	-4.2453	0.00542	0.01975	-3.0491
METTL7B	-1.0005	1.44425	-8.0666	0.00019	0.00204	0.70824
LINC0245	-1	-0.9835	-4.7174	0.00327	0.01374	-2.4848
SETX	-0.9978	4.15968	-9.0317	0.0001	0.00136	1.42563
DPY19L1	-0.9975	3.58636	-8.3802	0.00016	0.00178	0.94931
PLCB2	-0.9951	-1.4367	-6.3972	0.00069	0.00458	-0.7249
PRKACB	-0.9946	3.77467	-4.9896	0.00248	0.01123	-2.1743
HCFC2	-0.99	2.09693	-12.015	2.02E-05	0.00051	3.26776
PLD1	-0.9871	1.94636	-9.5438	7.58E-05	0.00111	1.77891
FGGY	-0.987	1.88877	-11.727	2.33E-05	0.00055	3.11008
PAQR6	-0.9869	1.28738	-3.6089	0.01126	0.03407	-3.8613
LMF1	-0.9867	0.81795	-5.1407	0.00214	0.01015	-2.0063

BAZ2B-AS	-0.9852	0.84676	-8.982	0.00011	0.00139	1.39042
BCL2L13	-0.9849	4.80183	-9.5236	7.67E-05	0.00112	1.76529
INTS6L	-0.9849	1.94749	-4.749	0.00317	0.01338	-2.4483
ZNF226	-0.9846	2.81501	-7.0407	0.00041	0.00326	-0.14
LINC0046	-0.9843	0.26578	-7.6416	0.00026	0.00244	0.36847
EIF4E3	-0.9841	1.63279	-6.3081	0.00074	0.00484	-0.8095
MGST2	-0.984	3.77758	-12.013	2.03E-05	0.00051	3.26682
CHKB-DT	-0.9825	1.80881	-9.0535	0.0001	0.00135	1.44108
TMEM144	-0.9817	0.87636	-6.5605	0.0006	0.00419	-0.5723
MILIP	-0.9803	2.37266	-7.9854	0.00021	0.0021	0.64452
NDRG2	-0.9796	1.8309	-8.436	0.00015	0.00174	0.99133
PRR7-AS	-0.9782	0.49255	-3.8118	0.00885	0.02843	-3.5961
WDR86-A	-0.978	1.80251	-7.0018	0.00042	0.00333	-0.1742
FRMD4B	-0.9774	2.48514	-5.2613	0.0019	0.00935	-1.8745
EXOC7	-0.9756	5.08644	-10.003	5.80E-05	0.00095	2.08091
AGA	-0.9755	1.96337	-7.8683	0.00022	0.00221	0.55166
SHC1	-0.9746	6.33478	-7.472	0.0003	0.00264	0.22839
DPM3	-0.9733	5.28189	-5.1915	0.00204	0.00979	-1.9505
PIGHP1	-0.973	1.52095	-7.3847	0.00032	0.00273	0.15526
TK2	-0.9724	1.63604	-5.3195	0.0018	0.00898	-1.8115
GABARAI	-0.9723	2.83428	-7.0404	0.00041	0.00326	-0.1403
HOMER3	-0.9722	3.58895	-4.9356	0.00262	0.01168	-2.235
EHHADH	-0.9714	2.18973	-6.7999	0.0005	0.00369	-0.3538
IL10RB	-0.9698	1.95589	-7.6454	0.00026	0.00244	0.37156
COPZ2	-0.9695	0.40903	-8.4438	0.00015	0.00174	0.99716
PCSK1N	-0.9694	3.40612	-8.5573	0.00014	0.00165	1.08191
CDHR1	-0.969	2.0624	-4.9083	0.00269	0.01193	-2.2658
PSMB3P1	-0.9689	2.44144	-3.5399	0.01223	0.03627	-3.9529
CARD6	-0.9682	2.04208	-10.107	5.47E-05	0.00092	2.14804
EME2	-0.9674	2.20037	-8.1764	0.00018	0.00194	0.79358
RAB5CP2	-0.9669	-0.3084	-4.277	0.00523	0.01924	-3.0102
ZFHX2	-0.9667	-0.2369	-3.8769	0.00821	0.02687	-3.5121
SLC26A1	-0.9657	-3.6215	-3.4204	0.01415	0.04054	-4.1129
NF2	-0.9649	5.8246	-5.013	0.00242	0.01103	-2.1479
TEX9	-0.9648	-0.3352	-5.7865	0.00117	0.00667	-1.323
SNCB	-0.9642	-0.9464	-3.2483	0.01752	0.04749	-4.3465
PLD6	-0.9634	1.23316	-5.3804	0.0017	0.00865	-1.7462
GAMT	-0.9627	2.3712	-12.898	1.34E-05	0.00039	3.72808
CRYZL2F	-0.9625	2.37213	-4.7609	0.00313	0.01329	-2.4345
G6PC3	-0.9621	4.21811	-10.08	5.56E-05	0.00093	2.13036
DNAH17-	-0.9617	0.06248	-5.7665	0.00119	0.00676	-1.3434
SLC38A1	-0.9604	4.81214	-9.4049	8.24E-05	0.00117	1.68486
DENND5I	-0.9604	4.12505	-10.53	4.33E-05	0.00079	2.41274
SFXN5	-0.96	2.87524	-4.2445	0.00542	0.01976	-3.05
KRT8P12	-0.9583	2.71927	-4.5574	0.00387	0.01544	-2.6725
GPR27	-0.9577	3.56343	-6.31	0.00074	0.00483	-0.8077
IL17RA	-0.9566	3.30246	-8.7007	0.00013	0.00156	1.18759
NRIP3	-0.9564	1.16365	-5.2873	0.00186	0.00917	-1.8463
CD302	-0.9564	2.65725	-4.5099	0.00407	0.016	-2.7289
BBS4	-0.9563	1.5116	-8.7457	0.00012	0.00153	1.22042
CCDC88A	-0.9523	4.62925	-10.889	3.57E-05	0.00071	2.62955
PHYHD1	-0.9513	0.14986	-4.4794	0.0042	0.01639	-2.7653
HSD17B1	-0.9513	2.68566	-6.6036	0.00058	0.00409	-0.5325
ARHGEF1	-0.9506	-0.6078	-4.6318	0.00358	0.01465	-2.5848
CBLL1-AS	-0.9503	1.46606	-3.559	0.01195	0.03564	-3.9274
PPM1M	-0.9499	2.63354	-3.9034	0.00796	0.02627	-3.4782
NKIRAS2	-0.9487	4.70052	-10.946	3.47E-05	0.00069	2.66328
RTL6	-0.9482	4.33954	-5.069	0.00229	0.01061	-2.0856

LINC0260	-0.947	1.11885	-6.6181	0.00057	0.00406	-0.5192
DAPK1	-0.9462	3.75326	-7.9647	0.00021	0.00212	0.62819
SLC35G2	-0.9452	2.23188	-7.4584	0.0003	0.00266	0.21698
EVA1B	-0.9447	3.06968	-7.6432	0.00026	0.00244	0.36974
ARMCX4	-0.9443	2.42409	-8.7725	0.00012	0.00151	1.23992
NBR2	-0.9437	-0.6178	-4.9167	0.00267	0.01187	-2.2564
TMEM104	-0.9429	2.82185	-5.2009	0.00202	0.00972	-1.9402
B3GNTL1	-0.9421	2.49627	-5.4755	0.00155	0.00814	-1.6452
INPP5F	-0.9419	2.51143	-11.653	2.42E-05	0.00056	3.06931
ABHD4	-0.9409	2.58139	-8.9963	0.00011	0.00138	1.40056
ZNF225	-0.9405	0.83565	-7.228	0.00036	0.00294	0.02211
HDAC10	-0.9391	-0.6127	-5.3253	0.00179	0.00894	-1.8054
ALPK1	-0.9387	1.82568	-3.8523	0.00845	0.02747	-3.5437
ANK2	-0.9384	2.20183	-3.5372	0.01227	0.03636	-3.9564
HTATIP2	-0.9355	3.72582	-9.4464	8.03E-05	0.00115	1.71306
ZNF285	-0.9355	0.30236	-7.3455	0.00033	0.00277	0.12216
HDHD5	-0.9352	3.78767	-5.9475	0.00101	0.00601	-1.161
ATP5MC1	-0.935	-0.2087	-3.4026	0.01446	0.04118	-4.1368
GALK1	-0.9341	2.68637	-7.7243	0.00025	0.00236	0.43579
ZNF850	-0.9332	-1.2423	-5.129	0.00216	0.01022	-2.0191
PRRX2	-0.9324	2.21036	-4.085	0.00647	0.02266	-3.2481
GSTT2B	-0.9323	0.15596	-6.1193	0.00087	0.00544	-0.9916
NME4	-0.932	4.22913	-5.5682	0.00142	0.0077	-1.5479
ZNF225-A	-0.9314	-0.0843	-4.528	0.00399	0.01582	-2.7073
SH3RF3	-0.9307	2.48113	-5.822	0.00113	0.00654	-1.287
NUDT17	-0.9305	1.35266	-5.6908	0.00127	0.00712	-1.4208
ZNF112	-0.9296	0.3456	-9.5969	7.35E-05	0.00109	1.81459
CCDC110	-0.9294	-1.6829	-4.7148	0.00328	0.01377	-2.4879
TXNRD2	-0.9283	3.55324	-5.4094	0.00165	0.00849	-1.7153
DPP3-DT	-0.928	0.42931	-7.3363	0.00033	0.00278	0.11441
SNCA	-0.928	2.57215	-9.5766	7.43E-05	0.0011	1.80097
THRA	-0.9274	2.60505	-8.2698	0.00017	0.00186	0.86536
SLC38A6	-0.927	2.02713	-6.2401	0.00079	0.00504	-0.8746
COX20P1	-0.9265	4.13101	-4.3361	0.0049	0.01833	-2.938
NEK6	-0.926	3.83158	-4.2767	0.00523	0.01924	-3.0105
PHKA1	-0.9245	0.16285	-6.4869	0.00064	0.00436	-0.6408
CHD1L	-0.9234	4.12387	-9.2352	9.13E-05	0.00125	1.56816
KLHL22	-0.9222	2.61837	-9.0725	0.0001	0.00134	1.45446
CYB5D2	-0.9217	0.88769	-4.5917	0.00373	0.01509	-2.6319
FAM166C	-0.9201	0.1397	-4.2286	0.00552	0.02	-3.0697
ZNF85	-0.9199	-0.0927	-4.3593	0.00478	0.01798	-2.9099
PIK3CG	-0.9199	1.76778	-6.9674	0.00044	0.00337	-0.2045
MRPL10	-0.9194	3.14294	-8.3964	0.00016	0.00178	0.96154
MKS1	-0.9185	2.05453	-8.9914	0.00011	0.00138	1.39708
STARD3	-0.9182	3.10782	-11.128	3.15E-05	0.00065	2.76986
SNF8	-0.9176	4.17707	-9.3766	8.38E-05	0.00118	1.66549
TTLL12	-0.917	5.88605	-5.9855	0.00098	0.00586	-1.1232
FXYD6	-0.9167	1.50649	-5.495	0.00152	0.00804	-1.6246
MFSD11	-0.9166	2.83095	-6.1373	0.00086	0.00538	-0.974
ZNFX1	-0.9165	4.602	-6.1569	0.00084	0.00532	-0.955
SIRT2	-0.9155	4.48797	-10.162	5.30E-05	0.0009	2.18318
ZNF529	-0.9147	1.91125	-6.7807	0.0005	0.00372	-0.3711
PCDHB12	-0.9141	-0.0807	-5.8039	0.00115	0.00661	-1.3053
DOCK4	-0.9133	4.4662	-3.3503	0.01543	0.04316	-4.2076
CEP290	-0.9131	3.63786	-8.8508	0.00012	0.00147	1.29656
CCDC47	-0.9127	4.88294	-6.9228	0.00045	0.00345	-0.244
MXD4	-0.9127	3.45105	-4.0498	0.00674	0.0233	-3.2924
CPQ	-0.9119	2.65108	-5.3429	0.00176	0.00884	-1.7864

ABHD14B	-0.9116	4.19591	-3.8458	0.00851	0.02762	-3.5521
SNF8P1	-0.9114	1.59729	-9.9082	6.13E-05	0.00098	2.01989
JAK2	-0.9111	1.37225	-8.7814	0.00012	0.00151	1.24635
LRRC20	-0.909	3.36683	-6.1036	0.00088	0.00548	-1.0069
MIRLET7	-0.9083	0.47963	-4.5106	0.00406	0.016	-2.728
XYLT2	-0.9082	3.25522	-6.9479	0.00044	0.00341	-0.2217
GAL3ST1	-0.9077	1.53018	-6.5114	0.00063	0.00429	-0.6178
RAB38	-0.9073	0.80291	-5.8023	0.00115	0.00662	-1.3069
SNAP25	-0.9071	1.23068	-3.4568	0.01353	0.03927	-4.0639
ANXA6	-0.9071	5.99892	-6.6375	0.00057	0.00403	-0.5014
OGDHL	-0.9062	4.12354	-4.4878	0.00416	0.0163	-2.7553
LINC0102	-0.9058	0.90327	-6.1367	0.00086	0.00538	-0.9746
BNC2-AS	-0.9037	-0.1325	-5.3981	0.00167	0.00853	-1.7273
THAP7	-0.903	4.44154	-12.146	1.90E-05	0.00048	3.33826
PCDHB6	-0.9005	-0.2343	-3.8581	0.00839	0.02734	-3.5363
ABHD14A	-0.9002	1.75381	-12.191	1.86E-05	0.00048	3.36208
PRXL2AP	-0.9	3.04657	-9.043	0.0001	0.00135	1.43366
ACOT2	-0.8998	1.75652	-7.1517	0.00038	0.00308	-0.0435
CNP	-0.8997	5.18258	-6.6798	0.00055	0.00394	-0.4627
DPYSL2	-0.8987	4.29336	-3.253	0.01741	0.04728	-4.34
AKR7A3	-0.898	-1.488	-3.9903	0.00721	0.02447	-3.3674
MPP2	-0.8979	2.33681	-9.5993	7.34E-05	0.00109	1.81615
P4HA1	-0.8979	4.53254	-8.2854	0.00017	0.00185	0.87725
LDLRAD3	-0.8978	2.6675	-7.1582	0.00038	0.00307	-0.0379
CCNY-AS	-0.8978	-0.773	-4.4269	0.00444	0.01708	-2.8282
SHF	-0.8966	0.07507	-6.2401	0.00079	0.00504	-0.8746
YPEL2	-0.8963	-0.5201	-3.3713	0.01503	0.04236	-4.1792
FLJ20021	-0.8955	0.715	-4.3194	0.00499	0.01858	-2.9584
SCRN3	-0.8955	2.08395	-6.8879	0.00046	0.00351	-0.275
ZNF595	-0.895	-1.0935	-4.424	0.00446	0.01711	-2.8317
NHS	-0.8936	2.34369	-4.4304	0.00443	0.01703	-2.824
PSMC5	-0.8936	6.26777	-11.18	3.07E-05	0.00064	2.80016
MAP3K12	-0.8936	3.19829	-5.1347	0.00215	0.01019	-2.0128
PRKCQ-A	-0.8933	0.90226	-5.7659	0.00119	0.00676	-1.344
ARHGAP	-0.8924	2.32622	-8.447	0.00015	0.00174	0.99962
NIPAL2	-0.8908	1.15217	-4.1469	0.00604	0.02148	-3.1708
PPP1R3B	-0.8905	1.9445	-4.4234	0.00446	0.01711	-2.8324
RAB30	-0.8879	2.15779	-4.8496	0.00286	0.01246	-2.3326
PIP4P2	-0.8876	2.87764	-8.7216	0.00013	0.00154	1.20288
HS1BP3	-0.8875	2.71206	-9.7692	6.64E-05	0.00103	1.92895
CDPF1	-0.8854	2.36676	-7.0831	0.0004	0.00318	-0.1031
TGFBR2	-0.8853	6.192	-3.9645	0.00742	0.02501	-3.4003
ARL17A	-0.8851	-1.3243	-4.2306	0.0055	0.01997	-3.0673
ZNF492	-0.8827	-0.8178	-3.5857	0.01158	0.0348	-3.8921
MTMR8	-0.8826	-1.4848	-3.9906	0.0072	0.02446	-3.3671
LTBP4	-0.8826	5.34371	-5.1217	0.00218	0.01026	-2.0272
NUDT16	-0.8821	5.28167	-11.579	2.51E-05	0.00057	3.02799
PEX12	-0.8818	1.5967	-5.6727	0.00129	0.0072	-1.4394
PPP1R12	-0.8813	-1.2804	-3.7996	0.00898	0.02872	-3.6119
PLLP	-0.881	2.1139	-8.6103	0.00014	0.00162	1.12119
GTF3C6P	-0.8798	4.73094	-4.5779	0.00378	0.01524	-2.6481
PFKFB3	-0.8792	3.97553	-4.5559	0.00387	0.01546	-2.6742
CHST14	-0.8789	4.09197	-5.8115	0.00114	0.00659	-1.2977
ZNF662	-0.878	1.5871	-5.0474	0.00234	0.0108	-2.1096
ZDHHC8	-0.8779	3.76394	-9.8752	6.24E-05	0.00099	1.99843
STOML1	-0.8772	1.62518	-7.6909	0.00025	0.0024	0.40864
RASSF10	-0.8771	2.07364	-3.3935	0.01462	0.04148	-4.1491
C17orf80	-0.8769	3.41569	-6.6198	0.00057	0.00406	-0.5176

TRABD-A	-0.8754	0.84521	-4.1544	0.00599	0.02132	-3.1615
ABCD1	-0.8751	3.80023	-6.4279	0.00067	0.0045	-0.696
GASAL1	-0.8743	-0.9133	-3.9021	0.00797	0.02629	-3.4799
KMT2E-A	-0.8738	2.96256	-5.85	0.0011	0.00641	-1.2587
TMX4	-0.8736	3.07458	-4.584	0.00376	0.01518	-2.641
SIPA1	-0.8714	4.42097	-9.2911	8.82E-05	0.00123	1.60681
HEXB	-0.8711	4.78891	-7.6344	0.00026	0.00245	0.36256
STARD9	-0.8698	0.84835	-5.9728	0.00099	0.00591	-1.1358
TEDC2	-0.8696	2.51355	-6.1185	0.00087	0.00544	-0.9924
TTC34	-0.8691	-2.141	-5.399	0.00167	0.00853	-1.7264
COX11	-0.8681	3.81342	-9.4199	8.16E-05	0.00116	1.69505
SIGLEC1(	-0.8678	-0.6531	-7.9548	0.00021	0.00212	0.62038
GSTT2	-0.8661	-0.4173	-3.3671	0.01511	0.04252	-4.1849
TTF1	-0.8655	3.3454	-9.4536	8.00E-05	0.00115	1.71796
PROS1	-0.8645	0.32006	-5.2878	0.00185	0.00917	-1.8457
IER5	-0.8643	5.79171	-5.5435	0.00146	0.00781	-1.5737
ATP6V1E	-0.8638	5.01532	-6.5304	0.00062	0.00427	-0.6002
ABHD6	-0.8614	0.95725	-5.8006	0.00115	0.00662	-1.3087
PSMD11	-0.8605	5.69953	-10.735	3.88E-05	0.00074	2.53713
SPR	-0.8604	3.69182	-6.9379	0.00045	0.00342	-0.2306
ORMDL3	-0.8591	3.48172	-8.3793	0.00016	0.00178	0.94859
SLC25A2	-0.8589	0.82958	-3.5984	0.0114	0.0344	-3.8752
SLC46A1	-0.8586	1.96292	-3.7278	0.00977	0.03055	-3.7052
HOXB4	-0.8586	3.9507	-4.3666	0.00474	0.0179	-2.901
WDR41	-0.8582	2.46199	-6.3978	0.00069	0.00458	-0.7243
MLX	-0.8576	4.49348	-10.933	3.49E-05	0.0007	2.65566
PON2	-0.8564	5.64353	-8.8291	0.00012	0.00148	1.28086
RNASE1	-0.8556	-0.0179	-4.2836	0.00519	0.01915	-3.0021
ADIRF-AS	-0.8549	1.66819	-4.0898	0.00644	0.02256	-3.2421
MRPS23	-0.8545	4.41314	-8.5533	0.00014	0.00166	1.07897
CPNE7	-0.8538	1.00142	-4.7856	0.00305	0.01306	-2.4061
FICD	-0.8535	0.78272	-10.409	4.62E-05	0.00083	2.33774
C22orf39	-0.8528	2.00905	-8.6769	0.00013	0.00158	1.17016
FBXO43	-0.8522	1.70416	-7.5927	0.00027	0.0025	0.32829
CTSC	-0.8519	6.46972	-8.3477	0.00016	0.0018	0.92469
SCPEP1	-0.8513	4.60952	-6.9153	0.00045	0.00347	-0.2506
CCPG1	-0.8511	1.39462	-7.1206	0.00039	0.00313	-0.0704
EEA1	-0.8502	3.97152	-10.354	4.77E-05	0.00084	2.30375
POLR3H	-0.8495	4.61155	-4.3396	0.00488	0.01827	-2.9338
ZNF337-A	-0.8495	-0.3218	-5.2311	0.00196	0.00951	-1.9072
DNAH1	-0.8495	1.44232	-4.5732	0.0038	0.01529	-2.6538
LDAF1	-0.8494	1.27465	-3.906	0.00794	0.02621	-3.4749
MAPRE3	-0.8478	1.48205	-9.8018	6.51E-05	0.00101	1.95035
EPB41L5	-0.8475	2.97825	-6.5517	0.00061	0.00421	-0.5804
NUDT19-	-0.8469	-0.643	-4.1727	0.00587	0.02099	-3.1388
ALG12	-0.8463	3.30059	-7.7734	0.00024	0.0023	0.47552
RNF43	-0.845	2.10636	-3.5026	0.0128	0.03761	-4.0026
ANKMY2	-0.8446	2.42619	-9.1069	9.88E-05	0.00132	1.47865
CAT	-0.8443	2.50516	-5.1212	0.00218	0.01026	-2.0277
GIT2	-0.844	3.69522	-9.5909	7.37E-05	0.0011	1.81052
CDK17	-0.842	3.34609	-6.3471	0.00072	0.00473	-0.7723
STAG3L1	-0.8418	-1.9866	-6.3431	0.00072	0.00474	-0.7761
PDZK1	-0.8411	-1.5775	-3.3684	0.01508	0.04248	-4.183
BASP1	-0.8411	5.35098	-7.8518	0.00023	0.00222	0.53849
PANX2	-0.8394	2.47594	-4.7923	0.00303	0.013	-2.3983
COL25A1	-0.8393	1.14842	-6.5216	0.00062	0.00428	-0.6084
RAD51D	-0.8392	2.33301	-8.2261	0.00017	0.0019	0.83183
RNF157	-0.8392	3.79416	-3.8156	0.00882	0.02834	-3.5912

RBMX2	-0.8381	3.54991	-5.8119	0.00114	0.00659	-1.2973
ENTPD1-	-0.8368	1.83831	-4.0233	0.00694	0.02382	-3.3258
DIP2C	-0.8355	3.42946	-3.9911	0.0072	0.02446	-3.3665
MSI2	-0.8352	5.15663	-6.8112	0.00049	0.00368	-0.3436
TMEM175	-0.8343	2.73091	-7.9706	0.00021	0.00211	0.63287
MECOM	-0.8341	6.96949	-5.8814	0.00107	0.00628	-1.2271
RIBC2	-0.8338	0.36052	-4.4573	0.0043	0.01667	-2.7917
C3	-0.8331	7.04513	-4.0658	0.00662	0.02303	-3.2723
METAP1D	-0.8319	0.69743	-5.7891	0.00117	0.00666	-1.3203
SRSF9P1	-0.8315	-0.1076	-3.2323	0.01787	0.04822	-4.3684
CHST12	-0.8313	2.05568	-5.5475	0.00145	0.0078	-1.5695
RXYLT1	-0.8307	3.33808	-6.5964	0.00058	0.00411	-0.5391
HPS4	-0.8301	3.36506	-8.8296	0.00012	0.00148	1.28123
KCND1	-0.83	-0.0239	-3.2589	0.01729	0.04699	-4.3321
PRXL2A	-0.8299	6.20073	-6.1691	0.00084	0.00527	-0.9432
NAT9	-0.8289	3.89475	-6.7397	0.00052	0.00382	-0.4082
PLPP2	-0.8287	3.40546	-5.1652	0.00209	0.00997	-1.9793
ACVR1B	-0.8287	3.88675	-6.1357	0.00086	0.00538	-0.9756
RASSF3	-0.8278	4.15079	-7.1245	0.00039	0.00312	-0.067
C17orf75	-0.8267	1.91621	-7.5011	0.00029	0.00261	0.2526
PRR5	-0.8264	2.02288	-8.298	0.00017	0.00184	0.88686
PANK2-A	-0.8252	0.95312	-3.2204	0.01814	0.04873	-4.3847
CALCOC(	-0.8248	3.87606	-3.6017	0.01135	0.03429	-3.8708
CGAS	-0.8245	2.7916	-3.6607	0.01058	0.03247	-3.7931
PDCD6	-0.824	3.09659	-6.6915	0.00054	0.00391	-0.452
AFMID	-0.8236	3.25736	-9.6567	7.09E-05	0.00107	1.85444
DLG4	-0.8235	2.07458	-4.1219	0.00621	0.02195	-3.202
SBK3	-0.8228	-2.2407	-6.7938	0.0005	0.0037	-0.3593
SGCB	-0.8228	2.29183	-4.6416	0.00354	0.01454	-2.5733
CAVIN3	-0.8228	2.84314	-6.8526	0.00048	0.00359	-0.3065
MRPL40	-0.8224	4.67317	-8.2294	0.00017	0.00189	0.83435
NBN	-0.8211	5.17814	-6.9937	0.00043	0.00334	-0.1813
CHMP6	-0.8207	2.64535	-5.9199	0.00104	0.00612	-1.1885
RBMXP4	-0.8198	-0.5137	-4.5962	0.00371	0.01504	-2.6267
ABCC3	-0.8195	1.49394	-5.7427	0.00121	0.00687	-1.3676
TMEM17	-0.8194	-0.0955	-5.8757	0.00108	0.00631	-1.2328
CABYR	-0.8194	2.27511	-8.0178	0.0002	0.00207	0.67004
SMG9	-0.8191	3.87418	-4.1948	0.00573	0.02059	-3.1114
STEAP3	-0.8187	1.48522	-3.7779	0.00921	0.02925	-3.6399
ENOSF1	-0.8184	3.53225	-5.602	0.00138	0.00756	-1.5126
FAM81A	-0.8179	-0.5068	-4.0693	0.00659	0.02296	-3.2679
CC2D1B	-0.8176	4.21198	-8.001	0.0002	0.00209	0.65678
FLCN	-0.816	2.32291	-8.9854	0.00011	0.00138	1.3928
SLC19A1	-0.8156	3.97054	-3.5868	0.01156	0.03476	-3.8906
ELK3	-0.8153	4.0769	-4.5338	0.00397	0.01574	-2.7005
ENDOV	-0.8141	2.22489	-4.7556	0.00315	0.01333	-2.4406
RIPK2-DT	-0.8137	1.84147	-9.535	7.62E-05	0.00111	1.77299
PARP10	-0.8133	4.21446	-5.4711	0.00156	0.00816	-1.6499
ANKRD7	-0.8127	-0.7626	-3.4251	0.01407	0.04038	-4.1065
DYRK4	-0.8119	-0.128	-8.4827	0.00015	0.00171	1.02633
PIGH	-0.8118	1.44924	-6.443	0.00066	0.00447	-0.6818
NDUFAF8	-0.8116	5.06832	-7.5924	0.00027	0.0025	0.32809
NFE2L1-D	-0.8115	-0.4072	-3.306	0.0163	0.04497	-4.2678
ERG	-0.8106	2.87495	-3.4129	0.01428	0.04078	-4.123
DUSP3	-0.8105	4.84635	-6.1701	0.00083	0.00527	-0.9422
GOLGA1	-0.8104	2.03265	-9.3584	8.47E-05	0.00118	1.65306
CUEDC1	-0.8102	3.50198	-10.219	5.14E-05	0.00088	2.21902
AMDHD2	-0.8098	3.02677	-7.0588	0.00041	0.00323	-0.1242

ATP1A3	-0.8088	1.06045	-4.0434	0.00679	0.0234	-3.3005
STAT5B	-0.8079	4.49424	-5.8006	0.00115	0.00662	-1.3087
UPRT	-0.8078	1.66209	-9.1974	9.34E-05	0.00127	1.54192
SPATC1L	-0.8064	1.64613	-6.6265	0.00057	0.00405	-0.5115
URB1-AS	-0.8057	2.6882	-6.004	0.00096	0.0058	-1.1049
SCRN2	-0.8045	4.1817	-3.3282	0.01585	0.04408	-4.2375
DUS1L	-0.8035	5.68254	-11.161	3.10E-05	0.00065	2.78949
ACSS3	-0.8032	-6.3856	-6.562	0.0006	0.00419	-0.5709
TRMT2A	-0.8032	5.11874	-10.355	4.76E-05	0.00084	2.30432
GNS	-0.8031	6.83506	-7.2206	0.00036	0.00296	0.01582
DTX3	-0.8031	4.05667	-4.9027	0.00271	0.01199	-2.2723
CDHR3	-0.8023	-0.6383	-4.3059	0.00507	0.01876	-2.9748
FKBP11	-0.8022	3.58232	-3.2983	0.01646	0.04527	-4.2782
FAM234B	-0.802	0.79634	-5.6116	0.00137	0.00751	-1.5027
AOC2	-0.8018	0.63329	-3.359	0.01526	0.04281	-4.1959
METTL23	-0.8017	3.577	-6.7237	0.00053	0.00385	-0.4227
HOXA1	-0.8008	1.79901	-6.2983	0.00075	0.00487	-0.8188
NFU1	-0.7987	2.64992	-8.3619	0.00016	0.00179	0.93543
EEPD1	-0.7985	0.98029	-5.0309	0.00238	0.01091	-2.128
ZFHX4	-0.7982	2.96434	-3.9633	0.00743	0.02502	-3.4018
GRB14	-0.7981	1.80471	-3.5019	0.01281	0.03763	-4.0035
RAPGEFL	-0.798	1.13889	-4.8497	0.00286	0.01246	-2.3326
FAM3C	-0.7977	5.14845	-6.0785	0.0009	0.00555	-1.0315
FAM174B	-0.7973	2.63399	-3.5148	0.01261	0.03721	-3.9864
DHX40	-0.7965	3.10746	-10.296	4.92E-05	0.00086	2.26724
BPTF	-0.7964	4.46289	-8.8731	0.00011	0.00145	1.31257
DSC2	-0.7964	1.38854	-3.3112	0.01619	0.04478	-4.2607
SYNJ1	-0.7961	2.96106	-8.4783	0.00015	0.00171	1.02309
PAK1	-0.7959	3.39833	-7.3536	0.00032	0.00276	0.12902
B3GAT3F	-0.7949	2.87715	-7.5044	0.00029	0.00261	0.25531
ZNF219	-0.7949	2.40835	-6.0119	0.00096	0.00578	-1.097
ZC3HAV1	-0.7947	4.69955	-6.1551	0.00085	0.00532	-0.9567
RBMX2P5	-0.7947	2.00493	-7.3896	0.00032	0.00272	0.15938
BDH2P1	-0.7945	4.17239	-4.8851	0.00276	0.01214	-2.2922
SLC20A1	-0.7944	1.13253	-4.7103	0.0033	0.01381	-2.4932
BECN1	-0.7942	4.02724	-11.067	3.25E-05	0.00067	2.73442
TBKBP1	-0.7942	2.34236	-7.6598	0.00026	0.00243	0.3833
MINPP1	-0.7939	3.81647	-7.8814	0.00022	0.00219	0.5621
MIF4GD-I	-0.7925	-0.6321	-3.1986	0.01865	0.04972	-4.4147
SLC25A1	-0.7918	2.07684	-9.9896	5.85E-05	0.00095	2.0726
FSD1L	-0.7916	2.36188	-4.8959	0.00273	0.01205	-2.28
ZNF512B	-0.7912	5.59132	-5.6833	0.00128	0.00715	-1.4286
SPATA24	-0.7901	0.93666	-5.5631	0.00143	0.00773	-1.5532
GRN	-0.7882	6.02922	-7.7386	0.00025	0.00235	0.4474
ENKD1	-0.7882	0.87776	-3.4118	0.0143	0.04081	-4.1245
GTF3C6	-0.7868	2.93188	-7.1119	0.00039	0.00314	-0.078
RNF144A	-0.7861	2.60358	-5.7357	0.00122	0.00691	-1.3748
TEX264	-0.7861	3.35313	-7.878	0.00022	0.0022	0.55941
LINC0260	-0.7839	0.76191	-4.4802	0.0042	0.01638	-2.7643
CSPG5	-0.7836	1.91238	-3.222	0.01811	0.04866	-4.3826
TUBG1P	-0.783	2.23037	-7.4331	0.00031	0.00269	0.19588
ZHX1	-0.7828	3.79829	-6.353	0.00071	0.00471	-0.7667
FUOM	-0.7819	3.20416	-4.8219	0.00294	0.01274	-2.3643
RECQL5	-0.7817	2.82412	-7.2987	0.00034	0.00283	0.08251
TERF1P4	-0.7808	4.2841	-8.3465	0.00016	0.0018	0.92376
ARFRP1	-0.7805	4.22983	-9.8307	6.41E-05	0.001	1.96932
SLC9A1	-0.7804	3.93569	-7.8242	0.00023	0.00225	0.51635
TRABD	-0.7802	5.09396	-8.7218	0.00013	0.00154	1.20296

ZFP41	-0.78	2.29092	-4.0079	0.00706	0.02411	-3.3452
WDR31	-0.7797	-1.497	-3.5153	0.0126	0.03719	-3.9856
LONRF3	-0.7797	1.13685	-6.1785	0.00083	0.00524	-0.9341
C12orf75	-0.7795	6.39153	-5.8894	0.00107	0.00625	-1.2191
DYNC2H1	-0.7793	4.44405	-8.2048	0.00018	0.00191	0.81542
TNFAIP8	-0.7789	1.43471	-6.3973	0.00069	0.00458	-0.7248
UBR7	-0.7788	4.15901	-8.3776	0.00016	0.00178	0.94734
DGLUCY	-0.7785	4.51362	-7.2276	0.00036	0.00294	0.02182
TIGD7	-0.7777	1.44334	-6.5908	0.00059	0.00412	-0.5443
TOM1L1	-0.7769	3.1198	-6.8229	0.00049	0.00365	-0.3331
FAM117A	-0.7763	3.47213	-8.1602	0.00018	0.00195	0.78107
SV2A	-0.7762	4.81286	-5.1507	0.00212	0.01006	-1.9953
NRG4	-0.7755	0.28933	-5.2051	0.00201	0.0097	-1.9356
GALM	-0.7754	3.26448	-8.2178	0.00018	0.0019	0.82547
SPACA9	-0.7749	-0.2264	-5.2901	0.00185	0.00916	-1.8433
MIEN1	-0.7748	4.80056	-6.1917	0.00082	0.0052	-0.9213
LCMT1-A	-0.7744	-2.7662	-3.872	0.00826	0.027	-3.5184
HEXD	-0.7739	2.35167	-5.9927	0.00097	0.00584	-1.116
PNPO	-0.7738	3.62494	-8.7766	0.00012	0.00151	1.2429
TYSND1	-0.773	4.29112	-7.076	0.0004	0.0032	-0.1092
PSD3	-0.7715	2.96895	-4.8363	0.0029	0.01259	-2.3478
PRDX5	-0.7713	6.70408	-5.4226	0.00163	0.00842	-1.7012
TAF15	-0.7711	5.2672	-9.0538	0.0001	0.00135	1.44131
FSD1	-0.7709	2.59173	-6.9452	0.00044	0.00342	-0.2241
FAM13A	-0.7707	2.27954	-4.1153	0.00626	0.02209	-3.2102
POC1B	-0.7687	1.42105	-3.4458	0.01372	0.03966	-4.0787
KIF3C	-0.7685	2.88575	-7.966	0.00021	0.00212	0.62923
HK2-DT	-0.7682	2.34863	-4.4089	0.00453	0.01729	-2.8499
VPS25	-0.7676	5.34188	-4.3672	0.00474	0.0179	-2.9003
ZNF471	-0.7673	-0.7935	-3.5195	0.01254	0.03703	-3.98
ADD2	-0.7659	2.43947	-3.1929	0.01878	0.04996	-4.4226
CTNS	-0.7656	2.53005	-5.9441	0.00102	0.00602	-1.1644
ACAP3	-0.765	3.67283	-5.0846	0.00226	0.0105	-2.0683
CADM1	-0.7649	4.23162	-4.8692	0.0028	0.01231	-2.3103
C10orf88	-0.7641	-0.1604	-4.0699	0.00658	0.02296	-3.267
SAP30BP	-0.7626	3.74612	-9.5713	7.46E-05	0.0011	1.79737
DOCK10	-0.7621	0.17312	-3.573	0.01175	0.03519	-3.9088
PRKACB-	-0.7619	1.2969	-4.8753	0.00278	0.01224	-2.3034
CHCHD10	-0.7613	6.86479	-6.4858	0.00064	0.00436	-0.6418
PTER	-0.7602	3.28787	-7.3616	0.00032	0.00275	0.13576
COX11P1	-0.7597	2.00785	-5.3781	0.0017	0.00866	-1.7487
CYSTM1	-0.7586	4.27921	-4.3932	0.00461	0.01751	-2.8689
JPT1	-0.7585	5.38758	-6.6936	0.00054	0.00391	-0.4501
LIMCH1	-0.7584	6.3171	-6.297	0.00075	0.00487	-0.82
ARHGEF4	-0.7578	4.0726	-5.3759	0.00171	0.00867	-1.751
GPR137	-0.7578	3.71563	-4.8577	0.00283	0.0124	-2.3235
CLTCL1	-0.7576	2.15049	-8.0766	0.00019	0.00203	0.71608
RSPO4	-0.7568	1.75926	-3.7814	0.00917	0.02918	-3.6354
SH3BGRL	-0.7564	4.16853	-8.6037	0.00014	0.00162	1.11628
ASB16-AS	-0.7562	2.40371	-6.3233	0.00073	0.0048	-0.795
IDUA	-0.7558	2.65096	-8.1125	0.00019	0.00199	0.74403
TMEM106	-0.7554	6.14303	-4.803	0.003	0.01291	-2.3861
CYTOR	-0.7538	1.80527	-5.0754	0.00228	0.01057	-2.0784
PYGB	-0.7534	5.57866	-6.109	0.00088	0.00546	-1.0016
ZNF605	-0.7529	-0.0771	-3.7734	0.00926	0.02936	-3.6457
DNAJC24	-0.7521	1.28488	-8.0605	0.0002	0.00204	0.70352
BTBD6	-0.7517	5.20728	-4.025	0.00693	0.02379	-3.3236
ZNF570	-0.7517	1.16699	-6.31	0.00074	0.00483	-0.8077

LUC7L3	-0.7514	5.27912	-5.4087	0.00165	0.0085	-1.7161
GJC1	-0.7514	3.59813	-6.5806	0.00059	0.00415	-0.5537
TCTN2	-0.7511	2.87844	-7.0649	0.0004	0.00321	-0.1189
SLC35B4	-0.751	3.80598	-5.1518	0.00212	0.01006	-1.994
TOP3B	-0.7508	3.53225	-5.6287	0.00135	0.00742	-1.485
ZDHHC12	-0.7506	0.14146	-3.243	0.01764	0.04768	-4.3538
ENGASE	-0.7496	3.8651	-5.275	0.00188	0.00925	-1.8596
SHISA8	-0.7488	-0.8987	-3.2071	0.01845	0.04938	-4.403
PRR19	-0.7484	0.77967	-5.2213	0.00198	0.00958	-1.9179
TMUB2	-0.7484	3.87711	-8.91	0.00011	0.00143	1.33906
WDR54	-0.7483	3.31222	-3.5227	0.01249	0.03691	-3.9757
TUBG1	-0.7478	5.09547	-8.7424	0.00012	0.00153	1.21805
ZNF529-A	-0.7477	-0.0731	-3.8979	0.00801	0.02639	-3.4852
ATP5MC2	-0.7467	5.04337	-7.0937	0.00039	0.00316	-0.0938
FAM3C2P	-0.7461	7.20263	-5.4211	0.00163	0.00843	-1.7028
POLG2	-0.7455	1.86189	-5.6005	0.00138	0.00756	-1.5142
ORAI2	-0.7451	5.48833	-7.5348	0.00028	0.00257	0.2805
C2CD2	-0.7445	3.00067	-7.3771	0.00032	0.00273	0.14883
VIM	-0.7439	8.39134	-4.1282	0.00617	0.02183	-3.1942
TRAF7	-0.7438	5.11381	-8.3915	0.00016	0.00178	0.95782
SNHG20	-0.7436	2.5248	-5.1316	0.00216	0.0102	-2.0163
C12orf43	-0.7435	2.61175	-9.6628	7.07E-05	0.00107	1.85853
GHDC	-0.7434	3.23807	-6.3311	0.00073	0.00478	-0.7875
MSH5	-0.7433	-0.6138	-3.5562	0.01199	0.03573	-3.9312
SH2B2	-0.7427	4.07604	-7.4414	0.0003	0.00268	0.20279
IGFBP7	-0.7406	6.43464	-6.4635	0.00065	0.00442	-0.6626
NCK1-DT	-0.7402	1.07468	-7.3764	0.00032	0.00273	0.14824
HEG1	-0.7388	4.26749	-3.7545	0.00947	0.02983	-3.6703
RAD51C	-0.738	3.33625	-4.6806	0.0034	0.0141	-2.5277
GXYLT2	-0.7378	2.9108	-6.9131	0.00045	0.00347	-0.2526
KIAA0930	-0.7359	4.7361	-7.0106	0.00042	0.00331	-0.1664
CD63-AS	-0.7358	3.0415	-5.2093	0.002	0.00966	-1.931
SRRD	-0.7354	3.32804	-9.3287	8.63E-05	0.0012	1.63269
BRCA1	-0.7353	2.68714	-5.3993	0.00167	0.00853	-1.7261
MAFG	-0.7351	5.14568	-7.7362	0.00025	0.00235	0.4454
CCDC102	-0.7348	1.79126	-5.5496	0.00145	0.0078	-1.5673
TRIM37	-0.7346	3.64961	-9.235	9.13E-05	0.00125	1.56803
GTF2IP20	-0.7341	1.29397	-4.1129	0.00627	0.02211	-3.2132
VLDLR	-0.7339	4.81977	-6.7825	0.0005	0.00372	-0.3694
RRAS2	-0.7333	4.65286	-8.3013	0.00017	0.00184	0.88939
RNF166	-0.7322	2.56575	-8.0131	0.0002	0.00208	0.66636
RASSF8	-0.7319	2.62649	-6.9077	0.00046	0.00348	-0.2574
APLP1	-0.7316	4.73305	-3.8906	0.00808	0.02654	-3.4946
EXTL3	-0.7313	4.53262	-9.6035	7.32E-05	0.00109	1.81899
TBC1D8-A	-0.7313	-0.5236	-4.0782	0.00652	0.02279	-3.2567
CA13	-0.731	1.03588	-3.695	0.01016	0.03144	-3.7481
RAB5C	-0.7306	3.57574	-7.2745	0.00034	0.00287	0.06188
MTERF2	-0.7292	2.27506	-7.8956	0.00022	0.00218	0.5734
PTRH2	-0.7286	3.05641	-5.9997	0.00097	0.00582	-1.1091
KLHL35	-0.7272	1.75995	-7.2183	0.00036	0.00296	0.01385
C1orf216	-0.7271	3.06279	-3.812	0.00885	0.02842	-3.5957
C1QBPP2	-0.7264	3.17355	-6.5516	0.00061	0.00421	-0.5805
CHPT1	-0.7261	1.94947	-5.1722	0.00207	0.00991	-1.9716
EPB41L4A	-0.7255	4.0378	-6.4556	0.00066	0.00444	-0.67
CHIC2	-0.7247	1.24564	-3.4023	0.01447	0.04118	-4.1372
TOMM40L	-0.7245	4.14448	-6.5043	0.00063	0.00431	-0.6244
LINC0278	-0.7245	0.5985	-4.2279	0.00552	0.02001	-3.0706
CFAP44	-0.7241	-0.2394	-5.9532	0.00101	0.00599	-1.1553

TMEM115	-0.7239	3.85152	-6.3988	0.00069	0.00458	-0.7234
RTEL1-TI	-0.7237	0.17129	-4.6557	0.00349	0.01438	-2.5567
GATD1	-0.7233	4.04601	-7.807	0.00023	0.00227	0.50256
ARRDC1-	-0.723	3.07303	-6.8037	0.0005	0.00369	-0.3504
NEIL2	-0.7229	1.48611	-6.6327	0.00057	0.00404	-0.5057
RPP25	-0.7228	4.74615	-5.9897	0.00098	0.00585	-1.1191
TRAPPC1	-0.7227	4.40351	-8.1003	0.00019	0.002	0.73456
RGS19	-0.7226	3.65339	-5.7896	0.00116	0.00666	-1.3199
IQSEC1	-0.7216	3.93642	-6.6709	0.00055	0.00396	-0.4708
LKAAEAR	-0.7215	0.17556	-4.1524	0.006	0.02137	-3.164
C22orf46	-0.7213	2.11131	-5.4474	0.00159	0.0083	-1.6749
NFU1P1	-0.7212	1.51228	-4.4566	0.0043	0.01667	-2.7925
GEMIN6	-0.7211	2.93169	-4.5916	0.00373	0.01509	-2.632
ARAP3	-0.7209	5.11131	-8.6053	0.00014	0.00162	1.11749
ZNF32	-0.7209	2.76796	-4.7557	0.00315	0.01333	-2.4406
PCED1A	-0.7204	4.14827	-5.2592	0.00191	0.00936	-1.8767
TIMP1	-0.72	5.64303	-3.8136	0.00884	0.02839	-3.5938
GPRASP2	-0.7199	2.05158	-5.0219	0.0024	0.01098	-2.1381
ACVR1	-0.7189	2.3853	-8.0679	0.00019	0.00203	0.70929
SGSH	-0.7185	4.24708	-7.6808	0.00026	0.00241	0.40044
ANTXR1	-0.7184	3.89992	-3.1991	0.01864	0.0497	-4.414
ANKRD37	-0.7184	-0.512	-3.676	0.01039	0.03203	-3.7729
MOB3C	-0.7179	1.44678	-4.3308	0.00493	0.01841	-2.9445
HYAL2	-0.7175	3.84207	-8.1504	0.00018	0.00196	0.77344
PPIL2	-0.7165	4.2062	-8.9793	0.00011	0.00139	1.38849
NMT1	-0.7154	6.63969	-9.6943	6.94E-05	0.00106	1.87944
SEPTIN6	-0.7138	3.21733	-6.2328	0.00079	0.00506	-0.8816
C4orf48	-0.7134	3.08138	-5.8006	0.00115	0.00662	-1.3087
SPOP	-0.7132	3.53891	-8.7068	0.00013	0.00156	1.19202
ZNF461	-0.7131	0.56886	-8.2593	0.00017	0.00187	0.85734
VMP1	-0.7127	6.18584	-3.9051	0.00795	0.02623	-3.476
RPL19P1	-0.7123	0.47266	-4.2463	0.00541	0.01974	-3.0479
PDK2	-0.7119	2.47242	-8.1638	0.00018	0.00195	0.78381
TRMU	-0.7117	4.5153	-6.8935	0.00046	0.0035	-0.27
CAPN15	-0.7116	4.48178	-4.7515	0.00316	0.01336	-2.4454
GSTO3P	-0.7113	0.88979	-4.9785	0.00251	0.01131	-2.1866
FCGRT	-0.7104	3.0949	-5.1379	0.00214	0.01016	-2.0094
ZNF703	-0.7103	4.64437	-5.8022	0.00115	0.00662	-1.3071
ZNF530	-0.7096	1.5298	-4.4836	0.00418	0.01635	-2.7603
ZFPM1	-0.7095	0.71787	-6.5708	0.0006	0.00417	-0.5628
SNX29	-0.7094	2.62127	-5.6584	0.00131	0.00727	-1.4542
NPAS1	-0.709	-1.0198	-4.6848	0.00338	0.01405	-2.5228
LGALS3B	-0.7089	7.74306	-7.0135	0.00042	0.00331	-0.1639
ARHGAP	-0.7075	1.6762	-3.4808	0.01314	0.0384	-4.0318
CCDC85B	-0.7073	6.02282	-4.9975	0.00246	0.01116	-2.1653
GPRC5C	-0.7071	3.6159	-3.6499	0.01072	0.0328	-3.8072
MANSC1	-0.7071	1.97159	-5.1604	0.0021	0.00999	-1.9846
DMPK	-0.7062	3.75027	-7.1413	0.00038	0.0031	-0.0525
SUPT4H1	-0.7057	1.50888	-7.9275	0.00021	0.00215	0.59874
KLHL20	-0.7056	2.43516	-5.5417	0.00146	0.00782	-1.5756
EIF2B1	-0.7054	4.96173	-8.122	0.00019	0.00198	0.75145
MOCS2	-0.7054	3.62955	-7.8903	0.00022	0.00218	0.56918
GNG11	-0.705	6.61268	-7.319	0.00033	0.0028	0.09971
MINDY1	-0.7047	2.20255	-4.5074	0.00408	0.01603	-2.7318
SPRED1	-0.704	2.72815	-8.4115	0.00015	0.00177	0.97291
SQOR	-0.7037	0.45381	-5.1378	0.00214	0.01016	-2.0095
TKFC	-0.7035	2.62171	-6.4423	0.00066	0.00447	-0.6825
BCAR1	-0.7009	5.08135	-3.7051	0.01004	0.03116	-3.7348

RBMX2P3	-0.7003	0.60964	-5.3182	0.0018	0.00898	-1.813
NECAB3	-0.6998	3.87883	-7.3763	0.00032	0.00273	0.14819
WASHC3	-0.6993	0.80605	-7.4211	0.00031	0.00269	0.1858
TPI1P2	-0.6993	0.95275	-4.2247	0.00554	0.02007	-3.0745
USP18	-0.6992	0.54651	-3.8615	0.00836	0.02726	-3.532
ZNF83	-0.6986	3.27554	-3.56	0.01194	0.03561	-3.9262
STX11	-0.6984	-1.5623	-3.8693	0.00828	0.02707	-3.5219
CD63	-0.6983	7.7841	-4.6371	0.00356	0.01459	-2.5786
BID	-0.6981	4.02081	-4.3884	0.00463	0.01755	-2.8746
SQSTM1	-0.698	7.5654	-4.351	0.00482	0.01809	-2.92
ERCC6L2	-0.6976	1.61445	-7.6502	0.00026	0.00244	0.37547
SYN1	-0.6973	-0.0589	-3.4177	0.0142	0.04062	-4.1165
CACNB3	-0.6963	2.91338	-7.1284	0.00038	0.00312	-0.0637
INO80D-A	-0.6961	0.70058	-3.5844	0.01159	0.03484	-3.8938
SBF1	-0.6956	5.89305	-5.7472	0.00121	0.00685	-1.363
DUSP8P5	-0.6946	0.55406	-5.2701	0.00189	0.00928	-1.8649
ILRUN-AS	-0.6939	-0.2982	-3.2743	0.01696	0.04623	-4.311
SLC4A2	-0.6932	6.40187	-5.0362	0.00237	0.01087	-2.1221
IAH1	-0.6929	2.0771	-5.5027	0.00151	0.00801	-1.6165
SH3GL1P	-0.6927	-1.132	-4.4053	0.00455	0.01734	-2.8542
KCTD2	-0.692	2.74339	-8.6175	0.00013	0.00161	1.1265
ZNF276	-0.6919	2.78885	-5.5928	0.00139	0.00759	-1.5222
SLC18B1	-0.6918	2.54786	-6.0736	0.00091	0.00556	-1.0363
MXRA7	-0.6916	6.19545	-5.1834	0.00205	0.00984	-1.9593
GPR75	-0.6912	-0.9722	-5.6896	0.00127	0.00713	-1.4221
ZNF830	-0.6909	3.30354	-5.2273	0.00197	0.00953	-1.9114
PALS2	-0.6908	3.2395	-3.8954	0.00804	0.02645	-3.4884
NUDT4	-0.6905	3.26255	-6.28	0.00076	0.00492	-0.8363
NGLY1	-0.6902	3.37372	-5.7919	0.00116	0.00666	-1.3175
SPAG9	-0.6892	4.67666	-6.6451	0.00056	0.00401	-0.4944
CASP8	-0.689	3.93508	-4.0541	0.0067	0.02324	-3.2869
LINC0020	-0.6887	2.95032	-8.5953	0.00014	0.00162	1.11011
TOP2A	-0.6879	7.07455	-9.0617	0.0001	0.00134	1.44685
CTSF	-0.6877	3.66921	-5.4881	0.00153	0.00806	-1.6319
NDUFV2P	-0.6857	6.78193	-8.6571	0.00013	0.00159	1.15563
BICC1	-0.6855	6.28622	-7.5271	0.00029	0.00258	0.27419
RNASEH2	-0.6855	4.86407	-5.0347	0.00237	0.01088	-2.1237
GBP2	-0.6849	3.3795	-6.9863	0.00043	0.00334	-0.1879
MPG	-0.6847	2.87622	-7.0249	0.00042	0.00329	-0.1539
SHC1P1	-0.6846	0.69817	-4.5691	0.00382	0.01535	-2.6586
OGFOD2	-0.6829	-0.2198	-3.4101	0.01433	0.04086	-4.1268
CHIC1	-0.6827	2.24733	-7.2775	0.00034	0.00287	0.06446
SYNGR1	-0.6826	2.81955	-7.4182	0.00031	0.00269	0.18341
SMIM10	-0.6824	2.24248	-4.49	0.00415	0.01627	-2.7525
TRIM5	-0.6823	2.90802	-6.5252	0.00062	0.00427	-0.605
ESS2	-0.6822	3.14529	-8.6753	0.00013	0.00158	1.16898
RAB35	-0.682	4.11249	-4.6728	0.00343	0.01419	-2.5367
MAP3K10	-0.682	3.04562	-3.3068	0.01628	0.04495	-4.2667
MTMR4	-0.6817	3.88588	-6.8581	0.00047	0.00357	-0.3016
PCDHGB	-0.6811	-2.5635	-3.3532	0.01537	0.04303	-4.2037
MED27	-0.6807	1.97808	-6.8486	0.00048	0.0036	-0.3101
PPP6R2	-0.6802	4.41902	-8.2132	0.00018	0.00191	0.8219
LINC0096	-0.6797	3.30725	-3.2826	0.01678	0.04594	-4.2997
SCMH1	-0.6795	3.40703	-6.0374	0.00094	0.00569	-1.0719
TACC1	-0.6792	4.43816	-4.2598	0.00533	0.01951	-3.0312
ZNF316	-0.6789	3.46937	-5.4327	0.00162	0.00838	-1.6905
ATOSA	-0.6789	1.64271	-4.0121	0.00703	0.02403	-3.3399
ZC3HC1	-0.6787	3.37555	-5.6046	0.00138	0.00755	-1.51

TRAPPC2	-0.6775	3.72948	-8.0237	0.0002	0.00207	0.67467
NEU1	-0.6772	6.0125	-5.3356	0.00177	0.00888	-1.7942
HSPA1A	-0.6772	5.0531	-4.9411	0.00261	0.01164	-2.2288
NAPEPLD	-0.677	2.72253	-3.5016	0.01281	0.03764	-4.0039
EAF2	-0.6761	-0.7081	-3.9462	0.00758	0.02539	-3.4235
TBC1D32	-0.676	0.26582	-4.7013	0.00333	0.01389	-2.5036
TOX	-0.6759	3.09505	-4.7185	0.00327	0.01373	-2.4836
EPB41L2	-0.675	5.87025	-6.7158	0.00053	0.00386	-0.4298
AGMAT	-0.6741	0.69236	-4.5354	0.00396	0.01572	-2.6985
AGPAT3	-0.674	4.17579	-6.1273	0.00087	0.00541	-0.9838
PRR34-A	-0.6738	1.48699	-6.6976	0.00054	0.00389	-0.4464
GOSR2	-0.6738	2.46963	-6.6565	0.00056	0.00399	-0.484
LIPE-AS1	-0.6736	-1.2541	-4.8074	0.00298	0.01286	-2.381
CDC42EF	-0.6727	3.90273	-6.2605	0.00077	0.00498	-0.855
C3orf62	-0.6723	0.85526	-5.097	0.00223	0.01042	-2.0545
DNM3	-0.6715	3.02719	-4.5259	0.004	0.01583	-2.7098
TMEM43	-0.6712	4.40002	-4.0566	0.00668	0.0232	-3.2838
AGPAT2	-0.6709	3.48804	-5.5116	0.0015	0.00796	-1.6071
NMD3	-0.6707	5.4507	-7.9624	0.00021	0.00212	0.62641
TRPM2	-0.6707	3.35319	-7.1047	0.00039	0.00315	-0.0842
TERF1P5	-0.67	2.5177	-6.6404	0.00056	0.00402	-0.4987
CEP128	-0.6699	3.41066	-6.5776	0.00059	0.00416	-0.5565
KLF10	-0.6694	4.89858	-4.0003	0.00712	0.02426	-3.3548
PCNX2	-0.6691	3.51369	-7.5822	0.00027	0.00252	0.31971
DNAJA2-I	-0.669	-1.6457	-4.7137	0.00328	0.01377	-2.4892
CDK11A	-0.6688	-2.119	-3.5068	0.01273	0.03749	-3.997
NRCAM	-0.6686	7.35523	-4.1778	0.00583	0.0209	-3.1324
PBLD	-0.6686	0.841	-5.3143	0.00181	0.00901	-1.8171
MED24	-0.6661	3.90853	-5.9117	0.00104	0.00616	-1.1968
C11orf1	-0.6652	1.98876	-7.4498	0.0003	0.00267	0.20983
DTWD2	-0.6645	1.93959	-5.268	0.00189	0.0093	-1.8671
PDE9A	-0.6641	2.37866	-3.8467	0.0085	0.02762	-3.551
CDC6	-0.6639	5.1167	-4.3105	0.00504	0.01872	-2.9692
RARA	-0.6639	2.42423	-7.6196	0.00027	0.00247	0.3504
RNF126P	-0.6638	0.59957	-3.9732	0.00735	0.02481	-3.3892
AMZ2	-0.6635	4.47665	-8.372	0.00016	0.00178	0.94309
ATP6V1E	-0.6631	3.37833	-5.0121	0.00243	0.01104	-2.149
UHMK1	-0.663	5.99164	-8.812	0.00012	0.00149	1.26851
C1QTNF6	-0.6627	2.09373	-6.1871	0.00082	0.00521	-0.9257
BIN1	-0.6627	4.25435	-6.5159	0.00062	0.00429	-0.6137
ZCCHC12	-0.6624	-2.205	-5.0671	0.0023	0.01063	-2.0877
PSMD12	-0.6622	4.53363	-6.7296	0.00053	0.00384	-0.4173
PRRG1	-0.6618	3.82038	-7.7792	0.00024	0.0023	0.48021
SNX8	-0.6616	2.68292	-6.4282	0.00067	0.0045	-0.6957
POLR2L	-0.6615	5.59771	-7.3663	0.00032	0.00275	0.13976
RAC2	-0.6613	1.75275	-5.1114	0.0022	0.01032	-2.0385
ZNF875	-0.6606	2.47888	-4.3051	0.00507	0.01877	-2.9758
THAP3	-0.6593	1.7178	-8.7885	0.00012	0.00151	1.25152
CENPM	-0.6593	4.42686	-3.5951	0.01145	0.0345	-3.8796
MLKL	-0.6588	0.97456	-3.9633	0.00743	0.02502	-3.4018
PSMD3	-0.6586	6.04626	-7.9539	0.00021	0.00212	0.6197
SBF2-AS1	-0.6583	0.61626	-5.0172	0.00241	0.01101	-2.1433
TERF1P2	-0.6577	0.34409	-4.1324	0.00614	0.02175	-3.1889
HEXIM2	-0.6571	2.57693	-8.4651	0.00015	0.00173	1.0132
CRYZ	-0.6567	5.21134	-5.5686	0.00142	0.0077	-1.5475
UTP18	-0.6564	2.90735	-5.9157	0.00104	0.00614	-1.1928
ZNF138	-0.6561	1.61134	-5.5484	0.00145	0.0078	-1.5685
BMT2	-0.6555	2.5305	-6.5397	0.00061	0.00424	-0.5915

HIGD1AP	-0.6554	5.17427	-5.1622	0.00209	0.00998	-1.9826
PMM1	-0.6552	3.35328	-6.1666	0.00084	0.00528	-0.9456
DOK1	-0.6551	2.38237	-5.7668	0.00119	0.00676	-1.3431
FBXW5	-0.6546	4.94776	-5.755	0.0012	0.00681	-1.3551
HEATR6	-0.6543	1.81313	-6.0784	0.0009	0.00555	-1.0316
LRFN1	-0.653	2.45562	-4.295	0.00513	0.01894	-2.9882
ZNF546	-0.6528	-1.0878	-4.3063	0.00506	0.01876	-2.9743
WWTR1	-0.6527	5.48041	-3.4128	0.01428	0.04078	-4.1231
METTL1F	-0.6527	0.98599	-3.4698	0.01332	0.03882	-4.0465
APPBP2	-0.6502	3.59252	-6.9823	0.00043	0.00335	-0.1914
LINC0046	-0.6502	0.45015	-3.7908	0.00907	0.02894	-3.6232
LMF2	-0.6491	5.68259	-5.4067	0.00166	0.00851	-1.7181
PCNX4	-0.649	4.04813	-6.5119	0.00063	0.00429	-0.6174
SOWAHC	-0.6489	6.02651	-8.3107	0.00017	0.00183	0.89651
STK32C	-0.6488	2.4847	-5.9563	0.001	0.00597	-1.1522
TRIM52	-0.6485	2.77492	-4.5138	0.00405	0.01597	-2.7243
NIFK-AS1	-0.6479	-0.3753	-5.4527	0.00159	0.00827	-1.6693
PAX9	-0.6477	-0.8027	-7.746	0.00024	0.00234	0.45339
DNAJC7	-0.6476	4.76468	-8.0418	0.0002	0.00205	0.68883
LRFN3	-0.6475	2.62681	-4.6465	0.00352	0.01449	-2.5675
RPS6KC1	-0.6471	2.69389	-5.4878	0.00154	0.00806	-1.6322
ABTB2	-0.6471	2.42817	-5.9748	0.00099	0.00591	-1.1339
WDCP	-0.647	2.60766	-4.3064	0.00506	0.01876	-2.9742
DRAM1	-0.647	3.04539	-6.4554	0.00066	0.00444	-0.6702
OSTCP4	-0.647	0.87721	-4.7628	0.00312	0.01327	-2.4323
LIX1L	-0.6463	4.16648	-8.1481	0.00018	0.00196	0.7717
TTC26	-0.6455	2.78901	-6.9765	0.00043	0.00336	-0.1965
SFXN3	-0.6454	3.0699	-7.867	0.00022	0.00221	0.55059
MIATNB	-0.645	-0.8874	-6.4189	0.00068	0.00453	-0.7045
NKIRAS1	-0.645	2.29123	-6.4494	0.00066	0.00446	-0.6758
ATP6V1E	-0.645	5.30635	-5.6096	0.00137	0.00752	-1.5047
CRYBB2F	-0.6445	2.22593	-6.5124	0.00063	0.00429	-0.6169
HSPA4L	-0.6444	3.60108	-4.7909	0.00303	0.01301	-2.3999
BBS5	-0.6441	0.82052	-3.7746	0.00925	0.02933	-3.6443
SOX12	-0.6429	5.78476	-7.2161	0.00036	0.00296	0.01196
VPS16	-0.6416	3.67352	-7.4291	0.00031	0.00269	0.19254
SUMF2	-0.6413	5.49257	-4.7783	0.00307	0.01313	-2.4144
RPL38	-0.6411	5.90671	-3.3581	0.01528	0.04284	-4.197
USP51	-0.6408	2.14602	-3.4469	0.0137	0.03962	-4.0772
PSMD12F	-0.6404	4.26058	-4.6879	0.00337	0.01401	-2.5192
FAM229A	-0.6404	2.17528	-3.8485	0.00848	0.02757	-3.5487
COPRS	-0.6397	3.91919	-6.4318	0.00067	0.0045	-0.6923
SPIRE2	-0.6396	1.16994	-3.8226	0.00874	0.02818	-3.5821
SDHAP3	-0.6396	-1.7924	-3.6102	0.01124	0.03403	-3.8597
LRRC37B	-0.6395	0.48855	-3.4208	0.01414	0.04053	-4.1123
YDJC	-0.6392	5.9285	-7.6835	0.00026	0.00241	0.40266
ADRM1	-0.6391	6.31045	-6.888	0.00046	0.00351	-0.2749
TRIM68	-0.6391	1.18959	-5.1489	0.00212	0.01008	-1.9973
NT5C	-0.639	3.34541	-4.583	0.00376	0.01519	-2.6422
PKNOX1	-0.639	4.3367	-4.8881	0.00275	0.01212	-2.2888
NRSN2	-0.6382	3.9804	-6.1107	0.00088	0.00546	-1
ZNF107	-0.638	2.23734	-3.344	0.01555	0.04341	-4.2161
KIF18B	-0.6377	4.54788	-4.9087	0.00269	0.01193	-2.2654
SMG8	-0.6374	3.25738	-7.6591	0.00026	0.00243	0.38274
NNT-AS1	-0.6366	2.29829	-6.5393	0.00061	0.00424	-0.5919
SNX11	-0.6361	2.17778	-6.501	0.00063	0.00432	-0.6276
B3GAT3	-0.6361	4.03042	-8.0657	0.0002	0.00204	0.70752
DESI1	-0.6352	5.08908	-6.7955	0.0005	0.0037	-0.3578

TEPSIN	-0.6349	2.96476	-5.3668	0.00172	0.00871	-1.7608
SNHG9	-0.6346	2.52191	-3.6194	0.01112	0.03375	-3.8475
CCDC117	-0.6345	4.21874	-6.6328	0.00057	0.00404	-0.5057
FAM107B	-0.6329	4.27048	-3.6833	0.0103	0.0318	-3.7634
PSEN2	-0.6327	1.3891	-4.5425	0.00393	0.01563	-2.6901
ALKBH8	-0.6326	1.16766	-5.4313	0.00162	0.00838	-1.692
MYOF	-0.6322	6.97603	-3.9212	0.0078	0.02588	-3.4554
GPC2	-0.6321	1.81753	-5.188	0.00204	0.00981	-1.9543
ARSA	-0.6318	4.09381	-5.1638	0.00209	0.00997	-1.9808
PURA	-0.6316	4.37723	-7.8371	0.00023	0.00223	0.52673
MRPS7	-0.6313	5.40241	-8.6537	0.00013	0.00159	1.15315
PARVB	-0.6313	3.65446	-8.4049	0.00016	0.00177	0.96791
SMARCE	-0.631	3.49135	-6.0587	0.00092	0.00561	-1.0509
SECTM1	-0.6307	0.34995	-5.8435	0.00111	0.00643	-1.2653
RAB11FIF	-0.6302	3.76243	-7.0288	0.00042	0.00328	-0.1505
SELENO(	-0.6295	4.05167	-6.6056	0.00058	0.00409	-0.5307
B4GALT7	-0.629	4.12496	-6.5181	0.00062	0.00429	-0.6116
L3MBTL3	-0.6284	1.32582	-3.204	0.01852	0.04949	-4.4073
PSMB3P2	-0.6284	3.02892	-6.8958	0.00046	0.0035	-0.268
SLC25A1	-0.628	3.94405	-3.9253	0.00776	0.02582	-3.4502
CTIF	-0.6279	4.57556	-5.7034	0.00126	0.00707	-1.4078
PHB1P21	-0.6277	3.25484	-6.2149	0.0008	0.00512	-0.8988
WDR5B-D	-0.6274	-1.9421	-3.9816	0.00728	0.02466	-3.3785
LINC0142	-0.6274	0.04699	-4.3808	0.00467	0.01768	-2.8839
PNISR-AS	-0.6272	-0.119	-6.9515	0.00044	0.00341	-0.2186
TRPM2-A	-0.6271	0.33497	-4.7886	0.00304	0.01303	-2.4026
RILPL2	-0.6267	1.84983	-5.993	0.00097	0.00584	-1.1157
LSM10	-0.6267	3.7821	-6.3562	0.00071	0.0047	-0.7637
CENPP	-0.6266	1.97171	-6.376	0.0007	0.00465	-0.7449
SEC61A2	-0.6262	1.73957	-7.801	0.00023	0.00228	0.49777
REEP2	-0.626	1.55657	-3.2434	0.01763	0.04767	-4.3532
UBE2O	-0.6251	4.51842	-5.9739	0.00099	0.00591	-1.1347
SGCE	-0.6251	4.75263	-6.0689	0.00091	0.00558	-1.0409
PLEKHM1	-0.6248	0.5855	-4.1625	0.00594	0.02116	-3.1515
MSRB2	-0.6247	2.62466	-3.8035	0.00894	0.02862	-3.6067
MRPL12	-0.6247	3.37172	-5.5082	0.00151	0.00798	-1.6107
INO80C	-0.6238	0.64777	-3.3147	0.01612	0.04467	-4.256
SORD	-0.6236	3.1067	-4.7059	0.00331	0.01386	-2.4982
MPHOSP	-0.6234	3.46308	-4.9322	0.00263	0.01171	-2.2388
TOR2A	-0.6232	2.36472	-6.8869	0.00046	0.00351	-0.2759
RMDN3	-0.6227	3.45423	-6.4351	0.00067	0.00449	-0.6892
GTF2IP13	-0.6221	2.35923	-3.5732	0.01175	0.03519	-3.9087
INSIG1-D	-0.622	1.14964	-3.444	0.01375	0.03972	-4.0811
PIGB	-0.6216	1.40875	-7.1335	0.00038	0.00311	-0.0593
RPP25L	-0.6216	3.23789	-4.1041	0.00634	0.02227	-3.2242
ANAPC7	-0.6208	4.40756	-7.1749	0.00037	0.00304	-0.0235
AP2B1	-0.6202	5.82519	-7.9719	0.00021	0.00211	0.63385
RPS24P1	-0.62	0.86879	-3.3077	0.01626	0.04493	-4.2654
CSGALNA	-0.62	-0.1068	-4.5105	0.00406	0.016	-2.7282
PNPLA3	-0.6197	2.73994	-3.5896	0.01152	0.03466	-3.8869
KSR1	-0.6191	2.10001	-7.3196	0.00033	0.0028	0.10024
FAM219A	-0.618	2.91581	-4.9535	0.00257	0.01153	-2.2148
EFTUD2	-0.6175	5.68122	-5.2126	0.00199	0.00964	-1.9275
DYRK3	-0.6167	0.53859	-4.0628	0.00664	0.02309	-3.276
ATP5MC2	-0.6167	4.98406	-5.4634	0.00157	0.00821	-1.658
ZSWIM8	-0.6167	4.34002	-6.3449	0.00072	0.00473	-0.7745
GRINA	-0.6167	7.5193	-7.8594	0.00023	0.00222	0.54453
DYNLT3	-0.6167	3.2836	-5.5268	0.00148	0.00791	-1.5912

SLC12A5	-0.6166	1.04678	-3.5194	0.01254	0.03703	-3.9802
SLC25A1	-0.6164	1.34668	-3.5375	0.01227	0.03636	-3.9561
ADPRM	-0.6161	0.02765	-7.7203	0.00025	0.00237	0.43259
MAP3K3	-0.6157	2.96447	-6.6431	0.00056	0.00402	-0.4962
SKA2	-0.6154	3.87965	-6.5529	0.00061	0.00421	-0.5793
PEX26	-0.6153	4.97817	-7.0345	0.00041	0.00327	-0.1455
FAM151B	-0.6152	0.52216	-3.2053	0.01849	0.04944	-4.4054
NCAPH2	-0.6152	4.87166	-5.4818	0.00154	0.00809	-1.6386
FAM241B	-0.6152	3.54207	-4.3489	0.00483	0.01812	-2.9224
TMEM135	-0.6145	2.90861	-3.2323	0.01787	0.04822	-4.3684
OSBPL1A	-0.6143	3.64025	-7.9842	0.00021	0.0021	0.64358
WWC3	-0.6141	4.19881	-7.3823	0.00032	0.00273	0.15325
TP53I3	-0.614	2.61175	-3.7136	0.00994	0.03091	-3.7237
FAM114A	-0.6134	2.6568	-4.9637	0.00255	0.01144	-2.2034
IFNAR2	-0.6133	2.69971	-7.1001	0.00039	0.00315	-0.0883
PLD3	-0.6133	6.56345	-5.1231	0.00218	0.01025	-2.0256
TMEM59L	-0.6131	-0.0954	-3.4108	0.01432	0.04084	-4.1259
VMA21	-0.6131	4.77865	-5.9368	0.00102	0.00605	-1.1716
CLMN	-0.613	1.68142	-4.7501	0.00316	0.01337	-2.447
ZC3H10	-0.6129	1.79964	-4.6264	0.0036	0.0147	-2.5911
UNKL	-0.6119	2.88904	-3.9785	0.0073	0.02471	-3.3825
MAN1B1	-0.6115	3.74097	-5.7737	0.00118	0.00674	-1.336
ZNF91	-0.6114	1.40408	-3.5166	0.01258	0.03714	-3.984
ARHGDIA	-0.6111	7.59207	-4.4422	0.00437	0.01685	-2.8098
ATP5MC1	-0.611	5.26412	-4.7351	0.00321	0.01353	-2.4643
IMPACT	-0.6107	2.74029	-4.7726	0.00309	0.01319	-2.421
SGPP1	-0.61	2.76784	-4.6393	0.00355	0.01456	-2.576
TSPAN10	-0.6099	0.9179	-4.4212	0.00447	0.01713	-2.8351
C19orf33	-0.6097	1.12308	-3.3288	0.01584	0.04406	-4.2368
EOGT	-0.6091	1.79471	-4.5589	0.00386	0.01543	-2.6706
C8orf76	-0.609	-1.3277	-6.7573	0.00051	0.00378	-0.3922
CDKL2	-0.6087	-2.6046	-3.8914	0.00807	0.02654	-3.4935
RRAS2P1	-0.6085	5.85998	-7.3419	0.00033	0.00277	0.11911
LAGE3	-0.6085	5.06132	-7.4738	0.0003	0.00264	0.22984
TCTN1	-0.6084	2.39828	-6.4353	0.00067	0.00449	-0.689
UBTF	-0.6074	5.25949	-3.5502	0.01208	0.03594	-3.9391
IQSEC2	-0.6067	2.52884	-3.7311	0.00973	0.03046	-3.7008
RBM19	-0.6063	3.32846	-6.6535	0.00056	0.00399	-0.4867
PNMA8A	-0.6063	2.82415	-5.79	0.00116	0.00666	-1.3195
MYO1B	-0.6062	5.2115	-4.4453	0.00436	0.01681	-2.8061
TAF9B	-0.606	3.9662	-5.6529	0.00132	0.0073	-1.4599
NFE2L1	-0.6054	5.77021	-5.1811	0.00206	0.00985	-1.9618
ATP5PD	-0.6048	4.53401	-5.2176	0.00198	0.0096	-1.922
CDK12	-0.6046	4.3927	-3.5456	0.01215	0.03609	-3.9453
EXOG	-0.6042	2.67801	-4.3136	0.00502	0.01868	-2.9655
C8orf58	-0.6041	1.46464	-4.8514	0.00285	0.01245	-2.3307
MRPL27	-0.6037	3.60561	-6.4472	0.00066	0.00446	-0.6779
C1orf56	-0.6037	2.00965	-4.9213	0.00266	0.01182	-2.2511
DCTN6-D	-0.6035	-0.0933	-3.7131	0.00994	0.03092	-3.7244
WASHC4	-0.6033	4.69288	-7.4279	0.00031	0.00269	0.19151
RAB3IL1	-0.6032	1.78395	-3.2859	0.01671	0.0458	-4.2952
L3HYPDH	-0.6025	1.77062	-4.6301	0.00358	0.01466	-2.5867
MAP3K11	-0.6025	5.09978	-6.0132	0.00096	0.00578	-1.0958
PHB1P11	-0.602	1.33684	-3.9899	0.00721	0.02447	-3.3679
KCTD21-/	-0.6009	0.04527	-4.6985	0.00334	0.01391	-2.5069
NCK1	-0.6005	3.40659	-5.6851	0.00128	0.00715	-1.4267
PGGT1BI	-0.6002	0.67376	-4.2827	0.0052	0.01916	-3.0032
FAAP24	-0.6	2.04888	-4.7569	0.00314	0.01332	-2.4391

ATP6V0A	-0.5997	3.30722	-3.5077	0.01272	0.03745	-3.9958
SCLY	-0.5992	-0.822	-3.2434	0.01763	0.04767	-4.3532
PARVG	-0.5991	0.50903	-3.2994	0.01643	0.04524	-4.2768
TERF2IP	-0.5982	3.57021	-8.1446	0.00018	0.00196	0.76892
HELZ	-0.598	3.50227	-7.4302	0.00031	0.00269	0.19342
SEPTIN7I	-0.5972	-1.1816	-5.1717	0.00207	0.00992	-1.9722
TRDMT1	-0.5971	1.57682	-4.6725	0.00343	0.0142	-2.5372
COMMD5	-0.5965	3.58534	-7.4757	0.0003	0.00264	0.23144
TWF2	-0.596	4.35246	-7.1857	0.00037	0.00302	-0.0142
DSTYK	-0.5958	2.56606	-7.6714	0.00026	0.00242	0.39281
NME2P1	-0.5958	7.27653	-4.6029	0.00369	0.01497	-2.6187
NBR1	-0.5956	4.36515	-5.0133	0.00242	0.01103	-2.1476
ZNF780B	-0.5956	1.97238	-5.1507	0.00212	0.01006	-1.9953
ZNF566-A	-0.5953	-0.3309	-4.4574	0.0043	0.01667	-2.7916
SRP68	-0.5934	4.71809	-7.381	0.00032	0.00273	0.1521
DCP1B	-0.5933	0.24544	-3.5724	0.01176	0.03521	-3.9097
ZNF280C	-0.5928	2.16818	-5.4412	0.0016	0.00834	-1.6815
GLMP	-0.5927	5.32106	-6.6472	0.00056	0.00401	-0.4924
ACTR3C	-0.5925	-0.1209	-3.9121	0.00788	0.02607	-3.4671
HSP90AA	-0.5923	2.99237	-4.4307	0.00443	0.01702	-2.8236
FAM86C1	-0.592	0.85218	-3.6681	0.01049	0.03228	-3.7834
SLC22A4	-0.5919	-0.096	-4.2639	0.00531	0.01945	-3.0262
FRMD8	-0.5918	3.33872	-4.2811	0.00521	0.01918	-3.0052
MATCAP1	-0.5916	1.8573	-6.1124	0.00088	0.00546	-0.9983
BTN3A2	-0.5914	2.60067	-5.3758	0.00171	0.00867	-1.7511
COL16A1	-0.5913	-0.0236	-3.3307	0.01581	0.04397	-4.2343
IPO13	-0.5905	4.07391	-3.5712	0.01178	0.03524	-3.9112
IFT46	-0.5901	2.96437	-5.1214	0.00218	0.01026	-2.0275
GCFC2	-0.5887	2.45179	-5.283	0.00186	0.0092	-1.8509
PDE7B	-0.5884	1.52507	-3.2907	0.01661	0.04558	-4.2887
JMJD6	-0.5878	4.24501	-4.7477	0.00317	0.01339	-2.4497
UROD	-0.5865	5.83744	-6.9015	0.00046	0.00349	-0.2629
TUBB6	-0.5864	5.6534	-5.804	0.00115	0.00661	-1.3052
TBC1D13	-0.5863	3.23277	-4.6014	0.00369	0.01498	-2.6205
UCK1	-0.5861	3.62048	-5.424	0.00163	0.00841	-1.6997
MIER2	-0.5856	2.22965	-6.5825	0.00059	0.00415	-0.5519
MRPL38	-0.5855	-0.5115	-6.3616	0.00071	0.00469	-0.7586
LAMP2	-0.5854	5.9502	-5.7323	0.00123	0.00692	-1.3783
DHX8	-0.5852	3.97542	-4.0159	0.007	0.02396	-3.335
PRELID1I	-0.5851	3.67014	-5.769	0.00119	0.00675	-1.3408
UBE2Z	-0.5848	5.30256	-8.1455	0.00018	0.00196	0.76961
TCIRG1	-0.5843	2.61933	-3.8106	0.00887	0.02844	-3.5976
DYNLT3P	-0.5843	0.32486	-7.8307	0.00023	0.00224	0.52159
LRIF1	-0.5836	2.87167	-6.1691	0.00084	0.00527	-0.9432
NMD3P2	-0.5827	1.18418	-3.8289	0.00868	0.02801	-3.5739
UFL1	-0.5822	3.7326	-4.2842	0.00519	0.01914	-3.0013
PHF20L1	-0.582	5.4468	-5.9445	0.00101	0.00602	-1.164
CCDC40	-0.5815	1.02496	-4.8113	0.00297	0.01283	-2.3765
TACO1	-0.5808	2.98914	-6.6701	0.00055	0.00396	-0.4715
CDC27	-0.5808	4.71595	-6.8293	0.00049	0.00363	-0.3274
MT1X	-0.5804	1.15305	-3.577	0.0117	0.03507	-3.9035
ALAD	-0.5802	2.88647	-5.7309	0.00123	0.00693	-1.3797
TMEM106	-0.58	-0.1227	-4.2784	0.00522	0.01922	-3.0085
HENMT1	-0.5797	2.02587	-6.3063	0.00074	0.00484	-0.8112
SMN1	-0.5795	-0.3388	-5.668	0.0013	0.00722	-1.4443
NDUFA12	-0.5791	3.87423	-4.4137	0.00451	0.01722	-2.8441
DCAF7	-0.579	5.07671	-7.0008	0.00042	0.00333	-0.1751
GREB1L	-0.5788	2.91541	-5.988	0.00098	0.00585	-1.1207

BLOC1S1	-0.5785	1.99347	-5.2345	0.00195	0.0095	-1.9036
COILP1	-0.5783	0.62237	-6.8338	0.00048	0.00363	-0.3234
SLC25A4	-0.5777	2.00815	-3.4293	0.014	0.04026	-4.1009
TMEM45A	-0.5776	2.19805	-3.6884	0.01024	0.03167	-3.7567
GSTA4	-0.5776	1.59202	-4.8978	0.00272	0.01203	-2.2778
DEPDC1B	-0.5772	4.00405	-4.4196	0.00448	0.01715	-2.837
LSM12	-0.5771	1.45934	-7.4222	0.00031	0.00269	0.18676
MED15	-0.5771	5.39451	-6.6641	0.00055	0.00397	-0.477
PEX11G	-0.576	-2.0288	-3.2135	0.0183	0.0491	-4.3943
GAD1	-0.5755	1.44063	-3.3062	0.0163	0.04497	-4.2676
SNAI3-AS	-0.5753	-0.1697	-3.5435	0.01218	0.03615	-3.948
GUCD1	-0.575	5.64089	-5.202	0.00201	0.00972	-1.939
MFSD1	-0.5743	3.83395	-6.4369	0.00067	0.00449	-0.6876
LAMC1	-0.5739	7.53578	-5.0737	0.00228	0.01058	-2.0803
SPATS2	-0.5734	4.70496	-4.656	0.00349	0.01438	-2.5564
C19orf44	-0.5723	1.58306	-5.4568	0.00158	0.00825	-1.6649
HEXA	-0.572	3.17369	-7.1383	0.00038	0.0031	-0.0551
QPRT	-0.5716	6.1697	-6.8537	0.00048	0.00359	-0.3055
TSPAN4	-0.5716	3.92265	-5.987	0.00098	0.00586	-1.1218
JMJD4	-0.5715	3.6809	-3.7504	0.00952	0.02994	-3.6757
NEIL3	-0.5715	2.04286	-4.6904	0.00336	0.014	-2.5163
MT1F	-0.5704	3.48047	-3.942	0.00762	0.02548	-3.4288
TYMS	-0.5702	6.1604	-3.9555	0.0075	0.0252	-3.4117
TMEM126	-0.5699	2.77771	-7.2524	0.00035	0.00291	0.043
JRK	-0.5697	3.57367	-3.6206	0.0111	0.03372	-3.8458
SLC23A2	-0.5688	3.44512	-5.0828	0.00226	0.01051	-2.0702
UFD1	-0.5681	4.06168	-5.5911	0.00139	0.00759	-1.524
CASP10	-0.5673	1.95134	-3.6707	0.01046	0.03219	-3.78
KPNB1	-0.5668	7.09658	-5.6181	0.00136	0.00748	-1.4959
TYMSOS	-0.5668	-0.4038	-4.7371	0.00321	0.01351	-2.462
NPLOC4	-0.5668	6.36951	-7.7047	0.00025	0.00238	0.41991
SLC25A4	-0.5667	3.10212	-6.6564	0.00056	0.00399	-0.484
CYBC1	-0.566	5.179	-6.5724	0.0006	0.00417	-0.5613
PODXL2	-0.5656	4.01762	-4.6289	0.00359	0.01467	-2.5882
NLE1	-0.5651	3.07462	-3.8266	0.0087	0.02808	-3.5769
GNPDA2	-0.5651	2.86303	-5.1111	0.0022	0.01032	-2.0389
ARHGAP	-0.5648	3.85066	-3.8897	0.00809	0.02656	-3.4958
TLCD5	-0.5645	2.0091	-5.8109	0.00114	0.00659	-1.2983
ZNF652	-0.5645	2.76441	-3.4816	0.01313	0.03838	-4.0307
PRPS1P2	-0.5645	1.3756	-3.7784	0.00921	0.02925	-3.6394
SLC66A1	-0.5644	3.00049	-6.7555	0.00051	0.00378	-0.3939
ZNF70	-0.5641	2.0414	-6.1095	0.00088	0.00546	-1.0011
EME1	-0.5638	2.60196	-4.2417	0.00544	0.01979	-3.0535
AMMECR	-0.5637	4.01077	-5.9942	0.00097	0.00584	-1.1146
TXNDC15	-0.5634	3.34896	-3.444	0.01375	0.03972	-4.0811
IQCG	-0.563	1.53457	-5.6309	0.00134	0.00741	-1.4827
PHB1P10	-0.5629	2.48395	-6.4403	0.00066	0.00448	-0.6844
C19orf25	-0.5627	1.77563	-4.4625	0.00428	0.01662	-2.7855
NSF	-0.5626	3.98461	-5.5915	0.00139	0.00759	-1.5235
FAM131A	-0.5621	1.96229	-3.7403	0.00963	0.03019	-3.6888
GNPDA1	-0.5621	5.55955	-5.26	0.0019	0.00935	-1.8758
ACAA2P1	-0.5618	3.35378	-5.0446	0.00235	0.01082	-2.1127
SLC35B1	-0.5618	4.37768	-6.6783	0.00055	0.00394	-0.464
FGF18	-0.5617	4.48011	-4.2644	0.0053	0.01944	-3.0256
SLC16A1	-0.561	1.5449	-5.797	0.00116	0.00664	-1.3123
C1GALT1	-0.5606	0.96075	-3.7925	0.00906	0.0289	-3.6211
TERF1	-0.5606	3.64282	-3.9491	0.00755	0.02533	-3.4199
CASP7	-0.5603	3.24613	-6.3941	0.00069	0.00459	-0.7278

MIDEAS-/	-0.56	0.51339	-4.0304	0.00689	0.02368	-3.3168
WAS	-0.5595	-2.0434	-3.8456	0.00851	0.02762	-3.5525
PYGO1	-0.5594	1.81502	-3.4231	0.0141	0.04045	-4.1093
CMTM8	-0.5593	1.6045	-3.428	0.01402	0.0403	-4.1027
ADGRL2	-0.5588	7.15882	-5.1043	0.00222	0.01037	-2.0465
TBX3	-0.5588	0.22144	-6.938	0.00045	0.00342	-0.2305
VAMP1	-0.5579	2.21869	-3.9735	0.00735	0.02481	-3.3888
TRIM65	-0.5577	2.55834	-4.2523	0.00537	0.01964	-3.0406
DPM2	-0.5567	4.36734	-4.6367	0.00356	0.0146	-2.5791
ENTPD5	-0.556	2.23711	-3.8858	0.00812	0.02665	-3.5007
TCEAL1	-0.5556	3.0625	-3.9217	0.0078	0.02588	-3.4548
PXN-AS1	-0.5556	0.85941	-3.8242	0.00873	0.02814	-3.58
NCKAP5L	-0.5555	4.17819	-6.4336	0.00067	0.00449	-0.6906
TOLLIP	-0.5555	3.40098	-3.9387	0.00765	0.02557	-3.4331
ACO1	-0.5555	4.3833	-3.5542	0.01202	0.03579	-3.9339
LINC0088	-0.5554	-0.7447	-4.251	0.00538	0.01966	-3.0421
SSNA1	-0.5551	5.11074	-5.4012	0.00167	0.00853	-1.724
RPTOR	-0.555	4.85294	-5.5928	0.00139	0.00759	-1.5222
TRPC1	-0.555	1.79695	-4.5577	0.00387	0.01544	-2.6721
SLC25A1	-0.5543	6.65793	-4.7878	0.00304	0.01303	-2.4036
FAAP100	-0.5542	4.47025	-4.1147	0.00626	0.0221	-3.211
TMEM92	-0.554	1.68958	-3.834	0.00863	0.0279	-3.5674
MIF4GD	-0.554	1.91524	-4.0982	0.00638	0.02239	-3.2316
THOC1	-0.5538	3.46491	-5.628	0.00135	0.00742	-1.4856
CENPX	-0.5536	5.56067	-4.3489	0.00483	0.01812	-2.9225
GPS1	-0.5536	6.34431	-7.4323	0.00031	0.00269	0.19522
UBA6	-0.5528	4.64273	-4.2722	0.00526	0.0193	-3.0161
NOL9	-0.5528	3.54133	-3.5474	0.01212	0.03604	-3.9428
RBM33-D	-0.5527	1.298	-5.1069	0.00221	0.01035	-2.0435
UTP6	-0.5524	3.77664	-6.1153	0.00087	0.00545	-0.9955
POP5	-0.5523	4.48561	-6.8017	0.0005	0.00369	-0.3521
APOBEC3	-0.5521	3.86012	-3.2855	0.01672	0.04581	-4.2956
TLE3	-0.5517	3.45998	-7.4782	0.0003	0.00264	0.23354
HDHD2	-0.5516	2.55766	-6.7196	0.00053	0.00385	-0.4264
FAM174C	-0.5515	2.78762	-3.8684	0.00829	0.02708	-3.5231
ARL16	-0.5514	5.131	-5.544	0.00146	0.00781	-1.5732
SNHG8	-0.551	5.42082	-3.372	0.01502	0.04234	-4.1783
BEX3	-0.5508	7.48032	-6.9006	0.00046	0.00349	-0.2637
GSTO1	-0.5507	3.68911	-3.9124	0.00788	0.02606	-3.4667
CCDC148	-0.5504	-2.2805	-4.6409	0.00354	0.01454	-2.574
ATP5PDF	-0.55	5.54215	-4.646	0.00352	0.01449	-2.5682
NUDT1	-0.5499	2.6305	-4.4827	0.00419	0.01635	-2.7613
AP5S1	-0.5499	2.97603	-3.2902	0.01662	0.04558	-4.2893
ATPAF2	-0.5497	1.29461	-3.8863	0.00812	0.02664	-3.5
ZNF566	-0.5493	1.60049	-4.4186	0.00448	0.01716	-2.8382
MT1XP1	-0.548	0.8965	-4.723	0.00325	0.01367	-2.4784
C11orf71	-0.5474	2.04301	-3.5418	0.0122	0.0362	-3.9504
GNA13	-0.546	4.35608	-5.5213	0.00149	0.00793	-1.5969
ELP6	-0.5449	2.82867	-7.4746	0.0003	0.00264	0.23056
ZNF426	-0.544	1.51879	-4.0434	0.00678	0.0234	-3.3003
TUBD1	-0.5433	1.51054	-5.5606	0.00143	0.00774	-1.5558
NCBP2	-0.5432	5.04172	-6.3721	0.0007	0.00466	-0.7486
MRPL17	-0.5423	5.37582	-6.2513	0.00078	0.005	-0.8638
AK1	-0.5422	-0.0098	-4.4249	0.00445	0.0171	-2.8306
CCDC34	-0.5419	2.98899	-4.0593	0.00666	0.02315	-3.2804
PSTK	-0.5416	0.91678	-3.7991	0.00899	0.02873	-3.6124
MFSD5	-0.5415	4.27257	-7.3365	0.00033	0.00278	0.11455
NOL11	-0.5414	3.80176	-3.2952	0.01652	0.04541	-4.2825

TMCO1	-0.5414	4.91377	-5.56	0.00144	0.00774	-1.5565
NINL	-0.5409	2.3315	-4.8463	0.00287	0.01249	-2.3364
ASB8	-0.5402	3.3535	-6.0631	0.00091	0.00559	-1.0466
SEC31B	-0.54	0.53459	-3.3452	0.01552	0.04338	-4.2145
RUSC2	-0.5399	2.97184	-4.3238	0.00497	0.01852	-2.953
NT5DC1	-0.5399	2.92154	-5.1398	0.00214	0.01015	-2.0072
NFS1	-0.5394	3.47599	-4.5287	0.00399	0.01582	-2.7065
PWWP3A	-0.5391	1.95657	-5.4303	0.00162	0.00838	-1.693
NMD3P1	-0.5388	4.39092	-5.6543	0.00132	0.00729	-1.4585
PWP1	-0.5385	4.72237	-5.4978	0.00152	0.00803	-1.6216
F2RL3	-0.5381	0.53584	-5.1146	0.00219	0.0103	-2.0351
KANSL1	-0.5379	3.53481	-6.9439	0.00044	0.00342	-0.2253
NUP50	-0.5375	5.33736	-5.7457	0.00121	0.00685	-1.3646
VEZT	-0.5374	3.35715	-4.107	0.00632	0.02223	-3.2206
WFS1	-0.5371	3.69542	-5.1959	0.00203	0.00976	-1.9457
SH3BP5-/	-0.537	0.33711	-4.4291	0.00443	0.01704	-2.8255
LIMS1	-0.537	5.71192	-6.371	0.0007	0.00466	-0.7497
RNF138	-0.5363	3.31618	-5.5117	0.0015	0.00796	-1.6071
MPV17	-0.536	3.88633	-4.0078	0.00706	0.02411	-3.3454
PINX1	-0.5359	0.85867	-4.9421	0.0026	0.01163	-2.2277
DUXAP8	-0.5359	1.97107	-3.431	0.01397	0.0402	-4.0985
GGA3	-0.5357	3.60655	-6.2226	0.0008	0.0051	-0.8914
HIGD2AP	-0.5354	1.16279	-3.8099	0.00887	0.02845	-3.5985
FAM229B	-0.5354	1.51644	-4.2427	0.00543	0.01977	-3.0523
NCALD	-0.5353	1.37882	-3.2904	0.01662	0.04558	-4.289
TIPARP	-0.5352	3.26163	-3.6556	0.01065	0.03264	-3.7997
SNX17	-0.5351	5.56754	-5.355	0.00174	0.00877	-1.7734
PPP3CB-	-0.535	0.10973	-3.9326	0.0077	0.02568	-3.4408
ZFYVE16	-0.5346	3.6859	-5.4473	0.00159	0.0083	-1.6751
KRCC1	-0.5343	3.59188	-5.0455	0.00235	0.01082	-2.1117
SUOX	-0.5341	3.74779	-3.9133	0.00787	0.02604	-3.4655
APPL2	-0.5341	2.91233	-5.8753	0.00108	0.00631	-1.2332
TMEM177	-0.534	2.37449	-5.2338	0.00195	0.0095	-1.9043
ACAA2	-0.5336	2.19946	-7.2284	0.00036	0.00294	0.02247
ACACB	-0.5335	1.57888	-3.3969	0.01456	0.04138	-4.1445
CAMK2N1	-0.5334	4.62986	-3.5942	0.01146	0.03452	-3.8808
SNHG7	-0.5334	3.17032	-4.3457	0.00485	0.01817	-2.9264
RNF216P	-0.5329	1.24405	-4.1863	0.00578	0.02074	-3.1219
DEPDC7	-0.5328	1.45492	-5.6487	0.00132	0.00732	-1.4643
NPHP3	-0.5322	1.3944	-5.9068	0.00105	0.00618	-1.2017
OGFOD3	-0.532	3.89762	-5.2783	0.00187	0.00923	-1.8561
LAMB1	-0.5319	7.46431	-4.3581	0.00479	0.01799	-2.9113
METTL2A	-0.5309	1.84155	-6.4465	0.00066	0.00446	-0.6785
EHD3	-0.5307	2.11857	-3.2993	0.01644	0.04524	-4.2769
PHLDB1	-0.5306	5.46756	-3.751	0.00951	0.02992	-3.6749
COG2	-0.5306	2.4627	-5.6294	0.00135	0.00741	-1.4842
SNRPD3	-0.5305	5.50093	-6.6462	0.00056	0.00401	-0.4933
CTBP1	-0.5304	4.96789	-5.7536	0.0012	0.00682	-1.3565
PPIAP22	-0.5304	9.36523	-5.7203	0.00124	0.00698	-1.3905
ZFP28	-0.5301	1.59052	-3.2046	0.01851	0.04947	-4.4065
NDUFC1	-0.5301	3.48249	-4.5333	0.00397	0.01574	-2.701
MRGBP	-0.53	5.2536	-6.0851	0.0009	0.00554	-1.0251
GALNT10	-0.528	6.34235	-3.2994	0.01643	0.04524	-4.2767
PSMC4	-0.5277	6.54231	-5.1388	0.00214	0.01016	-2.0083
CAMSAP2	-0.5271	5.11626	-4.088	0.00645	0.0226	-3.2444
RASA3	-0.527	3.21096	-4.4381	0.00439	0.01692	-2.8148
PSAP	-0.5268	7.76646	-4.281	0.00521	0.01918	-3.0054
COL27A1	-0.5267	2.6177	-3.3088	0.01624	0.04488	-4.264

ACOT4	-0.5266	0.72975	-4.0069	0.00707	0.02412	-3.3464
FBXL20	-0.5264	1.69314	-3.4433	0.01376	0.03974	-4.0821
SLC2A4R	-0.5261	6.65121	-3.997	0.00715	0.02433	-3.359
CERS5	-0.5259	3.50558	-4.1293	0.00616	0.02182	-3.1928
GSTM2	-0.5258	0.38268	-3.569	0.01181	0.03531	-3.9142
ERP29P1	-0.5256	3.09472	-5.3046	0.00183	0.00906	-1.8276
FAM217B	-0.5248	3.03808	-4.2445	0.00542	0.01976	-3.0501
LARP4	-0.5247	5.02346	-7.0254	0.00042	0.00329	-0.1535
C18orf32	-0.5237	1.22615	-3.9064	0.00793	0.02621	-3.4743
ATXN7L1	-0.5236	1.77544	-5.4886	0.00153	0.00806	-1.6313
MRPL41	-0.5234	5.11779	-5.2918	0.00185	0.00915	-1.8415
RETREG3	-0.5229	3.31585	-6.7824	0.0005	0.00372	-0.3695
ZNF197	-0.5229	1.13574	-4.7029	0.00332	0.01388	-2.5017
RMDN1	-0.5227	3.44868	-5.8621	0.00109	0.00636	-1.2465
MTHFS	-0.5224	-0.8398	-3.4703	0.01331	0.0388	-4.0458
DNAJC9-A	-0.5218	1.35683	-5.6679	0.0013	0.00722	-1.4445
PPP1R14	-0.5215	0.60739	-5.0982	0.00223	0.01041	-2.0531
DDX55	-0.5215	3.60472	-5.314	0.00181	0.00901	-1.8175
C15orf40	-0.5214	1.7104	-5.2659	0.00189	0.00932	-1.8694
ZBTB17	-0.5214	2.95879	-6.3687	0.00071	0.00467	-0.7518
ACSS1	-0.5208	2.35382	-6.408	0.00068	0.00456	-0.7147
SSTR2	-0.5201	1.14098	-3.2426	0.01764	0.04769	-4.3543
KCTD7	-0.5201	1.59624	-4.3575	0.00479	0.018	-2.912
LZTS3	-0.5197	4.77539	-4.6465	0.00352	0.01449	-2.5676
ZNF503	-0.5195	5.50624	-4.4238	0.00446	0.01711	-2.8319
POLR2H	-0.519	5.10425	-4.5424	0.00393	0.01563	-2.6903
KLHL14	-0.5188	3.24456	-3.7603	0.00941	0.0297	-3.6628
TPGS1	-0.5187	1.81381	-5.5491	0.00145	0.0078	-1.5678
ATP7A	-0.5186	1.86841	-4.9839	0.0025	0.01127	-2.1806
VAV2	-0.5186	3.37372	-4.0506	0.00673	0.0233	-3.2914
PI4KAP2	-0.5183	0.61642	-4.8444	0.00287	0.01251	-2.3386
TRMT112	-0.5181	4.77344	-5.6167	0.00136	0.00748	-1.4974
STAG3L3	-0.5179	0.28704	-3.8817	0.00816	0.02675	-3.506
ABCD4	-0.5169	3.4991	-5.2549	0.00191	0.00938	-1.8814
WDR82	-0.5166	6.02945	-4.4665	0.00426	0.01657	-2.7806
REX1BD	-0.5165	4.92345	-6.0642	0.00091	0.00559	-1.0455
ALDH7A1	-0.5165	6.52515	-5.1277	0.00217	0.01022	-2.0206
KIAA1328	-0.5163	0.62171	-5.045	0.00235	0.01082	-2.1123
BAG3	-0.5163	4.49243	-3.737	0.00967	0.03029	-3.6932
DIABLO	-0.5158	0.72682	-4.3338	0.00491	0.01837	-2.9408
ZNF264	-0.5155	2.77151	-3.4139	0.01426	0.04076	-4.1216
HIBCH	-0.5153	2.72094	-3.734	0.0097	0.03037	-3.6971
NT5DC1P	-0.5152	2.98871	-3.2246	0.01805	0.04855	-4.379
TNIP2	-0.5146	3.28565	-5.4049	0.00166	0.00851	-1.7201
COQ2	-0.5146	2.66754	-4.9798	0.00251	0.0113	-2.1852
FUT4	-0.5143	3.29419	-4.4746	0.00422	0.01645	-2.7709
GINM1	-0.5142	3.22171	-4.693	0.00336	0.01397	-2.5133
LPGAT1	-0.5135	3.47651	-5.0495	0.00234	0.01079	-2.1073
RCCD1	-0.513	3.13739	-5.4349	0.00161	0.00837	-1.6882
IMPA1	-0.5129	3.08826	-5.4661	0.00157	0.00819	-1.6551
DNAJB4	-0.5126	3.03956	-6.2543	0.00078	0.00499	-0.8609
ZNF503-A	-0.5119	1.72918	-4.3203	0.00499	0.01857	-2.9572
CCDC159	-0.5117	0.47535	-6.6077	0.00058	0.00409	-0.5287
CPE	-0.5115	2.74862	-4.0218	0.00695	0.02385	-3.3276
SFR1	-0.5111	1.67432	-6.103	0.00088	0.00548	-1.0075
SLC30A4	-0.5106	1.20096	-4.5481	0.00391	0.01557	-2.6835
UBE2L4	-0.5106	1.10698	-3.458	0.01351	0.03924	-4.0623
APRT	-0.5104	5.63806	-4.8907	0.00274	0.0121	-2.2859

STUB1	-0.5102	5.44735	-5.9082	0.00105	0.00617	-1.2003
SGTB	-0.5101	2.00494	-4.4748	0.00422	0.01645	-2.7708
MTRES1	-0.5098	1.45112	-5.7633	0.00119	0.00677	-1.3466
ABL1	-0.5094	4.56223	-4.1504	0.00602	0.0214	-3.1665
ZMYM6	-0.5091	2.19546	-4.9758	0.00252	0.01133	-2.1897
KIF7	-0.5086	0.74469	-3.9976	0.00715	0.02432	-3.3582
EID2B	-0.5086	1.91579	-6.8033	0.0005	0.00369	-0.3507
NME1	-0.5083	4.47389	-4.5649	0.00384	0.01539	-2.6636
RPS6KB1	-0.5079	3.0626	-4.047	0.00676	0.02334	-3.2959
BRF1	-0.5078	3.22532	-3.2814	0.01681	0.04597	-4.3013
GALNS	-0.5077	3.04161	-5.039	0.00236	0.01086	-2.1189
SIRT4	-0.5073	-1.0309	-3.7995	0.00898	0.02872	-3.6119
NAPG	-0.5066	3.183	-3.602	0.01135	0.03429	-3.8704
YBEY	-0.5065	2.13706	-3.5401	0.01223	0.03626	-3.9525
LSG1	-0.5063	4.39708	-5.0219	0.0024	0.01098	-2.1381
HSP90AA	-0.5063	8.6569	-3.9598	0.00746	0.0251	-3.4062
NR3C1	-0.5062	4.42105	-3.7435	0.00959	0.03011	-3.6847
TRIQK	-0.5062	1.40289	-4.2348	0.00548	0.01991	-3.0621
CSGALNA	-0.506	2.6224	-4.2739	0.00525	0.01928	-3.014
OGFR	-0.5059	4.29533	-4.2558	0.00535	0.01958	-3.0362
USF1	-0.5059	4.68905	-6.3944	0.00069	0.00459	-0.7275
KRT10-AS	-0.5053	2.32909	-3.7573	0.00944	0.02976	-3.6667
MDM1	-0.5048	1.85265	-3.6811	0.01033	0.03188	-3.7663
PAXX	-0.5044	4.59485	-5.5587	0.00144	0.00774	-1.5577
TMEM138	-0.5043	4.20133	-5.5966	0.00139	0.00758	-1.5182
ANAPC5	-0.5041	5.3593	-6.0259	0.00095	0.00573	-1.0833
MRPL28	-0.504	4.8732	-4.5574	0.00387	0.01544	-2.6724
EZH1	-0.5031	2.36844	-3.7242	0.00981	0.03064	-3.7099
SAMM50	-0.5031	4.45536	-6.0573	0.00092	0.00561	-1.0523
CRELD2	-0.5028	4.31046	-5.0026	0.00245	0.01112	-2.1596
SLC39A6	-0.5027	4.74445	-4.6985	0.00334	0.01391	-2.5069
PPIA	-0.5023	6.3798	-5.023	0.0024	0.01098	-2.1368
ARMC7	-0.5008	2.07384	-5.501	0.00152	0.00801	-1.6183
NUP85	-0.5005	4.15236	-6.1492	0.00085	0.00534	-0.9625
MIEF1	0.50032	4.44185	3.71567	0.00991	0.03086	-3.721
TAP2	0.50048	3.31387	3.63542	0.01091	0.03323	-3.8263
NAAA	0.501	1.62692	3.81996	0.00877	0.02824	-3.5855
ZBTB45	0.50113	2.83098	3.78497	0.00914	0.0291	-3.6308
SIRPAP1	0.50169	0.80957	4.10604	0.00632	0.02225	-3.2218
PIP4K2C	0.50203	4.99829	5.0936	0.00224	0.01044	-2.0583
WDTC1	0.50221	4.17941	4.95968	0.00256	0.01147	-2.2078
NSD3	0.50235	4.29676	5.48394	0.00154	0.00809	-1.6363
CREB3L4	0.50256	2.38285	5.51177	0.0015	0.00796	-1.607
UBE2H	0.50358	6.49009	5.12585	0.00217	0.01024	-2.0226
SLC39A1	0.50374	3.88069	5.69623	0.00127	0.0071	-1.4153
WDFY1	0.50375	2.86507	5.00074	0.00245	0.01114	-2.1617
DLGAP5	0.50409	4.98296	4.43887	0.00439	0.01691	-2.8138
FEM1C	0.50417	4.04512	6.32184	0.00073	0.0048	-0.7964
KLRA1P	0.50423	-0.673	4.85503	0.00284	0.01242	-2.3265
TRAF3IP2	0.50424	2.33091	4.32606	0.00496	0.01848	-2.9503
VPS4B	0.50443	2.54817	6.25624	0.00078	0.00498	-0.8591
FNBP1L	0.5049	4.48104	5.90614	0.00105	0.00618	-1.2023
DFFB	0.50605	0.64839	4.03475	0.00685	0.02359	-3.3113
NBEAL1	0.507	2.19686	5.35154	0.00175	0.00878	-1.7771
COTL1	0.50728	6.52261	3.57622	0.01171	0.03509	-3.9046
RPL12P1	0.50743	0.9621	4.07814	0.00652	0.02279	-3.2567
TRAM1L1	0.50807	0.31009	4.21676	0.00559	0.02022	-3.0843
STK38L	0.5082	2.97225	4.78934	0.00304	0.01302	-2.4017

DCAF4	0.50843	2.50605	4.58529	0.00376	0.01517	-2.6395
COMMD7	0.5086	4.32903	3.96858	0.00739	0.02492	-3.3951
ATIC	0.50888	5.08539	5.31181	0.00181	0.00902	-1.8198
ZNRF3	0.50963	2.95371	4.18188	0.00581	0.02082	-3.1274
HNRNPU	0.50978	4.76634	6.34004	0.00072	0.00475	-0.779
FKBP5	0.50997	3.2328	3.95279	0.00752	0.02524	-3.4151
KCNIP3	0.5102	1.35288	3.7409	0.00962	0.03019	-3.6881
PTPRK	0.51074	4.44868	5.98343	0.00098	0.00587	-1.1253
OSTF1	0.51132	2.25699	4.99612	0.00247	0.01117	-2.1669
ZNF487	0.51143	-0.3903	4.54323	0.00393	0.01563	-2.6892
SNX25	0.51199	1.80024	5.11033	0.0022	0.01032	-2.0397
FARSB	0.51214	3.82711	4.78401	0.00306	0.01307	-2.4079
ZRANB3	0.51393	1.9745	5.31178	0.00181	0.00902	-1.8199
ZNF432	0.51473	2.187	4.06841	0.0066	0.02298	-3.269
EPM2AIP	0.5159	3.07289	6.59902	0.00058	0.0041	-0.5367
RITA1	0.51601	4.60904	5.2283	0.00196	0.00953	-1.9103
MRPL48F	0.51613	1.76885	5.51293	0.0015	0.00796	-1.6058
TPMT	0.51647	4.37674	4.958	0.00256	0.01149	-2.2097
MEF2A	0.51648	3.41919	3.20519	0.01849	0.04944	-4.4056
MRTFB	0.51677	3.13506	4.58993	0.00374	0.01511	-2.634
TRAK1	0.51698	4.90672	4.7574	0.00314	0.01332	-2.4386
CARM1	0.51783	5.07026	5.79815	0.00116	0.00663	-1.3112
MMUT	0.51806	3.66626	5.36356	0.00173	0.00873	-1.7642
MAN2A2	0.51814	3.04396	5.21798	0.00198	0.0096	-1.9216
PCNT	0.51827	4.49205	3.73063	0.00974	0.03047	-3.7015
RPL12P1	0.51857	0.19911	3.24941	0.01749	0.04744	-4.345
ZNF766	0.5196	2.98315	4.50416	0.00409	0.01607	-2.7357
MAPK14	0.51985	4.45961	6.91186	0.00045	0.00347	-0.2537
LRRC49	0.51986	0.24563	3.92239	0.00779	0.02586	-3.4539
AKR1A1	0.51998	3.559	3.84654	0.0085	0.02762	-3.5512
REXO2	0.52061	5.17821	6.70669	0.00053	0.00387	-0.4381
CPT1A	0.52119	4.50335	5.64488	0.00133	0.00733	-1.4682
PNPLA6	0.52137	4.65749	6.0325	0.00094	0.00571	-1.0767
GOLGA2	0.52164	4.36776	3.28293	0.01678	0.04593	-4.2992
CSNK2B	0.52179	3.01397	3.97204	0.00736	0.02484	-3.3907
NOSIP	0.52282	4.97146	5.9896	0.00098	0.00585	-1.1192
LARP1B	0.523	3.17209	3.76623	0.00934	0.02955	-3.6551
CCDC149	0.52349	1.55356	4.03633	0.00684	0.02356	-3.3093
NOP9	0.52364	2.66066	3.36393	0.01517	0.04261	-4.1891
SHTN1	0.52378	4.09253	4.92772	0.00264	0.01175	-2.2439
DPP3P2	0.52416	0.01893	3.4867	0.01305	0.03817	-4.0238
PLA2G12	0.52497	1.97623	3.51145	0.01266	0.03731	-3.9908
EIF4BP7	0.52508	4.7267	4.71304	0.00329	0.01378	-2.4899
SMIM13	0.52545	4.37968	4.32771	0.00495	0.01846	-2.9483
NEK2	0.52548	2.30904	3.23964	0.01771	0.04783	-4.3584
ZNF770	0.52553	4.02555	5.88681	0.00107	0.00626	-1.2217
MAST3	0.52566	2.12434	5.35879	0.00173	0.00876	-1.7694
ASL	0.52578	2.48559	5.5484	0.00145	0.0078	-1.5686
GALNT7	0.52578	3.53581	6.15516	0.00085	0.00532	-0.9567
FCHO1	0.52595	2.28344	6.09412	0.00089	0.00551	-1.0162
RPS6KL1	0.52597	1.75204	5.58373	0.0014	0.00763	-1.5317
ZNF468	0.52634	2.72989	5.29645	0.00184	0.00912	-1.8364
CUL7	0.52666	3.63348	5.99658	0.00097	0.00583	-1.1122
RPS5	0.52669	7.1771	6.05134	0.00092	0.00564	-1.0582
LINC0298	0.52679	1.3591	3.37911	0.01489	0.04203	-4.1686
KCMF1	0.52727	4.89639	5.29368	0.00184	0.00914	-1.8394
GABPAP	0.52744	3.05804	3.84453	0.00852	0.02763	-3.5538
RPS11P5	0.52771	7.79749	6.02313	0.00095	0.00574	-1.086

ARL3	0.52783	2.17019	4.23709	0.00546	0.01986	-3.0592
SGMS2	0.52816	2.38298	3.703	0.01006	0.03122	-3.7376
PTGES3L	0.52821	-2.2797	3.58912	0.01153	0.03467	-3.8875
ACTR3	0.52827	6.09254	5.59425	0.00139	0.00758	-1.5207
MAD2L1E	0.52836	3.03325	5.66157	0.00131	0.00725	-1.4509
GPANK1	0.52887	2.70293	4.60198	0.00369	0.01498	-2.6198
SNHG12	0.52895	3.73016	5.15617	0.00211	0.01002	-1.9892
SLC35F5	0.52898	3.97875	4.37872	0.00468	0.0177	-2.8863
HERPUD	0.52994	3.18529	6.36636	0.00071	0.00467	-0.7541
PPP1R3C	0.53016	0.30586	3.4249	0.01407	0.04038	-4.1068
TMCO4	0.53095	2.68461	4.40912	0.00453	0.01729	-2.8496
SMCHD1	0.53099	4.12306	5.13533	0.00215	0.01018	-2.0122
C10orf95-	0.53103	1.40867	4.21312	0.00561	0.02027	-3.0888
UBA2	0.53237	5.39016	6.70249	0.00054	0.00388	-0.442
ADAM10	0.53256	5.11752	5.78838	0.00117	0.00666	-1.3211
TJP1	0.53288	5.25277	5.80926	0.00114	0.00659	-1.2999
PHF11	0.53294	0.76891	3.24441	0.0176	0.04765	-4.3518
EIF3LP3	0.53308	2.23145	3.66373	0.01054	0.03239	-3.7891
NCOA4	0.53327	6.51763	5.86836	0.00109	0.00634	-1.2402
ACTN4	0.53331	8.10627	3.30016	0.01642	0.04523	-4.2757
MRPL14	0.53354	3.92106	6.70604	0.00054	0.00387	-0.4387
MED25	0.53398	4.48958	7.09931	0.00039	0.00315	-0.0889
BAG5	0.53417	4.86619	6.94284	0.00044	0.00342	-0.2262
GXYLT1	0.53489	3.50341	6.33037	0.00073	0.00478	-0.7882
HMGB3	0.53507	4.86984	4.99035	0.00248	0.01123	-2.1734
WSB2	0.53538	4.74827	3.76234	0.00938	0.02965	-3.6602
OFD1	0.53573	2.39352	4.59984	0.0037	0.015	-2.6223
SIRT5	0.53636	2.81865	3.96694	0.0074	0.02495	-3.3971
GTPBP1	0.53649	2.57361	3.72498	0.0098	0.03061	-3.7088
LENG8-A	0.53672	1.01029	5.19046	0.00204	0.0098	-1.9516
OXR1	0.53719	4.77242	5.03669	0.00237	0.01087	-2.1215
ARHGAP	0.53744	4.47389	6.24063	0.00079	0.00504	-0.8741
UBAP1	0.53799	3.82381	6.35626	0.00071	0.0047	-0.7636
QTRT1	0.53822	4.31422	5.86445	0.00109	0.00635	-1.2442
LINC0113	0.53843	0.46137	5.1338	0.00215	0.01019	-2.0138
PTGFRN	0.53845	3.72848	3.35704	0.0153	0.04288	-4.1985
ANKLE2	0.53848	4.29451	6.67668	0.00055	0.00394	-0.4655
ERI2	0.53878	2.28502	4.51028	0.00407	0.016	-2.7284
CHST15	0.53911	2.91739	4.12808	0.00617	0.02183	-3.1943
SRD5A1F	0.53918	1.78516	5.09502	0.00224	0.01043	-2.0567
MYH10	0.53934	6.11206	5.33688	0.00177	0.00887	-1.7929
USP33	0.53937	5.36565	6.87585	0.00047	0.00354	-0.2857
KRBOX5	0.54092	2.09435	5.28397	0.00186	0.00919	-1.8499
SEMA4C	0.5411	3.29044	4.1876	0.00577	0.02072	-3.1203
GOT1	0.54128	4.67295	3.60384	0.01133	0.03424	-3.868
DEGS1	0.54135	5.43154	5.25527	0.00191	0.00938	-1.881
SCD	0.54152	6.51997	3.68514	0.01028	0.03175	-3.761
RANBP9	0.54178	5.04576	4.68875	0.00337	0.01401	-2.5182
EPN1	0.54185	6.4421	6.54441	0.00061	0.00423	-0.5872
NFKBIL1	0.54207	2.62447	5.24428	0.00193	0.00945	-1.8929
USP4	0.54208	3.26571	4.1447	0.00605	0.02152	-3.1736
TMEM30A	0.54249	6.29422	3.80876	0.00889	0.02848	-3.6
ZNRF2P2	0.54258	-1.4669	3.4363	0.01388	0.03997	-4.0915
SNX18	0.54278	3.09938	4.39143	0.00462	0.01752	-2.871
ZNF436	0.54282	1.75567	6.09033	0.00089	0.00552	-1.0199
ARL6IP1F	0.54406	1.23047	4.70261	0.00332	0.01388	-2.5021
COPS7A	0.5444	3.54585	6.24684	0.00078	0.00502	-0.8681
BAIAP2L1	0.54451	5.08474	4.6626	0.00346	0.0143	-2.5487

ARID2	0.54472	4.00216	6.92573	0.00045	0.00345	-0.2414
OARD1	0.54501	1.58496	5.18569	0.00205	0.00982	-1.9568
SPOPL	0.54676	2.72292	6.27243	0.00076	0.00494	-0.8435
KIF22	0.54788	5.46023	6.59911	0.00058	0.0041	-0.5366
TMSB4XI	0.54808	2.47682	5.0721	0.00229	0.01059	-2.0821
PTENP1	0.5487	4.31742	5.52504	0.00148	0.00791	-1.593
SIMC1	0.55021	3.03981	6.09626	0.00089	0.00551	-1.0141
ZNF283	0.55059	1.35956	5.09561	0.00223	0.01043	-2.056
CNOT9	0.55067	4.16468	5.54347	0.00146	0.00781	-1.5737
ZBTB42	0.55097	1.79243	3.59704	0.01142	0.03444	-3.877
PBX2P1	0.55118	3.06604	4.07788	0.00653	0.02279	-3.2571
RPL28	0.55138	7.70594	6.11556	0.00087	0.00545	-0.9952
DNAI4	0.55147	0.13134	3.99607	0.00716	0.02434	-3.3602
MRPL48	0.55168	1.87025	5.57337	0.00142	0.00768	-1.5425
PABIR2	0.55192	4.67414	5.38025	0.0017	0.00865	-1.7464
CRY1	0.55239	3.06937	6.31189	0.00074	0.00483	-0.8058
SNRPC	0.55275	3.96002	3.66672	0.01051	0.03232	-3.7851
KATNIP	0.55277	3.06362	7.11783	0.00039	0.00313	-0.0728
PRR14L	0.55313	3.60205	7.03965	0.00041	0.00326	-0.141
PPP2R1A	0.5536	7.00048	6.80584	0.00049	0.00368	-0.3484
NSUN5P1	0.55413	2.61934	3.72675	0.00978	0.03057	-3.7065
OSTF1P1	0.55437	1.91728	5.41298	0.00165	0.00848	-1.7115
SKIC2	0.55566	4.92752	5.58541	0.0014	0.00762	-1.5299
C18orf25	0.55608	4.30203	4.27366	0.00525	0.01928	-3.0143
KAZN	0.55627	5.02268	3.85158	0.00845	0.02749	-3.5447
EXOC4	0.55645	6.37607	4.538	0.00395	0.01568	-2.6954
UBE2J1	0.55658	4.60489	5.24674	0.00193	0.00943	-1.8903
PCDHGC	0.55683	2.91508	4.36719	0.00474	0.0179	-2.9003
ACTN1	0.55685	6.88373	4.44788	0.00435	0.01678	-2.803
NPDC1	0.5569	2.94268	7.09961	0.00039	0.00315	-0.0887
ARSK	0.55753	1.47504	6.2617	0.00077	0.00498	-0.8538
TBC1D14	0.55758	3.29715	5.8483	0.00111	0.00641	-1.2604
GSTP1	0.55777	8.57304	4.00501	0.00709	0.02416	-3.3489
FBLIM1	0.55869	5.63982	5.92654	0.00103	0.0061	-1.1819
RCOR1	0.55919	4.42409	5.9003	0.00106	0.0062	-1.2082
PRR12	0.55921	3.84662	3.22419	0.01806	0.04857	-4.3795
HLCS	0.55944	3.927	6.04928	0.00093	0.00565	-1.0602
MED20	0.55944	3.0894	4.78272	0.00306	0.01308	-2.4094
PTPN18	0.55996	2.92095	7.01111	0.00042	0.00331	-0.166
ROGDI	0.56031	1.99426	6.67653	0.00055	0.00394	-0.4656
MRPS18A	0.56086	3.41319	5.50489	0.00151	0.008	-1.6142
ST7-AS1	0.56116	0.45069	3.46923	0.01333	0.03884	-4.0473
UPF3AP2	0.56146	1.94488	3.79589	0.00902	0.02881	-3.6166
C6orf62	0.5617	6.21414	7.00045	0.00042	0.00333	-0.1754
MAP4K5	0.56208	4.01532	6.14628	0.00085	0.00535	-0.9653
MCUR1P	0.56286	4.08564	5.33374	0.00178	0.00889	-1.7962
ABCF1	0.5632	5.17902	5.48873	0.00153	0.00806	-1.6312
PDCD2L	0.56452	2.15425	5.2179	0.00198	0.0096	-1.9216
ARPC2	0.56503	5.96423	6.51522	0.00062	0.00429	-0.6143
TES	0.56519	6.11597	4.02844	0.0069	0.02372	-3.3193
CSNK1E	0.56556	4.77032	4.91402	0.00268	0.01189	-2.2594
AK3	0.56637	4.2787	4.54903	0.0039	0.01556	-2.6824
CARMIL1	0.56704	4.90186	4.52683	0.00399	0.01583	-2.7087
RPS11	0.56903	6.88094	4.83302	0.00291	0.01262	-2.3516
TMEM168	0.57071	3.2192	4.17254	0.00587	0.02099	-3.139
HSPB1P1	0.57087	3.70261	3.67865	0.01036	0.03195	-3.7695
HIVEP1	0.57114	3.46971	6.99078	0.00043	0.00334	-0.1839
ZNF618	0.57117	3.95243	4.7625	0.00312	0.01327	-2.4327

TTC3	0.57138	5.54247	5.43366	0.00161	0.00837	-1.6895
ELF4	0.57213	3.93532	5.99931	0.00097	0.00582	-1.1095
LDLR	0.57219	5.89226	5.57172	0.00142	0.00769	-1.5442
NIBAN2	0.57243	6.10382	5.8103	0.00114	0.00659	-1.2989
LHX6	0.57252	0.02532	3.5118	0.01266	0.0373	-3.9903
COBLL1	0.57262	2.46171	7.15961	0.00038	0.00307	-0.0367
CCNA2	0.57283	5.06453	4.46054	0.00429	0.01664	-2.7878
GCDH	0.57313	3.20519	4.91015	0.00269	0.01193	-2.2638
GOLPH3	0.57349	4.66457	5.37764	0.0017	0.00866	-1.7492
ZNF544	0.57418	3.00897	5.13095	0.00216	0.0102	-2.017
STRAP	0.57487	5.38552	6.20831	0.00081	0.00514	-0.9052
EEF1E1P	0.57577	1.60527	4.10515	0.00633	0.02226	-3.2229
ALDH4A1	0.57591	1.36471	3.89512	0.00804	0.02645	-3.4888
LIN54	0.57678	2.73523	6.65969	0.00056	0.00398	-0.481
FLII	0.57693	5.62507	4.87357	0.00279	0.01226	-2.3053
YWHAZP	0.57797	3.30658	4.9368	0.00262	0.01167	-2.2336
NCS1	0.57841	4.19454	5.54109	0.00146	0.00782	-1.5762
LEMD2	0.57844	4.73868	4.88801	0.00275	0.01212	-2.2889
YWHAH	0.57863	5.73408	4.45515	0.00431	0.01669	-2.7943
ARRDC2	0.57879	1.74135	5.39898	0.00167	0.00853	-1.7264
MRS2	0.57884	4.64488	6.0066	0.00096	0.00579	-1.1023
PPFIA3	0.57931	2.80015	4.76786	0.00311	0.01324	-2.4265
OPN3	0.57981	2.50998	4.07124	0.00657	0.02294	-3.2654
CITED2	0.58004	4.79224	5.15928	0.0021	0.01	-1.9858
DSTNP2	0.58133	3.24431	3.81431	0.00883	0.02837	-3.5928
DRG1P1	0.58152	3.83866	3.50423	0.01277	0.03757	-4.0004
TAGLN2P	0.58157	6.67335	4.35653	0.0048	0.01802	-2.9132
SLC4A7	0.58159	3.53811	3.22561	0.01802	0.04852	-4.3776
CTSH	0.58176	2.25417	4.31975	0.00499	0.01858	-2.958
BYSL	0.58334	3.56984	3.54209	0.0122	0.0362	-3.9499
YWHAZP	0.58593	5.13055	4.79943	0.00301	0.01293	-2.3901
SLC25A2	0.58632	2.57096	3.59201	0.01149	0.03458	-3.8837
EIF3LP1	0.5865	3.81586	5.0615	0.00231	0.01067	-2.0939
ERV3-1	0.58669	0.00141	3.67064	0.01046	0.03219	-3.78
ZCCHC2	0.58673	0.81882	3.82214	0.00875	0.02819	-3.5827
PRKCIP1	0.58687	5.09882	5.10299	0.00222	0.01038	-2.0479
TBC1D23	0.58696	3.33657	8.04386	0.0002	0.00205	0.69046
XRCC5	0.58752	6.5061	5.47642	0.00155	0.00813	-1.6442
LINC0298	0.58845	0.45745	3.28059	0.01682	0.04599	-4.3024
OSER1	0.58986	4.5406	6.07588	0.0009	0.00556	-1.0341
GOLGA2I	0.59	0.52876	5.42615	0.00163	0.0084	-1.6975
PRPF4BP	0.59009	1.64403	5.80326	0.00115	0.00662	-1.306
CRIM1	0.5907	6.04314	4.35431	0.00481	0.01805	-2.9159
CAPN2	0.59118	5.5728	3.36771	0.0151	0.0425	-4.184
LRRK1	0.59126	1.44723	3.36395	0.01517	0.04261	-4.1891
ANAPC15	0.59143	3.79774	6.0234	0.00095	0.00574	-1.0857
WDR25	0.59188	1.5426	3.8318	0.00865	0.02796	-3.5702
GIPC1	0.59198	5.91851	7.41959	0.00031	0.00269	0.18455
LINC0066	0.59199	1.87682	3.31127	0.01619	0.04478	-4.2606
ZFAND3	0.59201	5.1013	4.10439	0.00633	0.02227	-3.2239
NT5E	0.59232	4.21457	4.47825	0.00421	0.01641	-2.7666
SLC38A1	0.59247	7.29854	4.99006	0.00248	0.01123	-2.1737
PSPC1	0.5929	3.6039	6.83118	0.00048	0.00363	-0.3257
RPS5P8	0.59379	4.4995	6.25616	0.00078	0.00498	-0.8591
RANBP6	0.59387	4.86103	5.20121	0.00202	0.00972	-1.9399
FOXP4	0.59422	3.69235	4.23424	0.00548	0.01991	-3.0627
LSM2	0.59459	3.73169	6.27876	0.00076	0.00492	-0.8375
MRPS18B	0.59511	4.22725	5.35791	0.00173	0.00876	-1.7703

AGAP1	0.59577	4.25127	6.6398	0.00056	0.00402	-0.4992
TBCC	0.59801	3.56973	5.99714	0.00097	0.00583	-1.1117
RPL13AP	0.59807	1.46623	4.12151	0.00621	0.02196	-3.2025
FBXW9	0.59853	2.29606	4.4098	0.00453	0.01728	-2.8488
WWOX	0.59963	2.89886	5.10029	0.00222	0.0104	-2.0508
ABHD17C	0.59963	1.57655	5.45652	0.00158	0.00825	-1.6653
ENAH	0.59965	6.06883	6.99362	0.00043	0.00334	-0.1814
ACTR3B	0.60003	2.2507	4.57991	0.00378	0.01523	-2.6458
FBXO46	0.60038	3.25421	6.95843	0.00044	0.00339	-0.2124
TBC1D7	0.60168	4.41915	4.22565	0.00553	0.02005	-3.0733
ARHGEF	0.6017	4.5095	5.55416	0.00144	0.00777	-1.5625
PBX2	0.6031	3.32317	4.24711	0.0054	0.01973	-3.0469
RFX3	0.60342	2.27425	3.85858	0.00839	0.02733	-3.5357
PAX6	0.60423	1.39249	4.85042	0.00286	0.01246	-2.3317
ZYX	0.60437	6.8094	4.76726	0.00311	0.01324	-2.4272
DCDC2	0.6044	5.81796	3.45759	0.01352	0.03926	-4.0629
SEPTIN7	0.60495	0.55436	3.33115	0.0158	0.04396	-4.2336
BDH1	0.60523	3.42472	4.63353	0.00357	0.01463	-2.5827
SLC35E1	0.60686	4.60461	7.45649	0.0003	0.00266	0.21543
KDM1B	0.60755	3.75336	3.7171	0.0099	0.03085	-3.7191
UBE2MP	0.60759	6.46326	7.21699	0.00036	0.00296	0.0127
DAG1	0.60784	5.097	6.44825	0.00066	0.00446	-0.6769
DEPDC5	0.60792	1.80926	5.04884	0.00234	0.01079	-2.108
RPL10AP	0.60865	3.97317	6.91215	0.00045	0.00347	-0.2534
TRAF4	0.609	4.64599	7.2424	0.00035	0.00293	0.03447
SPART	0.60974	3.81853	6.58885	0.00059	0.00413	-0.5461
ZBTB33	0.60992	3.95905	5.50166	0.00152	0.00801	-1.6176
WWC1	0.61034	6.04531	3.39891	0.01453	0.04128	-4.1418
LAPTM4E	0.61117	3.93046	4.76895	0.0031	0.01323	-2.4252
IL6STP1	0.6112	0.98584	3.34462	0.01554	0.04341	-4.2153
B3GNT7	0.61233	0.37874	3.42544	0.01406	0.04037	-4.1061
LSM14A	0.61249	5.68313	6.66777	0.00055	0.00396	-0.4736
MAP3K14	0.61399	-0.6172	3.92149	0.0078	0.02588	-3.4551
GNL1	0.61418	4.5517	7.70485	0.00025	0.00238	0.42001
FGFR1OI	0.6142	2.97235	5.54372	0.00146	0.00781	-1.5735
GAB1	0.61481	1.37771	4.04548	0.00677	0.02338	-3.2978
HSPBP1	0.61512	5.02741	5.8958	0.00106	0.00622	-1.2127
FBXO41	0.61518	0.78546	4.02693	0.00691	0.02375	-3.3212
GCAT	0.61571	3.499	6.96892	0.00043	0.00337	-0.2031
PEX6	0.61589	3.4971	6.98401	0.00043	0.00335	-0.1898
ZNF765	0.61591	2.54453	5.64713	0.00133	0.00732	-1.4659
HMGN4	0.61594	4.5652	5.16411	0.00209	0.00997	-1.9805
PLXNA3	0.61688	3.02345	6.62118	0.00057	0.00406	-0.5163
RNF5P1	0.61712	4.47269	3.70543	0.01003	0.03116	-3.7344
NBL1	0.61728	1.1914	3.69493	0.01016	0.03144	-3.7481
ZNF554	0.61729	0.64972	4.57635	0.00379	0.01525	-2.65
SEPTIN8	0.61931	5.37531	3.25625	0.01734	0.04712	-4.3357
DERL3	0.61955	0.60566	3.93776	0.00765	0.02559	-3.4343
LRRC8B	0.61965	2.75436	6.99924	0.00042	0.00333	-0.1764
PHF21B	0.61969	-1.1112	3.53145	0.01236	0.03656	-3.9641
PUS7L	0.61976	3.31243	7.16835	0.00037	0.00305	-0.0291
UTP14C	0.62	3.27992	8.29039	0.00017	0.00184	0.88106
MEMO1	0.62085	-0.0317	4.20926	0.00564	0.02033	-3.0935
BRME1	0.62087	1.30681	3.54349	0.01218	0.03615	-3.9481
MRPL57	0.6216	4.33543	6.82722	0.00049	0.00364	-0.3292
FBXW11I	0.62175	2.99519	6.97573	0.00043	0.00336	-0.1971
TINAGL1	0.62294	6.15409	3.79032	0.00908	0.02894	-3.6238
DLG3	0.6231	2.72499	4.48688	0.00417	0.01631	-2.7563

PRKCI	0.62423	4.70124	5.35431	0.00174	0.00877	-1.7742
BAG6	0.62527	6.29979	8.37278	0.00016	0.00178	0.94367
PCDHB13	0.62542	1.17104	4.13212	0.00614	0.02176	-3.1892
GNAZ	0.62562	1.30151	3.22622	0.01801	0.0485	-4.3768
PHLDB2	0.62577	5.3773	3.7456	0.00957	0.03005	-3.682
WDFY2	0.62608	1.24322	3.97364	0.00735	0.02481	-3.3886
SCD5	0.62774	3.03488	3.72817	0.00977	0.03054	-3.7047
USP49	0.62858	1.68535	6.0955	0.00089	0.00551	-1.0148
CARS2	0.62859	4.15214	4.51381	0.00405	0.01597	-2.7242
EEF1E1	0.62861	2.84995	5.36808	0.00172	0.00871	-1.7594
FCSK	0.6289	1.90444	7.85102	0.00023	0.00222	0.53784
RPL7P22	0.62892	1.56997	3.24312	0.01763	0.04768	-4.3536
FOXO3	0.62903	3.08628	5.59994	0.00138	0.00756	-1.5148
COMMD6	0.62941	2.44393	7.79521	0.00024	0.00228	0.49308
TRIM38	0.62953	4.22143	3.29021	0.01662	0.04558	-4.2893
SEPTIN2	0.62979	0.96378	3.85947	0.00838	0.02731	-3.5346
PIK3AP1	0.63072	2.4948	4.0701	0.00658	0.02296	-3.2668
MRPL44	0.63075	2.18107	7.4187	0.00031	0.00269	0.1838
EXT1	0.63212	4.48242	5.62027	0.00136	0.00746	-1.4937
FKBP4	0.6328	5.77147	6.09168	0.00089	0.00552	-1.0186
UBE4B	0.63341	4.75208	7.78738	0.00024	0.00229	0.48677
SPRY1	0.63375	3.1891	4.51291	0.00405	0.01598	-2.7253
ZSCAN22	0.63455	1.57643	7.23964	0.00035	0.00293	0.03211
TRIM8	0.63469	3.04581	4.55543	0.00388	0.01547	-2.6748
ZNF451	0.63534	3.49023	5.07087	0.00229	0.0106	-2.0835
MPST	0.63624	4.06939	6.97798	0.00043	0.00336	-0.1952
DLX6	0.63646	2.76926	4.25646	0.00535	0.01957	-3.0354
ZNF613	0.63676	0.80017	5.48813	0.00153	0.00806	-1.6319
DBT	0.63717	2.29755	6.79245	0.0005	0.0037	-0.3605
ARHGAP	0.63727	2.02901	4.54801	0.00391	0.01557	-2.6836
CEP20	0.63728	4.43656	7.11895	0.00039	0.00313	-0.0719
C6orf89	0.63729	4.07287	8.01369	0.0002	0.00208	0.6668
CDYL	0.63767	4.16974	8.69382	0.00013	0.00156	1.18255
EIF3D	0.63782	5.94588	6.98641	0.00043	0.00334	-0.1877
FBXO7	0.63783	4.72727	7.88609	0.00022	0.00219	0.56583
SGPL1	0.63846	4.92254	5.70158	0.00126	0.00708	-1.4098
PASK	0.63865	1.7299	6.73496	0.00052	0.00383	-0.4125
UBE2HP1	0.63924	3.31723	4.14808	0.00603	0.02145	-3.1694
PWWP2A	0.63946	3.44077	8.3925	0.00016	0.00178	0.95857
TRIM47	0.63993	3.80806	3.40748	0.01438	0.04098	-4.1303
SOS2	0.64004	3.09565	6.66251	0.00055	0.00398	-0.4784
TCF7	0.64061	0.62799	3.38762	0.01473	0.0417	-4.1571
ZDHHC7	0.6408	4.08117	8.13297	0.00019	0.00198	0.75993
BICRA	0.64081	1.39044	6.83009	0.00048	0.00363	-0.3267
ALKBH7	0.64144	4.7874	3.9284	0.00774	0.02575	-3.4462
SFT2D2	0.64162	5.47023	6.07957	0.0009	0.00555	-1.0305
SLC44A3	0.64292	0.48569	4.71615	0.00328	0.01375	-2.4863
ANKRD13	0.64305	3.42421	4.39019	0.00462	0.01753	-2.8725
C1QL1	0.64311	1.76073	4.9975	0.00246	0.01116	-2.1653
METRNL	0.64317	3.02482	6.26036	0.00077	0.00498	-0.8551
TPM4	0.64321	7.6333	5.50441	0.00151	0.008	-1.6147
YAF2	0.64345	2.61565	6.70655	0.00054	0.00387	-0.4383
FADS2	0.64395	3.54044	3.88932	0.00809	0.02656	-3.4962
ARID3BP	0.64434	0.43406	6.07273	0.00091	0.00556	-1.0372
ZNF318	0.64452	3.016	5.76432	0.00119	0.00677	-1.3456
KLC4	0.64452	2.49201	6.09606	0.00089	0.00551	-1.0143
LRATD2	0.6447	5.90657	3.3571	0.0153	0.04288	-4.1984
G2E3	0.64511	3.41233	5.97324	0.00099	0.00591	-1.1354

TSNARE1	0.64521	1.90208	6.40036	0.00069	0.00458	-0.7219
PHKA2	0.64614	3.02791	4.55896	0.00386	0.01543	-2.6706
OTUD6B-	0.64614	3.78919	7.48292	0.0003	0.00264	0.23747
TRNAU1A	0.64637	1.68744	6.29974	0.00075	0.00486	-0.8174
TENT4B	0.64689	4.49411	7.97895	0.00021	0.00211	0.63944
CC2D1A	0.64757	4.55523	6.40113	0.00069	0.00458	-0.7212
PAPOLB	0.64771	1.02489	5.29289	0.00185	0.00915	-1.8403
NUFIP1P	0.64776	2.39051	4.83333	0.00291	0.01262	-2.3513
SETDB2	0.64865	0.99788	6.11749	0.00087	0.00544	-0.9934
CLCN2	0.65016	1.95829	3.65676	0.01063	0.0326	-3.7982
HPGD	0.65059	-6.3062	3.40148	0.01448	0.04118	-4.1384
PLS1	0.6517	4.53997	7.60129	0.00027	0.0025	0.33538
SORBS3	0.65175	3.59462	6.34455	0.00072	0.00473	-0.7748
PKP4	0.6525	5.05914	5.26344	0.0019	0.00933	-1.8721
BEND6	0.65276	1.41445	3.37229	0.01501	0.04233	-4.1778
AKAP12	0.65351	3.20702	3.57058	0.01179	0.03526	-3.9121
SNRNP48	0.65371	4.04502	5.6001	0.00138	0.00756	-1.5146
JADE3	0.65417	3.04778	4.82484	0.00293	0.0127	-2.361
VARS1	0.65424	5.7816	7.09978	0.00039	0.00315	-0.0885
ICE2P2	0.65462	-0.4966	4.69306	0.00336	0.01397	-2.5132
LTBP3	0.65538	3.54955	3.72201	0.00984	0.0307	-3.7127
NBPF3	0.65555	1.10628	8.68371	0.00013	0.00157	1.17515
ITGAV	0.6557	5.93504	4.56694	0.00383	0.01537	-2.6612
ZNF813	0.65767	2.21413	5.35742	0.00174	0.00876	-1.7708
NCOA4P4	0.65824	1.93485	4.42109	0.00447	0.01713	-2.8352
MTCH1	0.65867	6.00764	6.76657	0.00051	0.00376	-0.3838
CDK19	0.65876	2.47641	5.9263	0.00103	0.0061	-1.1821
PLAGL2	0.65915	3.87348	4.57975	0.00378	0.01523	-2.646
FBXO44	0.65972	2.74367	4.26295	0.00531	0.01946	-3.0274
KLHDC3	0.66086	5.45602	8.23208	0.00017	0.00189	0.83643
CDR2	0.66109	3.6678	4.80749	0.00298	0.01286	-2.3809
CYB5A	0.66111	2.79271	4.94209	0.0026	0.01163	-2.2277
FRAT1	0.66146	0.61183	5.17584	0.00207	0.00988	-1.9676
RPS18	0.6625	5.20599	3.38447	0.01479	0.04181	-4.1613
NUDT19	0.6634	3.63239	7.67289	0.00026	0.00242	0.394
SLC39A7	0.66364	6.31992	7.39854	0.00031	0.00271	0.16687
ACTR3P2	0.66365	4.2207	6.61804	0.00057	0.00406	-0.5192
MFSD13A	0.66368	-0.1814	4.39436	0.0046	0.0175	-2.8674
CTAGE1	0.66434	0.4312	3.3934	0.01463	0.04148	-4.1493
POMP	0.66439	4.53324	5.87109	0.00108	0.00633	-1.2375
ZBTB8B	0.66465	-2.1395	3.22698	0.01799	0.04847	-4.3757
SYT14	0.66537	2.01484	6.88907	0.00046	0.00351	-0.2739
RBM7P1	0.66671	3.44645	5.73389	0.00122	0.00691	-1.3766
GNAI1	0.66674	3.77744	4.61949	0.00362	0.01477	-2.5992
CTBP2P4	0.66744	3.56534	4.83193	0.00291	0.01263	-2.3529
TMEM183	0.66759	3.5517	6.43296	0.00067	0.00449	-0.6912
SPRYD7	0.66835	2.27938	7.41523	0.00031	0.0027	0.18089
WDR46	0.66868	4.81563	5.58066	0.00141	0.00764	-1.5349
SLC25A3	0.66873	2.7108	8.37798	0.00016	0.00178	0.9476
SPIN1	0.66874	4.60206	8.21053	0.00018	0.00191	0.81986
ETHE1	0.66936	3.54203	5.27603	0.00188	0.00924	-1.8585
NAT14	0.66965	4.86448	3.58034	0.01165	0.03499	-3.8991
ARFGEF1	0.66989	5.17552	8.76178	0.00012	0.00152	1.23212
GYG2	0.67082	2.39567	3.50114	0.01282	0.03764	-4.0045
PRICKLE	0.67112	3.21432	5.84305	0.00111	0.00643	-1.2657
ZNF579	0.67137	5.03207	5.89897	0.00106	0.00621	-1.2095
RAB27A	0.67148	2.98279	5.62484	0.00135	0.00744	-1.4889
FZD6	0.6716	4.3173	6.21355	0.0008	0.00512	-0.9001

TRMT12	0.67198	1.4257	7.74964	0.00024	0.00234	0.45632
SPECC1	0.67198	4.66632	6.60533	0.00058	0.00409	-0.5309
SAMD4A	0.67231	3.95554	3.31249	0.01617	0.04474	-4.2589
VPS52	0.67247	3.96305	7.31113	0.00034	0.00281	0.09304
RNF115	0.6738	4.66635	4.80358	0.00299	0.0129	-2.3854
MTMR6	0.67447	2.60201	5.03703	0.00237	0.01087	-2.1211
TP53BP2	0.67566	3.8016	7.83802	0.00023	0.00223	0.52743
SLC43A1	0.67636	-0.1267	3.42266	0.01411	0.04046	-4.1098
MCU	0.67706	4.8	4.51498	0.00404	0.01597	-2.7228
TNFAIP8I	0.67842	2.17782	4.62408	0.00361	0.01472	-2.5938
TPMTP1	0.67857	5.09367	6.045	0.00093	0.00566	-1.0644
ANXA11	0.67902	5.93957	6.29163	0.00075	0.00488	-0.8252
ZNF584	0.67935	3.18735	7.84868	0.00023	0.00223	0.53597
KCTD20	0.68022	4.38614	9.43259	8.10E-05	0.00116	1.7037
PRKCD	0.68126	3.38355	5.23666	0.00195	0.00949	-1.9012
SMURF2I	0.68131	-1.0711	3.46697	0.01337	0.03891	-4.0503
HERC3	0.68138	1.33882	5.18052	0.00206	0.00986	-1.9625
MYO9A	0.68186	2.9941	5.84726	0.00111	0.00642	-1.2615
FAM171A	0.68186	3.19642	6.06021	0.00092	0.0056	-1.0495
PRMT1	0.68245	5.73792	6.71884	0.00053	0.00385	-0.4271
ZNF761	0.68268	3.58884	7.94897	0.00021	0.00212	0.61576
SPEG	0.6827	2.0649	4.81773	0.00295	0.01279	-2.3691
SAP30	0.68365	2.13758	5.58833	0.0014	0.00761	-1.5269
DM1-AS	0.68527	-1.4021	3.93072	0.00772	0.02572	-3.4433
PABPC4L	0.68597	-0.1149	3.31313	0.01616	0.04473	-4.2581
HLA-E	0.68609	5.04009	4.01693	0.00699	0.02395	-3.3338
LRP5	0.68697	4.1752	5.50758	0.00151	0.00798	-1.6114
KIAA1522	0.68758	5.8832	4.89001	0.00274	0.0121	-2.2866
PPARD	0.68826	3.23827	3.98394	0.00726	0.02461	-3.3756
SAP18P2	0.68859	2.32656	4.09693	0.00639	0.02241	-3.2332
ITPRIPL2	0.68868	4.74698	5.01844	0.00241	0.01101	-2.1419
HERPUD	0.6888	4.58288	4.25997	0.00533	0.01951	-3.0311
BCL7A	0.6889	2.17328	4.05389	0.0067	0.02324	-3.2872
INKA2	0.68906	0.04473	3.51979	0.01253	0.03703	-3.9796
TUBGCP3	0.69	3.82923	6.22951	0.00079	0.00507	-0.8848
LITAF	0.69073	5.10921	4.30963	0.00505	0.01873	-2.9703
ZNF627	0.69085	1.85487	6.5659	0.0006	0.00418	-0.5673
BMP1	0.69181	2.2823	4.08619	0.00646	0.02263	-3.2466
VAV1	0.69274	2.6758	3.32145	0.01599	0.04439	-4.2468
DLEU1	0.69276	3.34604	4.06767	0.0066	0.02299	-3.2699
PLA2G12	0.69411	3.22988	6.12879	0.00086	0.00541	-0.9823
POLD1	0.69414	5.81296	7.95053	0.00021	0.00212	0.61699
DIAPH1	0.69445	6.9261	7.46643	0.0003	0.00265	0.22372
OSBPL6	0.69534	0.97047	3.70104	0.01009	0.03128	-3.7401
FOXO6	0.69592	-0.9275	4.40462	0.00455	0.01735	-2.855
LPP	0.69638	6.20568	4.01732	0.00699	0.02394	-3.3333
LINC0130	0.69649	0.20287	7.93328	0.00021	0.00214	0.60333
RPL13AP	0.69724	-0.0118	4.16772	0.0059	0.02108	-3.145
LIPE	0.6975	0.52505	7.51286	0.00029	0.0026	0.26236
CAB39	0.69786	3.4783	7.02521	0.00042	0.00329	-0.1536
KCTD9P3	0.69874	0.53303	5.39463	0.00168	0.00856	-1.731
IGF2BP2	0.69896	4.63263	4.25845	0.00534	0.01953	-3.0329
EIF2AK3	0.6992	2.5061	7.95662	0.00021	0.00212	0.62181
PKN3	0.69939	1.7153	4.36222	0.00477	0.01795	-2.9063
CD82	0.70036	0.37038	5.9029	0.00105	0.00619	-1.2056
THAP10	0.70167	0.77893	4.54075	0.00394	0.01564	-2.6922
RXRB	0.70202	3.79927	8.22305	0.00018	0.0019	0.82949
ATF6B	0.70207	4.67465	7.30606	0.00034	0.00282	0.08874

KCTD9P6	0.70255	0.49583	5.55731	0.00144	0.00775	-1.5592
CTBP2	0.70268	4.65888	9.47442	7.90E-05	0.00114	1.73209
RPS10	0.70289	0.69332	3.74763	0.00955	0.03	-3.6793
RNF24	0.70441	2.67077	4.05119	0.00673	0.02329	-3.2906
TNS2-AS	0.70714	1.41715	4.09906	0.00637	0.02237	-3.2305
P2RY2	0.70885	1.40269	4.78585	0.00305	0.01306	-2.4058
PPP2R5D	0.70904	4.24491	7.28874	0.00034	0.00285	0.07401
NFYA	0.71007	3.34307	7.63024	0.00027	0.00246	0.35914
PRMT1P1	0.71146	4.19044	7.42226	0.00031	0.00269	0.18679
SYT13	0.71158	2.57077	3.19713	0.01868	0.04978	-4.4167
KIF13A	0.71262	4.64518	4.37257	0.00471	0.01782	-2.8938
EPRS1	0.71364	5.49973	5.63561	0.00134	0.00738	-1.4778
DCLRE1C	0.71456	1.07655	8.17299	0.00018	0.00194	0.79092
MATK	0.71514	-0.4199	6.23075	0.00079	0.00507	-0.8836
USP12	0.71583	2.35281	7.30272	0.00034	0.00283	0.0859
CEBPG	0.71624	5.32107	3.21607	0.01824	0.04896	-4.3907
MCUR1	0.71674	4.64769	8.0549	0.0002	0.00204	0.69911
FCHO2	0.71716	2.6707	7.13218	0.00038	0.00311	-0.0604
ADPRHL1	0.71758	-0.2257	3.27979	0.01684	0.04602	-4.3035
BHLHE40	0.71767	5.83927	5.58967	0.0014	0.0076	-1.5255
SCDP1	0.71826	5.39287	7.47043	0.0003	0.00264	0.22706
EFCAB5	0.71928	-1.6835	3.40234	0.01447	0.04118	-4.1372
SEC24D	0.71983	3.81576	5.14229	0.00213	0.01013	-2.0045
DSP-AS1	0.71996	1.92487	3.47451	0.01324	0.03863	-4.0402
ATP11A	0.72041	5.14494	6.53478	0.00061	0.00425	-0.5961
DHX16	0.7207	4.11504	7.88886	0.00022	0.00219	0.56804
CDKN2D	0.72224	2.51844	5.38873	0.00168	0.0086	-1.7373
MTRF1	0.72237	1.00826	4.19282	0.00574	0.02063	-3.1139
JARID2	0.72257	3.97128	6.53712	0.00061	0.00425	-0.594
THADA	0.72265	3.92493	7.74112	0.00024	0.00234	0.44942
TRIM45	0.72297	0.99924	3.74835	0.00954	0.02999	-3.6784
TTC39A	0.72332	-0.6166	3.62923	0.01099	0.03343	-3.8345
PRCD	0.72453	1.5477	5.50754	0.00151	0.00798	-1.6114
EMSLR	0.72468	2.85767	5.91579	0.00104	0.00614	-1.1926
MYD88	0.72475	3.52054	5.23399	0.00195	0.0095	-1.9041
RHPN2	0.72681	5.32544	5.71765	0.00124	0.00699	-1.3933
CLDND2	0.72698	-0.0788	4.06537	0.00662	0.02304	-3.2728
PIWIL4	0.72738	0.44584	5.03855	0.00236	0.01086	-2.1194
HIPK3	0.72939	3.86379	7.14475	0.00038	0.00309	-0.0495
TTC3P1	0.72966	5.19858	7.61661	0.00027	0.00248	0.34796
INTS6	0.73188	2.18665	6.21722	0.0008	0.00511	-0.8966
LMO4	0.73248	4.93362	4.7978	0.00301	0.01295	-2.392
RGL2	0.73294	3.74869	7.47244	0.0003	0.00264	0.22874
MRPS31	0.73368	1.56428	6.93767	0.00045	0.00342	-0.2308
BARD1	0.73663	2.72421	6.89844	0.00046	0.00349	-0.2656
FAM86B1	0.7369	-0.6048	6.09411	0.00089	0.00551	-1.0162
ZBED6	0.73708	0.9114	4.34183	0.00487	0.01823	-2.9311
C9orf72	0.73714	0.97565	6.00814	0.00096	0.00579	-1.1008
KIF27	0.73771	-1.1355	4.23171	0.0055	0.01996	-3.0658
CILK1	0.73861	1.13128	9.64679	7.13E-05	0.00107	1.84787
ZMYM5	0.73867	1.60224	6.82224	0.00049	0.00365	-0.3337
FHL2	0.7405	3.05893	4.66745	0.00345	0.01425	-2.543
RBM7	0.74105	3.73055	9.67796	7.00E-05	0.00106	1.86861
ZNF845	0.74174	2.19192	6.06646	0.00091	0.00558	-1.0433
NCOA4P3	0.74303	2.98238	7.39512	0.00031	0.00271	0.164
TMEM63E	0.74375	2.67207	5.34376	0.00176	0.00884	-1.7855
TAPBP	0.74379	5.58797	7.12099	0.00039	0.00313	-0.0701
LMBRD1	0.7462	2.28388	5.40025	0.00167	0.00853	-1.725

CEACAM	0.74691	-0.3508	8.89694	0.00011	0.00144	1.32968
DANCR	0.74712	4.8398	7.16354	0.00037	0.00306	-0.0333
SPRY4	0.74724	3.89305	3.47194	0.01328	0.03874	-4.0436
MRPS10	0.74827	3.52896	5.57651	0.00141	0.00766	-1.5392
ZNF738	0.7492	1.11628	7.65799	0.00026	0.00243	0.38184
DNM2	0.74967	6.16186	8.07361	0.00019	0.00203	0.71373
DNAJC3-	0.75149	-1.9588	3.62518	0.01104	0.03355	-3.8398
PKP4P1	0.75169	2.49205	6.52726	0.00062	0.00427	-0.6031
ALG5	0.75172	1.91356	6.91771	0.00045	0.00347	-0.2485
GYS1	0.75257	4.49736	5.23879	0.00194	0.00948	-1.8989
FBLIM1P2	0.75266	1.29565	4.21127	0.00562	0.0203	-3.091
SERAC1	0.75295	1.3788	5.99602	0.00097	0.00583	-1.1128
POLR1H	0.75309	3.06213	6.29299	0.00075	0.00488	-0.8239
SYT17	0.75324	1.21062	3.36783	0.0151	0.0425	-4.1839
TPT1P9	0.7545	6.27045	3.87418	0.00824	0.02694	-3.5156
CAVIN4	0.75461	-0.4725	3.31098	0.0162	0.04479	-4.261
C18orf54	0.75525	1.45005	5.60356	0.00138	0.00755	-1.511
SRPK1	0.75593	5.07499	9.38688	8.33E-05	0.00118	1.67255
ITPKC	0.75614	2.1095	7.12384	0.00039	0.00312	-0.0676
TXNDC16	0.75629	2.41063	5.24643	0.00193	0.00943	-1.8906
GTF2F2	0.7566	2.78017	7.08404	0.0004	0.00318	-0.1022
CCDC80	0.75708	4.13296	6.49549	0.00064	0.00434	-0.6327
THOC5	0.75781	3.62784	4.68894	0.00337	0.01401	-2.518
LRRC8E	0.75848	3.06896	7.9067	0.00022	0.00217	0.58224
CDC5L	0.76132	4.19638	7.71182	0.00025	0.00238	0.42567
CA5B	0.76267	1.41045	3.27859	0.01687	0.04607	-4.3051
MROH1	0.76335	2.62823	6.4588	0.00065	0.00444	-0.667
AGFG1	0.76336	3.86508	8.03011	0.0002	0.00206	0.67968
CHD7	0.76456	4.71333	5.36959	0.00172	0.00871	-1.7578
USP2-AS	0.76495	-0.6108	5.73428	0.00122	0.00691	-1.3762
SIX4	0.76528	2.73967	6.41856	0.00068	0.00453	-0.7048
FBXO27	0.76532	3.52022	4.65507	0.00349	0.01439	-2.5575
PLP2	0.76573	6.07621	7.15575	0.00038	0.00307	-0.04
DOCK7	0.76683	4.32182	5.41432	0.00164	0.00847	-1.7101
HCG11	0.7669	1.86769	8.15175	0.00018	0.00196	0.77449
SIPA1L1	0.76705	4.45797	6.71514	0.00053	0.00386	-0.4304
CTTNBP2	0.76715	2.46389	10.073	5.58E-05	0.00093	2.12614
STXBP5	0.76853	2.07342	9.506	7.75E-05	0.00113	1.75344
LEMD3	0.76881	3.23208	6.78595	0.0005	0.00372	-0.3663
ZNF311	0.76937	-0.2005	5.03368	0.00238	0.01089	-2.1249
PITX1	0.76944	3.7486	3.82966	0.00867	0.028	-3.573
TMEM184	0.77007	4.43501	5.11592	0.00219	0.01029	-2.0336
SLC25A1	0.77025	1.54268	3.95458	0.00751	0.02522	-3.4129
HACD2	0.77265	4.7189	6.69255	0.00054	0.00391	-0.451
SPTLC2	0.77287	3.58662	7.94073	0.00021	0.00213	0.60924
PCDHGB	0.77325	-1.1883	3.93002	0.00772	0.02573	-3.4442
MYBPC2	0.77327	-1.1583	8.95442	0.00011	0.0014	1.37078
ALG11	0.7734	0.51627	4.42608	0.00445	0.01709	-2.8292
FAAH	0.77358	0.93831	3.41893	0.01418	0.04057	-4.1148
BLTP3A	0.77366	3.59114	5.32535	0.00179	0.00894	-1.8053
FBXO25	0.77573	1.16105	4.02679	0.00691	0.02375	-3.3214
SEC14L2	0.77599	1.55851	6.54404	0.00061	0.00423	-0.5875
TSPAN12	0.77606	2.53329	3.91555	0.00785	0.02601	-3.4626
CLIC1	0.7761	6.28588	9.6483	7.13E-05	0.00107	1.84888
SNHG32	0.77619	4.27408	3.86836	0.00829	0.02708	-3.5231
PTPRF	0.7765	5.00511	4.24061	0.00544	0.0198	-3.0549
MCF2L-A	0.7777	1.53414	4.95588	0.00257	0.0115	-2.2121
MMP14	0.77777	2.88687	4.28778	0.00517	0.01908	-2.997

PRR14	0.77807	4.18344	6.62327	0.00057	0.00406	-0.5144
RBM47	0.77941	3.71129	4.0552	0.00669	0.02322	-3.2856
TCEAL8	0.77965	3.65119	7.66424	0.00026	0.00243	0.38694
HNRNPH	0.78153	4.68089	9.19768	9.34E-05	0.00127	1.54211
SYNGAP1	0.78186	1.95038	3.84548	0.00851	0.02762	-3.5526
GPHN	0.78209	3.09277	6.57107	0.0006	0.00417	-0.5625
UGCG	0.78265	3.74645	3.99671	0.00715	0.02433	-3.3594
PDXDC1	0.78272	5.59787	10.1532	5.33E-05	0.0009	2.17733
PWWP2E	0.78295	2.44387	8.82068	0.00012	0.00149	1.2748
BCL2L11	0.7832	2.44621	4.26185	0.00532	0.01947	-3.0288
DDX47	0.78485	-1.9814	4.09083	0.00643	0.02255	-3.2408
SMAGP	0.78494	1.51453	4.85471	0.00284	0.01242	-2.3268
CIART	0.78515	1.66542	6.10893	0.00088	0.00546	-1.0017
ZNF48	0.78522	2.83236	6.38301	0.0007	0.00462	-0.7383
CD55	0.7853	2.11597	4.67046	0.00343	0.01422	-2.5395
THAP9	0.78584	0.02819	6.84666	0.00048	0.0036	-0.3118
TESMIN	0.78671	2.23946	7.81584	0.00023	0.00226	0.50965
C6orf136	0.78709	2.59048	7.84317	0.00023	0.00223	0.53156
ZNF524	0.78745	2.96276	5.19622	0.00203	0.00976	-1.9453
USPL1	0.78818	1.55882	6.72256	0.00053	0.00385	-0.4237
BAG1	0.78837	2.94833	5.07508	0.00228	0.01057	-2.0788
CXXC5	0.78875	4.81037	7.64511	0.00026	0.00244	0.37131
AMPD3	0.78899	-0.9699	3.98907	0.00722	0.02449	-3.3691
THSD4	0.78949	3.95187	5.77236	0.00118	0.00674	-1.3374
MED4	0.79037	2.51578	9.15176	9.61E-05	0.00129	1.51008
SHANK2	0.79063	1.94391	4.43357	0.00441	0.01699	-2.8202
TMEM143	0.79081	-0.101	4.50227	0.0041	0.01609	-2.7379
MAPKAP1	0.79107	4.57522	9.66257	7.07E-05	0.00107	1.85838
DIP2A	0.79117	4.36132	7.71044	0.00025	0.00238	0.42456
STMN3	0.79124	1.30097	4.9646	0.00254	0.01143	-2.2023
VPS36	0.79147	2.76989	9.05031	0.0001	0.00135	1.43881
GLCE	0.792	1.72959	7.32237	0.00033	0.0028	0.10257
USP2	0.79243	0.73771	5.7553	0.0012	0.00681	-1.3548
TEX30	0.79247	2.24647	9.27078	8.93E-05	0.00124	1.59279
CHMP2B	0.79326	3.7456	8.1754	0.00018	0.00194	0.79278
ITGB8	0.79475	5.49409	3.95338	0.00752	0.02523	-3.4144
SH3BP4	0.79499	4.84534	3.3461	0.01551	0.04335	-4.2133
N4BP2L2	0.79528	3.02665	4.61556	0.00364	0.01482	-2.6038
RPL21	0.79605	1.05477	5.01281	0.00243	0.01103	-2.1482
EIF3C	0.79646	-0.0025	5.74892	0.00121	0.00684	-1.3613
PANK1	0.79693	2.15084	7.93523	0.00021	0.00214	0.60488
CAMKMT	0.79718	2.17645	5.6941	0.00127	0.00711	-1.4174
RFC3	0.79777	3.66126	7.88538	0.00022	0.00219	0.56527
NTN1	0.79784	2.72749	6.35939	0.00071	0.00469	-0.7607
WDR90	0.79885	2.65224	9.88934	6.19E-05	0.00098	2.00761
ILRUNP1	0.79889	-0.0161	4.00993	0.00705	0.02407	-3.3426
TGDS	0.79961	0.92334	7.44206	0.0003	0.00268	0.20336
KLHL5	0.80171	3.60306	9.96452	5.93E-05	0.00096	2.05639
ANKRD10	0.80196	1.7633	3.49225	0.01296	0.03797	-4.0164
LIPG	0.80245	4.86983	4.95185	0.00258	0.01154	-2.2167
PTPRU	0.80279	2.11028	5.19265	0.00203	0.00978	-1.9492
NEK3	0.80308	-0.6817	7.56254	0.00028	0.00254	0.30347
GSTM3	0.8031	4.062	5.56368	0.00143	0.00773	-1.5526
RNASEH2	0.80339	2.28519	5.25665	0.00191	0.00938	-1.8795
DSTN	0.80339	5.68882	9.76484	6.66E-05	0.00103	1.92608
DCUN1D2	0.8039	2.49894	6.7632	0.00051	0.00376	-0.3869
GMDS	0.80539	5.23816	5.43668	0.00161	0.00836	-1.6863
LETM2	0.80584	1.81037	5.85792	0.0011	0.00638	-1.2507

IQCK	0.80676	1.30646	4.36591	0.00475	0.01791	-2.9018
H2AC25	0.80697	0.7697	3.22317	0.01808	0.04861	-4.3809
EBPL	0.80719	2.20868	9.90192	6.15E-05	0.00098	2.0158
NRL	0.80731	-2.0144	4.86401	0.00282	0.01235	-2.3162
RNF5	0.80755	3.12417	6.26916	0.00077	0.00495	-0.8467
PICK1	0.80813	2.54666	8.23612	0.00017	0.00189	0.83953
WEE1	0.80964	3.08595	4.79444	0.00302	0.01298	-2.3959
ZNF581	0.80968	3.46912	10.6578	4.04E-05	0.00076	2.49061
H1-10-AS	0.80998	-0.8005	4.51517	0.00404	0.01597	-2.7226
F12	0.81013	1.03001	8.34051	0.00016	0.00181	0.91921
MZF1	0.81061	3.09266	6.56873	0.0006	0.00417	-0.5647
MPND	0.81106	1.95649	8.61349	0.00014	0.00161	1.12352
PRKX	0.81346	3.56827	8.31959	0.00016	0.00182	0.90331
ZNF823	0.81395	1.18808	4.63154	0.00358	0.01465	-2.5851
PIBF1	0.81473	1.42415	8.12575	0.00019	0.00198	0.75433
MTHFD2	0.81489	5.33761	3.31295	0.01616	0.04474	-4.2583
MYO18A	0.81578	3.38456	7.61509	0.00027	0.00248	0.34672
LIG4	0.81718	2.6467	6.23158	0.00079	0.00506	-0.8828
FNDC3A	0.8173	3.75057	10.8044	3.73E-05	0.00072	2.57897
IFFO2	0.81738	2.02779	6.29072	0.00075	0.00488	-0.826
AKNA	0.81744	1.80244	3.60749	0.01128	0.03412	-3.8632
RAB11FIF	0.81759	4.45651	6.83373	0.00048	0.00363	-0.3234
BCL2L12	0.8179	3.74639	8.76666	0.00012	0.00152	1.23566
CAPN1	0.81825	5.59295	9.58923	7.38E-05	0.0011	1.80942
DCAF11	0.81871	3.80751	6.64328	0.00056	0.00402	-0.496
HMGB1P	0.81878	6.72216	9.83928	6.37E-05	0.001	1.97494
MRPS24	0.81891	-0.4277	5.72593	0.00123	0.00696	-1.3848
STX5	0.81935	3.45634	8.57817	0.00014	0.00164	1.0974
DEK	0.81991	6.99915	7.78911	0.00024	0.00229	0.48817
MCAM	0.82019	5.16661	4.86742	0.00281	0.01233	-2.3123
USP12PX	0.82207	0.61386	4.13706	0.00611	0.02166	-3.1831
ESYT2	0.82226	4.78984	11.0976	3.20E-05	0.00066	2.75237
PPP1R11	0.82243	-0.5001	3.41632	0.01422	0.04068	-4.1184
IQCA1	0.8234	-2.0106	5.18427	0.00205	0.00983	-1.9584
LARP1BF	0.82346	0.00623	3.68594	0.01027	0.03173	-3.7599
KLF13	0.82408	3.85059	9.12341	9.78E-05	0.00131	1.49023
ZNF844	0.82444	0.38928	7.641	0.00026	0.00244	0.36795
ZNF836	0.82497	0.38018	4.36328	0.00476	0.01794	-2.905
RPL7L1P	0.82543	1.0739	6.20847	0.00081	0.00514	-0.905
PSTPIP2	0.82616	0.37131	7.66002	0.00026	0.00243	0.3835
INPP5J	0.82792	0.58763	5.68363	0.00128	0.00715	-1.4282
SPRY2	0.82936	2.54187	5.8517	0.0011	0.0064	-1.257
STPG1	0.83104	1.56558	5.94504	0.00101	0.00602	-1.1634
TMEM102	0.83135	2.82348	9.21125	9.27E-05	0.00126	1.55154
HABP4	0.83161	2.22465	6.78416	0.0005	0.00372	-0.368
YIPF3	0.83215	4.86501	9.44677	8.03E-05	0.00115	1.71333
PPP1R15	0.83278	4.17605	3.64496	0.01078	0.03294	-3.8138
GAS6-DT	0.83451	-0.2778	3.78306	0.00916	0.02914	-3.6333
INAFM1	0.83563	2.19197	3.37435	0.01498	0.04225	-4.175
MMP7	0.83583	5.77097	4.36363	0.00476	0.01794	-2.9046
PRICKLE	0.83605	4.30711	5.37189	0.00171	0.0087	-1.7553
KLHL24	0.83643	2.24105	3.80736	0.0089	0.02851	-3.6018
CCDC9	0.83645	1.45721	9.25364	9.03E-05	0.00124	1.58093
ISOC2	0.83791	5.66759	9.43221	8.10E-05	0.00116	1.70344
USP53	0.83841	2.16066	6.27154	0.00077	0.00494	-0.8444
TMCC3	0.83949	1.78432	4.67775	0.00341	0.01413	-2.531
LGALS8	0.83957	3.1601	3.65814	0.01061	0.03256	-3.7964
PLAGL1	0.83965	3.64639	4.65007	0.00351	0.01445	-2.5634

FIZ1	0.83981	3.34586	7.43273	0.00031	0.00269	0.19556
H4C3	0.83993	-0.2378	5.28881	0.00185	0.00917	-1.8447
WNK2	0.84113	2.6483	6.79864	0.0005	0.00369	-0.3549
BMPR1A	0.84302	3.45224	5.59913	0.00138	0.00756	-1.5156
MYLK4	0.84407	-1.2859	3.81781	0.00879	0.02829	-3.5883
PROC	0.84493	-2.1512	3.87756	0.0082	0.02686	-3.5113
ZSWIM9	0.84586	0.83237	8.39476	0.00016	0.00178	0.96027
NFE2L3	0.84611	3.92903	7.46131	0.0003	0.00266	0.21945
S100A3	0.84637	1.28727	3.41806	0.01419	0.0406	-4.116
KLLN	0.84658	-0.8627	4.97304	0.00252	0.01136	-2.1928
DCTPP1	0.848	5.05563	6.87477	0.00047	0.00354	-0.2867
RAB6A	0.84876	5.02942	5.48206	0.00154	0.00809	-1.6383
PRDX2P4	0.84894	2.14188	4.47687	0.00421	0.01643	-2.7683
ARID5A	0.84897	1.40299	5.41921	0.00164	0.00844	-1.7048
ELF1	0.84942	2.90267	9.83387	6.39E-05	0.001	1.9714
TMEM63A	0.84948	2.28057	6.62381	0.00057	0.00406	-0.5139
SHISA9	0.8498	2.60313	3.2876	0.01668	0.04571	-4.2928
SUGT1	0.85049	2.94002	6.41728	0.00068	0.00453	-0.706
RBM26	0.85079	4.13769	9.2594	9.00E-05	0.00124	1.58492
LAYN	0.85142	1.63475	3.49833	0.01286	0.03774	-4.0083
ABHD16A	0.85191	-1.6149	6.98899	0.00043	0.00334	-0.1855
RAP1GAP	0.85385	1.52293	3.63483	0.01091	0.03325	-3.8271
COL9A2	0.85385	3.93206	4.61237	0.00365	0.01485	-2.6076
TSPAN5	0.85413	0.43542	5.77138	0.00118	0.00675	-1.3384
FCF1P8	0.85426	1.21075	6.49748	0.00063	0.00433	-0.6308
MICU2	0.85434	2.26427	10.2852	4.95E-05	0.00086	2.26066
PHEX	0.85495	-2.4923	3.75708	0.00944	0.02976	-3.667
MED21	0.85575	2.74065	8.95568	0.00011	0.0014	1.37168
STK11IP	0.85589	2.3761	8.00153	0.0002	0.00209	0.65723
PLEKHG3	0.85628	3.95492	5.3684	0.00172	0.00871	-1.7591
KHK	0.85874	-0.5978	3.56175	0.01191	0.03554	-3.9238
FUZ	0.85919	2.54588	7.64366	0.00026	0.00244	0.37012
RSPH3	0.8596	1.89938	8.74743	0.00012	0.00153	1.22168
ZNF385B	0.86047	-1.9346	6.52492	0.00062	0.00427	-0.6053
ANKS6	0.86189	3.49551	10.9985	3.37E-05	0.00068	2.69425
NKD1	0.86291	-1.4425	6.91142	0.00045	0.00347	-0.2541
TPT1	0.86323	7.2719	5.26358	0.0019	0.00933	-1.872
MRPL2	0.86381	4.31975	7.25765	0.00035	0.0029	0.0475
STAP2	0.86474	2.16892	5.32162	0.0018	0.00896	-1.8093
ANKRD10	0.86522	3.73998	8.93819	0.00011	0.00141	1.3592
NIPAL3	0.86561	3.50917	4.80134	0.003	0.01292	-2.3879
NUFIP1	0.86591	0.94696	6.41688	0.00068	0.00453	-0.7064
CLN5	0.86695	1.28159	6.93463	0.00045	0.00343	-0.2335
ZNF460	0.8676	0.1142	4.05934	0.00666	0.02315	-3.2804
SACS	0.86855	3.74572	7.35456	0.00032	0.00276	0.12981
DLEU2	0.86889	0.45228	6.77354	0.00051	0.00374	-0.3775
NIPSNAP	0.86915	4.68227	5.27782	0.00187	0.00923	-1.8566
ACSL3	0.86953	4.20981	10.9861	3.39E-05	0.00068	2.687
NFKB2	0.87042	3.2546	3.62529	0.01104	0.03355	-3.8397
ADAM19	0.87074	0.67701	4.56231	0.00385	0.0154	-2.6666
TTC7A	0.87113	3.39152	9.1239	9.78E-05	0.00131	1.49058
CCDC186	0.87118	3.62118	8.78682	0.00012	0.00151	1.25029
NHLRC3	0.87129	2.18286	5.48913	0.00153	0.00806	-1.6308
ZBED9	0.87232	0.46432	4.98709	0.00249	0.01125	-2.177
ZMYM2	0.87271	4.00031	5.67864	0.00129	0.00717	-1.4333
UPK2	0.8736	-0.9118	3.72225	0.00984	0.0307	-3.7124
MYO1E	0.87387	3.52673	8.29281	0.00017	0.00184	0.88291
CORO2A	0.87391	1.45801	7.69259	0.00025	0.0024	0.41004

SIX2	0.87612	0.87408	4.10354	0.00634	0.02228	-3.2249
NAA16	0.87742	1.73886	11.6025	2.48E-05	0.00056	3.04087
MAMSTR	0.87942	-1.0002	3.22835	0.01796	0.04841	-4.3738
NOP53	0.87947	2.9497	5.80976	0.00114	0.00659	-1.2994
ANO8	0.88009	1.97504	4.56493	0.00384	0.01539	-2.6635
TENM3	0.88022	5.19484	5.92426	0.00103	0.00611	-1.1842
TBC1D4	0.88102	1.64128	3.27446	0.01695	0.04623	-4.3108
C15orf39	0.88248	3.83771	4.13829	0.0061	0.02164	-3.1816
EGLN3	0.88549	4.12478	6.38525	0.0007	0.00462	-0.7362
TP53	0.88592	4.65045	6.80577	0.00049	0.00368	-0.3485
SLC37A1	0.88618	2.0982	7.14668	0.00038	0.00309	-0.0479
SUGT1P2	0.88882	2.6706	4.91452	0.00268	0.01189	-2.2588
ZNF69	0.88999	-1.5316	4.75303	0.00315	0.01335	-2.4436
LCA5	0.89097	0.4302	7.00307	0.00042	0.00332	-0.1731
TLE1	0.89099	4.59575	4.33193	0.00492	0.0184	-2.9431
TMC7	0.89159	-0.8571	4.08352	0.00648	0.02269	-3.25
INTS13	0.89223	4.48514	8.08273	0.00019	0.00202	0.72085
ICA1L	0.89251	-0.3072	4.81158	0.00297	0.01283	-2.3762
IRAK4	0.8932	2.17745	7.86417	0.00022	0.00221	0.54835
GPSM2	0.89325	2.61802	6.51478	0.00063	0.00429	-0.6147
ABCC4	0.89347	3.14068	5.52083	0.00149	0.00793	-1.5975
DLG1	0.89437	4.50711	5.68337	0.00128	0.00715	-1.4285
HMGB1P(	0.89467	5.21541	10.3641	4.74E-05	0.00084	2.31002
SELENOV	0.89534	-0.0656	3.78889	0.0091	0.02898	-3.6257
STK19	0.89573	1.34967	9.57075	7.46E-05	0.0011	1.79703
GPT2	0.89612	5.33285	3.89722	0.00802	0.0264	-3.4861
RIGI	0.89621	2.36051	5.14014	0.00214	0.01015	-2.0069
SOX18	0.89651	-1.0605	4.85147	0.00285	0.01245	-2.3305
STK3	0.89683	4.37444	4.58143	0.00377	0.01521	-2.644
PPP1R11	0.89709	4.94077	11.9885	2.05E-05	0.00051	3.25328
RPL13A	0.89758	5.20985	10.3011	4.91E-05	0.00086	2.27064
MYH9	0.89777	8.02871	3.77787	0.00921	0.02925	-3.64
FAM110A	0.90046	1.55236	7.45425	0.0003	0.00267	0.21355
REV3L	0.90175	3.76907	7.23048	0.00036	0.00294	0.02427
MTCH1P1	0.90246	4.32749	6.28132	0.00076	0.00491	-0.835
RBMS2	0.90528	4.02835	5.93566	0.00102	0.00605	-1.1728
SOGA1	0.90582	4.44724	6.42185	0.00067	0.00453	-0.7017
PLCB1	0.90653	2.88167	8.32844	0.00016	0.00182	0.91004
UPK3B	0.907	5.81996	3.67212	0.01044	0.03215	-3.778
NFE2L3P	0.90702	0.66587	5.22805	0.00196	0.00953	-1.9106
UGDH	0.90712	4.07471	6.07729	0.0009	0.00556	-1.0327
LATS2	0.90759	2.67357	8.45823	0.00015	0.00173	1.00802
PNPLA4	0.90956	1.11166	6.35386	0.00071	0.00471	-0.7659
MTMR12	0.91094	3.42538	10.7399	3.86E-05	0.00074	2.54022
METRN	0.91215	3.75157	3.9638	0.00743	0.02501	-3.4011
LINC0267	0.91239	-2.719	3.74048	0.00963	0.03019	-3.6886
ZBTB22	0.91247	3.25052	5.27831	0.00187	0.00923	-1.856
POLH	0.91265	3.19669	7.40128	0.00031	0.00271	0.16918
ZSCAN12	0.91364	1.39736	10.5854	4.20E-05	0.00078	2.44654
PHLPP1	0.91414	0.32838	10.3433	4.79E-05	0.00084	2.29701
TRPM4	0.9142	2.34268	7.29448	0.00034	0.00284	0.07889
TST	0.91435	2.96965	5.89983	0.00106	0.0062	-1.2086
AFAP1	0.91451	3.96379	10.4236	4.59E-05	0.00082	2.34699
SMTN	0.9149	2.8991	8.0838	0.00019	0.00202	0.72168
BRCA2	0.91663	1.88524	7.23235	0.00036	0.00294	0.02587
EYA3	0.91669	3.27912	6.18591	0.00082	0.00522	-0.9269
TIAM2	0.91705	1.36292	3.38455	0.01479	0.04181	-4.1612
PLXNB1	0.91709	4.22169	6.13526	0.00086	0.00538	-0.976

MINAR1	0.91838	-1.2423	3.66972	0.01047	0.03222	-3.7812
PDXK	0.91852	5.86865	7.11779	0.00039	0.00313	-0.0729
ZNF649	0.91882	1.44957	8.32459	0.00016	0.00182	0.90712
COG3	0.92059	2.01467	6.30041	0.00075	0.00486	-0.8168
YIPF2	0.92145	4.40043	7.23223	0.00036	0.00294	0.02576
TPCN1	0.9216	4.72214	4.48343	0.00418	0.01635	-2.7604
APOL1	0.92238	5.05509	4.26785	0.00528	0.01938	-3.0214
CDKAL1	0.92451	5.22773	11.7374	2.32E-05	0.00055	3.11589
TERC	0.92492	-0.1289	7.89036	0.00022	0.00218	0.56923
MEGF6	0.92547	1.55275	3.8494	0.00848	0.02755	-3.5475
C1QTNF1	0.92617	-1.3507	4.49463	0.00413	0.0162	-2.7471
PRDX2	0.92673	6.4503	8.48148	0.00015	0.00171	1.02544
OBI1	0.92768	1.86589	8.87307	0.00011	0.00145	1.31255
SPATA13	0.92893	2.04974	8.43661	0.00015	0.00174	0.99179
RHOBTB3	0.93026	6.14183	8.21554	0.00018	0.00191	0.82372
PTPN6	0.93039	1.67418	8.17588	0.00018	0.00194	0.79315
NUDT3	0.931	4.08004	9.21534	9.24E-05	0.00126	1.55438
STING1	0.93203	5.69263	4.96085	0.00255	0.01146	-2.2065
TMBIM1	0.93282	4.64253	6.59169	0.00059	0.00412	-0.5435
KIFC1	0.93285	4.90246	6.01337	0.00096	0.00578	-1.0956
SUPT20H	0.93352	3.34673	11.0045	3.36E-05	0.00068	2.6978
HACD1	0.93359	3.27647	11.7299	2.33E-05	0.00055	3.11176
RFXANK	0.93599	5.08564	4.75149	0.00316	0.01336	-2.4454
ING1	0.93688	3.52434	8.83354	0.00012	0.00148	1.28408
PSORS1C	0.93806	-0.4125	6.86297	0.00047	0.00357	-0.2972
MINDY2	0.93863	2.8407	5.67508	0.00129	0.00718	-1.437
TBC1D2	0.93867	2.93254	6.47808	0.00064	0.00438	-0.6489
UBR2	0.94126	3.58261	12.3651	1.71E-05	0.00046	3.45414
WBP4	0.94132	1.33139	8.64961	0.00013	0.00159	1.15012
APOBEC3	0.94218	2.74655	4.89704	0.00272	0.01204	-2.2787
ITGB5	0.94232	4.81358	6.90481	0.00046	0.00348	-0.2599
BTBD3	0.94334	4.12799	6.93781	0.00045	0.00342	-0.2307
FLOT1	0.94345	5.69036	7.24484	0.00035	0.00293	0.03655
EMP2	0.94383	2.91784	6.40367	0.00069	0.00457	-0.7188
MDC1	0.94407	4.20547	10.0043	5.80E-05	0.00095	2.08203
GPALPP1	0.94456	2.34068	10.644	4.07E-05	0.00076	2.48223
ILRUN	0.94701	5.00396	10.2464	5.06E-05	0.00088	2.23624
IDH2	0.94828	5.47175	4.66559	0.00345	0.01426	-2.5452
NUP58	0.95043	4.01325	9.91	6.12E-05	0.00098	2.02105
RAI14	0.95205	6.39007	10.1526	5.33E-05	0.0009	2.17693
RHBDD1	0.95208	1.50006	10.368	4.73E-05	0.00084	2.31241
TPT1P6	0.95388	1.30568	3.97998	0.00729	0.02468	-3.3806
BICRAL	0.95461	2.599	7.34814	0.00033	0.00277	0.12439
SHROOM	0.9548	6.07066	4.06201	0.00664	0.0231	-3.277
SRF	0.95484	4.70968	4.70396	0.00332	0.01387	-2.5005
MESP1	0.95585	-1.1493	3.29385	0.01655	0.04546	-4.2843
NR2F6	0.95695	5.72178	10.2412	5.07E-05	0.00088	2.23297
CUL3	0.95812	3.76451	9.91261	6.11E-05	0.00098	2.02274
ZNF132-D	0.95928	-0.5543	5.56744	0.00143	0.00771	-1.5487
ABAT	0.95959	-2.0502	5.58156	0.00141	0.00764	-1.5339
LRRC61	0.9618	3.61365	5.33452	0.00177	0.00889	-1.7954
NFIX	0.96197	3.72562	6.51595	0.00062	0.00429	-0.6136
ATXN1	0.96246	3.79914	4.4924	0.00414	0.01623	-2.7497
SLC1A4	0.96259	1.93355	5.23703	0.00195	0.00949	-1.9008
AIFM3	0.96356	0.33408	6.31917	0.00074	0.00481	-0.7989
ARHGEF1	0.96413	-0.0948	3.70101	0.01009	0.03128	-3.7402
FKBP4P1	0.96475	1.3839	3.41982	0.01416	0.04056	-4.1136
HYI	0.96495	2.44174	4.99471	0.00247	0.01119	-2.1685

HECTD1	0.96521	5.64231	8.64998	0.00013	0.00159	1.15039
ABHD11	0.96538	4.41807	7.02947	0.00041	0.00328	-0.1499
CAMK1D	0.9671	0.66456	4.84977	0.00286	0.01246	-2.3325
POU5F1	0.9684	0.42749	4.18379	0.0058	0.0208	-3.125
MTHFD2I	0.97012	3.94706	4.93032	0.00263	0.01173	-2.241
SLC29A2	0.97126	4.53821	6.94618	0.00044	0.00342	-0.2232
POGLUT	0.97171	0.60088	10.3102	4.88E-05	0.00086	2.27634
UCHL3	0.97205	0.29295	8.66707	0.00013	0.00158	1.16295
INTS6P1	0.97234	1.70416	6.79501	0.0005	0.0037	-0.3582
HM13-IT1	0.97315	0.20073	4.41533	0.0045	0.01721	-2.8421
LGALS3	0.97329	3.86573	8.59938	0.00014	0.00162	1.1131
STK24	0.97384	3.93519	10.4537	4.51E-05	0.00082	2.36561
OTUD3	0.97654	3.08049	5.51223	0.0015	0.00796	-1.6065
IL17RB	0.97668	0.39152	5.08474	0.00226	0.0105	-2.0681
MCF2L	0.97821	1.49671	8.9965	0.00011	0.00138	1.40071
CLIP2	0.97878	2.36178	3.57769	0.01169	0.03506	-3.9027
BCAR3	0.97886	3.51787	5.65093	0.00132	0.00731	-1.4619
FBXL3	0.97926	2.39216	7.44795	0.0003	0.00268	0.20829
TACC2	0.9798	2.17448	7.45685	0.0003	0.00266	0.21572
LTBP1	0.9803	2.02873	4.36813	0.00474	0.01789	-2.8992
EGFR	0.98044	5.06577	3.4544	0.01357	0.03934	-4.0671
LRCH1	0.98135	2.27102	9.21851	9.22E-05	0.00126	1.55659
TPT1P4	0.98172	4.8329	4.32136	0.00498	0.01856	-2.956
EBF4	0.98183	1.1488	3.79157	0.00907	0.02892	-3.6222
KBTBD6	0.98261	2.48552	4.72751	0.00324	0.01361	-2.4731
ZNF556	0.98371	0.6668	5.25235	0.00192	0.0094	-1.8842
CSRP1	0.98501	6.60661	7.37016	0.00032	0.00274	0.14298
PNKD	0.98719	4.43057	6.23369	0.00079	0.00506	-0.8807
KRT18P3	0.98764	2.95746	3.87462	0.00823	0.02693	-3.5151
NCOA3	0.98771	4.84624	3.55839	0.01196	0.03566	-3.9283
RAB4A	0.98844	2.71572	11.391	2.76E-05	0.0006	2.92152
ZNF185	0.98904	2.51906	4.96299	0.00255	0.01144	-2.2041
SMIM14	0.98969	2.25205	5.20235	0.00201	0.00972	-1.9386
SLC25A2	0.99001	5.09003	8.77847	0.00012	0.00151	1.24424
ZNF331	0.99016	3.59165	5.13131	0.00216	0.0102	-2.0166
ACTG1P2	0.9923	-1.5981	4.62518	0.0036	0.01472	-2.5925
HRH1	0.99248	1.76418	7.7129	0.00025	0.00238	0.42655
B4GALNT	0.99287	2.44288	7.56479	0.00028	0.00254	0.30532
HOXB5	0.99288	2.80138	5.23011	0.00196	0.00952	-1.9083
MAL	0.99438	-0.2498	4.27516	0.00524	0.01926	-3.0125
ERCC5	0.99525	0.7143	7.94973	0.00021	0.00212	0.61636
ARHGEF	0.99614	0.66028	5.01855	0.00241	0.01101	-2.1418
CERS4	0.99639	2.63789	3.89848	0.00801	0.02639	-3.4845
ADCY10F	0.99842	-1.0205	7.40672	0.00031	0.00271	0.17375
STK38	0.99842	4.01053	12.3298	1.74E-05	0.00046	3.43558
GPC1	0.9992	2.96087	4.5142	0.00405	0.01597	-2.7237
SNN	1.00179	3.12775	5.39306	0.00168	0.00857	-1.7327
TAP1	1.00219	2.30349	3.58263	0.01162	0.03491	-3.8961
MFF	1.00256	3.1181	9.98197	5.87E-05	0.00096	2.06766
EPAS1	1.00377	4.75416	4.43158	0.00442	0.01701	-2.8226
CENPJ	1.00409	2.32045	10.815	3.71E-05	0.00072	2.58534
FAM172A	1.00531	1.72351	8.5954	0.00014	0.00162	1.11016
IFT88	1.00563	0.33472	6.89706	0.00046	0.0035	-0.2668
AKAP11	1.00586	3.38447	8.79907	0.00012	0.0015	1.25917
CCNE1	1.00645	5.82798	3.76022	0.00941	0.0297	-3.6629
RNF6	1.00683	2.82857	12.6049	1.53E-05	0.00042	3.57893
S1PR2	1.00939	1.83276	8.50868	0.00014	0.00169	1.04576
ARSI	1.00978	-1.8338	3.72596	0.00979	0.03059	-3.7076

KRT18P1	1.01098	3.02952	4.90958	0.00269	0.01193	-2.2644
PRDX2P1	1.01225	2.62847	10.9513	3.46E-05	0.00069	2.66641
DOCK5	1.01242	2.27862	6.28208	0.00076	0.00491	-0.8343
RHBDD2	1.01262	3.26119	5.34259	0.00176	0.00884	-1.7867
SAP18	1.01398	4.46774	8.79722	0.00012	0.0015	1.25783
EML2	1.01402	3.53982	3.36889	0.01508	0.04247	-4.1824
LRCH2	1.01455	2.28524	4.55495	0.00388	0.01547	-2.6754
DLX5	1.01573	1.99913	4.58001	0.00378	0.01523	-2.6457
PM20D2	1.01641	3.77204	7.56433	0.00028	0.00254	0.30495
PORCN	1.01672	4.22744	3.96437	0.00742	0.02501	-3.4004
ANKDD1A	1.01768	-0.2222	5.56543	0.00143	0.00772	-1.5507
TBC1D22	1.01913	1.97944	6.26892	0.00077	0.00495	-0.8469
EDNRA	1.01931	-1.2451	3.95555	0.0075	0.0252	-3.4116
AP1S3	1.02073	0.46398	4.8313	0.00291	0.01263	-2.3536
DMC1	1.0235	-1.0863	5.31889	0.0018	0.00898	-1.8122
HECA	1.02375	1.86294	8.33046	0.00016	0.00182	0.91158
KHDRBS3	1.0239	1.72153	4.12306	0.0062	0.02193	-3.2005
MZT1	1.02508	3.29944	10.0711	5.58E-05	0.00093	2.12494
NACC2	1.02574	2.50613	4.74232	0.00319	0.01346	-2.456
ANKRD24	1.02642	-2.3205	10.2268	5.11E-05	0.00088	2.22388
NKX1-2	1.02672	1.73393	4.1611	0.00594	0.02118	-3.1532
GATA5	1.02702	-1.6218	3.22636	0.01801	0.0485	-4.3766
DLX6-AS	1.02839	1.56517	10.6856	3.98E-05	0.00075	2.50747
MYH14	1.02925	5.68872	7.9475	0.00021	0.00212	0.61459
TBC1D19	1.03149	0.77298	6.99734	0.00043	0.00333	-0.1781
LAMA5	1.03181	6.74172	5.51726	0.00149	0.00795	-1.6012
CSPG4	1.03329	-0.7046	3.31256	0.01617	0.04474	-4.2589
PROSER	1.03552	1.66361	4.71412	0.00328	0.01377	-2.4887
TMEM231	1.03682	0.79301	6.83801	0.00048	0.00362	-0.3196
DNASE2	1.03711	4.6857	4.59902	0.0037	0.01501	-2.6233
CRYL1	1.03802	0.7598	4.813	0.00297	0.01282	-2.3746
DNAJC3	1.03803	3.40524	12.3026	1.77E-05	0.00046	3.42122
PBX4	1.03976	-0.1552	8.65349	0.00013	0.00159	1.15297
ABLIM1	1.04091	5.55354	4.88713	0.00275	0.01212	-2.2899
HSD3B7	1.04148	0.51257	10.1849	5.24E-05	0.00089	2.19743
DSTNP3	1.04255	1.87608	5.62268	0.00136	0.00745	-1.4912
PLXNA2	1.04286	1.21895	3.26835	0.01708	0.04655	-4.3191
DHRS1	1.04319	1.14319	5.01804	0.00241	0.01101	-2.1423
NFKBID	1.0448	1.12552	5.526	0.00148	0.00791	-1.592
APOLD1	1.04592	0.47448	6.73362	0.00052	0.00383	-0.4137
MST1	1.04595	-1.5377	3.31343	0.01615	0.04473	-4.2577
NOTCH3	1.04678	4.02338	4.50869	0.00407	0.01601	-2.7303
RNF8	1.04693	2.63337	10.7392	3.87E-05	0.00074	2.5398
BMPR1AP	1.04792	3.18371	6.23976	0.00079	0.00504	-0.8749
ATP2B4	1.04803	4.95588	8.80408	0.00012	0.0015	1.26279
CDK8	1.04944	2.06349	10.2263	5.12E-05	0.00088	2.22358
NEDD9	1.04982	6.00226	4.93821	0.00261	0.01166	-2.232
SERINC2	1.05079	3.7929	4.7807	0.00307	0.0131	-2.4117
RDH13	1.05136	3.20015	9.47629	7.89E-05	0.00114	1.73336
NEK9	1.05175	2.55098	5.4622	0.00157	0.00821	-1.6593
TNK1	1.05281	1.09114	7.60615	0.00027	0.00249	0.33938
POLR1D	1.05283	3.75401	13.485	1.04E-05	0.00033	4.01728
PDS5B	1.0533	3.26245	13.4138	1.07E-05	0.00034	3.98293
PAN3	1.05639	2.48548	7.72734	0.00025	0.00236	0.43826
PRKCH	1.0569	1.9388	4.93862	0.00261	0.01166	-2.2316
HMGB1	1.05697	5.53408	11.4381	2.69E-05	0.00059	2.94828
IFNLR1	1.05873	0.86404	7.76888	0.00024	0.00231	0.47186
MAP9	1.05985	1.83082	5.00801	0.00244	0.01107	-2.1536

LRRC8D	1.05985	3.58221	6.95296	0.00044	0.0034	-0.2172
MYCL	1.0604	-0.4434	3.20854	0.01842	0.04932	-4.401
ENDOD1	1.06661	3.16607	6.63137	0.00057	0.00404	-0.507
MIPEP	1.0671	1.4153	7.78492	0.00024	0.00229	0.48479
TOGARA	1.06729	0.39029	6.72402	0.00053	0.00385	-0.4224
PARP4	1.06912	3.73467	8.79099	0.00012	0.00151	1.25332
PRECSIT	1.06935	1.26811	4.37979	0.00468	0.01769	-2.885
OPLAH	1.06984	3.46554	4.40096	0.00457	0.01739	-2.8595
TDRD3	1.07001	-0.9683	6.04907	0.00093	0.00565	-1.0604
VPREB3	1.07313	-1.0785	3.92897	0.00773	0.02575	-3.4455
MAT1A	1.07555	-2.0101	9.76117	6.67E-05	0.00103	1.92366
LINC0298	1.07585	1.24935	4.2075	0.00565	0.02036	-3.0957
PXK	1.0764	3.34996	3.57788	0.01168	0.03506	-3.9024
CD2AP	1.07904	5.23028	13.5746	9.96E-06	0.00032	4.0603
COLGALT	1.07945	-1.1974	5.10761	0.00221	0.01035	-2.0428
ATP7B	1.08142	0.16239	4.87942	0.00277	0.0122	-2.2987
ZNF93	1.08288	-1.1708	5.09259	0.00224	0.01045	-2.0594
RPL21P1	1.08313	3.7425	7.85099	0.00023	0.00222	0.53782
CD99P1	1.08598	-0.6849	10.0661	5.60E-05	0.00093	2.12174
MEST	1.08606	4.58582	13.3847	1.08E-05	0.00034	3.96881
VEGFA	1.08662	4.82553	3.60509	0.01131	0.03421	-3.8664
ERMP1	1.08698	4.86718	7.47088	0.0003	0.00264	0.22743
ATF4P4	1.089	1.9951	4.19982	0.00569	0.0205	-3.1052
IRF3	1.09017	4.68673	14.4134	7.02E-06	0.00026	4.44927
MYLK3	1.09114	-0.6693	6.28883	0.00075	0.00489	-0.8279
LYN	1.09347	3.23142	6.78486	0.0005	0.00372	-0.3673
HPDL	1.09396	1.62542	5.35191	0.00174	0.00878	-1.7767
MESTP3	1.09554	1.65597	8.03315	0.0002	0.00206	0.68207
RAB3D	1.09656	3.86101	6.49443	0.00064	0.00434	-0.6337
CDR2L	1.09689	4.83933	4.57781	0.00378	0.01524	-2.6483
WHRN	1.09757	0.83996	4.36057	0.00477	0.01797	-2.9083
DIS3	1.10398	3.12899	8.28729	0.00017	0.00185	0.87869
PMEL	1.10483	0.56019	6.35124	0.00072	0.00472	-0.7684
OSR2	1.10511	0.63427	8.33034	0.00016	0.00182	0.91149
CD2AP-D	1.10569	0.23697	6.00878	0.00096	0.00579	-1.1002
PPARGC	1.10688	1.76443	3.35968	0.01525	0.04278	-4.1949
SIM1	1.10886	-2.1191	3.42182	0.01412	0.04049	-4.111
RBMS2P1	1.10892	3.78516	5.67602	0.00129	0.00718	-1.436
BMPR1AI	1.11052	2.98452	5.49336	0.00153	0.00805	-1.6264
MIR3936I	1.11134	-0.5419	9.02657	0.0001	0.00136	1.42203
THOC3	1.11176	4.30241	6.32533	0.00073	0.00479	-0.793
HEY2	1.1129	2.4179	4.42183	0.00447	0.01713	-2.8343
PELI2	1.11342	1.12402	4.21552	0.0056	0.02024	-3.0858
CEMIP2	1.11462	4.57758	7.67517	0.00026	0.00242	0.39585
DDX39B	1.11569	3.99758	10.4229	4.59E-05	0.00082	2.34652
MYCBP2	1.11686	3.68186	11.4972	2.61E-05	0.00058	2.98171
DGKH	1.11912	3.62514	9.26269	8.98E-05	0.00124	1.58719
ZNF778	1.1193	3.01236	13.0082	1.28E-05	0.00037	3.78351
GALNT18	1.12117	0.16192	4.02219	0.00695	0.02384	-3.3272
P4HTM	1.12179	1.76739	6.64159	0.00056	0.00402	-0.4976
PDLIM7	1.12215	5.98481	10.3649	4.74E-05	0.00084	2.31051
RBMS3	1.1225	3.87706	6.41733	0.00068	0.00453	-0.7059
COL9A3	1.12259	-1.511	6.37816	0.0007	0.00464	-0.7429
CYP1A1	1.12531	-0.7289	3.25472	0.01738	0.04719	-4.3377
GRB7	1.12929	0.48154	3.49193	0.01297	0.03798	-4.0169
ARHGEF!	1.13033	2.97966	10.4261	4.58E-05	0.00082	2.34853
MESTP1	1.13047	3.98592	11.3305	2.84E-05	0.00061	2.88699
FARP1	1.1314	5.07079	9.91796	6.09E-05	0.00098	2.02622

P2RY6	1.13459	0.83116	4.52657	0.004	0.01583	-2.709
GTF2F2P	1.13479	-0.167	4.81449	0.00296	0.01281	-2.3728
UFM1P2	1.13598	1.01478	5.06884	0.00229	0.01061	-2.0857
SORL1	1.13818	3.2191	8.42117	0.00015	0.00176	0.98018
MIEF2	1.13965	1.60847	5.84533	0.00111	0.00642	-1.2634
FAM20C	1.14048	2.26168	3.23687	0.01777	0.04798	-4.3622
S100A2	1.14159	2.34766	8.23605	0.00017	0.00189	0.83948
LRRC56	1.14234	-1.0323	6.8586	0.00047	0.00357	-0.3011
JDP2	1.14318	2.63554	5.31109	0.00181	0.00902	-1.8206
RB1	1.1434	4.07632	10.8478	3.65E-05	0.00071	2.60491
ARRDC1	1.14387	2.94171	10.9276	3.50E-05	0.0007	2.65242
RCBTB2	1.14419	1.43387	6.80256	0.0005	0.00369	-0.3514
CMTM4	1.14587	3.34216	11.9947	2.04E-05	0.00051	3.25667
C11orf80	1.14688	0.67182	7.95789	0.00021	0.00212	0.62281
SEMA3F	1.14901	2.66759	7.07375	0.0004	0.0032	-0.1112
MFFP2	1.14958	3.0703	9.58537	7.40E-05	0.0011	1.80683
MUC3A	1.15049	-1.3504	5.31652	0.0018	0.00899	-1.8148
ALPK3	1.15168	-1.9338	5.64497	0.00133	0.00733	-1.4681
PRSS36	1.15277	-1.8833	5.20131	0.00202	0.00972	-1.9398
CD9	1.15285	5.26668	6.60315	0.00058	0.00409	-0.5329
SORT1	1.15366	3.63343	5.33792	0.00177	0.00887	-1.7917
CDKN2AI	1.15494	4.13953	12.4145	1.68E-05	0.00045	3.48008
ADHFE1	1.15537	-1.3069	6.19508	0.00082	0.00519	-0.918
TMEM191	1.15675	-0.5689	5.56418	0.00143	0.00772	-1.5521
SPNS2	1.15691	2.63559	8.94945	0.00011	0.0014	1.36724
ALDH1B1	1.1573	4.38714	12.0831	1.96E-05	0.0005	3.30435
CALD1	1.15792	6.98405	4.86244	0.00282	0.01237	-2.318
IL22RA1	1.15823	-0.3552	6.08401	0.0009	0.00554	-1.0261
IL3RA	1.15841	-1.6793	4.21805	0.00558	0.0202	-3.0827
RHOV	1.15865	0.12253	5.87499	0.00108	0.00631	-1.2336
XPO4	1.15964	3.15559	9.50867	7.74E-05	0.00113	1.75525
SLC43A2	1.15997	0.811	5.857	0.0011	0.00638	-1.2517
MIR193BI	1.16062	-1.8144	3.71375	0.00994	0.03091	-3.7235
PPP1R13	1.16265	3.13112	8.46763	0.00015	0.00172	1.01507
SKA3	1.16341	3.10319	6.1255	0.00087	0.00542	-0.9855
KSR2	1.16369	0.39755	8.38198	0.00016	0.00178	0.95062
TM9SF2	1.16491	4.81829	12.8392	1.38E-05	0.00039	3.69858
MORN3	1.16588	-1.6834	5.10203	0.00222	0.01039	-2.0489
RAB17-D	1.16634	-1.6054	3.73973	0.00964	0.0302	-3.6896
EPB41L4	1.16648	-0.3355	4.56216	0.00385	0.0154	-2.6668
CCSER1	1.16778	0.17069	4.79561	0.00302	0.01297	-2.3945
GLIS2	1.16804	1.79584	7.97186	0.00021	0.00211	0.63385
TLCD4	1.17008	2.09048	7.16952	0.00037	0.00305	-0.0281
SLC22A5	1.17119	2.51994	13.1298	1.21E-05	0.00036	3.84393
ASAP3	1.17154	2.05675	8.59777	0.00014	0.00162	1.1119
CLDN1	1.17155	4.62249	4.16306	0.00593	0.02116	-3.1507
EEF1AKM	1.17255	-0.1065	11.4385	2.69E-05	0.00059	2.94854
RAB6C	1.17339	0.52608	7.41045	0.00031	0.0027	0.17688
TMEM25	1.17368	2.28383	11.4731	2.64E-05	0.00058	2.96809
FBN1	1.17398	-1.2917	3.22334	0.01808	0.04861	-4.3807
KRT18P1	1.17436	3.20862	6.47924	0.00064	0.00438	-0.6479
SLC10A5	1.17471	-0.4064	7.61114	0.00027	0.00248	0.34348
ESD	1.17582	4.12277	13.6349	9.71E-06	0.00032	4.08903
OLFML2A	1.17688	0.53526	3.7991	0.00899	0.02873	-3.6125
CDK2AP2	1.17824	5.78035	8.2419	0.00017	0.00189	0.84397
CPAMD8	1.17863	1.87122	3.55408	0.01202	0.03579	-3.934
KBTBD7	1.18008	0.98536	7.59955	0.00027	0.0025	0.33396
PIK3IP1	1.18132	0.24113	3.44401	0.01375	0.03972	-4.0811

TNFRSF2	1.18167	3.81373	4.16454	0.00592	0.02113	-3.1489
TSC22D1	1.1822	3.97018	14.7298	6.18E-06	0.00024	4.58995
APLF	1.18362	-0.497	7.06202	0.0004	0.00322	-0.1214
IL12RB2	1.18415	-0.9023	4.5256	0.004	0.01583	-2.7102
LHFPL6	1.18477	0.59504	3.39603	0.01458	0.0414	-4.1457
PDE4DIP	1.18493	3.40211	12.4662	1.64E-05	0.00044	3.50706
HMGA2	1.18565	4.77063	6.71265	0.00053	0.00386	-0.4327
SLC7A5	1.1882	5.47853	8.3698	0.00016	0.00179	0.94141
AJAP1	1.18831	4.71691	9.95239	5.97E-05	0.00096	2.04854
TUBB3P1	1.18892	-1.0833	3.21003	0.01838	0.04925	-4.399
GLG1	1.1908	4.01331	11.2823	2.91E-05	0.00062	2.85934
TRAPPC9	1.19124	4.7873	12.387	1.70E-05	0.00045	3.46566
TMC6	1.19133	3.5745	3.61851	0.01113	0.03377	-3.8486
ZDHHC8E	1.19209	-0.8558	3.35128	0.01541	0.04312	-4.2063
IPO5	1.19262	5.13648	7.5514	0.00028	0.00255	0.29427
MTURN	1.19289	0.99011	11.4888	2.62E-05	0.00058	2.97697
KPNA3	1.1946	3.7635	12.2082	1.85E-05	0.00048	3.37123
DACT2	1.1946	1.63748	5.46893	0.00156	0.00817	-1.6521
RHOD	1.19478	2.49	12.0627	1.98E-05	0.0005	3.29336
COG6	1.19552	2.22069	6.18205	0.00083	0.00523	-0.9306
RNF165	1.19632	1.08381	5.60872	0.00137	0.00752	-1.5057
MICAL2	1.19689	4.18378	7.89367	0.00022	0.00218	0.57187
GPR180	1.19984	1.95311	12.301	1.77E-05	0.00046	3.42039
KRT18P6	1.20045	0.26494	6.50929	0.00063	0.0043	-0.6198
KRT18	1.20128	6.925	7.5133	0.00029	0.0026	0.26272
AJUBA-D	1.20151	-1.1286	8.92741	0.00011	0.00142	1.3515
SSH3	1.20179	3.07716	6.15128	0.00085	0.00533	-0.9604
BIVM	1.20248	0.4873	6.63206	0.00057	0.00404	-0.5063
MITF	1.2028	2.94026	11.8088	2.24E-05	0.00053	3.15527
LINC0148	1.20322	-1.9179	4.15787	0.00597	0.02125	-3.1572
MIR186	1.20366	-1.5376	4.24651	0.00541	0.01974	-3.0476
HEY1	1.20447	0.55926	3.20807	0.01843	0.04934	-4.4017
AVPI1	1.2052	2.63052	5.41038	0.00165	0.00849	-1.7142
YPEL1	1.20623	-1.5058	6.80992	0.00049	0.00368	-0.3448
RAB6D	1.20857	1.57487	7.92202	0.00022	0.00215	0.5944
HOMER2	1.20927	2.17551	9.43316	8.10E-05	0.00116	1.70409
POR	1.20999	4.57716	10.5839	4.20E-05	0.00078	2.44559
DMRT2	1.21012	-1.913	4.76393	0.00312	0.01327	-2.431
LEPR	1.21012	1.3397	8.00412	0.0002	0.00208	0.65927
COL5A1	1.21037	2.9698	3.5026	0.0128	0.03761	-4.0026
ANKS1A	1.21141	3.67611	7.53132	0.00028	0.00257	0.27766
DOP1B	1.21197	2.34457	7.10555	0.00039	0.00315	-0.0835
DCAF12L	1.21318	4.083	5.48225	0.00154	0.00809	-1.6381
FHDC1	1.21437	2.08644	4.09048	0.00643	0.02255	-3.2413
NDFIP2	1.21499	2.88913	13.7991	9.05E-06	0.0003	4.16673
LONRF1	1.21582	0.97333	4.79981	0.00301	0.01293	-2.3897
MTIF3	1.21654	1.5724	10.7326	3.88E-05	0.00074	2.53583
SLC20A2	1.21753	3.60514	7.35559	0.00032	0.00276	0.13068
TSPAN13	1.21782	3.48461	11.7987	2.25E-05	0.00054	3.14969
EFNB1	1.21811	2.57539	7.58737	0.00027	0.00251	0.32394
SOWAHD	1.22324	0.73731	4.51393	0.00405	0.01597	-2.7241
MFGE8	1.22482	6.21782	7.2928	0.00034	0.00284	0.07746
PPM1J	1.22707	-1.7616	5.595	0.00139	0.00758	-1.5199
PITPNM1	1.22898	5.04414	4.81534	0.00296	0.0128	-2.3719
CCDC153	1.22913	0.59454	4.27958	0.00521	0.0192	-3.007
KBTBD11	1.23115	-0.3283	4.84092	0.00288	0.01254	-2.3426
SLC7A5P	1.23188	-0.0073	6.0829	0.0009	0.00555	-1.0272
GDA	1.23565	4.90849	5.68807	0.00128	0.00713	-1.4236

RNF208	1.23797	0.9295	4.03232	0.00687	0.02364	-3.3144
MBNL2	1.23861	2.81251	5.68314	0.00128	0.00715	-1.4287
RDH10	1.2394	3.86525	8.17184	0.00018	0.00194	0.79003
OR2A1-A	1.23976	0.54387	3.72848	0.00976	0.03054	-3.7043
PLEKHA4	1.24031	2.38088	5.51675	0.00149	0.00795	-1.6017
ZNF117	1.24212	-0.1936	5.21041	0.002	0.00966	-1.9298
STARD13	1.24539	1.81815	7.41026	0.00031	0.0027	0.17671
LYPD3	1.24585	0.10383	4.65324	0.0035	0.01441	-2.5596
NT5DC2	1.25207	5.35801	14.0125	8.28E-06	0.00029	4.26631
GARRE1	1.25211	3.41245	11.7149	2.34E-05	0.00055	3.10346
REP15	1.25383	0.58563	8.33183	0.00016	0.00182	0.91262
RAP2A	1.25389	2.77345	12.8946	1.34E-05	0.00039	3.72653
AUTS2	1.25407	1.80773	4.21908	0.00557	0.02018	-3.0814
RIPPLY3	1.25463	-0.3142	4.49146	0.00415	0.01625	-2.7508
FAIM	1.25519	0.88	4.72805	0.00324	0.01361	-2.4725
ADAMTS1	1.25542	0.57714	7.87058	0.00022	0.00221	0.55347
IER5L	1.256	4.62225	6.78943	0.0005	0.00371	-0.3632
TEAD2	1.25763	4.43348	11.2189	3.01E-05	0.00064	2.82284
ARHGAP	1.25801	0.01552	5.05892	0.00232	0.0107	-2.0967
ELFN2	1.25919	2.42179	4.62408	0.00361	0.01472	-2.5938
FAT1	1.25937	3.91894	6.0767	0.0009	0.00556	-1.0333
EXOSC8	1.2619	3.36295	7.28647	0.00034	0.00285	0.07208
MAGIX	1.26197	1.76395	10.33	4.83E-05	0.00085	2.2887
MICB	1.26439	2.69086	14.6111	6.48E-06	0.00025	4.53752
ZNF525	1.26442	2.54084	10.4639	4.49E-05	0.00081	2.37189
ACBD7	1.26845	0.29796	7.20808	0.00036	0.00298	0.00506
KRT18P2	1.26939	-0.2333	6.68549	0.00054	0.00392	-0.4574
CASZ1	1.26961	0.41142	4.56283	0.00385	0.0154	-2.666
FUCA1	1.27172	2.95602	7.7433	0.00024	0.00234	0.45119
DDN	1.27232	-0.2068	5.96998	0.00099	0.00592	-1.1386
GSTO2	1.27299	1.45027	7.50673	0.00029	0.00261	0.25726
HDAC9	1.27365	5.66941	5.80937	0.00114	0.00659	-1.2998
CYP2S1	1.27379	1.97966	9.97203	5.91E-05	0.00096	2.06124
CKAP2	1.27409	4.16509	8.46844	0.00015	0.00172	1.01567
SIX1	1.27548	0.51203	5.71567	0.00124	0.007	-1.3953
CENPV	1.27657	1.45107	7.72776	0.00025	0.00236	0.4386
TPT1-AS1	1.27961	-0.0861	7.09838	0.00039	0.00315	-0.0897
SLC17A5	1.28028	2.5513	5.11674	0.00219	0.01029	-2.0327
SDC4	1.28218	5.37638	7.04333	0.00041	0.00326	-0.1378
ANXA3	1.28252	5.18536	4.77038	0.0031	0.01321	-2.4236
UFM1	1.28314	3.45317	11.8502	2.19E-05	0.00053	3.17797
SMIM24	1.28342	0.38188	3.65793	0.01062	0.03257	-3.7967
ARID3A	1.28399	1.67851	5.97767	0.00099	0.0059	-1.131
SERTAD4	1.2849	1.67939	9.00462	0.00011	0.00138	1.40647
SOX8	1.28581	-1.9944	3.50578	0.01275	0.03752	-3.9983
MXRA5	1.28581	3.93612	3.58055	0.01165	0.03499	-3.8989
KDM6A	1.28606	3.78468	14.3731	7.14E-06	0.00026	4.43108
TGFA	1.28617	0.57675	3.93361	0.00769	0.02567	-3.4396
FGFR3	1.28721	1.50085	6.57997	0.00059	0.00415	-0.5543
NELL2	1.28783	3.32747	3.76215	0.00938	0.02966	-3.6604
UBL3	1.28948	1.65531	12.3981	1.69E-05	0.00045	3.47145
CELSR3	1.29209	-0.2258	6.72806	0.00053	0.00384	-0.4187
GAS6	1.29239	2.78196	9.68061	6.99E-05	0.00106	1.87037
TMTC4	1.29243	2.73177	5.85285	0.0011	0.0064	-1.2559
IFIT5	1.29303	0.55224	4.73951	0.0032	0.01348	-2.4592
CDC42EF	1.29303	4.01706	6.67012	0.00055	0.00396	-0.4715
VASH2	1.29314	-1.9806	14.3666	7.15E-06	0.00026	4.42817
WNT5A	1.29325	3.10508	5.07664	0.00228	0.01057	-2.0771

ULK2	1.29971	-0.7015	6.80419	0.00049	0.00369	-0.3499
DEPTOR	1.30293	1.94315	8.12974	0.00019	0.00198	0.75743
VWA7	1.30403	-1.5452	8.11746	0.00019	0.00198	0.74789
ATF4P3	1.30451	2.36053	3.89374	0.00805	0.02648	-3.4905
ARID1B	1.3086	4.30792	12.0681	1.97E-05	0.0005	3.29625
CENPBD2	1.30877	3.0248	10.4828	4.44E-05	0.00081	2.38359
TTC39B	1.30991	1.19147	4.50958	0.00407	0.01601	-2.7292
MYT1	1.31074	-1.4357	7.32857	0.00033	0.00279	0.10782
RNF144B	1.31264	5.4114	5.48279	0.00154	0.00809	-1.6375
SYT15-AS	1.31471	-0.534	6.57556	0.00059	0.00416	-0.5584
STK24P1	1.31486	0.6424	14.6784	6.31E-06	0.00024	4.56729
TBX19	1.31626	0.19221	6.51482	0.00063	0.00429	-0.6147
MT1L	1.31831	1.73378	4.42103	0.00447	0.01713	-2.8353
GCH1	1.31931	0.70113	8.80575	0.00012	0.0015	1.264
ZC3H13	1.31973	4.43203	16.3455	3.36E-06	0.00017	5.2622
BORA	1.32023	1.8274	9.06382	0.0001	0.00134	1.44834
RAB20	1.3228	1.37833	8.05781	0.0002	0.00204	0.70139
CADM4	1.32295	2.77842	14.3149	7.31E-06	0.00026	4.40478
RELB	1.32359	1.83011	3.77469	0.00925	0.02933	-3.6441
DUSP9	1.33224	1.71232	8.28435	0.00017	0.00185	0.87645
ZDHHC20	1.33746	3.92367	14.2619	7.47E-06	0.00027	4.38073
MPZL3	1.33794	1.45837	6.94351	0.00044	0.00342	-0.2256
ARHGEF3	1.33929	1.60991	8.01598	0.0002	0.00208	0.6686
THBS3-A	1.34219	-1.2211	6.01305	0.00096	0.00578	-1.0959
BEX2	1.34297	2.54458	5.25329	0.00192	0.00939	-1.8831
SH3BGRL	1.34813	1.98874	10.1138	5.45E-05	0.00091	2.15225
MCTP2	1.35002	-0.2771	8.49719	0.00015	0.0017	1.03718
HAPLN3	1.35128	3.01433	8.49261	0.00015	0.0017	1.03376
PDK3	1.3547	0.49578	6.99215	0.00043	0.00334	-0.1827
KRT18P4	1.35665	-0.198	4.3278	0.00495	0.01846	-2.9482
CHST6	1.35747	1.95472	6.82401	0.00049	0.00365	-0.3321
RNF122	1.35914	-0.0485	4.06613	0.00661	0.02303	-3.2718
GALNT12	1.36072	0.85698	6.51191	0.00063	0.00429	-0.6174
GFOD1	1.36184	4.67548	10.675	4.00E-05	0.00075	2.50104
CTXN1	1.36313	4.81935	10.6147	4.13E-05	0.00077	2.46442
FGFBP1	1.368	-1.4542	4.8922	0.00274	0.01208	-2.2842
DAPP1	1.36902	0.0713	7.31305	0.00033	0.00281	0.09467
KRT18P1	1.36937	1.96339	10.5691	4.24E-05	0.00078	2.43657
LARGE2	1.37067	1.34506	8.51346	0.00014	0.00169	1.04932
SH3YL1	1.37111	2.41165	7.48226	0.0003	0.00264	0.23691
KLK14	1.37423	-1.3317	4.53401	0.00396	0.01574	-2.7002
CD74	1.37477	3.96681	9.83656	6.38E-05	0.001	1.97316
PCSK9	1.37504	4.05475	5.53189	0.00147	0.00788	-1.5859
GTF3A	1.37559	4.25616	16.0017	3.80E-06	0.00018	5.12525
RAB11FIF	1.37618	3.04462	10.4674	4.48E-05	0.00081	2.37404
FRG1BP	1.37718	-1.2268	11.2819	2.91E-05	0.00062	2.85914
TSPAN2	1.3776	0.52481	4.80259	0.003	0.01291	-2.3865
MAB21L3	1.37788	-0.8814	6.16801	0.00084	0.00528	-0.9442
PTCH1	1.38025	1.2244	6.03392	0.00094	0.0057	-1.0753
SHISA2	1.38082	-0.7945	3.35416	0.01535	0.04299	-4.2024
HMGA1	1.38105	6.7898	7.91149	0.00022	0.00216	0.58604
ZNF585B	1.38181	-1.3636	6.14896	0.00085	0.00534	-0.9627
BCAM	1.38446	6.56344	10.8496	3.65E-05	0.00071	2.60601
SUCLA2	1.38579	3.22347	16.3834	3.31E-06	0.00017	5.27713
KLC3	1.38738	1.58156	4.96091	0.00255	0.01146	-2.2064
DENND10	1.38818	-0.0247	4.31319	0.00503	0.01868	-2.966
KCNIP1	1.38857	3.81808	5.24986	0.00192	0.00941	-1.8869
KRT18P1	1.39035	0.76155	8.49711	0.00015	0.0017	1.03712

HSPA2	1.39058	2.94888	5.60147	0.00138	0.00756	-1.5132
ACTRT3	1.3919	1.17017	7.84358	0.00023	0.00223	0.53189
CLUHP3	1.39338	1.40533	14.7701	6.08E-06	0.00024	4.60761
TMEM169	1.39822	-0.5456	7.1552	0.00038	0.00307	-0.0405
CCN5	1.3988	-1.2806	4.50364	0.00409	0.01607	-2.7363
RNF207	1.39918	1.84146	4.81388	0.00296	0.01281	-2.3735
MBP	1.40121	2.40329	6.15048	0.00085	0.00534	-0.9612
GPR176	1.4014	1.9619	13.7475	9.25E-06	0.00031	4.14245
EPHB3	1.40188	-1.3392	4.41894	0.00448	0.01716	-2.8378
HOXC9	1.40272	-1.8456	7.42595	0.00031	0.00269	0.18988
SERTM1	1.40472	-1.6104	4.97077	0.00253	0.01138	-2.1954
TMSB15A	1.40577	-0.3081	3.43665	0.01387	0.03996	-4.091
GGT5	1.41075	-1.4751	3.50048	0.01283	0.03767	-4.0054
PCOTH	1.41156	-1.1734	4.40725	0.00454	0.01731	-2.8519
KLF12	1.41232	2.17802	18.7756	1.48E-06	0.00011	6.14931
SCHIP1	1.41492	-1.4426	5.86584	0.00109	0.00635	-1.2428
LINC0163	1.41835	-1.5782	6.41977	0.00068	0.00453	-0.7036
LDB3	1.41866	-1.1003	3.70768	0.01001	0.03108	-3.7314
LRRC1	1.41994	1.66512	7.70116	0.00025	0.00238	0.41701
SRSF12	1.42159	-0.1871	7.58245	0.00027	0.00252	0.31988
CDK8P2	1.42298	-0.4286	5.24734	0.00193	0.00943	-1.8896
KRT18P2	1.42424	0.72191	7.35036	0.00033	0.00276	0.12626
ZDHHC20	1.42672	2.24493	9.75998	6.68E-05	0.00103	1.92287
EFNB2	1.4311	4.32957	4.34765	0.00484	0.01814	-2.924
KRT18P2	1.432	-0.2018	7.35334	0.00032	0.00276	0.12878
SLC6A8	1.43966	2.11048	7.77817	0.00024	0.0023	0.47935
GALNT6	1.44035	2.52415	8.79177	0.00012	0.00151	1.25388
CBR3-AS	1.441	0.00277	9.59211	7.37E-05	0.0011	1.81135
IQCN	1.44207	0.43663	6.71806	0.00053	0.00385	-0.4278
UGGT2	1.44417	2.58118	9.37718	8.38E-05	0.00118	1.66591
S100A1	1.44692	2.305	4.14276	0.00607	0.02154	-3.176
SPTBN2	1.45014	2.78079	7.37757	0.00032	0.00273	0.14923
PLK2	1.45049	6.17293	5.50169	0.00152	0.00801	-1.6176
VWA8	1.45467	2.86631	18.192	1.79E-06	0.00012	5.94829
PTK7	1.45608	5.15536	11.9173	2.12E-05	0.00052	3.21464
HES4	1.45784	3.35447	6.81708	0.00049	0.00366	-0.3383
PPP2R3A	1.46002	3.86778	11.5818	2.50E-05	0.00057	3.02931
AP1M2	1.46018	5.32091	12.3954	1.69E-05	0.00045	3.47003
GAL	1.46072	-0.2238	6.44946	0.00066	0.00446	-0.6757
KRT8P36	1.4609	-0.5435	9.27459	8.91E-05	0.00123	1.59542
TPP2	1.46103	3.39431	18.0827	1.85E-06	0.00012	5.90983
GJB3	1.46176	1.20316	7.28214	0.00034	0.00286	0.06839
CRABP2	1.46249	4.31449	7.36357	0.00032	0.00275	0.13742
WNT7A	1.46401	5.32619	8.63032	0.00013	0.0016	1.13592
REM1	1.46423	-1.8535	6.65411	0.00056	0.00399	-0.4861
PPP1R14	1.46638	0.86252	10.4201	4.60E-05	0.00082	2.34477
TUBA5P	1.46779	0.74921	6.59492	0.00059	0.00411	-0.5405
APOL6	1.46797	2.42971	5.09389	0.00224	0.01044	-2.0579
PARD3B	1.46866	3.28332	3.58524	0.01158	0.03481	-3.8926
CDA	1.46924	0.82443	3.29222	0.01658	0.04552	-4.2865
ERFE	1.47052	2.61893	9.96338	5.94E-05	0.00096	2.05565
SPON1	1.47074	6.30607	7.07577	0.0004	0.0032	-0.1094
PROSER	1.47164	3.57418	16.6139	3.05E-06	0.00016	5.36701
ELL2	1.47176	0.46662	11.9867	2.05E-05	0.00051	3.25234
LRRC8D-	1.47719	0.23606	5.29269	0.00185	0.00915	-1.8405
MEOX1	1.47768	2.56591	3.45588	0.01355	0.03929	-4.0652
USP43	1.47975	0.51824	7.21122	0.00036	0.00297	0.00775
TMEM52	1.47988	0.36191	10.7122	3.92E-05	0.00074	2.52353

RASAL1	1.48091	2.39183	5.95483	0.00101	0.00598	-1.1537
MREG	1.48116	0.8381	5.26314	0.0019	0.00933	-1.8725
HMGA1P	1.48353	0.874	7.42787	0.00031	0.00269	0.19148
VWA1	1.48578	1.8742	6.1401	0.00086	0.00537	-0.9713
SLC4A11	1.49134	3.0834	14.3917	7.08E-06	0.00026	4.43949
ARHGEF	1.49594	2.59364	8.4254	0.00015	0.00175	0.98336
PRSS23	1.5024	3.83535	3.30271	0.01637	0.04514	-4.2723
DTX4	1.50464	0.90816	5.97335	0.00099	0.00591	-1.1353
MAP1LC	1.50566	0.42874	3.21404	0.01829	0.04907	-4.3935
RFXAP	1.50809	0.698	10.014	5.77E-05	0.00095	2.08833
ELL2P1	1.51075	0.36791	7.23251	0.00036	0.00294	0.026
ZNNT1	1.51158	-0.4296	3.47735	0.0132	0.03853	-4.0364
TENT5B	1.51168	2.75314	6.29735	0.00075	0.00487	-0.8197
ITPR3	1.51169	5.34544	12.488	1.62E-05	0.00044	3.51841
MYADM	1.51415	6.14696	13.5052	1.03E-05	0.00033	4.02699
DUSP8	1.51957	0.73606	10.1907	5.22E-05	0.00089	2.20111
PRRT2	1.52212	-1.5976	9.58287	7.41E-05	0.0011	1.80516
TUT7	1.52438	3.30345	11.7374	2.32E-05	0.00055	3.11591
MPZL2	1.52463	4.18515	7.78049	0.00024	0.0023	0.48122
SUCLA2F	1.52635	2.54803	13.0658	1.24E-05	0.00037	3.81221
SLC4A3	1.52715	1.51136	10.6181	4.13E-05	0.00077	2.46648
SVIP	1.5274	1.31302	10.5567	4.27E-05	0.00079	2.42899
MMP15	1.53287	2.25155	8.92843	0.00011	0.00142	1.35223
CCL26	1.53321	-1.1306	3.97473	0.00734	0.02479	-3.3872
FAM107A	1.54233	2.47144	3.89728	0.00802	0.0264	-3.486
EYA1	1.54417	-5.8595	4.54427	0.00392	0.01562	-2.688
KCNK1	1.54556	0.97288	8.36124	0.00016	0.00179	0.93493
CERCAM	1.55113	3.40319	10.8268	3.69E-05	0.00072	2.59241
SMIM10L	1.55214	-0.7269	6.20902	0.00081	0.00514	-0.9045
FERMT1	1.55721	0.95701	5.96258	0.001	0.00595	-1.146
ARHGEF	1.55928	2.36884	17.6362	2.15E-06	0.00013	5.75008
MAFF	1.56653	0.38189	7.00841	0.00042	0.00331	-0.1684
LINC015C	1.56748	-3.2496	4.14075	0.00608	0.02159	-3.1785
CCDC122	1.56836	-0.7295	6.53679	0.00061	0.00425	-0.5943
RAB32	1.57149	3.53058	15.9002	3.95E-06	0.00018	5.08418
FSCN2	1.57194	-1.5946	6.79826	0.0005	0.00369	-0.3553
P3H2	1.57574	4.36646	8.6012	0.00014	0.00162	1.11444
LIMS2	1.57593	0.84569	4.4861	0.00417	0.01632	-2.7572
NAV2	1.57738	3.67288	8.34257	0.00016	0.00181	0.92078
RYR2	1.57838	-0.3932	5.93955	0.00102	0.00604	-1.1689
PDE1C	1.57958	0.98556	3.37286	0.015	0.04231	-4.177
HES6	1.58246	2.19872	10.1892	5.22E-05	0.00089	2.20014
PLEKHO	1.58465	3.30546	12.9184	1.33E-05	0.00038	3.73852
MAPRE2	1.58474	-1.4647	3.54336	0.01218	0.03615	-3.9482
PRTFDC	1.58908	-0.1698	19.9087	1.05E-06	9.09E-05	6.5204
DOCK9	1.59117	2.60613	14.3869	7.10E-06	0.00026	4.43733
RALGPS	1.59225	0.07035	7.56443	0.00028	0.00254	0.30503
LIMK2	1.5964	4.00606	7.69213	0.00025	0.0024	0.40967
SEMA7A	1.59653	1.40743	14.2314	7.56E-06	0.00027	4.36685
FZD3	1.59906	0.94233	9.20329	9.31E-05	0.00127	1.54601
ALPL	1.60317	1.31376	15.9271	3.91E-06	0.00018	5.0951
HMGA1P	1.60722	1.804	13.4303	1.06E-05	0.00034	3.99089
EBI3	1.60926	-0.4896	5.06942	0.00229	0.01061	-2.0851
KCNH2	1.61157	-2.676	4.84376	0.00287	0.01251	-2.3393
ZSWIM4	1.61421	1.44537	4.19764	0.00571	0.02054	-3.1079
FILIP1L	1.61602	0.8226	4.16705	0.00591	0.02109	-3.1458
ARID5B	1.62447	4.52521	14.2107	7.63E-06	0.00027	4.3574
TMPRSS	1.62469	-2.0943	5.32611	0.00179	0.00894	-1.8044

NXNL2	1.62647	-1.1595	7.78845	0.00024	0.00229	0.48763
PATJ	1.62719	4.8406	10.8579	3.63E-05	0.00071	2.61095
PACSIN1	1.62807	2.18999	9.28011	8.88E-05	0.00123	1.59923
HDAC5	1.63075	0.95092	5.3243	0.00179	0.00895	-1.8064
CELSR2	1.63095	3.33855	7.64943	0.00026	0.00244	0.37484
OCLN	1.63195	1.03362	9.65123	7.11E-05	0.00107	1.85083
SLC9A2	1.63323	1.87959	8.89815	0.00011	0.00144	1.33056
KLK1	1.63337	-1.2894	4.01312	0.00702	0.02402	-3.3386
FBLN2	1.63359	2.03198	5.36915	0.00172	0.00871	-1.7583
ATP6V1B	1.63363	-1.5022	4.90549	0.0027	0.01196	-2.2691
AKR1E2	1.63439	-1.9179	4.81568	0.00296	0.0128	-2.3715
CYFIP2	1.63613	1.46756	5.54268	0.00146	0.00782	-1.5745
WFDC2	1.63808	4.13201	5.51409	0.0015	0.00796	-1.6045
IGDCC4	1.63978	0.57595	5.24053	0.00194	0.00947	-1.897
NXPH4	1.63989	1.88444	8.02558	0.0002	0.00207	0.67613
TENM1	1.64254	-0.6969	3.23561	0.0178	0.04805	-4.3639
LBP	1.64617	-2.5786	3.34276	0.01557	0.04347	-4.2178
LRRC34	1.64715	-1.7343	10.0166	5.76E-05	0.00095	2.08996
SLC7A1	1.64808	4.16437	15.344	4.87E-06	0.00021	4.85425
SLC6A15	1.649	-5.0497	7.3874	0.00032	0.00273	0.1575
NFKBIE	1.65126	1.105	5.65296	0.00132	0.0073	-1.4598
RTKN2	1.65179	3.61297	6.43623	0.00067	0.00449	-0.6882
ALS2CL	1.65404	1.82749	6.0444	0.00093	0.00566	-1.065
BCL2L15	1.65707	-1.1893	8.04262	0.0002	0.00205	0.6895
INSR	1.66006	2.80942	7.78686	0.00024	0.00229	0.48635
PCDH1	1.66472	1.50406	6.51855	0.00062	0.00429	-0.6112
SUCLA2F	1.66889	0.36863	9.93396	6.04E-05	0.00097	2.0366
ST8SIA1	1.67019	0.21885	4.60389	0.00368	0.01496	-2.6176
GPR39	1.67934	-0.3748	5.5373	0.00147	0.00784	-1.5802
BMAL2	1.67964	2.05132	9.25293	9.03E-05	0.00124	1.58045
OR2A7	1.68131	0.71284	8.27204	0.00017	0.00186	0.86705
CRISPLD	1.68424	-0.0879	5.03099	0.00238	0.01091	-2.1279
SERPINA	1.69112	0.5961	5.76831	0.00119	0.00676	-1.3415
INA	1.69258	2.50374	4.97952	0.00251	0.0113	-2.1855
TESK2	1.69579	-0.7626	9.06972	0.0001	0.00134	1.4525
MPP7	1.70343	2.06322	5.03805	0.00237	0.01086	-2.12
FOXL2NE	1.70446	-1.3399	8.69888	0.00013	0.00156	1.18625
PHLDA3	1.70973	4.26343	12.7719	1.42E-05	0.0004	3.6644
ZSCAN5D	1.71856	-3.3454	3.6342	0.01092	0.03326	-3.8279
KLF5	1.72023	3.90046	21.1612	7.30E-07	7.40E-05	6.90362
PTGER2	1.72081	-0.4769	8.00873	0.0002	0.00208	0.6629
SP6	1.72309	-3.1906	9.88936	6.19E-05	0.00098	2.00762
CYP26A1	1.73015	-2.0926	4.1906	0.00575	0.02066	-3.1166
RASL11B	1.73235	-1.1677	7.60447	0.00027	0.00249	0.338
MDGA1	1.73568	-0.7664	8.16362	0.00018	0.00195	0.78368
SMIM10L	1.73865	-1.1336	5.67814	0.00129	0.00717	-1.4339
FBLN5	1.74409	2.20243	4.34753	0.00484	0.01814	-2.9242
MOB3B	1.74491	2.14032	4.42498	0.00445	0.0171	-2.8305
PNMA3	1.74786	1.00682	8.96485	0.00011	0.0014	1.37821
OPHN1	1.75243	3.94951	7.83723	0.00023	0.00223	0.5268
MSI1	1.75384	0.86396	6.34797	0.00072	0.00473	-0.7715
CLDN3	1.76199	3.22233	7.68772	0.00025	0.0024	0.40608
FOXI3	1.763	-1.9506	4.46052	0.00429	0.01664	-2.7878
IL17D	1.76475	-2.0366	4.4669	0.00426	0.01657	-2.7802
KRT18P1	1.76703	-0.5849	9.70598	6.89E-05	0.00105	1.88719
TNS1	1.77156	2.17938	9.40576	8.23E-05	0.00117	1.68543
PCK2	1.7732	2.43569	3.66131	0.01057	0.03246	-3.7922
SYTL1	1.77358	2.67088	6.08067	0.0009	0.00555	-1.0294

FHIP1A	1.77418	-0.1707	8.81246	0.00012	0.00149	1.26886
SIM2	1.77507	2.61587	12.7998	1.40E-05	0.0004	3.67857
LOXL1-AS	1.77859	1.78233	6.94886	0.00044	0.00341	-0.2209
SDCBP2	1.7814	-2.336	6.0811	0.0009	0.00555	-1.029
DMKN	1.7885	4.89673	10.2029	5.18E-05	0.00088	2.2088
PRSS16	1.78968	1.84821	11.7352	2.32E-05	0.00055	3.11467
HMGA1P2	1.7965	2.85494	15.2197	5.10E-06	0.00021	4.80166
CDKN2B	1.79667	3.20355	10.2331	5.10E-05	0.00088	2.22787
N4BP3	1.79768	1.99689	13.3892	1.08E-05	0.00034	3.97101
ROBO1	1.80475	-0.7136	4.85833	0.00283	0.0124	-2.3227
VASN	1.80643	3.09305	9.49769	7.79E-05	0.00113	1.74783
TGFB2	1.80704	0.39801	5.49803	0.00152	0.00803	-1.6214
OBSL1	1.80707	3.53999	13.4588	1.05E-05	0.00034	4.00467
PCP4L1	1.81068	-3.4075	9.56083	7.50E-05	0.00111	1.79037
PRR15-D	1.81377	-2.1428	4.76709	0.00311	0.01324	-2.4274
SMAD9	1.81848	0.18023	6.31588	0.00074	0.00482	-0.802
CHCHD7	1.82001	4.04606	22.6323	4.90E-07	5.80E-05	7.32141
HOXA10-	1.82078	0.95098	10.6224	4.12E-05	0.00077	2.46907
EMX2	1.82235	4.24687	9.10055	9.92E-05	0.00132	1.47419
MSR1	1.82908	-2.8584	4.10361	0.00634	0.02228	-3.2248
CRABP1	1.82983	-0.717	4.98215	0.0025	0.01128	-2.1826
IL18R1	1.83265	-1.2092	16.3293	3.38E-06	0.00017	5.25584
EPS8L2	1.83879	4.42603	16.335	3.37E-06	0.00017	5.25807
MBNL1-A	1.84213	0.20996	4.39102	0.00462	0.01752	-2.8715
CSF1	1.84314	1.05123	4.13972	0.00609	0.02161	-3.1798
TMEM86A	1.84529	-0.258	9.99436	5.83E-05	0.00095	2.07565
DES	1.84678	-1.5693	4.00205	0.00711	0.02422	-3.3526
AK7	1.84823	-1.2869	3.72556	0.0098	0.0306	-3.7081
FRY	1.84876	-1.9827	4.98601	0.00249	0.01126	-2.1782
LLGL2	1.85221	2.82165	12.8118	1.40E-05	0.0004	3.68469
CYP24A1	1.85353	-1.6218	5.54179	0.00146	0.00782	-1.5755
HOXC10	1.86014	-0.5577	8.44722	0.00015	0.00174	0.99976
MBOAT1	1.86183	0.86963	13.7457	9.26E-06	0.00031	4.14157
TMEM51-	1.8647	-1.8348	7.77835	0.00024	0.0023	0.4795
TBC1D8B	1.86595	-1.271	10.4703	4.47E-05	0.00081	2.37588
NYNRIN	1.86646	-1.7339	3.89606	0.00803	0.02643	-3.4876
ANKRD1	1.87097	6.08716	4.11176	0.00628	0.02214	-3.2147
QSOX1	1.87181	6.38821	13.3941	1.08E-05	0.00034	3.97336
MYO6	1.87209	3.48754	8.99571	0.00011	0.00138	1.40015
JPH1	1.87767	2.09124	14.9168	5.74E-06	0.00023	4.6716
MYL2	1.88022	-2.6696	4.68975	0.00337	0.014	-2.517
MYO5B	1.88721	0.23578	4.21734	0.00558	0.02021	-3.0836
IL17RE	1.88811	-0.6032	15.628	4.37E-06	0.00019	4.97276
TBC1D30	1.88969	1.08399	13.8592	8.83E-06	0.00029	4.19497
NRAD1	1.89071	-2.9947	4.05789	0.00667	0.02318	-3.2822
C1orf116	1.89765	0.01065	11.516	2.59E-05	0.00058	2.99231
SYT12	1.89814	-2.0908	3.77475	0.00925	0.02933	-3.644
C1orf198	1.90009	3.44224	19.5346	1.17E-06	9.42E-05	6.40059
HOOK2	1.9073	3.34396	22.922	4.55E-07	5.60E-05	7.3999
LPAR2	1.908	2.17717	14.4192	7.00E-06	0.00026	4.45184
ACKR2	1.90915	0.19307	13.6302	9.73E-06	0.00032	4.08682
TTC9	1.91259	-1.3612	8.24624	0.00017	0.00188	0.84729
RIPK4	1.91786	3.33496	5.71875	0.00124	0.00699	-1.3921
TEAD3	1.92	2.75878	24.1971	3.30E-07	4.82E-05	7.73182
DOK5	1.92147	0.41632	7.0484	0.00041	0.00325	-0.1333
LRRC32	1.9218	-2.0405	6.88454	0.00046	0.00352	-0.278
FAM169A	1.92773	-0.795	6.93901	0.00045	0.00342	-0.2296
PHOSPH	1.93268	0.2868	3.91477	0.00786	0.02602	-3.4636

ST6GALN	1.93833	0.86913	3.74065	0.00963	0.03019	-3.6884
NLRP4	1.93873	-2.0504	3.85527	0.00842	0.02741	-3.5399
LOXL3	1.94054	-0.2467	9.80904	6.49E-05	0.00101	1.95513
SLC9A3R	1.94165	2.54947	12.8171	1.39E-05	0.0004	3.68737
HES2	1.94725	-0.4985	10.6375	4.08E-05	0.00076	2.47824
SLITRK5	1.95122	1.42345	11.6538	2.42E-05	0.00056	3.06952
CCDC3	1.95149	2.38788	7.11664	0.00039	0.00313	-0.0739
HMGA1P	1.95214	4.00193	17.3436	2.37E-06	0.00014	5.64296
JPH2	1.953	1.82313	16.0662	3.71E-06	0.00018	5.15116
HCP5	1.95468	-0.724	4.63319	0.00357	0.01463	-2.5831
PODXL	1.95498	4.55345	9.08917	9.99E-05	0.00133	1.46619
RAB11FII	1.95962	3.59067	4.77161	0.00309	0.0132	-2.4222
CHMP4C	1.96635	1.86578	12.7376	1.44E-05	0.0004	3.64696
PRR36	1.9664	0.78902	10.8764	3.59E-05	0.00071	2.62196
DIAPH3	1.96918	4.58635	12.8401	1.38E-05	0.00039	3.69901
IL6	1.97159	1.82705	3.7643	0.00936	0.02959	-3.6576
ICAM5	1.97439	2.63238	5.42779	0.00162	0.0084	-1.6957
APOE	-1.976	3.02986	4.49553	0.00413	0.01619	-2.746
NAALAD2	1.97647	-5.027	7.41702	0.00031	0.0027	0.18239
NMU	1.97969	2.71333	6.37495	0.0007	0.00465	-0.7459
MAP7	1.98166	2.60136	8.18318	0.00018	0.00194	0.79879
DUTP6	1.98252	-0.8193	3.99824	0.00714	0.02431	-3.3574
NME3	1.98438	0.93357	13.5067	1.03E-05	0.00033	4.02771
TRIL	1.98717	-1.5749	10.221	5.13E-05	0.00088	2.22023
ZNF711	1.9936	-0.9504	5.44814	0.00159	0.0083	-1.6741
FBXO2	1.99433	3.6382	9.54585	7.57E-05	0.00111	1.7803
HCAR1	1.99604	-1.6297	12.2492	1.81E-05	0.00047	3.393
SMPD5	1.99962	-0.5211	6.75269	0.00052	0.00379	-0.3964
GPR20	2.00462	-4.17	3.61966	0.01111	0.03375	-3.8471
B4GALNT	2.00502	1.51693	5.82109	0.00113	0.00654	-1.2879
FGD3	2.00551	-0.5411	13.2774	1.13E-05	0.00035	3.91656
BBC3	2.0065	-1.21	4.56762	0.00383	0.01536	-2.6603
NUDT16L	2.01099	-0.6557	4.56504	0.00384	0.01539	-2.6634
SULT1A2	2.01198	-0.4099	10.8	3.74E-05	0.00072	2.57634
CILP2	2.01553	-0.7853	10.1575	5.32E-05	0.0009	2.18005
NUDT15	2.01982	3.89596	17.9669	1.92E-06	0.00012	5.86882
WNT7B	2.02349	1.29655	9.66584	7.05E-05	0.00107	1.86055
FREM1	2.02407	-1.4726	7.58671	0.00027	0.00251	0.32339
SYNGR3	2.02573	-0.1017	8.30721	0.00017	0.00183	0.89388
TMEM117	2.028	1.12065	16.2692	3.45E-06	0.00017	5.23208
MUC20	2.03078	-0.0508	7.83482	0.00023	0.00224	0.52487
ALDH1L2	2.03381	2.98783	3.19767	0.01867	0.04976	-4.4159
PDLIM2	2.0501	0.2214	13.2407	1.15E-05	0.00035	3.89856
DDR1	2.05436	3.53908	14.8736	5.84E-06	0.00023	4.65284
BEX4	2.06007	0.22171	5.68554	0.00128	0.00715	-1.4262
ERVMER	2.06231	-0.4851	8.46054	0.00015	0.00173	1.00975
SMPDL3E	2.06815	1.55844	14.821	5.96E-06	0.00024	4.6299
GRTP1	2.07155	0.01654	4.648	0.00352	0.01448	-2.5658
ID4	2.07239	5.51413	6.90614	0.00046	0.00348	-0.2588
IQCD	2.07312	0.10291	5.66608	0.0013	0.00723	-1.4463
XDH	2.07792	-0.6622	18.1539	1.81E-06	0.00012	5.93488
CGN	2.08111	3.80509	20.5345	8.73E-07	8.36E-05	6.71522
ARTN	2.08255	0.44454	4.2819	0.0052	0.01917	-3.0042
PMEPA1	2.08568	1.54029	6.18068	0.00083	0.00523	-0.9319
NANOS1	2.08644	2.56101	7.0654	0.0004	0.00321	-0.1185
MYO1D	2.0897	3.47719	7.91079	0.00022	0.00216	0.58549
RND3	2.09368	4.35534	7.36446	0.00032	0.00275	0.13817
LNX2	2.09371	1.51621	14.3798	7.12E-06	0.00026	4.43411

SBK2	2.0983	-2.4162	4.21961	0.00557	0.02018	-3.0807
SOX9	2.10122	0.10075	9.36532	8.44E-05	0.00118	1.6578
RCN3	2.1066	2.46805	5.20156	0.00202	0.00972	-1.9395
CHPF	2.11223	3.57098	19.3701	1.23E-06	9.61E-05	6.34705
SUSD3	2.12058	-1.8337	3.82924	0.00868	0.028	-3.5735
MDFIC	2.12764	-2.6232	11.9369	2.10E-05	0.00051	3.2253
JUP	2.12844	4.84654	8.86437	0.00012	0.00146	1.3063
ENTPD2	2.13032	-2.3079	7.79215	0.00024	0.00229	0.49061
IRS1	2.13603	2.01264	9.80399	6.51E-05	0.00101	1.95182
SUSD2	2.13617	2.96463	7.43132	0.00031	0.00269	0.19437
SELENOI	2.14029	0.09387	15.8308	4.05E-06	0.00019	5.05597
ZP3	2.14317	-3.0083	5.64484	0.00133	0.00733	-1.4682
L1CAM	2.14914	5.52542	8.57572	0.00014	0.00164	1.09559
ESPN	2.15283	-1.7058	21.9085	5.95E-07	6.37E-05	7.11996
C21orf91	2.15428	1.68939	13.859	8.83E-06	0.00029	4.19487
KCNK6	2.15461	1.10007	8.44275	0.00015	0.00174	0.99641
ZNF204P	2.16388	0.0556	5.03917	0.00236	0.01086	-2.1188
NEXN	2.16736	3.16094	7.85018	0.00023	0.00222	0.53717
ACTL8	2.17094	-0.7457	6.01194	0.00096	0.00578	-1.097
RINL	2.17307	0.44547	8.51254	0.00014	0.00169	1.04863
WNT6	2.17468	-1.4166	4.60921	0.00366	0.01489	-2.6113
GLS2	2.18702	-1.7645	7.63875	0.00026	0.00245	0.3661
TMEM198	2.1874	-0.4443	9.19132	9.38E-05	0.00127	1.53768
FER1L4	2.19255	0.36875	3.91925	0.00782	0.02592	-3.4579
DTNA	2.199	-2.9071	6.00296	0.00096	0.00581	-1.1059
ERBB4	2.20299	4.20879	5.05706	0.00232	0.01071	-2.0988
MUC1	2.20543	2.26506	11.4807	2.63E-05	0.00058	2.97243
ADGRF1	2.20978	-1.5962	7.4795	0.0003	0.00264	0.23462
ADA	2.21342	2.69728	8.32453	0.00016	0.00182	0.90707
LINC0123	2.22206	-1.3286	7.98072	0.00021	0.0021	0.64084
MYEF2	2.2233	-1.0977	18.2788	1.74E-06	0.00012	5.97862
ERICH5	2.22601	-2.1505	7.97685	0.00021	0.00211	0.63779
SLC44A2	2.23272	5.10956	16.0824	3.69E-06	0.00018	5.15766
SFRP1	2.23294	2.13345	7.0333	0.00041	0.00327	-0.1465
CACNG6	2.23725	-0.9757	6.13083	0.00086	0.0054	-0.9804
GBP5	2.24374	1.11149	8.38876	0.00016	0.00178	0.95574
ARHGEF3	2.2438	0.94164	16.4969	3.18E-06	0.00016	5.32155
CLDN10	2.24861	-0.033	4.94333	0.0026	0.01163	-2.2263
BICDL1	2.25667	0.50679	5.61074	0.00137	0.00752	-1.5036
FABP5	2.25914	-0.8723	13.9611	8.46E-06	0.00029	4.24247
ZNF853	2.26023	0.34675	6.11058	0.00088	0.00546	-1.0001
COL7A1	2.26675	0.48797	9.43936	8.07E-05	0.00116	1.7083
RPS6KA2	2.27594	3.79655	10.7735	3.80E-05	0.00073	2.56046
LBH	2.27604	1.61989	5.12559	0.00217	0.01024	-2.0229
EPSTI1	2.27656	0.81748	5.92963	0.00103	0.00608	-1.1788
SH2D3A	2.27704	2.96531	16.3009	3.41E-06	0.00017	5.2446
NOTUM	2.27811	2.02436	5.79485	0.00116	0.00664	-1.3145
LURAP1	2.279	-2.5751	4.98441	0.00249	0.01127	-2.18
IL1R1	2.28401	4.59531	8.67053	0.00013	0.00158	1.16548
MYEOV	2.28567	-3.3561	5.59921	0.00138	0.00756	-1.5155
KRT8P13	2.28866	-0.4263	8.48356	0.00015	0.00171	1.02699
HPSE	2.29073	0.69027	14.4948	6.79E-06	0.00026	4.48572
SCARA3	2.29089	2.37059	16.762	2.89E-06	0.00016	5.42406
LACC1	2.29133	-1.2969	8.89498	0.00011	0.00144	1.32828
THEMIS2	2.29477	1.36244	6.04353	0.00093	0.00566	-1.0659
CLIC3	2.29527	1.92401	11.0301	3.32E-05	0.00067	2.71286
LRRC41	2.29683	2.76821	25.9191	2.19E-07	4.00E-05	8.14767
KRT80	2.31903	4.68286	6.88306	0.00047	0.00352	-0.2793

DNAJC15	2.32584	-1.5647	14.6859	6.29E-06	0.00024	4.57059
ELF3	2.32996	3.41751	11.0308	3.31E-05	0.00067	2.71329
FGF1	2.33079	-1.4908	3.56635	0.01185	0.03539	-3.9177
RIMS4	2.33673	0.28055	5.639	0.00133	0.00737	-1.4743
TSPOAP1	2.33702	-1.4692	10.6723	4.01E-05	0.00075	2.49937
CAPS	2.33987	1.83734	22.3687	5.26E-07	6.04E-05	7.24893
KRT86	2.34005	-1.9924	7.66477	0.00026	0.00243	0.38738
C1orf115	2.34258	-2.8485	11.6441	2.43E-05	0.00056	3.06409
ADGRG1	2.34363	4.35612	19.6561	1.13E-06	9.28E-05	6.43977
IFITM1	2.34437	1.69491	7.75565	0.00024	0.00233	0.46118
SSC5D	2.36254	-0.6397	12.1183	1.93E-05	0.00049	3.32322
PRSS21	2.36503	2.07228	11.4666	2.65E-05	0.00058	2.96443
LMTK3	2.36994	-0.2657	12.9731	1.30E-05	0.00038	3.76594
EMB	2.37025	-2.4016	4.77888	0.00307	0.01312	-2.4138
DENND2D	2.37104	0.48489	22.7544	4.75E-07	5.80E-05	7.35464
MYCN	2.37112	-1.2299	7.42053	0.00031	0.00269	0.18533
PRKCG	2.3727	-0.3938	5.19557	0.00203	0.00976	-1.946
HMGA1P(	2.38414	0.17616	8.35151	0.00016	0.0018	0.92755
IL17RD	2.39496	1.4957	7.1152	0.00039	0.00313	-0.0751
ASNS	2.39872	4.621	3.2992	0.01644	0.04524	-4.277
NEURL1E	2.40117	1.50347	8.70127	0.00013	0.00156	1.188
SLC35F3	2.40253	-3.8557	5.40516	0.00166	0.00851	-1.7198
UNC5B	2.40364	-0.2005	6.11886	0.00087	0.00544	-0.992
IL32	2.40721	1.27381	9.1042	9.90E-05	0.00132	1.47675
TIMP3	2.40902	4.14408	9.70064	6.91E-05	0.00105	1.88366
ATP8B1	2.42288	1.1121	15.6386	4.35E-06	0.00019	4.97715
FAR2P1	2.42863	-2.7469	4.28618	0.00518	0.0191	-2.999
BMF	2.43656	-1.5646	8.78722	0.00012	0.00151	1.25058
STARD10	2.43869	1.67772	10.36	4.75E-05	0.00084	2.30747
HOXC4	2.43951	-2.7566	7.80063	0.00023	0.00228	0.49743
RBP7	2.44002	-0.9146	6.4182	0.00068	0.00453	-0.7051
CKMT1B	2.4479	-2.3388	7.3136	0.00033	0.00281	0.09514
HOXC8	2.45331	-2.3014	5.70695	0.00125	0.00705	-1.4042
C2orf15	2.45603	-1.469	9.27719	8.90E-05	0.00123	1.59722
SMOC2	2.46427	1.00151	6.08508	0.0009	0.00554	-1.0251
SCNN1A	2.47284	0.89576	5.24393	0.00193	0.00945	-1.8933
FABP5P7	2.48073	3.4928	8.54161	0.00014	0.00166	1.07028
TACSTD2	2.48252	5.49001	13.4474	1.05E-05	0.00034	3.99913
PTK2B	2.48581	0.22342	10.6965	3.96E-05	0.00075	2.51402
SLC27A3	2.49159	2.64785	9.79194	6.55E-05	0.00102	1.9439
SPDEF	2.49912	-2.6973	5.47003	0.00156	0.00817	-1.651
ARHGAP	2.50601	0.30019	24.0937	3.38E-07	4.82E-05	7.70568
S100A9	2.5062	0.5269	5.47234	0.00156	0.00815	-1.6485
NPR3	2.50961	1.00855	3.99718	0.00715	0.02433	-3.3588
PLEKHS1	2.51856	-1.6751	4.85432	0.00284	0.01242	-2.3273
TUNAR	2.52535	-1.82	4.50568	0.00409	0.01606	-2.7339
PLEKHN1	2.52675	-0.4	9.96051	5.95E-05	0.00096	2.0538
RBP1	2.53013	2.88941	13.0979	1.23E-05	0.00037	3.82815
CATSPEF	2.53629	-1.9209	9.20183	9.32E-05	0.00127	1.54499
PRRG4	2.53972	0.85227	13.0551	1.25E-05	0.00037	3.80686
ITGB4	2.54315	1.37595	12.3557	1.72E-05	0.00046	3.44919
MYO3B	2.54456	1.22395	15.2055	5.13E-06	0.00021	4.79563
ZNF165	2.54488	0.31565	10.598	4.17E-05	0.00077	2.45422
LINC0070	2.54994	-3.0693	3.78713	0.00911	0.02904	-3.628
CILP	2.55036	2.75271	3.94269	0.00761	0.02548	-3.428
SLC22A3	2.55433	0.03882	12.1701	1.88E-05	0.00048	3.35094
DNAAF3	2.55466	-0.2789	8.2819	0.00017	0.00185	0.87458
KRT8P33	2.56396	2.6967	20.2866	9.38E-07	8.61E-05	6.63888

MFSD6	2.5654	0.43026	9.41711	8.18E-05	0.00116	1.69317
C17orf107	2.56914	-0.1047	7.40351	0.00031	0.00271	0.17105
ARMCX2	2.5708	1.81544	14.8761	5.83E-06	0.00023	4.65391
RAP1GAP	2.57213	1.88222	5.88651	0.00107	0.00626	-1.222
KRT8P3	2.57915	5.71132	16.7245	2.93E-06	0.00016	5.40967
SPSB4	2.58321	-2.0502	6.21857	0.0008	0.00511	-0.8953
KRT8	2.58379	5.79433	16.4906	3.19E-06	0.00016	5.3191
THRB	2.59387	2.23837	13.0965	1.23E-05	0.00037	3.82743
FUT1	2.60781	-0.1999	14.1731	7.74E-06	0.00027	4.34023
NTF4	2.60917	-2.1085	10.0933	5.51E-05	0.00092	2.13914
CCNA1	2.63408	1.86495	4.93899	0.00261	0.01166	-2.2312
INAVA	2.63447	1.95844	8.99899	0.00011	0.00138	1.40248
SIGLEC1	2.63626	-2.9805	5.72445	0.00124	0.00696	-1.3863
ARFGEF3	2.64052	-0.3465	16.1771	3.57E-06	0.00017	5.19552
GLB1L2	2.64584	-1.6569	6.08754	0.0009	0.00553	-1.0226
ACTA2	2.66646	0.4018	8.24429	0.00017	0.00188	0.8458
FOXL2	2.6698	-0.5564	15.9147	3.93E-06	0.00018	5.09007
DNAJC6	2.68806	1.14718	6.46617	0.00065	0.00442	-0.6601
HOXB9	2.69842	4.22348	10.6628	4.03E-05	0.00076	2.49364
MYO5C	2.70641	1.10939	13.1709	1.19E-05	0.00036	3.86424
GRHL1	2.70717	-1.1535	12.2721	1.79E-05	0.00047	3.4051
ELAPOR2	2.71276	-3.6291	5.79584	0.00116	0.00664	-1.3135
PDE3A	2.71398	-1.9756	6.4071	0.00068	0.00456	-0.7156
FABP5P1	2.71596	-0.7306	9.5302	7.64E-05	0.00112	1.76977
KCNQ3	2.72006	-0.6983	21.9039	5.95E-07	6.37E-05	7.11866
SAMD12	2.72364	-0.008	14.0074	8.30E-06	0.00029	4.26398
IL2RG	2.7294	-3.8081	3.5136	0.01263	0.03725	-3.9879
GCNT4	2.73388	0.13533	5.12314	0.00218	0.01025	-2.0256
DHDH	2.73621	-2.3571	6.29157	0.00075	0.00488	-0.8252
KRT23	2.75015	1.34456	4.2433	0.00543	0.01976	-3.0516
LMO7	2.75745	2.3519	7.66618	0.00026	0.00243	0.38853
HRK	2.75748	-3.3806	6.95878	0.00044	0.00339	-0.2121
RPSAP52	2.76967	-0.684	9.6727	7.02E-05	0.00107	1.86511
DSP	2.77476	6.29478	16.278	3.44E-06	0.00017	5.23557
S100A4	2.77903	3.94127	27.2893	1.61E-07	3.37E-05	8.45469
SEMA4A	2.78093	0.81497	7.30122	0.00034	0.00283	0.08462
PCDHGC	2.78267	-1.8899	11.1841	3.06E-05	0.00064	2.80269
MLXIPL	2.78546	-1.3744	5.69517	0.00127	0.00711	-1.4163
LRRN2	2.79458	-1.9595	5.46225	0.00157	0.00821	-1.6592
RND2	2.79477	-3.8791	4.21645	0.00559	0.02022	-3.0846
SLC8A1	2.8008	2.85264	13.056	1.25E-05	0.00037	3.80731
POU2F3	2.81016	-3.5761	6.72151	0.00053	0.00385	-0.4247
COL4A4	2.81877	-0.6251	9.87085	6.26E-05	0.00099	1.99556
CREG2	2.81906	-4.2455	5.23913	0.00194	0.00948	-1.8985
KRT8P32	2.81943	-0.086	16.7523	2.90E-06	0.00016	5.42032
KRT8P45	2.83216	2.92408	17.6045	2.17E-06	0.00013	5.73855
NAPSB	2.84863	-2.396	5.5144	0.0015	0.00796	-1.6042
CACNA2D	2.85435	-0.7366	6.31493	0.00074	0.00482	-0.8029
SYNDIG1	2.85521	-3.1401	4.85557	0.00284	0.01242	-2.3259
SYT5	2.8554	-2.2175	6.61823	0.00057	0.00406	-0.519
TMEM45B	2.85917	-2.5504	12.3278	1.74E-05	0.00046	3.43454
XKR8	2.8783	-0.0981	11.5378	2.56E-05	0.00058	3.00458
MARVELD	2.88323	0.82105	10.737	3.87E-05	0.00074	2.53852
PRRG2	2.8877	-0.1838	23.2255	4.20E-07	5.30E-05	7.4809
ABCG4	2.89027	-1.9796	6.02499	0.00095	0.00573	-1.0841
SLC7A8	2.89028	-1.2813	5.96686	0.001	0.00593	-1.1417
F3	2.89042	2.18137	12.4914	1.62E-05	0.00044	3.52018
NPTX2	2.89568	4.94594	18.9656	1.40E-06	0.0001	6.21328

COL8A1	2.89802	1.29373	9.56072	7.50E-05	0.00111	1.7903
IL11	2.90161	-1.0521	5.92301	0.00103	0.00611	-1.1854
EMILIN2	2.90724	1.27109	4.04428	0.00678	0.02339	-3.2993
RGCC	2.92343	-0.9849	7.71623	0.00025	0.00237	0.42925
C10orf82	2.92346	-4.1823	7.19212	0.00037	0.00301	-0.0087
RASIP1	2.9297	-0.3217	7.4765	0.0003	0.00264	0.23212
TMEM238	2.93207	1.9469	14.5616	6.61E-06	0.00025	4.51553
IGSF9	2.93284	-0.9896	6.71368	0.00053	0.00386	-0.4318
CRYBG1	2.93756	-0.407	22.0411	5.74E-07	6.37E-05	7.15744
EVPL	2.93972	-0.4139	11.8331	2.21E-05	0.00053	3.1686
KRT8P10	2.94502	1.78985	13.36	1.09E-05	0.00034	3.95683
MYB	2.96247	-1.7758	16.813	2.84E-06	0.00016	5.44358
PNCK	2.97271	-1.8179	15.1746	5.19E-06	0.00021	4.78247
COL26A1	2.97971	3.4194	16.6728	2.99E-06	0.00016	5.38975
NOXO1	2.99146	-2.0267	10.8224	3.70E-05	0.00072	2.58974
LAMA3	2.99624	6.17623	10.3607	4.75E-05	0.00084	2.30787
CRYBG2	2.99658	1.20334	23.8002	3.64E-07	4.95E-05	7.6308
PLAG1	3.00189	0.97814	7.40193	0.00031	0.00271	0.16972
IRS2	3.01328	1.73502	11.911	2.13E-05	0.00052	3.21119
ELMO3	3.02393	1.46742	15.7866	4.12E-06	0.00019	5.03793
RNF152	3.03647	-1.8694	11.7945	2.25E-05	0.00054	3.14737
LINC0304	3.04293	2.71907	8.60628	0.00014	0.00162	1.11819
ATP10A	3.04988	-1.6649	7.44484	0.0003	0.00268	0.20569
LINC0111	3.0522	-3.9544	4.79086	0.00303	0.01301	-2.4
MDFI	3.06518	-1.6509	10.127	5.41E-05	0.00091	2.16063
COL8A2	3.0824	-0.4903	15.6545	4.33E-06	0.00019	4.98371
IGFBP2	3.08603	4.5238	11.3335	2.84E-05	0.00061	2.8887
TMPRSS1	3.09016	-2.4419	8.05779	0.0002	0.00204	0.70136
ZDHHC2	3.09959	-0.0101	8.04648	0.0002	0.00205	0.69252
RGL3	3.09967	2.57321	11.7592	2.29E-05	0.00054	3.12791
KIF1A	3.11405	1.0843	10.9497	3.46E-05	0.00069	2.6655
ELOVL3	3.12707	-0.8684	10.2858	4.95E-05	0.00086	2.26101
IL1RL2	3.14226	-0.6651	30.4576	8.37E-08	2.41E-05	9.09438
CLDN9	3.16056	-0.9538	14.7602	6.11E-06	0.00024	4.60328
UPK1B	3.16123	-0.5402	3.92519	0.00776	0.02582	-3.4503
TNF	3.1623	0.01354	7.68019	0.00026	0.00241	0.39995
TACR1	3.16625	0.64507	5.74197	0.00122	0.00687	-1.3684
EXPH5	3.16961	-0.2256	9.86059	6.30E-05	0.00099	1.98887
HMX2	3.17038	-1.6222	14.2058	7.64E-06	0.00027	4.35519
AMN	3.18324	-0.3198	15.894	3.96E-06	0.00018	5.08166
HPD	3.18404	-1.1507	14.3766	7.13E-06	0.00026	4.4327
ASIC2	3.1876	0.31079	5.00797	0.00244	0.01107	-2.1536
GPR15LG	3.19414	-2.1214	24.9295	2.76E-07	4.41E-05	7.91301
BAGE2	3.19848	-5.9528	5.54052	0.00146	0.00782	-1.5768
CAMSAP3	3.19854	1.14352	15.005	5.55E-06	0.00022	4.70976
MX2	3.19891	-3.0271	11.686	2.38E-05	0.00055	3.08738
TSPAN1	3.20935	1.74428	9.8378	6.38E-05	0.001	1.97397
NOS3	3.21111	-1.578	10.031	5.71E-05	0.00094	2.09924
FOXA1	3.21737	0.34548	7.03154	0.00041	0.00328	-0.1481
PITPNM3	3.22074	0.27273	20.5794	8.62E-07	8.30E-05	6.72893
STC2	3.23375	3.88435	6.39274	0.00069	0.00459	-0.7291
FOLR1	3.23651	0.56046	11.0435	3.29E-05	0.00067	2.72071
AMZ1	3.23872	-1.8731	14.4914	6.80E-06	0.00026	4.48422
GPR143	3.24617	-0.5478	14.0323	8.21E-06	0.00028	4.2755
FAAH2	3.24919	-2.5841	11.0969	3.20E-05	0.00066	2.75195
MIR4664	3.2517	-3.6806	3.96823	0.00739	0.02493	-3.3955
TENM2	3.27781	-1.0659	8.63141	0.00013	0.0016	1.13673
DSEL	3.27881	-2.6046	11.973	2.07E-05	0.00051	3.2449

EFHD1	3.29312	2.13168	16.6517	3.01E-06	0.00016	5.38161
KRT8P35	3.29618	-0.3414	12.5203	1.59E-05	0.00043	3.53518
ARHGEF	3.30033	-0.0421	19.4659	1.20E-06	9.43E-05	6.3783
LINC0259	3.30547	-1.6379	18.9557	1.40E-06	0.0001	6.20994
CFTR	3.31232	-2.216	19.7779	1.09E-06	9.19E-05	6.47881
GNGT2	3.31323	-3.0249	5.38261	0.00169	0.00864	-1.7439
MARCHF	3.34734	-0.8071	3.7485	0.00954	0.02999	-3.6782
RD3	3.34919	-1.6758	11.886	2.16E-05	0.00052	3.19757
SCGB2A1	3.34991	-0.5415	9.78846	6.57E-05	0.00102	1.94162
LRG1	3.35305	1.82715	5.76153	0.00119	0.00678	-1.3484
ANKRD2	3.37164	-1.0674	10.7201	3.91E-05	0.00074	2.52833
ITGB6	3.38067	1.25768	17.5442	2.21E-06	0.00013	5.71659
HS3ST3A	3.38914	-3.1034	10.1769	5.26E-05	0.00089	2.19237
NPFFR1	3.39018	-2.1823	8.64006	0.00013	0.00159	1.1431
CPA4	3.41815	1.63309	11.4357	2.69E-05	0.00059	2.94692
SOWAHA	3.42022	1.20635	9.95322	5.97E-05	0.00096	2.04908
SGIP1	3.42825	2.00933	19.9548	1.03E-06	9.09E-05	6.53497
MAGI2-AS	3.43477	-1.9937	23.9801	3.48E-07	4.82E-05	7.67685
PNPLA5	3.44154	-3.4512	14.6874	6.29E-06	0.00024	4.57126
ADAP1	3.44865	-1.3008	15.3014	4.95E-06	0.00021	4.83627
TMEM47	3.45752	1.17875	22.3954	5.22E-07	6.04E-05	7.25633
TTC22	3.46651	-1.3495	10.8507	3.64E-05	0.00071	2.60666
PID1	3.47489	-1.6411	8.29965	0.00017	0.00184	0.88813
MST1R	3.47533	-0.0705	10.8024	3.74E-05	0.00072	2.57778
PRR15	3.48142	1.60444	7.51062	0.00029	0.0026	0.26049
NPR1	3.48958	2.02768	11.7549	2.30E-05	0.00054	3.12556
PDE5A	3.49458	-2.4164	7.53252	0.00028	0.00257	0.27866
SAMD11	3.50503	-0.5598	14.4174	7.01E-06	0.00026	4.45106
GPRIN2	3.50784	-0.973	9.3595	8.47E-05	0.00118	1.65381
DEPP1	3.51854	-0.7231	7.76744	0.00024	0.00231	0.47069
SMIM5	3.53602	-3.4679	7.20731	0.00036	0.00298	0.00439
SEMA3B	3.53684	1.59564	18.0796	1.85E-06	0.00012	5.90874
C8orf34	3.53772	0.02138	11.9786	2.06E-05	0.00051	3.24794
B4GALNT	3.54971	-1.3583	10.7592	3.83E-05	0.00074	2.55185
TMTC2	3.55121	-0.052	24.836	2.82E-07	4.47E-05	7.89024
GPR87	3.5541	-4.7213	4.36647	0.00474	0.0179	-2.9012
FAM83H	3.56796	4.53717	23.2237	4.21E-07	5.30E-05	7.48042
FOXS1	3.57478	-1.1003	11.3619	2.80E-05	0.0006	2.90494
TJP3	3.58494	0.04692	8.38286	0.00016	0.00178	0.95129
MAP7-AS	3.6159	-0.8552	17.2929	2.41E-06	0.00014	5.62418
HR	3.62224	1.02485	4.76645	0.00311	0.01324	-2.4281
FSTL1	3.63762	5.1551	9.53683	7.61E-05	0.00111	1.77423
ZNF439	3.66059	-2.2577	15.0179	5.52E-06	0.00022	4.71533
LYPD6B	3.70523	-0.1042	8.42482	0.00015	0.00175	0.98292
B3GALT4	3.7172	-2.7029	8.83213	0.00012	0.00148	1.28307
BLNK	3.72035	-3.799	5.1129	0.0022	0.0103	-2.0369
SLC12A8	3.74418	0.96803	33.5289	4.72E-08	2.01E-05	9.63484
IL1R2	3.74529	2.38565	11.2065	3.03E-05	0.00064	2.81564
MROH2A	3.75774	-3.1347	7.0924	0.0004	0.00316	-0.0949
KLK10	3.76066	2.5152	12.3802	1.70E-05	0.00045	3.46206
IGFBP3	3.78272	4.5006	16.5644	3.10E-06	0.00016	5.34782
RASSF9	3.79665	-2.3724	6.74363	0.00052	0.00381	-0.4046
PGAP4	3.82584	-3.9407	4.45695	0.0043	0.01667	-2.7921
CARD14	3.829	-1.8474	10.0301	5.71E-05	0.00094	2.09867
KLK7	3.82942	-2.3733	5.12849	0.00216	0.01022	-2.0197
ANXA9	3.83481	0.09875	14.4512	6.91E-06	0.00026	4.46623
EMX2OS	3.84234	3.46773	19.596	1.15E-06	9.35E-05	6.42043
MGAT3	3.8525	0.21736	15.3197	4.91E-06	0.00021	4.84401

TMC4	3.86128	1.62136	13.1595	1.19E-05	0.00036	3.85863
EHF	3.88981	-1.9044	8.15637	0.00018	0.00196	0.77807
GJB6	3.89738	-1.0042	6.64443	0.00056	0.00401	-0.495
ANO9	3.91422	0.56382	17.0725	2.60E-06	0.00015	5.54194
NR3C2	3.93259	-1.2828	8.74868	0.00012	0.00153	1.22259
EGFL6	3.95016	-3.067	11.6183	2.46E-05	0.00056	3.0497
ADAMTS2	3.95139	-2.711	5.11556	0.00219	0.01029	-2.034
PAK6	3.95251	-2.0749	9.28103	8.88E-05	0.00123	1.59987
STK26	3.96397	0.95517	16.1935	3.55E-06	0.00017	5.20201
KRT81	3.98782	-1.078	11.4441	2.68E-05	0.00059	2.95168
FNDC1	4.001	2.83077	8.46359	0.00015	0.00173	1.01204
PDZRN3	4.01381	-2.8692	10.2943	4.93E-05	0.00086	2.26638
GPRC5A	4.01867	3.01088	16.0003	3.80E-06	0.00018	5.12468
SUN3	4.03692	-2.3206	7.73429	0.00025	0.00235	0.4439
FBN3	4.03783	-0.891	9.54213	7.59E-05	0.00111	1.7778
MAPK15	4.08771	-2.2311	8.04101	0.0002	0.00205	0.68823
SYBU	4.09356	-1.7891	22.7252	4.78E-07	5.80E-05	7.34672
LSR	4.09602	2.97842	13.3216	1.11E-05	0.00035	3.93813
CDC42BF	4.10792	0.72773	33.3455	4.88E-08	2.01E-05	9.6045
EHD2	4.11079	-3.807	8.20907	0.00018	0.00191	0.81874
SIGIRR	4.13325	0.0259	11.4619	2.66E-05	0.00058	2.96176
INHBB	4.15702	0.15235	8.4965	0.00015	0.0017	1.03666
PROM1	4.1581	2.86652	29.6386	9.85E-08	2.63E-05	8.93766
MSLN	4.17593	-0.1995	16.6721	2.99E-06	0.00016	5.38947
EDNRB	4.1824	-3.3933	9.19154	9.38E-05	0.00127	1.53783
HID1	4.2058	-0.1036	23.2653	4.16E-07	5.30E-05	7.49142
ERP27	4.21243	-0.1515	11.0711	3.25E-05	0.00067	2.73688
OVOL2	4.22658	-0.9776	12.8294	1.38E-05	0.00039	3.69362
COL1A2	4.23635	-0.7268	14.7184	6.21E-06	0.00024	4.58494
SYK	4.24145	-2.1812	9.18327	9.43E-05	0.00127	1.53207
CNGB1	4.25255	0.30214	33.1796	5.03E-08	2.01E-05	9.57687
PALM3	4.26173	0.64908	10.4774	4.45E-05	0.00081	2.38025
MARVELI	4.27128	-0.5026	28.6091	1.22E-07	2.92E-05	8.73238
KLRG2	4.28935	1.84561	13.026	1.27E-05	0.00037	3.79237
BARX1	4.3062	-0.0718	7.12205	0.00039	0.00313	-0.0692
MTARC2	4.30745	-1.323	11.574	2.51E-05	0.00057	3.02494
SOWAHE	4.31453	-3.0336	19.0109	1.38E-06	0.0001	6.22841
TRIM17	4.32196	-1.9975	14.036	8.20E-06	0.00028	4.27722
FLNC	4.3328	1.56321	25.8165	2.24E-07	4.02E-05	8.12386
GJB1	4.33357	-2.5251	7.57955	0.00027	0.00252	0.31749
KCNK15	4.35023	0.15073	8.78344	0.00012	0.00151	1.24784
EPHB1	4.37901	-1.7208	6.63073	0.00057	0.00404	-0.5076
NLGN4X	4.40393	0.02709	19.7473	1.10E-06	9.21E-05	6.46903
LAMB3	4.42789	0.86052	14.0384	8.19E-06	0.00028	4.27829
ESRP2	4.45554	1.21957	12.2257	1.83E-05	0.00047	3.38051
CYP26C1	4.49454	-2.867	10.1413	5.37E-05	0.0009	2.16976
CARMN	4.49888	-3.0568	17.2373	2.45E-06	0.00014	5.60352
RNF180	4.5345	-3.0971	44.3191	8.91E-09	9.87E-06	11.08
LAMC2	4.53601	2.25165	23.3378	4.09E-07	5.30E-05	7.51052
DLX3	4.553	-0.1841	19.4864	1.19E-06	9.43E-05	6.38495
PRDM5	4.55837	-3.0938	11.3252	2.85E-05	0.00061	2.88397
NUP210L	4.5688	-3.503	12.7583	1.43E-05	0.0004	3.65749
TMEM178	4.57555	-1.6955	7.4051	0.00031	0.00271	0.17238
CRACDL	4.58966	-0.7244	25.2123	2.58E-07	4.27E-05	7.98123
CHSY3	4.60975	-1.8711	14.822	5.96E-06	0.00024	4.63036
CXCL16	4.61767	1.47757	15.7707	4.14E-06	0.00019	5.03143
NPPB	4.61911	-1.8115	12.0803	1.96E-05	0.0005	3.30282
ZBED2	4.62579	-0.2442	4.13538	0.00612	0.0217	-3.1852

GPX7	4.64106	-1.6681	20.9448	7.76E-07	7.77E-05	6.8393
SLC6A12	4.64434	-1.2294	12.6835	1.48E-05	0.00041	3.61928
CALHM2	4.64876	-2.4942	9.80479	6.50E-05	0.00101	1.95234
KLHL30	4.65299	-1.9009	15.0393	5.47E-06	0.00022	4.72454
MSLNL	4.66993	-2.1236	16.515	3.16E-06	0.00016	5.32861
NCF2	4.68106	-2.1228	15.3301	4.89E-06	0.00021	4.84842
LMOD1	4.69452	-0.6622	15.478	4.62E-06	0.0002	4.91047
FUT3	4.69544	-2.2764	7.17911	0.00037	0.00303	-0.0199
LCK	4.69846	-2.1309	11.4524	2.67E-05	0.00059	2.95642
XG	4.70365	-3.2774	25.7171	2.29E-07	4.04E-05	8.10068
SLC35G6	4.71068	-2.2155	13.1148	1.22E-05	0.00037	3.83649
RASD2	4.72484	-3.7281	9.48247	7.86E-05	0.00114	1.73754
COL4A3	4.72789	-3.8568	10.709	3.93E-05	0.00074	2.52158
CD164L2	4.73151	-3.4932	10.4043	4.64E-05	0.00083	2.33502
UNC13D	4.75143	0.57679	33.0471	5.15E-08	2.01E-05	9.55466
GLDC	4.77373	0.66805	25.2048	2.59E-07	4.27E-05	7.97943
CNN1	4.79763	-0.5545	18.8793	1.43E-06	0.0001	6.18432
EPCAM	4.80893	3.57741	23.5421	3.88E-07	5.14E-05	7.56403
LINC0162	4.82292	-2.0746	14.3672	7.15E-06	0.00026	4.42844
ZNF469	4.83051	-2.7197	13.3892	1.08E-05	0.00034	3.971
PLEKHG6	4.83751	-1.4373	25.0633	2.67E-07	4.32E-05	7.9454
IGFL1	4.8395	0.07514	6.98205	0.00043	0.00335	-0.1916
EMC10	4.87941	3.33254	9.82655	6.42E-05	0.00101	1.96661
INHBE	4.88577	-1.9268	4.08018	0.00651	0.02275	-3.2542
SFTA1P	4.90752	-2.6515	9.52052	7.69E-05	0.00112	1.76324
SEC14L4	4.92669	-3.9882	47.9263	5.58E-09	7.87E-06	11.4464
PTGS1	4.95088	3.30525	21.2361	7.15E-07	7.30E-05	6.92569
GLDCP1	4.95743	2.55853	24.4956	3.06E-07	4.65E-05	7.80647
KLK5	5.03427	-3.3337	9.42481	8.14E-05	0.00116	1.6984
RNF39	5.04711	-2.4908	11.6316	2.44E-05	0.00056	3.05713
IQANK1	5.08443	-0.8207	19.1513	1.32E-06	0.0001	6.27508
FUT2	5.10781	-3.0757	10.2233	5.12E-05	0.00088	2.22172
PTGER4	5.11827	-3.3134	11.4813	2.63E-05	0.00058	2.97275
ERBB3	5.12166	1.36957	9.54166	7.59E-05	0.00111	1.77748
LTB	5.13865	-1.6052	20.4085	9.05E-07	8.46E-05	6.67655
KRT5	5.19518	-3.3864	32.8823	5.30E-08	2.01E-05	9.52685
DOK7	5.20886	-2.0655	17.2901	2.41E-06	0.00014	5.62315
PTAFR	5.21427	-1.3134	17.6116	2.16E-06	0.00013	5.74113
CRB3	5.21924	1.15771	32.0672	6.16E-08	2.17E-05	9.38647
SYT7	5.22196	-0.9157	15.4502	4.67E-06	0.00021	4.89882
COBL	5.22509	-2.418	10.8206	3.70E-05	0.00072	2.58869
SBK1	5.23011	-1.893	18.2081	1.78E-06	0.00012	5.95389
CRYAB	5.23659	3.05786	12.3403	1.73E-05	0.00046	3.44113
CNKSR1	5.23846	0.03306	37.668	2.36E-08	1.46E-05	10.2621
PPL	5.26253	0.41275	38.6249	2.03E-08	1.35E-05	10.393
NYAP2	5.28552	-1.8605	16.0102	3.79E-06	0.00018	5.12867
ALDH3B2	5.29203	-3.9573	15.2908	4.97E-06	0.00021	4.83182
GARIN5A	5.29232	-1.007	14.0556	8.13E-06	0.00028	4.28624
CAND2	5.3045	-2.1413	32.2737	5.93E-08	2.14E-05	9.42249
LAD1	5.34264	2.49869	40.5576	1.51E-08	1.24E-05	10.6429
KRT13	5.34759	-2.5403	11.8326	2.21E-05	0.00053	3.1683
CLDN7	5.35768	2.6032	19.6633	1.13E-06	9.28E-05	6.4421
KRTAP2-	5.35874	-0.5257	6.72822	0.00053	0.00384	-0.4186
SORCS2	5.36405	0.54814	12.4044	1.68E-05	0.00045	3.47476
KRTCAP3	5.36666	0.98209	17.7801	2.04E-06	0.00013	5.80204
MAP1LC3	5.36819	-1.2182	18.2582	1.75E-06	0.00012	5.97143
NECTIN4	5.37034	-2.7899	9.64327	7.15E-05	0.00107	1.84553
SPINT1	5.3809	1.79245	17.7743	2.05E-06	0.00013	5.79993

MTARC1	5.41861	-2.516	12.6881	1.48E-05	0.00041	3.62164
C8orf34-A	5.42297	-2.4131	7.89898	0.00022	0.00218	0.57609
FGF9	5.4241	-1.9962	22.1058	5.64E-07	6.37E-05	7.17564
C6orf132	5.43344	1.37988	20.0732	9.98E-07	9.00E-05	6.5723
CDH3	5.4354	1.59149	17.896	1.97E-06	0.00012	5.84354
LCP1	5.44755	4.13276	44.5154	8.68E-09	9.87E-06	11.1012
CALB1	5.44831	-1.6172	10.2354	5.09E-05	0.00088	2.22933
NUP62CL	5.48977	-3.5801	9.65185	7.11E-05	0.00107	1.85124
HSH2D	5.53977	-3.5845	11.8601	2.18E-05	0.00053	3.18341
KRT6A	5.54639	-3.2108	24.2286	3.27E-07	4.82E-05	7.73974
NLRP7	5.54817	-3.982	10.7314	3.88E-05	0.00074	2.53512
PLA2G3	5.55456	-1.542	12.2372	1.82E-05	0.00047	3.38662
CDCP1	5.56117	1.3483	52.2513	3.33E-09	5.74E-06	11.8288
PTN	5.57521	-4.1185	8.38702	0.00016	0.00178	0.95443
TMEM184	5.58624	0.00126	30.909	7.67E-08	2.36E-05	9.17836
SYNE4	5.61155	-0.0314	22.0685	5.69E-07	6.37E-05	7.16518
GRAMD2	5.61772	-3.2707	9.71006	6.87E-05	0.00105	1.88989
MAB21L4	5.6411	-2.9643	11.0204	3.33E-05	0.00068	2.70716
ADAMTS1	5.64615	-2.928	11.5821	2.50E-05	0.00057	3.02947
ADGRG5	5.65496	-3.5514	9.75965	6.68E-05	0.00103	1.92265
CST6	5.67223	-0.8817	9.94251	6.01E-05	0.00097	2.04214
TNNC1	5.68753	4.3575	13.9044	8.66E-06	0.00029	4.21609
FXYD3	5.69066	-3.2488	28.8039	1.17E-07	2.92E-05	8.77195
SLC6A9	5.69201	-0.8344	7.42923	0.00031	0.00269	0.19262
SPINT1-A	5.71133	-1.5114	12.9706	1.30E-05	0.00038	3.76471
VGLL1	5.72368	-3.5642	20.2253	9.55E-07	8.71E-05	6.61981
EPN3	5.79135	-2.0922	14.7996	6.01E-06	0.00024	4.62057
PROM2	5.81793	-1.2011	15.4224	4.72E-06	0.00021	4.88722
VEGFC	5.82956	-3.2013	14.7151	6.22E-06	0.00024	4.58347
B3GNT3	5.85531	-0.6163	11.8903	2.15E-05	0.00052	3.19988
TC2N	5.89023	0.7196	25.4052	2.47E-07	4.17E-05	8.02721
CLDN22	5.91164	-2.3792	25.7921	2.25E-07	4.02E-05	8.11818
ADAMTS5	5.93051	-2.6906	19.9611	1.03E-06	9.09E-05	6.53699
CLIC5	5.94508	-1.8976	8.68044	0.00013	0.00157	1.17275
CNTNAP2	5.9455	-0.6721	18.5766	1.58E-06	0.00011	6.08156
KLK6	5.97943	-1.5242	12.7372	1.44E-05	0.0004	3.64674
RARRES2	6.04214	-1.3702	21.9972	5.80E-07	6.37E-05	7.14505
PLD5	6.11261	-2.8346	18.9313	1.41E-06	0.0001	6.20178
LXN	6.1162	-3.1122	16.3169	3.39E-06	0.00017	5.25094
ALOX12P	6.16109	-3.0926	10.8523	3.64E-05	0.00071	2.60765
MAL2	6.18108	2.3454	29.2719	1.06E-07	2.74E-05	8.86563
LOXL1	6.1904	1.74281	19.9069	1.05E-06	9.09E-05	6.51983
CAPN13	6.26806	-0.5769	28.2447	1.31E-07	3.07E-05	8.65738
MUC16	6.32437	1.43352	12.2294	1.83E-05	0.00047	3.38249
LRRN1	6.35287	-3.298	8.36328	0.00016	0.00179	0.93647
NTNG1	6.37264	-2.6397	18.7063	1.52E-06	0.00011	6.12581
UCA1	6.45541	-1.1202	8.76427	0.00012	0.00152	1.23392
PKP3	6.46335	0.7696	24.4641	3.09E-07	4.65E-05	7.79865
VIL1	6.48597	-3.8998	18.1862	1.79E-06	0.00012	5.94625
ACP7	6.52997	-2.9716	11.5525	2.54E-05	0.00057	3.01288
NID2	6.54146	1.20651	13.2802	1.13E-05	0.00035	3.91793
KRT19P1	6.55758	-0.8765	14.5733	6.58E-06	0.00025	4.52074
CDS1	6.6048	-0.5249	22.3867	5.23E-07	6.04E-05	7.25392
ADAMTS1	6.62206	1.61336	29.5292	1.01E-07	2.65E-05	8.9163
IRF6	6.63776	-0.5083	37.2086	2.54E-08	1.48E-05	10.1976
EYA2	6.69195	1.03045	16.763	2.89E-06	0.00016	5.42442
TAGLN	6.72792	4.78319	19.7969	1.08E-06	9.18E-05	6.48487
BSPRY	6.74568	-1.8815	43.2629	1.03E-08	1.03E-05	10.9634

SPINT2	6.75734	3.46937	22.6649	4.86E-07	5.80E-05	7.33029
SLCO3A1	6.77774	-1.1681	10.9757	3.41E-05	0.00069	2.68083
STXBP6	6.78408	-2.827	26.6604	1.85E-07	3.46E-05	8.31624
MUC4	6.86024	-2.3865	7.22912	0.00036	0.00294	0.0231
MISP	7.06281	-0.7813	36.5636	2.81E-08	1.48E-05	10.1049
ST14	7.0987	2.07416	48.1631	5.42E-09	7.87E-06	11.4689
TRIM29	7.11968	-1.6952	6.83809	0.00048	0.00362	-0.3195
RBBP8NL	7.12053	-3.8965	31.3487	7.05E-08	2.27E-05	9.25859
BICDL2	7.14518	-2.0536	14.333	7.25E-06	0.00026	4.41298
OVOL1	7.17481	-2.1677	19.7333	1.10E-06	9.21E-05	6.46454
SCEL	7.18121	-3.9597	26.6636	1.85E-07	3.46E-05	8.31696
NRXN3	7.18717	-0.6313	27.7859	1.45E-07	3.16E-05	8.56117
EPHA1	7.23251	0.36142	32.9367	5.25E-08	2.01E-05	9.53606
TRPS1	7.26961	-0.6329	9.44567	8.04E-05	0.00115	1.71259
TMEM125	7.31076	-0.9905	12.5157	1.60E-05	0.00043	3.53278
SFTA2	7.34025	-0.1458	23.972	3.48E-07	4.82E-05	7.67478
MYL9	7.42118	2.74844	23.908	3.54E-07	4.86E-05	7.65845
KDF1	7.42646	-1.2855	16.5886	3.08E-06	0.00016	5.35719
SFN	7.43593	2.01942	77.4811	3.14E-10	1.92E-06	13.2524
IGFBP5	7.43596	-0.6941	11.9668	2.07E-05	0.00051	3.24155
DMD	7.44446	1.197	25.4145	2.46E-07	4.17E-05	8.02942
PCDH7	7.50198	-2.0913	19.5063	1.18E-06	9.43E-05	6.3914
MBNL3	7.55085	-1.6782	31.5198	6.83E-08	2.27E-05	9.2894
TRHDE-A	7.65579	-2.696	33.8593	4.45E-08	2.01E-05	9.6889
FRAS1	7.70387	-0.5007	12.7641	1.43E-05	0.0004	3.66044
TNS4	7.73514	-0.4927	17.3567	2.36E-06	0.00014	5.64779
COL11A1	7.78026	-1.5086	19.8008	1.08E-06	9.18E-05	6.48612
VTCN1	7.88982	0.14775	16.03	3.76E-06	0.00018	5.13663
HS6ST2	7.90469	-1.2788	20.8397	8.00E-07	7.95E-05	6.8078
SMIM22	7.9332	-2.0721	39.2201	1.85E-08	1.35E-05	10.4719
C1orf210	7.96365	-1.6455	54.2549	2.66E-09	5.15E-06	11.9877
CLDN4	7.97093	1.25588	24.4596	3.09E-07	4.65E-05	7.79752
FAT3	7.98889	-0.7488	18.2338	1.76E-06	0.00012	5.96289
ATP2C2	8.03789	-1.5556	9.80344	6.51E-05	0.00101	1.95146
FLRT2	8.06204	-3.0104	21.9675	5.85E-07	6.37E-05	7.13667
CDH1	8.14383	0.55488	27.8471	1.43E-07	3.16E-05	8.57412
PRSS8	8.14414	1.52795	23.0457	4.40E-07	5.46E-05	7.43307
HEPHL1	8.25991	-3.2519	13.9855	8.37E-06	0.00029	4.2538
RAB25	8.58665	-1.8008	32.655	5.53E-08	2.04E-05	9.48819
S100A14	8.59818	1.84034	20.1015	9.90E-07	8.98E-05	6.58116
MACC1	8.6308	-2.1613	24.6781	2.93E-07	4.59E-05	7.85155
KRT19	8.65323	1.87885	27.5001	1.54E-07	3.32E-05	8.50018
TRHDE	8.65662	-1.0487	30.5738	8.19E-08	2.40E-05	9.11614
PRSS22	8.75649	-0.9823	18.6359	1.55E-06	0.00011	6.10184
KRT7	8.84897	3.85686	36.5066	2.84E-08	1.48E-05	10.0966
KRT17	9.09994	-0.2889	15.2523	5.04E-06	0.00021	4.8155
TMPRSS4	9.15818	-1.2147	16.1543	3.60E-06	0.00017	5.1864
EPS8L1	9.30901	0.00459	47.0582	6.23E-09	8.05E-06	11.3625
GALNT3	9.32962	-1.2198	18.5165	1.61E-06	0.00011	6.06092
TMEM30B	9.35363	-0.6607	24.5468	3.03E-07	4.65E-05	7.81916
ESRP1	9.39537	0.41541	22.4245	5.18E-07	6.04E-05	7.26437
CBLC	9.49805	-0.8881	17.9647	1.92E-06	0.00012	5.86804
GRHL2	9.51914	-1.3309	31.4876	6.87E-08	2.27E-05	9.28362
EPPK1	9.69086	0.09465	18.6716	1.53E-06	0.00011	6.114
FBN2	9.93886	0.83574	28.0628	1.36E-07	3.07E-05	8.61947

Supplementary Table 4: Overlapped gene in GSE98230 and GSE141630

Up-regulated overlapped gene	Down-regulated overlapped gene
GALNT14	SSC5D
TDO2	CRYAB
RUNX3	SLC44A2
LINC00882	STC2
COL12A1	EEF1AKMT1
HSPA12A	SNN
MMP1	ARID3BP1
HTRA1	MYLK4
LMX1B	TMEM169
PLPP2	CTTNBP2NL
HOXB13	TNS1
PRDM16	STARD13
MIRLET7BHG	LRCH2
PRKCB	DNAJC3-DT
CA13	TMSB15A
KIF26B	WDR25
DPP4	ADAMTS1
STK32B	SORT1
MLKL	ATXN1
HMX1	TRIM38
COL14A1	ANKRD24
RTN4RL2	CRIM1
GRIN2B	ZNF204P
PLPP3	C2orf15
AGPAT3	ERBB4
TNFSF14	UGCG
OSGIN1	ESPN
CCPG1	MISP
SLC1A1	ERP27
MAMLD1	NRXN3
LAMC3	PDZRN3
RNF144A	EBI3
KCNH3	EDNRB
ACSS1	ERVMER34-1
FOXF2	ACTL8
KITLG	ZBTB8B
LDAF1	RINL
MAPT	EDNRA
CAMK2N1	PRSS8
TFCP2L1	ZBED9
SH3RF3	ASNS
SFXN3	FAT3
CACHD1	FZD3
ZNF804A	NTF4
AKR1C2	SBK1
CDHR1	THSD4
GPR27	SERTAD4
TESC	NECTIN4
SDK2	MYLK3
TBXAS1	DMD
EEF1A2	FBLIM1
CFAP20DC	RNF152
AR	LCP1
C3	DLG3
FAM81A	S100A1
NPTX1	TSPAN2

SYNE3	BAGE2
MCOLN3	FBXO27
JUN	PTGER2
C6orf141	TBC1D8B
MCC	CALD1
RASSF2	LRG1
TTLL7	EXPH5
NEK10	CACNG6
GBX2	ADGRF1
PHLDA2	CARMN
ITGA6	LINC01629
OGFRL1	SYBU
ADORA1	RIGI
MCOLN2	HOXB5
NPAS1	FNBP1L
PLD1	BMF
KIF5C	FUT1
TMEM64	COBLL1
CLDN11	LRRC8D-DT
CA12	SLC6A15
SNHG26	INHBE
LGI2	CSF1
PMP22	APLF
DAPK2	ARID3A
LINC00645	FRAS1
ZNF703	ADPRHL1
TRABD2A	CBLC
MSRA	RNF144B
GDPD5	SH3BGRL2
TMX4	ADGRG1
TRNP1	JUP
ANTXR1	PAX6
KLF2	IGFBP5
STAT1	EPAS1
GMPR	CASZ1
TMEM98	TAGLN
GCAWKR	GCNT4
PLAAT3	FOLR1
GALNT16	RCBTB2
EGR1	F3
TNFRSF8	SERTM1
CLMN	LINC01234
COL16A1	ANXA3
SLC30A3	TGFA
IL6R	CYB5A
DNAAF11	TSPAN12
CSPG5	SAP30
TIMP1	TMTC2
MRAP2	RND3
CHRD	MGAT3
KIF7	MESTP3
RIPK2	FBLN2
ARHGAP31	ADAM19
A4GALT	SIX1
PPP1R16B	NLGN4X
FRMD8	CPA4
NIPAL2	PTPRK
ITPR1	EGLN3

NR2F2	ST8SIA1
STEAP3	PNMA3
KAT2B	NELL2
CNRIP1	TENM1
GAB3	FLRT2
MAN1C1	MPZL2
CABYR	P2RY2
AKR1C3	SH3BP4
MEGF11	FOXL2NB
CASP10	RBM47
SEMA3G	RNF180
PLEKHA7	FADS2
MCIDAS	INA
MAP3K7CL	IL32
MMP10	MAP1LC3A
YPEL2	TENM3
ZCCHC12	TGFB2
MYO7A	CCDC80
EDA	NID2
FUT4	FBN2
SERINC5	MSR1
EEPD1	PRKCH
SPATA20	DSEL
PDK1-AS1	MYO3B
PRKCA	PLAG1
KCNC4	MBP
CCDC148	BEX4
TMEM144	ACTA2
DYNLT3	ZDHHC2
PDE6A	APOL1
OSGIN2	ELF3
SLC19A3	ANKRD1
DLK2	SUN3
HSPA4L	PRICKLE1
CDO1	DTNA
MTSS1	SLC7A8
MORC4	PDE3A
RHBDL3	TMCC3
ABTB2	IFITM1
NDRG2	NEDD9
FXYD6	APOE
RNF157	JPH1
REEP2	ADAMTS5
SEPTIN4	DNAJC15
	TAP1
	JPH2
	THRB
	APOL6
	TRPS1
	SCHIP1
	EPM2AIP1
	HOXB9
	ZNF439
	LMO4
	MDGA1
	MESTP1
	TRHDE-AS1
	PRTFDC1

	TMEM47
	FOXL2
	MDFI
	EFNB2
	CCSER1
	SHROOM3
	MAT1A
	NAALAD2
	ZNF432
	CNTNAP2
	FILIP1L
	CNN1
	RASSF9
	MBNL3
	LACC1
	CRABP2
	FREM1
	CRABP1
	USP53
	FRY
	ZNF738
	SOX9
	GBP5
	TRHDE
	CEMIP2
	NTNG1
	TMEM30B
	TIMP3
	FLNC
	LAYN
	COL1A2
	DLX6
	DLX5
	HDAC9
	MEST
	DLX6-AS1

Supplementary Table 5: The result of GO analysis

id	num	pvalue	per	ratio	class	ID	Descrption	Pvalue	Qvalue	Up	Down	fg_num	bg_num
GO:0048731 system development	158	9.21E-16	48.916	0.032	Biological	GO:00487	system development	9.21E-16	4.37E-12	0	0	158	4969
GO:0009653 anatomical structure morphogenesis	107	1.75E-15	33.127	0.039	Biological	GO:00096	anatomical structure morphogenesis	1.75E-15	4.37E-12	0	0	107	2749
GO:0048856 anatomical structure development	176	3.17E-15	54.489	0.03	Biological	GO:00488	anatomical structure development	3.17E-15	5.29E-12	0	0	176	5916
GO:0007275 multicellular organism development	160	5.28E-14	49.536	0.03	Biological	GO:00072	multicellular organism development	5.28E-14	6.59E-11	0	0	160	5283
GO:0032502 developmental process	183	2.50E-13	56.656	0.028	Biological	GO:00325	developmental process	2.50E-13	2.50E-10	0	0	183	6532
GO:0009888 tissue development	82	7.31E-13	25.387	0.041	Biological	GO:00098	tissue development	7.31E-13	6.08E-10	0	0	82	1997
GO:0009887 animal organ morphogenesis	55	2.33E-12	17.028	0.051	Biological	GO:00098	animal organ morphogenesis	2.33E-12	1.67E-09	0	0	55	1070
GO:0048513 animal organ development	120	2.86E-12	37.152	0.033	Biological	GO:00485	animal organ development	2.86E-12	1.79E-09	0	0	120	3637
GO:0048646 anatomical structure formation involved in morphogenesis	56	2.73E-11	17.337	0.048	Biological	GO:00486	anatomical structure formation involved in morphogenesis	2.73E-11	1.52E-08	0	0	56	1174
GO:0016477 cell migration	65	3.80E-11	20.124	0.043	Biological	GO:00164	cell migration	3.80E-11	1.90E-08	0	0	65	1504
GO:0032501 multicellular organismal process	198	4.97E-11	61.3	0.026	Biological	GO:00325	multicellular organismal process	4.97E-11	2.02E-08	0	0	198	7687
GO:0030154 cell differentiation	131	5.25E-11	40.557	0.03	Biological	GO:00301	cell differentiation	5.25E-11	2.02E-08	0	0	131	4301
GO:0001568 blood vessel development	41	5.27E-11	12.693	0.058	Biological	GO:00015	blood vessel development	5.27E-11	2.02E-08	0	0	41	706
GO:0048869 cellular developmental process	131	1.81E-10	40.557	0.03	Biological	GO:00488	cellular developmental process	1.81E-10	6.44E-08	0	0	131	4376
GO:0001944 vasculature development	41	1.95E-10	12.693	0.056	Biological	GO:00019	vasculature development	1.95E-10	6.51E-08	0	0	41	737
GO:0060429 epithelium development	55	3.51E-10	17.028	0.045	Biological	GO:00604	epithelium development	3.51E-10	1.10E-07	0	0	55	1220
GO:0007155 cell adhesion	63	5.88E-10	19.505	0.041	Biological	GO:00071	cell adhesion	5.88E-10	1.64E-07	0	0	63	1530
GO:0030334 regulation of cell migration	46	5.91E-10	14.241	0.05	Biological	GO:00303	regulation of cell migration	5.91E-10	1.64E-07	0	0	46	927
GO:0022610 biological adhesion	63	7.06E-10	19.505	0.041	Biological	GO:00226	biological adhesion	7.06E-10	1.86E-07	0	0	63	1537
GO:0006928 movement of cell or subcellular component	78	9.94E-10	24.149	0.036	Biological	GO:00069	movement of cell or subcellular component	9.94E-10	2.48E-07	0	0	78	2138
GO:0072359 circulatory system development	51	1.33E-09	15.789	0.045	Biological	GO:00723	circulatory system development	1.33E-09	3.17E-07	0	0	51	1123
GO:0032879 regulation of localization	93	1.90E-09	28.793	0.033	Biological	GO:00328	regulation of localization	1.90E-09	4.31E-07	0	0	93	2798
GO:0071944 cell periphery	164	1.98E-09	48.665	0.026	Cellular C	GO:00719	cell periphery	1.98E-09	1.06E-06	0	0	164	6197
GO:0035239 tube morphogenesis	43	2.34E-09	13.313	0.05	Biological	GO:00352	tube morphogenesis	2.34E-09	4.83E-07	0	0	43	867
GO:0048870 cell motility	66	2.42E-09	20.433	0.039	Biological	GO:00488	cell motility	2.42E-09	4.83E-07	0	0	66	1701
GO:0051674 localization of cell	66	2.42E-09	20.433	0.039	Biological	GO:00516	localization of cell	2.42E-09	4.83E-07	0	0	66	1701
GO:0035295 tube development	49	3.04E-09	15.17	0.045	Biological	GO:00352	tube development	3.04E-09	5.65E-07	0	0	49	1080
GO:0042127 regulation of cell population proliferation	65	3.05E-09	20.124	0.039	Biological	GO:00421	regulation of cell population proliferation	3.05E-09	5.65E-07	0	0	65	1672
GO:2000145 regulation of cell motility	46	4.05E-09	14.241	0.047	Biological	GO:20001	regulation of cell motility	4.05E-09	7.22E-07	0	0	46	985
GO:0040011 locomotion	70	4.77E-09	21.672	0.037	Biological	GO:00400	locomotion	4.77E-09	8.21E-07	0	0	70	1888
GO:0050678 regulation of epithelial cell proliferation	26	5.53E-09	8.05	0.07	Biological	GO:00506	regulation of epithelial cell proliferation	5.53E-09	8.99E-07	0	0	26	373
GO:0040012 regulation of locomotion	47	5.59E-09	14.551	0.046	Biological	GO:00400	regulation of locomotion	5.59E-09	8.99E-07	0	0	47	1030
GO:0022008 neurogenesis	65	5.76E-09	20.124	0.038	Biological	GO:00220	neurogenesis	5.76E-09	8.99E-07	0	0	65	1699
GO:0007399 nervous system development	85	8.85E-09	26.316	0.033	Biological	GO:00073	nervous system development	8.85E-09	1.34E-06	0	0	85	2538
GO:0048699 generation of neurons	61	9.71E-09	18.885	0.039	Biological	GO:00486	generation of neurons	9.71E-09	1.43E-06	0	0	61	1566
GO:0030855 epithelial cell differentiation	37	1.40E-08	11.455	0.051	Biological	GO:00308	epithelial cell differentiation	1.40E-08	2.00E-06	0	0	37	721
GO:0048518 positive regulation of biological process	164	1.45E-08	50.774	0.026	Biological	GO:00485	positive regulation of biological process	1.45E-08	2.02E-06	0	0	164	6318
GO:0034330 cell junction organization	37	2.76E-08	11.455	0.05	Biological	GO:00343	cell junction organization	2.76E-08	3.73E-06	0	0	37	740
GO:0051270 regulation of cellular component movement	46	3.21E-08	14.241	0.044	Biological	GO:00512	regulation of cellular component movement	3.21E-08	4.22E-06	0	0	46	1054
GO:0048514 blood vessel morphogenesis	33	3.67E-08	10.217	0.053	Biological	GO:00485	blood vessel morphogenesis	3.67E-08	4.70E-06	0	0	33	619
GO:0048522 positive regulation of cellular process	152	4.58E-08	47.059	0.026	Biological	GO:00485	positive regulation of cellular process	4.58E-08	5.72E-06	0	0	152	5796
GO:0051239 regulation of multicellular organismal process	88	4.98E-08	27.245	0.032	Biological	GO:00512	regulation of multicellular organismal process	4.98E-08	6.07E-06	0	0	88	2765
GO:0050673 epithelial cell proliferation	27	5.40E-08	8.359	0.061	Biological	GO:00506	epithelial cell proliferation	5.40E-08	6.42E-06	0	0	27	446
GO:0030182 neuron differentiation	55	5.58E-08	17.028	0.039	Biological	GO:00301	neuron differentiation	5.58E-08	6.49E-06	0	0	55	1409
GO:0008283 cell population proliferation	69	8.16E-08	21.362	0.035	Biological	GO:00082	cell population proliferation	8.16E-08	9.26E-06	0	0	69	1984
GO:0001667 ameboidal-type cell migration	26	9.41E-08	8.05	0.061	Biological	GO:00016	ameboidal-type cell migration	9.41E-08	1.04E-05	0	0	26	429
GO:0030198 extracellular matrix organization	22	1.36E-07	6.811	0.068	Biological	GO:00301	extracellular matrix organization	1.36E-07	1.47E-05	0	0	22	324
GO:0043062 extracellular structure organization	22	1.43E-07	6.811	0.068	Biological	GO:00430	extracellular structure organization	1.43E-07	1.52E-05	0	0	22	325
GO:0045229 external encapsulating structure organization	22	1.59E-07	6.811	0.067	Biological	GO:00452	external encapsulating structure organization	1.59E-07	1.66E-05	0	0	22	327
GO:0090596 sensory organ morphogenesis	20	2.40E-07	6.192	0.071	Biological	GO:00905	sensory organ morphogenesis	2.40E-07	2.45E-05	0	0	20	281
GO:0062023 collagen-containing extracellular matrix	25	3.04E-07	7.418	0.058	Cellular C	GO:00620	collagen-containing extracellular matrix	3.04E-07	6.88E-05	0	0	25	430
GO:0031012 extracellular matrix	29	4.96E-07	8.605	0.051	Cellular C	GO:00310	extracellular matrix	4.96E-07	6.88E-05	0	0	29	567
GO:0001525 angiogenesis	28	5.04E-07	8.669	0.053	Biological	GO:00015	angiogenesis	5.04E-07	5.03E-05	0	0	28	530
GO:0030312 external encapsulating structure	29	5.14E-07	8.605	0.051	Cellular C	GO:00303	external encapsulating structure	5.14E-07	6.88E-05	0	0	29	568
GO:0050793 regulation of developmental process	79	6.61E-07	24.458	0.031	Biological	GO:00507	regulation of developmental process	6.61E-07	6.36E-05	0	0	79	2528
GO:0048468 cell development	71	6.62E-07	21.981	0.033	Biological	GO:00484	cell development	6.62E-07	6.36E-05	0	0	71	2182
GO:0008284 positive regulation of cell population proliferation	39	1.60E-06	12.074	0.041	Biological	GO:00082	positive regulation of cell population proliferation	1.60E-06	0.00015	0	0	39	945
GO:0009719 response to endogenous stimulus	56	1.61E-06	17.337	0.035	Biological	GO:00097	response to endogenous stimulus	1.61E-06	0.00015	0	0	56	1605
GO:0007423 sensory organ development	29	1.66E-06	8.978	0.049	Biological	GO:00074	sensory organ development	1.66E-06	0.00015	0	0	29	596
GO:0098609 cell-cell adhesion	39	1.82E-06	12.074	0.041	Biological	GO:00986	cell-cell adhesion	1.82E-06	0.00016	0	0	39	950
GO:0048666 neuron development	44	1.89E-06	13.622	0.039	Biological	GO:00486	neuron development	1.89E-06	0.00017	0	0	44	1139
GO:0051240 positive regulation of multicellular organismal process	53	2.92E-06	16.409	0.035	Biological	GO:00512	positive regulation of multicellular organismal process	2.92E-06	0.00025	0	0	53	1514
GO:0010033 response to organic substance	88	3.62E-06	27.245	0.029	Biological	GO:00100	response to organic substance	3.62E-06	0.00031	0	0	88	3047
GO:0051179 localization	155	5.02E-06	47.988	0.024	Biological	GO:00511	localization	5.02E-06	0.00042	0	0	155	6369
GO:0009966 regulation of signal transduction	86	6.87E-06	26.625	0.029	Biological	GO:00099	regulation of signal transduction	6.87E-06	0.00056	0	0	86	3003
GO:0050808 synapse organization	24	6.95E-06	7.43	0.051	Biological	GO:00508	synapse organization	6.95E-06	0.00056	0	0	24	474
GO:0045785 positive regulation of cell adhesion	24	7.73E-06	7.43	0.05	Biological	GO:00457	positive regulation of cell adhesion	7.73E-06	0.00061	0	0	24	477
GO:0048592 eye morphogenesis	13	9.38E-06	4.025	0.08	Biological	GO:00485	eye morphogenesis	9.38E-06	0.00073	0	0	13	163
GO:0010646 regulation of cell communication	94	9.86E-06	29.102	0.028	Biological	GO:00106	regulation of cell communication	9.86E-06	0.00075	0	0	94	3403
GO:0050680 negative regulation of epithelial cell proliferation	12	9.96E-06	3.715	0.086	Biological	GO:00506	negative regulation of epithelial cell proliferation	9.96E-06	0.00075	0	0	12	140
GO:0005886 plasma membrane	140	1.07E-05	41.543	0.025	Cellular C	GO:00058	plasma membrane	1.07E-05	0.00114	0	0	140	5713
GO:0023051 regulation of signaling	94	1.13E-05	29.102	0.028	Biological	GO:00230	regulation of signaling	1.13E-05	0.00084	0	0	94	3414
GO:0001655 urogenital system development	20	1.27E-05	6.192	0.055	Biological	GO:00016	urogenital system development	1.27E-05	0.00094	0	0	20	364
GO:0005604 basement membrane	10	1.30E-05	2.967	0.1	Cellular C	GO:00056	basement membrane	1.30E-05	0.00116	0	0	10	100
GO:0048568 embryonic organ development	23	1.42E-05	7.121	0.05	Biological	GO:00485	embryonic organ development	1.42E-05	0.00103	0	0	23	462
GO:0030054 cell junction	67	1.63E-05	19.881	0.03	Cellular C	GO:00300	cell junction	1.63E-05	0.00124	0	0	67	2230
GO:0048729 tissue morphogenesis	28	1.64E-05	8.669	0.044	Biological	GO:00487	tissue morphogenesis	1.64E-05	0.00117	0	0	28	636
GO:0060485 mesenchyme development	18	1.66E-05	5.573	0.058	Biological	GO:00604	mesenchyme development	1.66E-05	0.00117	0	0	18	310
GO:0150063 visual system development	21	1.72E-05	6.502	0.052	Biological	GO:01500	visual system development	1.72E-05	0.00119	0	0	21	403
GO:0090130 tissue migration	18	1.74E-05	5.573	0.058	Biological	GO:00901	tissue migration	1.74E-05	0.00119	0	0	18	311
GO:0000902 cell morphogenesis	39	1.85E-05	12.074	0.037	Biological	GO:00009	cell morphogenesis	1.85E-05	0.00125	0	0	39	1049
GO:0022612 gland morphogenesis	11	1.96E-05	3.406	0.087	Biological	GO:00226	gland morphogenesis	1.96E-05	0.00131	0	0	11	126
GO:0048880 sensory system development	21	2.14E-05	6.502	0.051	Biological	GO:00488	sensory system development	2.14E-05	0.00141	0	0	21	409
GO:1902952 positive regulation of dendritic spine maintenance	3	2.37E-05	0.929	0.75	Biological	GO:19029	positive regulation of dendritic spine maintenance	2.37E-05	0.00154	0	0	3	4
GO:0043010 camera-type eye development	19	2.58E-05	5.882	0.054	Biological	GO:00430	camera-type eye development	2.58E-05	0.00165	0	0	19	351
GO:0048598 embryonic morphogenesis	27	2.66E-05	8.359	0.044	Biological	GO:00485	embryonic morphogenesis	2.66E-05	0.00168	0	0	27	618
GO:0071495 cellular response to endogenous stimulus	46	3.18E-05	14.241	0.034	Biological	GO:00714	cellular response to endogenous stimulus	3.18E-05	0.00197	0	0	46	1356
GO:0071559 response to transforming growth factor beta	16	3.20E-05	4.954	0.06	Biological	GO:00715	response to transforming growth factor beta	3.20E-05	0.00197	0	0	16	266
GO:0031252 cell leading edge	21	3.23E-05	6.231	0.049	Cellular C	GO:00312	cell leading edge	3.23E-05	0.00216	0	0	21	425
GO:1902950 regulation of dendritic spine maintenance	4	3.23E-05	1.238	0.364	Biological	GO:19029	regulation of dendritic spine maintenance	3.23E-05	0.00197	0	0	4	11
GO:0022603 regulation of anatomical structure morphogenesis	35	3.28E-05	10.836	0.038	Biological	GO:00226	regulation of anatomical structure morphogenesis	3.28E-05	0.00197	0	0	35	921
GO:0002009 morphogenesis of an epithelium	24	3.92E-05	7.43	0.046	Biological	GO:00020	morphogenesis of an epithelium	3.92E-05	0.00232	0	0	24	527
GO:0030155 regulation of cell adhesion	31	3.94E-05	9.598	0.04	Biological	GO:00301	regulation of cell adhesion	3.94E-05	0.00232	0	0	31	778
GO:0048762 mesenchymal cell differentiation	15	4.20E-05	4.644	0.062	Biological	GO:00487	mesenchymal cell differentiation	4.20E-05	0.00242	0	0	15	243
GO:0010631 epithelial cell migration	17	4.22E-05	5.263	0.056	Biological	GO:00106	epithelial cell migration	4.22E-05	0.00242	0	0	17	302
GO:0048562 embryonic organ morphogenesis	17	4.40E-05	5.263	0.056	Biological	GO:00485	embryonic organ morphogenesis	4.40E-05	0.00249	0	0	17	303
GO:0008285 negative regulation of cell population proliferation	29	4.43E-05	8.978	0.041	Biological	GO:00082	negative regulation of cell population proliferation	4.43E-05	0.00249	0	0	29	709
GO:0070848 response to growth factor	29	4.55E-05	8.978	0.041	Biological	GO:00708	response to growth factor	4.55E-05	0.00252	0	0	29	710
GO:0001654 eye development	20	4.71E-05	6.192	0.05	Biological	GO:00016	eye development	4.71E-05	0.00259	0	0	20	399
GO:0090132 epithelium migration	17	4.77E-05	5.263	0.056	Biological	GO:00901	epithelium migration	4.77E-05	0.00259	0	0	17	305
GO:0031175 neuron projection development	36	6.27E-05	11.146	0.036	Biological	GO:00311	neuron projection development	6.27E-05	0.00337	0	0	36	990
GO:0061564 axon development	23	6.41E-05	7.121	0.045	Biological	GO:00615	axon development	6.41E-05	0.00337	0	0	23	509
GO:0014033 neural crest cell differentiation	9	6.42E-05	2.786	0.094	Biological	GO:00140	neural crest cell differentiation	6.42E-05	0.00337	0	0	9	96
GO:0034329 cell junction assembly	21	7.03E-05	6.502	0.047	Biological	GO:00343	cell junction assembly	7.03E-05	0.00366	0	0	21	444
GO:0048608 reproductive structure development	21	7.50E-05	6.502	0.047	Biological	GO:00486	reproductive structure development	7.50E-05	0.00386	0	0	21	446
GO:0045765 regulation of angiogenesis	16	7.93E-05	4.954	0.056	Biological	GO:00457	regulation of angiogenesis	7.93E-05	0.00404	0	0	16	287
GO:1901700 response to oxygen-containing compound	52	8.23E-05	16.099	0.031	Biological	GO:19017	response to oxygen-containing compound	8.23E-05	0.00415	0	0	52	1665
GO:0061458 reproductive system development	21	8.50E-05	6.502	0.047	Biological	GO:00614	reproductive system development	8.50E-05	0.00425	0	0	21	450
GO:0061061 muscle structure development	27	9.02E-05	8.359	0.041	Biological	GO:00610	muscle structure development	9.02E-05	0.00442	0	0	27	664
GO:0071560 cellular response to transforming growth factor beta stimul	15	9.03E-05	4.644	0.058	Biological	GO:00715	cellular response to transforming growth factor beta stimulus	9.03E-05	0.00442	0	0	15	260
GO:1901342 regulation of vasculature development	16	9.33E-05	4.954	0.055	Biological	GO:19013	regulation of vasculature development	9.33E-05	0.0045	0	0	16	291
GO:0010842 retina layer formation	5	9.37E-05	1.548	0.192	Biological	GO:00108	retina layer formation	9.37E-05	0.0045	0	0	5	26
GO:0001755 neural crest cell migration	7	9.49E-05	2.167	0.119	Biological	GO:00017	neural crest cell migration	9.49E-05	0.00451	0	0	7	59
GO:0072001 renal system development	17	0.0001	5.263	0.052	Biological	GO:00720	renal system development	0.0001	0.00473	0	0	17	324
GO:0005593 FACIT collagen trimer	3	0.00011	0.89	0.5	Cellular C	GO:00055	FACIT collagen trimer	0.00011	0.00663	0	0	3	6
GO:0042221 response to chemical	110	0.00011	34.056	0.025	Biological	GO:00422	response to chemical	0.00011	0.00532	0	0	110	4401
GO:1904428 negative regulation of tubulin deacetylation	3	0.00012	0.929	0.5	Biological	GO:19044	negative regulation of tubulin deacetylation	0.00012	0.00533	0	0	3	6
GO:0051094 positive regulation of developmental process	44	0.00012	13.622	0.033	Biological	GO:00510	positive regulation of developmental process	0.00012	0.00553	0	0	44	1351
GO:0003407 neural retina development	8	0.00012	2.477	0.098	Biological	GO:00034	neural retina development	0.00012	0.00553	0	0	8	82
GO:0072006 nephron development	11	0.00012	3.406	0.071	Biological	GO:00720	nephron development	0.00012	0.00553	0	0	11	154
GO:0007166 cell surface receptor signaling pathway	77	0.00013	23.839	0.027	Biological	GO:00071	cell surface receptor signaling pathway	0.00013	0.00553	0	0	77	2820
GO:0050679 positive regulation of epithelial cell proliferation	13	0.00013	4.025	0.062	Biological	GO:00506	positive regulation of epithelial cell proliferation	0.00013	0.00553	0	0	13	209
GO:1905048 regulation of metallopeptidase activity	4	0.00013	1.238	0.267	Biological	GO:19050	regulation of metallopeptidase activity	0.00013	0.00553	0	0	4	15
GO:0048523 negative regulation of cellular process	120	0.00013	37.152	0.024	Biological	GO:00485	negative regulation of cellular process	0.00013	0.00553	0	0	120	4913
GO:0071363 cellular response to growth factor stimulus	27	0.00013	8.359	0.04	Biological	GO:00713	cellular response to growth factor stimulus	0.00013	0.00574	0	0	27	680
GO:0014032 neural crest cell development	8	0.00013	2.477	0.096	Biological	GO:00140	neural crest cell development	0.00013	0.00575	0	0	8	83
GO:0060070 canonical Wnt signaling pathway	16	0.00014	4.954	0.053	Biological	GO:00600	canonical Wnt signaling pathway	0.00014	0.0058	0	0	16	301
GO:0007167 enzyme linked receptor protein signaling pathway	35	0.00014	10.836	0.035	Biological	GO:00071	enzyme linked receptor protein signaling pathway	0.00014	0.0058	0	0	35	990
GO:0002064 epithelial cell development	14	0.00014	4.334	0.058	Biological	GO:00020	epithelial cell development	0.00014	0.00597	0	0	14	241
GO:0048593 camera-type eye morphogenesis	10	0.00015	3.096	0.076	Biological	GO:00485	camera-type eye morphogenesis	0.00015	0.00638	0	0	10	132
GO:0015629 actin cytoskeleton	22	0.00015	6.528	0.043	Cellular C	GO:00156	actin cytoskeleton	0.00015	0.00829	0	0	22	510
GO:0007154 cell communication	151	0.00016	46.749	0.023	Biological	GO:00071	cell communication	0.00016	0.00649	0	0	151	6536
GO:0048519 negative regulation of biological process	128	0.00017	39.628	0.024	Biological	GO:00485	negative regulation of biological process	0.00017	0.00677	0	0	128	5349
GO:0060042 retina morphogenesis in camera-type eye	7	0.00018	2.167	0.108	Biological	GO:00600	retina morphogenesis in camera-type eye	0.00018	0.0071	0	0	7	65
GO:0044093 positive regulation of molecular function	47	0.00018	14.551	0.031	Biological	GO:00440	positive regulation of molecular function	0.00018	0.00717	0	0	47	1502
GO:0045597 positive regulation of cell differentiation	32	0.00018	9.907	0.036	Biological	GO:00455	positive regulation of cell differentiation	0.00018	0.00717	0	0	32	885
GO:0045595 regulation of cell differentiation	49	0.00019	15.17	0.031	Biological	GO:00455	regulation of cell differentiation	0.00019	0.00757	0	0	49	1592
GO:0008015 blood circulation	22	0.00019	6.811	0.043	Biological	GO:00080	blood circulation	0.00019	0.0076	0	0	22	513
GO:0014031 mesenchymal cell development	8	0.0002	2.477	0.091	Biological	GO:00140	mesenchymal cell development	0.0002	0.00777	0	0	8	88
GO:0008219 cell death	61	0.0002	18.885	0.029	Biological	GO:00082	cell death	0.0002	0.00777	0	0	61	2126
GO:0065009 regulation of molecular function	71	0.0002	21.981	0.027	Biological	GO:00650	regulation of molecular function	0.0002	0.00777	0	0	71	2583
GO:0048732 gland development	20	0.00022	6.192	0.045	Biological	GO:00487	gland development	0.00022	0.00824	0	0	20	447
GO:0048864 stem cell development	8	0.00022	2.477	0.09	Biological	GO:00488	stem cell development	0.00022	0.00824	0	0	8	89
GO:0001822 kidney development	16	0.00022	4.954	0.051	Biological	GO:00018	kidney development	0.00022	0.00832	0	0	16	314
GO:0060041 retina development in camera-type eye	11	0.00023	3.406	0.067	Biological	GO:00600	retina development in camera-type eye	0.00023	0.0084	0	0	11	165
GO:0071310 cellular response to organic substance	67	0.00023	20.743	0.028	Biological	GO:00713	cellular response to organic substance	0.00023	0.00847	0	0	67	2409
GO:0030879 mammary gland development	10	0.00024	3.096	0.072	Biological	GO:00308	mammary gland development	0.00024	0.0086	0	0	10	139
GO:0085029 extracellular matrix assembly	6	0.00025	1.858	0.122	Biological	GO:00850	extracellular matrix assembly	0.00025	0.00916	0	0	6	49
GO:0030336 negative regulation of cell migration	15	0.00027	4.644	0.052	Biological	GO:00303	negative regulation of cell migration	0.00027	0.00957	0	0	15	287
GO:0010632 regulation of epithelial cell migration	13	0.00028	4.025	0.057	Biological	GO:00106	regulation of epithelial cell migration	0.00028	0.0101	0	0	13	227

GO:0007165 signal transduction	139	0.00029	43.034	0.023	Biological	GO:0007	signal transduction	0.00029	0.01031	0	0	139	5982
GO:0120036 plasma membrane bounded cell projection organization	47	0.0003	14.551	0.031	Biological	GO:0120	plasma membrane bounded cell projection organization	0.0003	0.01044	0	0	47	1535
GO:0003779 actin binding	20	0.00031	5.848	0.044	Molecular	GO:0003	actin binding	0.00031	0.09031	0	0	20	451
GO:0009967 positive regulation of signal transduction	47	0.00031	14.551	0.031	Biological	GO:0009	positive regulation of signal transduction	0.00031	0.01082	0	0	47	1538
GO:0050896 response to stimulus	194	0.00031	60.062	0.022	Biological	GO:0050	response to stimulus	0.00031	0.01082	0	0	194	8949
GO:0030424 axon	25	0.00031	7.418	0.039	Cellular C	GO:0030	axon	0.00031	0.01518	0	0	25	648
GO:0060509 type I pneumocyte differentiation	3	0.00031	0.929	0.375	Biological	GO:0060	type I pneumocyte differentiation	0.00031	0.01082	0	0	3	8
GO:0030335 positive regulation of cell migration	22	0.00032	6.811	0.041	Biological	GO:0030	positive regulation of cell migration	0.00032	0.01102	0	0	22	532
GO:0007382 specification of segmental identity, maxillary segment	2	0.00033	0.619	1	Biological	GO:0007	specification of segmental identity, maxillary segment	0.00033	0.01118	0	0	2	2
GO:0018993 somatic sex determination	2	0.00033	0.619	1	Biological	GO:0018	somatic sex determination	0.00033	0.01118	0	0	2	2
GO:0004962 endothelin receptor activity	2	0.00034	0.585	1	Molecular	GO:0004	endothelin receptor activity	0.00034	0.09031	0	0	2	2
GO:0035403 histone kinase activity (H3-T6 specific)	2	0.00034	0.585	1	Molecular	GO:0035	histone kinase activity (H3-T6 specific)	0.00034	0.09031	0	0	2	2
GO:0007416 synapse assembly	12	0.00035	3.715	0.059	Biological	GO:0007	synapse assembly	0.00035	0.01161	0	0	12	202
GO:0006915 apoptotic process	55	0.00036	17.028	0.029	Biological	GO:0006	apoptotic process	0.00036	0.012	0	0	55	1901
GO:0010647 positive regulation of cell communication	51	0.00038	15.789	0.03	Biological	GO:0010	positive regulation of cell communication	0.00038	0.01252	0	0	51	1727
GO:0070887 cellular response to chemical stimulus	80	0.00038	24.768	0.026	Biological	GO:0070	cellular response to chemical stimulus	0.00038	0.01252	0	0	80	3063
GO:0043954 cellular component maintenance	7	0.0004	2.167	0.095	Biological	GO:0043	cellular component maintenance	0.0004	0.01287	0	0	7	74
GO:1905114 cell surface receptor signaling pathway involved in cell-cell	23	0.0004	7.121	0.04	Biological	GO:1905	cell surface receptor signaling pathway involved in cell-cell signaling	0.0004	0.01287	0	0	23	577
GO:0023056 positive regulation of signaling	51	0.0004	15.789	0.029	Biological	GO:0023	positive regulation of signaling	0.0004	0.01287	0	0	51	1731
GO:0003008 system process	63	0.00041	19.505	0.028	Biological	GO:0003	system process	0.00041	0.01299	0	0	63	2273
GO:0097062 dendritic spine maintenance	4	0.00042	1.238	0.2	Biological	GO:0097	dendritic spine maintenance	0.00042	0.01326	0	0	4	20
GO:0012501 programmed cell death	56	0.00042	17.337	0.029	Biological	GO:0012	programmed cell death	0.00042	0.01332	0	0	56	1958
GO:0031589 cell-substrate adhesion	17	0.00043	5.263	0.046	Biological	GO:0031	cell-substrate adhesion	0.00043	0.01365	0	0	17	367
GO:0046872 metal ion binding	107	0.00046	31.287	0.025	Molecular	GO:0046	metal ion binding	0.00046	0.09031	0	0	107	4317
GO:2000146 negative regulation of cell motility	15	0.00047	4.644	0.05	Biological	GO:2000	negative regulation of cell motility	0.00047	0.01474	0	0	15	303
GO:0010811 positive regulation of cell-substrate adhesion	9	0.00051	2.786	0.071	Biological	GO:0010	positive regulation of cell-substrate adhesion	0.00051	0.01564	0	0	9	126
GO:0090043 regulation of tubulin deacetylation	4	0.00051	1.238	0.19	Biological	GO:0090	regulation of tubulin deacetylation	0.00051	0.01564	0	0	4	21
GO:0030030 cell projection organization	47	0.00051	14.551	0.03	Biological	GO:0030	cell projection organization	0.00051	0.01564	0	0	47	1573
GO:0060562 epithelial tube morphogenesis	16	0.00052	4.954	0.047	Biological	GO:0060	epithelial tube morphogenesis	0.00052	0.01583	0	0	16	339
GO:0051271 negative regulation of cellular component movement	15	0.00052	4.644	0.049	Biological	GO:0051	negative regulation of cellular component movement	0.00052	0.01583	0	0	15	306
GO:1903670 regulation of sprouting angiogenesis	5	0.00053	1.548	0.135	Biological	GO:1903	regulation of sprouting angiogenesis	0.00053	0.01583	0	0	5	37
GO:0060537 muscle tissue development	18	0.00053	5.573	0.044	Biological	GO:0060	muscle tissue development	0.00053	0.01583	0	0	18	408
GO:0045667 regulation of osteoblast differentiation	9	0.00054	2.786	0.071	Biological	GO:0045	regulation of osteoblast differentiation	0.00054	0.01588	0	0	9	127
GO:0040013 negative regulation of locomotion	16	0.00054	4.954	0.047	Biological	GO:0040	negative regulation of locomotion	0.00054	0.01588	0	0	16	340
GO:0010942 positive regulation of cell death	23	0.00055	7.121	0.039	Biological	GO:0010	positive regulation of cell death	0.00055	0.01626	0	0	23	591
GO:2000147 positive regulation of cell motility	22	0.00057	6.811	0.04	Biological	GO:2000	positive regulation of cell motility	0.00057	0.01666	0	0	22	555
GO:0042471 ear morphogenesis	9	0.0006	2.786	0.07	Biological	GO:0042	ear morphogenesis	0.0006	0.01742	0	0	9	129
GO:0016055 Wnt signaling pathway	19	0.0006	5.882	0.042	Biological	GO:0016	Wnt signaling pathway	0.0006	0.01742	0	0	19	448
GO:0198738 cell-cell signaling by wnt	19	0.00064	5.882	0.042	Biological	GO:0198	cell-cell signaling by wnt	0.00064	0.01824	0	0	19	450
GO:0002763 positive regulation of myeloid leukocyte differentiation	6	0.00064	1.858	0.103	Biological	GO:0002	positive regulation of myeloid leukocyte differentiation	0.00064	0.01824	0	0	6	58
GO:0097386 glial cell projection	5	0.00064	1.484	0.128	Cellular C	GO:0097	glial cell projection	0.00064	0.02592	0	0	5	39
GO:0043169 cation binding	108	0.00065	31.579	0.025	Molecular	GO:0043	cation binding	0.00065	0.09031	0	0	108	4406
GO:0061626 pharyngeal arch artery morphogenesis	3	0.00065	0.929	0.3	Biological	GO:0061	pharyngeal arch artery morphogenesis	0.00065	0.01848	0	0	3	10
GO:0086100 endothelin receptor signaling pathway	3	0.00065	0.929	0.3	Biological	GO:0086	endothelin receptor signaling pathway	0.00065	0.01848	0	0	3	10
GO:0019899 enzyme binding	59	0.00066	17.251	0.028	Molecular	GO:0019	enzyme binding	0.00066	0.09031	0	0	59	2092
GO:0003013 circulatory system process	23	0.00066	7.121	0.038	Biological	GO:0003	circulatory system process	0.00066	0.01865	0	0	23	599
GO:1902533 positive regulation of intracellular signal transduction	33	0.0007	10.217	0.033	Biological	GO:1902	positive regulation of intracellular signal transduction	0.0007	0.01961	0	0	33	998
GO:0032989 cellular component morphogenesis	28	0.00071	8.669	0.035	Biological	GO:0032	cellular component morphogenesis	0.00071	0.01964	0	0	28	796
GO:0090042 tubulin deacetylation	4	0.00073	1.238	0.174	Biological	GO:0090	tubulin deacetylation	0.00073	0.02014	0	0	4	23
GO:0051272 positive regulation of cellular component movement	22	0.00074	6.811	0.039	Biological	GO:0051	positive regulation of cellular component movement	0.00074	0.02029	0	0	22	566
GO:0023052 signaling	146	0.00077	45.201	0.023	Biological	GO:0023	signaling	0.00077	0.02106	0	0	146	6474
GO:0042472 inner ear morphogenesis	8	0.00081	2.477	0.074	Biological	GO:0042	inner ear morphogenesis	0.00081	0.02203	0	0	8	108
GO:0040017 positive regulation of locomotion	22	0.00083	6.811	0.039	Biological	GO:0040	positive regulation of locomotion	0.00083	0.02239	0	0	22	571
GO:0060322 head development	29	0.00084	8.978	0.034	Biological	GO:0060	head development	0.00084	0.02252	0	0	29	845
GO:0005883 neurofilament	3	0.00086	0.89	0.273	Cellular C	GO:0005	neurofilament	0.00086	0.02592	0	0	3	11
GO:0034992 microtubule organizing center attachment site	3	0.00086	0.89	0.273	Cellular C	GO:0034	microtubule organizing center attachment site	0.00086	0.02592	0	0	3	11
GO:0034993 meiotic nuclear membrane microtubule tethering complex	3	0.00086	0.89	0.273	Cellular C	GO:0034	meiotic nuclear membrane microtubule tethering complex	0.00086	0.02592	0	0	3	11
GO:0106083 nuclear membrane protein complex	3	0.00086	0.89	0.273	Cellular C	GO:0106	nuclear membrane protein complex	0.00086	0.02592	0	0	3	11
GO:0106094 nuclear membrane microtubule tethering complex	3	0.00086	0.89	0.273	Cellular C	GO:0106	nuclear membrane microtubule tethering complex	0.00086	0.02592	0	0	3	11
GO:0016020 membrane	205	0.00087	60.831	0.021	Cellular C	GO:0016	membrane	0.00087	0.02592	0	0	205	9774
GO:0030397 membrane disassembly	3	0.00089	0.929	0.273	Biological	GO:0030	membrane disassembly	0.00089	0.0236	0	0	3	11
GO:0060736 prostate gland growth	3	0.00089	0.929	0.273	Biological	GO:0060	prostate gland growth	0.00089	0.0236	0	0	3	11
GO:0014070 response to organic cyclic compound	31	0.00093	9.598	0.033	Biological	GO:0014	response to organic cyclic compound	0.00093	0.02453	0	0	31	932
GO:0045862 positive regulation of proteolysis	16	0.00096	4.954	0.045	Biological	GO:0045	positive regulation of proteolysis	0.00096	0.02521	0	0	16	359
GO:0030934 anchoring collagen complex	2	0.00096	0.593	0.667	Cellular C	GO:0030	anchoring collagen complex	0.00096	0.02704	0	0	2	3
GO:0045726 positive regulation of integrin biosynthetic process	2	0.00098	0.619	0.667	Biological	GO:0045	positive regulation of integrin biosynthetic process	0.00098	0.02558	0	0	2	3
GO:0009790 embryo development	36	0.00098	11.146	0.031	Biological	GO:0009	embryo development	0.00098	0.02558	0	0	36	1144
GO:0008083 growth factor activity	10	0.00099	2.924	0.061	Molecular	GO:0008	growth factor activity	0.00099	0.09031	0	0	10	164
GO:0030111 regulation of Wnt signaling pathway	15	0.001	4.644	0.046	Biological	GO:0030	regulation of Wnt signaling pathway	0.001	0.02583	0	0	15	326
GO:0002053 positive regulation of mesenchymal cell proliferation	4	0.00101	1.238	0.16	Biological	GO:0002	positive regulation of mesenchymal cell proliferation	0.00101	0.02608	0	0	4	25
GO:0018636 phenanthrene 9,10-monooxygenase activity	2	0.00102	0.585	0.667	Molecular	GO:0018	phenanthrene 9,10-monooxygenase activity	0.00102	0.09031	0	0	2	3
GO:0106235 ceramide-1-phosphate phosphatase activity	2	0.00102	0.585	0.667	Molecular	GO:0106	ceramide-1-phosphate phosphatase activity	0.00102	0.09031	0	0	2	3
GO:2000026 regulation of multicellular organismal development	42	0.00104	13.003	0.03	Biological	GO:2000	regulation of multicellular organismal development	0.00104	0.02668	0	0	42	1406
GO:0001936 regulation of endothelial cell proliferation	9	0.00108	2.786	0.064	Biological	GO:0001	regulation of endothelial cell proliferation	0.00108	0.02751	0	0	9	140
GO:0005102 signaling receptor binding	46	0.00109	13.45	0.03	Molecular	GO:0005	signaling receptor binding	0.00109	0.09031	0	0	46	1557
GO:0042981 regulation of apoptotic process	43	0.00112	13.313	0.03	Biological	GO:0042	regulation of apoptotic process	0.00112	0.02836	0	0	43	1455
GO:0065008 regulation of biological quality	95	0.00115	29.412	0.024	Biological	GO:0065	regulation of biological quality	0.00115	0.02892	0	0	95	3911
GO:0015175 neutral amino acid transmembrane transporter activity	5	0.00115	1.462	0.116	Molecular	GO:0015	neutral amino acid transmembrane transporter activity	0.00115	0.09031	0	0	5	43
GO:0010604 positive regulation of macromolecule metabolic process	87	0.00118	26.935	0.025	Biological	GO:0010	positive regulation of macromolecule metabolic process	0.00118	0.02972	0	0	87	3520
GO:0099604 ligand-gated calcium channel activity	4	0.00126	1.17	0.154	Molecular	GO:0099	ligand-gated calcium channel activity	0.00126	0.09062	0	0	4	26
GO:0065007 biological regulation	250	0.00126	77.399	0.02	Biological	GO:0065	biological regulation	0.00126	0.03153	0	0	250	12363
GO:0046530 photoreceptor cell differentiation	6	0.00127	1.858	0.091	Biological	GO:0046	photoreceptor cell differentiation	0.00127	0.03156	0	0	6	66
GO:0035272 exocrine system development	5	0.00131	1.548	0.111	Biological	GO:0035	exocrine system development	0.00131	0.03243	0	0	5	45
GO:0010941 regulation of cell death	47	0.00132	14.551	0.029	Biological	GO:0010	regulation of cell death	0.00132	0.03244	0	0	47	1645
GO:0033002 muscle cell proliferation	11	0.00134	3.406	0.054	Biological	GO:0033	muscle cell proliferation	0.00134	0.03279	0	0	11	204
GO:0045745 positive regulation of G protein-coupled receptor signaling	4	0.00137	1.238	0.148	Biological	GO:0045	positive regulation of G protein-coupled receptor signaling pathway	0.00137	0.03323	0	0	4	27
GO:0000904 cell morphogenesis involved in differentiation	26	0.00137	8.05	0.035	Biological	GO:0000	cell morphogenesis involved in differentiation	0.00137	0.03323	0	0	26	751
GO:0007389 pattern specification process	19	0.00139	5.882	0.04	Biological	GO:0007	pattern specification process	0.00139	0.03362	0	0	19	481
GO:0048583 regulation of response to stimulus	97	0.00144	30.031	0.024	Biological	GO:0048	regulation of response to stimulus	0.00144	0.03444	0	0	97	4038
GO:0007179 transforming growth factor beta receptor signaling pathwa	11	0.00145	3.406	0.053	Biological	GO:0007	transforming growth factor beta receptor signaling pathway	0.00145	0.03444	0	0	11	206
GO:0045216 cell-cell junction organization	11	0.00145	3.406	0.053	Biological	GO:0045	cell-cell junction organization	0.00145	0.03444	0	0	11	206
GO:0005581 collagen trimer	7	0.00146	2.077	0.075	Cellular C	GO:0005	collagen trimer	0.00146	0.03797	0	0	7	93
GO:0032835 glomerulus development	6	0.00148	1.858	0.088	Biological	GO:0032	glomerulus development	0.00148	0.03513	0	0	6	68
GO:0042415 norepinephrine metabolic process	3	0.0015	0.929	0.231	Biological	GO:0042	norepinephrine metabolic process	0.0015	0.03531	0	0	3	13
GO:0010952 positive regulation of peptidase activity	10	0.00155	3.096	0.056	Biological	GO:0010	positive regulation of peptidase activity	0.00155	0.03624	0	0	10	177
GO:0007417 central nervous system development	34	0.00155	10.526	0.031	Biological	GO:0007	central nervous system development	0.00155	0.03624	0	0	34	1089
GO:0001223 transcription coactivator binding	5	0.00156	1.462	0.109	Molecular	GO:0001	transcription coactivator binding	0.00156	0.104	0	0	5	46
GO:0072012 glomerulus vasculature development	4	0.00157	1.238	0.143	Biological	GO:0072	glomerulus vasculature development	0.00157	0.03649	0	0	4	28
GO:0031256 leading edge membrane	10	0.00162	2.967	0.056	Cellular C	GO:0031	leading edge membrane	0.00162	0.03797	0	0	10	180
GO:0007178 transmembrane receptor protein serine/threonine kinase s	16	0.00163	4.954	0.042	Biological	GO:0007	transmembrane receptor protein serine/threonine kinase signaling pa	0.00163	0.03779	0	0	16	378
GO:0043209 myelin sheath	5	0.00168	1.484	0.104	Cellular C	GO:0043	myelin sheath	0.00168	0.03797	0	0	5	48
GO:0120025 plasma membrane bounded cell projection	60	0.00169	17.804	0.026	Cellular C	GO:0120	plasma membrane bounded cell projection	0.00169	0.03797	0	0	60	2285
GO:0005788 endoplasmic reticulum lumen	14	0.00172	4.154	0.045	Cellular C	GO:0005	endoplasmic reticulum lumen	0.00172	0.03797	0	0	14	313
GO:0043067 regulation of programmed cell death	43	0.00173	13.313	0.029	Biological	GO:0043	regulation of programmed cell death	0.00173	0.03975	0	0	43	1488
GO:0060560 developmental growth involved in morphogenesis	12	0.00174	3.715	0.049	Biological	GO:0060	developmental growth involved in morphogenesis	0.00174	0.03989	0	0	12	243
GO:0045668 negative regulation of osteoblast differentiation	5	0.00176	1.548	0.104	Biological	GO:0045	negative regulation of osteoblast differentiation	0.00176	0.03992	0	0	5	48
GO:0048483 autonomic nervous system development	5	0.00176	1.548	0.104	Biological	GO:0048	autonomic nervous system development	0.00176	0.03992	0	0	5	48
GO:0060037 pharyngeal system development	4	0.0018	1.238	0.138	Biological	GO:0060	pharyngeal system development	0.0018	0.0406	0	0	4	29
GO:0030673 axolemma	3	0.00182	0.89	0.214	Cellular C	GO:0030	axolemma	0.00182	0.03797	0	0	3	14
GO:0048844 artery morphogenesis	6	0.00186	1.858	0.085	Biological	GO:0048	artery morphogenesis	0.00186	0.0407	0	0	6	71
GO:0005783 endoplasmic reticulum	55	0.00186	16.32	0.027	Cellular C	GO:0005	endoplasmic reticulum	0.00186	0.03797	0	0	55	2059
GO:0043588 skin development	14	0.0019	4.334	0.045	Biological	GO:0043	skin development	0.0019	0.0407	0	0	14	313
GO:1901701 cellular response to oxygen-containing compound	36	0.00192	11.146	0.03	Biological	GO:1901	cellular response to oxygen-containing compound	0.00192	0.0407	0	0	36	1189
GO:0042692 muscle cell differentiation	16	0.00192	4.954	0.042	Biological	GO:0042	muscle cell differentiation	0.00192	0.0407	0	0	16	384
GO:0070161 anchoring junction	29	0.00193	8.605	0.032	Cellular C	GO:0070	anchoring junction	0.00193	0.03797	0	0	29	902
GO:0035850 epithelial cell differentiation involved in kidney development	5	0.00193	1.548	0.102	Biological	GO:0035	epithelial cell differentiation involved in kidney development	0.00193	0.0407	0	0	5	49
GO:0002159 desmosome assembly	2	0.00194	0.619	0.5	Biological	GO:0002	desmosome assembly	0.00194	0.0407	0	0	2	4
GO:0007380 specification of segmental identity, head	2	0.00194	0.619	0.5	Biological	GO:0007	specification of segmental identity, head	0.00194	0.0407	0	0	2	4
GO:0009753 response to jasmonic acid	2	0.00194	0.619	0.5	Biological	GO:0009	response to jasmonic acid	0.00194	0.0407	0	0	2	4
GO:0035287 head segmentation	2	0.00194	0.619	0.5	Biological	GO:0035	head segmentation	0.00194	0.0407	0	0	2	4
GO:0035289 posterior head segmentation	2	0.00194	0.619	0.5	Biological	GO:0035	posterior head segmentation	0.00194	0.0407	0	0	2	4
GO:0071395 cellular response to jasmonic acid stimulus	2	0.00194	0.619	0.5	Biological	GO:0071	cellular response to jasmonic acid stimulus	0.00194	0.0407	0	0	2	4
GO:0071798 response to prostaglandin D	2	0.00194	0.619	0.5	Biological	GO:0071	response to prostaglandin D	0.00194	0.0407	0	0	2	4
GO:0071799 cellular response to prostaglandin D stimulus	2	0.00194	0.619	0.5	Biological	GO:0071	cellular response to prostaglandin D stimulus	0.00194	0.0407	0	0	2	4
GO:0072191 ureter smooth muscle development	2	0.00194	0.619	0.5	Biological	GO:0072	ureter smooth muscle development	0.00194	0.0407	0	0	2	4
GO:0072193 ureter smooth muscle cell differentiation	2	0.00194	0.619	0.5	Biological	GO:0072	ureter smooth muscle cell differentiation	0.00194	0.0407	0	0	2	4
GO:0097018 renal albumin absorption	2	0.00194	0.619	0.5	Biological	GO:0097	renal albumin absorption	0.00194	0.0407	0	0	2	4
GO:0106071 positive regulation of adenylate cyclase-activating G protei	2	0.00194	0.619	0.5	Biological	GO:0106	positive regulation of adenylate cyclase-activating G protein-coupled	0.00194	0.0407	0	0	2	4
GO:0040007 growth	30	0.00195	9.288	0.032	Biological	GO:0040	growth	0.00195	0.0407	0	0	30	935
GO:0008270 zinc ion binding	28	0.00197	8.187	0.033	Molecular	GO:0008	zinc ion binding	0.00197	0.11576	0	0	28	838
GO:0098590 plasma membrane region	38	0.002	11.276	0.029	Cellular C	GO:0098	plasma membrane region	0.002	0.03797	0	0	38	1292
GO:0004687 myosin light chain kinase activity	2	0.00201	0.585	0.5	Molecular	GO:0004	myosin light chain kinase activity	0.00201	0.11576	0	0	2	4
GO:0051347 positive regulation of transferase activity	20	0.00202	6.192	0.037	Biological	GO:0051	positive regulation of transferase activity	0.00202	0.04166	0	0	20	535
GO:0014706 striated muscle tissue development	16	0.00202	4.954	0.041	Biological	GO:0014	striated muscle tissue development	0.00202	0.04166	0	0	16	386
GO:0051146 striated muscle cell differentiation	13	0.00203	4.025	0.046	Biological	GO:0051	striated muscle cell differentiation	0.00203	0.04166	0	0	13	281
GO:0061437 renal system vasculature development	4	0.00204	1.238	0.133	Biological	GO:0061	renal system vasculature development	0.00204	0.04166	0	0	4	30
GO:0061440 kidney vasculature development	4	0.00204	1.238	0.133	Biological	GO:0061	kidney vasculature development	0.00204	0.04166	0	0	4	30
GO:0007409 axonogenesis	18	0.00205	5.573	0.039	Biological	GO:0007	axonogenesis	0.00205	0.04166	0	0	18	460
GO:0050789 regulation of biological process	238	0.00205	73.684	0.02	Biological	GO:0050	regulation of biological process	0.00205	0.04166	0	0	238	11725
GO:0042995 cell projection	62	0.00206	18.398	0.026	Cellular C	GO:0042	cell projection	0.00206	0.03797	0	0	62	2399
GO:1902531 regulation of intracellular signal transduction	48	0.00206	14.861	0.028	Biological	GO:1902	regulation of intracellular signal transduction	0.00206	0.04166	0	0	48	1727
GO:0002040 sprouting angiogenesis	8	0.00208	2.477	0.064	Biological	GO:0002	sprouting angiogenesis	0.00208	0.04188	0	0	8	125
GO:0001754 eye photoreceptor cell differentiation	5	0.00211	1.548	0.1	Biological	GO:0001	eye photoreceptor cell differentiation	0.00211	0.04237	0	0	5	50
GO:0060828 regulation of canonical Wnt signaling pathway	12	0.00213	3.715	0.048	Biological	GO:0060	regulation of canonical Wnt signaling pathway	0.00213	0.04262	0	0	12	249
GO:0051716 cellular response to stimulus	162	0.00216	50.155	0.022	Biological	GO:0051	cellular response to stimulus	0.00216	0.043	0	0	162	7475
GO:0008092 cytoskeletal protein binding	32	0.00216	9.357	0.032	Molecular	GO:0008	cytoskeletal protein binding	0.00216	0.11704	0	0	32	1008
GO:0043542 endothelial cell migration	11	0.00218	3.406	0.051	Biological	GO:0043	endothelial cell migration	0.00218	0.04327	0	0	11	217
GO:0050890 cognition	14	0.00226	4.334	0.044	Biological	GO:0050	cognition	0.00226	0.04459	0	0	14	319
GO:0035148 tube formation	9	0.00228	2.786	0.058	Biological	GO:0035	tube formation	0.00228	0.04491	0	0	9	156
GO:0070293 renal absorption	4	0.00231	1.238	0.129	Biological	GO:0070	renal absorption	0.00231	0.045	0	0	4	31
GO:0042063 gliogenesis	14	0.00232	4.334	0.044	Biological	GO:0042	gliogenesis	0.00232	0.045	0	0	14	320
GO:0048736 appendage development	10	0.00233	3.096	0.053	Biological	GO:0048	appendage development	0.00233	0.045	0	0	10	187

GO:0060173 limb development	10	0.00233	3.096	0.053	Biological	GO:00601	limb development	0.00233	0.045	0	0	10	187
GO:0009612 response to mechanical stimulus	11	0.00235	3.406	0.05	Biological	GO:00096	response to mechanical stimulus	0.00235	0.045	0	0	11	219
GO:0045639 positive regulation of myeloid cell differentiation	7	0.00236	2.167	0.07	Biological	GO:00456	positive regulation of myeloid cell differentiation	0.00236	0.045	0	0	7	100
GO:0060840 artery development	7	0.00236	2.167	0.07	Biological	GO:00608	artery development	0.00236	0.045	0	0	7	100
GO:0060993 kidney morphogenesis	7	0.00236	2.167	0.07	Biological	GO:00609	kidney morphogenesis	0.00236	0.045	0	0	7	100
GO:0043005 neuron projection	40	0.00237	11.869	0.029	Cellular C	GO:00430	neuron projection	0.00237	0.04166	0	0	40	1394
GO:0016331 morphogenesis of embryonic epithelium	9	0.00239	2.786	0.057	Biological	GO:00163	morphogenesis of embryonic epithelium	0.00239	0.04529	0	0	9	157
GO:0099512 supramolecular fiber	32	0.00241	9.496	0.031	Cellular C	GO:00995	supramolecular fiber	0.00241	0.04166	0	0	32	1044
GO:0031624 ubiquitin conjugating enzyme binding	4	0.00246	1.17	0.129	Molecular	GO:00316	ubiquitin conjugating enzyme binding	0.00246	0.12518	0	0	4	31
GO:0001935 endothelial cell proliferation	9	0.0026	2.786	0.057	Biological	GO:00019	endothelial cell proliferation	0.0026	0.04892	0	0	9	159
GO:0003338 metanephros morphogenesis	4	0.00261	1.238	0.125	Biological	GO:00033	metanephros morphogenesis	0.00261	0.04892	0	0	4	32
GO:0010464 regulation of mesenchymal cell proliferation	4	0.00261	1.238	0.125	Biological	GO:00104	regulation of mesenchymal cell proliferation	0.00261	0.04892	0	0	4	32
GO:0001838 embryonic epithelial tube formation	8	0.00266	2.477	0.062	Biological	GO:00018	embryonic epithelial tube formation	0.00266	0.04969	0	0	8	130
GO:0099081 supramolecular polymer	32	0.00275	9.496	0.03	Cellular C	GO:00990	supramolecular polymer	0.00275	0.04603	0	0	32	1053
GO:0072109 glomerular mesangium development	3	0.00282	0.929	0.188	Biological	GO:00721	glomerular mesangium development	0.00282	0.05252	0	0	3	16
GO:0006935 chemotaxis	22	0.00285	6.811	0.035	Biological	GO:00069	chemotaxis	0.00285	0.05283	0	0	22	630
GO:0012505 endomembrane system	109	0.00285	32.344	0.023	Cellular C	GO:00125	endomembrane system	0.00285	0.04619	0	0	109	4776
GO:0051015 actin filament binding	11	0.00286	3.216	0.05	Molecular	GO:00510	actin filament binding	0.00286	0.13324	0	0	11	221
GO:0048858 cell projection morphogenesis	23	0.0029	7.121	0.034	Biological	GO:00488	cell projection morphogenesis	0.0029	0.05364	0	0	23	671
GO:0007431 salivary gland development	4	0.00292	1.238	0.121	Biological	GO:00074	salivary gland development	0.00292	0.05387	0	0	4	33
GO:0004698 calcium-dependent protein kinase C activity	3	0.00295	0.877	0.188	Molecular	GO:00046	calcium-dependent protein kinase C activity	0.00295	0.13324	0	0	3	16
GO:0009893 positive regulation of metabolic process	91	0.00297	28.173	0.024	Biological	GO:00098	positive regulation of metabolic process	0.00297	0.05451	0	0	91	3828
GO:0042327 positive regulation of phosphorylation	26	0.00299	8.05	0.033	Biological	GO:00423	positive regulation of phosphorylation	0.00299	0.05471	0	0	26	795
GO:0048469 cell maturation	10	0.00303	3.096	0.052	Biological	GO:00484	cell maturation	0.00303	0.05516	0	0	10	194
GO:0003007 heart morphogenesis	12	0.00304	3.715	0.046	Biological	GO:00030	heart morphogenesis	0.00304	0.05516	0	0	12	260
GO:0032270 positive regulation of cellular protein metabolic process	39	0.00305	12.074	0.029	Biological	GO:00322	positive regulation of cellular protein metabolic process	0.00305	0.05516	0	0	39	1355
GO:0003002 regionalization	15	0.00307	4.644	0.041	Biological	GO:00030	regionalization	0.00307	0.05535	0	0	15	366
GO:0042330 taxis	22	0.00313	6.811	0.035	Biological	GO:00423	taxis	0.00313	0.05598	0	0	22	635
GO:0033674 positive regulation of kinase activity	17	0.00315	5.263	0.039	Biological	GO:00336	positive regulation of kinase activity	0.00315	0.05598	0	0	17	441
GO:0120035 regulation of plasma membrane bounded cell projection or	22	0.00319	6.811	0.035	Biological	GO:01200	regulation of plasma membrane bounded cell projection organization	0.00319	0.05598	0	0	22	636
GO:0003151 outflow tract morphogenesis	6	0.00319	1.858	0.076	Biological	GO:00031	outflow tract morphogenesis	0.00319	0.05598	0	0	6	79
GO:0034343 type III interferon production	2	0.00319	0.619	0.4	Biological	GO:00343	type III interferon production	0.00319	0.05598	0	0	2	5
GO:0034344 regulation of type III interferon production	2	0.00319	0.619	0.4	Biological	GO:00343	regulation of type III interferon production	0.00319	0.05598	0	0	2	5
GO:0045113 regulation of integrin biosynthetic process	2	0.00319	0.619	0.4	Biological	GO:00451	regulation of integrin biosynthetic process	0.00319	0.05598	0	0	2	5
GO:0071504 cellular response to heparin	2	0.00319	0.619	0.4	Biological	GO:00715	cellular response to heparin	0.00319	0.05598	0	0	2	5
GO:0030175 filopodium	7	0.00325	2.077	0.065	Cellular C	GO:00301	filopodium	0.00325	0.05116	0	0	7	107
GO:0047086 ketosteroid monooxygenase activity	2	0.0033	0.585	0.4	Molecular	GO:00470	ketosteroid monooxygenase activity	0.0033	0.13324	0	0	2	5
GO:0072345 NAADP-sensitive calcium-release channel activity	2	0.0033	0.585	0.4	Molecular	GO:00723	NAADP-sensitive calcium-release channel activity	0.0033	0.13324	0	0	2	5
GO:0042592 homeostatic process	50	0.00331	15.48	0.027	Biological	GO:00425	homeostatic process	0.00331	0.0577	0	0	50	1861
GO:0045766 positive regulation of angiogenesis	9	0.00333	2.786	0.055	Biological	GO:00457	positive regulation of angiogenesis	0.00333	0.0577	0	0	9	165
GO:1904018 positive regulation of vasculature development	9	0.00333	2.786	0.055	Biological	GO:19040	positive regulation of vasculature development	0.00333	0.0577	0	0	9	165
GO:0042448 progesterone metabolic process	3	0.00338	0.929	0.176	Biological	GO:00424	progesterone metabolic process	0.00338	0.05834	0	0	3	17
GO:0070997 neuron death	15	0.0034	4.644	0.041	Biological	GO:00709	neuron death	0.0034	0.05851	0	0	15	370
GO:0001954 positive regulation of cell-matrix adhesion	5	0.00349	1.548	0.089	Biological	GO:00019	positive regulation of cell-matrix adhesion	0.00349	0.05982	0	0	5	56
GO:0004697 protein kinase C activity	3	0.00354	0.877	0.176	Molecular	GO:00046	protein kinase C activity	0.00354	0.13324	0	0	3	17
GO:0015278 calcium-release channel activity	3	0.00354	0.877	0.176	Molecular	GO:00152	calcium-release channel activity	0.00354	0.13324	0	0	3	17
GO:0003018 vascular process in circulatory system	12	0.00354	3.715	0.045	Biological	GO:00030	vascular process in circulatory system	0.00354	0.06062	0	0	12	265
GO:0051247 positive regulation of protein metabolic process	41	0.00356	12.693	0.028	Biological	GO:00512	positive regulation of protein metabolic process	0.00356	0.06073	0	0	41	1457
GO:0010594 regulation of endothelial cell migration	9	0.0036	2.786	0.054	Biological	GO:00105	regulation of endothelial cell migration	0.0036	0.06092	0	0	9	167
GO:0010950 positive regulation of endopeptidase activity	9	0.0036	2.786	0.054	Biological	GO:00109	positive regulation of endopeptidase activity	0.0036	0.06092	0	0	9	167
GO:0035418 protein localization to synapse	6	0.00362	1.858	0.074	Biological	GO:00354	protein localization to synapse	0.00362	0.06092	0	0	6	81
GO:0050794 regulation of cellular process	228	0.00363	70.588	0.02	Biological	GO:00507	regulation of cellular process	0.00363	0.06092	0	0	228	11233
GO:0048048 embryonic eye morphogenesis	4	0.00364	1.238	0.114	Biological	GO:00480	embryonic eye morphogenesis	0.00364	0.06092	0	0	4	35
GO:0008406 gonad development	11	0.00365	3.406	0.047	Biological	GO:00084	gonad development	0.00365	0.06092	0	0	11	232
GO:0008544 epidermis development	15	0.00366	4.644	0.04	Biological	GO:00085	epidermis development	0.00366	0.06096	0	0	15	373
GO:0021915 neural tube development	9	0.00375	2.786	0.054	Biological	GO:00219	neural tube development	0.00375	0.06189	0	0	9	168
GO:0006584 catecholamine metabolic process	5	0.00376	1.548	0.088	Biological	GO:00065	catecholamine metabolic process	0.00376	0.06189	0	0	5	57
GO:0009712 catechol-containing compound metabolic process	5	0.00376	1.548	0.088	Biological	GO:00097	catechol-containing compound metabolic process	0.00376	0.06189	0	0	5	57
GO:0043583 ear development	11	0.00377	3.406	0.047	Biological	GO:00435	ear development	0.00377	0.06189	0	0	11	233
GO:0007173 epidermal growth factor receptor signaling pathway	7	0.00383	2.167	0.064	Biological	GO:00071	epidermal growth factor receptor signaling pathway	0.00383	0.06266	0	0	7	109
GO:0043177 organic acid binding	8	0.00388	2.339	0.059	Molecular	GO:00431	organic acid binding	0.00388	0.14001	0	0	8	136
GO:0030027 lamellipodium	10	0.00388	2.967	0.049	Cellular C	GO:00300	lamellipodium	0.00388	0.05931	0	0	10	203
GO:0030178 negative regulation of Wnt signaling pathway	9	0.0039	2.786	0.053	Biological	GO:00301	negative regulation of Wnt signaling pathway	0.0039	0.06361	0	0	9	169
GO:0010744 positive regulation of macrophage derived foam cell differe	3	0.004	0.929	0.167	Biological	GO:00107	positive regulation of macrophage derived foam cell differentiation	0.004	0.06454	0	0	3	18
GO:0072077 renal vesicle morphogenesis	3	0.004	0.929	0.167	Biological	GO:00720	renal vesicle morphogenesis	0.004	0.06454	0	0	3	18
GO:0010573 vascular endothelial growth factor production	4	0.00403	1.238	0.111	Biological	GO:00105	vascular endothelial growth factor production	0.00403	0.06454	0	0	4	36
GO:0010742 macrophage derived foam cell differentiation	4	0.00403	1.238	0.111	Biological	GO:00107	macrophage derived foam cell differentiation	0.00403	0.06454	0	0	4	36
GO:0090077 foam cell differentiation	4	0.00403	1.238	0.111	Biological	GO:00900	foam cell differentiation	0.00403	0.06454	0	0	4	36
GO:0110110 positive regulation of animal organ morphogenesis	4	0.00403	1.238	0.111	Biological	GO:01101	positive regulation of animal organ morphogenesis	0.00403	0.06454	0	0	4	36
GO:0031344 regulation of cell projection organization	22	0.00412	6.811	0.034	Biological	GO:00313	regulation of cell projection organization	0.00412	0.06558	0	0	22	650
GO:0048812 neuron projection morphogenesis	22	0.00412	6.811	0.034	Biological	GO:00488	neuron projection morphogenesis	0.00412	0.06558	0	0	22	650
GO:0032990 cell part morphogenesis	23	0.00415	7.121	0.033	Biological	GO:00329	cell part morphogenesis	0.00415	0.06578	0	0	23	691
GO:0044057 regulation of system process	20	0.00421	6.192	0.035	Biological	GO:00440	regulation of system process	0.00421	0.06659	0	0	20	571
GO:0048589 developmental growth	22	0.00427	6.811	0.034	Biological	GO:00485	developmental growth	0.00427	0.06722	0	0	22	652
GO:0045137 development of primary sexual characteristics	11	0.00428	3.406	0.046	Biological	GO:00451	development of primary sexual characteristics	0.00428	0.06722	0	0	11	237
GO:0048660 regulation of smooth muscle cell proliferation	8	0.00436	2.477	0.057	Biological	GO:00486	regulation of smooth muscle cell proliferation	0.00436	0.06779	0	0	8	141
GO:0015804 neutral amino acid transport	5	0.00437	1.548	0.085	Biological	GO:00158	neutral amino acid transport	0.00437	0.06779	0	0	5	59
GO:0035904 aorta development	5	0.00437	1.548	0.085	Biological	GO:00359	aorta development	0.00437	0.06779	0	0	5	59
GO:0061005 cell differentiation involved in kidney development	5	0.00437	1.548	0.085	Biological	GO:00610	cell differentiation involved in kidney development	0.00437	0.06779	0	0	5	59
GO:0048839 inner ear development	10	0.00448	3.096	0.049	Biological	GO:00488	inner ear development	0.00448	0.06933	0	0	10	205
GO:0048708 astrocyte differentiation	6	0.00459	1.858	0.071	Biological	GO:00487	astrocyte differentiation	0.00459	0.06974	0	0	6	85
GO:0030314 junctional membrane complex	2	0.00463	0.593	0.333	Cellular C	GO:00303	junctional membrane complex	0.00463	0.06883	0	0	2	6
GO:0008016 regulation of heart contraction	10	0.00464	3.096	0.049	Biological	GO:00080	regulation of heart contraction	0.00464	0.06974	0	0	10	206
GO:0051235 maintenance of location	14	0.00466	4.334	0.04	Biological	GO:00512	maintenance of location	0.00466	0.06974	0	0	14	346
GO:0043473 pigmentation	7	0.00467	2.167	0.062	Biological	GO:00434	pigmentation	0.00467	0.06974	0	0	7	113
GO:0071625 vocalization behavior	3	0.00468	0.929	0.158	Biological	GO:00716	vocalization behavior	0.00468	0.06974	0	0	3	19
GO:0072087 renal vesicle development	3	0.00468	0.929	0.158	Biological	GO:00720	renal vesicle development	0.00468	0.06974	0	0	3	19
GO:0008237 metallopeptidase activity	10	0.00471	2.924	0.049	Molecular	GO:00082	metallopeptidase activity	0.00471	0.15397	0	0	10	203
GO:0044390 ubiquitin-like protein conjugating enzyme binding	4	0.00473	1.17	0.108	Molecular	GO:00443	ubiquitin-like protein conjugating enzyme binding	0.00473	0.15397	0	0	4	37
GO:0009597 detection of virus	2	0.00473	0.619	0.333	Biological	GO:00095	detection of virus	0.00473	0.06974	0	0	2	6
GO:0014034 neural crest cell fate commitment	2	0.00473	0.619	0.333	Biological	GO:00140	neural crest cell fate commitment	0.00473	0.06974	0	0	2	6
GO:0045112 integrin biosynthetic process	2	0.00473	0.619	0.333	Biological	GO:00451	integrin biosynthetic process	0.00473	0.06974	0	0	2	6
GO:0051005 negative regulation of lipoprotein lipase activity	2	0.00473	0.619	0.333	Biological	GO:00510	negative regulation of lipoprotein lipase activity	0.00473	0.06974	0	0	2	6
GO:0051890 regulation of cardioblast differentiation	2	0.00473	0.619	0.333	Biological	GO:00518	regulation of cardioblast differentiation	0.00473	0.06974	0	0	2	6
GO:0061343 cell adhesion involved in heart morphogenesis	2	0.00473	0.619	0.333	Biological	GO:00613	cell adhesion involved in heart morphogenesis	0.00473	0.06974	0	0	2	6
GO:1905050 positive regulation of metallopeptidase activity	2	0.00473	0.619	0.333	Biological	GO:19050	positive regulation of metallopeptidase activity	0.00473	0.06974	0	0	2	6
GO:0035296 regulation of tube diameter	8	0.00475	2.477	0.056	Biological	GO:00352	regulation of tube diameter	0.00475	0.06974	0	0	8	143
GO:0072175 epithelial tube formation	8	0.00475	2.477	0.056	Biological	GO:00721	epithelial tube formation	0.00475	0.06974	0	0	8	143
GO:0097746 blood vessel diameter maintenance	8	0.00475	2.477	0.056	Biological	GO:00977	blood vessel diameter maintenance	0.00475	0.06974	0	0	8	143
GO:2001056 positive regulation of cysteine-type endopeptidase activity	8	0.00475	2.477	0.056	Biological	GO:20010	positive regulation of cysteine-type endopeptidase activity	0.00475	0.06974	0	0	8	143
GO:0031253 cell projection membrane	14	0.00481	4.154	0.04	Cellular C	GO:00312	cell projection membrane	0.00481	0.06911	0	0	14	351
GO:0000979 RNA polymerase II core promoter sequence-specific DNA	3	0.00491	0.877	0.158	Molecular	GO:00009	RNA polymerase II core promoter sequence-specific DNA binding	0.00491	0.15397	0	0	3	19
GO:1901698 response to nitrogen compound	33	0.00491	10.217	0.029	Biological	GO:19016	response to nitrogen compound	0.00491	0.07129	0	0	33	1127
GO:0006730 one-carbon metabolic process	4	0.00491	1.238	0.105	Biological	GO:00067	one-carbon metabolic process	0.00491	0.07129	0	0	4	38
GO:0008207 C21-steroid hormone metabolic process	4	0.00491	1.238	0.105	Biological	GO:00082	C21-steroid hormone metabolic process	0.00491	0.07129	0	0	4	38
GO:0071711 basement membrane organization	4	0.00491	1.238	0.105	Biological	GO:00717	basement membrane organization	0.00491	0.07129	0	0	4	38
GO:0035150 regulation of tube size	8	0.00495	2.477	0.056	Biological	GO:00351	regulation of tube size	0.00495	0.07165	0	0	8	144
GO:0098588 bounding membrane of organelle	55	0.00495	16.32	0.026	Cellular C	GO:00985	bounding membrane of organelle	0.00495	0.06911	0	0	55	2156
GO:0007611 learning or memory	12	0.00503	3.715	0.043	Biological	GO:00076	learning or memory	0.00503	0.07258	0	0	12	277
GO:0031674 I band	8	0.00504	2.374	0.055	Cellular C	GO:00316	I band	0.00504	0.06911	0	0	8	146
GO:0022617 extracellular matrix disassembly	5	0.00504	1.548	0.082	Biological	GO:00226	extracellular matrix disassembly	0.00504	0.07258	0	0	5	61
GO:0048659 smooth muscle cell proliferation	8	0.00516	2.477	0.055	Biological	GO:00486	smooth muscle cell proliferation	0.00516	0.07402	0	0	8	145
GO:0010562 positive regulation of phosphorus metabolic process	27	0.00521	8.359	0.031	Biological	GO:00105	positive regulation of phosphorus metabolic process	0.00521	0.07441	0	0	27	872
GO:0045937 positive regulation of phosphate metabolic process	27	0.00521	8.359	0.031	Biological	GO:00459	positive regulation of phosphate metabolic process	0.00521	0.07441	0	0	27	872
GO:0005201 extracellular matrix structural constituent	9	0.00525	2.632	0.052	Molecular	GO:00052	extracellular matrix structural constituent	0.00525	0.15397	0	0	9	174
GO:0007517 muscle organ development	14	0.00528	4.334	0.04	Biological	GO:00075	muscle organ development	0.00528	0.07508	0	0	14	351
GO:0043292 contractile fiber	11	0.00537	3.264	0.045	Cellular C	GO:00432	contractile fiber	0.00537	0.07094	0	0	11	247
GO:0008584 male gonad development	8	0.00537	2.477	0.055	Biological	GO:00085	male gonad development	0.00537	0.07623	0	0	8	146
GO:0043618 regulation of transcription from RNA polymerase II promot	4	0.00539	1.238	0.103	Biological	GO:00436	regulation of transcription from RNA polymerase II promoter in respo	0.00539	0.07626	0	0	4	39
GO:1904705 regulation of vascular associated smooth muscle cell prolif	5	0.00541	1.548	0.081	Biological	GO:19047	regulation of vascular associated smooth muscle cell proliferation	0.00541	0.07626	0	0	5	62
GO:0016757 glycosyltransferase activity	12	0.00543	3.509	0.044	Molecular	GO:00167	glycosyltransferase activity	0.00543	0.15397	0	0	12	275
GO:0001764 neuron migration	9	0.00546	2.786	0.051	Biological	GO:00017	neuron migration	0.00546	0.07664	0	0	9	178
GO:0120039 plasma membrane bounded cell projection morphogenesis	22	0.00546	6.811	0.033	Biological	GO:01200	plasma membrane bounded cell projection morphogenesis	0.00546	0.07664	0	0	22	666
GO:0044304 main axon	5	0.00553	1.484	0.079	Cellular C	GO:00443	main axon	0.00553	0.07094	0	0	5	63
GO:0045202 synapse	40	0.00557	11.869	0.027	Cellular C	GO:00452	synapse	0.00557	0.07094	0	0	40	1465
GO:0046546 development of primary male sexual characteristics	8	0.0056	2.477	0.054	Biological	GO:00465	development of primary male sexual characteristics	0.0056	0.07827	0	0	8	147
GO:0001221 transcription coregulator binding	7	0.00562	2.047	0.061	Molecular	GO:00012	transcription coregulator binding	0.00562	0.15397	0	0	7	115
GO:0048863 stem cell differentiation	11	0.00564	3.406	0.045	Biological	GO:00488	stem cell differentiation	0.00564	0.07866	0	0	11	246
GO:0043065 positive regulation of apoptotic process	18	0.00566	5.573	0.036	Biological	GO:00430	positive regulation of apoptotic process	0.00566	0.07867	0	0	18	507
GO:0001972 retinoic acid binding	3	0.00569	0.877	0.15	Molecular	GO:00019	retinoic acid binding	0.00569	0.15397	0	0	3	20
GO:0005520 insulin-like growth factor binding	3	0.00569	0.877	0.15	Molecular	GO:00055	insulin-like growth factor binding	0.00569	0.15397	0	0	3	20
GO:0033627 cell adhesion mediated by integrin	6	0.00574	1.858	0.067	Biological	GO:00336	cell adhesion mediated by integrin	0.00574	0.07961	0	0	6	89
GO:0031325 positive regulation of cellular metabolic process	79	0.00576	24.458	0.024	Biological	GO:00313	positive regulation of cellular metabolic process	0.00576	0.07975	0	0	79	3316
GO:0070542 response to fatty acid	5	0.00579	1.548	0.079	Biological	GO:00705	response to fatty acid	0.00579	0.07982	0	0	5	63
GO:0030055 cell-substrate junction	16	0.00579	4.748	0.037	Cellular C	GO:00300	cell-substrate junction	0.00579	0.07141	0	0	16	435
GO:0005912 adherens junction	9	0.00587	2.671	0.049	Cellular C	GO:00059	adherens junction	0.00587	0.07141	0	0	9	182
GO:0048667 cell morphogenesis involved in neuron differentiation	20	0.00601	6.192	0.034	Biological	GO:00486	cell morphogenesis involved in neuron differentiation	0.00601	0.08267	0	0	20	590
GO:0010634 positive regulation of epithelial cell migration	8	0.00606	2.477	0.054	Biological	GO:00106	positive regulation of epithelial cell migration	0.00606	0.08291	0	0	8	149
GO:0072073 kidney epithelium development	8	0.00606	2.477	0.054	Biological	GO:00720	kidney epithelium development	0.00606	0.08291	0	0	8	149
GO:0043083 synaptic cleft	3	0.00607	0.89	0.143	Cellular C	GO:00430	synaptic cleft	0.00607	0.07214	0	0	3	21
GO:0050731 positive regulation of peptidyl-tyrosine phosphorylation	9	0.00608	2.786	0.05	Biological	GO:00507	positive regulation of peptidyl-tyrosine phosphorylation	0.00608	0.08291	0	0	9	181
GO:0048018 receptor ligand activity	18	0.00608	5.263	0.036	Molecular	GO:00480	receptor ligand activity	0.00608	0.15397	0	0	18	502
GO:0007411 axon guidance	11	0.00616	3.406	0.044	Biological	GO:00074	axon guidance	0.00616	0.0837	0	0	11	249
GO:0072009 nephron epithelium development	7	0.00617	2.167	0.059	Biological	GO:00720	nephron epithelium development	0.00617	0.0837	0	0	7	119
GO:1990874 vascular associated smooth muscle cell proliferation	5	0.00618	1.548	0.078	Biological	GO:19908	vascular associated smooth muscle cell proliferation	0.00618	0.0837	0	0	5	64
GO:0060749 mammary gland alveolus development	3	0.00626	0.929	0.143	Biological	GO:00607	mammary gland alveolus development	0.00626	0.08401	0	0	3	21
GO:0061377 mammary gland lobule development	3	0.00626	0.929	0.143	Biological	GO:00613	mammary gland lobule development	0.00626	0.08401	0	0	3	21
GO:0072111 cell proliferation involved in kidney development	3	0.00626	0.929	0.143	Biological	GO:00721	cell proliferation involved in kidney development	0.00626	0.08401	0	0	3	21
GO:0098742 cell-cell adhesion via plasma-membrane adhesion molecul	12	0.00628	3.715	0.042	Biological	GO:00987	cell-cell adhesion via plasma-membrane adhesion molecules	0.00628	0.08402	0	0	12	285
GO:0097485 neuron projection guidance	11	0.00634	3.406	0.044	Biological	GO:00974	neuron projection guidance	0.00634	0.08434	0	0	11	250
GO:1901216 positive regulation of neuron death	6	0.00639	1.858	0.066	Biological	GO:19012	positive regulation of neuron death	0.00639	0.08434	0	0	6	91
GO:0072210 metanephric nephron development	4	0.00646	1.238	0.098	Biological	GO:00722	metanephric nephron development	0.00646	0.08434	0	0	4	41

GO:0007077 mitotic nuclear membrane disassembly	2	0.00655	0.619	0.286	Biological	GO:00070	mitotic nuclear membrane disassembly	0.00655	0.08434	0	0	2	7
GO:0021780 glial cell fate specification	2	0.00655	0.619	0.286	Biological	GO:00217	glial cell fate specification	0.00655	0.08434	0	0	2	7
GO:0021796 cerebral cortex regionalization	2	0.00655	0.619	0.286	Biological	GO:00217	cerebral cortex regionalization	0.00655	0.08434	0	0	2	7
GO:0032808 lacrimal gland development	2	0.00655	0.619	0.286	Biological	GO:00328	lacrimal gland development	0.00655	0.08434	0	0	2	7
GO:0034334 adherens junction maintenance	2	0.00655	0.619	0.286	Biological	GO:00343	adherens junction maintenance	0.00655	0.08434	0	0	2	7
GO:0071503 response to heparin	2	0.00655	0.619	0.286	Biological	GO:00715	response to heparin	0.00655	0.08434	0	0	2	7
GO:0072126 positive regulation of glomerular mesangial cell proliferation	2	0.00655	0.619	0.286	Biological	GO:00721	positive regulation of glomerular mesangial cell proliferation	0.00655	0.08434	0	0	2	7
GO:0097017 renal protein absorption	2	0.00655	0.619	0.286	Biological	GO:00970	renal protein absorption	0.00655	0.08434	0	0	2	7
GO:1904683 regulation of metalloendopeptidase activity	2	0.00655	0.619	0.286	Biological	GO:19046	regulation of metalloendopeptidase activity	0.00655	0.08434	0	0	2	7
GO:1905049 negative regulation of metallopeptidase activity	2	0.00655	0.619	0.286	Biological	GO:19050	negative regulation of metallopeptidase activity	0.00655	0.08434	0	0	2	7
GO:0035107 appendage morphogenesis	8	0.00655	2.477	0.053	Biological	GO:00351	appendage morphogenesis	0.00655	0.08434	0	0	8	151
GO:0035108 limb morphogenesis	8	0.00655	2.477	0.053	Biological	GO:00351	limb morphogenesis	0.00655	0.08434	0	0	8	151
GO:0007548 sex differentiation	12	0.00662	3.715	0.042	Biological	GO:00075	sex differentiation	0.00662	0.08501	0	0	12	287
GO:0030545 signaling receptor regulator activity	19	0.00667	5.556	0.035	Molecular	GO:00305	signaling receptor regulator activity	0.00667	0.15397	0	0	19	546
GO:0022407 regulation of cell-cell adhesion	17	0.00668	5.263	0.036	Biological	GO:00224	regulation of cell-cell adhesion	0.00668	0.08548	0	0	17	476
GO:1901214 regulation of neuron death	13	0.00673	4.025	0.04	Biological	GO:19012	regulation of neuron death	0.00673	0.08594	0	0	13	324
GO:0002761 regulation of myeloid leukocyte differentiation	7	0.00675	2.167	0.058	Biological	GO:00027	regulation of myeloid leukocyte differentiation	0.00675	0.08597	0	0	7	121
GO:0031406 carboxylic acid binding	9	0.00676	2.632	0.05	Molecular	GO:00314	carboxylic acid binding	0.00676	0.15397	0	0	9	181
GO:0042392 sphingosine-1-phosphate phosphatase activity	2	0.00676	0.585	0.286	Molecular	GO:00423	sphingosine-1-phosphate phosphatase activity	0.00676	0.15397	0	0	2	7
GO:0043167 ion binding	135	0.0068	39.474	0.022	Molecular	GO:00431	ion binding	0.0068	0.15397	0	0	135	6096
GO:0007610 behavior	21	0.00683	6.502	0.033	Biological	GO:00076	behavior	0.00683	0.08677	0	0	21	638
GO:0030020 extracellular matrix structural constituent conferring tensile	4	0.00684	1.17	0.098	Molecular	GO:00300	extracellular matrix structural constituent conferring tensile strength	0.00684	0.15397	0	0	4	41
GO:0052548 regulation of endopeptidase activity	13	0.0069	4.025	0.04	Biological	GO:00525	regulation of endopeptidase activity	0.0069	0.08742	0	0	13	325
GO:1903522 regulation of blood circulation	11	0.00691	3.406	0.043	Biological	GO:19035	regulation of blood circulation	0.00691	0.08742	0	0	11	253
GO:0098858 actin-based cell projection	10	0.00698	2.967	0.045	Cellular C	GO:00988	actin-based cell projection	0.00698	0.08116	0	0	10	221
GO:0001656 metanephros development	6	0.00709	1.858	0.065	Biological	GO:00016	metanephros development	0.00709	0.08939	0	0	6	93
GO:0001228 DNA-binding transcription activator activity, RNA polymera	17	0.0071	4.971	0.036	Molecular	GO:00012	DNA-binding transcription activator activity, RNA polymerase II-spec	0.0071	0.15397	0	0	17	471
GO:0030546 signaling receptor activator activity	18	0.00711	5.263	0.035	Molecular	GO:00305	signaling receptor activator activity	0.00711	0.15397	0	0	18	510
GO:0007530 sex determination	3	0.00715	0.929	0.136	Biological	GO:00075	sex determination	0.00715	0.08971	0	0	3	22
GO:0071379 cellular response to prostaglandin stimulus	3	0.00715	0.929	0.136	Biological	GO:00713	cellular response to prostaglandin stimulus	0.00715	0.08971	0	0	3	22
GO:0043296 apical junction complex	8	0.00716	2.374	0.052	Cellular C	GO:00432	apical junction complex	0.00716	0.08155	0	0	8	155
GO:0030326 embryonic limb morphogenesis	7	0.00736	2.167	0.057	Biological	GO:00303	embryonic limb morphogenesis	0.00736	0.09192	0	0	7	123
GO:0035113 embryonic appendage morphogenesis	7	0.00736	2.167	0.057	Biological	GO:00351	embryonic appendage morphogenesis	0.00736	0.09192	0	0	7	123
GO:0001843 neural tube closure	6	0.00746	1.858	0.064	Biological	GO:00018	neural tube closure	0.00746	0.09277	0	0	6	94
GO:0055001 muscle cell development	9	0.00747	2.786	0.048	Biological	GO:00550	muscle cell development	0.00747	0.09277	0	0	9	187
GO:0010633 negative regulation of epithelial cell migration	5	0.0075	1.548	0.075	Biological	GO:00106	negative regulation of epithelial cell migration	0.0075	0.09289	0	0	5	67
GO:0021700 developmental maturation	13	0.00761	4.025	0.04	Biological	GO:00217	developmental maturation	0.00761	0.09404	0	0	13	329
GO:0007613 memory	7	0.00768	2.167	0.056	Biological	GO:00076	memory	0.00768	0.09468	0	0	7	124
GO:0001216 DNA-binding transcription activator activity	17	0.0077	4.971	0.036	Molecular	GO:00012	DNA-binding transcription activator activity	0.0077	0.15736	0	0	17	475
GO:0043068 positive regulation of programmed cell death	18	0.0077	5.573	0.034	Biological	GO:00430	positive regulation of programmed cell death	0.0077	0.09468	0	0	18	523
GO:0071695 anatomical structure maturation	11	0.00774	3.406	0.043	Biological	GO:00716	anatomical structure maturation	0.00774	0.09495	0	0	11	257
GO:0060606 tube closure	6	0.00784	1.858	0.063	Biological	GO:00606	tube closure	0.00784	0.096	0	0	6	95
GO:0007267 cell-cell signaling	44	0.0079	13.622	0.026	Biological	GO:00072	cell-cell signaling	0.0079	0.09649	0	0	44	1666
GO:1902893 regulation of pri-miRNA transcription by RNA polymerase	5	0.00797	1.548	0.074	Biological	GO:19028	regulation of pri-miRNA transcription by RNA polymerase II	0.00797	0.097	0	0	5	68
GO:0018209 peptidyl-serine modification	13	0.00798	4.025	0.039	Biological	GO:00182	peptidyl-serine modification	0.00798	0.097	0	0	13	331
GO:0043085 positive regulation of catalytic activity	32	0.008	9.907	0.029	Biological	GO:00430	positive regulation of catalytic activity	0.008	0.09701	0	0	32	1121
GO:0006509 membrane protein ectodomain proteolysis	4	0.00829	1.238	0.091	Biological	GO:00065	membrane protein ectodomain proteolysis	0.00829	0.10021	0	0	4	44
GO:0051173 positive regulation of nitrogen compound metabolic proces	74	0.00832	22.91	0.024	Biological	GO:00511	positive regulation of nitrogen compound metabolic process	0.00832	0.10021	0	0	74	3116
GO:0038127 ERBB signaling pathway	7	0.00836	2.167	0.056	Biological	GO:00381	ERBB signaling pathway	0.00836	0.10021	0	0	7	126
GO:0009968 negative regulation of signal transduction	35	0.00837	10.836	0.028	Biological	GO:00099	negative regulation of signal transduction	0.00837	0.10021	0	0	35	1259
GO:0042325 regulation of phosphorylation	34	0.00837	10.526	0.028	Biological	GO:00423	regulation of phosphorylation	0.00837	0.10021	0	0	34	1214
GO:0061673 mitotic spindle astral microtubule	2	0.00844	0.593	0.25	Cellular C	GO:00616	mitotic spindle astral microtubule	0.00844	0.09409	0	0	2	8
GO:0008134 transcription factor binding	20	0.00844	5.848	0.033	Molecular	GO:00081	transcription factor binding	0.00844	0.15736	0	0	20	599
GO:0002573 myeloid leukocyte differentiation	10	0.00847	3.096	0.044	Biological	GO:00025	myeloid leukocyte differentiation	0.00847	0.10073	0	0	10	225
GO:0061614 pri-miRNA transcription by RNA polymerase II	5	0.00847	1.548	0.072	Biological	GO:00616	pri-miRNA transcription by RNA polymerase II	0.00847	0.10073	0	0	5	69
GO:0097120 receptor localization to synapse	5	0.00847	1.548	0.072	Biological	GO:00971	receptor localization to synapse	0.00847	0.10073	0	0	5	69
GO:0009931 calcium-dependent protein serine/threonine kinase activity	3	0.00849	0.877	0.13	Molecular	GO:00099	calcium-dependent protein serine/threonine kinase activity	0.00849	0.15736	0	0	3	23
GO:0035035 histone acetyltransferase binding	3	0.00849	0.877	0.13	Molecular	GO:00350	histone acetyltransferase binding	0.00849	0.15736	0	0	3	23
GO:0099173 postsynapse organization	9	0.00852	2.786	0.047	Biological	GO:00991	postsynapse organization	0.00852	0.1009	0	0	9	191
GO:0010243 response to organonitrogen compound	30	0.00858	9.288	0.029	Biological	GO:00102	response to organonitrogen compound	0.00858	0.1009	0	0	30	1038
GO:0005856 cytoskeleton	59	0.00862	17.507	0.024	Cellular C	GO:00058	cytoskeleton	0.00862	0.09411	0	0	59	2411
GO:0007207 phospholipase C-activating G protein-coupled acetylcholin	2	0.00863	0.619	0.25	Biological	GO:00072	phospholipase C-activating G protein-coupled acetylcholine receptor	0.00863	0.1009	0	0	2	8
GO:0050861 positive regulation of B cell receptor signaling pathway	2	0.00863	0.619	0.25	Biological	GO:00508	positive regulation of B cell receptor signaling pathway	0.00863	0.1009	0	0	2	8
GO:0051045 negative regulation of membrane protein ectodomain prote	2	0.00863	0.619	0.25	Biological	GO:00510	negative regulation of membrane protein ectodomain proteolysis	0.00863	0.1009	0	0	2	8
GO:0070255 regulation of mucus secretion	2	0.00863	0.619	0.25	Biological	GO:00702	regulation of mucus secretion	0.00863	0.1009	0	0	2	8
GO:0072033 renal vesicle formation	2	0.00863	0.619	0.25	Biological	GO:00720	renal vesicle formation	0.00863	0.1009	0	0	2	8
GO:0017124 SH3 domain binding	7	0.00875	2.047	0.056	Molecular	GO:00171	SH3 domain binding	0.00875	0.15736	0	0	7	125
GO:0035184 histone threonine kinase activity	2	0.00891	0.585	0.25	Molecular	GO:00351	histone threonine kinase activity	0.00891	0.15736	0	0	2	8
GO:0047023 androsterone dehydrogenase activity	2	0.00891	0.585	0.25	Molecular	GO:00470	androsterone dehydrogenase activity	0.00891	0.15736	0	0	2	8
GO:0010463 mesenchymal cell proliferation	4	0.00898	1.238	0.089	Biological	GO:00104	mesenchymal cell proliferation	0.00898	0.10417	0	0	4	45
GO:0043620 regulation of DNA-templated transcription in response to s	4	0.00898	1.238	0.089	Biological	GO:00436	regulation of DNA-templated transcription in response to stress	0.00898	0.10417	0	0	4	45
GO:0061098 positive regulation of protein tyrosine kinase activity	4	0.00898	1.238	0.089	Biological	GO:00610	positive regulation of protein tyrosine kinase activity	0.00898	0.10417	0	0	4	45
GO:0032722 positive regulation of chemokine production	5	0.00899	1.548	0.071	Biological	GO:00327	positive regulation of chemokine production	0.00899	0.10417	0	0	5	70
GO:0051128 regulation of cellular component organization	59	0.00902	18.266	0.025	Biological	GO:00511	regulation of cellular component organization	0.00902	0.10426	0	0	59	2392
GO:1901699 cellular response to nitrogen compound	22	0.00909	6.811	0.032	Biological	GO:19016	cellular response to nitrogen compound	0.00909	0.10481	0	0	22	697
GO:0004222 metalloendopeptidase activity	7	0.00912	2.047	0.056	Molecular	GO:00042	metalloendopeptidase activity	0.00912	0.15736	0	0	7	126
GO:0002070 epithelial cell maturation	3	0.00915	0.929	0.125	Biological	GO:00020	epithelial cell maturation	0.00915	0.10502	0	0	3	24
GO:0051043 regulation of membrane protein ectodomain proteolysis	3	0.00915	0.929	0.125	Biological	GO:00510	regulation of membrane protein ectodomain proteolysis	0.00915	0.10502	0	0	3	24
GO:0001649 osteoblast differentiation	10	0.00924	3.096	0.044	Biological	GO:00016	osteoblast differentiation	0.00924	0.10588	0	0	10	228
GO:0044297 cell body	19	0.00927	5.638	0.033	Cellular C	GO:00442	cell body	0.00927	0.09923	0	0	19	580
GO:0000302 response to reactive oxygen species	9	0.00939	2.786	0.046	Biological	GO:00003	response to reactive oxygen species	0.00939	0.10726	0	0	9	194
GO:0001503 ossification	15	0.00948	4.644	0.036	Biological	GO:00015	ossification	0.00948	0.10807	0	0	15	415
GO:0044087 regulation of cellular component biogenesis	29	0.00953	8.978	0.029	Biological	GO:00440	regulation of cellular component biogenesis	0.00953	0.10836	0	0	29	1002
GO:0010857 calcium-dependent protein kinase activity	3	0.00957	0.877	0.125	Molecular	GO:00108	calcium-dependent protein kinase activity	0.00957	0.15736	0	0	3	24
GO:0070412 R-SMAD binding	3	0.00957	0.877	0.125	Molecular	GO:00704	R-SMAD binding	0.00957	0.15736	0	0	3	24
GO:0050790 regulation of catalytic activity	48	0.00967	14.861	0.026	Biological	GO:00507	regulation of catalytic activity	0.00967	0.10951	0	0	48	1874
GO:0021762 substantia nigra development	4	0.00969	1.238	0.087	Biological	GO:00217	substantia nigra development	0.00969	0.10951	0	0	4	46
GO:0042551 neuron maturation	4	0.00969	1.238	0.087	Biological	GO:00425	neuron maturation	0.00969	0.10951	0	0	4	46
GO:0001816 cytokine production	23	0.00992	7.121	0.031	Biological	GO:00018	cytokine production	0.00992	0.11186	0	0	23	745
GO:0014020 primary neural tube formation	6	0.00998	1.858	0.06	Biological	GO:00140	primary neural tube formation	0.00998	0.11225	0	0	6	100
GO:0098984 neuron to neuron synapse	14	0.01002	4.154	0.037	Cellular C	GO:00989	neuron to neuron synapse	0.01002	0.10511	0	0	14	383
GO:0003158 endothelium development	7	0.01024	2.167	0.053	Biological	GO:00031	endothelium development	0.01024	0.11408	0	0	7	131
GO:0032727 positive regulation of interferon-alpha production	3	0.01026	0.929	0.12	Biological	GO:00327	positive regulation of interferon-alpha production	0.01026	0.11408	0	0	3	25
GO:0072273 metanephric nephron morphogenesis	3	0.01026	0.929	0.12	Biological	GO:00722	metanephric nephron morphogenesis	0.01026	0.11408	0	0	3	25
GO:0090103 cochlea morphogenesis	3	0.01026	0.929	0.12	Biological	GO:00901	cochlea morphogenesis	0.01026	0.11408	0	0	3	25
GO:1905207 regulation of cardiocyte differentiation	3	0.01026	0.929	0.12	Biological	GO:19052	regulation of cardiocyte differentiation	0.01026	0.11408	0	0	3	25
GO:0006468 protein phosphorylation	41	0.01034	12.693	0.026	Biological	GO:00064	protein phosphorylation	0.01034	0.1147	0	0	41	1553
GO:0051091 positive regulation of DNA-binding transcription factor activ	11	0.0104	3.406	0.041	Biological	GO:00510	positive regulation of DNA-binding transcription factor activity	0.0104	0.11515	0	0	11	268
GO:0060443 mammary gland morphogenesis	4	0.01044	1.238	0.085	Biological	GO:00604	mammary gland morphogenesis	0.01044	0.11515	0	0	4	47
GO:1903539 protein localization to postsynaptic membrane	4	0.01044	1.238	0.085	Biological	GO:19035	protein localization to postsynaptic membrane	0.01044	0.11515	0	0	4	47
GO:0005925 focal adhesion	15	0.01059	4.451	0.035	Cellular C	GO:00059	focal adhesion	0.01059	0.1062	0	0	15	425
GO:0010648 negative regulation of cell communication	37	0.0106	11.455	0.027	Biological	GO:00106	negative regulation of cell communication	0.0106	0.11645	0	0	37	1371
GO:2000116 regulation of cysteine-type endopeptidase activity	10	0.01066	3.096	0.043	Biological	GO:20001	regulation of cysteine-type endopeptidase activity	0.01066	0.11645	0	0	10	233
GO:0005915 zonula adherens	2	0.01073	0.593	0.222	Cellular C	GO:00059	zonula adherens	0.01073	0.1062	0	0	2	9
GO:0051246 regulation of protein metabolic process	61	0.01082	18.885	0.024	Biological	GO:00512	regulation of protein metabolic process	0.01082	0.11645	0	0	61	2511
GO:0030162 regulation of proteolysis	22	0.01093	6.811	0.031	Biological	GO:00301	regulation of proteolysis	0.01093	0.11645	0	0	22	709
GO:0000098 sulfur amino acid catabolic process	2	0.01096	0.619	0.222	Biological	GO:00000	sulfur amino acid catabolic process	0.01096	0.11645	0	0	2	9
GO:0016115 terpenoid catabolic process	2	0.01096	0.619	0.222	Biological	GO:00161	terpenoid catabolic process	0.01096	0.11645	0	0	2	9
GO:0019317 fucose catabolic process	2	0.01096	0.619	0.222	Biological	GO:00193	fucose catabolic process	0.01096	0.11645	0	0	2	9
GO:0019371 cyclooxygenase pathway	2	0.01096	0.619	0.222	Biological	GO:00193	cyclooxygenase pathway	0.01096	0.11645	0	0	2	9
GO:0034351 negative regulation of glial cell apoptotic process	2	0.01096	0.619	0.222	Biological	GO:00343	negative regulation of glial cell apoptotic process	0.01096	0.11645	0	0	2	9
GO:0035332 positive regulation of hippo signaling	2	0.01096	0.619	0.222	Biological	GO:00353	positive regulation of hippo signaling	0.01096	0.11645	0	0	2	9
GO:0042355 L-fucose catabolic process	2	0.01096	0.619	0.222	Biological	GO:00423	L-fucose catabolic process	0.01096	0.11645	0	0	2	9
GO:0044597 daunorubicin metabolic process	2	0.01096	0.619	0.222	Biological	GO:00445	daunorubicin metabolic process	0.01096	0.11645	0	0	2	9
GO:0060385 axonogenesis involved in innervation	2	0.01096	0.619	0.222	Biological	GO:00603	axonogenesis involved in innervation	0.01096	0.11645	0	0	2	9
GO:0060677 ureteric bud elongation	2	0.01096	0.619	0.222	Biological	GO:00606	ureteric bud elongation	0.01096	0.11645	0	0	2	9
GO:0097021 lymphocyte migration into lymphoid organs	2	0.01096	0.619	0.222	Biological	GO:00970	lymphocyte migration into lymphoid organs	0.01096	0.11645	0	0	2	9
GO:1901724 positive regulation of cell proliferation involved in kidney de	2	0.01096	0.619	0.222	Biological	GO:19017	positive regulation of cell proliferation involved in kidney development	0.01096	0.11645	0	0	2	9
GO:2000343 positive regulation of chemokine (C-X-C motif) ligand 2 pro	2	0.01096	0.619	0.222	Biological	GO:20003	positive regulation of chemokine (C-X-C motif) ligand 2 production	0.01096	0.11645	0	0	2	9
GO:0001101 response to acid chemical	7	0.01107	2.167	0.053	Biological	GO:00011	response to acid chemical	0.01107	0.11713	0	0	7	133
GO:0070555 response to interleukin-1	7	0.01107	2.167	0.053	Biological	GO:00705	response to interleukin-1	0.01107	0.11713	0	0	7	133
GO:0016323 basolateral plasma membrane	10	0.0111	2.967	0.042	Cellular C	GO:00163	basolateral plasma membrane	0.0111	0.1062	0	0	10	237
GO:0030425 dendrite	20	0.01117	5.935	0.032	Cellular C	GO:00304	dendrite	0.01117	0.1062	0	0	20	633
GO:0023057 negative regulation of signaling	37	0.01119	11.455	0.027	Biological	GO:00230	negative regulation of signaling	0.01119	0.11815	0	0	37	1376
GO:0062237 protein localization to postsynapse	4	0.01123	1.238	0.083	Biological	GO:00622	protein localization to postsynapse	0.01123	0.1182	0	0	4	48
GO:0072507 divalent inorganic cation homeostasis	18	0.01124	5.573	0.033	Biological	GO:00725	divalent inorganic cation homeostasis	0.01124	0.1182	0	0	18	544
GO:0050801 ion homeostasis	25	0.01131	7.74	0.03	Biological	GO:00508	ion homeostasis	0.01131	0.11833	0	0	25	840
GO:0005114 type II transforming growth factor beta receptor binding	2	0.01131	0.585	0.222	Molecular	GO:00051	type II transforming growth factor beta receptor binding	0.01131	0.15736	0	0	2	9
GO:0030023 extracellular matrix constituent conferring elasticity	2	0.01131	0.585	0.222	Molecular	GO:00300	extracellular matrix constituent conferring elasticity	0.01131	0.15736	0	0	2	9
GO:0047044 androstan-3-alpha,17-beta-diol dehydrogenase activity	2	0.01131	0.585	0.222	Molecular	GO:00470	androstan-3-alpha,17-beta-diol dehydrogenase activity	0.01131	0.15736	0	0	2	9
GO:0030016 myofibril	10	0.01141	2.967	0.042	Cellular C	GO:00300	myofibril	0.01141	0.1062	0	0	10	238
GO:0014829 vascular associated smooth muscle contraction	3	0.01144	0.929	0.115	Biological	GO:00148	vascular associated smooth muscle contraction	0.01144	0.11833	0	0	3	26
GO:0035994 response to muscle stretch	3	0.01144	0.929	0.115	Biological	GO:00359	response to muscle stretch	0.01144	0.11833	0	0	3	26
GO:0045662 negative regulation of myoblast differentiation	3	0.01144	0.929	0.115	Biological	GO:00456	negative regulation of myoblast differentiation	0.01144	0.11833	0	0	3	26
GO:0045672 positive regulation of osteoclast differentiation	3	0.01144	0.929	0.115	Biological	GO:00456	positive regulation of osteoclast differentiation	0.01144	0.11833	0	0	3	26
GO:0002027 regulation of heart rate	6	0.01144	1.858	0.058	Biological	GO:00020	regulation of heart rate	0.01144	0.11833	0	0	6	103
GO:0006576 cellular biogenic amine metabolic process	6	0.01144	1.858	0.058	Biological	GO:00065	cellular biogenic amine metabolic process	0.01144	0.11833	0	0	6	103
GO:0061326 renal tubule development	6	0.01144	1.858	0.058	Biological	GO:00613	renal tubule development	0.01144	0.11833	0	0	6	103
GO:0098771 inorganic ion homeostasis	24	0.01151	7.43	0.03	Biological	GO:00987	inorganic ion homeostasis	0.01151	0.11872	0	0	24	798
GO:0097447 dendritic tree	20	0.01153	5.935	0.031	Cellular C	GO:00974	dendritic tree	0.01153	0.1062	0	0	20	635
GO:0032268 regulation of cellular protein metabolic process	57	0.01157	17.647	0.025	Biological	GO:00322	regulation of cellular protein metabolic process	0.01157	0.11917	0	0	57	2325
GO:0019838 growth factor binding	7	0.0116	2.047	0.053	Molecular	GO:00198	growth factor binding	0.0116	0.15736	0	0	7	132
GO:0006979 response to oxidative stress	15	0.0116	4.644	0.035	Biological	GO:00069	response to oxidative stress	0.0116	0.11921	0	0	15	425
GO:0005911 cell-cell junction	17	0.0117	5.045	0.033	Cellular C	GO:00059	cell-cell junction	0.0117	0.1062	0	0	17	511
GO:0032279 asymmetric synapse	13	0.01186	3.858	0.037	Cellular C	GO:00322	asymmetric synapse	0.01186	0.1062	0	0	13	352
GO:0043025 neuronal cell body	17	0.01191	5.045	0.033	Cellular C	GO:00430	neuronal cell body	0.01191	0.1062	0	0	17	512
GO:0001736 establishment of planar polarity	5	0.01192	1.548	0.067	Biological	GO:00017	establishment of planar polarity	0.01192	0.12194	0	0	5	75
GO:0007164 establishment of tissue polarity	5	0.01192	1.548	0.067	Biological	GO:00071	establishment of tissue polarity	0.01192	0.12194	0	0	5	75
GO:0030850 prostate gland development	4	0.01206	1.238	0.082	Biological	GO:00308	prostate gland development	0.01206	0.12268	0	0	4	49
GO:0031103 axon regeneration	4	0.01206	1.238	0.082	Biological	GO:00311	axon regeneration	0.01206	0.12268	0	0	4	49
GO:0042311 vasodilation	4	0.01206	1.238	0.082	Biological	GO:00423	vasodilation	0.01206	0.12268	0	0	4	49
GO:0016758 hexosyltransferase activity	9	0.01214	2.632	0.045	Molecular	GO:00167	hexosyltransferase activity	0.01214	0.15736	0	0	9	199

GO:0043009 chordate embryonic development	21	0.01222	6.502	0.031	Biological	GO:00430	chordate embryonic development	0.01222	0.12376	0	0	21	674
GO:0007160 cell-matrix adhesion	10	0.01224	3.096	0.042	Biological	GO:00071	cell-matrix adhesion	0.01224	0.12376	0	0	10	238
GO:0007169 transmembrane receptor protein tyrosine kinase signaling	20	0.01224	6.192	0.032	Biological	GO:00071	transmembrane receptor protein tyrosine kinase signaling pathway	0.01224	0.12376	0	0	20	632
GO:0090090 negative regulation of canonical Wnt signaling pathway	7	0.01241	2.167	0.051	Biological	GO:00900	negative regulation of canonical Wnt signaling pathway	0.01241	0.12503	0	0	7	136
GO:0090092 regulation of transmembrane receptor protein serine/threo	11	0.01243	3.406	0.04	Biological	GO:00900	regulation of transmembrane receptor protein serine/threonine kinase	0.01243	0.12503	0	0	11	275
GO:0007519 skeletal muscle tissue development	8	0.01247	2.477	0.047	Biological	GO:00075	skeletal muscle tissue development	0.01247	0.12503	0	0	8	169
GO:0046661 male sex differentiation	8	0.01247	2.477	0.047	Biological	GO:00466	male sex differentiation	0.01247	0.12503	0	0	8	169
GO:0018105 peptidyl-serine phosphorylation	12	0.01263	3.715	0.038	Biological	GO:00181	peptidyl-serine phosphorylation	0.01263	0.12634	0	0	12	313
GO:0060314 regulation of ryanodine-sensitive calcium-release channel a	3	0.0127	0.929	0.111	Biological	GO:00603	regulation of ryanodine-sensitive calcium-release channel activity	0.0127	0.12634	0	0	3	27
GO:0060384 innervation	3	0.0127	0.929	0.111	Biological	GO:00603	innervation	0.0127	0.12634	0	0	3	27
GO:0060740 prostate gland epithelium morphogenesis	3	0.0127	0.929	0.111	Biological	GO:00607	prostate gland epithelium morphogenesis	0.0127	0.12634	0	0	3	27
GO:0032813 tumor necrosis factor receptor superfamily binding	4	0.01276	1.17	0.082	Molecular	GO:00328	tumor necrosis factor receptor superfamily binding	0.01276	0.15736	0	0	4	49
GO:0007507 heart development	19	0.01301	5.882	0.032	Biological	GO:00075	heart development	0.01301	0.12875	0	0	19	594
GO:0014701 junctional sarcoplasmic reticulum membrane	2	0.01325	0.593	0.2	Cellular C	GO:00147	junctional sarcoplasmic reticulum membrane	0.01325	0.1162	0	0	2	10
GO:0071813 lipoprotein particle binding	3	0.01328	0.877	0.111	Molecular	GO:00718	lipoprotein particle binding	0.01328	0.15736	0	0	3	27
GO:0071814 protein-lipid complex binding	3	0.01328	0.877	0.111	Molecular	GO:00718	protein-lipid complex binding	0.01328	0.15736	0	0	3	27
GO:0048771 tissue remodeling	8	0.01331	2.477	0.047	Biological	GO:00487	tissue remodeling	0.01331	0.12875	0	0	8	171
GO:0042803 protein homodimerization activity	22	0.01332	6.433	0.031	Molecular	GO:00428	protein homodimerization activity	0.01332	0.15736	0	0	22	710
GO:0034599 cellular response to oxidative stress	11	0.01339	3.406	0.04	Biological	GO:00345	cellular response to oxidative stress	0.01339	0.12875	0	0	11	278
GO:0043549 regulation of kinase activity	22	0.01346	6.811	0.03	Biological	GO:00435	regulation of kinase activity	0.01346	0.12875	0	0	22	723
GO:0090287 regulation of cellular response to growth factor stimulus	12	0.01353	3.715	0.038	Biological	GO:00902	regulation of cellular response to growth factor stimulus	0.01353	0.12875	0	0	12	316
GO:0002138 retinoic acid biosynthetic process	2	0.01354	0.619	0.2	Biological	GO:00021	retinoic acid biosynthetic process	0.01354	0.12875	0	0	2	10
GO:0002934 desmosome organization	2	0.01354	0.619	0.2	Biological	GO:00029	desmosome organization	0.01354	0.12875	0	0	2	10
GO:0003253 cardiac neural crest cell migration involved in outflow tract	2	0.01354	0.619	0.2	Biological	GO:00032	cardiac neural crest cell migration involved in outflow tract morphoge	0.01354	0.12875	0	0	2	10
GO:0015820 leucine transport	2	0.01354	0.619	0.2	Biological	GO:00158	leucine transport	0.01354	0.12875	0	0	2	10
GO:0030638 polyketide metabolic process	2	0.01354	0.619	0.2	Biological	GO:00306	polyketide metabolic process	0.01354	0.12875	0	0	2	10
GO:0030647 aminoglycoside antibiotic metabolic process	2	0.01354	0.619	0.2	Biological	GO:00306	aminoglycoside antibiotic metabolic process	0.01354	0.12875	0	0	2	10
GO:0042354 L-fucose metabolic process	2	0.01354	0.619	0.2	Biological	GO:00423	L-fucose metabolic process	0.01354	0.12875	0	0	2	10
GO:0043653 mitochondrial fragmentation involved in apoptotic process	2	0.01354	0.619	0.2	Biological	GO:00436	mitochondrial fragmentation involved in apoptotic process	0.01354	0.12875	0	0	2	10
GO:0044598 doxorubicin metabolic process	2	0.01354	0.619	0.2	Biological	GO:00445	doxorubicin metabolic process	0.01354	0.12875	0	0	2	10
GO:0048865 stem cell fate commitment	2	0.01354	0.619	0.2	Biological	GO:00488	stem cell fate commitment	0.01354	0.12875	0	0	2	10
GO:0051081 nuclear membrane disassembly	2	0.01354	0.619	0.2	Biological	GO:00510	nuclear membrane disassembly	0.01354	0.12875	0	0	2	10
GO:0060056 mammary gland involution	2	0.01354	0.619	0.2	Biological	GO:00600	mammary gland involution	0.01354	0.12875	0	0	2	10
GO:0070294 renal sodium ion absorption	2	0.01354	0.619	0.2	Biological	GO:00702	renal sodium ion absorption	0.01354	0.12875	0	0	2	10
GO:0072124 regulation of glomerular mesangial cell proliferation	2	0.01354	0.619	0.2	Biological	GO:00721	regulation of glomerular mesangial cell proliferation	0.01354	0.12875	0	0	2	10
GO:0072203 cell proliferation involved in metanephros development	2	0.01354	0.619	0.2	Biological	GO:00722	cell proliferation involved in metanephros development	0.01354	0.12875	0	0	2	10
GO:0097113 AMPA glutamate receptor clustering	2	0.01354	0.619	0.2	Biological	GO:00971	AMPA glutamate receptor clustering	0.01354	0.12875	0	0	2	10
GO:0097688 glutamate receptor clustering	2	0.01354	0.619	0.2	Biological	GO:00976	glutamate receptor clustering	0.01354	0.12875	0	0	2	10
GO:1903054 negative regulation of extracellular matrix organization	2	0.01354	0.619	0.2	Biological	GO:19030	negative regulation of extracellular matrix organization	0.01354	0.12875	0	0	2	10
GO:0060047 heart contraction	10	0.01362	3.096	0.041	Biological	GO:00600	heart contraction	0.01362	0.12932	0	0	10	242
GO:0045165 cell fate commitment	11	0.01372	3.406	0.039	Biological	GO:00451	cell fate commitment	0.01372	0.13	0	0	11	279
GO:0034332 adherens junction organization	4	0.01383	1.238	0.078	Biological	GO:00343	adherens junction organization	0.01383	0.13032	0	0	4	51
GO:0042461 photoreceptor cell development	4	0.01383	1.238	0.078	Biological	GO:00424	photoreceptor cell development	0.01383	0.13032	0	0	4	51
GO:1902895 positive regulation of pri-miRNA transcription by RNA poly	4	0.01383	1.238	0.078	Biological	GO:19028	positive regulation of pri-miRNA transcription by RNA polymerase II	0.01383	0.13032	0	0	4	51
GO:0071417 cellular response to organonitrogen compound	20	0.01387	6.192	0.031	Biological	GO:00714	cellular response to organonitrogen compound	0.01387	0.13047	0	0	20	640
GO:0008239 dipeptidyl-peptidase activity	2	0.01397	0.585	0.2	Molecular	GO:00082	dipeptidyl-peptidase activity	0.01397	0.15736	0	0	2	10
GO:0060479 lung cell differentiation	3	0.01404	0.929	0.107	Biological	GO:00604	lung cell differentiation	0.01404	0.13102	0	0	3	28
GO:0060487 lung epithelial cell differentiation	3	0.01404	0.929	0.107	Biological	GO:00604	lung epithelial cell differentiation	0.01404	0.13102	0	0	3	28
GO:0099633 protein localization to postsynaptic specialization membran	3	0.01404	0.929	0.107	Biological	GO:00996	protein localization to postsynaptic specialization membrane	0.01404	0.13102	0	0	3	28
GO:0099645 neurotransmitter receptor localization to postsynaptic spec	3	0.01404	0.929	0.107	Biological	GO:00996	neurotransmitter receptor localization to postsynaptic specialization m	0.01404	0.13102	0	0	3	28
GO:0001841 neural tube formation	6	0.01421	1.858	0.056	Biological	GO:00018	neural tube formation	0.01421	0.13242	0	0	6	108
GO:0010977 negative regulation of neuron projection development	7	0.01437	2.167	0.05	Biological	GO:00109	negative regulation of neuron projection development	0.01437	0.13337	0	0	7	140
GO:0017015 regulation of transforming growth factor beta receptor sign	7	0.01437	2.167	0.05	Biological	GO:00170	regulation of transforming growth factor beta receptor signaling pathw	0.01437	0.13337	0	0	7	140
GO:0072503 cellular divalent inorganic cation homeostasis	17	0.01456	5.263	0.033	Biological	GO:00725	cellular divalent inorganic cation homeostasis	0.01456	0.13495	0	0	17	518
GO:0000165 MAPK cascade	23	0.01464	7.121	0.03	Biological	GO:00001	MAPK cascade	0.01464	0.13537	0	0	23	772
GO:0045499 chemorepellent activity	3	0.01467	0.877	0.107	Molecular	GO:00454	chemorepellent activity	0.01467	0.15736	0	0	3	28
GO:0010596 negative regulation of endothelial cell migration	4	0.01477	1.238	0.077	Biological	GO:00105	negative regulation of endothelial cell migration	0.01477	0.13612	0	0	4	52
GO:0051489 regulation of filopodium assembly	4	0.01477	1.238	0.077	Biological	GO:00514	regulation of filopodium assembly	0.01477	0.13612	0	0	4	52
GO:0019220 regulation of phosphate metabolic process	36	0.01488	11.146	0.027	Biological	GO:00192	regulation of phosphate metabolic process	0.01488	0.13686	0	0	36	1357
GO:0051174 regulation of phosphorus metabolic process	36	0.01504	11.146	0.027	Biological	GO:00511	regulation of phosphorus metabolic process	0.01504	0.13752	0	0	36	1358
GO:0001819 positive regulation of cytokine production	16	0.01533	4.954	0.033	Biological	GO:00018	positive regulation of cytokine production	0.01533	0.13752	0	0	16	480
GO:0097435 supramolecular fiber organization	24	0.01535	7.43	0.029	Biological	GO:00974	supramolecular fiber organization	0.01535	0.13752	0	0	24	819
GO:1902414 protein localization to cell junction	6	0.01544	1.858	0.055	Biological	GO:19024	protein localization to cell junction	0.01544	0.13752	0	0	6	110
GO:0003094 glomerular filtration	3	0.01545	0.929	0.103	Biological	GO:00030	glomerular filtration	0.01545	0.13752	0	0	3	29
GO:0060512 prostate gland morphogenesis	3	0.01545	0.929	0.103	Biological	GO:00605	prostate gland morphogenesis	0.01545	0.13752	0	0	3	29
GO:0016043 cellular component organization	136	0.01547	42.105	0.021	Biological	GO:00160	cellular component organization	0.01547	0.13752	0	0	136	6412
GO:0030864 cortical actin cytoskeleton	5	0.01552	1.484	0.062	Cellular C	GO:00308	cortical actin cytoskeleton	0.01552	0.13058	0	0	5	81
GO:0002020 protease binding	7	0.01562	2.047	0.05	Molecular	GO:00020	protease binding	0.01562	0.15736	0	0	7	140
GO:0000139 Golgi membrane	20	0.01567	5.935	0.031	Cellular C	GO:00001	Golgi membrane	0.01567	0.13058	0	0	20	655
GO:1903844 regulation of cellular response to transforming growth facto	7	0.01598	2.167	0.049	Biological	GO:19038	regulation of cellular response to transforming growth factor beta stin	0.01598	0.13752	0	0	7	143
GO:0000235 astral microtubule	2	0.016	0.593	0.182	Cellular C	GO:00002	astral microtubule	0.016	0.13058	0	0	2	11
GO:0005818 aster	2	0.016	0.593	0.182	Cellular C	GO:00058	aster	0.016	0.13058	0	0	2	11
GO:0043410 positive regulation of MAPK cascade	16	0.01616	4.954	0.033	Biological	GO:00434	positive regulation of MAPK cascade	0.01616	0.13752	0	0	16	483
GO:0045178 basal part of cell	11	0.01616	3.264	0.038	Cellular C	GO:00451	basal part of cell	0.01616	0.13058	0	0	11	289
GO:0061097 regulation of protein tyrosine kinase activity	5	0.01621	1.548	0.062	Biological	GO:00610	regulation of protein tyrosine kinase activity	0.01621	0.13752	0	0	5	81
GO:0046914 transition metal ion binding	31	0.01634	9.064	0.028	Molecular	GO:00469	transition metal ion binding	0.01634	0.15736	0	0	31	1117
GO:0009950 dorsal/ventral axis specification	2	0.01635	0.619	0.182	Biological	GO:00099	dorsal/ventral axis specification	0.01635	0.13752	0	0	2	11
GO:0016102 diterpenoid biosynthetic process	2	0.01635	0.619	0.182	Biological	GO:00161	diterpenoid biosynthetic process	0.01635	0.13752	0	0	2	11
GO:0021889 olfactory bulb interneuron differentiation	2	0.01635	0.619	0.182	Biological	GO:00218	olfactory bulb interneuron differentiation	0.01635	0.13752	0	0	2	11
GO:0021903 rostrocaudal neural tube patterning	2	0.01635	0.619	0.182	Biological	GO:00219	rostrocaudal neural tube patterning	0.01635	0.13752	0	0	2	11
GO:0033210 leptin-mediated signaling pathway	2	0.01635	0.619	0.182	Biological	GO:00332	leptin-mediated signaling pathway	0.01635	0.13752	0	0	2	11
GO:0034350 regulation of glial cell apoptotic process	2	0.01635	0.619	0.182	Biological	GO:00343	regulation of glial cell apoptotic process	0.01635	0.13752	0	0	2	11
GO:0035878 nail development	2	0.01635	0.619	0.182	Biological	GO:00358	nail development	0.01635	0.13752	0	0	2	11
GO:0042045 epithelial fluid transport	2	0.01635	0.619	0.182	Biological	GO:00420	epithelial fluid transport	0.01635	0.13752	0	0	2	11
GO:0046689 response to mercury ion	2	0.01635	0.619	0.182	Biological	GO:00466	response to mercury ion	0.01635	0.13752	0	0	2	11
GO:0060742 epithelial cell differentiation involved in prostate gland deve	2	0.01635	0.619	0.182	Biological	GO:00607	epithelial cell differentiation involved in prostate gland development	0.01635	0.13752	0	0	2	11
GO:0060767 epithelial cell proliferation involved in prostate gland develo	2	0.01635	0.619	0.182	Biological	GO:00607	epithelial cell proliferation involved in prostate gland development	0.01635	0.13752	0	0	2	11
GO:0071492 cellular response to UV-A	2	0.01635	0.619	0.182	Biological	GO:00714	cellular response to UV-A	0.01635	0.13752	0	0	2	11
GO:0072110 glomerular mesangial cell proliferation	2	0.01635	0.619	0.182	Biological	GO:00721	glomerular mesangial cell proliferation	0.01635	0.13752	0	0	2	11
GO:1900272 negative regulation of long-term synaptic potentiation	2	0.01635	0.619	0.182	Biological	GO:19002	negative regulation of long-term synaptic potentiation	0.01635	0.13752	0	0	2	11
GO:0031399 regulation of protein modification process	39	0.01637	12.074	0.026	Biological	GO:00313	regulation of protein modification process	0.01637	0.13752	0	0	39	1506
GO:0035051 cardiocyte differentiation	7	0.01654	2.167	0.049	Biological	GO:00350	cardiocyte differentiation	0.01654	0.13752	0	0	7	144
GO:0022414 reproductive process	41	0.01654	12.693	0.026	Biological	GO:00224	reproductive process	0.01654	0.13752	0	0	41	1601
GO:0045860 positive regulation of protein kinase activity	13	0.01656	4.025	0.036	Biological	GO:00458	positive regulation of protein kinase activity	0.01656	0.13752	0	0	13	364
GO:0001817 regulation of cytokine production	22	0.01666	6.811	0.03	Biological	GO:00018	regulation of cytokine production	0.01666	0.13752	0	0	22	738
GO:0045446 endothelial cell differentiation	6	0.01674	1.858	0.054	Biological	GO:00454	endothelial cell differentiation	0.01674	0.13752	0	0	6	112
GO:0035176 social behavior	4	0.01678	1.238	0.074	Biological	GO:00351	social behavior	0.01678	0.13752	0	0	4	54
GO:0048662 negative regulation of smooth muscle cell proliferation	4	0.01678	1.238	0.074	Biological	GO:00486	negative regulation of smooth muscle cell proliferation	0.01678	0.13752	0	0	4	54
GO:0015658 branched-chain amino acid transmembrane transporter ac	2	0.01687	0.585	0.182	Molecular	GO:00156	branched-chain amino acid transmembrane transporter activity	0.01687	0.15736	0	0	2	11
GO:0016595 glutamate binding	2	0.01687	0.585	0.182	Molecular	GO:00165	glutamate binding	0.01687	0.15736	0	0	2	11
GO:0022889 serine transmembrane transporter activity	2	0.01687	0.585	0.182	Molecular	GO:00228	serine transmembrane transporter activity	0.01687	0.15736	0	0	2	11
GO:0032052 bile acid binding	2	0.01687	0.585	0.182	Molecular	GO:00320	bile acid binding	0.01687	0.15736	0	0	2	11
GO:0097493 structural molecule activity conferring elasticity	2	0.01687	0.585	0.182	Molecular	GO:00974	structural molecule activity conferring elasticity	0.01687	0.15736	0	0	2	11
GO:0009792 embryo development ending in birth or egg hatching	21	0.01693	6.502	0.03	Biological	GO:00097	embryo development ending in birth or egg hatching	0.01693	0.13752	0	0	21	696
GO:0003401 axis elongation	3	0.01694	0.929	0.1	Biological	GO:00034	axis elongation	0.01694	0.13752	0	0	3	30
GO:0007176 regulation of epidermal growth factor-activated receptor ac	3	0.01694	0.929	0.1	Biological	GO:00071	regulation of epidermal growth factor-activated receptor activity	0.01694	0.13752	0	0	3	30
GO:0051647 nucleus localization	3	0.01694	0.929	0.1	Biological	GO:00516	nucleus localization	0.01694	0.13752	0	0	3	30
GO:0060219 camera-type eye photoreceptor cell differentiation	3	0.01694	0.929	0.1	Biological	GO:00602	camera-type eye photoreceptor cell differentiation	0.01694	0.13752	0	0	3	30
GO:0006919 activation of cysteine-type endopeptidase activity involved	5	0.01701	1.548	0.061	Biological	GO:00069	activation of cysteine-type endopeptidase activity involved in apoptoti	0.01701	0.13752	0	0	5	82
GO:0007422 peripheral nervous system development	5	0.01701	1.548	0.061	Biological	GO:00074	peripheral nervous system development	0.01701	0.13752	0	0	5	82
GO:0000003 reproduction	41	0.01702	12.693	0.026	Biological	GO:00000	reproduction	0.01702	0.13752	0	0	41	1604
GO:0050730 regulation of peptidyl-tyrosine phosphorylation	10	0.01716	3.096	0.04	Biological	GO:00507	regulation of peptidyl-tyrosine phosphorylation	0.01716	0.13752	0	0	10	251
GO:0030017 sarcomere	9	0.01722	2.671	0.041	Cellular C	GO:00300	sarcomere	0.01722	0.13058	0	0	9	217
GO:0033293 monocarboxylic acid binding	5	0.0173	1.462	0.062	Molecular	GO:00332	monocarboxylic acid binding	0.0173	0.15736	0	0	5	81
GO:0052547 regulation of peptidase activity	14	0.01735	4.334	0.034	Biological	GO:00525	regulation of peptidase activity	0.01735	0.13752	0	0	14	406
GO:0018958 phenol-containing compound metabolic process	6	0.01742	1.858	0.053	Biological	GO:00189	phenol-containing compound metabolic process	0.01742	0.13752	0	0	6	113
GO:0016310 phosphorylation	46	0.01758	14.241	0.025	Biological	GO:00163	phosphorylation	0.01758	0.13752	0	0	46	1845
GO:0003785 actin monomer binding	3	0.0177	0.877	0.1	Molecular	GO:00037	actin monomer binding	0.0177	0.15736	0	0	3	30
GO:0005217 intracellular ligand-gated ion channel activity	3	0.0177	0.877	0.1	Molecular	GO:00052	intracellular ligand-gated ion channel activity	0.0177	0.15736	0	0	3	30
GO:0015101 organic cation transmembrane transporter activity	3	0.0177	0.877	0.1	Molecular	GO:00151	organic cation transmembrane transporter activity	0.0177	0.15736	0	0	3	30
GO:0043495 protein-membrane adaptor activity	3	0.0177	0.877	0.1	Molecular	GO:00434	protein-membrane adaptor activity	0.0177	0.15736	0	0	3	30
GO:0009725 response to hormone	25	0.01771	7.74	0.029	Biological	GO:00097	response to hormone	0.01771	0.13752	0	0	25	874
GO:0032880 regulation of protein localization	25	0.01771	7.74	0.029	Biological	GO:00328	regulation of protein localization	0.01771	0.13752	0	0	25	874
GO:0005246 calcium channel regulator activity	4	0.01773	1.17	0.074	Molecular	GO:00052	calcium channel regulator activity	0.01773	0.15736	0	0	4	54
GO:0009628 response to abiotic stimulus	31	0.01774	9.598	0.027	Biological	GO:00096	response to abiotic stimulus	0.01774	0.13752	0	0	31	1144
GO:0050839 cell adhesion molecule binding	18	0.01777	5.263	0.032	Molecular	GO:00508	cell adhesion molecule binding	0.01777	0.15736	0	0	18	562
GO:0048678 response to axon injury	5	0.01784	1.548	0.06	Biological	GO:00486	response to axon injury	0.01784	0.13752	0	0	5	83
GO:0071229 cellular response to acid chemical	5	0.01784	1.548	0.06	Biological	GO:00712	cellular response to acid chemical	0.01784	0.13752	0	0	5	83
GO:0072028 nephron morphogenesis	5	0.01784	1.548	0.06	Biological	GO:00720	nephron morphogenesis	0.01784	0.13752	0	0	5	83
GO:0030225 macrophage differentiation	4	0.01784	1.238	0.073	Biological	GO:00302	macrophage differentiation	0.01784	0.13752	0	0	4	55
GO:0010810 regulation of cell-substrate adhesion	9	0.01787	2.786	0.042	Biological	GO:00108	regulation of cell-substrate adhesion	0.01787	0.13752	0	0	9	216
GO:0055065 metal ion homeostasis	21	0.01792	6.502	0.03	Biological	GO:00550	metal ion homeostasis	0.01792	0.13752	0	0	21	700
GO:0005595 collagen type XII trimer	1	0.01803	0.297	1	Cellular C	GO:00055	collagen type XII trimer	0.01803	0.13058	0	0	1	1
GO:0005596 collagen type XIV trimer	1	0.01803	0.297	1	Cellular C	GO:00055	collagen type XIV trimer	0.01803	0.13058	0	0	1	1
GO:0005597 collagen type XVI trimer	1	0.01803	0.297	1	Cellular C	GO:00055	collagen type XVI trimer	0.01803	0.13058	0	0	1	1
GO:0034423 autophagosome lumen	1	0.01803	0.297	1	Cellular C	GO:00344	autophagosome lumen	0.01803	0.13058	0	0	1	1
GO:0045298 tubulin complex	1	0.01803	0.297	1	Cellular C	GO:00452	tubulin complex	0.01803	0.13058	0	0	1	1
GO:0071665 gamma-catenin-TCF7L2 complex	1	0.01803	0.297	1	Cellular C	GO:00716	gamma-catenin-TCF7L2 complex	0.01803	0.13058	0	0	1	1
GO:0044106 cellular amine metabolic process	6	0.01811	1.858	0.053	Biological	GO:00441	cellular amine metabolic process	0.01811	0.13752	0	0	6	114
GO:0015171 amino acid transmembrane transporter activity	5	0.01815	1.462	0.061	Molecular	GO:00151	amino acid transmembrane transporter activity	0.01815	0.15736	0	0	5	82
GO:0003147 neural crest cell migration involved in heart formation	1	0.01823	0.31	1	Biological	GO:00031	neural crest cell migration involved in heart formation	0.01823	0.13752	0	0	1	1
GO:0003242 cardiac chamber ballooning	1	0.01823	0.31	1	Biological	GO:00032	cardiac chamber ballooning	0.01823	0.13752	0	0	1	1
GO:0003322 pancreatic A cell development	1	0.01823	0.31	1	Biological	GO:00033	pancreatic A cell development	0.01823	0.13752	0	0	1	1
GO:0008052 sensory organ boundary specification	1	0.01823	0.31	1	Biological	GO:00080	sensory organ boundary specification	0.01823	0.13752	0	0	1	1
GO:0009087 methionine catabolic process	1	0.01823	0.31	1	Biological	GO:00090	methionine catabolic process	0.01823	0.13752	0	0	1	1
GO:0009956 radial pattern formation	1	0.01823	0.31	1	Biological	GO:00099	radial pattern formation	0.01823	0.13752	0	0	1	1
GO:0010160 formation of animal organ boundary	1	0.01823	0.31	1	Biological	GO:00101	formation of animal organ boundary	0.01823	0.13752	0	0	1	1
GO:0019101 female somatic sex determination	1	0.01823	0.31	1	Biological	GO:00191	female somatic sex determination	0.01823	0.13752	0	0	1	1
GO:0019102 male somatic sex determination	1	0.01823	0.31	1	Biological	GO:00191	male somatic sex determination	0.01823	0.13752	0	0	1	1
GO:0019413 acetate biosynthetic process	1	0.01823	0.31	1	Biological	GO:00194	acetate biosynthetic process	0.01823	0.13752	0	0	1	1
GO:0019442 tryptophan catabolic process to acetyl-CoA	1	0.01823	0.31	1	Biological	GO:00194	tryptophan catabolic process to acetyl-CoA	0.01823	0.13752	0	0	1	1
GO:0019542 propionate biosynthetic process	1	0.01823	0.31	1	Biological	GO:00195	propionate biosynthetic process	0.01823	0.13752	0	0	1	1

GO:0021629 olfactory nerve structural organization	1	0.01823	0.31	1	Biological	GO:00216	olfactory nerve structural organization	0.01823	0.13752	0	0	1	1
GO:0021905 forebrain-midbrain boundary formation	1	0.01823	0.31	1	Biological	GO:00219	forebrain-midbrain boundary formation	0.01823	0.13752	0	0	1	1
GO:0021919 BMP signaling pathway involved in spinal cord dorsal/vent	1	0.01823	0.31	1	Biological	GO:00219	BMP signaling pathway involved in spinal cord dorsal/ventral patterni	0.01823	0.13752	0	0	1	1
GO:0030034 microvillar actin bundle assembly	1	0.01823	0.31	1	Biological	GO:00300	microvillar actin bundle assembly	0.01823	0.13752	0	0	1	1
GO:0032244 positive regulation of nucleoside transport	1	0.01823	0.31	1	Biological	GO:00322	positive regulation of nucleoside transport	0.01823	0.13752	0	0	1	1
GO:0032900 negative regulation of neurotrophin production	1	0.01823	0.31	1	Biological	GO:00329	negative regulation of neurotrophin production	0.01823	0.13752	0	0	1	1
GO:0033026 negative regulation of mast cell apoptotic process	1	0.01823	0.31	1	Biological	GO:00330	negative regulation of mast cell apoptotic process	0.01823	0.13752	0	0	1	1
GO:0035645 enteric smooth muscle cell differentiation	1	0.01823	0.31	1	Biological	GO:00356	enteric smooth muscle cell differentiation	0.01823	0.13752	0	0	1	1
GO:0038167 epidermal growth factor receptor signaling pathway via pos	1	0.01823	0.31	1	Biological	GO:00381	epidermal growth factor receptor signaling pathway via positive regul	0.01823	0.13752	0	0	1	1
GO:0038168 epidermal growth factor receptor signaling pathway via I-ka	1	0.01823	0.31	1	Biological	GO:00381	epidermal growth factor receptor signaling pathway via I-kappaB kina	0.01823	0.13752	0	0	1	1
GO:0042323 negative regulation of circadian sleep/wake cycle, non-REI	1	0.01823	0.31	1	Biological	GO:00423	negative regulation of circadian sleep/wake cycle, non-REM sleep	0.01823	0.13752	0	0	1	1
GO:0060514 prostate induction	1	0.01823	0.31	1	Biological	GO:00605	prostate induction	0.01823	0.13752	0	0	1	1
GO:0060515 prostate field specification	1	0.01823	0.31	1	Biological	GO:00605	prostate field specification	0.01823	0.13752	0	0	1	1
GO:0060517 epithelial cell proliferation involved in prostatic bud elongati	1	0.01823	0.31	1	Biological	GO:00605	epithelial cell proliferation involved in prostatic bud elongation	0.01823	0.13752	0	0	1	1
GO:0060520 activation of prostate induction by androgen receptor signa	1	0.01823	0.31	1	Biological	GO:00605	activation of prostate induction by androgen receptor signaling pathw	0.01823	0.13752	0	0	1	1
GO:0060611 mammary gland fat development	1	0.01823	0.31	1	Biological	GO:00606	mammary gland fat development	0.01823	0.13752	0	0	1	1
GO:0060721 regulation of spongiotrophoblast cell proliferation	1	0.01823	0.31	1	Biological	GO:00607	regulation of spongiotrophoblast cell proliferation	0.01823	0.13752	0	0	1	1
GO:0060723 regulation of cell proliferation involved in embryonic placen	1	0.01823	0.31	1	Biological	GO:00607	regulation of cell proliferation involved in embryonic placenta develop	0.01823	0.13752	0	0	1	1
GO:0060784 regulation of cell proliferation involved in tissue homeostas	1	0.01823	0.31	1	Biological	GO:00607	regulation of cell proliferation involved in tissue homeostasis	0.01823	0.13752	0	0	1	1
GO:0061193 taste bud development	1	0.01823	0.31	1	Biological	GO:00611	taste bud development	0.01823	0.13752	0	0	1	1
GO:0061864 basement membrane constituent secretion	1	0.01823	0.31	1	Biological	GO:00618	basement membrane constituent secretion	0.01823	0.13752	0	0	1	1
GO:0061865 polarized secretion of basement membrane proteins in epi	1	0.01823	0.31	1	Biological	GO:00618	polarized secretion of basement membrane proteins in epithelium	0.01823	0.13752	0	0	1	1
GO:0061872 hepatic stellate cell contraction	1	0.01823	0.31	1	Biological	GO:00618	hepatic stellate cell contraction	0.01823	0.13752	0	0	1	1
GO:0061873 regulation of hepatic stellate cell contraction	1	0.01823	0.31	1	Biological	GO:00618	regulation of hepatic stellate cell contraction	0.01823	0.13752	0	0	1	1
GO:0061874 positive regulation of hepatic stellate cell contraction	1	0.01823	0.31	1	Biological	GO:00618	positive regulation of hepatic stellate cell contraction	0.01823	0.13752	0	0	1	1
GO:0070208 protein heterotrimerization	1	0.01823	0.31	1	Biological	GO:00702	protein heterotrimerization	0.01823	0.13752	0	0	1	1
GO:0070981 L-asparagine biosynthetic process	1	0.01823	0.31	1	Biological	GO:00709	L-asparagine biosynthetic process	0.01823	0.13752	0	0	1	1
GO:0071831 intermediate-density lipoprotein particle clearance	1	0.01823	0.31	1	Biological	GO:00718	intermediate-density lipoprotein particle clearance	0.01823	0.13752	0	0	1	1
GO:0072303 positive regulation of glomerular metanephric mesangial ce	1	0.01823	0.31	1	Biological	GO:00723	positive regulation of glomerular metanephric mesangial cell proliferat	0.01823	0.13752	0	0	1	1
GO:0072308 negative regulation of metanephric nephron tubule epitheli	1	0.01823	0.31	1	Biological	GO:00723	negative regulation of metanephric nephron tubule epithelial cell differ	0.01823	0.13752	0	0	1	1
GO:0072347 response to anesthetic	1	0.01823	0.31	1	Biological	GO:00723	response to anesthetic	0.01823	0.13752	0	0	1	1
GO:0090731 cellular response to very-low-density lipoprotein particle sti	1	0.01823	0.31	1	Biological	GO:00907	cellular response to very-low-density lipoprotein particle stimulus	0.01823	0.13752	0	0	1	1
GO:0120116 glucagon processing	1	0.01823	0.31	1	Biological	GO:01201	glucagon processing	0.01823	0.13752	0	0	1	1
GO:0150200 regulation of transport across blood-brain barrier	1	0.01823	0.31	1	Biological	GO:01502	regulation of transport across blood-brain barrier	0.01823	0.13752	0	0	1	1
GO:0150201 positive regulation of transport across blood-brain barrier	1	0.01823	0.31	1	Biological	GO:01502	positive regulation of transport across blood-brain barrier	0.01823	0.13752	0	0	1	1
GO:1901202 negative regulation of extracellular matrix assembly	1	0.01823	0.31	1	Biological	GO:19012	negative regulation of extracellular matrix assembly	0.01823	0.13752	0	0	1	1
GO:1901875 positive regulation of post-translational protein modification	1	0.01823	0.31	1	Biological	GO:19018	positive regulation of post-translational protein modification	0.01823	0.13752	0	0	1	1
GO:1902228 positive regulation of macrophage colony-stimulating facto	1	0.01823	0.31	1	Biological	GO:19022	positive regulation of macrophage colony-stimulating factor signaling	0.01823	0.13752	0	0	1	1
GO:1903000 regulation of lipid transport across blood-brain barrier	1	0.01823	0.31	1	Biological	GO:19030	regulation of lipid transport across blood-brain barrier	0.01823	0.13752	0	0	1	1
GO:1903002 positive regulation of lipid transport across blood-brain bar	1	0.01823	0.31	1	Biological	GO:19030	positive regulation of lipid transport across blood-brain barrier	0.01823	0.13752	0	0	1	1
GO:1903701 substantia propria of cornea development	1	0.01823	0.31	1	Biological	GO:19037	substantia propria of cornea development	0.01823	0.13752	0	0	1	1
GO:1904205 negative regulation of skeletal muscle hypertrophy	1	0.01823	0.31	1	Biological	GO:19042	negative regulation of skeletal muscle hypertrophy	0.01823	0.13752	0	0	1	1
GO:1905070 anterior visceral endoderm cell migration	1	0.01823	0.31	1	Biological	GO:19050	anterior visceral endoderm cell migration	0.01823	0.13752	0	0	1	1
GO:1905687 regulation of diacylglycerol kinase activity	1	0.01823	0.31	1	Biological	GO:19056	regulation of diacylglycerol kinase activity	0.01823	0.13752	0	0	1	1
GO:1905689 positive regulation of diacylglycerol kinase activity	1	0.01823	0.31	1	Biological	GO:19056	positive regulation of diacylglycerol kinase activity	0.01823	0.13752	0	0	1	1
GO:1905853 regulation of heparan sulfate binding	1	0.01823	0.31	1	Biological	GO:19058	regulation of heparan sulfate binding	0.01823	0.13752	0	0	1	1
GO:1905855 positive regulation of heparan sulfate binding	1	0.01823	0.31	1	Biological	GO:19058	positive regulation of heparan sulfate binding	0.01823	0.13752	0	0	1	1
GO:1905858 regulation of heparan sulfate proteoglycan binding	1	0.01823	0.31	1	Biological	GO:19058	regulation of heparan sulfate proteoglycan binding	0.01823	0.13752	0	0	1	1
GO:1905860 positive regulation of heparan sulfate proteoglycan binding	1	0.01823	0.31	1	Biological	GO:19058	positive regulation of heparan sulfate proteoglycan binding	0.01823	0.13752	0	0	1	1
GO:1905890 regulation of cellular response to very-low-density lipoprote	1	0.01823	0.31	1	Biological	GO:19058	regulation of cellular response to very-low-density lipoprotein particle	0.01823	0.13752	0	0	1	1
GO:1990678 histone H4-K16 deacetylation	1	0.01823	0.31	1	Biological	GO:19906	histone H4-K16 deacetylation	0.01823	0.13752	0	0	1	1
GO:2000690 regulation of cardiac muscle cell myoblast differentiation	1	0.01823	0.31	1	Biological	GO:20006	regulation of cardiac muscle cell myoblast differentiation	0.01823	0.13752	0	0	1	1
GO:2000691 negative regulation of cardiac muscle cell myoblast differe	1	0.01823	0.31	1	Biological	GO:20006	negative regulation of cardiac muscle cell myoblast differentiation	0.01823	0.13752	0	0	1	1
GO:0003015 heart process	10	0.01848	3.096	0.039	Biological	GO:00030	heart process	0.01848	0.13813	0	0	10	254
GO:0007435 salivary gland morphogenesis	3	0.0185	0.929	0.097	Biological	GO:00074	salivary gland morphogenesis	0.0185	0.13813	0	0	3	31
GO:0010743 regulation of macrophage derived foam cell differentiation	3	0.0185	0.929	0.097	Biological	GO:00107	regulation of macrophage derived foam cell differentiation	0.0185	0.13813	0	0	3	31
GO:0032607 interferon-alpha production	3	0.0185	0.929	0.097	Biological	GO:00326	interferon-alpha production	0.0185	0.13813	0	0	3	31
GO:0032647 regulation of interferon-alpha production	3	0.0185	0.929	0.097	Biological	GO:00326	regulation of interferon-alpha production	0.0185	0.13813	0	0	3	31
GO:0051491 positive regulation of filopodium assembly	3	0.0185	0.929	0.097	Biological	GO:00514	positive regulation of filopodium assembly	0.0185	0.13813	0	0	3	31
GO:0097205 renal filtration	3	0.0185	0.929	0.097	Biological	GO:00972	renal filtration	0.0185	0.13813	0	0	3	31
GO:0003830 beta-1,4-mannosylglycoprotein 4-beta-N-acetylglucosamin	1	0.01853	0.292	1	Molecular	GO:00038	beta-1,4-mannosylglycoprotein 4-beta-N-acetylglucosaminyltransfera	0.01853	0.15736	0	0	1	1
GO:0008120 ceramide glucosyltransferase activity	1	0.01853	0.292	1	Molecular	GO:00081	ceramide glucosyltransferase activity	0.01853	0.15736	0	0	1	1
GO:0017172 cysteine dioxygenase activity	1	0.01853	0.292	1	Molecular	GO:00171	cysteine dioxygenase activity	0.01853	0.15736	0	0	1	1
GO:0030379 neurotensin receptor activity, non-G protein-coupled	1	0.01853	0.292	1	Molecular	GO:00303	neurotensin receptor activity, non-G protein-coupled	0.01853	0.15736	0	0	1	1
GO:0031078 histone deacetylase activity (H3-K14 specific)	1	0.01853	0.292	1	Molecular	GO:00310	histone deacetylase activity (H3-K14 specific)	0.01853	0.15736	0	0	1	1
GO:0031714 C5a anaphylatoxin chemotactic receptor binding	1	0.01853	0.292	1	Molecular	GO:00317	C5a anaphylatoxin chemotactic receptor binding	0.01853	0.15736	0	0	1	1
GO:0031715 C5L2 anaphylatoxin chemotactic receptor binding	1	0.01853	0.292	1	Molecular	GO:00317	C5L2 anaphylatoxin chemotactic receptor binding	0.01853	0.15736	0	0	1	1
GO:0031717 cannabinoid receptor binding	1	0.01853	0.292	1	Molecular	GO:00317	cannabinoid receptor binding	0.01853	0.15736	0	0	1	1
GO:0031718 type 1 cannabinoid receptor binding	1	0.01853	0.292	1	Molecular	GO:00317	type 1 cannabinoid receptor binding	0.01853	0.15736	0	0	1	1
GO:0031883 taste receptor binding	1	0.01853	0.292	1	Molecular	GO:00318	taste receptor binding	0.01853	0.15736	0	0	1	1
GO:0033744 L-methionine:thioredoxin-disulfide S-oxidoreductase activit	1	0.01853	0.292	1	Molecular	GO:00337	L-methionine:thioredoxin-disulfide S-oxidoreductase activity	0.01853	0.15736	0	0	1	1
GO:0036131 prostaglandin D2 11-ketoreductase activity	1	0.01853	0.292	1	Molecular	GO:00361	prostaglandin D2 11-ketoreductase activity	0.01853	0.15736	0	0	1	1
GO:0036456 L-methionine-(S)-S-oxide reductase activity	1	0.01853	0.292	1	Molecular	GO:00364	L-methionine-(S)-S-oxide reductase activity	0.01853	0.15736	0	0	1	1
GO:0038177 death receptor agonist activity	1	0.01853	0.292	1	Molecular	GO:00381	death receptor agonist activity	0.01853	0.15736	0	0	1	1
GO:0042943 D-amino acid transmembrane transporter activity	1	0.01853	0.292	1	Molecular	GO:00429	D-amino acid transmembrane transporter activity	0.01853	0.15736	0	0	1	1
GO:0045703 ketoreductase activity	1	0.01853	0.292	1	Molecular	GO:00457	ketoreductase activity	0.01853	0.15736	0	0	1	1
GO:0046911 metal chelating activity	1	0.01853	0.292	1	Molecular	GO:00469	metal chelating activity	0.01853	0.15736	0	0	1	1
GO:0050512 lactosylceramide 4-alpha-galactosyltransferase activity	1	0.01853	0.292	1	Molecular	GO:00505	lactosylceramide 4-alpha-galactosyltransferase activity	0.01853	0.15736	0	0	1	1
GO:0070119 ciliary neurotrophic factor binding	1	0.01853	0.292	1	Molecular	GO:00701	ciliary neurotrophic factor binding	0.01853	0.15736	0	0	1	1
GO:0070974 POU domain binding	1	0.01853	0.292	1	Molecular	GO:00709	POU domain binding	0.01853	0.15736	0	0	1	1
GO:0098695 inositol 1,4,5-trisphosphate receptor activity involved in reg	1	0.01853	0.292	1	Molecular	GO:00986	inositol 1,4,5-trisphosphate receptor activity involved in regulation of	0.01853	0.15736	0	0	1	1
GO:0099077 histone-dependent DNA binding	1	0.01853	0.292	1	Molecular	GO:00990	histone-dependent DNA binding	0.01853	0.15736	0	0	1	1
GO:0099130 estrogen binding	1	0.01853	0.292	1	Molecular	GO:00991	estrogen binding	0.01853	0.15736	0	0	1	1
GO:0099609 microtubule lateral binding	1	0.01853	0.292	1	Molecular	GO:00996	microtubule lateral binding	0.01853	0.15736	0	0	1	1
GO:0102769 dihydroceramide glucosyltransferase activity	1	0.01853	0.292	1	Molecular	GO:01027	dihydroceramide glucosyltransferase activity	0.01853	0.15736	0	0	1	1
GO:0140010 D-aspartate transmembrane transporter activity	1	0.01853	0.292	1	Molecular	GO:01400	D-aspartate transmembrane transporter activity	0.01853	0.15736	0	0	1	1
GO:0048661 positive regulation of smooth muscle cell proliferation	5	0.01869	1.548	0.06	Biological	GO:00486	positive regulation of smooth muscle cell proliferation	0.01869	0.13913	0	0	5	84
GO:0060538 skeletal muscle organ development	8	0.01869	2.477	0.044	Biological	GO:00605	skeletal muscle organ development	0.01869	0.13913	0	0	8	182
GO:0043534 blood vessel endothelial cell migration	6	0.01883	1.858	0.052	Biological	GO:00435	blood vessel endothelial cell migration	0.01883	0.13992	0	0	6	115
GO:0031102 neuron projection regeneration	4	0.01895	1.238	0.071	Biological	GO:00311	neuron projection regeneration	0.01895	0.14038	0	0	4	56
GO:0051703 biological process involved in intraspecies interaction betw	4	0.01895	1.238	0.071	Biological	GO:00517	biological process involved in intraspecies interaction between organi	0.01895	0.14038	0	0	4	56
GO:0001527 microfibril	2	0.01898	0.593	0.167	Cellular C	GO:00015	microfibril	0.01898	0.13058	0	0	2	12
GO:0005583 fibrillar collagen trimer	2	0.01898	0.593	0.167	Cellular C	GO:00055	fibrillar collagen trimer	0.01898	0.13058	0	0	2	12
GO:0005614 interstitial matrix	2	0.01898	0.593	0.167	Cellular C	GO:00056	interstitial matrix	0.01898	0.13058	0	0	2	12
GO:0098643 banded collagen fibril	2	0.01898	0.593	0.167	Cellular C	GO:00986	banded collagen fibril	0.01898	0.13058	0	0	2	12
GO:0005576 extracellular region	94	0.01904	27.893	0.022	Cellular C	GO:00055	extracellular region	0.01904	0.13058	0	0	94	4292
GO:1902107 positive regulation of leukocyte differentiation	8	0.01925	2.477	0.044	Biological	GO:19021	positive regulation of leukocyte differentiation	0.01925	0.1413	0	0	8	183
GO:1903708 positive regulation of hemopoiesis	8	0.01925	2.477	0.044	Biological	GO:19037	positive regulation of hemopoiesis	0.01925	0.1413	0	0	8	183
GO:0001968 fibronectin binding	3	0.01933	0.877	0.097	Molecular	GO:00019	fibronectin binding	0.01933	0.15944	0	0	3	31
GO:0016628 oxidoreductase activity, acting on the CH-CH group of don	3	0.01933	0.877	0.097	Molecular	GO:00166	oxidoreductase activity, acting on the CH-CH group of donors, NAD	0.01933	0.15944	0	0	3	31
GO:0051721 protein phosphatase 2A binding	3	0.01933	0.877	0.097	Molecular	GO:00517	protein phosphatase 2A binding	0.01933	0.15944	0	0	3	31
GO:0008300 isoprenoid catabolic process	2	0.01938	0.619	0.167	Biological	GO:00083	isoprenoid catabolic process	0.01938	0.1413	0	0	2	12
GO:0021527 spinal cord association neuron differentiation	2	0.01938	0.619	0.167	Biological	GO:00215	spinal cord association neuron differentiation	0.01938	0.1413	0	0	2	12
GO:0021794 thalamus development	2	0.01938	0.619	0.167	Biological	GO:00217	thalamus development	0.01938	0.1413	0	0	2	12
GO:0032329 serine transport	2	0.01938	0.619	0.167	Biological	GO:00323	serine transport	0.01938	0.1413	0	0	2	12
GO:0038180 nerve growth factor signaling pathway	2	0.01938	0.619	0.167	Biological	GO:00381	nerve growth factor signaling pathway	0.01938	0.1413	0	0	2	12
GO:0043589 skin morphogenesis	2	0.01938	0.619	0.167	Biological	GO:00435	skin morphogenesis	0.01938	0.1413	0	0	2	12
GO:0061303 cornea development in camera-type eye	2	0.01938	0.619	0.167	Biological	GO:00613	cornea development in camera-type eye	0.01938	0.1413	0	0	2	12
GO:0061309 cardiac neural crest cell development involved in outflow tr	2	0.01938	0.619	0.167	Biological	GO:00613	cardiac neural crest cell development involved in outflow tract morph	0.01938	0.1413	0	0	2	12
GO:1905907 negative regulation of amyloid fibril formation	2	0.01938	0.619	0.167	Biological	GO:19059	negative regulation of amyloid fibril formation	0.01938	0.1413	0	0	2	12
GO:0036211 protein modification process	82	0.01948	25.387	0.023	Biological	GO:00362	protein modification process	0.01948	0.14178	0	0	82	3640
GO:0035556 intracellular signal transduction	62	0.0195	19.195	0.024	Biological	GO:00355	intracellular signal transduction	0.0195	0.14178	0	0	62	2637
GO:0009605 response to external stimulus	66	0.01958	20.433	0.023	Biological	GO:00096	response to external stimulus	0.01958	0.14215	0	0	66	2836
GO:0007420 brain development	23	0.01993	7.121	0.029	Biological	GO:00074	brain development	0.01993	0.14446	0	0	23	795
GO:0001540 amyloid-beta binding	5	0.01994	1.462	0.06	Molecular	GO:00015	amyloid-beta binding	0.01994	0.16034	0	0	5	84
GO:0004857 enzyme inhibitor activity	5	0.01994	1.462	0.06	Molecular	GO:00048	enzyme inhibitor activity	0.01994	0.16034	0	0	5	84
GO:0004032 alditol:NADP+ 1-oxidoreductase activity	2	0.02	0.585	0.167	Molecular	GO:00040	alditol:NADP+ 1-oxidoreductase activity	0.02	0.16034	0	0	2	12
GO:0000096 sulfur amino acid metabolic process	3	0.02015	0.929	0.094	Biological	GO:00000	sulfur amino acid metabolic process	0.02015	0.14562	0	0	3	32
GO:0001975 response to amphetamine	3	0.02015	0.929	0.094	Biological	GO:00019	response to amphetamine	0.02015	0.14562	0	0	3	32
GO:0002687 positive regulation of leukocyte migration	7	0.02022	2.167	0.047	Biological	GO:00026	positive regulation of leukocyte migration	0.02022	0.14591	0	0	7	150
GO:0048738 cardiac muscle tissue development	9	0.02039	2.786	0.041	Biological	GO:00487	cardiac muscle tissue development	0.02039	0.14696	0	0	9	221
GO:0055074 calcium ion homeostasis	16	0.02051	4.954	0.032	Biological	GO:00550	calcium ion homeostasis	0.02051	0.14761	0	0	16	497
GO:0005262 calcium channel activity	6	0.02104	1.754	0.052	Molecular	GO:00052	calcium channel activity	0.02104	0.1655	0	0	6	116
GO:0005164 tumor necrosis factor receptor binding	3	0.02105	0.877	0.094	Molecular	GO:00051	tumor necrosis factor receptor binding	0.02105	0.1655	0	0	3	32
GO:0002526 acute inflammatory response	6	0.02109	1.858	0.051	Biological	GO:00025	acute inflammatory response	0.02109	0.1513	0	0	6	118
GO:0007229 integrin-mediated signaling pathway	6	0.02109	1.858	0.051	Biological	GO:00072	integrin-mediated signaling pathway	0.02109	0.1513	0	0	6	118
GO:0050840 extracellular matrix binding	4	0.02121	1.17	0.07	Molecular	GO:00508	extracellular matrix binding	0.02121	0.1655	0	0	4	57
GO:0036293 response to decreased oxygen levels	11	0.02126	3.406	0.037	Biological	GO:00362	response to decreased oxygen levels	0.02126	0.15203	0	0	11	298
GO:0033619 membrane protein proteolysis	4	0.02128	1.238	0.069	Biological	GO:00336	membrane protein proteolysis	0.02128	0.15203	0	0	4	58
GO:0072132 mesenchyme morphogenesis	4	0.02128	1.238	0.069	Biological	GO:00721	mesenchyme morphogenesis	0.02128	0.15203	0	0	4	58
GO:0019935 cyclic-nucleotide-mediated signaling	5	0.02142	1.548	0.057	Biological	GO:00199	cyclic-nucleotide-mediated signaling	0.02142	0.15281	0	0	5	87
GO:0008104 protein localization	59	0.02178	18.266	0.024	Biological	GO:00081	protein localization	0.02178	0.15518	0	0	59	2504
GO:0035909 aorta morphogenesis	3	0.02187	0.929	0.091	Biological	GO:00359	aorta morphogenesis	0.02187	0.1554	0	0	3	33
GO:0009308 amine metabolic process	6	0.02188	1.858	0.05	Biological	GO:00093	amine metabolic process	0.02188	0.1554	0	0	6	119
GO:0040008 regulation of growth	19	0.02191	5.882	0.03	Biological	GO:00400	regulation of growth	0.02191	0.1554	0	0	19	628
GO:0050920 regulation of chemotaxis	9	0.02203	2.786	0.04	Biological	GO:00509	regulation of chemotaxis	0.02203	0.15592	0	0	9	224
GO:0001934 positive regulation of protein phosphorylation	21	0.02204	6.502	0.029	Biological	GO:00019	positive regulation of protein phosphorylation	0.02204	0.15592	0	0	21	715
GO:0034362 low-density lipoprotein particle	2	0.02216	0.593	0.154	Cellular C	GO:00343	low-density lipoprotein particle	0.02216	0.14986	0	0	2	13
GO:0043408 regulation of MAPK cascade	20	0.02218	6.192	0.03	Biological	GO:00434	regulation of MAPK cascade	0.02218	0.15595	0	0	20	672
GO:0001890 placenta development	7	0.02226	2.167	0.046	Biological	GO:00018	placenta development	0.02226	0.15595	0	0	7	153
GO:0016049 cell growth	16	0.02227	4.954	0.032	Biological	GO:00160	cell growth	0.02227	0.15595	0	0	16	502
GO:0021675 nerve development	5	0.02238	1.548	0.057	Biological	GO:00216	nerve development	0.02238	0.15595	0	0	5	88
GO:0060291 long-term synaptic potentiation	5	0.02238	1.548	0.057	Biological	GO:00602	long-term synaptic potentiation	0.02238	0.15595	0	0	5	88
GO:0098982 GABA-ergic synapse	5	0.02241	1.484	0.056	Cellular C	GO:00989	GABA-ergic synapse	0.02241	0.14986	0	0	5	89
GO:0090311 regulation of protein deacetylation	4	0.02251	1.238	0.068	Biological	GO:00903	regulation of protein deacetylation	0.02251	0.15595	0	0	4	59
GO:0003096 renal sodium ion transport	2	0.02263	0.619	0.154	Biological	GO:00030	renal sodium ion transport	0.02263	0.15595	0	0	2	13
GO:0021781 glial cell fate commitment	2	0.02263	0.619	0.154	Biological	GO:00217	glial cell fate commitment	0.02263	0.15595	0	0	2	13
GO:0021978 telencephalon regionalization	2	0.02263	0.619	0.154	Biological	GO:00219	telencephalon regionalization	0.02263	0.15595	0	0	2	13
GO:0043568 positive regulation of insulin-like growth factor receptor sig	2	0.02263	0.619	0.154	Biological	GO:00435	positive regulation of insulin-like growth factor receptor signaling path	0.02263	0.15595	0	0	2	13
GO:0043951 negative regulation of cAMP-mediated signaling	2	0.02263	0.619	0.154	Biological	GO:00439	negative regulation of cAMP-mediated signaling	0.02263	0.15595	0	0	2	13
GO:0045741 positive regulation of epidermal growth factor-activated rec	2	0.02263	0.619	0.154	Biological	GO:00457	positive regulation of epidermal growth factor-activated receptor activ	0.02263	0.15595	0	0	2	13
GO:0060707 trophoblast giant cell differentiation	2	0.02263	0.619	0.154	Biological	GO:00607	trophoblast giant cell differentiation	0.02263	0.15595	0	0	2	13

GO:0060788 ectodermal placode formation	2	0.02263	0.619	0.154	Biological	GO:00601	ectodermal placode formation	0.02263	0.15595	0	0	2	13
GO:0070254 mucus secretion	2	0.02263	0.619	0.154	Biological	GO:00702	mucus secretion	0.02263	0.15595	0	0	2	13
GO:0071697 ectodermal placode morphogenesis	2	0.02263	0.619	0.154	Biological	GO:00716	ectodermal placode morphogenesis	0.02263	0.15595	0	0	2	13
GO:1904754 positive regulation of vascular associated smooth muscle c	2	0.02263	0.619	0.154	Biological	GO:19047	positive regulation of vascular associated smooth muscle cell migratio	0.02263	0.15595	0	0	2	13
GO:1905209 positive regulation of cardiocyte differentiation	2	0.02263	0.619	0.154	Biological	GO:19052	positive regulation of cardiocyte differentiation	0.02263	0.15595	0	0	2	13
GO:0001932 regulation of protein phosphorylation	29	0.02264	8.978	0.027	Biological	GO:00019	regulation of protein phosphorylation	0.02264	0.15595	0	0	29	1075
GO:0032589 neuron projection membrane	4	0.02294	1.187	0.067	Cellular C	GO:00325	neuron projection membrane	0.02294	0.15153	0	0	4	60
GO:0001501 skeletal system development	17	0.02298	5.263	0.031	Biological	GO:00015	skeletal system development	0.02298	0.15809	0	0	17	546
GO:0006464 cellular protein modification process	81	0.02333	25.077	0.022	Biological	GO:00064	cellular protein modification process	0.02333	0.15989	0	0	81	3618
GO:0048871 multicellular organismal homeostasis	17	0.02334	5.263	0.031	Biological	GO:00488	multicellular organismal homeostasis	0.02334	0.15989	0	0	17	547
GO:0008417 fucosyltransferase activity	2	0.02335	0.585	0.154	Molecular	GO:00084	fucosyltransferase activity	0.02335	0.17892	0	0	2	13
GO:0031994 insulin-like growth factor I binding	2	0.02335	0.585	0.154	Molecular	GO:00319	insulin-like growth factor I binding	0.02335	0.17892	0	0	2	13
GO:0007589 body fluid secretion	5	0.02337	1.548	0.056	Biological	GO:00075	body fluid secretion	0.02337	0.15989	0	0	5	89
GO:0043535 regulation of blood vessel endothelial cell migration	5	0.02337	1.548	0.056	Biological	GO:00435	regulation of blood vessel endothelial cell migration	0.02337	0.15989	0	0	5	89
GO:0031345 negative regulation of cell projection organization	8	0.02348	2.477	0.042	Biological	GO:00313	negative regulation of cell projection organization	0.02348	0.15997	0	0	8	190
GO:0006688 glycosphingolipid biosynthetic process	3	0.02367	0.929	0.088	Biological	GO:00066	glycosphingolipid biosynthetic process	0.02367	0.15997	0	0	3	34
GO:0010765 positive regulation of sodium ion transport	3	0.02367	0.929	0.088	Biological	GO:00107	positive regulation of sodium ion transport	0.02367	0.15997	0	0	3	34
GO:0021772 olfactory bulb development	3	0.02367	0.929	0.088	Biological	GO:00217	olfactory bulb development	0.02367	0.15997	0	0	3	34
GO:0034694 response to prostaglandin	3	0.02367	0.929	0.088	Biological	GO:00346	response to prostaglandin	0.02367	0.15997	0	0	3	34
GO:0042573 retinoic acid metabolic process	3	0.02367	0.929	0.088	Biological	GO:00425	retinoic acid metabolic process	0.02367	0.15997	0	0	3	34
GO:0043537 negative regulation of blood vessel endothelial cell migratio	3	0.02367	0.929	0.088	Biological	GO:00435	negative regulation of blood vessel endothelial cell migration	0.02367	0.15997	0	0	3	34
GO:0070633 transepithelial transport	3	0.02367	0.929	0.088	Biological	GO:00706	transepithelial transport	0.02367	0.15997	0	0	3	34
GO:0090183 regulation of kidney development	3	0.02367	0.929	0.088	Biological	GO:00901	regulation of kidney development	0.02367	0.15997	0	0	3	34
GO:0051338 regulation of transferase activity	24	0.02396	7.43	0.028	Biological	GO:00513	regulation of transferase activity	0.02396	0.16171	0	0	24	854
GO:0031099 regeneration	8	0.02414	2.477	0.042	Biological	GO:00310	regeneration	0.02414	0.16267	0	0	8	191
GO:0042802 identical protein binding	52	0.02415	15.205	0.024	Molecular	GO:00428	identical protein binding	0.02415	0.18346	0	0	52	2136
GO:0048872 homeostasis of number of cells	11	0.02417	3.406	0.036	Biological	GO:00488	homeostasis of number of cells	0.02417	0.16269	0	0	11	304
GO:0045944 positive regulation of transcription by RNA polymerase II	33	0.02423	10.217	0.026	Biological	GO:00459	positive regulation of transcription by RNA polymerase II	0.02423	0.16283	0	0	33	1266
GO:0002685 regulation of leukocyte migration	9	0.02435	2.786	0.039	Biological	GO:00026	regulation of leukocyte migration	0.02435	0.16301	0	0	9	228
GO:0006486 protein glycosylation	9	0.02435	2.786	0.039	Biological	GO:00064	protein glycosylation	0.02435	0.16301	0	0	9	228
GO:0043413 macromolecule glycosylation	9	0.02435	2.786	0.039	Biological	GO:00434	macromolecule glycosylation	0.02435	0.16301	0	0	9	228
GO:0005154 epidermal growth factor receptor binding	3	0.02471	0.877	0.088	Molecular	GO:00051	epidermal growth factor receptor binding	0.02471	0.18586	0	0	3	34
GO:0042562 hormone binding	5	0.0249	1.462	0.056	Molecular	GO:00425	hormone binding	0.0249	0.18586	0	0	5	89
GO:0048588 developmental cell growth	9	0.02496	2.786	0.039	Biological	GO:00485	developmental cell growth	0.02496	0.16685	0	0	9	229
GO:0051145 smooth muscle cell differentiation	4	0.0251	1.238	0.066	Biological	GO:00511	smooth muscle cell differentiation	0.0251	0.16755	0	0	4	61
GO:0009925 basal plasma membrane	10	0.0251	2.967	0.037	Cellular C	GO:00099	basal plasma membrane	0.0251	0.16374	0	0	10	270
GO:0140096 catalytic activity, acting on a protein	56	0.02514	16.374	0.024	Molecular	GO:01400	catalytic activity, acting on a protein	0.02514	0.18607	0	0	56	2334
GO:0008217 regulation of blood pressure	8	0.02548	2.477	0.041	Biological	GO:00082	regulation of blood pressure	0.02548	0.16939	0	0	8	193
GO:0019934 cGMP-mediated signaling	3	0.02555	0.929	0.086	Biological	GO:00199	cGMP-mediated signaling	0.02555	0.16939	0	0	3	35
GO:0035633 maintenance of blood-brain barrier	3	0.02555	0.929	0.086	Biological	GO:00356	maintenance of blood-brain barrier	0.02555	0.16939	0	0	3	35
GO:0048665 neuron fate specification	3	0.02555	0.929	0.086	Biological	GO:00486	neuron fate specification	0.02555	0.16939	0	0	3	35
GO:0070528 protein kinase C signaling	3	0.02555	0.929	0.086	Biological	GO:00705	protein kinase C signaling	0.02555	0.16939	0	0	3	35
GO:0043218 compact myelin	2	0.02555	0.593	0.143	Cellular C	GO:00432	compact myelin	0.02555	0.1647	0	0	2	14
GO:0051651 maintenance of location in cell	9	0.02557	2.786	0.039	Biological	GO:00516	maintenance of location in cell	0.02557	0.16939	0	0	9	230
GO:0008081 phosphoric diester hydrolase activity	5	0.02597	1.462	0.056	Molecular	GO:00080	phosphoric diester hydrolase activity	0.02597	0.19062	0	0	5	90
GO:0098978 glutamatergic synapse	14	0.026	4.154	0.032	Cellular C	GO:00989	glutamatergic synapse	0.026	0.16557	0	0	14	433
GO:0003084 positive regulation of systemic arterial blood pressure	2	0.02609	0.619	0.143	Biological	GO:00030	positive regulation of systemic arterial blood pressure	0.02609	0.17077	0	0	2	14
GO:0016322 neuron remodeling	2	0.02609	0.619	0.143	Biological	GO:00163	neuron remodeling	0.02609	0.17077	0	0	2	14
GO:0030238 male sex determination	2	0.02609	0.619	0.143	Biological	GO:00302	male sex determination	0.02609	0.17077	0	0	2	14
GO:0061029 eyelid development in camera-type eye	2	0.02609	0.619	0.143	Biological	GO:00610	eyelid development in camera-type eye	0.02609	0.17077	0	0	2	14
GO:0070141 response to UV-A	2	0.02609	0.619	0.143	Biological	GO:00701	response to UV-A	0.02609	0.17077	0	0	2	14
GO:0071696 ectodermal placode development	2	0.02609	0.619	0.143	Biological	GO:00716	ectodermal placode development	0.02609	0.17077	0	0	2	14
GO:0106070 regulation of adenylate cyclase-activating G protein-couple	2	0.02609	0.619	0.143	Biological	GO:01060	regulation of adenylate cyclase-activating G protein-coupled receptor	0.02609	0.17077	0	0	2	14
GO:1900452 regulation of long-term synaptic depression	2	0.02609	0.619	0.143	Biological	GO:19004	regulation of long-term synaptic depression	0.02609	0.17077	0	0	2	14
GO:1901722 regulation of cell proliferation involved in kidney developme	2	0.02609	0.619	0.143	Biological	GO:19017	regulation of cell proliferation involved in kidney development	0.02609	0.17077	0	0	2	14
GO:0030029 actin filament-based process	23	0.0263	7.121	0.028	Biological	GO:00300	actin filament-based process	0.0263	0.17189	0	0	23	817
GO:0060021 roof of mouth development	5	0.02651	1.548	0.054	Biological	GO:00600	roof of mouth development	0.02651	0.17308	0	0	5	92
GO:0008195 phosphatidate phosphatase activity	2	0.02691	0.585	0.143	Molecular	GO:00081	phosphatidate phosphatase activity	0.02691	0.191	0	0	2	14
GO:0042577 lipid phosphatase activity	2	0.02691	0.585	0.143	Molecular	GO:00425	lipid phosphatase activity	0.02691	0.191	0	0	2	14
GO:0044594 17-beta-hydroxysteroid dehydrogenase (NAD+) activity	2	0.02691	0.585	0.143	Molecular	GO:00445	17-beta-hydroxysteroid dehydrogenase (NAD+) activity	0.02691	0.191	0	0	2	14
GO:0071837 HMG box domain binding	2	0.02691	0.585	0.143	Molecular	GO:00718	HMG box domain binding	0.02691	0.191	0	0	2	14
GO:0002688 regulation of leukocyte chemotaxis	6	0.02705	1.858	0.048	Biological	GO:00026	regulation of leukocyte chemotaxis	0.02705	0.17614	0	0	6	125
GO:0043280 positive regulation of cysteine-type endopeptidase activity	6	0.02705	1.858	0.048	Biological	GO:00432	positive regulation of cysteine-type endopeptidase activity involved in	0.02705	0.17614	0	0	6	125
GO:0007616 long-term memory	3	0.02751	0.929	0.083	Biological	GO:00076	long-term memory	0.02751	0.17771	0	0	3	36
GO:0021988 olfactory lobe development	3	0.02751	0.929	0.083	Biological	GO:00219	olfactory lobe development	0.02751	0.17771	0	0	3	36
GO:0031128 developmental induction	3	0.02751	0.929	0.083	Biological	GO:00311	developmental induction	0.02751	0.17771	0	0	3	36
GO:0042462 eye photoreceptor cell development	3	0.02751	0.929	0.083	Biological	GO:00424	eye photoreceptor cell development	0.02751	0.17771	0	0	3	36
GO:1904707 positive regulation of vascular associated smooth muscle c	3	0.02751	0.929	0.083	Biological	GO:19047	positive regulation of vascular associated smooth muscle cell prolifer	0.02751	0.17771	0	0	3	36
GO:1905144 response to acetylcholine	3	0.02751	0.929	0.083	Biological	GO:19051	response to acetylcholine	0.02751	0.17771	0	0	3	36
GO:0045664 regulation of neuron differentiation	8	0.0276	2.477	0.041	Biological	GO:00456	regulation of neuron differentiation	0.0276	0.17773	0	0	8	196
GO:0030901 midbrain development	5	0.02762	1.548	0.054	Biological	GO:00309	midbrain development	0.02762	0.17773	0	0	5	93
GO:0099072 regulation of postsynaptic membrane neurotransmitter rec	5	0.02762	1.548	0.054	Biological	GO:00990	regulation of postsynaptic membrane neurotransmitter receptor levels	0.02762	0.17773	0	0	5	93
GO:0048878 chemical homeostasis	32	0.0277	9.907	0.026	Biological	GO:00488	chemical homeostasis	0.0277	0.17802	0	0	32	1233
GO:0003014 renal system process	6	0.02799	1.858	0.048	Biological	GO:00030	renal system process	0.02799	0.17965	0	0	6	126
GO:0005178 integrin binding	7	0.02809	2.047	0.044	Molecular	GO:00051	integrin binding	0.02809	0.19512	0	0	7	158
GO:0001837 epithelial to mesenchymal transition	7	0.02838	2.167	0.043	Biological	GO:00018	epithelial to mesenchymal transition	0.02838	0.18168	0	0	7	161
GO:0007605 sensory perception of sound	7	0.02838	2.167	0.043	Biological	GO:00076	sensory perception of sound	0.02838	0.18168	0	0	7	161
GO:1990778 protein localization to cell periphery	12	0.02854	3.715	0.034	Biological	GO:19907	protein localization to cell periphery	0.02854	0.18249	0	0	12	352
GO:0032436 positive regulation of proteasomal ubiquitin-dependent prot	5	0.02875	1.548	0.053	Biological	GO:00324	positive regulation of proteasomal ubiquitin-dependent protein catabo	0.02875	0.18313	0	0	5	94
GO:0032602 chemokine production	5	0.02875	1.548	0.053	Biological	GO:00326	chemokine production	0.02875	0.18313	0	0	5	94
GO:0032642 regulation of chemokine production	5	0.02875	1.548	0.053	Biological	GO:00326	regulation of chemokine production	0.02875	0.18313	0	0	5	94
GO:0042175 nuclear outer membrane-endoplasmic reticulum membran	31	0.02876	9.199	0.026	Cellular C	GO:00421	nuclear outer membrane-endoplasmic reticulum membrane network	0.02876	0.18099	0	0	31	1203
GO:0010001 glial cell differentiation	9	0.02883	2.786	0.038	Biological	GO:00100	glial cell differentiation	0.02883	0.18338	0	0	9	235
GO:0014902 myotube differentiation	6	0.02894	1.858	0.047	Biological	GO:00149	myotube differentiation	0.02894	0.18366	0	0	6	127
GO:0051209 release of sequestered calcium ion into cytosol	6	0.02894	1.858	0.047	Biological	GO:00512	release of sequestered calcium ion into cytosol	0.02894	0.18366	0	0	6	127
GO:0030199 collagen fibril organization	4	0.0293	1.238	0.062	Biological	GO:00301	collagen fibril organization	0.0293	0.18447	0	0	4	64
GO:0060341 regulation of cellular localization	23	0.02931	7.121	0.028	Biological	GO:00603	regulation of cellular localization	0.02931	0.18447	0	0	23	826
GO:0001941 postsynaptic membrane organization	3	0.02954	0.929	0.081	Biological	GO:00019	postsynaptic membrane organization	0.02954	0.18447	0	0	3	37
GO:0097242 amyloid-beta clearance	3	0.02954	0.929	0.081	Biological	GO:00972	amyloid-beta clearance	0.02954	0.18447	0	0	3	37
GO:1905332 positive regulation of morphogenesis of an epithelium	3	0.02954	0.929	0.081	Biological	GO:19053	positive regulation of morphogenesis of an epithelium	0.02954	0.18447	0	0	3	37
GO:0006874 cellular calcium ion homeostasis	15	0.02969	4.644	0.031	Biological	GO:00068	cellular calcium ion homeostasis	0.02969	0.18447	0	0	15	478
GO:0014029 neural crest formation	2	0.02975	0.619	0.133	Biological	GO:00140	neural crest formation	0.02975	0.18447	0	0	2	15
GO:0043584 nose development	2	0.02975	0.619	0.133	Biological	GO:00435	nose development	0.02975	0.18447	0	0	2	15
GO:0048484 enteric nervous system development	2	0.02975	0.619	0.133	Biological	GO:00484	enteric nervous system development	0.02975	0.18447	0	0	2	15
GO:0061307 cardiac neural crest cell differentiation involved in heart de	2	0.02975	0.619	0.133	Biological	GO:00613	cardiac neural crest cell differentiation involved in heart development	0.02975	0.18447	0	0	2	15
GO:0061308 cardiac neural crest cell development involved in heart dev	2	0.02975	0.619	0.133	Biological	GO:00613	cardiac neural crest cell development involved in heart development	0.02975	0.18447	0	0	2	15
GO:0071599 otic vesicle development	2	0.02975	0.619	0.133	Biological	GO:00715	otic vesicle development	0.02975	0.18447	0	0	2	15
GO:0072075 metanephric mesenchyme development	2	0.02975	0.619	0.133	Biological	GO:00720	metanephric mesenchyme development	0.02975	0.18447	0	0	2	15
GO:0072160 nephron tubule epithelial cell differentiation	2	0.02975	0.619	0.133	Biological	GO:00721	nephron tubule epithelial cell differentiation	0.02975	0.18447	0	0	2	15
GO:0072283 metanephric renal vesicle morphogenesis	2	0.02975	0.619	0.133	Biological	GO:00722	metanephric renal vesicle morphogenesis	0.02975	0.18447	0	0	2	15
GO:0097152 mesenchymal cell apoptotic process	2	0.02975	0.619	0.133	Biological	GO:00971	mesenchymal cell apoptotic process	0.02975	0.18447	0	0	2	15
GO:1901201 regulation of extracellular matrix assembly	2	0.02975	0.619	0.133	Biological	GO:19012	regulation of extracellular matrix assembly	0.02975	0.18447	0	0	2	15
GO:1904936 interneuron migration	2	0.02975	0.619	0.133	Biological	GO:19049	interneuron migration	0.02975	0.18447	0	0	2	15
GO:0050900 leukocyte migration	13	0.02977	4.025	0.033	Biological	GO:00509	leukocyte migration	0.02977	0.18447	0	0	13	395
GO:0001942 hair follicle development	5	0.02991	1.548	0.053	Biological	GO:00019	hair follicle development	0.02991	0.1848	0	0	5	95
GO:0051283 negative regulation of sequestering of calcium ion	6	0.02992	1.858	0.047	Biological	GO:00512	negative regulation of sequestering of calcium ion	0.02992	0.1848	0	0	6	128
GO:0055080 cation homeostasis	22	0.02994	6.811	0.028	Biological	GO:00550	cation homeostasis	0.02994	0.1848	0	0	22	783
GO:0018193 peptidyl-amino acid modification	32	0.03052	9.907	0.026	Biological	GO:00181	peptidyl-amino acid modification	0.03052	0.18597	0	0	32	1243
GO:0004089 carbonate dehydratase activity	2	0.03067	0.585	0.133	Molecular	GO:00040	carbonate dehydratase activity	0.03067	0.19512	0	0	2	15
GO:0034358 plasma lipoprotein particle	3	0.03076	0.89	0.079	Cellular C	GO:00343	plasma lipoprotein particle	0.03076	0.18918	0	0	3	38
GO:1990777 lipoprotein particle	3	0.03076	0.89	0.079	Cellular C	GO:19907	lipoprotein particle	0.03076	0.18918	0	0	3	38
GO:0003006 developmental process involved in reproduction	28	0.03079	8.669	0.026	Biological	GO:00030	developmental process involved in reproduction	0.03079	0.18597	0	0	28	1058
GO:0060760 positive regulation of response to cytokine stimulus	4	0.03079	1.238	0.062	Biological	GO:00607	positive regulation of response to cytokine stimulus	0.03079	0.18597	0	0	4	65
GO:0035265 organ growth	7	0.03094	2.167	0.043	Biological	GO:00352	organ growth	0.03094	0.18597	0	0	7	164
GO:0060255 regulation of macromolecule metabolic process	130	0.03094	40.248	0.021	Biological	GO:00602	regulation of macromolecule metabolic process	0.03094	0.18597	0	0	130	6227
GO:0034109 homotypic cell-cell adhesion	5	0.0311	1.548	0.052	Biological	GO:00341	homotypic cell-cell adhesion	0.0311	0.18597	0	0	5	96
GO:0022409 positive regulation of cell-cell adhesion	11	0.03148	3.406	0.035	Biological	GO:00224	positive regulation of cell-cell adhesion	0.03148	0.18597	0	0	11	317
GO:0008236 serine-type peptidase activity	8	0.03166	2.339	0.04	Molecular	GO:00082	serine-type peptidase activity	0.03166	0.19512	0	0	8	198
GO:0051282 regulation of sequestering of calcium ion	6	0.03194	1.858	0.046	Biological	GO:00512	regulation of sequestering of calcium ion	0.03194	0.18597	0	0	6	130
GO:0001738 morphogenesis of a polarized epithelium	5	0.03232	1.548	0.052	Biological	GO:00017	morphogenesis of a polarized epithelium	0.03232	0.18597	0	0	5	97
GO:0001938 positive regulation of endothelial cell proliferation	5	0.03232	1.548	0.052	Biological	GO:00019	positive regulation of endothelial cell proliferation	0.03232	0.18597	0	0	5	97
GO:0002690 positive regulation of leukocyte chemotaxis	5	0.03232	1.548	0.052	Biological	GO:00026	positive regulation of leukocyte chemotaxis	0.03232	0.18597	0	0	5	97
GO:0097306 cellular response to alcohol	5	0.03232	1.548	0.052	Biological	GO:00973	cellular response to alcohol	0.03232	0.18597	0	0	5	97
GO:0030018 Z disc	6	0.03251	1.78	0.045	Cellular C	GO:00300	Z disc	0.03251	0.1942	0	0	6	132
GO:0042622 photoreceptor outer segment membrane	2	0.03291	0.593	0.125	Cellular C	GO:00426	photoreceptor outer segment membrane	0.03291	0.1942	0	0	2	16
GO:0098985 asymmetric, glutamatergic, excitatory synapse	2	0.03291	0.593	0.125	Cellular C	GO:00989	asymmetric, glutamatergic, excitatory synapse	0.03291	0.1942	0	0	2	16
GO:2000027 regulation of animal organ morphogenesis	6	0.03298	1.858	0.046	Biological	GO:20000	regulation of animal organ morphogenesis	0.03298	0.18597	0	0	6	131
GO:0043281 regulation of cysteine-type endopeptidase activity involved	8	0.033	2.477	0.039	Biological	GO:00432	regulation of cysteine-type endopeptidase activity involved in apoptoti	0.033	0.18597	0	0	8	203
GO:0005501 retinoid binding	3	0.03302	0.877	0.079	Molecular	GO:00055	retinoid binding	0.03302	0.19512	0	0	3	38
GO:0019840 isoprenoid binding	3	0.03302	0.877	0.079	Molecular	GO:00198	isoprenoid binding	0.03302	0.19512	0	0	3	38
GO:0005938 cell cortex	11	0.03303	3.264	0.034	Cellular C	GO:00059	cell cortex	0.03303	0.1942	0	0	11	323
GO:0097529 myeloid leukocyte migration	9	0.0331	2.786	0.037	Biological	GO:00975	myeloid leukocyte migration	0.0331	0.18597	0	0	9	241
GO:0010975 regulation of neuron projection development	14	0.03321	4.334	0.032	Biological	GO:00109	regulation of neuron projection development	0.03321	0.18597	0	0	14	443
GO:0098685 Schaffer collateral - CA1 synapse	5	0.03346	1.484	0.051	Cellular C	GO:00986	Schaffer collateral - CA1 synapse	0.03346	0.1946	0	0	5	99
GO:0031401 positive regulation of protein modification process	26	0.03349	8.05	0.027	Biological	GO:00314	positive regulation of protein modification process	0.03349	0.18597	0	0	26	974
GO:0022404 molting cycle process	5	0.03357	1.548	0.051	Biological	GO:00224	molting cycle process	0.03357	0.18597	0	0	5	98
GO:0022405 hair cycle process	5	0.03357	1.548	0.051	Biological	GO:00224	hair cycle process	0.03357	0.18597	0	0	5	98
GO:0006004 fucose metabolic process	2	0.03359	0.619	0.125	Biological	GO:00060	fucose metabolic process	0.03359	0.18597	0	0	2	16
GO:0006555 methionine metabolic process	2	0.03359	0.619	0.125	Biological	GO:00065	methionine metabolic process	0.03359	0.18597	0	0	2	16
GO:0007158 neuron cell-cell adhesion	2	0.03359	0.619	0.125	Biological	GO:00071	neuron cell-cell adhesion	0.03359	0.18597	0	0	2	16
GO:0030033 microvillus assembly	2	0.03359	0.619	0.125	Biological	GO:00300	microvillus assembly	0.03359	0.18597	0	0	2	16
GO:0034349 glial cell apoptotic process	2	0.03359	0.619	0.125	Biological	GO:00343	glial cell apoptotic process	0.03359	0.18597	0	0	2	16
GO:0035641 locomotory exploration behavior	2	0.03359	0.619	0.125	Biological	GO:00356	locomotory exploration behavior	0.03359	0.18597	0	0	2	16
GO:0044849 estrous cycle	2	0.03359	0.619	0.125	Biological	GO:00448	estrous cycle	0.03359	0.18597	0	0	2	16
GO:0045651 positive regulation of macrophage differentiation	2	0.03359	0.619	0.125	Biological	GO:00456	positive regulation of macrophage differentiation	0.03359	0.18597	0	0	2	16
GO:0048385 regulation of retinoic acid receptor signaling pathway	2	0.03359	0.619	0.125	Biological	GO:00483	regulation of retinoic acid receptor signaling pathway	0.03359	0.18597	0	0	2	16
GO:0055089 fatty acid homeostasis	2	0.03359	0.619	0.125	Biological	GO:00550	fatty acid homeostasis	0.03359	0.18597	0	0	2	16
GO:0060192 negative regulation of lipase activity	2	0.03359	0.619	0.125	Biological	GO:00601	negative regulation of lipase activity	0.03359	0.18597	0	0	2	16
GO:0070431 nucleotide-binding oligomerization domain containing 2 sig	2	0.03359	0.619	0.125	Biological	GO:00704	nucleotide-binding oligomerization domain containing 2 signaling path	0.03359	0.18597	0	0	2	16
GO:0072520 seminiferous tubule development	2	0.03359	0.619	0.125	Biological	GO:00725	seminiferous tubule development	0.03359	0.18597	0	0	2	16
GO:0072567 chemokine (C-X-C motif) ligand 2 production	2	0.03359	0.619	0.125	Biological	GO:00725	chemokine (C-X-C motif) ligand 2 production	0.03359	0.18597	0	0	2	16
GO:1905906 regulation of amyloid fibril formation	2	0.03359	0.619	0.125	Biological	GO:19059	regulation of amyloid fibril formation	0.03359	0.18597	0	0	2	16

GO:2000341 regulation of chemokine (C-X-C motif) ligand 2 production	2	0.03359	0.619	0.125	Biological	GO:20003	regulation of chemokine (C-X-C motif) ligand 2 production	0.03359	0.18597	0	0	2	16
GO:0097553 calcium ion transmembrane import into cytosol	7	0.03365	2.167	0.042	Biological	GO:00975	calcium ion transmembrane import into cytosol	0.03365	0.18597	0	0	7	167
GO:0032570 response to progesterone	3	0.03384	0.929	0.077	Biological	GO:00325	response to progesterone	0.03384	0.18597	0	0	3	39
GO:0001885 endothelial cell development	4	0.0339	1.238	0.06	Biological	GO:00018	endothelial cell development	0.0339	0.18597	0	0	4	67
GO:0048857 neural nucleus development	4	0.0339	1.238	0.06	Biological	GO:00488	neural nucleus development	0.0339	0.18597	0	0	4	67
GO:0042176 regulation of protein catabolic process	13	0.03417	4.025	0.032	Biological	GO:00421	regulation of protein catabolic process	0.03417	0.18597	0	0	13	403
GO:0045859 regulation of protein kinase activity	18	0.0344	5.573	0.029	Biological	GO:00458	regulation of protein kinase activity	0.0344	0.18597	0	0	18	617
GO:0008191 metalloendopeptidase inhibitor activity	2	0.03463	0.585	0.125	Molecular	GO:00081	metalloendopeptidase inhibitor activity	0.03463	0.19512	0	0	2	16
GO:0019841 retinol binding	2	0.03463	0.585	0.125	Molecular	GO:00198	retinol binding	0.03463	0.19512	0	0	2	16
GO:0047555 3',5'-cyclic-GMP phosphodiesterase activity	2	0.03463	0.585	0.125	Molecular	GO:00475	3',5'-cyclic-GMP phosphodiesterase activity	0.03463	0.19512	0	0	2	16
GO:0072080 nephron tubule development	5	0.03485	1.548	0.051	Biological	GO:00720	nephron tubule development	0.03485	0.18597	0	0	5	99
GO:0017171 serine hydrolase activity	8	0.03502	2.339	0.04	Molecular	GO:00171	serine hydrolase activity	0.03502	0.19512	0	0	8	202
GO:0005126 cytokine receptor binding	10	0.03509	2.924	0.036	Molecular	GO:00051	cytokine receptor binding	0.03509	0.19512	0	0	10	278
GO:0006873 cellular ion homeostasis	20	0.03519	6.192	0.028	Biological	GO:00068	cellular ion homeostasis	0.03519	0.18597	0	0	20	707
GO:0042445 hormone metabolic process	9	0.03539	2.786	0.037	Biological	GO:00424	hormone metabolic process	0.03539	0.18597	0	0	9	244
GO:0030239 myofibril assembly	4	0.03552	1.238	0.059	Biological	GO:00302	myofibril assembly	0.03552	0.18597	0	0	4	68
GO:0060249 anatomical structure homeostasis	12	0.03553	3.715	0.033	Biological	GO:00602	anatomical structure homeostasis	0.03553	0.18597	0	0	12	364
GO:0009611 response to wounding	17	0.03565	5.263	0.03	Biological	GO:00096	response to wounding	0.03565	0.18597	0	0	17	576
GO:0005584 collagen type I trimer	1	0.03573	0.297	0.5	Cellular C	GO:00055	collagen type I trimer	0.03573	0.19705	0	0	1	2
GO:0034365 discoidal high-density lipoprotein particle	1	0.03573	0.297	0.5	Cellular C	GO:00343	discoidal high-density lipoprotein particle	0.03573	0.19705	0	0	1	2
GO:0150002 distal dendrite	1	0.03573	0.297	0.5	Cellular C	GO:01500	distal dendrite	0.03573	0.19705	0	0	1	2
GO:1902560 GMP reductase complex	1	0.03573	0.297	0.5	Cellular C	GO:19025	GMP reductase complex	0.03573	0.19705	0	0	1	2
GO:1990682 CSF1-CSF1R complex	1	0.03573	0.297	0.5	Cellular C	GO:19906	CSF1-CSF1R complex	0.03573	0.19705	0	0	1	2
GO:0001894 tissue homeostasis	10	0.03602	3.096	0.035	Biological	GO:00018	tissue homeostasis	0.03602	0.18597	0	0	10	284
GO:0001662 behavioral fear response	3	0.0361	0.929	0.075	Biological	GO:00016	behavioral fear response	0.0361	0.18597	0	0	3	40
GO:0032350 regulation of hormone metabolic process	3	0.0361	0.929	0.075	Biological	GO:00323	regulation of hormone metabolic process	0.0361	0.18597	0	0	3	40
GO:1903115 regulation of actin filament-based movement	3	0.0361	0.929	0.075	Biological	GO:19031	regulation of actin filament-based movement	0.0361	0.18597	0	0	3	40
GO:1990000 amyloid fibril formation	3	0.0361	0.929	0.075	Biological	GO:19900	amyloid fibril formation	0.0361	0.18597	0	0	3	40
GO:0001575 globoside metabolic process	1	0.03613	0.31	0.5	Biological	GO:00015	globoside metabolic process	0.03613	0.18597	0	0	1	2
GO:0001576 globoside biosynthetic process	1	0.03613	0.31	0.5	Biological	GO:00015	globoside biosynthetic process	0.03613	0.18597	0	0	1	2
GO:0001970 positive regulation of activation of membrane attack compl	1	0.03613	0.31	0.5	Biological	GO:00019	positive regulation of activation of membrane attack complex	0.03613	0.18597	0	0	1	2
GO:0002121 inter-male aggressive behavior	1	0.03613	0.31	0.5	Biological	GO:00021	inter-male aggressive behavior	0.03613	0.18597	0	0	1	2
GO:0002384 hepatic immune response	1	0.03613	0.31	0.5	Biological	GO:00023	hepatic immune response	0.03613	0.18597	0	0	1	2
GO:0002479 antigen processing and presentation of exogenous peptide	1	0.03613	0.31	0.5	Biological	GO:00024	antigen processing and presentation of exogenous peptide antigen vi	0.03613	0.18597	0	0	1	2
GO:0002543 activation of blood coagulation via clotting cascade	1	0.03613	0.31	0.5	Biological	GO:00025	activation of blood coagulation via clotting cascade	0.03613	0.18597	0	0	1	2
GO:0003340 negative regulation of mesenchymal to epithelial transition	1	0.03613	0.31	0.5	Biological	GO:00033	negative regulation of mesenchymal to epithelial transition involved in	0.03613	0.18597	0	0	1	2
GO:0006083 acetate metabolic process	1	0.03613	0.31	0.5	Biological	GO:00060	acetate metabolic process	0.03613	0.18597	0	0	1	2
GO:0006529 asparagine biosynthetic process	1	0.03613	0.31	0.5	Biological	GO:00065	asparagine biosynthetic process	0.03613	0.18597	0	0	1	2
GO:0007402 ganglion mother cell fate determination	1	0.03613	0.31	0.5	Biological	GO:00074	ganglion mother cell fate determination	0.03613	0.18597	0	0	1	2
GO:0007497 posterior midgut development	1	0.03613	0.31	0.5	Biological	GO:00074	posterior midgut development	0.03613	0.18597	0	0	1	2
GO:0007542 primary sex determination, germ-line	1	0.03613	0.31	0.5	Biological	GO:00075	primary sex determination, germ-line	0.03613	0.18597	0	0	1	2
GO:0008050 female courtship behavior	1	0.03613	0.31	0.5	Biological	GO:00080	female courtship behavior	0.03613	0.18597	0	0	1	2
GO:0010877 lipid transport involved in lipid storage	1	0.03613	0.31	0.5	Biological	GO:00108	lipid transport involved in lipid storage	0.03613	0.18597	0	0	1	2
GO:0014036 neural crest cell fate specification	1	0.03613	0.31	0.5	Biological	GO:00140	neural crest cell fate specification	0.03613	0.18597	0	0	1	2
GO:0016107 sesquiterpenoid catabolic process	1	0.03613	0.31	0.5	Biological	GO:00161	sesquiterpenoid catabolic process	0.03613	0.18597	0	0	1	2
GO:0016487 farnesol metabolic process	1	0.03613	0.31	0.5	Biological	GO:00164	farnesol metabolic process	0.03613	0.18597	0	0	1	2
GO:0016488 farnesol catabolic process	1	0.03613	0.31	0.5	Biological	GO:00164	farnesol catabolic process	0.03613	0.18597	0	0	1	2
GO:0018992 germ-line sex determination	1	0.03613	0.31	0.5	Biological	GO:00189	germ-line sex determination	0.03613	0.18597	0	0	1	2
GO:0019100 male germ-line sex determination	1	0.03613	0.31	0.5	Biological	GO:00191	male germ-line sex determination	0.03613	0.18597	0	0	1	2
GO:0019427 acetyl-CoA biosynthetic process from acetate	1	0.03613	0.31	0.5	Biological	GO:00194	acetyl-CoA biosynthetic process from acetate	0.03613	0.18597	0	0	1	2
GO:0021568 rhombomere 2 development	1	0.03613	0.31	0.5	Biological	GO:00215	rhombomere 2 development	0.03613	0.18597	0	0	1	2
GO:0030237 female sex determination	1	0.03613	0.31	0.5	Biological	GO:00302	female sex determination	0.03613	0.18597	0	0	1	2
GO:0032242 regulation of nucleoside transport	1	0.03613	0.31	0.5	Biological	GO:00322	regulation of nucleoside transport	0.03613	0.18597	0	0	1	2
GO:0032639 TRAIL production	1	0.03613	0.31	0.5	Biological	GO:00326	TRAIL production	0.03613	0.18597	0	0	1	2
GO:0032679 regulation of TRAIL production	1	0.03613	0.31	0.5	Biological	GO:00326	regulation of TRAIL production	0.03613	0.18597	0	0	1	2
GO:0032759 positive regulation of TRAIL production	1	0.03613	0.31	0.5	Biological	GO:00327	positive regulation of TRAIL production	0.03613	0.18597	0	0	1	2
GO:0034346 positive regulation of type III interferon production	1	0.03613	0.31	0.5	Biological	GO:00343	positive regulation of type III interferon production	0.03613	0.18597	0	0	1	2
GO:0035026 leading edge cell differentiation	1	0.03613	0.31	0.5	Biological	GO:00350	leading edge cell differentiation	0.03613	0.18597	0	0	1	2
GO:0035622 intrahepatic bile duct development	1	0.03613	0.31	0.5	Biological	GO:00356	intrahepatic bile duct development	0.03613	0.18597	0	0	1	2
GO:0036515 serotonergic neuron axon guidance	1	0.03613	0.31	0.5	Biological	GO:00365	serotonergic neuron axon guidance	0.03613	0.18597	0	0	1	2
GO:0042488 positive regulation of odontogenesis of dentin-containing to	1	0.03613	0.31	0.5	Biological	GO:00424	positive regulation of odontogenesis of dentin-containing tooth	0.03613	0.18597	0	0	1	2
GO:0042704 uterine wall breakdown	1	0.03613	0.31	0.5	Biological	GO:00427	uterine wall breakdown	0.03613	0.18597	0	0	1	2
GO:0044751 cellular response to human chorionic gonadotropin stimulu	1	0.03613	0.31	0.5	Biological	GO:00447	cellular response to human chorionic gonadotropin stimulus	0.03613	0.18597	0	0	1	2
GO:0045213 neurotransmitter receptor metabolic process	1	0.03613	0.31	0.5	Biological	GO:00452	neurotransmitter receptor metabolic process	0.03613	0.18597	0	0	1	2
GO:0045720 negative regulation of integrin biosynthetic process	1	0.03613	0.31	0.5	Biological	GO:00457	negative regulation of integrin biosynthetic process	0.03613	0.18597	0	0	1	2
GO:0045827 negative regulation of isoprenoid metabolic process	1	0.03613	0.31	0.5	Biological	GO:00458	negative regulation of isoprenoid metabolic process	0.03613	0.18597	0	0	1	2
GO:0045976 negative regulation of mitotic cell cycle, embryonic	1	0.03613	0.31	0.5	Biological	GO:00459	negative regulation of mitotic cell cycle, embryonic	0.03613	0.18597	0	0	1	2
GO:0046725 negative regulation by virus of viral protein levels in host ce	1	0.03613	0.31	0.5	Biological	GO:00467	negative regulation by virus of viral protein levels in host cell	0.03613	0.18597	0	0	1	2
GO:0051892 negative regulation of cardioblast differentiation	1	0.03613	0.31	0.5	Biological	GO:00518	negative regulation of cardioblast differentiation	0.03613	0.18597	0	0	1	2
GO:0052312 modulation of transcription in other organism involved in sy	1	0.03613	0.31	0.5	Biological	GO:00523	modulation of transcription in other organism involved in symbiotic int	0.03613	0.18597	0	0	1	2
GO:0052472 modulation by host of symbiont transcription	1	0.03613	0.31	0.5	Biological	GO:00524	modulation by host of symbiont transcription	0.03613	0.18597	0	0	1	2
GO:0060014 granulosa cell differentiation	1	0.03613	0.31	0.5	Biological	GO:00600	granulosa cell differentiation	0.03613	0.18597	0	0	1	2
GO:0060282 positive regulation of oocyte development	1	0.03613	0.31	0.5	Biological	GO:00602	positive regulation of oocyte development	0.03613	0.18597	0	0	1	2
GO:0060516 primary prostatic bud elongation	1	0.03613	0.31	0.5	Biological	GO:00605	primary prostatic bud elongation	0.03613	0.18597	0	0	1	2
GO:0060532 bronchus cartilage development	1	0.03613	0.31	0.5	Biological	GO:00605	bronchus cartilage development	0.03613	0.18597	0	0	1	2
GO:0060599 lateral sprouting involved in mammary gland duct morphog	1	0.03613	0.31	0.5	Biological	GO:00605	lateral sprouting involved in mammary gland duct morphogenesis	0.03613	0.18597	0	0	1	2
GO:0060720 spongiotrophoblast cell proliferation	1	0.03613	0.31	0.5	Biological	GO:00607	spongiotrophoblast cell proliferation	0.03613	0.18597	0	0	1	2
GO:0060722 cell proliferation involved in embryonic placenta developme	1	0.03613	0.31	0.5	Biological	GO:00607	cell proliferation involved in embryonic placenta development	0.03613	0.18597	0	0	1	2
GO:0060857 establishment of glial blood-brain barrier	1	0.03613	0.31	0.5	Biological	GO:00608	establishment of glial blood-brain barrier	0.03613	0.18597	0	0	1	2
GO:0060974 cell migration involved in heart formation	1	0.03613	0.31	0.5	Biological	GO:00609	cell migration involved in heart formation	0.03613	0.18597	0	0	1	2
GO:0061145 lung smooth muscle development	1	0.03613	0.31	0.5	Biological	GO:00611	lung smooth muscle development	0.03613	0.18597	0	0	1	2
GO:0061152 trachea submucosa development	1	0.03613	0.31	0.5	Biological	GO:00611	trachea submucosa development	0.03613	0.18597	0	0	1	2
GO:0061153 trachea gland development	1	0.03613	0.31	0.5	Biological	GO:00611	trachea gland development	0.03613	0.18597	0	0	1	2
GO:0061159 establishment of bipolar cell polarity involved in cell morph	1	0.03613	0.31	0.5	Biological	GO:00611	establishment of bipolar cell polarity involved in cell morphogenesis	0.03613	0.18597	0	0	1	2
GO:0061868 hepatic stellate cell migration	1	0.03613	0.31	0.5	Biological	GO:00618	hepatic stellate cell migration	0.03613	0.18597	0	0	1	2
GO:0061869 regulation of hepatic stellate cell migration	1	0.03613	0.31	0.5	Biological	GO:00618	regulation of hepatic stellate cell migration	0.03613	0.18597	0	0	1	2
GO:0061870 positive regulation of hepatic stellate cell migration	1	0.03613	0.31	0.5	Biological	GO:00618	positive regulation of hepatic stellate cell migration	0.03613	0.18597	0	0	1	2
GO:0062044 negative regulation of cardiac epithelial to mesenchymal tra	1	0.03613	0.31	0.5	Biological	GO:00620	negative regulation of cardiac epithelial to mesenchymal transition	0.03613	0.18597	0	0	1	2
GO:0070256 negative regulation of mucus secretion	1	0.03613	0.31	0.5	Biological	GO:00702	negative regulation of mucus secretion	0.03613	0.18597	0	0	1	2
GO:0070982 L-asparagine metabolic process	1	0.03613	0.31	0.5	Biological	GO:00709	L-asparagine metabolic process	0.03613	0.18597	0	0	1	2
GO:0071109 superior temporal gyrus development	1	0.03613	0.31	0.5	Biological	GO:00711	superior temporal gyrus development	0.03613	0.18597	0	0	1	2
GO:0071231 cellular response to folic acid	1	0.03613	0.31	0.5	Biological	GO:00712	cellular response to folic acid	0.03613	0.18597	0	0	1	2
GO:0071409 cellular response to cycloheximide	1	0.03613	0.31	0.5	Biological	GO:00714	cellular response to cycloheximide	0.03613	0.18597	0	0	1	2
GO:0071505 response to mycophenolic acid	1	0.03613	0.31	0.5	Biological	GO:00715	response to mycophenolic acid	0.03613	0.18597	0	0	1	2
GO:0071506 cellular response to mycophenolic acid	1	0.03613	0.31	0.5	Biological	GO:00715	cellular response to mycophenolic acid	0.03613	0.18597	0	0	1	2
GO:0071642 positive regulation of macrophage inflammatory protein 1 a	1	0.03613	0.31	0.5	Biological	GO:00716	positive regulation of macrophage inflammatory protein 1 alpha produ	0.03613	0.18597	0	0	1	2
GO:0072046 establishment of planar polarity involved in nephron morph	1	0.03613	0.31	0.5	Biological	GO:00720	establishment of planar polarity involved in nephron morphogenesis	0.03613	0.18597	0	0	1	2
GO:0072183 negative regulation of nephron tubule epithelial cell differen	1	0.03613	0.31	0.5	Biological	GO:00721	negative regulation of nephron tubule epithelial cell differentiation	0.03613	0.18597	0	0	1	2
GO:0072301 regulation of metanephric glomerular mesangial cell prolife	1	0.03613	0.31	0.5	Biological	GO:00723	regulation of metanephric glomerular mesangial cell proliferation	0.03613	0.18597	0	0	1	2
GO:0090214 spongiotrophoblast layer developmental growth	1	0.03613	0.31	0.5	Biological	GO:00902	spongiotrophoblast layer developmental growth	0.03613	0.18597	0	0	1	2
GO:0097474 retinal cone cell apoptotic process	1	0.03613	0.31	0.5	Biological	GO:00974	retinal cone cell apoptotic process	0.03613	0.18597	0	0	1	2
GO:0097498 endothelial tube lumen extension	1	0.03613	0.31	0.5	Biological	GO:00974	endothelial tube lumen extension	0.03613	0.18597	0	0	1	2
GO:0097528 execution phase of necroptosis	1	0.03613	0.31	0.5	Biological	GO:00975	execution phase of necroptosis	0.03613	0.18597	0	0	1	2
GO:0097532 stress response to acid chemical	1	0.03613	0.31	0.5	Biological	GO:00975	stress response to acid chemical	0.03613	0.18597	0	0	1	2
GO:0097533 cellular stress response to acid chemical	1	0.03613	0.31	0.5	Biological	GO:00975	cellular stress response to acid chemical	0.03613	0.18597	0	0	1	2
GO:0099180 zinc ion import into synaptic vesicle	1	0.03613	0.31	0.5	Biological	GO:00991	zinc ion import into synaptic vesicle	0.03613	0.18597	0	0	1	2
GO:1900053 negative regulation of retinoic acid biosynthetic process	1	0.03613	0.31	0.5	Biological	GO:19000	negative regulation of retinoic acid biosynthetic process	0.03613	0.18597	0	0	1	2
GO:1901628 positive regulation of postsynaptic membrane organization	1	0.03613	0.31	0.5	Biological	GO:19016	positive regulation of postsynaptic membrane organization	0.03613	0.18597	0	0	1	2
GO:1902068 regulation of sphingolipid mediated signaling pathway	1	0.03613	0.31	0.5	Biological	GO:19020	regulation of sphingolipid mediated signaling pathway	0.03613	0.18597	0	0	1	2
GO:1902957 negative regulation of mitochondrial electron transport, NA	1	0.03613	0.31	0.5	Biological	GO:19029	negative regulation of mitochondrial electron transport, NADH to ubiq	0.03613	0.18597	0	0	1	2
GO:1903116 positive regulation of actin filament-based movement	1	0.03613	0.31	0.5	Biological	GO:19031	positive regulation of actin filament-based movement	0.03613	0.18597	0	0	1	2
GO:1903547 regulation of growth hormone activity	1	0.03613	0.31	0.5	Biological	GO:19035	regulation of growth hormone activity	0.03613	0.18597	0	0	1	2
GO:1903847 regulation of aorta morphogenesis	1	0.03613	0.31	0.5	Biological	GO:19038	regulation of aorta morphogenesis	0.03613	0.18597	0	0	1	2
GO:1903849 positive regulation of aorta morphogenesis	1	0.03613	0.31	0.5	Biological	GO:19038	positive regulation of aorta morphogenesis	0.03613	0.18597	0	0	1	2
GO:1904204 regulation of skeletal muscle hypertrophy	1	0.03613	0.31	0.5	Biological	GO:19042	regulation of skeletal muscle hypertrophy	0.03613	0.18597	0	0	1	2
GO:1904217 regulation of CDP-diacylglycerol-serine O-phosphatidyltran	1	0.03613	0.31	0.5	Biological	GO:19042	regulation of CDP-diacylglycerol-serine O-phosphatidyltransferase ac	0.03613	0.18597	0	0	1	2
GO:1904219 positive regulation of CDP-diacylglycerol-serine O-phospha	1	0.03613	0.31	0.5	Biological	GO:19042	positive regulation of CDP-diacylglycerol-serine O-phosphatidyltransf	0.03613	0.18597	0	0	1	2
GO:1904222 positive regulation of serine C-palmitoyltransferase activity	1	0.03613	0.31	0.5	Biological	GO:19042	positive regulation of serine C-palmitoyltransferase activity	0.03613	0.18597	0	0	1	2
GO:1904685 positive regulation of metalloendopeptidase activity	1	0.03613	0.31	0.5	Biological	GO:19046	positive regulation of metalloendopeptidase activity	0.03613	0.18597	0	0	1	2
GO:1904719 positive regulation of AMPA glutamate receptor clustering	1	0.03613	0.31	0.5	Biological	GO:19047	positive regulation of AMPA glutamate receptor clustering	0.03613	0.18597	0	0	1	2
GO:1904806 regulation of protein oxidation	1	0.03613	0.31	0.5	Biological	GO:19048	regulation of protein oxidation	0.03613	0.18597	0	0	1	2
GO:1904808 positive regulation of protein oxidation	1	0.03613	0.31	0.5	Biological	GO:19048	positive regulation of protein oxidation	0.03613	0.18597	0	0	1	2
GO:1904842 response to nitroglycerin	1	0.03613	0.31	0.5	Biological	GO:19048	response to nitroglycerin	0.03613	0.18597	0	0	1	2
GO:1904863 regulation of beta-catenin-TCF complex assembly	1	0.03613	0.31	0.5	Biological	GO:19048	regulation of beta-catenin-TCF complex assembly	0.03613	0.18597	0	0	1	2
GO:1904864 negative regulation of beta-catenin-TCF complex assembly	1	0.03613	0.31	0.5	Biological	GO:19048	negative regulation of beta-catenin-TCF complex assembly	0.03613	0.18597	0	0	1	2
GO:1905006 negative regulation of epithelial to mesenchymal transition	1	0.03613	0.31	0.5	Biological	GO:19050	negative regulation of epithelial to mesenchymal transition involved in	0.03613	0.18597	0	0	1	2
GO:1905243 cellular response to 3,3',5-triiodo-L-thyronine	1	0.03613	0.31	0.5	Biological	GO:19052	cellular response to 3,3',5-triiodo-L-thyronine	0.03613	0.18597	0	0	1	2
GO:1905447 negative regulation of mitochondrial ATP synthesis couple	1	0.03613	0.31	0.5	Biological	GO:19054	negative regulation of mitochondrial ATP synthesis coupled electron t	0.03613	0.18597	0	0	1	2
GO:1905751 positive regulation of endosome to plasma membrane prot	1	0.03613	0.31	0.5	Biological	GO:19057	positive regulation of endosome to plasma membrane protein transpo	0.03613	0.18597	0	0	1	2
GO:1905871 regulation of protein localization to cell leading edge	1	0.03613	0.31	0.5	Biological	GO:19058	regulation of protein localization to cell leading edge	0.03613	0.18597	0	0	1	2
GO:1990839 response to endothelin	1	0.03613	0.31	0.5	Biological	GO:19908	response to endothelin	0.03613	0.18597	0	0	1	2
GO:2000422 regulation of eosinophil chemotaxis	1	0.03613	0.31	0.5	Biological	GO:20004	regulation of eosinophil chemotaxis	0.03613	0.18597	0	0	1	2
GO:2000424 positive regulation of eosinophil chemotaxis	1	0.03613	0.31	0.5	Biological	GO:20004	positive regulation of eosinophil chemotaxis	0.03613	0.18597	0	0	1	2
GO:2000522 positive regulation of immunological synapse formation	1	0.03613	0.31	0.5	Biological	GO:20005	positive regulation of immunological synapse formation	0.03613	0.18597	0	0	1	2
GO:2000707 positive regulation of dense core granule biogenesis	1	0.03613	0.31	0.5	Biological	GO:20007	positive regulation of dense core granule biogenesis	0.03613	0.18597	0	0	1	2
GO:2000729 positive regulation of mesenchymal cell proliferation involve	1	0.03613	0.31	0.5	Biological	GO:20007	positive regulation of mesenchymal cell proliferation involved in ureter	0.03613	0.18597	0	0	1	2
GO:2001106 regulation of Rho guanyl-nucleotide exchange factor activit	1	0.03613	0.31	0.5	Biological	GO:20011	regulation of Rho guanyl-nucleotide exchange factor activity	0.03613	0.18597	0	0	1	2
GO:0098773 skin epidermis development	5	0.03616	1.548	0.05	Biological	GO:00987	skin epidermis development	0.03616	0.18597	0	0	5	100
GO:0051208 sequestering of calcium ion	6	0.03623	1.858	0.045	Biological	GO:00512	sequestering of calcium ion	0.03623	0.18617	0	0	6	134
GO:0015085 calcium ion transmembrane transporter activity	6	0.03647	1.754	0.045	Molecular	GO:00150	calcium ion transmembrane transporter activity	0.03647	0.19512	0	0	6	132
GO:0070371 ERK1 and ERK2 cascade	11	0.03668	3.406	0.034	Biological	GO:00703	ERK1 and ERK2 cascade	0.03668	0.1881	0	0	11	325
GO:0070482 response to oxygen levels	11	0.03668	3.406	0.034	Biological	GO:00704	response to oxygen levels	0.03668	0.1881	0	0	11	325
GO:0001883 purine nucleoside binding	1	0.03673	0.292	0.5	Molecular	GO:00018	purine nucleoside binding	0.03673	0.19512	0	0	1	2
GO:0003875 ADP-ribosylarginine hydrolase activity	1	0.03673	0.292	0.5	Molecular	GO:00038	ADP-ribosylarginine hydrolase activity	0.03673	0.19512	0	0	1	2
GO:0003920 GMP reductase activity	1	0.03673	0.292	0.5	Molecular	GO:00039	GMP reductase activity	0.03673	0.19512	0	0	1	2
GO:0004066 asparagine synthase (glutamine-hydrolyzing) activity	1	0.03673	0.292	0.5	Molecular	GO:00040	asparagine synthase (glutamine-hydrolyzing) activity	0.03673	0.19512	0	0	1	2
GO:0004119 cGMP-inhibited cyclic-nucleotide phosphodiesterase activi	1	0.03673	0.292	0.5	Molecular	GO:00041	cGMP-inhibited cyclic-nucleotide phosphodiesterase activity	0.03673	0.19512	0	0	1	2
GO:0004422 hypoxanthine phosphoribosyltransferase activity	1	0.03673	0.292	0.5	Molecular	GO:00044	hypoxanthine phosphoribosyltransferase activity	0.03673	0.19512	0	0	1	2
GO:0004478 methionine adenosyltransferase activity	1	0.03673	0.292	0.5	Molecular	GO:00044	methionine adenosyltransferase activity	0.03673	0.19512	0	0	1	2
GO:0004796 thromboxane-A synthase activity	1	0.03673	0.292	0.5	Molecular	GO:00047	thromboxane-A synthase activity	0.03673	0.19512	0	0	1	2
GO:0004915 interleukin-6 receptor activity	1	0.03673	0.292	0.5	Molecular	GO:00049	interleukin-6 receptor activity	0.03673	0.19512	0	0	1	2
GO:0008107 galactoside 2-alpha-L-fucosyltransferase activity	1	0.03673	0.292	0.5	Molecular	GO:00081	galactoside 2-alpha-L-fucosyltransferase activity	0.03673	0.19512	0	0	1	2

GO:0008113 peptide-methionine (S)-S-oxide reductase activity	1	0.03673	0.292	0.5	Molecular	GO:0008	peptide-methionine (S)-S-oxide reductase activity	0.03673	0.19512	0	0	1	2
GO:0010465 nerve growth factor receptor activity	1	0.03673	0.292	0.5	Molecular	GO:0010	nerve growth factor receptor activity	0.03673	0.19512	0	0	1	2
GO:0015657 branched-chain amino acid:sodium symporter activity	1	0.03673	0.292	0.5	Molecular	GO:0015	branched-chain amino acid:sodium symporter activity	0.03673	0.19512	0	0	1	2
GO:0016213 linoleoyl-CoA desaturase activity	1	0.03673	0.292	0.5	Molecular	GO:0016	linoleoyl-CoA desaturase activity	0.03673	0.19512	0	0	1	2
GO:0019981 interleukin-6 binding	1	0.03673	0.292	0.5	Molecular	GO:0019	interleukin-6 binding	0.03673	0.19512	0	0	1	2
GO:0031127 alpha-(1,2)-fucosyltransferase activity	1	0.03673	0.292	0.5	Molecular	GO:0031	alpha-(1,2)-fucosyltransferase activity	0.03673	0.19512	0	0	1	2
GO:0031686 A1 adenosine receptor binding	1	0.03673	0.292	0.5	Molecular	GO:0031	A1 adenosine receptor binding	0.03673	0.19512	0	0	1	2
GO:0031780 corticotropin hormone receptor binding	1	0.03673	0.292	0.5	Molecular	GO:0031	corticotropin hormone receptor binding	0.03673	0.19512	0	0	1	2
GO:0031783 type 5 melanocortin receptor binding	1	0.03673	0.292	0.5	Molecular	GO:0031	type 5 melanocortin receptor binding	0.03673	0.19512	0	0	1	2
GO:0036130 prostaglandin H2 endoperoxidase reductase activity	1	0.03673	0.292	0.5	Molecular	GO:0036	prostaglandin H2 endoperoxidase reductase activity	0.03673	0.19512	0	0	1	2
GO:0043992 histone acetyltransferase activity (H3-K9 specific)	1	0.03673	0.292	0.5	Molecular	GO:0043	histone acetyltransferase activity (H3-K9 specific)	0.03673	0.19512	0	0	1	2
GO:0045523 interleukin-27 receptor binding	1	0.03673	0.292	0.5	Molecular	GO:0045	interleukin-27 receptor binding	0.03673	0.19512	0	0	1	2
GO:0045550 geranylgeranyl reductase activity	1	0.03673	0.292	0.5	Molecular	GO:0045	geranylgeranyl reductase activity	0.03673	0.19512	0	0	1	2
GO:0047017 prostaglandin-F synthase activity	1	0.03673	0.292	0.5	Molecular	GO:0047	prostaglandin-F synthase activity	0.03673	0.19512	0	0	1	2
GO:0047020 15-hydroxyprostaglandin-D dehydrogenase (NADP+) activ	1	0.03673	0.292	0.5	Molecular	GO:0047	15-hydroxyprostaglandin-D dehydrogenase (NADP+) activity	0.03673	0.19512	0	0	1	2
GO:0047389 glycerophosphocholine phosphodiesterase activity	1	0.03673	0.292	0.5	Molecular	GO:0047	glycerophosphocholine phosphodiesterase activity	0.03673	0.19512	0	0	1	2
GO:0047787 delta4-3-oxosteroid 5beta-reductase activity	1	0.03673	0.292	0.5	Molecular	GO:0047	delta4-3-oxosteroid 5beta-reductase activity	0.03673	0.19512	0	0	1	2
GO:0047975 guanosine phosphorylase activity	1	0.03673	0.292	0.5	Molecular	GO:0047	guanosine phosphorylase activity	0.03673	0.19512	0	0	1	2
GO:0061714 folic acid receptor activity	1	0.03673	0.292	0.5	Molecular	GO:0061	folic acid receptor activity	0.03673	0.19512	0	0	1	2
GO:0140625 opioid growth factor receptor activity	1	0.03673	0.292	0.5	Molecular	GO:0140	opioid growth factor receptor activity	0.03673	0.19512	0	0	1	2
GO:0001666 response to hypoxia	10	0.03675	3.096	0.035	Biological	GO:0001	response to hypoxia	0.03675	0.18825	0	0	10	285
GO:0033036 macromolecule localization	67	0.03682	20.743	0.022	Biological	GO:0033	macromolecule localization	0.03682	0.18842	0	0	67	2982
GO:0005869 dynactin complex	2	0.03686	0.593	0.118	Cellular C	GO:0005	dynactin complex	0.03686	0.19752	0	0	2	17
GO:0043034 costamere	2	0.03686	0.593	0.118	Cellular C	GO:0043	costamere	0.03686	0.19752	0	0	2	17
GO:0045121 membrane raft	11	0.03697	3.264	0.033	Cellular C	GO:0045	membrane raft	0.03697	0.19752	0	0	11	329
GO:0045670 regulation of osteoclast differentiation	4	0.03718	1.238	0.058	Biological	GO:0045	regulation of osteoclast differentiation	0.03718	0.18922	0	0	4	69
GO:0048645 animal organ formation	4	0.03718	1.238	0.058	Biological	GO:0048	animal organ formation	0.03718	0.18922	0	0	4	69
GO:0055002 striated muscle cell development	4	0.03718	1.238	0.058	Biological	GO:0055	striated muscle cell development	0.03718	0.18922	0	0	4	69
GO:1903827 regulation of cellular protein localization	16	0.03737	4.954	0.03	Biological	GO:1903	regulation of cellular protein localization	0.03737	0.18922	0	0	16	536
GO:0032994 protein-lipid complex	3	0.03738	0.89	0.073	Cellular C	GO:0032	protein-lipid complex	0.03738	0.19752	0	0	3	41
GO:0071772 response to BMP	7	0.03751	2.167	0.041	Biological	GO:0071	response to BMP	0.03751	0.18922	0	0	7	171
GO:0071773 cellular response to BMP stimulus	7	0.03751	2.167	0.041	Biological	GO:0071	cellular response to BMP stimulus	0.03751	0.18922	0	0	7	171
GO:0010566 regulation of ketone biosynthetic process	2	0.03762	0.619	0.118	Biological	GO:0010	regulation of ketone biosynthetic process	0.03762	0.18922	0	0	2	17
GO:0016114 terpenoid biosynthetic process	2	0.03762	0.619	0.118	Biological	GO:0016	terpenoid biosynthetic process	0.03762	0.18922	0	0	2	17
GO:0030202 heparin metabolic process	2	0.03762	0.619	0.118	Biological	GO:0030	heparin metabolic process	0.03762	0.18922	0	0	2	17
GO:0033631 cell-cell adhesion mediated by integrin	2	0.03762	0.619	0.118	Biological	GO:0033	cell-cell adhesion mediated by integrin	0.03762	0.18922	0	0	2	17
GO:0036065 fucosylation	2	0.03762	0.619	0.118	Biological	GO:0036	fucosylation	0.03762	0.18922	0	0	2	17
GO:0042953 lipoprotein transport	2	0.03762	0.619	0.118	Biological	GO:0042	lipoprotein transport	0.03762	0.18922	0	0	2	17
GO:0043117 positive regulation of vascular permeability	2	0.03762	0.619	0.118	Biological	GO:0043	positive regulation of vascular permeability	0.03762	0.18922	0	0	2	17
GO:0045217 cell-cell junction maintenance	2	0.03762	0.619	0.118	Biological	GO:0045	cell-cell junction maintenance	0.03762	0.18922	0	0	2	17
GO:0061548 ganglion development	2	0.03762	0.619	0.118	Biological	GO:0061	ganglion development	0.03762	0.18922	0	0	2	17
GO:0072578 neurotransmitter-gated ion channel clustering	2	0.03762	0.619	0.118	Biological	GO:0072	neurotransmitter-gated ion channel clustering	0.03762	0.18922	0	0	2	17
GO:2000651 positive regulation of sodium ion transmembrane transport	2	0.03762	0.619	0.118	Biological	GO:2000	positive regulation of sodium ion transmembrane transporter activity	0.03762	0.18922	0	0	2	17
GO:0098857 membrane microdomain	11	0.03766	3.264	0.033	Cellular C	GO:0098	membrane microdomain	0.03766	0.19752	0	0	11	330
GO:0070085 glycosylation	9	0.03779	2.786	0.036	Biological	GO:0070	glycosylation	0.03779	0.18985	0	0	9	247
GO:0002209 behavioral defense response	3	0.03844	0.929	0.073	Biological	GO:0002	behavioral defense response	0.03844	0.19219	0	0	3	41
GO:0003203 endocardial cushion morphogenesis	3	0.03844	0.929	0.073	Biological	GO:0003	endocardial cushion morphogenesis	0.03844	0.19219	0	0	3	41
GO:0014002 astrocyte development	3	0.03844	0.929	0.073	Biological	GO:0014	astrocyte development	0.03844	0.19219	0	0	3	41
GO:0032941 secretion by tissue	3	0.03844	0.929	0.073	Biological	GO:0032	secretion by tissue	0.03844	0.19219	0	0	3	41
GO:0099068 postsynapse assembly	3	0.03844	0.929	0.073	Biological	GO:0099	postsynapse assembly	0.03844	0.19219	0	0	3	41
GO:0048663 neuron fate commitment	4	0.03889	1.238	0.057	Biological	GO:0048	neuron fate commitment	0.03889	0.19422	0	0	4	70
GO:0048584 positive regulation of response to stimulus	52	0.0393	16.099	0.023	Biological	GO:0048	positive regulation of response to stimulus	0.0393	0.19605	0	0	52	2241
GO:0042476 odontogenesis	6	0.03969	1.858	0.044	Biological	GO:0042	odontogenesis	0.03969	0.19782	0	0	6	137
GO:0140297 DNA-binding transcription factor binding	15	0.03985	4.386	0.031	Molecular	GO:0140	DNA-binding transcription factor binding	0.03985	0.21044	0	0	15	489
GO:0045637 regulation of myeloid cell differentiation	8	0.04002	2.477	0.038	Biological	GO:0045	regulation of myeloid cell differentiation	0.04002	0.19904	0	0	8	211
GO:1901654 response to ketone	8	0.04002	2.477	0.038	Biological	GO:1901	response to ketone	0.04002	0.19904	0	0	8	211
GO:0007009 plasma membrane organization	7	0.04059	2.167	0.04	Biological	GO:0007	plasma membrane organization	0.04059	0.2017	0	0	7	174
GO:0046847 filopodium assembly	4	0.04064	1.238	0.056	Biological	GO:0046	filopodium assembly	0.04064	0.20176	0	0	4	71
GO:0051480 regulation of cytosolic calcium ion concentration	12	0.04083	3.715	0.032	Biological	GO:0051	regulation of cytosolic calcium ion concentration	0.04083	0.20244	0	0	12	372
GO:0003351 epithelial cilium movement involved in extracellular fluid mo	3	0.04086	0.929	0.071	Biological	GO:0003	epithelial cilium movement involved in extracellular fluid movement	0.04086	0.20244	0	0	3	42
GO:0005518 collagen binding	4	0.04094	1.17	0.057	Molecular	GO:0005	collagen binding	0.04094	0.21485	0	0	4	70
GO:0070588 calcium ion transmembrane transport	11	0.04097	3.406	0.033	Biological	GO:0070	calcium ion transmembrane transport	0.04097	0.20278	0	0	11	331
GO:0031527 filopodium membrane	2	0.04098	0.593	0.111	Cellular C	GO:0031	filopodium membrane	0.04098	0.21288	0	0	2	18
GO:0034613 cellular protein localization	41	0.04134	12.693	0.024	Biological	GO:0034	cellular protein localization	0.04134	0.20439	0	0	41	1708
GO:0019896 axonal transport of mitochondrion	2	0.04183	0.619	0.111	Biological	GO:0019	axonal transport of mitochondrion	0.04183	0.20439	0	0	2	18
GO:0030050 vesicle transport along actin filament	2	0.04183	0.619	0.111	Biological	GO:0030	vesicle transport along actin filament	0.04183	0.20439	0	0	2	18
GO:0038128 ERBB2 signaling pathway	2	0.04183	0.619	0.111	Biological	GO:0038	ERBB2 signaling pathway	0.04183	0.20439	0	0	2	18
GO:0044320 cellular response to leptin stimulus	2	0.04183	0.619	0.111	Biological	GO:0044	cellular response to leptin stimulus	0.04183	0.20439	0	0	2	18
GO:0044872 lipoprotein localization	2	0.04183	0.619	0.111	Biological	GO:0044	lipoprotein localization	0.04183	0.20439	0	0	2	18
GO:0051004 regulation of lipoprotein lipase activity	2	0.04183	0.619	0.111	Biological	GO:0051	regulation of lipoprotein lipase activity	0.04183	0.20439	0	0	2	18
GO:0060252 positive regulation of glial cell proliferation	2	0.04183	0.619	0.111	Biological	GO:0060	positive regulation of glial cell proliferation	0.04183	0.20439	0	0	2	18
GO:0060644 mammary gland epithelial cell differentiation	2	0.04183	0.619	0.111	Biological	GO:0060	mammary gland epithelial cell differentiation	0.04183	0.20439	0	0	2	18
GO:0070206 protein trimerization	2	0.04183	0.619	0.111	Biological	GO:0070	protein trimerization	0.04183	0.20439	0	0	2	18
GO:0072189 ureter development	2	0.04183	0.619	0.111	Biological	GO:0072	ureter development	0.04183	0.20439	0	0	2	18
GO:0090136 epithelial cell-cell adhesion	2	0.04183	0.619	0.111	Biological	GO:0090	epithelial cell-cell adhesion	0.04183	0.20439	0	0	2	18
GO:2000136 regulation of cell proliferation involved in heart morphogene	2	0.04183	0.619	0.111	Biological	GO:2000	regulation of cell proliferation involved in heart morphogenesis	0.04183	0.20439	0	0	2	18
GO:0043254 regulation of protein-containing complex assembly	14	0.04198	4.334	0.031	Biological	GO:0043	regulation of protein-containing complex assembly	0.04198	0.20495	0	0	14	458
GO:0036477 somatodendritic compartment	23	0.04204	6.825	0.027	Cellular C	GO:0036	somatodendritic compartment	0.04204	0.21624	0	0	23	867
GO:0035914 skeletal muscle cell differentiation	4	0.04244	1.238	0.056	Biological	GO:0035	skeletal muscle cell differentiation	0.04244	0.20678	0	0	4	72
GO:0061180 mammary gland epithelium development	4	0.04244	1.238	0.056	Biological	GO:0061	mammary gland epithelium development	0.04244	0.20678	0	0	4	72
GO:0005488 binding	322	0.04254	94.152	0.019	Molecular	GO:0005	binding	0.04254	0.22088	0	0	322	16887
GO:0008201 heparin binding	7	0.04263	2.047	0.04	Molecular	GO:0008	heparin binding	0.04263	0.22088	0	0	7	173
GO:0016529 sarcoplasmic reticulum	4	0.0428	1.187	0.055	Cellular C	GO:0016	sarcoplasmic reticulum	0.0428	0.21739	0	0	4	73
GO:0060541 respiratory system development	8	0.04288	2.477	0.037	Biological	GO:0060	respiratory system development	0.04288	0.20873	0	0	8	214
GO:0010595 positive regulation of endothelial cell migration	5	0.04316	1.548	0.048	Biological	GO:0010	positive regulation of endothelial cell migration	0.04316	0.20985	0	0	5	105
GO:0071840 cellular component organization or biogenesis	136	0.04327	42.105	0.021	Biological	GO:0071	cellular component organization or biogenesis	0.04327	0.20999	0	0	136	6619
GO:0001937 negative regulation of endothelial cell proliferation	3	0.04335	0.929	0.07	Biological	GO:0001	negative regulation of endothelial cell proliferation	0.04335	0.20999	0	0	3	43
GO:0015695 organic cation transport	3	0.04335	0.929	0.07	Biological	GO:0015	organic cation transport	0.04335	0.20999	0	0	3	43
GO:0060428 lung epithelium development	3	0.04335	0.929	0.07	Biological	GO:0060	lung epithelium development	0.04335	0.20999	0	0	3	43
GO:0009986 cell surface	25	0.04337	7.418	0.026	Cellular C	GO:0009	cell surface	0.04337	0.21739	0	0	25	963
GO:0014069 postsynaptic density	11	0.04348	3.264	0.033	Cellular C	GO:0014	postsynaptic density	0.04348	0.21739	0	0	11	338
GO:0045936 negative regulation of phosphate metabolic process	13	0.04364	4.025	0.031	Biological	GO:0045	negative regulation of phosphate metabolic process	0.04364	0.21119	0	0	13	418
GO:0140678 molecular function inhibitor activity	7	0.04375	2.047	0.04	Molecular	GO:0140	molecular function inhibitor activity	0.04375	0.22088	0	0	7	174
GO:1904888 cranial skeletal system development	4	0.04428	1.238	0.055	Biological	GO:1904	cranial skeletal system development	0.04428	0.21391	0	0	4	73
GO:0010563 negative regulation of phosphorus metabolic process	13	0.04433	4.025	0.031	Biological	GO:0010	negative regulation of phosphorus metabolic process	0.04433	0.21391	0	0	13	419
GO:0030099 myeloid cell differentiation	13	0.04433	4.025	0.031	Biological	GO:0030	myeloid cell differentiation	0.04433	0.21391	0	0	13	419
GO:0071347 cellular response to interleukin-1	5	0.04465	1.548	0.047	Biological	GO:0071	cellular response to interleukin-1	0.04465	0.21521	0	0	5	106
GO:0001046 core promoter sequence-specific DNA binding	3	0.04517	0.877	0.07	Molecular	GO:0001	core promoter sequence-specific DNA binding	0.04517	0.22088	0	0	3	43
GO:0032433 filopodium tip	2	0.04527	0.593	0.105	Cellular C	GO:0032	filopodium tip	0.04527	0.2189	0	0	2	19
GO:0044305 calyx of Held	2	0.04527	0.593	0.105	Cellular C	GO:0044	calyx of Held	0.04527	0.2189	0	0	2	19
GO:0010628 positive regulation of gene expression	29	0.04531	8.978	0.025	Biological	GO:0010	positive regulation of gene expression	0.04531	0.21785	0	0	29	1144
GO:0070727 cellular macromolecule localization	41	0.04533	12.693	0.024	Biological	GO:0070	cellular macromolecule localization	0.04533	0.21785	0	0	41	1720
GO:0051402 neuron apoptotic process	9	0.04565	2.786	0.035	Biological	GO:0051	neuron apoptotic process	0.04565	0.21785	0	0	9	256
GO:1901652 response to peptide	15	0.04577	4.644	0.03	Biological	GO:1901	response to peptide	0.04577	0.21785	0	0	15	507
GO:0032434 regulation of proteasomal ubiquitin-dependent protein cata	6	0.0459	1.858	0.042	Biological	GO:0032	regulation of proteasomal ubiquitin-dependent protein catabolic proce	0.0459	0.21785	0	0	6	142
GO:0034614 cellular response to reactive oxygen species	6	0.0459	1.858	0.042	Biological	GO:0034	cellular response to reactive oxygen species	0.0459	0.21785	0	0	6	142
GO:0034754 cellular hormone metabolic process	6	0.0459	1.858	0.042	Biological	GO:0034	cellular hormone metabolic process	0.0459	0.21785	0	0	6	142
GO:0031952 regulation of protein autophosphorylation	3	0.04592	0.929	0.068	Biological	GO:0031	regulation of protein autophosphorylation	0.04592	0.21785	0	0	3	44
GO:0045214 sarcomere organization	3	0.04592	0.929	0.068	Biological	GO:0045	sarcomere organization	0.04592	0.21785	0	0	3	44
GO:0034097 response to cytokine	24	0.04593	7.43	0.026	Biological	GO:0034	response to cytokine	0.04593	0.21785	0	0	24	912
GO:0019222 regulation of metabolic process	138	0.04601	42.724	0.02	Biological	GO:0019	regulation of metabolic process	0.04601	0.21785	0	0	138	6740
GO:0005515 protein binding	288	0.04609	84.211	0.019	Molecular	GO:0005	protein binding	0.04609	0.22088	0	0	288	14863
GO:0003184 pulmonary valve morphogenesis	2	0.0462	0.619	0.105	Biological	GO:0003	pulmonary valve morphogenesis	0.0462	0.21785	0	0	2	19
GO:0010002 cardioblast differentiation	2	0.0462	0.619	0.105	Biological	GO:0010	cardioblast differentiation	0.0462	0.21785	0	0	2	19
GO:0032717 negative regulation of interleukin-8 production	2	0.0462	0.619	0.105	Biological	GO:0032	negative regulation of interleukin-8 production	0.0462	0.21785	0	0	2	19
GO:0045780 positive regulation of bone resorption	2	0.0462	0.619	0.105	Biological	GO:0045	positive regulation of bone resorption	0.0462	0.21785	0	0	2	19
GO:0055003 cardiac myofibril assembly	2	0.0462	0.619	0.105	Biological	GO:0055	cardiac myofibril assembly	0.0462	0.21785	0	0	2	19
GO:0060438 trachea development	2	0.0462	0.619	0.105	Biological	GO:0060	trachea development	0.0462	0.21785	0	0	2	19
GO:0060602 branch elongation of an epithelium	2	0.0462	0.619	0.105	Biological	GO:0060	branch elongation of an epithelium	0.0462	0.21785	0	0	2	19
GO:0061323 cell proliferation involved in heart morphogenesis	2	0.0462	0.619	0.105	Biological	GO:0061	cell proliferation involved in heart morphogenesis	0.0462	0.21785	0	0	2	19
GO:0061744 motor behavior	2	0.0462	0.619	0.105	Biological	GO:0061	motor behavior	0.0462	0.21785	0	0	2	19
GO:0072074 kidney mesenchyme development	2	0.0462	0.619	0.105	Biological	GO:0072	kidney mesenchyme development	0.0462	0.21785	0	0	2	19
GO:0090190 positive regulation of branching involved in ureteric bud mo	2	0.0462	0.619	0.105	Biological	GO:0090	positive regulation of branching involved in ureteric bud morphogenes	0.0462	0.21785	0	0	2	19
GO:1901522 positive regulation of transcription from RNA polymerase II	2	0.0462	0.619	0.105	Biological	GO:1901	positive regulation of transcription from RNA polymerase II promoter	0.0462	0.21785	0	0	2	19
GO:0062197 cellular response to chemical stress	11	0.04639	3.406	0.033	Biological	GO:0062	cellular response to chemical stress	0.04639	0.21857	0	0	11	338
GO:0009987 cellular process	313	0.04741	96.904	0.019	Biological	GO:0009	cellular process	0.04741	0.22168	0	0	313	16795
GO:0031090 organelle membrane	80	0.04745	23.739	0.021	Cellular C	GO:0031	organelle membrane	0.04745	0.2189	0	0	80	3728
GO:0051130 positive regulation of cellular component organization	28	0.04751	8.669	0.025	Biological	GO:0051	positive regulation of cellular component organization	0.04751	0.22168	0	0	28	1102
GO:0004303 estradiol 17-beta-dehydrogenase activity	2	0.0476	0.585	0.105	Molecular	GO:0004	estradiol 17-beta-dehydrogenase activity	0.0476	0.22088	0	0	2	19
GO:0016918 retinal binding	2	0.0476	0.585	0.105	Molecular	GO:0016	retinal binding	0.0476	0.22088	0	0	2	19
GO:0042698 ovulation cycle	4	0.0481	1.238	0.053	Biological	GO:0042	ovulation cycle	0.0481	0.22168	0	0	4	75
GO:0050688 regulation of defense response to virus	4	0.0481	1.238	0.053	Biological	GO:0050	regulation of defense response to virus	0.0481	0.22168	0	0	4	75
GO:0071230 cellular response to amino acid stimulus	4	0.0481	1.238	0.053	Biological	GO:0071	cellular response to amino acid stimulus	0.0481	0.22168	0	0	4	75
GO:0071260 cellular response to mechanical stimulus	4	0.0481	1.238	0.053	Biological	GO:0071	cellular response to mechanical stimulus	0.0481	0.22168	0	0	4	75
GO:0005794 Golgi apparatus	39	0.04815	11.573	0.024	Cellular C	GO:0005	Golgi apparatus	0.04815	0.2189	0	0	39	1648
GO:0050921 positive regulation of chemotaxis	6	0.04854	1.858	0.042	Biological	GO:0050	positive regulation of chemotaxis	0.04854	0.22168	0	0	6	144
GO:0006858 extracellular transport	3	0.04856	0.929	0.067	Biological	GO:0006	extracellular transport	0.04856	0.22168	0	0	3	45
GO:0010837 regulation of keratinocyte proliferation	3	0.04856	0.929	0.067	Biological	GO:0010	regulation of keratinocyte proliferation	0.04856	0.22168	0	0	3	45
GO:0031295 T cell costimulation	3	0.04856	0.929	0.067	Biological	GO:0031	T cell costimulation	0.04856	0.22168	0	0	3	45
GO:0099080 supramolecular complex	34	0.04941	10.089	0.024	Cellular C	GO:0099	supramolecular complex	0.04941	0.2189	0	0	34	1407
GO:0048585 negative regulation of response to stimulus	39	0.04945	12.074	0.024	Biological	GO:0048	negative regulation of response to stimulus	0.04945	0.22168	0	0	39	1634
GO:0032426 stereocilium tip	2	0.04972	0.593	0.1	Cellular C	GO:0032	stereocilium tip	0.04972	0.2189	0	0	2	20
GO:0097449 astrocyte projection	2	0.04972	0.593	0.1	Cellular C	GO:0097	astrocyte projection	0.04972	0.2189	0	0	2	20
GO:0050714 positive regulation of protein secretion	6	0.0499	1.858	0.041	Biological	GO:0050	positive regulation of protein secretion	0.0499	0.22168	0	0	6	145
GO:0070372 regulation of ERK1 and ERK2 cascade	10	0.04994	3.096	0.033	Biological	GO:0070	regulation of ERK1 and ERK2 cascade	0.04994	0.22168	0	0	10	301
GO:0045661 regulation of myoblast differentiation	4	0.05007	1.238	0.053	Biological	GO:0045	regulation of myoblast differentiation	0.05007	0.22168	0	0	4	76
GO:0043434 response to peptide hormone	13	0.05014	4.025	0.03	Biological	GO:0043	response to peptide hormone	0.05014	0.22168	0	0	13	427
GO:0030863 cortical cytoskeleton	5	0.05055	1.484	0.045	Cellular C	GO:0030	cortical cytoskeleton	0.05055	0.2189	0	0	5	111
GO:0002888 positive regulation of myeloid leukocyte mediated immunity	2	0.05073	0.619	0.1	Biological	GO:0002	positive regulation of myeloid leukocyte mediated immunity	0.05073	0.22168	0	0	2	20
GO:0016137 glycoside metabolic process	2	0.05073	0.619	0.1	Biological	GO:0016	glycoside metabolic process	0.05073	0.22168	0	0	2	20
GO:0021516 dorsal spinal cord development	2	0.05073	0.619	0.1	Biological	GO:0021	dorsal spinal cord development	0.05073	0.22168	0	0	2	20
GO:0035813 regulation of renal sodium excretion	2	0.05073	0.619	0.1	Biological	GO:0035	regulation of renal sodium excretion	0.05073	0.22168	0	0	2	20

GO:0036342 post-anal tail morphogenesis	2	0.05073	0.619	0.1	Biological	GO:00363	post-anal tail morphogenesis	0.05073	0.22168	0	0	2	20
GO:0042474 middle ear morphogenesis	2	0.05073	0.619	0.1	Biological	GO:00424	middle ear morphogenesis	0.05073	0.22168	0	0	2	20
GO:0043586 tongue development	2	0.05073	0.619	0.1	Biological	GO:00435	tongue development	0.05073	0.22168	0	0	2	20
GO:0055064 chloride ion homeostasis	2	0.05073	0.619	0.1	Biological	GO:00550	chloride ion homeostasis	0.05073	0.22168	0	0	2	20
GO:0055083 monovalent inorganic anion homeostasis	2	0.05073	0.619	0.1	Biological	GO:00550	monovalent inorganic anion homeostasis	0.05073	0.22168	0	0	2	20
GO:0060973 cell migration involved in heart development	2	0.05073	0.619	0.1	Biological	GO:00609	cell migration involved in heart development	0.05073	0.22168	0	0	2	20
GO:0071498 cellular response to fluid shear stress	2	0.05073	0.619	0.1	Biological	GO:00714	cellular response to fluid shear stress	0.05073	0.22168	0	0	2	20
GO:0072079 nephron tubule formation	2	0.05073	0.619	0.1	Biological	GO:00720	nephron tubule formation	0.05073	0.22168	0	0	2	20
GO:0099515 actin filament-based transport	2	0.05073	0.619	0.1	Biological	GO:00995	actin filament-based transport	0.05073	0.22168	0	0	2	20
GO:0060402 calcium ion transport into cytosol	7	0.05079	2.167	0.038	Biological	GO:00604	calcium ion transport into cytosol	0.05079	0.22168	0	0	7	183
GO:0050804 modulation of chemical synaptic transmission	14	0.05082	4.334	0.03	Biological	GO:00508	modulation of chemical synaptic transmission	0.05082	0.22168	0	0	14	471
GO:0004252 serine-type endopeptidase activity	7	0.05083	2.047	0.039	Molecular	GO:00042	serine-type endopeptidase activity	0.05083	0.22088	0	0	7	180
GO:0006939 smooth muscle contraction	5	0.05091	1.548	0.045	Biological	GO:00069	smooth muscle contraction	0.05091	0.22168	0	0	5	110
GO:1901655 cellular response to ketone	5	0.05091	1.548	0.045	Biological	GO:19016	cellular response to ketone	0.05091	0.22168	0	0	5	110
GO:0014075 response to amine	3	0.05127	0.929	0.065	Biological	GO:00140	response to amine	0.05127	0.22168	0	0	3	46
GO:0014904 myotube cell development	3	0.05127	0.929	0.065	Biological	GO:00149	myotube cell development	0.05127	0.22168	0	0	3	46
GO:0042596 fear response	3	0.05127	0.929	0.065	Biological	GO:00425	fear response	0.05127	0.22168	0	0	3	46
GO:1900271 regulation of long-term synaptic potentiation	3	0.05127	0.929	0.065	Biological	GO:19002	regulation of long-term synaptic potentiation	0.05127	0.22168	0	0	3	46
GO:1990090 cellular response to nerve growth factor stimulus	3	0.05127	0.929	0.065	Biological	GO:19900	cellular response to nerve growth factor stimulus	0.05127	0.22168	0	0	3	46
GO:0006865 amino acid transport	6	0.05128	1.858	0.041	Biological	GO:00068	amino acid transport	0.05128	0.22168	0	0	6	146
GO:0007272 ensheathment of neurons	6	0.05128	1.858	0.041	Biological	GO:00072	ensheathment of neurons	0.05128	0.22168	0	0	6	146
GO:0008366 axon ensheathment	6	0.05128	1.858	0.041	Biological	GO:00083	axon ensheathment	0.05128	0.22168	0	0	6	146
GO:0090101 negative regulation of transmembrane receptor protein ser	6	0.05128	1.858	0.041	Biological	GO:00901	negative regulation of transmembrane receptor protein serine/threoni	0.05128	0.22168	0	0	6	146
GO:0099177 regulation of trans-synaptic signaling	14	0.05155	4.334	0.03	Biological	GO:00991	regulation of trans-synaptic signaling	0.05155	0.22168	0	0	14	472
GO:0019538 protein metabolic process	113	0.05161	34.985	0.021	Biological	GO:00195	protein metabolic process	0.05161	0.22168	0	0	113	5432
GO:0050954 sensory perception of mechanical stimulus	7	0.05202	2.167	0.038	Biological	GO:00509	sensory perception of mechanical stimulus	0.05202	0.22168	0	0	7	184
GO:0043412 macromolecule modification	83	0.05223	25.697	0.021	Biological	GO:00434	macromolecule modification	0.05223	0.22168	0	0	83	3863
GO:0004653 polypeptide N-acetylgalactosaminyltransferase activity	2	0.05225	0.585	0.1	Molecular	GO:00046	polypeptide N-acetylgalactosaminyltransferase activity	0.05225	0.22088	0	0	2	20
GO:0005123 death receptor binding	2	0.05225	0.585	0.1	Molecular	GO:00051	death receptor binding	0.05225	0.22088	0	0	2	20
GO:0005283 amino acid:sodium symporter activity	2	0.05225	0.585	0.1	Molecular	GO:00052	amino acid:sodium symporter activity	0.05225	0.22088	0	0	2	20
GO:0001708 cell fate specification	5	0.05255	1.548	0.045	Biological	GO:00017	cell fate specification	0.05255	0.22168	0	0	5	111
GO:0007200 phospholipase C-activating G protein-coupled receptor sig	5	0.05255	1.548	0.045	Biological	GO:00072	phospholipase C-activating G protein-coupled receptor signaling path	0.05255	0.22168	0	0	5	111
GO:0030148 sphingolipid biosynthetic process	5	0.05255	1.548	0.045	Biological	GO:00301	sphingolipid biosynthetic process	0.05255	0.22168	0	0	5	111
GO:0034446 substrate adhesion-dependent cell spreading	5	0.05255	1.548	0.045	Biological	GO:00344	substrate adhesion-dependent cell spreading	0.05255	0.22168	0	0	5	111
GO:0000122 negative regulation of transcription by RNA polymerase II	25	0.05262	7.74	0.026	Biological	GO:00001	negative regulation of transcription by RNA polymerase II	0.05262	0.22168	0	0	25	972
GO:0014074 response to purine-containing compound	6	0.05268	1.858	0.041	Biological	GO:00140	response to purine-containing compound	0.05268	0.22168	0	0	6	147
GO:0005896 interleukin-6 receptor complex	1	0.05311	0.297	0.333	Cellular C	GO:00058	interleukin-6 receptor complex	0.05311	0.2189	0	0	1	3
GO:0016513 core-binding factor complex	1	0.05311	0.297	0.333	Cellular C	GO:00165	core-binding factor complex	0.05311	0.2189	0	0	1	3
GO:0030485 smooth muscle contractile fiber	1	0.05311	0.297	0.333	Cellular C	GO:00304	smooth muscle contractile fiber	0.05311	0.2189	0	0	1	3
GO:0035866 alphav-beta3 integrin-PKCalpha complex	1	0.05311	0.297	0.333	Cellular C	GO:00358	alphav-beta3 integrin-PKCalpha complex	0.05311	0.2189	0	0	1	3
GO:0042825 TAP complex	1	0.05311	0.297	0.333	Cellular C	GO:00428	TAP complex	0.05311	0.2189	0	0	1	3
GO:0048269 methionine adenosyltransferase complex	1	0.05311	0.297	0.333	Cellular C	GO:00482	methionine adenosyltransferase complex	0.05311	0.2189	0	0	1	3
GO:0070110 ciliary neurotrophic factor receptor complex	1	0.05311	0.297	0.333	Cellular C	GO:00701	ciliary neurotrophic factor receptor complex	0.05311	0.2189	0	0	1	3
GO:0070721 ISGF3 complex	1	0.05311	0.297	0.333	Cellular C	GO:00707	ISGF3 complex	0.05311	0.2189	0	0	1	3
GO:0090651 apical cytoplasm	1	0.05311	0.297	0.333	Cellular C	GO:00906	apical cytoplasm	0.05311	0.2189	0	0	1	3
GO:0097451 glial limiting end-foot	1	0.05311	0.297	0.333	Cellular C	GO:00974	glial limiting end-foot	0.05311	0.2189	0	0	1	3
GO:0099544 perisynaptic space	1	0.05311	0.297	0.333	Cellular C	GO:00995	perisynaptic space	0.05311	0.2189	0	0	1	3
GO:1901588 dendritic microtubule	1	0.05311	0.297	0.333	Cellular C	GO:19015	dendritic microtubule	0.05311	0.2189	0	0	1	3
GO:1905721 mitotic spindle astral microtubule end	1	0.05311	0.297	0.333	Cellular C	GO:19057	mitotic spindle astral microtubule end	0.05311	0.2189	0	0	1	3
GO:0007565 female pregnancy	7	0.05326	2.167	0.038	Biological	GO:00075	female pregnancy	0.05326	0.22168	0	0	7	185
GO:0008194 UDP-glycosyltransferase activity	6	0.0533	1.754	0.041	Molecular	GO:00081	UDP-glycosyltransferase activity	0.0533	0.22088	0	0	6	145
GO:0001555 oocyte growth	1	0.0537	0.31	0.333	Biological	GO:00015	oocyte growth	0.0537	0.22168	0	0	1	3
GO:0001798 positive regulation of type IIa hypersensitivity	1	0.0537	0.31	0.333	Biological	GO:00017	positive regulation of type IIa hypersensitivity	0.0537	0.22168	0	0	1	3
GO:0001905 activation of membrane attack complex	1	0.0537	0.31	0.333	Biological	GO:00019	activation of membrane attack complex	0.0537	0.22168	0	0	1	3
GO:0001969 regulation of activation of membrane attack complex	1	0.0537	0.31	0.333	Biological	GO:00019	regulation of activation of membrane attack complex	0.0537	0.22168	0	0	1	3
GO:0002074 extraocular skeletal muscle development	1	0.0537	0.31	0.333	Biological	GO:00020	extraocular skeletal muscle development	0.0537	0.22168	0	0	1	3
GO:0002157 positive regulation of thyroid hormone mediated signaling p	1	0.0537	0.31	0.333	Biological	GO:00021	positive regulation of thyroid hormone mediated signaling pathway	0.0537	0.22168	0	0	1	3
GO:0002541 activation of plasma proteins involved in acute inflammator	1	0.0537	0.31	0.333	Biological	GO:00025	activation of plasma proteins involved in acute inflammatory response	0.0537	0.22168	0	0	1	3
GO:0002894 positive regulation of type II hypersensitivity	1	0.0537	0.31	0.333	Biological	GO:00028	positive regulation of type II hypersensitivity	0.0537	0.22168	0	0	1	3
GO:0003220 left ventricular cardiac muscle tissue morphogenesis	1	0.0537	0.31	0.333	Biological	GO:00032	left ventricular cardiac muscle tissue morphogenesis	0.0537	0.22168	0	0	1	3
GO:0003275 apoptotic process involved in outflow tract morphogenesis	1	0.0537	0.31	0.333	Biological	GO:00032	apoptotic process involved in outflow tract morphogenesis	0.0537	0.22168	0	0	1	3
GO:0003430 growth plate cartilage chondrocyte growth	1	0.0537	0.31	0.333	Biological	GO:00034	growth plate cartilage chondrocyte growth	0.0537	0.22168	0	0	1	3
GO:0006679 glucosylceramide biosynthetic process	1	0.0537	0.31	0.333	Biological	GO:00066	glucosylceramide biosynthetic process	0.0537	0.22168	0	0	1	3
GO:0006714 sesquiterpenoid metabolic process	1	0.0537	0.31	0.333	Biological	GO:00067	sesquiterpenoid metabolic process	0.0537	0.22168	0	0	1	3
GO:0007538 primary sex determination	1	0.0537	0.31	0.333	Biological	GO:00075	primary sex determination	0.0537	0.22168	0	0	1	3
GO:0007619 courtship behavior	1	0.0537	0.31	0.333	Biological	GO:00076	courtship behavior	0.0537	0.22168	0	0	1	3
GO:0010693 negative regulation of alkaline phosphatase activity	1	0.0537	0.31	0.333	Biological	GO:00106	negative regulation of alkaline phosphatase activity	0.0537	0.22168	0	0	1	3
GO:0010716 negative regulation of extracellular matrix disassembly	1	0.0537	0.31	0.333	Biological	GO:00107	negative regulation of extracellular matrix disassembly	0.0537	0.22168	0	0	1	3
GO:0014043 negative regulation of neuron maturation	1	0.0537	0.31	0.333	Biological	GO:00140	negative regulation of neuron maturation	0.0537	0.22168	0	0	1	3
GO:0014734 skeletal muscle hypertrophy	1	0.0537	0.31	0.333	Biological	GO:00147	skeletal muscle hypertrophy	0.0537	0.22168	0	0	1	3
GO:0021538 epithalamus development	1	0.0537	0.31	0.333	Biological	GO:00215	epithalamus development	0.0537	0.22168	0	0	1	3
GO:0021551 central nervous system morphogenesis	1	0.0537	0.31	0.333	Biological	GO:00215	central nervous system morphogenesis	0.0537	0.22168	0	0	1	3
GO:0021555 midbrain-hindbrain boundary morphogenesis	1	0.0537	0.31	0.333	Biological	GO:00215	midbrain-hindbrain boundary morphogenesis	0.0537	0.22168	0	0	1	3
GO:0021627 olfactory nerve morphogenesis	1	0.0537	0.31	0.333	Biological	GO:00216	olfactory nerve morphogenesis	0.0537	0.22168	0	0	1	3
GO:0021986 habenula development	1	0.0537	0.31	0.333	Biological	GO:00219	habenula development	0.0537	0.22168	0	0	1	3
GO:0030910 olfactory placode formation	1	0.0537	0.31	0.333	Biological	GO:00309	olfactory placode formation	0.0537	0.22168	0	0	1	3
GO:0032805 positive regulation of low-density lipoprotein particle recept	1	0.0537	0.31	0.333	Biological	GO:00328	positive regulation of low-density lipoprotein particle receptor cataboli	0.0537	0.22168	0	0	1	3
GO:0032899 regulation of neurotrophin production	1	0.0537	0.31	0.333	Biological	GO:00328	regulation of neurotrophin production	0.0537	0.22168	0	0	1	3
GO:0033024 mast cell apoptotic process	1	0.0537	0.31	0.333	Biological	GO:00330	mast cell apoptotic process	0.0537	0.22168	0	0	1	3
GO:0033025 regulation of mast cell apoptotic process	1	0.0537	0.31	0.333	Biological	GO:00330	regulation of mast cell apoptotic process	0.0537	0.22168	0	0	1	3
GO:0035702 monocyte homeostasis	1	0.0537	0.31	0.333	Biological	GO:00357	monocyte homeostasis	0.0537	0.22168	0	0	1	3
GO:0035846 oviduct epithelium development	1	0.0537	0.31	0.333	Biological	GO:00358	oviduct epithelium development	0.0537	0.22168	0	0	1	3
GO:0036514 dopaminergic neuron axon guidance	1	0.0537	0.31	0.333	Biological	GO:00365	dopaminergic neuron axon guidance	0.0537	0.22168	0	0	1	3
GO:0038154 interleukin-11-mediated signaling pathway	1	0.0537	0.31	0.333	Biological	GO:00381	interleukin-11-mediated signaling pathway	0.0537	0.22168	0	0	1	3
GO:0042305 specification of segmental identity, mandibular segment	1	0.0537	0.31	0.333	Biological	GO:00423	specification of segmental identity, mandibular segment	0.0537	0.22168	0	0	1	3
GO:0042883 cysteine transport	1	0.0537	0.31	0.333	Biological	GO:00428	cysteine transport	0.0537	0.22168	0	0	1	3
GO:0045218 zonula adherens maintenance	1	0.0537	0.31	0.333	Biological	GO:00452	zonula adherens maintenance	0.0537	0.22168	0	0	1	3
GO:0048769 sarcomerogenesis	1	0.0537	0.31	0.333	Biological	GO:00487	sarcomerogenesis	0.0537	0.22168	0	0	1	3
GO:0051790 short-chain fatty acid biosynthetic process	1	0.0537	0.31	0.333	Biological	GO:00517	short-chain fatty acid biosynthetic process	0.0537	0.22168	0	0	1	3
GO:0051795 positive regulation of timing of catagen	1	0.0537	0.31	0.333	Biological	GO:00517	positive regulation of timing of catagen	0.0537	0.22168	0	0	1	3
GO:0060086 circadian temperature homeostasis	1	0.0537	0.31	0.333	Biological	GO:00600	circadian temperature homeostasis	0.0537	0.22168	0	0	1	3
GO:0060166 olfactory pit development	1	0.0537	0.31	0.333	Biological	GO:00601	olfactory pit development	0.0537	0.22168	0	0	1	3
GO:0060281 regulation of oocyte development	1	0.0537	0.31	0.333	Biological	GO:00602	regulation of oocyte development	0.0537	0.22168	0	0	1	3
GO:0060743 epithelial cell maturation involved in prostate gland develop	1	0.0537	0.31	0.333	Biological	GO:00607	epithelial cell maturation involved in prostate gland development	0.0537	0.22168	0	0	1	3
GO:0060748 tertiary branching involved in mammary gland duct morpho	1	0.0537	0.31	0.333	Biological	GO:00607	tertiary branching involved in mammary gland duct morphogenesis	0.0537	0.22168	0	0	1	3
GO:0060769 positive regulation of epithelial cell proliferation involved in	1	0.0537	0.31	0.333	Biological	GO:00607	positive regulation of epithelial cell proliferation involved in prostate gl	0.0537	0.22168	0	0	1	3
GO:0060838 lymphatic endothelial cell fate commitment	1	0.0537	0.31	0.333	Biological	GO:00608	lymphatic endothelial cell fate commitment	0.0537	0.22168	0	0	1	3
GO:0061110 dense core granule biogenesis	1	0.0537	0.31	0.333	Biological	GO:00611	dense core granule biogenesis	0.0537	0.22168	0	0	1	3
GO:0061171 establishment of bipolar cell polarity	1	0.0537	0.31	0.333	Biological	GO:00611	establishment of bipolar cell polarity	0.0537	0.22168	0	0	1	3
GO:0061519 macrophage homeostasis	1	0.0537	0.31	0.333	Biological	GO:00615	macrophage homeostasis	0.0537	0.22168	0	0	1	3
GO:0061713 anterior neural tube closure	1	0.0537	0.31	0.333	Biological	GO:00617	anterior neural tube closure	0.0537	0.22168	0	0	1	3
GO:0061771 response to caloric restriction	1	0.0537	0.31	0.333	Biological	GO:00617	response to caloric restriction	0.0537	0.22168	0	0	1	3
GO:0070075 tear secretion	1	0.0537	0.31	0.333	Biological	GO:00700	tear secretion	0.0537	0.22168	0	0	1	3
GO:0071288 cellular response to mercury ion	1	0.0537	0.31	0.333	Biological	GO:00712	cellular response to mercury ion	0.0537	0.22168	0	0	1	3
GO:0071698 olfactory placode development	1	0.0537	0.31	0.333	Biological	GO:00716	olfactory placode development	0.0537	0.22168	0	0	1	3
GO:0071699 olfactory placode morphogenesis	1	0.0537	0.31	0.333	Biological	GO:00716	olfactory placode morphogenesis	0.0537	0.22168	0	0	1	3
GO:0072034 renal vesicle induction	1	0.0537	0.31	0.333	Biological	GO:00720	renal vesicle induction	0.0537	0.22168	0	0	1	3
GO:0072092 ureteric bud invasion	1	0.0537	0.31	0.333	Biological	GO:00720	ureteric bud invasion	0.0537	0.22168	0	0	1	3
GO:0072135 kidney mesenchymal cell proliferation	1	0.0537	0.31	0.333	Biological	GO:00721	kidney mesenchymal cell proliferation	0.0537	0.22168	0	0	1	3
GO:0072136 metanephric mesenchymal cell proliferation involved in me	1	0.0537	0.31	0.333	Biological	GO:00721	metanephric mesenchymal cell proliferation involved in metanephros	0.0537	0.22168	0	0	1	3
GO:0072190 ureter urothelium development	1	0.0537	0.31	0.333	Biological	GO:00721	ureter urothelium development	0.0537	0.22168	0	0	1	3
GO:0072198 mesenchymal cell proliferation involved in ureter developm	1	0.0537	0.31	0.333	Biological	GO:00721	mesenchymal cell proliferation involved in ureter development	0.0537	0.22168	0	0	1	3
GO:0072199 regulation of mesenchymal cell proliferation involved in ure	1	0.0537	0.31	0.333	Biological	GO:00721	regulation of mesenchymal cell proliferation involved in ureter develop	0.0537	0.22168	0	0	1	3
GO:0072262 metanephric glomerular mesangial cell proliferation involve	1	0.0537	0.31	0.333	Biological	GO:00722	metanephric glomerular mesangial cell proliferation involved in metan	0.0537	0.22168	0	0	1	3
GO:0072513 positive regulation of secondary heart field cardioblast prol	1	0.0537	0.31	0.333	Biological	GO:00725	positive regulation of secondary heart field cardioblast proliferation	0.0537	0.22168	0	0	1	3
GO:0072740 cellular response to anisomycin	1	0.0537	0.31	0.333	Biological	GO:00727	cellular response to anisomycin	0.0537	0.22168	0	0	1	3
GO:0086101 endothelin receptor signaling pathway involved in heart pro	1	0.0537	0.31	0.333	Biological	GO:00861	endothelin receptor signaling pathway involved in heart process	0.0537	0.22168	0	0	1	3
GO:0090360 platelet-derived growth factor production	1	0.0537	0.31	0.333	Biological	GO:00903	platelet-derived growth factor production	0.0537	0.22168	0	0	1	3
GO:0090361 regulation of platelet-derived growth factor production	1	0.0537	0.31	0.333	Biological	GO:00903	regulation of platelet-derived growth factor production	0.0537	0.22168	0	0	1	3
GO:0097324 melanocyte migration	1	0.0537	0.31	0.333	Biological	GO:00973	melanocyte migration	0.0537	0.22168	0	0	1	3
GO:0098758 response to interleukin-8	1	0.0537	0.31	0.333	Biological	GO:00987	response to interleukin-8	0.0537	0.22168	0	0	1	3
GO:0098759 cellular response to interleukin-8	1	0.0537	0.31	0.333	Biological	GO:00987	cellular response to interleukin-8	0.0537	0.22168	0	0	1	3
GO:0099185 postsynaptic intermediate filament cytoskeleton organizatio	1	0.0537	0.31	0.333	Biological	GO:00991	postsynaptic intermediate filament cytoskeleton organization	0.0537	0.22168	0	0	1	3
GO:0106091 glial cell projection elongation	1	0.0537	0.31	0.333	Biological	GO:01060	glial cell projection elongation	0.0537	0.22168	0	0	1	3
GO:0140300 serine import into mitochondrion	1	0.0537	0.31	0.333	Biological	GO:01403	serine import into mitochondrion	0.0537	0.22168	0	0	1	3
GO:0150064 vertebrate eye-specific patterning	1	0.0537	0.31	0.333	Biological	GO:01500	vertebrate eye-specific patterning	0.0537	0.22168	0	0	1	3
GO:1900052 regulation of retinoic acid biosynthetic process	1	0.0537	0.31	0.333	Biological	GO:19000	regulation of retinoic acid biosynthetic process	0.0537	0.22168	0	0	1	3
GO:1900453 negative regulation of long-term synaptic depression	1	0.0537	0.31	0.333	Biological	GO:19004	negative regulation of long-term synaptic depression	0.0537	0.22168	0	0	1	3
GO:1901626 regulation of postsynaptic membrane organization	1	0.0537	0.31	0.333	Biological	GO:19016	regulation of postsynaptic membrane organization	0.0537	0.22168	0	0	1	3
GO:1902256 regulation of apoptotic process involved in outflow tract mo	1	0.0537	0.31	0.333	Biological	GO:19022	regulation of apoptotic process involved in outflow tract morphogenes	0.0537	0.22168	0	0	1	3
GO:1902309 negative regulation of peptidyl-serine dephosphorylation	1	0.0537	0.31	0.333	Biological	GO:19023	negative regulation of peptidyl-serine dephosphorylation	0.0537	0.22168	0	0	1	3
GO:1902951 negative regulation of dendritic spine maintenance	1	0.0537	0.31	0.333	Biological	GO:19029	negative regulation of dendritic spine maintenance	0.0537	0.22168	0	0	1	3
GO:1902988 neurofibrillary tangle assembly	1	0.0537	0.31	0.333	Biological	GO:19029	neurofibrillary tangle assembly	0.0537	0.22168	0	0	1	3
GO:1903385 regulation of homophilic cell adhesion	1	0.0537	0.31	0.333	Biological	GO:19033	regulation of homophilic cell adhesion	0.0537	0.22168	0	0	1	3
GO:1903712 cysteine transmembrane transport	1	0.0537	0.31	0.333	Biological	GO:19037	cysteine transmembrane transport	0.0537	0.22168	0	0	1	3
GO:1903984 positive regulation of TRAIL-activated apoptotic signaling p	1	0.0537	0.31	0.333	Biological	GO:19039	positive regulation of TRAIL-activated apoptotic signaling pathway	0.0537	0.22168	0	0	1	3
GO:1904381 Golgi apparatus mannose trimming	1	0.0537	0.31	0.333	Biological	GO:19043	Golgi apparatus mannose trimming	0.0537	0.22168	0	0	1	3
GO:1904693 midbrain morphogenesis	1	0.0537	0.31	0.333	Biological	GO:19046	midbrain morphogenesis	0.0537	0.22168	0	0	1	3
GO:1904837 beta-catenin-TCF complex assembly	1	0.0537	0.31	0.333	Biological	GO:19048	beta-catenin-TCF complex assembly	0.0537	0.22168	0	0	1	3
GO:1904938 planar cell polarity pathway involved in axon guidance	1	0.0537	0.31	0.333	Biological	GO:19049	planar cell polarity pathway involved in axon guidance	0.0537	0.22168	0	0	1	3
GO:1905242 response to 3,3',5-triiodo-L-thyronine	1	0.0537	0.31	0.333	Biological	GO:19052	response to 3,3',5-triiodo-L-thyronine	0.0537	0.22168	0	0	1	3
GO:1905584 outer hair cell apoptotic process	1	0.0537	0.31	0.333	Biological	GO:19055	outer hair cell apoptotic process	0.0537	0.22168	0	0	1	3
GO:1905879 regulation of oogenesis	1	0.0537	0.31	0.333	Biological	GO:19058	regulation of oogenesis	0.0537	0.22168	0	0	1	3
GO:2000182 regulation of progesterone biosynthetic process	1	0.0537	0.31	0.333	Biological	GO:20001	regulation of progesterone biosynthetic process	0.0537	0.22168	0	0	1	3
GO:2000224 regulation of testosterone biosynthetic process	1	0.0537	0.31	0.333	Biological	GO:20002	regulation of testosterone biosynthetic process	0.0537	0.22168	0	0	1	3
GO:2000233 negative regulation of rRNA processing	1	0.0537	0.31	0.333	Biological	GO:20002	negative regulation of rRNA processing	0.0537	0.22168	0	0	1	3
GO:2000705 regulation of dense core granule biogenesis	1	0.0537	0.31	0.333	Biological	GO:20007	regulation of dense core granule biogenesis	0.0537	0.22168	0	0	1	3
GO:0002673 regulation of acute inflammatory response	3	0.05405	0.929	0.064	Biological	GO:00026	regulation of acute inflammatory response	0.05405	0.22168	0	0	3	47
GO:0009066 aspartate family amino acid metabolic process	3	0.05405	0.929	0.064	Biological	GO:00090	aspartate family amino acid metabolic process	0.05405	0.22168	0	0	3	47
GO:0010259 multicellular organism aging	3	0.05405	0.929	0.064	Biological	GO:00102	multicellular organism aging	0.05405	0.22168	0	0	3	47
GO:0031294 lymphocyte costimulation	3	0.05405	0.929	0.064	Biological	GO:00312	lymphocyte costimulation	0.05405	0.22168	0	0	3	47
GO:0042088 T-helper 1 type immune response	3	0.05405	0.929	0.064	Biological	GO:00420	T-helper 1 type immune response	0.05405	0.22168	0	0	3	47

GO:0050919 negative chemotaxis	3	0.05405	0.929	0.064	Biological	GO:00509	negative chemotaxis	0.05405	0.22168	0	0	3	47
GO:1902622 regulation of neutrophil migration	3	0.05405	0.929	0.064	Biological	GO:19026	regulation of neutrophil migration	0.05405	0.22168	0	0	3	47
GO:1905314 semi-lunar valve development	3	0.05405	0.929	0.064	Biological	GO:19053	semi-lunar valve development	0.05405	0.22168	0	0	3	47
GO:2001222 regulation of neuron migration	3	0.05405	0.929	0.064	Biological	GO:20012	regulation of neuron migration	0.05405	0.22168	0	0	3	47
GO:0030534 adult behavior	6	0.05411	1.858	0.041	Biological	GO:00305	adult behavior	0.05411	0.22168	0	0	6	148
GO:1903364 positive regulation of cellular protein catabolic process	6	0.05411	1.858	0.041	Biological	GO:19033	positive regulation of cellular protein catabolic process	0.05411	0.22168	0	0	6	148
GO:0045445 myoblast differentiation	5	0.05423	1.548	0.045	Biological	GO:00454	myoblast differentiation	0.05423	0.22179	0	0	5	112
GO:2000060 positive regulation of ubiquitin-dependent protein catabolic	5	0.05423	1.548	0.045	Biological	GO:20000	positive regulation of ubiquitin-dependent protein catabolic process	0.05423	0.22179	0	0	5	112
GO:0043066 negative regulation of apoptotic process	23	0.05428	7.121	0.026	Biological	GO:00430	negative regulation of apoptotic process	0.05428	0.22185	0	0	23	882
GO:0034361 very-low-density lipoprotein particle	2	0.05431	0.593	0.095	Cellular C	GO:00343	very-low-density lipoprotein particle	0.05431	0.2189	0	0	2	21
GO:0034385 triglyceride-rich plasma lipoprotein particle	2	0.05431	0.593	0.095	Cellular C	GO:00343	triglyceride-rich plasma lipoprotein particle	0.05431	0.2189	0	0	2	21
GO:0042827 platelet dense granule	2	0.05431	0.593	0.095	Cellular C	GO:00428	platelet dense granule	0.05431	0.2189	0	0	2	21
GO:0032432 actin filament bundle	4	0.05442	1.187	0.051	Cellular C	GO:00324	actin filament bundle	0.05442	0.2189	0	0	4	79
GO:0042641 actomyosin	4	0.05442	1.187	0.051	Cellular C	GO:00426	actomyosin	0.05442	0.2189	0	0	4	79
GO:0000293 ferric-chelate reductase activity	1	0.05458	0.292	0.333	Molecular	GO:00002	ferric-chelate reductase activity	0.05458	0.22088	0	0	1	3
GO:0003829 beta-1,3-galactosyl-O-glycosyl-glycoprotein beta-1,6-N-ac	1	0.05458	0.292	0.333	Molecular	GO:00038	beta-1,3-galactosyl-O-glycosyl-glycoprotein beta-1,6-N-acetylglucosa	0.05458	0.22088	0	0	1	3
GO:0004145 diamine N-acetyltransferase activity	1	0.05458	0.292	0.333	Molecular	GO:00041	diamine N-acetyltransferase activity	0.05458	0.22088	0	0	1	3
GO:0004699 calcium-independent protein kinase C activity	1	0.05458	0.292	0.333	Molecular	GO:00046	calcium-independent protein kinase C activity	0.05458	0.22088	0	0	1	3
GO:0004768 stearoyl-CoA 9-desaturase activity	1	0.05458	0.292	0.333	Molecular	GO:00047	stearoyl-CoA 9-desaturase activity	0.05458	0.22088	0	0	1	3
GO:0004833 tryptophan 2,3-dioxygenase activity	1	0.05458	0.292	0.333	Molecular	GO:00048	tryptophan 2,3-dioxygenase activity	0.05458	0.22088	0	0	1	3
GO:0004921 interleukin-11 receptor activity	1	0.05458	0.292	0.333	Molecular	GO:00049	interleukin-11 receptor activity	0.05458	0.22088	0	0	1	3
GO:0005006 epidermal growth factor-activated receptor activity	1	0.05458	0.292	0.333	Molecular	GO:00050	epidermal growth factor-activated receptor activity	0.05458	0.22088	0	0	1	3
GO:0005010 insulin-like growth factor-activated receptor activity	1	0.05458	0.292	0.333	Molecular	GO:00050	insulin-like growth factor-activated receptor activity	0.05458	0.22088	0	0	1	3
GO:0005157 macrophage colony-stimulating factor receptor binding	1	0.05458	0.292	0.333	Molecular	GO:00051	macrophage colony-stimulating factor receptor binding	0.05458	0.22088	0	0	1	3
GO:0005220 inositol 1,4,5-trisphosphate-sensitive calcium-release char	1	0.05458	0.292	0.333	Molecular	GO:00052	inositol 1,4,5-trisphosphate-sensitive calcium-release channel activity	0.05458	0.22088	0	0	1	3
GO:0008109 N-acetyllactosaminide beta-1,6-N-acetylglucosaminyltrans	1	0.05458	0.292	0.333	Molecular	GO:00081	N-acetyllactosaminide beta-1,6-N-acetylglucosaminyltransferase acti	0.05458	0.22088	0	0	1	3
GO:0008427 calcium-dependent protein kinase inhibitor activity	1	0.05458	0.292	0.333	Molecular	GO:00084	calcium-dependent protein kinase inhibitor activity	0.05458	0.22088	0	0	1	3
GO:0008823 cupric reductase activity	1	0.05458	0.292	0.333	Molecular	GO:00088	cupric reductase activity	0.05458	0.22088	0	0	1	3
GO:0010858 calcium-dependent protein kinase regulator activity	1	0.05458	0.292	0.333	Molecular	GO:00108	calcium-dependent protein kinase regulator activity	0.05458	0.22088	0	0	1	3
GO:0016151 nickel cation binding	1	0.05458	0.292	0.333	Molecular	GO:00161	nickel cation binding	0.05458	0.22088	0	0	1	3
GO:0016657 oxidoreductase activity, acting on NAD(P)H, nitrogenous g	1	0.05458	0.292	0.333	Molecular	GO:00166	oxidoreductase activity, acting on NAD(P)H, nitrogenous group as ac	0.05458	0.22088	0	0	1	3
GO:0016920 pyroglutamyl-peptidase activity	1	0.05458	0.292	0.333	Molecular	GO:00169	pyroglutamyl-peptidase activity	0.05458	0.22088	0	0	1	3
GO:0017020 myosin phosphatase regulator activity	1	0.05458	0.292	0.333	Molecular	GO:00170	myosin phosphatase regulator activity	0.05458	0.22088	0	0	1	3
GO:0017061 S-methyl-5-thioadenosine phosphorylase activity	1	0.05458	0.292	0.333	Molecular	GO:00170	S-methyl-5-thioadenosine phosphorylase activity	0.05458	0.22088	0	0	1	3
GO:0019970 interleukin-11 binding	1	0.05458	0.292	0.333	Molecular	GO:00199	interleukin-11 binding	0.05458	0.22088	0	0	1	3
GO:0032129 histone deacetylase activity (H3-K9 specific)	1	0.05458	0.292	0.333	Molecular	GO:00321	histone deacetylase activity (H3-K9 specific)	0.05458	0.22088	0	0	1	3
GO:0033229 cysteine transmembrane transporter activity	1	0.05458	0.292	0.333	Molecular	GO:00332	cysteine transmembrane transporter activity	0.05458	0.22088	0	0	1	3
GO:0034714 type III transforming growth factor beta receptor binding	1	0.05458	0.292	0.333	Molecular	GO:00347	type III transforming growth factor beta receptor binding	0.05458	0.22088	0	0	1	3
GO:0036134 12-hydroxyheptadecatrienoic acid synthase activity	1	0.05458	0.292	0.333	Molecular	GO:00361	12-hydroxyheptadecatrienoic acid synthase activity	0.05458	0.22088	0	0	1	3
GO:0038131 neuregulin receptor activity	1	0.05458	0.292	0.333	Molecular	GO:00381	neuregulin receptor activity	0.05458	0.22088	0	0	1	3
GO:0044729 hemi-methylated DNA-binding	1	0.05458	0.292	0.333	Molecular	GO:00447	hemi-methylated DNA-binding	0.05458	0.22088	0	0	1	3
GO:0045030 G protein-coupled UTP receptor activity	1	0.05458	0.292	0.333	Molecular	GO:00450	G protein-coupled UTP receptor activity	0.05458	0.22088	0	0	1	3
GO:0046979 TAP2 binding	1	0.05458	0.292	0.333	Molecular	GO:00469	TAP2 binding	0.05458	0.22088	0	0	1	3
GO:0047115 trans-1,2-dihydrobenzene-1,2-diol dehydrogenase activity	1	0.05458	0.292	0.333	Molecular	GO:00471	trans-1,2-dihydrobenzene-1,2-diol dehydrogenase activity	0.05458	0.22088	0	0	1	3
GO:0047690 aspartyltransferase activity	1	0.05458	0.292	0.333	Molecular	GO:00476	aspartyltransferase activity	0.05458	0.22088	0	0	1	3
GO:0047718 indanol dehydrogenase activity	1	0.05458	0.292	0.333	Molecular	GO:00477	indanol dehydrogenase activity	0.05458	0.22088	0	0	1	3
GO:0047757 chondroitin-glucuronate 5-epimerase activity	1	0.05458	0.292	0.333	Molecular	GO:00477	chondroitin-glucuronate 5-epimerase activity	0.05458	0.22088	0	0	1	3
GO:0052851 ferric-chelate reductase (NADPH) activity	1	0.05458	0.292	0.333	Molecular	GO:00528	ferric-chelate reductase (NADPH) activity	0.05458	0.22088	0	0	1	3
GO:0070290 N-acylphosphatidylethanolamine-specific phospholipase D	1	0.05458	0.292	0.333	Molecular	GO:00702	N-acylphosphatidylethanolamine-specific phospholipase D activity	0.05458	0.22088	0	0	1	3
GO:0099184 structural constituent of postsynaptic intermediate filament	1	0.05458	0.292	0.333	Molecular	GO:00991	structural constituent of postsynaptic intermediate filament cytoskele	0.05458	0.22088	0	0	1	3
GO:0106130 purine phosphoribosyltransferase activity	1	0.05458	0.292	0.333	Molecular	GO:01061	purine phosphoribosyltransferase activity	0.05458	0.22088	0	0	1	3
GO:0006491 N-glycan processing	2	0.05541	0.619	0.095	Biological	GO:00064	N-glycan processing	0.05541	0.22441	0	0	2	21
GO:0006670 sphingosine metabolic process	2	0.05541	0.619	0.095	Biological	GO:00066	sphingosine metabolic process	0.05541	0.22441	0	0	2	21
GO:0007213 G protein-coupled acetylcholine receptor signaling pathway	2	0.05541	0.619	0.095	Biological	GO:00072	G protein-coupled acetylcholine receptor signaling pathway	0.05541	0.22441	0	0	2	21
GO:0007379 segment specification	2	0.05541	0.619	0.095	Biological	GO:00073	segment specification	0.05541	0.22441	0	0	2	21
GO:0050855 regulation of B cell receptor signaling pathway	2	0.05541	0.619	0.095	Biological	GO:00508	regulation of B cell receptor signaling pathway	0.05541	0.22441	0	0	2	21
GO:0051000 positive regulation of nitric-oxide synthase activity	2	0.05541	0.619	0.095	Biological	GO:00510	positive regulation of nitric-oxide synthase activity	0.05541	0.22441	0	0	2	21
GO:0072234 metanephric nephron tubule development	2	0.05541	0.619	0.095	Biological	GO:00722	metanephric nephron tubule development	0.05541	0.22441	0	0	2	21
GO:0140131 positive regulation of lymphocyte chemotaxis	2	0.05541	0.619	0.095	Biological	GO:01401	positive regulation of lymphocyte chemotaxis	0.05541	0.22441	0	0	2	21
GO:1902307 positive regulation of sodium ion transmembrane transport	2	0.05541	0.619	0.095	Biological	GO:19023	positive regulation of sodium ion transmembrane transport	0.05541	0.22441	0	0	2	21
GO:1903672 positive regulation of sprouting angiogenesis	2	0.05541	0.619	0.095	Biological	GO:19036	positive regulation of sprouting angiogenesis	0.05541	0.22441	0	0	2	21
GO:2000738 positive regulation of stem cell differentiation	2	0.05541	0.619	0.095	Biological	GO:20007	positive regulation of stem cell differentiation	0.05541	0.22441	0	0	2	21
GO:0005789 endoplasmic reticulum membrane	29	0.05591	8.605	0.025	Cellular C	GO:00057	endoplasmic reticulum membrane	0.05591	0.22323	0	0	29	1180
GO:0022607 cellular component assembly	67	0.05592	20.743	0.022	Biological	GO:00226	cellular component assembly	0.05592	0.22631	0	0	67	3053
GO:0033116 endoplasmic reticulum-Golgi intermediate compartment m	4	0.05651	1.187	0.05	Cellular C	GO:00331	endoplasmic reticulum-Golgi intermediate compartment membrane	0.05651	0.22393	0	0	4	80
GO:0006875 cellular metal ion homeostasis	17	0.05685	5.263	0.028	Biological	GO:00068	cellular metal ion homeostasis	0.05685	0.22918	0	0	17	612
GO:0046332 SMAD binding	4	0.05689	1.17	0.051	Molecular	GO:00463	SMAD binding	0.05689	0.22917	0	0	4	78
GO:0043114 regulation of vascular permeability	3	0.05691	0.929	0.062	Biological	GO:00431	regulation of vascular permeability	0.05691	0.22918	0	0	3	48
GO:0048009 insulin-like growth factor receptor signaling pathway	3	0.05691	0.929	0.062	Biological	GO:00480	insulin-like growth factor receptor signaling pathway	0.05691	0.22918	0	0	3	48
GO:0055081 anion homeostasis	3	0.05691	0.929	0.062	Biological	GO:00550	anion homeostasis	0.05691	0.22918	0	0	3	48
GO:0061383 trabecula morphogenesis	3	0.05691	0.929	0.062	Biological	GO:00613	trabecula morphogenesis	0.05691	0.22918	0	0	3	48
GO:1990089 response to nerve growth factor	3	0.05691	0.929	0.062	Biological	GO:19900	response to nerve growth factor	0.05691	0.22918	0	0	3	48
GO:0007043 cell-cell junction assembly	6	0.05703	1.858	0.04	Biological	GO:00070	cell-cell junction assembly	0.05703	0.22951	0	0	6	150
GO:0061138 morphogenesis of a branching epithelium	7	0.0571	2.167	0.037	Biological	GO:00611	morphogenesis of a branching epithelium	0.0571	0.22961	0	0	7	188
GO:0031346 positive regulation of cell projection organization	11	0.05773	3.406	0.031	Biological	GO:00313	positive regulation of cell projection organization	0.05773	0.23192	0	0	11	351
GO:0098827 endoplasmic reticulum subcompartment	29	0.05787	8.605	0.024	Cellular C	GO:00988	endoplasmic reticulum subcompartment	0.05787	0.22765	0	0	29	1184
GO:0072088 nephron epithelium morphogenesis	4	0.0584	1.238	0.05	Biological	GO:00720	nephron epithelium morphogenesis	0.0584	0.23427	0	0	4	80
GO:0097061 dendritic spine organization	4	0.0584	1.238	0.05	Biological	GO:00970	dendritic spine organization	0.0584	0.23427	0	0	4	80
GO:0031644 regulation of nervous system process	6	0.05853	1.858	0.04	Biological	GO:00316	regulation of nervous system process	0.05853	0.23458	0	0	6	151
GO:0031258 lamellipodium membrane	2	0.05905	0.593	0.091	Cellular C	GO:00312	lamellipodium membrane	0.05905	0.22892	0	0	2	22
GO:0098644 complex of collagen trimers	2	0.05905	0.593	0.091	Cellular C	GO:00986	complex of collagen trimers	0.05905	0.22892	0	0	2	22
GO:0045892 negative regulation of transcription, DNA-templated	32	0.0593	9.907	0.024	Biological	GO:00458	negative regulation of transcription, DNA-templated	0.0593	0.23749	0	0	32	1318
GO:0006636 unsaturated fatty acid biosynthetic process	3	0.05983	0.929	0.061	Biological	GO:00066	unsaturated fatty acid biosynthetic process	0.05983	0.23757	0	0	3	49
GO:0006692 prostanoid metabolic process	3	0.05983	0.929	0.061	Biological	GO:00066	prostanoid metabolic process	0.05983	0.23757	0	0	3	49
GO:0006693 prostaglandin metabolic process	3	0.05983	0.929	0.061	Biological	GO:00066	prostaglandin metabolic process	0.05983	0.23757	0	0	3	49
GO:0033628 regulation of cell adhesion mediated by integrin	3	0.05983	0.929	0.061	Biological	GO:00336	regulation of cell adhesion mediated by integrin	0.05983	0.23757	0	0	3	49
GO:0034331 cell junction maintenance	3	0.05983	0.929	0.061	Biological	GO:00343	cell junction maintenance	0.05983	0.23757	0	0	3	49
GO:0048066 developmental pigmentation	3	0.05983	0.929	0.061	Biological	GO:00480	developmental pigmentation	0.05983	0.23757	0	0	3	49
GO:0061028 establishment of endothelial barrier	3	0.05983	0.929	0.061	Biological	GO:00610	establishment of endothelial barrier	0.05983	0.23757	0	0	3	49
GO:0061082 myeloid leukocyte cytokine production	3	0.05983	0.929	0.061	Biological	GO:00610	myeloid leukocyte cytokine production	0.05983	0.23757	0	0	3	49
GO:0001780 neutrophil homeostasis	2	0.06023	0.619	0.091	Biological	GO:00017	neutrophil homeostasis	0.06023	0.23757	0	0	2	22
GO:0010288 response to lead ion	2	0.06023	0.619	0.091	Biological	GO:00102	response to lead ion	0.06023	0.23757	0	0	2	22
GO:0035461 vitamin transmembrane transport	2	0.06023	0.619	0.091	Biological	GO:00354	vitamin transmembrane transport	0.06023	0.23757	0	0	2	22
GO:0035812 renal sodium excretion	2	0.06023	0.619	0.091	Biological	GO:00358	renal sodium excretion	0.06023	0.23757	0	0	2	22
GO:0044321 response to leptin	2	0.06023	0.619	0.091	Biological	GO:00443	response to leptin	0.06023	0.23757	0	0	2	22
GO:0045063 T-helper 1 cell differentiation	2	0.06023	0.619	0.091	Biological	GO:00450	T-helper 1 cell differentiation	0.06023	0.23757	0	0	2	22
GO:0048485 sympathetic nervous system development	2	0.06023	0.619	0.091	Biological	GO:00484	sympathetic nervous system development	0.06023	0.23757	0	0	2	22
GO:0061318 renal filtration cell differentiation	2	0.06023	0.619	0.091	Biological	GO:00613	renal filtration cell differentiation	0.06023	0.23757	0	0	2	22
GO:0072112 glomerular visceral epithelial cell differentiation	2	0.06023	0.619	0.091	Biological	GO:00721	glomerular visceral epithelial cell differentiation	0.06023	0.23757	0	0	2	22
GO:0090189 regulation of branching involved in ureteric bud morphoger	2	0.06023	0.619	0.091	Biological	GO:00901	regulation of branching involved in ureteric bud morphogenesis	0.06023	0.23757	0	0	2	22
GO:1902644 tertiary alcohol metabolic process	2	0.06023	0.619	0.091	Biological	GO:19026	tertiary alcohol metabolic process	0.06023	0.23757	0	0	2	22
GO:1903507 negative regulation of nucleic acid-templated transcription	32	0.06027	9.907	0.024	Biological	GO:19035	negative regulation of nucleic acid-templated transcription	0.06027	0.23757	0	0	32	1320
GO:0051129 negative regulation of cellular component organization	19	0.06051	5.882	0.027	Biological	GO:00511	negative regulation of cellular component organization	0.06051	0.23831	0	0	19	708
GO:0042310 vasoconstriction	4	0.06059	1.238	0.049	Biological	GO:00423	vasoconstriction	0.06059	0.23846	0	0	4	81
GO:0006816 calcium ion transport	13	0.06065	4.025	0.03	Biological	GO:00068	calcium ion transport	0.06065	0.23848	0	0	13	440
GO:0005871 kinesin complex	3	0.06116	0.89	0.06	Cellular C	GO:00058	kinesin complex	0.06116	0.23541	0	0	3	50
GO:1902679 negative regulation of RNA biosynthetic process	32	0.06125	9.907	0.024	Biological	GO:19026	negative regulation of RNA biosynthetic process	0.06125	0.24068	0	0	32	1322
GO:0051222 positive regulation of protein transport	10	0.06169	3.096	0.032	Biological	GO:00512	positive regulation of protein transport	0.06169	0.24218	0	0	10	313
GO:0042605 peptide antigen binding	2	0.06202	0.585	0.091	Molecular	GO:00426	peptide antigen binding	0.06202	0.24751	0	0	2	22
GO:0140103 catalytic activity, acting on a glycoprotein	2	0.06202	0.585	0.091	Molecular	GO:01401	catalytic activity, acting on a glycoprotein	0.06202	0.24751	0	0	2	22
GO:0055082 cellular chemical homeostasis	22	0.06218	6.811	0.026	Biological	GO:00550	cellular chemical homeostasis	0.06218	0.24395	0	0	22	849
GO:0045893 positive regulation of transcription, DNA-templated	40	0.06259	12.384	0.023	Biological	GO:00458	positive regulation of transcription, DNA-templated	0.06259	0.24517	0	0	40	1715
GO:1903508 positive regulation of nucleic acid-templated transcription	40	0.06259	12.384	0.023	Biological	GO:19035	positive regulation of nucleic acid-templated transcription	0.06259	0.24517	0	0	40	1715
GO:0009101 glycoprotein biosynthetic process	10	0.06274	3.096	0.032	Biological	GO:00091	glycoprotein biosynthetic process	0.06274	0.24533	0	0	10	314
GO:0031018 endocrine pancreas development	3	0.06282	0.929	0.06	Biological	GO:00310	endocrine pancreas development	0.06282	0.24533	0	0	3	50
GO:0099054 presynapse assembly	3	0.06282	0.929	0.06	Biological	GO:00990	presynapse assembly	0.06282	0.24533	0	0	3	50
GO:0061333 renal tubule morphogenesis	4	0.06283	1.238	0.049	Biological	GO:00613	renal tubule morphogenesis	0.06283	0.24533	0	0	4	82
GO:0003774 cytoskeletal motor activity	5	0.06291	1.462	0.043	Molecular	GO:00037	cytoskeletal motor activity	0.06291	0.24903	0	0	5	115
GO:0042277 peptide binding	10	0.06298	2.924	0.032	Molecular	GO:00422	peptide binding	0.06298	0.24903	0	0	10	309
GO:0006476 protein deacetylation	5	0.06305	1.548	0.043	Biological	GO:00064	protein deacetylation	0.06305	0.24582	0	0	5	117
GO:0043200 response to amino acid	5	0.06305	1.548	0.043	Biological	GO:00432	response to amino acid	0.06305	0.24582	0	0	5	117
GO:0035264 multicellular organism growth	6	0.06316	1.858	0.039	Biological	GO:00352	multicellular organism growth	0.06316	0.24602	0	0	6	154
GO:0030001 metal ion transport	23	0.06362	7.121	0.026	Biological	GO:00300	metal ion transport	0.06362	0.24764	0	0	23	898
GO:0031400 negative regulation of protein modification process	14	0.06421	4.334	0.029	Biological	GO:00314	negative regulation of protein modification process	0.06421	0.24976	0	0	14	488
GO:0021537 telencephalon development	9	0.06446	2.786	0.033	Biological	GO:00215	telencephalon development	0.06446	0.25052	0	0	9	274
GO:0021782 glial cell development	5	0.06491	1.548	0.042	Biological	GO:00217	glial cell development	0.06491	0.25078	0	0	5	118
GO:0019915 lipid storage	4	0.06511	1.238	0.048	Biological	GO:00199	lipid storage	0.06511	0.25078	0	0	4	83
GO:0051241 negative regulation of multicellular organismal process	26	0.06514	8.05	0.025	Biological	GO:00512	negative regulation of multicellular organismal process	0.06514	0.25078	0	0	26	1042
GO:0003177 pulmonary valve development	2	0.0652	0.619	0.087	Biological	GO:00031	pulmonary valve development	0.0652	0.25078	0	0	2	23
GO:0010839 negative regulation of keratinocyte proliferation	2	0.0652	0.619	0.087	Biological	GO:00108	negative regulation of keratinocyte proliferation	0.0652	0.25078	0	0	2	23
GO:0043567 regulation of insulin-like growth factor receptor signaling pa	2	0.0652	0.619	0.087	Biological	GO:00435	regulation of insulin-like growth factor receptor signaling pathway	0.0652	0.25078	0	0	2	23
GO:0044062 regulation of excretion	2	0.0652	0.619	0.087	Biological	GO:00440	regulation of excretion	0.0652	0.25078	0	0	2	23
GO:0045649 regulation of macrophage differentiation	2	0.0652	0.619	0.087	Biological	GO:00456	regulation of macrophage differentiation	0.0652	0.25078	0	0	2	23
GO:0046519 sphingoid metabolic process	2	0.0652	0.619	0.087	Biological	GO:00465	sphingoid metabolic process	0.0652	0.25078	0	0	2	23
GO:0060444 branching involved in mammary gland duct morphogenesis	2	0.0652	0.619	0.087	Biological	GO:00604	branching involved in mammary gland duct morphogenesis	0.0652	0.25078	0	0	2	23
GO:0072311 glomerular epithelial cell differentiation	2	0.0652	0.619	0.087	Biological	GO:00723	glomerular epithelial cell differentiation	0.0652	0.25078	0	0	2	23
GO:0097107 postsynaptic density assembly	2	0.0652	0.619	0.087	Biological	GO:00971	postsynaptic density assembly	0.0652	0.25078	0	0	2	23
GO:1902680 positive regulation of RNA biosynthetic process	40	0.0653	12.384	0.023	Biological	GO:19026	positive regulation of RNA biosynthetic process	0.0653	0.25078	0	0	40	1721
GO:0005044 scavenger receptor activity	3	0.06536	0.877	0.06	Molecular	GO:00050	scavenger receptor activity	0.06536	0.25163	0	0	3	50
GO:0016597 amino acid binding	3	0.06536	0.877	0.06	Molecular	GO:00165	amino acid binding	0.06536	0.25163	0	0	3	50
GO:0048705 skeletal system morphogenesis	8	0.06547	2.477	0.034	Biological	GO:00487	skeletal system morphogenesis	0.06547	0.25078	0	0	8	234
GO:0099572 postsynaptic specialization	11	0.06557	3.264	0.03	Cellular C	GO:00995	postsynaptic specialization	0.06557	0.24865	0	0	11	363
GO:0099106 ion channel regulator activity	6	0.06566	1.754	0.039	Molecular	GO:00991	ion channel regulator activity	0.06566	0.25163	0	0	6	153
GO:0090102 cochlea development	3	0.06589	0.929	0.059	Biological	GO:00901	cochlea development	0.06589	0.25078	0	0	3	51
GO:0043069 negative regulation of programmed cell death	23	0.06676	7.121	0.025	Biological	GO:00430	negative regulation of programmed cell death	0.06676	0.25078	0	0	23	903
GO:1901800 positive regulation of proteasomal protein catabolic proces	5	0.0668	1.548	0.042	Biological	GO:19018	positive regulation of proteasomal protein catabolic process	0.0668	0.25078	0	0	5	119
GO:0031323 regulation of cellular metabolic process	123	0.06699	38.08	0.02	Biological	GO:00313	regulation of cellular metabolic process	0.06699	0.25078	0	0	123	6023
GO:0035173 histone kinase activity	2	0.06712	0.585	0.087	Molecular	GO:00351	histone kinase activity	0.06712	0.25163	0	0	2	23
GO:0051253 negative regulation of RNA metabolic process	34	0.06781	10.526	0.024	Biological	GO:00512	negative regulation of RNA metabolic process	0.06781	0.25078	0	0	34	1432
GO:0048754 branching morphogenesis of an epithelial tube	6	0.06799	1.858	0.038	Biological	GO:00487	branching morphogenesis of an epithelial tube	0.06799	0.25078	0	0	6	157
GO:0051092 positive regulation of NF-kappaB transcription factor activi	6	0.06799	1.858	0.038	Biological	GO:00510	positive regulation of NF-kappaB transcription factor activity	0.06799	0.25078	0	0	6	157
GO:0043204 perikaryon	6	0.0684	1.78	0.038	Cellular C	GO:00432	perikaryon	0.0684	0.24865	0	0	6	159

GO:0034219 carbohydrate transmembrane transport	5	0.06872	1.548	0.042	Biological	GO:00342	carbohydrate transmembrane transport	0.06872	0.25078	0	0	5	120
GO:0042303 molting cycle	5	0.06872	1.548	0.042	Biological	GO:00423	molting cycle	0.06872	0.25078	0	0	5	120
GO:0042633 hair cycle	5	0.06872	1.548	0.042	Biological	GO:00426	hair cycle	0.06872	0.25078	0	0	5	120
GO:0003197 endocardial cushion development	3	0.06902	0.929	0.058	Biological	GO:00031	endocardial cushion development	0.06902	0.25078	0	0	3	52
GO:0060688 regulation of morphogenesis of a branching structure	3	0.06902	0.929	0.058	Biological	GO:00606	regulation of morphogenesis of a branching structure	0.06902	0.25078	0	0	3	52
GO:0071622 regulation of granulocyte chemotaxis	3	0.06902	0.929	0.058	Biological	GO:00716	regulation of granulocyte chemotaxis	0.06902	0.25078	0	0	3	52
GO:0061136 regulation of proteasomal protein catabolic process	7	0.06964	2.167	0.036	Biological	GO:0061	regulation of proteasomal protein catabolic process	0.06964	0.25078	0	0	7	197
GO:0032637 interleukin-8 production	4	0.06979	1.238	0.047	Biological	GO:00326	interleukin-8 production	0.06979	0.25078	0	0	4	85
GO:0032677 regulation of interleukin-8 production	4	0.06979	1.238	0.047	Biological	GO:00326	regulation of interleukin-8 production	0.06979	0.25078	0	0	4	85
GO:0008324 cation transmembrane transporter activity	18	0.06992	5.263	0.027	Molecular	GO:00083	cation transmembrane transporter activity	0.06992	0.25163	0	0	18	664
GO:0005588 collagen type V trimer	1	0.07018	0.297	0.25	Cellular C	GO:00055	collagen type V trimer	0.07018	0.24865	0	0	1	4
GO:0005853 eukaryotic translation elongation factor 1 complex	1	0.07018	0.297	0.25	Cellular C	GO:00058	eukaryotic translation elongation factor 1 complex	0.07018	0.24865	0	0	1	4
GO:0016013 syntrophin complex	1	0.07018	0.297	0.25	Cellular C	GO:00160	syntrophin complex	0.07018	0.24865	0	0	1	4
GO:0030478 actin cap	1	0.07018	0.297	0.25	Cellular C	GO:00304	actin cap	0.07018	0.24865	0	0	1	4
GO:0033269 internode region of axon	1	0.07018	0.297	0.25	Cellular C	GO:00332	internode region of axon	0.07018	0.24865	0	0	1	4
GO:0034363 intermediate-density lipoprotein particle	1	0.07018	0.297	0.25	Cellular C	GO:00343	intermediate-density lipoprotein particle	0.07018	0.24865	0	0	1	4
GO:0042567 insulin-like growth factor ternary complex	1	0.07018	0.297	0.25	Cellular C	GO:00425	insulin-like growth factor ternary complex	0.07018	0.24865	0	0	1	4
GO:0071664 catenin-TCF7L2 complex	1	0.07018	0.297	0.25	Cellular C	GO:00716	catenin-TCF7L2 complex	0.07018	0.24865	0	0	1	4
GO:0098983 symmetric, GABA-ergic, inhibitory synapse	1	0.07018	0.297	0.25	Cellular C	GO:00989	symmetric, GABA-ergic, inhibitory synapse	0.07018	0.24865	0	0	1	4
GO:0099160 postsynaptic intermediate filament cytoskeleton	1	0.07018	0.297	0.25	Cellular C	GO:00991	postsynaptic intermediate filament cytoskeleton	0.07018	0.24865	0	0	1	4
GO:0007097 nuclear migration	2	0.07029	0.619	0.083	Biological	GO:00070	nuclear migration	0.07029	0.25078	0	0	2	24
GO:0010884 positive regulation of lipid storage	2	0.07029	0.619	0.083	Biological	GO:00108	positive regulation of lipid storage	0.07029	0.25078	0	0	2	24
GO:0021871 forebrain regionalization	2	0.07029	0.619	0.083	Biological	GO:00218	forebrain regionalization	0.07029	0.25078	0	0	2	24
GO:0032528 microvillus organization	2	0.07029	0.619	0.083	Biological	GO:00325	microvillus organization	0.07029	0.25078	0	0	2	24
GO:0042430 indole-containing compound metabolic process	2	0.07029	0.619	0.083	Biological	GO:00424	indole-containing compound metabolic process	0.07029	0.25078	0	0	2	24
GO:0048745 smooth muscle tissue development	2	0.07029	0.619	0.083	Biological	GO:00487	smooth muscle tissue development	0.07029	0.25078	0	0	2	24
GO:0050857 positive regulation of antigen receptor-mediated signaling p	2	0.07029	0.619	0.083	Biological	GO:00508	positive regulation of antigen receptor-mediated signaling pathway	0.07029	0.25078	0	0	2	24
GO:0060065 uterus development	2	0.07029	0.619	0.083	Biological	GO:00600	uterus development	0.07029	0.25078	0	0	2	24
GO:0061484 hematopoietic stem cell homeostasis	2	0.07029	0.619	0.083	Biological	GO:00614	hematopoietic stem cell homeostasis	0.07029	0.25078	0	0	2	24
GO:0072170 metanephric tubule development	2	0.07029	0.619	0.083	Biological	GO:00721	metanephric tubule development	0.07029	0.25078	0	0	2	24
GO:0072243 metanephric nephron epithelium development	2	0.07029	0.619	0.083	Biological	GO:00722	metanephric nephron epithelium development	0.07029	0.25078	0	0	2	24
GO:0001952 regulation of cell-matrix adhesion	5	0.07066	1.548	0.041	Biological	GO:00019	regulation of cell-matrix adhesion	0.07066	0.25078	0	0	5	121
GO:0001796 regulation of type IIa hypersensitivity	1	0.07096	0.31	0.25	Biological	GO:00017	regulation of type IIa hypersensitivity	0.07096	0.25078	0	0	1	4
GO:0002528 regulation of vascular permeability involved in acute inflam	1	0.07096	0.31	0.25	Biological	GO:00025	regulation of vascular permeability involved in acute inflammatory res	0.07096	0.25078	0	0	1	4
GO:0002892 regulation of type II hypersensitivity	1	0.07096	0.31	0.25	Biological	GO:00028	regulation of type II hypersensitivity	0.07096	0.25078	0	0	1	4
GO:0003241 growth involved in heart morphogenesis	1	0.07096	0.31	0.25	Biological	GO:00032	growth involved in heart morphogenesis	0.07096	0.25078	0	0	1	4
GO:0006556 S-adenosylmethionine biosynthetic process	1	0.07096	0.31	0.25	Biological	GO:00065	S-adenosylmethionine biosynthetic process	0.07096	0.25078	0	0	1	4
GO:0007621 negative regulation of female receptivity	1	0.07096	0.31	0.25	Biological	GO:00076	negative regulation of female receptivity	0.07096	0.25078	0	0	1	4
GO:0008065 establishment of blood-nerve barrier	1	0.07096	0.31	0.25	Biological	GO:00080	establishment of blood-nerve barrier	0.07096	0.25078	0	0	1	4
GO:0009093 cysteine catabolic process	1	0.07096	0.31	0.25	Biological	GO:00090	cysteine catabolic process	0.07096	0.25078	0	0	1	4
GO:0009794 regulation of mitotic cell cycle, embryonic	1	0.07096	0.31	0.25	Biological	GO:00097	regulation of mitotic cell cycle, embryonic	0.07096	0.25078	0	0	1	4
GO:0014005 microglia development	1	0.07096	0.31	0.25	Biological	GO:00140	microglia development	0.07096	0.25078	0	0	1	4
GO:0014809 regulation of skeletal muscle contraction by regulation of re	1	0.07096	0.31	0.25	Biological	GO:00148	regulation of skeletal muscle contraction by regulation of release of se	0.07096	0.25078	0	0	1	4
GO:0014826 vein smooth muscle contraction	1	0.07096	0.31	0.25	Biological	GO:00148	vein smooth muscle contraction	0.07096	0.25078	0	0	1	4
GO:0015829 valine transport	1	0.07096	0.31	0.25	Biological	GO:00158	valine transport	0.07096	0.25078	0	0	1	4
GO:0016095 polyprenol catabolic process	1	0.07096	0.31	0.25	Biological	GO:00160	polyprenol catabolic process	0.07096	0.25078	0	0	1	4
GO:0018076 N-terminal peptidyl-lysine acetylation	1	0.07096	0.31	0.25	Biological	GO:00180	N-terminal peptidyl-lysine acetylation	0.07096	0.25078	0	0	1	4
GO:0019448 L-cysteine catabolic process	1	0.07096	0.31	0.25	Biological	GO:00194	L-cysteine catabolic process	0.07096	0.25078	0	0	1	4
GO:0019541 propionate metabolic process	1	0.07096	0.31	0.25	Biological	GO:00195	propionate metabolic process	0.07096	0.25078	0	0	1	4
GO:0019747 regulation of isoprenoid metabolic process	1	0.07096	0.31	0.25	Biological	GO:00197	regulation of isoprenoid metabolic process	0.07096	0.25078	0	0	1	4
GO:0021778 oligodendrocyte cell fate specification	1	0.07096	0.31	0.25	Biological	GO:00217	oligodendrocyte cell fate specification	0.07096	0.25078	0	0	1	4
GO:0021779 oligodendrocyte cell fate commitment	1	0.07096	0.31	0.25	Biological	GO:00217	oligodendrocyte cell fate commitment	0.07096	0.25078	0	0	1	4
GO:0033023 mast cell homeostasis	1	0.07096	0.31	0.25	Biological	GO:00330	mast cell homeostasis	0.07096	0.25078	0	0	1	4
GO:0033686 positive regulation of luteinizing hormone secretion	1	0.07096	0.31	0.25	Biological	GO:00336	positive regulation of luteinizing hormone secretion	0.07096	0.25078	0	0	1	4
GO:0035583 sequestering of TGFbeta in extracellular matrix	1	0.07096	0.31	0.25	Biological	GO:00355	sequestering of TGFbeta in extracellular matrix	0.07096	0.25078	0	0	1	4
GO:0035814 negative regulation of renal sodium excretion	1	0.07096	0.31	0.25	Biological	GO:00358	negative regulation of renal sodium excretion	0.07096	0.25078	0	0	1	4
GO:0035880 embryonic nail plate morphogenesis	1	0.07096	0.31	0.25	Biological	GO:00358	embryonic nail plate morphogenesis	0.07096	0.25078	0	0	1	4
GO:0035910 ascending aorta morphogenesis	1	0.07096	0.31	0.25	Biological	GO:00359	ascending aorta morphogenesis	0.07096	0.25078	0	0	1	4
GO:0036343 psychomotor behavior	1	0.07096	0.31	0.25	Biological	GO:00363	psychomotor behavior	0.07096	0.25078	0	0	1	4
GO:0042321 negative regulation of circadian sleep/wake cycle, sleep	1	0.07096	0.31	0.25	Biological	GO:00423	negative regulation of circadian sleep/wake cycle, sleep	0.07096	0.25078	0	0	1	4
GO:0042412 taurine biosynthetic process	1	0.07096	0.31	0.25	Biological	GO:00424	taurine biosynthetic process	0.07096	0.25078	0	0	1	4
GO:0042637 catagen	1	0.07096	0.31	0.25	Biological	GO:00426	catagen	0.07096	0.25078	0	0	1	4
GO:0045163 clustering of voltage-gated potassium channels	1	0.07096	0.31	0.25	Biological	GO:00451	clustering of voltage-gated potassium channels	0.07096	0.25078	0	0	1	4
GO:0045448 mitotic cell cycle, embryonic	1	0.07096	0.31	0.25	Biological	GO:00454	mitotic cell cycle, embryonic	0.07096	0.25078	0	0	1	4
GO:0046305 alkanesulfonate biosynthetic process	1	0.07096	0.31	0.25	Biological	GO:00463	alkanesulfonate biosynthetic process	0.07096	0.25078	0	0	1	4
GO:0046439 L-cysteine metabolic process	1	0.07096	0.31	0.25	Biological	GO:00464	L-cysteine metabolic process	0.07096	0.25078	0	0	1	4
GO:0046967 cytosol to endoplasmic reticulum transport	1	0.07096	0.31	0.25	Biological	GO:00469	cytosol to endoplasmic reticulum transport	0.07096	0.25078	0	0	1	4
GO:0048808 male genitalia morphogenesis	1	0.07096	0.31	0.25	Biological	GO:00488	male genitalia morphogenesis	0.07096	0.25078	0	0	1	4
GO:0051794 regulation of timing of catagen	1	0.07096	0.31	0.25	Biological	GO:00517	regulation of timing of catagen	0.07096	0.25078	0	0	1	4
GO:0051891 positive regulation of cardioblast differentiation	1	0.07096	0.31	0.25	Biological	GO:00518	positive regulation of cardioblast differentiation	0.07096	0.25078	0	0	1	4
GO:0060018 astrocyte fate commitment	1	0.07096	0.31	0.25	Biological	GO:00600	astrocyte fate commitment	0.07096	0.25078	0	0	1	4
GO:0060298 positive regulation of sarcomere organization	1	0.07096	0.31	0.25	Biological	GO:00602	positive regulation of sarcomere organization	0.07096	0.25078	0	0	1	4
GO:0060465 pharynx development	1	0.07096	0.31	0.25	Biological	GO:00604	pharynx development	0.07096	0.25078	0	0	1	4
GO:0060737 prostate gland morphogenetic growth	1	0.07096	0.31	0.25	Biological	GO:00607	prostate gland morphogenetic growth	0.07096	0.25078	0	0	1	4
GO:0060763 mammary duct terminal end bud growth	1	0.07096	0.31	0.25	Biological	GO:00607	mammary duct terminal end bud growth	0.07096	0.25078	0	0	1	4
GO:0061055 myotome development	1	0.07096	0.31	0.25	Biological	GO:00610	myotome development	0.07096	0.25078	0	0	1	4
GO:0061551 trigeminal ganglion development	1	0.07096	0.31	0.25	Biological	GO:00615	trigeminal ganglion development	0.07096	0.25078	0	0	1	4
GO:0070384 Harderian gland development	1	0.07096	0.31	0.25	Biological	GO:00703	Harderian gland development	0.07096	0.25078	0	0	1	4
GO:0070668 positive regulation of mast cell proliferation	1	0.07096	0.31	0.25	Biological	GO:00706	positive regulation of mast cell proliferation	0.07096	0.25078	0	0	1	4
GO:0071205 protein localization to juxtaparanode region of axon	1	0.07096	0.31	0.25	Biological	GO:00712	protein localization to juxtaparanode region of axon	0.07096	0.25078	0	0	1	4
GO:0071603 endothelial cell-cell adhesion	1	0.07096	0.31	0.25	Biological	GO:00716	endothelial cell-cell adhesion	0.07096	0.25078	0	0	1	4
GO:0071623 negative regulation of granulocyte chemotaxis	1	0.07096	0.31	0.25	Biological	GO:00716	negative regulation of granulocyte chemotaxis	0.07096	0.25078	0	0	1	4
GO:0072011 glomerular endothelium development	1	0.07096	0.31	0.25	Biological	GO:00720	glomerular endothelium development	0.07096	0.25078	0	0	1	4
GO:0072093 metanephric renal vesicle formation	1	0.07096	0.31	0.25	Biological	GO:00720	metanephric renal vesicle formation	0.07096	0.25078	0	0	1	4
GO:0072095 regulation of branch elongation involved in ureteric bud bra	1	0.07096	0.31	0.25	Biological	GO:00720	regulation of branch elongation involved in ureteric bud branching	0.07096	0.25078	0	0	1	4
GO:0072106 regulation of ureteric bud formation	1	0.07096	0.31	0.25	Biological	GO:00721	regulation of ureteric bud formation	0.07096	0.25078	0	0	1	4
GO:0072107 positive regulation of ureteric bud formation	1	0.07096	0.31	0.25	Biological	GO:00721	positive regulation of ureteric bud formation	0.07096	0.25078	0	0	1	4
GO:0072289 metanephric nephron tubule formation	1	0.07096	0.31	0.25	Biological	GO:00722	metanephric nephron tubule formation	0.07096	0.25078	0	0	1	4
GO:0072739 response to anisomycin	1	0.07096	0.31	0.25	Biological	GO:00727	response to anisomycin	0.07096	0.25078	0	0	1	4
GO:0090024 negative regulation of neutrophil chemotaxis	1	0.07096	0.31	0.25	Biological	GO:00900	negative regulation of neutrophil chemotaxis	0.07096	0.25078	0	0	1	4
GO:0090071 negative regulation of ribosome biogenesis	1	0.07096	0.31	0.25	Biological	GO:00900	negative regulation of ribosome biogenesis	0.07096	0.25078	0	0	1	4
GO:0090258 negative regulation of mitochondrial fission	1	0.07096	0.31	0.25	Biological	GO:00902	negative regulation of mitochondrial fission	0.07096	0.25078	0	0	1	4
GO:0090598 male anatomical structure morphogenesis	1	0.07096	0.31	0.25	Biological	GO:00905	male anatomical structure morphogenesis	0.07096	0.25078	0	0	1	4
GO:0097022 lymphocyte migration into lymph node	1	0.07096	0.31	0.25	Biological	GO:00970	lymphocyte migration into lymph node	0.07096	0.25078	0	0	1	4
GO:0097376 interneuron axon guidance	1	0.07096	0.31	0.25	Biological	GO:00973	interneuron axon guidance	0.07096	0.25078	0	0	1	4
GO:0097492 sympathetic neuron axon guidance	1	0.07096	0.31	0.25	Biological	GO:00974	sympathetic neuron axon guidance	0.07096	0.25078	0	0	1	4
GO:0099179 regulation of synaptic membrane adhesion	1	0.07096	0.31	0.25	Biological	GO:00991	regulation of synaptic membrane adhesion	0.07096	0.25078	0	0	1	4
GO:0120229 protein localization to motile cilium	1	0.07096	0.31	0.25	Biological	GO:01202	protein localization to motile cilium	0.07096	0.25078	0	0	1	4
GO:0150062 complement-mediated synapse pruning	1	0.07096	0.31	0.25	Biological	GO:01500	complement-mediated synapse pruning	0.07096	0.25078	0	0	1	4
GO:1900738 positive regulation of phospholipase C-activating G protein	1	0.07096	0.31	0.25	Biological	GO:19007	positive regulation of phospholipase C-activating G protein-coupled re	0.07096	0.25078	0	0	1	4
GO:1902226 regulation of macrophage colony-stimulating factor signalir	1	0.07096	0.31	0.25	Biological	GO:19022	regulation of macrophage colony-stimulating factor signaling pathway	0.07096	0.25078	0	0	1	4
GO:1902308 regulation of peptidyl-serine dephosphorylation	1	0.07096	0.31	0.25	Biological	GO:19023	regulation of peptidyl-serine dephosphorylation	0.07096	0.25078	0	0	1	4
GO:1902994 regulation of phospholipid efflux	1	0.07096	0.31	0.25	Biological	GO:19029	regulation of phospholipid efflux	0.07096	0.25078	0	0	1	4
GO:1902995 positive regulation of phospholipid efflux	1	0.07096	0.31	0.25	Biological	GO:19029	positive regulation of phospholipid efflux	0.07096	0.25078	0	0	1	4
GO:1903121 regulation of TRAIL-activated apoptotic signaling pathway	1	0.07096	0.31	0.25	Biological	GO:19031	regulation of TRAIL-activated apoptotic signaling pathway	0.07096	0.25078	0	0	1	4
GO:1903210 glomerular visceral epithelial cell apoptotic process	1	0.07096	0.31	0.25	Biological	GO:19032	glomerular visceral epithelial cell apoptotic process	0.07096	0.25078	0	0	1	4
GO:1903925 response to bisphenol A	1	0.07096	0.31	0.25	Biological	GO:19039	response to bisphenol A	0.07096	0.25078	0	0	1	4
GO:1903926 cellular response to bisphenol A	1	0.07096	0.31	0.25	Biological	GO:19039	cellular response to bisphenol A	0.07096	0.25078	0	0	1	4
GO:1904220 regulation of serine C-palmitoyltransferase activity	1	0.07096	0.31	0.25	Biological	GO:19042	regulation of serine C-palmitoyltransferase activity	0.07096	0.25078	0	0	1	4
GO:1904273 L-alanine import across plasma membrane	1	0.07096	0.31	0.25	Biological	GO:19042	L-alanine import across plasma membrane	0.07096	0.25078	0	0	1	4
GO:1904684 negative regulation of metalloendopeptidase activity	1	0.07096	0.31	0.25	Biological	GO:19046	negative regulation of metalloendopeptidase activity	0.07096	0.25078	0	0	1	4
GO:1990441 negative regulation of transcription from RNA polymerase	1	0.07096	0.31	0.25	Biological	GO:19904	negative regulation of transcription from RNA polymerase II promoter	0.07096	0.25078	0	0	1	4
GO:1990708 conditioned place preference	1	0.07096	0.31	0.25	Biological	GO:19907	conditioned place preference	0.07096	0.25078	0	0	1	4
GO:2000418 positive regulation of eosinophil migration	1	0.07096	0.31	0.25	Biological	GO:20004	positive regulation of eosinophil migration	0.07096	0.25078	0	0	1	4
GO:2000520 regulation of immunological synapse formation	1	0.07096	0.31	0.25	Biological	GO:20005	regulation of immunological synapse formation	0.07096	0.25078	0	0	1	4
GO:2000697 negative regulation of epithelial cell differentiation involved	1	0.07096	0.31	0.25	Biological	GO:20006	negative regulation of epithelial cell differentiation involved in kidney d	0.07096	0.25078	0	0	1	4
GO:2000741 positive regulation of mesenchymal stem cell differentiatior	1	0.07096	0.31	0.25	Biological	GO:20007	positive regulation of mesenchymal stem cell differentiation	0.07096	0.25078	0	0	1	4
GO:2001013 epithelial cell proliferation involved in renal tubule morphoge	1	0.07096	0.31	0.25	Biological	GO:20010	epithelial cell proliferation involved in renal tubule morphogenesis	0.07096	0.25078	0	0	1	4
GO:0051017 actin filament bundle assembly	6	0.07133	1.858	0.038	Biological	GO:00510	actin filament bundle assembly	0.07133	0.25158	0	0	6	159
GO:0097530 granulocyte migration	6	0.07133	1.858	0.038	Biological	GO:00975	granulocyte migration	0.07133	0.25158	0	0	6	159
GO:0120254 olefinic compound metabolic process	6	0.07133	1.858	0.038	Biological	GO:01202	olefinic compound metabolic process	0.07133	0.25158	0	0	6	159
GO:0001933 negative regulation of protein phosphorylation	10	0.07158	3.096	0.031	Biological	GO:00019	negative regulation of protein phosphorylation	0.07158	0.25211	0	0	10	322
GO:0051047 positive regulation of secretion	10	0.07158	3.096	0.031	Biological	GO:00510	positive regulation of secretion	0.07158	0.25211	0	0	10	322
GO:0004879 nuclear receptor activity	3	0.07177	0.877	0.058	Molecular	GO:00048	nuclear receptor activity	0.07177	0.25163	0	0	3	52
GO:0098531 ligand-activated transcription factor activity	3	0.07177	0.877	0.058	Molecular	GO:00985	ligand-activated transcription factor activity	0.07177	0.25163	0	0	3	52
GO:0140296 general transcription initiation factor binding	3	0.07177	0.877	0.058	Molecular	GO:01402	general transcription initiation factor binding	0.07177	0.25163	0	0	3	52
GO:0042326 negative regulation of phosphorylation	11	0.07184	3.406	0.03	Biological	GO:00423	negative regulation of phosphorylation	0.07184	0.2528	0	0	11	365
GO:0030595 leukocyte chemotaxis	8	0.07208	2.477	0.033	Biological	GO:00305	leukocyte chemotaxis	0.07208	0.2535	0	0	8	239
GO:0003987 acetate-CoA ligase activity	1	0.07211	0.292	0.25	Molecular	GO:00039	acetate-CoA ligase activity	0.07211	0.25163	0	0	1	4
GO:0004731 purine-nucleoside phosphorylase activity	1	0.07211	0.292	0.25	Molecular	GO:00047	purine-nucleoside phosphorylase activity	0.07211	0.25163	0	0	1	4
GO:0004949 cannabinoid receptor activity	1	0.07211	0.292	0.25	Molecular	GO:00049	cannabinoid receptor activity	0.07211	0.25163	0	0	1	4
GO:0005173 stem cell factor receptor binding	1	0.07211	0.292	0.25	Molecular	GO:00051	stem cell factor receptor binding	0.07211	0.25163	0	0	1	4
GO:0016215 acyl-CoA desaturase activity	1	0.07211	0.292	0.25	Molecular	GO:00162	acyl-CoA desaturase activity	0.07211	0.25163	0	0	1	4
GO:0031781 type 3 melanocortin receptor binding	1	0.07211	0.292	0.25	Molecular	GO:00317	type 3 melanocortin receptor binding	0.07211	0.25163	0	0	1	4
GO:0031782 type 4 melanocortin receptor binding	1	0.07211	0.292	0.25	Molecular	GO:00317	type 4 melanocortin receptor binding	0.07211	0.25163	0	0	1	4
GO:0032795 heterotrimeric G-protein binding	1	0.07211	0.292	0.25	Molecular	GO:00327	heterotrimeric G-protein binding	0.07211	0.25163	0	0	1	4
GO:0034739 histone deacetylase activity (H4-K16 specific)	1	0.07211	0.292	0.25	Molecular	GO:00347	histone deacetylase activity (H4-K16 specific)	0.07211	0.25163	0	0	1	4
GO:0035410 dihydrotestosterone 17-beta-dehydrogenase activity	1	0.07211	0.292	0.25	Molecular	GO:00354	dihydrotestosterone 17-beta-dehydrogenase activity	0.07211	0.25163	0	0	1	4
GO:0035877 death effector domain binding	1	0.07211	0.292	0.25	Molecular	GO:00358	death effector domain binding	0.07211	0.25163	0	0	1	4
GO:0046936 2'-deoxyadenosine deaminase activity	1	0.07211	0.292	0.25	Molecular	GO:00469	2'-deoxyadenosine deaminase activity	0.07211	0.25163	0	0	1	4
GO:0050218 propionate-CoA ligase activity	1	0.07211	0.292	0.25	Molecular	GO:00502	propionate-CoA ligase activity	0.07211	0.25163	0	0	1	4
GO:0070326 very-low-density lipoprotein particle receptor binding	1	0.07211	0.292	0.25	Molecular	GO:00703	very-low-density lipoprotein particle receptor binding	0.07211	0.25163	0	0	1	4
GO:0070996 type 1 melanocortin receptor binding	1	0.07211	0.292	0.25	Molecular	GO:00709	type 1 melanocortin receptor binding	0.07211	0.25163	0	0	1	4
GO:0071553 G protein-coupled pyrimidinergic nucleotide receptor activi	1	0.07211	0.292	0.25	Molecular	GO:00715	G protein-coupled pyrimidinergic nucleotide receptor activity	0.07211	0.25163	0	0	1	4
GO:0106006 cytoskeletal protein-membrane anchor activity	1	0.07211	0.292	0.25	Molecular	GO:01060	cytoskeletal protein-membrane anchor activity	0.07211	0.25163	0	0	1	4
GO:0160002 ADP-D-ribose modification-dependent protein binding	1	0.07211	0.292	0.25	Molecular	GO:01600	ADP-D-ribose modification-dependent protein binding	0.07211	0.25163	0	0	1	4
GO:1902945 metalloendopeptidase activity involved in amyloid precurso	1	0.07211	0.292	0.25	Molecular	GO:19029	metalloendopeptidase activity involved in amyloid precursor protein ca	0.07211	0.25163	0	0	1	4
GO:0060324 face development	3	0.07221	0.929	0.057	Biological	GO:00603	face development	0.07221	0.25378	0	0	3	53
GO:0005160 transforming growth factor beta receptor binding	2	0.07235	0.585	0.083	Molecular	GO:00051	transforming growth factor beta receptor binding	0.07235	0.25163	0	0	2	24
GO:0008106 alcohol dehydrogenase (NADP+) activity	2	0.07235	0.585	0.083	Molecular	GO:00081	alcohol dehydrogenase (NADP+) activity	0.07235	0.25163	0	0	2	24
GO:0016702 oxidoreductase activity, acting on single donors with incorp	2	0.07235	0.585	0.083	Molecular	GO:00167	oxidoreductase activity, acting on single donors with incorporation of	0.07235	0.25163	0	0	2	24
GO:0048638 regulation of developmental growth	10	0.07274	3.096	0.031	Biological	GO:00486	regulation of developmental growth	0.07274	0.25547	0	0	10	323

GO:0061448 connective tissue development	9	0.07294	2.786	0.032	Biological	GO:00614	connective tissue development	0.07294	0.25597	0	0	9	281
GO:0030509 BMP signaling pathway	6	0.07304	1.858	0.037	Biological	GO:00305	BMP signaling pathway	0.07304	0.25615	0	0	6	160
GO:0010817 regulation of hormone levels	15	0.07326	4.644	0.028	Biological	GO:00108	regulation of hormone levels	0.07326	0.25674	0	0	15	543
GO:0015849 organic acid transport	10	0.07391	3.096	0.031	Biological	GO:00158	organic acid transport	0.07391	0.25885	0	0	10	324
GO:0051336 regulation of hydrolase activity	25	0.07412	7.74	0.025	Biological	GO:00513	regulation of hydrolase activity	0.07412	0.25938	0	0	25	1009
GO:0016247 channel regulator activity	6	0.07416	1.754	0.038	Molecular	GO:00162	channel regulator activity	0.07416	0.2569	0	0	6	158
GO:0005125 cytokine activity	8	0.07482	2.339	0.034	Molecular	GO:00051	cytokine activity	0.07482	0.25816	0	0	8	237
GO:0098772 molecular function regulator	47	0.07522	13.743	0.023	Molecular	GO:00987	molecular function regulator	0.07522	0.25848	0	0	47	2055
GO:0002042 cell migration involved in sprouting angiogenesis	3	0.07547	0.929	0.056	Biological	GO:00020	cell migration involved in sprouting angiogenesis	0.07547	0.26152	0	0	3	54
GO:0007157 heterophilic cell-cell adhesion via plasma membrane cell ad	3	0.07547	0.929	0.056	Biological	GO:00071	heterophilic cell-cell adhesion via plasma membrane cell adhesion mo	0.07547	0.26152	0	0	3	54
GO:0010718 positive regulation of epithelial to mesenchymal transition	3	0.07547	0.929	0.056	Biological	GO:00107	positive regulation of epithelial to mesenchymal transition	0.07547	0.26152	0	0	3	54
GO:0014009 glial cell proliferation	3	0.07547	0.929	0.056	Biological	GO:00140	glial cell proliferation	0.07547	0.26152	0	0	3	54
GO:0099172 presynapse organization	3	0.07547	0.929	0.056	Biological	GO:00991	presynapse organization	0.07547	0.26152	0	0	3	54
GO:0001759 organ induction	2	0.07551	0.619	0.08	Biological	GO:00017	organ induction	0.07551	0.26152	0	0	2	25
GO:0003071 renal system process involved in regulation of systemic art	2	0.07551	0.619	0.08	Biological	GO:00030	renal system process involved in regulation of systemic arterial blood	0.07551	0.26152	0	0	2	25
GO:0030318 melanocyte differentiation	2	0.07551	0.619	0.08	Biological	GO:00303	melanocyte differentiation	0.07551	0.26152	0	0	2	25
GO:0035330 regulation of hippo signaling	2	0.07551	0.619	0.08	Biological	GO:00353	regulation of hippo signaling	0.07551	0.26152	0	0	2	25
GO:0046641 positive regulation of alpha-beta T cell proliferation	2	0.07551	0.619	0.08	Biological	GO:00466	positive regulation of alpha-beta T cell proliferation	0.07551	0.26152	0	0	2	25
GO:0046885 regulation of hormone biosynthetic process	2	0.07551	0.619	0.08	Biological	GO:00468	regulation of hormone biosynthetic process	0.07551	0.26152	0	0	2	25
GO:0070423 nucleotide-binding oligomerization domain containing signa	2	0.07551	0.619	0.08	Biological	GO:00704	nucleotide-binding oligomerization domain containing signaling pathw	0.07551	0.26152	0	0	2	25
GO:0120255 olefinic compound biosynthetic process	2	0.07551	0.619	0.08	Biological	GO:01202	olefinic compound biosynthetic process	0.07551	0.26152	0	0	2	25
GO:1904752 regulation of vascular associated smooth muscle cell migra	2	0.07551	0.619	0.08	Biological	GO:19047	regulation of vascular associated smooth muscle cell migration	0.07551	0.26152	0	0	2	25
GO:2000679 positive regulation of transcription regulatory region DNA b	2	0.07551	0.619	0.08	Biological	GO:20006	positive regulation of transcription regulatory region DNA binding	0.07551	0.26152	0	0	2	25
GO:0060401 cytosolic calcium ion transport	7	0.0757	2.167	0.035	Biological	GO:00604	cytosolic calcium ion transport	0.0757	0.26197	0	0	7	201
GO:0061572 actin filament bundle organization	6	0.07652	1.858	0.037	Biological	GO:00615	actin filament bundle organization	0.07652	0.26464	0	0	6	162
GO:0010927 cellular component assembly involved in morphogenesis	5	0.07669	1.548	0.04	Biological	GO:00109	cellular component assembly involved in morphogenesis	0.07669	0.26504	0	0	5	124
GO:0032420 stereocilium	3	0.07677	0.89	0.055	Cellular C	GO:00324	stereocilium	0.07677	0.27019	0	0	3	55
GO:0030036 actin cytoskeleton organization	19	0.07732	5.882	0.026	Biological	GO:00300	actin cytoskeleton organization	0.07732	0.26703	0	0	19	731
GO:0001091 RNA polymerase II general transcription initiation factor bir	2	0.07771	0.585	0.08	Molecular	GO:00010	RNA polymerase II general transcription initiation factor binding	0.07771	0.26494	0	0	2	25
GO:0016701 oxidoreductase activity, acting on single donors with incorp	2	0.07771	0.585	0.08	Molecular	GO:00167	oxidoreductase activity, acting on single donors with incorporation of	0.07771	0.26494	0	0	2	25
GO:0010469 regulation of signaling receptor activity	6	0.0783	1.858	0.037	Biological	GO:00104	regulation of signaling receptor activity	0.0783	0.27022	0	0	6	163
GO:0007566 embryo implantation	3	0.0788	0.929	0.055	Biological	GO:00075	embryo implantation	0.0788	0.27134	0	0	3	55
GO:0019933 cAMP-mediated signaling	3	0.0788	0.929	0.055	Biological	GO:00199	cAMP-mediated signaling	0.0788	0.27134	0	0	3	55
GO:0050873 brown fat cell differentiation	3	0.0788	0.929	0.055	Biological	GO:00508	brown fat cell differentiation	0.0788	0.27134	0	0	3	55
GO:0001763 morphogenesis of a branching structure	7	0.07884	2.167	0.034	Biological	GO:00017	morphogenesis of a branching structure	0.07884	0.27134	0	0	7	203
GO:0051050 positive regulation of transport	23	0.07894	7.121	0.025	Biological	GO:00510	positive regulation of transport	0.07894	0.27149	0	0	23	921
GO:0051641 cellular localization	65	0.07961	20.124	0.022	Biological	GO:00516	cellular localization	0.07961	0.27362	0	0	65	3016
GO:2000243 positive regulation of reproductive process	4	0.07966	1.238	0.045	Biological	GO:20002	positive regulation of reproductive process	0.07966	0.27362	0	0	4	89
GO:0016528 sarcoplasm	4	0.07968	1.187	0.044	Cellular C	GO:00165	sarcoplasm	0.07968	0.2786	0	0	4	90
GO:1904951 positive regulation of establishment of protein localization	10	0.07994	3.096	0.03	Biological	GO:19049	positive regulation of establishment of protein localization	0.07994	0.27437	0	0	10	329
GO:0006665 sphingolipid metabolic process	6	0.08009	1.858	0.037	Biological	GO:00066	sphingolipid metabolic process	0.08009	0.27472	0	0	6	164
GO:0044089 positive regulation of cellular component biogenesis	15	0.08059	4.644	0.027	Biological	GO:00440	positive regulation of cellular component biogenesis	0.08059	0.27505	0	0	15	551
GO:0010875 positive regulation of cholesterol efflux	2	0.08085	0.619	0.077	Biological	GO:00108	positive regulation of cholesterol efflux	0.08085	0.27505	0	0	2	26
GO:0033622 integrin activation	2	0.08085	0.619	0.077	Biological	GO:00336	integrin activation	0.08085	0.27505	0	0	2	26
GO:0035872 nucleotide-binding domain, leucine rich repeat containing r	2	0.08085	0.619	0.077	Biological	GO:00358	nucleotide-binding domain, leucine rich repeat containing receptor sig	0.08085	0.27505	0	0	2	26
GO:0042659 regulation of cell fate specification	2	0.08085	0.619	0.077	Biological	GO:00426	regulation of cell fate specification	0.08085	0.27505	0	0	2	26
GO:0048596 embryonic camera-type eye morphogenesis	2	0.08085	0.619	0.077	Biological	GO:00485	embryonic camera-type eye morphogenesis	0.08085	0.27505	0	0	2	26
GO:0050687 negative regulation of defense response to virus	2	0.08085	0.619	0.077	Biological	GO:00506	negative regulation of defense response to virus	0.08085	0.27505	0	0	2	26
GO:0060343 trabecula formation	2	0.08085	0.619	0.077	Biological	GO:00603	trabecula formation	0.08085	0.27505	0	0	2	26
GO:0072010 glomerular epithelium development	2	0.08085	0.619	0.077	Biological	GO:00720	glomerular epithelium development	0.08085	0.27505	0	0	2	26
GO:1901623 regulation of lymphocyte chemotaxis	2	0.08085	0.619	0.077	Biological	GO:19016	regulation of lymphocyte chemotaxis	0.08085	0.27505	0	0	2	26
GO:1903901 negative regulation of viral life cycle	2	0.08085	0.619	0.077	Biological	GO:19039	negative regulation of viral life cycle	0.08085	0.27505	0	0	2	26
GO:1904706 negative regulation of vascular associated smooth muscle	2	0.08085	0.619	0.077	Biological	GO:19047	negative regulation of vascular associated smooth muscle cell prolife	0.08085	0.27505	0	0	2	26
GO:0038024 cargo receptor activity	4	0.08086	1.17	0.045	Molecular	GO:00380	cargo receptor activity	0.08086	0.27043	0	0	4	88
GO:0071496 cellular response to external stimulus	10	0.08118	3.096	0.03	Biological	GO:00714	cellular response to external stimulus	0.08118	0.27598	0	0	10	330
GO:0030003 cellular cation homeostasis	18	0.08176	5.573	0.026	Biological	GO:00300	cellular cation homeostasis	0.08176	0.27737	0	0	18	690
GO:0005080 protein kinase C binding	3	0.08189	0.877	0.055	Molecular	GO:00050	protein kinase C binding	0.08189	0.27043	0	0	3	55
GO:0032608 interferon-beta production	3	0.08218	0.929	0.054	Biological	GO:00326	interferon-beta production	0.08218	0.27737	0	0	3	56
GO:0032648 regulation of interferon-beta production	3	0.08218	0.929	0.054	Biological	GO:00326	regulation of interferon-beta production	0.08218	0.27737	0	0	3	56
GO:0043616 keratinocyte proliferation	3	0.08218	0.929	0.054	Biological	GO:00436	keratinocyte proliferation	0.08218	0.27737	0	0	3	56
GO:0055078 sodium ion homeostasis	3	0.08218	0.929	0.054	Biological	GO:00550	sodium ion homeostasis	0.08218	0.27737	0	0	3	56
GO:1903053 regulation of extracellular matrix organization	3	0.08218	0.929	0.054	Biological	GO:19030	regulation of extracellular matrix organization	0.08218	0.27737	0	0	3	56
GO:2000179 positive regulation of neural precursor cell proliferation	3	0.08218	0.929	0.054	Biological	GO:20001	positive regulation of neural precursor cell proliferation	0.08218	0.27737	0	0	3	56
GO:0001892 embryonic placenta development	4	0.08223	1.238	0.044	Biological	GO:00018	embryonic placenta development	0.08223	0.27737	0	0	4	90
GO:0043367 CD4-positive, alpha-beta T cell differentiation	4	0.08223	1.238	0.044	Biological	GO:00433	CD4-positive, alpha-beta T cell differentiation	0.08223	0.27737	0	0	4	90
GO:0106027 neuron projection organization	4	0.08223	1.238	0.044	Biological	GO:01060	neuron projection organization	0.08223	0.27737	0	0	4	90
GO:0004114 3',5'-cyclic-nucleotide phosphodiesterase activity	2	0.08319	0.585	0.077	Molecular	GO:00041	3',5'-cyclic-nucleotide phosphodiesterase activity	0.08319	0.27043	0	0	2	26
GO:0004435 phosphatidylinositol phospholipase C activity	2	0.08319	0.585	0.077	Molecular	GO:00044	phosphatidylinositol phospholipase C activity	0.08319	0.27043	0	0	2	26
GO:0005416 amino acid:cation symporter activity	2	0.08319	0.585	0.077	Molecular	GO:00054	amino acid:cation symporter activity	0.08319	0.27043	0	0	2	26
GO:0007249 I-kappaB kinase/NF-kappaB signaling	9	0.08343	2.786	0.031	Biological	GO:00072	I-kappaB kinase/NF-kappaB signaling	0.08343	0.27737	0	0	9	289
GO:0016459 myosin complex	3	0.08345	0.89	0.053	Cellular C	GO:00164	myosin complex	0.08345	0.28363	0	0	3	57
GO:0007204 positive regulation of cytosolic calcium ion concentration	10	0.0837	3.096	0.03	Biological	GO:00072	positive regulation of cytosolic calcium ion concentration	0.0837	0.27737	0	0	10	332
GO:0005509 calcium ion binding	19	0.08418	5.556	0.026	Molecular	GO:00055	calcium ion binding	0.08418	0.27043	0	0	19	727
GO:0005923 bicellular tight junction	5	0.08422	1.484	0.039	Cellular C	GO:00059	bicellular tight junction	0.08422	0.28363	0	0	5	129
GO:0072657 protein localization to membrane	16	0.08469	4.954	0.027	Biological	GO:00726	protein localization to membrane	0.08469	0.27737	0	0	16	601
GO:0006493 protein O-linked glycosylation	4	0.08484	1.238	0.044	Biological	GO:00064	protein O-linked glycosylation	0.08484	0.27737	0	0	4	91
GO:0051262 protein tetramerization	4	0.08484	1.238	0.044	Biological	GO:00512	protein tetramerization	0.08484	0.27737	0	0	4	91
GO:0045926 negative regulation of growth	8	0.08497	2.477	0.032	Biological	GO:00459	negative regulation of growth	0.08497	0.27737	0	0	8	248
GO:0005539 glycosaminoglycan binding	8	0.08514	2.339	0.033	Molecular	GO:00055	glycosaminoglycan binding	0.08514	0.27043	0	0	8	244
GO:0035601 protein deacylation	5	0.08514	1.548	0.039	Biological	GO:00356	protein deacylation	0.08514	0.27737	0	0	5	128
GO:0043687 post-translational protein modification	3	0.08563	0.929	0.053	Biological	GO:00436	post-translational protein modification	0.08563	0.27737	0	0	3	57
GO:0048168 regulation of neuronal synaptic plasticity	3	0.08563	0.929	0.053	Biological	GO:00481	regulation of neuronal synaptic plasticity	0.08563	0.27737	0	0	3	57
GO:0031324 negative regulation of cellular metabolic process	55	0.08618	17.028	0.022	Biological	GO:00313	negative regulation of cellular metabolic process	0.08618	0.27737	0	0	55	2519
GO:0001702 gastrulation with mouth forming second	2	0.0863	0.619	0.074	Biological	GO:00017	gastrulation with mouth forming second	0.0863	0.27737	0	0	2	27
GO:0019098 reproductive behavior	2	0.0863	0.619	0.074	Biological	GO:00190	reproductive behavior	0.0863	0.27737	0	0	2	27
GO:0034643 establishment of mitochondrion localization, microtubule-m	2	0.0863	0.619	0.074	Biological	GO:00346	establishment of mitochondrion localization, microtubule-mediated	0.0863	0.27737	0	0	2	27
GO:0047497 mitochondrion transport along microtubule	2	0.0863	0.619	0.074	Biological	GO:00474	mitochondrion transport along microtubule	0.0863	0.27737	0	0	2	27
GO:0051291 protein heterooligomerization	2	0.0863	0.619	0.074	Biological	GO:00512	protein heterooligomerization	0.0863	0.27737	0	0	2	27
GO:0060561 apoptotic process involved in morphogenesis	2	0.0863	0.619	0.074	Biological	GO:00605	apoptotic process involved in morphogenesis	0.0863	0.27737	0	0	2	27
GO:0060706 cell differentiation involved in embryonic placenta developn	2	0.0863	0.619	0.074	Biological	GO:00607	cell differentiation involved in embryonic placenta development	0.0863	0.27737	0	0	2	27
GO:1904738 vascular associated smooth muscle cell migration	2	0.0863	0.619	0.074	Biological	GO:19047	vascular associated smooth muscle cell migration	0.0863	0.27737	0	0	2	27
GO:0008289 lipid binding	21	0.08633	6.14	0.026	Molecular	GO:00082	lipid binding	0.08633	0.27043	0	0	21	822
GO:0009892 negative regulation of metabolic process	64	0.08663	19.814	0.021	Biological	GO:00098	negative regulation of metabolic process	0.08663	0.27737	0	0	64	2981
GO:0001405 PAM complex, Tim23 associated import motor	1	0.08694	0.297	0.2	Cellular C	GO:00014	PAM complex, Tim23 associated import motor	0.08694	0.28363	0	0	1	5
GO:0016942 insulin-like growth factor binding protein complex	1	0.08694	0.297	0.2	Cellular C	GO:00169	insulin-like growth factor binding protein complex	0.08694	0.28363	0	0	1	5
GO:0030895 apolipoprotein B mRNA editing enzyme complex	1	0.08694	0.297	0.2	Cellular C	GO:00308	apolipoprotein B mRNA editing enzyme complex	0.08694	0.28363	0	0	1	5
GO:0035976 transcription factor AP-1 complex	1	0.08694	0.297	0.2	Cellular C	GO:00359	transcription factor AP-1 complex	0.08694	0.28363	0	0	1	5
GO:0097227 sperm annulus	1	0.08694	0.297	0.2	Cellular C	GO:00972	sperm annulus	0.08694	0.28363	0	0	1	5
GO:0097418 neurofibrillary tangle	1	0.08694	0.297	0.2	Cellular C	GO:00974	neurofibrillary tangle	0.08694	0.28363	0	0	1	5
GO:0097457 hippocampal mossy fiber	1	0.08694	0.297	0.2	Cellular C	GO:00974	hippocampal mossy fiber	0.08694	0.28363	0	0	1	5
GO:0099617 matrix side of mitochondrial inner membrane	1	0.08694	0.297	0.2	Cellular C	GO:00996	matrix side of mitochondrial inner membrane	0.08694	0.28363	0	0	1	5
GO:0120044 stereocilium base	1	0.08694	0.297	0.2	Cellular C	GO:01200	stereocilium base	0.08694	0.28363	0	0	1	5
GO:0061630 ubiquitin protein ligase activity	10	0.08698	2.924	0.03	Molecular	GO:00616	ubiquitin protein ligase activity	0.08698	0.27043	0	0	10	329
GO:0021987 cerebral cortex development	5	0.08733	1.548	0.039	Biological	GO:00219	cerebral cortex development	0.08733	0.27737	0	0	5	129
GO:0030048 actin filament-based movement	5	0.08733	1.548	0.039	Biological	GO:00300	actin filament-based movement	0.08733	0.27737	0	0	5	129
GO:0033555 multicellular organismal response to stress	4	0.0875	1.238	0.043	Biological	GO:00335	multicellular organismal response to stress	0.0875	0.27737	0	0	4	92
GO:0005875 microtubule associated complex	6	0.08775	1.78	0.035	Cellular C	GO:00058	microtubule associated complex	0.08775	0.28453	0	0	6	170
GO:0003274 endocardial cushion fusion	1	0.0879	0.31	0.2	Biological	GO:00032	endocardial cushion fusion	0.0879	0.27737	0	0	1	5
GO:0003284 septum primum development	1	0.0879	0.31	0.2	Biological	GO:00032	septum primum development	0.0879	0.27737	0	0	1	5
GO:0003289 atrial septum primum morphogenesis	1	0.0879	0.31	0.2	Biological	GO:00032	atrial septum primum morphogenesis	0.0879	0.27737	0	0	1	5
GO:0003431 growth plate cartilage chondrocyte development	1	0.0879	0.31	0.2	Biological	GO:00034	growth plate cartilage chondrocyte development	0.0879	0.27737	0	0	1	5
GO:0006528 asparagine metabolic process	1	0.0879	0.31	0.2	Biological	GO:00065	asparagine metabolic process	0.0879	0.27737	0	0	1	5
GO:0008594 photoreceptor cell morphogenesis	1	0.0879	0.31	0.2	Biological	GO:00085	photoreceptor cell morphogenesis	0.0879	0.27737	0	0	1	5
GO:0009786 regulation of asymmetric cell division	1	0.0879	0.31	0.2	Biological	GO:00097	regulation of asymmetric cell division	0.0879	0.27737	0	0	1	5
GO:0010641 positive regulation of platelet-derived growth factor recepto	1	0.0879	0.31	0.2	Biological	GO:00106	positive regulation of platelet-derived growth factor receptor signaling	0.0879	0.27737	0	0	1	5
GO:0014050 negative regulation of glutamate secretion	1	0.0879	0.31	0.2	Biological	GO:00140	negative regulation of glutamate secretion	0.0879	0.27737	0	0	1	5
GO:0014722 regulation of skeletal muscle contraction by calcium ion sig	1	0.0879	0.31	0.2	Biological	GO:00147	regulation of skeletal muscle contraction by calcium ion signaling	0.0879	0.27737	0	0	1	5
GO:0016103 diterpenoid catabolic process	1	0.0879	0.31	0.2	Biological	GO:00161	diterpenoid catabolic process	0.0879	0.27737	0	0	1	5
GO:0019441 tryptophan catabolic process to kynurenine	1	0.0879	0.31	0.2	Biological	GO:00194	tryptophan catabolic process to kynurenine	0.0879	0.27737	0	0	1	5
GO:0030035 microspike assembly	1	0.0879	0.31	0.2	Biological	GO:00300	microspike assembly	0.0879	0.27737	0	0	1	5
GO:0030208 dermatan sulfate biosynthetic process	1	0.0879	0.31	0.2	Biological	GO:00302	dermatan sulfate biosynthetic process	0.0879	0.27737	0	0	1	5
GO:0032417 positive regulation of sodium:proton antiporter activity	1	0.0879	0.31	0.2	Biological	GO:00324	positive regulation of sodium:proton antiporter activity	0.0879	0.27737	0	0	1	5
GO:0032596 protein transport into membrane raft	1	0.0879	0.31	0.2	Biological	GO:00325	protein transport into membrane raft	0.0879	0.27737	0	0	1	5
GO:0032898 neurotrophin production	1	0.0879	0.31	0.2	Biological	GO:00328	neurotrophin production	0.0879	0.27737	0	0	1	5
GO:0034653 retinoic acid catabolic process	1	0.0879	0.31	0.2	Biological	GO:00346	retinoic acid catabolic process	0.0879	0.27737	0	0	1	5
GO:0035905 ascending aorta development	1	0.0879	0.31	0.2	Biological	GO:00359	ascending aorta development	0.0879	0.27737	0	0	1	5
GO:0038135 ERBB2-ERBB4 signaling pathway	1	0.0879	0.31	0.2	Biological	GO:00381	ERBB2-ERBB4 signaling pathway	0.0879	0.27737	0	0	1	5
GO:0042297 vocal learning	1	0.0879	0.31	0.2	Biological	GO:00422	vocal learning	0.0879	0.27737	0	0	1	5
GO:0042494 detection of bacterial lipoprotein	1	0.0879	0.31	0.2	Biological	GO:00424	detection of bacterial lipoprotein	0.0879	0.27737	0	0	1	5
GO:0043378 positive regulation of CD8-positive, alpha-beta T cell differe	1	0.0879	0.31	0.2	Biological	GO:00433	positive regulation of CD8-positive, alpha-beta T cell differentiation	0.0879	0.27737	0	0	1	5
GO:0044752 response to human chorionic gonadotropin	1	0.0879	0.31	0.2	Biological	GO:00447	response to human chorionic gonadotropin	0.0879	0.27737	0	0	1	5
GO:0045541 negative regulation of cholesterol biosynthetic process	1	0.0879	0.31	0.2	Biological	GO:00455	negative regulation of cholesterol biosynthetic process	0.0879	0.27737	0	0	1	5
GO:0045636 positive regulation of melanocyte differentiation	1	0.0879	0.31	0.2	Biological	GO:00456	positive regulation of melanocyte differentiation	0.0879	0.27737	0	0	1	5
GO:0045917 positive regulation of complement activation	1	0.0879	0.31	0.2	Biological	GO:00459	positive regulation of complement activation	0.0879	0.27737	0	0	1	5
GO:0046898 response to cycloheximide	1	0.0879	0.31	0.2	Biological	GO:00468	response to cycloheximide	0.0879	0.27737	0	0	1	5
GO:0048386 positive regulation of retinoic acid receptor signaling pathw	1	0.0879	0.31	0.2	Biological	GO:00483	positive regulation of retinoic acid receptor signaling pathway	0.0879	0.27737	0	0	1	5
GO:0048818 positive regulation of hair follicle maturation	1	0.0879	0.31	0.2	Biological	GO:00488	positive regulation of hair follicle maturation	0.0879	0.27737	0	0	1	5
GO:0048859 formation of anatomical boundary	1	0.0879	0.31	0.2	Biological	GO:00488	formation of anatomical boundary	0.0879	0.27737	0	0	1	5
GO:0051106 positive regulation of DNA ligation	1	0.0879	0.31	0.2	Biological	GO:00511	positive regulation of DNA ligation	0.0879	0.27737	0	0	1	5
GO:0055024 regulation of cardiac muscle tissue development	1	0.0879	0.31	0.2	Biological	GO:00550	regulation of cardiac muscle tissue development	0.0879	0.27737	0	0	1	5
GO:0060066 oviduct development	1	0.0879	0.31	0.2	Biological	GO:00600	oviduct development	0.0879	0.27737	0	0	1	5
GO:0060406 positive regulation of penile erection	1	0.0879	0.31	0.2	Biological	GO:00604	positive regulation of penile erection	0.0879	0.27737	0	0	1	5
GO:0060605 tube lumen cavitation	1	0.0879	0.31	0.2	Biological	GO:00606	tube lumen cavitation	0.0879	0.27737	0	0	1	5
GO:0060662 salivary gland cavitation	1	0.0879	0.31	0.2	Biological	GO:00606	salivary gland cavitation	0.0879	0.27737	0	0	1	5
GO:0060681 branch elongation involved in ureteric bud branching	1	0.0879	0.31	0.2	Biological	GO:00606	branch elongation involved in ureteric bud branching	0.0879	0.27737	0	0	1	5
GO:0061009 common bile duct development	1	0.0879	0.31	0.2	Biological	GO:00610	common bile duct development	0.0879	0.27737	0	0	1	5
GO:0061088 regulation of sequestering of zinc ion	1	0.0879	0.31	0.2	Biological	GO:00610	regulation of sequestering of zinc ion	0.0879	0.27737	0	0	1	5
GO:0061197 fungiform papilla morphogenesis	1	0.0879	0.31	0.2	Biological	GO:00611	fungiform papilla morphogenesis	0.0879	0.27737	0	0	1	5
GO:0061217 regulation of mesonephros development	1	0.0879	0.31	0.2	Biological	GO:00612	regulation of mesonephros development	0.0879	0.27737	0	0	1	5
GO:0061523 cilium disassembly	1	0.0879	0.31	0.2	Biological	GO:00615	cilium disassembly	0.0879	0.27737	0	0	1	5
GO:0061550 cranial ganglion development	1	0.0879	0.31	0.2	Biological	GO:00615	cranial ganglion development	0.0879	0.27737	0	0	1	5
GO:0070120 ciliary neurotrophic factor-mediated signaling pathway	1	0.0879	0.31	0.2	Biological	GO:00701	ciliary neurotrophic factor-mediated signaling pathway	0.0879	0.27737	0	0	1	5

GO:0070257 positive regulation of mucus secretion	1	0.0879	0.31	0.2	Biological	GO:00702	positive regulation of mucus secretion	0.0879	0.27737	0	0	1	5
GO:0070777 D-aspartate transport	1	0.0879	0.31	0.2	Biological	GO:00707	D-aspartate transport	0.0879	0.27737	0	0	1	5
GO:0070779 D-aspartate import across plasma membrane	1	0.0879	0.31	0.2	Biological	GO:00707	D-aspartate import across plasma membrane	0.0879	0.27737	0	0	1	5
GO:0071242 cellular response to ammonium ion	1	0.0879	0.31	0.2	Biological	GO:00712	cellular response to ammonium ion	0.0879	0.27737	0	0	1	5
GO:0071681 cellular response to indole-3-methanol	1	0.0879	0.31	0.2	Biological	GO:00716	cellular response to indole-3-methanol	0.0879	0.27737	0	0	1	5
GO:0072144 glomerular mesangial cell development	1	0.0879	0.31	0.2	Biological	GO:00721	glomerular mesangial cell development	0.0879	0.27737	0	0	1	5
GO:0072162 metanephric mesenchymal cell differentiation	1	0.0879	0.31	0.2	Biological	GO:00721	metanephric mesenchymal cell differentiation	0.0879	0.27737	0	0	1	5
GO:0072223 metanephric glomerular mesangium development	1	0.0879	0.31	0.2	Biological	GO:00722	metanephric glomerular mesangium development	0.0879	0.27737	0	0	1	5
GO:0090069 regulation of ribosome biogenesis	1	0.0879	0.31	0.2	Biological	GO:00900	regulation of ribosome biogenesis	0.0879	0.27737	0	0	1	5
GO:0090131 mesenchyme migration	1	0.0879	0.31	0.2	Biological	GO:00901	mesenchyme migration	0.0879	0.27737	0	0	1	5
GO:0090185 negative regulation of kidney development	1	0.0879	0.31	0.2	Biological	GO:00901	negative regulation of kidney development	0.0879	0.27737	0	0	1	5
GO:0090206 negative regulation of cholesterol metabolic process	1	0.0879	0.31	0.2	Biological	GO:00902	negative regulation of cholesterol metabolic process	0.0879	0.27737	0	0	1	5
GO:0090461 glutamate homeostasis	1	0.0879	0.31	0.2	Biological	GO:00904	glutamate homeostasis	0.0879	0.27737	0	0	1	5
GO:0097114 NMDA glutamate receptor clustering	1	0.0879	0.31	0.2	Biological	GO:00971	NMDA glutamate receptor clustering	0.0879	0.27737	0	0	1	5
GO:0098596 imitative learning	1	0.0879	0.31	0.2	Biological	GO:00985	imitative learning	0.0879	0.27737	0	0	1	5
GO:0098713 leucine import across plasma membrane	1	0.0879	0.31	0.2	Biological	GO:00987	leucine import across plasma membrane	0.0879	0.27737	0	0	1	5
GO:0098971 anterograde dendritic transport of neurotransmitter recepto	1	0.0879	0.31	0.2	Biological	GO:00989	anterograde dendritic transport of neurotransmitter receptor complex	0.0879	0.27737	0	0	1	5
GO:0106119 negative regulation of sterol biosynthetic process	1	0.0879	0.31	0.2	Biological	GO:01061	negative regulation of sterol biosynthetic process	0.0879	0.27737	0	0	1	5
GO:1900736 regulation of phospholipase C-activating G protein-couplec	1	0.0879	0.31	0.2	Biological	GO:19007	regulation of phospholipase C-activating G protein-coupled receptor s	0.0879	0.27737	0	0	1	5
GO:1902463 protein localization to cell leading edge	1	0.0879	0.31	0.2	Biological	GO:19024	protein localization to cell leading edge	0.0879	0.27737	0	0	1	5
GO:1902623 negative regulation of neutrophil migration	1	0.0879	0.31	0.2	Biological	GO:19026	negative regulation of neutrophil migration	0.0879	0.27737	0	0	1	5
GO:1902956 regulation of mitochondrial electron transport, NADH to ub	1	0.0879	0.31	0.2	Biological	GO:19029	regulation of mitochondrial electron transport, NADH to ubiquinone	0.0879	0.27737	0	0	1	5
GO:1903801 L-leucine import across plasma membrane	1	0.0879	0.31	0.2	Biological	GO:19038	L-leucine import across plasma membrane	0.0879	0.27737	0	0	1	5
GO:1904351 negative regulation of protein catabolic process in the vacu	1	0.0879	0.31	0.2	Biological	GO:19043	negative regulation of protein catabolic process in the vacuole	0.0879	0.27737	0	0	1	5
GO:1904447 folate import across plasma membrane	1	0.0879	0.31	0.2	Biological	GO:19044	folate import across plasma membrane	0.0879	0.27737	0	0	1	5
GO:1904557 L-alanine transmembrane transport	1	0.0879	0.31	0.2	Biological	GO:19045	L-alanine transmembrane transport	0.0879	0.27737	0	0	1	5
GO:1904906 positive regulation of endothelial cell-matrix adhesion via fib	1	0.0879	0.31	0.2	Biological	GO:19049	positive regulation of endothelial cell-matrix adhesion via fibronectin	0.0879	0.27737	0	0	1	5
GO:1905007 positive regulation of epithelial to mesenchymal transition i	1	0.0879	0.31	0.2	Biological	GO:19050	positive regulation of epithelial to mesenchymal transition involved in	0.0879	0.27737	0	0	1	5
GO:1905166 negative regulation of lysosomal protein catabolic process	1	0.0879	0.31	0.2	Biological	GO:19051	negative regulation of lysosomal protein catabolic process	0.0879	0.27737	0	0	1	5
GO:1905636 positive regulation of RNA polymerase II regulatory region	1	0.0879	0.31	0.2	Biological	GO:19056	positive regulation of RNA polymerase II regulatory region sequence-	0.0879	0.27737	0	0	1	5
GO:1905651 regulation of artery morphogenesis	1	0.0879	0.31	0.2	Biological	GO:19056	regulation of artery morphogenesis	0.0879	0.27737	0	0	1	5
GO:1905653 positive regulation of artery morphogenesis	1	0.0879	0.31	0.2	Biological	GO:19056	positive regulation of artery morphogenesis	0.0879	0.27737	0	0	1	5
GO:1905749 regulation of endosome to plasma membrane protein trans	1	0.0879	0.31	0.2	Biological	GO:19057	regulation of endosome to plasma membrane protein transport	0.0879	0.27737	0	0	1	5
GO:1905908 positive regulation of amyloid fibril formation	1	0.0879	0.31	0.2	Biological	GO:19059	positive regulation of amyloid fibril formation	0.0879	0.27737	0	0	1	5
GO:2000467 positive regulation of glycogen (starch) synthase activity	1	0.0879	0.31	0.2	Biological	GO:20004	positive regulation of glycogen (starch) synthase activity	0.0879	0.27737	0	0	1	5
GO:2000491 positive regulation of hepatic stellate cell activation	1	0.0879	0.31	0.2	Biological	GO:20004	positive regulation of hepatic stellate cell activation	0.0879	0.27737	0	0	1	5
GO:0050877 nervous system process	35	0.08792	10.836	0.023	Biological	GO:00508	nervous system process	0.08792	0.27737	0	0	35	1517
GO:0008198 ferrous iron binding	2	0.08878	0.585	0.074	Molecular	GO:00081	ferrous iron binding	0.08878	0.27043	0	0	2	27
GO:0001961 positive regulation of cytokine-mediated signaling pathway	3	0.08914	0.929	0.052	Biological	GO:00019	positive regulation of cytokine-mediated signaling pathway	0.08914	0.2807	0	0	3	58
GO:0030514 negative regulation of BMP signaling pathway	3	0.08914	0.929	0.052	Biological	GO:00305	negative regulation of BMP signaling pathway	0.08914	0.2807	0	0	3	58
GO:0043113 receptor clustering	3	0.08914	0.929	0.052	Biological	GO:00431	receptor clustering	0.08914	0.2807	0	0	3	58
GO:0001093 TFIIB-class transcription factor binding	1	0.08931	0.292	0.2	Molecular	GO:00010	TFIIB-class transcription factor binding	0.08931	0.27043	0	0	1	5
GO:0001609 G protein-coupled adenosine receptor activity	1	0.08931	0.292	0.2	Molecular	GO:00016	G protein-coupled adenosine receptor activity	0.08931	0.27043	0	0	1	5
GO:0004468 lysine N-acetyltransferase activity, acting on acetyl phosph	1	0.08931	0.292	0.2	Molecular	GO:00044	lysine N-acetyltransferase activity, acting on acetyl phosphate as dor	0.08931	0.27043	0	0	1	5
GO:0004630 phospholipase D activity	1	0.08931	0.292	0.2	Molecular	GO:00046	phospholipase D activity	0.08931	0.27043	0	0	1	5
GO:0004957 prostaglandin E receptor activity	1	0.08931	0.292	0.2	Molecular	GO:00049	prostaglandin E receptor activity	0.08931	0.27043	0	0	1	5
GO:0005030 neurotrophin receptor activity	1	0.08931	0.292	0.2	Molecular	GO:00050	neurotrophin receptor activity	0.08931	0.27043	0	0	1	5
GO:0005163 nerve growth factor receptor binding	1	0.08931	0.292	0.2	Molecular	GO:00051	nerve growth factor receptor binding	0.08931	0.27043	0	0	1	5
GO:0005298 proline:sodium symporter activity	1	0.08931	0.292	0.2	Molecular	GO:00052	proline:sodium symporter activity	0.08931	0.27043	0	0	1	5
GO:0005314 high-affinity glutamate transmembrane transporter activity	1	0.08931	0.292	0.2	Molecular	GO:00053	high-affinity glutamate transmembrane transporter activity	0.08931	0.27043	0	0	1	5
GO:0005497 androgen binding	1	0.08931	0.292	0.2	Molecular	GO:00054	androgen binding	0.08931	0.27043	0	0	1	5
GO:0008889 glycerophosphodiester phosphodiesterase activity	1	0.08931	0.292	0.2	Molecular	GO:00088	glycerophosphodiester phosphodiesterase activity	0.08931	0.27043	0	0	1	5
GO:0015433 ABC-type peptide antigen transporter activity	1	0.08931	0.292	0.2	Molecular	GO:00154	ABC-type peptide antigen transporter activity	0.08931	0.27043	0	0	1	5
GO:0015440 ABC-type peptide transporter activity	1	0.08931	0.292	0.2	Molecular	GO:00154	ABC-type peptide transporter activity	0.08931	0.27043	0	0	1	5
GO:0015501 glutamate:sodium symporter activity	1	0.08931	0.292	0.2	Molecular	GO:00155	glutamate:sodium symporter activity	0.08931	0.27043	0	0	1	5
GO:0016723 oxidoreductase activity, acting on metal ions, NAD or NAD	1	0.08931	0.292	0.2	Molecular	GO:00167	oxidoreductase activity, acting on metal ions, NAD or NADP as acce	0.08931	0.27043	0	0	1	5
GO:0019834 phospholipase A2 inhibitor activity	1	0.08931	0.292	0.2	Molecular	GO:00198	phospholipase A2 inhibitor activity	0.08931	0.27043	0	0	1	5
GO:0022849 glutamate-gated calcium ion channel activity	1	0.08931	0.292	0.2	Molecular	GO:00228	glutamate-gated calcium ion channel activity	0.08931	0.27043	0	0	1	5
GO:0031779 melanocortin receptor binding	1	0.08931	0.292	0.2	Molecular	GO:00317	melanocortin receptor binding	0.08931	0.27043	0	0	1	5
GO:0038132 neuregulin binding	1	0.08931	0.292	0.2	Molecular	GO:00381	neuregulin binding	0.08931	0.27043	0	0	1	5
GO:0086083 cell adhesive protein binding involved in bundle of His cell-	1	0.08931	0.292	0.2	Molecular	GO:00860	cell adhesive protein binding involved in bundle of His cell-Purkinje m	0.08931	0.27043	0	0	1	5
GO:0097109 neuroligin family protein binding	1	0.08931	0.292	0.2	Molecular	GO:00971	neuroligin family protein binding	0.08931	0.27043	0	0	1	5
GO:0140444 cytoskeleton-nuclear membrane anchor activity	1	0.08931	0.292	0.2	Molecular	GO:01404	cytoskeleton-nuclear membrane anchor activity	0.08931	0.27043	0	0	1	5
GO:0048706 embryonic skeletal system development	5	0.08954	1.548	0.038	Biological	GO:00487	embryonic skeletal system development	0.08954	0.28146	0	0	5	130
GO:1990266 neutrophil migration	5	0.08954	1.548	0.038	Biological	GO:19902	neutrophil migration	0.08954	0.28146	0	0	5	130
GO:1903362 regulation of cellular protein catabolic process	8	0.08955	2.477	0.032	Biological	GO:19033	regulation of cellular protein catabolic process	0.08955	0.28146	0	0	8	251
GO:0009953 dorsal/ventral pattern formation	4	0.09019	1.238	0.043	Biological	GO:00099	dorsal/ventral pattern formation	0.09019	0.28309	0	0	4	93
GO:0034644 cellular response to UV	4	0.09019	1.238	0.043	Biological	GO:00346	cellular response to UV	0.09019	0.28309	0	0	4	93
GO:0060284 regulation of cell development	14	0.09108	4.334	0.027	Biological	GO:00602	regulation of cell development	0.09108	0.28571	0	0	14	516
GO:0003012 muscle system process	12	0.09123	3.715	0.028	Biological	GO:00030	muscle system process	0.09123	0.28601	0	0	12	426
GO:0019904 protein domain specific binding	18	0.09124	5.263	0.026	Molecular	GO:00199	protein domain specific binding	0.09124	0.27531	0	0	18	689
GO:0007156 homophilic cell adhesion via plasma membrane adhesion r	6	0.09137	1.858	0.035	Biological	GO:00071	homophilic cell adhesion via plasma membrane adhesion molecules	0.09137	0.28625	0	0	6	170
GO:0005770 late endosome	9	0.09146	2.671	0.03	Cellular C	GO:00057	late endosome	0.09146	0.29439	0	0	9	298
GO:0010996 response to auditory stimulus	2	0.09186	0.619	0.071	Biological	GO:00109	response to auditory stimulus	0.09186	0.28637	0	0	2	28
GO:0014072 response to isoquinoline alkaloid	2	0.09186	0.619	0.071	Biological	GO:00140	response to isoquinoline alkaloid	0.09186	0.28637	0	0	2	28
GO:0031954 positive regulation of protein autophosphorylation	2	0.09186	0.619	0.071	Biological	GO:00319	positive regulation of protein autophosphorylation	0.09186	0.28637	0	0	2	28
GO:0043278 response to morphine	2	0.09186	0.619	0.071	Biological	GO:00432	response to morphine	0.09186	0.28637	0	0	2	28
GO:0044331 cell-cell adhesion mediated by cadherin	2	0.09186	0.619	0.071	Biological	GO:00443	cell-cell adhesion mediated by cadherin	0.09186	0.28637	0	0	2	28
GO:0071577 zinc ion transmembrane transport	2	0.09186	0.619	0.071	Biological	GO:00715	zinc ion transmembrane transport	0.09186	0.28637	0	0	2	28
GO:0072207 metanephric epithelium development	2	0.09186	0.619	0.071	Biological	GO:00722	metanephric epithelium development	0.09186	0.28637	0	0	2	28
GO:0150146 cell junction disassembly	2	0.09186	0.619	0.071	Biological	GO:01501	cell junction disassembly	0.09186	0.28637	0	0	2	28
GO:0098793 presynapse	15	0.09223	4.451	0.026	Cellular C	GO:00987	presynapse	0.09223	0.29439	0	0	15	569
GO:0005667 transcription regulator complex	14	0.09244	4.154	0.027	Cellular C	GO:00056	transcription regulator complex	0.09244	0.29439	0	0	14	523
GO:0097305 response to alcohol	8	0.09269	2.477	0.032	Biological	GO:00973	response to alcohol	0.09269	0.28812	0	0	8	253
GO:0008347 glial cell migration	3	0.09271	0.929	0.051	Biological	GO:00083	glial cell migration	0.09271	0.28812	0	0	3	59
GO:0048599 oocyte development	3	0.09271	0.929	0.051	Biological	GO:00485	oocyte development	0.09271	0.28812	0	0	3	59
GO:0051289 protein homotetramerization	3	0.09271	0.929	0.051	Biological	GO:00512	protein homotetramerization	0.09271	0.28812	0	0	3	59
GO:0055008 cardiac muscle tissue morphogenesis	3	0.09271	0.929	0.051	Biological	GO:00550	cardiac muscle tissue morphogenesis	0.09271	0.28812	0	0	3	59
GO:0005874 microtubule	13	0.09338	3.858	0.027	Cellular C	GO:00058	microtubule	0.09338	0.29561	0	0	13	478
GO:0002521 leukocyte differentiation	16	0.0934	4.954	0.026	Biological	GO:00025	leukocyte differentiation	0.0934	0.29008	0	0	16	610
GO:0046683 response to organophosphorus	5	0.09406	1.548	0.038	Biological	GO:00466	response to organophosphorus	0.09406	0.29176	0	0	5	132
GO:0098732 macromolecule deacylation	5	0.09406	1.548	0.038	Biological	GO:00987	macromolecule deacylation	0.09406	0.29176	0	0	5	132
GO:0006790 sulfur compound metabolic process	10	0.09424	3.096	0.029	Biological	GO:00067	sulfur compound metabolic process	0.09424	0.29213	0	0	10	340
GO:0004112 cyclic-nucleotide phosphodiesterase activity	2	0.09448	0.585	0.071	Molecular	GO:00041	cyclic-nucleotide phosphodiesterase activity	0.09448	0.2841	0	0	2	28
GO:0098794 postsynapse	17	0.09468	5.045	0.026	Cellular C	GO:00987	postsynapse	0.09468	0.29764	0	0	17	665
GO:1901564 organonitrogen compound metabolic process	129	0.09478	39.938	0.02	Biological	GO:19015	organonitrogen compound metabolic process	0.09478	0.29364	0	0	129	6431
GO:0002262 myeloid cell homeostasis	6	0.09531	1.858	0.035	Biological	GO:00022	myeloid cell homeostasis	0.09531	0.29508	0	0	6	172
GO:0098839 postsynaptic density membrane	4	0.09551	1.187	0.042	Cellular C	GO:00988	postsynaptic density membrane	0.09551	0.29764	0	0	4	96
GO:0006936 muscle contraction	10	0.09561	3.096	0.029	Biological	GO:00069	muscle contraction	0.09561	0.29571	0	0	10	341
GO:0030512 negative regulation of transforming growth factor beta rece	4	0.09569	1.238	0.042	Biological	GO:00305	negative regulation of transforming growth factor beta receptor signal	0.09569	0.29571	0	0	4	95
GO:0048704 embryonic skeletal system morphogenesis	4	0.09569	1.238	0.042	Biological	GO:00487	embryonic skeletal system morphogenesis	0.09569	0.29571	0	0	4	95
GO:0034364 high-density lipoprotein particle	2	0.09569	0.593	0.069	Cellular C	GO:00343	high-density lipoprotein particle	0.09569	0.29764	0	0	2	29
GO:0001558 regulation of cell growth	12	0.09604	3.715	0.028	Biological	GO:00015	regulation of cell growth	0.09604	0.29662	0	0	12	430
GO:0008610 lipid biosynthetic process	18	0.09613	5.573	0.025	Biological	GO:00086	lipid biosynthetic process	0.09613	0.29669	0	0	18	706
GO:0002753 cytoplasmic pattern recognition receptor signaling pathway	3	0.09634	0.929	0.05	Biological	GO:00027	cytoplasmic pattern recognition receptor signaling pathway	0.09634	0.29669	0	0	3	60
GO:0003179 heart valve morphogenesis	3	0.09634	0.929	0.05	Biological	GO:00031	heart valve morphogenesis	0.09634	0.29669	0	0	3	60
GO:0060135 maternal process involved in female pregnancy	3	0.09634	0.929	0.05	Biological	GO:00601	maternal process involved in female pregnancy	0.09634	0.29669	0	0	3	60
GO:1904375 regulation of protein localization to cell periphery	5	0.09636	1.548	0.038	Biological	GO:19043	regulation of protein localization to cell periphery	0.09636	0.29669	0	0	5	133
GO:0071214 cellular response to abiotic stimulus	10	0.09699	3.096	0.029	Biological	GO:00712	cellular response to abiotic stimulus	0.09699	0.29826	0	0	10	342
GO:0104004 cellular response to environmental stimulus	10	0.09699	3.096	0.029	Biological	GO:01040	cellular response to environmental stimulus	0.09699	0.29826	0	0	10	342
GO:0006508 proteolysis	40	0.09706	12.384	0.022	Biological	GO:00065	proteolysis	0.09706	0.2983	0	0	40	1781
GO:0030427 site of polarized growth	6	0.09744	1.78	0.034	Cellular C	GO:00304	site of polarized growth	0.09744	0.2999	0	0	6	175
GO:0051259 protein complex oligomerization	8	0.0975	2.477	0.031	Biological	GO:00512	protein complex oligomerization	0.0975	0.29861	0	0	8	256
GO:0002052 positive regulation of neuroblast proliferation	2	0.09752	0.619	0.069	Biological	GO:00020	positive regulation of neuroblast proliferation	0.09752	0.29861	0	0	2	29
GO:0009312 oligosaccharide biosynthetic process	2	0.09752	0.619	0.069	Biological	GO:00093	oligosaccharide biosynthetic process	0.09752	0.29861	0	0	2	29
GO:0033137 negative regulation of peptidyl-serine phosphorylation	2	0.09752	0.619	0.069	Biological	GO:00331	negative regulation of peptidyl-serine phosphorylation	0.09752	0.29861	0	0	2	29
GO:0071480 cellular response to gamma radiation	2	0.09752	0.619	0.069	Biological	GO:00714	cellular response to gamma radiation	0.09752	0.29861	0	0	2	29
GO:0099170 postsynaptic modulation of chemical synaptic transmissior	2	0.09752	0.619	0.069	Biological	GO:00991	postsynaptic modulation of chemical synaptic transmission	0.09752	0.29861	0	0	2	29
GO:0070160 tight junction	5	0.09754	1.484	0.037	Cellular C	GO:00701	tight junction	0.09754	0.2999	0	0	5	135
GO:0031329 regulation of cellular catabolic process	21	0.09888	6.502	0.025	Biological	GO:00313	regulation of cellular catabolic process	0.09888	0.29898	0	0	21	851
GO:0000977 RNA polymerase II transcription regulatory region sequenc	33	0.09897	9.649	0.023	Molecular	GO:00009	RNA polymerase II transcription regulatory region sequence-specific	0.09897	0.29657	0	0	33	1411
GO:0006687 glycosphingolipid metabolic process	3	0.10003	0.929	0.049	Biological	GO:00066	glycosphingolipid metabolic process	0.10003	0.29898	0	0	3	61
GO:0030858 positive regulation of epithelial cell differentiation	3	0.10003	0.929	0.049	Biological	GO:00308	positive regulation of epithelial cell differentiation	0.10003	0.29898	0	0	3	61
GO:0032731 positive regulation of interleukin-1 beta production	3	0.10003	0.929	0.049	Biological	GO:00327	positive regulation of interleukin-1 beta production	0.10003	0.29898	0	0	3	61
GO:1905517 macrophage migration	3	0.10003	0.929	0.049	Biological	GO:19055	macrophage migration	0.10003	0.29898	0	0	3	61
GO:0000981 DNA-binding transcription factor activity, RNA polymerase	32	0.10008	9.357	0.023	Molecular	GO:00009	DNA-binding transcription factor activity, RNA polymerase II-specific	0.10008	0.29666	0	0	32	1364
GO:0004629 phospholipase C activity	2	0.10028	0.585	0.069	Molecular	GO:00046	phospholipase C activity	0.10028	0.29666	0	0	2	29
GO:0080090 regulation of primary metabolic process	117	0.10067	36.223	0.02	Biological	GO:00800	regulation of primary metabolic process	0.10067	0.29898	0	0	117	5803
GO:0140694 non-membrane-bounded organelle assembly	11	0.10078	3.406	0.028	Biological	GO:01406	non-membrane-bounded organelle assembly	0.10078	0.29898	0	0	11	389
GO:0044706 multi-multicellular organism process	7	0.10108	2.167	0.032	Biological	GO:00447	multi-multicellular organism process	0.10108	0.29898	0	0	7	216
GO:1903510 mucopolysaccharide metabolic process	4	0.10134	1.238	0.041	Biological	GO:19035	mucopolysaccharide metabolic process	0.10134	0.29898	0	0	4	97
GO:0005640 nuclear outer membrane	2	0.10135	0.593	0.067	Cellular C	GO:00056	nuclear outer membrane	0.10135	0.30174	0	0	2	30
GO:2000058 regulation of ubiquitin-dependent protein catabolic process	6	0.10138	1.858	0.034	Biological	GO:20000	regulation of ubiquitin-dependent protein catabolic process	0.10138	0.29898	0	0	6	175
GO:0033993 response to lipid	22	0.10163	6.811	0.024	Biological	GO:00339	response to lipid	0.10163	0.29898	0	0	22	902
GO:0051093 negative regulation of developmental process	23	0.10242	7.121	0.024	Biological	GO:00510	negative regulation of developmental process	0.10242	0.29898	0	0	23	951
GO:0043523 regulation of neuron apoptotic process	7	0.10292	2.167	0.032	Biological	GO:00435	regulation of neuron apoptotic process	0.10292	0.29898	0	0	7	217
GO:0019725 cellular homeostasis	23	0.10327	7.121	0.024	Biological	GO:00197	cellular homeostasis	0.10327	0.29898	0	0	23	952
GO:0001516 prostaglandin biosynthetic process	2	0.10328	0.619	0.067	Biological	GO:00015	prostaglandin biosynthetic process	0.10328	0.29898	0	0	2	30
GO:0003156 regulation of animal organ formation	2	0.10328	0.619	0.067	Biological	GO:00031	regulation of animal organ formation	0.10328	0.29898	0	0	2	30
GO:0006829 zinc ion transport	2	0.10328	0.619	0.067	Biological	GO:00068	zinc ion transport	0.10328	0.29898	0	0	2	30
GO:0021602 cranial nerve morphogenesis	2	0.10328	0.619	0.067	Biological	GO:00216	cranial nerve morphogenesis	0.10328	0.29898	0	0	2	30
GO:0030878 thyroid gland development	2	0.10328	0.619	0.067	Biological	GO:00308	thyroid gland development	0.10328	0.29898	0	0	2	30
GO:0035640 exploration behavior	2	0.10328	0.619	0.067	Biological	GO:00356	exploration behavior	0.10328	0.29898	0	0	2	30
GO:0045736 negative regulation of cyclin-dependent protein serine/thre	2	0.10328	0.619	0.067	Biological	GO:00457	negative regulation of cyclin-dependent protein serine/threonine kinas	0.10328	0.29898	0	0	2	30
GO:0046457 prostanoid biosynthetic process	2	0.10328	0.619	0.067	Biological	GO:00464	prostanoid biosynthetic process	0.10328	0.29898	0	0	2	30
GO:0051654 establishment of mitochondrion localization	2	0.10328	0.619	0.067	Biological	GO:00516	establishment of mitochondrion localization	0.10328	0.29898	0	0	2	30
GO:0098698 postsynaptic specialization assembly	2	0.10328	0.619	0.067	Biological	GO:00986	postsynaptic specialization assembly	0.10328	0.29898	0	0	2	30
GO:0099560 synaptic membrane adhesion	2	0.10328	0.619	0.067	Biological	GO:00995	synaptic membrane adhesion	0.10328	0.29898	0	0	2	30
GO:1904030 negative regulation of cyclin-dependent protein kinase acti	2	0.10328	0.619	0.067	Biological	GO:19040	negative regulation of cyclin-dependent protein kinase activity	0.10328	0.29898	0	0	2	30
GO:0005639 integral component of nuclear inner membrane	1	0.10341	0.297	0.167	Cellular C	GO:00056	integral component of nuclear inner membrane	0.10341	0.30174	0	0	1	6
GO:0005955 calcineurin complex	1	0.10341	0.297	0.167	Cellular C	GO:00059	calcineurin complex	0.10341	0.30174	0	0	1	6
GO:0031088 platelet dense granule membrane	1	0.10341	0.297	0.167	Cellular C	GO:00310	platelet dense granule membrane	0.10341	0.30174	0	0	1	6

GO:0031229 intrinsic component of nuclear inner membrane	1	0.10341	0.297	0.167	Cellular C	GO:00312	intrinsic component of nuclear inner membrane	0.10341	0.30174	0	0	1	6
GO:0031265 CD95 death-inducing signaling complex	1	0.10341	0.297	0.167	Cellular C	GO:00312	CD95 death-inducing signaling complex	0.10341	0.30174	0	0	1	6
GO:0044302 dentate gyrus mossy fiber	1	0.10341	0.297	0.167	Cellular C	GO:00443	dentate gyrus mossy fiber	0.10341	0.30174	0	0	1	6
GO:0097342 ripoptosome	1	0.10341	0.297	0.167	Cellular C	GO:00973	ripoptosome	0.10341	0.30174	0	0	1	6
GO:0097487 multivesicular body, internal vesicle	1	0.10341	0.297	0.167	Cellular C	GO:00974	multivesicular body, internal vesicle	0.10341	0.30174	0	0	1	6
GO:0061659 ubiquitin-like protein ligase activity	10	0.10373	2.924	0.029	Molecular	GO:00616	ubiquitin-like protein ligase activity	0.10373	0.29666	0	0	10	341
GO:0006998 nuclear envelope organization	3	0.10377	0.929	0.048	Biological	GO:00069	nuclear envelope organization	0.10377	0.29898	0	0	3	62
GO:0016836 hydro-lyase activity	3	0.10383	0.877	0.049	Molecular	GO:00168	hydro-lyase activity	0.10383	0.29666	0	0	3	61
GO:0031982 vesicle	82	0.10414	24.332	0.021	Cellular C	GO:00319	vesicle	0.10414	0.30174	0	0	82	3998
GO:0010717 regulation of epithelial to mesenchymal transition	4	0.10423	1.238	0.041	Biological	GO:00107	regulation of epithelial to mesenchymal transition	0.10423	0.29898	0	0	4	98
GO:0032755 positive regulation of interleukin-6 production	4	0.10423	1.238	0.041	Biological	GO:00327	positive regulation of interleukin-6 production	0.10423	0.29898	0	0	4	98
GO:0001794 type IIa hypersensitivity	1	0.10453	0.31	0.167	Biological	GO:0001	type IIa hypersensitivity	0.10453	0.29898	0	0	1	6
GO:0001999 renal response to blood flow involved in circulatory renin-a	1	0.10453	0.31	0.167	Biological	GO:00019	renal response to blood flow involved in circulatory renin-angiotensin	0.10453	0.29898	0	0	1	6
GO:0002001 renin secretion into blood stream	1	0.10453	0.31	0.167	Biological	GO:00020	renin secretion into blood stream	0.10453	0.29898	0	0	1	6
GO:0002155 regulation of thyroid hormone mediated signaling pathway	1	0.10453	0.31	0.167	Biological	GO:0002	regulation of thyroid hormone mediated signaling pathway	0.10453	0.29898	0	0	1	6
GO:0002248 connective tissue replacement involved in inflammatory res	1	0.10453	0.31	0.167	Biological	GO:00022	connective tissue replacement involved in inflammatory response wo	0.10453	0.29898	0	0	1	6
GO:0002372 myeloid dendritic cell cytokine production	1	0.10453	0.31	0.167	Biological	GO:00023	myeloid dendritic cell cytokine production	0.10453	0.29898	0	0	1	6
GO:0002445 type II hypersensitivity	1	0.10453	0.31	0.167	Biological	GO:00024	type II hypersensitivity	0.10453	0.29898	0	0	1	6
GO:0002733 regulation of myeloid dendritic cell cytokine production	1	0.10453	0.31	0.167	Biological	GO:0002	regulation of myeloid dendritic cell cytokine production	0.10453	0.29898	0	0	1	6
GO:0002735 positive regulation of myeloid dendritic cell cytokine produc	1	0.10453	0.31	0.167	Biological	GO:0002	positive regulation of myeloid dendritic cell cytokine production	0.10453	0.29898	0	0	1	6
GO:0002885 positive regulation of hypersensitivity	1	0.10453	0.31	0.167	Biological	GO:00028	positive regulation of hypersensitivity	0.10453	0.29898	0	0	1	6
GO:0003278 apoptotic process involved in heart morphogenesis	1	0.10453	0.31	0.167	Biological	GO:00032	apoptotic process involved in heart morphogenesis	0.10453	0.29898	0	0	1	6
GO:0003402 planar cell polarity pathway involved in axis elongation	1	0.10453	0.31	0.167	Biological	GO:00034	planar cell polarity pathway involved in axis elongation	0.10453	0.29898	0	0	1	6
GO:0003415 chondrocyte hypertrophy	1	0.10453	0.31	0.167	Biological	GO:00034	chondrocyte hypertrophy	0.10453	0.29898	0	0	1	6
GO:0006564 L-serine biosynthetic process	1	0.10453	0.31	0.167	Biological	GO:00065	L-serine biosynthetic process	0.10453	0.29898	0	0	1	6
GO:0006678 glucosylceramide metabolic process	1	0.10453	0.31	0.167	Biological	GO:00066	glucosylceramide metabolic process	0.10453	0.29898	0	0	1	6
GO:0008582 regulation of synaptic assembly at neuromuscular junction	1	0.10453	0.31	0.167	Biological	GO:00085	regulation of synaptic assembly at neuromuscular junction	0.10453	0.29898	0	0	1	6
GO:0010040 response to iron(II) ion	1	0.10453	0.31	0.167	Biological	GO:00100	response to iron(II) ion	0.10453	0.29898	0	0	1	6
GO:0010668 ectodermal cell differentiation	1	0.10453	0.31	0.167	Biological	GO:00106	ectodermal cell differentiation	0.10453	0.29898	0	0	1	6
GO:0015677 copper ion import	1	0.10453	0.31	0.167	Biological	GO:00156	copper ion import	0.10453	0.29898	0	0	1	6
GO:0015827 tryptophan transport	1	0.10453	0.31	0.167	Biological	GO:00158	tryptophan transport	0.10453	0.29898	0	0	1	6
GO:0021902 commitment of neuronal cell to specific neuron type in fore	1	0.10453	0.31	0.167	Biological	GO:00219	commitment of neuronal cell to specific neuron type in forebrain	0.10453	0.29898	0	0	1	6
GO:0032229 negative regulation of synaptic transmission, GABAergic	1	0.10453	0.31	0.167	Biological	GO:00322	negative regulation of synaptic transmission, GABAergic	0.10453	0.29898	0	0	1	6
GO:0032241 positive regulation of nucleobase-containing compound tra	1	0.10453	0.31	0.167	Biological	GO:00322	positive regulation of nucleobase-containing compound transport	0.10453	0.29898	0	0	1	6
GO:0032534 regulation of microvillus assembly	1	0.10453	0.31	0.167	Biological	GO:00325	regulation of microvillus assembly	0.10453	0.29898	0	0	1	6
GO:0032799 low-density lipoprotein receptor particle metabolic process	1	0.10453	0.31	0.167	Biological	GO:00327	low-density lipoprotein receptor particle metabolic process	0.10453	0.29898	0	0	1	6
GO:0032802 low-density lipoprotein particle receptor catabolic process	1	0.10453	0.31	0.167	Biological	GO:00328	low-density lipoprotein particle receptor catabolic process	0.10453	0.29898	0	0	1	6
GO:0032803 regulation of low-density lipoprotein particle receptor catab	1	0.10453	0.31	0.167	Biological	GO:00328	regulation of low-density lipoprotein particle receptor catabolic proces	0.10453	0.29898	0	0	1	6
GO:0033091 positive regulation of immature T cell proliferation	1	0.10453	0.31	0.167	Biological	GO:00330	positive regulation of immature T cell proliferation	0.10453	0.29898	0	0	1	6
GO:0033092 positive regulation of immature T cell proliferation in thymu	1	0.10453	0.31	0.167	Biological	GO:00330	positive regulation of immature T cell proliferation in thymus	0.10453	0.29898	0	0	1	6
GO:0033684 regulation of luteinizing hormone secretion	1	0.10453	0.31	0.167	Biological	GO:00336	regulation of luteinizing hormone secretion	0.10453	0.29898	0	0	1	6
GO:0034382 chylomicron remnant clearance	1	0.10453	0.31	0.167	Biological	GO:00343	chylomicron remnant clearance	0.10453	0.29898	0	0	1	6
GO:0034447 very-low-density lipoprotein particle clearance	1	0.10453	0.31	0.167	Biological	GO:00344	very-low-density lipoprotein particle clearance	0.10453	0.29898	0	0	1	6
GO:0038138 ERBB4-ERBB4 signaling pathway	1	0.10453	0.31	0.167	Biological	GO:0038	ERBB4-ERBB4 signaling pathway	0.10453	0.29898	0	0	1	6
GO:0042482 positive regulation of odontogenesis	1	0.10453	0.31	0.167	Biological	GO:00424	positive regulation of odontogenesis	0.10453	0.29898	0	0	1	6
GO:0045188 regulation of circadian sleep/wake cycle, non-REM sleep	1	0.10453	0.31	0.167	Biological	GO:0045	regulation of circadian sleep/wake cycle, non-REM sleep	0.10453	0.29898	0	0	1	6
GO:0045627 positive regulation of T-helper 1 cell differentiation	1	0.10453	0.31	0.167	Biological	GO:00456	positive regulation of T-helper 1 cell differentiation	0.10453	0.29898	0	0	1	6
GO:0045634 regulation of melanocyte differentiation	1	0.10453	0.31	0.167	Biological	GO:00456	regulation of melanocyte differentiation	0.10453	0.29898	0	0	1	6
GO:0046532 regulation of photoreceptor cell differentiation	1	0.10453	0.31	0.167	Biological	GO:00465	regulation of photoreceptor cell differentiation	0.10453	0.29898	0	0	1	6
GO:0046533 negative regulation of photoreceptor cell differentiation	1	0.10453	0.31	0.167	Biological	GO:00465	negative regulation of photoreceptor cell differentiation	0.10453	0.29898	0	0	1	6
GO:0046880 regulation of follicle-stimulating hormone secretion	1	0.10453	0.31	0.167	Biological	GO:00468	regulation of follicle-stimulating hormone secretion	0.10453	0.29898	0	0	1	6
GO:0046881 positive regulation of follicle-stimulating hormone secretion	1	0.10453	0.31	0.167	Biological	GO:00468	positive regulation of follicle-stimulating hormone secretion	0.10453	0.29898	0	0	1	6
GO:0048625 myoblast fate commitment	1	0.10453	0.31	0.167	Biological	GO:00486	myoblast fate commitment	0.10453	0.29898	0	0	1	6
GO:0048866 stem cell fate specification	1	0.10453	0.31	0.167	Biological	GO:00488	stem cell fate specification	0.10453	0.29898	0	0	1	6
GO:0051105 regulation of DNA ligation	1	0.10453	0.31	0.167	Biological	GO:0051	regulation of DNA ligation	0.10453	0.29898	0	0	1	6
GO:0051725 protein de-ADP-ribosylation	1	0.10453	0.31	0.167	Biological	GO:0051	protein de-ADP-ribosylation	0.10453	0.29898	0	0	1	6
GO:0060359 response to ammonium ion	1	0.10453	0.31	0.167	Biological	GO:00603	response to ammonium ion	0.10453	0.29898	0	0	1	6
GO:0060526 prostate glandular acinus morphogenesis	1	0.10453	0.31	0.167	Biological	GO:00605	prostate glandular acinus morphogenesis	0.10453	0.29898	0	0	1	6
GO:0060527 prostate epithelial cord arborization involved in prostate gla	1	0.10453	0.31	0.167	Biological	GO:00605	prostate epithelial cord arborization involved in prostate glandular aci	0.10453	0.29898	0	0	1	6
GO:0060685 regulation of prostatic bud formation	1	0.10453	0.31	0.167	Biological	GO:00606	regulation of prostatic bud formation	0.10453	0.29898	0	0	1	6
GO:0060718 chorionic trophoblast cell differentiation	1	0.10453	0.31	0.167	Biological	GO:00607	chorionic trophoblast cell differentiation	0.10453	0.29898	0	0	1	6
GO:0060789 hair follicle placode formation	1	0.10453	0.31	0.167	Biological	GO:00607	hair follicle placode formation	0.10453	0.29898	0	0	1	6
GO:0061026 cardiac muscle tissue regeneration	1	0.10453	0.31	0.167	Biological	GO:00610	cardiac muscle tissue regeneration	0.10453	0.29898	0	0	1	6
GO:0061046 regulation of branching involved in lung morphogenesis	1	0.10453	0.31	0.167	Biological	GO:00610	regulation of branching involved in lung morphogenesis	0.10453	0.29898	0	0	1	6
GO:0061196 fungiform papilla development	1	0.10453	0.31	0.167	Biological	GO:0061	fungiform papilla development	0.10453	0.29898	0	0	1	6
GO:0070427 nucleotide-binding oligomerization domain containing 1 sig	1	0.10453	0.31	0.167	Biological	GO:00704	nucleotide-binding oligomerization domain containing 1 signaling path	0.10453	0.29898	0	0	1	6
GO:0071373 cellular response to luteinizing hormone stimulus	1	0.10453	0.31	0.167	Biological	GO:00713	cellular response to luteinizing hormone stimulus	0.10453	0.29898	0	0	1	6
GO:0071608 macrophage inflammatory protein-1 alpha production	1	0.10453	0.31	0.167	Biological	GO:00716	macrophage inflammatory protein-1 alpha production	0.10453	0.29898	0	0	1	6
GO:0071640 regulation of macrophage inflammatory protein 1 alpha pro	1	0.10453	0.31	0.167	Biological	GO:00716	regulation of macrophage inflammatory protein 1 alpha production	0.10453	0.29898	0	0	1	6
GO:0071680 response to indole-3-methanol	1	0.10453	0.31	0.167	Biological	GO:00716	response to indole-3-methanol	0.10453	0.29898	0	0	1	6
GO:0071830 triglyceride-rich lipoprotein particle clearance	1	0.10453	0.31	0.167	Biological	GO:00718	triglyceride-rich lipoprotein particle clearance	0.10453	0.29898	0	0	1	6
GO:0072008 glomerular mesangial cell differentiation	1	0.10453	0.31	0.167	Biological	GO:00720	glomerular mesangial cell differentiation	0.10453	0.29898	0	0	1	6
GO:0072143 mesangial cell development	1	0.10453	0.31	0.167	Biological	GO:0072	mesangial cell development	0.10453	0.29898	0	0	1	6
GO:0072174 metanephric tubule formation	1	0.10453	0.31	0.167	Biological	GO:0072	metanephric tubule formation	0.10453	0.29898	0	0	1	6
GO:0086073 bundle of His cell-Purkinje myocyte adhesion involved in c	1	0.10453	0.31	0.167	Biological	GO:00860	bundle of His cell-Purkinje myocyte adhesion involved in cell commu	0.10453	0.29898	0	0	1	6
GO:0090674 endothelial cell-matrix adhesion via fibronectin	1	0.10453	0.31	0.167	Biological	GO:00906	endothelial cell-matrix adhesion via fibronectin	0.10453	0.29898	0	0	1	6
GO:0097402 neuroblast migration	1	0.10453	0.31	0.167	Biological	GO:00974	neuroblast migration	0.10453	0.29898	0	0	1	6
GO:0098597 observational learning	1	0.10453	0.31	0.167	Biological	GO:00985	observational learning	0.10453	0.29898	0	0	1	6
GO:0120041 positive regulation of macrophage proliferation	1	0.10453	0.31	0.167	Biological	GO:01200	positive regulation of macrophage proliferation	0.10453	0.29898	0	0	1	6
GO:0140009 L-aspartate import across plasma membrane	1	0.10453	0.31	0.167	Biological	GO:01400	L-aspartate import across plasma membrane	0.10453	0.29898	0	0	1	6
GO:1901164 negative regulation of trophoblast cell migration	1	0.10453	0.31	0.167	Biological	GO:1901	negative regulation of trophoblast cell migration	0.10453	0.29898	0	0	1	6
GO:1901534 positive regulation of hematopoietic progenitor cell differen	1	0.10453	0.31	0.167	Biological	GO:19015	positive regulation of hematopoietic progenitor cell differentiation	0.10453	0.29898	0	0	1	6
GO:1901873 regulation of post-translational protein modification	1	0.10453	0.31	0.167	Biological	GO:19018	regulation of post-translational protein modification	0.10453	0.29898	0	0	1	6
GO:1902083 negative regulation of peptidyl-cysteine S-nitrosylation	1	0.10453	0.31	0.167	Biological	GO:19020	negative regulation of peptidyl-cysteine S-nitrosylation	0.10453	0.29898	0	0	1	6
GO:1902474 positive regulation of protein localization to synapse	1	0.10453	0.31	0.167	Biological	GO:19024	positive regulation of protein localization to synapse	0.10453	0.29898	0	0	1	6
GO:1902949 positive regulation of tau-protein kinase activity	1	0.10453	0.31	0.167	Biological	GO:19029	positive regulation of tau-protein kinase activity	0.10453	0.29898	0	0	1	6
GO:1903537 meiotic cell cycle process involved in oocyte maturation	1	0.10453	0.31	0.167	Biological	GO:19035	meiotic cell cycle process involved in oocyte maturation	0.10453	0.29898	0	0	1	6
GO:1903575 cornified envelope assembly	1	0.10453	0.31	0.167	Biological	GO:19035	cornified envelope assembly	0.10453	0.29898	0	0	1	6
GO:1903969 regulation of response to macrophage colony-stimulating f	1	0.10453	0.31	0.167	Biological	GO:19039	regulation of response to macrophage colony-stimulating factor	0.10453	0.29898	0	0	1	6
GO:1903972 regulation of cellular response to macrophage colony-stimu	1	0.10453	0.31	0.167	Biological	GO:19039	regulation of cellular response to macrophage colony-stimulating fact	0.10453	0.29898	0	0	1	6
GO:1904798 positive regulation of core promoter binding	1	0.10453	0.31	0.167	Biological	GO:1904	positive regulation of core promoter binding	0.10453	0.29898	0	0	1	6
GO:1904904 regulation of endothelial cell-matrix adhesion via fibronecti	1	0.10453	0.31	0.167	Biological	GO:19049	regulation of endothelial cell-matrix adhesion via fibronectin	0.10453	0.29898	0	0	1	6
GO:1905005 regulation of epithelial to mesenchymal transition involved	1	0.10453	0.31	0.167	Biological	GO:19050	regulation of epithelial to mesenchymal transition involved in endocar	0.10453	0.29898	0	0	1	6
GO:1905446 regulation of mitochondrial ATP synthesis coupled electror	1	0.10453	0.31	0.167	Biological	GO:19054	regulation of mitochondrial ATP synthesis coupled electron transport	0.10453	0.29898	0	0	1	6
GO:1990009 retinal cell apoptotic process	1	0.10453	0.31	0.167	Biological	GO:19900	retinal cell apoptotic process	0.10453	0.29898	0	0	1	6
GO:1990379 lipid transport across blood-brain barrier	1	0.10453	0.31	0.167	Biological	GO:1990	lipid transport across blood-brain barrier	0.10453	0.29898	0	0	1	6
GO:2000416 regulation of eosinophil migration	1	0.10453	0.31	0.167	Biological	GO:20004	regulation of eosinophil migration	0.10453	0.29898	0	0	1	6
GO:0032421 stereocilium bundle	3	0.1049	0.89	0.048	Cellular C	GO:00324	stereocilium bundle	0.1049	0.30174	0	0	3	63
GO:1904115 axon cytoplasm	3	0.1049	0.89	0.048	Cellular C	GO:1904	axon cytoplasm	0.1049	0.30174	0	0	3	63
GO:0060548 negative regulation of cell death	24	0.10554	7.43	0.024	Biological	GO:00605	negative regulation of cell death	0.10554	0.30169	0	0	24	1003
GO:0033218 amide binding	11	0.10554	3.216	0.028	Molecular	GO:00332	amide binding	0.10554	0.29666	0	0	11	386
GO:0004181 metallocarboxypeptidase activity	2	0.10618	0.585	0.067	Molecular	GO:0004	metallocarboxypeptidase activity	0.10618	0.29666	0	0	2	30
GO:0017147 Wnt-protein binding	2	0.10618	0.585	0.067	Molecular	GO:0017	Wnt-protein binding	0.10618	0.29666	0	0	2	30
GO:0009100 glycoprotein metabolic process	11	0.10619	3.406	0.028	Biological	GO:0009	glycoprotein metabolic process	0.10619	0.30339	0	0	11	393
GO:0003828 alpha-N-acetylneuraminate alpha-2,8-sialyltransferase acti	1	0.10619	0.292	0.167	Molecular	GO:00038	alpha-N-acetylneuraminate alpha-2,8-sialyltransferase activity	0.10619	0.29666	0	0	1	6
GO:0010385 double-stranded methylated DNA binding	1	0.10619	0.292	0.167	Molecular	GO:00103	double-stranded methylated DNA binding	0.10619	0.29666	0	0	1	6
GO:0017083 4-galactosyl-N-acetylglucosaminide 3-alpha-L-fucosyltrans	1	0.10619	0.292	0.167	Molecular	GO:00170	4-galactosyl-N-acetylglucosaminide 3-alpha-L-fucosyltransferase act	0.10619	0.29666	0	0	1	6
GO:0019705 protein-cysteine S-myristoyltransferase activity	1	0.10619	0.292	0.167	Molecular	GO:00197	protein-cysteine S-myristoyltransferase activity	0.10619	0.29666	0	0	1	6
GO:0023029 MHC class Ib protein binding	1	0.10619	0.292	0.167	Molecular	GO:00230	MHC class Ib protein binding	0.10619	0.29666	0	0	1	6
GO:0030274 LIM domain binding	1	0.10619	0.292	0.167	Molecular	GO:00302	LIM domain binding	0.10619	0.29666	0	0	1	6
GO:0031702 type 1 angiotensin receptor binding	1	0.10619	0.292	0.167	Molecular	GO:00317	type 1 angiotensin receptor binding	0.10619	0.29666	0	0	1	6
GO:0035671 enone reductase activity	1	0.10619	0.292	0.167	Molecular	GO:00356	enone reductase activity	0.10619	0.29666	0	0	1	6
GO:0046978 TAP1 binding	1	0.10619	0.292	0.167	Molecular	GO:00469	TAP1 binding	0.10619	0.29666	0	0	1	6
GO:0047024 5alpha-androstane-3beta,17beta-diol dehydrogenase activ	1	0.10619	0.292	0.167	Molecular	GO:00470	5alpha-androstane-3beta,17beta-diol dehydrogenase activity	0.10619	0.29666	0	0	1	6
GO:0048406 nerve growth factor binding	1	0.10619	0.292	0.167	Molecular	GO:00484	nerve growth factor binding	0.10619	0.29666	0	0	1	6
GO:0060228 phosphatidylcholine-sterol O-acyltransferase activator acti	1	0.10619	0.292	0.167	Molecular	GO:00602	phosphatidylcholine-sterol O-acyltransferase activator activity	0.10619	0.29666	0	0	1	6
GO:0070324 thyroid hormone binding	1	0.10619	0.292	0.167	Molecular	GO:00703	thyroid hormone binding	0.10619	0.29666	0	0	1	6
GO:0072572 poly-ADP-D-ribose binding	1	0.10619	0.292	0.167	Molecular	GO:00725	poly-ADP-D-ribose binding	0.10619	0.29666	0	0	1	6
GO:0006796 phosphate-containing compound metabolic process	59	0.1073	18.266	0.021	Biological	GO:00067	phosphate-containing compound metabolic process	0.1073	0.30638	0	0	59	2766
GO:0099536 synaptic signaling	19	0.10746	5.882	0.025	Biological	GO:00995	synaptic signaling	0.10746	0.30643	0	0	19	765
GO:0009994 oocyte differentiation	3	0.10756	0.929	0.048	Biological	GO:00099	oocyte differentiation	0.10756	0.30643	0	0	3	63
GO:0032481 positive regulation of type I interferon production	3	0.10756	0.929	0.048	Biological	GO:00324	positive regulation of type I interferon production	0.10756	0.30643	0	0	3	63
GO:0043090 amino acid import	3	0.10756	0.929	0.048	Biological	GO:00430	amino acid import	0.10756	0.30643	0	0	3	63
GO:0005615 extracellular space	70	0.10761	20.772	0.021	Cellular C	GO:00056	extracellular space	0.10761	0.30788	0	0	70	3372
GO:1990138 neuron projection extension	6	0.10766	1.858	0.034	Biological	GO:1990	neuron projection extension	0.10766	0.30653	0	0	6	178
GO:0009894 regulation of catabolic process	24	0.10809	7.43	0.024	Biological	GO:00098	regulation of catabolic process	0.10809	0.3075	0	0	24	1006
GO:0009890 negative regulation of biosynthetic process	37	0.10813	11.455	0.022	Biological	GO:00098	negative regulation of biosynthetic process	0.10813	0.3075	0	0	37	1648
GO:0008277 regulation of G protein-coupled receptor signaling pathway	5	0.1083	1.548	0.036	Biological	GO:00082	regulation of G protein-coupled receptor signaling pathway	0.1083	0.30781	0	0	5	138
GO:0010605 negative regulation of macromolecule metabolic process	59	0.10837	18.266	0.021	Biological	GO:00106	negative regulation of macromolecule metabolic process	0.10837	0.30785	0	0	59	2768
GO:0071407 cellular response to organic cyclic compound	15	0.10878	4.644	0.026	Biological	GO:00714	cellular response to organic cyclic compound	0.10878	0.30789	0	0	15	578
GO:0001556 oocyte maturation	2	0.10912	0.619	0.065	Biological	GO:00015	oocyte maturation	0.10912	0.30789	0	0	2	31
GO:0001945 lymph vessel development	2	0.10912	0.619	0.065	Biological	GO:00019	lymph vessel development	0.10912	0.30789	0	0	2	31
GO:0006359 regulation of transcription by RNA polymerase III	2	0.10912	0.619	0.065	Biological	GO:00063	regulation of transcription by RNA polymerase III	0.10912	0.30789	0	0	2	31
GO:0007350 blastoderm segmentation	2	0.10912	0.619	0.065	Biological	GO:00073	blastoderm segmentation	0.10912	0.30789	0	0	2	31
GO:0008045 motor neuron axon guidance	2	0.10912	0.619	0.065	Biological	GO:00080	motor neuron axon guidance	0.10912	0.30789	0	0	2	31
GO:0008299 isoprenoid biosynthetic process	2	0.10912	0.619	0.065	Biological	GO:00082	isoprenoid biosynthetic process	0.10912	0.30789	0	0	2	31
GO:0031069 hair follicle morphogenesis	2	0.10912	0.619	0.065	Biological	GO:00310	hair follicle morphogenesis	0.10912	0.30789	0	0	2	31
GO:0032770 positive regulation of monooxygenase activity	2	0.10912	0.619	0.065	Biological	GO:00327	positive regulation of monooxygenase activity	0.10912	0.30789	0	0	2	31
GO:0034311 diol metabolic process	2	0.10912	0.619	0.065	Biological	GO:00343	diol metabolic process	0.10912	0.30789	0	0	2	31
GO:0043949 regulation of cAMP-mediated signaling	2	0.10912	0.619	0.065	Biological	GO:00439	regulation of cAMP-mediated signaling	0.10912	0.30789	0	0	2	31
GO:0095500 acetylcholine receptor signaling pathway	2	0.10912	0.619	0.065	Biological	GO:00955	acetylcholine receptor signaling pathway	0.10912	0.30789	0	0	2	31
GO:0001659 temperature homeostasis	6	0.1098	1.858	0.034	Biological	GO:00016	temperature homeostasis	0.1098	0.30961	0	0	6	179
GO:0003073 regulation of systemic arterial blood pressure	4	0.1101	1.238	0.04	Biological	GO:00030	regulation of systemic arterial blood pressure	0.1101	0.31013	0	0	4	100
GO:0021510 spinal cord development	4	0.1101	1.238	0.04	Biological	GO:00215	spinal cord development	0.1101	0.31013	0	0	4	100
GO:0007601 visual perception	7	0.11046	2.167	0.032	Biological	GO:00076	visual perception	0.11046	0.31096	0	0	7	221
GO:1903052 positive regulation of proteolysis involved in cellular protein	5	0.11077	1.548	0.036	Biological	GO:19030	positive regulation of proteolysis involved in cellular protein catabolic	0.11077	0.31164	0	0	5	139
GO:0033059 cellular pigmentation	3	0.11141	0.929	0.047	Biological	GO:00330	cellular pigmentation	0.11141	0.31311	0	0	3	64
GO:0046579 positive regulation of Ras protein signal transduction	3	0.11141	0.929	0.047	Biological	GO:00465	positive regulation of Ras protein signal transduction	0.11141	0.31311	0	0	3	64
GO:0015179 L-amino acid transmembrane transporter activity	3	0.11161	0.877	0.048	Molecular	GO:00151	L-amino acid transmembrane transporter activity	0.11161	0.31077	0	0	3	63
GO:0001726 ruffle	6	0.11192	1.78	0.033	Cellular C	GO:0001	ruffle	0.11192	0.31736	0	0	6	182
GO:0004033 aldo-keto reductase (NADP) activity	2	0.11217	0.585	0.065	Molecular	GO:00040	aldo-keto reductase (NADP) activity	0.11217	0.31135	0	0	2	31
GO:0005637 nuclear inner membrane	3	0.11249	0.89	0.046	Cellular C	GO:00056	nuclear inner membrane	0.11249	0.31736	0	0	3	65

GO:0031965 nuclear membrane	9	0.11271	2.671	0.029	Cellular C	GO:00319	nuclear membrane	0.11271	0.31736	0	0	9	312
GO:0051090 regulation of DNA-binding transcription factor activity	12	0.11272	3.715	0.027	Biological	GO:00510	regulation of DNA-binding transcription factor activity	0.11272	0.31661	0	0	12	443
GO:0032760 positive regulation of tumor necrosis factor production	4	0.1131	1.238	0.04	Biological	GO:00327	positive regulation of tumor necrosis factor production	0.1131	0.31731	0	0	4	101
GO:0048709 oligodendrocyte differentiation	4	0.1131	1.238	0.04	Biological	GO:00487	oligodendrocyte differentiation	0.1131	0.31731	0	0	4	101
GO:0006812 cation transport	29	0.1132	8.978	0.023	Biological	GO:00068	cation transport	0.1132	0.31741	0	0	29	1257
GO:0042157 lipoprotein metabolic process	5	0.11326	1.548	0.036	Biological	GO:00421	lipoprotein metabolic process	0.11326	0.31741	0	0	5	140
GO:0045177 apical part of cell	12	0.11415	3.561	0.027	Cellular C	GO:00451	apical part of cell	0.11415	0.31974	0	0	12	449
GO:0030522 intracellular receptor signaling pathway	8	0.11455	2.477	0.03	Biological	GO:00305	intracellular receptor signaling pathway	0.11455	0.3194	0	0	8	266
GO:0010574 regulation of vascular endothelial growth factor production	2	0.11506	0.619	0.062	Biological	GO:00105	regulation of vascular endothelial growth factor production	0.11506	0.3194	0	0	2	32
GO:0048384 retinoic acid receptor signaling pathway	2	0.11506	0.619	0.062	Biological	GO:00483	retinoic acid receptor signaling pathway	0.11506	0.3194	0	0	2	32
GO:0048873 homeostasis of number of cells within a tissue	2	0.11506	0.619	0.062	Biological	GO:00488	homeostasis of number of cells within a tissue	0.11506	0.3194	0	0	2	32
GO:0060292 long-term synaptic depression	2	0.11506	0.619	0.062	Biological	GO:00602	long-term synaptic depression	0.11506	0.3194	0	0	2	32
GO:0060325 face morphogenesis	2	0.11506	0.619	0.062	Biological	GO:00603	face morphogenesis	0.11506	0.3194	0	0	2	32
GO:0010720 positive regulation of cell development	9	0.11507	2.786	0.029	Biological	GO:00107	positive regulation of cell development	0.11507	0.3194	0	0	9	310
GO:0098930 axonal transport	3	0.11531	0.929	0.046	Biological	GO:00989	axonal transport	0.11531	0.3194	0	0	3	65
GO:1905330 regulation of morphogenesis of an epithelium	3	0.11531	0.929	0.046	Biological	GO:19053	regulation of morphogenesis of an epithelium	0.11531	0.3194	0	0	3	65
GO:0032101 regulation of response to external stimulus	23	0.11569	7.121	0.024	Biological	GO:00321	regulation of response to external stimulus	0.11569	0.3194	0	0	23	966
GO:0005811 lipid droplet	4	0.11569	1.187	0.039	Cellular C	GO:00058	lipid droplet	0.11569	0.32238	0	0	4	103
GO:0003333 amino acid transmembrane transport	4	0.11613	1.238	0.039	Biological	GO:00033	amino acid transmembrane transport	0.11613	0.3194	0	0	4	102
GO:0032479 regulation of type I interferon production	4	0.11613	1.238	0.039	Biological	GO:00324	regulation of type I interferon production	0.11613	0.3194	0	0	4	102
GO:0032606 type I interferon production	4	0.11613	1.238	0.039	Biological	GO:00326	type I interferon production	0.11613	0.3194	0	0	4	102
GO:0099175 regulation of postsynapse organization	4	0.11613	1.238	0.039	Biological	GO:00991	regulation of postsynapse organization	0.11613	0.3194	0	0	4	102
GO:0050953 sensory perception of light stimulus	7	0.1163	2.167	0.031	Biological	GO:00509	sensory perception of light stimulus	0.1163	0.3194	0	0	7	224
GO:0046873 metal ion transmembrane transporter activity	12	0.11738	3.509	0.027	Molecular	GO:00468	metal ion transmembrane transporter activity	0.11738	0.32217	0	0	12	439
GO:0008238 exopeptidase activity	4	0.11823	1.17	0.04	Molecular	GO:00082	exopeptidase activity	0.11823	0.32217	0	0	4	101
GO:0033764 steroid dehydrogenase activity, acting on the CH-OH grou	2	0.11825	0.585	0.062	Molecular	GO:00337	steroid dehydrogenase activity, acting on the CH-OH group of donors	0.11825	0.32217	0	0	2	32
GO:0097191 extrinsic apoptotic signaling pathway	7	0.11829	2.167	0.031	Biological	GO:00971	extrinsic apoptotic signaling pathway	0.11829	0.3194	0	0	7	225
GO:0033135 regulation of peptidyl-serine phosphorylation	5	0.11834	1.548	0.035	Biological	GO:00331	regulation of peptidyl-serine phosphorylation	0.11834	0.3194	0	0	5	142
GO:0035270 endocrine system development	5	0.11834	1.548	0.035	Biological	GO:00352	endocrine system development	0.11834	0.3194	0	0	5	142
GO:0007010 cytoskeleton organization	34	0.11896	10.526	0.022	Biological	GO:00070	cytoskeleton organization	0.11896	0.3194	0	0	34	1513
GO:0030900 forebrain development	11	0.11898	3.406	0.027	Biological	GO:00309	forebrain development	0.11898	0.3194	0	0	11	402
GO:0016525 negative regulation of angiogenesis	4	0.11919	1.238	0.039	Biological	GO:00165	negative regulation of angiogenesis	0.11919	0.3194	0	0	4	103
GO:0032963 collagen metabolic process	4	0.11919	1.238	0.039	Biological	GO:00329	collagen metabolic process	0.11919	0.3194	0	0	4	103
GO:0150115 cell-substrate junction organization	4	0.11919	1.238	0.039	Biological	GO:01501	cell-substrate junction organization	0.11919	0.3194	0	0	4	103
GO:0009311 oligosaccharide metabolic process	3	0.11927	0.929	0.045	Biological	GO:00093	oligosaccharide metabolic process	0.11927	0.3194	0	0	3	66
GO:0044458 motile cilium assembly	3	0.11927	0.929	0.045	Biological	GO:00444	motile cilium assembly	0.11927	0.3194	0	0	3	66
GO:0071806 protein transmembrane transport	3	0.11927	0.929	0.045	Biological	GO:00718	protein transmembrane transport	0.11927	0.3194	0	0	3	66
GO:0035686 sperm fibrous sheath	1	0.11957	0.297	0.143	Cellular C	GO:00356	sperm fibrous sheath	0.11957	0.32473	0	0	1	7
GO:0046581 intercellular canaliculus	1	0.11957	0.297	0.143	Cellular C	GO:00465	intercellular canaliculus	0.11957	0.32473	0	0	1	7
GO:0097512 cardiac myofibril	1	0.11957	0.297	0.143	Cellular C	GO:00975	cardiac myofibril	0.11957	0.32473	0	0	1	7
GO:0150053 cerebellar climbing fiber to Purkinje cell synapse	1	0.11957	0.297	0.143	Cellular C	GO:01500	cerebellar climbing fiber to Purkinje cell synapse	0.11957	0.32473	0	0	1	7
GO:1990635 proximal dendrite	1	0.11957	0.297	0.143	Cellular C	GO:19906	proximal dendrite	0.11957	0.32473	0	0	1	7
GO:1903039 positive regulation of leukocyte cell-cell adhesion	8	0.11996	2.477	0.03	Biological	GO:19030	positive regulation of leukocyte cell-cell adhesion	0.11996	0.3194	0	0	8	269
GO:0002684 positive regulation of immune system process	23	0.12034	7.121	0.024	Biological	GO:00026	positive regulation of immune system process	0.12034	0.3194	0	0	23	971
GO:0046983 protein dimerization activity	26	0.12036	7.602	0.024	Molecular	GO:00469	protein dimerization activity	0.12036	0.32217	0	0	26	1099
GO:0043405 regulation of MAP kinase activity	6	0.1208	1.858	0.033	Biological	GO:00434	regulation of MAP kinase activity	0.1208	0.3194	0	0	6	184
GO:0002154 thyroid hormone mediated signaling pathway	1	0.12086	0.31	0.143	Biological	GO:00021	thyroid hormone mediated signaling pathway	0.12086	0.3194	0	0	1	7
GO:0003339 regulation of mesenchymal to epithelial transition involved i	1	0.12086	0.31	0.143	Biological	GO:00033	regulation of mesenchymal to epithelial transition involved in metanep	0.12086	0.3194	0	0	1	7
GO:0006166 purine ribonucleoside salvage	1	0.12086	0.31	0.143	Biological	GO:00061	purine ribonucleoside salvage	0.12086	0.3194	0	0	1	7
GO:0006701 progesterone biosynthetic process	1	0.12086	0.31	0.143	Biological	GO:00067	progesterone biosynthetic process	0.12086	0.3194	0	0	1	7
GO:0009624 response to nematode	1	0.12086	0.31	0.143	Biological	GO:00096	response to nematode	0.12086	0.3194	0	0	1	7
GO:0010536 positive regulation of activation of Janus kinase activity	1	0.12086	0.31	0.143	Biological	GO:00105	positive regulation of activation of Janus kinase activity	0.12086	0.3194	0	0	1	7
GO:0010886 positive regulation of cholesterol storage	1	0.12086	0.31	0.143	Biological	GO:00108	positive regulation of cholesterol storage	0.12086	0.3194	0	0	1	7
GO:0021546 rhombomere development	1	0.12086	0.31	0.143	Biological	GO:00215	rhombomere development	0.12086	0.3194	0	0	1	7
GO:0021798 forebrain dorsal/ventral pattern formation	1	0.12086	0.31	0.143	Biological	GO:00217	forebrain dorsal/ventral pattern formation	0.12086	0.3194	0	0	1	7
GO:0021877 forebrain neuron fate commitment	1	0.12086	0.31	0.143	Biological	GO:00218	forebrain neuron fate commitment	0.12086	0.3194	0	0	1	7
GO:0030046 parallel actin filament bundle assembly	1	0.12086	0.31	0.143	Biological	GO:00300	parallel actin filament bundle assembly	0.12086	0.3194	0	0	1	7
GO:0030091 protein repair	1	0.12086	0.31	0.143	Biological	GO:00300	protein repair	0.12086	0.3194	0	0	1	7
GO:0030917 midbrain-hindbrain boundary development	1	0.12086	0.31	0.143	Biological	GO:00309	midbrain-hindbrain boundary development	0.12086	0.3194	0	0	1	7
GO:0032278 positive regulation of gonadotropin secretion	1	0.12086	0.31	0.143	Biological	GO:00322	positive regulation of gonadotropin secretion	0.12086	0.3194	0	0	1	7
GO:0032415 regulation of sodium:proton antiporter activity	1	0.12086	0.31	0.143	Biological	GO:00324	regulation of sodium:proton antiporter activity	0.12086	0.3194	0	0	1	7
GO:0032594 protein transport within lipid bilayer	1	0.12086	0.31	0.143	Biological	GO:00325	protein transport within lipid bilayer	0.12086	0.3194	0	0	1	7
GO:0032741 positive regulation of interleukin-18 production	1	0.12086	0.31	0.143	Biological	GO:00327	positive regulation of interleukin-18 production	0.12086	0.3194	0	0	1	7
GO:0038145 macrophage colony-stimulating factor signaling pathway	1	0.12086	0.31	0.143	Biological	GO:00381	macrophage colony-stimulating factor signaling pathway	0.12086	0.3194	0	0	1	7
GO:0042723 thiamine-containing compound metabolic process	1	0.12086	0.31	0.143	Biological	GO:00427	thiamine-containing compound metabolic process	0.12086	0.3194	0	0	1	7
GO:0042748 circadian sleep/wake cycle, non-REM sleep	1	0.12086	0.31	0.143	Biological	GO:00427	circadian sleep/wake cycle, non-REM sleep	0.12086	0.3194	0	0	1	7
GO:0042940 D-amino acid transport	1	0.12086	0.31	0.143	Biological	GO:00429	D-amino acid transport	0.12086	0.3194	0	0	1	7
GO:0043569 negative regulation of insulin-like growth factor receptor sig	1	0.12086	0.31	0.143	Biological	GO:00435	negative regulation of insulin-like growth factor receptor signaling pat	0.12086	0.3194	0	0	1	7
GO:0043619 regulation of transcription from RNA polymerase II promot	1	0.12086	0.31	0.143	Biological	GO:00436	regulation of transcription from RNA polymerase II promoter in respo	0.12086	0.3194	0	0	1	7
GO:0044691 tooth eruption	1	0.12086	0.31	0.143	Biological	GO:00446	tooth eruption	0.12086	0.3194	0	0	1	7
GO:0045924 regulation of female receptivity	1	0.12086	0.31	0.143	Biological	GO:00459	regulation of female receptivity	0.12086	0.3194	0	0	1	7
GO:0046137 negative regulation of vitamin metabolic process	1	0.12086	0.31	0.143	Biological	GO:00461	negative regulation of vitamin metabolic process	0.12086	0.3194	0	0	1	7
GO:0046600 negative regulation of centriole replication	1	0.12086	0.31	0.143	Biological	GO:00466	negative regulation of centriole replication	0.12086	0.3194	0	0	1	7
GO:0046813 receptor-mediated virion attachment to host cell	1	0.12086	0.31	0.143	Biological	GO:00468	receptor-mediated virion attachment to host cell	0.12086	0.3194	0	0	1	7
GO:0048312 intracellular distribution of mitochondria	1	0.12086	0.31	0.143	Biological	GO:00483	intracellular distribution of mitochondria	0.12086	0.3194	0	0	1	7
GO:0050882 voluntary musculoskeletal movement	1	0.12086	0.31	0.143	Biological	GO:00508	voluntary musculoskeletal movement	0.12086	0.3194	0	0	1	7
GO:0050916 sensory perception of sweet taste	1	0.12086	0.31	0.143	Biological	GO:00509	sensory perception of sweet taste	0.12086	0.3194	0	0	1	7
GO:0050942 positive regulation of pigment cell differentiation	1	0.12086	0.31	0.143	Biological	GO:00509	positive regulation of pigment cell differentiation	0.12086	0.3194	0	0	1	7
GO:0060087 relaxation of vascular associated smooth muscle	1	0.12086	0.31	0.143	Biological	GO:00600	relaxation of vascular associated smooth muscle	0.12086	0.3194	0	0	1	7
GO:0060180 female mating behavior	1	0.12086	0.31	0.143	Biological	GO:00601	female mating behavior	0.12086	0.3194	0	0	1	7
GO:0060297 regulation of sarcomere organization	1	0.12086	0.31	0.143	Biological	GO:00602	regulation of sarcomere organization	0.12086	0.3194	0	0	1	7
GO:0060405 regulation of penile erection	1	0.12086	0.31	0.143	Biological	GO:00604	regulation of penile erection	0.12086	0.3194	0	0	1	7
GO:0060534 trachea cartilage development	1	0.12086	0.31	0.143	Biological	GO:00605	trachea cartilage development	0.12086	0.3194	0	0	1	7
GO:0060676 ureteric bud formation	1	0.12086	0.31	0.143	Biological	GO:00606	ureteric bud formation	0.12086	0.3194	0	0	1	7
GO:0060729 intestinal epithelial structure maintenance	1	0.12086	0.31	0.143	Biological	GO:00607	intestinal epithelial structure maintenance	0.12086	0.3194	0	0	1	7
GO:0060745 mammary gland branching involved in pregnancy	1	0.12086	0.31	0.143	Biological	GO:00607	mammary gland branching involved in pregnancy	0.12086	0.3194	0	0	1	7
GO:0061370 testosterone biosynthetic process	1	0.12086	0.31	0.143	Biological	GO:00613	testosterone biosynthetic process	0.12086	0.3194	0	0	1	7
GO:0070106 interleukin-27-mediated signaling pathway	1	0.12086	0.31	0.143	Biological	GO:00701	interleukin-27-mediated signaling pathway	0.12086	0.3194	0	0	1	7
GO:0070671 response to interleukin-12	1	0.12086	0.31	0.143	Biological	GO:00706	response to interleukin-12	0.12086	0.3194	0	0	1	7
GO:0070933 histone H4 deacetylation	1	0.12086	0.31	0.143	Biological	GO:00709	histone H4 deacetylation	0.12086	0.3194	0	0	1	7
GO:0071224 cellular response to peptidoglycan	1	0.12086	0.31	0.143	Biological	GO:00712	cellular response to peptidoglycan	0.12086	0.3194	0	0	1	7
GO:0071321 cellular response to cGMP	1	0.12086	0.31	0.143	Biological	GO:00713	cellular response to cGMP	0.12086	0.3194	0	0	1	7
GO:0071651 positive regulation of chemokine (C-C motif) ligand 5 produ	1	0.12086	0.31	0.143	Biological	GO:00716	positive regulation of chemokine (C-C motif) ligand 5 production	0.12086	0.3194	0	0	1	7
GO:0071694 maintenance of protein location in extracellular region	1	0.12086	0.31	0.143	Biological	GO:00716	maintenance of protein location in extracellular region	0.12086	0.3194	0	0	1	7
GO:0071934 thiamine transmembrane transport	1	0.12086	0.31	0.143	Biological	GO:00719	thiamine transmembrane transport	0.12086	0.3194	0	0	1	7
GO:0072007 mesangial cell differentiation	1	0.12086	0.31	0.143	Biological	GO:00720	mesangial cell differentiation	0.12086	0.3194	0	0	1	7
GO:0072197 ureter morphogenesis	1	0.12086	0.31	0.143	Biological	GO:00721	ureter morphogenesis	0.12086	0.3194	0	0	1	7
GO:0072257 metanephric nephron tubule epithelial cell differentiation	1	0.12086	0.31	0.143	Biological	GO:00722	metanephric nephron tubule epithelial cell differentiation	0.12086	0.3194	0	0	1	7
GO:0072307 regulation of metanephric nephron tubule epithelial cell diff	1	0.12086	0.31	0.143	Biological	GO:00723	regulation of metanephric nephron tubule epithelial cell differentiation	0.12086	0.3194	0	0	1	7
GO:0072386 plus-end-directed organelle transport along microtubule	1	0.12086	0.31	0.143	Biological	GO:00723	plus-end-directed organelle transport along microtubule	0.12086	0.3194	0	0	1	7
GO:0086042 cardiac muscle cell-cardiac muscle cell adhesion	1	0.12086	0.31	0.143	Biological	GO:00860	cardiac muscle cell-cardiac muscle cell adhesion	0.12086	0.3194	0	0	1	7
GO:0090673 endothelial cell-matrix adhesion	1	0.12086	0.31	0.143	Biological	GO:00906	endothelial cell-matrix adhesion	0.12086	0.3194	0	0	1	7
GO:0097065 anterior head development	1	0.12086	0.31	0.143	Biological	GO:00970	anterior head development	0.12086	0.3194	0	0	1	7
GO:0097187 dentinogenesis	1	0.12086	0.31	0.143	Biological	GO:00971	dentinogenesis	0.12086	0.3194	0	0	1	7
GO:0098838 folate transmembrane transport	1	0.12086	0.31	0.143	Biological	GO:00988	folate transmembrane transport	0.12086	0.3194	0	0	1	7
GO:0106104 regulation of glutamate receptor clustering	1	0.12086	0.31	0.143	Biological	GO:01061	regulation of glutamate receptor clustering	0.12086	0.3194	0	0	1	7
GO:0120040 regulation of macrophage proliferation	1	0.12086	0.31	0.143	Biological	GO:01200	regulation of macrophage proliferation	0.12086	0.3194	0	0	1	7
GO:0140331 aminophospholipid translocation	1	0.12086	0.31	0.143	Biological	GO:01403	aminophospholipid translocation	0.12086	0.3194	0	0	1	7
GO:1903598 positive regulation of gap junction assembly	1	0.12086	0.31	0.143	Biological	GO:19035	positive regulation of gap junction assembly	0.12086	0.3194	0	0	1	7
GO:1903911 positive regulation of receptor clustering	1	0.12086	0.31	0.143	Biological	GO:19039	positive regulation of receptor clustering	0.12086	0.3194	0	0	1	7
GO:1904141 positive regulation of microglial cell migration	1	0.12086	0.31	0.143	Biological	GO:19041	positive regulation of microglial cell migration	0.12086	0.3194	0	0	1	7
GO:1904717 regulation of AMPA glutamate receptor clustering	1	0.12086	0.31	0.143	Biological	GO:19047	regulation of AMPA glutamate receptor clustering	0.12086	0.3194	0	0	1	7
GO:1904796 regulation of core promoter binding	1	0.12086	0.31	0.143	Biological	GO:19047	regulation of core promoter binding	0.12086	0.3194	0	0	1	7
GO:2000138 positive regulation of cell proliferation involved in heart mor	1	0.12086	0.31	0.143	Biological	GO:20001	positive regulation of cell proliferation involved in heart morphogenesi	0.12086	0.3194	0	0	1	7
GO:2000489 regulation of hepatic stellate cell activation	1	0.12086	0.31	0.143	Biological	GO:20004	regulation of hepatic stellate cell activation	0.12086	0.3194	0	0	1	7
GO:2000646 positive regulation of receptor catabolic process	1	0.12086	0.31	0.143	Biological	GO:20006	positive regulation of receptor catabolic process	0.12086	0.3194	0	0	1	7
GO:2000727 positive regulation of cardiac muscle cell differentiation	1	0.12086	0.31	0.143	Biological	GO:20007	positive regulation of cardiac muscle cell differentiation	0.12086	0.3194	0	0	1	7
GO:2000794 regulation of epithelial cell proliferation involved in lung mor	1	0.12086	0.31	0.143	Biological	GO:20007	regulation of epithelial cell proliferation involved in lung morphogenesi	0.12086	0.3194	0	0	1	7
GO:0002230 positive regulation of defense response to virus by host	2	0.12107	0.619	0.061	Biological	GO:00022	positive regulation of defense response to virus by host	0.12107	0.3194	0	0	2	33
GO:0006084 acetyl-CoA metabolic process	2	0.12107	0.619	0.061	Biological	GO:00060	acetyl-CoA metabolic process	0.12107	0.3194	0	0	2	33
GO:0006654 phosphatidic acid biosynthetic process	2	0.12107	0.619	0.061	Biological	GO:00066	phosphatidic acid biosynthetic process	0.12107	0.3194	0	0	2	33
GO:0035456 response to interferon-beta	2	0.12107	0.619	0.061	Biological	GO:00354	response to interferon-beta	0.12107	0.3194	0	0	2	33
GO:0042044 fluid transport	2	0.12107	0.619	0.061	Biological	GO:00420	fluid transport	0.12107	0.3194	0	0	2	33
GO:0048566 embryonic digestive tract development	2	0.12107	0.619	0.061	Biological	GO:00485	embryonic digestive tract development	0.12107	0.3194	0	0	2	33
GO:0060603 mammary gland duct morphogenesis	2	0.12107	0.619	0.061	Biological	GO:00606	mammary gland duct morphogenesis	0.12107	0.3194	0	0	2	33
GO:0061384 heart trabecula morphogenesis	2	0.12107	0.619	0.061	Biological	GO:00613	heart trabecula morphogenesis	0.12107	0.3194	0	0	2	33
GO:0090022 regulation of neutrophil chemotaxis	2	0.12107	0.619	0.061	Biological	GO:00900	regulation of neutrophil chemotaxis	0.12107	0.3194	0	0	2	33
GO:1905145 cellular response to acetylcholine	2	0.12107	0.619	0.061	Biological	GO:19051	cellular response to acetylcholine	0.12107	0.3194	0	0	2	33
GO:0006793 phosphorus metabolic process	59	0.12119	18.266	0.021	Biological	GO:00067	phosphorus metabolic process	0.12119	0.31954	0	0	59	2791
GO:0070201 regulation of establishment of protein localization	14	0.12155	4.334	0.026	Biological	GO:00702	regulation of establishment of protein localization	0.12155	0.32032	0	0	14	542
GO:0019932 second-messenger-mediated signaling	9	0.12176	2.786	0.029	Biological	GO:00199	second-messenger-mediated signaling	0.12176	0.32071	0	0	9	314
GO:0005881 cytoplasmic microtubule	4	0.12178	1.187	0.038	Cellular C	GO:00058	cytoplasmic microtubule	0.12178	0.32905	0	0	4	105
GO:0030316 osteoclast differentiation	4	0.12229	1.238	0.038	Biological	GO:00303	osteoclast differentiation	0.12229	0.32161	0	0	4	104
GO:0045833 negative regulation of lipid metabolic process	4	0.12229	1.238	0.038	Biological	GO:00458	negative regulation of lipid metabolic process	0.12229	0.32161	0	0	4	104
GO:2000181 negative regulation of blood vessel morphogenesis	4	0.12229	1.238	0.038	Biological	GO:20001	negative regulation of blood vessel morphogenesis	0.12229	0.32161	0	0	4	104
GO:0032103 positive regulation of response to external stimulus	12	0.12236	3.715	0.027	Biological	GO:00321	positive regulation of response to external stimulus	0.12236	0.32162	0	0	12	450
GO:0004169 dolichyl-phosphate-mannose-protein mannosyltransferase	1	0.12277	0.292	0.143	Molecular	GO:00041	dolichyl-phosphate-mannose-protein mannosyltransferase activity	0.12277	0.32217	0	0	1	7
GO:0004571 mannosyl-oligosaccharide 1,2-alpha-mannosidase activity	1	0.12277	0.292	0.143	Molecular	GO:00045	mannosyl-oligosaccharide 1,2-alpha-mannosidase activity	0.12277	0.32217	0	0	1	7
GO:0005138 interleukin-6 receptor binding	1	0.12277	0.292	0.143	Molecular	GO:00051	interleukin-6 receptor binding	0.12277	0.32217	0	0	1	7
GO:0005295 neutral amino acid:sodium symporter activity	1	0.12277	0.292	0.143	Molecular	GO:00052	neutral amino acid:sodium symporter activity	0.12277	0.32217	0	0	1	7
GO:0015190 L-leucine transmembrane transporter activity	1	0.12277	0.292	0.143	Molecular	GO:00151	L-leucine transmembrane transporter activity	0.12277	0.32217	0	0	1	7
GO:0015220 choline transmembrane transporter activity	1	0.12277	0.292	0.143	Molecular	GO:00152	choline transmembrane transporter activity	0.12277	0.32217	0	0	1	7
GO:0015234 thiamine transmembrane transporter activity	1	0.12277	0.292	0.143	Molecular	GO:00152	thiamine transmembrane transporter activity	0.12277	0.32217	0	0	1	7
GO:0015247 aminophospholipid flippase activity	1	0.12277	0.292	0.143	Molecular	GO:00152	aminophospholipid flippase activity	0.12277	0.32217	0	0	1	7
GO:0015349 thyroid hormone transmembrane transporter activity	1	0.12277	0.292	0.143	Molecular	GO:00153	thyroid hormone transmembrane transporter activity	0.12277	0.32217	0	0	1	7
GO:0031685 adenosine receptor binding	1	0.12277	0.292	0.143	Molecular	GO:00316	adenosine receptor binding	0.12277	0.32217	0	0	1	7
GO:0031701 angiotensin receptor binding	1	0.12277	0.292	0.143	Molecular	GO:00317	angiotensin receptor binding	0.12277	0.32217	0	0	1	7
GO:0031730 CCR5 chemokine receptor binding	1	0.12277	0.292	0.143	Molecular	GO:00317	CCR5 chemokine receptor binding	0.12277	0.32217	0	0	1	7
GO:0106256 hydroperoxy icosatetraenoate dehydratase activity	1	0.12277	0.292	0.143	Molecular	GO:01062	hydroperoxy icosatetraenoate dehydratase activity	0.12277	0.32217	0	0	1	7
GO:0140439 protein-cysteine S-stearoyltransferase activity	1	0.12277	0.292	0.143	Molecular	GO:01404	protein-cysteine S-stearoyltransferase activity	0.12277	0.32217	0	0	1	7
GO:0051171 regulation of nitrogen compound metabolic process	113	0.12292	34.985	0.02	Biological	GO:00511	regulation of nitrogen compound metabolic process	0.12292	0.32293	0	0	113	5642
GO:0042552 myelination	5	0.12352	1.548	0.035	Biological	GO:00425	myelination	0.12352	0.32431	0	0	5	144

GO:0016627 oxidoreductase activity, acting on the CH-CH group of don	3	0.12367	0.877	0.045	Molecular	GO:0016	oxidoreductase activity, acting on the CH-CH group of donors	0.12367	0.32356	0	0	3	66
GO:0005343 organic acid:sodium symporter activity	2	0.1244	0.585	0.061	Molecular	GO:0005	organic acid:sodium symporter activity	0.1244	0.32379	0	0	2	33
GO:0070851 growth factor receptor binding	5	0.12451	1.462	0.035	Molecular	GO:0070	growth factor receptor binding	0.12451	0.32379	0	0	5	142
GO:0022411 cellular component disassembly	13	0.12488	4.025	0.026	Biological	GO:0022	cellular component disassembly	0.12488	0.32771	0	0	13	498
GO:0090066 regulation of anatomical structure size	14	0.12541	4.334	0.026	Biological	GO:0090	regulation of anatomical structure size	0.12541	0.32847	0	0	14	545
GO:0008585 female gonad development	4	0.12543	1.238	0.038	Biological	GO:0008	female gonad development	0.12543	0.32847	0	0	4	105
GO:1901343 negative regulation of vasculature development	4	0.12543	1.238	0.038	Biological	GO:1901	negative regulation of vasculature development	0.12543	0.32847	0	0	4	105
GO:1903557 positive regulation of tumor necrosis factor superfamily cyt	4	0.12543	1.238	0.038	Biological	GO:1903	positive regulation of tumor necrosis factor superfamily cytokine prod	0.12543	0.32847	0	0	4	105
GO:0044272 sulfur compound biosynthetic process	5	0.12615	1.548	0.034	Biological	GO:0044	sulfur compound biosynthetic process	0.12615	0.33017	0	0	5	145
GO:0060326 cell chemotaxis	9	0.12691	2.786	0.028	Biological	GO:0060	cell chemotaxis	0.12691	0.33057	0	0	9	317
GO:1902105 regulation of leukocyte differentiation	9	0.12691	2.786	0.028	Biological	GO:1902	regulation of leukocyte differentiation	0.12691	0.33057	0	0	9	317
GO:0001569 branching involved in blood vessel morphogenesis	2	0.12716	0.619	0.059	Biological	GO:0001	branching involved in blood vessel morphogenesis	0.12716	0.33057	0	0	2	34
GO:0034405 response to fluid shear stress	2	0.12716	0.619	0.059	Biological	GO:0034	response to fluid shear stress	0.12716	0.33057	0	0	2	34
GO:0035590 purinergic nucleotide receptor signaling pathway	2	0.12716	0.619	0.059	Biological	GO:0035	purinergic nucleotide receptor signaling pathway	0.12716	0.33057	0	0	2	34
GO:0060914 heart formation	2	0.12716	0.619	0.059	Biological	GO:0060	heart formation	0.12716	0.33057	0	0	2	34
GO:0061081 positive regulation of myeloid leukocyte cytokine productio	2	0.12716	0.619	0.059	Biological	GO:0061	positive regulation of myeloid leukocyte cytokine production involved	0.12716	0.33057	0	0	2	34
GO:0070873 regulation of glycogen metabolic process	2	0.12716	0.619	0.059	Biological	GO:0070	regulation of glycogen metabolic process	0.12716	0.33057	0	0	2	34
GO:0086004 regulation of cardiac muscle cell contraction	2	0.12716	0.619	0.059	Biological	GO:0086	regulation of cardiac muscle cell contraction	0.12716	0.33057	0	0	2	34
GO:0098801 regulation of renal system process	2	0.12716	0.619	0.059	Biological	GO:0098	regulation of renal system process	0.12716	0.33057	0	0	2	34
GO:1904861 excitatory synapse assembly	2	0.12716	0.619	0.059	Biological	GO:1904	excitatory synapse assembly	0.12716	0.33057	0	0	2	34
GO:0007268 chemical synaptic transmission	18	0.12716	5.573	0.024	Biological	GO:0007	chemical synaptic transmission	0.12716	0.33057	0	0	18	736
GO:0098916 anterograde trans-synaptic signaling	18	0.12716	5.573	0.024	Biological	GO:0098	anterograde trans-synaptic signaling	0.12716	0.33057	0	0	18	736
GO:0045669 positive regulation of osteoblast differentiation	3	0.12732	0.929	0.044	Biological	GO:0045	positive regulation of osteoblast differentiation	0.12732	0.33081	0	0	3	68
GO:0021543 pallium development	6	0.12765	1.858	0.032	Biological	GO:0021	pallium development	0.12765	0.3315	0	0	6	187
GO:0008235 metalloexopeptidase activity	3	0.12779	0.877	0.045	Molecular	GO:0008	metalloexopeptidase activity	0.12779	0.33135	0	0	3	67
GO:0043232 intracellular non-membrane-bounded organelle	105	0.12785	31.157	0.02	Cellular C	GO:0043	intracellular non-membrane-bounded organelle	0.12785	0.34324	0	0	105	5280
GO:0045596 negative regulation of cell differentiation	17	0.1281	5.263	0.025	Biological	GO:0045	negative regulation of cell differentiation	0.1281	0.33249	0	0	17	689
GO:0043228 non-membrane-bounded organelle	105	0.12831	31.157	0.02	Cellular C	GO:0043	non-membrane-bounded organelle	0.12831	0.34324	0	0	105	5281
GO:0001776 leukocyte homeostasis	4	0.1286	1.238	0.038	Biological	GO:0001	leukocyte homeostasis	0.1286	0.33362	0	0	4	106
GO:0005737 cytoplasm	229	0.13004	67.953	0.019	Cellular C	GO:0005	cytoplasm	0.13004	0.34614	0	0	229	12135
GO:0051049 regulation of transport	39	0.13029	12.074	0.022	Biological	GO:0051	regulation of transport	0.13029	0.33783	0	0	39	1780
GO:0008376 acetylgalactosaminyltransferase activity	2	0.13063	0.585	0.059	Molecular	GO:0008	acetylgalactosaminyltransferase activity	0.13063	0.33669	0	0	2	34
GO:0030296 protein tyrosine kinase activator activity	2	0.13063	0.585	0.059	Molecular	GO:0030	protein tyrosine kinase activator activity	0.13063	0.33669	0	0	2	34
GO:0014855 striated muscle cell proliferation	3	0.13141	0.929	0.043	Biological	GO:0014	striated muscle cell proliferation	0.13141	0.33849	0	0	3	69
GO:0042093 T-helper cell differentiation	3	0.13141	0.929	0.043	Biological	GO:0042	T-helper cell differentiation	0.13141	0.33849	0	0	3	69
GO:0060675 ureteric bud morphogenesis	3	0.13141	0.929	0.043	Biological	GO:0060	ureteric bud morphogenesis	0.13141	0.33849	0	0	3	69
GO:0006954 inflammatory response	19	0.13152	5.882	0.024	Biological	GO:0006	inflammatory response	0.13152	0.33849	0	0	19	788
GO:0004672 protein kinase activity	15	0.13172	4.386	0.026	Molecular	GO:0004	protein kinase activity	0.13172	0.33812	0	0	15	587
GO:1903076 regulation of protein localization to plasma membrane	4	0.1318	1.238	0.037	Biological	GO:1903	regulation of protein localization to plasma membrane	0.1318	0.33849	0	0	4	107
GO:0003777 microtubule motor activity	3	0.13197	0.877	0.044	Molecular	GO:0003	microtubule motor activity	0.13197	0.33812	0	0	3	68
GO:0150034 distal axon	8	0.13294	2.374	0.029	Cellular C	GO:0150	distal axon	0.13294	0.34924	0	0	8	279
GO:0048534 hematopoietic or lymphoid organ development	23	0.13297	7.121	0.023	Biological	GO:0048	hematopoietic or lymphoid organ development	0.13297	0.33849	0	0	23	984
GO:0001893 maternal placenta development	2	0.13331	0.619	0.057	Biological	GO:0001	maternal placenta development	0.13331	0.33849	0	0	2	35
GO:0048730 epidermis morphogenesis	2	0.13331	0.619	0.057	Biological	GO:0048	epidermis morphogenesis	0.13331	0.33849	0	0	2	35
GO:0060251 regulation of glial cell proliferation	2	0.13331	0.619	0.057	Biological	GO:0060	regulation of glial cell proliferation	0.13331	0.33849	0	0	2	35
GO:0071398 cellular response to fatty acid	2	0.13331	0.619	0.057	Biological	GO:0071	cellular response to fatty acid	0.13331	0.33849	0	0	2	35
GO:0099171 presynaptic modulation of chemical synaptic transmission	2	0.13331	0.619	0.057	Biological	GO:0099	presynaptic modulation of chemical synaptic transmission	0.13331	0.33849	0	0	2	35
GO:1902624 positive regulation of neutrophil migration	2	0.13331	0.619	0.057	Biological	GO:1902	positive regulation of neutrophil migration	0.13331	0.33849	0	0	2	35
GO:0004175 endopeptidase activity	12	0.13334	3.509	0.027	Molecular	GO:0004	endopeptidase activity	0.13334	0.33916	0	0	12	450
GO:0097190 apoptotic signaling pathway	15	0.13436	4.644	0.025	Biological	GO:0097	apoptotic signaling pathway	0.13436	0.33849	0	0	15	599
GO:0030308 negative regulation of cell growth	6	0.13469	1.858	0.032	Biological	GO:0030	negative regulation of cell growth	0.13469	0.33849	0	0	6	190
GO:0034308 primary alcohol metabolic process	4	0.13503	1.238	0.037	Biological	GO:0034	primary alcohol metabolic process	0.13503	0.33849	0	0	4	108
GO:0099537 trans-synaptic signaling	18	0.13516	5.573	0.024	Biological	GO:0099	trans-synaptic signaling	0.13516	0.33849	0	0	18	743
GO:0000976 transcription cis-regulatory region binding	34	0.13536	9.942	0.023	Molecular	GO:0000	transcription cis-regulatory region binding	0.13536	0.33916	0	0	34	1508
GO:0033010 paranodal junction	1	0.13545	0.297	0.125	Cellular C	GO:0033	paranodal junction	0.13545	0.34924	0	0	1	8
GO:0036454 growth factor complex	1	0.13545	0.297	0.125	Cellular C	GO:0036	growth factor complex	0.13545	0.34924	0	0	1	8
GO:0097450 astrocyte end-foot	1	0.13545	0.297	0.125	Cellular C	GO:0097	astrocyte end-foot	0.13545	0.34924	0	0	1	8
GO:0098837 postsynaptic recycling endosome	1	0.13545	0.297	0.125	Cellular C	GO:0098	postsynaptic recycling endosome	0.13545	0.34924	0	0	1	8
GO:0045934 negative regulation of nucleobase-containing compound m	34	0.13545	10.526	0.022	Biological	GO:0045	negative regulation of nucleobase-containing compound metabolic pr	0.13545	0.33849	0	0	34	1534
GO:0106310 protein serine kinase activity	10	0.13549	2.924	0.028	Molecular	GO:0106	protein serine kinase activity	0.13549	0.33916	0	0	10	361
GO:0046635 positive regulation of alpha-beta T cell activation	3	0.13556	0.929	0.043	Biological	GO:0046	positive regulation of alpha-beta T cell activation	0.13556	0.33849	0	0	3	70
GO:0050810 regulation of steroid biosynthetic process	3	0.13556	0.929	0.043	Biological	GO:0050	regulation of steroid biosynthetic process	0.13556	0.33849	0	0	3	70
GO:0060415 muscle tissue morphogenesis	3	0.13556	0.929	0.043	Biological	GO:0060	muscle tissue morphogenesis	0.13556	0.33849	0	0	3	70
GO:0072171 mesonephric tubule morphogenesis	3	0.13556	0.929	0.043	Biological	GO:0072	mesonephric tubule morphogenesis	0.13556	0.33849	0	0	3	70
GO:0044267 cellular protein metabolic process	96	0.13643	29.721	0.02	Biological	GO:0044	cellular protein metabolic process	0.13643	0.33849	0	0	96	4763
GO:0001725 stress fiber	3	0.13643	0.89	0.042	Cellular C	GO:0001	stress fiber	0.13643	0.34924	0	0	3	71
GO:0016328 lateral plasma membrane	3	0.13643	0.89	0.042	Cellular C	GO:0016	lateral plasma membrane	0.13643	0.34924	0	0	3	71
GO:0097517 contractile actin filament bundle	3	0.13643	0.89	0.042	Cellular C	GO:0097	contractile actin filament bundle	0.13643	0.34924	0	0	3	71
GO:0002118 aggressive behavior	1	0.13689	0.31	0.125	Biological	GO:0002	aggressive behavior	0.13689	0.33849	0	0	1	8
GO:0002246 wound healing involved in inflammatory response	1	0.13689	0.31	0.125	Biological	GO:0002	wound healing involved in inflammatory response	0.13689	0.33849	0	0	1	8
GO:0002866 positive regulation of acute inflammatory response to antig	1	0.13689	0.31	0.125	Biological	GO:0002	positive regulation of acute inflammatory response to antigenic stimu	0.13689	0.33849	0	0	1	8
GO:0003149 membranous septum morphogenesis	1	0.13689	0.31	0.125	Biological	GO:0003	membranous septum morphogenesis	0.13689	0.33849	0	0	1	8
GO:0003360 brainstem development	1	0.13689	0.31	0.125	Biological	GO:0003	brainstem development	0.13689	0.33849	0	0	1	8
GO:0003433 chondrocyte development involved in endochondral bone r	1	0.13689	0.31	0.125	Biological	GO:0003	chondrocyte development involved in endochondral bone morphogen	0.13689	0.33849	0	0	1	8
GO:0009414 response to water deprivation	1	0.13689	0.31	0.125	Biological	GO:0009	response to water deprivation	0.13689	0.33849	0	0	1	8
GO:0014004 microglia differentiation	1	0.13689	0.31	0.125	Biological	GO:0014	microglia differentiation	0.13689	0.33849	0	0	1	8
GO:0014041 regulation of neuron maturation	1	0.13689	0.31	0.125	Biological	GO:0014	regulation of neuron maturation	0.13689	0.33849	0	0	1	8
GO:0015888 thiamine transport	1	0.13689	0.31	0.125	Biological	GO:0015	thiamine transport	0.13689	0.33849	0	0	1	8
GO:0021553 olfactory nerve development	1	0.13689	0.31	0.125	Biological	GO:0021	olfactory nerve development	0.13689	0.33849	0	0	1	8
GO:0030854 positive regulation of granulocyte differentiation	1	0.13689	0.31	0.125	Biological	GO:0030	positive regulation of granulocyte differentiation	0.13689	0.33849	0	0	1	8
GO:0030916 otic vesicle formation	1	0.13689	0.31	0.125	Biological	GO:0030	otic vesicle formation	0.13689	0.33849	0	0	1	8
GO:0032119 sequestering of zinc ion	1	0.13689	0.31	0.125	Biological	GO:0032	sequestering of zinc ion	0.13689	0.33849	0	0	1	8
GO:0032489 regulation of Cdc42 protein signal transduction	1	0.13689	0.31	0.125	Biological	GO:0032	regulation of Cdc42 protein signal transduction	0.13689	0.33849	0	0	1	8
GO:0032493 response to bacterial lipoprotein	1	0.13689	0.31	0.125	Biological	GO:0032	response to bacterial lipoprotein	0.13689	0.33849	0	0	1	8
GO:0033278 cell proliferation in midbrain	1	0.13689	0.31	0.125	Biological	GO:0033	cell proliferation in midbrain	0.13689	0.33849	0	0	1	8
GO:0033601 positive regulation of mammary gland epithelial cell prolifer	1	0.13689	0.31	0.125	Biological	GO:0033	positive regulation of mammary gland epithelial cell proliferation	0.13689	0.33849	0	0	1	8
GO:0034699 response to luteinizing hormone	1	0.13689	0.31	0.125	Biological	GO:0034	response to luteinizing hormone	0.13689	0.33849	0	0	1	8
GO:0034983 peptidyl-lysine deacetylation	1	0.13689	0.31	0.125	Biological	GO:0034	peptidyl-lysine deacetylation	0.13689	0.33849	0	0	1	8
GO:0038134 ERBB2-EGFR signaling pathway	1	0.13689	0.31	0.125	Biological	GO:0038	ERBB2-EGFR signaling pathway	0.13689	0.33849	0	0	1	8
GO:0043376 regulation of CD8-positive, alpha-beta T cell differentiation	1	0.13689	0.31	0.125	Biological	GO:0043	regulation of CD8-positive, alpha-beta T cell differentiation	0.13689	0.33849	0	0	1	8
GO:0046476 glycosylceramide biosynthetic process	1	0.13689	0.31	0.125	Biological	GO:0046	glycosylceramide biosynthetic process	0.13689	0.33849	0	0	1	8
GO:0046719 regulation by virus of viral protein levels in host cell	1	0.13689	0.31	0.125	Biological	GO:0046	regulation by virus of viral protein levels in host cell	0.13689	0.33849	0	0	1	8
GO:0046884 follicle-stimulating hormone secretion	1	0.13689	0.31	0.125	Biological	GO:0046	follicle-stimulating hormone secretion	0.13689	0.33849	0	0	1	8
GO:0048227 plasma membrane to endosome transport	1	0.13689	0.31	0.125	Biological	GO:0048	plasma membrane to endosome transport	0.13689	0.33849	0	0	1	8
GO:0050651 dermatan sulfate proteoglycan biosynthetic process	1	0.13689	0.31	0.125	Biological	GO:0050	dermatan sulfate proteoglycan biosynthetic process	0.13689	0.33849	0	0	1	8
GO:0050932 regulation of pigment cell differentiation	1	0.13689	0.31	0.125	Biological	GO:0050	regulation of pigment cell differentiation	0.13689	0.33849	0	0	1	8
GO:0051124 synaptic assembly at neuromuscular junction	1	0.13689	0.31	0.125	Biological	GO:0051	synaptic assembly at neuromuscular junction	0.13689	0.33849	0	0	1	8
GO:0051801 cytolysis in other organism involved in symbiotic interactio	1	0.13689	0.31	0.125	Biological	GO:0051	cytolysis in other organism involved in symbiotic interaction	0.13689	0.33849	0	0	1	8
GO:0051838 cytolysis by host of symbiont cells	1	0.13689	0.31	0.125	Biological	GO:0051	cytolysis by host of symbiont cells	0.13689	0.33849	0	0	1	8
GO:0060836 lymphatic endothelial cell differentiation	1	0.13689	0.31	0.125	Biological	GO:0060	lymphatic endothelial cell differentiation	0.13689	0.33849	0	0	1	8
GO:0060839 endothelial cell fate commitment	1	0.13689	0.31	0.125	Biological	GO:0060	endothelial cell fate commitment	0.13689	0.33849	0	0	1	8
GO:0061518 microglial cell proliferation	1	0.13689	0.31	0.125	Biological	GO:0061	microglial cell proliferation	0.13689	0.33849	0	0	1	8
GO:0062111 zinc ion import into organelle	1	0.13689	0.31	0.125	Biological	GO:0062	zinc ion import into organelle	0.13689	0.33849	0	0	1	8
GO:0070662 mast cell proliferation	1	0.13689	0.31	0.125	Biological	GO:0070	mast cell proliferation	0.13689	0.33849	0	0	1	8
GO:0070666 regulation of mast cell proliferation	1	0.13689	0.31	0.125	Biological	GO:0070	regulation of mast cell proliferation	0.13689	0.33849	0	0	1	8
GO:0071314 cellular response to cocaine	1	0.13689	0.31	0.125	Biological	GO:0071	cellular response to cocaine	0.13689	0.33849	0	0	1	8
GO:0071499 cellular response to laminar fluid shear stress	1	0.13689	0.31	0.125	Biological	GO:0071	cellular response to laminar fluid shear stress	0.13689	0.33849	0	0	1	8
GO:0072161 mesenchymal cell differentiation involved in kidney develop	1	0.13689	0.31	0.125	Biological	GO:0072	mesenchymal cell differentiation involved in kidney development	0.13689	0.33849	0	0	1	8
GO:0072282 metanephric nephron tubule morphogenesis	1	0.13689	0.31	0.125	Biological	GO:0072	metanephric nephron tubule morphogenesis	0.13689	0.33849	0	0	1	8
GO:0080144 amino acid homeostasis	1	0.13689	0.31	0.125	Biological	GO:0080	amino acid homeostasis	0.13689	0.33849	0	0	1	8
GO:0090324 negative regulation of oxidative phosphorylation	1	0.13689	0.31	0.125	Biological	GO:0090	negative regulation of oxidative phosphorylation	0.13689	0.33849	0	0	1	8
GO:0097527 necroptotic signaling pathway	1	0.13689	0.31	0.125	Biological	GO:0097	necroptotic signaling pathway	0.13689	0.33849	0	0	1	8
GO:0098598 learned vocalization behavior or vocal learning	1	0.13689	0.31	0.125	Biological	GO:0098	learned vocalization behavior or vocal learning	0.13689	0.33849	0	0	1	8
GO:0120180 cell-substrate junction disassembly	1	0.13689	0.31	0.125	Biological	GO:0120	cell-substrate junction disassembly	0.13689	0.33849	0	0	1	8
GO:0120181 focal adhesion disassembly	1	0.13689	0.31	0.125	Biological	GO:0120	focal adhesion disassembly	0.13689	0.33849	0	0	1	8
GO:0120197 mucociliary clearance	1	0.13689	0.31	0.125	Biological	GO:0120	mucociliary clearance	0.13689	0.33849	0	0	1	8
GO:1900223 positive regulation of amyloid-beta clearance	1	0.13689	0.31	0.125	Biological	GO:1900	positive regulation of amyloid-beta clearance	0.13689	0.33849	0	0	1	8
GO:1902423 regulation of attachment of mitotic spindle microtubules to	1	0.13689	0.31	0.125	Biological	GO:1902	regulation of attachment of mitotic spindle microtubules to kinetochor	0.13689	0.33849	0	0	1	8
GO:1902425 positive regulation of attachment of mitotic spindle microtu	1	0.13689	0.31	0.125	Biological	GO:1902	positive regulation of attachment of mitotic spindle microtubules to ki	0.13689	0.33849	0	0	1	8
GO:1903430 negative regulation of cell maturation	1	0.13689	0.31	0.125	Biological	GO:1903	negative regulation of cell maturation	0.13689	0.33849	0	0	1	8
GO:1903748 negative regulation of establishment of protein localization	1	0.13689	0.31	0.125	Biological	GO:1903	negative regulation of establishment of protein localization to mitocho	0.13689	0.33849	0	0	1	8
GO:1904124 microglial cell migration	1	0.13689	0.31	0.125	Biological	GO:1904	microglial cell migration	0.13689	0.33849	0	0	1	8
GO:1904139 regulation of microglial cell migration	1	0.13689	0.31	0.125	Biological	GO:1904	regulation of microglial cell migration	0.13689	0.33849	0	0	1	8
GO:1904383 response to sodium phosphate	1	0.13689	0.31	0.125	Biological	GO:1904	response to sodium phosphate	0.13689	0.33849	0	0	1	8
GO:1904396 regulation of neuromuscular junction development	1	0.13689	0.31	0.125	Biological	GO:1904	regulation of neuromuscular junction development	0.13689	0.33849	0	0	1	8
GO:1904415 regulation of xenophagy	1	0.13689	0.31	0.125	Biological	GO:1904	regulation of xenophagy	0.13689	0.33849	0	0	1	8
GO:1904417 positive regulation of xenophagy	1	0.13689	0.31	0.125	Biological	GO:1904	positive regulation of xenophagy	0.13689	0.33849	0	0	1	8
GO:1904714 regulation of chaperone-mediated autophagy	1	0.13689	0.31	0.125	Biological	GO:1904	regulation of chaperone-mediated autophagy	0.13689	0.33849	0	0	1	8
GO:1905918 regulation of CoA-transferase activity	1	0.13689	0.31	0.125	Biological	GO:1905	regulation of CoA-transferase activity	0.13689	0.33849	0	0	1	8
GO:1905920 positive regulation of CoA-transferase activity	1	0.13689	0.31	0.125	Biological	GO:1905	positive regulation of CoA-transferase activity	0.13689	0.33849	0	0	1	8
GO:1990416 cellular response to brain-derived neurotrophic factor stimu	1	0.13689	0.31	0.125	Biological	GO:1990	cellular response to brain-derived neurotrophic factor stimulus	0.13689	0.33849	0	0	1	8
GO:2000020 positive regulation of male gonad development	1	0.13689	0.31	0.125	Biological	GO:2000	positive regulation of male gonad development	0.13689	0.33849	0	0	1	8
GO:2000171 negative regulation of dendrite development	1	0.13689	0.31	0.125	Biological	GO:2000	negative regulation of dendrite development	0.13689	0.33849	0	0	1	8
GO:2000427 positive regulation of apoptotic cell clearance	1	0.13689	0.31	0.125	Biological	GO:2000	positive regulation of apoptotic cell clearance	0.13689	0.33849	0	0	1	8
GO:2000739 regulation of mesenchymal stem cell differentiation	1	0.13689	0.31	0.125	Biological	GO:2000	regulation of mesenchymal stem cell differentiation	0.13689	0.33849	0	0	1	8
GO:2001012 mesenchymal cell differentiation involved in renal system c	1	0.13689	0.31	0.125	Biological	GO:2001	mesenchymal cell differentiation involved in renal system developme	0.13689	0.33849	0	0	1	8
GO:2001137 positive regulation of endocytic recycling	1	0.13689	0.31	0.125	Biological	GO:2001	positive regulation of endocytic recycling	0.13689	0.33849	0	0	1	8
GO:0045732 positive regulation of protein catabolic process	7	0.13691	2.167	0.03	Biological	GO:0045	positive regulation of protein catabolic process	0.13691	0.33849	0	0	7	234
GO:1903050 regulation of proteolysis involved in cellular protein cataboli	7	0.13691	2.167	0.03	Biological	GO:1903	regulation of proteolysis involved in cellular protein catabolic process	0.13691	0.33849	0	0	7	234
GO:0010976 positive regulation of neuron projection development	5	0.13692	1.548	0.034	Biological	GO:0010	positive regulation of neuron projection development	0.13692	0.33849	0	0	5	149
GO:0001067 transcription regulatory region nucleic acid binding	34	0.13702	9.942	0.023	Molecular	GO:0001	transcription regulatory region nucleic acid binding	0.13702	0.33916	0	0	34	1510
GO:0043123 positive regulation of I-kappaB kinase/NF-kappaB signalin	6	0.13707	1.858	0.031	Biological	GO:0043	positive regulation of I-kappaB kinase/NF-kappaB signaling	0.13707	0.33872	0	0	6	191
GO:0044403 biological process involved in symbiotic interaction	9	0.13756	2.786	0.028	Biological	GO:0044	biological process involved in symbiotic interaction	0.13756	0.33975	0	0	9	323
GO:0051172 negative regulation of nitrogen compound metabolic proce	50	0.13786	15.48	0.021	Biological	GO:0051	negative regulation of nitrogen compound metabolic process	0.13786	0.34033	0	0	50	2353
GO:0005774 vacuolar membrane	12	0.13821	3.561	0.026	Cellular C	GO:0005	vacuolar membrane	0.13821	0.35173	0	0	12	466
GO:0006766 vitamin metabolic process	4	0.1383	1.238	0.037	Biological	GO:0006	vitamin metabolic process	0.1383	0.3409	0	0	4	109
GO:0046545 development of primary female sexual characteristics	4	0.1383	1.238	0.037	Biological	GO:0046	development of primary female sexual characteristics	0.1383	0.3409	0	0	4	109
GO:1905954 positive regulation of lipid localization	4	0.1383	1.238	0.037	Biological	GO:1905	positive regulation of lipid localization	0.1383	0.3409	0	0	4	109
GO:0008233 peptidase activity	16	0.13853	4.678	0.025	Molecular	GO:0008	peptidase activity	0.13853	0.33916	0	0	16	639
GO:0045211 postsynaptic membrane	8	0.13872	2.374	0.028	Cellular C	GO:0045	postsynaptic membrane	0.13872	0.35173	0	0	8	282
GO:0001758 retinal dehydrogenase activity	1	0.13903	0.292	0.125	Molecular	GO:0001	retinal dehydrogenase activity	0.13903	0.33916	0	0	1	8
GO:0001882 nucleoside binding	1	0.13903	0.292	0.125	Molecular	GO:0001	nucleoside binding	0.13903	0.33916	0	0	1	8

GO:0004415 hyaluronoglucosaminidase activity	1	0.13903	0.292	0.125	Molecular	GO:0004	hyaluronoglucosaminidase activity	0.13903	0.33916	0	0	1	8
GO:0004897 ciliary neurotrophic factor receptor activity	1	0.13903	0.292	0.125	Molecular	GO:0004	ciliary neurotrophic factor receptor activity	0.13903	0.33916	0	0	1	8
GO:0004972 NMDA glutamate receptor activity	1	0.13903	0.292	0.125	Molecular	GO:0004	NMDA glutamate receptor activity	0.13903	0.33916	0	0	1	8
GO:0016717 oxidoreductase activity, acting on paired donors, with oxida	1	0.13903	0.292	0.125	Molecular	GO:0016	oxidoreductase activity, acting on paired donors, with oxidation of a p	0.13903	0.33916	0	0	1	8
GO:0019534 toxin transmembrane transporter activity	1	0.13903	0.292	0.125	Molecular	GO:0019	toxin transmembrane transporter activity	0.13903	0.33916	0	0	1	8
GO:0031995 insulin-like growth factor II binding	1	0.13903	0.292	0.125	Molecular	GO:0031	insulin-like growth factor II binding	0.13903	0.33916	0	0	1	8
GO:0034046 poly(G) binding	1	0.13903	0.292	0.125	Molecular	GO:0034	poly(G) binding	0.13903	0.33916	0	0	1	8
GO:0046920 alpha-(1->3)-fucosyltransferase activity	1	0.13903	0.292	0.125	Molecular	GO:0046	alpha-(1->3)-fucosyltransferase activity	0.13903	0.33916	0	0	1	8
GO:0047035 testosterone dehydrogenase (NAD+) activity	1	0.13903	0.292	0.125	Molecular	GO:0047	testosterone dehydrogenase (NAD+) activity	0.13903	0.33916	0	0	1	8
GO:0099107 ion channel regulator activity involved in G protein-coupled	1	0.13903	0.292	0.125	Molecular	GO:0099	ion channel regulator activity involved in G protein-coupled receptor s	0.13903	0.33916	0	0	1	8
GO:0071478 cellular response to radiation	6	0.13948	1.858	0.031	Biological	GO:0071	cellular response to radiation	0.13948	0.34243	0	0	6	192
GO:0000132 establishment of mitotic spindle orientation	2	0.13954	0.619	0.056	Biological	GO:0000	establishment of mitotic spindle orientation	0.13954	0.34243	0	0	2	36
GO:0010934 macrophage cytokine production	2	0.13954	0.619	0.056	Biological	GO:0010	macrophage cytokine production	0.13954	0.34243	0	0	2	36
GO:0010935 regulation of macrophage cytokine production	2	0.13954	0.619	0.056	Biological	GO:0010	regulation of macrophage cytokine production	0.13954	0.34243	0	0	2	36
GO:0030224 monocyte differentiation	2	0.13954	0.619	0.056	Biological	GO:0030	monocyte differentiation	0.13954	0.34243	0	0	2	36
GO:0043276 anoikis	2	0.13954	0.619	0.056	Biological	GO:0043	anoikis	0.13954	0.34243	0	0	2	36
GO:0045777 positive regulation of blood pressure	2	0.13954	0.619	0.056	Biological	GO:0045	positive regulation of blood pressure	0.13954	0.34243	0	0	2	36
GO:0050931 pigment cell differentiation	2	0.13954	0.619	0.056	Biological	GO:0050	pigment cell differentiation	0.13954	0.34243	0	0	2	36
GO:0071276 cellular response to cadmium ion	2	0.13954	0.619	0.056	Biological	GO:0071	cellular response to cadmium ion	0.13954	0.34243	0	0	2	36
GO:0046467 membrane lipid biosynthetic process	5	0.13967	1.548	0.033	Biological	GO:0046	membrane lipid biosynthetic process	0.13967	0.34243	0	0	5	150
GO:0002294 CD4-positive, alpha-beta T cell differentiation involved in in	3	0.13974	0.929	0.042	Biological	GO:0002	CD4-positive, alpha-beta T cell differentiation involved in immune res	0.13974	0.34243	0	0	3	71
GO:0015807 L-amino acid transport	3	0.13974	0.929	0.042	Biological	GO:0015	L-amino acid transport	0.13974	0.34243	0	0	3	71
GO:0099513 polymeric cytoskeletal fiber	19	0.14076	5.638	0.024	Cellular C	GO:0099	polymeric cytoskeletal fiber	0.14076	0.35523	0	0	19	805
GO:0014066 regulation of phosphatidylinositol 3-kinase signaling	4	0.14159	1.238	0.036	Biological	GO:0014	regulation of phosphatidylinositol 3-kinase signaling	0.14159	0.34663	0	0	4	110
GO:1904659 glucose transmembrane transport	4	0.14159	1.238	0.036	Biological	GO:1904	glucose transmembrane transport	0.14159	0.34663	0	0	4	110
GO:0048167 regulation of synaptic plasticity	6	0.14191	1.858	0.031	Biological	GO:0048	regulation of synaptic plasticity	0.14191	0.34722	0	0	6	193
GO:0005765 lysosomal membrane	11	0.14268	3.264	0.026	Cellular C	GO:0005	lysosomal membrane	0.14268	0.35671	0	0	11	422
GO:0098852 lytic vacuole membrane	11	0.14268	3.264	0.026	Cellular C	GO:0098	lytic vacuole membrane	0.14268	0.35671	0	0	11	422
GO:0002520 immune system development	24	0.14286	7.43	0.023	Biological	GO:0002	immune system development	0.14286	0.34939	0	0	24	1043
GO:0003700 DNA-binding transcription factor activity	32	0.14303	9.357	0.023	Molecular	GO:0003	DNA-binding transcription factor activity	0.14303	0.34793	0	0	32	1418
GO:0018108 peptidyl-tyrosine phosphorylation	10	0.14386	3.096	0.027	Biological	GO:0018	peptidyl-tyrosine phosphorylation	0.14386	0.35062	0	0	10	372
GO:0002287 alpha-beta T cell activation involved in immune response	3	0.14397	0.929	0.042	Biological	GO:0002	alpha-beta T cell activation involved in immune response	0.14397	0.35062	0	0	3	72
GO:0002293 alpha-beta T cell differentiation involved in immune respon	3	0.14397	0.929	0.042	Biological	GO:0002	alpha-beta T cell differentiation involved in immune response	0.14397	0.35062	0	0	3	72
GO:0032732 positive regulation of interleukin-1 production	3	0.14397	0.929	0.042	Biological	GO:0032	positive regulation of interleukin-1 production	0.14397	0.35062	0	0	3	72
GO:0042058 regulation of epidermal growth factor receptor signaling pa	3	0.14397	0.929	0.042	Biological	GO:0042	regulation of epidermal growth factor receptor signaling pathway	0.14397	0.35062	0	0	3	72
GO:0051057 positive regulation of small GTPase mediated signal transd	3	0.14397	0.929	0.042	Biological	GO:0051	positive regulation of small GTPase mediated signal transduction	0.14397	0.35062	0	0	3	72
GO:0002793 positive regulation of peptide secretion	4	0.14492	1.238	0.036	Biological	GO:0002	positive regulation of peptide secretion	0.14492	0.35062	0	0	4	111
GO:0055007 cardiac muscle cell differentiation	4	0.14492	1.238	0.036	Biological	GO:0055	cardiac muscle cell differentiation	0.14492	0.35062	0	0	4	111
GO:0031333 negative regulation of protein-containing complex assembl	5	0.14525	1.548	0.033	Biological	GO:0031	negative regulation of protein-containing complex assembly	0.14525	0.35062	0	0	5	152
GO:0046718 viral entry into host cell	5	0.14525	1.548	0.033	Biological	GO:0046	viral entry into host cell	0.14525	0.35062	0	0	5	152
GO:1905039 carboxylic acid transmembrane transport	5	0.14525	1.548	0.033	Biological	GO:1905	carboxylic acid transmembrane transport	0.14525	0.35062	0	0	5	152
GO:0071692 protein localization to extracellular region	10	0.1456	3.096	0.027	Biological	GO:0071	protein localization to extracellular region	0.1456	0.35062	0	0	10	373
GO:0061024 membrane organization	21	0.14564	6.502	0.023	Biological	GO:0061	membrane organization	0.14564	0.35062	0	0	21	898
GO:0051254 positive regulation of RNA metabolic process	40	0.14569	12.384	0.022	Biological	GO:0051	positive regulation of RNA metabolic process	0.14569	0.35062	0	0	40	1851
GO:0010035 response to inorganic substance	14	0.14573	4.334	0.025	Biological	GO:0010	response to inorganic substance	0.14573	0.35062	0	0	14	560
GO:1901615 organic hydroxy compound metabolic process	14	0.14573	4.334	0.025	Biological	GO:1901	organic hydroxy compound metabolic process	0.14573	0.35062	0	0	14	560
GO:0031224 intrinsic component of membrane	49	0.14574	14.54	0.021	Cellular C	GO:0031	intrinsic component of membrane	0.14574	0.36074	0	0	49	2338
GO:0000785 chromatin	29	0.14575	8.605	0.022	Cellular C	GO:0000	chromatin	0.14575	0.36074	0	0	29	1309
GO:0002446 neutrophil mediated immunity	2	0.14582	0.619	0.054	Biological	GO:0002	neutrophil mediated immunity	0.14582	0.35062	0	0	2	37
GO:0007588 excretion	2	0.14582	0.619	0.054	Biological	GO:0007	excretion	0.14582	0.35062	0	0	2	37
GO:0010874 regulation of cholesterol efflux	2	0.14582	0.619	0.054	Biological	GO:0010	regulation of cholesterol efflux	0.14582	0.35062	0	0	2	37
GO:0014911 positive regulation of smooth muscle cell migration	2	0.14582	0.619	0.054	Biological	GO:0014	positive regulation of smooth muscle cell migration	0.14582	0.35062	0	0	2	37
GO:0021799 cerebral cortex radially oriented cell migration	2	0.14582	0.619	0.054	Biological	GO:0021	cerebral cortex radially oriented cell migration	0.14582	0.35062	0	0	2	37
GO:0031076 embryonic camera-type eye development	2	0.14582	0.619	0.054	Biological	GO:0031	embryonic camera-type eye development	0.14582	0.35062	0	0	2	37
GO:0045742 positive regulation of epidermal growth factor receptor sign	2	0.14582	0.619	0.054	Biological	GO:0045	positive regulation of epidermal growth factor receptor signaling pathw	0.14582	0.35062	0	0	2	37
GO:0046473 phosphatidic acid metabolic process	2	0.14582	0.619	0.054	Biological	GO:0046	phosphatidic acid metabolic process	0.14582	0.35062	0	0	2	37
GO:0050999 regulation of nitric-oxide synthase activity	2	0.14582	0.619	0.054	Biological	GO:0050	regulation of nitric-oxide synthase activity	0.14582	0.35062	0	0	2	37
GO:0051930 regulation of sensory perception of pain	2	0.14582	0.619	0.054	Biological	GO:0051	regulation of sensory perception of pain	0.14582	0.35062	0	0	2	37
GO:0051931 regulation of sensory perception	2	0.14582	0.619	0.054	Biological	GO:0051	regulation of sensory perception	0.14582	0.35062	0	0	2	37
GO:0055090 acylglycerol homeostasis	2	0.14582	0.619	0.054	Biological	GO:0055	acylglycerol homeostasis	0.14582	0.35062	0	0	2	37
GO:0070328 triglyceride homeostasis	2	0.14582	0.619	0.054	Biological	GO:0070	triglyceride homeostasis	0.14582	0.35062	0	0	2	37
GO:0090218 positive regulation of lipid kinase activity	2	0.14582	0.619	0.054	Biological	GO:0090	positive regulation of lipid kinase activity	0.14582	0.35062	0	0	2	37
GO:0018212 peptidyl-tyrosine modification	10	0.14735	3.096	0.027	Biological	GO:0018	peptidyl-tyrosine modification	0.14735	0.35062	0	0	10	374
GO:2000113 negative regulation of cellular macromolecule biosynthetic	34	0.14805	10.526	0.022	Biological	GO:2000	negative regulation of cellular macromolecule biosynthetic process	0.14805	0.35062	0	0	34	1549
GO:0008643 carbohydrate transport	5	0.14808	1.548	0.033	Biological	GO:0008	carbohydrate transport	0.14808	0.35062	0	0	5	153
GO:0032635 interleukin-6 production	5	0.14808	1.548	0.033	Biological	GO:0032	interleukin-6 production	0.14808	0.35062	0	0	5	153
GO:0032675 regulation of interleukin-6 production	5	0.14808	1.548	0.033	Biological	GO:0032	regulation of interleukin-6 production	0.14808	0.35062	0	0	5	153
GO:0050806 positive regulation of synaptic transmission	5	0.14808	1.548	0.033	Biological	GO:0050	positive regulation of synaptic transmission	0.14808	0.35062	0	0	5	153
GO:1903825 organic acid transmembrane transport	5	0.14808	1.548	0.033	Biological	GO:1903	organic acid transmembrane transport	0.14808	0.35062	0	0	5	153
GO:0003170 heart valve development	3	0.14825	0.929	0.041	Biological	GO:0003	heart valve development	0.14825	0.35062	0	0	3	73
GO:0009247 glycolipid biosynthetic process	3	0.14825	0.929	0.041	Biological	GO:0009	glycolipid biosynthetic process	0.14825	0.35062	0	0	3	73
GO:0010827 regulation of glucose transmembrane transport	3	0.14825	0.929	0.041	Biological	GO:0010	regulation of glucose transmembrane transport	0.14825	0.35062	0	0	3	73
GO:0070373 negative regulation of ERK1 and ERK2 cascade	3	0.14825	0.929	0.041	Biological	GO:0070	negative regulation of ERK1 and ERK2 cascade	0.14825	0.35062	0	0	3	73
GO:0033559 unsaturated fatty acid metabolic process	4	0.14828	1.238	0.036	Biological	GO:0033	unsaturated fatty acid metabolic process	0.14828	0.35062	0	0	4	112
GO:0051897 positive regulation of protein kinase B signaling	4	0.14828	1.238	0.036	Biological	GO:0051	positive regulation of protein kinase B signaling	0.14828	0.35062	0	0	4	112
GO:0061387 regulation of extent of cell growth	4	0.14828	1.238	0.036	Biological	GO:0061	regulation of extent of cell growth	0.14828	0.35062	0	0	4	112
GO:0031968 organelle outer membrane	7	0.14855	2.077	0.029	Cellular C	GO:0031	organelle outer membrane	0.14855	0.36074	0	0	7	242
GO:0051260 protein homooligomerization	6	0.1493	1.858	0.031	Biological	GO:0051	protein homooligomerization	0.1493	0.35062	0	0	6	196
GO:0043198 dendritic shaft	2	0.14947	0.593	0.053	Cellular C	GO:0043	dendritic shaft	0.14947	0.36074	0	0	2	38
GO:0019903 protein phosphatase binding	5	0.14958	1.462	0.033	Molecular	GO:0019	protein phosphatase binding	0.14958	0.36017	0	0	5	151
GO:0022843 voltage-gated cation channel activity	5	0.14958	1.462	0.033	Molecular	GO:0022	voltage-gated cation channel activity	0.14958	0.36017	0	0	5	151
GO:0000146 microfilament motor activity	2	0.14973	0.585	0.054	Molecular	GO:0000	microfilament motor activity	0.14973	0.36017	0	0	2	37
GO:0016229 steroid dehydrogenase activity	2	0.14973	0.585	0.054	Molecular	GO:0016	steroid dehydrogenase activity	0.14973	0.36017	0	0	2	37
GO:0050807 regulation of synapse organization	7	0.15008	2.167	0.029	Biological	GO:0050	regulation of synapse organization	0.15008	0.35062	0	0	7	240
GO:0043524 negative regulation of neuron apoptotic process	5	0.15093	1.548	0.032	Biological	GO:0043	negative regulation of neuron apoptotic process	0.15093	0.35062	0	0	5	154
GO:0010557 positive regulation of macromolecule biosynthetic process	42	0.15098	13.003	0.021	Biological	GO:0010	positive regulation of macromolecule biosynthetic process	0.15098	0.35062	0	0	42	1960
GO:0005916 fascia adherens	1	0.15104	0.297	0.111	Cellular C	GO:0005	fascia adherens	0.15104	0.36074	0	0	1	9
GO:0008091 spectrin	1	0.15104	0.297	0.111	Cellular C	GO:0008	spectrin	0.15104	0.36074	0	0	1	9
GO:0017146 NMDA selective glutamate receptor complex	1	0.15104	0.297	0.111	Cellular C	GO:0017	NMDA selective glutamate receptor complex	0.15104	0.36074	0	0	1	9
GO:0031095 platelet dense tubular network membrane	1	0.15104	0.297	0.111	Cellular C	GO:0031	platelet dense tubular network membrane	0.15104	0.36074	0	0	1	9
GO:0031264 death-inducing signaling complex	1	0.15104	0.297	0.111	Cellular C	GO:0031	death-inducing signaling complex	0.15104	0.36074	0	0	1	9
GO:0042824 MHC class I peptide loading complex	1	0.15104	0.297	0.111	Cellular C	GO:0042	MHC class I peptide loading complex	0.15104	0.36074	0	0	1	9
GO:0004620 phospholipase activity	4	0.15118	1.17	0.036	Molecular	GO:0004	phospholipase activity	0.15118	0.36266	0	0	4	111
GO:0002444 myeloid leukocyte mediated immunity	4	0.15167	1.238	0.035	Biological	GO:0002	myeloid leukocyte mediated immunity	0.15167	0.35062	0	0	4	113
GO:0046578 regulation of Ras protein signal transduction	6	0.1518	1.858	0.03	Biological	GO:0046	regulation of Ras protein signal transduction	0.1518	0.35062	0	0	6	197
GO:0051223 regulation of protein transport	13	0.15213	4.025	0.025	Biological	GO:0051	regulation of protein transport	0.15213	0.35062	0	0	13	517
GO:0003180 aortic valve morphogenesis	2	0.15217	0.619	0.053	Biological	GO:0003	aortic valve morphogenesis	0.15217	0.35062	0	0	2	38
GO:0010092 specification of animal organ identity	2	0.15217	0.619	0.053	Biological	GO:0010	specification of animal organ identity	0.15217	0.35062	0	0	2	38
GO:0010737 protein kinase A signaling	2	0.15217	0.619	0.053	Biological	GO:0010	protein kinase A signaling	0.15217	0.35062	0	0	2	38
GO:0032373 positive regulation of sterol transport	2	0.15217	0.619	0.053	Biological	GO:0032	positive regulation of sterol transport	0.15217	0.35062	0	0	2	38
GO:0032376 positive regulation of cholesterol transport	2	0.15217	0.619	0.053	Biological	GO:0032	positive regulation of cholesterol transport	0.15217	0.35062	0	0	2	38
GO:0034110 regulation of homotypic cell-cell adhesion	2	0.15217	0.619	0.053	Biological	GO:0034	regulation of homotypic cell-cell adhesion	0.15217	0.35062	0	0	2	38
GO:0038179 neurotrophin signaling pathway	2	0.15217	0.619	0.053	Biological	GO:0038	neurotrophin signaling pathway	0.15217	0.35062	0	0	2	38
GO:0060323 head morphogenesis	2	0.15217	0.619	0.053	Biological	GO:0060	head morphogenesis	0.15217	0.35062	0	0	2	38
GO:0090049 regulation of cell migration involved in sprouting angiogene	2	0.15217	0.619	0.053	Biological	GO:0090	regulation of cell migration involved in sprouting angiogenesis	0.15217	0.35062	0	0	2	38
GO:0042490 mechanoreceptor differentiation	3	0.15256	0.929	0.041	Biological	GO:0042	mechanoreceptor differentiation	0.15256	0.35062	0	0	3	74
GO:0045665 negative regulation of neuron differentiation	3	0.15256	0.929	0.041	Biological	GO:0045	negative regulation of neuron differentiation	0.15256	0.35062	0	0	3	74
GO:0050805 negative regulation of synaptic transmission	3	0.15256	0.929	0.041	Biological	GO:0050	negative regulation of synaptic transmission	0.15256	0.35062	0	0	3	74
GO:0071677 positive regulation of mononuclear cell migration	3	0.15256	0.929	0.041	Biological	GO:0071	positive regulation of mononuclear cell migration	0.15256	0.35062	0	0	3	74
GO:0001542 ovulation from ovarian follicle	1	0.15263	0.31	0.111	Biological	GO:0001	ovulation from ovarian follicle	0.15263	0.35062	0	0	1	9
GO:0002883 regulation of hypersensitivity	1	0.15263	0.31	0.111	Biological	GO:0002	regulation of hypersensitivity	0.15263	0.35062	0	0	1	9
GO:0003266 regulation of secondary heart field cardioblast proliferation	1	0.15263	0.31	0.111	Biological	GO:0003	regulation of secondary heart field cardioblast proliferation	0.15263	0.35062	0	0	1	9
GO:0006069 ethanol oxidation	1	0.15263	0.31	0.111	Biological	GO:0006	ethanol oxidation	0.15263	0.35062	0	0	1	9
GO:0006569 tryptophan catabolic process	1	0.15263	0.31	0.111	Biological	GO:0006	tryptophan catabolic process	0.15263	0.35062	0	0	1	9
GO:0010533 regulation of activation of Janus kinase activity	1	0.15263	0.31	0.111	Biological	GO:0010	regulation of activation of Janus kinase activity	0.15263	0.35062	0	0	1	9
GO:0010692 regulation of alkaline phosphatase activity	1	0.15263	0.31	0.111	Biological	GO:0010	regulation of alkaline phosphatase activity	0.15263	0.35062	0	0	1	9
GO:0010835 regulation of protein ADP-ribosylation	1	0.15263	0.31	0.111	Biological	GO:0010	regulation of protein ADP-ribosylation	0.15263	0.35062	0	0	1	9
GO:0014745 negative regulation of muscle adaptation	1	0.15263	0.31	0.111	Biological	GO:0014	negative regulation of muscle adaptation	0.15263	0.35062	0	0	1	9
GO:0014877 response to muscle inactivity involved in regulation of mus	1	0.15263	0.31	0.111	Biological	GO:0014	response to muscle inactivity involved in regulation of muscle adapta	0.15263	0.35062	0	0	1	9
GO:0014894 response to denervation involved in regulation of muscle a	1	0.15263	0.31	0.111	Biological	GO:0014	response to denervation involved in regulation of muscle adaptation	0.15263	0.35062	0	0	1	9
GO:0015825 L-serine transport	1	0.15263	0.31	0.111	Biological	GO:0015	L-serine transport	0.15263	0.35062	0	0	1	9
GO:0015858 nucleoside transport	1	0.15263	0.31	0.111	Biological	GO:0015	nucleoside transport	0.15263	0.35062	0	0	1	9
GO:0015871 choline transport	1	0.15263	0.31	0.111	Biological	GO:0015	choline transport	0.15263	0.35062	0	0	1	9
GO:0015884 folic acid transport	1	0.15263	0.31	0.111	Biological	GO:0015	folic acid transport	0.15263	0.35062	0	0	1	9
GO:0015917 aminophospholipid transport	1	0.15263	0.31	0.111	Biological	GO:0015	aminophospholipid transport	0.15263	0.35062	0	0	1	9
GO:0019530 taurine metabolic process	1	0.15263	0.31	0.111	Biological	GO:0019	taurine metabolic process	0.15263	0.35062	0	0	1	9
GO:0019694 alkanesulfonate metabolic process	1	0.15263	0.31	0.111	Biological	GO:0019	alkanesulfonate metabolic process	0.15263	0.35062	0	0	1	9
GO:0031620 regulation of fever generation	1	0.15263	0.31	0.111	Biological	GO:0031	regulation of fever generation	0.15263	0.35062	0	0	1	9
GO:0032906 transforming growth factor beta2 production	1	0.15263	0.31	0.111	Biological	GO:0032	transforming growth factor beta2 production	0.15263	0.35062	0	0	1	9
GO:0032909 regulation of transforming growth factor beta2 production	1	0.15263	0.31	0.111	Biological	GO:0032	regulation of transforming growth factor beta2 production	0.15263	0.35062	0	0	1	9
GO:0035733 hepatic stellate cell activation	1	0.15263	0.31	0.111	Biological	GO:0035	hepatic stellate cell activation	0.15263	0.35062	0	0	1	9
GO:0042436 indole-containing compound catabolic process	1	0.15263	0.31	0.111	Biological	GO:0042	indole-containing compound catabolic process	0.15263	0.35062	0	0	1	9
GO:0042590 antigen processing and presentation of exogenous peptide	1	0.15263	0.31	0.111	Biological	GO:0042	antigen processing and presentation of exogenous peptide antigen vi	0.15263	0.35062	0	0	1	9
GO:0043174 nucleoside salvage	1	0.15263	0.31	0.111	Biological	GO:0043	nucleoside salvage	0.15263	0.35062	0	0	1	9
GO:0044848 biological phase	1	0.15263	0.31	0.111	Biological	GO:0044	biological phase	0.15263	0.35062	0	0	1	9
GO:0044851 hair cycle phase	1	0.15263	0.31	0.111	Biological	GO:0044	hair cycle phase	0.15263	0.35062	0	0	1	9
GO:0046218 indolalkylamine catabolic process	1	0.15263	0.31	0.111	Biological	GO:0046	indolalkylamine catabolic process	0.15263	0.35062	0	0	1	9
GO:0048087 positive regulation of developmental pigmentation	1	0.15263	0.31	0.111	Biological	GO:0048	positive regulation of developmental pigmentation	0.15263	0.35062	0	0	1	9
GO:0048819 regulation of hair follicle maturation	1	0.15263	0.31	0.111	Biological	GO:0048	regulation of hair follicle maturation	0.15263	0.35062	0	0	1	9
GO:0048845 venous blood vessel morphogenesis	1	0.15263	0.31	0.111	Biological	GO:0048	venous blood vessel morphogenesis	0.15263	0.35062	0	0	1	9
GO:0050957 equilibrioception	1	0.15263	0.31	0.111	Biological	GO:0050	equilibrioception	0.15263	0.35062	0	0	1	9
GO:0051639 actin filament network formation	1	0.15263	0.31	0.111	Biological	GO:0051	actin filament network formation	0.15263	0.35062	0	0	1	9
GO:0051715 cytolysis in other organism	1	0.15263	0.31	0.111	Biological	GO:0051	cytolysis in other organism	0.15263	0.35062	0	0	1	9
GO:0060013 righting reflex	1	0.15263	0.31	0.111	Biological	GO:0060	righting reflex	0.15263	0.35062	0	0	1	9
GO:0060052 neurofilament cytoskeleton organization	1	0.15263	0.31	0.111	Biological	GO:0060	neurofilament cytoskeleton organization	0.15263	0.35062	0	0	1	9
GO:0060316 positive regulation of ryanodine-sensitive calcium-release c	1	0.15263	0.31	0.111	Biological	GO:0060	positive regulation of ryanodine-sensitive calcium-release channel act	0.15263	0.35062	0	0	1	9
GO:0060700 regulation of ribonuclease activity	1	0.15263	0.31	0.111	Biological	GO:0060	regulation of ribonuclease activity	0.15263	0.35062	0	0	1	9
GO:0060717 chorion development	1	0.15263	0.31	0.111	Biological	GO:0060	chorion development	0.15263	0.35062	0	0	1	9
GO:0061072 iris morphogenesis	1	0.15263	0.31	0.111	Biological	GO:0061	iris morphogenesis	0.15263	0.35062	0	0	1	9
GO:0061549 sympathetic ganglion development	1	0.15263	0.31	0.111	Biological	GO:0061	sympathetic ganglion development	0.15263	0.35062	0	0	1	9
GO:0062043 positive regulation of cardiac epithelial to mesenchymal tra	1	0.15263	0.31	0.111	Biological	GO:0062	positive regulation of cardiac epithelial to mesenchymal transition	0.15263	0.35062	0	0	1	9
GO:0070292 N-acylphosphatidylethanolamine metabolic process	1	0.15263	0.31	0.111	Biological	GO:0070	N-acylphosphatidylethanolamine metabolic process	0.15263	0.35062	0	0	1	9

GO:0070305 response to cGMP	1	0.15263	0.31	0.111	Biological	GO:00703	response to cGMP	0.15263	0.35062	0	0	1	9
GO:0071225 cellular response to muramyl dipeptide	1	0.15263	0.31	0.111	Biological	GO:00712	cellular response to muramyl dipeptide	0.15263	0.35062	0	0	1	9
GO:0072102 glomerulus morphogenesis	1	0.15263	0.31	0.111	Biological	GO:00721	glomerulus morphogenesis	0.15263	0.35062	0	0	1	9
GO:0072239 metanephric glomerulus vasculature development	1	0.15263	0.31	0.111	Biological	GO:00722	metanephric glomerulus vasculature development	0.15263	0.35062	0	0	1	9
GO:0090394 negative regulation of excitatory postsynaptic potential	1	0.15263	0.31	0.111	Biological	GO:00903	negative regulation of excitatory postsynaptic potential	0.15263	0.35062	0	0	1	9
GO:0098937 anterograde dendritic transport	1	0.15263	0.31	0.111	Biological	GO:00989	anterograde dendritic transport	0.15263	0.35062	0	0	1	9
GO:0099612 protein localization to axon	1	0.15263	0.31	0.111	Biological	GO:00996	protein localization to axon	0.15263	0.35062	0	0	1	9
GO:0099641 anterograde axonal protein transport	1	0.15263	0.31	0.111	Biological	GO:00996	anterograde axonal protein transport	0.15263	0.35062	0	0	1	9
GO:1900118 negative regulation of execution phase of apoptosis	1	0.15263	0.31	0.111	Biological	GO:19001	negative regulation of execution phase of apoptosis	0.15263	0.35062	0	0	1	9
GO:1902035 positive regulation of hematopoietic stem cell proliferation	1	0.15263	0.31	0.111	Biological	GO:19020	positive regulation of hematopoietic stem cell proliferation	0.15263	0.35062	0	0	1	9
GO:1902732 positive regulation of chondrocyte proliferation	1	0.15263	0.31	0.111	Biological	GO:19027	positive regulation of chondrocyte proliferation	0.15263	0.35062	0	0	1	9
GO:1902965 regulation of protein localization to early endosome	1	0.15263	0.31	0.111	Biological	GO:19029	regulation of protein localization to early endosome	0.15263	0.35062	0	0	1	9
GO:1902966 positive regulation of protein localization to early endosome	1	0.15263	0.31	0.111	Biological	GO:19029	positive regulation of protein localization to early endosome	0.15263	0.35062	0	0	1	9
GO:1903044 protein localization to membrane raft	1	0.15263	0.31	0.111	Biological	GO:19030	protein localization to membrane raft	0.15263	0.35062	0	0	1	9
GO:1903251 multi-ciliated epithelial cell differentiation	1	0.15263	0.31	0.111	Biological	GO:19032	multi-ciliated epithelial cell differentiation	0.15263	0.35062	0	0	1	9
GO:1903589 positive regulation of blood vessel endothelial cell proliferat	1	0.15263	0.31	0.111	Biological	GO:19035	positive regulation of blood vessel endothelial cell proliferation involve	0.15263	0.35062	0	0	1	9
GO:1904776 regulation of protein localization to cell cortex	1	0.15263	0.31	0.111	Biological	GO:19047	regulation of protein localization to cell cortex	0.15263	0.35062	0	0	1	9
GO:1905276 regulation of epithelial tube formation	1	0.15263	0.31	0.111	Biological	GO:19052	regulation of epithelial tube formation	0.15263	0.35062	0	0	1	9
GO:1905278 positive regulation of epithelial tube formation	1	0.15263	0.31	0.111	Biological	GO:19052	positive regulation of epithelial tube formation	0.15263	0.35062	0	0	1	9
GO:1905941 positive regulation of gonad development	1	0.15263	0.31	0.111	Biological	GO:19059	positive regulation of gonad development	0.15263	0.35062	0	0	1	9
GO:1990806 ligand-gated ion channel signaling pathway	1	0.15263	0.31	0.111	Biological	GO:19908	ligand-gated ion channel signaling pathway	0.15263	0.35062	0	0	1	9
GO:2000018 regulation of male gonad development	1	0.15263	0.31	0.111	Biological	GO:20000	regulation of male gonad development	0.15263	0.35062	0	0	1	9
GO:2000169 regulation of peptidyl-cysteine S-nitrosylation	1	0.15263	0.31	0.111	Biological	GO:20001	regulation of peptidyl-cysteine S-nitrosylation	0.15263	0.35062	0	0	1	9
GO:2000465 regulation of glycogen (starch) synthase activity	1	0.15263	0.31	0.111	Biological	GO:20004	regulation of glycogen (starch) synthase activity	0.15263	0.35062	0	0	1	9
GO:2001187 positive regulation of CD8-positive, alpha-beta T cell activa	1	0.15263	0.31	0.111	Biological	GO:20011	positive regulation of CD8-positive, alpha-beta T cell activation	0.15263	0.35062	0	0	1	9
GO:0019867 outer membrane	7	0.153	2.077	0.029	Cellular C	GO:00198	outer membrane	0.153	0.36284	0	0	7	244
GO:0016234 inclusion body	3	0.15327	0.89	0.04	Cellular C	GO:00162	inclusion body	0.15327	0.36284	0	0	3	75
GO:0004860 protein kinase inhibitor activity	3	0.15351	0.877	0.041	Molecular	GO:00048	protein kinase inhibitor activity	0.15351	0.36277	0	0	3	73
GO:0007612 learning	5	0.1538	1.548	0.032	Biological	GO:00076	learning	0.1538	0.35314	0	0	5	155
GO:0120032 regulation of plasma membrane bounded cell projection as	6	0.15432	1.858	0.03	Biological	GO:01200	regulation of plasma membrane bounded cell projection assembly	0.15432	0.35418	0	0	6	198
GO:0015194 L-serine transmembrane transporter activity	1	0.155	0.292	0.111	Molecular	GO:00151	L-serine transmembrane transporter activity	0.155	0.36277	0	0	1	9
GO:0015924 mannosyl-oligosaccharide mannosidase activity	1	0.155	0.292	0.111	Molecular	GO:00159	mannosyl-oligosaccharide mannosidase activity	0.155	0.36277	0	0	1	9
GO:0016671 oxidoreductase activity, acting on a sulfur group of donors,	1	0.155	0.292	0.111	Molecular	GO:00166	oxidoreductase activity, acting on a sulfur group of donors, disulfide a	0.155	0.36277	0	0	1	9
GO:0031545 peptidyl-proline 4-dioxygenase activity	1	0.155	0.292	0.111	Molecular	GO:00315	peptidyl-proline 4-dioxygenase activity	0.155	0.36277	0	0	1	9
GO:0046977 TAP binding	1	0.155	0.292	0.111	Molecular	GO:00469	TAP binding	0.155	0.36277	0	0	1	9
GO:0047045 testosterone 17-beta-dehydrogenase (NADP+) activity	1	0.155	0.292	0.111	Molecular	GO:00470	testosterone 17-beta-dehydrogenase (NADP+) activity	0.155	0.36277	0	0	1	9
GO:0097200 cysteine-type endopeptidase activity involved in execution	1	0.155	0.292	0.111	Molecular	GO:00972	cysteine-type endopeptidase activity involved in execution phase of a	0.155	0.36277	0	0	1	9
GO:1905394 retromer complex binding	1	0.155	0.292	0.111	Molecular	GO:19053	retromer complex binding	0.155	0.36277	0	0	1	9
GO:0008645 hexose transmembrane transport	4	0.15509	1.238	0.035	Biological	GO:00086	hexose transmembrane transport	0.15509	0.35577	0	0	4	114
GO:1902532 negative regulation of intracellular signal transduction	13	0.15517	4.025	0.025	Biological	GO:19025	negative regulation of intracellular signal transduction	0.15517	0.3558	0	0	13	519
GO:0031672 A band	2	0.15577	0.593	0.051	Cellular C	GO:00316	A band	0.15577	0.36712	0	0	2	39
GO:0043028 cysteine-type endopeptidase regulator activity involved in a	2	0.15621	0.585	0.053	Molecular	GO:00430	cysteine-type endopeptidase regulator activity involved in apoptotic p	0.15621	0.36463	0	0	2	38
GO:0010558 negative regulation of macromolecule biosynthetic process	34	0.15683	10.526	0.022	Biological	GO:00105	negative regulation of macromolecule biosynthetic process	0.15683	0.35934	0	0	34	1559
GO:1901605 alpha-amino acid metabolic process	6	0.15686	1.858	0.03	Biological	GO:19016	alpha-amino acid metabolic process	0.15686	0.35934	0	0	6	199
GO:0042581 specific granule	5	0.15741	1.484	0.032	Cellular C	GO:00425	specific granule	0.15741	0.36935	0	0	5	158
GO:0043406 positive regulation of MAP kinase activity	4	0.15854	1.238	0.035	Biological	GO:00434	positive regulation of MAP kinase activity	0.15854	0.36208	0	0	4	115
GO:0071901 negative regulation of protein serine/threonine kinase activ	4	0.15854	1.238	0.035	Biological	GO:00719	negative regulation of protein serine/threonine kinase activity	0.15854	0.36208	0	0	4	115
GO:0007618 mating	2	0.15856	0.619	0.051	Biological	GO:00076	mating	0.15856	0.36208	0	0	2	39
GO:0044273 sulfur compound catabolic process	2	0.15856	0.619	0.051	Biological	GO:00442	sulfur compound catabolic process	0.15856	0.36208	0	0	2	39
GO:0045823 positive regulation of heart contraction	2	0.15856	0.619	0.051	Biological	GO:00458	positive regulation of heart contraction	0.15856	0.36208	0	0	2	39
GO:0098926 postsynaptic signal transduction	2	0.15856	0.619	0.051	Biological	GO:00989	postsynaptic signal transduction	0.15856	0.36208	0	0	2	39
GO:1901186 positive regulation of ERBB signaling pathway	2	0.15856	0.619	0.051	Biological	GO:19011	positive regulation of ERBB signaling pathway	0.15856	0.36208	0	0	2	39
GO:0009913 epidermal cell differentiation	7	0.15918	2.167	0.029	Biological	GO:00099	epidermal cell differentiation	0.15918	0.36332	0	0	7	244
GO:0007568 aging	8	0.15937	2.477	0.028	Biological	GO:00075	aging	0.15937	0.36354	0	0	8	289
GO:0016324 apical plasma membrane	10	0.1594	2.967	0.026	Cellular C	GO:00163	apical plasma membrane	0.1594	0.37241	0	0	10	385
GO:0060491 regulation of cell projection assembly	6	0.15942	1.858	0.03	Biological	GO:00604	regulation of cell projection assembly	0.15942	0.36354	0	0	6	200
GO:0007189 adenylate cyclase-activating G protein-coupled receptor si	5	0.15961	1.548	0.032	Biological	GO:00071	adenylate cyclase-activating G protein-coupled receptor signaling pat	0.15961	0.36365	0	0	5	157
GO:0061351 neural precursor cell proliferation	5	0.15961	1.548	0.032	Biological	GO:00613	neural precursor cell proliferation	0.15961	0.36365	0	0	5	157
GO:0019902 phosphatase binding	6	0.1602	1.754	0.03	Molecular	GO:00199	phosphatase binding	0.1602	0.37294	0	0	6	197
GO:0007585 respiratory gaseous exchange by respiratory system	3	0.1613	0.929	0.039	Biological	GO:00075	respiratory gaseous exchange by respiratory system	0.1613	0.36666	0	0	3	76
GO:0055013 cardiac muscle cell development	3	0.1613	0.929	0.039	Biological	GO:00550	cardiac muscle cell development	0.1613	0.36666	0	0	3	76
GO:0055117 regulation of cardiac muscle contraction	3	0.1613	0.929	0.039	Biological	GO:00551	regulation of cardiac muscle contraction	0.1613	0.36666	0	0	3	76
GO:0060337 type I interferon signaling pathway	3	0.1613	0.929	0.039	Biological	GO:00603	type I interferon signaling pathway	0.1613	0.36666	0	0	3	76
GO:0097194 execution phase of apoptosis	3	0.1613	0.929	0.039	Biological	GO:00971	execution phase of apoptosis	0.1613	0.36666	0	0	3	76
GO:0032414 positive regulation of ion transmembrane transporter activi	4	0.16201	1.238	0.034	Biological	GO:00324	positive regulation of ion transmembrane transporter activity	0.16201	0.3681	0	0	4	116
GO:0008328 ionotropic glutamate receptor complex	2	0.16212	0.593	0.05	Cellular C	GO:00083	ionotropic glutamate receptor complex	0.16212	0.37711	0	0	2	40
GO:0004177 aminopeptidase activity	2	0.16275	0.585	0.051	Molecular	GO:00041	aminopeptidase activity	0.16275	0.3769	0	0	2	39
GO:0140110 transcription regulator activity	42	0.16332	12.281	0.022	Molecular	GO:01401	transcription regulator activity	0.16332	0.3769	0	0	42	1942
GO:0007188 adenylate cyclase-modulating G protein-coupled receptor s	7	0.16382	2.167	0.028	Biological	GO:00071	adenylate cyclase-modulating G protein-coupled receptor signaling p	0.16382	0.36817	0	0	7	246
GO:0050803 regulation of synapse structure or activity	7	0.16382	2.167	0.028	Biological	GO:00508	regulation of synapse structure or activity	0.16382	0.36817	0	0	7	246
GO:0050870 positive regulation of T cell activation	7	0.16382	2.167	0.028	Biological	GO:00508	positive regulation of T cell activation	0.16382	0.36817	0	0	7	246
GO:0031327 negative regulation of cellular biosynthetic process	35	0.16415	10.836	0.022	Biological	GO:00313	negative regulation of cellular biosynthetic process	0.16415	0.36817	0	0	35	1618
GO:0010453 regulation of cell fate commitment	2	0.16501	0.619	0.05	Biological	GO:00104	regulation of cell fate commitment	0.16501	0.36817	0	0	2	40
GO:0010907 positive regulation of glucose metabolic process	2	0.16501	0.619	0.05	Biological	GO:00109	positive regulation of glucose metabolic process	0.16501	0.36817	0	0	2	40
GO:0032728 positive regulation of interferon-beta production	2	0.16501	0.619	0.05	Biological	GO:00327	positive regulation of interferon-beta production	0.16501	0.36817	0	0	2	40
GO:0034381 plasma lipoprotein particle clearance	2	0.16501	0.619	0.05	Biological	GO:00343	plasma lipoprotein particle clearance	0.16501	0.36817	0	0	2	40
GO:0090207 regulation of triglyceride metabolic process	2	0.16501	0.619	0.05	Biological	GO:00902	regulation of triglyceride metabolic process	0.16501	0.36817	0	0	2	40
GO:0097106 postsynaptic density organization	2	0.16501	0.619	0.05	Biological	GO:00971	postsynaptic density organization	0.16501	0.36817	0	0	2	40
GO:1901661 quinone metabolic process	2	0.16501	0.619	0.05	Biological	GO:19016	quinone metabolic process	0.16501	0.36817	0	0	2	40
GO:1903524 positive regulation of blood circulation	2	0.16501	0.619	0.05	Biological	GO:19035	positive regulation of blood circulation	0.16501	0.36817	0	0	2	40
GO:0002367 cytokine production involved in immune response	4	0.16552	1.238	0.034	Biological	GO:00023	cytokine production involved in immune response	0.16552	0.36817	0	0	4	117
GO:0002718 regulation of cytokine production involved in immune respo	4	0.16552	1.238	0.034	Biological	GO:00027	regulation of cytokine production involved in immune response	0.16552	0.36817	0	0	4	117
GO:0015749 monosaccharide transmembrane transport	4	0.16552	1.238	0.034	Biological	GO:00157	monosaccharide transmembrane transport	0.16552	0.36817	0	0	4	117
GO:0035710 CD4-positive, alpha-beta T cell activation	4	0.16552	1.238	0.034	Biological	GO:00357	CD4-positive, alpha-beta T cell activation	0.16552	0.36817	0	0	4	117
GO:0044409 entry into host	5	0.16552	1.548	0.031	Biological	GO:00444	entry into host	0.16552	0.36817	0	0	5	159
GO:0048644 muscle organ morphogenesis	3	0.16573	0.929	0.039	Biological	GO:00486	muscle organ morphogenesis	0.16573	0.36817	0	0	3	77
GO:0031902 late endosome membrane	5	0.16614	1.484	0.031	Cellular C	GO:00319	late endosome membrane	0.16614	0.38196	0	0	5	161
GO:0044224 juxtaparanode region of axon	1	0.16635	0.297	0.1	Cellular C	GO:00442	juxtaparanode region of axon	0.16635	0.38196	0	0	1	10
GO:0099571 postsynaptic cytoskeleton	1	0.16635	0.297	0.1	Cellular C	GO:00995	postsynaptic cytoskeleton	0.16635	0.38196	0	0	1	10
GO:0003093 regulation of glomerular filtration	1	0.16809	0.31	0.1	Biological	GO:00030	regulation of glomerular filtration	0.16809	0.36817	0	0	1	10
GO:0003228 atrial cardiac muscle tissue development	1	0.16809	0.31	0.1	Biological	GO:00032	atrial cardiac muscle tissue development	0.16809	0.36817	0	0	1	10
GO:0003263 cardioblast proliferation	1	0.16809	0.31	0.1	Biological	GO:00032	cardioblast proliferation	0.16809	0.36817	0	0	1	10
GO:0003264 regulation of cardioblast proliferation	1	0.16809	0.31	0.1	Biological	GO:00032	regulation of cardioblast proliferation	0.16809	0.36817	0	0	1	10
GO:0003310 pancreatic A cell differentiation	1	0.16809	0.31	0.1	Biological	GO:00033	pancreatic A cell differentiation	0.16809	0.36817	0	0	1	10
GO:0003418 growth plate cartilage chondrocyte differentiation	1	0.16809	0.31	0.1	Biological	GO:00034	growth plate cartilage chondrocyte differentiation	0.16809	0.36817	0	0	1	10
GO:0006707 cholesterol catabolic process	1	0.16809	0.31	0.1	Biological	GO:00067	cholesterol catabolic process	0.16809	0.36817	0	0	1	10
GO:0007021 tubulin complex assembly	1	0.16809	0.31	0.1	Biological	GO:00070	tubulin complex assembly	0.16809	0.36817	0	0	1	10
GO:0010917 negative regulation of mitochondrial membrane potential	1	0.16809	0.31	0.1	Biological	GO:00109	negative regulation of mitochondrial membrane potential	0.16809	0.36817	0	0	1	10
GO:0014824 artery smooth muscle contraction	1	0.16809	0.31	0.1	Biological	GO:00148	artery smooth muscle contraction	0.16809	0.36817	0	0	1	10
GO:0014870 response to muscle inactivity	1	0.16809	0.31	0.1	Biological	GO:00148	response to muscle inactivity	0.16809	0.36817	0	0	1	10
GO:0015803 branched-chain amino acid transport	1	0.16809	0.31	0.1	Biological	GO:00158	branched-chain amino acid transport	0.16809	0.36817	0	0	1	10
GO:0016093 polyprenol metabolic process	1	0.16809	0.31	0.1	Biological	GO:00160	polyprenol metabolic process	0.16809	0.36817	0	0	1	10
GO:0016127 sterol catabolic process	1	0.16809	0.31	0.1	Biological	GO:00161	sterol catabolic process	0.16809	0.36817	0	0	1	10
GO:0018242 protein O-linked glycosylation via serine	1	0.16809	0.31	0.1	Biological	GO:00182	protein O-linked glycosylation via serine	0.16809	0.36817	0	0	1	10
GO:0018243 protein O-linked glycosylation via threonine	1	0.16809	0.31	0.1	Biological	GO:00182	protein O-linked glycosylation via threonine	0.16809	0.36817	0	0	1	10
GO:0021561 facial nerve development	1	0.16809	0.31	0.1	Biological	GO:00215	facial nerve development	0.16809	0.36817	0	0	1	10
GO:0021610 facial nerve morphogenesis	1	0.16809	0.31	0.1	Biological	GO:00216	facial nerve morphogenesis	0.16809	0.36817	0	0	1	10
GO:0030205 dermatan sulfate metabolic process	1	0.16809	0.31	0.1	Biological	GO:00302	dermatan sulfate metabolic process	0.16809	0.36817	0	0	1	10
GO:0031642 negative regulation of myelination	1	0.16809	0.31	0.1	Biological	GO:00316	negative regulation of myelination	0.16809	0.36817	0	0	1	10
GO:0031666 positive regulation of lipopolysaccharide-mediated signaling	1	0.16809	0.31	0.1	Biological	GO:00316	positive regulation of lipopolysaccharide-mediated signaling pathway	0.16809	0.36817	0	0	1	10
GO:0032275 luteinizing hormone secretion	1	0.16809	0.31	0.1	Biological	GO:00322	luteinizing hormone secretion	0.16809	0.36817	0	0	1	10
GO:0033572 transferrin transport	1	0.16809	0.31	0.1	Biological	GO:00335	transferrin transport	0.16809	0.36817	0	0	1	10
GO:0034115 negative regulation of heterotypic cell-cell adhesion	1	0.16809	0.31	0.1	Biological	GO:00341	negative regulation of heterotypic cell-cell adhesion	0.16809	0.36817	0	0	1	10
GO:0035581 sequestering of extracellular ligand from receptor	1	0.16809	0.31	0.1	Biological	GO:00355	sequestering of extracellular ligand from receptor	0.16809	0.36817	0	0	1	10
GO:0036109 alpha-linolenic acid metabolic process	1	0.16809	0.31	0.1	Biological	GO:00361	alpha-linolenic acid metabolic process	0.16809	0.36817	0	0	1	10
GO:0038130 ERBB4 signaling pathway	1	0.16809	0.31	0.1	Biological	GO:00381	ERBB4 signaling pathway	0.16809	0.36817	0	0	1	10
GO:0038171 cannabinoid signaling pathway	1	0.16809	0.31	0.1	Biological	GO:00381	cannabinoid signaling pathway	0.16809	0.36817	0	0	1	10
GO:0043587 tongue morphogenesis	1	0.16809	0.31	0.1	Biological	GO:00435	tongue morphogenesis	0.16809	0.36817	0	0	1	10
GO:0044557 relaxation of smooth muscle	1	0.16809	0.31	0.1	Biological	GO:00445	relaxation of smooth muscle	0.16809	0.36817	0	0	1	10
GO:0045625 regulation of T-helper 1 cell differentiation	1	0.16809	0.31	0.1	Biological	GO:00456	regulation of T-helper 1 cell differentiation	0.16809	0.36817	0	0	1	10
GO:0045657 positive regulation of monocyte differentiation	1	0.16809	0.31	0.1	Biological	GO:00456	positive regulation of monocyte differentiation	0.16809	0.36817	0	0	1	10
GO:0045837 negative regulation of membrane potential	1	0.16809	0.31	0.1	Biological	GO:00458	negative regulation of membrane potential	0.16809	0.36817	0	0	1	10
GO:0046886 positive regulation of hormone biosynthetic process	1	0.16809	0.31	0.1	Biological	GO:00468	positive regulation of hormone biosynthetic process	0.16809	0.36817	0	0	1	10
GO:0051660 establishment of centrosome localization	1	0.16809	0.31	0.1	Biological	GO:00516	establishment of centrosome localization	0.16809	0.36817	0	0	1	10
GO:0051967 negative regulation of synaptic transmission, glutamatergic	1	0.16809	0.31	0.1	Biological	GO:00519	negative regulation of synaptic transmission, glutamatergic	0.16809	0.36817	0	0	1	10
GO:0060054 positive regulation of epithelial cell proliferation involved in	1	0.16809	0.31	0.1	Biological	GO:00600	positive regulation of epithelial cell proliferation involved in wound hea	0.16809	0.36817	0	0	1	10
GO:0060346 bone trabecula formation	1	0.16809	0.31	0.1	Biological	GO:00603	bone trabecula formation	0.16809	0.36817	0	0	1	10
GO:0060502 epithelial cell proliferation involved in lung morphogenesis	1	0.16809	0.31	0.1	Biological	GO:00605	epithelial cell proliferation involved in lung morphogenesis	0.16809	0.36817	0	0	1	10
GO:0060513 prostatic bud formation	1	0.16809	0.31	0.1	Biological	GO:00605	prostatic bud formation	0.16809	0.36817	0	0	1	10
GO:0060525 prostate glandular acinus development	1	0.16809	0.31	0.1	Biological	GO:00605	prostate glandular acinus development	0.16809	0.36817	0	0	1	10
GO:0060768 regulation of epithelial cell proliferation involved in prostate	1	0.16809	0.31	0.1	Biological	GO:00607	regulation of epithelial cell proliferation involved in prostate gland deve	0.16809	0.36817	0	0	1	10
GO:0062042 regulation of cardiac epithelial to mesenchymal transition	1	0.16809	0.31	0.1	Biological	GO:00620	regulation of cardiac epithelial to mesenchymal transition	0.16809	0.36817	0	0	1	10
GO:0070391 response to lipoteichoic acid	1	0.16809	0.31	0.1	Biological	GO:00703	response to lipoteichoic acid	0.16809	0.36817	0	0	1	10
GO:0070587 regulation of cell-cell adhesion involved in gastrulation	1	0.16809	0.31	0.1	Biological	GO:00705	regulation of cell-cell adhesion involved in gastrulation	0.16809	0.36817	0	0	1	10
GO:0071223 cellular response to lipoteichoic acid	1	0.16809	0.31	0.1	Biological	GO:00712	cellular response to lipoteichoic acid	0.16809	0.36817	0	0	1	10
GO:0071600 otic vesicle morphogenesis	1	0.16809	0.31	0.1	Biological	GO:00716	otic vesicle morphogenesis	0.16809	0.36817	0	0	1	10
GO:0071803 positive regulation of podosome assembly	1	0.16809	0.31	0.1	Biological	GO:00718	positive regulation of podosome assembly	0.16809	0.36817	0	0	1	10
GO:0072172 mesonephric tubule formation	1	0.16809	0.31	0.1	Biological	GO:00721	mesonephric tubule formation	0.16809	0.36817	0	0	1	10
GO:0090209 negative regulation of triglyceride metabolic process	1	0.16809	0.31	0.1	Biological	GO:00902	negative regulation of triglyceride metabolic process	0.16809	0.36817	0	0	1	10
GO:0097104 postsynaptic membrane assembly	1	0.16809	0.31	0.1	Biological	GO:00971	postsynaptic membrane assembly	0.16809	0.36817	0	0	1	10
GO:0097105 presynaptic membrane assembly	1	0.16809	0.31	0.1	Biological	GO:00971	presynaptic membrane assembly	0.16809	0.36817	0	0	1	10
GO:0097201 negative regulation of transcription from RNA polymerase	1	0.16809	0.31	0.1	Biological	GO:00972	negative regulation of transcription from RNA polymerase II promoter	0.16809	0.36817	0	0	1	10
GO:0098976 excitatory chemical synaptic transmission	1	0.16809	0.31	0.1	Biological	GO:00989	excitatory chemical synaptic transmission	0.16809	0.36817	0	0	1	10
GO:0099640 axo-dendritic protein transport	1	0.16809	0.31	0.1	Biological	GO:00996	axo-dendritic protein transport	0.16809	0.36817	0	0	1	10
GO:1901203 positive regulation of extracellular matrix assembly	1	0.16809	0.31	0.1	Biological	GO:19012	positive regulation of extracellular matrix assembly	0.16809	0.36817	0	0	1	10
GO:1901856 negative regulation of cellular respiration	1	0.16809	0.31	0.1	Biological	GO:19018	negative regulation of cellular respiration	0.16809	0.36817	0	0	1	10
GO:1903596 regulation of gap junction assembly	1	0.16809	0.31	0.1	Biological	GO:19035	regulation of gap junction assembly	0.16809	0.36817	0	0	1	10
GO:1903977 positive regulation of glial cell migration	1	0.16809	0.31	0.1	Biological	GO:19039	positive regulation of glial cell migration	0.16809	0.36817	0	0	1	10
GO:1905097 regulation of guanyl-nucleotide exchange factor activity	1	0.16809	0.31	0.1	Biological	GO:19050	regulation of guanyl-nucleotide exchange factor activity	0.16809	0.36817	0	0	1	10
GO:2000425 regulation of apoptotic cell clearance	1	0.16809	0.31	0.1	Biological	GO:20004	regulation of apoptotic cell clearance	0.16809	0.36817	0	0	1	10
GO:2000563 positive regulation of CD4-positive, alpha-beta T cell prolife	1	0.16809	0.31	0.1	Biological	GO:20005	positive regulation of CD4-positive, alpha-beta T cell proliferation	0.16809	0.36817	0	0	1	10
GO:2000822 regulation of behavioral fear response	1	0.16809	0.31	0.1	Biological	GO:20008	regulation of behavioral fear response	0.16809	0.36817	0	0	1	10

GO:2001054 negative regulation of mesenchymal cell apoptotic process	1	0.16809	0.31	0.1	Biological	GO:20010	negative regulation of mesenchymal cell apoptotic process	0.16809	0.36817	0	0	1	10
GO:0030139 endocytic vesicle	9	0.1685	2.671	0.026	Cellular C	GO:00301	endocytic vesicle	0.1685	0.38365	0	0	9	343
GO:0030140 trans-Golgi network transport vesicle	2	0.16852	0.593	0.049	Cellular C	GO:00301	trans-Golgi network transport vesicle	0.16852	0.38365	0	0	2	41
GO:0071345 cellular response to cytokine stimulus	19	0.16854	5.882	0.023	Biological	GO:00713	cellular response to cytokine stimulus	0.16854	0.36901	0	0	19	819
GO:0019901 protein kinase binding	17	0.1686	4.971	0.024	Molecular	GO:00199	protein kinase binding	0.1686	0.3769	0	0	17	709
GO:0099094 ligand-gated cation channel activity	4	0.16883	1.17	0.034	Molecular	GO:00990	ligand-gated cation channel activity	0.16883	0.3769	0	0	4	116
GO:0002286 T cell activation involved in immune response	4	0.16905	1.238	0.034	Biological	GO:00022	T cell activation involved in immune response	0.16905	0.36978	0	0	4	118
GO:0030203 glycosaminoglycan metabolic process	4	0.16905	1.238	0.034	Biological	GO:00302	glycosaminoglycan metabolic process	0.16905	0.36978	0	0	4	118
GO:0019216 regulation of lipid metabolic process	9	0.17011	2.786	0.026	Biological	GO:00192	regulation of lipid metabolic process	0.17011	0.37132	0	0	9	340
GO:0008088 axo-dendritic transport	3	0.1702	0.929	0.038	Biological	GO:00080	axo-dendritic transport	0.1702	0.37132	0	0	3	78
GO:0021536 diencephalon development	3	0.1702	0.929	0.038	Biological	GO:00215	diencephalon development	0.1702	0.37132	0	0	3	78
GO:0071357 cellular response to type I interferon	3	0.1702	0.929	0.038	Biological	GO:00713	cellular response to type I interferon	0.1702	0.37132	0	0	3	78
GO:0072078 nephron tubule morphogenesis	3	0.1702	0.929	0.038	Biological	GO:00720	nephron tubule morphogenesis	0.1702	0.37132	0	0	3	78
GO:2000736 regulation of stem cell differentiation	3	0.1702	0.929	0.038	Biological	GO:20007	regulation of stem cell differentiation	0.1702	0.37132	0	0	3	78
GO:0000099 sulfur amino acid transmembrane transporter activity	1	0.17066	0.292	0.1	Molecular	GO:00000	sulfur amino acid transmembrane transporter activity	0.17066	0.3769	0	0	1	10
GO:0003680 minor groove of adenine-thymine-rich DNA binding	1	0.17066	0.292	0.1	Molecular	GO:00036	minor groove of adenine-thymine-rich DNA binding	0.17066	0.3769	0	0	1	10
GO:0004955 prostaglandin receptor activity	1	0.17066	0.292	0.1	Molecular	GO:00049	prostaglandin receptor activity	0.17066	0.3769	0	0	1	10
GO:0015180 L-alanine transmembrane transporter activity	1	0.17066	0.292	0.1	Molecular	GO:00151	L-alanine transmembrane transporter activity	0.17066	0.3769	0	0	1	10
GO:0015183 L-aspartate transmembrane transporter activity	1	0.17066	0.292	0.1	Molecular	GO:00151	L-aspartate transmembrane transporter activity	0.17066	0.3769	0	0	1	10
GO:0015187 glycine transmembrane transporter activity	1	0.17066	0.292	0.1	Molecular	GO:00151	glycine transmembrane transporter activity	0.17066	0.3769	0	0	1	10
GO:0015643 toxic substance binding	1	0.17066	0.292	0.1	Molecular	GO:00156	toxic substance binding	0.17066	0.3769	0	0	1	10
GO:0019911 structural constituent of myelin sheath	1	0.17066	0.292	0.1	Molecular	GO:00199	structural constituent of myelin sheath	0.17066	0.3769	0	0	1	10
GO:0034713 type I transforming growth factor beta receptor binding	1	0.17066	0.292	0.1	Molecular	GO:00347	type I transforming growth factor beta receptor binding	0.17066	0.3769	0	0	1	10
GO:0043121 neurotrophin binding	1	0.17066	0.292	0.1	Molecular	GO:00431	neurotrophin binding	0.17066	0.3769	0	0	1	10
GO:0046974 histone methyltransferase activity (H3-K9 specific)	1	0.17066	0.292	0.1	Molecular	GO:00469	histone methyltransferase activity (H3-K9 specific)	0.17066	0.3769	0	0	1	10
GO:0052740 1-acyl-2-lysophosphatidylserine acylhydrolase activity	1	0.17066	0.292	0.1	Molecular	GO:00527	1-acyl-2-lysophosphatidylserine acylhydrolase activity	0.17066	0.3769	0	0	1	10
GO:0070739 protein-glutamic acid ligase activity	1	0.17066	0.292	0.1	Molecular	GO:00707	protein-glutamic acid ligase activity	0.17066	0.3769	0	0	1	10
GO:0070740 tubulin-glutamic acid ligase activity	1	0.17066	0.292	0.1	Molecular	GO:00707	tubulin-glutamic acid ligase activity	0.17066	0.3769	0	0	1	10
GO:0097199 cysteine-type endopeptidase activity involved in apoptotic s	1	0.17066	0.292	0.1	Molecular	GO:00971	cysteine-type endopeptidase activity involved in apoptotic signaling p	0.17066	0.3769	0	0	1	10
GO:0001618 virus receptor activity	3	0.17147	0.877	0.039	Molecular	GO:00016	virus receptor activity	0.17147	0.3769	0	0	3	77
GO:0016835 carbon-oxygen lyase activity	3	0.17147	0.877	0.039	Molecular	GO:00168	carbon-oxygen lyase activity	0.17147	0.3769	0	0	3	77
GO:0019210 kinase inhibitor activity	3	0.17147	0.877	0.039	Molecular	GO:00192	kinase inhibitor activity	0.17147	0.3769	0	0	3	77
GO:0003016 respiratory system process	2	0.1715	0.619	0.049	Biological	GO:00030	respiratory system process	0.1715	0.37271	0	0	2	41
GO:0006882 cellular zinc ion homeostasis	2	0.1715	0.619	0.049	Biological	GO:00068	cellular zinc ion homeostasis	0.1715	0.37271	0	0	2	41
GO:0009069 serine family amino acid metabolic process	2	0.1715	0.619	0.049	Biological	GO:00090	serine family amino acid metabolic process	0.1715	0.37271	0	0	2	41
GO:0021532 neural tube patterning	2	0.1715	0.619	0.049	Biological	GO:00215	neural tube patterning	0.1715	0.37271	0	0	2	41
GO:0032881 regulation of polysaccharide metabolic process	2	0.1715	0.619	0.049	Biological	GO:00328	regulation of polysaccharide metabolic process	0.1715	0.37271	0	0	2	41
GO:0042417 dopamine metabolic process	2	0.1715	0.619	0.049	Biological	GO:00424	dopamine metabolic process	0.1715	0.37271	0	0	2	41
GO:0048741 skeletal muscle fiber development	2	0.1715	0.619	0.049	Biological	GO:00487	skeletal muscle fiber development	0.1715	0.37271	0	0	2	41
GO:1902742 apoptotic process involved in development	2	0.1715	0.619	0.049	Biological	GO:19027	apoptotic process involved in development	0.1715	0.37271	0	0	2	41
GO:0050905 neuromuscular process	5	0.1715	1.548	0.031	Biological	GO:00509	neuromuscular process	0.1715	0.37271	0	0	5	161
GO:0042734 presynaptic membrane	5	0.17207	1.484	0.031	Cellular C	GO:00427	presynaptic membrane	0.17207	0.39006	0	0	5	163
GO:0019221 cytokine-mediated signaling pathway	12	0.17207	3.715	0.025	Biological	GO:00192	cytokine-mediated signaling pathway	0.17207	0.37378	0	0	12	482
GO:0000209 protein polyubiquitination	7	0.17328	2.167	0.028	Biological	GO:00002	protein polyubiquitination	0.17328	0.37609	0	0	7	250
GO:0031334 positive regulation of protein-containing complex assembly	7	0.17328	2.167	0.028	Biological	GO:00313	positive regulation of protein-containing complex assembly	0.17328	0.37609	0	0	7	250
GO:0048471 perinuclear region of cytoplasm	17	0.17397	5.045	0.023	Cellular C	GO:00484	perinuclear region of cytoplasm	0.17397	0.39272	0	0	17	733
GO:0048639 positive regulation of developmental growth	5	0.17453	1.548	0.031	Biological	GO:00486	positive regulation of developmental growth	0.17453	0.37832	0	0	5	162
GO:0002720 positive regulation of cytokine production involved in immu	3	0.17469	0.929	0.038	Biological	GO:00027	positive regulation of cytokine production involved in immune respons	0.17469	0.37832	0	0	3	79
GO:0009880 embryonic pattern specification	3	0.17469	0.929	0.038	Biological	GO:00098	embryonic pattern specification	0.17469	0.37832	0	0	3	79
GO:0043502 regulation of muscle adaptation	3	0.17469	0.929	0.038	Biological	GO:00435	regulation of muscle adaptation	0.17469	0.37832	0	0	3	79
GO:1901184 regulation of ERBB signaling pathway	3	0.17469	0.929	0.038	Biological	GO:19011	regulation of ERBB signaling pathway	0.17469	0.37832	0	0	3	79
GO:0033017 sarcoplasmic reticulum membrane	2	0.17496	0.593	0.048	Cellular C	GO:00330	sarcoplasmic reticulum membrane	0.17496	0.39328	0	0	2	42
GO:0016021 integral component of membrane	48	0.17581	14.243	0.021	Cellular C	GO:00160	integral component of membrane	0.17581	0.39355	0	0	48	2326
GO:0140272 exogenous protein binding	3	0.17606	0.877	0.038	Molecular	GO:01402	exogenous protein binding	0.17606	0.38599	0	0	3	78
GO:0046632 alpha-beta T cell differentiation	4	0.17618	1.238	0.033	Biological	GO:00466	alpha-beta T cell differentiation	0.17618	0.38139	0	0	4	120
GO:0046943 carboxylic acid transmembrane transporter activity	5	0.17659	1.462	0.031	Molecular	GO:00469	carboxylic acid transmembrane transporter activity	0.17659	0.38619	0	0	5	160
GO:0007595 lactation	2	0.17803	0.619	0.048	Biological	GO:00075	lactation	0.17803	0.38182	0	0	2	42
GO:0019320 hexose catabolic process	2	0.17803	0.619	0.048	Biological	GO:00193	hexose catabolic process	0.17803	0.38182	0	0	2	42
GO:0040001 establishment of mitotic spindle localization	2	0.17803	0.619	0.048	Biological	GO:00400	establishment of mitotic spindle localization	0.17803	0.38182	0	0	2	42
GO:0048246 macrophage chemotaxis	2	0.17803	0.619	0.048	Biological	GO:00482	macrophage chemotaxis	0.17803	0.38182	0	0	2	42
GO:0051155 positive regulation of striated muscle cell differentiation	2	0.17803	0.619	0.048	Biological	GO:00511	positive regulation of striated muscle cell differentiation	0.17803	0.38182	0	0	2	42
GO:0055069 zinc ion homeostasis	2	0.17803	0.619	0.048	Biological	GO:00550	zinc ion homeostasis	0.17803	0.38182	0	0	2	42
GO:1901998 toxin transport	2	0.17803	0.619	0.048	Biological	GO:19019	toxin transport	0.17803	0.38182	0	0	2	42
GO:2000403 positive regulation of lymphocyte migration	2	0.17803	0.619	0.048	Biological	GO:20004	positive regulation of lymphocyte migration	0.17803	0.38182	0	0	2	42
GO:0001664 G protein-coupled receptor binding	8	0.1787	2.339	0.027	Molecular	GO:00016	G protein-coupled receptor binding	0.1787	0.3898	0	0	8	293
GO:0002292 T cell differentiation involved in immune response	3	0.17922	0.929	0.037	Biological	GO:00022	T cell differentiation involved in immune response	0.17922	0.38182	0	0	3	80
GO:0014068 positive regulation of phosphatidylinositol 3-kinase signalin	3	0.17922	0.929	0.037	Biological	GO:00140	positive regulation of phosphatidylinositol 3-kinase signaling	0.17922	0.38182	0	0	3	80
GO:0005342 organic acid transmembrane transporter activity	5	0.1797	1.462	0.031	Molecular	GO:00053	organic acid transmembrane transporter activity	0.1797	0.39101	0	0	5	161
GO:0018210 peptidyl-threonine modification	4	0.17979	1.238	0.033	Biological	GO:00182	peptidyl-threonine modification	0.17979	0.38182	0	0	4	121
GO:0045931 positive regulation of mitotic cell cycle	4	0.17979	1.238	0.033	Biological	GO:00459	positive regulation of mitotic cell cycle	0.17979	0.38182	0	0	4	121
GO:0051101 regulation of DNA binding	4	0.17979	1.238	0.033	Biological	GO:00511	regulation of DNA binding	0.17979	0.38182	0	0	4	121
GO:0005635 nuclear envelope	12	0.17986	3.561	0.024	Cellular C	GO:00056	nuclear envelope	0.17986	0.39759	0	0	12	492
GO:0030705 cytoskeleton-dependent intracellular transport	6	0.18052	1.858	0.029	Biological	GO:00307	cytoskeleton-dependent intracellular transport	0.18052	0.38182	0	0	6	208
GO:0031094 platelet dense tubular network	1	0.18138	0.297	0.091	Cellular C	GO:00310	platelet dense tubular network	0.18138	0.39759	0	0	1	11
GO:0032280 symmetric synapse	1	0.18138	0.297	0.091	Cellular C	GO:00322	symmetric synapse	0.18138	0.39759	0	0	1	11
GO:0098981 cholinergic synapse	1	0.18138	0.297	0.091	Cellular C	GO:00989	cholinergic synapse	0.18138	0.39759	0	0	1	11
GO:0097060 synaptic membrane	10	0.18176	2.967	0.025	Cellular C	GO:00970	synaptic membrane	0.18176	0.39759	0	0	10	397
GO:0099634 postsynaptic specialization membrane	4	0.18207	1.187	0.033	Cellular C	GO:00996	postsynaptic specialization membrane	0.18207	0.39759	0	0	4	123
GO:1903320 regulation of protein modification by small protein conjugat	7	0.18298	2.167	0.028	Biological	GO:19033	regulation of protein modification by small protein conjugation or rem	0.18298	0.38182	0	0	7	254
GO:2000241 regulation of reproductive process	6	0.18323	1.858	0.029	Biological	GO:20002	regulation of reproductive process	0.18323	0.38182	0	0	6	209
GO:0001660 fever generation	1	0.18326	0.31	0.091	Biological	GO:00016	fever generation	0.18326	0.38182	0	0	1	11
GO:0002158 osteoclast proliferation	1	0.18326	0.31	0.091	Biological	GO:00021	osteoclast proliferation	0.18326	0.38182	0	0	1	11
GO:0002732 positive regulation of dendritic cell cytokine production	1	0.18326	0.31	0.091	Biological	GO:00027	positive regulation of dendritic cell cytokine production	0.18326	0.38182	0	0	1	11
GO:0003357 noradrenergic neuron differentiation	1	0.18326	0.31	0.091	Biological	GO:00033	noradrenergic neuron differentiation	0.18326	0.38182	0	0	1	11
GO:0006568 tryptophan metabolic process	1	0.18326	0.31	0.091	Biological	GO:00065	tryptophan metabolic process	0.18326	0.38182	0	0	1	11
GO:0006995 cellular response to nitrogen starvation	1	0.18326	0.31	0.091	Biological	GO:00069	cellular response to nitrogen starvation	0.18326	0.38182	0	0	1	11
GO:0007406 negative regulation of neuroblast proliferation	1	0.18326	0.31	0.091	Biological	GO:00074	negative regulation of neuroblast proliferation	0.18326	0.38182	0	0	1	11
GO:0007494 midgut development	1	0.18326	0.31	0.091	Biological	GO:00074	midgut development	0.18326	0.38182	0	0	1	11
GO:0010936 negative regulation of macrophage cytokine production	1	0.18326	0.31	0.091	Biological	GO:00109	negative regulation of macrophage cytokine production	0.18326	0.38182	0	0	1	11
GO:0014820 tonic smooth muscle contraction	1	0.18326	0.31	0.091	Biological	GO:00148	tonic smooth muscle contraction	0.18326	0.38182	0	0	1	11
GO:0018401 peptidyl-proline hydroxylation to 4-hydroxy-L-proline	1	0.18326	0.31	0.091	Biological	GO:00184	peptidyl-proline hydroxylation to 4-hydroxy-L-proline	0.18326	0.38182	0	0	1	11
GO:0019062 virion attachment to host cell	1	0.18326	0.31	0.091	Biological	GO:00190	virion attachment to host cell	0.18326	0.38182	0	0	1	11
GO:0019065 receptor-mediated endocytosis of virus by host cell	1	0.18326	0.31	0.091	Biological	GO:00190	receptor-mediated endocytosis of virus by host cell	0.18326	0.38182	0	0	1	11
GO:0021520 spinal cord motor neuron cell fate specification	1	0.18326	0.31	0.091	Biological	GO:00215	spinal cord motor neuron cell fate specification	0.18326	0.38182	0	0	1	11
GO:0021604 cranial nerve structural organization	1	0.18326	0.31	0.091	Biological	GO:00216	cranial nerve structural organization	0.18326	0.38182	0	0	1	11
GO:0030320 cellular monovalent inorganic anion homeostasis	1	0.18326	0.31	0.091	Biological	GO:00303	cellular monovalent inorganic anion homeostasis	0.18326	0.38182	0	0	1	11
GO:0030644 cellular chloride ion homeostasis	1	0.18326	0.31	0.091	Biological	GO:00306	cellular chloride ion homeostasis	0.18326	0.38182	0	0	1	11
GO:0031223 auditory behavior	1	0.18326	0.31	0.091	Biological	GO:00312	auditory behavior	0.18326	0.38182	0	0	1	11
GO:0032060 bleb assembly	1	0.18326	0.31	0.091	Biological	GO:00320	bleb assembly	0.18326	0.38182	0	0	1	11
GO:0032276 regulation of gonadotropin secretion	1	0.18326	0.31	0.091	Biological	GO:00322	regulation of gonadotropin secretion	0.18326	0.38182	0	0	1	11
GO:0032621 interleukin-18 production	1	0.18326	0.31	0.091	Biological	GO:00326	interleukin-18 production	0.18326	0.38182	0	0	1	11
GO:0032661 regulation of interleukin-18 production	1	0.18326	0.31	0.091	Biological	GO:00326	regulation of interleukin-18 production	0.18326	0.38182	0	0	1	11
GO:0032966 negative regulation of collagen biosynthetic process	1	0.18326	0.31	0.091	Biological	GO:00329	negative regulation of collagen biosynthetic process	0.18326	0.38182	0	0	1	11
GO:0033504 floor plate development	1	0.18326	0.31	0.091	Biological	GO:00335	floor plate development	0.18326	0.38182	0	0	1	11
GO:0033632 regulation of cell-cell adhesion mediated by integrin	1	0.18326	0.31	0.091	Biological	GO:00336	regulation of cell-cell adhesion mediated by integrin	0.18326	0.38182	0	0	1	11
GO:0034384 high-density lipoprotein particle clearance	1	0.18326	0.31	0.091	Biological	GO:00343	high-density lipoprotein particle clearance	0.18326	0.38182	0	0	1	11
GO:0035524 proline transmembrane transport	1	0.18326	0.31	0.091	Biological	GO:00355	proline transmembrane transport	0.18326	0.38182	0	0	1	11
GO:0036152 phosphatidylethanolamine acyl-chain remodeling	1	0.18326	0.31	0.091	Biological	GO:00361	phosphatidylethanolamine acyl-chain remodeling	0.18326	0.38182	0	0	1	11
GO:0036462 TRAIL-activated apoptotic signaling pathway	1	0.18326	0.31	0.091	Biological	GO:00364	TRAIL-activated apoptotic signaling pathway	0.18326	0.38182	0	0	1	11
GO:0042428 serotonin metabolic process	1	0.18326	0.31	0.091	Biological	GO:00424	serotonin metabolic process	0.18326	0.38182	0	0	1	11
GO:0042451 purine nucleoside biosynthetic process	1	0.18326	0.31	0.091	Biological	GO:00424	purine nucleoside biosynthetic process	0.18326	0.38182	0	0	1	11
GO:0042455 ribonucleoside biosynthetic process	1	0.18326	0.31	0.091	Biological	GO:00424	ribonucleoside biosynthetic process	0.18326	0.38182	0	0	1	11
GO:0042487 regulation of odontogenesis of dentin-containing tooth	1	0.18326	0.31	0.091	Biological	GO:00424	regulation of odontogenesis of dentin-containing tooth	0.18326	0.38182	0	0	1	11
GO:0043084 penile erection	1	0.18326	0.31	0.091	Biological	GO:00430	penile erection	0.18326	0.38182	0	0	1	11
GO:0043562 cellular response to nitrogen levels	1	0.18326	0.31	0.091	Biological	GO:00435	cellular response to nitrogen levels	0.18326	0.38182	0	0	1	11
GO:0045654 positive regulation of megakaryocyte differentiation	1	0.18326	0.31	0.091	Biological	GO:00456	positive regulation of megakaryocyte differentiation	0.18326	0.38182	0	0	1	11
GO:0046129 purine ribonucleoside biosynthetic process	1	0.18326	0.31	0.091	Biological	GO:00461	purine ribonucleoside biosynthetic process	0.18326	0.38182	0	0	1	11
GO:0048505 regulation of timing of cell differentiation	1	0.18326	0.31	0.091	Biological	GO:00485	regulation of timing of cell differentiation	0.18326	0.38182	0	0	1	11
GO:0048672 positive regulation of collateral sprouting	1	0.18326	0.31	0.091	Biological	GO:00486	positive regulation of collateral sprouting	0.18326	0.38182	0	0	1	11
GO:0051593 response to folic acid	1	0.18326	0.31	0.091	Biological	GO:00515	response to folic acid	0.18326	0.38182	0	0	1	11
GO:0051987 positive regulation of attachment of spindle microtubules to	1	0.18326	0.31	0.091	Biological	GO:00519	positive regulation of attachment of spindle microtubules to kinetocho	0.18326	0.38182	0	0	1	11
GO:0060020 Bergmann glial cell differentiation	1	0.18326	0.31	0.091	Biological	GO:00600	Bergmann glial cell differentiation	0.18326	0.38182	0	0	1	11
GO:0060174 limb bud formation	1	0.18326	0.31	0.091	Biological	GO:00601	limb bud formation	0.18326	0.38182	0	0	1	11
GO:0060433 bronchus development	1	0.18326	0.31	0.091	Biological	GO:00604	bronchus development	0.18326	0.38182	0	0	1	11
GO:0060856 establishment of blood-brain barrier	1	0.18326	0.31	0.091	Biological	GO:00608	establishment of blood-brain barrier	0.18326	0.38182	0	0	1	11
GO:0061418 regulation of transcription from RNA polymerase II promote	1	0.18326	0.31	0.091	Biological	GO:00614	regulation of transcription from RNA polymerase II promoter in respo	0.18326	0.38182	0	0	1	11
GO:0061517 macrophage proliferation	1	0.18326	0.31	0.091	Biological	GO:00615	macrophage proliferation	0.18326	0.38182	0	0	1	11
GO:0070586 cell-cell adhesion involved in gastrulation	1	0.18326	0.31	0.091	Biological	GO:00705	cell-cell adhesion involved in gastrulation	0.18326	0.38182	0	0	1	11
GO:0070673 response to interleukin-18	1	0.18326	0.31	0.091	Biological	GO:00706	response to interleukin-18	0.18326	0.38182	0	0	1	11
GO:0071374 cellular response to parathyroid hormone stimulus	1	0.18326	0.31	0.091	Biological	GO:00713	cellular response to parathyroid hormone stimulus	0.18326	0.38182	0	0	1	11
GO:0071679 commissural neuron axon guidance	1	0.18326	0.31	0.091	Biological	GO:00716	commissural neuron axon guidance	0.18326	0.38182	0	0	1	11
GO:0072173 metanephric tubule morphogenesis	1	0.18326	0.31	0.091	Biological	GO:00721	metanephric tubule morphogenesis	0.18326	0.38182	0	0	1	11
GO:0072497 mesenchymal stem cell differentiation	1	0.18326	0.31	0.091	Biological	GO:00724	mesenchymal stem cell differentiation	0.18326	0.38182	0	0	1	11
GO:0072697 protein localization to cell cortex	1	0.18326	0.31	0.091	Biological	GO:00726	protein localization to cell cortex	0.18326	0.38182	0	0	1	11
GO:0090009 primitive streak formation	1	0.18326	0.31	0.091	Biological	GO:00900	primitive streak formation	0.18326	0.38182	0	0	1	11
GO:0090160 Golgi to lysosome transport	1	0.18326	0.31	0.091	Biological	GO:00901	Golgi to lysosome transport	0.18326	0.38182	0	0	1	11
GO:0090184 positive regulation of kidney development	1	0.18326	0.31	0.091	Biological	GO:00901	positive regulation of kidney development	0.18326	0.38182	0	0	1	11
GO:0090527 actin filament reorganization	1	0.18326	0.31	0.091	Biological	GO:00905	actin filament reorganization	0.18326	0.38182	0	0	1	11
GO:0097090 presynaptic membrane organization	1	0.18326	0.31	0.091	Biological	GO:00970	presynaptic membrane organization	0.18326	0.38182	0	0	1	11
GO:0097278 complement-dependent cytotoxicity	1	0.18326	0.31	0.091	Biological	GO:00972	complement-dependent cytotoxicity	0.18326	0.38182	0	0	1	11
GO:0097709 connective tissue replacement	1	0.18326	0.31	0.091	Biological	GO:00977	connective tissue replacement	0.18326	0.38182	0	0	1	11
GO:0099105 ion channel modulating, G protein-coupled receptor signali	1	0.18326	0.31	0.091	Biological	GO:00991	ion channel modulating, G protein-coupled receptor signaling pathwa	0.18326	0.38182	0	0	1	11
GO:0099566 regulation of postsynaptic cytosolic calcium ion concentrat	1	0.18326	0.31	0.091	Biological	GO:00995	regulation of postsynaptic cytosolic calcium ion concentration	0.18326	0.38182	0	0	1	11
GO:0106072 negative regulation of adenylate cyclase-activating G prote	1	0.18326	0.31	0.091	Biological	GO:01060	negative regulation of adenylate cyclase-activating G protein-coupled	0.18326	0.38182	0	0	1	11
GO:1902946 protein localization to early endosome	1	0.18326	0.31	0.091	Biological	GO:19029	protein localization to early endosome	0.18326	0.38182	0	0	1	11
GO:1902947 regulation of tau-protein kinase activity	1	0.18326	0.31	0.091	Biological	GO:19029	regulation of tau-protein kinase activity	0.18326	0.38182	0	0	1	11
GO:1903365 regulation of fear response	1	0.18326	0.31	0.091	Biological	GO:19033	regulation of fear response	0.18326	0.38182	0	0	1	11
GO:1903671 negative regulation of sprouting angiogenesis	1	0.18326	0.31	0.091	Biological	GO:19036	negative regulation of sprouting angiogenesis	0.18326	0.38182	0	0	1	11
GO:1904238 pericyte cell differentiation	1	0.18326	0.31	0.091	Biological	GO:19042	pericyte cell differentiation	0.18326	0.38182	0	0	1	11
GO:1905165 regulation of lysosomal protein catabolic process	1	0.18326	0.31	0.091	Biological	GO:19051	regulation of lysosomal protein catabolic process	0.18326	0.38182	0	0	1	11
GO:1905383 protein localization to presynapse	1	0.18326	0.31	0.091	Biological	GO:19053	protein localization to presynapse	0.18326	0.38182	0	0	1	11
GO:2000049 positive regulation of cell-cell adhesion mediated by cadhe	1	0.18326	0.31	0.091	Biological	GO:20000	positive regulation of cell-cell adhesion mediated by cadherin	0.18326	0.38182	0	0	1	11

GO:2000644 regulation of receptor catabolic process	1	0.18326	0.31	0.091	Biological	GO:20006	regulation of receptor catabolic process	0.18326	0.38182	0	0	1	11
GO:2001223 negative regulation of neuron migration	1	0.18326	0.31	0.091	Biological	GO:20012	negative regulation of neuron migration	0.18326	0.38182	0	0	1	11
GO:0030282 bone mineralization	4	0.18342	1.238	0.033	Biological	GO:00302	bone mineralization	0.18342	0.38183	0	0	4	122
GO:0031398 positive regulation of protein ubiquitination	4	0.18342	1.238	0.033	Biological	GO:00313	positive regulation of protein ubiquitination	0.18342	0.38183	0	0	4	122
GO:1901657 glycosyl compound metabolic process	3	0.18379	0.929	0.037	Biological	GO:19016	glycosyl compound metabolic process	0.18379	0.38243	0	0	3	81
GO:0051234 establishment of localization	95	0.18407	29.412	0.02	Biological	GO:00512	establishment of localization	0.18407	0.38286	0	0	95	4794
GO:0001974 blood vessel remodeling	2	0.1846	0.619	0.047	Biological	GO:00019	blood vessel remodeling	0.1846	0.383	0	0	2	43
GO:0003176 aortic valve development	2	0.1846	0.619	0.047	Biological	GO:00031	aortic valve development	0.1846	0.383	0	0	2	43
GO:0016266 O-glycan processing	2	0.1846	0.619	0.047	Biological	GO:00162	O-glycan processing	0.1846	0.383	0	0	2	43
GO:0033574 response to testosterone	2	0.1846	0.619	0.047	Biological	GO:00335	response to testosterone	0.1846	0.383	0	0	2	43
GO:0051294 establishment of spindle orientation	2	0.1846	0.619	0.047	Biological	GO:00512	establishment of spindle orientation	0.1846	0.383	0	0	2	43
GO:0071364 cellular response to epidermal growth factor stimulus	2	0.1846	0.619	0.047	Biological	GO:00713	cellular response to epidermal growth factor stimulus	0.1846	0.383	0	0	2	43
GO:0002376 immune system process	59	0.18585	18.266	0.02	Biological	GO:00023	immune system process	0.18585	0.38545	0	0	59	2888
GO:0002162 dystroglycan binding	1	0.18604	0.292	0.091	Molecular	GO:00021	dystroglycan binding	0.18604	0.39414	0	0	1	11
GO:0004954 prostanoid receptor activity	1	0.18604	0.292	0.091	Molecular	GO:00049	prostanoid receptor activity	0.18604	0.39414	0	0	1	11
GO:0016884 carbon-nitrogen ligase activity, with glutamine as amido-N-	1	0.18604	0.292	0.091	Molecular	GO:00168	carbon-nitrogen ligase activity, with glutamine as amido-N-donor	0.18604	0.39414	0	0	1	11
GO:0019107 myristoyltransferase activity	1	0.18604	0.292	0.091	Molecular	GO:00191	myristoyltransferase activity	0.18604	0.39414	0	0	1	11
GO:0030283 testosterone dehydrogenase [NAD(P)] activity	1	0.18604	0.292	0.091	Molecular	GO:00302	testosterone dehydrogenase [NAD(P)] activity	0.18604	0.39414	0	0	1	11
GO:0045294 alpha-catenin binding	1	0.18604	0.292	0.091	Molecular	GO:00452	alpha-catenin binding	0.18604	0.39414	0	0	1	11
GO:0048407 platelet-derived growth factor binding	1	0.18604	0.292	0.091	Molecular	GO:00484	platelet-derived growth factor binding	0.18604	0.39414	0	0	1	11
GO:0052739 phosphatidylserine 1-acylhydrolase activity	1	0.18604	0.292	0.091	Molecular	GO:00527	phosphatidylserine 1-acylhydrolase activity	0.18604	0.39414	0	0	1	11
GO:0070097 delta-catenin binding	1	0.18604	0.292	0.091	Molecular	GO:00700	delta-catenin binding	0.18604	0.39414	0	0	1	11
GO:0070410 co-SMAD binding	1	0.18604	0.292	0.091	Molecular	GO:00704	co-SMAD binding	0.18604	0.39414	0	0	1	11
GO:0042542 response to hydrogen peroxide	4	0.18708	1.238	0.033	Biological	GO:00425	response to hydrogen peroxide	0.18708	0.38783	0	0	4	123
GO:0003713 transcription coactivator activity	8	0.18783	2.339	0.027	Molecular	GO:00037	transcription coactivator activity	0.18783	0.39414	0	0	8	297
GO:0006898 receptor-mediated endocytosis	7	0.18791	2.167	0.027	Biological	GO:00068	receptor-mediated endocytosis	0.18791	0.38922	0	0	7	256
GO:0043122 regulation of I-kappaB kinase/NF-kappaB signaling	7	0.18791	2.167	0.027	Biological	GO:00431	regulation of I-kappaB kinase/NF-kappaB signaling	0.18791	0.38922	0	0	7	256
GO:0055006 cardiac cell development	3	0.18838	0.929	0.037	Biological	GO:00550	cardiac cell development	0.18838	0.39004	0	0	3	82
GO:1990837 sequence-specific double-stranded DNA binding	34	0.18862	9.942	0.022	Molecular	GO:19908	sequence-specific double-stranded DNA binding	0.18862	0.39414	0	0	34	1566
GO:0010506 regulation of autophagy	9	0.18868	2.786	0.026	Biological	GO:00105	regulation of autophagy	0.18868	0.39004	0	0	9	349
GO:0003690 double-stranded DNA binding	36	0.18871	10.526	0.022	Molecular	GO:00036	double-stranded DNA binding	0.18871	0.39414	0	0	36	1667
GO:0006643 membrane lipid metabolic process	6	0.18871	1.858	0.028	Biological	GO:00066	membrane lipid metabolic process	0.18871	0.39004	0	0	6	211
GO:0007626 locomotory behavior	6	0.18871	1.858	0.028	Biological	GO:00076	locomotory behavior	0.18871	0.39004	0	0	6	211
GO:0099111 microtubule-based transport	6	0.18871	1.858	0.028	Biological	GO:00991	microtubule-based transport	0.18871	0.39004	0	0	6	211
GO:0032269 negative regulation of cellular protein metabolic process	21	0.18877	6.502	0.022	Biological	GO:00322	negative regulation of cellular protein metabolic process	0.18877	0.39004	0	0	21	934
GO:0005245 voltage-gated calcium channel activity	2	0.18933	0.585	0.047	Molecular	GO:00052	voltage-gated calcium channel activity	0.18933	0.39414	0	0	2	43
GO:0008307 structural constituent of muscle	2	0.18933	0.585	0.047	Molecular	GO:00083	structural constituent of muscle	0.18933	0.39414	0	0	2	43
GO:0008375 acetylglucosaminyltransferase activity	2	0.18933	0.585	0.047	Molecular	GO:00083	acetylglucosaminyltransferase activity	0.18933	0.39414	0	0	2	43
GO:0016709 oxidoreductase activity, acting on paired donors, with incor	2	0.18933	0.585	0.047	Molecular	GO:00167	oxidoreductase activity, acting on paired donors, with incorporation o	0.18933	0.39414	0	0	2	43
GO:0043014 alpha-tubulin binding	2	0.18933	0.585	0.047	Molecular	GO:00430	alpha-tubulin binding	0.18933	0.39414	0	0	2	43
GO:1903561 extracellular vesicle	44	0.18971	13.056	0.021	Cellular C	GO:19035	extracellular vesicle	0.18971	0.40782	0	0	44	2133
GO:0004674 protein serine/threonine kinase activity	11	0.18995	3.216	0.025	Molecular	GO:00046	protein serine/threonine kinase activity	0.18995	0.39447	0	0	11	437
GO:0030426 growth cone	5	0.19032	1.484	0.03	Cellular C	GO:00304	growth cone	0.19032	0.40782	0	0	5	169
GO:0050878 regulation of body fluid levels	10	0.1904	3.096	0.025	Biological	GO:00508	regulation of body fluid levels	0.1904	0.39325	0	0	10	397
GO:0030136 clathrin-coated vesicle	6	0.19041	1.78	0.028	Cellular C	GO:00301	clathrin-coated vesicle	0.19041	0.40782	0	0	6	214
GO:0043230 extracellular organelle	44	0.19057	13.056	0.021	Cellular C	GO:00432	extracellular organelle	0.19057	0.40782	0	0	44	2134
GO:0065010 extracellular membrane-bounded organelle	44	0.19057	13.056	0.021	Cellular C	GO:00650	extracellular membrane-bounded organelle	0.19057	0.40782	0	0	44	2134
GO:0003206 cardiac chamber morphogenesis	4	0.19076	1.238	0.032	Biological	GO:00032	cardiac chamber morphogenesis	0.19076	0.39366	0	0	4	124
GO:0046660 female sex differentiation	4	0.19076	1.238	0.032	Biological	GO:00466	female sex differentiation	0.19076	0.39366	0	0	4	124
GO:0045124 regulation of bone resorption	2	0.1912	0.619	0.045	Biological	GO:00451	regulation of bone resorption	0.1912	0.39392	0	0	2	44
GO:0050691 regulation of defense response to virus by host	2	0.1912	0.619	0.045	Biological	GO:00506	regulation of defense response to virus by host	0.1912	0.39392	0	0	2	44
GO:1900026 positive regulation of substrate adhesion-dependent cell sp	2	0.1912	0.619	0.045	Biological	GO:19000	positive regulation of substrate adhesion-dependent cell spreading	0.1912	0.39392	0	0	2	44
GO:2000300 regulation of synaptic vesicle exocytosis	2	0.1912	0.619	0.045	Biological	GO:20003	regulation of synaptic vesicle exocytosis	0.1912	0.39392	0	0	2	44
GO:0044085 cellular component biogenesis	67	0.19236	20.743	0.02	Biological	GO:00440	cellular component biogenesis	0.19236	0.39443	0	0	67	3318
GO:0031016 pancreas development	3	0.193	0.929	0.036	Biological	GO:00310	pancreas development	0.193	0.39443	0	0	3	83
GO:0051153 regulation of striated muscle cell differentiation	3	0.193	0.929	0.036	Biological	GO:00511	regulation of striated muscle cell differentiation	0.193	0.39443	0	0	3	83
GO:0051668 localization within membrane	16	0.19301	4.954	0.023	Biological	GO:00516	localization within membrane	0.19301	0.39443	0	0	16	689
GO:0003205 cardiac chamber development	5	0.19309	1.548	0.03	Biological	GO:00032	cardiac chamber development	0.19309	0.39443	0	0	5	168
GO:0070374 positive regulation of ERK1 and ERK2 cascade	6	0.19425	1.858	0.028	Biological	GO:00703	positive regulation of ERK1 and ERK2 cascade	0.19425	0.39443	0	0	6	213
GO:0098878 neurotransmitter receptor complex	2	0.19449	0.593	0.044	Cellular C	GO:00988	neurotransmitter receptor complex	0.19449	0.41153	0	0	2	45
GO:0042405 nuclear inclusion body	1	0.19615	0.297	0.083	Cellular C	GO:00424	nuclear inclusion body	0.19615	0.41153	0	0	1	12
GO:0043256 laminin complex	1	0.19615	0.297	0.083	Cellular C	GO:00432	laminin complex	0.19615	0.41153	0	0	1	12
GO:0044754 autolysosome	1	0.19615	0.297	0.083	Cellular C	GO:00447	autolysosome	0.19615	0.41153	0	0	1	12
GO:1990454 L-type voltage-gated calcium channel complex	1	0.19615	0.297	0.083	Cellular C	GO:19904	L-type voltage-gated calcium channel complex	0.19615	0.41153	0	0	1	12
GO:1901888 regulation of cell junction assembly	6	0.19704	1.858	0.028	Biological	GO:19018	regulation of cell junction assembly	0.19704	0.39443	0	0	6	214
GO:0098655 cation transmembrane transport	22	0.19758	6.811	0.022	Biological	GO:00986	cation transmembrane transport	0.19758	0.39443	0	0	22	991
GO:0032024 positive regulation of insulin secretion	3	0.19765	0.929	0.036	Biological	GO:00320	positive regulation of insulin secretion	0.19765	0.39443	0	0	3	84
GO:0034340 response to type I interferon	3	0.19765	0.929	0.036	Biological	GO:00343	response to type I interferon	0.19765	0.39443	0	0	3	84
GO:0051279 regulation of release of sequestered calcium ion into cytos	3	0.19765	0.929	0.036	Biological	GO:00512	regulation of release of sequestered calcium ion into cytosol	0.19765	0.39443	0	0	3	84
GO:0060419 heart growth	3	0.19765	0.929	0.036	Biological	GO:00604	heart growth	0.19765	0.39443	0	0	3	84
GO:1901606 alpha-amino acid catabolic process	3	0.19765	0.929	0.036	Biological	GO:19016	alpha-amino acid catabolic process	0.19765	0.39443	0	0	3	84
GO:0001709 cell fate determination	2	0.19784	0.619	0.044	Biological	GO:00017	cell fate determination	0.19784	0.39443	0	0	2	45
GO:0021879 forebrain neuron differentiation	2	0.19784	0.619	0.044	Biological	GO:00218	forebrain neuron differentiation	0.19784	0.39443	0	0	2	45
GO:0030574 collagen catabolic process	2	0.19784	0.619	0.044	Biological	GO:00305	collagen catabolic process	0.19784	0.39443	0	0	2	45
GO:0035329 hippo signaling	2	0.19784	0.619	0.044	Biological	GO:00353	hippo signaling	0.19784	0.39443	0	0	2	45
GO:0042181 ketone biosynthetic process	2	0.19784	0.619	0.044	Biological	GO:00421	ketone biosynthetic process	0.19784	0.39443	0	0	2	45
GO:0046839 phospholipid dephosphorylation	2	0.19784	0.619	0.044	Biological	GO:00468	phospholipid dephosphorylation	0.19784	0.39443	0	0	2	45
GO:0048512 circadian behavior	2	0.19784	0.619	0.044	Biological	GO:00485	circadian behavior	0.19784	0.39443	0	0	2	45
GO:0051602 response to electrical stimulus	2	0.19784	0.619	0.044	Biological	GO:00516	response to electrical stimulus	0.19784	0.39443	0	0	2	45
GO:0071526 semaphorin-plexin signaling pathway	2	0.19784	0.619	0.044	Biological	GO:00715	semaphorin-plexin signaling pathway	0.19784	0.39443	0	0	2	45
GO:0071763 nuclear membrane organization	2	0.19784	0.619	0.044	Biological	GO:00717	nuclear membrane organization	0.19784	0.39443	0	0	2	45
GO:0098815 modulation of excitatory postsynaptic potential	2	0.19784	0.619	0.044	Biological	GO:00988	modulation of excitatory postsynaptic potential	0.19784	0.39443	0	0	2	45
GO:1902692 regulation of neuroblast proliferation	2	0.19784	0.619	0.044	Biological	GO:19026	regulation of neuroblast proliferation	0.19784	0.39443	0	0	2	45
GO:0051345 positive regulation of hydrolase activity	14	0.19791	4.334	0.024	Biological	GO:00513	positive regulation of hydrolase activity	0.19791	0.39443	0	0	14	594
GO:0045927 positive regulation of growth	7	0.19793	2.167	0.027	Biological	GO:00459	positive regulation of growth	0.19793	0.39443	0	0	7	260
GO:0002674 negative regulation of acute inflammatory response	1	0.19816	0.31	0.083	Biological	GO:00026	negative regulation of acute inflammatory response	0.19816	0.39443	0	0	1	12
GO:0003334 keratinocyte development	1	0.19816	0.31	0.083	Biological	GO:00033	keratinocyte development	0.19816	0.39443	0	0	1	12
GO:0003337 mesenchymal to epithelial transition involved in metanephr	1	0.19816	0.31	0.083	Biological	GO:00033	mesenchymal to epithelial transition involved in metanephros morpho	0.19816	0.39443	0	0	1	12
GO:0006534 cysteine metabolic process	1	0.19816	0.31	0.083	Biological	GO:00065	cysteine metabolic process	0.19816	0.39443	0	0	1	12
GO:0006563 L-serine metabolic process	1	0.19816	0.31	0.083	Biological	GO:00065	L-serine metabolic process	0.19816	0.39443	0	0	1	12
GO:0010713 negative regulation of collagen metabolic process	1	0.19816	0.31	0.083	Biological	GO:00107	negative regulation of collagen metabolic process	0.19816	0.39443	0	0	1	12
GO:0010826 negative regulation of centrosome duplication	1	0.19816	0.31	0.083	Biological	GO:00108	negative regulation of centrosome duplication	0.19816	0.39443	0	0	1	12
GO:0014733 regulation of skeletal muscle adaptation	1	0.19816	0.31	0.083	Biological	GO:00147	regulation of skeletal muscle adaptation	0.19816	0.39443	0	0	1	12
GO:0014842 regulation of skeletal muscle satellite cell proliferation	1	0.19816	0.31	0.083	Biological	GO:00148	regulation of skeletal muscle satellite cell proliferation	0.19816	0.39443	0	0	1	12
GO:0015808 L-alanine transport	1	0.19816	0.31	0.083	Biological	GO:00158	L-alanine transport	0.19816	0.39443	0	0	1	12
GO:0016554 cytidine to uridine editing	1	0.19816	0.31	0.083	Biological	GO:00165	cytidine to uridine editing	0.19816	0.39443	0	0	1	12
GO:0030656 regulation of vitamin metabolic process	1	0.19816	0.31	0.083	Biological	GO:00306	regulation of vitamin metabolic process	0.19816	0.39443	0	0	1	12
GO:0031650 regulation of heat generation	1	0.19816	0.31	0.083	Biological	GO:00316	regulation of heat generation	0.19816	0.39443	0	0	1	12
GO:0031953 negative regulation of protein autophosphorylation	1	0.19816	0.31	0.083	Biological	GO:00319	negative regulation of protein autophosphorylation	0.19816	0.39443	0	0	1	12
GO:0032351 negative regulation of hormone metabolic process	1	0.19816	0.31	0.083	Biological	GO:00323	negative regulation of hormone metabolic process	0.19816	0.39443	0	0	1	12
GO:0032490 detection of molecule of bacterial origin	1	0.19816	0.31	0.083	Biological	GO:00324	detection of molecule of bacterial origin	0.19816	0.39443	0	0	1	12
GO:0033327 Leydig cell differentiation	1	0.19816	0.31	0.083	Biological	GO:00333	Leydig cell differentiation	0.19816	0.39443	0	0	1	12
GO:0034370 triglyceride-rich lipoprotein particle remodeling	1	0.19816	0.31	0.083	Biological	GO:00343	triglyceride-rich lipoprotein particle remodeling	0.19816	0.39443	0	0	1	12
GO:0034372 very-low-density lipoprotein particle remodeling	1	0.19816	0.31	0.083	Biological	GO:00343	very-low-density lipoprotein particle remodeling	0.19816	0.39443	0	0	1	12
GO:0035112 genitalia morphogenesis	1	0.19816	0.31	0.083	Biological	GO:00351	genitalia morphogenesis	0.19816	0.39443	0	0	1	12
GO:0035810 positive regulation of urine volume	1	0.19816	0.31	0.083	Biological	GO:00358	positive regulation of urine volume	0.19816	0.39443	0	0	1	12
GO:0040034 regulation of development, heterochronic	1	0.19816	0.31	0.083	Biological	GO:00400	regulation of development, heterochronic	0.19816	0.39443	0	0	1	12
GO:0042416 dopamine biosynthetic process	1	0.19816	0.31	0.083	Biological	GO:00424	dopamine biosynthetic process	0.19816	0.39443	0	0	1	12
GO:0042711 maternal behavior	1	0.19816	0.31	0.083	Biological	GO:00427	maternal behavior	0.19816	0.39443	0	0	1	12
GO:0042989 sequestering of actin monomers	1	0.19816	0.31	0.083	Biological	GO:00429	sequestering of actin monomers	0.19816	0.39443	0	0	1	12
GO:0043312 neutrophil degranulation	1	0.19816	0.31	0.083	Biological	GO:00433	neutrophil degranulation	0.19816	0.39443	0	0	1	12
GO:0043476 pigment accumulation	1	0.19816	0.31	0.083	Biological	GO:00434	pigment accumulation	0.19816	0.39443	0	0	1	12
GO:0043482 cellular pigment accumulation	1	0.19816	0.31	0.083	Biological	GO:00434	cellular pigment accumulation	0.19816	0.39443	0	0	1	12
GO:0045161 neuronal ion channel clustering	1	0.19816	0.31	0.083	Biological	GO:00451	neuronal ion channel clustering	0.19816	0.39443	0	0	1	12
GO:0046549 retinal cone cell development	1	0.19816	0.31	0.083	Biological	GO:00465	retinal cone cell development	0.19816	0.39443	0	0	1	12
GO:0046606 negative regulation of centrosome cycle	1	0.19816	0.31	0.083	Biological	GO:00466	negative regulation of centrosome cycle	0.19816	0.39443	0	0	1	12
GO:0048103 somatic stem cell division	1	0.19816	0.31	0.083	Biological	GO:00481	somatic stem cell division	0.19816	0.39443	0	0	1	12
GO:0048251 elastic fiber assembly	1	0.19816	0.31	0.083	Biological	GO:00482	elastic fiber assembly	0.19816	0.39443	0	0	1	12
GO:0048521 negative regulation of behavior	1	0.19816	0.31	0.083	Biological	GO:00485	negative regulation of behavior	0.19816	0.39443	0	0	1	12
GO:0048563 post-embryonic animal organ morphogenesis	1	0.19816	0.31	0.083	Biological	GO:00485	post-embryonic animal organ morphogenesis	0.19816	0.39443	0	0	1	12
GO:0051798 positive regulation of hair follicle development	1	0.19816	0.31	0.083	Biological	GO:00517	positive regulation of hair follicle development	0.19816	0.39443	0	0	1	12
GO:0060379 cardiac muscle cell myoblast differentiation	1	0.19816	0.31	0.083	Biological	GO:00603	cardiac muscle cell myoblast differentiation	0.19816	0.39443	0	0	1	12
GO:0060442 branching involved in prostate gland morphogenesis	1	0.19816	0.31	0.083	Biological	GO:00604	branching involved in prostate gland morphogenesis	0.19816	0.39443	0	0	1	12
GO:0060601 lateral sprouting from an epithelium	1	0.19816	0.31	0.083	Biological	GO:00606	lateral sprouting from an epithelium	0.19816	0.39443	0	0	1	12
GO:0060746 parental behavior	1	0.19816	0.31	0.083	Biological	GO:00607	parental behavior	0.19816	0.39443	0	0	1	12
GO:0070189 kynurenine metabolic process	1	0.19816	0.31	0.083	Biological	GO:00701	kynurenine metabolic process	0.19816	0.39443	0	0	1	12
GO:0070327 thyroid hormone transport	1	0.19816	0.31	0.083	Biological	GO:00703	thyroid hormone transport	0.19816	0.39443	0	0	1	12
GO:0070778 L-aspartate transmembrane transport	1	0.19816	0.31	0.083	Biological	GO:00707	L-aspartate transmembrane transport	0.19816	0.39443	0	0	1	12
GO:0071372 cellular response to follicle-stimulating hormone stimulus	1	0.19816	0.31	0.083	Biological	GO:00713	cellular response to follicle-stimulating hormone stimulus	0.19816	0.39443	0	0	1	12
GO:0071394 cellular response to testosterone stimulus	1	0.19816	0.31	0.083	Biological	GO:00713	cellular response to testosterone stimulus	0.19816	0.39443	0	0	1	12
GO:0071609 chemokine (C-C motif) ligand 5 production	1	0.19816	0.31	0.083	Biological	GO:00716	chemokine (C-C motif) ligand 5 production	0.19816	0.39443	0	0	1	12
GO:0071649 regulation of chemokine (C-C motif) ligand 5 production	1	0.19816	0.31	0.083	Biological	GO:00716	regulation of chemokine (C-C motif) ligand 5 production	0.19816	0.39443	0	0	1	12
GO:0072178 nephric duct morphogenesis	1	0.19816	0.31	0.083	Biological	GO:00721	nephric duct morphogenesis	0.19816	0.39443	0	0	1	12
GO:0097084 vascular associated smooth muscle cell development	1	0.19816	0.31	0.083	Biological	GO:00970	vascular associated smooth muscle cell development	0.19816	0.39443	0	0	1	12
GO:0098911 regulation of ventricular cardiac muscle cell action potentia	1	0.19816	0.31	0.083	Biological	GO:00989	regulation of ventricular cardiac muscle cell action potential	0.19816	0.39443	0	0	1	12
GO:1900115 extracellular regulation of signal transduction	1	0.19816	0.31	0.083	Biological	GO:19001	extracellular regulation of signal transduction	0.19816	0.39443	0	0	1	12
GO:1900116 extracellular negative regulation of signal transduction	1	0.19816	0.31	0.083	Biological	GO:19001	extracellular negative regulation of signal transduction	0.19816	0.39443	0	0	1	12
GO:1901862 negative regulation of muscle tissue development	1	0.19816	0.31	0.083	Biological	GO:19018	negative regulation of muscle tissue development	0.19816	0.39443	0	0	1	12
GO:1903238 positive regulation of leukocyte tethering or rolling	1	0.19816	0.31	0.083	Biological	GO:19032	positive regulation of leukocyte tethering or rolling	0.19816	0.39443	0	0	1	12
GO:1903867 extraembryonic membrane development	1	0.19816	0.31	0.083	Biological	GO:19038	extraembryonic membrane development	0.19816	0.39443	0	0	1	12
GO:1904177 regulation of adipose tissue development	1	0.19816	0.31	0.083	Biological	GO:19041	regulation of adipose tissue development	0.19816	0.39443	0	0	1	12
GO:1905208 negative regulation of cardiocyte differentiation	1	0.19816	0.31	0.083	Biological	GO:19052	negative regulation of cardiocyte differentiation	0.19816	0.39443	0	0	1	12
GO:2000288 positive regulation of myoblast proliferation	1	0.19816	0.31	0.083	Biological	GO:20002	positive regulation of myoblast proliferation	0.19816	0.39443	0	0	1	12
GO:0043409 negative regulation of MAPK cascade	5	0.19943	1.548	0.029	Biological	GO:00434	negative regulation of MAPK cascade	0.19943	0.3968	0	0	5	170
GO:0044306 neuron projection terminus	4	0.20033	1.187	0.031	Cellular C	GO:00443	neuron projection terminus	0.20033	0.41865	0	0	4	128
GO:0030097 hemopoiesis	21	0.20048	6.502	0.022	Biological	GO:00300	hemopoiesis	0.20048	0.39873	0	0	21	943
GO:0004706 JUN kinase kinase kinase activity	1	0.20114	0.292	0.083	Molecular	GO:00047	JUN kinase kinase kinase activity	0.20114	0.40603	0	0	1	12
GO:0004859 phospholipase inhibitor activity	1	0.20114	0.292	0.083	Molecular	GO:00048	phospholipase inhibitor activity	0.20114	0.40603	0	0	1	12
GO:0004861 cyclin-dependent protein serine/threonine kinase inhibitor a	1	0.20114	0.292	0.083	Molecular	GO:00048	cyclin-dependent protein serine/threonine kinase inhibitor activity	0.20114	0.40603	0	0	1	12
GO:0005165 neurotrophin receptor binding	1	0.20114	0.292	0.083	Molecular	GO:00051	neurotrophin receptor binding	0.20114	0.40603	0	0	1	12
GO:0005542 folic acid binding	1	0.20114	0.292	0.083	Molecular	GO:00055	folic acid binding	0.20114	0.40603	0	0	1	12
GO:0016594 glycine binding	1	0.20114	0.292	0.083	Molecular	GO:00165	glycine binding	0.20114	0.40603	0	0	1	12

GO:0016857 racemase and epimerase activity, acting on carbohydrates	1	0.20114	0.292	0.083	Molecular	GO:0016	racemase and epimerase activity, acting on carbohydrates and deriva	0.20114	0.40603	0	0	1	12
GO:0017166 vinculin binding	1	0.20114	0.292	0.083	Molecular	GO:0017	vinculin binding	0.20114	0.40603	0	0	1	12
GO:0034452 dynactin binding	1	0.20114	0.292	0.083	Molecular	GO:0034	dynactin binding	0.20114	0.40603	0	0	1	12
GO:0035251 UDP-glucosyltransferase activity	1	0.20114	0.292	0.083	Molecular	GO:0035	UDP-glucosyltransferase activity	0.20114	0.40603	0	0	1	12
GO:0086080 protein binding involved in heterotypic cell-cell adhesion	1	0.20114	0.292	0.083	Molecular	GO:0086	protein binding involved in heterotypic cell-cell adhesion	0.20114	0.40603	0	0	1	12
GO:0089720 caspase binding	1	0.20114	0.292	0.083	Molecular	GO:0089	caspase binding	0.20114	0.40603	0	0	1	12
GO:0032411 positive regulation of transporter activity	4	0.20193	1.238	0.031	Biological	GO:0032	positive regulation of transporter activity	0.20193	0.40114	0	0	4	127
GO:0032874 positive regulation of stress-activated MAPK cascade	4	0.20193	1.238	0.031	Biological	GO:0032	positive regulation of stress-activated MAPK cascade	0.20193	0.40114	0	0	4	127
GO:0048675 axon extension	4	0.20193	1.238	0.031	Biological	GO:0048	axon extension	0.20193	0.40114	0	0	4	127
GO:0005198 structural molecule activity	19	0.20211	5.556	0.023	Molecular	GO:0005	structural molecule activity	0.20211	0.40704	0	0	19	830
GO:0010468 regulation of gene expression	96	0.20215	29.721	0.02	Biological	GO:0010	regulation of gene expression	0.20215	0.40141	0	0	96	4876
GO:0002028 regulation of sodium ion transport	3	0.20233	0.929	0.035	Biological	GO:0002	regulation of sodium ion transport	0.20233	0.40161	0	0	3	85
GO:0042180 cellular ketone metabolic process	6	0.20267	1.858	0.028	Biological	GO:0042	cellular ketone metabolic process	0.20267	0.40211	0	0	6	216
GO:0004842 ubiquitin-protein transferase activity	11	0.20346	3.216	0.025	Molecular	GO:0004	ubiquitin-protein transferase activity	0.20346	0.40882	0	0	11	444
GO:0001774 microglial cell activation	2	0.2045	0.619	0.043	Biological	GO:0001	microglial cell activation	0.2045	0.4043	0	0	2	46
GO:0021517 ventral spinal cord development	2	0.2045	0.619	0.043	Biological	GO:0021	ventral spinal cord development	0.2045	0.4043	0	0	2	46
GO:0046365 monosaccharide catabolic process	2	0.2045	0.619	0.043	Biological	GO:0046	monosaccharide catabolic process	0.2045	0.4043	0	0	2	46
GO:0046640 regulation of alpha-beta T cell proliferation	2	0.2045	0.619	0.043	Biological	GO:0046	regulation of alpha-beta T cell proliferation	0.2045	0.4043	0	0	2	46
GO:0055010 ventricular cardiac muscle tissue morphogenesis	2	0.2045	0.619	0.043	Biological	GO:0055	ventricular cardiac muscle tissue morphogenesis	0.2045	0.4043	0	0	2	46
GO:0098534 centriole assembly	2	0.2045	0.619	0.043	Biological	GO:0098	centriole assembly	0.2045	0.4043	0	0	2	46
GO:0099084 postsynaptic specialization organization	2	0.2045	0.619	0.043	Biological	GO:0099	postsynaptic specialization organization	0.2045	0.4043	0	0	2	46
GO:2000273 positive regulation of signaling receptor activity	2	0.2045	0.619	0.043	Biological	GO:2000	positive regulation of signaling receptor activity	0.2045	0.4043	0	0	2	46
GO:2000648 positive regulation of stem cell proliferation	2	0.2045	0.619	0.043	Biological	GO:2000	positive regulation of stem cell proliferation	0.2045	0.4043	0	0	2	46
GO:1901215 negative regulation of neuron death	6	0.2055	1.858	0.028	Biological	GO:1901	negative regulation of neuron death	0.2055	0.40612	0	0	6	217
GO:0031503 protein-containing complex localization	7	0.20558	2.167	0.027	Biological	GO:0031	protein-containing complex localization	0.20558	0.40612	0	0	7	263
GO:0071482 cellular response to light stimulus	4	0.2057	1.238	0.031	Biological	GO:0071	cellular response to light stimulus	0.2057	0.4062	0	0	4	128
GO:0002831 regulation of response to biotic stimulus	9	0.2059	2.786	0.025	Biological	GO:0002	regulation of response to biotic stimulus	0.2059	0.4063	0	0	9	357
GO:0043086 negative regulation of catalytic activity	15	0.20592	4.644	0.023	Biological	GO:0043	negative regulation of catalytic activity	0.20592	0.4063	0	0	15	648
GO:0042826 histone deacetylase binding	4	0.2061	1.17	0.032	Molecular	GO:0042	histone deacetylase binding	0.2061	0.41316	0	0	4	126
GO:0022604 regulation of cell morphogenesis	8	0.2064	2.477	0.026	Biological	GO:0022	regulation of cell morphogenesis	0.2064	0.40697	0	0	8	310
GO:0007015 actin filament organization	11	0.20642	3.406	0.024	Biological	GO:0007	actin filament organization	0.20642	0.40697	0	0	11	453
GO:0001523 retinoid metabolic process	3	0.20703	0.929	0.035	Biological	GO:0001	retinoid metabolic process	0.20703	0.40786	0	0	3	86
GO:0060395 SMAD protein signal transduction	3	0.20703	0.929	0.035	Biological	GO:0060	SMAD protein signal transduction	0.20703	0.40786	0	0	3	86
GO:0048786 presynaptic active zone	3	0.20717	0.89	0.034	Cellular C	GO:0048	presynaptic active zone	0.20717	0.43062	0	0	3	87
GO:0005891 voltage-gated calcium channel complex	2	0.20766	0.593	0.043	Cellular C	GO:0005	voltage-gated calcium channel complex	0.20766	0.43062	0	0	2	47
GO:0006629 lipid metabolic process	30	0.20892	9.288	0.021	Biological	GO:0006	lipid metabolic process	0.20892	0.40872	0	0	30	1407
GO:0030165 PDZ domain binding	3	0.20902	0.877	0.035	Molecular	GO:0030	PDZ domain binding	0.20902	0.41803	0	0	3	85
GO:0030216 keratinocyte differentiation	5	0.20907	1.548	0.029	Biological	GO:0030	keratinocyte differentiation	0.20907	0.40872	0	0	5	173
GO:0006022 aminoglycan metabolic process	4	0.20949	1.238	0.031	Biological	GO:0006	aminoglycan metabolic process	0.20949	0.40872	0	0	4	129
GO:0070304 positive regulation of stress-activated protein kinase signal	4	0.20949	1.238	0.031	Biological	GO:0070	positive regulation of stress-activated protein kinase signaling cascad	0.20949	0.40872	0	0	4	129
GO:0061629 RNA polymerase II-specific DNA-binding transcription fact	9	0.21015	2.632	0.025	Molecular	GO:0061	RNA polymerase II-specific DNA-binding transcription factor binding	0.21015	0.41934	0	0	9	353
GO:0035253 ciliary rootlet	1	0.21065	0.297	0.077	Cellular C	GO:0035	ciliary rootlet	0.21065	0.43345	0	0	1	13
GO:0099523 presynaptic cytosol	1	0.21065	0.297	0.077	Cellular C	GO:0099	presynaptic cytosol	0.21065	0.43345	0	0	1	13
GO:0050708 regulation of protein secretion	7	0.21074	2.167	0.026	Biological	GO:0050	regulation of protein secretion	0.21074	0.40872	0	0	7	265
GO:0007622 rhythmic behavior	2	0.21119	0.619	0.043	Biological	GO:0007	rhythmic behavior	0.21119	0.40872	0	0	2	47
GO:0046427 positive regulation of receptor signaling pathway via JAK-S	2	0.21119	0.619	0.043	Biological	GO:0046	positive regulation of receptor signaling pathway via JAK-STAT	0.21119	0.40872	0	0	2	47
GO:0061756 leukocyte adhesion to vascular endothelial cell	2	0.21119	0.619	0.043	Biological	GO:0061	leukocyte adhesion to vascular endothelial cell	0.21119	0.40872	0	0	2	47
GO:0070849 response to epidermal growth factor	2	0.21119	0.619	0.043	Biological	GO:0070	response to epidermal growth factor	0.21119	0.40872	0	0	2	47
GO:1901861 regulation of muscle tissue development	2	0.21119	0.619	0.043	Biological	GO:1901	regulation of muscle tissue development	0.21119	0.40872	0	0	2	47
GO:0006112 energy reserve metabolic process	3	0.21176	0.929	0.034	Biological	GO:0006	energy reserve metabolic process	0.21176	0.40872	0	0	3	87
GO:0010232 vascular transport	3	0.21176	0.929	0.034	Biological	GO:0010	vascular transport	0.21176	0.40872	0	0	3	87
GO:0014812 muscle cell migration	3	0.21176	0.929	0.034	Biological	GO:0014	muscle cell migration	0.21176	0.40872	0	0	3	87
GO:0150104 transport across blood-brain barrier	3	0.21176	0.929	0.034	Biological	GO:0150	transport across blood-brain barrier	0.21176	0.40872	0	0	3	87
GO:0000012 single strand break repair	1	0.21279	0.31	0.077	Biological	GO:0000	single strand break repair	0.21279	0.40872	0	0	1	13
GO:0000101 sulfur amino acid transport	1	0.21279	0.31	0.077	Biological	GO:0000	sulfur amino acid transport	0.21279	0.40872	0	0	1	13
GO:0001973 G protein-coupled adenosine receptor signaling pathway	1	0.21279	0.31	0.077	Biological	GO:0001	G protein-coupled adenosine receptor signaling pathway	0.21279	0.40872	0	0	1	13
GO:0002087 regulation of respiratory gaseous exchange by nervous sys	1	0.21279	0.31	0.077	Biological	GO:0002	regulation of respiratory gaseous exchange by nervous system proce	0.21279	0.40872	0	0	1	13
GO:0002863 positive regulation of inflammatory response to antigenic s	1	0.21279	0.31	0.077	Biological	GO:0002	positive regulation of inflammatory response to antigenic stimulus	0.21279	0.40872	0	0	1	13
GO:0006586 indolalkylamine metabolic process	1	0.21279	0.31	0.077	Biological	GO:0006	indolalkylamine metabolic process	0.21279	0.40872	0	0	1	13
GO:0007638 mechanosensory behavior	1	0.21279	0.31	0.077	Biological	GO:0007	mechanosensory behavior	0.21279	0.40872	0	0	1	13
GO:0014854 response to inactivity	1	0.21279	0.31	0.077	Biological	GO:0014	response to inactivity	0.21279	0.40872	0	0	1	13
GO:0015801 aromatic amino acid transport	1	0.21279	0.31	0.077	Biological	GO:0015	aromatic amino acid transport	0.21279	0.40872	0	0	1	13
GO:0015824 proline transport	1	0.21279	0.31	0.077	Biological	GO:0015	proline transport	0.21279	0.40872	0	0	1	13
GO:0016024 CDP-diacylglycerol biosynthetic process	1	0.21279	0.31	0.077	Biological	GO:0016	CDP-diacylglycerol biosynthetic process	0.21279	0.40872	0	0	1	13
GO:0018095 protein polyglutamylation	1	0.21279	0.31	0.077	Biological	GO:0018	protein polyglutamylation	0.21279	0.40872	0	0	1	13
GO:0019885 antigen processing and presentation of endogenous peptic	1	0.21279	0.31	0.077	Biological	GO:0019	antigen processing and presentation of endogenous peptide antigen	0.21279	0.40872	0	0	1	13
GO:0021514 ventral spinal cord interneuron differentiation	1	0.21279	0.31	0.077	Biological	GO:0021	ventral spinal cord interneuron differentiation	0.21279	0.40872	0	0	1	13
GO:0032341 aldosterone metabolic process	1	0.21279	0.31	0.077	Biological	GO:0032	aldosterone metabolic process	0.21279	0.40872	0	0	1	13
GO:0032530 regulation of microvillus organization	1	0.21279	0.31	0.077	Biological	GO:0032	regulation of microvillus organization	0.21279	0.40872	0	0	1	13
GO:0033083 regulation of immature T cell proliferation	1	0.21279	0.31	0.077	Biological	GO:0033	regulation of immature T cell proliferation	0.21279	0.40872	0	0	1	13
GO:0033084 regulation of immature T cell proliferation in thymus	1	0.21279	0.31	0.077	Biological	GO:0033	regulation of immature T cell proliferation in thymus	0.21279	0.40872	0	0	1	13
GO:0033148 positive regulation of intracellular estrogen receptor signali	1	0.21279	0.31	0.077	Biological	GO:0033	positive regulation of intracellular estrogen receptor signaling pathwa	0.21279	0.40872	0	0	1	13
GO:0034333 adherens junction assembly	1	0.21279	0.31	0.077	Biological	GO:0034	adherens junction assembly	0.21279	0.40872	0	0	1	13
GO:0035588 G protein-coupled purinergic receptor signaling pathway	1	0.21279	0.31	0.077	Biological	GO:0035	G protein-coupled purinergic receptor signaling pathway	0.21279	0.40872	0	0	1	13
GO:0035815 positive regulation of renal sodium excretion	1	0.21279	0.31	0.077	Biological	GO:0035	positive regulation of renal sodium excretion	0.21279	0.40872	0	0	1	13
GO:0035865 cellular response to potassium ion	1	0.21279	0.31	0.077	Biological	GO:0035	cellular response to potassium ion	0.21279	0.40872	0	0	1	13
GO:0036006 cellular response to macrophage colony-stimulating factor	1	0.21279	0.31	0.077	Biological	GO:0036	cellular response to macrophage colony-stimulating factor stimulus	0.21279	0.40872	0	0	1	13
GO:0040015 negative regulation of multicellular organism growth	1	0.21279	0.31	0.077	Biological	GO:0040	negative regulation of multicellular organism growth	0.21279	0.40872	0	0	1	13
GO:0042363 fat-soluble vitamin catabolic process	1	0.21279	0.31	0.077	Biological	GO:0042	fat-soluble vitamin catabolic process	0.21279	0.40872	0	0	1	13
GO:0042670 retinal cone cell differentiation	1	0.21279	0.31	0.077	Biological	GO:0042	retinal cone cell differentiation	0.21279	0.40872	0	0	1	13
GO:0044650 adhesion of symbiont to host cell	1	0.21279	0.31	0.077	Biological	GO:0044	adhesion of symbiont to host cell	0.21279	0.40872	0	0	1	13
GO:0048742 regulation of skeletal muscle fiber development	1	0.21279	0.31	0.077	Biological	GO:0048	regulation of skeletal muscle fiber development	0.21279	0.40872	0	0	1	13
GO:0050655 dermatan sulfate proteoglycan metabolic process	1	0.21279	0.31	0.077	Biological	GO:0050	dermatan sulfate proteoglycan metabolic process	0.21279	0.40872	0	0	1	13
GO:0051451 myoblast migration	1	0.21279	0.31	0.077	Biological	GO:0051	myoblast migration	0.21279	0.40872	0	0	1	13
GO:0060287 epithelial cilium movement involved in determination of left/	1	0.21279	0.31	0.077	Biological	GO:0060	epithelial cilium movement involved in determination of left/right asym	0.21279	0.40872	0	0	1	13
GO:0061430 bone trabecula morphogenesis	1	0.21279	0.31	0.077	Biological	GO:0061	bone trabecula morphogenesis	0.21279	0.40872	0	0	1	13
GO:0070863 positive regulation of protein exit from endoplasmic reticulu	1	0.21279	0.31	0.077	Biological	GO:0070	positive regulation of protein exit from endoplasmic reticulum	0.21279	0.40872	0	0	1	13
GO:0071801 regulation of podosome assembly	1	0.21279	0.31	0.077	Biological	GO:0071	regulation of podosome assembly	0.21279	0.40872	0	0	1	13
GO:0072182 regulation of nephron tubule epithelial cell differentiation	1	0.21279	0.31	0.077	Biological	GO:0072	regulation of nephron tubule epithelial cell differentiation	0.21279	0.40872	0	0	1	13
GO:0072537 fibroblast activation	1	0.21279	0.31	0.077	Biological	GO:0072	fibroblast activation	0.21279	0.40872	0	0	1	13
GO:0075509 endocytosis involved in viral entry into host cell	1	0.21279	0.31	0.077	Biological	GO:0075	endocytosis involved in viral entry into host cell	0.21279	0.40872	0	0	1	13
GO:0090205 positive regulation of cholesterol metabolic process	1	0.21279	0.31	0.077	Biological	GO:0090	positive regulation of cholesterol metabolic process	0.21279	0.40872	0	0	1	13
GO:0090235 regulation of metaphase plate congression	1	0.21279	0.31	0.077	Biological	GO:0090	regulation of metaphase plate congression	0.21279	0.40872	0	0	1	13
GO:0098792 xenophagy	1	0.21279	0.31	0.077	Biological	GO:0098	xenophagy	0.21279	0.40872	0	0	1	13
GO:0098935 dendritic transport	1	0.21279	0.31	0.077	Biological	GO:0098	dendritic transport	0.21279	0.40872	0	0	1	13
GO:0099550 trans-synaptic signaling, modulating synaptic transmission	1	0.21279	0.31	0.077	Biological	GO:0099	trans-synaptic signaling, modulating synaptic transmission	0.21279	0.40872	0	0	1	13
GO:0140042 lipid droplet formation	1	0.21279	0.31	0.077	Biological	GO:0140	lipid droplet formation	0.21279	0.40872	0	0	1	13
GO:1902337 regulation of apoptotic process involved in morphogenesis	1	0.21279	0.31	0.077	Biological	GO:1902	regulation of apoptotic process involved in morphogenesis	0.21279	0.40872	0	0	1	13
GO:1902931 negative regulation of alcohol biosynthetic process	1	0.21279	0.31	0.077	Biological	GO:1902	negative regulation of alcohol biosynthetic process	0.21279	0.40872	0	0	1	13
GO:1904350 regulation of protein catabolic process in the vacuole	1	0.21279	0.31	0.077	Biological	GO:1904	regulation of protein catabolic process in the vacuole	0.21279	0.40872	0	0	1	13
GO:1904748 regulation of apoptotic process involved in development	1	0.21279	0.31	0.077	Biological	GO:1904	regulation of apoptotic process involved in development	0.21279	0.40872	0	0	1	13
GO:1905668 positive regulation of protein localization to endosome	1	0.21279	0.31	0.077	Biological	GO:1905	positive regulation of protein localization to endosome	0.21279	0.40872	0	0	1	13
GO:1905939 regulation of gonad development	1	0.21279	0.31	0.077	Biological	GO:1905	regulation of gonad development	0.21279	0.40872	0	0	1	13
GO:2001053 regulation of mesenchymal cell apoptotic process	1	0.21279	0.31	0.077	Biological	GO:2001	regulation of mesenchymal cell apoptotic process	0.21279	0.40872	0	0	1	13
GO:0000323 lytic vacuole	17	0.21286	5.045	0.022	Cellular C	GO:0000	lytic vacuole	0.21286	0.43466	0	0	17	760
GO:0005764 lysosome	17	0.21286	5.045	0.022	Cellular C	GO:0005	lysosome	0.21286	0.43466	0	0	17	760
GO:0071621 granulocyte chemotaxis	4	0.2133	1.238	0.031	Biological	GO:0071	granulocyte chemotaxis	0.2133	0.40954	0	0	4	130
GO:0070062 extracellular exosome	43	0.21537	12.76	0.02	Cellular C	GO:0070	extracellular exosome	0.21537	0.43705	0	0	43	2109
GO:0045935 positive regulation of nucleobase-containing compound me	42	0.21553	13.003	0.021	Biological	GO:0045	positive regulation of nucleobase-containing compound metabolic pro	0.21553	0.41361	0	0	42	2034
GO:0009267 cellular response to starvation	5	0.21558	1.548	0.029	Biological	GO:0009	cellular response to starvation	0.21558	0.41361	0	0	5	175
GO:0043197 dendritic spine	5	0.21567	1.484	0.028	Cellular C	GO:0043	dendritic spine	0.21567	0.43705	0	0	5	177
GO:0003906 DNA-(apurinic or apyrimidinic site) endonuclease activity	1	0.21596	0.292	0.077	Molecular	GO:0003	DNA-(apurinic or apyrimidinic site) endonuclease activity	0.21596	0.42217	0	0	1	13
GO:0004000 adenosine deaminase activity	1	0.21596	0.292	0.077	Molecular	GO:0004	adenosine deaminase activity	0.21596	0.42217	0	0	1	13
GO:0031432 titin binding	1	0.21596	0.292	0.077	Molecular	GO:0031	titin binding	0.21596	0.42217	0	0	1	13
GO:0031543 peptidyl-proline dioxygenase activity	1	0.21596	0.292	0.077	Molecular	GO:0031	peptidyl-proline dioxygenase activity	0.21596	0.42217	0	0	1	13
GO:0034236 protein kinase A catalytic subunit binding	1	0.21596	0.292	0.077	Molecular	GO:0034	protein kinase A catalytic subunit binding	0.21596	0.42217	0	0	1	13
GO:0050998 nitric-oxide synthase binding	1	0.21596	0.292	0.077	Molecular	GO:0050	nitric-oxide synthase binding	0.21596	0.42217	0	0	1	13
GO:0070679 inositol 1,4,5 trisphosphate binding	1	0.21596	0.292	0.077	Molecular	GO:0070	inositol 1,4,5 trisphosphate binding	0.21596	0.42217	0	0	1	13
GO:0097157 pre-mRNA intronic binding	1	0.21596	0.292	0.077	Molecular	GO:0097	pre-mRNA intronic binding	0.21596	0.42217	0	0	1	13
GO:0001222 transcription corepressor binding	2	0.21645	0.585	0.043	Molecular	GO:0001	transcription corepressor binding	0.21645	0.42217	0	0	2	47
GO:0004180 carboxypeptidase activity	2	0.21645	0.585	0.043	Molecular	GO:0004	carboxypeptidase activity	0.21645	0.42217	0	0	2	47
GO:0035023 regulation of Rho protein signal transduction	3	0.21651	0.929	0.034	Biological	GO:0035	regulation of Rho protein signal transduction	0.21651	0.41508	0	0	3	88
GO:0060191 regulation of lipase activity	3	0.21651	0.929	0.034	Biological	GO:0060	regulation of lipase activity	0.21651	0.41508	0	0	3	88
GO:0006810 transport	91	0.21686	28.173	0.02	Biological	GO:0006	transport	0.21686	0.41558	0	0	91	4630
GO:0018198 peptidyl-cysteine modification	2	0.21789	0.619	0.042	Biological	GO:0018	peptidyl-cysteine modification	0.21789	0.41628	0	0	2	48
GO:0035305 negative regulation of dephosphorylation	2	0.21789	0.619	0.042	Biological	GO:0035	negative regulation of dephosphorylation	0.21789	0.41628	0	0	2	48
GO:0035307 positive regulation of protein dephosphorylation	2	0.21789	0.619	0.042	Biological	GO:0035	positive regulation of protein dephosphorylation	0.21789	0.41628	0	0	2	48
GO:0042491 inner ear auditory receptor cell differentiation	2	0.21789	0.619	0.042	Biological	GO:0042	inner ear auditory receptor cell differentiation	0.21789	0.41628	0	0	2	48
GO:0042789 mRNA transcription by RNA polymerase II	2	0.21789	0.619	0.042	Biological	GO:0042	mRNA transcription by RNA polymerase II	0.21789	0.41628	0	0	2	48
GO:0046596 regulation of viral entry into host cell	2	0.21789	0.619	0.042	Biological	GO:0046	regulation of viral entry into host cell	0.21789	0.41628	0	0	2	48
GO:0048806 genitalia development	2	0.21789	0.619	0.042	Biological	GO:0048	genitalia development	0.21789	0.41628	0	0	2	48
GO:0071985 multivesicular body sorting pathway	2	0.21789	0.619	0.042	Biological	GO:0071	multivesicular body sorting pathway	0.21789	0.41628	0	0	2	48
GO:0019900 kinase binding	18	0.2186	5.263	0.023	Molecular	GO:0019	kinase binding	0.2186	0.42542	0	0	18	792
GO:0046631 alpha-beta T cell activation	5	0.21886	1.548	0.028	Biological	GO:0046	alpha-beta T cell activation	0.21886	0.41797	0	0	5	176
GO:1903706 regulation of hemopoiesis	10	0.21908	3.096	0.024	Biological	GO:1903	regulation of hemopoiesis	0.21908	0.41822	0	0	10	411
GO:0051248 negative regulation of protein metabolic process	22	0.2199	6.811	0.022	Biological	GO:0051	negative regulation of protein metabolic process	0.2199	0.41963	0	0	22	1008
GO:0043467 regulation of generation of precursor metabolites and ener	4	0.22097	1.238	0.03	Biological	GO:0043	regulation of generation of precursor metabolites and energy	0.22097	0.42151	0	0	4	132
GO:0002088 lens development in camera-type eye	3	0.22129	0.929	0.034	Biological	GO:0002	lens development in camera-type eye	0.22129	0.4218	0	0	3	89
GO:0016101 diterpenoid metabolic process	3	0.22129	0.929	0.034	Biological	GO:0016	diterpenoid metabolic process	0.22129	0.4218	0	0	3	89
GO:0051960 regulation of nervous system development	11	0.2222	3.406	0.024	Biological	GO:0051	regulation of nervous system development	0.2222	0.42337	0	0	11	461
GO:0009306 protein secretion	9	0.22374	2.786	0.025	Biological	GO:0009	protein secretion	0.22374	0.4236	0	0	9	365
GO:0002686 negative regulation of leukocyte migration	2	0.22462	0.619	0.041	Biological	GO:0002	negative regulation of leukocyte migration	0.22462	0.4236	0	0	2	49
GO:0010862 positive regulation of pathway-restricted SMAD protein pho	2	0.22462	0.619	0.041	Biological	GO:0010	positive regulation of pathway-restricted SMAD protein phosphorylati	0.22462	0.4236	0	0	2	49
GO:0021795 cerebral cortex cell migration	2	0.22462	0.619	0.041	Biological	GO:0021	cerebral cortex cell migration	0.22462	0.4236	0	0	2	49
GO:0030857 negative regulation of epithelial cell differentiation	2	0.22462	0.619	0.041	Biological	GO:0030	negative regulation of epithelial cell differentiation	0.22462	0.4236	0	0	2	49
GO:0034198 cellular response to amino acid starvation	2	0.22462	0.619	0.041	Biological	GO:0034	cellular response to amino acid starvation	0.22462	0.4236	0	0	2	49
GO:0043457 regulation of cellular respiration	2	0.22462	0.619	0.041	Biological	GO:0043	regulation of cellular respiration	0.22462	0.4236	0	0	2	49
GO:0046633 alpha-beta T cell proliferation	2	0.22462	0.619	0.041	Biological	GO:0046	alpha-beta T cell proliferation	0.22462	0.4236	0	0	2	49
GO:0048538 thymus development	2	0.22462	0.619	0.041	Biological	GO:0048	thymus development	0.22462	0.4236	0	0	2	49
GO:0051180 vitamin transport	2	0.22462	0.619	0.041	Biological	GO:0051	vitamin transport	0.22462	0.4236	0	0	2	49
GO:0045598 regulation of fat cell differentiation	4	0.22483	1.238	0.03	Biological	GO:0045	regulation of fat cell differentiation	0.22483	0.4236	0	0	4	133
GO:0051702 biological process involved in interaction with symbiont	4	0.22483	1.238	0.03	Biological	GO:0051	biological process involved in interaction with symbiont	0.22483	0.4236	0	0	4	133

GO:0005940 septin ring	1	0.22489	0.297	0.071	Cellular C	GO:00059	septin ring	0.22489	0.43967	0	0	1	14
GO:0031089 platelet dense granule lumen	1	0.22489	0.297	0.071	Cellular C	GO:00310	platelet dense granule lumen	0.22489	0.43967	0	0	1	14
GO:0031105 septin complex	1	0.22489	0.297	0.071	Cellular C	GO:00311	septin complex	0.22489	0.43967	0	0	1	14
GO:0031254 cell trailing edge	1	0.22489	0.297	0.071	Cellular C	GO:00312	cell trailing edge	0.22489	0.43967	0	0	1	14
GO:0042627 chylomicron	1	0.22489	0.297	0.071	Cellular C	GO:00426	chylomicron	0.22489	0.43967	0	0	1	14
GO:0098574 cytoplasmic side of lysosomal membrane	1	0.22489	0.297	0.071	Cellular C	GO:00985	cytoplasmic side of lysosomal membrane	0.22489	0.43967	0	0	1	14
GO:0099092 postsynaptic density, intracellular component	1	0.22489	0.297	0.071	Cellular C	GO:00990	postsynaptic density, intracellular component	0.22489	0.43967	0	0	1	14
GO:0140672 ATAC complex	1	0.22489	0.297	0.071	Cellular C	GO:01406	ATAC complex	0.22489	0.43967	0	0	1	14
GO:1990531 phospholipid-translocating ATPase complex	1	0.22489	0.297	0.071	Cellular C	GO:19905	phospholipid-translocating ATPase complex	0.22489	0.43967	0	0	1	14
GO:0032580 Golgi cisterna membrane	3	0.226	0.89	0.033	Cellular C	GO:00325	Golgi cisterna membrane	0.226	0.43967	0	0	3	91
GO:0120111 neuron projection cytoplasm	3	0.226	0.89	0.033	Cellular C	GO:01201	neuron projection cytoplasm	0.226	0.43967	0	0	3	91
GO:0035592 establishment of protein localization to extracellular region	9	0.22601	2.786	0.025	Biological	GO:00355	establishment of protein localization to extracellular region	0.22601	0.4236	0	0	9	366
GO:0032868 response to insulin	7	0.22652	2.167	0.026	Biological	GO:00328	response to insulin	0.22652	0.4236	0	0	7	271
GO:0002524 hypersensitivity	1	0.22715	0.31	0.071	Biological	GO:00025	hypersensitivity	0.22715	0.4236	0	0	1	14
GO:0003207 cardiac chamber formation	1	0.22715	0.31	0.071	Biological	GO:00032	cardiac chamber formation	0.22715	0.4236	0	0	1	14
GO:0003222 ventricular trabecula myocardium morphogenesis	1	0.22715	0.31	0.071	Biological	GO:00032	ventricular trabecula myocardium morphogenesis	0.22715	0.4236	0	0	1	14
GO:0007175 negative regulation of epidermal growth factor-activated re	1	0.22715	0.31	0.071	Biological	GO:00071	negative regulation of epidermal growth factor-activated receptor acti	0.22715	0.4236	0	0	1	14
GO:0008212 mineralocorticoid metabolic process	1	0.22715	0.31	0.071	Biological	GO:00082	mineralocorticoid metabolic process	0.22715	0.4236	0	0	1	14
GO:0010715 regulation of extracellular matrix disassembly	1	0.22715	0.31	0.071	Biological	GO:00107	regulation of extracellular matrix disassembly	0.22715	0.4236	0	0	1	14
GO:0010838 positive regulation of keratinocyte proliferation	1	0.22715	0.31	0.071	Biological	GO:00108	positive regulation of keratinocyte proliferation	0.22715	0.4236	0	0	1	14
GO:0014841 skeletal muscle satellite cell proliferation	1	0.22715	0.31	0.071	Biological	GO:00148	skeletal muscle satellite cell proliferation	0.22715	0.4236	0	0	1	14
GO:0014874 response to stimulus involved in regulation of muscle adap	1	0.22715	0.31	0.071	Biological	GO:00148	response to stimulus involved in regulation of muscle adaptation	0.22715	0.4236	0	0	1	14
GO:0018158 protein oxidation	1	0.22715	0.31	0.071	Biological	GO:00181	protein oxidation	0.22715	0.4236	0	0	1	14
GO:0030949 positive regulation of vascular endothelial growth factor rec	1	0.22715	0.31	0.071	Biological	GO:00309	positive regulation of vascular endothelial growth factor receptor sign	0.22715	0.4236	0	0	1	14
GO:0032494 response to peptidoglycan	1	0.22715	0.31	0.071	Biological	GO:00324	response to peptidoglycan	0.22715	0.4236	0	0	1	14
GO:0033079 immature T cell proliferation	1	0.22715	0.31	0.071	Biological	GO:00330	immature T cell proliferation	0.22715	0.4236	0	0	1	14
GO:0033080 immature T cell proliferation in thymus	1	0.22715	0.31	0.071	Biological	GO:00330	immature T cell proliferation in thymus	0.22715	0.4236	0	0	1	14
GO:0033145 positive regulation of intracellular steroid hormone receptor	1	0.22715	0.31	0.071	Biological	GO:00331	positive regulation of intracellular steroid hormone receptor signaling	0.22715	0.4236	0	0	1	14
GO:0033700 phospholipid efflux	1	0.22715	0.31	0.071	Biological	GO:00337	phospholipid efflux	0.22715	0.4236	0	0	1	14
GO:0034111 negative regulation of homotypic cell-cell adhesion	1	0.22715	0.31	0.071	Biological	GO:00341	negative regulation of homotypic cell-cell adhesion	0.22715	0.4236	0	0	1	14
GO:0034380 high-density lipoprotein particle assembly	1	0.22715	0.31	0.071	Biological	GO:00343	high-density lipoprotein particle assembly	0.22715	0.4236	0	0	1	14
GO:0034616 response to laminar fluid shear stress	1	0.22715	0.31	0.071	Biological	GO:00346	response to laminar fluid shear stress	0.22715	0.4236	0	0	1	14
GO:0035589 G protein-coupled purinergic nucleotide receptor signaling	1	0.22715	0.31	0.071	Biological	GO:00355	G protein-coupled purinergic nucleotide receptor signaling pathway	0.22715	0.4236	0	0	1	14
GO:0035845 photoreceptor cell outer segment organization	1	0.22715	0.31	0.071	Biological	GO:00358	photoreceptor cell outer segment organization	0.22715	0.4236	0	0	1	14
GO:0042117 monocyte activation	1	0.22715	0.31	0.071	Biological	GO:00421	monocyte activation	0.22715	0.4236	0	0	1	14
GO:0042754 negative regulation of circadian rhythm	1	0.22715	0.31	0.071	Biological	GO:00427	negative regulation of circadian rhythm	0.22715	0.4236	0	0	1	14
GO:0043101 purine-containing compound salvage	1	0.22715	0.31	0.071	Biological	GO:00431	purine-containing compound salvage	0.22715	0.4236	0	0	1	14
GO:0046341 CDP-diacylglycerol metabolic process	1	0.22715	0.31	0.071	Biological	GO:00463	CDP-diacylglycerol metabolic process	0.22715	0.4236	0	0	1	14
GO:0046655 folic acid metabolic process	1	0.22715	0.31	0.071	Biological	GO:00466	folic acid metabolic process	0.22715	0.4236	0	0	1	14
GO:0048715 negative regulation of oligodendrocyte differentiation	1	0.22715	0.31	0.071	Biological	GO:00487	negative regulation of oligodendrocyte differentiation	0.22715	0.4236	0	0	1	14
GO:0051764 actin crosslink formation	1	0.22715	0.31	0.071	Biological	GO:00517	actin crosslink formation	0.22715	0.4236	0	0	1	14
GO:0060100 positive regulation of phagocytosis, engulfment	1	0.22715	0.31	0.071	Biological	GO:00601	positive regulation of phagocytosis, engulfment	0.22715	0.4236	0	0	1	14
GO:0060221 retinal rod cell differentiation	1	0.22715	0.31	0.071	Biological	GO:00602	retinal rod cell differentiation	0.22715	0.4236	0	0	1	14
GO:0060712 spongiotrophoblast layer development	1	0.22715	0.31	0.071	Biological	GO:00607	spongiotrophoblast layer development	0.22715	0.4236	0	0	1	14
GO:0071639 positive regulation of monocyte chemotactic protein-1 prod	1	0.22715	0.31	0.071	Biological	GO:00716	positive regulation of monocyte chemotactic protein-1 production	0.22715	0.4236	0	0	1	14
GO:0072540 T-helper 17 cell lineage commitment	1	0.22715	0.31	0.071	Biological	GO:00725	T-helper 17 cell lineage commitment	0.22715	0.4236	0	0	1	14
GO:0097475 motor neuron migration	1	0.22715	0.31	0.071	Biological	GO:00974	motor neuron migration	0.22715	0.4236	0	0	1	14
GO:0098693 regulation of synaptic vesicle cycle	1	0.22715	0.31	0.071	Biological	GO:00986	regulation of synaptic vesicle cycle	0.22715	0.4236	0	0	1	14
GO:1901160 primary amino compound metabolic process	1	0.22715	0.31	0.071	Biological	GO:19011	primary amino compound metabolic process	0.22715	0.4236	0	0	1	14
GO:1902033 regulation of hematopoietic stem cell proliferation	1	0.22715	0.31	0.071	Biological	GO:19020	regulation of hematopoietic stem cell proliferation	0.22715	0.4236	0	0	1	14
GO:1902043 positive regulation of extrinsic apoptotic signaling pathway	1	0.22715	0.31	0.071	Biological	GO:19020	positive regulation of extrinsic apoptotic signaling pathway via death c	0.22715	0.4236	0	0	1	14
GO:1902287 semaphorin-plexin signaling pathway involved in axon guid	1	0.22715	0.31	0.071	Biological	GO:19022	semaphorin-plexin signaling pathway involved in axon guidance	0.22715	0.4236	0	0	1	14
GO:1902514 regulation of calcium ion transmembrane transport via high	1	0.22715	0.31	0.071	Biological	GO:19025	regulation of calcium ion transmembrane transport via high voltage-g	0.22715	0.4236	0	0	1	14
GO:1905155 positive regulation of membrane invagination	1	0.22715	0.31	0.071	Biological	GO:19051	positive regulation of membrane invagination	0.22715	0.4236	0	0	1	14
GO:1905666 regulation of protein localization to endosome	1	0.22715	0.31	0.071	Biological	GO:19056	regulation of protein localization to endosome	0.22715	0.4236	0	0	1	14
GO:2001140 positive regulation of phospholipid transport	1	0.22715	0.31	0.071	Biological	GO:20011	positive regulation of phospholipid transport	0.22715	0.4236	0	0	1	14
GO:0032970 regulation of actin filament-based process	10	0.22757	3.096	0.024	Biological	GO:00329	regulation of actin filament-based process	0.22757	0.42423	0	0	10	415
GO:0009952 anterior/posterior pattern specification	6	0.22869	1.858	0.027	Biological	GO:00099	anterior/posterior pattern specification	0.22869	0.42615	0	0	6	225
GO:0021953 central nervous system neuron differentiation	5	0.22879	1.548	0.028	Biological	GO:00219	central nervous system neuron differentiation	0.22879	0.42617	0	0	5	179
GO:0005504 fatty acid binding	2	0.23014	0.585	0.041	Molecular	GO:00055	fatty acid binding	0.23014	0.43774	0	0	2	49
GO:0005313 L-glutamate transmembrane transporter activity	1	0.2305	0.292	0.071	Molecular	GO:00053	L-glutamate transmembrane transporter activity	0.2305	0.43774	0	0	1	14
GO:0010484 H3 histone acetyltransferase activity	1	0.2305	0.292	0.071	Molecular	GO:00104	H3 histone acetyltransferase activity	0.2305	0.43774	0	0	1	14
GO:0019855 calcium channel inhibitor activity	1	0.2305	0.292	0.071	Molecular	GO:00198	calcium channel inhibitor activity	0.2305	0.43774	0	0	1	14
GO:0031005 filamin binding	1	0.2305	0.292	0.071	Molecular	GO:00310	filamin binding	0.2305	0.43774	0	0	1	14
GO:0031404 chloride ion binding	1	0.2305	0.292	0.071	Molecular	GO:00314	chloride ion binding	0.2305	0.43774	0	0	1	14
GO:0042043 neurexin family protein binding	1	0.2305	0.292	0.071	Molecular	GO:00420	neurexin family protein binding	0.2305	0.43774	0	0	1	14
GO:0044548 S100 protein binding	1	0.2305	0.292	0.071	Molecular	GO:00445	S100 protein binding	0.2305	0.43774	0	0	1	14
GO:0045295 gamma-catenin binding	1	0.2305	0.292	0.071	Molecular	GO:00452	gamma-catenin binding	0.2305	0.43774	0	0	1	14
GO:0052650 NADP-retinol dehydrogenase activity	1	0.2305	0.292	0.071	Molecular	GO:00526	NADP-retinol dehydrogenase activity	0.2305	0.43774	0	0	1	14
GO:1901474 azole transmembrane transporter activity	1	0.2305	0.292	0.071	Molecular	GO:19014	azole transmembrane transporter activity	0.2305	0.43774	0	0	1	14
GO:0005793 endoplasmic reticulum-Golgi intermediate compartment	4	0.23059	1.187	0.029	Cellular C	GO:00057	endoplasmic reticulum-Golgi intermediate compartment	0.23059	0.44697	0	0	4	136
GO:0046849 bone remodeling	3	0.2309	0.929	0.033	Biological	GO:00468	bone remodeling	0.2309	0.42921	0	0	3	91
GO:0051591 response to cAMP	3	0.2309	0.929	0.033	Biological	GO:00515	response to cAMP	0.2309	0.42921	0	0	3	91
GO:0006953 acute-phase response	2	0.23136	0.619	0.04	Biological	GO:00069	acute-phase response	0.23136	0.42921	0	0	2	50
GO:0007520 myoblast fusion	2	0.23136	0.619	0.04	Biological	GO:00075	myoblast fusion	0.23136	0.42921	0	0	2	50
GO:0007528 neuromuscular junction development	2	0.23136	0.619	0.04	Biological	GO:00075	neuromuscular junction development	0.23136	0.42921	0	0	2	50
GO:0010171 body morphogenesis	2	0.23136	0.619	0.04	Biological	GO:00101	body morphogenesis	0.23136	0.42921	0	0	2	50
GO:0010883 regulation of lipid storage	2	0.23136	0.619	0.04	Biological	GO:00108	regulation of lipid storage	0.23136	0.42921	0	0	2	50
GO:0032768 regulation of monooxygenase activity	2	0.23136	0.619	0.04	Biological	GO:00327	regulation of monooxygenase activity	0.23136	0.42921	0	0	2	50
GO:0043330 response to exogenous dsRNA	2	0.23136	0.619	0.04	Biological	GO:00433	response to exogenous dsRNA	0.23136	0.42921	0	0	2	50
GO:0060038 cardiac muscle cell proliferation	2	0.23136	0.619	0.04	Biological	GO:00600	cardiac muscle cell proliferation	0.23136	0.42921	0	0	2	50
GO:1904894 positive regulation of receptor signaling pathway via STAT	2	0.23136	0.619	0.04	Biological	GO:19048	positive regulation of receptor signaling pathway via STAT	0.23136	0.42921	0	0	2	50
GO:0044309 neuron spine	5	0.23202	1.484	0.027	Cellular C	GO:00443	neuron spine	0.23202	0.44813	0	0	5	182
GO:0048017 inositol lipid-mediated signaling	5	0.23213	1.548	0.028	Biological	GO:00480	inositol lipid-mediated signaling	0.23213	0.43046	0	0	5	180
GO:0048565 digestive tract development	4	0.23261	1.238	0.03	Biological	GO:00485	digestive tract development	0.23261	0.43121	0	0	4	135
GO:0016053 organic acid biosynthetic process	8	0.23301	2.477	0.025	Biological	GO:00160	organic acid biosynthetic process	0.23301	0.43179	0	0	8	321
GO:1901681 sulfur compound binding	7	0.23323	2.047	0.026	Molecular	GO:19016	sulfur compound binding	0.23323	0.44148	0	0	7	269
GO:0016298 lipase activity	4	0.23349	1.17	0.03	Molecular	GO:00162	lipase activity	0.23349	0.44148	0	0	4	133
GO:0000421 autophagosome membrane	2	0.23425	0.593	0.039	Cellular C	GO:00004	autophagosome membrane	0.23425	0.45075	0	0	2	51
GO:0051098 regulation of binding	9	0.23518	2.786	0.024	Biological	GO:00510	regulation of binding	0.23518	0.43495	0	0	9	370
GO:0005902 microvillus	3	0.23553	0.89	0.032	Cellular C	GO:00059	microvillus	0.23553	0.45075	0	0	3	93
GO:0030658 transport vesicle membrane	6	0.23591	1.78	0.026	Cellular C	GO:00306	transport vesicle membrane	0.23591	0.45075	0	0	6	230
GO:0098662 inorganic cation transmembrane transport	19	0.237	5.882	0.022	Biological	GO:00986	inorganic cation transmembrane transport	0.237	0.43495	0	0	19	868
GO:0018024 histone-lysine N-methyltransferase activity	2	0.237	0.585	0.04	Molecular	GO:00180	histone-lysine N-methyltransferase activity	0.237	0.44715	0	0	2	50
GO:0021515 cell differentiation in spinal cord	2	0.23812	0.619	0.039	Biological	GO:00215	cell differentiation in spinal cord	0.23812	0.43495	0	0	2	51
GO:0043392 negative regulation of DNA binding	2	0.23812	0.619	0.039	Biological	GO:00433	negative regulation of DNA binding	0.23812	0.43495	0	0	2	51
GO:0046638 positive regulation of alpha-beta T cell differentiation	2	0.23812	0.619	0.039	Biological	GO:00466	positive regulation of alpha-beta T cell differentiation	0.23812	0.43495	0	0	2	51
GO:0046850 regulation of bone remodeling	2	0.23812	0.619	0.039	Biological	GO:00468	regulation of bone remodeling	0.23812	0.43495	0	0	2	51
GO:0051646 mitochondrion localization	2	0.23812	0.619	0.039	Biological	GO:00516	mitochondrion localization	0.23812	0.43495	0	0	2	51
GO:0060612 adipose tissue development	2	0.23812	0.619	0.039	Biological	GO:00606	adipose tissue development	0.23812	0.43495	0	0	2	51
GO:2000272 negative regulation of signaling receptor activity	2	0.23812	0.619	0.039	Biological	GO:20002	negative regulation of signaling receptor activity	0.23812	0.43495	0	0	2	51
GO:0043178 alcohol binding	3	0.23825	0.877	0.033	Molecular	GO:00431	alcohol binding	0.23825	0.44767	0	0	3	91
GO:0004866 endopeptidase inhibitor activity	5	0.23883	1.462	0.028	Molecular	GO:00048	endopeptidase inhibitor activity	0.23883	0.44767	0	0	5	179
GO:0016705 oxidoreductase activity, acting on paired donors, with incor	5	0.23883	1.462	0.028	Molecular	GO:00167	oxidoreductase activity, acting on paired donors, with incorporation o	0.23883	0.44767	0	0	5	179
GO:0031616 spindle pole centrosome	1	0.23887	0.297	0.067	Cellular C	GO:00316	spindle pole centrosome	0.23887	0.45157	0	0	1	15
GO:0097440 apical dendrite	1	0.23887	0.297	0.067	Cellular C	GO:00974	apical dendrite	0.23887	0.45157	0	0	1	15
GO:0098845 postsynaptic endosome	1	0.23887	0.297	0.067	Cellular C	GO:00988	postsynaptic endosome	0.23887	0.45157	0	0	1	15
GO:0044877 protein-containing complex binding	28	0.23946	8.187	0.021	Molecular	GO:00448	protein-containing complex binding	0.23946	0.44789	0	0	28	1308
GO:0005798 Golgi-associated vesicle	3	0.24033	0.89	0.032	Cellular C	GO:00057	Golgi-associated vesicle	0.24033	0.45273	0	0	3	94
GO:0042475 odontogenesis of dentin-containing tooth	3	0.24058	0.929	0.032	Biological	GO:00424	odontogenesis of dentin-containing tooth	0.24058	0.43495	0	0	3	93
GO:2000177 regulation of neural precursor cell proliferation	3	0.24058	0.929	0.032	Biological	GO:20001	regulation of neural precursor cell proliferation	0.24058	0.43495	0	0	3	93
GO:0033157 regulation of intracellular protein transport	6	0.24059	1.858	0.026	Biological	GO:00331	regulation of intracellular protein transport	0.24059	0.43495	0	0	6	229
GO:0033673 negative regulation of kinase activity	6	0.24059	1.858	0.026	Biological	GO:00336	negative regulation of kinase activity	0.24059	0.43495	0	0	6	229
GO:0001771 immunological synapse formation	1	0.24125	0.31	0.067	Biological	GO:00017	immunological synapse formation	0.24125	0.43495	0	0	1	15
GO:0001977 renal system process involved in regulation of blood volum	1	0.24125	0.31	0.067	Biological	GO:00019	renal system process involved in regulation of blood volume	0.24125	0.43495	0	0	1	15
GO:0002864 regulation of acute inflammatory response to antigenic stin	1	0.24125	0.31	0.067	Biological	GO:00028	regulation of acute inflammatory response to antigenic stimulus	0.24125	0.43495	0	0	1	15
GO:0003214 cardiac left ventricle morphogenesis	1	0.24125	0.31	0.067	Biological	GO:00032	cardiac left ventricle morphogenesis	0.24125	0.43495	0	0	1	15
GO:0006750 glutathione biosynthetic process	1	0.24125	0.31	0.067	Biological	GO:00067	glutathione biosynthetic process	0.24125	0.43495	0	0	1	15
GO:0009111 vitamin catabolic process	1	0.24125	0.31	0.067	Biological	GO:00091	vitamin catabolic process	0.24125	0.43495	0	0	1	15
GO:0009163 nucleoside biosynthetic process	1	0.24125	0.31	0.067	Biological	GO:00091	nucleoside biosynthetic process	0.24125	0.43495	0	0	1	15
GO:0009415 response to water	1	0.24125	0.31	0.067	Biological	GO:00094	response to water	0.24125	0.43495	0	0	1	15
GO:0010820 positive regulation of T cell chemotaxis	1	0.24125	0.31	0.067	Biological	GO:00108	positive regulation of T cell chemotaxis	0.24125	0.43495	0	0	1	15
GO:0014857 regulation of skeletal muscle cell proliferation	1	0.24125	0.31	0.067	Biological	GO:00148	regulation of skeletal muscle cell proliferation	0.24125	0.43495	0	0	1	15
GO:0015816 glycine transport	1	0.24125	0.31	0.067	Biological	GO:00158	glycine transport	0.24125	0.43495	0	0	1	15
GO:0017014 protein nitrosylation	1	0.24125	0.31	0.067	Biological	GO:00170	protein nitrosylation	0.24125	0.43495	0	0	1	15
GO:0018119 peptidyl-cysteine S-nitrosylation	1	0.24125	0.31	0.067	Biological	GO:00181	peptidyl-cysteine S-nitrosylation	0.24125	0.43495	0	0	1	15
GO:0021819 layer formation in cerebral cortex	1	0.24125	0.31	0.067	Biological	GO:00218	layer formation in cerebral cortex	0.24125	0.43495	0	0	1	15
GO:0030174 regulation of DNA-dependent DNA replication initiation	1	0.24125	0.31	0.067	Biological	GO:00301	regulation of DNA-dependent DNA replication initiation	0.24125	0.43495	0	0	1	15
GO:0030214 hyaluronan catabolic process	1	0.24125	0.31	0.067	Biological	GO:00302	hyaluronan catabolic process	0.24125	0.43495	0	0	1	15
GO:0032239 regulation of nucleobase-containing compound transport	1	0.24125	0.31	0.067	Biological	GO:00322	regulation of nucleobase-containing compound transport	0.24125	0.43495	0	0	1	15
GO:0032352 positive regulation of hormone metabolic process	1	0.24125	0.31	0.067	Biological	GO:00323	positive regulation of hormone metabolic process	0.24125	0.43495	0	0	1	15
GO:0032488 Cdc42 protein signal transduction	1	0.24125	0.31	0.067	Biological	GO:00324	Cdc42 protein signal transduction	0.24125	0.43495	0	0	1	15
GO:0032725 positive regulation of granulocyte macrophage colony-stim	1	0.24125	0.31	0.067	Biological	GO:00327	positive regulation of granulocyte macrophage colony-stimulating fact	0.24125	0.43495	0	0	1	15
GO:0034310 primary alcohol catabolic process	1	0.24125	0.31	0.067	Biological	GO:00343	primary alcohol catabolic process	0.24125	0.43495	0	0	1	15
GO:0034404 nucleobase-containing small molecule biosynthetic proces	1	0.24125	0.31	0.067	Biological	GO:00344	nucleobase-containing small molecule biosynthetic process	0.24125	0.43495	0	0	1	15
GO:0042976 activation of Janus kinase activity	1	0.24125	0.31	0.067	Biological	GO:00429	activation of Janus kinase activity	0.24125	0.43495	0	0	1	15
GO:0043922 negative regulation by host of viral transcription	1	0.24125	0.31	0.067	Biological	GO:00439	negative regulation by host of viral transcription	0.24125	0.43495	0	0	1	15
GO:0045117 azole transmembrane transport	1	0.24125	0.31	0.067	Biological	GO:00451	azole transmembrane transport	0.24125	0.43495	0	0	1	15
GO:0045176 apical protein localization	1	0.24125	0.31	0.067	Biological	GO:00451	apical protein localization	0.24125	0.43495	0	0	1	15
GO:0045725 positive regulation of glycogen biosynthetic process	1	0.24125	0.31	0.067	Biological	GO:00457	positive regulation of glycogen biosynthetic process	0.24125	0.43495	0	0	1	15
GO:0045986 negative regulation of smooth muscle contraction	1	0.24125	0.31	0.067	Biological	GO:00459	negative regulation of smooth muscle contraction	0.24125	0.43495	0	0	1	15
GO:0046322 negative regulation of fatty acid oxidation	1	0.24125	0.31	0.067	Biological	GO:00463	negative regulation of fatty acid oxidation	0.24125	0.43495	0	0	1	15
GO:0046459 short-chain fatty acid metabolic process	1	0.24125	0.31	0.067	Biological	GO:00464	short-chain fatty acid metabolic process	0.24125	0.43495	0	0	1	15
GO:0046500 S-adenosylmethionine metabolic process	1	0.24125	0.31	0.067	Biological	GO:00465	S-adenosylmethionine metabolic process	0.24125	0.43495	0	0	1	15
GO:0046598 positive regulation of viral entry into host cell	1	0.24125	0.31	0.067	Biological	GO:00465	positive regulation of viral entry into host cell	0.24125	0.43495	0	0	1	15
GO:0048070 regulation of developmental pigmentation	1	0.24125	0.31	0.067	Biological	GO:00480	regulation of developmental pigmentation	0.24125	0.43495	0	0	1	15
GO:0048311 mitochondrion distribution	1	0.24125	0.31	0.067	Biological	GO:00483	mitochondrion distribution	0.24125	0.43495	0	0	1	15
GO:0048820 hair follicle maturation	1	0.24125	0.31	0.067	Biological	GO:00488	hair follicle maturation	0.24125	0.43495	0	0	1	15
GO:0048934 peripheral nervous system neuron differentiation	1	0.24125	0.31	0.067	Biological	GO:00489	peripheral nervous system neuron differentiation	0.24125	0.43495	0	0	1	15
GO:0048935 peripheral nervous system neuron development	1	0.24125	0.31	0.067	Biological	GO:00489	peripheral nervous system neuron development	0.24125	0.43495	0	0	1	15
GO:0051290 protein heterotetramerization	1	0.24125	0.31	0.067	Biological	GO:00512	protein heterotetramerization	0.24125	0.43495	0	0	1	15
GO:0051956 negative regulation of amino acid transport	1	0.24125	0.31	0.067	Biological	GO:00519	negative regulation of amino acid transport	0.24125	0.43495	0	0	1	15

GO:0060009 Sertoli cell development	1	0.24125	0.31	0.067	Biological	GO:00600	Sertoli cell development	0.24125	0.43495	0	0	1	15
GO:0060347 heart trabecula formation	1	0.24125	0.31	0.067	Biological	GO:00603	heart trabecula formation	0.24125	0.43495	0	0	1	15
GO:0060413 atrial septum morphogenesis	1	0.24125	0.31	0.067	Biological	GO:00604	atrial septum morphogenesis	0.24125	0.43495	0	0	1	15
GO:0070262 peptidyl-serine dephosphorylation	1	0.24125	0.31	0.067	Biological	GO:00702	peptidyl-serine dephosphorylation	0.24125	0.43495	0	0	1	15
GO:0070278 extracellular matrix constituent secretion	1	0.24125	0.31	0.067	Biological	GO:00702	extracellular matrix constituent secretion	0.24125	0.43495	0	0	1	15
GO:0070831 basement membrane assembly	1	0.24125	0.31	0.067	Biological	GO:00708	basement membrane assembly	0.24125	0.43495	0	0	1	15
GO:0071107 response to parathyroid hormone	1	0.24125	0.31	0.067	Biological	GO:00711	response to parathyroid hormone	0.24125	0.43495	0	0	1	15
GO:0072531 pyrimidine-containing compound transmembrane transpor	1	0.24125	0.31	0.067	Biological	GO:00725	pyrimidine-containing compound transmembrane transport	0.24125	0.43495	0	0	1	15
GO:0075294 positive regulation by symbiont of entry into host	1	0.24125	0.31	0.067	Biological	GO:00752	positive regulation by symbiont of entry into host	0.24125	0.43495	0	0	1	15
GO:0086069 bundle of His cell to Purkinje myocyte communication	1	0.24125	0.31	0.067	Biological	GO:00860	bundle of His cell to Purkinje myocyte communication	0.24125	0.43495	0	0	1	15
GO:0086103 G protein-coupled receptor signaling pathway involved in h	1	0.24125	0.31	0.067	Biological	GO:00861	G protein-coupled receptor signaling pathway involved in heart proce	0.24125	0.43495	0	0	1	15
GO:0090051 negative regulation of cell migration involved in sprouting a	1	0.24125	0.31	0.067	Biological	GO:00900	negative regulation of cell migration involved in sprouting angiogenes	0.24125	0.43495	0	0	1	15
GO:0090660 cerebrospinal fluid circulation	1	0.24125	0.31	0.067	Biological	GO:00906	cerebrospinal fluid circulation	0.24125	0.43495	0	0	1	15
GO:0097531 mast cell migration	1	0.24125	0.31	0.067	Biological	GO:00975	mast cell migration	0.24125	0.43495	0	0	1	15
GO:0098840 protein transport along microtubule	1	0.24125	0.31	0.067	Biological	GO:00988	protein transport along microtubule	0.24125	0.43495	0	0	1	15
GO:0098883 synapse pruning	1	0.24125	0.31	0.067	Biological	GO:00988	synapse pruning	0.24125	0.43495	0	0	1	15
GO:0099118 microtubule-based protein transport	1	0.24125	0.31	0.067	Biological	GO:00991	microtubule-based protein transport	0.24125	0.43495	0	0	1	15
GO:0120305 regulation of pigmentation	1	0.24125	0.31	0.067	Biological	GO:01203	regulation of pigmentation	0.24125	0.43495	0	0	1	15
GO:0150011 regulation of neuron projection arborization	1	0.24125	0.31	0.067	Biological	GO:01500	regulation of neuron projection arborization	0.24125	0.43495	0	0	1	15
GO:1901163 regulation of trophoblast cell migration	1	0.24125	0.31	0.067	Biological	GO:19011	regulation of trophoblast cell migration	0.24125	0.43495	0	0	1	15
GO:1903025 regulation of RNA polymerase II regulatory region sequenc	1	0.24125	0.31	0.067	Biological	GO:19030	regulation of RNA polymerase II regulatory region sequence-specific	0.24125	0.43495	0	0	1	15
GO:2000725 regulation of cardiac muscle cell differentiation	1	0.24125	0.31	0.067	Biological	GO:20007	regulation of cardiac muscle cell differentiation	0.24125	0.43495	0	0	1	15
GO:2001138 regulation of phospholipid transport	1	0.24125	0.31	0.067	Biological	GO:20011	regulation of phospholipid transport	0.24125	0.43495	0	0	1	15
GO:1905475 regulation of protein localization to membrane	5	0.24222	1.548	0.027	Biological	GO:19054	regulation of protein localization to membrane	0.24222	0.43654	0	0	5	183
GO:0031328 positive regulation of cellular biosynthetic process	42	0.24318	13.003	0.02	Biological	GO:00313	positive regulation of cellular biosynthetic process	0.24318	0.43811	0	0	42	2062
GO:0008013 beta-catenin binding	3	0.24319	0.877	0.033	Molecular	GO:00080	beta-catenin binding	0.24319	0.44909	0	0	3	92
GO:0016651 oxidoreductase activity, acting on NAD(P)H	3	0.24319	0.877	0.033	Molecular	GO:00166	oxidoreductase activity, acting on NAD(P)H	0.24319	0.44909	0	0	3	92
GO:0031984 organelle subcompartment	31	0.24438	9.199	0.021	Cellular C	GO:00319	organelle subcompartment	0.24438	0.45875	0	0	31	1506
GO:0004129 cytochrome-c oxidase activity	1	0.24477	0.292	0.067	Molecular	GO:00041	cytochrome-c oxidase activity	0.24477	0.44909	0	0	1	15
GO:0004953 icosanoid receptor activity	1	0.24477	0.292	0.067	Molecular	GO:00049	icosanoid receptor activity	0.24477	0.44909	0	0	1	15
GO:0016208 AMP binding	1	0.24477	0.292	0.067	Molecular	GO:00162	AMP binding	0.24477	0.44909	0	0	1	15
GO:0042813 Wnt-activated receptor activity	1	0.24477	0.292	0.067	Molecular	GO:00428	Wnt-activated receptor activity	0.24477	0.44909	0	0	1	15
GO:0045028 G protein-coupled purinergic nucleotide receptor activity	1	0.24477	0.292	0.067	Molecular	GO:00450	G protein-coupled purinergic nucleotide receptor activity	0.24477	0.44909	0	0	1	15
GO:0097153 cysteine-type endopeptidase activity involved in apoptotic p	1	0.24477	0.292	0.067	Molecular	GO:00971	cysteine-type endopeptidase activity involved in apoptotic process	0.24477	0.44909	0	0	1	15
GO:0099103 channel activator activity	1	0.24477	0.292	0.067	Molecular	GO:00991	channel activator activity	0.24477	0.44909	0	0	1	15
GO:0007215 glutamate receptor signaling pathway	2	0.24488	0.619	0.038	Biological	GO:00072	glutamate receptor signaling pathway	0.24488	0.43969	0	0	2	52
GO:0022602 ovulation cycle process	2	0.24488	0.619	0.038	Biological	GO:00226	ovulation cycle process	0.24488	0.43969	0	0	2	52
GO:0032007 negative regulation of TOR signaling	2	0.24488	0.619	0.038	Biological	GO:00320	negative regulation of TOR signaling	0.24488	0.43969	0	0	2	52
GO:0043536 positive regulation of blood vessel endothelial cell migratior	2	0.24488	0.619	0.038	Biological	GO:00435	positive regulation of blood vessel endothelial cell migration	0.24488	0.43969	0	0	2	52
GO:0050435 amyloid-beta metabolic process	2	0.24488	0.619	0.038	Biological	GO:00504	amyloid-beta metabolic process	0.24488	0.43969	0	0	2	52
GO:0089718 amino acid import across plasma membrane	2	0.24488	0.619	0.038	Biological	GO:00897	amino acid import across plasma membrane	0.24488	0.43969	0	0	2	52
GO:1990928 response to amino acid starvation	2	0.24488	0.619	0.038	Biological	GO:19909	response to amino acid starvation	0.24488	0.43969	0	0	2	52
GO:2000649 regulation of sodium ion transmembrane transporter activit	2	0.24488	0.619	0.038	Biological	GO:20006	regulation of sodium ion transmembrane transporter activity	0.24488	0.43969	0	0	2	52
GO:2000677 regulation of transcription regulatory region DNA binding	2	0.24488	0.619	0.038	Biological	GO:20006	regulation of transcription regulatory region DNA binding	0.24488	0.43969	0	0	2	52
GO:0007018 microtubule-based movement	10	0.24494	3.096	0.024	Biological	GO:00070	microtubule-based movement	0.24494	0.43969	0	0	10	423
GO:0006835 dicarboxylic acid transport	3	0.24545	0.929	0.032	Biological	GO:00068	dicarboxylic acid transport	0.24545	0.4403	0	0	3	94
GO:0019218 regulation of steroid metabolic process	3	0.24545	0.929	0.032	Biological	GO:00192	regulation of steroid metabolic process	0.24545	0.4403	0	0	3	94
GO:0006950 response to stress	78	0.24613	24.149	0.02	Biological	GO:00069	response to stress	0.24613	0.44135	0	0	78	3974
GO:0043565 sequence-specific DNA binding	35	0.24783	10.234	0.021	Molecular	GO:00435	sequence-specific DNA binding	0.24783	0.45375	0	0	35	1672
GO:0007266 Rho protein signal transduction	4	0.24836	1.238	0.029	Biological	GO:00072	Rho protein signal transduction	0.24836	0.44504	0	0	4	139
GO:0071456 cellular response to hypoxia	4	0.24836	1.238	0.029	Biological	GO:00714	cellular response to hypoxia	0.24836	0.44504	0	0	4	139
GO:0031669 cellular response to nutrient levels	6	0.24963	1.858	0.026	Biological	GO:00316	cellular response to nutrient levels	0.24963	0.44715	0	0	6	232
GO:0008017 microtubule binding	7	0.24982	2.047	0.025	Molecular	GO:00080	microtubule binding	0.24982	0.45457	0	0	7	275
GO:0006942 regulation of striated muscle contraction	3	0.25034	0.929	0.032	Biological	GO:00069	regulation of striated muscle contraction	0.25034	0.44724	0	0	3	95
GO:0032611 interleukin-1 beta production	3	0.25034	0.929	0.032	Biological	GO:00326	interleukin-1 beta production	0.25034	0.44724	0	0	3	95
GO:0032651 regulation of interleukin-1 beta production	3	0.25034	0.929	0.032	Biological	GO:00326	regulation of interleukin-1 beta production	0.25034	0.44724	0	0	3	95
GO:0016763 pentosyltransferase activity	2	0.25076	0.585	0.038	Molecular	GO:00167	pentosyltransferase activity	0.25076	0.45457	0	0	2	52
GO:0016879 ligase activity, forming carbon-nitrogen bonds	2	0.25076	0.585	0.038	Molecular	GO:00168	ligase activity, forming carbon-nitrogen bonds	0.25076	0.45457	0	0	2	52
GO:0034703 cation channel complex	6	0.25077	1.78	0.026	Cellular C	GO:00347	cation channel complex	0.25077	0.466	0	0	6	235
GO:0015075 ion transmembrane transporter activity	19	0.2516	5.556	0.022	Molecular	GO:00150	ion transmembrane transporter activity	0.2516	0.45457	0	0	19	863
GO:0010543 regulation of platelet activation	2	0.25165	0.619	0.038	Biological	GO:00105	regulation of platelet activation	0.25165	0.44724	0	0	2	53
GO:0060071 Wnt signaling pathway, planar cell polarity pathway	2	0.25165	0.619	0.038	Biological	GO:00600	Wnt signaling pathway, planar cell polarity pathway	0.25165	0.44724	0	0	2	53
GO:0061462 protein localization to lysosome	2	0.25165	0.619	0.038	Biological	GO:00614	protein localization to lysosome	0.25165	0.44724	0	0	2	53
GO:0030133 transport vesicle	10	0.25202	2.967	0.023	Cellular C	GO:00301	transport vesicle	0.25202	0.466	0	0	10	431
GO:0046887 positive regulation of hormone secretion	4	0.25234	1.238	0.029	Biological	GO:00468	positive regulation of hormone secretion	0.25234	0.44724	0	0	4	140
GO:0009266 response to temperature stimulus	5	0.25244	1.548	0.027	Biological	GO:00092	response to temperature stimulus	0.25244	0.44724	0	0	5	186
GO:0010721 negative regulation of cell development	5	0.25244	1.548	0.027	Biological	GO:00107	negative regulation of cell development	0.25244	0.44724	0	0	5	186
GO:0005744 TIM23 mitochondrial import inner membrane translocase c	1	0.2526	0.297	0.062	Cellular C	GO:00057	TIM23 mitochondrial import inner membrane translocase complex	0.2526	0.466	0	0	1	16
GO:0032156 septin cytoskeleton	1	0.2526	0.297	0.062	Cellular C	GO:00321	septin cytoskeleton	0.2526	0.466	0	0	1	16
GO:0098554 cytoplasmic side of endoplasmic reticulum membrane	1	0.2526	0.297	0.062	Cellular C	GO:00985	cytoplasmic side of endoplasmic reticulum membrane	0.2526	0.466	0	0	1	16
GO:0006952 defense response	36	0.25387	11.146	0.02	Biological	GO:00069	defense response	0.25387	0.44724	0	0	36	1758
GO:0000097 sulfur amino acid biosynthetic process	1	0.25509	0.31	0.062	Biological	GO:00000	sulfur amino acid biosynthetic process	0.25509	0.44724	0	0	1	16
GO:0002371 dendritic cell cytokine production	1	0.25509	0.31	0.062	Biological	GO:00023	dendritic cell cytokine production	0.25509	0.44724	0	0	1	16
GO:0002730 regulation of dendritic cell cytokine production	1	0.25509	0.31	0.062	Biological	GO:00027	regulation of dendritic cell cytokine production	0.25509	0.44724	0	0	1	16
GO:0003188 heart valve formation	1	0.25509	0.31	0.062	Biological	GO:00031	heart valve formation	0.25509	0.44724	0	0	1	16
GO:0003413 chondrocyte differentiation involved in endochondral bone	1	0.25509	0.31	0.062	Biological	GO:00034	chondrocyte differentiation involved in endochondral bone morphoger	0.25509	0.44724	0	0	1	16
GO:0006266 DNA ligation	1	0.25509	0.31	0.062	Biological	GO:00062	DNA ligation	0.25509	0.44724	0	0	1	16
GO:0006677 glycosylceramide metabolic process	1	0.25509	0.31	0.062	Biological	GO:00066	glycosylceramide metabolic process	0.25509	0.44724	0	0	1	16
GO:0006825 copper ion transport	1	0.25509	0.31	0.062	Biological	GO:00068	copper ion transport	0.25509	0.44724	0	0	1	16
GO:0007028 cytoplasm organization	1	0.25509	0.31	0.062	Biological	GO:00070	cytoplasm organization	0.25509	0.44724	0	0	1	16
GO:0009074 aromatic amino acid family catabolic process	1	0.25509	0.31	0.062	Biological	GO:00090	aromatic amino acid family catabolic process	0.25509	0.44724	0	0	1	16
GO:0010763 positive regulation of fibroblast migration	1	0.25509	0.31	0.062	Biological	GO:00107	positive regulation of fibroblast migration	0.25509	0.44724	0	0	1	16
GO:0010819 regulation of T cell chemotaxis	1	0.25509	0.31	0.062	Biological	GO:00108	regulation of T cell chemotaxis	0.25509	0.44724	0	0	1	16
GO:0014819 regulation of skeletal muscle contraction	1	0.25509	0.31	0.062	Biological	GO:00148	regulation of skeletal muscle contraction	0.25509	0.44724	0	0	1	16
GO:0019511 peptidyl-proline hydroxylation	1	0.25509	0.31	0.062	Biological	GO:00195	peptidyl-proline hydroxylation	0.25509	0.44724	0	0	1	16
GO:0021783 preganglionic parasympathetic fiber development	1	0.25509	0.31	0.062	Biological	GO:00217	preganglionic parasympathetic fiber development	0.25509	0.44724	0	0	1	16
GO:0030852 regulation of granulocyte differentiation	1	0.25509	0.31	0.062	Biological	GO:00308	regulation of granulocyte differentiation	0.25509	0.44724	0	0	1	16
GO:0034374 low-density lipoprotein particle remodeling	1	0.25509	0.31	0.062	Biological	GO:00343	low-density lipoprotein particle remodeling	0.25509	0.44724	0	0	1	16
GO:0034375 high-density lipoprotein particle remodeling	1	0.25509	0.31	0.062	Biological	GO:00343	high-density lipoprotein particle remodeling	0.25509	0.44724	0	0	1	16
GO:0042159 lipoprotein catabolic process	1	0.25509	0.31	0.062	Biological	GO:00421	lipoprotein catabolic process	0.25509	0.44724	0	0	1	16
GO:0042249 establishment of planar polarity of embryonic epithelium	1	0.25509	0.31	0.062	Biological	GO:00422	establishment of planar polarity of embryonic epithelium	0.25509	0.44724	0	0	1	16
GO:0042994 cytoplasmic sequestering of transcription factor	1	0.25509	0.31	0.062	Biological	GO:00429	cytoplasmic sequestering of transcription factor	0.25509	0.44724	0	0	1	16
GO:0043374 CD8-positive, alpha-beta T cell differentiation	1	0.25509	0.31	0.062	Biological	GO:00433	CD8-positive, alpha-beta T cell differentiation	0.25509	0.44724	0	0	1	16
GO:0044065 regulation of respiratory system process	1	0.25509	0.31	0.062	Biological	GO:00440	regulation of respiratory system process	0.25509	0.44724	0	0	1	16
GO:0044154 histone H3-K14 acetylation	1	0.25509	0.31	0.062	Biological	GO:00441	histone H3-K14 acetylation	0.25509	0.44724	0	0	1	16
GO:0044406 adhesion of symbiont to host	1	0.25509	0.31	0.062	Biological	GO:00444	adhesion of symbiont to host	0.25509	0.44724	0	0	1	16
GO:0045187 regulation of circadian sleep/wake cycle, sleep	1	0.25509	0.31	0.062	Biological	GO:00451	regulation of circadian sleep/wake cycle, sleep	0.25509	0.44724	0	0	1	16
GO:0045475 locomotor rhythm	1	0.25509	0.31	0.062	Biological	GO:00454	locomotor rhythm	0.25509	0.44724	0	0	1	16
GO:0051044 positive regulation of membrane protein ectodomain protec	1	0.25509	0.31	0.062	Biological	GO:00510	positive regulation of membrane protein ectodomain proteolysis	0.25509	0.44724	0	0	1	16
GO:0060099 regulation of phagocytosis, engulfment	1	0.25509	0.31	0.062	Biological	GO:00600	regulation of phagocytosis, engulfment	0.25509	0.44724	0	0	1	16
GO:0060572 morphogenesis of an epithelial bud	1	0.25509	0.31	0.062	Biological	GO:00605	morphogenesis of an epithelial bud	0.25509	0.44724	0	0	1	16
GO:0060841 venous blood vessel development	1	0.25509	0.31	0.062	Biological	GO:00608	venous blood vessel development	0.25509	0.44724	0	0	1	16
GO:0061450 trophoblast cell migration	1	0.25509	0.31	0.062	Biological	GO:00614	trophoblast cell migration	0.25509	0.44724	0	0	1	16
GO:0061577 calcium ion transmembrane transport via high voltage-gate	1	0.25509	0.31	0.062	Biological	GO:00615	calcium ion transmembrane transport via high voltage-gated calcium	0.25509	0.44724	0	0	1	16
GO:0061684 chaperone-mediated autophagy	1	0.25509	0.31	0.062	Biological	GO:00616	chaperone-mediated autophagy	0.25509	0.44724	0	0	1	16
GO:0070207 protein homotrimerization	1	0.25509	0.31	0.062	Biological	GO:00702	protein homotrimerization	0.25509	0.44724	0	0	1	16
GO:0070875 positive regulation of glycogen metabolic process	1	0.25509	0.31	0.062	Biological	GO:00708	positive regulation of glycogen metabolic process	0.25509	0.44724	0	0	1	16
GO:0072176 nephric duct development	1	0.25509	0.31	0.062	Biological	GO:00721	nephric duct development	0.25509	0.44724	0	0	1	16
GO:0090336 positive regulation of brown fat cell differentiation	1	0.25509	0.31	0.062	Biological	GO:00903	positive regulation of brown fat cell differentiation	0.25509	0.44724	0	0	1	16
GO:0090594 inflammatory response to wounding	1	0.25509	0.31	0.062	Biological	GO:00905	inflammatory response to wounding	0.25509	0.44724	0	0	1	16
GO:0099188 postsynaptic cytoskeleton organization	1	0.25509	0.31	0.062	Biological	GO:00991	postsynaptic cytoskeleton organization	0.25509	0.44724	0	0	1	16
GO:0099638 endosome to plasma membrane protein transport	1	0.25509	0.31	0.062	Biological	GO:00996	endosome to plasma membrane protein transport	0.25509	0.44724	0	0	1	16
GO:1901741 positive regulation of myoblast fusion	1	0.25509	0.31	0.062	Biological	GO:19017	positive regulation of myoblast fusion	0.25509	0.44724	0	0	1	16
GO:1902285 semaphorin-plexin signaling pathway involved in neuron pr	1	0.25509	0.31	0.062	Biological	GO:19022	semaphorin-plexin signaling pathway involved in neuron projection gu	0.25509	0.44724	0	0	1	16
GO:1903909 regulation of receptor clustering	1	0.25509	0.31	0.062	Biological	GO:19039	regulation of receptor clustering	0.25509	0.44724	0	0	1	16
GO:1904424 regulation of GTP binding	1	0.25509	0.31	0.062	Biological	GO:19044	regulation of GTP binding	0.25509	0.44724	0	0	1	16
GO:1905153 regulation of membrane invagination	1	0.25509	0.31	0.062	Biological	GO:19051	regulation of membrane invagination	0.25509	0.44724	0	0	1	16
GO:2000232 regulation of rRNA processing	1	0.25509	0.31	0.062	Biological	GO:20002	regulation of rRNA processing	0.25509	0.44724	0	0	1	16
GO:2000291 regulation of myoblast proliferation	1	0.25509	0.31	0.062	Biological	GO:20002	regulation of myoblast proliferation	0.25509	0.44724	0	0	1	16
GO:2001256 regulation of store-operated calcium entry	1	0.25509	0.31	0.062	Biological	GO:20012	regulation of store-operated calcium entry	0.25509	0.44724	0	0	1	16
GO:0007044 cell-substrate junction assembly	3	0.25523	0.929	0.031	Biological	GO:00070	cell-substrate junction assembly	0.25523	0.44724	0	0	3	96
GO:0046330 positive regulation of JNK cascade	3	0.25523	0.929	0.031	Biological	GO:00463	positive regulation of JNK cascade	0.25523	0.44724	0	0	3	96
GO:0048525 negative regulation of viral process	3	0.25523	0.929	0.031	Biological	GO:00485	negative regulation of viral process	0.25523	0.44724	0	0	3	96
GO:0050769 positive regulation of neurogenesis	6	0.25571	1.858	0.026	Biological	GO:00507	positive regulation of neurogenesis	0.25571	0.44772	0	0	6	234
GO:0090257 regulation of muscle system process	6	0.25571	1.858	0.026	Biological	GO:00902	regulation of muscle system process	0.25571	0.44772	0	0	6	234
GO:0009896 positive regulation of catabolic process	12	0.2558	3.715	0.023	Biological	GO:00098	positive regulation of catabolic process	0.2558	0.44772	0	0	12	527
GO:0002700 regulation of production of molecular mediator of immune r	5	0.25586	1.548	0.027	Biological	GO:00027	regulation of production of molecular mediator of immune response	0.25586	0.44772	0	0	5	187
GO:0050767 regulation of neurogenesis	9	0.25628	2.786	0.024	Biological	GO:00507	regulation of neurogenesis	0.25628	0.44821	0	0	9	379
GO:1903322 positive regulation of protein modification by small protein c	4	0.25632	1.238	0.028	Biological	GO:19033	positive regulation of protein modification by small protein conjugatior	0.25632	0.44821	0	0	4	141
GO:0019787 ubiquitin-like protein transferase activity	11	0.25703	3.216	0.023	Molecular	GO:00197	ubiquitin-like protein transferase activity	0.25703	0.45457	0	0	11	470
GO:0017046 peptide hormone binding	2	0.25765	0.585	0.038	Molecular	GO:00170	peptide hormone binding	0.25765	0.45457	0	0	2	53
GO:0062012 regulation of small molecule metabolic process	8	0.25819	2.477	0.024	Biological	GO:00620	regulation of small molecule metabolic process	0.25819	0.45062	0	0	8	331
GO:0009299 mRNA transcription	2	0.25843	0.619	0.037	Biological	GO:00092	mRNA transcription	0.25843	0.45062	0	0	2	54
GO:0010676 positive regulation of cellular carbohydrate metabolic proce	2	0.25843	0.619	0.037	Biological	GO:00106	positive regulation of cellular carbohydrate metabolic process	0.25843	0.45062	0	0	2	54
GO:0010761 fibroblast migration	2	0.25843	0.619	0.037	Biological	GO:00107	fibroblast migration	0.25843	0.45062	0	0	2	54
GO:0021872 forebrain generation of neurons	2	0.25843	0.619	0.037	Biological	GO:00218	forebrain generation of neurons	0.25843	0.45062	0	0	2	54
GO:0032330 regulation of chondrocyte differentiation	2	0.25843	0.619	0.037	Biological	GO:00323	regulation of chondrocyte differentiation	0.25843	0.45062	0	0	2	54
GO:0043370 regulation of CD4-positive, alpha-beta T cell differentiation	2	0.25843	0.619	0.037	Biological	GO:00433	regulation of CD4-positive, alpha-beta T cell differentiation	0.25843	0.45062	0	0	2	54
GO:0045744 negative regulation of G protein-coupled receptor signaling	2	0.25843	0.619	0.037	Biological	GO:00457	negative regulation of G protein-coupled receptor signaling pathway	0.25843	0.45062	0	0	2	54
GO:0004559 alpha-mannosidase activity	1	0.25878	0.292	0.062	Molecular	GO:00045	alpha-mannosidase activity	0.25878	0.45457	0	0	1	16
GO:0005092 GDP-dissociation inhibitor activity	1	0.25878	0.292	0.062	Molecular	GO:00050	GDP-dissociation inhibitor activity	0.25878	0.45457	0	0	1	16
GO:0005523 tropomyosin binding	1	0.25878	0.292	0.062	Molecular	GO:00055	tropomyosin binding	0.25878	0.45457	0	0	1	16
GO:0015108 chloride transmembrane transporter activity	1	0.25878	0.292	0.062	Molecular	GO:00151	chloride transmembrane transporter activity	0.25878	0.45457	0	0	1	16
GO:0015929 hexosaminidase activity	1	0.25878	0.292	0.062	Molecular	GO:00159	hexosaminidase activity	0.25878	0.45457	0	0	1	16
GO:0016675 oxidoreductase activity, acting on a heme group of donors	1	0.25878	0.292	0.062	Molecular	GO:00166	oxidoreductase activity, acting on a heme group of donors	0.25878	0.45457	0	0	1	16
GO:0016722 oxidoreductase activity, acting on metal ions	1	0.25878	0.292	0.062	Molecular	GO:00167	oxidoreductase activity, acting on metal ions	0.25878	0.45457	0	0	1	16
GO:0016805 dipeptidase activity	1	0.25878	0.292	0.062	Molecular	GO:00168	dipeptidase activity	0.25878	0.45457	0	0	1	16
GO:0022858 alanine transmembrane transporter activity	1	0.25878	0.292	0.062	Molecular	GO:00228	alanine transmembrane transporter activity	0.25878	0.45457	0	0	1	16
GO:0030169 low-density lipoprotein particle binding	1	0.25878	0.292	0.062	Molecular	GO:00301	low-density lipoprotein particle binding	0.25878	0.45457	0	0	1	16
GO:0030297 transmembrane receptor protein tyrosine kinase activator a	1	0.25878	0.292	0.062	Molecular	GO:00302	transmembrane receptor protein tyrosine kinase activator activity	0.25878	0.45457	0	0	1	16
GO:0050700 CARD domain binding	1	0.25878	0.292	0.062	Molecular	GO:00507	CARD domain binding	0.25878	0.45457	0	0	1	16
GO:0055102 lipase inhibitor activity	1	0.25878	0.292	0.062	Molecular	GO:00551	lipase inhibitor activity	0.25878	0.45457	0	0	1	16

GO:1904680 peptide transmembrane transporter activity	1	0.25878	0.292	0.062	Molecular	GO:19046	peptide transmembrane transporter activity	0.25878	0.45457	0	0	1	16
GO:0030414 peptidase inhibitor activity	5	0.25963	1.462	0.027	Molecular	GO:00304	peptidase inhibitor activity	0.25963	0.45514	0	0	5	185
GO:0001750 photoreceptor outer segment	3	0.25966	0.89	0.031	Cellular C	GO:00017	photoreceptor outer segment	0.25966	0.47739	0	0	3	98
GO:0000041 transition metal ion transport	3	0.26014	0.929	0.031	Biological	GO:00000	transition metal ion transport	0.26014	0.45299	0	0	3	97
GO:0009798 axis specification	3	0.26014	0.929	0.031	Biological	GO:00097	axis specification	0.26014	0.45299	0	0	3	97
GO:0030516 regulation of axon extension	3	0.26014	0.929	0.031	Biological	GO:00305	regulation of axon extension	0.26014	0.45299	0	0	3	97
GO:1901019 regulation of calcium ion transmembrane transporter activi	3	0.26014	0.929	0.031	Biological	GO:19010	regulation of calcium ion transmembrane transporter activity	0.26014	0.45299	0	0	3	97
GO:0045444 fat cell differentiation	6	0.26183	1.858	0.025	Biological	GO:00454	fat cell differentiation	0.26183	0.45572	0	0	6	236
GO:0051962 positive regulation of nervous system development	7	0.26199	2.167	0.025	Biological	GO:00519	positive regulation of nervous system development	0.26199	0.45572	0	0	7	284
GO:1903829 positive regulation of cellular protein localization	7	0.26199	2.167	0.025	Biological	GO:19038	positive regulation of cellular protein localization	0.26199	0.45572	0	0	7	284
GO:0030324 lung development	5	0.26275	1.548	0.026	Biological	GO:00303	lung development	0.26275	0.45689	0	0	5	189
GO:0051213 dioxygenase activity	3	0.26312	0.877	0.031	Molecular	GO:00512	dioxygenase activity	0.26312	0.46032	0	0	3	96
GO:0055076 transition metal ion homeostasis	4	0.26433	1.238	0.028	Biological	GO:00550	transition metal ion homeostasis	0.26433	0.45937	0	0	4	143
GO:0055038 recycling endosome membrane	3	0.26453	0.89	0.03	Cellular C	GO:00550	recycling endosome membrane	0.26453	0.48256	0	0	3	99
GO:0045185 maintenance of protein location	3	0.26507	0.929	0.031	Biological	GO:00451	maintenance of protein location	0.26507	0.45937	0	0	3	98
GO:0062014 negative regulation of small molecule metabolic process	3	0.26507	0.929	0.031	Biological	GO:00620	negative regulation of small molecule metabolic process	0.26507	0.45937	0	0	3	98
GO:0003229 ventricular cardiac muscle tissue development	2	0.26521	0.619	0.036	Biological	GO:00032	ventricular cardiac muscle tissue development	0.26521	0.45937	0	0	2	55
GO:0033344 cholesterol efflux	2	0.26521	0.619	0.036	Biological	GO:00333	cholesterol efflux	0.26521	0.45937	0	0	2	55
GO:0051055 negative regulation of lipid biosynthetic process	2	0.26521	0.619	0.036	Biological	GO:00510	negative regulation of lipid biosynthetic process	0.26521	0.45937	0	0	2	55
GO:0052372 modulation by symbiont of entry into host	2	0.26521	0.619	0.036	Biological	GO:00523	modulation by symbiont of entry into host	0.26521	0.45937	0	0	2	55
GO:0030666 endocytic vesicle membrane	5	0.26571	1.484	0.026	Cellular C	GO:00306	endocytic vesicle membrane	0.26571	0.48256	0	0	5	192
GO:0001650 fibrillar center	4	0.26585	1.187	0.028	Cellular C	GO:00016	fibrillar center	0.26585	0.48256	0	0	4	145
GO:0099091 postsynaptic specialization, intracellular component	1	0.26608	0.297	0.059	Cellular C	GO:00990	postsynaptic specialization, intracellular component	0.26608	0.48256	0	0	1	17
GO:0010876 lipid localization	11	0.26791	3.406	0.023	Biological	GO:00108	lipid localization	0.26791	0.45937	0	0	11	483
GO:0001991 regulation of systemic arterial blood pressure by circulatory	1	0.26869	0.31	0.059	Biological	GO:00019	regulation of systemic arterial blood pressure by circulatory renin-ang	0.26869	0.45937	0	0	1	17
GO:0003198 epithelial to mesenchymal transition involved in endocardia	1	0.26869	0.31	0.059	Biological	GO:00031	epithelial to mesenchymal transition involved in endocardial cushion f	0.26869	0.45937	0	0	1	17
GO:0006474 N-terminal protein amino acid acetylation	1	0.26869	0.31	0.059	Biological	GO:00064	N-terminal protein amino acid acetylation	0.26869	0.45937	0	0	1	17
GO:0006895 Golgi to endosome transport	1	0.26869	0.31	0.059	Biological	GO:00068	Golgi to endosome transport	0.26869	0.45937	0	0	1	17
GO:0006957 complement activation, alternative pathway	1	0.26869	0.31	0.059	Biological	GO:00069	complement activation, alternative pathway	0.26869	0.45937	0	0	1	17
GO:0009068 aspartate family amino acid catabolic process	1	0.26869	0.31	0.059	Biological	GO:00090	aspartate family amino acid catabolic process	0.26869	0.45937	0	0	1	17
GO:0009071 serine family amino acid catabolic process	1	0.26869	0.31	0.059	Biological	GO:00090	serine family amino acid catabolic process	0.26869	0.45937	0	0	1	17
GO:0009886 post-embryonic animal morphogenesis	1	0.26869	0.31	0.059	Biological	GO:00098	post-embryonic animal morphogenesis	0.26869	0.45937	0	0	1	17
GO:0010885 regulation of cholesterol storage	1	0.26869	0.31	0.059	Biological	GO:00108	regulation of cholesterol storage	0.26869	0.45937	0	0	1	17
GO:0016045 detection of bacterium	1	0.26869	0.31	0.059	Biological	GO:00160	detection of bacterium	0.26869	0.45937	0	0	1	17
GO:0019184 nonribosomal peptide biosynthetic process	1	0.26869	0.31	0.059	Biological	GO:00191	nonribosomal peptide biosynthetic process	0.26869	0.45937	0	0	1	17
GO:0021513 spinal cord dorsal/ventral patterning	1	0.26869	0.31	0.059	Biological	GO:00215	spinal cord dorsal/ventral patterning	0.26869	0.45937	0	0	1	17
GO:0031649 heat generation	1	0.26869	0.31	0.059	Biological	GO:00316	heat generation	0.26869	0.45937	0	0	1	17
GO:0032274 gonadotropin secretion	1	0.26869	0.31	0.059	Biological	GO:00322	gonadotropin secretion	0.26869	0.45937	0	0	1	17
GO:0033599 regulation of mammary gland epithelial cell proliferation	1	0.26869	0.31	0.059	Biological	GO:00335	regulation of mammary gland epithelial cell proliferation	0.26869	0.45937	0	0	1	17
GO:0033623 regulation of integrin activation	1	0.26869	0.31	0.059	Biological	GO:00336	regulation of integrin activation	0.26869	0.45937	0	0	1	17
GO:0034134 toll-like receptor 2 signaling pathway	1	0.26869	0.31	0.059	Biological	GO:00341	toll-like receptor 2 signaling pathway	0.26869	0.45937	0	0	1	17
GO:0035269 protein O-linked mannosylation	1	0.26869	0.31	0.059	Biological	GO:00352	protein O-linked mannosylation	0.26869	0.45937	0	0	1	17
GO:0036005 response to macrophage colony-stimulating factor	1	0.26869	0.31	0.059	Biological	GO:00360	response to macrophage colony-stimulating factor	0.26869	0.45937	0	0	1	17
GO:0036159 inner dynein arm assembly	1	0.26869	0.31	0.059	Biological	GO:00361	inner dynein arm assembly	0.26869	0.45937	0	0	1	17
GO:0036507 protein demannosylation	1	0.26869	0.31	0.059	Biological	GO:00365	protein demannosylation	0.26869	0.45937	0	0	1	17
GO:0036508 protein alpha-1,2-demannosylation	1	0.26869	0.31	0.059	Biological	GO:00365	protein alpha-1,2-demannosylation	0.26869	0.45937	0	0	1	17
GO:0043129 surfactant homeostasis	1	0.26869	0.31	0.059	Biological	GO:00431	surfactant homeostasis	0.26869	0.45937	0	0	1	17
GO:0043249 erythrocyte maturation	1	0.26869	0.31	0.059	Biological	GO:00432	erythrocyte maturation	0.26869	0.45937	0	0	1	17
GO:0044764 multi-organism cellular process	1	0.26869	0.31	0.059	Biological	GO:00447	multi-organism cellular process	0.26869	0.45937	0	0	1	17
GO:0045722 positive regulation of gluconeogenesis	1	0.26869	0.31	0.059	Biological	GO:00457	positive regulation of gluconeogenesis	0.26869	0.45937	0	0	1	17
GO:0048532 anatomical structure arrangement	1	0.26869	0.31	0.059	Biological	GO:00485	anatomical structure arrangement	0.26869	0.45937	0	0	1	17
GO:0048875 chemical homeostasis within a tissue	1	0.26869	0.31	0.059	Biological	GO:00488	chemical homeostasis within a tissue	0.26869	0.45937	0	0	1	17
GO:0071380 cellular response to prostaglandin E stimulus	1	0.26869	0.31	0.059	Biological	GO:00713	cellular response to prostaglandin E stimulus	0.26869	0.45937	0	0	1	17
GO:0072224 metanephric glomerulus development	1	0.26869	0.31	0.059	Biological	GO:00722	metanephric glomerulus development	0.26869	0.45937	0	0	1	17
GO:0098712 L-glutamate import across plasma membrane	1	0.26869	0.31	0.059	Biological	GO:00987	L-glutamate import across plasma membrane	0.26869	0.45937	0	0	1	17
GO:0098877 neurotransmitter receptor transport to plasma membrane	1	0.26869	0.31	0.059	Biological	GO:00988	neurotransmitter receptor transport to plasma membrane	0.26869	0.45937	0	0	1	17
GO:0099149 regulation of postsynaptic neurotransmitter receptor intern	1	0.26869	0.31	0.059	Biological	GO:00991	regulation of postsynaptic neurotransmitter receptor internalization	0.26869	0.45937	0	0	1	17
GO:0099509 regulation of presynaptic cytosolic calcium ion concentratio	1	0.26869	0.31	0.059	Biological	GO:00995	regulation of presynaptic cytosolic calcium ion concentration	0.26869	0.45937	0	0	1	17
GO:1900034 regulation of cellular response to heat	1	0.26869	0.31	0.059	Biological	GO:19000	regulation of cellular response to heat	0.26869	0.45937	0	0	1	17
GO:1900117 regulation of execution phase of apoptosis	1	0.26869	0.31	0.059	Biological	GO:19001	regulation of execution phase of apoptosis	0.26869	0.45937	0	0	1	17
GO:1903236 regulation of leukocyte tethering or rolling	1	0.26869	0.31	0.059	Biological	GO:19032	regulation of leukocyte tethering or rolling	0.26869	0.45937	0	0	1	17
GO:1903587 regulation of blood vessel endothelial cell proliferation invol	1	0.26869	0.31	0.059	Biological	GO:19035	regulation of blood vessel endothelial cell proliferation involved in spro	0.26869	0.45937	0	0	1	17
GO:1903830 magnesium ion transmembrane transport	1	0.26869	0.31	0.059	Biological	GO:19038	magnesium ion transmembrane transport	0.26869	0.45937	0	0	1	17
GO:1990403 embryonic brain development	1	0.26869	0.31	0.059	Biological	GO:19904	embryonic brain development	0.26869	0.45937	0	0	1	17
GO:2000696 regulation of epithelial cell differentiation involved in kidney	1	0.26869	0.31	0.059	Biological	GO:20006	regulation of epithelial cell differentiation involved in kidney developme	0.26869	0.45937	0	0	1	17
GO:0020037 heme binding	4	0.26986	1.17	0.028	Molecular	GO:00200	heme binding	0.26986	0.46643	0	0	4	142
GO:0051851 modulation by host of symbiont process	3	0.27	0.929	0.03	Biological	GO:00518	modulation by host of symbiont process	0.27	0.46131	0	0	3	99
GO:1901222 regulation of NIK/NF-kappaB signaling	3	0.27	0.929	0.03	Biological	GO:19012	regulation of NIK/NF-kappaB signaling	0.27	0.46131	0	0	3	99
GO:0098632 cell-cell adhesion mediator activity	2	0.27143	0.585	0.036	Molecular	GO:00986	cell-cell adhesion mediator activity	0.27143	0.46643	0	0	2	55
GO:0006383 transcription by RNA polymerase III	2	0.27198	0.619	0.036	Biological	GO:00063	transcription by RNA polymerase III	0.27198	0.46406	0	0	2	56
GO:0010332 response to gamma radiation	2	0.27198	0.619	0.036	Biological	GO:00103	response to gamma radiation	0.27198	0.46406	0	0	2	56
GO:0042987 amyloid precursor protein catabolic process	2	0.27198	0.619	0.036	Biological	GO:00429	amyloid precursor protein catabolic process	0.27198	0.46406	0	0	2	56
GO:0046164 alcohol catabolic process	2	0.27198	0.619	0.036	Biological	GO:00461	alcohol catabolic process	0.27198	0.46406	0	0	2	56
GO:0014065 phosphatidylinositol 3-kinase signaling	4	0.27239	1.238	0.028	Biological	GO:00140	phosphatidylinositol 3-kinase signaling	0.27239	0.46459	0	0	4	145
GO:0005001 transmembrane receptor protein tyrosine phosphatase act	1	0.27253	0.292	0.059	Molecular	GO:00050	transmembrane receptor protein tyrosine phosphatase activity	0.27253	0.46643	0	0	1	17
GO:0005521 lamin binding	1	0.27253	0.292	0.059	Molecular	GO:00055	lamin binding	0.27253	0.46643	0	0	1	17
GO:0008574 plus-end-directed microtubule motor activity	1	0.27253	0.292	0.059	Molecular	GO:00085	plus-end-directed microtubule motor activity	0.27253	0.46643	0	0	1	17
GO:0015095 magnesium ion transmembrane transporter activity	1	0.27253	0.292	0.059	Molecular	GO:00150	magnesium ion transmembrane transporter activity	0.27253	0.46643	0	0	1	17
GO:0015923 mannosidase activity	1	0.27253	0.292	0.059	Molecular	GO:00159	mannosidase activity	0.27253	0.46643	0	0	1	17
GO:0019198 transmembrane receptor protein phosphatase activity	1	0.27253	0.292	0.059	Molecular	GO:00191	transmembrane receptor protein phosphatase activity	0.27253	0.46643	0	0	1	17
GO:0035497 cAMP response element binding	1	0.27253	0.292	0.059	Molecular	GO:00354	cAMP response element binding	0.27253	0.46643	0	0	1	17
GO:0099507 ligand-gated ion channel activity involved in regulation of p	1	0.27253	0.292	0.059	Molecular	GO:00995	ligand-gated ion channel activity involved in regulation of presynaptic	0.27253	0.46643	0	0	1	17
GO:0140327 flippase activity	1	0.27253	0.292	0.059	Molecular	GO:01403	flippase activity	0.27253	0.46643	0	0	1	17
GO:0072659 protein localization to plasma membrane	7	0.27321	2.167	0.024	Biological	GO:00726	protein localization to plasma membrane	0.27321	0.46583	0	0	7	288
GO:0032587 ruffle membrane	3	0.27429	0.89	0.03	Cellular C	GO:00325	ruffle membrane	0.27429	0.49577	0	0	3	101
GO:0001657 ureteric bud development	3	0.27495	0.929	0.03	Biological	GO:00016	ureteric bud development	0.27495	0.46831	0	0	3	100
GO:0006721 terpenoid metabolic process	3	0.27495	0.929	0.03	Biological	GO:00067	terpenoid metabolic process	0.27495	0.46831	0	0	3	100
GO:0070252 actin-mediated cell contraction	3	0.27495	0.929	0.03	Biological	GO:00702	actin-mediated cell contraction	0.27495	0.46831	0	0	3	100
GO:0051346 negative regulation of hydrolase activity	8	0.2763	2.477	0.024	Biological	GO:00513	negative regulation of hydrolase activity	0.2763	0.47045	0	0	8	338
GO:0051656 establishment of organelle localization	10	0.2764	3.096	0.023	Biological	GO:00516	establishment of organelle localization	0.2764	0.47046	0	0	10	437
GO:0030323 respiratory tube development	5	0.27666	1.548	0.026	Biological	GO:00303	respiratory tube development	0.27666	0.47059	0	0	5	193
GO:0052126 movement in host environment	5	0.27666	1.548	0.026	Biological	GO:00521	movement in host environment	0.27666	0.47059	0	0	5	193
GO:0002931 response to ischemia	2	0.27876	0.619	0.035	Biological	GO:00029	response to ischemia	0.27876	0.47106	0	0	2	57
GO:0014743 regulation of muscle hypertrophy	2	0.27876	0.619	0.035	Biological	GO:00147	regulation of muscle hypertrophy	0.27876	0.47106	0	0	2	57
GO:0019369 arachidonic acid metabolic process	2	0.27876	0.619	0.035	Biological	GO:00193	arachidonic acid metabolic process	0.27876	0.47106	0	0	2	57
GO:0035315 hair cell differentiation	2	0.27876	0.619	0.035	Biological	GO:00353	hair cell differentiation	0.27876	0.47106	0	0	2	57
GO:0043331 response to dsRNA	2	0.27876	0.619	0.035	Biological	GO:00433	response to dsRNA	0.27876	0.47106	0	0	2	57
GO:0043388 positive regulation of DNA binding	2	0.27876	0.619	0.035	Biological	GO:00433	positive regulation of DNA binding	0.27876	0.47106	0	0	2	57
GO:0043550 regulation of lipid kinase activity	2	0.27876	0.619	0.035	Biological	GO:00435	regulation of lipid kinase activity	0.27876	0.47106	0	0	2	57
GO:0046456 icosanoid biosynthetic process	2	0.27876	0.619	0.035	Biological	GO:00464	icosanoid biosynthetic process	0.27876	0.47106	0	0	2	57
GO:0050881 musculoskeletal movement	2	0.27876	0.619	0.035	Biological	GO:00508	musculoskeletal movement	0.27876	0.47106	0	0	2	57
GO:0090175 regulation of establishment of planar polarity	2	0.27876	0.619	0.035	Biological	GO:00901	regulation of establishment of planar polarity	0.27876	0.47106	0	0	2	57
GO:0032386 regulation of intracellular transport	8	0.27891	2.477	0.024	Biological	GO:00323	regulation of intracellular transport	0.27891	0.47106	0	0	8	339
GO:0005767 secondary lysosome	1	0.27932	0.297	0.056	Cellular C	GO:00057	secondary lysosome	0.27932	0.49979	0	0	1	18
GO:0016010 dystrophin-associated glycoprotein complex	1	0.27932	0.297	0.056	Cellular C	GO:00160	dystrophin-associated glycoprotein complex	0.27932	0.49979	0	0	1	18
GO:1905286 serine-type peptidase complex	1	0.27932	0.297	0.056	Cellular C	GO:19052	serine-type peptidase complex	0.27932	0.49979	0	0	1	18
GO:0072163 mesonephric epithelium development	3	0.2799	0.929	0.03	Biological	GO:00721	mesonephric epithelium development	0.2799	0.47106	0	0	3	101
GO:0072164 mesonephric tubule development	3	0.2799	0.929	0.03	Biological	GO:00721	mesonephric tubule development	0.2799	0.47106	0	0	3	101
GO:0010508 positive regulation of autophagy	4	0.28049	1.238	0.027	Biological	GO:00105	positive regulation of autophagy	0.28049	0.47106	0	0	4	147
GO:0036294 cellular response to decreased oxygen levels	4	0.28049	1.238	0.027	Biological	GO:00362	cellular response to decreased oxygen levels	0.28049	0.47106	0	0	4	147
GO:0055123 digestive system development	4	0.28049	1.238	0.027	Biological	GO:00551	digestive system development	0.28049	0.47106	0	0	4	147
GO:0009891 positive regulation of biosynthetic process	42	0.28101	13.003	0.02	Biological	GO:00098	positive regulation of biosynthetic process	0.28101	0.47106	0	0	42	2098
GO:0012507 ER to Golgi transport vesicle membrane	2	0.28112	0.593	0.034	Cellular C	GO:00125	ER to Golgi transport vesicle membrane	0.28112	0.50133	0	0	2	58
GO:0042578 phosphoric ester hydrolase activity	9	0.28145	2.632	0.023	Molecular	GO:00425	phosphoric ester hydrolase activity	0.28145	0.47612	0	0	9	383
GO:0002295 T-helper cell lineage commitment	1	0.28203	0.31	0.056	Biological	GO:00022	T-helper cell lineage commitment	0.28203	0.47106	0	0	1	18
GO:0002827 positive regulation of T-helper 1 type immune response	1	0.28203	0.31	0.056	Biological	GO:00028	positive regulation of T-helper 1 type immune response	0.28203	0.47106	0	0	1	18
GO:0003159 morphogenesis of an endothelium	1	0.28203	0.31	0.056	Biological	GO:00031	morphogenesis of an endothelium	0.28203	0.47106	0	0	1	18
GO:0006085 acetyl-CoA biosynthetic process	1	0.28203	0.31	0.056	Biological	GO:00060	acetyl-CoA biosynthetic process	0.28203	0.47106	0	0	1	18
GO:0010829 negative regulation of glucose transmembrane transport	1	0.28203	0.31	0.056	Biological	GO:00108	negative regulation of glucose transmembrane transport	0.28203	0.47106	0	0	1	18
GO:0015693 magnesium ion transport	1	0.28203	0.31	0.056	Biological	GO:00156	magnesium ion transport	0.28203	0.47106	0	0	1	18
GO:0016264 gap junction assembly	1	0.28203	0.31	0.056	Biological	GO:00162	gap junction assembly	0.28203	0.47106	0	0	1	18
GO:0021534 cell proliferation in hindbrain	1	0.28203	0.31	0.056	Biological	GO:00215	cell proliferation in hindbrain	0.28203	0.47106	0	0	1	18
GO:0021924 cell proliferation in external granule layer	1	0.28203	0.31	0.056	Biological	GO:00219	cell proliferation in external granule layer	0.28203	0.47106	0	0	1	18
GO:0021930 cerebellar granule cell precursor proliferation	1	0.28203	0.31	0.056	Biological	GO:00219	cerebellar granule cell precursor proliferation	0.28203	0.47106	0	0	1	18
GO:0032354 response to follicle-stimulating hormone	1	0.28203	0.31	0.056	Biological	GO:00323	response to follicle-stimulating hormone	0.28203	0.47106	0	0	1	18
GO:0032604 granulocyte macrophage colony-stimulating factor product	1	0.28203	0.31	0.056	Biological	GO:00326	granulocyte macrophage colony-stimulating factor production	0.28203	0.47106	0	0	1	18
GO:0032645 regulation of granulocyte macrophage colony-stimulating fa	1	0.28203	0.31	0.056	Biological	GO:00326	regulation of granulocyte macrophage colony-stimulating factor produ	0.28203	0.47106	0	0	1	18
GO:0032930 positive regulation of superoxide anion generation	1	0.28203	0.31	0.056	Biological	GO:00329	positive regulation of superoxide anion generation	0.28203	0.47106	0	0	1	18
GO:0033033 negative regulation of myeloid cell apoptotic process	1	0.28203	0.31	0.056	Biological	GO:00330	negative regulation of myeloid cell apoptotic process	0.28203	0.47106	0	0	1	18
GO:0035234 ectopic germ cell programmed cell death	1	0.28203	0.31	0.056	Biological	GO:00352	ectopic germ cell programmed cell death	0.28203	0.47106	0	0	1	18
GO:0035864 response to potassium ion	1	0.28203	0.31	0.056	Biological	GO:00358	response to potassium ion	0.28203	0.47106	0	0	1	18
GO:0042481 regulation of odontogenesis	1	0.28203	0.31	0.056	Biological	GO:00424	regulation of odontogenesis	0.28203	0.47106	0	0	1	18
GO:0042574 retinal metabolic process	1	0.28203	0.31	0.056	Biological	GO:00425	retinal metabolic process	0.28203	0.47106	0	0	1	18
GO:0043691 reverse cholesterol transport	1	0.28203	0.31	0.056	Biological	GO:00436	reverse cholesterol transport	0.28203	0.47106	0	0	1	18
GO:0043923 positive regulation by host of viral transcription	1	0.28203	0.31	0.056	Biological	GO:00439	positive regulation by host of viral transcription	0.28203	0.47106	0	0	1	18
GO:0045618 positive regulation of keratinocyte differentiation	1	0.28203	0.31	0.056	Biological	GO:00456	positive regulation of keratinocyte differentiation	0.28203	0.47106	0	0	1	18
GO:0048486 parasympathetic nervous system development	1	0.28203	0.31	0.056	Biological	GO:00484	parasympathetic nervous system development	0.28203	0.47106	0	0	1	18
GO:0050802 circadian sleep/wake cycle, sleep	1	0.28203	0.31	0.056	Biological	GO:00508	circadian sleep/wake cycle, sleep	0.28203	0.47106	0	0	1	18
GO:0051770 positive regulation of nitric-oxide synthase biosynthetic pro	1	0.28203	0.31	0.056	Biological	GO:00517	positive regulation of nitric-oxide synthase biosynthetic process	0.28203	0.47106	0	0	1	18
GO:0060088 auditory receptor cell stereocilium organization	1	0.28203	0.31	0.056	Biological	GO:00600	auditory receptor cell stereocilium organization	0.28203	0.47106	0	0	1	18
GO:0061154 endothelial tube morphogenesis	1	0.28203	0.31	0.056	Biological	GO:00611	endothelial tube morphogenesis	0.28203	0.47106	0	0	1	18
GO:0070102 interleukin-6-mediated signaling pathway	1	0.28203	0.31	0.056	Biological	GO:00701	interleukin-6-mediated signaling pathway	0.28203	0.47106	0	0	1	18
GO:0071318 cellular response to ATP	1	0.28203	0.31	0.056	Biological	GO:00713	cellular response to ATP	0.28203	0.47106	0	0	1	18
GO:0071391 cellular response to estrogen stimulus	1	0.28203	0.31	0.056	Biological	GO:00713	cellular response to estrogen stimulus	0.28203	0.47106	0	0	1	18
GO:0072148 epithelial cell fate commitment	1	0.28203	0.31	0.056	Biological	GO:00721	epithelial cell fate commitment	0.28203	0.47106	0	0	1	18
GO:0090520 sphingolipid mediated signaling pathway	1	0.28203	0.31	0.056	Biological	GO:00905	sphingolipid mediated signaling pathway	0.28203	0.47106	0	0	1	18
GO:0097067 cellular response to thyroid hormone stimulus	1	0.28203	0.31	0.056	Biological	GO:00970	cellular response to thyroid hormone stimulus	0.28203	0.47106	0	0	1	18
GO:1900221 regulation of amyloid-beta clearance	1	0.28203	0.31	0.056	Biological	GO:19002	regulation of amyloid-beta clearance	0.28203	0.47106	0	0	1	18
GO:1902430 negative regulation of amyloid-beta formation	1	0.28203	0.31	0.056	Biological	GO:19024	negative regulation of amyloid-beta formation	0.28203	0.47106	0	0	1	18
GO:1903975 regulation of glial cell migration	1	0.28203	0.31	0.056	Biological	GO:19039	regulation of glial cell migration	0.28203	0.47106	0	0	1	18
GO:2000353 positive regulation of endothelial cell apoptotic process	1	0.28203	0.31	0.056	Biological	GO:20003	positive regulation of endothelial cell apoptotic process	0.28203	0.47106	0	0	1	18
GO:0005773 vacuole	18	0.2822	5.341	0.021	Cellular C	GO:00057	vacuole	0.2822	0.50159	0	0	18	855

GO:0004896 cytokine receptor activity	3	0.28323	0.877	0.03	Molecular	GO:00048	cytokine receptor activity	0.28323	0.47612	0	0	3	100
GO:0007163 establishment or maintenance of cell polarity	6	0.28355	1.858	0.025	Biological	GO:00071	establishment or maintenance of cell polarity	0.28355	0.47343	0	0	6	243
GO:0032946 positive regulation of mononuclear cell proliferation	4	0.28455	1.238	0.027	Biological	GO:00329	positive regulation of mononuclear cell proliferation	0.28455	0.4748	0	0	4	148
GO:0050768 negative regulation of neurogenesis	4	0.28455	1.238	0.027	Biological	GO:00507	negative regulation of neurogenesis	0.28455	0.4748	0	0	4	148
GO:0006641 triglyceride metabolic process	3	0.28487	0.929	0.029	Biological	GO:00066	triglyceride metabolic process	0.28487	0.47484	0	0	3	102
GO:0014013 regulation of gliogenesis	3	0.28487	0.929	0.029	Biological	GO:00140	regulation of gliogenesis	0.28487	0.47484	0	0	3	102
GO:0042158 lipoprotein biosynthetic process	3	0.28487	0.929	0.029	Biological	GO:00421	lipoprotein biosynthetic process	0.28487	0.47484	0	0	3	102
GO:0016655 oxidoreductase activity, acting on NAD(P)H, quinone or sir	2	0.28521	0.585	0.035	Molecular	GO:00166	oxidoreductase activity, acting on NAD(P)H, quinone or similar comp	0.28521	0.47612	0	0	2	57
GO:0001541 ovarian follicle development	2	0.28553	0.619	0.034	Biological	GO:00015	ovarian follicle development	0.28553	0.47516	0	0	2	58
GO:0006767 water-soluble vitamin metabolic process	2	0.28553	0.619	0.034	Biological	GO:00067	water-soluble vitamin metabolic process	0.28553	0.47516	0	0	2	58
GO:0043407 negative regulation of MAP kinase activity	2	0.28553	0.619	0.034	Biological	GO:00434	negative regulation of MAP kinase activity	0.28553	0.47516	0	0	2	58
GO:0050879 multicellular organismal movement	2	0.28553	0.619	0.034	Biological	GO:00508	multicellular organismal movement	0.28553	0.47516	0	0	2	58
GO:0051293 establishment of spindle localization	2	0.28553	0.619	0.034	Biological	GO:00512	establishment of spindle localization	0.28553	0.47516	0	0	2	58
GO:0034399 nuclear periphery	4	0.28586	1.187	0.027	Cellular C	GO:00343	nuclear periphery	0.28586	0.5064	0	0	4	150
GO:0004115 3',5'-cyclic-AMP phosphodiesterase activity	1	0.28603	0.292	0.056	Molecular	GO:00041	3',5'-cyclic-AMP phosphodiesterase activity	0.28603	0.47612	0	0	1	18
GO:0008970 phospholipase A1 activity	1	0.28603	0.292	0.056	Molecular	GO:00089	phospholipase A1 activity	0.28603	0.47612	0	0	1	18
GO:0015172 acidic amino acid transmembrane transporter activity	1	0.28603	0.292	0.056	Molecular	GO:00151	acidic amino acid transmembrane transporter activity	0.28603	0.47612	0	0	1	18
GO:0015556 C4-dicarboxylate transmembrane transporter activity	1	0.28603	0.292	0.056	Molecular	GO:00155	C4-dicarboxylate transmembrane transporter activity	0.28603	0.47612	0	0	1	18
GO:0016854 racemase and epimerase activity	1	0.28603	0.292	0.056	Molecular	GO:00168	racemase and epimerase activity	0.28603	0.47612	0	0	1	18
GO:0034185 apolipoprotein binding	1	0.28603	0.292	0.056	Molecular	GO:00341	apolipoprotein binding	0.28603	0.47612	0	0	1	18
GO:0043395 heparan sulfate proteoglycan binding	1	0.28603	0.292	0.056	Molecular	GO:00433	heparan sulfate proteoglycan binding	0.28603	0.47612	0	0	1	18
GO:0050811 GABA receptor binding	1	0.28603	0.292	0.056	Molecular	GO:00508	GABA receptor binding	0.28603	0.47612	0	0	1	18
GO:0099186 structural constituent of postsynapse	1	0.28603	0.292	0.056	Molecular	GO:00991	structural constituent of postsynapse	0.28603	0.47612	0	0	1	18
GO:0015276 ligand-gated ion channel activity	4	0.28635	1.17	0.027	Molecular	GO:00152	ligand-gated ion channel activity	0.28635	0.47612	0	0	4	146
GO:0022834 ligand-gated channel activity	4	0.28635	1.17	0.027	Molecular	GO:00228	ligand-gated channel activity	0.28635	0.47612	0	0	4	146
GO:0015631 tubulin binding	9	0.28644	2.632	0.023	Molecular	GO:00156	tubulin binding	0.28644	0.47612	0	0	9	385
GO:0043491 protein kinase B signaling	5	0.28719	1.548	0.026	Biological	GO:00434	protein kinase B signaling	0.28719	0.47775	0	0	5	196
GO:0001775 cell activation	24	0.2874	7.43	0.021	Biological	GO:00017	cell activation	0.2874	0.47781	0	0	24	1159
GO:1903532 positive regulation of secretion by cell	7	0.28742	2.167	0.024	Biological	GO:19035	positive regulation of secretion by cell	0.28742	0.47781	0	0	7	293
GO:0030660 Golgi-associated vesicle membrane	2	0.28781	0.593	0.034	Cellular C	GO:00306	Golgi-associated vesicle membrane	0.28781	0.50818	0	0	2	59
GO:0061135 endopeptidase regulator activity	5	0.28799	1.462	0.026	Molecular	GO:00611	endopeptidase regulator activity	0.28799	0.47777	0	0	5	193
GO:0015908 fatty acid transport	3	0.28984	0.929	0.029	Biological	GO:00159	fatty acid transport	0.28984	0.48152	0	0	3	103
GO:0060349 bone morphogenesis	3	0.28984	0.929	0.029	Biological	GO:00603	bone morphogenesis	0.28984	0.48152	0	0	3	103
GO:0005261 cation channel activity	8	0.29119	2.339	0.024	Molecular	GO:00052	cation channel activity	0.29119	0.48216	0	0	8	338
GO:1901682 sulfur compound transmembrane transporter activity	2	0.29209	0.585	0.034	Molecular	GO:19016	sulfur compound transmembrane transporter activity	0.29209	0.48273	0	0	2	58
GO:0046686 response to cadmium ion	2	0.2923	0.619	0.034	Biological	GO:00466	response to cadmium ion	0.2923	0.48309	0	0	2	59
GO:0051353 positive regulation of oxidoreductase activity	2	0.2923	0.619	0.034	Biological	GO:00513	positive regulation of oxidoreductase activity	0.2923	0.48309	0	0	2	59
GO:0099522 cytosolic region	1	0.29233	0.297	0.053	Cellular C	GO:00995	cytosolic region	0.29233	0.51446	0	0	1	19
GO:0051607 defense response to virus	7	0.29315	2.167	0.024	Biological	GO:00516	defense response to virus	0.29315	0.48309	0	0	7	295
GO:0140546 defense response to symbiont	7	0.29315	2.167	0.024	Biological	GO:01405	defense response to symbiont	0.29315	0.48309	0	0	7	295
GO:0042102 positive regulation of T cell proliferation	3	0.29481	0.929	0.029	Biological	GO:00421	positive regulation of T cell proliferation	0.29481	0.48309	0	0	3	104
GO:0090288 negative regulation of cellular response to growth factor sti	3	0.29481	0.929	0.029	Biological	GO:00902	negative regulation of cellular response to growth factor stimulus	0.29481	0.48309	0	0	3	104
GO:0031410 cytoplasmic vesicle	49	0.29504	14.54	0.02	Cellular C	GO:00314	cytoplasmic vesicle	0.29504	0.51753	0	0	49	2510
GO:0001845 phagolysosome assembly	1	0.29513	0.31	0.053	Biological	GO:00018	phagolysosome assembly	0.29513	0.48309	0	0	1	19
GO:0002283 neutrophil activation involved in immune response	1	0.29513	0.31	0.053	Biological	GO:00022	neutrophil activation involved in immune response	0.29513	0.48309	0	0	1	19
GO:0002483 antigen processing and presentation of endogenous peptic	1	0.29513	0.31	0.053	Biological	GO:00024	antigen processing and presentation of endogenous peptide antigen	0.29513	0.48309	0	0	1	19
GO:0003215 cardiac right ventricle morphogenesis	1	0.29513	0.31	0.053	Biological	GO:00032	cardiac right ventricle morphogenesis	0.29513	0.48309	0	0	1	19
GO:0006044 N-acetylglucosamine metabolic process	1	0.29513	0.31	0.053	Biological	GO:00060	N-acetylglucosamine metabolic process	0.29513	0.48309	0	0	1	19
GO:0006309 apoptotic DNA fragmentation	1	0.29513	0.31	0.053	Biological	GO:00063	apoptotic DNA fragmentation	0.29513	0.48309	0	0	1	19
GO:0007617 mating behavior	1	0.29513	0.31	0.053	Biological	GO:00076	mating behavior	0.29513	0.48309	0	0	1	19
GO:0009070 serine family amino acid biosynthetic process	1	0.29513	0.31	0.053	Biological	GO:00090	serine family amino acid biosynthetic process	0.29513	0.48309	0	0	1	19
GO:0010544 negative regulation of platelet activation	1	0.29513	0.31	0.053	Biological	GO:00105	negative regulation of platelet activation	0.29513	0.48309	0	0	1	19
GO:0010759 positive regulation of macrophage chemotaxis	1	0.29513	0.31	0.053	Biological	GO:00107	positive regulation of macrophage chemotaxis	0.29513	0.48309	0	0	1	19
GO:0010878 cholesterol storage	1	0.29513	0.31	0.053	Biological	GO:00108	cholesterol storage	0.29513	0.48309	0	0	1	19
GO:0014048 regulation of glutamate secretion	1	0.29513	0.31	0.053	Biological	GO:00140	regulation of glutamate secretion	0.29513	0.48309	0	0	1	19
GO:0016553 base conversion or substitution editing	1	0.29513	0.31	0.053	Biological	GO:00165	base conversion or substitution editing	0.29513	0.48309	0	0	1	19
GO:0021511 spinal cord patterning	1	0.29513	0.31	0.053	Biological	GO:00215	spinal cord patterning	0.29513	0.48309	0	0	1	19
GO:0030502 negative regulation of bone mineralization	1	0.29513	0.31	0.053	Biological	GO:00305	negative regulation of bone mineralization	0.29513	0.48309	0	0	1	19
GO:0031645 negative regulation of nervous system process	1	0.29513	0.31	0.053	Biological	GO:00316	negative regulation of nervous system process	0.29513	0.48309	0	0	1	19
GO:0032328 alanine transport	1	0.29513	0.31	0.053	Biological	GO:00323	alanine transport	0.29513	0.48309	0	0	1	19
GO:0032793 positive regulation of CREB transcription factor activity	1	0.29513	0.31	0.053	Biological	GO:00327	positive regulation of CREB transcription factor activity	0.29513	0.48309	0	0	1	19
GO:0036003 positive regulation of transcription from RNA polymerase II	1	0.29513	0.31	0.053	Biological	GO:00360	positive regulation of transcription from RNA polymerase II promoter	0.29513	0.48309	0	0	1	19
GO:0043116 negative regulation of vascular permeability	1	0.29513	0.31	0.053	Biological	GO:00431	negative regulation of vascular permeability	0.29513	0.48309	0	0	1	19
GO:0043517 positive regulation of DNA damage response, signal transc	1	0.29513	0.31	0.053	Biological	GO:00435	positive regulation of DNA damage response, signal transduction by	0.29513	0.48309	0	0	1	19
GO:0045540 regulation of cholesterol biosynthetic process	1	0.29513	0.31	0.053	Biological	GO:00455	regulation of cholesterol biosynthetic process	0.29513	0.48309	0	0	1	19
GO:0045655 regulation of monocyte differentiation	1	0.29513	0.31	0.053	Biological	GO:00456	regulation of monocyte differentiation	0.29513	0.48309	0	0	1	19
GO:0045945 positive regulation of transcription by RNA polymerase III	1	0.29513	0.31	0.053	Biological	GO:00459	positive regulation of transcription by RNA polymerase III	0.29513	0.48309	0	0	1	19
GO:0048245 eosinophil chemotaxis	1	0.29513	0.31	0.053	Biological	GO:00482	eosinophil chemotaxis	0.29513	0.48309	0	0	1	19
GO:0050965 detection of temperature stimulus involved in sensory perc	1	0.29513	0.31	0.053	Biological	GO:00509	detection of temperature stimulus involved in sensory perception of p	0.29513	0.48309	0	0	1	19
GO:0051938 L-glutamate import	1	0.29513	0.31	0.053	Biological	GO:00519	L-glutamate import	0.29513	0.48309	0	0	1	19
GO:0051988 regulation of attachment of spindle microtubules to kinetoc	1	0.29513	0.31	0.053	Biological	GO:00519	regulation of attachment of spindle microtubules to kinetochore	0.29513	0.48309	0	0	1	19
GO:0071360 cellular response to exogenous dsRNA	1	0.29513	0.31	0.053	Biological	GO:00713	cellular response to exogenous dsRNA	0.29513	0.48309	0	0	1	19
GO:0071786 endoplasmic reticulum tubular network organization	1	0.29513	0.31	0.053	Biological	GO:00717	endoplasmic reticulum tubular network organization	0.29513	0.48309	0	0	1	19
GO:0071800 podosome assembly	1	0.29513	0.31	0.053	Biological	GO:00718	podosome assembly	0.29513	0.48309	0	0	1	19
GO:0097094 craniofacial suture morphogenesis	1	0.29513	0.31	0.053	Biological	GO:00970	craniofacial suture morphogenesis	0.29513	0.48309	0	0	1	19
GO:0097202 activation of cysteine-type endopeptidase activity	1	0.29513	0.31	0.053	Biological	GO:00972	activation of cysteine-type endopeptidase activity	0.29513	0.48309	0	0	1	19
GO:0106118 regulation of sterol biosynthetic process	1	0.29513	0.31	0.053	Biological	GO:01061	regulation of sterol biosynthetic process	0.29513	0.48309	0	0	1	19
GO:1901659 glycosyl compound biosynthetic process	1	0.29513	0.31	0.053	Biological	GO:19016	glycosyl compound biosynthetic process	0.29513	0.48309	0	0	1	19
GO:1904862 inhibitory synapse assembly	1	0.29513	0.31	0.053	Biological	GO:19048	inhibitory synapse assembly	0.29513	0.48309	0	0	1	19
GO:1905146 lysosomal protein catabolic process	1	0.29513	0.31	0.053	Biological	GO:19051	lysosomal protein catabolic process	0.29513	0.48309	0	0	1	19
GO:2001135 regulation of endocytic recycling	1	0.29513	0.31	0.053	Biological	GO:20011	regulation of endocytic recycling	0.29513	0.48309	0	0	1	19
GO:2001185 regulation of CD8-positive, alpha-beta T cell activation	1	0.29513	0.31	0.053	Biological	GO:20011	regulation of CD8-positive, alpha-beta T cell activation	0.29513	0.48309	0	0	1	19
GO:0045834 positive regulation of lipid metabolic process	4	0.2968	1.238	0.026	Biological	GO:00458	positive regulation of lipid metabolic process	0.2968	0.48565	0	0	4	151
GO:0002682 regulation of immune system process	31	0.29694	9.598	0.02	Biological	GO:00026	regulation of immune system process	0.29694	0.48572	0	0	31	1532
GO:0045787 positive regulation of cell cycle	8	0.2974	2.477	0.023	Biological	GO:00457	positive regulation of cell cycle	0.2974	0.48631	0	0	8	346
GO:0097708 intracellular vesicle	49	0.29809	14.54	0.019	Cellular C	GO:00977	intracellular vesicle	0.29809	0.52117	0	0	49	2513
GO:0016279 protein-lysine N-methyltransferase activity	2	0.29896	0.585	0.034	Molecular	GO:00162	protein-lysine N-methyltransferase activity	0.29896	0.48808	0	0	2	59
GO:0002886 regulation of myeloid leukocyte mediated immunity	2	0.29906	0.619	0.033	Biological	GO:00028	regulation of myeloid leukocyte mediated immunity	0.29906	0.48823	0	0	2	60
GO:0019748 secondary metabolic process	2	0.29906	0.619	0.033	Biological	GO:00197	secondary metabolic process	0.29906	0.48823	0	0	2	60
GO:0021545 cranial nerve development	2	0.29906	0.619	0.033	Biological	GO:00215	cranial nerve development	0.29906	0.48823	0	0	2	60
GO:0034113 heterotypic cell-cell adhesion	2	0.29906	0.619	0.033	Biological	GO:00341	heterotypic cell-cell adhesion	0.29906	0.48823	0	0	2	60
GO:0071709 membrane assembly	2	0.29906	0.619	0.033	Biological	GO:00717	membrane assembly	0.29906	0.48823	0	0	2	60
GO:0004745 NAD-retinol dehydrogenase activity	1	0.29927	0.292	0.053	Molecular	GO:00047	NAD-retinol dehydrogenase activity	0.29927	0.48808	0	0	1	19
GO:0004970 ionotropic glutamate receptor activity	1	0.29927	0.292	0.053	Molecular	GO:00049	ionotropic glutamate receptor activity	0.29927	0.48808	0	0	1	19
GO:0005326 neurotransmitter transmembrane transporter activity	1	0.29927	0.292	0.053	Molecular	GO:00053	neurotransmitter transmembrane transporter activity	0.29927	0.48808	0	0	1	19
GO:0030506 ankyrin binding	1	0.29927	0.292	0.053	Molecular	GO:00305	ankyrin binding	0.29927	0.48808	0	0	1	19
GO:0036122 BMP binding	1	0.29927	0.292	0.053	Molecular	GO:00361	BMP binding	0.29927	0.48808	0	0	1	19
GO:0046527 glucosyltransferase activity	1	0.29927	0.292	0.053	Molecular	GO:00465	glucosyltransferase activity	0.29927	0.48808	0	0	1	19
GO:0001823 mesonephros development	3	0.29979	0.929	0.029	Biological	GO:00018	mesonephros development	0.29979	0.48832	0	0	3	105
GO:0006664 glycolipid metabolic process	3	0.29979	0.929	0.029	Biological	GO:00066	glycolipid metabolic process	0.29979	0.48832	0	0	3	105
GO:0006672 ceramide metabolic process	3	0.29979	0.929	0.029	Biological	GO:00066	ceramide metabolic process	0.29979	0.48832	0	0	3	105
GO:0030510 regulation of BMP signaling pathway	3	0.29979	0.929	0.029	Biological	GO:00305	regulation of BMP signaling pathway	0.29979	0.48832	0	0	3	105
GO:0030593 neutrophil chemotaxis	3	0.29979	0.929	0.029	Biological	GO:00305	neutrophil chemotaxis	0.29979	0.48832	0	0	3	105
GO:0043500 muscle adaptation	3	0.29979	0.929	0.029	Biological	GO:00435	muscle adaptation	0.29979	0.48832	0	0	3	105
GO:0090263 positive regulation of canonical Wnt signaling pathway	3	0.29979	0.929	0.029	Biological	GO:00902	positive regulation of canonical Wnt signaling pathway	0.29979	0.48832	0	0	3	105
GO:0005216 ion channel activity	10	0.30046	2.924	0.023	Molecular	GO:00052	ion channel activity	0.30046	0.4891	0	0	10	440
GO:0000791 euchromatin	2	0.30118	0.593	0.033	Cellular C	GO:00007	euchromatin	0.30118	0.52315	0	0	2	61
GO:0035580 specific granule lumen	2	0.30118	0.593	0.033	Cellular C	GO:00355	specific granule lumen	0.30118	0.52315	0	0	2	61
GO:0046942 carboxylic acid transport	7	0.30179	2.167	0.023	Biological	GO:00469	carboxylic acid transport	0.30179	0.4914	0	0	7	298
GO:0019842 vitamin binding	4	0.30297	1.17	0.027	Molecular	GO:00198	vitamin binding	0.30297	0.49226	0	0	4	150
GO:0098660 inorganic ion transmembrane transport	20	0.30386	6.192	0.021	Biological	GO:00986	inorganic ion transmembrane transport	0.30386	0.49315	0	0	20	962
GO:0008637 apoptotic mitochondrial changes	3	0.30478	0.929	0.028	Biological	GO:00086	apoptotic mitochondrial changes	0.30478	0.49315	0	0	3	106
GO:0048477 oogenesis	3	0.30478	0.929	0.028	Biological	GO:00484	oogenesis	0.30478	0.49315	0	0	3	106
GO:0051817 modulation of process of other organism involved in symbi	3	0.30478	0.929	0.028	Biological	GO:00518	modulation of process of other organism involved in symbiotic interac	0.30478	0.49315	0	0	3	106
GO:1903509 liposaccharide metabolic process	3	0.30478	0.929	0.028	Biological	GO:19035	liposaccharide metabolic process	0.30478	0.49315	0	0	3	106
GO:0006836 neurotransmitter transport	5	0.30489	1.548	0.025	Biological	GO:00068	neurotransmitter transport	0.30489	0.49315	0	0	5	201
GO:0031032 actomyosin structure organization	5	0.30489	1.548	0.025	Biological	GO:00310	actomyosin structure organization	0.30489	0.49315	0	0	5	201
GO:0071674 mononuclear cell migration	5	0.30489	1.548	0.025	Biological	GO:00716	mononuclear cell migration	0.30489	0.49315	0	0	5	201
GO:0008360 regulation of cell shape	4	0.305	1.238	0.026	Biological	GO:00083	regulation of cell shape	0.305	0.49315	0	0	4	153
GO:0010951 negative regulation of endopeptidase activity	4	0.305	1.238	0.026	Biological	GO:00109	negative regulation of endopeptidase activity	0.305	0.49315	0	0	4	153
GO:0030856 regulation of epithelial cell differentiation	4	0.305	1.238	0.026	Biological	GO:00308	regulation of epithelial cell differentiation	0.305	0.49315	0	0	4	153
GO:0043194 axon initial segment	1	0.3051	0.297	0.05	Cellular C	GO:00431	axon initial segment	0.3051	0.52654	0	0	1	20
GO:0120293 dynein axonemal particle	1	0.3051	0.297	0.05	Cellular C	GO:01202	dynein axonemal particle	0.3051	0.52654	0	0	1	20
GO:0002237 response to molecule of bacterial origin	8	0.30541	2.477	0.023	Biological	GO:00022	response to molecule of bacterial origin	0.30541	0.49315	0	0	8	349
GO:0007265 Ras protein signal transduction	8	0.30541	2.477	0.023	Biological	GO:00072	Ras protein signal transduction	0.30541	0.49315	0	0	8	349
GO:0006814 sodium ion transport	6	0.30564	1.858	0.024	Biological	GO:00068	sodium ion transport	0.30564	0.49315	0	0	6	250
GO:0006940 regulation of smooth muscle contraction	2	0.3058	0.619	0.033	Biological	GO:00069	regulation of smooth muscle contraction	0.3058	0.49315	0	0	2	61
GO:0060350 endochondral bone morphogenesis	2	0.3058	0.619	0.033	Biological	GO:00603	endochondral bone morphogenesis	0.3058	0.49315	0	0	2	61
GO:1900024 regulation of substrate adhesion-dependent cell spreading	2	0.3058	0.619	0.033	Biological	GO:19000	regulation of substrate adhesion-dependent cell spreading	0.3058	0.49315	0	0	2	61
GO:1902305 regulation of sodium ion transmembrane transport	2	0.3058	0.619	0.033	Biological	GO:19023	regulation of sodium ion transmembrane transport	0.3058	0.49315	0	0	2	61
GO:1903078 positive regulation of protein localization to plasma membra	2	0.3058	0.619	0.033	Biological	GO:19030	positive regulation of protein localization to plasma membrane	0.3058	0.49315	0	0	2	61
GO:0016278 lysine N-methyltransferase activity	2	0.30582	0.585	0.033	Molecular	GO:00162	lysine N-methyltransferase activity	0.30582	0.49411	0	0	2	60
GO:0016667 oxidoreductase activity, acting on a sulfur group of donors	2	0.30582	0.585	0.033	Molecular	GO:00166	oxidoreductase activity, acting on a sulfur group of donors	0.30582	0.49411	0	0	2	60
GO:0044183 protein folding chaperone	2	0.30582	0.585	0.033	Molecular	GO:00441	protein folding chaperone	0.30582	0.49411	0	0	2	60
GO:0002093 auditory receptor cell morphogenesis	1	0.308	0.31	0.05	Biological	GO:00020	auditory receptor cell morphogenesis	0.308	0.49315	0	0	1	20
GO:0002689 negative regulation of leukocyte chemotaxis	1	0.308	0.31	0.05	Biological	GO:00026	negative regulation of leukocyte chemotaxis	0.308	0.49315	0	0	1	20
GO:0003417 growth plate cartilage development	1	0.308	0.31	0.05	Biological	GO:00034	growth plate cartilage development	0.308	0.49315	0	0	1	20
GO:0006067 ethanol metabolic process	1	0.308	0.31	0.05	Biological	GO:00060	ethanol metabolic process	0.308	0.49315	0	0	1	20
GO:0006144 purine nucleobase metabolic process	1	0.308	0.31	0.05	Biological	GO:00061	purine nucleobase metabolic process	0.308	0.49315	0	0	1	20
GO:0006662 glycerol ether metabolic process	1	0.308	0.31	0.05	Biological	GO:00066	glycerol ether metabolic process	0.308	0.49315	0	0	1	20
GO:0007398 ectoderm development	1	0.308	0.31	0.05	Biological	GO:00073	ectoderm development	0.308	0.49315	0	0	1	20
GO:0008356 asymmetric cell division	1	0.308	0.31	0.05	Biological	GO:00083	asymmetric cell division	0.308	0.49315	0	0	1	20
GO:0010866 regulation of triglyceride biosynthetic process	1	0.308	0.31	0.05	Biological	GO:00108	regulation of triglyceride biosynthetic process	0.308	0.49315	0	0	1	20
GO:0014856 skeletal muscle cell proliferation	1	0.308	0.31	0.05	Biological	GO:00148	skeletal muscle cell proliferation	0.308	0.49315	0	0	1	20
GO:0030002 cellular anion homeostasis	1	0.308	0.31	0.05	Biological	GO:00300	cellular anion homeostasis	0.308	0.49315	0	0	1	20
GO:0030150 protein import into mitochondrial matrix	1	0.308	0.31	0.05	Biological	GO:00301	protein import into mitochondrial matrix	0.308	0.49315	0	0	1	20
GO:0032495 response to muramyl dipeptide	1	0.308	0.31	0.05	Biological	GO:00324	response to muramyl dipeptide	0.308	0.49315	0	0	1	20
GO:0042749 regulation of circadian sleep/wake cycle	1	0.308	0.31	0.05	Biological	GO:00427	regulation of circadian sleep/wake cycle	0.308	0.49315	0	0	1	20
GO:0046485 ether lipid metabolic process	1	0.308	0.31	0.05	Biological	GO:00464	ether lipid metabolic process	0.308	0.49315	0	0	1	20
GO:0046931 pore complex assembly	1	0.308	0.31	0.05	Biological	GO:00469	pore complex assembly	0.308	0.49315	0	0	1	20
GO:0048569 post-embryonic animal organ development	1	0.308	0.31	0.05	Biological	GO:00485	post-embryonic animal organ development	0.308	0.49315	0	0	1	20
GO:0050849 negative regulation of calcium-mediated signaling	1	0.308	0.31	0.05	Biological	GO:00508	negative regulation of calcium-mediated signaling	0.308	0.49315	0	0	1	20

GO:0051546 keratinocyte migration	1	0.308	0.31	0.05	Biological	GO:00515	keratinocyte migration	0.308	0.49315	0	0	1	20
GO:0051797 regulation of hair follicle development	1	0.308	0.31	0.05	Biological	GO:00517	regulation of hair follicle development	0.308	0.49315	0	0	1	20
GO:0060004 reflex	1	0.308	0.31	0.05	Biological	GO:00600	reflex	0.308	0.49315	0	0	1	20
GO:0060231 mesenchymal to epithelial transition	1	0.308	0.31	0.05	Biological	GO:00602	mesenchymal to epithelial transition	0.308	0.49315	0	0	1	20
GO:0060340 positive regulation of type I interferon-mediated signaling p	1	0.308	0.31	0.05	Biological	GO:00603	positive regulation of type I interferon-mediated signaling pathway	0.308	0.49315	0	0	1	20
GO:0071371 cellular response to gonadotropin stimulus	1	0.308	0.31	0.05	Biological	GO:00713	cellular response to gonadotropin stimulus	0.308	0.49315	0	0	1	20
GO:0071605 monocyte chemotactic protein-1 production	1	0.308	0.31	0.05	Biological	GO:00716	monocyte chemotactic protein-1 production	0.308	0.49315	0	0	1	20
GO:0071637 regulation of monocyte chemotactic protein-1 production	1	0.308	0.31	0.05	Biological	GO:00716	regulation of monocyte chemotactic protein-1 production	0.308	0.49315	0	0	1	20
GO:0097320 plasma membrane tubulation	1	0.308	0.31	0.05	Biological	GO:00973	plasma membrane tubulation	0.308	0.49315	0	0	1	20
GO:0098543 detection of other organism	1	0.308	0.31	0.05	Biological	GO:00985	detection of other organism	0.308	0.49315	0	0	1	20
GO:1900227 positive regulation of NLRP3 inflammasome complex asse	1	0.308	0.31	0.05	Biological	GO:19002	positive regulation of NLRP3 inflammasome complex assembly	0.308	0.49315	0	0	1	20
GO:2000010 positive regulation of protein localization to cell surface	1	0.308	0.31	0.05	Biological	GO:20000	positive regulation of protein localization to cell surface	0.308	0.49315	0	0	1	20
GO:2000434 regulation of protein neddylation	1	0.308	0.31	0.05	Biological	GO:20004	regulation of protein neddylation	0.308	0.49315	0	0	1	20
GO:2000810 regulation of bicellular tight junction assembly	1	0.308	0.31	0.05	Biological	GO:20008	regulation of bicellular tight junction assembly	0.308	0.49315	0	0	1	20
GO:2001014 regulation of skeletal muscle cell differentiation	1	0.308	0.31	0.05	Biological	GO:20010	regulation of skeletal muscle cell differentiation	0.308	0.49315	0	0	1	20
GO:0071248 cellular response to metal ion	5	0.30845	1.548	0.025	Biological	GO:00712	cellular response to metal ion	0.30845	0.49372	0	0	5	202
GO:0004497 monooxygenase activity	3	0.30854	0.877	0.029	Molecular	GO:00044	monooxygenase activity	0.30854	0.49758	0	0	3	105
GO:0009411 response to UV	4	0.3091	1.238	0.026	Biological	GO:00094	response to UV	0.3091	0.4943	0	0	4	154
GO:0051961 negative regulation of nervous system development	4	0.3091	1.238	0.026	Biological	GO:00519	negative regulation of nervous system development	0.3091	0.4943	0	0	4	154
GO:0009617 response to bacterium	16	0.30911	4.954	0.021	Biological	GO:00096	response to bacterium	0.30911	0.4943	0	0	16	758
GO:0021549 cerebellum development	3	0.30977	0.929	0.028	Biological	GO:00215	cerebellum development	0.30977	0.49504	0	0	3	107
GO:1901890 positive regulation of cell junction assembly	3	0.30977	0.929	0.028	Biological	GO:19018	positive regulation of cell junction assembly	0.30977	0.49504	0	0	3	107
GO:0022836 gated channel activity	8	0.31017	2.339	0.023	Molecular	GO:00228	gated channel activity	0.31017	0.49774	0	0	8	345
GO:0046906 tetrapyrrole binding	4	0.31133	1.17	0.026	Molecular	GO:00469	tetrapyrrole binding	0.31133	0.49774	0	0	4	152
GO:0016773 phosphotransferase activity, alcohol group as acceptor	15	0.31143	4.386	0.022	Molecular	GO:00167	phosphotransferase activity, alcohol group as acceptor	0.31143	0.49774	0	0	15	696
GO:0032870 cellular response to hormone stimulus	13	0.31159	4.025	0.021	Biological	GO:00328	cellular response to hormone stimulus	0.31159	0.49778	0	0	13	605
GO:0016755 aminoacyltransferase activity	1	0.31227	0.292	0.05	Molecular	GO:00167	aminoacyltransferase activity	0.31227	0.49774	0	0	1	20
GO:0070300 phosphatidic acid binding	1	0.31227	0.292	0.05	Molecular	GO:00703	phosphatidic acid binding	0.31227	0.49774	0	0	1	20
GO:0001658 branching involved in ureteric bud morphogenesis	2	0.31254	0.619	0.032	Biological	GO:00016	branching involved in ureteric bud morphogenesis	0.31254	0.49866	0	0	2	62
GO:0050922 negative regulation of chemotaxis	2	0.31254	0.619	0.032	Biological	GO:00509	negative regulation of chemotaxis	0.31254	0.49866	0	0	2	62
GO:0060393 regulation of pathway-restricted SMAD protein phosphoryl	2	0.31254	0.619	0.032	Biological	GO:00603	regulation of pathway-restricted SMAD protein phosphorylation	0.31254	0.49866	0	0	2	62
GO:0072348 sulfur compound transport	2	0.31254	0.619	0.032	Biological	GO:00723	sulfur compound transport	0.31254	0.49866	0	0	2	62
GO:0001098 basal transcription machinery binding	2	0.31267	0.585	0.033	Molecular	GO:00010	basal transcription machinery binding	0.31267	0.49774	0	0	2	61
GO:0001099 basal RNA polymerase II transcription machinery binding	2	0.31267	0.585	0.033	Molecular	GO:00010	basal RNA polymerase II transcription machinery binding	0.31267	0.49774	0	0	2	61
GO:0007159 leukocyte cell-cell adhesion	9	0.31269	2.786	0.022	Biological	GO:00071	leukocyte cell-cell adhesion	0.31269	0.49875	0	0	9	402
GO:0031331 positive regulation of cellular catabolic process	10	0.31373	3.096	0.022	Biological	GO:00313	positive regulation of cellular catabolic process	0.31373	0.50024	0	0	10	453
GO:0032147 activation of protein kinase activity	3	0.31476	0.929	0.028	Biological	GO:00321	activation of protein kinase activity	0.31476	0.50173	0	0	3	108
GO:0005244 voltage-gated ion channel activity	5	0.31688	1.462	0.025	Molecular	GO:00052	voltage-gated ion channel activity	0.31688	0.50352	0	0	5	201
GO:0090316 positive regulation of intracellular protein transport	4	0.31734	1.238	0.026	Biological	GO:00903	positive regulation of intracellular protein transport	0.31734	0.50226	0	0	4	156
GO:0060077 inhibitory synapse	1	0.31763	0.297	0.048	Cellular C	GO:00600	inhibitory synapse	0.31763	0.54641	0	0	1	21
GO:0006355 regulation of transcription, DNA-templated	67	0.31842	20.743	0.019	Biological	GO:00063	regulation of transcription, DNA-templated	0.31842	0.50226	0	0	67	3469
GO:0090575 RNA polymerase II transcription regulator complex	6	0.3188	1.78	0.023	Cellular C	GO:00905	RNA polymerase II transcription regulator complex	0.3188	0.54666	0	0	6	257
GO:0000768 syncytium formation by plasma membrane fusion	2	0.31926	0.619	0.032	Biological	GO:00007	syncytium formation by plasma membrane fusion	0.31926	0.50226	0	0	2	63
GO:0002712 regulation of B cell mediated immunity	2	0.31926	0.619	0.032	Biological	GO:00027	regulation of B cell mediated immunity	0.31926	0.50226	0	0	2	63
GO:0002889 regulation of immunoglobulin mediated immune response	2	0.31926	0.619	0.032	Biological	GO:00028	regulation of immunoglobulin mediated immune response	0.31926	0.50226	0	0	2	63
GO:0022029 telencephalon cell migration	2	0.31926	0.619	0.032	Biological	GO:00220	telencephalon cell migration	0.31926	0.50226	0	0	2	63
GO:0030865 cortical cytoskeleton organization	2	0.31926	0.619	0.032	Biological	GO:00308	cortical cytoskeleton organization	0.31926	0.50226	0	0	2	63
GO:0031663 lipopolysaccharide-mediated signaling pathway	2	0.31926	0.619	0.032	Biological	GO:00316	lipopolysaccharide-mediated signaling pathway	0.31926	0.50226	0	0	2	63
GO:0034605 cellular response to heat	2	0.31926	0.619	0.032	Biological	GO:00346	cellular response to heat	0.31926	0.50226	0	0	2	63
GO:0035306 positive regulation of dephosphorylation	2	0.31926	0.619	0.032	Biological	GO:00353	positive regulation of dephosphorylation	0.31926	0.50226	0	0	2	63
GO:0048247 lymphocyte chemotaxis	2	0.31926	0.619	0.032	Biological	GO:00482	lymphocyte chemotaxis	0.31926	0.50226	0	0	2	63
GO:0051653 spindle localization	2	0.31926	0.619	0.032	Biological	GO:00516	spindle localization	0.31926	0.50226	0	0	2	63
GO:0140253 cell-cell fusion	2	0.31926	0.619	0.032	Biological	GO:01402	cell-cell fusion	0.31926	0.50226	0	0	2	63
GO:0030301 cholesterol transport	3	0.31976	0.929	0.028	Biological	GO:00303	cholesterol transport	0.31976	0.50226	0	0	3	109
GO:0033138 positive regulation of peptidyl-serine phosphorylation	3	0.31976	0.929	0.028	Biological	GO:00331	positive regulation of peptidyl-serine phosphorylation	0.31976	0.50226	0	0	3	109
GO:0090277 positive regulation of peptide hormone secretion	3	0.31976	0.929	0.028	Biological	GO:00902	positive regulation of peptide hormone secretion	0.31976	0.50226	0	0	3	109
GO:1903506 regulation of nucleic acid-templated transcription	67	0.32028	20.743	0.019	Biological	GO:19035	regulation of nucleic acid-templated transcription	0.32028	0.50226	0	0	67	3471
GO:0001502 cartilage condensation	1	0.32063	0.31	0.048	Biological	GO:00015	cartilage condensation	0.32063	0.50226	0	0	1	21
GO:0006700 C21-steroid hormone biosynthetic process	1	0.32063	0.31	0.048	Biological	GO:00067	C21-steroid hormone biosynthetic process	0.32063	0.50226	0	0	1	21
GO:0006896 Golgi to vacuole transport	1	0.32063	0.31	0.048	Biological	GO:00068	Golgi to vacuole transport	0.32063	0.50226	0	0	1	21
GO:0007194 negative regulation of adenylate cyclase activity	1	0.32063	0.31	0.048	Biological	GO:00071	negative regulation of adenylate cyclase activity	0.32063	0.50226	0	0	1	21
GO:0007625 grooming behavior	1	0.32063	0.31	0.048	Biological	GO:00076	grooming behavior	0.32063	0.50226	0	0	1	21
GO:0008340 determination of adult lifespan	1	0.32063	0.31	0.048	Biological	GO:00083	determination of adult lifespan	0.32063	0.50226	0	0	1	21
GO:0009067 aspartate family amino acid biosynthetic process	1	0.32063	0.31	0.048	Biological	GO:00090	aspartate family amino acid biosynthetic process	0.32063	0.50226	0	0	1	21
GO:0009713 catechol-containing compound biosynthetic process	1	0.32063	0.31	0.048	Biological	GO:00097	catechol-containing compound biosynthetic process	0.32063	0.50226	0	0	1	21
GO:0010881 regulation of cardiac muscle contraction by regulation of th	1	0.32063	0.31	0.048	Biological	GO:00108	regulation of cardiac muscle contraction by regulation of the release	0.32063	0.50226	0	0	1	21
GO:0021756 striatum development	1	0.32063	0.31	0.048	Biological	GO:00217	striatum development	0.32063	0.50226	0	0	1	21
GO:0022410 circadian sleep/wake cycle process	1	0.32063	0.31	0.048	Biological	GO:00224	circadian sleep/wake cycle process	0.32063	0.50226	0	0	1	21
GO:0030277 maintenance of gastrointestinal epithelium	1	0.32063	0.31	0.048	Biological	GO:00302	maintenance of gastrointestinal epithelium	0.32063	0.50226	0	0	1	21
GO:0030449 regulation of complement activation	1	0.32063	0.31	0.048	Biological	GO:00304	regulation of complement activation	0.32063	0.50226	0	0	1	21
GO:0030728 ovulation	1	0.32063	0.31	0.048	Biological	GO:00307	ovulation	0.32063	0.50226	0	0	1	21
GO:0030903 notochord development	1	0.32063	0.31	0.048	Biological	GO:00309	notochord development	0.32063	0.50226	0	0	1	21
GO:0032332 positive regulation of chondrocyte differentiation	1	0.32063	0.31	0.048	Biological	GO:00323	positive regulation of chondrocyte differentiation	0.32063	0.50226	0	0	1	21
GO:0032928 regulation of superoxide anion generation	1	0.32063	0.31	0.048	Biological	GO:00329	regulation of superoxide anion generation	0.32063	0.50226	0	0	1	21
GO:0033762 response to glucagon	1	0.32063	0.31	0.048	Biological	GO:00337	response to glucagon	0.32063	0.50226	0	0	1	21
GO:0035313 wound healing, spreading of epidermal cells	1	0.32063	0.31	0.048	Biological	GO:00353	wound healing, spreading of epidermal cells	0.32063	0.50226	0	0	1	21
GO:0035809 regulation of urine volume	1	0.32063	0.31	0.048	Biological	GO:00358	regulation of urine volume	0.32063	0.50226	0	0	1	21
GO:0042423 catecholamine biosynthetic process	1	0.32063	0.31	0.048	Biological	GO:00424	catecholamine biosynthetic process	0.32063	0.50226	0	0	1	21
GO:0043373 CD4-positive, alpha-beta T cell lineage commitment	1	0.32063	0.31	0.048	Biological	GO:00433	CD4-positive, alpha-beta T cell lineage commitment	0.32063	0.50226	0	0	1	21
GO:0043651 linoleic acid metabolic process	1	0.32063	0.31	0.048	Biological	GO:00436	linoleic acid metabolic process	0.32063	0.50226	0	0	1	21
GO:0044794 positive regulation by host of viral process	1	0.32063	0.31	0.048	Biological	GO:00447	positive regulation by host of viral process	0.32063	0.50226	0	0	1	21
GO:0045624 positive regulation of T-helper cell differentiation	1	0.32063	0.31	0.048	Biological	GO:00456	positive regulation of T-helper cell differentiation	0.32063	0.50226	0	0	1	21
GO:0048670 regulation of collateral sprouting	1	0.32063	0.31	0.048	Biological	GO:00486	regulation of collateral sprouting	0.32063	0.50226	0	0	1	21
GO:0051315 attachment of mitotic spindle microtubules to kinetochore	1	0.32063	0.31	0.048	Biological	GO:00513	attachment of mitotic spindle microtubules to kinetochore	0.32063	0.50226	0	0	1	21
GO:0051767 nitric-oxide synthase biosynthetic process	1	0.32063	0.31	0.048	Biological	GO:00517	nitric-oxide synthase biosynthetic process	0.32063	0.50226	0	0	1	21
GO:0051769 regulation of nitric-oxide synthase biosynthetic process	1	0.32063	0.31	0.048	Biological	GO:00517	regulation of nitric-oxide synthase biosynthetic process	0.32063	0.50226	0	0	1	21
GO:0090050 positive regulation of cell migration involved in sprouting ar	1	0.32063	0.31	0.048	Biological	GO:00900	positive regulation of cell migration involved in sprouting angiogenesis	0.32063	0.50226	0	0	1	21
GO:0097049 motor neuron apoptotic process	1	0.32063	0.31	0.048	Biological	GO:00970	motor neuron apoptotic process	0.32063	0.50226	0	0	1	21
GO:0097503 sialylation	1	0.32063	0.31	0.048	Biological	GO:00975	sialylation	0.32063	0.50226	0	0	1	21
GO:1900120 regulation of receptor binding	1	0.32063	0.31	0.048	Biological	GO:19001	regulation of receptor binding	0.32063	0.50226	0	0	1	21
GO:1901739 regulation of myoblast fusion	1	0.32063	0.31	0.048	Biological	GO:19017	regulation of myoblast fusion	0.32063	0.50226	0	0	1	21
GO:1902992 negative regulation of amyloid precursor protein catabolic p	1	0.32063	0.31	0.048	Biological	GO:19029	negative regulation of amyloid precursor protein catabolic process	0.32063	0.50226	0	0	1	21
GO:1903429 regulation of cell maturation	1	0.32063	0.31	0.048	Biological	GO:19034	regulation of cell maturation	0.32063	0.50226	0	0	1	21
GO:2000047 regulation of cell-cell adhesion mediated by cadherin	1	0.32063	0.31	0.048	Biological	GO:20000	regulation of cell-cell adhesion mediated by cadherin	0.32063	0.50226	0	0	1	21
GO:2001044 regulation of integrin-mediated signaling pathway	1	0.32063	0.31	0.048	Biological	GO:20010	regulation of integrin-mediated signaling pathway	0.32063	0.50226	0	0	1	21
GO:0006633 fatty acid biosynthetic process	4	0.32146	1.238	0.025	Biological	GO:00066	fatty acid biosynthetic process	0.32146	0.50325	0	0	4	157
GO:0030010 establishment of cell polarity	4	0.32146	1.238	0.025	Biological	GO:00300	establishment of cell polarity	0.32146	0.50325	0	0	4	157
GO:0006469 negative regulation of protein kinase activity	5	0.32275	1.548	0.024	Biological	GO:00064	negative regulation of protein kinase activity	0.32275	0.50505	0	0	5	206
GO:1901137 carbohydrate derivative biosynthetic process	14	0.32281	4.334	0.021	Biological	GO:19011	carbohydrate derivative biosynthetic process	0.32281	0.50505	0	0	14	662
GO:0043170 macromolecule metabolic process	175	0.32324	54.18	0.019	Biological	GO:00431	macromolecule metabolic process	0.32324	0.50556	0	0	175	9348
GO:0051649 establishment of localization in cell	45	0.3235	13.932	0.02	Biological	GO:00516	establishment of localization in cell	0.3235	0.50581	0	0	45	2296
GO:0022832 voltage-gated channel activity	5	0.32416	1.462	0.025	Molecular	GO:00228	voltage-gated channel activity	0.32416	0.50809	0	0	5	203
GO:0055085 transmembrane transport	32	0.3244	9.907	0.02	Biological	GO:00550	transmembrane transport	0.3244	0.50706	0	0	32	1606
GO:0001676 long-chain fatty acid metabolic process	3	0.32475	0.929	0.027	Biological	GO:00016	long-chain fatty acid metabolic process	0.32475	0.50745	0	0	3	110
GO:0003841 1-acylglycerol-3-phosphate O-acyltransferase activity	1	0.32503	0.292	0.048	Molecular	GO:00038	1-acylglycerol-3-phosphate O-acyltransferase activity	0.32503	0.50809	0	0	1	21
GO:0004407 histone deacetylase activity	1	0.32503	0.292	0.048	Molecular	GO:00044	histone deacetylase activity	0.32503	0.50809	0	0	1	21
GO:0008373 sialyltransferase activity	1	0.32503	0.292	0.048	Molecular	GO:00083	sialyltransferase activity	0.32503	0.50809	0	0	1	21
GO:0010314 phosphatidylinositol-5-phosphate binding	1	0.32503	0.292	0.048	Molecular	GO:00103	phosphatidylinositol-5-phosphate binding	0.32503	0.50809	0	0	1	21
GO:0017075 syntaxin-1 binding	1	0.32503	0.292	0.048	Molecular	GO:00170	syntaxin-1 binding	0.32503	0.50809	0	0	1	21
GO:0031418 L-ascorbic acid binding	1	0.32503	0.292	0.048	Molecular	GO:00314	L-ascorbic acid binding	0.32503	0.50809	0	0	1	21
GO:0042288 MHC class I protein binding	1	0.32503	0.292	0.048	Molecular	GO:00422	MHC class I protein binding	0.32503	0.50809	0	0	1	21
GO:0043027 cysteine-type endopeptidase inhibitor activity involved in ap	1	0.32503	0.292	0.048	Molecular	GO:00430	cysteine-type endopeptidase inhibitor activity involved in apoptotic pr	0.32503	0.50809	0	0	1	21
GO:0000381 regulation of alternative mRNA splicing, via spliceosome	2	0.32596	0.619	0.031	Biological	GO:00003	regulation of alternative mRNA splicing, via spliceosome	0.32596	0.50823	0	0	2	64
GO:0032371 regulation of sterol transport	2	0.32596	0.619	0.031	Biological	GO:00323	regulation of sterol transport	0.32596	0.50823	0	0	2	64
GO:0032374 regulation of cholesterol transport	2	0.32596	0.619	0.031	Biological	GO:00323	regulation of cholesterol transport	0.32596	0.50823	0	0	2	64
GO:0032757 positive regulation of interleukin-8 production	2	0.32596	0.619	0.031	Biological	GO:00327	positive regulation of interleukin-8 production	0.32596	0.50823	0	0	2	64
GO:0050854 regulation of antigen receptor-mediated signaling pathway	2	0.32596	0.619	0.031	Biological	GO:00508	regulation of antigen receptor-mediated signaling pathway	0.32596	0.50823	0	0	2	64
GO:0051149 positive regulation of muscle cell differentiation	2	0.32596	0.619	0.031	Biological	GO:00511	positive regulation of muscle cell differentiation	0.32596	0.50823	0	0	2	64
GO:0070050 neuron cellular homeostasis	2	0.32596	0.619	0.031	Biological	GO:00700	neuron cellular homeostasis	0.32596	0.50823	0	0	2	64
GO:0004714 transmembrane receptor protein tyrosine kinase activity	2	0.32632	0.585	0.032	Molecular	GO:00047	transmembrane receptor protein tyrosine kinase activity	0.32632	0.50826	0	0	2	63
GO:1990841 promoter-specific chromatin binding	2	0.32632	0.585	0.032	Molecular	GO:19908	promoter-specific chromatin binding	0.32632	0.50826	0	0	2	63
GO:0002285 lymphocyte activation involved in immune response	5	0.32634	1.548	0.024	Biological	GO:00022	lymphocyte activation involved in immune response	0.32634	0.50865	0	0	5	207
GO:0005516 calmodulin binding	5	0.32781	1.462	0.025	Molecular	GO:00055	calmodulin binding	0.32781	0.50966	0	0	5	204
GO:2001141 regulation of RNA biosynthetic process	67	0.32868	20.743	0.019	Biological	GO:20011	regulation of RNA biosynthetic process	0.32868	0.51215	0	0	67	3480
GO:0007584 response to nutrient	4	0.32972	1.238	0.025	Biological	GO:00075	response to nutrient	0.32972	0.51317	0	0	4	159
GO:0050770 regulation of axonogenesis	4	0.32972	1.238	0.025	Biological	GO:00507	regulation of axonogenesis	0.32972	0.51317	0	0	4	159
GO:0009408 response to heat	3	0.32975	0.929	0.027	Biological	GO:00094	response to heat	0.32975	0.51317	0	0	3	111
GO:0018107 peptidyl-threonine phosphorylation	3	0.32975	0.929	0.027	Biological	GO:00181	peptidyl-threonine phosphorylation	0.32975	0.51317	0	0	3	111
GO:0019722 calcium-mediated signaling	5	0.32993	1.548	0.024	Biological	GO:00197	calcium-mediated signaling	0.32993	0.51329	0	0	5	208
GO:0012510 trans-Golgi network transport vesicle membrane	1	0.32995	0.297	0.045	Cellular C	GO:00125	trans-Golgi network transport vesicle membrane	0.32995	0.56039	0	0	1	22
GO:0016580 Sin3 complex	1	0.32995	0.297	0.045	Cellular C	GO:00165	Sin3 complex	0.32995	0.56039	0	0	1	22
GO:0070822 Sin3-type complex	1	0.32995	0.297	0.045	Cellular C	GO:00708	Sin3-type complex	0.32995	0.56039	0	0	1	22
GO:0042531 positive regulation of tyrosine phosphorylation of STAT prc	2	0.33265	0.619	0.031	Biological	GO:00425	positive regulation of tyrosine phosphorylation of STAT protein	0.33265	0.51506	0	0	2	65
GO:0044091 membrane biogenesis	2	0.33265	0.619	0.031	Biological	GO:00440	membrane biogenesis	0.33265	0.51506	0	0	2	65
GO:0002082 regulation of oxidative phosphorylation	1	0.33303	0.31	0.045	Biological	GO:00020	regulation of oxidative phosphorylation	0.33303	0.51506	0	0	1	22
GO:0002115 store-operated calcium entry	1	0.33303	0.31	0.045	Biological	GO:00021	store-operated calcium entry	0.33303	0.51506	0	0	1	22
GO:0002922 positive regulation of humoral immune response	1	0.33303	0.31	0.045	Biological	GO:00029	positive regulation of humoral immune response	0.33303	0.51506	0	0	1	22
GO:0006471 protein ADP-ribosylation	1	0.33303	0.31	0.045	Biological	GO:00064	protein ADP-ribosylation	0.33303	0.51506	0	0	1	22
GO:0007620 copulation	1	0.33303	0.31	0.045	Biological	GO:00076	copulation	0.33303	0.51506	0	0	1	22
GO:0032069 regulation of nuclease activity	1	0.33303	0.31	0.045	Biological	GO:00320	regulation of nuclease activity	0.33303	0.51506	0	0	1	22
GO:0033630 positive regulation of cell adhesion mediated by integrin	1	0.33303	0.31	0.045	Biological	GO:00336	positive regulation of cell adhesion mediated by integrin	0.33303	0.51506	0	0	1	22
GO:0035268 protein mannosylation	1	0.33303	0.31	0.045	Biological	GO:00352	protein mannosylation	0.33303	0.51506	0	0	1	22
GO:0035455 response to interferon-alpha	1	0.33303	0.31	0.045	Biological	GO:00354	response to interferon-alpha	0.33303	0.51506	0	0	1	22
GO:0043371 negative regulation of CD4-positive, alpha-beta T cell differ	1	0.33303	0.31	0.045	Biological	GO:00433	negative regulation of CD4-positive, alpha-beta T cell differentiation	0.33303	0.51506	0	0	1	22
GO:0046128 purine ribonucleoside metabolic process	1	0.33303	0.31	0.045	Biological	GO:00461	purine ribonucleoside metabolic process	0.33303	0.51506	0	0	1	22
GO:0046597 negative regulation of viral entry into host cell	1	0.33303	0.31	0.045	Biological	GO:00465	negative regulation of viral entry into host cell	0.33303	0.51506	0	0	1	22
GO:0048143 astrocyte activation	1	0.33303	0.31	0.045	Biological	GO:00481	astrocyte activation	0.33303	0.51506	0	0	1	22
GO:0060008 Sertoli cell differentiation	1	0.33303	0.31	0.045	Biological	GO:00600	Sertoli cell differentiation	0.33303	0.51506	0	0	1	22
GO:0098962 regulation of postsynaptic neurotransmitter receptor activit	1	0.33303	0.31	0.045	Biological	GO:00989	regulation of postsynaptic neurotransmitter receptor activity	0.33303	0.51506	0	0	1	22
GO:1902894 negative regulation of pri-miRNA transcription by RNA poly	1	0.33303	0.31	0.045	Biological	GO:19028	negative regulation of pri-miRNA transcription by RNA polymerase II	0.33303	0.51506	0	0	1	22
GO:1903861 positive regulation of dendrite extension	1	0.33303	0.31	0.045	Biological	GO:19038	positive regulation of dendrite extension	0.33303	0.51506	0	0	1	22
GO:0009566 fertilization	5	0.33352	1.548	0.024	Biological	GO:00095	fertilization	0.33352	0.51551	0	0	5	209

GO:0071222 cellular response to lipopolysaccharide	5	0.33352	1.548	0.024	Biological	GO:00712	cellular response to lipopolysaccharide	0.33352	0.51551	0	0	5	209
GO:1904064 positive regulation of cation transmembrane transport	4	0.33385	1.238	0.025	Biological	GO:19040	positive regulation of cation transmembrane transport	0.33385	0.51577	0	0	4	160
GO:0051056 regulation of small GTPase mediated signal transduction	7	0.3339	2.167	0.023	Biological	GO:00510	regulation of small GTPase mediated signal transduction	0.3339	0.51577	0	0	7	309
GO:0005771 multivesicular body	2	0.33436	0.593	0.03	Cellular C	GO:00057	multivesicular body	0.33436	0.56431	0	0	2	66
GO:0031093 platelet alpha granule lumen	2	0.33436	0.593	0.03	Cellular C	GO:00310	platelet alpha granule lumen	0.33436	0.56431	0	0	2	66
GO:0032612 interleukin-1 production	3	0.33474	0.929	0.027	Biological	GO:00326	interleukin-1 production	0.33474	0.5166	0	0	3	112
GO:0032652 regulation of interleukin-1 production	3	0.33474	0.929	0.027	Biological	GO:00326	regulation of interleukin-1 production	0.33474	0.5166	0	0	3	112
GO:0120034 positive regulation of plasma membrane bounded cell proje	3	0.33474	0.929	0.027	Biological	GO:01200	positive regulation of plasma membrane bounded cell projection asse	0.33474	0.5166	0	0	3	112
GO:0072330 monocarboxylic acid biosynthetic process	5	0.33712	1.548	0.024	Biological	GO:00723	monocarboxylic acid biosynthetic process	0.33712	0.52011	0	0	5	210
GO:0001614 purinergic nucleotide receptor activity	1	0.33756	0.292	0.045	Molecular	GO:00016	purinergic nucleotide receptor activity	0.33756	0.52132	0	0	1	22
GO:0016502 nucleotide receptor activity	1	0.33756	0.292	0.045	Molecular	GO:00165	nucleotide receptor activity	0.33756	0.52132	0	0	1	22
GO:0031683 G-protein beta/gamma-subunit complex binding	1	0.33756	0.292	0.045	Molecular	GO:00316	G-protein beta/gamma-subunit complex binding	0.33756	0.52132	0	0	1	22
GO:0030674 protein-macromolecule adaptor activity	8	0.33772	2.339	0.023	Molecular	GO:00306	protein-macromolecule adaptor activity	0.33772	0.52132	0	0	8	355
GO:0043679 axon terminus	3	0.33833	0.89	0.026	Cellular C	GO:00436	axon terminus	0.33833	0.56794	0	0	3	114
GO:0008333 endosome to lysosome transport	2	0.33932	0.619	0.03	Biological	GO:00083	endosome to lysosome transport	0.33932	0.52269	0	0	2	66
GO:0014015 positive regulation of gliogenesis	2	0.33932	0.619	0.03	Biological	GO:00140	positive regulation of gliogenesis	0.33932	0.52269	0	0	2	66
GO:0015800 acidic amino acid transport	2	0.33932	0.619	0.03	Biological	GO:00158	acidic amino acid transport	0.33932	0.52269	0	0	2	66
GO:0021885 forebrain cell migration	2	0.33932	0.619	0.03	Biological	GO:00218	forebrain cell migration	0.33932	0.52269	0	0	2	66
GO:1901224 positive regulation of NIK/NF-kappaB signaling	2	0.33932	0.619	0.03	Biological	GO:19012	positive regulation of NIK/NF-kappaB signaling	0.33932	0.52269	0	0	2	66
GO:0009063 cellular amino acid catabolic process	3	0.33974	0.929	0.027	Biological	GO:00090	cellular amino acid catabolic process	0.33974	0.52285	0	0	3	113
GO:0010522 regulation of calcium ion transport into cytosol	3	0.33974	0.929	0.027	Biological	GO:00105	regulation of calcium ion transport into cytosol	0.33974	0.52285	0	0	3	113
GO:0046634 regulation of alpha-beta T cell activation	3	0.33974	0.929	0.027	Biological	GO:00466	regulation of alpha-beta T cell activation	0.33974	0.52285	0	0	3	113
GO:0051701 biological process involved in interaction with host	5	0.34073	1.548	0.024	Biological	GO:00517	biological process involved in interaction with host	0.34073	0.52421	0	0	5	211
GO:0060076 excitatory synapse	2	0.34095	0.593	0.03	Cellular C	GO:00600	excitatory synapse	0.34095	0.56794	0	0	2	67
GO:0005868 cytoplasmic dynein complex	1	0.34204	0.297	0.043	Cellular C	GO:00058	cytoplasmic dynein complex	0.34204	0.56794	0	0	1	23
GO:0071682 endocytic vesicle lumen	1	0.34204	0.297	0.043	Cellular C	GO:00716	endocytic vesicle lumen	0.34204	0.56794	0	0	1	23
GO:0090665 glycoprotein complex	1	0.34204	0.297	0.043	Cellular C	GO:00906	glycoprotein complex	0.34204	0.56794	0	0	1	23
GO:0005741 mitochondrial outer membrane	5	0.34288	1.484	0.023	Cellular C	GO:00057	mitochondrial outer membrane	0.34288	0.56794	0	0	5	214
GO:0051020 GTPase binding	7	0.34314	2.047	0.023	Molecular	GO:00510	GTPase binding	0.34314	0.52876	0	0	7	307
GO:0031668 cellular response to extracellular stimulus	6	0.34416	1.858	0.023	Biological	GO:00316	cellular response to extracellular stimulus	0.34416	0.52609	0	0	6	262
GO:0031396 regulation of protein ubiquitination	5	0.34433	1.548	0.024	Biological	GO:00313	regulation of protein ubiquitination	0.34433	0.52609	0	0	5	212
GO:0002363 alpha-beta T cell lineage commitment	1	0.3452	0.31	0.043	Biological	GO:00023	alpha-beta T cell lineage commitment	0.3452	0.52609	0	0	1	23
GO:0003085 negative regulation of systemic arterial blood pressure	1	0.3452	0.31	0.043	Biological	GO:00030	negative regulation of systemic arterial blood pressure	0.3452	0.52609	0	0	1	23
GO:0003283 atrial septum development	1	0.3452	0.31	0.043	Biological	GO:00032	atrial septum development	0.3452	0.52609	0	0	1	23
GO:0007039 protein catabolic process in the vacuole	1	0.3452	0.31	0.043	Biological	GO:00070	protein catabolic process in the vacuole	0.3452	0.52609	0	0	1	23
GO:0016338 calcium-independent cell-cell adhesion via plasma membra	1	0.3452	0.31	0.043	Biological	GO:00163	calcium-independent cell-cell adhesion via plasma membrane cell-ad	0.3452	0.52609	0	0	1	23
GO:0023019 signal transduction involved in regulation of gene expressio	1	0.3452	0.31	0.043	Biological	GO:00230	signal transduction involved in regulation of gene expression	0.3452	0.52609	0	0	1	23
GO:0031280 negative regulation of cyclase activity	1	0.3452	0.31	0.043	Biological	GO:00312	negative regulation of cyclase activity	0.3452	0.52609	0	0	1	23
GO:0032288 myelin assembly	1	0.3452	0.31	0.043	Biological	GO:00322	myelin assembly	0.3452	0.52609	0	0	1	23
GO:0032891 negative regulation of organic acid transport	1	0.3452	0.31	0.043	Biological	GO:00328	negative regulation of organic acid transport	0.3452	0.52609	0	0	1	23
GO:0036158 outer dynein arm assembly	1	0.3452	0.31	0.043	Biological	GO:00361	outer dynein arm assembly	0.3452	0.52609	0	0	1	23
GO:0042745 circadian sleep/wake cycle	1	0.3452	0.31	0.043	Biological	GO:00427	circadian sleep/wake cycle	0.3452	0.52609	0	0	1	23
GO:0045822 negative regulation of heart contraction	1	0.3452	0.31	0.043	Biological	GO:00458	negative regulation of heart contraction	0.3452	0.52609	0	0	1	23
GO:0046599 regulation of centriole replication	1	0.3452	0.31	0.043	Biological	GO:00465	regulation of centriole replication	0.3452	0.52609	0	0	1	23
GO:0046794 transport of virus	1	0.3452	0.31	0.043	Biological	GO:00467	transport of virus	0.3452	0.52609	0	0	1	23
GO:0050961 detection of temperature stimulus involved in sensory perc	1	0.3452	0.31	0.043	Biological	GO:00509	detection of temperature stimulus involved in sensory perception	0.3452	0.52609	0	0	1	23
GO:0051904 pigment granule transport	1	0.3452	0.31	0.043	Biological	GO:00519	pigment granule transport	0.3452	0.52609	0	0	1	23
GO:0060045 positive regulation of cardiac muscle cell proliferation	1	0.3452	0.31	0.043	Biological	GO:00600	positive regulation of cardiac muscle cell proliferation	0.3452	0.52609	0	0	1	23
GO:0072677 eosinophil migration	1	0.3452	0.31	0.043	Biological	GO:00726	eosinophil migration	0.3452	0.52609	0	0	1	23
GO:0097164 ammonium ion metabolic process	1	0.3452	0.31	0.043	Biological	GO:00971	ammonium ion metabolic process	0.3452	0.52609	0	0	1	23
GO:0098856 intestinal lipid absorption	1	0.3452	0.31	0.043	Biological	GO:00988	intestinal lipid absorption	0.3452	0.52609	0	0	1	23
GO:1900746 regulation of vascular endothelial growth factor signaling pa	1	0.3452	0.31	0.043	Biological	GO:19007	regulation of vascular endothelial growth factor signaling pathway	0.3452	0.52609	0	0	1	23
GO:1902473 regulation of protein localization to synapse	1	0.3452	0.31	0.043	Biological	GO:19024	regulation of protein localization to synapse	0.3452	0.52609	0	0	1	23
GO:1904996 positive regulation of leukocyte adhesion to vascular endot	1	0.3452	0.31	0.043	Biological	GO:19049	positive regulation of leukocyte adhesion to vascular endothelial cell	0.3452	0.52609	0	0	1	23
GO:0006801 superoxide metabolic process	2	0.34597	0.619	0.03	Biological	GO:00068	superoxide metabolic process	0.34597	0.52609	0	0	2	67
GO:0006949 syncytium formation	2	0.34597	0.619	0.03	Biological	GO:00069	syncytium formation	0.34597	0.52609	0	0	2	67
GO:0014910 regulation of smooth muscle cell migration	2	0.34597	0.619	0.03	Biological	GO:00149	regulation of smooth muscle cell migration	0.34597	0.52609	0	0	2	67
GO:0016925 protein sumoylation	2	0.34597	0.619	0.03	Biological	GO:00169	protein sumoylation	0.34597	0.52609	0	0	2	67
GO:0040014 regulation of multicellular organism growth	2	0.34597	0.619	0.03	Biological	GO:00400	regulation of multicellular organism growth	0.34597	0.52609	0	0	2	67
GO:0045453 bone resorption	2	0.34597	0.619	0.03	Biological	GO:00454	bone resorption	0.34597	0.52609	0	0	2	67
GO:0045600 positive regulation of fat cell differentiation	2	0.34597	0.619	0.03	Biological	GO:00456	positive regulation of fat cell differentiation	0.34597	0.52609	0	0	2	67
GO:0050771 negative regulation of axonogenesis	2	0.34597	0.619	0.03	Biological	GO:00507	negative regulation of axonogenesis	0.34597	0.52609	0	0	2	67
GO:0060389 pathway-restricted SMAD protein phosphorylation	2	0.34597	0.619	0.03	Biological	GO:00603	pathway-restricted SMAD protein phosphorylation	0.34597	0.52609	0	0	2	67
GO:0070830 bicellular tight junction assembly	2	0.34597	0.619	0.03	Biological	GO:00708	bicellular tight junction assembly	0.34597	0.52609	0	0	2	67
GO:2000379 positive regulation of reactive oxygen species metabolic pr	2	0.34597	0.619	0.03	Biological	GO:20003	positive regulation of reactive oxygen species metabolic process	0.34597	0.52609	0	0	2	67
GO:1903037 regulation of leukocyte cell-cell adhesion	8	0.34609	2.477	0.022	Biological	GO:19030	regulation of leukocyte cell-cell adhesion	0.34609	0.52609	0	0	8	364
GO:0032640 tumor necrosis factor production	4	0.34627	1.238	0.025	Biological	GO:00326	tumor necrosis factor production	0.34627	0.52609	0	0	4	163
GO:0032680 regulation of tumor necrosis factor production	4	0.34627	1.238	0.025	Biological	GO:00326	regulation of tumor necrosis factor production	0.34627	0.52609	0	0	4	163
GO:0060759 regulation of response to cytokine stimulus	4	0.34627	1.238	0.025	Biological	GO:00607	regulation of response to cytokine stimulus	0.34627	0.52609	0	0	4	163
GO:0071453 cellular response to oxygen levels	4	0.34627	1.238	0.025	Biological	GO:00714	cellular response to oxygen levels	0.34627	0.52609	0	0	4	163
GO:0042054 histone methyltransferase activity	2	0.34665	0.585	0.03	Molecular	GO:00420	histone methyltransferase activity	0.34665	0.52967	0	0	2	66
GO:0098631 cell adhesion mediator activity	2	0.34665	0.585	0.03	Molecular	GO:00986	cell adhesion mediator activity	0.34665	0.52967	0	0	2	66
GO:0042594 response to starvation	5	0.34794	1.548	0.023	Biological	GO:00425	response to starvation	0.34794	0.52847	0	0	5	213
GO:0044283 small molecule biosynthetic process	12	0.34876	3.715	0.021	Biological	GO:00442	small molecule biosynthetic process	0.34876	0.52949	0	0	12	571
GO:0071900 regulation of protein serine/threonine kinase activity	8	0.34883	2.477	0.022	Biological	GO:00719	regulation of protein serine/threonine kinase activity	0.34883	0.52949	0	0	8	365
GO:0051046 regulation of secretion	13	0.34893	4.025	0.021	Biological	GO:00510	regulation of secretion	0.34893	0.52949	0	0	13	623
GO:0022890 inorganic cation transmembrane transporter activity	13	0.3495	3.801	0.021	Molecular	GO:00228	inorganic cation transmembrane transporter activity	0.3495	0.52967	0	0	13	613
GO:0051963 regulation of synapse assembly	3	0.34971	0.929	0.026	Biological	GO:00519	regulation of synapse assembly	0.34971	0.53052	0	0	3	115
GO:0016881 acid-amino acid ligase activity	1	0.34985	0.292	0.043	Molecular	GO:00168	acid-amino acid ligase activity	0.34985	0.52967	0	0	1	23
GO:0030215 semaphorin receptor binding	1	0.34985	0.292	0.043	Molecular	GO:00302	semaphorin receptor binding	0.34985	0.52967	0	0	1	23
GO:0033558 protein deacetylase activity	1	0.34985	0.292	0.043	Molecular	GO:00335	protein deacetylase activity	0.34985	0.52967	0	0	1	23
GO:0042171 lysophosphatidic acid acyltransferase activity	1	0.34985	0.292	0.043	Molecular	GO:00421	lysophosphatidic acid acyltransferase activity	0.34985	0.52967	0	0	1	23
GO:0071617 lysophospholipid acyltransferase activity	1	0.34985	0.292	0.043	Molecular	GO:00716	lysophospholipid acyltransferase activity	0.34985	0.52967	0	0	1	23
GO:0098918 structural constituent of synapse	1	0.34985	0.292	0.043	Molecular	GO:00989	structural constituent of synapse	0.34985	0.52967	0	0	1	23
GO:0120020 cholesterol transfer activity	1	0.34985	0.292	0.043	Molecular	GO:01200	cholesterol transfer activity	0.34985	0.52967	0	0	1	23
GO:0006811 ion transport	32	0.3507	9.907	0.02	Biological	GO:00068	ion transport	0.3507	0.53186	0	0	32	1626
GO:0098862 cluster of actin-based cell projections	4	0.35097	1.187	0.024	Cellular C	GO:00988	cluster of actin-based cell projections	0.35097	0.57576	0	0	4	166
GO:0031100 animal organ regeneration	2	0.35259	0.619	0.029	Biological	GO:00311	animal organ regeneration	0.35259	0.53407	0	0	2	68
GO:0035567 non-canonical Wnt signaling pathway	2	0.35259	0.619	0.029	Biological	GO:00355	non-canonical Wnt signaling pathway	0.35259	0.53407	0	0	2	68
GO:0071300 cellular response to retinoic acid	2	0.35259	0.619	0.029	Biological	GO:00713	cellular response to retinoic acid	0.35259	0.53407	0	0	2	68
GO:2000401 regulation of lymphocyte migration	2	0.35259	0.619	0.029	Biological	GO:20004	regulation of lymphocyte migration	0.35259	0.53407	0	0	2	68
GO:0005776 autophagosome	3	0.35312	0.89	0.026	Cellular C	GO:00057	autophagosome	0.35312	0.57576	0	0	3	117
GO:0000124 SAGA complex	1	0.35391	0.297	0.042	Cellular C	GO:00001	SAGA complex	0.35391	0.57576	0	0	1	24
GO:0016327 apicolateral plasma membrane	1	0.35391	0.297	0.042	Cellular C	GO:00163	apicolateral plasma membrane	0.35391	0.57576	0	0	1	24
GO:0031430 M band	1	0.35391	0.297	0.042	Cellular C	GO:00314	M band	0.35391	0.57576	0	0	1	24
GO:0030669 clathrin-coated endocytic vesicle membrane	2	0.35406	0.593	0.029	Cellular C	GO:00306	clathrin-coated endocytic vesicle membrane	0.35406	0.57576	0	0	2	69
GO:0006066 alcohol metabolic process	8	0.35432	2.477	0.022	Biological	GO:00060	alcohol metabolic process	0.35432	0.53514	0	0	8	367
GO:0007292 female gamete generation	4	0.35455	1.238	0.024	Biological	GO:00072	female gamete generation	0.35455	0.53514	0	0	4	165
GO:0070665 positive regulation of leukocyte proliferation	4	0.35455	1.238	0.024	Biological	GO:00706	positive regulation of leukocyte proliferation	0.35455	0.53514	0	0	4	165
GO:1905952 regulation of lipid localization	4	0.35455	1.238	0.024	Biological	GO:19059	regulation of lipid localization	0.35455	0.53514	0	0	4	165
GO:0006357 regulation of transcription by RNA polymerase II	50	0.35465	15.48	0.019	Biological	GO:00063	regulation of transcription by RNA polymerase II	0.35465	0.53514	0	0	50	2592
GO:1902905 positive regulation of supramolecular fiber organization	5	0.35516	1.548	0.023	Biological	GO:19029	positive regulation of supramolecular fiber organization	0.35516	0.53514	0	0	5	215
GO:0016740 transferase activity	46	0.35705	13.45	0.02	Molecular	GO:00167	transferase activity	0.35705	0.53962	0	0	46	2340
GO:0010038 response to metal ion	8	0.35707	2.477	0.022	Biological	GO:00100	response to metal ion	0.35707	0.53514	0	0	8	368
GO:0002043 blood vessel endothelial cell proliferation involved in sprout	1	0.35715	0.31	0.042	Biological	GO:00020	blood vessel endothelial cell proliferation involved in sprouting angiog	0.35715	0.53514	0	0	1	24
GO:0010640 regulation of platelet-derived growth factor receptor signali	1	0.35715	0.31	0.042	Biological	GO:00106	regulation of platelet-derived growth factor receptor signaling pathwa	0.35715	0.53514	0	0	1	24
GO:0010894 negative regulation of steroid biosynthetic process	1	0.35715	0.31	0.042	Biological	GO:00108	negative regulation of steroid biosynthetic process	0.35715	0.53514	0	0	1	24
GO:0014821 phasic smooth muscle contraction	1	0.35715	0.31	0.042	Biological	GO:00148	phasic smooth muscle contraction	0.35715	0.53514	0	0	1	24
GO:0018230 peptidyl-L-cysteine S-palmitoylation	1	0.35715	0.31	0.042	Biological	GO:00182	peptidyl-L-cysteine S-palmitoylation	0.35715	0.53514	0	0	1	24
GO:0018231 peptidyl-S-diacylglycerol-L-cysteine biosynthetic process f	1	0.35715	0.31	0.042	Biological	GO:00182	peptidyl-S-diacylglycerol-L-cysteine biosynthetic process from peptid	0.35715	0.53514	0	0	1	24
GO:0030539 male genitalia development	1	0.35715	0.31	0.042	Biological	GO:00305	male genitalia development	0.35715	0.53514	0	0	1	24
GO:0034114 regulation of heterotypic cell-cell adhesion	1	0.35715	0.31	0.042	Biological	GO:00341	regulation of heterotypic cell-cell adhesion	0.35715	0.53514	0	0	1	24
GO:0035162 embryonic hemopoiesis	1	0.35715	0.31	0.042	Biological	GO:00351	embryonic hemopoiesis	0.35715	0.53514	0	0	1	24
GO:0035739 CD4-positive, alpha-beta T cell proliferation	1	0.35715	0.31	0.042	Biological	GO:00357	CD4-positive, alpha-beta T cell proliferation	0.35715	0.53514	0	0	1	24
GO:0043369 CD4-positive or CD8-positive, alpha-beta T cell lineage co	1	0.35715	0.31	0.042	Biological	GO:00433	CD4-positive or CD8-positive, alpha-beta T cell lineage commitment	0.35715	0.53514	0	0	1	24
GO:0043501 skeletal muscle adaptation	1	0.35715	0.31	0.042	Biological	GO:00435	skeletal muscle adaptation	0.35715	0.53514	0	0	1	24
GO:0044766 multi-organism transport	1	0.35715	0.31	0.042	Biological	GO:00447	multi-organism transport	0.35715	0.53514	0	0	1	24
GO:0045932 negative regulation of muscle contraction	1	0.35715	0.31	0.042	Biological	GO:00459	negative regulation of muscle contraction	0.35715	0.53514	0	0	1	24
GO:0051905 establishment of pigment granule localization	1	0.35715	0.31	0.042	Biological	GO:00519	establishment of pigment granule localization	0.35715	0.53514	0	0	1	24
GO:0060143 positive regulation of syncytium formation by plasma mem	1	0.35715	0.31	0.042	Biological	GO:00601	positive regulation of syncytium formation by plasma membrane fusio	0.35715	0.53514	0	0	1	24
GO:0060907 positive regulation of macrophage cytokine production	1	0.35715	0.31	0.042	Biological	GO:00609	positive regulation of macrophage cytokine production	0.35715	0.53514	0	0	1	24
GO:0071359 cellular response to dsRNA	1	0.35715	0.31	0.042	Biological	GO:00713	cellular response to dsRNA	0.35715	0.53514	0	0	1	24
GO:0072574 hepatocyte proliferation	1	0.35715	0.31	0.042	Biological	GO:00725	hepatocyte proliferation	0.35715	0.53514	0	0	1	24
GO:0072575 epithelial cell proliferation involved in liver morphogenesis	1	0.35715	0.31	0.042	Biological	GO:00725	epithelial cell proliferation involved in liver morphogenesis	0.35715	0.53514	0	0	1	24
GO:0090335 regulation of brown fat cell differentiation	1	0.35715	0.31	0.042	Biological	GO:00903	regulation of brown fat cell differentiation	0.35715	0.53514	0	0	1	24
GO:0098884 postsynaptic neurotransmitter receptor internalization	1	0.35715	0.31	0.042	Biological	GO:00988	postsynaptic neurotransmitter receptor internalization	0.35715	0.53514	0	0	1	24
GO:0140239 postsynaptic endocytosis	1	0.35715	0.31	0.042	Biological	GO:01402	postsynaptic endocytosis	0.35715	0.53514	0	0	1	24
GO:1900017 positive regulation of cytokine production involved in inflam	1	0.35715	0.31	0.042	Biological	GO:19000	positive regulation of cytokine production involved in inflammatory res	0.35715	0.53514	0	0	1	24
GO:1900273 positive regulation of long-term synaptic potentiation	1	0.35715	0.31	0.042	Biological	GO:19002	positive regulation of long-term synaptic potentiation	0.35715	0.53514	0	0	1	24
GO:1902579 multi-organism localization	1	0.35715	0.31	0.042	Biological	GO:19025	multi-organism localization	0.35715	0.53514	0	0	1	24
GO:1903523 negative regulation of blood circulation	1	0.35715	0.31	0.042	Biological	GO:19035	negative regulation of blood circulation	0.35715	0.53514	0	0	1	24
GO:2000178 negative regulation of neural precursor cell proliferation	1	0.35715	0.31	0.042	Biological	GO:20001	negative regulation of neural precursor cell proliferation	0.35715	0.53514	0	0	1	24
GO:2000561 regulation of CD4-positive, alpha-beta T cell proliferation	1	0.35715	0.31	0.042	Biological	GO:20005	regulation of CD4-positive, alpha-beta T cell proliferation	0.35715	0.53514	0	0	1	24
GO:0046394 carboxylic acid biosynthetic process	7	0.35755	2.167	0.022	Biological	GO:00463	carboxylic acid biosynthetic process	0.35755	0.53558	0	0	7	317
GO:0031985 Golgi cisterna	3	0.35805	0.89	0.025	Cellular C	GO:00319	Golgi cisterna	0.35805	0.58047	0	0	3	118
GO:0051896 regulation of protein kinase B signaling	4	0.35869	1.238	0.024	Biological	GO:00518	regulation of protein kinase B signaling	0.35869	0.53707	0	0	4	166
GO:0042446 hormone biosynthetic process	2	0.35919	0.619	0.029	Biological	GO:00424	hormone biosynthetic process	0.35919	0.53707	0	0	2	69
GO:0060113 inner ear receptor cell differentiation	2	0.35919	0.619	0.029	Biological	GO:00601	inner ear receptor cell differentiation	0.35919	0.53707	0	0	2	69
GO:0070936 protein K48-linked ubiquitination	2	0.35919	0.619	0.029	Biological	GO:00709	protein K48-linked ubiquitination	0.35919	0.53707	0	0	2	69
GO:1901607 alpha-amino acid biosynthetic process	2	0.35919	0.619	0.029	Biological	GO:19016	alpha-amino acid biosynthetic process	0.35919	0.53707	0	0	2	69
GO:1904377 positive regulation of protein localization to cell periphery	2	0.35919	0.619	0.029	Biological	GO:19043	positive regulation of protein localization to cell periphery	0.35919	0.53707	0	0	2	69
GO:0022037 metencephalon development	3	0.35968	0.929	0.026	Biological	GO:00220	metencephalon development	0.35968	0.53763	0	0	3	117
GO:0000030 mannosyltransferase activity	1	0.36192	0.292	0.042	Molecular	GO:00000	mannosyltransferase activity	0.36192	0.54131	0	0	1	24
GO:0005212 structural constituent of eye lens	1	0.36192	0.292	0.042	Molecular	GO:00052	structural constituent of eye lens	0.36192	0.54131	0	0	1	24
GO:0008093 cytoskeletal anchor activity	1	0.36192	0.292	0.042	Molecular	GO:00080	cytoskeletal anchor activity	0.36192	0.54131	0	0	1	24
GO:0008327 methyl-CpG binding	1	0.36192	0.292	0.042	Molecular	GO:00083	methyl-CpG binding	0.36192	0.54131	0	0	1	24
GO:0050750 low-density lipoprotein particle receptor binding	1	0.36192	0.292	0.042	Molecular	GO:00507	low-density lipoprotein particle receptor binding	0.36192	0.54131	0	0	1	24
GO:0120015 sterol transfer activity	1	0.36192	0.292	0.042	Molecular	GO:01200	sterol transfer activity	0.36192	0.54131	0	0	1	24
GO:0120031 plasma membrane bounded cell projection assembly	12	0.36195	3.715	0.021	Biological	GO:01200	plasma membrane bounded cell projection assembly	0.36195	0.54087	0	0	12	577
GO:0034220 ion transmembrane transport	24	0.3622	7.43	0.02	Biological	GO:00342	ion transmembrane transport	0.3622	0.54107	0	0	24	1209
GO:0030307 positive regulation of cell growth	4	0.36283	1.238	0.024	Biological	GO:00303	positive regulation of cell growth	0.36283	0.5417	0	0	4	167
GO:0050792 regulation of viral process	4	0.36283	1.238	0.024	Biological	GO:00507	regulation of viral process	0.36283	0.5417	0	0	4	167

GO:0005884 actin filament	3	0.36297	0.89	0.025	Cellular C	GO:00058	actin filament	0.36297	0.58667	0	0	3	119
GO:0032504 multicellular organism reproduction	19	0.36313	5.882	0.02	Biological	GO:00325	multicellular organism reproduction	0.36313	0.54198	0	0	19	945
GO:0051348 negative regulation of transferase activity	6	0.36361	1.858	0.022	Biological	GO:00513	negative regulation of transferase activity	0.36361	0.54254	0	0	6	268
GO:0010906 regulation of glucose metabolic process	3	0.36465	0.929	0.025	Biological	GO:00109	regulation of glucose metabolic process	0.36465	0.54279	0	0	3	118
GO:0090100 positive regulation of transmembrane receptor protein seri	3	0.36465	0.929	0.025	Biological	GO:00901	positive regulation of transmembrane receptor protein serine/threonir	0.36465	0.54279	0	0	3	118
GO:0044419 biological process involved in interspecies interaction betw	33	0.36488	10.217	0.02	Biological	GO:00444	biological process involved in interspecies interaction between organi	0.36488	0.54279	0	0	33	1690
GO:0003208 cardiac ventricle morphogenesis	2	0.36577	0.619	0.029	Biological	GO:00032	cardiac ventricle morphogenesis	0.36577	0.54279	0	0	2	70
GO:0006081 cellular aldehyde metabolic process	2	0.36577	0.619	0.029	Biological	GO:00060	cellular aldehyde metabolic process	0.36577	0.54279	0	0	2	70
GO:0030193 regulation of blood coagulation	2	0.36577	0.619	0.029	Biological	GO:00301	regulation of blood coagulation	0.36577	0.54279	0	0	2	70
GO:0034394 protein localization to cell surface	2	0.36577	0.619	0.029	Biological	GO:00343	protein localization to cell surface	0.36577	0.54279	0	0	2	70
GO:0099518 vesicle cytoskeletal trafficking	2	0.36577	0.619	0.029	Biological	GO:00995	vesicle cytoskeletal trafficking	0.36577	0.54279	0	0	2	70
GO:0071219 cellular response to molecule of bacterial origin	5	0.366	1.548	0.023	Biological	GO:00712	cellular response to molecule of bacterial origin	0.366	0.54279	0	0	5	218
GO:0006475 internal protein amino acid acetylation	4	0.36697	1.238	0.024	Biological	GO:00064	internal protein amino acid acetylation	0.36697	0.54279	0	0	4	168
GO:0006937 regulation of muscle contraction	4	0.36697	1.238	0.024	Biological	GO:00069	regulation of muscle contraction	0.36697	0.54279	0	0	4	168
GO:0055067 monovalent inorganic cation homeostasis	4	0.36697	1.238	0.024	Biological	GO:00550	monovalent inorganic cation homeostasis	0.36697	0.54279	0	0	4	168
GO:0071706 tumor necrosis factor superfamily cytokine production	4	0.36697	1.238	0.024	Biological	GO:00717	tumor necrosis factor superfamily cytokine production	0.36697	0.54279	0	0	4	168
GO:1903555 regulation of tumor necrosis factor superfamily cytokine pr	4	0.36697	1.238	0.024	Biological	GO:19035	regulation of tumor necrosis factor superfamily cytokine production	0.36697	0.54279	0	0	4	168
GO:0001917 photoreceptor inner segment	2	0.36708	0.593	0.028	Cellular C	GO:00019	photoreceptor inner segment	0.36708	0.59153	0	0	2	71
GO:0044389 ubiquitin-like protein ligase binding	7	0.36725	2.047	0.022	Molecular	GO:00443	ubiquitin-like protein ligase binding	0.36725	0.54835	0	0	7	315
GO:0003081 regulation of systemic arterial blood pressure by renin-ang	1	0.36889	0.31	0.04	Biological	GO:00030	regulation of systemic arterial blood pressure by renin-angiotensin	0.36889	0.54279	0	0	1	25
GO:0003148 outflow tract septum morphogenesis	1	0.36889	0.31	0.04	Biological	GO:00031	outflow tract septum morphogenesis	0.36889	0.54279	0	0	1	25
GO:0003181 atrioventricular valve morphogenesis	1	0.36889	0.31	0.04	Biological	GO:00031	atrioventricular valve morphogenesis	0.36889	0.54279	0	0	1	25
GO:0006517 protein deglycosylation	1	0.36889	0.31	0.04	Biological	GO:00065	protein deglycosylation	0.36889	0.54279	0	0	1	25
GO:0006658 phosphatidylserine metabolic process	1	0.36889	0.31	0.04	Biological	GO:00066	phosphatidylserine metabolic process	0.36889	0.54279	0	0	1	25
GO:0006706 steroid catabolic process	1	0.36889	0.31	0.04	Biological	GO:00067	steroid catabolic process	0.36889	0.54279	0	0	1	25
GO:0010155 regulation of proton transport	1	0.36889	0.31	0.04	Biological	GO:00101	regulation of proton transport	0.36889	0.54279	0	0	1	25
GO:0014047 glutamate secretion	1	0.36889	0.31	0.04	Biological	GO:00140	glutamate secretion	0.36889	0.54279	0	0	1	25
GO:0018904 ether metabolic process	1	0.36889	0.31	0.04	Biological	GO:00189	ether metabolic process	0.36889	0.54279	0	0	1	25
GO:0021884 forebrain neuron development	1	0.36889	0.31	0.04	Biological	GO:00218	forebrain neuron development	0.36889	0.54279	0	0	1	25
GO:0032331 negative regulation of chondrocyte differentiation	1	0.36889	0.31	0.04	Biological	GO:00323	negative regulation of chondrocyte differentiation	0.36889	0.54279	0	0	1	25
GO:0035235 ionotropic glutamate receptor signaling pathway	1	0.36889	0.31	0.04	Biological	GO:00352	ionotropic glutamate receptor signaling pathway	0.36889	0.54279	0	0	1	25
GO:0035458 cellular response to interferon-beta	1	0.36889	0.31	0.04	Biological	GO:00354	cellular response to interferon-beta	0.36889	0.54279	0	0	1	25
GO:0043576 regulation of respiratory gaseous exchange	1	0.36889	0.31	0.04	Biological	GO:00435	regulation of respiratory gaseous exchange	0.36889	0.54279	0	0	1	25
GO:0046697 decidualization	1	0.36889	0.31	0.04	Biological	GO:00466	decidualization	0.36889	0.54279	0	0	1	25
GO:0046716 muscle cell cellular homeostasis	1	0.36889	0.31	0.04	Biological	GO:00467	muscle cell cellular homeostasis	0.36889	0.54279	0	0	1	25
GO:0048011 neurotrophin TRK receptor signaling pathway	1	0.36889	0.31	0.04	Biological	GO:00480	neurotrophin TRK receptor signaling pathway	0.36889	0.54279	0	0	1	25
GO:0051350 negative regulation of lyase activity	1	0.36889	0.31	0.04	Biological	GO:00513	negative regulation of lyase activity	0.36889	0.54279	0	0	1	25
GO:0051450 myoblast proliferation	1	0.36889	0.31	0.04	Biological	GO:00514	myoblast proliferation	0.36889	0.54279	0	0	1	25
GO:0060571 morphogenesis of an epithelial fold	1	0.36889	0.31	0.04	Biological	GO:00605	morphogenesis of an epithelial fold	0.36889	0.54279	0	0	1	25
GO:0060575 intestinal epithelial cell differentiation	1	0.36889	0.31	0.04	Biological	GO:00605	intestinal epithelial cell differentiation	0.36889	0.54279	0	0	1	25
GO:0070841 inclusion body assembly	1	0.36889	0.31	0.04	Biological	GO:00708	inclusion body assembly	0.36889	0.54279	0	0	1	25
GO:0070861 regulation of protein exit from endoplasmic reticulum	1	0.36889	0.31	0.04	Biological	GO:00708	regulation of protein exit from endoplasmic reticulum	0.36889	0.54279	0	0	1	25
GO:0072202 cell differentiation involved in metanephros development	1	0.36889	0.31	0.04	Biological	GO:00722	cell differentiation involved in metanephros development	0.36889	0.54279	0	0	1	25
GO:0072576 liver morphogenesis	1	0.36889	0.31	0.04	Biological	GO:00725	liver morphogenesis	0.36889	0.54279	0	0	1	25
GO:0090023 positive regulation of neutrophil chemotaxis	1	0.36889	0.31	0.04	Biological	GO:00900	positive regulation of neutrophil chemotaxis	0.36889	0.54279	0	0	1	25
GO:0098581 detection of external biotic stimulus	1	0.36889	0.31	0.04	Biological	GO:00985	detection of external biotic stimulus	0.36889	0.54279	0	0	1	25
GO:0098743 cell aggregation	1	0.36889	0.31	0.04	Biological	GO:00987	cell aggregation	0.36889	0.54279	0	0	1	25
GO:1902455 negative regulation of stem cell population maintenance	1	0.36889	0.31	0.04	Biological	GO:19024	negative regulation of stem cell population maintenance	0.36889	0.54279	0	0	1	25
GO:1902547 regulation of cellular response to vascular endothelial grow	1	0.36889	0.31	0.04	Biological	GO:19025	regulation of cellular response to vascular endothelial growth factor s	0.36889	0.54279	0	0	1	25
GO:1903792 negative regulation of anion transport	1	0.36889	0.31	0.04	Biological	GO:19037	negative regulation of anion transport	0.36889	0.54279	0	0	1	25
GO:1903859 regulation of dendrite extension	1	0.36889	0.31	0.04	Biological	GO:19038	regulation of dendrite extension	0.36889	0.54279	0	0	1	25
GO:1903902 positive regulation of viral life cycle	1	0.36889	0.31	0.04	Biological	GO:19039	positive regulation of viral life cycle	0.36889	0.54279	0	0	1	25
GO:0019058 viral life cycle	7	0.36944	2.167	0.022	Biological	GO:00190	viral life cycle	0.36944	0.54339	0	0	7	321
GO:0002062 chondrocyte differentiation	3	0.36961	0.929	0.025	Biological	GO:00020	chondrocyte differentiation	0.36961	0.54339	0	0	3	119
GO:0002440 production of molecular mediator of immune response	5	0.36962	1.548	0.023	Biological	GO:00024	production of molecular mediator of immune response	0.36962	0.54339	0	0	5	219
GO:0043903 regulation of biological process involved in symbiotic intera	2	0.37232	0.619	0.028	Biological	GO:00439	regulation of biological process involved in symbiotic interaction	0.37232	0.54704	0	0	2	71
GO:0097006 regulation of plasma lipoprotein particle levels	2	0.37232	0.619	0.028	Biological	GO:00970	regulation of plasma lipoprotein particle levels	0.37232	0.54704	0	0	2	71
GO:0000978 RNA polymerase II cis-regulatory region sequence-specific	24	0.37313	7.018	0.02	Molecular	GO:00009	RNA polymerase II cis-regulatory region sequence-specific DNA bind	0.37313	0.55141	0	0	24	1196
GO:0005112 Notch binding	1	0.37376	0.292	0.04	Molecular	GO:00051	Notch binding	0.37376	0.55141	0	0	1	25
GO:0005385 zinc ion transmembrane transporter activity	1	0.37376	0.292	0.04	Molecular	GO:00053	zinc ion transmembrane transporter activity	0.37376	0.55141	0	0	1	25
GO:0008266 poly(U) RNA binding	1	0.37376	0.292	0.04	Molecular	GO:00082	poly(U) RNA binding	0.37376	0.55141	0	0	1	25
GO:0043225 ATPase-coupled inorganic anion transmembrane transpor	1	0.37376	0.292	0.04	Molecular	GO:00432	ATPase-coupled inorganic anion transmembrane transporter activity	0.37376	0.55141	0	0	1	25
GO:0070006 metalloaminopeptidase activity	1	0.37376	0.292	0.04	Molecular	GO:00700	metalloaminopeptidase activity	0.37376	0.55141	0	0	1	25
GO:0140416 transcription regulator inhibitor activity	1	0.37376	0.292	0.04	Molecular	GO:01404	transcription regulator inhibitor activity	0.37376	0.55141	0	0	1	25
GO:0016032 viral process	9	0.37408	2.786	0.021	Biological	GO:00160	viral process	0.37408	0.54943	0	0	9	426
GO:0045184 establishment of protein localization	33	0.37417	10.217	0.019	Biological	GO:00451	establishment of protein localization	0.37417	0.54943	0	0	33	1697
GO:0030278 regulation of ossification	3	0.37457	0.929	0.025	Biological	GO:00302	regulation of ossification	0.37457	0.5497	0	0	3	120
GO:0050830 defense response to Gram-positive bacterium	3	0.37457	0.929	0.025	Biological	GO:00508	defense response to Gram-positive bacterium	0.37457	0.5497	0	0	3	120
GO:1903169 regulation of calcium ion transmembrane transport	4	0.37525	1.238	0.024	Biological	GO:19031	regulation of calcium ion transmembrane transport	0.37525	0.55052	0	0	4	170
GO:0031347 regulation of defense response	13	0.37641	4.025	0.02	Biological	GO:00313	regulation of defense response	0.37641	0.55206	0	0	13	636
GO:0032281 AMPA glutamate receptor complex	1	0.37703	0.297	0.038	Cellular C	GO:00322	AMPA glutamate receptor complex	0.37703	0.60032	0	0	1	26
GO:0044295 axonal growth cone	1	0.37703	0.297	0.038	Cellular C	GO:00442	axonal growth cone	0.37703	0.60032	0	0	1	26
GO:0046930 pore complex	1	0.37703	0.297	0.038	Cellular C	GO:00469	pore complex	0.37703	0.60032	0	0	1	26
GO:0097381 photoreceptor disc membrane	1	0.37703	0.297	0.038	Cellular C	GO:00973	photoreceptor disc membrane	0.37703	0.60032	0	0	1	26
GO:0046637 regulation of alpha-beta T cell differentiation	2	0.37885	0.619	0.028	Biological	GO:00466	regulation of alpha-beta T cell differentiation	0.37885	0.55226	0	0	2	72
GO:0086003 cardiac muscle cell contraction	2	0.37885	0.619	0.028	Biological	GO:00860	cardiac muscle cell contraction	0.37885	0.55226	0	0	2	72
GO:0120192 tight junction assembly	2	0.37885	0.619	0.028	Biological	GO:01201	tight junction assembly	0.37885	0.55226	0	0	2	72
GO:1900046 regulation of hemostasis	2	0.37885	0.619	0.028	Biological	GO:19000	regulation of hemostasis	0.37885	0.55226	0	0	2	72
GO:1902475 L-alpha-amino acid transmembrane transport	2	0.37885	0.619	0.028	Biological	GO:19024	L-alpha-amino acid transmembrane transport	0.37885	0.55226	0	0	2	72
GO:0072676 lymphocyte migration	3	0.37953	0.929	0.025	Biological	GO:00726	lymphocyte migration	0.37953	0.55226	0	0	3	121
GO:0006760 folic acid-containing compound metabolic process	1	0.38041	0.31	0.038	Biological	GO:00067	folic acid-containing compound metabolic process	0.38041	0.55226	0	0	1	26
GO:0010460 positive regulation of heart rate	1	0.38041	0.31	0.038	Biological	GO:00104	positive regulation of heart rate	0.38041	0.55226	0	0	1	26
GO:0010613 positive regulation of cardiac muscle hypertrophy	1	0.38041	0.31	0.038	Biological	GO:00106	positive regulation of cardiac muscle hypertrophy	0.38041	0.55226	0	0	1	26
GO:0010623 programmed cell death involved in cell development	1	0.38041	0.31	0.038	Biological	GO:00106	programmed cell death involved in cell development	0.38041	0.55226	0	0	1	26
GO:0010882 regulation of cardiac muscle contraction by calcium ion sig	1	0.38041	0.31	0.038	Biological	GO:00108	regulation of cardiac muscle contraction by calcium ion signaling	0.38041	0.55226	0	0	1	26
GO:0019883 antigen processing and presentation of endogenous antige	1	0.38041	0.31	0.038	Biological	GO:00198	antigen processing and presentation of endogenous antigen	0.38041	0.55226	0	0	1	26
GO:0033233 regulation of protein sumoylation	1	0.38041	0.31	0.038	Biological	GO:00332	regulation of protein sumoylation	0.38041	0.55226	0	0	1	26
GO:0034377 plasma lipoprotein particle assembly	1	0.38041	0.31	0.038	Biological	GO:00343	plasma lipoprotein particle assembly	0.38041	0.55226	0	0	1	26
GO:0034695 response to prostaglandin E	1	0.38041	0.31	0.038	Biological	GO:00346	response to prostaglandin E	0.38041	0.55226	0	0	1	26
GO:0035988 chondrocyte proliferation	1	0.38041	0.31	0.038	Biological	GO:00359	chondrocyte proliferation	0.38041	0.55226	0	0	1	26
GO:0036010 protein localization to endosome	1	0.38041	0.31	0.038	Biological	GO:00360	protein localization to endosome	0.38041	0.55226	0	0	1	26
GO:0045686 negative regulation of glial cell differentiation	1	0.38041	0.31	0.038	Biological	GO:00456	negative regulation of glial cell differentiation	0.38041	0.55226	0	0	1	26
GO:0045815 positive regulation of gene expression, epigenetic	1	0.38041	0.31	0.038	Biological	GO:00458	positive regulation of gene expression, epigenetic	0.38041	0.55226	0	0	1	26
GO:0045940 positive regulation of steroid metabolic process	1	0.38041	0.31	0.038	Biological	GO:00459	positive regulation of steroid metabolic process	0.38041	0.55226	0	0	1	26
GO:0050927 positive regulation of positive chemotaxis	1	0.38041	0.31	0.038	Biological	GO:00509	positive regulation of positive chemotaxis	0.38041	0.55226	0	0	1	26
GO:0050995 negative regulation of lipid catabolic process	1	0.38041	0.31	0.038	Biological	GO:00509	negative regulation of lipid catabolic process	0.38041	0.55226	0	0	1	26
GO:0051220 cytoplasmic sequestering of protein	1	0.38041	0.31	0.038	Biological	GO:00512	cytoplasmic sequestering of protein	0.38041	0.55226	0	0	1	26
GO:0051238 sequestering of metal ion	1	0.38041	0.31	0.038	Biological	GO:00512	sequestering of metal ion	0.38041	0.55226	0	0	1	26
GO:0051875 pigment granule localization	1	0.38041	0.31	0.038	Biological	GO:00518	pigment granule localization	0.38041	0.55226	0	0	1	26
GO:0051894 positive regulation of focal adhesion assembly	1	0.38041	0.31	0.038	Biological	GO:00518	positive regulation of focal adhesion assembly	0.38041	0.55226	0	0	1	26
GO:0060117 auditory receptor cell development	1	0.38041	0.31	0.038	Biological	GO:00601	auditory receptor cell development	0.38041	0.55226	0	0	1	26
GO:0062009 secondary palate development	1	0.38041	0.31	0.038	Biological	GO:00620	secondary palate development	0.38041	0.55226	0	0	1	26
GO:0070935 3'-UTR-mediated mRNA stabilization	1	0.38041	0.31	0.038	Biological	GO:00709	3'-UTR-mediated mRNA stabilization	0.38041	0.55226	0	0	1	26
GO:0071280 cellular response to copper ion	1	0.38041	0.31	0.038	Biological	GO:00712	cellular response to copper ion	0.38041	0.55226	0	0	1	26
GO:0099637 neurotransmitter receptor transport	1	0.38041	0.31	0.038	Biological	GO:00996	neurotransmitter receptor transport	0.38041	0.55226	0	0	1	26
GO:0140374 antiviral innate immune response	1	0.38041	0.31	0.038	Biological	GO:01403	antiviral innate immune response	0.38041	0.55226	0	0	1	26
GO:1901071 glucosamine-containing compound metabolic process	1	0.38041	0.31	0.038	Biological	GO:19010	glucosamine-containing compound metabolic process	0.38041	0.55226	0	0	1	26
GO:1902074 response to salt	1	0.38041	0.31	0.038	Biological	GO:19020	response to salt	0.38041	0.55226	0	0	1	26
GO:1904262 negative regulation of TORC1 signaling	1	0.38041	0.31	0.038	Biological	GO:19042	negative regulation of TORC1 signaling	0.38041	0.55226	0	0	1	26
GO:0034762 regulation of transmembrane transport	12	0.38188	3.715	0.02	Biological	GO:00347	regulation of transmembrane transport	0.38188	0.55423	0	0	12	586
GO:0097733 photoreceptor cell cilium	3	0.38257	0.89	0.024	Cellular C	GO:00977	photoreceptor cell cilium	0.38257	0.60732	0	0	3	123
GO:0005543 phospholipid binding	10	0.38366	2.924	0.021	Molecular	GO:00055	phospholipid binding	0.38366	0.56091	0	0	10	474
GO:0000922 spindle pole	4	0.38369	1.187	0.023	Cellular C	GO:00009	spindle pole	0.38369	0.60732	0	0	4	174
GO:0006690 icosanoid metabolic process	3	0.38447	0.929	0.025	Biological	GO:00066	icosanoid metabolic process	0.38447	0.5575	0	0	3	122
GO:0006720 isoprenoid metabolic process	3	0.38447	0.929	0.025	Biological	GO:00067	isoprenoid metabolic process	0.38447	0.5575	0	0	3	122
GO:0071675 regulation of mononuclear cell migration	3	0.38447	0.929	0.025	Biological	GO:00716	regulation of mononuclear cell migration	0.38447	0.5575	0	0	3	122
GO:0003281 ventricular septum development	2	0.38534	0.619	0.027	Biological	GO:00032	ventricular septum development	0.38534	0.55812	0	0	2	73
GO:0007405 neuroblast proliferation	2	0.38534	0.619	0.027	Biological	GO:00074	neuroblast proliferation	0.38534	0.55812	0	0	2	73
GO:0042982 amyloid precursor protein metabolic process	2	0.38534	0.619	0.027	Biological	GO:00429	amyloid precursor protein metabolic process	0.38534	0.55812	0	0	2	73
GO:0061035 regulation of cartilage development	2	0.38534	0.619	0.027	Biological	GO:00610	regulation of cartilage development	0.38534	0.55812	0	0	2	73
GO:0001671 ATPase activator activity	1	0.38538	0.292	0.038	Molecular	GO:00016	ATPase activator activity	0.38538	0.56091	0	0	1	26
GO:0003746 translation elongation factor activity	1	0.38538	0.292	0.038	Molecular	GO:00037	translation elongation factor activity	0.38538	0.56091	0	0	1	26
GO:0005104 fibroblast growth factor receptor binding	1	0.38538	0.292	0.038	Molecular	GO:00051	fibroblast growth factor receptor binding	0.38538	0.56091	0	0	1	26
GO:0019706 protein-cysteine S-palmitoyltransferase activity	1	0.38538	0.292	0.038	Molecular	GO:00197	protein-cysteine S-palmitoyltransferase activity	0.38538	0.56091	0	0	1	26
GO:0019707 protein-cysteine S-acyltransferase activity	1	0.38538	0.292	0.038	Molecular	GO:00197	protein-cysteine S-acyltransferase activity	0.38538	0.56091	0	0	1	26
GO:0030507 spectrin binding	1	0.38538	0.292	0.038	Molecular	GO:00305	spectrin binding	0.38538	0.56091	0	0	1	26
GO:0050681 androgen receptor binding	1	0.38538	0.292	0.038	Molecular	GO:00506	androgen receptor binding	0.38538	0.56091	0	0	1	26
GO:0046903 secretion	19	0.38581	5.882	0.02	Biological	GO:00469	secretion	0.38581	0.55864	0	0	19	958
GO:0030031 cell projection assembly	12	0.38633	3.715	0.02	Biological	GO:00300	cell projection assembly	0.38633	0.55922	0	0	12	588
GO:0005905 clathrin-coated pit	2	0.38641	0.593	0.027	Cellular C	GO:00059	clathrin-coated pit	0.38641	0.60803	0	0	2	74
GO:0034704 calcium channel complex	2	0.38641	0.593	0.027	Cellular C	GO:00347	calcium channel complex	0.38641	0.60803	0	0	2	74
GO:0051604 protein maturation	7	0.38732	2.167	0.021	Biological	GO:00516	protein maturation	0.38732	0.56049	0	0	7	327
GO:0046890 regulation of lipid biosynthetic process	4	0.38764	1.238	0.023	Biological	GO:00468	regulation of lipid biosynthetic process	0.38764	0.56066	0	0	4	173
GO:0030057 desmosome	1	0.38827	0.297	0.037	Cellular C	GO:00300	desmosome	0.38827	0.60916	0	0	1	27
GO:0015918 sterol transport	3	0.38941	0.929	0.024	Biological	GO:00159	sterol transport	0.38941	0.56066	0	0	3	123
GO:0032355 response to estradiol	3	0.38941	0.929	0.024	Biological	GO:00323	response to estradiol	0.38941	0.56066	0	0	3	123
GO:0044255 cellular lipid metabolic process	20	0.39069	6.192	0.02	Biological	GO:00442	cellular lipid metabolic process	0.39069	0.56066	0	0	20	1014
GO:0000737 DNA catabolic process, endonucleolytic	1	0.39172	0.31	0.037	Biological	GO:00007	DNA catabolic process, endonucleolytic	0.39172	0.56066	0	0	1	27
GO:0002438 acute inflammatory response to antigenic stimulus	1	0.39172	0.31	0.037	Biological	GO:00024	acute inflammatory response to antigenic stimulus	0.39172	0.56066	0	0	1	27
GO:0002675 positive regulation of acute inflammatory response	1	0.39172	0.31	0.037	Biological	GO:00026	positive regulation of acute inflammatory response	0.39172	0.56066	0	0	1	27
GO:0006027 glycosaminoglycan catabolic process	1	0.39172	0.31	0.037	Biological	GO:00060	glycosaminoglycan catabolic process	0.39172	0.56066	0	0	1	27
GO:0006541 glutamine metabolic process	1	0.39172	0.31	0.037	Biological	GO:00065	glutamine metabolic process	0.39172	0.56066	0	0	1	27
GO:0007274 neuromuscular synaptic transmission	1	0.39172	0.31	0.037	Biological	GO:00072	neuromuscular synaptic transmission	0.39172	0.56066	0	0	1	27
GO:0010818 T cell chemotaxis	1	0.39172	0.31	0.037	Biological	GO:00108	T cell chemotaxis	0.39172	0.56066	0	0	1	27
GO:0014742 positive regulation of muscle hypertrophy	1	0.39172	0.31	0.037	Biological	GO:00147	positive regulation of muscle hypertrophy	0.39172	0.56066	0	0	1	27
GO:0014912 negative regulation of smooth muscle cell migration	1	0.39172	0.31	0.037	Biological	GO:00149	negative regulation of smooth muscle cell migration	0.39172	0.56066	0	0	1	27
GO:0016188 synaptic vesicle maturation	1	0.39172	0.31	0.037	Biological	GO:00161	synaptic vesicle maturation	0.39172	0.56066	0	0	1	27
GO:0021544 subpallium development	1	0.39172	0.31	0.037	Biological	GO:00215	subpallium development	0.39172	0.56066	0	0	1	27
GO:0032801 receptor catabolic process	1	0.39172	0.31	0.037	Biological	GO:00328	receptor catabolic process	0.39172	0.56066	0	0	1	27
GO:0042026 protein refolding	1	0.39172	0.31	0.037	Biological	GO:00420	protein refolding	0.39172	0.56066	0	0	1	27
GO:0042059 negative regulation of epidermal growth factor receptor sig	1	0.39172	0.31	0.037	Biological	GO:00420	negative regulation of epidermal growth factor receptor signaling path	0.39172	0.56066	0	0	1	27
GO:0042278 purine nucleoside metabolic process	1	0.39172	0.31	0.037	Biological	GO:00422	purine nucleoside metabolic process	0.39172	0.56066	0	0	1	27

GO:0042402 cellular biogenic amine catabolic process	1	0.39172	0.31	0.037	Biological	GO:00424	cellular biogenic amine catabolic process	0.39172	0.56066	0	0	1	27
GO:0042403 thyroid hormone metabolic process	1	0.39172	0.31	0.037	Biological	GO:00424	thyroid hormone metabolic process	0.39172	0.56066	0	0	1	27
GO:0045606 positive regulation of epidermal cell differentiation	1	0.39172	0.31	0.037	Biological	GO:0045(	positive regulation of epidermal cell differentiation	0.39172	0.56066	0	0	1	27
GO:0045939 negative regulation of steroid metabolic process	1	0.39172	0.31	0.037	Biological	GO:00459	negative regulation of steroid metabolic process	0.39172	0.56066	0	0	1	27
GO:0046639 negative regulation of alpha-beta T cell differentiation	1	0.39172	0.31	0.037	Biological	GO:0046(	negative regulation of alpha-beta T cell differentiation	0.39172	0.56066	0	0	1	27
GO:0050926 regulation of positive chemotaxis	1	0.39172	0.31	0.037	Biological	GO:00509	regulation of positive chemotaxis	0.39172	0.56066	0	0	1	27
GO:0051953 negative regulation of amine transport	1	0.39172	0.31	0.037	Biological	GO:00519	negative regulation of amine transport	0.39172	0.56066	0	0	1	27
GO:0070498 interleukin-1-mediated signaling pathway	1	0.39172	0.31	0.037	Biological	GO:00704	interleukin-1-mediated signaling pathway	0.39172	0.56066	0	0	1	27
GO:0090200 positive regulation of release of cytochrome c from mitocho	1	0.39172	0.31	0.037	Biological	GO:00902	positive regulation of release of cytochrome c from mitochondria	0.39172	0.56066	0	0	1	27
GO:1903055 positive regulation of extracellular matrix organization	1	0.39172	0.31	0.037	Biological	GO:1903(	positive regulation of extracellular matrix organization	0.39172	0.56066	0	0	1	27
GO:1903077 negative regulation of protein localization to plasma membr	1	0.39172	0.31	0.037	Biological	GO:1903(	negative regulation of protein localization to plasma membrane	0.39172	0.56066	0	0	1	27
GO:1905523 positive regulation of macrophage migration	1	0.39172	0.31	0.037	Biological	GO:19055	positive regulation of macrophage migration	0.39172	0.56066	0	0	1	27
GO:2000737 negative regulation of stem cell differentiation	1	0.39172	0.31	0.037	Biological	GO:20007	negative regulation of stem cell differentiation	0.39172	0.56066	0	0	1	27
GO:0031214 biomineral tissue development	4	0.39177	1.238	0.023	Biological	GO:00312	biomineral tissue development	0.39177	0.56066	0	0	4	174
GO:0110148 biomineralization	4	0.39177	1.238	0.023	Biological	GO:01101	biomineralization	0.39177	0.56066	0	0	4	174
GO:0005977 glycogen metabolic process	2	0.39181	0.619	0.027	Biological	GO:00059	glycogen metabolic process	0.39181	0.56066	0	0	2	74
GO:0006487 protein N-linked glycosylation	2	0.39181	0.619	0.027	Biological	GO:00064	protein N-linked glycosylation	0.39181	0.56066	0	0	2	74
GO:0014909 smooth muscle cell migration	2	0.39181	0.619	0.027	Biological	GO:00149	smooth muscle cell migration	0.39181	0.56066	0	0	2	74
GO:0042246 tissue regeneration	2	0.39181	0.619	0.027	Biological	GO:00422	tissue regeneration	0.39181	0.56066	0	0	2	74
GO:0086001 cardiac muscle cell action potential	2	0.39181	0.619	0.027	Biological	GO:0086(	cardiac muscle cell action potential	0.39181	0.56066	0	0	2	74
GO:0010008 endosome membrane	11	0.39204	3.264	0.02	Cellular C	GO:0010(	endosome membrane	0.39204	0.61328	0	0	11	544
GO:0060079 excitatory postsynaptic potential	3	0.39433	0.929	0.024	Biological	GO:0060(	excitatory postsynaptic potential	0.39433	0.5641	0	0	3	124
GO:0001227 DNA-binding transcription repressor activity, RNA polymer	7	0.39451	2.047	0.022	Molecular	GO:00012	DNA-binding transcription repressor activity, RNA polymerase II-spe	0.39451	0.57175	0	0	7	324
GO:0009410 response to xenobiotic stimulus	9	0.39484	2.786	0.021	Biological	GO:00094	response to xenobiotic stimulus	0.39484	0.56447	0	0	9	434
GO:0034764 positive regulation of transmembrane transport	5	0.39493	1.548	0.022	Biological	GO:00347	positive regulation of transmembrane transport	0.39493	0.56447	0	0	5	226
GO:0099003 vesicle-mediated transport in synapse	5	0.39493	1.548	0.022	Biological	GO:0099(	vesicle-mediated transport in synapse	0.39493	0.56447	0	0	5	226
GO:0006575 cellular modified amino acid metabolic process	4	0.39589	1.238	0.023	Biological	GO:00065	cellular modified amino acid metabolic process	0.39589	0.56568	0	0	4	175
GO:0032496 response to lipopolysaccharide	7	0.39626	2.167	0.021	Biological	GO:00324	response to lipopolysaccharide	0.39626	0.56606	0	0	7	330
GO:0004683 calmodulin-dependent protein kinase activity	1	0.39679	0.292	0.037	Molecular	GO:0004(	calmodulin-dependent protein kinase activity	0.39679	0.57175	0	0	1	27
GO:0004709 MAP kinase kinase kinase activity	1	0.39679	0.292	0.037	Molecular	GO:00047	MAP kinase kinase kinase activity	0.39679	0.57175	0	0	1	27
GO:0008066 glutamate receptor activity	1	0.39679	0.292	0.037	Molecular	GO:0008(	glutamate receptor activity	0.39679	0.57175	0	0	1	27
GO:0035250 UDP-galactosyltransferase activity	1	0.39679	0.292	0.037	Molecular	GO:00352	UDP-galactosyltransferase activity	0.39679	0.57175	0	0	1	27
GO:0140326 ATPase-coupled intramembrane lipid transporter activity	1	0.39679	0.292	0.037	Molecular	GO:01403	ATPase-coupled intramembrane lipid transporter activity	0.39679	0.57175	0	0	1	27
GO:0006869 lipid transport	9	0.39744	2.786	0.021	Biological	GO:00068	lipid transport	0.39744	0.56727	0	0	9	435
GO:0002067 glandular epithelial cell differentiation	2	0.39824	0.619	0.027	Biological	GO:0002(	glandular epithelial cell differentiation	0.39824	0.56727	0	0	2	75
GO:0006073 cellular glucan metabolic process	2	0.39824	0.619	0.027	Biological	GO:0006(	cellular glucan metabolic process	0.39824	0.56727	0	0	2	75
GO:0034103 regulation of tissue remodeling	2	0.39824	0.619	0.027	Biological	GO:00341	regulation of tissue remodeling	0.39824	0.56727	0	0	2	75
GO:0043297 apical junction assembly	2	0.39824	0.619	0.027	Biological	GO:00432	apical junction assembly	0.39824	0.56727	0	0	2	75
GO:0044042 glucan metabolic process	2	0.39824	0.619	0.027	Biological	GO:0044(	glucan metabolic process	0.39824	0.56727	0	0	2	75
GO:0050795 regulation of behavior	2	0.39824	0.619	0.027	Biological	GO:00507	regulation of behavior	0.39824	0.56727	0	0	2	75
GO:0050818 regulation of coagulation	2	0.39824	0.619	0.027	Biological	GO:00508	regulation of coagulation	0.39824	0.56727	0	0	2	75
GO:0055017 cardiac muscle tissue growth	2	0.39824	0.619	0.027	Biological	GO:0055(	cardiac muscle tissue growth	0.39824	0.56727	0	0	2	75
GO:0071479 cellular response to ionizing radiation	2	0.39824	0.619	0.027	Biological	GO:00714	cellular response to ionizing radiation	0.39824	0.56727	0	0	2	75
GO:0003712 transcription coregulator activity	11	0.39903	3.216	0.021	Molecular	GO:00037	transcription coregulator activity	0.39903	0.57317	0	0	11	532
GO:0001891 phagocytic cup	1	0.39931	0.297	0.036	Cellular C	GO:00018	phagocytic cup	0.39931	0.61923	0	0	1	28
GO:0032809 neuronal cell body membrane	1	0.39931	0.297	0.036	Cellular C	GO:00328	neuronal cell body membrane	0.39931	0.61923	0	0	1	28
GO:0071782 endoplasmic reticulum tubular network	1	0.39931	0.297	0.036	Cellular C	GO:00717	endoplasmic reticulum tubular network	0.39931	0.61923	0	0	1	28
GO:1990782 protein tyrosine kinase binding	3	0.39951	0.877	0.024	Molecular	GO:19907	protein tyrosine kinase binding	0.39951	0.57317	0	0	3	123
GO:0017022 myosin binding	2	0.39977	0.585	0.027	Molecular	GO:0017(	myosin binding	0.39977	0.57317	0	0	2	74
GO:0034767 positive regulation of ion transmembrane transport	4	0.4	1.238	0.023	Biological	GO:00347	positive regulation of ion transmembrane transport	0.4	0.56764	0	0	4	176
GO:0048015 phosphatidylinositol-mediated signaling	4	0.4	1.238	0.023	Biological	GO:0048(	phosphatidylinositol-mediated signaling	0.4	0.56764	0	0	4	176
GO:0051252 regulation of RNA metabolic process	71	0.40127	21.981	0.019	Biological	GO:00512	regulation of RNA metabolic process	0.40127	0.56764	0	0	71	3772
GO:0016363 nuclear matrix	3	0.40204	0.89	0.024	Cellular C	GO:00163	nuclear matrix	0.40204	0.62165	0	0	3	127
GO:0044281 small molecule metabolic process	35	0.40279	10.836	0.019	Biological	GO:00442	small molecule metabolic process	0.40279	0.56764	0	0	35	1826
GO:0003171 atrioventricular valve development	1	0.40283	0.31	0.036	Biological	GO:00031	atrioventricular valve development	0.40283	0.56764	0	0	1	28
GO:0003272 endocardial cushion formation	1	0.40283	0.31	0.036	Biological	GO:00032	endocardial cushion formation	0.40283	0.56764	0	0	1	28
GO:0005979 regulation of glycogen biosynthetic process	1	0.40283	0.31	0.036	Biological	GO:00059	regulation of glycogen biosynthetic process	0.40283	0.56764	0	0	1	28
GO:0007263 nitric oxide mediated signal transduction	1	0.40283	0.31	0.036	Biological	GO:00072	nitric oxide mediated signal transduction	0.40283	0.56764	0	0	1	28
GO:0007342 fusion of sperm to egg plasma membrane involved in singl	1	0.40283	0.31	0.036	Biological	GO:00073	fusion of sperm to egg plasma membrane involved in single fertilizati	0.40283	0.56764	0	0	1	28
GO:0009310 amine catabolic process	1	0.40283	0.31	0.036	Biological	GO:00093	amine catabolic process	0.40283	0.56764	0	0	1	28
GO:0010575 positive regulation of vascular endothelial growth factor pro	1	0.40283	0.31	0.036	Biological	GO:00105	positive regulation of vascular endothelial growth factor production	0.40283	0.56764	0	0	1	28
GO:0010800 positive regulation of peptidyl-threonine phosphorylation	1	0.40283	0.31	0.036	Biological	GO:00108	positive regulation of peptidyl-threonine phosphorylation	0.40283	0.56764	0	0	1	28
GO:0010962 regulation of glucan biosynthetic process	1	0.40283	0.31	0.036	Biological	GO:00109	regulation of glucan biosynthetic process	0.40283	0.56764	0	0	1	28
GO:0018126 protein hydroxylation	1	0.40283	0.31	0.036	Biological	GO:00181	protein hydroxylation	0.40283	0.56764	0	0	1	28
GO:0021801 cerebral cortex radial glia-guided migration	1	0.40283	0.31	0.036	Biological	GO:00218	cerebral cortex radial glia-guided migration	0.40283	0.56764	0	0	1	28
GO:0022030 telencephalon glial cell migration	1	0.40283	0.31	0.036	Biological	GO:0022(	telencephalon glial cell migration	0.40283	0.56764	0	0	1	28
GO:0030194 positive regulation of blood coagulation	1	0.40283	0.31	0.036	Biological	GO:00301	positive regulation of blood coagulation	0.40283	0.56764	0	0	1	28
GO:0031664 regulation of lipopolysaccharide-mediated signaling pathwa	1	0.40283	0.31	0.036	Biological	GO:0031(	regulation of lipopolysaccharide-mediated signaling pathway	0.40283	0.56764	0	0	1	28
GO:0032469 endoplasmic reticulum calcium ion homeostasis	1	0.40283	0.31	0.036	Biological	GO:00324	endoplasmic reticulum calcium ion homeostasis	0.40283	0.56764	0	0	1	28
GO:0033598 mammary gland epithelial cell proliferation	1	0.40283	0.31	0.036	Biological	GO:00335	mammary gland epithelial cell proliferation	0.40283	0.56764	0	0	1	28
GO:0034063 stress granule assembly	1	0.40283	0.31	0.036	Biological	GO:0034(	stress granule assembly	0.40283	0.56764	0	0	1	28
GO:0036037 CD8-positive, alpha-beta T cell activation	1	0.40283	0.31	0.036	Biological	GO:0036(	CD8-positive, alpha-beta T cell activation	0.40283	0.56764	0	0	1	28
GO:0039529 RIG-I signaling pathway	1	0.40283	0.31	0.036	Biological	GO:00395	RIG-I signaling pathway	0.40283	0.56764	0	0	1	28
GO:0042537 benzene-containing compound metabolic process	1	0.40283	0.31	0.036	Biological	GO:00425	benzene-containing compound metabolic process	0.40283	0.56764	0	0	1	28
GO:0045116 protein neddylation	1	0.40283	0.31	0.036	Biological	GO:00451	protein neddylation	0.40283	0.56764	0	0	1	28
GO:0048668 collateral sprouting	1	0.40283	0.31	0.036	Biological	GO:0048(	collateral sprouting	0.40283	0.56764	0	0	1	28
GO:0048843 negative regulation of axon extension involved in axon guid	1	0.40283	0.31	0.036	Biological	GO:00488	negative regulation of axon extension involved in axon guidance	0.40283	0.56764	0	0	1	28
GO:0050996 positive regulation of lipid catabolic process	1	0.40283	0.31	0.036	Biological	GO:00509	positive regulation of lipid catabolic process	0.40283	0.56764	0	0	1	28
GO:0051984 positive regulation of chromosome segregation	1	0.40283	0.31	0.036	Biological	GO:00519	positive regulation of chromosome segregation	0.40283	0.56764	0	0	1	28
GO:0055023 positive regulation of cardiac muscle tissue growth	1	0.40283	0.31	0.036	Biological	GO:0055(	positive regulation of cardiac muscle tissue growth	0.40283	0.56764	0	0	1	28
GO:0060333 interferon-gamma-mediated signaling pathway	1	0.40283	0.31	0.036	Biological	GO:00603	interferon-gamma-mediated signaling pathway	0.40283	0.56764	0	0	1	28
GO:0062149 detection of stimulus involved in sensory perception of pai	1	0.40283	0.31	0.036	Biological	GO:00621	detection of stimulus involved in sensory perception of pain	0.40283	0.56764	0	0	1	28
GO:0070168 negative regulation of biomineral tissue development	1	0.40283	0.31	0.036	Biological	GO:00701	negative regulation of biomineral tissue development	0.40283	0.56764	0	0	1	28
GO:0071624 positive regulation of granulocyte chemotaxis	1	0.40283	0.31	0.036	Biological	GO:0071(	positive regulation of granulocyte chemotaxis	0.40283	0.56764	0	0	1	28
GO:0110150 negative regulation of biomineralization	1	0.40283	0.31	0.036	Biological	GO:01101	negative regulation of biomineralization	0.40283	0.56764	0	0	1	28
GO:1900048 positive regulation of hemostasis	1	0.40283	0.31	0.036	Biological	GO:1900(	positive regulation of hemostasis	0.40283	0.56764	0	0	1	28
GO:1901223 negative regulation of NIK/NF-kappaB signaling	1	0.40283	0.31	0.036	Biological	GO:19012	negative regulation of NIK/NF-kappaB signaling	0.40283	0.56764	0	0	1	28
GO:1903579 negative regulation of ATP metabolic process	1	0.40283	0.31	0.036	Biological	GO:19035	negative regulation of ATP metabolic process	0.40283	0.56764	0	0	1	28
GO:0003231 cardiac ventricle development	3	0.40415	0.929	0.024	Biological	GO:00032	cardiac ventricle development	0.40415	0.56902	0	0	3	126
GO:0035821 modulation of process of other organism	3	0.40415	0.929	0.024	Biological	GO:00358	modulation of process of other organism	0.40415	0.56902	0	0	3	126
GO:0038061 NIK/NF-kappaB signaling	3	0.40415	0.929	0.024	Biological	GO:0038(	NIK/NF-kappaB signaling	0.40415	0.56902	0	0	3	126
GO:0009988 cell-cell recognition	2	0.40465	0.619	0.026	Biological	GO:00099	cell-cell recognition	0.40465	0.56908	0	0	2	76
GO:0032729 positive regulation of interferon-gamma production	2	0.40465	0.619	0.026	Biological	GO:00327	positive regulation of interferon-gamma production	0.40465	0.56908	0	0	2	76
GO:0045913 positive regulation of carbohydrate metabolic process	2	0.40465	0.619	0.026	Biological	GO:00459	positive regulation of carbohydrate metabolic process	0.40465	0.56908	0	0	2	76
GO:0090307 mitotic spindle assembly	2	0.40465	0.619	0.026	Biological	GO:00903	mitotic spindle assembly	0.40465	0.56908	0	0	2	76
GO:0000987 cis-regulatory region sequence-specific DNA binding	24	0.40484	7.018	0.02	Molecular	GO:00009	cis-regulatory region sequence-specific DNA binding	0.40484	0.57949	0	0	24	1216
GO:0031594 neuromuscular junction	2	0.40548	0.593	0.026	Cellular C	GO:00315	neuromuscular junction	0.40548	0.62337	0	0	2	77
GO:0060170 ciliary membrane	2	0.40548	0.593	0.026	Cellular C	GO:00601	ciliary membrane	0.40548	0.62337	0	0	2	77
GO:0008187 poly-pyrimidine tract binding	1	0.40799	0.292	0.036	Molecular	GO:00081	poly-pyrimidine tract binding	0.40799	0.58016	0	0	1	28
GO:0016405 CoA-ligase activity	1	0.40799	0.292	0.036	Molecular	GO:00164	CoA-ligase activity	0.40799	0.58016	0	0	1	28
GO:0043236 laminin binding	1	0.40799	0.292	0.036	Molecular	GO:00432	laminin binding	0.40799	0.58016	0	0	1	28
GO:0080025 phosphatidylinositol-3,5-bisphosphate binding	1	0.40799	0.292	0.036	Molecular	GO:0080(	phosphatidylinositol-3,5-bisphosphate binding	0.40799	0.58016	0	0	1	28
GO:0006941 striated muscle contraction	4	0.40822	1.238	0.022	Biological	GO:00069	striated muscle contraction	0.40822	0.57379	0	0	4	178
GO:0051099 positive regulation of binding	4	0.40822	1.238	0.022	Biological	GO:0051(	positive regulation of binding	0.40822	0.57379	0	0	4	178
GO:0006612 protein targeting to membrane	3	0.40904	0.929	0.024	Biological	GO:0006(	protein targeting to membrane	0.40904	0.57478	0	0	3	127
GO:0001217 DNA-binding transcription repressor activity	7	0.40967	2.047	0.021	Molecular	GO:00012	DNA-binding transcription repressor activity	0.40967	0.5816	0	0	7	329
GO:0042509 regulation of tyrosine phosphorylation of STAT protein	2	0.41102	0.619	0.026	Biological	GO:00425	regulation of tyrosine phosphorylation of STAT protein	0.41102	0.57596	0	0	2	77
GO:0043154 negative regulation of cysteine-type endopeptidase activity	2	0.41102	0.619	0.026	Biological	GO:00431	negative regulation of cysteine-type endopeptidase activity involved ir	0.41102	0.57596	0	0	2	77
GO:0046928 regulation of neurotransmitter secretion	2	0.41102	0.619	0.026	Biological	GO:00469	regulation of neurotransmitter secretion	0.41102	0.57596	0	0	2	77
GO:2000514 regulation of CD4-positive, alpha-beta T cell activation	2	0.41102	0.619	0.026	Biological	GO:20005	regulation of CD4-positive, alpha-beta T cell activation	0.41102	0.57596	0	0	2	77
GO:0017053 transcription repressor complex	2	0.41178	0.593	0.026	Cellular C	GO:0017(	transcription repressor complex	0.41178	0.62943	0	0	2	78
GO:0030670 phagocytic vesicle membrane	2	0.41178	0.593	0.026	Cellular C	GO:0030(	phagocytic vesicle membrane	0.41178	0.62943	0	0	2	78
GO:0001818 negative regulation of cytokine production	6	0.41238	1.858	0.021	Biological	GO:00018	negative regulation of cytokine production	0.41238	0.57596	0	0	6	283
GO:0046777 protein autophosphorylation	5	0.41296	1.548	0.022	Biological	GO:00467	protein autophosphorylation	0.41296	0.57596	0	0	5	231
GO:0048609 multicellular organismal reproductive process	18	0.41313	5.573	0.02	Biological	GO:0048(	multicellular organismal reproductive process	0.41313	0.57596	0	0	18	920
GO:0042110 T cell activation	11	0.4133	3.406	0.02	Biological	GO:00421	T cell activation	0.4133	0.57596	0	0	11	547
GO:0001964 startle response	1	0.41373	0.31	0.034	Biological	GO:00019	startle response	0.41373	0.57596	0	0	1	29
GO:0002474 antigen processing and presentation of peptide antigen via	1	0.41373	0.31	0.034	Biological	GO:00024	antigen processing and presentation of peptide antigen via MHC clas	0.41373	0.57596	0	0	1	29
GO:0003209 cardiac atrium morphogenesis	1	0.41373	0.31	0.034	Biological	GO:00032	cardiac atrium morphogenesis	0.41373	0.57596	0	0	1	29
GO:0003416 endochondral bone growth	1	0.41373	0.31	0.034	Biological	GO:00034	endochondral bone growth	0.41373	0.57596	0	0	1	29
GO:0007190 activation of adenylate cyclase activity	1	0.41373	0.31	0.034	Biological	GO:00071	activation of adenylate cyclase activity	0.41373	0.57596	0	0	1	29
GO:0010758 regulation of macrophage chemotaxis	1	0.41373	0.31	0.034	Biological	GO:00107	regulation of macrophage chemotaxis	0.41373	0.57596	0	0	1	29
GO:0010880 regulation of release of sequestered calcium ion into cytos	1	0.41373	0.31	0.034	Biological	GO:00108	regulation of release of sequestered calcium ion into cytosol by sarco	0.41373	0.57596	0	0	1	29
GO:0010955 negative regulation of protein processing	1	0.41373	0.31	0.034	Biological	GO:00109	negative regulation of protein processing	0.41373	0.57596	0	0	1	29
GO:0016048 detection of temperature stimulus	1	0.41373	0.31	0.034	Biological	GO:0016(	detection of temperature stimulus	0.41373	0.57596	0	0	1	29
GO:0030262 apoptotic nuclear changes	1	0.41373	0.31	0.034	Biological	GO:00302	apoptotic nuclear changes	0.41373	0.57596	0	0	1	29
GO:0030431 sleep	1	0.41373	0.31	0.034	Biological	GO:00304	sleep	0.41373	0.57596	0	0	1	29
GO:0032148 activation of protein kinase B activity	1	0.41373	0.31	0.034	Biological	GO:00321	activation of protein kinase B activity	0.41373	0.57596	0	0	1	29
GO:0035886 vascular associated smooth muscle cell differentiation	1	0.41373	0.31	0.034	Biological	GO:00358	vascular associated smooth muscle cell differentiation	0.41373	0.57596	0	0	1	29
GO:0042634 regulation of hair cycle	1	0.41373	0.31	0.034	Biological	GO:0042(	regulation of hair cycle	0.41373	0.57596	0	0	1	29
GO:0046337 phosphatidylethanolamine metabolic process	1	0.41373	0.31	0.034	Biological	GO:00463	phosphatidylethanolamine metabolic process	0.41373	0.57596	0	0	1	29
GO:0051873 killing by host of symbiont cells	1	0.41373	0.31	0.034	Biological	GO:00518	killing by host of symbiont cells	0.41373	0.57596	0	0	1	29
GO:0055094 response to lipoprotein particle	1	0.41373	0.31	0.034	Biological	GO:0055(	response to lipoprotein particle	0.41373	0.57596	0	0	1	29
GO:0060142 regulation of syncytium formation by plasma membrane fu	1	0.41373	0.31	0.034	Biological	GO:00601	regulation of syncytium formation by plasma membrane fusion	0.41373	0.57596	0	0	1	29
GO:0060441 epithelial tube branching involved in lung morphogenesis	1	0.41373	0.31	0.034	Biological	GO:00604	epithelial tube branching involved in lung morphogenesis	0.41373	0.57596	0	0	1	29
GO:0065005 protein-lipid complex assembly	1	0.41373	0.31	0.034	Biological	GO:0065(	protein-lipid complex assembly	0.41373	0.57596	0	0	1	29
GO:0090322 regulation of superoxide metabolic process	1	0.41373	0.31	0.034	Biological	GO:00903	regulation of superoxide metabolic process	0.41373	0.57596	0	0	1	29
GO:1902914 regulation of protein polyubiquitination	1	0.41373	0.31	0.034	Biological	GO:19029	regulation of protein polyubiquitination	0.41373	0.57596	0	0	1	29
GO:1903318 negative regulation of protein maturation	1	0.41373	0.31	0.034	Biological	GO:19033	negative regulation of protein maturation	0.41373	0.57596	0	0	1	29
GO:1904376 negative regulation of protein localization to cell periphery	1	0.41373	0.31	0.034	Biological	GO:19043	negative regulation of protein localization to cell periphery	0.41373	0.57596	0	0	1	29
GO:0042177 negative regulation of protein catabolic process	3	0.41392	0.929	0.023	Biological	GO:00421	negative regulation of protein catabolic process	0.41392	0.57596	0	0	3	128
GO:0045471 response to ethanol	3	0.41392	0.929	0.023	Biological	GO:00454	response to ethanol	0.41392	0.57596	0	0	3	128
GO:1903828 negative regulation of cellular protein localization	3	0.41392	0.929	0.023	Biological	GO:19038	negative regulation of cellular protein localization	0.41392	0.57596	0	0	3	128
GO:0030665 clathrin-coated vesicle membrane	3	0.41653	0.89	0.023	Cellular C	GO:0030(	clathrin-coated vesicle membrane	0.41653	0.63307	0	0	3	130
GO:1905368 peptidase complex	3	0.41653	0.89	0.023	Cellular C	GO:19053	peptidase complex	0.41653	0.63307	0	0	3	130
GO:0051707 response to other organism	29	0.41674	8.978	0.019	Biological	GO:00517	response to other organism	0.41674	0.57972	0	0	29	1513
GO:1901135 carbohydrate derivative metabolic process	21	0.41707	6.502	0.019	Biological	GO:19011	carbohydrate derivative metabolic process	0.41707	0.57993	0	0	21	1083
GO:0007193 adenylate cyclase-inhibiting G protein-coupled receptor sig	2	0.41736	0.619	0.026	Biological	GO:00071	adenylate cyclase-inhibiting G protein-coupled receptor signaling patl	0.41736	0.57993	0	0	2	78
GO:0046470 phosphatidylcholine metabolic process	2	0.41736	0.619	0.026	Biological	GO:00464	phosphatidylcholine metabolic process	0.41736	0.57993	0	0	2	78
GO:0120193 tight junction organization	2	0.41736	0.619	0.026	Biological	GO:01201	tight junction organization	0.41736	0.57993	0	0	2	78
GO:0042060 wound healing	9	0.41824	2.786	0.02	Biological	GO:0042(	wound healing	0.41824	0.581	0	0	9	443
GO:0030218 erythrocyte differentiation	3	0.41879	0.929	0.023	Biological	GO:00302	erythrocyte differentiation	0.41879	0.5816	0	0	3	129

GO:0008157 protein phosphatase 1 binding	1	0.41898	0.292	0.034	Molecular	GO:0008	protein phosphatase 1 binding	0.41898	0.59217	0	0	1	29
GO:0070325 lipoprotein particle receptor binding	1	0.41898	0.292	0.034	Molecular	GO:0070	lipoprotein particle receptor binding	0.41898	0.59217	0	0	1	29
GO:0015370 solute:sodium symporter activity	2	0.41917	0.585	0.026	Molecular	GO:0015	solute:sodium symporter activity	0.41917	0.59217	0	0	2	77
GO:0071241 cellular response to inorganic substance	5	0.42015	1.548	0.021	Biological	GO:0071	cellular response to inorganic substance	0.42015	0.5828	0	0	5	233
GO:0005963 magnesium-dependent protein serine/threonine phosphata	1	0.42081	0.297	0.033	Cellular C	GO:0005	magnesium-dependent protein serine/threonine phosphatase comple	0.42081	0.63386	0	0	1	30
GO:0031528 microvillus membrane	1	0.42081	0.297	0.033	Cellular C	GO:0031	microvillus membrane	0.42081	0.63386	0	0	1	30
GO:0032839 dendrite cytoplasm	1	0.42081	0.297	0.033	Cellular C	GO:0032	dendrite cytoplasm	0.42081	0.63386	0	0	1	30
GO:0043207 response to external biotic stimulus	29	0.42106	8.978	0.019	Biological	GO:0043	response to external biotic stimulus	0.42106	0.5828	0	0	29	1516
GO:0015267 channel activity	10	0.42109	2.924	0.02	Molecular	GO:0015	channel activity	0.42109	0.59392	0	0	10	489
GO:0061134 peptidase regulator activity	5	0.42316	1.462	0.022	Molecular	GO:0061	peptidase regulator activity	0.42316	0.5955	0	0	5	230
GO:0044282 small molecule catabolic process	8	0.42346	2.477	0.02	Biological	GO:0044	small molecule catabolic process	0.42346	0.5828	0	0	8	392
GO:0022803 passive transmembrane transporter activity	10	0.42359	2.924	0.02	Molecular	GO:0022	passive transmembrane transporter activity	0.42359	0.5955	0	0	10	490
GO:0006639 acylglycerol metabolic process	3	0.42364	0.929	0.023	Biological	GO:0006	acylglycerol metabolic process	0.42364	0.5828	0	0	3	130
GO:0010565 regulation of cellular ketone metabolic process	3	0.42364	0.929	0.023	Biological	GO:0010	regulation of cellular ketone metabolic process	0.42364	0.5828	0	0	3	130
GO:0061041 regulation of wound healing	3	0.42364	0.929	0.023	Biological	GO:0061	regulation of wound healing	0.42364	0.5828	0	0	3	130
GO:0008652 cellular amino acid biosynthetic process	2	0.42366	0.619	0.025	Biological	GO:0008	cellular amino acid biosynthetic process	0.42366	0.5828	0	0	2	79
GO:0030500 regulation of bone mineralization	2	0.42366	0.619	0.025	Biological	GO:0030	regulation of bone mineralization	0.42366	0.5828	0	0	2	79
GO:0051966 regulation of synaptic transmission, glutamatergic	2	0.42366	0.619	0.025	Biological	GO:0051	regulation of synaptic transmission, glutamatergic	0.42366	0.5828	0	0	2	79
GO:0009636 response to toxic substance	5	0.42374	1.548	0.021	Biological	GO:0009	response to toxic substance	0.42374	0.5828	0	0	5	234
GO:0031300 intrinsic component of organelle membrane	2	0.42427	0.593	0.025	Cellular C	GO:0031	intrinsic component of organelle membrane	0.42427	0.63386	0	0	2	80
GO:0031301 integral component of organelle membrane	2	0.42427	0.593	0.025	Cellular C	GO:0031	integral component of organelle membrane	0.42427	0.63386	0	0	2	80
GO:0002360 T cell lineage commitment	1	0.42444	0.31	0.033	Biological	GO:0002	T cell lineage commitment	0.42444	0.5828	0	0	1	30
GO:0002825 regulation of T-helper 1 type immune response	1	0.42444	0.31	0.033	Biological	GO:0002	regulation of T-helper 1 type immune response	0.42444	0.5828	0	0	1	30
GO:0006622 protein targeting to lysosome	1	0.42444	0.31	0.033	Biological	GO:0006	protein targeting to lysosome	0.42444	0.5828	0	0	1	30
GO:0006656 phosphatidylcholine biosynthetic process	1	0.42444	0.31	0.033	Biological	GO:0006	phosphatidylcholine biosynthetic process	0.42444	0.5828	0	0	1	30
GO:0007202 activation of phospholipase C activity	1	0.42444	0.31	0.033	Biological	GO:0007	activation of phospholipase C activity	0.42444	0.5828	0	0	1	30
GO:0014741 negative regulation of muscle hypertrophy	1	0.42444	0.31	0.033	Biological	GO:0014	negative regulation of muscle hypertrophy	0.42444	0.5828	0	0	1	30
GO:0015740 C4-dicarboxylate transport	1	0.42444	0.31	0.033	Biological	GO:0015	C4-dicarboxylate transport	0.42444	0.5828	0	0	1	30
GO:0015813 L-glutamate transmembrane transport	1	0.42444	0.31	0.033	Biological	GO:0015	L-glutamate transmembrane transport	0.42444	0.5828	0	0	1	30
GO:0018200 peptidyl-glutamic acid modification	1	0.42444	0.31	0.033	Biological	GO:0018	peptidyl-glutamic acid modification	0.42444	0.5828	0	0	1	30
GO:0030947 regulation of vascular endothelial growth factor receptor si	1	0.42444	0.31	0.033	Biological	GO:0030	regulation of vascular endothelial growth factor receptor signaling pat	0.42444	0.5828	0	0	1	30
GO:0031365 N-terminal protein amino acid modification	1	0.42444	0.31	0.033	Biological	GO:0031	N-terminal protein amino acid modification	0.42444	0.5828	0	0	1	30
GO:0034067 protein localization to Golgi apparatus	1	0.42444	0.31	0.033	Biological	GO:0034	protein localization to Golgi apparatus	0.42444	0.5828	0	0	1	30
GO:0034368 protein-lipid complex remodeling	1	0.42444	0.31	0.033	Biological	GO:0034	protein-lipid complex remodeling	0.42444	0.5828	0	0	1	30
GO:0034369 plasma lipoprotein particle remodeling	1	0.42444	0.31	0.033	Biological	GO:0034	plasma lipoprotein particle remodeling	0.42444	0.5828	0	0	1	30
GO:0034698 response to gonadotropin	1	0.42444	0.31	0.033	Biological	GO:0034	response to gonadotropin	0.42444	0.5828	0	0	1	30
GO:0035521 monoubiquitinated histone deubiquitination	1	0.42444	0.31	0.033	Biological	GO:0035	monoubiquitinated histone deubiquitination	0.42444	0.5828	0	0	1	30
GO:0035522 monoubiquitinated histone H2A deubiquitination	1	0.42444	0.31	0.033	Biological	GO:0035	monoubiquitinated histone H2A deubiquitination	0.42444	0.5828	0	0	1	30
GO:0035774 positive regulation of insulin secretion involved in cellular re	1	0.42444	0.31	0.033	Biological	GO:0035	positive regulation of insulin secretion involved in cellular response to	0.42444	0.5828	0	0	1	30
GO:0043094 cellular metabolic compound salvage	1	0.42444	0.31	0.033	Biological	GO:0043	cellular metabolic compound salvage	0.42444	0.5828	0	0	1	30
GO:0043171 peptide catabolic process	1	0.42444	0.31	0.033	Biological	GO:0043	peptide catabolic process	0.42444	0.5828	0	0	1	30
GO:0044342 type B pancreatic cell proliferation	1	0.42444	0.31	0.033	Biological	GO:0044	type B pancreatic cell proliferation	0.42444	0.5828	0	0	1	30
GO:0050820 positive regulation of coagulation	1	0.42444	0.31	0.033	Biological	GO:0050	positive regulation of coagulation	0.42444	0.5828	0	0	1	30
GO:0060674 placenta blood vessel development	1	0.42444	0.31	0.033	Biological	GO:0060	placenta blood vessel development	0.42444	0.5828	0	0	1	30
GO:0072539 T-helper 17 cell differentiation	1	0.42444	0.31	0.033	Biological	GO:0072	T-helper 17 cell differentiation	0.42444	0.5828	0	0	1	30
GO:0090140 regulation of mitochondrial fission	1	0.42444	0.31	0.033	Biological	GO:0090	regulation of mitochondrial fission	0.42444	0.5828	0	0	1	30
GO:0090382 phagosome maturation	1	0.42444	0.31	0.033	Biological	GO:0090	phagosome maturation	0.42444	0.5828	0	0	1	30
GO:0097066 response to thyroid hormone	1	0.42444	0.31	0.033	Biological	GO:0097	response to thyroid hormone	0.42444	0.5828	0	0	1	30
GO:0098901 regulation of cardiac muscle cell action potential	1	0.42444	0.31	0.033	Biological	GO:0098	regulation of cardiac muscle cell action potential	0.42444	0.5828	0	0	1	30
GO:0099505 regulation of presynaptic membrane potential	1	0.42444	0.31	0.033	Biological	GO:0099	regulation of presynaptic membrane potential	0.42444	0.5828	0	0	1	30
GO:0099558 maintenance of synapse structure	1	0.42444	0.31	0.033	Biological	GO:0099	maintenance of synapse structure	0.42444	0.5828	0	0	1	30
GO:1904994 regulation of leukocyte adhesion to vascular endothelial ce	1	0.42444	0.31	0.033	Biological	GO:1904	regulation of leukocyte adhesion to vascular endothelial cell	0.42444	0.5828	0	0	1	30
GO:0045296 cadherin binding	7	0.42481	2.047	0.021	Molecular	GO:0045	cadherin binding	0.42481	0.59624	0	0	7	334
GO:0043270 positive regulation of ion transport	6	0.42536	1.858	0.021	Biological	GO:0043	positive regulation of ion transport	0.42536	0.5839	0	0	6	287
GO:0009615 response to virus	8	0.42623	2.477	0.02	Biological	GO:0009	response to virus	0.42623	0.58494	0	0	8	393
GO:0072686 mitotic spindle	4	0.42833	1.187	0.022	Cellular C	GO:0072	mitotic spindle	0.42833	0.63386	0	0	4	185
GO:0032940 secretion by cell	16	0.42834	4.954	0.019	Biological	GO:0032	secretion by cell	0.42834	0.58755	0	0	16	821
GO:0006638 neutral lipid metabolic process	3	0.42848	0.929	0.023	Biological	GO:0006	neutral lipid metabolic process	0.42848	0.58755	0	0	3	131
GO:0099565 chemical synaptic transmission, postsynaptic	3	0.42848	0.929	0.023	Biological	GO:0099	chemical synaptic transmission, postsynaptic	0.42848	0.58755	0	0	3	131
GO:0098797 plasma membrane protein complex	12	0.42895	3.561	0.02	Cellular C	GO:0098	plasma membrane protein complex	0.42895	0.63386	0	0	12	614
GO:0005540 hyaluronic acid binding	1	0.42976	0.292	0.033	Molecular	GO:0005	hyaluronic acid binding	0.42976	0.59835	0	0	1	30
GO:0008378 galactosyltransferase activity	1	0.42976	0.292	0.033	Molecular	GO:0008	galactosyltransferase activity	0.42976	0.59835	0	0	1	30
GO:0016417 S-acyltransferase activity	1	0.42976	0.292	0.033	Molecular	GO:0016	S-acyltransferase activity	0.42976	0.59835	0	0	1	30
GO:0046875 ephrin receptor binding	1	0.42976	0.292	0.033	Molecular	GO:0046	ephrin receptor binding	0.42976	0.59835	0	0	1	30
GO:0070717 poly-purine tract binding	1	0.42976	0.292	0.033	Molecular	GO:0070	poly-purine tract binding	0.42976	0.59835	0	0	1	30
GO:0007260 tyrosine phosphorylation of STAT protein	2	0.42993	0.619	0.025	Biological	GO:0007	tyrosine phosphorylation of STAT protein	0.42993	0.58888	0	0	2	80
GO:0050772 positive regulation of axonogenesis	2	0.42993	0.619	0.025	Biological	GO:0050	positive regulation of axonogenesis	0.42993	0.58888	0	0	2	80
GO:0072091 regulation of stem cell proliferation	2	0.42993	0.619	0.025	Biological	GO:0072	regulation of stem cell proliferation	0.42993	0.58888	0	0	2	80
GO:1901616 organic hydroxy compound catabolic process	2	0.42993	0.619	0.025	Biological	GO:1901	organic hydroxy compound catabolic process	0.42993	0.58888	0	0	2	80
GO:0002102 podosome	1	0.43126	0.297	0.032	Cellular C	GO:0002	podosome	0.43126	0.63386	0	0	1	31
GO:0008305 integrin complex	1	0.43126	0.297	0.032	Cellular C	GO:0008	integrin complex	0.43126	0.63386	0	0	1	31
GO:0031941 filamentous actin	1	0.43126	0.297	0.032	Cellular C	GO:0031	filamentous actin	0.43126	0.63386	0	0	1	31
GO:0044298 cell body membrane	1	0.43126	0.297	0.032	Cellular C	GO:0044	cell body membrane	0.43126	0.63386	0	0	1	31
GO:0070971 endoplasmic reticulum exit site	1	0.43126	0.297	0.032	Cellular C	GO:0070	endoplasmic reticulum exit site	0.43126	0.63386	0	0	1	31
GO:0048545 response to steroid hormone	7	0.43202	2.167	0.02	Biological	GO:0048	response to steroid hormone	0.43202	0.59158	0	0	7	342
GO:0007219 Notch signaling pathway	4	0.43273	1.238	0.022	Biological	GO:0007	Notch signaling pathway	0.43273	0.5917	0	0	4	184
GO:0002702 positive regulation of production of molecular mediator of ir	3	0.43331	0.929	0.023	Biological	GO:0002	positive regulation of production of molecular mediator of immune res	0.43331	0.5917	0	0	3	132
GO:0035303 regulation of dephosphorylation	3	0.43331	0.929	0.023	Biological	GO:0035	regulation of dephosphorylation	0.43331	0.5917	0	0	3	132
GO:0006351 transcription, DNA-templated	67	0.43431	20.743	0.019	Biological	GO:0006	transcription, DNA-templated	0.43431	0.5917	0	0	67	3588
GO:0051403 stress-activated MAPK cascade	5	0.43449	1.548	0.021	Biological	GO:0051	stress-activated MAPK cascade	0.43449	0.5917	0	0	5	237
GO:0001958 endochondral ossification	1	0.43495	0.31	0.032	Biological	GO:0001	endochondral ossification	0.43495	0.5917	0	0	1	31
GO:0006921 cellular component disassembly involved in execution phas	1	0.43495	0.31	0.032	Biological	GO:0006	cellular component disassembly involved in execution phase of apopt	0.43495	0.5917	0	0	1	31
GO:0008156 negative regulation of DNA replication	1	0.43495	0.31	0.032	Biological	GO:0008	negative regulation of DNA replication	0.43495	0.5917	0	0	1	31
GO:0010039 response to iron ion	1	0.43495	0.31	0.032	Biological	GO:0010	response to iron ion	0.43495	0.5917	0	0	1	31
GO:0010669 epithelial structure maintenance	1	0.43495	0.31	0.032	Biological	GO:0010	epithelial structure maintenance	0.43495	0.5917	0	0	1	31
GO:0021522 spinal cord motor neuron differentiation	1	0.43495	0.31	0.032	Biological	GO:0021	spinal cord motor neuron differentiation	0.43495	0.5917	0	0	1	31
GO:0036075 replacement ossification	1	0.43495	0.31	0.032	Biological	GO:0036	replacement ossification	0.43495	0.5917	0	0	1	31
GO:0045648 positive regulation of erythrocyte differentiation	1	0.43495	0.31	0.032	Biological	GO:0045	positive regulation of erythrocyte differentiation	0.43495	0.5917	0	0	1	31
GO:0045907 positive regulation of vasoconstriction	1	0.43495	0.31	0.032	Biological	GO:0045	positive regulation of vasoconstriction	0.43495	0.5917	0	0	1	31
GO:0050951 sensory perception of temperature stimulus	1	0.43495	0.31	0.032	Biological	GO:0050	sensory perception of temperature stimulus	0.43495	0.5917	0	0	1	31
GO:0051883 killing of cells in other organism involved in symbiotic intera	1	0.43495	0.31	0.032	Biological	GO:0051	killing of cells in other organism involved in symbiotic interaction	0.43495	0.5917	0	0	1	31
GO:0061037 negative regulation of cartilage development	1	0.43495	0.31	0.032	Biological	GO:0061	negative regulation of cartilage development	0.43495	0.5917	0	0	1	31
GO:0061099 negative regulation of protein tyrosine kinase activity	1	0.43495	0.31	0.032	Biological	GO:0061	negative regulation of protein tyrosine kinase activity	0.43495	0.5917	0	0	1	31
GO:0090330 regulation of platelet aggregation	1	0.43495	0.31	0.032	Biological	GO:0090	regulation of platelet aggregation	0.43495	0.5917	0	0	1	31
GO:0099590 neurotransmitter receptor internalization	1	0.43495	0.31	0.032	Biological	GO:0099	neurotransmitter receptor internalization	0.43495	0.5917	0	0	1	31
GO:0150117 positive regulation of cell-substrate junction organization	1	0.43495	0.31	0.032	Biological	GO:0150	positive regulation of cell-substrate junction organization	0.43495	0.5917	0	0	1	31
GO:1900225 regulation of NLRP3 inflammasome complex assembly	1	0.43495	0.31	0.032	Biological	GO:1900	regulation of NLRP3 inflammasome complex assembly	0.43495	0.5917	0	0	1	31
GO:1903715 regulation of aerobic respiration	1	0.43495	0.31	0.032	Biological	GO:1903	regulation of aerobic respiration	0.43495	0.5917	0	0	1	31
GO:2000463 positive regulation of excitatory postsynaptic potential	1	0.43495	0.31	0.032	Biological	GO:2000	positive regulation of excitatory postsynaptic potential	0.43495	0.5917	0	0	1	31
GO:0097659 nucleic acid-templated transcription	67	0.43532	20.743	0.019	Biological	GO:0097	nucleic acid-templated transcription	0.43532	0.59204	0	0	67	3589
GO:0072665 protein localization to vacuole	2	0.43616	0.619	0.025	Biological	GO:0072	protein localization to vacuole	0.43616	0.59286	0	0	2	81
GO:2001259 positive regulation of cation channel activity	2	0.43616	0.619	0.025	Biological	GO:2001	positive regulation of cation channel activity	0.43616	0.59286	0	0	2	81
GO:0005310 dicarboxylic acid transmembrane transporter activity	1	0.44035	0.292	0.032	Molecular	GO:0005	dicarboxylic acid transmembrane transporter activity	0.44035	0.60917	0	0	1	31
GO:0016411 acylglycerol O-acyltransferase activity	1	0.44035	0.292	0.032	Molecular	GO:0016	acylglycerol O-acyltransferase activity	0.44035	0.60917	0	0	1	31
GO:0043425 bHLH transcription factor binding	1	0.44035	0.292	0.032	Molecular	GO:0043	bHLH transcription factor binding	0.44035	0.60917	0	0	1	31
GO:0070273 phosphatidylinositol-4-phosphate binding	1	0.44035	0.292	0.032	Molecular	GO:0070	phosphatidylinositol-4-phosphate binding	0.44035	0.60917	0	0	1	31
GO:0097731 9+0 non-motile cilium	3	0.44041	0.89	0.022	Cellular C	GO:0097	9+0 non-motile cilium	0.44041	0.64541	0	0	3	135
GO:2001257 regulation of cation channel activity	4	0.44085	1.238	0.022	Biological	GO:2001	regulation of cation channel activity	0.44085	0.59907	0	0	4	186
GO:0060090 molecular adaptor activity	10	0.44106	2.924	0.02	Molecular	GO:0060	molecular adaptor activity	0.44106	0.60919	0	0	10	497
GO:0032409 regulation of transporter activity	6	0.44151	1.858	0.021	Biological	GO:0032	regulation of transporter activity	0.44151	0.59981	0	0	6	292
GO:0016342 catenin complex	1	0.44153	0.297	0.031	Cellular C	GO:0016	catenin complex	0.44153	0.64541	0	0	1	32
GO:0050848 regulation of calcium-mediated signaling	2	0.44235	0.619	0.024	Biological	GO:0050	regulation of calcium-mediated signaling	0.44235	0.60034	0	0	2	82
GO:0051865 protein autoubiquitination	2	0.44235	0.619	0.024	Biological	GO:0051	protein autoubiquitination	0.44235	0.60034	0	0	2	82
GO:0015031 protein transport	30	0.44248	9.288	0.019	Biological	GO:0015	protein transport	0.44248	0.60034	0	0	30	1585
GO:0007264 small GTPase mediated signal transduction	10	0.44279	3.096	0.02	Biological	GO:0007	small GTPase mediated signal transduction	0.44279	0.60034	0	0	10	506
GO:0006897 endocytosis	13	0.44487	4.025	0.019	Biological	GO:0006	endocytosis	0.44487	0.60034	0	0	13	668
GO:0003309 type B pancreatic cell differentiation	1	0.44527	0.31	0.031	Biological	GO:0003	type B pancreatic cell differentiation	0.44527	0.60034	0	0	1	32
GO:0007271 synaptic transmission, cholinergic	1	0.44527	0.31	0.031	Biological	GO:0007	synaptic transmission, cholinergic	0.44527	0.60034	0	0	1	32
GO:0009072 aromatic amino acid family metabolic process	1	0.44527	0.31	0.031	Biological	GO:0009	aromatic amino acid family metabolic process	0.44527	0.60034	0	0	1	32
GO:0017145 stem cell division	1	0.44527	0.31	0.031	Biological	GO:0017	stem cell division	0.44527	0.60034	0	0	1	32
GO:0019835 cytolysis	1	0.44527	0.31	0.031	Biological	GO:0019	cytolysis	0.44527	0.60034	0	0	1	32
GO:0030511 positive regulation of transforming growth factor beta rece	1	0.44527	0.31	0.031	Biological	GO:0030	positive regulation of transforming growth factor beta receptor signali	0.44527	0.60034	0	0	1	32
GO:0031116 positive regulation of microtubule polymerization	1	0.44527	0.31	0.031	Biological	GO:0031	positive regulation of microtubule polymerization	0.44527	0.60034	0	0	1	32
GO:0033032 regulation of myeloid cell apoptotic process	1	0.44527	0.31	0.031	Biological	GO:0033	regulation of myeloid cell apoptotic process	0.44527	0.60034	0	0	1	32
GO:0034367 protein-containing complex remodeling	1	0.44527	0.31	0.031	Biological	GO:0034	protein-containing complex remodeling	0.44527	0.60034	0	0	1	32
GO:0035115 embryonic forelimb morphogenesis	1	0.44527	0.31	0.031	Biological	GO:0035	embryonic forelimb morphogenesis	0.44527	0.60034	0	0	1	32
GO:0045026 plasma membrane fusion	1	0.44527	0.31	0.031	Biological	GO:0045	plasma membrane fusion	0.44527	0.60034	0	0	1	32
GO:0045070 positive regulation of viral genome replication	1	0.44527	0.31	0.031	Biological	GO:0045	positive regulation of viral genome replication	0.44527	0.60034	0	0	1	32
GO:0045684 positive regulation of epidermis development	1	0.44527	0.31	0.031	Biological	GO:0045	positive regulation of epidermis development	0.44527	0.60034	0	0	1	32
GO:0048240 sperm capacitation	1	0.44527	0.31	0.031	Biological	GO:0048	sperm capacitation	0.44527	0.60034	0	0	1	32
GO:0060421 positive regulation of heart growth	1	0.44527	0.31	0.031	Biological	GO:0060	positive regulation of heart growth	0.44527	0.60034	0	0	1	32
GO:0061436 establishment of skin barrier	1	0.44527	0.31	0.031	Biological	GO:0061	establishment of skin barrier	0.44527	0.60034	0	0	1	32
GO:0071295 cellular response to vitamin	1	0.44527	0.31	0.031	Biological	GO:0071	cellular response to vitamin	0.44527	0.60034	0	0	1	32
GO:0071402 cellular response to lipoprotein particle stimulus	1	0.44527	0.31	0.031	Biological	GO:0071	cellular response to lipoprotein particle stimulus	0.44527	0.60034	0	0	1	32
GO:0098868 bone growth	1	0.44527	0.31	0.031	Biological	GO:0098	bone growth	0.44527	0.60034	0	0	1	32
GO:0140058 neuron projection arborization	1	0.44527	0.31	0.031	Biological	GO:0140	neuron projection arborization	0.44527	0.60034	0	0	1	32
GO:1901185 negative regulation of ERBB signaling pathway	1	0.44527	0.31	0.031	Biological	GO:1901	negative regulation of ERBB signaling pathway	0.44527	0.60034	0	0	1	32
GO:1901798 positive regulation of signal transduction by p53 class med	1	0.44527	0.31	0.031	Biological	GO:1901	positive regulation of signal transduction by p53 class mediator	0.44527	0.60034	0	0	1	32
GO:1903846 positive regulation of cellular response to transforming gro	1	0.44527	0.31	0.031	Biological	GO:1903	positive regulation of cellular response to transforming growth factor	0.44527	0.60034	0	0	1	32
GO:0000380 alternative mRNA splicing, via spliceosome	2	0.44851	0.619	0.024	Biological	GO:0000	alternative mRNA splicing, via spliceosome	0.44851	0.60406	0	0	2	83
GO:0008625 extrinsic apoptotic signaling pathway via death domain rec	2	0.44851	0.619	0.024	Biological	GO:0008	extrinsic apoptotic signaling pathway via death domain receptors	0.44851	0.60406	0	0	2	83
GO:0014897 striated muscle hypertrophy	2	0.44851	0.619	0.024	Biological	GO:0014	striated muscle hypertrophy	0.44851	0.60406	0	0	2	83
GO:0061515 myeloid cell development	2	0.44851	0.619	0.024	Biological	GO:0061	myeloid cell development	0.44851	0.60406	0	0	2	83
GO:0008361 regulation of cell size	4	0.44892	1.238	0.021	Biological	GO:0008	regulation of cell size	0.44892	0.60446	0	0	4	188
GO:0072562 blood microparticle	3	0.44986	0.89	0.022	Cellular C	GO:0072	blood microparticle	0.44986	0.65579	0	0	3	137
GO:0016878 acid-thiol ligase activity	1	0.45074	0.292	0.031	Molecular	GO:0016	acid-thiol ligase activity	0.45074	0.61965	0	0	1	32
GO:0019199 transmembrane receptor protein kinase activity	2	0.45078	0.585	0.024	Molecular	GO:0019	transmembrane receptor protein kinase activity	0.45078	0.61965	0	0	2	82
GO:0048306 calcium-dependent protein binding	2	0.45078	0.585	0.024	Molecular	GO:0048	calcium-dependent protein binding	0.45078	0.61965	0	0	2	82
GO:0098576 lumenal side of membrane	1	0.45162	0.297	0.03	Cellular C	GO:0098	lumenal side of membrane	0.45162	0.65594	0	0	1	33
GO:0060048 cardiac muscle contraction	3	0.45246	0.929	0.022	Biological	GO:0060	cardiac muscle contraction	0.45246	0.60858	0	0	3	136
GO:1903530 regulation of secretion by cell	11	0.45301	3.406	0.02	Biological	GO:1903	regulation of secretion by cell	0.45301	0.60858	0	0	11	564
GO:0032774 RNA biosynthetic process	67	0.45448	20.743	0.019	Biological	GO:0032	RNA biosynthetic process	0.45448	0.60858	0	0	67	3608

GO:0042383 sarcolemma	3	0.45456	0.89	0.022	Cellular C	GO:00423	sarcolemma	0.45456	0.65594	0	0	3	138
GO:0015630 microtubule cytoskeleton	26	0.45458	7.715	0.019	Cellular C	GO:00156	microtubule cytoskeleton	0.45458	0.65594	0	0	26	1392
GO:0010770 positive regulation of cell morphogenesis involved in differe	2	0.45463	0.619	0.024	Biological	GO:00107	positive regulation of cell morphogenesis involved in differentiation	0.45463	0.60858	0	0	2	84
GO:0000118 histone deacetylase complex	2	0.45487	0.593	0.024	Cellular C	GO:00001	histone deacetylase complex	0.45487	0.65594	0	0	2	85
GO:0002063 chondrocyte development	1	0.4554	0.31	0.03	Biological	GO:00020	chondrocyte development	0.4554	0.60858	0	0	1	33
GO:0002719 negative regulation of cytokine production involved in immu	1	0.4554	0.31	0.03	Biological	GO:00027	negative regulation of cytokine production involved in immune respon	0.4554	0.60858	0	0	1	33
GO:0006026 aminoglycan catabolic process	1	0.4554	0.31	0.03	Biological	GO:00060	aminoglycan catabolic process	0.4554	0.60858	0	0	1	33
GO:0006308 DNA catabolic process	1	0.4554	0.31	0.03	Biological	GO:00063	DNA catabolic process	0.4554	0.60858	0	0	1	33
GO:0006516 glycoprotein catabolic process	1	0.4554	0.31	0.03	Biological	GO:00065	glycoprotein catabolic process	0.4554	0.60858	0	0	1	33
GO:0014808 release of sequestered calcium ion into cytosol by sarcopla	1	0.4554	0.31	0.03	Biological	GO:00148	release of sequestered calcium ion into cytosol by sarcoplasmic retic	0.4554	0.60858	0	0	1	33
GO:0018345 protein palmitoylation	1	0.4554	0.31	0.03	Biological	GO:00183	protein palmitoylation	0.4554	0.60858	0	0	1	33
GO:0030204 chondroitin sulfate metabolic process	1	0.4554	0.31	0.03	Biological	GO:00302	chondroitin sulfate metabolic process	0.4554	0.60858	0	0	1	33
GO:0033198 response to ATP	1	0.4554	0.31	0.03	Biological	GO:00331	response to ATP	0.4554	0.60858	0	0	1	33
GO:0034260 negative regulation of GTPase activity	1	0.4554	0.31	0.03	Biological	GO:00342	negative regulation of GTPase activity	0.4554	0.60858	0	0	1	33
GO:0034389 lipid droplet organization	1	0.4554	0.31	0.03	Biological	GO:00343	lipid droplet organization	0.4554	0.60858	0	0	1	33
GO:0040018 positive regulation of multicellular organism growth	1	0.4554	0.31	0.03	Biological	GO:00400	positive regulation of multicellular organism growth	0.4554	0.60858	0	0	1	33
GO:0040020 regulation of meiotic nuclear division	1	0.4554	0.31	0.03	Biological	GO:00400	regulation of meiotic nuclear division	0.4554	0.60858	0	0	1	33
GO:0042558 pteridine-containing compound metabolic process	1	0.4554	0.31	0.03	Biological	GO:00425	pteridine-containing compound metabolic process	0.4554	0.60858	0	0	1	33
GO:0043372 positive regulation of CD4-positive, alpha-beta T cell differe	1	0.4554	0.31	0.03	Biological	GO:00433	positive regulation of CD4-positive, alpha-beta T cell differentiation	0.4554	0.60858	0	0	1	33
GO:0043552 positive regulation of phosphatidylinositol 3-kinase activity	1	0.4554	0.31	0.03	Biological	GO:00435	positive regulation of phosphatidylinositol 3-kinase activity	0.4554	0.60858	0	0	1	33
GO:0045761 regulation of adenylate cyclase activity	1	0.4554	0.31	0.03	Biological	GO:00457	regulation of adenylate cyclase activity	0.4554	0.60858	0	0	1	33
GO:0050901 leukocyte tethering or rolling	1	0.4554	0.31	0.03	Biological	GO:00509	leukocyte tethering or rolling	0.4554	0.60858	0	0	1	33
GO:0051642 centrosome localization	1	0.4554	0.31	0.03	Biological	GO:00516	centrosome localization	0.4554	0.60858	0	0	1	33
GO:0060317 cardiac epithelial to mesenchymal transition	1	0.4554	0.31	0.03	Biological	GO:00603	cardiac epithelial to mesenchymal transition	0.4554	0.60858	0	0	1	33
GO:0061036 positive regulation of cartilage development	1	0.4554	0.31	0.03	Biological	GO:00610	positive regulation of cartilage development	0.4554	0.60858	0	0	1	33
GO:0061842 microtubule organizing center localization	1	0.4554	0.31	0.03	Biological	GO:00618	microtubule organizing center localization	0.4554	0.60858	0	0	1	33
GO:0071425 hematopoietic stem cell proliferation	1	0.4554	0.31	0.03	Biological	GO:00714	hematopoietic stem cell proliferation	0.4554	0.60858	0	0	1	33
GO:0086005 ventricular cardiac muscle cell action potential	1	0.4554	0.31	0.03	Biological	GO:00860	ventricular cardiac muscle cell action potential	0.4554	0.60858	0	0	1	33
GO:0019887 protein kinase regulator activity	5	0.4558	1.462	0.021	Molecular	GO:00198	protein kinase regulator activity	0.4558	0.62555	0	0	5	239
GO:0051302 regulation of cell division	4	0.45697	1.238	0.021	Biological	GO:00513	regulation of cell division	0.45697	0.61034	0	0	4	190
GO:0007586 digestion	3	0.45721	0.929	0.022	Biological	GO:00075	digestion	0.45721	0.61034	0	0	3	137
GO:0051147 regulation of muscle cell differentiation	3	0.45721	0.929	0.022	Biological	GO:00511	regulation of muscle cell differentiation	0.45721	0.61034	0	0	3	137
GO:1904019 epithelial cell apoptotic process	3	0.45721	0.929	0.022	Biological	GO:19040	epithelial cell apoptotic process	0.45721	0.61034	0	0	3	137
GO:0044092 negative regulation of molecular function	19	0.45827	5.882	0.019	Biological	GO:00440	negative regulation of molecular function	0.45827	0.61159	0	0	19	999
GO:0019318 hexose metabolic process	5	0.45938	1.548	0.02	Biological	GO:00193	hexose metabolic process	0.45938	0.61292	0	0	5	244
GO:0014896 muscle hypertrophy	2	0.46071	0.619	0.024	Biological	GO:00148	muscle hypertrophy	0.46071	0.61429	0	0	2	85
GO:0045069 regulation of viral genome replication	2	0.46071	0.619	0.024	Biological	GO:00450	regulation of viral genome replication	0.46071	0.61429	0	0	2	85
GO:0002366 leukocyte activation involved in immune response	6	0.46078	1.858	0.02	Biological	GO:00023	leukocyte activation involved in immune response	0.46078	0.61429	0	0	6	298
GO:0005251 delayed rectifier potassium channel activity	1	0.46094	0.292	0.03	Molecular	GO:00052	delayed rectifier potassium channel activity	0.46094	0.62664	0	0	1	33
GO:0017080 sodium channel regulator activity	1	0.46094	0.292	0.03	Molecular	GO:00170	sodium channel regulator activity	0.46094	0.62664	0	0	1	33
GO:0019239 deaminase activity	1	0.46094	0.292	0.03	Molecular	GO:00192	deaminase activity	0.46094	0.62664	0	0	1	33
GO:0030332 cyclin binding	1	0.46094	0.292	0.03	Molecular	GO:00303	cyclin binding	0.46094	0.62664	0	0	1	33
GO:0042287 MHC protein binding	1	0.46094	0.292	0.03	Molecular	GO:00422	MHC protein binding	0.46094	0.62664	0	0	1	33
GO:0140662 ATP-dependent protein folding chaperone	1	0.46094	0.292	0.03	Molecular	GO:01406	ATP-dependent protein folding chaperone	0.46094	0.62664	0	0	1	33
GO:0032872 regulation of stress-activated MAPK cascade	4	0.46097	1.238	0.021	Biological	GO:00328	regulation of stress-activated MAPK cascade	0.46097	0.61438	0	0	4	191
GO:0090543 Flemming body	1	0.46152	0.297	0.029	Cellular C	GO:00905	Flemming body	0.46152	0.66374	0	0	1	34
GO:0006986 response to unfolded protein	3	0.46193	0.929	0.022	Biological	GO:00069	response to unfolded protein	0.46193	0.61534	0	0	3	138
GO:0034101 erythrocyte homeostasis	3	0.46193	0.929	0.022	Biological	GO:00341	erythrocyte homeostasis	0.46193	0.61534	0	0	3	138
GO:0006914 autophagy	11	0.46233	3.406	0.019	Biological	GO:00069	autophagy	0.46233	0.61553	0	0	11	568
GO:0061919 process utilizing autophagic mechanism	11	0.46233	3.406	0.019	Biological	GO:00619	process utilizing autophagic mechanism	0.46233	0.61553	0	0	11	568
GO:0031098 stress-activated protein kinase signaling cascade	5	0.46292	1.548	0.02	Biological	GO:00310	stress-activated protein kinase signaling cascade	0.46292	0.61593	0	0	5	245
GO:0071216 cellular response to biotic stimulus	5	0.46292	1.548	0.02	Biological	GO:00712	cellular response to biotic stimulus	0.46292	0.61593	0	0	5	245
GO:0006520 cellular amino acid metabolic process	6	0.46398	1.858	0.02	Biological	GO:00065	cellular amino acid metabolic process	0.46398	0.61593	0	0	6	299
GO:0030662 coated vesicle membrane	4	0.46422	1.187	0.021	Cellular C	GO:00306	coated vesicle membrane	0.46422	0.66583	0	0	4	194
GO:0010959 regulation of metal ion transport	8	0.46482	2.477	0.02	Biological	GO:00109	regulation of metal ion transport	0.46482	0.61593	0	0	8	407
GO:0007369 gastrulation	4	0.46497	1.238	0.021	Biological	GO:00073	gastrulation	0.46497	0.61593	0	0	4	192
GO:0002021 response to dietary excess	1	0.46534	0.31	0.029	Biological	GO:00020	response to dietary excess	0.46534	0.61593	0	0	1	34
GO:0003382 epithelial cell morphogenesis	1	0.46534	0.31	0.029	Biological	GO:00033	epithelial cell morphogenesis	0.46534	0.61593	0	0	1	34
GO:0009112 nucleobase metabolic process	1	0.46534	0.31	0.029	Biological	GO:00091	nucleobase metabolic process	0.46534	0.61593	0	0	1	34
GO:0030212 hyaluronan metabolic process	1	0.46534	0.31	0.029	Biological	GO:00302	hyaluronan metabolic process	0.46534	0.61593	0	0	1	34
GO:0033561 regulation of water loss via skin	1	0.46534	0.31	0.029	Biological	GO:00335	regulation of water loss via skin	0.46534	0.61593	0	0	1	34
GO:0035025 positive regulation of Rho protein signal transduction	1	0.46534	0.31	0.029	Biological	GO:00350	positive regulation of Rho protein signal transduction	0.46534	0.61593	0	0	1	34
GO:0035308 negative regulation of protein dephosphorylation	1	0.46534	0.31	0.029	Biological	GO:00353	negative regulation of protein dephosphorylation	0.46534	0.61593	0	0	1	34
GO:0036475 neuron death in response to oxidative stress	1	0.46534	0.31	0.029	Biological	GO:00364	neuron death in response to oxidative stress	0.46534	0.61593	0	0	1	34
GO:0039528 cytoplasmic pattern recognition receptor signaling pathway	1	0.46534	0.31	0.029	Biological	GO:00395	cytoplasmic pattern recognition receptor signaling pathway in respon	0.46534	0.61593	0	0	1	34
GO:0044546 NLRP3 inflammasome complex assembly	1	0.46534	0.31	0.029	Biological	GO:00445	NLRP3 inflammasome complex assembly	0.46534	0.61593	0	0	1	34
GO:0048265 response to pain	1	0.46534	0.31	0.029	Biological	GO:00482	response to pain	0.46534	0.61593	0	0	1	34
GO:0048841 regulation of axon extension involved in axon guidance	1	0.46534	0.31	0.029	Biological	GO:00488	regulation of axon extension involved in axon guidance	0.46534	0.61593	0	0	1	34
GO:0060074 synapse maturation	1	0.46534	0.31	0.029	Biological	GO:00600	synapse maturation	0.46534	0.61593	0	0	1	34
GO:0071354 cellular response to interleukin-6	1	0.46534	0.31	0.029	Biological	GO:00713	cellular response to interleukin-6	0.46534	0.61593	0	0	1	34
GO:0080111 DNA demethylation	1	0.46534	0.31	0.029	Biological	GO:00801	DNA demethylation	0.46534	0.61593	0	0	1	34
GO:1903514 release of sequestered calcium ion into cytosol by endopla	1	0.46534	0.31	0.029	Biological	GO:19035	release of sequestered calcium ion into cytosol by endoplasmic reticu	0.46534	0.61593	0	0	1	34
GO:2000406 positive regulation of T cell migration	1	0.46534	0.31	0.029	Biological	GO:20004	positive regulation of T cell migration	0.46534	0.61593	0	0	1	34
GO:0021954 central nervous system neuron development	2	0.46675	0.619	0.023	Biological	GO:00219	central nervous system neuron development	0.46675	0.6173	0	0	2	86
GO:0035050 embryonic heart tube development	2	0.46675	0.619	0.023	Biological	GO:00350	embryonic heart tube development	0.46675	0.6173	0	0	2	86
GO:0048041 focal adhesion assembly	2	0.46675	0.619	0.023	Biological	GO:00480	focal adhesion assembly	0.46675	0.6173	0	0	2	86
GO:0045334 clathrin-coated endocytic vesicle	2	0.46685	0.593	0.023	Cellular C	GO:00453	clathrin-coated endocytic vesicle	0.46685	0.66782	0	0	2	87
GO:0051640 organelle localization	11	0.46698	3.406	0.019	Biological	GO:00516	organelle localization	0.46698	0.61744	0	0	11	570
GO:0001701 in utero embryonic development	8	0.46756	2.477	0.02	Biological	GO:00017	in utero embryonic development	0.46756	0.61804	0	0	8	408
GO:0006366 transcription by RNA polymerase II	50	0.46772	15.48	0.019	Biological	GO:00063	transcription by RNA polymerase II	0.46772	0.6181	0	0	50	2693
GO:0016810 hydrolase activity, acting on carbon-nitrogen (but not pepti	3	0.46799	0.877	0.022	Molecular	GO:00168	hydrolase activity, acting on carbon-nitrogen (but not peptide) bonds	0.46799	0.63524	0	0	3	137
GO:0071855 neuropeptide receptor binding	1	0.47095	0.292	0.029	Molecular	GO:00718	neuropeptide receptor binding	0.47095	0.63625	0	0	1	34
GO:0071889 14-3-3 protein binding	1	0.47095	0.292	0.029	Molecular	GO:00718	14-3-3 protein binding	0.47095	0.63625	0	0	1	34
GO:0090482 vitamin transmembrane transporter activity	1	0.47095	0.292	0.029	Molecular	GO:00904	vitamin transmembrane transporter activity	0.47095	0.63625	0	0	1	34
GO:1990752 microtubule end	1	0.47124	0.297	0.029	Cellular C	GO:19907	microtubule end	0.47124	0.6723	0	0	1	35
GO:0007269 neurotransmitter secretion	3	0.47134	0.929	0.021	Biological	GO:00072	neurotransmitter secretion	0.47134	0.62255	0	0	3	140
GO:0099643 signal release from synapse	3	0.47134	0.929	0.021	Biological	GO:00996	signal release from synapse	0.47134	0.62255	0	0	3	140
GO:0032370 positive regulation of lipid transport	2	0.47275	0.619	0.023	Biological	GO:00323	positive regulation of lipid transport	0.47275	0.62392	0	0	2	87
GO:0042632 cholesterol homeostasis	2	0.47275	0.619	0.023	Biological	GO:00426	cholesterol homeostasis	0.47275	0.62392	0	0	2	87
GO:0070301 cellular response to hydrogen peroxide	2	0.47275	0.619	0.023	Biological	GO:00703	cellular response to hydrogen peroxide	0.47275	0.62392	0	0	2	87
GO:0016798 hydrolase activity, acting on glycosyl bonds	3	0.47276	0.877	0.022	Molecular	GO:00167	hydrolase activity, acting on glycosyl bonds	0.47276	0.63756	0	0	3	138
GO:0008076 voltage-gated potassium channel complex	2	0.47278	0.593	0.023	Cellular C	GO:00080	voltage-gated potassium channel complex	0.47278	0.6727	0	0	2	88
GO:0070302 regulation of stress-activated protein kinase signaling casc	4	0.47293	1.238	0.021	Biological	GO:00703	regulation of stress-activated protein kinase signaling cascade	0.47293	0.62399	0	0	4	194
GO:0031625 ubiquitin protein ligase binding	6	0.47339	1.754	0.02	Molecular	GO:00316	ubiquitin protein ligase binding	0.47339	0.63756	0	0	6	297
GO:0002263 cell activation involved in immune response	6	0.47354	1.858	0.02	Biological	GO:00022	cell activation involved in immune response	0.47354	0.62406	0	0	6	302
GO:0009954 proximal/distal pattern formation	1	0.47511	0.31	0.029	Biological	GO:00099	proximal/distal pattern formation	0.47511	0.62406	0	0	1	35
GO:0010719 negative regulation of epithelial to mesenchymal transition	1	0.47511	0.31	0.029	Biological	GO:00107	negative regulation of epithelial to mesenchymal transition	0.47511	0.62406	0	0	1	35
GO:0032743 positive regulation of interleukin-2 production	1	0.47511	0.31	0.029	Biological	GO:00327	positive regulation of interleukin-2 production	0.47511	0.62406	0	0	1	35
GO:0032885 regulation of polysaccharide biosynthetic process	1	0.47511	0.31	0.029	Biological	GO:00328	regulation of polysaccharide biosynthetic process	0.47511	0.62406	0	0	1	35
GO:0045879 negative regulation of smoothened signaling pathway	1	0.47511	0.31	0.029	Biological	GO:00458	negative regulation of smoothened signaling pathway	0.47511	0.62406	0	0	1	35
GO:0046627 negative regulation of insulin receptor signaling pathway	1	0.47511	0.31	0.029	Biological	GO:00466	negative regulation of insulin receptor signaling pathway	0.47511	0.62406	0	0	1	35
GO:0051968 positive regulation of synaptic transmission, glutamatergic	1	0.47511	0.31	0.029	Biological	GO:00519	positive regulation of synaptic transmission, glutamatergic	0.47511	0.62406	0	0	1	35
GO:0060351 cartilage development involved in endochondral bone morp	1	0.47511	0.31	0.029	Biological	GO:00603	cartilage development involved in endochondral bone morphogenesis	0.47511	0.62406	0	0	1	35
GO:0090075 relaxation of muscle	1	0.47511	0.31	0.029	Biological	GO:00900	relaxation of muscle	0.47511	0.62406	0	0	1	35
GO:0090181 regulation of cholesterol metabolic process	1	0.47511	0.31	0.029	Biological	GO:00901	regulation of cholesterol metabolic process	0.47511	0.62406	0	0	1	35
GO:0090313 regulation of protein targeting to membrane	1	0.47511	0.31	0.029	Biological	GO:00903	regulation of protein targeting to membrane	0.47511	0.62406	0	0	1	35
GO:0097484 dendrite extension	1	0.47511	0.31	0.029	Biological	GO:00974	dendrite extension	0.47511	0.62406	0	0	1	35
GO:0097502 mannosylation	1	0.47511	0.31	0.029	Biological	GO:00975	mannosylation	0.47511	0.62406	0	0	1	35
GO:0099174 regulation of presynapse organization	1	0.47511	0.31	0.029	Biological	GO:00991	regulation of presynapse organization	0.47511	0.62406	0	0	1	35
GO:0140632 inflammasome complex assembly	1	0.47511	0.31	0.029	Biological	GO:01406	inflammasome complex assembly	0.47511	0.62406	0	0	1	35
GO:1905606 regulation of presynapse assembly	1	0.47511	0.31	0.029	Biological	GO:19056	regulation of presynapse assembly	0.47511	0.62406	0	0	1	35
GO:0003727 single-stranded RNA binding	2	0.47536	0.585	0.023	Molecular	GO:00037	single-stranded RNA binding	0.47536	0.63823	0	0	2	86
GO:0051117 ATPase binding	2	0.47536	0.585	0.023	Molecular	GO:00511	ATPase binding	0.47536	0.63823	0	0	2	86
GO:0009607 response to biotic stimulus	29	0.476	8.978	0.019	Biological	GO:00096	response to biotic stimulus	0.476	0.62459	0	0	29	1554
GO:0055037 recycling endosome	4	0.47601	1.187	0.02	Cellular C	GO:00550	recycling endosome	0.47601	0.6755	0	0	4	197
GO:0046328 regulation of JNK cascade	3	0.47601	0.929	0.021	Biological	GO:00463	regulation of JNK cascade	0.47601	0.62459	0	0	3	141
GO:0062013 positive regulation of small molecule metabolic process	3	0.47601	0.929	0.021	Biological	GO:00620	positive regulation of small molecule metabolic process	0.47601	0.62459	0	0	3	141
GO:1903008 organelle disassembly	3	0.47601	0.929	0.021	Biological	GO:19030	organelle disassembly	0.47601	0.62459	0	0	3	141
GO:0071902 positive regulation of protein serine/threonine kinase activit	4	0.47689	1.238	0.021	Biological	GO:00719	positive regulation of protein serine/threonine kinase activity	0.47689	0.62558	0	0	4	195
GO:0005876 spindle microtubule	2	0.47867	0.593	0.022	Cellular C	GO:00058	spindle microtubule	0.47867	0.67748	0	0	2	89
GO:0055072 iron ion homeostasis	2	0.47871	0.619	0.023	Biological	GO:00550	iron ion homeostasis	0.47871	0.62764	0	0	2	88
GO:0055092 sterol homeostasis	2	0.47871	0.619	0.023	Biological	GO:00550	sterol homeostasis	0.47871	0.62764	0	0	2	88
GO:0030177 positive regulation of Wnt signaling pathway	3	0.48067	0.929	0.021	Biological	GO:00301	positive regulation of Wnt signaling pathway	0.48067	0.63004	0	0	3	142
GO:0036002 pre-mRNA binding	1	0.48077	0.292	0.029	Molecular	GO:00360	pre-mRNA binding	0.48077	0.6435	0	0	1	35
GO:0043531 ADP binding	1	0.48077	0.292	0.029	Molecular	GO:00435	ADP binding	0.48077	0.6435	0	0	1	35
GO:0016235 aggresome	1	0.48079	0.297	0.028	Cellular C	GO:00162	aggresome	0.48079	0.67869	0	0	1	36
GO:0016311 dephosphorylation	7	0.48212	2.167	0.019	Biological	GO:00163	dephosphorylation	0.48212	0.63178	0	0	7	359
GO:0016485 protein processing	5	0.48396	1.548	0.02	Biological	GO:00164	protein processing	0.48396	0.63194	0	0	5	251
GO:0031091 platelet alpha granule	2	0.48452	0.593	0.022	Cellular C	GO:00310	platelet alpha granule	0.48452	0.68036	0	0	2	90
GO:0035579 specific granule membrane	2	0.48452	0.593	0.022	Cellular C	GO:00355	specific granule membrane	0.48452	0.68036	0	0	2	90
GO:0032092 positive regulation of protein binding	2	0.48462	0.619	0.022	Biological	GO:00320	positive regulation of protein binding	0.48462	0.63194	0	0	2	89
GO:0072384 organelle transport along microtubule	2	0.48462	0.619	0.022	Biological	GO:00723	organelle transport along microtubule	0.48462	0.63194	0	0	2	89
GO:0002068 glandular epithelial cell development	1	0.4847	0.31	0.028	Biological	GO:00020	glandular epithelial cell development	0.4847	0.63194	0	0	1	36
GO:0009119 ribonucleoside metabolic process	1	0.4847	0.31	0.028	Biological	GO:00091	ribonucleoside metabolic process	0.4847	0.63194	0	0	1	36
GO:0031112 positive regulation of microtubule polymerization or depolyr	1	0.4847	0.31	0.028	Biological	GO:00311	positive regulation of microtubule polymerization or depolymerization	0.4847	0.63194	0	0	1	36
GO:0032228 regulation of synaptic transmission, GABAergic	1	0.4847	0.31	0.028	Biological	GO:00322	regulation of synaptic transmission, GABAergic	0.4847	0.63194	0	0	1	36
GO:0033146 regulation of intracellular estrogen receptor signaling pathw	1	0.4847	0.31	0.028	Biological	GO:00331	regulation of intracellular estrogen receptor signaling pathway	0.4847	0.63194	0	0	1	36
GO:0035520 monoubiquitinated protein deubiquitination	1	0.4847	0.31	0.028	Biological	GO:00355	monoubiquitinated protein deubiquitination	0.4847	0.63194	0	0	1	36
GO:0044060 regulation of endocrine process	1	0.4847	0.31	0.028	Biological	GO:00440	regulation of endocrine process	0.4847	0.63194	0	0	1	36
GO:0044319 wound healing, spreading of cells	1	0.4847	0.31	0.028	Biological	GO:00443	wound healing, spreading of cells	0.4847	0.63194	0	0	1	36
GO:0045922 negative regulation of fatty acid metabolic process	1	0.4847	0.31	0.028	Biological	GO:00459	negative regulation of fatty acid metabolic process	0.4847	0.63194	0	0	1	36
GO:0060999 positive regulation of dendritic spine development	1	0.4847	0.31	0.028	Biological	GO:00609	positive regulation of dendritic spine development	0.4847	0.63194	0	0	1	36
GO:0071312 cellular response to alkaloid	1	0.4847	0.31	0.028	Biological	GO:00713	cellular response to alkaloid	0.4847	0.63194	0	0	1	36
GO:0090505 epiboly involved in wound healing	1	0.4847	0.31	0.028	Biological	GO:00905	epiboly involved in wound healing	0.4847	0.63194	0	0	1	36
GO:1900181 negative regulation of protein localization to nucleus	1	0.4847	0.31	0.028	Biological	GO:19001	negative regulation of protein localization to nucleus	0.4847	0.63194	0	0	1	36
GO:1902003 regulation of amyloid-beta formation	1	0.4847	0.31	0.028	Biological	GO:19020	regulation of amyloid-beta formation	0.4847	0.63194	0	0	1	36
GO:1905476 negative regulation of protein localization to membrane	1	0.4847	0.31	0.028	Biological	GO:19054	negative regulation of protein localization to membrane	0.4847	0.63194	0	0	1	36
GO:2000515 negative regulation of CD4-positive, alpha-beta T cell activ	1	0.4847	0.31	0.028	Biological	GO:20005	negative regulation of CD4-positive, alpha-beta T cell activation	0.4847	0.63194	0	0	1	36
GO:0045321 leukocyte activation	19	0.48478	5.882	0.019	Biological	GO:00453	leukocyte activation	0.48478	0.63194	0	0	19	1014
GO:0032446 protein modification by small protein conjugation	17	0.48504	5.263	0.019	Biological	GO:00324	protein modification by small protein conjugation	0.48504	0.63212	0	0	17	905
GO:2000377 regulation of reactive oxygen species metabolic process	3	0.4853	0.929	0.021	Biological	GO:20003	regulation of reactive oxygen species metabolic process	0.4853	0.6323	0	0	3	143
GO:0016922 nuclear receptor binding	3	0.48694	0.877	0.021	Molecular	GO:00169	nuclear receptor binding	0.48694	0.65075	0	0	3	141

GO:0045111 intermediate filament cytoskeleton	5	0.48787	1.484	0.02	Cellular C	GO:00451	intermediate filament cytoskeleton	0.48787	0.68217	0	0	5	255
GO:0032388 positive regulation of intracellular transport	4	0.48871	1.238	0.02	Biological	GO:00323	positive regulation of intracellular transport	0.48871	0.63657	0	0	4	198
GO:0001508 action potential	3	0.48992	0.929	0.021	Biological	GO:00015	action potential	0.48992	0.63781	0	0	3	144
GO:0071322 cellular response to carbohydrate stimulus	3	0.48992	0.929	0.021	Biological	GO:00713	cellular response to carbohydrate stimulus	0.48992	0.63781	0	0	3	144
GO:0048787 presynaptic active zone membrane	1	0.49017	0.297	0.027	Cellular C	GO:00487	presynaptic active zone membrane	0.49017	0.68217	0	0	1	37
GO:0030134 COPII-coated ER to Golgi transport vesicle	2	0.49033	0.593	0.022	Cellular C	GO:00301	COPII-coated ER to Golgi transport vesicle	0.49033	0.68217	0	0	2	91
GO:0016814 hydrolase activity, acting on carbon-nitrogen (but not pepti	1	0.49041	0.292	0.028	Molecular	GO:00168	hydrolase activity, acting on carbon-nitrogen (but not peptide) bonds,	0.49041	0.65238	0	0	1	36
GO:0030291 protein serine/threonine kinase inhibitor activity	1	0.49041	0.292	0.028	Molecular	GO:00302	protein serine/threonine kinase inhibitor activity	0.49041	0.65238	0	0	1	36
GO:0043394 proteoglycan binding	1	0.49041	0.292	0.028	Molecular	GO:00433	proteoglycan binding	0.49041	0.65238	0	0	1	36
GO:0033273 response to vitamin	2	0.4905	0.619	0.022	Biological	GO:00332	response to vitamin	0.4905	0.63791	0	0	2	90
GO:0050886 endocrine process	2	0.4905	0.619	0.022	Biological	GO:00508	endocrine process	0.4905	0.63791	0	0	2	90
GO:0071277 cellular response to calcium ion	2	0.4905	0.619	0.022	Biological	GO:00712	cellular response to calcium ion	0.4905	0.63791	0	0	2	90
GO:2000117 negative regulation of cysteine-type endopeptidase activity	2	0.4905	0.619	0.022	Biological	GO:20001	negative regulation of cysteine-type endopeptidase activity	0.4905	0.63791	0	0	2	90
GO:0034702 ion channel complex	6	0.49091	1.78	0.019	Cellular C	GO:00347	ion channel complex	0.49091	0.68217	0	0	6	311
GO:0070925 organelle assembly	18	0.49124	5.573	0.019	Biological	GO:00709	organelle assembly	0.49124	0.6387	0	0	18	963
GO:0003341 cilium movement	4	0.49263	1.238	0.02	Biological	GO:00033	cilium movement	0.49263	0.63963	0	0	4	199
GO:0017157 regulation of exocytosis	4	0.49263	1.238	0.02	Biological	GO:00171	regulation of exocytosis	0.49263	0.63963	0	0	4	199
GO:0003714 transcription corepressor activity	4	0.49368	1.17	0.02	Molecular	GO:00037	transcription corepressor activity	0.49368	0.65406	0	0	4	196
GO:0008514 organic anion transmembrane transporter activity	4	0.49368	1.17	0.02	Molecular	GO:00085	organic anion transmembrane transporter activity	0.49368	0.65406	0	0	4	196
GO:0030135 coated vesicle	6	0.49402	1.78	0.019	Cellular C	GO:00301	coated vesicle	0.49402	0.68471	0	0	6	312
GO:0003230 cardiac atrium development	1	0.49411	0.31	0.027	Biological	GO:00032	cardiac atrium development	0.49411	0.63963	0	0	1	37
GO:0009595 detection of biotic stimulus	1	0.49411	0.31	0.027	Biological	GO:00095	detection of biotic stimulus	0.49411	0.63963	0	0	1	37
GO:0016486 peptide hormone processing	1	0.49411	0.31	0.027	Biological	GO:00164	peptide hormone processing	0.49411	0.63963	0	0	1	37
GO:0019068 virion assembly	1	0.49411	0.31	0.027	Biological	GO:00190	virion assembly	0.49411	0.63963	0	0	1	37
GO:0021696 cerebellar cortex morphogenesis	1	0.49411	0.31	0.027	Biological	GO:00216	cerebellar cortex morphogenesis	0.49411	0.63963	0	0	1	37
GO:0032965 regulation of collagen biosynthetic process	1	0.49411	0.31	0.027	Biological	GO:00329	regulation of collagen biosynthetic process	0.49411	0.63963	0	0	1	37
GO:0033280 response to vitamin D	1	0.49411	0.31	0.027	Biological	GO:00332	response to vitamin D	0.49411	0.63963	0	0	1	37
GO:0045191 regulation of isotype switching	1	0.49411	0.31	0.027	Biological	GO:00451	regulation of isotype switching	0.49411	0.63963	0	0	1	37
GO:0045652 regulation of megakaryocyte differentiation	1	0.49411	0.31	0.027	Biological	GO:00456	regulation of megakaryocyte differentiation	0.49411	0.63963	0	0	1	37
GO:0046320 regulation of fatty acid oxidation	1	0.49411	0.31	0.027	Biological	GO:00463	regulation of fatty acid oxidation	0.49411	0.63963	0	0	1	37
GO:0060122 inner ear receptor cell stereocilium organization	1	0.49411	0.31	0.027	Biological	GO:00601	inner ear receptor cell stereocilium organization	0.49411	0.63963	0	0	1	37
GO:0070741 response to interleukin-6	1	0.49411	0.31	0.027	Biological	GO:00707	response to interleukin-6	0.49411	0.63963	0	0	1	37
GO:0090504 epiboly	1	0.49411	0.31	0.027	Biological	GO:00905	epiboly	0.49411	0.63963	0	0	1	37
GO:1900077 negative regulation of cellular response to insulin stimulus	1	0.49411	0.31	0.027	Biological	GO:19000	negative regulation of cellular response to insulin stimulus	0.49411	0.63963	0	0	1	37
GO:0043543 protein acylation	5	0.49438	1.548	0.02	Biological	GO:00435	protein acylation	0.49438	0.63963	0	0	5	254
GO:0010324 membrane invagination	3	0.49451	0.929	0.021	Biological	GO:00103	membrane invagination	0.49451	0.63963	0	0	3	145
GO:0034341 response to interferon-gamma	3	0.49451	0.929	0.021	Biological	GO:00343	response to interferon-gamma	0.49451	0.63963	0	0	3	145
GO:0050671 positive regulation of lymphocyte proliferation	3	0.49451	0.929	0.021	Biological	GO:00506	positive regulation of lymphocyte proliferation	0.49451	0.63963	0	0	3	145
GO:0019955 cytokine binding	3	0.49628	0.877	0.021	Molecular	GO:00199	cytokine binding	0.49628	0.65406	0	0	3	143
GO:0140375 immune receptor activity	3	0.49628	0.877	0.021	Molecular	GO:01403	immune receptor activity	0.49628	0.65406	0	0	3	143
GO:0046883 regulation of hormone secretion	5	0.49783	1.548	0.02	Biological	GO:00468	regulation of hormone secretion	0.49783	0.64375	0	0	5	255
GO:0001889 liver development	3	0.49909	0.929	0.021	Biological	GO:00018	liver development	0.49909	0.64521	0	0	3	146
GO:0000217 DNA secondary structure binding	1	0.49987	0.292	0.027	Molecular	GO:00002	DNA secondary structure binding	0.49987	0.65406	0	0	1	37
GO:0008200 ion channel inhibitor activity	1	0.49987	0.292	0.027	Molecular	GO:00082	ion channel inhibitor activity	0.49987	0.65406	0	0	1	37
GO:0015248 sterol transporter activity	1	0.49987	0.292	0.027	Molecular	GO:00152	sterol transporter activity	0.49987	0.65406	0	0	1	37
GO:0016409 palmitoyltransferase activity	1	0.49987	0.292	0.027	Molecular	GO:00164	palmitoyltransferase activity	0.49987	0.65406	0	0	1	37
GO:0045505 dynein intermediate chain binding	1	0.49987	0.292	0.027	Molecular	GO:00455	dynein intermediate chain binding	0.49987	0.65406	0	0	1	37
GO:0038023 signaling receptor activity	28	0.49999	8.187	0.019	Molecular	GO:00380	signaling receptor activity	0.49999	0.65406	0	0	28	1491
GO:0060089 molecular transducer activity	28	0.49999	8.187	0.019	Molecular	GO:00600	molecular transducer activity	0.49999	0.65406	0	0	28	1491
GO:0035304 regulation of protein dephosphorylation	2	0.50213	0.619	0.022	Biological	GO:00353	regulation of protein dephosphorylation	0.50213	0.64758	0	0	2	92
GO:0045666 positive regulation of neuron differentiation	2	0.50213	0.619	0.022	Biological	GO:00456	positive regulation of neuron differentiation	0.50213	0.64758	0	0	2	92
GO:0006270 DNA replication initiation	1	0.50335	0.31	0.026	Biological	GO:00062	DNA replication initiation	0.50335	0.64758	0	0	1	38
GO:0010923 negative regulation of phosphatase activity	1	0.50335	0.31	0.026	Biological	GO:00109	negative regulation of phosphatase activity	0.50335	0.64758	0	0	1	38
GO:0019432 triglyceride biosynthetic process	1	0.50335	0.31	0.026	Biological	GO:00194	triglyceride biosynthetic process	0.50335	0.64758	0	0	1	38
GO:0030279 negative regulation of ossification	1	0.50335	0.31	0.026	Biological	GO:00302	negative regulation of ossification	0.50335	0.64758	0	0	1	38
GO:0035883 enteroendocrine cell differentiation	1	0.50335	0.31	0.026	Biological	GO:00358	enteroendocrine cell differentiation	0.50335	0.64758	0	0	1	38
GO:0042401 cellular biogenic amine biosynthetic process	1	0.50335	0.31	0.026	Biological	GO:00424	cellular biogenic amine biosynthetic process	0.50335	0.64758	0	0	1	38
GO:0043368 positive T cell selection	1	0.50335	0.31	0.026	Biological	GO:00433	positive T cell selection	0.50335	0.64758	0	0	1	38
GO:0044743 protein transmembrane import into intracellular organelle	1	0.50335	0.31	0.026	Biological	GO:00447	protein transmembrane import into intracellular organelle	0.50335	0.64758	0	0	1	38
GO:0045616 regulation of keratinocyte differentiation	1	0.50335	0.31	0.026	Biological	GO:00456	regulation of keratinocyte differentiation	0.50335	0.64758	0	0	1	38
GO:0045746 negative regulation of Notch signaling pathway	1	0.50335	0.31	0.026	Biological	GO:00457	negative regulation of Notch signaling pathway	0.50335	0.64758	0	0	1	38
GO:0045773 positive regulation of axon extension	1	0.50335	0.31	0.026	Biological	GO:00457	positive regulation of axon extension	0.50335	0.64758	0	0	1	38
GO:0048846 axon extension involved in axon guidance	1	0.50335	0.31	0.026	Biological	GO:00488	axon extension involved in axon guidance	0.50335	0.64758	0	0	1	38
GO:0050654 chondroitin sulfate proteoglycan metabolic process	1	0.50335	0.31	0.026	Biological	GO:00506	chondroitin sulfate proteoglycan metabolic process	0.50335	0.64758	0	0	1	38
GO:0060043 regulation of cardiac muscle cell proliferation	1	0.50335	0.31	0.026	Biological	GO:00600	regulation of cardiac muscle cell proliferation	0.50335	0.64758	0	0	1	38
GO:0070306 lens fiber cell differentiation	1	0.50335	0.31	0.026	Biological	GO:00703	lens fiber cell differentiation	0.50335	0.64758	0	0	1	38
GO:1902284 neuron projection extension involved in neuron projection g	1	0.50335	0.31	0.026	Biological	GO:19022	neuron projection extension involved in neuron projection guidance	0.50335	0.64758	0	0	1	38
GO:0060078 regulation of postsynaptic membrane potential	3	0.50364	0.929	0.02	Biological	GO:00600	regulation of postsynaptic membrane potential	0.50364	0.64758	0	0	3	147
GO:0120161 regulation of cold-induced thermogenesis	3	0.50364	0.929	0.02	Biological	GO:01201	regulation of cold-induced thermogenesis	0.50364	0.64758	0	0	3	147
GO:1903900 regulation of viral life cycle	3	0.50364	0.929	0.02	Biological	GO:19039	regulation of viral life cycle	0.50364	0.64758	0	0	3	147
GO:0071396 cellular response to lipid	11	0.5039	3.406	0.019	Biological	GO:00713	cellular response to lipid	0.5039	0.64774	0	0	11	586
GO:0043393 regulation of protein binding	4	0.5043	1.238	0.02	Biological	GO:00433	regulation of protein binding	0.5043	0.64809	0	0	4	202
GO:0051704 multi-organism process	21	0.5046	6.502	0.019	Biological	GO:00517	multi-organism process	0.5046	0.6483	0	0	21	1135
GO:0048511 rhythmic process	6	0.50503	1.858	0.019	Biological	GO:00485	rhythmic process	0.50503	0.6487	0	0	6	312
GO:0008276 protein methyltransferase activity	2	0.50512	0.585	0.022	Molecular	GO:00082	protein methyltransferase activity	0.50512	0.65901	0	0	2	91
GO:0140657 ATP-dependent activity	11	0.50529	3.216	0.019	Molecular	GO:01406	ATP-dependent activity	0.50529	0.65901	0	0	11	577
GO:0030659 cytoplasmic vesicle membrane	22	0.50551	6.528	0.018	Cellular C	GO:00306	cytoplasmic vesicle membrane	0.50551	0.69883	0	0	22	1204
GO:0045995 regulation of embryonic development	2	0.50787	0.619	0.022	Biological	GO:00459	regulation of embryonic development	0.50787	0.65156	0	0	2	93
GO:0050829 defense response to Gram-negative bacterium	2	0.50787	0.619	0.022	Biological	GO:00508	defense response to Gram-negative bacterium	0.50787	0.65156	0	0	2	93
GO:0051781 positive regulation of cell division	2	0.50787	0.619	0.022	Biological	GO:00517	positive regulation of cell division	0.50787	0.65156	0	0	2	93
GO:0002791 regulation of peptide secretion	4	0.50817	1.238	0.02	Biological	GO:00027	regulation of peptide secretion	0.50817	0.65156	0	0	4	203
GO:0099504 synaptic vesicle cycle	4	0.50817	1.238	0.02	Biological	GO:00995	synaptic vesicle cycle	0.50817	0.65156	0	0	4	203
GO:0006997 nucleus organization	3	0.50818	0.929	0.02	Biological	GO:00069	nucleus organization	0.50818	0.65156	0	0	3	148
GO:0106106 cold-induced thermogenesis	3	0.50818	0.929	0.02	Biological	GO:01061	cold-induced thermogenesis	0.50818	0.65156	0	0	3	148
GO:0070461 SAGA-type complex	1	0.50842	0.297	0.026	Cellular C	GO:00704	SAGA-type complex	0.50842	0.70104	0	0	1	39
GO:0004623 phospholipase A2 activity	1	0.50916	0.292	0.026	Molecular	GO:00046	phospholipase A2 activity	0.50916	0.66033	0	0	1	38
GO:0016248 channel inhibitor activity	1	0.50916	0.292	0.026	Molecular	GO:00162	channel inhibitor activity	0.50916	0.66033	0	0	1	38
GO:0042887 amide transmembrane transporter activity	1	0.50916	0.292	0.026	Molecular	GO:00428	amide transmembrane transporter activity	0.50916	0.66033	0	0	1	38
GO:0016301 kinase activity	15	0.50935	4.386	0.019	Molecular	GO:00163	kinase activity	0.50935	0.66033	0	0	15	795
GO:0005768 endosome	19	0.50982	5.638	0.018	Cellular C	GO:00057	endosome	0.50982	0.70117	0	0	19	1040
GO:0005253 anion channel activity	2	0.51094	0.585	0.022	Molecular	GO:00052	anion channel activity	0.51094	0.66041	0	0	2	92
GO:0072341 modified amino acid binding	2	0.51094	0.585	0.022	Molecular	GO:00723	modified amino acid binding	0.51094	0.66041	0	0	2	92
GO:0022857 transmembrane transporter activity	21	0.51236	6.14	0.019	Molecular	GO:00228	transmembrane transporter activity	0.51236	0.66102	0	0	21	1121
GO:0001990 regulation of systemic arterial blood pressure by hormone	1	0.51242	0.31	0.026	Biological	GO:00019	regulation of systemic arterial blood pressure by hormone	0.51242	0.65415	0	0	1	39
GO:0002478 antigen processing and presentation of exogenous peptide	1	0.51242	0.31	0.026	Biological	GO:00024	antigen processing and presentation of exogenous peptide antigen	0.51242	0.65415	0	0	1	39
GO:0007031 peroxisome organization	1	0.51242	0.31	0.026	Biological	GO:00070	peroxisome organization	0.51242	0.65415	0	0	1	39
GO:0007212 dopamine receptor signaling pathway	1	0.51242	0.31	0.026	Biological	GO:00072	dopamine receptor signaling pathway	0.51242	0.65415	0	0	1	39
GO:0009309 amine biosynthetic process	1	0.51242	0.31	0.026	Biological	GO:00093	amine biosynthetic process	0.51242	0.65415	0	0	1	39
GO:0010762 regulation of fibroblast migration	1	0.51242	0.31	0.026	Biological	GO:00107	regulation of fibroblast migration	0.51242	0.65415	0	0	1	39
GO:0030851 granulocyte differentiation	1	0.51242	0.31	0.026	Biological	GO:00308	granulocyte differentiation	0.51242	0.65415	0	0	1	39
GO:0032438 melanosome organization	1	0.51242	0.31	0.026	Biological	GO:00324	melanosome organization	0.51242	0.65415	0	0	1	39
GO:0033028 myeloid cell apoptotic process	1	0.51242	0.31	0.026	Biological	GO:00330	myeloid cell apoptotic process	0.51242	0.65415	0	0	1	39
GO:0035510 DNA dealkylation	1	0.51242	0.31	0.026	Biological	GO:00355	DNA dealkylation	0.51242	0.65415	0	0	1	39
GO:0045880 positive regulation of smoothened signaling pathway	1	0.51242	0.31	0.026	Biological	GO:00458	positive regulation of smoothened signaling pathway	0.51242	0.65415	0	0	1	39
GO:0048821 erythrocyte development	1	0.51242	0.31	0.026	Biological	GO:00488	erythrocyte development	0.51242	0.65415	0	0	1	39
GO:0048854 brain morphogenesis	1	0.51242	0.31	0.026	Biological	GO:00488	brain morphogenesis	0.51242	0.65415	0	0	1	39
GO:0060416 response to growth hormone	1	0.51242	0.31	0.026	Biological	GO:00604	response to growth hormone	0.51242	0.65415	0	0	1	39
GO:0070266 necroptotic process	1	0.51242	0.31	0.026	Biological	GO:00702	necroptotic process	0.51242	0.65415	0	0	1	39
GO:0071634 regulation of transforming growth factor beta production	1	0.51242	0.31	0.026	Biological	GO:00716	regulation of transforming growth factor beta production	0.51242	0.65415	0	0	1	39
GO:0072337 modified amino acid transport	1	0.51242	0.31	0.026	Biological	GO:00723	modified amino acid transport	0.51242	0.65415	0	0	1	39
GO:0061008 hepaticobiliary system development	3	0.51269	0.929	0.02	Biological	GO:00610	hepaticobiliary system development	0.51269	0.65432	0	0	3	149
GO:0009791 post-embryonic development	2	0.51358	0.619	0.021	Biological	GO:00097	post-embryonic development	0.51358	0.65512	0	0	2	94
GO:0051588 regulation of neurotransmitter transport	2	0.51358	0.619	0.021	Biological	GO:00515	regulation of neurotransmitter transport	0.51358	0.65512	0	0	2	94
GO:1901653 cellular response to peptide	7	0.51391	2.167	0.019	Biological	GO:19016	cellular response to peptide	0.51391	0.65521	0	0	7	370
GO:1904062 regulation of cation transmembrane transport	7	0.51391	2.167	0.019	Biological	GO:19040	regulation of cation transmembrane transport	0.51391	0.65521	0	0	7	370
GO:0042886 amide transport	6	0.51436	1.858	0.019	Biological	GO:00428	amide transport	0.51436	0.65561	0	0	6	315
GO:0098562 cytoplasmic side of membrane	4	0.51456	1.187	0.019	Cellular C	GO:00985	cytoplasmic side of membrane	0.51456	0.70246	0	0	4	207
GO:0098802 plasma membrane signaling receptor complex	4	0.51456	1.187	0.019	Cellular C	GO:00988	plasma membrane signaling receptor complex	0.51456	0.70246	0	0	4	207
GO:0004713 protein tyrosine kinase activity	3	0.51472	0.877	0.02	Molecular	GO:00047	protein tyrosine kinase activity	0.51472	0.66102	0	0	3	147
GO:0034976 response to endoplasmic reticulum stress	5	0.51497	1.548	0.019	Biological	GO:00349	response to endoplasmic reticulum stress	0.51497	0.65623	0	0	5	260
GO:0005215 transporter activity	23	0.5154	6.725	0.019	Molecular	GO:00052	transporter activity	0.5154	0.66102	0	0	23	1231
GO:0090087 regulation of peptide transport	4	0.51586	1.238	0.02	Biological	GO:00900	regulation of peptide transport	0.51586	0.65719	0	0	4	205
GO:0016746 acyltransferase activity	5	0.51588	1.462	0.02	Molecular	GO:00167	acyltransferase activity	0.51588	0.66102	0	0	5	256
GO:0016410 N-acyltransferase activity	2	0.51672	0.585	0.022	Molecular	GO:00164	N-acyltransferase activity	0.51672	0.66102	0	0	2	93
GO:0030176 integral component of endoplasmic reticulum membrane	1	0.5173	0.297	0.025	Cellular C	GO:00301	integral component of endoplasmic reticulum membrane	0.5173	0.70246	0	0	1	40
GO:0031227 intrinsic component of endoplasmic reticulum membrane	1	0.5173	0.297	0.025	Cellular C	GO:00312	intrinsic component of endoplasmic reticulum membrane	0.5173	0.70246	0	0	1	40
GO:0010629 negative regulation of gene expression	18	0.51819	5.573	0.018	Biological	GO:00106	negative regulation of gene expression	0.51819	0.65996	0	0	18	978
GO:0004520 endodeoxyribonuclease activity	1	0.51828	0.292	0.026	Molecular	GO:00045	endodeoxyribonuclease activity	0.51828	0.66102	0	0	1	39
GO:0015036 disulfide oxidoreductase activity	1	0.51828	0.292	0.026	Molecular	GO:00150	disulfide oxidoreductase activity	0.51828	0.66102	0	0	1	39
GO:0016799 hydrolase activity, hydrolyzing N-glycosyl compounds	1	0.51828	0.292	0.026	Molecular	GO:00167	hydrolase activity, hydrolyzing N-glycosyl compounds	0.51828	0.66102	0	0	1	39
GO:0019825 oxygen binding	1	0.51828	0.292	0.026	Molecular	GO:00198	oxygen binding	0.51828	0.66102	0	0	1	39
GO:0008654 phospholipid biosynthetic process	5	0.51837	1.548	0.019	Biological	GO:00086	phospholipid biosynthetic process	0.51837	0.65996	0	0	5	261
GO:0005795 Golgi stack	3	0.51844	0.89	0.02	Cellular C	GO:00057	Golgi stack	0.51844	0.70246	0	0	3	152
GO:0034774 secretory granule lumen	6	0.51864	1.78	0.019	Cellular C	GO:00347	secretory granule lumen	0.51864	0.70246	0	0	6	320
GO:0006637 acyl-CoA metabolic process	2	0.51924	0.619	0.021	Biological	GO:00066	acyl-CoA metabolic process	0.51924	0.65996	0	0	2	95
GO:0008593 regulation of Notch signaling pathway	2	0.51924	0.619	0.021	Biological	GO:00085	regulation of Notch signaling pathway	0.51924	0.65996	0	0	2	95
GO:0035383 thioester metabolic process	2	0.51924	0.619	0.021	Biological	GO:00353	thioester metabolic process	0.51924	0.65996	0	0	2	95
GO:1990830 cellular response to leukemia inhibitory factor	2	0.51924	0.619	0.021	Biological	GO:19908	cellular response to leukemia inhibitory factor	0.51924	0.65996	0	0	2	95
GO:0050863 regulation of T cell activation	7	0.51962	2.167	0.019	Biological	GO:00508	regulation of T cell activation	0.51962	0.65996	0	0	7	372
GO:0008152 metabolic process	208	0.52064	64.396	0.018	Biological	GO:00081	metabolic process	0.52064	0.65996	0	0	208	11404
GO:0031667 response to nutrient levels	9	0.52088	2.786	0.019	Biological	GO:00316	response to nutrient levels	0.52088	0.65996	0	0	9	483
GO:0000266 mitochondrial fission	1	0.52133	0.31	0.025	Biological	GO:00002	mitochondrial fission	0.52133	0.65996	0	0	1	40
GO:0002691 regulation of cellular extravasation	1	0.52133	0.31	0.025	Biological	GO:00026	regulation of cellular extravasation	0.52133	0.65996	0	0	1	40
GO:0010863 positive regulation of phospholipase C activity	1	0.52133	0.31	0.025	Biological	GO:00108	positive regulation of phospholipase C activity	0.52133	0.65996	0	0	1	40
GO:0034142 toll-like receptor 4 signaling pathway	1	0.52133	0.31	0.025	Biological	GO:00341	toll-like receptor 4 signaling pathway	0.52133	0.65996	0	0	1	40
GO:0035136 forelimb morphogenesis	1	0.52133	0.31	0.025	Biological	GO:00351	forelimb morphogenesis	0.52133	0.65996	0	0	1	40
GO:0042307 positive regulation of protein import into nucleus	1	0.52133	0.31	0.025	Biological	GO:00423	positive regulation of protein import into nucleus	0.52133	0.65996	0	0	1	40
GO:0043516 regulation of DNA damage response, signal transduction b	1	0.52133	0.31	0.025	Biological	GO:00435	regulation of DNA damage response, signal transduction by p53 clas	0.52133	0.65996	0	0	1	40
GO:0046688 response to copper ion	1	0.52133	0.31	0.025	Biological	GO:00466	response to copper ion	0.52133	0.65996	0	0	1	40
GO:0048489 synaptic vesicle transport	1	0.52133	0.31	0.025	Biological	GO:00484	synaptic vesicle transport	0.52133	0.65996	0	0	1	40
GO:0048753 pigment granule organization	1	0.52133	0.31	0.025	Biological	GO:00487	pigment granule organization	0.52133	0.65996	0	0	1	40
GO:0070286 axonemal dynein complex assembly	1	0.52133	0.31	0.025	Biological	GO:00702	axonemal dynein complex assembly	0.52133	0.65996	0	0	1	40

GO:0070296 sarcoplasmic reticulum calcium ion transport	1	0.52133	0.31	0.025	Biological	GO:00702	sarcoplasmic reticulum calcium ion transport	0.52133	0.65996	0	0	1	40
GO:0071392 cellular response to estradiol stimulus	1	0.52133	0.31	0.025	Biological	GO:00713	cellular response to estradiol stimulus	0.52133	0.65996	0	0	1	40
GO:0140448 signaling receptor ligand precursor processing	1	0.52133	0.31	0.025	Biological	GO:01404	signaling receptor ligand precursor processing	0.52133	0.65996	0	0	1	40
GO:1901532 regulation of hematopoietic progenitor cell differentiation	1	0.52133	0.31	0.025	Biological	GO:19015	regulation of hematopoietic progenitor cell differentiation	0.52133	0.65996	0	0	1	40
GO:2000279 negative regulation of DNA biosynthetic process	1	0.52133	0.31	0.025	Biological	GO:20002	negative regulation of DNA biosynthetic process	0.52133	0.65996	0	0	1	40
GO:0015318 inorganic molecular entity transmembrane transporter acti	14	0.52177	4.094	0.019	Molecular	GO:00153	inorganic molecular entity transmembrane transporter activity	0.52177	0.66449	0	0	14	747
GO:0032956 regulation of actin cytoskeleton organization	7	0.52247	2.167	0.019	Biological	GO:00329	regulation of actin cytoskeleton organization	0.52247	0.66122	0	0	7	373
GO:1990823 response to leukemia inhibitory factor	2	0.52486	0.619	0.021	Biological	GO:19908	response to leukemia inhibitory factor	0.52486	0.66408	0	0	2	96
GO:0005996 monosaccharide metabolic process	5	0.52514	1.548	0.019	Biological	GO:00059	monosaccharide metabolic process	0.52514	0.66427	0	0	5	263
GO:0032391 photoreceptor connecting cilium	1	0.52602	0.297	0.024	Cellular C	GO:00323	photoreceptor connecting cilium	0.52602	0.70886	0	0	1	41
GO:0032590 dendrite membrane	1	0.52602	0.297	0.024	Cellular C	GO:00325	dendrite membrane	0.52602	0.70886	0	0	1	41
GO:0001959 regulation of cytokine-mediated signaling pathway	3	0.52609	0.929	0.02	Biological	GO:00019	regulation of cytokine-mediated signaling pathway	0.52609	0.66514	0	0	3	152
GO:0010675 regulation of cellular carbohydrate metabolic process	3	0.52609	0.929	0.02	Biological	GO:00106	regulation of cellular carbohydrate metabolic process	0.52609	0.66514	0	0	3	152
GO:0005547 phosphatidylinositol-3,4,5-trisphosphate binding	1	0.52723	0.292	0.025	Molecular	GO:00055	phosphatidylinositol-3,4,5-trisphosphate binding	0.52723	0.66849	0	0	1	40
GO:0016620 oxidoreductase activity, acting on the aldehyde or oxo grou	1	0.52723	0.292	0.025	Molecular	GO:00166	oxidoreductase activity, acting on the aldehyde or oxo group of donor	0.52723	0.66849	0	0	1	40
GO:0019213 deacetylase activity	1	0.52723	0.292	0.025	Molecular	GO:00192	deacetylase activity	0.52723	0.66849	0	0	1	40
GO:0060205 cytoplasmic vesicle lumen	6	0.52775	1.78	0.019	Cellular C	GO:00602	cytoplasmic vesicle lumen	0.52775	0.70942	0	0	6	323
GO:0042391 regulation of membrane potential	8	0.52953	2.477	0.019	Biological	GO:00423	regulation of membrane potential	0.52953	0.66725	0	0	8	431
GO:0034705 potassium channel complex	2	0.52982	0.593	0.02	Cellular C	GO:00347	potassium channel complex	0.52982	0.71041	0	0	2	98
GO:0002714 positive regulation of B cell mediated immunity	1	0.53008	0.31	0.024	Biological	GO:00027	positive regulation of B cell mediated immunity	0.53008	0.66725	0	0	1	41
GO:0002891 positive regulation of immunoglobulin mediated immune re	1	0.53008	0.31	0.024	Biological	GO:00028	positive regulation of immunoglobulin mediated immune response	0.53008	0.66725	0	0	1	41
GO:0030866 cortical actin cytoskeleton organization	1	0.53008	0.31	0.024	Biological	GO:00308	cortical actin cytoskeleton organization	0.53008	0.66725	0	0	1	41
GO:0031063 regulation of histone deacetylation	1	0.53008	0.31	0.024	Biological	GO:00310	regulation of histone deacetylation	0.53008	0.66725	0	0	1	41
GO:0032509 endosome transport via multivesicular body sorting pathwa	1	0.53008	0.31	0.024	Biological	GO:00325	endosome transport via multivesicular body sorting pathway	0.53008	0.66725	0	0	1	41
GO:0032733 positive regulation of interleukin-10 production	1	0.53008	0.31	0.024	Biological	GO:00327	positive regulation of interleukin-10 production	0.53008	0.66725	0	0	1	41
GO:0042554 superoxide anion generation	1	0.53008	0.31	0.024	Biological	GO:00425	superoxide anion generation	0.53008	0.66725	0	0	1	41
GO:0043403 skeletal muscle tissue regeneration	1	0.53008	0.31	0.024	Biological	GO:00434	skeletal muscle tissue regeneration	0.53008	0.66725	0	0	1	41
GO:0045429 positive regulation of nitric oxide biosynthetic process	1	0.53008	0.31	0.024	Biological	GO:00454	positive regulation of nitric oxide biosynthetic process	0.53008	0.66725	0	0	1	41
GO:0045622 regulation of T-helper cell differentiation	1	0.53008	0.31	0.024	Biological	GO:00456	regulation of T-helper cell differentiation	0.53008	0.66725	0	0	1	41
GO:0060338 regulation of type I interferon-mediated signaling pathway	1	0.53008	0.31	0.024	Biological	GO:00603	regulation of type I interferon-mediated signaling pathway	0.53008	0.66725	0	0	1	41
GO:0060412 ventricular septum morphogenesis	1	0.53008	0.31	0.024	Biological	GO:00604	ventricular septum morphogenesis	0.53008	0.66725	0	0	1	41
GO:0086091 regulation of heart rate by cardiac conduction	1	0.53008	0.31	0.024	Biological	GO:00860	regulation of heart rate by cardiac conduction	0.53008	0.66725	0	0	1	41
GO:0140467 integrated stress response signaling	1	0.53008	0.31	0.024	Biological	GO:01404	integrated stress response signaling	0.53008	0.66725	0	0	1	41
GO:1904037 positive regulation of epithelial cell apoptotic process	1	0.53008	0.31	0.024	Biological	GO:19040	positive regulation of epithelial cell apoptotic process	0.53008	0.66725	0	0	1	41
GO:0006497 protein lipidation	2	0.53043	0.619	0.021	Biological	GO:00064	protein lipidation	0.53043	0.66725	0	0	2	97
GO:0016079 synaptic vesicle exocytosis	2	0.53043	0.619	0.021	Biological	GO:00160	synaptic vesicle exocytosis	0.53043	0.66725	0	0	2	97
GO:0016575 histone deacetylation	2	0.53043	0.619	0.021	Biological	GO:00165	histone deacetylation	0.53043	0.66725	0	0	2	97
GO:0043255 regulation of carbohydrate biosynthetic process	2	0.53043	0.619	0.021	Biological	GO:00432	regulation of carbohydrate biosynthetic process	0.53043	0.66725	0	0	2	97
GO:0015711 organic anion transport	7	0.53096	2.167	0.019	Biological	GO:00157	organic anion transport	0.53096	0.66775	0	0	7	376
GO:0032412 regulation of ion transmembrane transporter activity	5	0.53187	1.548	0.019	Biological	GO:00324	regulation of ion transmembrane transporter activity	0.53187	0.66872	0	0	5	265
GO:0008202 steroid metabolic process	6	0.53279	1.858	0.019	Biological	GO:00082	steroid metabolic process	0.53279	0.66971	0	0	6	321
GO:0001653 peptide receptor activity	3	0.5328	0.877	0.02	Molecular	GO:00016	peptide receptor activity	0.5328	0.67456	0	0	3	151
GO:0031983 vesicle lumen	6	0.53379	1.78	0.018	Cellular C	GO:00319	vesicle lumen	0.53379	0.71296	0	0	6	325
GO:0098686 hippocampal mossy fiber to CA3 synapse	1	0.53458	0.297	0.024	Cellular C	GO:00986	hippocampal mossy fiber to CA3 synapse	0.53458	0.71296	0	0	1	42
GO:0032869 cellular response to insulin stimulus	4	0.53483	1.238	0.019	Biological	GO:00328	cellular response to insulin stimulus	0.53483	0.67187	0	0	4	210
GO:0051216 cartilage development	4	0.53483	1.238	0.019	Biological	GO:00512	cartilage development	0.53483	0.67187	0	0	4	210
GO:2001236 regulation of extrinsic apoptotic signaling pathway	3	0.53491	0.929	0.019	Biological	GO:20012	regulation of extrinsic apoptotic signaling pathway	0.53491	0.67187	0	0	3	154
GO:0051924 regulation of calcium ion transport	5	0.53522	1.548	0.019	Biological	GO:00519	regulation of calcium ion transport	0.53522	0.672	0	0	5	266
GO:0140352 export from cell	16	0.53542	4.954	0.018	Biological	GO:01403	export from cell	0.53542	0.672	0	0	16	877
GO:0012506 vesicle membrane	22	0.53594	6.528	0.018	Cellular C	GO:00125	vesicle membrane	0.53594	0.71296	0	0	22	1223
GO:0043279 response to alkaloid	2	0.53596	0.619	0.02	Biological	GO:00432	response to alkaloid	0.53596	0.672	0	0	2	98
GO:0044264 cellular polysaccharide metabolic process	2	0.53596	0.619	0.02	Biological	GO:00442	cellular polysaccharide metabolic process	0.53596	0.672	0	0	2	98
GO:0045807 positive regulation of endocytosis	2	0.53596	0.619	0.02	Biological	GO:00458	positive regulation of endocytosis	0.53596	0.672	0	0	2	98
GO:0120162 positive regulation of cold-induced thermogenesis	2	0.53596	0.619	0.02	Biological	GO:01201	positive regulation of cold-induced thermogenesis	0.53596	0.672	0	0	2	98
GO:2001237 negative regulation of extrinsic apoptotic signaling pathway	2	0.53596	0.619	0.02	Biological	GO:20012	negative regulation of extrinsic apoptotic signaling pathway	0.53596	0.672	0	0	2	98
GO:0046915 transition metal ion transmembrane transporter activity	1	0.53601	0.292	0.024	Molecular	GO:00469	transition metal ion transmembrane transporter activity	0.53601	0.67665	0	0	1	41
GO:0048487 beta-tubulin binding	1	0.53601	0.292	0.024	Molecular	GO:00484	beta-tubulin binding	0.53601	0.67665	0	0	1	41
GO:0008021 synaptic vesicle	4	0.53705	1.187	0.019	Cellular C	GO:00080	synaptic vesicle	0.53705	0.71296	0	0	4	213
GO:0006040 amino sugar metabolic process	1	0.53867	0.31	0.024	Biological	GO:00060	amino sugar metabolic process	0.53867	0.6732	0	0	1	42
GO:0007099 centriole replication	1	0.53867	0.31	0.024	Biological	GO:00070	centriole replication	0.53867	0.6732	0	0	1	42
GO:0010712 regulation of collagen metabolic process	1	0.53867	0.31	0.024	Biological	GO:00107	regulation of collagen metabolic process	0.53867	0.6732	0	0	1	42
GO:0010830 regulation of myotube differentiation	1	0.53867	0.31	0.024	Biological	GO:00108	regulation of myotube differentiation	0.53867	0.6732	0	0	1	42
GO:0014014 negative regulation of gliogenesis	1	0.53867	0.31	0.024	Biological	GO:00140	negative regulation of gliogenesis	0.53867	0.6732	0	0	1	42
GO:0042119 neutrophil activation	1	0.53867	0.31	0.024	Biological	GO:00421	neutrophil activation	0.53867	0.6732	0	0	1	42
GO:0050892 intestinal absorption	1	0.53867	0.31	0.024	Biological	GO:00508	intestinal absorption	0.53867	0.6732	0	0	1	42
GO:0050913 sensory perception of bitter taste	1	0.53867	0.31	0.024	Biological	GO:00509	sensory perception of bitter taste	0.53867	0.6732	0	0	1	42
GO:0120178 steroid hormone biosynthetic process	1	0.53867	0.31	0.024	Biological	GO:01201	steroid hormone biosynthetic process	0.53867	0.6732	0	0	1	42
GO:1900274 regulation of phospholipase C activity	1	0.53867	0.31	0.024	Biological	GO:19002	regulation of phospholipase C activity	0.53867	0.6732	0	0	1	42
GO:1902991 regulation of amyloid precursor protein catabolic process	1	0.53867	0.31	0.024	Biological	GO:19029	regulation of amyloid precursor protein catabolic process	0.53867	0.6732	0	0	1	42
GO:2000008 regulation of protein localization to cell surface	1	0.53867	0.31	0.024	Biological	GO:20000	regulation of protein localization to cell surface	0.53867	0.6732	0	0	1	42
GO:2000516 positive regulation of CD4-positive, alpha-beta T cell activa	1	0.53867	0.31	0.024	Biological	GO:20005	positive regulation of CD4-positive, alpha-beta T cell activation	0.53867	0.6732	0	0	1	42
GO:0009914 hormone transport	6	0.53888	1.858	0.019	Biological	GO:00099	hormone transport	0.53888	0.67329	0	0	6	323
GO:0008170 N-methyltransferase activity	2	0.53937	0.585	0.021	Molecular	GO:00081	N-methyltransferase activity	0.53937	0.6799	0	0	2	97
GO:0043269 regulation of ion transport	13	0.53993	4.025	0.018	Biological	GO:00432	regulation of ion transport	0.53993	0.67444	0	0	13	713
GO:0071702 organic substance transport	45	0.54074	13.932	0.018	Biological	GO:00717	organic substance transport	0.54074	0.67528	0	0	45	2482
GO:0051341 regulation of oxidoreductase activity	2	0.54144	0.619	0.02	Biological	GO:00513	regulation of oxidoreductase activity	0.54144	0.67599	0	0	2	99
GO:0098656 anion transmembrane transport	5	0.54188	1.548	0.019	Biological	GO:00986	anion transmembrane transport	0.54188	0.67637	0	0	5	268
GO:0006473 protein acetylation	4	0.54232	1.238	0.019	Biological	GO:00064	protein acetylation	0.54232	0.67641	0	0	4	212
GO:0016051 carbohydrate biosynthetic process	4	0.54232	1.238	0.019	Biological	GO:00160	carbohydrate biosynthetic process	0.54232	0.67641	0	0	4	212
GO:0050821 protein stabilization	4	0.54232	1.238	0.019	Biological	GO:00508	protein stabilization	0.54232	0.67641	0	0	4	212
GO:0003824 catalytic activity	106	0.54323	30.994	0.018	Molecular	GO:00038	catalytic activity	0.54323	0.68291	0	0	106	5745
GO:0016877 ligase activity, forming carbon-sulfur bonds	1	0.54463	0.292	0.024	Molecular	GO:00168	ligase activity, forming carbon-sulfur bonds	0.54463	0.68291	0	0	1	42
GO:0030331 estrogen receptor binding	1	0.54463	0.292	0.024	Molecular	GO:00303	estrogen receptor binding	0.54463	0.68291	0	0	1	42
GO:0071705 nitrogen compound transport	37	0.54478	11.455	0.018	Biological	GO:00717	nitrogen compound transport	0.54478	0.67847	0	0	37	2044
GO:0004553 hydrolase activity, hydrolyzing O-glycosyl compounds	2	0.54491	0.585	0.02	Molecular	GO:00045	hydrolase activity, hydrolyzing O-glycosyl compounds	0.54491	0.68291	0	0	2	98
GO:0018205 peptidyl-lysine modification	7	0.545	2.167	0.018	Biological	GO:00182	peptidyl-lysine modification	0.545	0.67847	0	0	7	381
GO:0031330 negative regulation of cellular catabolic process	5	0.54519	1.548	0.019	Biological	GO:00313	negative regulation of cellular catabolic process	0.54519	0.67847	0	0	5	269
GO:0005887 integral component of plasma membrane	8	0.54529	2.374	0.018	Cellular C	GO:00058	integral component of plasma membrane	0.54529	0.7221	0	0	8	442
GO:0042098 T cell proliferation	4	0.54604	1.238	0.019	Biological	GO:00420	T cell proliferation	0.54604	0.67847	0	0	4	213
GO:0050865 regulation of cell activation	13	0.54611	4.025	0.018	Biological	GO:00508	regulation of cell activation	0.54611	0.67847	0	0	13	716
GO:0010256 endomembrane system organization	11	0.54683	3.406	0.018	Biological	GO:00102	endomembrane system organization	0.54683	0.67847	0	0	11	605
GO:0070167 regulation of biomineral tissue development	2	0.54688	0.619	0.02	Biological	GO:00701	regulation of biomineral tissue development	0.54688	0.67847	0	0	2	100
GO:0110149 regulation of biomineralization	2	0.54688	0.619	0.02	Biological	GO:01101	regulation of biomineralization	0.54688	0.67847	0	0	2	100
GO:0002251 organ or tissue specific immune response	1	0.5471	0.31	0.023	Biological	GO:00022	organ or tissue specific immune response	0.5471	0.67847	0	0	1	43
GO:0002861 regulation of inflammatory response to antigenic stimulus	1	0.5471	0.31	0.023	Biological	GO:00028	regulation of inflammatory response to antigenic stimulus	0.5471	0.67847	0	0	1	43
GO:0010799 regulation of peptidyl-threonine phosphorylation	1	0.5471	0.31	0.023	Biological	GO:00107	regulation of peptidyl-threonine phosphorylation	0.5471	0.67847	0	0	1	43
GO:0010828 positive regulation of glucose transmembrane transport	1	0.5471	0.31	0.023	Biological	GO:00108	positive regulation of glucose transmembrane transport	0.5471	0.67847	0	0	1	43
GO:0016202 regulation of striated muscle tissue development	1	0.5471	0.31	0.023	Biological	GO:00162	regulation of striated muscle tissue development	0.5471	0.67847	0	0	1	43
GO:0021983 pituitary gland development	1	0.5471	0.31	0.023	Biological	GO:00219	pituitary gland development	0.5471	0.67847	0	0	1	43
GO:0030501 positive regulation of bone mineralization	1	0.5471	0.31	0.023	Biological	GO:00305	positive regulation of bone mineralization	0.5471	0.67847	0	0	1	43
GO:0031670 cellular response to nutrient	1	0.5471	0.31	0.023	Biological	GO:00316	cellular response to nutrient	0.5471	0.67847	0	0	1	43
GO:0032735 positive regulation of interleukin-12 production	1	0.5471	0.31	0.023	Biological	GO:00327	positive regulation of interleukin-12 production	0.5471	0.67847	0	0	1	43
GO:0038084 vascular endothelial growth factor signaling pathway	1	0.5471	0.31	0.023	Biological	GO:00380	vascular endothelial growth factor signaling pathway	0.5471	0.67847	0	0	1	43
GO:0046622 positive regulation of organ growth	1	0.5471	0.31	0.023	Biological	GO:00466	positive regulation of organ growth	0.5471	0.67847	0	0	1	43
GO:0051281 positive regulation of release of sequestered calcium ion in	1	0.5471	0.31	0.023	Biological	GO:00512	positive regulation of release of sequestered calcium ion into cytosol	0.5471	0.67847	0	0	1	43
GO:0071542 dopaminergic neuron differentiation	1	0.5471	0.31	0.023	Biological	GO:00715	dopaminergic neuron differentiation	0.5471	0.67847	0	0	1	43
GO:0071604 transforming growth factor beta production	1	0.5471	0.31	0.023	Biological	GO:00716	transforming growth factor beta production	0.5471	0.67847	0	0	1	43
GO:1904407 positive regulation of nitric oxide metabolic process	1	0.5471	0.31	0.023	Biological	GO:19044	positive regulation of nitric oxide metabolic process	0.5471	0.67847	0	0	1	43
GO:0140677 molecular function activator activity	4	0.54789	1.17	0.019	Molecular	GO:01406	molecular function activator activity	0.54789	0.68565	0	0	4	210
GO:0006605 protein targeting	6	0.54794	1.858	0.018	Biological	GO:00066	protein targeting	0.54794	0.67934	0	0	6	326
GO:0090150 establishment of protein localization to membrane	5	0.5485	1.548	0.019	Biological	GO:00901	establishment of protein localization to membrane	0.5485	0.67979	0	0	5	270
GO:0051493 regulation of cytoskeleton organization	10	0.54857	3.096	0.018	Biological	GO:00514	regulation of cytoskeleton organization	0.54857	0.67979	0	0	10	550
GO:0019219 regulation of nucleobase-containing compound metabolic p	73	0.54985	22.601	0.018	Biological	GO:00192	regulation of nucleobase-containing compound metabolic process	0.54985	0.6812	0	0	73	4033
GO:0019207 kinase regulator activity	5	0.54991	1.462	0.019	Molecular	GO:00192	kinase regulator activity	0.54991	0.68621	0	0	5	266
GO:0015297 antiporter activity	2	0.55041	0.585	0.02	Molecular	GO:00152	antiporter activity	0.55041	0.68621	0	0	2	99
GO:0003682 chromatin binding	11	0.55111	3.216	0.018	Molecular	GO:00036	chromatin binding	0.55111	0.68621	0	0	11	597
GO:0001772 immunological synapse	1	0.55124	0.297	0.023	Cellular C	GO:00017	immunological synapse	0.55124	0.72707	0	0	1	44
GO:0016567 protein ubiquitination	15	0.5515	4.644	0.018	Biological	GO:00165	protein ubiquitination	0.5515	0.68309	0	0	15	830
GO:0005882 intermediate filament	4	0.55175	1.187	0.018	Cellular C	GO:00058	intermediate filament	0.55175	0.72707	0	0	4	217
GO:0070647 protein modification by small protein conjugation or remova	19	0.55259	5.882	0.018	Biological	GO:00706	protein modification by small protein conjugation or removal	0.55259	0.68427	0	0	19	1053
GO:0015144 carbohydrate transmembrane transporter activity	1	0.55309	0.292	0.023	Molecular	GO:0015	carbohydrate transmembrane transporter activity	0.55309	0.68621	0	0	1	43
GO:0048156 tau protein binding	1	0.55309	0.292	0.023	Molecular	GO:0048	tau protein binding	0.55309	0.68621	0	0	1	43
GO:0140303 intramembrane lipid transporter activity	1	0.55309	0.292	0.023	Molecular	GO:01403	intramembrane lipid transporter activity	0.55309	0.68621	0	0	1	43
GO:0007017 microtubule-based process	17	0.55468	5.263	0.018	Biological	GO:00070	microtubule-based process	0.55468	0.68431	0	0	17	943
GO:0044703 multi-organism reproductive process	20	0.55502	6.192	0.018	Biological	GO:00447	multi-organism reproductive process	0.55502	0.68431	0	0	20	1110
GO:0003009 skeletal muscle contraction	1	0.55537	0.31	0.023	Biological	GO:00030	skeletal muscle contraction	0.55537	0.68431	0	0	1	44
GO:0005978 glycogen biosynthetic process	1	0.55537	0.31	0.023	Biological	GO:00059	glycogen biosynthetic process	0.55537	0.68431	0	0	1	44
GO:0006623 protein targeting to vacuole	1	0.55537	0.31	0.023	Biological	GO:00066	protein targeting to vacuole	0.55537	0.68431	0	0	1	44
GO:0008608 attachment of spindle microtubules to kinetochore	1	0.55537	0.31	0.023	Biological	GO:00086	attachment of spindle microtubules to kinetochore	0.55537	0.68431	0	0	1	44
GO:0009250 glucan biosynthetic process	1	0.55537	0.31	0.023	Biological	GO:00092	glucan biosynthetic process	0.55537	0.68431	0	0	1	44
GO:0021587 cerebellum morphogenesis	1	0.55537	0.31	0.023	Biological	GO:00215	cerebellum morphogenesis	0.55537	0.68431	0	0	1	44
GO:0034205 amyloid-beta formation	1	0.55537	0.31	0.023	Biological	GO:00342	amyloid-beta formation	0.55537	0.68431	0	0	1	44
GO:0042398 cellular modified amino acid biosynthetic process	1	0.55537	0.31	0.023	Biological	GO:00423	cellular modified amino acid biosynthetic process	0.55537	0.68431	0	0	1	44
GO:0043462 regulation of ATPase activity	1	0.55537	0.31	0.023	Biological	GO:00434	regulation of ATPase activity	0.55537	0.68431	0	0	1	44
GO:0045494 photoreceptor cell maintenance	1	0.55537	0.31	0.023	Biological	GO:00454	photoreceptor cell maintenance	0.55537	0.68431	0	0	1	44
GO:0048713 regulation of oligodendrocyte differentiation	1	0.55537	0.31	0.023	Biological	GO:00487	regulation of oligodendrocyte differentiation	0.55537	0.68431	0	0	1	44
GO:0051955 regulation of amino acid transport	1	0.55537	0.31	0.023	Biological	GO:00519	regulation of amino acid transport	0.55537	0.68431	0	0	1	44
GO:0072538 T-helper 17 type immune response	1	0.55537	0.31	0.023	Biological	GO:00725	T-helper 17 type immune response	0.55537	0.68431	0	0	1	44
GO:1900087 positive regulation of G1/S transition of mitotic cell cycle	1	0.55537	0.31	0.023	Biological	GO:19000	positive regulation of G1/S transition of mitotic cell cycle	0.55537	0.68431	0	0	1	44
GO:1901385 regulation of voltage-gated calcium channel activity	1	0.55537	0.31	0.023	Biological	GO:19013	regulation of voltage-gated calcium channel activity	0.55537	0.68431	0	0	1	44
GO:1902116 negative regulation of organelle assembly	1	0.55537	0.31	0.023	Biological	GO:1902	negative regulation of organelle assembly	0.55537	0.68431	0	0	1	44
GO:1904591 positive regulation of protein import	1	0.55537	0.31	0.023	Biological	GO:19045	positive regulation of protein import	0.55537	0.68431	0	0	1	44
GO:1905521 regulation of macrophage migration	1	0.55537	0.31	0.023	Biological	GO:19055	regulation of macrophage migration	0.55537	0.68431	0	0	1	44
GO:0005249 voltage-gated potassium channel activity	2	0.55586	0.585	0.02	Molecular	GO:00052	voltage-gated potassium channel activity	0.55586	0.68867	0	0	2	100
GO:0016573 histone acetylation	3	0.55655	0.929	0.019	Biological	GO:00165	histone acetylation	0.55655	0.68543	0	0	3	159
GO:0055088 lipid homeostasis	3	0.55655	0.929	0.019	Biological	GO:00550	lipid homeostasis	0.55655	0.68543	0	0	3	159
GO:0010769 regulation of cell morphogenesis involved in differentiation	2	0.55762	0.619	0.02	Biological	GO:00107	regulation of cell morphogenesis involved in differentiation	0.55762	0.68641	0	0	2	102
GO:0046425 regulation of receptor signaling pathway via JAK-STAT	2	0.55762	0.619	0.02	Biological	GO:00464	regulation of receptor signaling pathway via JAK-STAT	0.55762	0.68641	0	0	2	102
GO:0035966 response to topologically incorrect protein	3	0.5608	0.929	0.019	Biological	GO:00359	response to topologically incorrect protein	0.5608	0.69016	0	0	3	160
GO:0004402 histone acetyltransferase activity	1	0.56139	0.292	0.023	Molecular	GO:00044	histone acetyltransferase activity	0.56139	0.69452	0	0	1	44
GO:0015833 peptide transport	5	0.56159	1.548	0.018	Biological	GO:00158	peptide transport	0.56159	0.69096	0	0	5	274
GO:0006885 regulation of pH	2	0.56292	0.619	0.019	Biological	GO:00068	regulation of pH	0.56292	0.69126	0	0	2	103

GO:1902476 chloride transmembrane transport	2	0.56292	0.619	0.019	Biological	GO:19024	chloride transmembrane transport	0.56292	0.69126	0	0	2	103
GO:0098552 side of membrane	12	0.56306	3.561	0.018	Cellular C	GO:00985	side of membrane	0.56306	0.73849	0	0	12	676
GO:0031226 intrinsic component of plasma membrane	8	0.56318	2.374	0.018	Cellular C	GO:00312	intrinsic component of plasma membrane	0.56318	0.73849	0	0	8	449
GO:0005506 iron ion binding	3	0.56349	0.877	0.019	Molecular	GO:00055	iron ion binding	0.56349	0.69613	0	0	3	158
GO:0002701 negative regulation of production of molecular mediator of i	1	0.5635	0.31	0.022	Biological	GO:00027	negative regulation of production of molecular mediator of immune re	0.5635	0.69126	0	0	1	45
GO:0031279 regulation of cyclase activity	1	0.5635	0.31	0.022	Biological	GO:00312	regulation of cyclase activity	0.5635	0.69126	0	0	1	45
GO:0043551 regulation of phosphatidylinositol 3-kinase activity	1	0.5635	0.31	0.022	Biological	GO:00435	regulation of phosphatidylinositol 3-kinase activity	0.5635	0.69126	0	0	1	45
GO:0045646 regulation of erythrocyte differentiation	1	0.5635	0.31	0.022	Biological	GO:00456	regulation of erythrocyte differentiation	0.5635	0.69126	0	0	1	45
GO:0045663 positive regulation of myoblast differentiation	1	0.5635	0.31	0.022	Biological	GO:00456	positive regulation of myoblast differentiation	0.5635	0.69126	0	0	1	45
GO:0045840 positive regulation of mitotic nuclear division	1	0.5635	0.31	0.022	Biological	GO:00458	positive regulation of mitotic nuclear division	0.5635	0.69126	0	0	1	45
GO:0048701 embryonic cranial skeleton morphogenesis	1	0.5635	0.31	0.022	Biological	GO:00487	embryonic cranial skeleton morphogenesis	0.5635	0.69126	0	0	1	45
GO:0071827 plasma lipoprotein particle organization	1	0.5635	0.31	0.022	Biological	GO:00718	plasma lipoprotein particle organization	0.5635	0.69126	0	0	1	45
GO:0097009 energy homeostasis	1	0.5635	0.31	0.022	Biological	GO:00970	energy homeostasis	0.5635	0.69126	0	0	1	45
GO:2000378 negative regulation of reactive oxygen species metabolic p	1	0.5635	0.31	0.022	Biological	GO:20003	negative regulation of reactive oxygen species metabolic process	0.5635	0.69126	0	0	1	45
GO:0022898 regulation of transmembrane transporter activity	5	0.56484	1.548	0.018	Biological	GO:00228	regulation of transmembrane transporter activity	0.56484	0.69246	0	0	5	275
GO:0030902 hindbrain development	3	0.56504	0.929	0.019	Biological	GO:00309	hindbrain development	0.56504	0.69246	0	0	3	161
GO:1903034 regulation of response to wounding	3	0.56504	0.929	0.019	Biological	GO:19030	regulation of response to wounding	0.56504	0.69246	0	0	3	161
GO:1990845 adaptive thermogenesis	3	0.56504	0.929	0.019	Biological	GO:19908	adaptive thermogenesis	0.56504	0.69246	0	0	3	161
GO:0034765 regulation of ion transmembrane transport	9	0.56522	2.786	0.018	Biological	GO:00347	regulation of ion transmembrane transport	0.56522	0.69252	0	0	9	501
GO:0019233 sensory perception of pain	2	0.56818	0.619	0.019	Biological	GO:00192	sensory perception of pain	0.56818	0.69598	0	0	2	104
GO:0004715 non-membrane spanning protein tyrosine kinase activity	1	0.56954	0.292	0.022	Molecular	GO:00047	non-membrane spanning protein tyrosine kinase activity	0.56954	0.70159	0	0	1	45
GO:0051879 Hsp90 protein binding	1	0.56954	0.292	0.022	Molecular	GO:00518	Hsp90 protein binding	0.56954	0.70159	0	0	1	45
GO:0002920 regulation of humoral immune response	1	0.57148	0.31	0.022	Biological	GO:00029	regulation of humoral immune response	0.57148	0.6983	0	0	1	46
GO:0014888 striated muscle adaptation	1	0.57148	0.31	0.022	Biological	GO:00148	striated muscle adaptation	0.57148	0.6983	0	0	1	46
GO:0016578 histone deubiquitination	1	0.57148	0.31	0.022	Biological	GO:00165	histone deubiquitination	0.57148	0.6983	0	0	1	46
GO:0043029 T cell homeostasis	1	0.57148	0.31	0.022	Biological	GO:00430	T cell homeostasis	0.57148	0.6983	0	0	1	46
GO:0045747 positive regulation of Notch signaling pathway	1	0.57148	0.31	0.022	Biological	GO:00457	positive regulation of Notch signaling pathway	0.57148	0.6983	0	0	1	46
GO:0046460 neutral lipid biosynthetic process	1	0.57148	0.31	0.022	Biological	GO:00464	neutral lipid biosynthetic process	0.57148	0.6983	0	0	1	46
GO:0046463 acylglycerol biosynthetic process	1	0.57148	0.31	0.022	Biological	GO:00464	acylglycerol biosynthetic process	0.57148	0.6983	0	0	1	46
GO:0046636 negative regulation of alpha-beta T cell activation	1	0.57148	0.31	0.022	Biological	GO:00466	negative regulation of alpha-beta T cell activation	0.57148	0.6983	0	0	1	46
GO:0097300 programmed necrotic cell death	1	0.57148	0.31	0.022	Biological	GO:00973	programmed necrotic cell death	0.57148	0.6983	0	0	1	46
GO:1901021 positive regulation of calcium ion transmembrane transpor	1	0.57148	0.31	0.022	Biological	GO:19010	positive regulation of calcium ion transmembrane transporter activity	0.57148	0.6983	0	0	1	46
GO:0006457 protein folding	4	0.57163	1.238	0.018	Biological	GO:00064	protein folding	0.57163	0.69832	0	0	4	220
GO:0015914 phospholipid transport	2	0.57339	0.619	0.019	Biological	GO:00159	phospholipid transport	0.57339	0.69965	0	0	2	105
GO:0022600 digestive system process	2	0.57339	0.619	0.019	Biological	GO:00226	digestive system process	0.57339	0.69965	0	0	2	105
GO:0035249 synaptic transmission, glutamatergic	2	0.57339	0.619	0.019	Biological	GO:00352	synaptic transmission, glutamatergic	0.57339	0.69965	0	0	2	105
GO:1905477 positive regulation of protein localization to membrane	2	0.57339	0.619	0.019	Biological	GO:19054	positive regulation of protein localization to membrane	0.57339	0.69965	0	0	2	105
GO:0040029 regulation of gene expression, epigenetic	3	0.57342	0.929	0.018	Biological	GO:00400	regulation of gene expression, epigenetic	0.57342	0.69965	0	0	3	163
GO:0000776 kinetochore	3	0.57397	0.89	0.018	Cellular C	GO:00007	kinetochore	0.57397	0.75048	0	0	3	165
GO:0043195 terminal bouton	1	0.57513	0.297	0.021	Cellular C	GO:00431	terminal bouton	0.57513	0.75048	0	0	1	47
GO:1902903 regulation of supramolecular fiber organization	7	0.57523	2.167	0.018	Biological	GO:19029	regulation of supramolecular fiber organization	0.57523	0.70169	0	0	7	392
GO:0001784 phosphotyrosine residue binding	1	0.57754	0.292	0.022	Molecular	GO:00017	phosphotyrosine residue binding	0.57754	0.70943	0	0	1	46
GO:0032266 phosphatidylinositol-3-phosphate binding	1	0.57754	0.292	0.022	Molecular	GO:00322	phosphatidylinositol-3-phosphate binding	0.57754	0.70943	0	0	1	46
GO:0006644 phospholipid metabolic process	7	0.57793	2.167	0.018	Biological	GO:00066	phospholipid metabolic process	0.57793	0.70339	0	0	7	393
GO:0051726 regulation of cell cycle	20	0.57813	6.192	0.018	Biological	GO:00517	regulation of cell cycle	0.57813	0.70339	0	0	20	1124
GO:0042116 macrophage activation	2	0.57856	0.619	0.019	Biological	GO:00421	macrophage activation	0.57856	0.70339	0	0	2	106
GO:0006775 fat-soluble vitamin metabolic process	1	0.57931	0.31	0.021	Biological	GO:00067	fat-soluble vitamin metabolic process	0.57931	0.70339	0	0	1	47
GO:0010824 regulation of centrosome duplication	1	0.57931	0.31	0.021	Biological	GO:00108	regulation of centrosome duplication	0.57931	0.70339	0	0	1	47
GO:0021575 hindbrain morphogenesis	1	0.57931	0.31	0.021	Biological	GO:00215	hindbrain morphogenesis	0.57931	0.70339	0	0	1	47
GO:0030517 negative regulation of axon extension	1	0.57931	0.31	0.021	Biological	GO:00305	negative regulation of axon extension	0.57931	0.70339	0	0	1	47
GO:0030521 androgen receptor signaling pathway	1	0.57931	0.31	0.021	Biological	GO:00305	androgen receptor signaling pathway	0.57931	0.70339	0	0	1	47
GO:0032527 protein exit from endoplasmic reticulum	1	0.57931	0.31	0.021	Biological	GO:00325	protein exit from endoplasmic reticulum	0.57931	0.70339	0	0	1	47
GO:0032964 collagen biosynthetic process	1	0.57931	0.31	0.021	Biological	GO:00329	collagen biosynthetic process	0.57931	0.70339	0	0	1	47
GO:0035384 thioester biosynthetic process	1	0.57931	0.31	0.021	Biological	GO:00353	thioester biosynthetic process	0.57931	0.70339	0	0	1	47
GO:0042220 response to cocaine	1	0.57931	0.31	0.021	Biological	GO:00422	response to cocaine	0.57931	0.70339	0	0	1	47
GO:0046189 phenol-containing compound biosynthetic process	1	0.57931	0.31	0.021	Biological	GO:00461	phenol-containing compound biosynthetic process	0.57931	0.70339	0	0	1	47
GO:0048634 regulation of muscle organ development	1	0.57931	0.31	0.021	Biological	GO:00486	regulation of muscle organ development	0.57931	0.70339	0	0	1	47
GO:0071616 acyl-CoA biosynthetic process	1	0.57931	0.31	0.021	Biological	GO:00716	acyl-CoA biosynthetic process	0.57931	0.70339	0	0	1	47
GO:0072583 clathrin-dependent endocytosis	1	0.57931	0.31	0.021	Biological	GO:00725	clathrin-dependent endocytosis	0.57931	0.70339	0	0	1	47
GO:0090199 regulation of release of cytochrome c from mitochondria	1	0.57931	0.31	0.021	Biological	GO:00901	regulation of release of cytochrome c from mitochondria	0.57931	0.70339	0	0	1	47
GO:0120163 negative regulation of cold-induced thermogenesis	1	0.57931	0.31	0.021	Biological	GO:01201	negative regulation of cold-induced thermogenesis	0.57931	0.70339	0	0	1	47
GO:1902459 positive regulation of stem cell population maintenance	1	0.57931	0.31	0.021	Biological	GO:19024	positive regulation of stem cell population maintenance	0.57931	0.70339	0	0	1	47
GO:0009895 negative regulation of catabolic process	6	0.58045	1.858	0.018	Biological	GO:00098	negative regulation of catabolic process	0.58045	0.70461	0	0	6	337
GO:0016787 hydrolase activity	45	0.58113	13.158	0.018	Molecular	GO:00167	hydrolase activity	0.58113	0.71283	0	0	45	2476
GO:0031267 small GTPase binding	5	0.58276	1.462	0.018	Molecular	GO:00312	small GTPase binding	0.58276	0.71372	0	0	5	276
GO:0097542 ciliary tip	1	0.58281	0.297	0.021	Cellular C	GO:00975	ciliary tip	0.58281	0.75865	0	0	1	48
GO:0016042 lipid catabolic process	6	0.58335	1.858	0.018	Biological	GO:00160	lipid catabolic process	0.58335	0.70795	0	0	6	338
GO:0003279 cardiac septum development	2	0.58368	0.619	0.019	Biological	GO:00032	cardiac septum development	0.58368	0.70804	0	0	2	107
GO:0002683 negative regulation of immune system process	8	0.5837	2.477	0.018	Biological	GO:00026	negative regulation of immune system process	0.5837	0.70804	0	0	8	452
GO:0019894 kinesin binding	1	0.58539	0.292	0.021	Molecular	GO:00198	kinesin binding	0.58539	0.71372	0	0	1	47
GO:0061733 peptide-lysine-N-acetyltransferase activity	1	0.58539	0.292	0.021	Molecular	GO:00617	peptide-lysine-N-acetyltransferase activity	0.58539	0.71372	0	0	1	47
GO:0005524 ATP binding	27	0.58558	7.895	0.018	Molecular	GO:00055	ATP binding	0.58558	0.71372	0	0	27	1495
GO:0018393 internal peptidyl-lysine acetylation	3	0.58581	0.929	0.018	Biological	GO:00183	internal peptidyl-lysine acetylation	0.58581	0.70963	0	0	3	166
GO:0051494 negative regulation of cytoskeleton organization	3	0.58581	0.929	0.018	Biological	GO:00514	negative regulation of cytoskeleton organization	0.58581	0.70963	0	0	3	166
GO:0010466 negative regulation of peptidase activity	4	0.58589	1.238	0.018	Biological	GO:00104	negative regulation of peptidase activity	0.58589	0.70963	0	0	4	224
GO:0035091 phosphatidylinositol binding	5	0.58597	1.462	0.018	Molecular	GO:00350	phosphatidylinositol binding	0.58597	0.71372	0	0	5	277
GO:0019884 antigen processing and presentation of exogenous antigen	1	0.587	0.31	0.021	Biological	GO:00198	antigen processing and presentation of exogenous antigen	0.587	0.70963	0	0	1	48
GO:0031641 regulation of myelination	1	0.587	0.31	0.021	Biological	GO:00316	regulation of myelination	0.587	0.70963	0	0	1	48
GO:0035987 endodermal cell differentiation	1	0.587	0.31	0.021	Biological	GO:00359	endodermal cell differentiation	0.587	0.70963	0	0	1	48
GO:0036230 granulocyte activation	1	0.587	0.31	0.021	Biological	GO:00362	granulocyte activation	0.587	0.70963	0	0	1	48
GO:0043124 negative regulation of I-kappaB kinase/NF-kappaB signalir	1	0.587	0.31	0.021	Biological	GO:00431	negative regulation of I-kappaB kinase/NF-kappaB signaling	0.587	0.70963	0	0	1	48
GO:0051339 regulation of lyase activity	1	0.587	0.31	0.021	Biological	GO:00513	regulation of lyase activity	0.587	0.70963	0	0	1	48
GO:0060976 coronary vasculature development	1	0.587	0.31	0.021	Biological	GO:00609	coronary vasculature development	0.587	0.70963	0	0	1	48
GO:0071825 protein-lipid complex subunit organization	1	0.587	0.31	0.021	Biological	GO:00718	protein-lipid complex subunit organization	0.587	0.70963	0	0	1	48
GO:1902041 regulation of extrinsic apoptotic signaling pathway via deat	1	0.587	0.31	0.021	Biological	GO:19020	regulation of extrinsic apoptotic signaling pathway via death domain r	0.587	0.70963	0	0	1	48
GO:1902930 regulation of alcohol biosynthetic process	1	0.587	0.31	0.021	Biological	GO:19029	regulation of alcohol biosynthetic process	0.587	0.70963	0	0	1	48
GO:2000404 regulation of T cell migration	1	0.587	0.31	0.021	Biological	GO:20004	regulation of T cell migration	0.587	0.70963	0	0	1	48
GO:0046649 lymphocyte activation	15	0.58728	4.644	0.018	Biological	GO:00466	lymphocyte activation	0.58728	0.7098	0	0	15	849
GO:0005903 brush border	2	0.58756	0.593	0.018	Cellular C	GO:00059	brush border	0.58756	0.76297	0	0	2	109
GO:0046486 glycerolipid metabolic process	7	0.58866	2.167	0.018	Biological	GO:00464	glycerolipid metabolic process	0.58866	0.71072	0	0	7	397
GO:0002824 positive regulation of adaptive immune response based on	2	0.58875	0.619	0.019	Biological	GO:00028	positive regulation of adaptive immune response based on somatic re	0.58875	0.71072	0	0	2	108
GO:0005976 polysaccharide metabolic process	2	0.58875	0.619	0.019	Biological	GO:00059	polysaccharide metabolic process	0.58875	0.71072	0	0	2	108
GO:0032006 regulation of TOR signaling	2	0.58875	0.619	0.019	Biological	GO:00320	regulation of TOR signaling	0.58875	0.71072	0	0	2	108
GO:1904892 regulation of receptor signaling pathway via STAT	2	0.58875	0.619	0.019	Biological	GO:19048	regulation of receptor signaling pathway via STAT	0.58875	0.71072	0	0	2	108
GO:0007259 receptor signaling pathway via JAK-STAT	3	0.58989	0.929	0.018	Biological	GO:00072	receptor signaling pathway via JAK-STAT	0.58989	0.71157	0	0	3	167
GO:0007338 single fertilization	3	0.58989	0.929	0.018	Biological	GO:00073	single fertilization	0.58989	0.71157	0	0	3	167
GO:0031960 response to corticosteroid	3	0.58989	0.929	0.018	Biological	GO:00319	response to corticosteroid	0.58989	0.71157	0	0	3	167
GO:0097730 non-motile cilium	3	0.59023	0.89	0.018	Cellular C	GO:00977	non-motile cilium	0.59023	0.76459	0	0	3	169
GO:0043226 organelle	254	0.59296	75.371	0.018	Cellular C	GO:00432	organelle	0.59296	0.76626	0	0	254	14161
GO:0044260 cellular macromolecule metabolic process	143	0.59305	44.272	0.018	Biological	GO:00442	cellular macromolecule metabolic process	0.59305	0.71521	0	0	143	7932
GO:0016538 cyclin-dependent protein serine/threonine kinase regulator	1	0.59309	0.292	0.021	Molecular	GO:00165	cyclin-dependent protein serine/threonine kinase regulator activity	0.59309	0.72036	0	0	1	48
GO:0048020 CCR chemokine receptor binding	1	0.59309	0.292	0.021	Molecular	GO:00480	CCR chemokine receptor binding	0.59309	0.72036	0	0	1	48
GO:0009991 response to extracellular stimulus	9	0.59381	2.786	0.018	Biological	GO:00099	response to extracellular stimulus	0.59381	0.71563	0	0	9	513
GO:1902904 negative regulation of supramolecular fiber organization	3	0.59394	0.929	0.018	Biological	GO:19029	negative regulation of supramolecular fiber organization	0.59394	0.71563	0	0	3	168
GO:0006631 fatty acid metabolic process	7	0.59397	2.167	0.018	Biological	GO:00066	fatty acid metabolic process	0.59397	0.71563	0	0	7	399
GO:0051251 positive regulation of lymphocyte activation	7	0.59397	2.167	0.018	Biological	GO:00512	positive regulation of lymphocyte activation	0.59397	0.71563	0	0	7	399
GO:0008038 neuron recognition	1	0.59455	0.31	0.02	Biological	GO:00080	neuron recognition	0.59455	0.71563	0	0	1	49
GO:0045581 negative regulation of T cell differentiation	1	0.59455	0.31	0.02	Biological	GO:00455	negative regulation of T cell differentiation	0.59455	0.71563	0	0	1	49
GO:0048286 lung alveolus development	1	0.59455	0.31	0.02	Biological	GO:00482	lung alveolus development	0.59455	0.71563	0	0	1	49
GO:1903747 regulation of establishment of protein localization to mitoch	1	0.59455	0.31	0.02	Biological	GO:19037	regulation of establishment of protein localization to mitochondrion	0.59455	0.71563	0	0	1	49
GO:0043161 proteasome-mediated ubiquitin-dependent protein catabol	8	0.59617	2.477	0.018	Biological	GO:00431	proteasome-mediated ubiquitin-dependent protein catabolic process	0.59617	0.7178	0	0	8	457
GO:0009743 response to carbohydrate	4	0.5964	1.238	0.018	Biological	GO:00097	response to carbohydrate	0.5964	0.71718	0	0	4	227
GO:0017038 protein import	4	0.5964	1.238	0.018	Biological	GO:00170	protein import	0.5964	0.71718	0	0	4	227
GO:0072593 reactive oxygen species metabolic process	4	0.5964	1.238	0.018	Biological	GO:00725	reactive oxygen species metabolic process	0.5964	0.71718	0	0	4	227
GO:0050796 regulation of insulin secretion	3	0.59797	0.929	0.018	Biological	GO:00507	regulation of insulin secretion	0.59797	0.71872	0	0	3	169
GO:1902850 microtubule cytoskeleton organization involved in mitosis	3	0.59797	0.929	0.018	Biological	GO:19028	microtubule cytoskeleton organization involved in mitosis	0.59797	0.71872	0	0	3	169
GO:0007218 neuropeptide signaling pathway	2	0.59876	0.619	0.018	Biological	GO:00072	neuropeptide signaling pathway	0.59876	0.71932	0	0	2	110
GO:0048640 negative regulation of developmental growth	2	0.59876	0.619	0.018	Biological	GO:00486	negative regulation of developmental growth	0.59876	0.71932	0	0	2	110
GO:0016903 oxidoreductase activity, acting on the aldehyde or oxo grou	1	0.60065	0.292	0.02	Molecular	GO:00169	oxidoreductase activity, acting on the aldehyde or oxo group of donor	0.60065	0.72808	0	0	1	49
GO:0007186 G protein-coupled receptor signaling pathway	22	0.6018	6.811	0.018	Biological	GO:00071	G protein-coupled receptor signaling pathway	0.6018	0.7211	0	0	22	1251
GO:0030246 carbohydrate binding	5	0.60184	1.462	0.018	Molecular	GO:00302	carbohydrate binding	0.60184	0.72808	0	0	5	282
GO:0002066 columnar/cuboidal epithelial cell development	1	0.60196	0.31	0.02	Biological	GO:00020	columnar/cuboidal epithelial cell development	0.60196	0.7211	0	0	1	50
GO:0003044 regulation of systemic arterial blood pressure mediated by	1	0.60196	0.31	0.02	Biological	GO:00030	regulation of systemic arterial blood pressure mediated by a chemica	0.60196	0.7211	0	0	1	50
GO:0006111 regulation of gluconeogenesis	1	0.60196	0.31	0.02	Biological	GO:00061	regulation of gluconeogenesis	0.60196	0.7211	0	0	1	50
GO:0008089 anterograde axonal transport	1	0.60196	0.31	0.02	Biological	GO:00080	anterograde axonal transport	0.60196	0.7211	0	0	1	50
GO:0030195 negative regulation of blood coagulation	1	0.60196	0.31	0.02	Biological	GO:00301	negative regulation of blood coagulation	0.60196	0.7211	0	0	1	50
GO:0032715 negative regulation of interleukin-6 production	1	0.60196	0.31	0.02	Biological	GO:00327	negative regulation of interleukin-6 production	0.60196	0.7211	0	0	1	50
GO:0086002 cardiac muscle cell action potential involved in contraction	1	0.60196	0.31	0.02	Biological	GO:00860	cardiac muscle cell action potential involved in contraction	0.60196	0.7211	0	0	1	50
GO:1903432 regulation of TORC1 signaling	1	0.60196	0.31	0.02	Biological	GO:19034	regulation of TORC1 signaling	0.60196	0.7211	0	0	1	50
GO:2000351 regulation of endothelial cell apoptotic process	1	0.60196	0.31	0.02	Biological	GO:20003	regulation of endothelial cell apoptotic process	0.60196	0.7211	0	0	1	50
GO:2001238 positive regulation of extrinsic apoptotic signaling pathway	1	0.60196	0.31	0.02	Biological	GO:20012	positive regulation of extrinsic apoptotic signaling pathway	0.60196	0.7211	0	0	1	50
GO:0010970 transport along microtubule	3	0.60197	0.929	0.018	Biological	GO:00109	transport along microtubule	0.60197	0.7211	0	0	3	170
GO:0042470 melanosome	2	0.60236	0.593	0.018	Cellular C	GO:00424	melanosome	0.60236	0.77467	0	0	2	112
GO:0048770 pigment granule	2	0.60236	0.593	0.018	Cellular C	GO:00487	pigment granule	0.60236	0.77467	0	0	2	112
GO:0016747 acyltransferase activity, transferring groups other than am	4	0.60245	1.17	0.018	Molecular	GO:00167	acyltransferase activity, transferring groups other than amino-acyl gr	0.60245	0.72808	0	0	4	225
GO:0005200 structural constituent of cytoskeleton	2	0.60281	0.585	0.018	Molecular	GO:00052	structural constituent of cytoskeleton	0.60281	0.72808	0	0	2	109
GO:0000079 regulation of cyclin-dependent protein serine/threonine kina	2	0.6037	0.619	0.018	Biological	GO:00000	regulation of cyclin-dependent protein serine/threonine kinase activity	0.6037	0.72247	0	0	2	111
GO:0048024 regulation of mRNA splicing, via spliceosome	2	0.6037	0.619	0.018	Biological	GO:00480	regulation of mRNA splicing, via spliceosome	0.6037	0.72247	0	0	2	111
GO:0048259 regulation of receptor-mediated endocytosis	2	0.6037	0.619	0.018	Biological	GO:00482	regulation of receptor-mediated endocytosis	0.6037	0.72247	0	0	2	111
GO:0072089 stem cell proliferation	2	0.6037	0.619	0.018	Biological	GO:00720	stem cell proliferation	0.6037	0.72247	0	0	2	111
GO:0005694 chromosome	33	0.60416	9.792	0.017	Cellular C	GO:00056	chromosome	0.60416	0.77512	0	0	33	1890
GO:0015294 solute:cation symporter activity	2	0.60779	0.585	0.018	Molecular	GO:00152	solute:cation symporter activity	0.60779	0.73138	0	0	2	110
GO:1902936 phosphatidylinositol bisphosphate binding	2	0.60779	0.585	0.018	Molecular	GO:19029	phosphatidylinositol bisphosphate binding	0.60779	0.73138	0	0	2	110
GO:0070382 exocytic vesicle	4	0.60798	1.187	0.017	Cellular C	GO:00703	exocytic vesicle	0.60798	0.77816	0	0	4	233
GO:0140359 ABC-type transporter activity	1	0.60807	0.292	0.02	Molecular	GO:01403	ABC-type transporter activity	0.60807	0.73138	0	0	1	50
GO:0050867 positive regulation of cell activation	8	0.60845	2.477	0.017	Biological	GO:00508	positive regulation of cell activation	0.60845	0.72701	0	0	8	462
GO:0002832 negative regulation of response to biotic stimulus	2	0.60859	0.619	0.018	Biological	GO:00028	negative regulation of response to biotic stimulus	0.60859	0.72701	0	0	2	112
GO:0035282 segmentation	2	0.60859	0.619	0.018	Biological	GO:00352	segmentation	0.60859	0.72701	0	0	2	112
GO:0006120 mitochondrial electron transport, NADH to ubiquinone	1	0.60924	0.31	0.02	Biological	GO:00061	mitochondrial electron transport, NADH to ubiquinone	0.60924	0.72701	0	0	1	51
GO:0018208 peptidyl-proline modification	1	0.60924	0.31	0.02	Biological	GO:00182	peptidyl-proline modification	0.60924	0.72701	0	0	1	51
GO:0031146 SCF-dependent proteasomal ubiquitin-dependent protein c	1	0.60924	0.31	0.02	Biological	GO:00311	SCF-dependent proteasomal ubiquitin-dependent protein catabolic pr	0.60924	0.72701	0	0	1	51
GO:0043268 positive regulation of potassium ion transport	1	0.60924	0.31	0.02	Biological	GO:00432	positive regulation of potassium ion transport	0.60924	0.72701	0	0	1	51

GO:0043277 apoptotic cell clearance	1	0.60924	0.31	0.02	Biological	GO:00432	apoptotic cell clearance	0.60924	0.72701	0	0	1	51
GO:0045058 T cell selection	1	0.60924	0.31	0.02	Biological	GO:00450	T cell selection	0.60924	0.72701	0	0	1	51
GO:0045776 negative regulation of blood pressure	1	0.60924	0.31	0.02	Biological	GO:00457	negative regulation of blood pressure	0.60924	0.72701	0	0	1	51
GO:0060119 inner ear receptor cell development	1	0.60924	0.31	0.02	Biological	GO:00601	inner ear receptor cell development	0.60924	0.72701	0	0	1	51
GO:1900047 negative regulation of hemostasis	1	0.60924	0.31	0.02	Biological	GO:19000	negative regulation of hemostasis	0.60924	0.72701	0	0	1	51
GO:0000287 magnesium ion binding	4	0.60942	1.17	0.018	Molecular	GO:00002	magnesium ion binding	0.60942	0.73198	0	0	4	227
GO:0014704 intercalated disc	1	0.61216	0.297	0.019	Cellular C	GO:00147	intercalated disc	0.61216	0.78164	0	0	1	52
GO:0032526 response to retinoic acid	2	0.61343	0.619	0.018	Biological	GO:00325	response to retinoic acid	0.61343	0.73184	0	0	2	113
GO:0097696 receptor signaling pathway via STAT	3	0.61382	0.929	0.017	Biological	GO:00976	receptor signaling pathway via STAT	0.61382	0.73213	0	0	3	173
GO:0032535 regulation of cellular component size	7	0.61487	2.167	0.017	Biological	GO:00325	regulation of cellular component size	0.61487	0.73321	0	0	7	407
GO:0010043 response to zinc ion	1	0.61638	0.31	0.019	Biological	GO:00100	response to zinc ion	0.61638	0.73395	0	0	1	52
GO:0045778 positive regulation of ossification	1	0.61638	0.31	0.019	Biological	GO:00457	positive regulation of ossification	0.61638	0.73395	0	0	1	52
GO:0046580 negative regulation of Ras protein signal transduction	1	0.61638	0.31	0.019	Biological	GO:00465	negative regulation of Ras protein signal transduction	0.61638	0.73395	0	0	1	52
GO:0048013 ephrin receptor signaling pathway	1	0.61638	0.31	0.019	Biological	GO:00480	ephrin receptor signaling pathway	0.61638	0.73395	0	0	1	52
GO:0060425 lung morphogenesis	1	0.61638	0.31	0.019	Biological	GO:00604	lung morphogenesis	0.61638	0.73395	0	0	1	52
GO:0120009 intermembrane lipid transfer	1	0.61638	0.31	0.019	Biological	GO:01200	intermembrane lipid transfer	0.61638	0.73395	0	0	1	52
GO:0006694 steroid biosynthetic process	3	0.61771	0.929	0.017	Biological	GO:00066	steroid biosynthetic process	0.61771	0.73519	0	0	3	174
GO:0007254 JNK cascade	3	0.61771	0.929	0.017	Biological	GO:00072	JNK cascade	0.61771	0.73519	0	0	3	174
GO:0000779 condensed chromosome, centromeric region	3	0.61773	0.89	0.017	Cellular C	GO:00007	condensed chromosome, centromeric region	0.61773	0.78687	0	0	3	176
GO:0002821 positive regulation of adaptive immune response	2	0.61823	0.619	0.018	Biological	GO:00028	positive regulation of adaptive immune response	0.61823	0.73546	0	0	2	114
GO:1904029 regulation of cyclin-dependent protein kinase activity	2	0.61823	0.619	0.018	Biological	GO:19040	regulation of cyclin-dependent protein kinase activity	0.61823	0.73546	0	0	2	114
GO:0071704 organic substance metabolic process	197	0.61986	60.991	0.018	Biological	GO:00717	organic substance metabolic process	0.61986	0.73722	0	0	197	10922
GO:0051495 positive regulation of cytoskeleton organization	4	0.62031	1.238	0.017	Biological	GO:00514	positive regulation of cytoskeleton organization	0.62031	0.73758	0	0	4	234
GO:0034451 centriolar satellite	2	0.62148	0.593	0.017	Cellular C	GO:00344	centriolar satellite	0.62148	0.78977	0	0	2	116
GO:0034212 peptide N-acetyltransferase activity	1	0.62251	0.292	0.019	Molecular	GO:00342	peptide N-acetyltransferase activity	0.62251	0.7446	0	0	1	52
GO:0051018 protein kinase A binding	1	0.62251	0.292	0.019	Molecular	GO:00510	protein kinase A binding	0.62251	0.7446	0	0	1	52
GO:0120013 lipid transfer activity	1	0.62251	0.292	0.019	Molecular	GO:01200	lipid transfer activity	0.62251	0.7446	0	0	1	52
GO:0031532 actin cytoskeleton reorganization	2	0.62298	0.619	0.017	Biological	GO:00315	actin cytoskeleton reorganization	0.62298	0.73826	0	0	2	115
GO:0046916 cellular transition metal ion homeostasis	2	0.62298	0.619	0.017	Biological	GO:00469	cellular transition metal ion homeostasis	0.62298	0.73826	0	0	2	115
GO:0002204 somatic recombination of immunoglobulin genes involved i	1	0.62339	0.31	0.019	Biological	GO:00022	somatic recombination of immunoglobulin genes involved in immune	0.62339	0.73826	0	0	1	53
GO:0002208 somatic diversification of immunoglobulins involved in imm	1	0.62339	0.31	0.019	Biological	GO:00022	somatic diversification of immunoglobulins involved in immune respo	0.62339	0.73826	0	0	1	53
GO:0006749 glutathione metabolic process	1	0.62339	0.31	0.019	Biological	GO:00067	glutathione metabolic process	0.62339	0.73826	0	0	1	53
GO:0006890 retrograde vesicle-mediated transport, Golgi to endoplasm	1	0.62339	0.31	0.019	Biological	GO:00068	retrograde vesicle-mediated transport, Golgi to endoplasmic reticulum	0.62339	0.73826	0	0	1	53
GO:0010823 negative regulation of mitochondrion organization	1	0.62339	0.31	0.019	Biological	GO:00108	negative regulation of mitochondrion organization	0.62339	0.73826	0	0	1	53
GO:0019083 viral transcription	1	0.62339	0.31	0.019	Biological	GO:00190	viral transcription	0.62339	0.73826	0	0	1	53
GO:0045190 isotype switching	1	0.62339	0.31	0.019	Biological	GO:00451	isotype switching	0.62339	0.73826	0	0	1	53
GO:0045197 establishment or maintenance of epithelial cell apical/basal	1	0.62339	0.31	0.019	Biological	GO:00451	establishment or maintenance of epithelial cell apical/basal polarity	0.62339	0.73826	0	0	1	53
GO:0045332 phospholipid translocation	1	0.62339	0.31	0.019	Biological	GO:00453	phospholipid translocation	0.62339	0.73826	0	0	1	53
GO:0045599 negative regulation of fat cell differentiation	1	0.62339	0.31	0.019	Biological	GO:00455	negative regulation of fat cell differentiation	0.62339	0.73826	0	0	1	53
GO:0048146 positive regulation of fibroblast proliferation	1	0.62339	0.31	0.019	Biological	GO:00481	positive regulation of fibroblast proliferation	0.62339	0.73826	0	0	1	53
GO:0050732 negative regulation of peptidyl-tyrosine phosphorylation	1	0.62339	0.31	0.019	Biological	GO:00507	negative regulation of peptidyl-tyrosine phosphorylation	0.62339	0.73826	0	0	1	53
GO:0060986 endocrine hormone secretion	1	0.62339	0.31	0.019	Biological	GO:00609	endocrine hormone secretion	0.62339	0.73826	0	0	1	53
GO:0070169 positive regulation of biomineral tissue development	1	0.62339	0.31	0.019	Biological	GO:00701	positive regulation of biomineral tissue development	0.62339	0.73826	0	0	1	53
GO:0110151 positive regulation of biomineralization	1	0.62339	0.31	0.019	Biological	GO:01101	positive regulation of biomineralization	0.62339	0.73826	0	0	1	53
GO:0031348 negative regulation of defense response	4	0.62365	1.238	0.017	Biological	GO:00313	negative regulation of defense response	0.62365	0.73838	0	0	4	235
GO:0031349 positive regulation of defense response	5	0.62405	1.548	0.017	Biological	GO:00313	positive regulation of defense response	0.62405	0.73869	0	0	5	294
GO:0005775 vacuolar lumen	3	0.62535	0.89	0.017	Cellular C	GO:00057	vacuolar lumen	0.62535	0.78995	0	0	3	178
GO:0002822 regulation of adaptive immune response based on somatic	3	0.62543	0.929	0.017	Biological	GO:00028	regulation of adaptive immune response based on somatic recombina	0.62543	0.74014	0	0	3	176
GO:0010498 proteasomal protein catabolic process	9	0.626	2.786	0.017	Biological	GO:00104	proteasomal protein catabolic process	0.626	0.74064	0	0	9	527
GO:0030286 dynein complex	1	0.62606	0.297	0.019	Cellular C	GO:00302	dynein complex	0.62606	0.78995	0	0	1	54
GO:0030315 T-tubule	1	0.62606	0.297	0.019	Cellular C	GO:00303	T-tubule	0.62606	0.78995	0	0	1	54
GO:0021761 limbic system development	2	0.62769	0.619	0.017	Biological	GO:00217	limbic system development	0.62769	0.74229	0	0	2	116
GO:0045582 positive regulation of T cell differentiation	2	0.62769	0.619	0.017	Biological	GO:00455	positive regulation of T cell differentiation	0.62769	0.74229	0	0	2	116
GO:0018394 peptidyl-lysine acetylation	3	0.62924	0.929	0.017	Biological	GO:00183	peptidyl-lysine acetylation	0.62924	0.74306	0	0	3	177
GO:0004869 cysteine-type endopeptidase inhibitor activity	1	0.62952	0.292	0.019	Molecular	GO:00048	cysteine-type endopeptidase inhibitor activity	0.62952	0.75092	0	0	1	53
GO:0005544 calcium-dependent phospholipid binding	1	0.62952	0.292	0.019	Molecular	GO:00055	calcium-dependent phospholipid binding	0.62952	0.75092	0	0	1	53
GO:0006695 cholesterol biosynthetic process	1	0.63028	0.31	0.019	Biological	GO:00066	cholesterol biosynthetic process	0.63028	0.74306	0	0	1	54
GO:0009409 response to cold	1	0.63028	0.31	0.019	Biological	GO:00094	response to cold	0.63028	0.74306	0	0	1	54
GO:0010611 regulation of cardiac muscle hypertrophy	1	0.63028	0.31	0.019	Biological	GO:00106	regulation of cardiac muscle hypertrophy	0.63028	0.74306	0	0	1	54
GO:0021695 cerebellar cortex development	1	0.63028	0.31	0.019	Biological	GO:00216	cerebellar cortex development	0.63028	0.74306	0	0	1	54
GO:0042149 cellular response to glucose starvation	1	0.63028	0.31	0.019	Biological	GO:00421	cellular response to glucose starvation	0.63028	0.74306	0	0	1	54
GO:0043525 positive regulation of neuron apoptotic process	1	0.63028	0.31	0.019	Biological	GO:00435	positive regulation of neuron apoptotic process	0.63028	0.74306	0	0	1	54
GO:0046605 regulation of centrosome cycle	1	0.63028	0.31	0.019	Biological	GO:00466	regulation of centrosome cycle	0.63028	0.74306	0	0	1	54
GO:0048260 positive regulation of receptor-mediated endocytosis	1	0.63028	0.31	0.019	Biological	GO:00482	positive regulation of receptor-mediated endocytosis	0.63028	0.74306	0	0	1	54
GO:0050819 negative regulation of coagulation	1	0.63028	0.31	0.019	Biological	GO:00508	negative regulation of coagulation	0.63028	0.74306	0	0	1	54
GO:0055021 regulation of cardiac muscle tissue growth	1	0.63028	0.31	0.019	Biological	GO:00550	regulation of cardiac muscle tissue growth	0.63028	0.74306	0	0	1	54
GO:0061178 regulation of insulin secretion involved in cellular response	1	0.63028	0.31	0.019	Biological	GO:00611	regulation of insulin secretion involved in cellular response to glucose	0.63028	0.74306	0	0	1	54
GO:1902653 secondary alcohol biosynthetic process	1	0.63028	0.31	0.019	Biological	GO:19026	secondary alcohol biosynthetic process	0.63028	0.74306	0	0	1	54
GO:0005769 early endosome	7	0.6308	2.077	0.017	Cellular C	GO:00057	early endosome	0.6308	0.79061	0	0	7	418
GO:0031967 organelle envelope	22	0.63101	6.528	0.017	Cellular C	GO:00319	organelle envelope	0.63101	0.79061	0	0	22	1285
GO:0031975 envelope	22	0.63101	6.528	0.017	Cellular C	GO:00319	envelope	0.63101	0.79061	0	0	22	1285
GO:0097367 carbohydrate derivative binding	41	0.63203	11.988	0.018	Molecular	GO:00973	carbohydrate derivative binding	0.63203	0.75287	0	0	41	2301
GO:1904724 tertiary granule lumen	1	0.63282	0.297	0.018	Cellular C	GO:19047	tertiary granule lumen	0.63282	0.79102	0	0	1	55
GO:0035725 sodium ion transmembrane transport	3	0.63303	0.929	0.017	Biological	GO:00357	sodium ion transmembrane transport	0.63303	0.74595	0	0	3	178
GO:0051650 establishment of vesicle localization	3	0.63303	0.929	0.017	Biological	GO:00516	establishment of vesicle localization	0.63303	0.74595	0	0	3	178
GO:0042593 glucose homeostasis	4	0.63356	1.238	0.017	Biological	GO:00425	glucose homeostasis	0.63356	0.74641	0	0	4	238
GO:0008374 O-acyltransferase activity	1	0.63641	0.292	0.019	Molecular	GO:00083	O-acyltransferase activity	0.63641	0.75632	0	0	1	54
GO:0042626 ATPase-coupled transmembrane transporter activity	2	0.63667	0.585	0.017	Molecular	GO:00426	ATPase-coupled transmembrane transporter activity	0.63667	0.75632	0	0	2	116
GO:0033500 carbohydrate homeostasis	4	0.63683	1.238	0.017	Biological	GO:00335	carbohydrate homeostasis	0.63683	0.74733	0	0	4	239
GO:0032649 regulation of interferon-gamma production	2	0.63697	0.619	0.017	Biological	GO:00326	regulation of interferon-gamma production	0.63697	0.74733	0	0	2	118
GO:0009395 phospholipid catabolic process	1	0.63704	0.31	0.018	Biological	GO:00093	phospholipid catabolic process	0.63704	0.74733	0	0	1	55
GO:0010518 positive regulation of phospholipase activity	1	0.63704	0.31	0.018	Biological	GO:00105	positive regulation of phospholipase activity	0.63704	0.74733	0	0	1	55
GO:0030520 intracellular estrogen receptor signaling pathway	1	0.63704	0.31	0.018	Biological	GO:00305	intracellular estrogen receptor signaling pathway	0.63704	0.74733	0	0	1	55
GO:0031113 regulation of microtubule polymerization	1	0.63704	0.31	0.018	Biological	GO:00311	regulation of microtubule polymerization	0.63704	0.74733	0	0	1	55
GO:0033047 regulation of mitotic sister chromatid segregation	1	0.63704	0.31	0.018	Biological	GO:00330	regulation of mitotic sister chromatid segregation	0.63704	0.74733	0	0	1	55
GO:0035036 sperm-egg recognition	1	0.63704	0.31	0.018	Biological	GO:00350	sperm-egg recognition	0.63704	0.74733	0	0	1	55
GO:0042572 retinol metabolic process	1	0.63704	0.31	0.018	Biological	GO:00425	retinol metabolic process	0.63704	0.74733	0	0	1	55
GO:0045071 negative regulation of viral genome replication	1	0.63704	0.31	0.018	Biological	GO:00450	negative regulation of viral genome replication	0.63704	0.74733	0	0	1	55
GO:0048010 vascular endothelial growth factor receptor signaling pathw	1	0.63704	0.31	0.018	Biological	GO:00480	vascular endothelial growth factor receptor signaling pathway	0.63704	0.74733	0	0	1	55
GO:0050982 detection of mechanical stimulus	1	0.63704	0.31	0.018	Biological	GO:00509	detection of mechanical stimulus	0.63704	0.74733	0	0	1	55
GO:0060998 regulation of dendritic spine development	1	0.63704	0.31	0.018	Biological	GO:00609	regulation of dendritic spine development	0.63704	0.74733	0	0	1	55
GO:0065002 intracellular protein transmembrane transport	1	0.63704	0.31	0.018	Biological	GO:00650	intracellular protein transmembrane transport	0.63704	0.74733	0	0	1	55
GO:0071320 cellular response to cAMP	1	0.63704	0.31	0.018	Biological	GO:00713	cellular response to cAMP	0.63704	0.74733	0	0	1	55
GO:0072577 endothelial cell apoptotic process	1	0.63704	0.31	0.018	Biological	GO:00725	endothelial cell apoptotic process	0.63704	0.74733	0	0	1	55
GO:0097479 synaptic vesicle localization	1	0.63704	0.31	0.018	Biological	GO:00974	synaptic vesicle localization	0.63704	0.74733	0	0	1	55
GO:2000107 negative regulation of leukocyte apoptotic process	1	0.63704	0.31	0.018	Biological	GO:20001	negative regulation of leukocyte apoptotic process	0.63704	0.74733	0	0	1	55
GO:0033554 cellular response to stress	34	0.63724	10.526	0.017	Biological	GO:00335	cellular response to stress	0.63724	0.74739	0	0	34	1951
GO:0002274 myeloid leukocyte activation	4	0.64008	1.238	0.017	Biological	GO:00022	myeloid leukocyte activation	0.64008	0.75054	0	0	4	240
GO:0006886 intracellular protein transport	15	0.64125	4.644	0.017	Biological	GO:00068	intracellular protein transport	0.64125	0.75154	0	0	15	879
GO:0019751 polyol metabolic process	2	0.64154	0.619	0.017	Biological	GO:00197	polyol metabolic process	0.64154	0.75154	0	0	2	119
GO:0032609 interferon-gamma production	2	0.64154	0.619	0.017	Biological	GO:00326	interferon-gamma production	0.64154	0.75154	0	0	2	119
GO:0097237 cellular response to toxic substance	2	0.64154	0.619	0.017	Biological	GO:00972	cellular response to toxic substance	0.64154	0.75154	0	0	2	119
GO:0008146 sulfotransferase activity	1	0.64317	0.292	0.018	Molecular	GO:00081	sulfotransferase activity	0.64317	0.76091	0	0	1	55
GO:0016860 intramolecular oxidoreductase activity	1	0.64317	0.292	0.018	Molecular	GO:00168	intramolecular oxidoreductase activity	0.64317	0.76091	0	0	1	55
GO:0019212 phosphatase inhibitor activity	1	0.64317	0.292	0.018	Molecular	GO:00192	phosphatase inhibitor activity	0.64317	0.76091	0	0	1	55
GO:0001836 release of cytochrome c from mitochondria	1	0.64368	0.31	0.018	Biological	GO:00018	release of cytochrome c from mitochondria	0.64368	0.75352	0	0	1	56
GO:0006826 iron ion transport	1	0.64368	0.31	0.018	Biological	GO:00068	iron ion transport	0.64368	0.75352	0	0	1	56
GO:0050885 neuromuscular process controlling balance	1	0.64368	0.31	0.018	Biological	GO:00508	neuromuscular process controlling balance	0.64368	0.75352	0	0	1	56
GO:0016874 ligase activity	3	0.6441	0.877	0.017	Molecular	GO:00168	ligase activity	0.6441	0.76097	0	0	3	178
GO:0002460 adaptive immune response based on somatic recombinatio	6	0.64438	1.858	0.017	Biological	GO:00024	adaptive immune response based on somatic recombination of immu	0.64438	0.75416	0	0	6	360
GO:0098542 defense response to other organism	20	0.64453	6.192	0.017	Biological	GO:00985	defense response to other organism	0.64453	0.75417	0	0	20	1166
GO:0008287 protein serine/threonine phosphatase complex	1	0.64597	0.297	0.018	Cellular C	GO:00082	protein serine/threonine phosphatase complex	0.64597	0.80385	0	0	1	57
GO:0002065 columnar/cuboidal epithelial cell differentiation	2	0.64606	0.619	0.017	Biological	GO:00020	columnar/cuboidal epithelial cell differentiation	0.64606	0.75546	0	0	2	120
GO:0006821 chloride transport	2	0.64606	0.619	0.017	Biological	GO:00068	chloride transport	0.64606	0.75546	0	0	2	120
GO:0019752 carboxylic acid metabolic process	16	0.64609	4.954	0.017	Biological	GO:00197	carboxylic acid metabolic process	0.64609	0.75546	0	0	16	939
GO:0023061 signal release	8	0.64643	2.477	0.017	Biological	GO:00230	signal release	0.64643	0.75569	0	0	8	478
GO:0110165 cellular anatomical entity	335	0.64688	99.407	0.018	Cellular C	GO:01101	cellular anatomical entity	0.64688	0.80385	0	0	335	18579
GO:0030217 T cell differentiation	5	0.6475	1.548	0.017	Biological	GO:00302	T cell differentiation	0.6475	0.75671	0	0	5	302
GO:0031901 early endosome membrane	3	0.64759	0.89	0.016	Cellular C	GO:00319	early endosome membrane	0.64759	0.80385	0	0	3	184
GO:0006109 regulation of carbohydrate metabolic process	3	0.64792	0.929	0.016	Biological	GO:00061	regulation of carbohydrate metabolic process	0.64792	0.75671	0	0	3	182
GO:0042129 regulation of T cell proliferation	3	0.64792	0.929	0.016	Biological	GO:00421	regulation of T cell proliferation	0.64792	0.75671	0	0	3	182
GO:0043484 regulation of RNA splicing	3	0.64792	0.929	0.016	Biological	GO:00434	regulation of RNA splicing	0.64792	0.75671	0	0	3	182
GO:0060627 regulation of vesicle-mediated transport	9	0.64815	2.786	0.017	Biological	GO:00606	regulation of vesicle-mediated transport	0.64815	0.75681	0	0	9	537
GO:0032944 regulation of mononuclear cell proliferation	4	0.64971	1.238	0.016	Biological	GO:00329	regulation of mononuclear cell proliferation	0.64971	0.75742	0	0	4	243
GO:0001706 endoderm formation	1	0.65019	0.31	0.018	Biological	GO:00017	endoderm formation	0.65019	0.75742	0	0	1	57
GO:0002381 immunoglobulin production involved in immunoglobulin-me	1	0.65019	0.31	0.018	Biological	GO:00023	immunoglobulin production involved in immunoglobulin-mediated imm	0.65019	0.75742	0	0	1	57
GO:0002534 cytokine production involved in inflammatory response	1	0.65019	0.31	0.018	Biological	GO:00025	cytokine production involved in inflammatory response	0.65019	0.75742	0	0	1	57
GO:0009116 nucleoside metabolic process	1	0.65019	0.31	0.018	Biological	GO:00091	nucleoside metabolic process	0.65019	0.75742	0	0	1	57
GO:0045428 regulation of nitric oxide biosynthetic process	1	0.65019	0.31	0.018	Biological	GO:00454	regulation of nitric oxide biosynthetic process	0.65019	0.75742	0	0	1	57
GO:0048016 inositol phosphate-mediated signaling	1	0.65019	0.31	0.018	Biological	GO:00480	inositol phosphate-mediated signaling	0.65019	0.75742	0	0	1	57
GO:0090329 regulation of DNA-dependent DNA replication	1	0.65019	0.31	0.018	Biological	GO:00903	regulation of DNA-dependent DNA replication	0.65019	0.75742	0	0	1	57
GO:0098900 regulation of action potential	1	0.65019	0.31	0.018	Biological	GO:00989	regulation of action potential	0.65019	0.75742	0	0	1	57
GO:1900015 regulation of cytokine production involved in inflammatory r	1	0.65019	0.31	0.018	Biological	GO:19000	regulation of cytokine production involved in inflammatory response	0.65019	0.75742	0	0	1	57
GO:0031490 chromatin DNA binding	2	0.65048	0.585	0.017	Molecular	GO:00314	chromatin DNA binding	0.65048	0.76746	0	0	2	119
GO:0090630 activation of GTPase activity	2	0.65054	0.619	0.017	Biological	GO:00906	activation of GTPase activity	0.65054	0.75765	0	0	2	121
GO:1903131 mononuclear cell differentiation	8	0.65103	2.477	0.017	Biological	GO:19031	mononuclear cell differentiation	0.65103	0.75795	0	0	8	480
GO:0033365 protein localization to organelle	16	0.65111	4.954	0.017	Biological	GO:00333	protein localization to organelle	0.65111	0.75795	0	0	16	942
GO:0031526 brush border membrane	1	0.65237	0.297	0.017	Cellular C	GO:00315	brush border membrane	0.65237	0.80605	0	0	1	58
GO:0098636 protein complex involved in cell adhesion	1	0.65237	0.297	0.017	Cellular C	GO:00986	protein complex involved in cell adhesion	0.65237	0.80605	0	0	1	58
GO:0033865 nucleoside bisphosphate metabolic process	2	0.65498	0.619	0.016	Biological	GO:00338	nucleoside bisphosphate metabolic process	0.65498	0.76193	0	0	2	122
GO:0033875 ribonucleoside bisphosphate metabolic process	2	0.65498	0.619	0.016	Biological	GO:00338	ribonucleoside bisphosphate metabolic process	0.65498	0.76193	0	0	2	122
GO:0034032 purine nucleoside bisphosphate metabolic process	2	0.65498	0.619	0.016	Biological	GO:00340	purine nucleoside bisphosphate metabolic process	0.65498	0.76193	0	0	2	122
GO:0005975 carbohydrate metabolic process	10	0.65631	3.096	0.017	Biological	GO:00059	carbohydrate metabolic process	0.65631	0.76203	0	0	10	599
GO:0008408 3'-5' exonuclease activity	1	0.65631	0.292	0.018	Molecular	GO:00084	3'-5' exonuclease activity	0.65631	0.77127	0	0	1	57
GO:0030374 nuclear receptor coactivator activity	1	0.65631	0.292	0.018	Molecular	GO:00303	nuclear receptor coactivator activity	0.65631	0.77127	0	0	1	57
GO:0043168 anion binding	43	0.65639	12.573	0.018	Molecular	GO:00431	anion binding	0.65639	0.77127	0	0	43	2433
GO:0007080 mitotic metaphase plate congression	1	0.65659	0.31	0.017	Biological	GO:00070	mitotic metaphase plate congression	0.65659	0.76203	0	0	1	58
GO:0033866 nucleoside bisphosphate biosynthetic process	1	0.65659	0.31	0.017	Biological	GO:00338	nucleoside bisphosphate biosynthetic process	0.65659	0.76203	0	0	1	58
GO:0034030 ribonucleoside bisphosphate biosynthetic process	1	0.65659	0.31	0.017	Biological	GO:00340	ribonucleoside bisphosphate biosynthetic process	0.65659	0.76203	0	0	1	58
GO:0034033 purine nucleoside bisphosphate biosynthetic process	1	0.65659	0.31	0.017	Biological	GO:00340	purine nucleoside bisphosphate biosynthetic process	0.65659	0.76203	0	0	1	58
GO:0035088 establishment or maintenance of apical/basal cell polarity	1	0.65659	0.31	0.017	Biological	GO:00350	establishment or maintenance of apical/basal cell polarity	0.65659	0.76203	0	0	1	58
GO:0048008 platelet-derived growth factor receptor signaling pathway	1	0.65659	0.31	0.017	Biological	GO:00480	platelet-derived growth factor receptor signaling pathway	0.65659	0.76203	0	0	1	58

GO:0051932 synaptic transmission, GABAergic	1	0.65659	0.31	0.017	Biological	GO:00519	synaptic transmission, GABAergic	0.65659	0.76203	0	0	1	58
GO:0061245 establishment or maintenance of bipolar cell polarity	1	0.65659	0.31	0.017	Biological	GO:00612	establishment or maintenance of bipolar cell polarity	0.65659	0.76203	0	0	1	58
GO:0070534 protein K63-linked ubiquitination	1	0.65659	0.31	0.017	Biological	GO:00705	protein K63-linked ubiquitination	0.65659	0.76203	0	0	1	58
GO:1904813 ficolin-1-rich granule lumen	2	0.65757	0.593	0.016	Cellular C	GO:19048	ficolin-1-rich granule lumen	0.65757	0.80771	0	0	2	124
GO:0001533 cornified envelope	1	0.65866	0.297	0.017	Cellular C	GO:00015	cornified envelope	0.65866	0.80771	0	0	1	59
GO:1903293 phosphatase complex	1	0.65866	0.297	0.017	Cellular C	GO:19032	phosphatase complex	0.65866	0.80771	0	0	1	59
GO:0002833 positive regulation of response to biotic stimulus	3	0.6588	0.929	0.016	Biological	GO:00028	positive regulation of response to biotic stimulus	0.6588	0.76441	0	0	3	185
GO:0015399 primary active transmembrane transporter activity	3	0.65891	0.877	0.016	Molecular	GO:00153	primary active transmembrane transporter activity	0.65891	0.77214	0	0	3	182
GO:1901981 phosphatidylinositol phosphate binding	3	0.65891	0.877	0.016	Molecular	GO:19019	phosphatidylinositol phosphate binding	0.65891	0.77214	0	0	3	182
GO:0071346 cellular response to interferon-gamma	2	0.65937	0.619	0.016	Biological	GO:00713	cellular response to interferon-gamma	0.65937	0.76489	0	0	2	123
GO:2000112 regulation of cellular macromolecule biosynthetic process	70	0.66121	21.672	0.018	Biological	GO:2000	regulation of cellular macromolecule biosynthetic process	0.66121	0.76685	0	0	70	3986
GO:0030672 synaptic vesicle membrane	2	0.66189	0.593	0.016	Cellular C	GO:00306	synaptic vesicle membrane	0.66189	0.80771	0	0	2	125
GO:0099501 exocytic vesicle membrane	2	0.66189	0.593	0.016	Cellular C	GO:00995	exocytic vesicle membrane	0.66189	0.80771	0	0	2	125
GO:0034612 response to tumor necrosis factor	4	0.66228	1.238	0.016	Biological	GO:00346	response to tumor necrosis factor	0.66228	0.767	0	0	4	247
GO:0046395 carboxylic acid catabolic process	4	0.66228	1.238	0.016	Biological	GO:00463	carboxylic acid catabolic process	0.66228	0.767	0	0	4	247
GO:0002221 pattern recognition receptor signaling pathway	3	0.66237	0.929	0.016	Biological	GO:00022	pattern recognition receptor signaling pathway	0.66237	0.767	0	0	3	186
GO:0045309 protein phosphorylated amino acid binding	1	0.6627	0.292	0.017	Molecular	GO:00453	protein phosphorylated amino acid binding	0.6627	0.77554	0	0	1	58
GO:0031514 motile cilium	4	0.66278	1.187	0.016	Cellular C	GO:00315	motile cilium	0.66278	0.80771	0	0	4	250
GO:0002011 morphogenesis of an epithelial sheet	1	0.66287	0.31	0.017	Biological	GO:00020	morphogenesis of an epithelial sheet	0.66287	0.767	0	0	1	59
GO:0032387 negative regulation of intracellular transport	1	0.66287	0.31	0.017	Biological	GO:00323	negative regulation of intracellular transport	0.66287	0.767	0	0	1	59
GO:0034204 lipid translocation	1	0.66287	0.31	0.017	Biological	GO:00342	lipid translocation	0.66287	0.767	0	0	1	59
GO:0045604 regulation of epidermal cell differentiation	1	0.66287	0.31	0.017	Biological	GO:00456	regulation of epidermal cell differentiation	0.66287	0.767	0	0	1	59
GO:0045620 negative regulation of lymphocyte differentiation	1	0.66287	0.31	0.017	Biological	GO:00456	negative regulation of lymphocyte differentiation	0.66287	0.767	0	0	1	59
GO:0051058 negative regulation of small GTPase mediated signal trans	1	0.66287	0.31	0.017	Biological	GO:00510	negative regulation of small GTPase mediated signal transduction	0.66287	0.767	0	0	1	59
GO:1902808 positive regulation of cell cycle G1/S phase transition	1	0.66287	0.31	0.017	Biological	GO:19028	positive regulation of cell cycle G1/S phase transition	0.66287	0.767	0	0	1	59
GO:0034763 negative regulation of transmembrane transport	2	0.66371	0.619	0.016	Biological	GO:00347	negative regulation of transmembrane transport	0.66371	0.76762	0	0	2	124
GO:0072655 establishment of protein localization to mitochondrion	2	0.66371	0.619	0.016	Biological	GO:00726	establishment of protein localization to mitochondrion	0.66371	0.76762	0	0	2	124
GO:0009749 response to glucose	3	0.66592	0.929	0.016	Biological	GO:00097	response to glucose	0.66592	0.76981	0	0	3	187
GO:0043112 receptor metabolic process	3	0.66592	0.929	0.016	Biological	GO:00431	receptor metabolic process	0.66592	0.76981	0	0	3	187
GO:0071375 cellular response to peptide hormone stimulus	5	0.66725	1.548	0.016	Biological	GO:00713	cellular response to peptide hormone stimulus	0.66725	0.77117	0	0	5	309
GO:0019208 phosphatase regulator activity	2	0.66823	0.585	0.016	Molecular	GO:00192	phosphatase regulator activity	0.66823	0.77972	0	0	2	123
GO:0004536 deoxyribonuclease activity	1	0.66897	0.292	0.017	Molecular	GO:00045	deoxyribonuclease activity	0.66897	0.77972	0	0	1	59
GO:0016765 transferase activity, transferring alkyl or aryl (other than m	1	0.66897	0.292	0.017	Molecular	GO:00167	transferase activity, transferring alkyl or aryl (other than methyl) grou	0.66897	0.77972	0	0	1	59
GO:0009948 anterior/posterior axis specification	1	0.66904	0.31	0.017	Biological	GO:00099	anterior/posterior axis specification	0.66904	0.77164	0	0	1	60
GO:0032613 interleukin-10 production	1	0.66904	0.31	0.017	Biological	GO:00326	interleukin-10 production	0.66904	0.77164	0	0	1	60
GO:0032653 regulation of interleukin-10 production	1	0.66904	0.31	0.017	Biological	GO:00326	regulation of interleukin-10 production	0.66904	0.77164	0	0	1	60
GO:0038202 TORC1 signaling	1	0.66904	0.31	0.017	Biological	GO:00382	TORC1 signaling	0.66904	0.77164	0	0	1	60
GO:0080164 regulation of nitric oxide metabolic process	1	0.66904	0.31	0.017	Biological	GO:00801	regulation of nitric oxide metabolic process	0.66904	0.77164	0	0	1	60
GO:0086065 cell communication involved in cardiac conduction	1	0.66904	0.31	0.017	Biological	GO:00860	cell communication involved in cardiac conduction	0.66904	0.77164	0	0	1	60
GO:0090303 positive regulation of wound healing	1	0.66904	0.31	0.017	Biological	GO:00903	positive regulation of wound healing	0.66904	0.77164	0	0	1	60
GO:0097352 autophagosome maturation	1	0.66904	0.31	0.017	Biological	GO:00973	autophagosome maturation	0.66904	0.77164	0	0	1	60
GO:2001244 positive regulation of intrinsic apoptotic signaling pathway	1	0.66904	0.31	0.017	Biological	GO:20012	positive regulation of intrinsic apoptotic signaling pathway	0.66904	0.77164	0	0	1	60
GO:0006650 glycerophospholipid metabolic process	5	0.67001	1.548	0.016	Biological	GO:00066	glycerophospholipid metabolic process	0.67001	0.77257	0	0	5	310
GO:0002790 peptide secretion	4	0.67151	1.238	0.016	Biological	GO:00027	peptide secretion	0.67151	0.77413	0	0	4	250
GO:0032559 adenyl ribonucleotide binding	27	0.67211	7.895	0.017	Molecular	GO:00325	adenyl ribonucleotide binding	0.67211	0.78224	0	0	27	1558
GO:0019079 viral genome replication	2	0.67227	0.619	0.016	Biological	GO:00190	viral genome replication	0.67227	0.77482	0	0	2	126
GO:0046982 protein heterodimerization activity	6	0.67294	1.754	0.016	Molecular	GO:00469	protein heterodimerization activity	0.67294	0.78224	0	0	6	365
GO:1990351 transporter complex	7	0.67401	2.077	0.016	Cellular C	GO:19903	transporter complex	0.67401	0.81954	0	0	7	436
GO:0016054 organic acid catabolic process	4	0.67455	1.238	0.016	Biological	GO:00160	organic acid catabolic process	0.67455	0.77611	0	0	4	251
GO:0045017 glycerolipid biosynthetic process	4	0.67455	1.238	0.016	Biological	GO:00450	glycerolipid biosynthetic process	0.67455	0.77611	0	0	4	251
GO:0016126 sterol biosynthetic process	1	0.67509	0.31	0.016	Biological	GO:00161	sterol biosynthetic process	0.67509	0.77611	0	0	1	61
GO:0016447 somatic recombination of immunoglobulin gene segments	1	0.67509	0.31	0.016	Biological	GO:00164	somatic recombination of immunoglobulin gene segments	0.67509	0.77611	0	0	1	61
GO:0042306 regulation of protein import into nucleus	1	0.67509	0.31	0.016	Biological	GO:00423	regulation of protein import into nucleus	0.67509	0.77611	0	0	1	61
GO:0042733 embryonic digit morphogenesis	1	0.67509	0.31	0.016	Biological	GO:00427	embryonic digit morphogenesis	0.67509	0.77611	0	0	1	61
GO:0044788 modulation by host of viral process	1	0.67509	0.31	0.016	Biological	GO:00447	modulation by host of viral process	0.67509	0.77611	0	0	1	61
GO:0046824 positive regulation of nucleocytoplasmic transport	1	0.67509	0.31	0.016	Biological	GO:00468	positive regulation of nucleocytoplasmic transport	0.67509	0.77611	0	0	1	61
GO:0051785 positive regulation of nuclear division	1	0.67509	0.31	0.016	Biological	GO:00517	positive regulation of nuclear division	0.67509	0.77611	0	0	1	61
GO:0060420 regulation of heart growth	1	0.67509	0.31	0.016	Biological	GO:00604	regulation of heart growth	0.67509	0.77611	0	0	1	61
GO:0070265 necrotic cell death	1	0.67509	0.31	0.016	Biological	GO:00702	necrotic cell death	0.67509	0.77611	0	0	1	61
GO:0046879 hormone secretion	5	0.67549	1.548	0.016	Biological	GO:00468	hormone secretion	0.67549	0.77638	0	0	5	312
GO:0016570 histone modification	8	0.6757	2.477	0.016	Biological	GO:00165	histone modification	0.6757	0.77645	0	0	8	491
GO:0098661 inorganic anion transmembrane transport	2	0.67648	0.619	0.016	Biological	GO:00986	inorganic anion transmembrane transport	0.67648	0.77698	0	0	2	127
GO:1904950 negative regulation of establishment of protein localization	2	0.67648	0.619	0.016	Biological	GO:19049	negative regulation of establishment of protein localization	0.67648	0.77698	0	0	2	127
GO:0005267 potassium channel activity	2	0.67683	0.585	0.016	Molecular	GO:00052	potassium channel activity	0.67683	0.78571	0	0	2	125
GO:0002697 regulation of immune effector process	6	0.67781	1.858	0.016	Biological	GO:00026	regulation of immune effector process	0.67781	0.77803	0	0	6	373
GO:0050727 regulation of inflammatory response	6	0.67781	1.858	0.016	Biological	GO:00507	regulation of inflammatory response	0.67781	0.77803	0	0	6	373
GO:0031326 regulation of cellular biosynthetic process	73	0.67786	22.601	0.017	Biological	GO:00313	regulation of cellular biosynthetic process	0.67786	0.77803	0	0	73	4172
GO:0034504 protein localization to nucleus	5	0.6782	1.548	0.016	Biological	GO:00345	protein localization to nucleus	0.6782	0.77825	0	0	5	313
GO:0022408 negative regulation of cell-cell adhesion	3	0.67983	0.929	0.016	Biological	GO:00224	negative regulation of cell-cell adhesion	0.67983	0.77953	0	0	3	191
GO:0007041 lysosomal transport	2	0.68065	0.619	0.016	Biological	GO:00070	lysosomal transport	0.68065	0.77953	0	0	2	128
GO:0071333 cellular response to glucose stimulus	2	0.68065	0.619	0.016	Biological	GO:00713	cellular response to glucose stimulus	0.68065	0.77953	0	0	2	128
GO:0032943 mononuclear cell proliferation	5	0.6809	1.548	0.016	Biological	GO:00329	mononuclear cell proliferation	0.6809	0.77953	0	0	5	314
GO:0019229 regulation of vasoconstriction	1	0.68104	0.31	0.016	Biological	GO:00192	regulation of vasoconstriction	0.68104	0.77953	0	0	1	62
GO:0031122 cytoplasmic microtubule organization	1	0.68104	0.31	0.016	Biological	GO:00311	cytoplasmic microtubule organization	0.68104	0.77953	0	0	1	62
GO:0032615 interleukin-12 production	1	0.68104	0.31	0.016	Biological	GO:00326	interleukin-12 production	0.68104	0.77953	0	0	1	62
GO:0032655 regulation of interleukin-12 production	1	0.68104	0.31	0.016	Biological	GO:00326	regulation of interleukin-12 production	0.68104	0.77953	0	0	1	62
GO:0048255 mRNA stabilization	1	0.68104	0.31	0.016	Biological	GO:00482	mRNA stabilization	0.68104	0.77953	0	0	1	62
GO:0050994 regulation of lipid catabolic process	1	0.68104	0.31	0.016	Biological	GO:00509	regulation of lipid catabolic process	0.68104	0.77953	0	0	1	62
GO:0072666 establishment of protein localization to vacuole	1	0.68104	0.31	0.016	Biological	GO:00726	establishment of protein localization to vacuole	0.68104	0.77953	0	0	1	62
GO:0001786 phosphatidylserine binding	1	0.68117	0.292	0.016	Molecular	GO:00017	phosphatidylserine binding	0.68117	0.78863	0	0	1	61
GO:0005507 copper ion binding	1	0.68117	0.292	0.016	Molecular	GO:00055	copper ion binding	0.68117	0.78863	0	0	1	61
GO:0043436 oxoacid metabolic process	16	0.68202	4.954	0.017	Biological	GO:00434	oxoacid metabolic process	0.68202	0.78048	0	0	16	961
GO:0002819 regulation of adaptive immune response	3	0.68324	0.929	0.016	Biological	GO:00028	regulation of adaptive immune response	0.68324	0.78135	0	0	3	192
GO:0009746 response to hexose	3	0.68324	0.929	0.016	Biological	GO:00097	response to hexose	0.68324	0.78135	0	0	3	192
GO:0009889 regulation of biosynthetic process	74	0.68325	22.91	0.017	Biological	GO:00098	regulation of biosynthetic process	0.68325	0.78135	0	0	74	4234
GO:0002699 positive regulation of immune effector process	4	0.68354	1.238	0.016	Biological	GO:00026	positive regulation of immune effector process	0.68354	0.78138	0	0	4	254
GO:0006909 phagocytosis	5	0.68359	1.548	0.016	Biological	GO:00069	phagocytosis	0.68359	0.78138	0	0	5	315
GO:0006911 phagocytosis, engulfment	2	0.68477	0.619	0.016	Biological	GO:00069	phagocytosis, engulfment	0.68477	0.78237	0	0	2	129
GO:0031109 microtubule polymerization or depolymerization	2	0.68477	0.619	0.016	Biological	GO:00311	microtubule polymerization or depolymerization	0.68477	0.78237	0	0	2	129
GO:0005179 hormone activity	2	0.68525	0.585	0.016	Molecular	GO:00051	hormone activity	0.68525	0.79229	0	0	2	127
GO:0030554 adenyl nucleotide binding	27	0.68624	7.895	0.017	Molecular	GO:00305	adenyl nucleotide binding	0.68624	0.79231	0	0	27	1569
GO:0010556 regulation of macromolecule biosynthetic process	70	0.68667	21.672	0.016	Biological	GO:00105	regulation of macromolecule biosynthetic process	0.68667	0.78334	0	0	70	4015
GO:0002090 regulation of receptor internalization	1	0.68687	0.31	0.016	Biological	GO:00020	regulation of receptor internalization	0.68687	0.78334	0	0	1	63
GO:0032233 positive regulation of actin filament bundle assembly	1	0.68687	0.31	0.016	Biological	GO:00322	positive regulation of actin filament bundle assembly	0.68687	0.78334	0	0	1	63
GO:0032623 interleukin-2 production	1	0.68687	0.31	0.016	Biological	GO:00326	interleukin-2 production	0.68687	0.78334	0	0	1	63
GO:0032663 regulation of interleukin-2 production	1	0.68687	0.31	0.016	Biological	GO:00326	regulation of interleukin-2 production	0.68687	0.78334	0	0	1	63
GO:0035924 cellular response to vascular endothelial growth factor stim	1	0.68687	0.31	0.016	Biological	GO:00359	cellular response to vascular endothelial growth factor stimulus	0.68687	0.78334	0	0	1	63
GO:0048002 antigen processing and presentation of peptide antigen	1	0.68687	0.31	0.016	Biological	GO:00480	antigen processing and presentation of peptide antigen	0.68687	0.78334	0	0	1	63
GO:0070059 intrinsic apoptotic signaling pathway in response to endopl	1	0.68687	0.31	0.016	Biological	GO:00700	intrinsic apoptotic signaling pathway in response to endoplasmic retic	0.68687	0.78334	0	0	1	63
GO:0016706 2-oxoglutarate-dependent dioxygenase activity	1	0.6871	0.292	0.016	Molecular	GO:00167	2-oxoglutarate-dependent dioxygenase activity	0.6871	0.79231	0	0	1	62
GO:0031929 TOR signaling	2	0.68885	0.619	0.015	Biological	GO:00319	TOR signaling	0.68885	0.78488	0	0	2	130
GO:0070585 protein localization to mitochondrion	2	0.68885	0.619	0.015	Biological	GO:00705	protein localization to mitochondrion	0.68885	0.78488	0	0	2	130
GO:0071331 cellular response to hexose stimulus	2	0.68885	0.619	0.015	Biological	GO:00713	cellular response to hexose stimulus	0.68885	0.78488	0	0	2	130
GO:2001235 positive regulation of apoptotic signaling pathway	2	0.68885	0.619	0.015	Biological	GO:20012	positive regulation of apoptotic signaling pathway	0.68885	0.78488	0	0	2	130
GO:0031072 heat shock protein binding	2	0.68939	0.585	0.016	Molecular	GO:00310	heat shock protein binding	0.68939	0.7939	0	0	2	128
GO:0016192 vesicle-mediated transport	28	0.69024	8.669	0.017	Biological	GO:00161	vesicle-mediated transport	0.69024	0.78628	0	0	28	1655
GO:0006303 double-strand break repair via nonhomologous end joining	1	0.6926	0.31	0.016	Biological	GO:00063	double-strand break repair via nonhomologous end joining	0.6926	0.78732	0	0	1	64
GO:0006414 translational elongation	1	0.6926	0.31	0.016	Biological	GO:00064	translational elongation	0.6926	0.78732	0	0	1	64
GO:0035773 insulin secretion involved in cellular response to glucose st	1	0.6926	0.31	0.016	Biological	GO:00357	insulin secretion involved in cellular response to glucose stimulus	0.6926	0.78732	0	0	1	64
GO:0051445 regulation of meiotic cell cycle	1	0.6926	0.31	0.016	Biological	GO:00514	regulation of meiotic cell cycle	0.6926	0.78732	0	0	1	64
GO:0051893 regulation of focal adhesion assembly	1	0.6926	0.31	0.016	Biological	GO:00518	regulation of focal adhesion assembly	0.6926	0.78732	0	0	1	64
GO:0051965 positive regulation of synapse assembly	1	0.6926	0.31	0.016	Biological	GO:00519	positive regulation of synapse assembly	0.6926	0.78732	0	0	1	64
GO:0061951 establishment of protein localization to plasma membrane	1	0.6926	0.31	0.016	Biological	GO:00619	establishment of protein localization to plasma membrane	0.6926	0.78732	0	0	1	64
GO:0090109 regulation of cell-substrate junction assembly	1	0.6926	0.31	0.016	Biological	GO:00901	regulation of cell-substrate junction assembly	0.6926	0.78732	0	0	1	64
GO:0097035 regulation of membrane lipid distribution	1	0.6926	0.31	0.016	Biological	GO:00970	regulation of membrane lipid distribution	0.6926	0.78732	0	0	1	64
GO:0045621 positive regulation of lymphocyte differentiation	2	0.69289	0.619	0.015	Biological	GO:00456	positive regulation of lymphocyte differentiation	0.69289	0.78732	0	0	2	131
GO:0051225 spindle assembly	2	0.69289	0.619	0.015	Biological	GO:00512	spindle assembly	0.69289	0.78732	0	0	2	131
GO:0003677 DNA binding	44	0.69307	12.865	0.017	Molecular	GO:00036	DNA binding	0.69307	0.79708	0	0	44	2523
GO:0045861 negative regulation of proteolysis	5	0.69418	1.548	0.016	Biological	GO:00458	negative regulation of proteolysis	0.69418	0.78861	0	0	5	319
GO:0036094 small molecule binding	44	0.69512	12.865	0.017	Molecular	GO:00360	small molecule binding	0.69512	0.79821	0	0	44	2525
GO:0015291 secondary active transmembrane transporter activity	4	0.6959	1.17	0.016	Molecular	GO:00152	secondary active transmembrane transporter activity	0.6959	0.79821	0	0	4	254
GO:0071326 cellular response to monosaccharide stimulus	2	0.69688	0.619	0.015	Biological	GO:00713	cellular response to monosaccharide stimulus	0.69688	0.79132	0	0	2	132
GO:1903305 regulation of regulated secretory pathway	2	0.69688	0.619	0.015	Biological	GO:19033	regulation of regulated secretory pathway	0.69688	0.79132	0	0	2	132
GO:0010524 positive regulation of calcium ion transport into cytosol	1	0.69822	0.31	0.015	Biological	GO:00105	positive regulation of calcium ion transport into cytosol	0.69822	0.79159	0	0	1	65
GO:0030219 megakaryocyte differentiation	1	0.69822	0.31	0.015	Biological	GO:00302	megakaryocyte differentiation	0.69822	0.79159	0	0	1	65
GO:0031638 zymogen activation	1	0.69822	0.31	0.015	Biological	GO:00316	zymogen activation	0.69822	0.79159	0	0	1	65
GO:0033692 cellular polysaccharide biosynthetic process	1	0.69822	0.31	0.015	Biological	GO:00336	cellular polysaccharide biosynthetic process	0.69822	0.79159	0	0	1	65
GO:0048524 positive regulation of viral process	1	0.69822	0.31	0.015	Biological	GO:00485	positive regulation of viral process	0.69822	0.79159	0	0	1	65
GO:0071384 cellular response to corticosteroid stimulus	1	0.69822	0.31	0.015	Biological	GO:00713	cellular response to corticosteroid stimulus	0.69822	0.79159	0	0	1	65
GO:0099601 regulation of neurotransmitter receptor activity	1	0.69822	0.31	0.015	Biological	GO:00996	regulation of neurotransmitter receptor activity	0.69822	0.79159	0	0	1	65
GO:0019005 SCF ubiquitin ligase complex	1	0.6996	0.297	0.015	Cellular C	GO:00190	SCF ubiquitin ligase complex	0.6996	0.84872	0	0	1	66
GO:0051648 vesicle localization	3	0.69989	0.929	0.015	Biological	GO:00516	vesicle localization	0.69989	0.79329	0	0	3	197
GO:0006807 nitrogen compound metabolic process	173	0.70018	53.56	0.018	Biological	GO:00068	nitrogen compound metabolic process	0.70018	0.79344	0	0	173	9717
GO:0030234 enzyme regulator activity	22	0.70152	6.433	0.017	Molecular	GO:00302	enzyme regulator activity	0.70152	0.80257	0	0	22	1299
GO:0016616 oxidoreductase activity, acting on the CH-OH group of dor	2	0.70155	0.585	0.015	Molecular	GO:00166	oxidoreductase activity, acting on the CH-OH group of donors, NAD	0.70155	0.80257	0	0	2	131
GO:0098739 import across plasma membrane	3	0.70314	0.929	0.015	Biological	GO:00987	import across plasma membrane	0.70314	0.79568	0	0	3	198
GO:0001947 heart looping	1	0.70375	0.31	0.015	Biological	GO:00019	heart looping	0.70375	0.79568	0	0	1	66
GO:0010517 regulation of phospholipase activity	1	0.70375	0.31	0.015	Biological	GO:00105	regulation of phospholipase activity	0.70375	0.79568	0	0	1	66
GO:0030166 proteoglycan biosynthetic process	1	0.70375	0.31	0.015	Biological	GO:00301	proteoglycan biosynthetic process	0.70375	0.79568	0	0	1	66
GO:0043966 histone H3 acetylation	1	0.70375	0.31	0.015	Biological	GO:00439	histone H3 acetylation	0.70375	0.79568	0	0	1	66
GO:0045682 regulation of epidermis development	1	0.70375	0.31	0.015	Biological	GO:00456	regulation of epidermis development	0.70375	0.79568	0	0	1	66
GO:0050709 negative regulation of protein secretion	1	0.70375	0.31	0.015	Biological	GO:00507	negative regulation of protein secretion	0.70375	0.79568	0	0	1	66
GO:0050918 positive chemotaxis	1	0.70375	0.31	0.015	Biological	GO:00509	positive chemotaxis	0.70375	0.79568	0	0	1	66
GO:1902017 regulation of cilium assembly	1	0.70375	0.31	0.015	Biological	GO:19020	regulation of cilium assembly	0.70375	0.79568	0	0	1	66
GO:1904589 regulation of protein import	1	0.70375	0.31	0.015	Biological	GO:19045	regulation of protein import	0.70375	0.79568	0	0	1	66
GO:0022824 transmitter-gated ion channel activity	1	0.70423	0.292	0.015	Molecular	GO:00228	transmitter-gated ion channel activity	0.70423	0.80286	0	0	1	65
GO:0022835 transmitter-gated channel activity	1	0.70423	0.292	0.015	Molecular	GO:00228	transmitter-gated channel activity	0.70423	0.80286	0	0	1	65
GO:0042393 histone binding	4	0.70461	1.17	0.016	Molecular	GO:00423	histone binding	0.70461	0.80286	0	0	4	257
GO:0000502 proteasome complex	1	0.70503	0.297	0.015	Cellular C	GO:00005	proteasome complex	0.70503	0.85337	0	0	1	67
GO:0008242 omega peptidase activity	2	0.70552	0.585	0.015	Molecular	GO:00082	omega peptidase activity	0.70552	0.80286	0	0	2	132
GO:0030832 regulation of actin filament length	3	0.70636	0.929	0.015	Biological	GO:00308	regulation of actin filament length	0.70636	0.79845	0	0	3	199
GO:0009897 external side of plasma membrane	7	0.70753	2.077	0.016	Cellular C	GO:00098	external side of plasma membrane	0.70753	0.85404	0	0	7	451

GO:0002696 positive regulation of leukocyte activation	7	0.70784	2.167	0.016	Biological	GO:0002	positive regulation of leukocyte activation	0.70784	0.79994	0	0	7	446
GO:0030168 platelet activation	2	0.7086	0.619	0.015	Biological	GO:0030	platelet activation	0.7086	0.80018	0	0	2	135
GO:0016445 somatic diversification of immunoglobulins	1	0.70917	0.31	0.015	Biological	GO:0016	somatic diversification of immunoglobulins	0.70917	0.80018	0	0	1	67
GO:0032507 maintenance of protein location in cell	1	0.70917	0.31	0.015	Biological	GO:0032	maintenance of protein location in cell	0.70917	0.80018	0	0	1	67
GO:0035019 somatic stem cell population maintenance	1	0.70917	0.31	0.015	Biological	GO:0035	somatic stem cell population maintenance	0.70917	0.80018	0	0	1	67
GO:0050891 multicellular organismal water homeostasis	1	0.70917	0.31	0.015	Biological	GO:0050	multicellular organismal water homeostasis	0.70917	0.80018	0	0	1	67
GO:0060193 positive regulation of lipase activity	1	0.70917	0.31	0.015	Biological	GO:0060	positive regulation of lipase activity	0.70917	0.80018	0	0	1	67
GO:0070613 regulation of protein processing	1	0.70917	0.31	0.015	Biological	GO:0070	regulation of protein processing	0.70917	0.80018	0	0	1	67
GO:0034284 response to monosaccharide	3	0.70955	0.929	0.015	Biological	GO:0034	response to monosaccharide	0.70955	0.80025	0	0	3	200
GO:0090276 regulation of peptide hormone secretion	3	0.70955	0.929	0.015	Biological	GO:0090	regulation of peptide hormone secretion	0.70955	0.80025	0	0	3	200
GO:0005548 phospholipid transporter activity	1	0.70974	0.292	0.015	Molecular	GO:0005	phospholipid transporter activity	0.70974	0.80449	0	0	1	66
GO:0015453 oxidoreduction-driven active transmembrane transporter a	1	0.70974	0.292	0.015	Molecular	GO:0015	oxidoreduction-driven active transmembrane transporter activity	0.70974	0.80449	0	0	1	66
GO:0097110 scaffold protein binding	1	0.70974	0.292	0.015	Molecular	GO:0097	scaffold protein binding	0.70974	0.80449	0	0	1	66
GO:0005778 peroxisomal membrane	1	0.71037	0.297	0.015	Cellular C	GO:0005	peroxisomal membrane	0.71037	0.85404	0	0	1	68
GO:0031903 microbody membrane	1	0.71037	0.297	0.015	Cellular C	GO:0031	microbody membrane	0.71037	0.85404	0	0	1	68
GO:0006082 organic acid metabolic process	16	0.71135	4.954	0.016	Biological	GO:0006	organic acid metabolic process	0.71135	0.80207	0	0	16	980
GO:0045786 negative regulation of cell cycle	6	0.71149	1.858	0.016	Biological	GO:0045	negative regulation of cell cycle	0.71149	0.80207	0	0	6	387
GO:0005622 intracellular anatomical structure	267	0.71223	79.228	0.018	Cellular C	GO:0005	intracellular anatomical structure	0.71223	0.85435	0	0	267	15006
GO:0006470 protein dephosphorylation	4	0.71226	1.238	0.015	Biological	GO:0006	protein dephosphorylation	0.71226	0.8024	0	0	4	264
GO:0002705 positive regulation of leukocyte mediated immunity	2	0.71243	0.619	0.015	Biological	GO:0002	positive regulation of leukocyte mediated immunity	0.71243	0.8024	0	0	2	136
GO:0007052 mitotic spindle organization	2	0.71243	0.619	0.015	Biological	GO:0007	mitotic spindle organization	0.71243	0.8024	0	0	2	136
GO:0007368 determination of left/right symmetry	2	0.71243	0.619	0.015	Biological	GO:0007	determination of left/right symmetry	0.71243	0.8024	0	0	2	136
GO:0030295 protein kinase activator activity	2	0.71331	0.585	0.015	Molecular	GO:0030	protein kinase activator activity	0.71331	0.80749	0	0	2	134
GO:0015909 long-chain fatty acid transport	1	0.71449	0.31	0.015	Biological	GO:0015	long-chain fatty acid transport	0.71449	0.80346	0	0	1	68
GO:0030837 negative regulation of actin filament polymerization	1	0.71449	0.31	0.015	Biological	GO:0030	negative regulation of actin filament polymerization	0.71449	0.80346	0	0	1	68
GO:0046513 ceramide biosynthetic process	1	0.71449	0.31	0.015	Biological	GO:0046	ceramide biosynthetic process	0.71449	0.80346	0	0	1	68
GO:0046626 regulation of insulin receptor signaling pathway	1	0.71449	0.31	0.015	Biological	GO:0046	regulation of insulin receptor signaling pathway	0.71449	0.80346	0	0	1	68
GO:0046888 negative regulation of hormone secretion	1	0.71449	0.31	0.015	Biological	GO:0046	negative regulation of hormone secretion	0.71449	0.80346	0	0	1	68
GO:1900076 regulation of cellular response to insulin stimulus	1	0.71449	0.31	0.015	Biological	GO:1900	regulation of cellular response to insulin stimulus	0.71449	0.80346	0	0	1	68
GO:2000144 positive regulation of DNA-templated transcription, initiatio	1	0.71449	0.31	0.015	Biological	GO:2000	positive regulation of DNA-templated transcription, initiation	0.71449	0.80346	0	0	1	68
GO:0008080 N-acetyltransferase activity	1	0.71513	0.292	0.015	Molecular	GO:0008	N-acetyltransferase activity	0.71513	0.80849	0	0	1	67
GO:0051249 regulation of lymphocyte activation	9	0.71578	2.786	0.016	Biological	GO:0051	regulation of lymphocyte activation	0.71578	0.80446	0	0	9	570
GO:0006006 glucose metabolic process	3	0.71586	0.929	0.015	Biological	GO:0006	glucose metabolic process	0.71586	0.80446	0	0	3	202
GO:0030073 insulin secretion	3	0.71586	0.929	0.015	Biological	GO:0030	insulin secretion	0.71586	0.80446	0	0	3	202
GO:0032368 regulation of lipid transport	2	0.71621	0.619	0.015	Biological	GO:0032	regulation of lipid transport	0.71621	0.80448	0	0	2	137
GO:0050853 B cell receptor signaling pathway	2	0.71621	0.619	0.015	Biological	GO:0050	B cell receptor signaling pathway	0.71621	0.80448	0	0	2	137
GO:0002548 monocyte chemotaxis	1	0.71971	0.31	0.014	Biological	GO:0002	monocyte chemotaxis	0.71971	0.80687	0	0	1	69
GO:0038034 signal transduction in absence of ligand	1	0.71971	0.31	0.014	Biological	GO:0038	signal transduction in absence of ligand	0.71971	0.80687	0	0	1	69
GO:0050909 sensory perception of taste	1	0.71971	0.31	0.014	Biological	GO:0050	sensory perception of taste	0.71971	0.80687	0	0	1	69
GO:0097192 extrinsic apoptotic signaling pathway in absence of ligand	1	0.71971	0.31	0.014	Biological	GO:0097	extrinsic apoptotic signaling pathway in absence of ligand	0.71971	0.80687	0	0	1	69
GO:0150116 regulation of cell-substrate junction organization	1	0.71971	0.31	0.014	Biological	GO:0150	regulation of cell-substrate junction organization	0.71971	0.80687	0	0	1	69
GO:0006820 anion transport	8	0.71975	2.477	0.016	Biological	GO:0006	anion transport	0.71975	0.80687	0	0	8	512
GO:0008203 cholesterol metabolic process	2	0.71995	0.619	0.014	Biological	GO:0008	cholesterol metabolic process	0.71995	0.80687	0	0	2	138
GO:0030317 flagellated sperm motility	2	0.71995	0.619	0.014	Biological	GO:0030	flagellated sperm motility	0.71995	0.80687	0	0	2	138
GO:0097722 sperm motility	2	0.71995	0.619	0.014	Biological	GO:0097	sperm motility	0.71995	0.80687	0	0	2	138
GO:0099024 plasma membrane invagination	2	0.71995	0.619	0.014	Biological	GO:0099	plasma membrane invagination	0.71995	0.80687	0	0	2	138
GO:0070663 regulation of leukocyte proliferation	4	0.7205	1.238	0.015	Biological	GO:0070	regulation of leukocyte proliferation	0.7205	0.8073	0	0	4	267
GO:0008509 anion transmembrane transporter activity	5	0.72051	1.462	0.015	Molecular	GO:0008	anion transmembrane transporter activity	0.72051	0.81351	0	0	5	324
GO:0045087 innate immune response	15	0.72109	4.644	0.016	Biological	GO:0045	innate immune response	0.72109	0.80779	0	0	15	928
GO:0001669 acrosomal vesicle	2	0.72145	0.593	0.014	Cellular C	GO:0001	acrosomal vesicle	0.72145	0.86348	0	0	2	140
GO:0002244 hematopoietic progenitor cell differentiation	2	0.72364	0.619	0.014	Biological	GO:0002	hematopoietic progenitor cell differentiation	0.72364	0.81047	0	0	2	139
GO:0006305 DNA alkylation	1	0.72484	0.31	0.014	Biological	GO:0006	DNA alkylation	0.72484	0.81054	0	0	1	70
GO:0006306 DNA methylation	1	0.72484	0.31	0.014	Biological	GO:0006	DNA methylation	0.72484	0.81054	0	0	1	70
GO:0045109 intermediate filament organization	1	0.72484	0.31	0.014	Biological	GO:0045	intermediate filament organization	0.72484	0.81054	0	0	1	70
GO:0051881 regulation of mitochondrial membrane potential	1	0.72484	0.31	0.014	Biological	GO:0051	regulation of mitochondrial membrane potential	0.72484	0.81054	0	0	1	70
GO:0060411 cardiac septum morphogenesis	1	0.72484	0.31	0.014	Biological	GO:0060	cardiac septum morphogenesis	0.72484	0.81054	0	0	1	70
GO:0061512 protein localization to cilium	1	0.72484	0.31	0.014	Biological	GO:0061	protein localization to cilium	0.72484	0.81054	0	0	1	70
GO:1903317 regulation of protein maturation	1	0.72484	0.31	0.014	Biological	GO:1903	regulation of protein maturation	0.72484	0.81054	0	0	1	70
GO:1900180 regulation of protein localization to nucleus	2	0.7273	0.619	0.014	Biological	GO:1900	regulation of protein localization to nucleus	0.7273	0.81303	0	0	2	140
GO:0032102 negative regulation of response to external stimulus	6	0.7274	1.858	0.015	Biological	GO:0032	negative regulation of response to external stimulus	0.7274	0.81303	0	0	6	394
GO:0045335 phagocytic vesicle	2	0.72869	0.593	0.014	Cellular C	GO:0045	phagocytic vesicle	0.72869	0.8688	0	0	2	142
GO:0000271 polysaccharide biosynthetic process	1	0.72988	0.31	0.014	Biological	GO:0000	polysaccharide biosynthetic process	0.72988	0.81435	0	0	1	71
GO:0002562 somatic diversification of immune receptors via germline re	1	0.72988	0.31	0.014	Biological	GO:0002	somatic diversification of immune receptors via germline recombinati	0.72988	0.81435	0	0	1	71
GO:0016444 somatic cell DNA recombination	1	0.72988	0.31	0.014	Biological	GO:0016	somatic cell DNA recombination	0.72988	0.81435	0	0	1	71
GO:0032922 circadian regulation of gene expression	1	0.72988	0.31	0.014	Biological	GO:0032	circadian regulation of gene expression	0.72988	0.81435	0	0	1	71
GO:0043489 RNA stabilization	1	0.72988	0.31	0.014	Biological	GO:0043	RNA stabilization	0.72988	0.81435	0	0	1	71
GO:0072678 T cell migration	1	0.72988	0.31	0.014	Biological	GO:0072	T cell migration	0.72988	0.81435	0	0	1	71
GO:1903036 positive regulation of response to wounding	1	0.72988	0.31	0.014	Biological	GO:1903	positive regulation of response to wounding	0.72988	0.81435	0	0	1	71
GO:2000036 regulation of stem cell population maintenance	1	0.72988	0.31	0.014	Biological	GO:2000	regulation of stem cell population maintenance	0.72988	0.81435	0	0	1	71
GO:0019905 syntaxin binding	1	0.73074	0.292	0.014	Molecular	GO:0019	syntaxin binding	0.73074	0.82398	0	0	1	70
GO:0035869 ciliary transition zone	1	0.73077	0.297	0.014	Cellular C	GO:0035	ciliary transition zone	0.73077	0.8688	0	0	1	72
GO:0070821 tertiary granule membrane	1	0.73077	0.297	0.014	Cellular C	GO:0070	tertiary granule membrane	0.73077	0.8688	0	0	1	72
GO:0030258 lipid modification	3	0.73118	0.929	0.014	Biological	GO:0030	lipid modification	0.73118	0.81561	0	0	3	207
GO:0006955 immune response	33	0.73381	10.217	0.017	Biological	GO:0006	immune response	0.73381	0.81785	0	0	33	1979
GO:0007049 cell cycle	30	0.73381	9.288	0.017	Biological	GO:0007	cell cycle	0.73381	0.81785	0	0	30	1807
GO:0050684 regulation of mRNA processing	2	0.73449	0.619	0.014	Biological	GO:0050	regulation of mRNA processing	0.73449	0.81785	0	0	2	142
GO:0003143 embryonic heart tube morphogenesis	1	0.73482	0.31	0.014	Biological	GO:0003	embryonic heart tube morphogenesis	0.73482	0.81785	0	0	1	72
GO:0006024 glycosaminoglycan biosynthetic process	1	0.73482	0.31	0.014	Biological	GO:0006	glycosaminoglycan biosynthetic process	0.73482	0.81785	0	0	1	72
GO:0006809 nitric oxide biosynthetic process	1	0.73482	0.31	0.014	Biological	GO:0006	nitric oxide biosynthetic process	0.73482	0.81785	0	0	1	72
GO:0043627 response to estrogen	1	0.73482	0.31	0.014	Biological	GO:0043	response to estrogen	0.73482	0.81785	0	0	1	72
GO:0050766 positive regulation of phagocytosis	1	0.73482	0.31	0.014	Biological	GO:0050	positive regulation of phagocytosis	0.73482	0.81785	0	0	1	72
GO:0061371 determination of heart left/right asymmetry	1	0.73482	0.31	0.014	Biological	GO:0061	determination of heart left/right asymmetry	0.73482	0.81785	0	0	1	72
GO:1902373 negative regulation of mRNA catabolic process	1	0.73482	0.31	0.014	Biological	GO:1902	negative regulation of mRNA catabolic process	0.73482	0.81785	0	0	1	72
GO:0008234 cysteine-type peptidase activity	3	0.73537	0.877	0.015	Molecular	GO:0008	cysteine-type peptidase activity	0.73537	0.82533	0	0	3	205
GO:0032153 cell division site	1	0.73564	0.297	0.014	Cellular C	GO:0032	cell division site	0.73564	0.87265	0	0	1	73
GO:0019209 kinase activator activity	2	0.73567	0.585	0.014	Molecular	GO:0019	kinase activator activity	0.73567	0.82533	0	0	2	140
GO:0002039 p53 binding	1	0.73575	0.292	0.014	Molecular	GO:0002	p53 binding	0.73575	0.82533	0	0	1	71
GO:0043621 protein self-association	1	0.73575	0.292	0.014	Molecular	GO:0043	protein self-association	0.73575	0.82533	0	0	1	71
GO:0015980 energy derivation by oxidation of organic compounds	5	0.73647	1.548	0.015	Biological	GO:0015	energy derivation by oxidation of organic compounds	0.73647	0.81951	0	0	5	336
GO:1902115 regulation of organelle assembly	3	0.73712	0.929	0.014	Biological	GO:1902	regulation of organelle assembly	0.73712	0.82004	0	0	3	209
GO:0010212 response to ionizing radiation	2	0.73803	0.619	0.014	Biological	GO:0010	response to ionizing radiation	0.73803	0.82069	0	0	2	143
GO:0051384 response to glucocorticoid	2	0.73803	0.619	0.014	Biological	GO:0051	response to glucocorticoid	0.73803	0.82069	0	0	2	143
GO:0016829 lyase activity	3	0.73836	0.877	0.015	Molecular	GO:0016	lyase activity	0.73836	0.8272	0	0	3	206
GO:0002260 lymphocyte homeostasis	1	0.73968	0.31	0.014	Biological	GO:0002	lymphocyte homeostasis	0.73968	0.82088	0	0	1	73
GO:0010822 positive regulation of mitochondrion organization	1	0.73968	0.31	0.014	Biological	GO:0010	positive regulation of mitochondrion organization	0.73968	0.82088	0	0	1	73
GO:0030104 water homeostasis	1	0.73968	0.31	0.014	Biological	GO:0030	water homeostasis	0.73968	0.82088	0	0	1	73
GO:0031640 killing of cells of other organism	1	0.73968	0.31	0.014	Biological	GO:0031	killing of cells of other organism	0.73968	0.82088	0	0	1	73
GO:0033143 regulation of intracellular steroid hormone receptor signalin	1	0.73968	0.31	0.014	Biological	GO:0033	regulation of intracellular steroid hormone receptor signaling pathway	0.73968	0.82088	0	0	1	73
GO:0048488 synaptic vesicle endocytosis	1	0.73968	0.31	0.014	Biological	GO:0048	synaptic vesicle endocytosis	0.73968	0.82088	0	0	1	73
GO:0051298 centrosome duplication	1	0.73968	0.31	0.014	Biological	GO:0051	centrosome duplication	0.73968	0.82088	0	0	1	73
GO:0051310 metaphase plate congression	1	0.73968	0.31	0.014	Biological	GO:0051	metaphase plate congression	0.73968	0.82088	0	0	1	73
GO:0061045 negative regulation of wound healing	1	0.73968	0.31	0.014	Biological	GO:0061	negative regulation of wound healing	0.73968	0.82088	0	0	1	73
GO:0035097 histone methyltransferase complex	1	0.74042	0.297	0.014	Cellular C	GO:0035	histone methyltransferase complex	0.74042	0.87327	0	0	1	74
GO:0044291 cell-cell contact zone	1	0.74042	0.297	0.014	Cellular C	GO:0044	cell-cell contact zone	0.74042	0.87327	0	0	1	74
GO:0016491 oxidoreductase activity	12	0.74066	3.509	0.016	Molecular	GO:0016	oxidoreductase activity	0.74066	0.8287	0	0	12	751
GO:0032273 positive regulation of protein polymerization	2	0.74152	0.619	0.014	Biological	GO:0032	positive regulation of protein polymerization	0.74152	0.82274	0	0	2	144
GO:0046907 intracellular transport	26	0.7421	8.05	0.016	Biological	GO:0046	intracellular transport	0.7421	0.8232	0	0	26	1584
GO:0005777 peroxisome	2	0.74268	0.593	0.014	Cellular C	GO:0005	peroxisome	0.74268	0.87327	0	0	2	146
GO:0042579 microbody	2	0.74268	0.593	0.014	Cellular C	GO:0042	microbody	0.74268	0.87327	0	0	2	146
GO:0015293 symporter activity	2	0.74279	0.585	0.014	Molecular	GO:0015	symporter activity	0.74279	0.83001	0	0	2	142
GO:0045123 cellular extravasation	1	0.74444	0.31	0.014	Biological	GO:0045	cellular extravasation	0.74444	0.8247	0	0	1	74
GO:0046323 glucose import	1	0.74444	0.31	0.014	Biological	GO:0046	glucose import	0.74444	0.8247	0	0	1	74
GO:0070527 platelet aggregation	1	0.74444	0.31	0.014	Biological	GO:0070	platelet aggregation	0.74444	0.8247	0	0	1	74
GO:0070988 demethylation	1	0.74444	0.31	0.014	Biological	GO:0070	demethylation	0.74444	0.8247	0	0	1	74
GO:1903363 negative regulation of cellular protein catabolic process	1	0.74444	0.31	0.014	Biological	GO:1903	negative regulation of cellular protein catabolic process	0.74444	0.8247	0	0	1	74
GO:2000242 negative regulation of reproductive process	1	0.74444	0.31	0.014	Biological	GO:2000	negative regulation of reproductive process	0.74444	0.8247	0	0	1	74
GO:0009855 determination of bilateral symmetry	2	0.74498	0.619	0.014	Biological	GO:0009	determination of bilateral symmetry	0.74498	0.82492	0	0	2	145
GO:0045089 positive regulation of innate immune response	2	0.74498	0.619	0.014	Biological	GO:0045	positive regulation of innate immune response	0.74498	0.82492	0	0	2	145
GO:0016782 transferase activity, transferring sulphur-containing groups	1	0.74549	0.292	0.014	Molecular	GO:0016	transferase activity, transferring sulphur-containing groups	0.74549	0.83081	0	0	1	73
GO:0016811 hydrolase activity, acting on carbon-nitrogen (but not pepti	1	0.74549	0.292	0.014	Molecular	GO:0016	hydrolase activity, acting on carbon-nitrogen (but not peptide) bonds,	0.74549	0.83081	0	0	1	73
GO:0001505 regulation of neurotransmitter levels	3	0.74584	0.929	0.014	Biological	GO:0001	regulation of neurotransmitter levels	0.74584	0.82569	0	0	3	212
GO:0044782 cilium organization	6	0.74694	1.858	0.015	Biological	GO:0044	cilium organization	0.74694	0.82674	0	0	6	403
GO:0009581 detection of external stimulus	2	0.74839	0.619	0.014	Biological	GO:0009	detection of external stimulus	0.74839	0.82713	0	0	2	146
GO:0009799 specification of symmetry	2	0.74839	0.619	0.014	Biological	GO:0009	specification of symmetry	0.74839	0.82713	0	0	2	146
GO:0050729 positive regulation of inflammatory response	2	0.74839	0.619	0.014	Biological	GO:0050	positive regulation of inflammatory response	0.74839	0.82713	0	0	2	146
GO:0051783 regulation of nuclear division	2	0.74839	0.619	0.014	Biological	GO:0051	regulation of nuclear division	0.74839	0.82713	0	0	2	146
GO:1903531 negative regulation of secretion by cell	2	0.74839	0.619	0.014	Biological	GO:1903	negative regulation of secretion by cell	0.74839	0.82713	0	0	2	146
GO:0035639 purine ribonucleoside triphosphate binding	31	0.74843	9.064	0.017	Molecular	GO:0035	purine ribonucleoside triphosphate binding	0.74843	0.83081	0	0	31	1848
GO:0030100 regulation of endocytosis	3	0.74869	0.929	0.014	Biological	GO:0030	regulation of endocytosis	0.74869	0.82713	0	0	3	213
GO:0002437 inflammatory response to antigenic stimulus	1	0.74912	0.31	0.013	Biological	GO:0002	inflammatory response to antigenic stimulus	0.74912	0.82713	0	0	1	75
GO:0002637 regulation of immunoglobulin production	1	0.74912	0.31	0.013	Biological	GO:0002	regulation of immunoglobulin production	0.74912	0.82713	0	0	1	75
GO:0016239 positive regulation of macroautophagy	1	0.74912	0.31	0.013	Biological	GO:0016	positive regulation of macroautophagy	0.74912	0.82713	0	0	1	75
GO:0045685 regulation of glial cell differentiation	1	0.74912	0.31	0.013	Biological	GO:0045	regulation of glial cell differentiation	0.74912	0.82713	0	0	1	75
GO:0140238 presynaptic endocytosis	1	0.74912	0.31	0.013	Biological	GO:0140	presynaptic endocytosis	0.74912	0.82713	0	0	1	75
GO:0016614 oxidoreductase activity, acting on CH-OH group of donors	2	0.74974	0.585	0.014	Molecular	GO:0016	oxidoreductase activity, acting on CH-OH group of donors	0.74974	0.83081	0	0	2	144
GO:1902495 transmembrane transporter complex	6	0.74974	1.78	0.015	Cellular C	GO:1902	transmembrane transporter complex	0.74974	0.87963	0	0	6	409
GO:0005230 extracellular ligand-gated ion channel activity	1	0.75023	0.292	0.014	Molecular	GO:0005	extracellular ligand-gated ion channel activity	0.75023	0.83081	0	0	1	74
GO:0042379 chemokine receptor binding	1	0.75023	0.292	0.014	Molecular	GO:0042	chemokine receptor binding	0.75023	0.83081	0	0	1	74
GO:0048029 monosaccharide binding	1	0.75023	0.292	0.014	Molecular	GO:0048	monosaccharide binding	0.75023	0.83081	0	0	1	74
GO:0034654 nucleobase-containing compound biosynthetic process	69	0.75039	21.362	0.017	Biological	GO:0034	nucleobase-containing compound biosynthetic process	0.75039	0.82835	0	0	69	4037
GO:0046474 glycerophospholipid biosynthetic process	3	0.75152	0.929	0.014	Biological	GO:0046	glycerophospholipid biosynthetic process	0.75152	0.82941	0	0	3	214
GO:0008528 G protein-coupled peptide receptor activity	2	0.75316	0.585	0.014	Molecular	GO:0008	G protein-coupled peptide receptor activity	0.75316	0.83299	0	0	2	145
GO:0006023 aminoglycan biosynthetic process	1	0.75371	0.31	0.013	Biological	GO:0006	aminoglycan biosynthetic process	0.75371	0.83147	0	0	1	76
GO:0032890 regulation of organic acid transport	1	0.75371	0.31	0.013	Biological	GO:0032	regulation of organic acid transport	0.75371	0.83147	0	0	1	76
GO:0035861 site of double-strand break	1	0.75426	0.297	0.013	Cellular C	GO:0035	site of double-strand break	0.75426	0.883	0	0	1	77
GO:0019953 sexual reproduction	15	0.75452	4.644	0.016	Biological	GO:0019	sexual reproduction	0.75452	0.83217	0	0	15	951
GO:0001678 cellular glucose homeostasis	2	0.7551	0.619	0.014	Biological	GO:0001	cellular glucose homeostasis	0.7551	0.83245	0	0	2	148
GO:1902652 secondary alcohol metabolic process	2	0.7551	0.619	0.014	Biological	GO:1902	secondary alcohol metabolic process	0.7551	0.83245	0	0	2	148
GO:0030330 DNA damage response, signal transduction by p53 class r	1	0.75822	0.31	0.013	Biological	GO:0030	DNA damage response, signal transduction by p53 class mediator	0.75822	0.83516	0	0	1	77
GO:0030968 endoplasmic reticulum unfolded protein response	1	0.75822	0.31	0.013	Biological	GO:0030	endoplasmic reticulum unfolded protein response	0.75822	0.83516	0	0	1	77
GO:0034637 cellular carbohydrate biosynthetic process	1	0.75822	0.31	0.013	Biological	GO:0034	cellular carbohydrate biosynthetic process	0.75822	0.83516	0	0	1	77
GO:0009582 detection of abiotic stimulus	2	0.7584	0.619	0.013	Biological	GO:0009	detection of abiotic stimulus	0.7584	0.83516	0	0	2	149

GO:0010821 regulation of mitochondrion organization	2	0.7584	0.619	0.013	Biological	GO:0010	regulation of mitochondrion organization	0.7584	0.83516	0	0	2	149
GO:0036126 sperm flagellum	2	0.75931	0.593	0.013	Cellular C	GO:0036	sperm flagellum	0.75931	0.88697	0	0	2	151
GO:0003724 RNA helicase activity	1	0.75944	0.292	0.013	Molecular	GO:0003	RNA helicase activity	0.75944	0.8378	0	0	1	76
GO:0003725 double-stranded RNA binding	1	0.75944	0.292	0.013	Molecular	GO:0003	double-stranded RNA binding	0.75944	0.8378	0	0	1	76
GO:0007224 smoothened signaling pathway	2	0.76166	0.619	0.013	Biological	GO:0007	smoothened signaling pathway	0.76166	0.83799	0	0	2	150
GO:0031023 microtubule organizing center organization	2	0.76166	0.619	0.013	Biological	GO:0031	microtubule organizing center organization	0.76166	0.83799	0	0	2	150
GO:0050728 negative regulation of inflammatory response	2	0.76166	0.619	0.013	Biological	GO:0050	negative regulation of inflammatory response	0.76166	0.83799	0	0	2	150
GO:0060294 cilium movement involved in cell motility	2	0.76166	0.619	0.013	Biological	GO:0060	cilium movement involved in cell motility	0.76166	0.83799	0	0	2	150
GO:0002200 somatic diversification of immune receptors	1	0.76265	0.31	0.013	Biological	GO:0002	somatic diversification of immune receptors	0.76265	0.83799	0	0	1	78
GO:0019226 transmission of nerve impulse	1	0.76265	0.31	0.013	Biological	GO:0019	transmission of nerve impulse	0.76265	0.83799	0	0	1	78
GO:0045814 negative regulation of gene expression, epigenetic	1	0.76265	0.31	0.013	Biological	GO:0045	negative regulation of gene expression, epigenetic	0.76265	0.83799	0	0	1	78
GO:0046209 nitric oxide metabolic process	1	0.76265	0.31	0.013	Biological	GO:0046	nitric oxide metabolic process	0.76265	0.83799	0	0	1	78
GO:0070828 heterochromatin organization	1	0.76265	0.31	0.013	Biological	GO:0070	heterochromatin organization	0.76265	0.83799	0	0	1	78
GO:1903533 regulation of protein targeting	1	0.76265	0.31	0.013	Biological	GO:1903	regulation of protein targeting	0.76265	0.83799	0	0	1	78
GO:0070661 leukocyte proliferation	5	0.76367	1.548	0.014	Biological	GO:0070	leukocyte proliferation	0.76367	0.83892	0	0	5	348
GO:0015748 organophosphate ester transport	2	0.76488	0.619	0.013	Biological	GO:0015	organophosphate ester transport	0.76488	0.83989	0	0	2	151
GO:0051592 response to calcium ion	2	0.76488	0.619	0.013	Biological	GO:0051	response to calcium ion	0.76488	0.83989	0	0	2	151
GO:2001057 reactive nitrogen species metabolic process	1	0.767	0.31	0.013	Biological	GO:2001	reactive nitrogen species metabolic process	0.767	0.84203	0	0	1	79
GO:0008186 ATP-dependent activity, acting on RNA	1	0.76831	0.292	0.013	Molecular	GO:0008	ATP-dependent activity, acting on RNA	0.76831	0.84543	0	0	1	78
GO:0035064 methylated histone binding	1	0.76831	0.292	0.013	Molecular	GO:0035	methylated histone binding	0.76831	0.84543	0	0	1	78
GO:0007005 mitochondrion organization	8	0.76854	2.477	0.015	Biological	GO:0007	mitochondrion organization	0.76854	0.84353	0	0	8	538
GO:0098876 vesicle-mediated transport to the plasma membrane	2	0.77121	0.619	0.013	Biological	GO:0098	vesicle-mediated transport to the plasma membrane	0.77121	0.8456	0	0	2	153
GO:0001895 retina homeostasis	1	0.77126	0.31	0.013	Biological	GO:0001	retina homeostasis	0.77126	0.8456	0	0	1	80
GO:0003300 cardiac muscle hypertrophy	1	0.77126	0.31	0.013	Biological	GO:0003	cardiac muscle hypertrophy	0.77126	0.8456	0	0	1	80
GO:0006900 vesicle budding from membrane	1	0.77126	0.31	0.013	Biological	GO:0006	vesicle budding from membrane	0.77126	0.8456	0	0	1	80
GO:2000142 regulation of DNA-templated transcription, initiation	1	0.77126	0.31	0.013	Biological	GO:2000	regulation of DNA-templated transcription, initiation	0.77126	0.8456	0	0	1	80
GO:0005254 chloride channel activity	1	0.77262	0.292	0.013	Molecular	GO:0005	chloride channel activity	0.77262	0.84802	0	0	1	79
GO:0030971 receptor tyrosine kinase binding	1	0.77262	0.292	0.013	Molecular	GO:0030	receptor tyrosine kinase binding	0.77262	0.84802	0	0	1	79
GO:0016607 nuclear speck	6	0.77373	1.78	0.014	Cellular C	GO:0016	nuclear speck	0.77373	0.90184	0	0	6	421
GO:0016125 sterol metabolic process	2	0.77432	0.619	0.013	Biological	GO:0016	sterol metabolic process	0.77432	0.84876	0	0	2	154
GO:0002532 production of molecular mediator involved in inflammatory	1	0.77545	0.31	0.012	Biological	GO:0002	production of molecular mediator involved in inflammatory response	0.77545	0.84907	0	0	1	81
GO:0008543 fibroblast growth factor receptor signaling pathway	1	0.77545	0.31	0.012	Biological	GO:0008	fibroblast growth factor receptor signaling pathway	0.77545	0.84907	0	0	1	81
GO:0009064 glutamine family amino acid metabolic process	1	0.77545	0.31	0.012	Biological	GO:0009	glutamine family amino acid metabolic process	0.77545	0.84907	0	0	1	81
GO:1901264 carbohydrate derivative transport	1	0.77545	0.31	0.012	Biological	GO:1901	carbohydrate derivative transport	0.77545	0.84907	0	0	1	81
GO:1902117 positive regulation of organelle assembly	1	0.77545	0.31	0.012	Biological	GO:1902	positive regulation of organelle assembly	0.77545	0.84907	0	0	1	81
GO:0140034 methylation-dependent protein binding	1	0.77685	0.292	0.013	Molecular	GO:0140	methylation-dependent protein binding	0.77685	0.85159	0	0	1	80
GO:0043229 intracellular organelle	235	0.77742	69.733	0.018	Cellular C	GO:0043	intracellular organelle	0.77742	0.90298	0	0	235	13356
GO:0034645 cellular macromolecule biosynthetic process	83	0.77748	25.697	0.017	Biological	GO:0034	cellular macromolecule biosynthetic process	0.77748	0.8511	0	0	83	4860
GO:0005929 cilium	11	0.77817	3.264	0.015	Cellular C	GO:0005	cilium	0.77817	0.90298	0	0	11	736
GO:0005901 caveola	1	0.77977	0.297	0.012	Cellular C	GO:0005	caveola	0.77977	0.90298	0	0	1	83
GO:0044242 cellular lipid catabolic process	3	0.78097	0.929	0.013	Biological	GO:0044	cellular lipid catabolic process	0.78097	0.85455	0	0	3	225
GO:0071356 cellular response to tumor necrosis factor	3	0.78097	0.929	0.013	Biological	GO:0071	cellular response to tumor necrosis factor	0.78097	0.85455	0	0	3	225
GO:0006887 exocytosis	5	0.78264	1.548	0.014	Biological	GO:0006	exocytosis	0.78264	0.85612	0	0	5	357
GO:1990542 mitochondrial transmembrane transport	2	0.78343	0.619	0.013	Biological	GO:1990	mitochondrial transmembrane transport	0.78343	0.85612	0	0	2	157
GO:0032272 negative regulation of protein polymerization	1	0.7836	0.31	0.012	Biological	GO:0032	negative regulation of protein polymerization	0.7836	0.85612	0	0	1	83
GO:0032410 negative regulation of transporter activity	1	0.7836	0.31	0.012	Biological	GO:0032	negative regulation of transporter activity	0.7836	0.85612	0	0	1	83
GO:0072527 pyrimidine-containing compound metabolic process	1	0.7836	0.31	0.012	Biological	GO:0072	pyrimidine-containing compound metabolic process	0.7836	0.85612	0	0	1	83
GO:1900542 regulation of purine nucleotide metabolic process	1	0.7836	0.31	0.012	Biological	GO:1900	regulation of purine nucleotide metabolic process	0.7836	0.85612	0	0	1	83
GO:1904427 positive regulation of calcium ion transmembrane transpor	1	0.7836	0.31	0.012	Biological	GO:1904	positive regulation of calcium ion transmembrane transport	0.7836	0.85612	0	0	1	83
GO:0015103 inorganic anion transmembrane transporter activity	2	0.78515	0.585	0.013	Molecular	GO:0015	inorganic anion transmembrane transporter activity	0.78515	0.8596	0	0	2	155
GO:0007600 sensory perception	15	0.78522	4.644	0.015	Biological	GO:0007	sensory perception	0.78522	0.8577	0	0	15	974
GO:0006094 gluconeogenesis	1	0.78756	0.31	0.012	Biological	GO:0006	gluconeogenesis	0.78756	0.85913	0	0	1	84
GO:0032456 endocytic recycling	1	0.78756	0.31	0.012	Biological	GO:0032	endocytic recycling	0.78756	0.85913	0	0	1	84
GO:0036465 synaptic vesicle recycling	1	0.78756	0.31	0.012	Biological	GO:0036	synaptic vesicle recycling	0.78756	0.85913	0	0	1	84
GO:0043299 leukocyte degranulation	1	0.78756	0.31	0.012	Biological	GO:0043	leukocyte degranulation	0.78756	0.85913	0	0	1	84
GO:0045921 positive regulation of exocytosis	1	0.78756	0.31	0.012	Biological	GO:0045	positive regulation of exocytosis	0.78756	0.85913	0	0	1	84
GO:1902369 negative regulation of RNA catabolic process	1	0.78756	0.31	0.012	Biological	GO:1902	negative regulation of RNA catabolic process	0.78756	0.85913	0	0	1	84
GO:0005791 rough endoplasmic reticulum	1	0.78768	0.297	0.012	Cellular C	GO:0005	rough endoplasmic reticulum	0.78768	0.91016	0	0	1	85
GO:0004527 exonuclease activity	1	0.78908	0.292	0.012	Molecular	GO:0004	exonuclease activity	0.78908	0.86172	0	0	1	83
GO:0016209 antioxidant activity	1	0.78908	0.292	0.012	Molecular	GO:0016	antioxidant activity	0.78908	0.86172	0	0	1	83
GO:0007596 blood coagulation	3	0.79095	0.929	0.013	Biological	GO:0007	blood coagulation	0.79095	0.86225	0	0	3	229
GO:0015081 sodium ion transmembrane transporter activity	2	0.7911	0.585	0.013	Molecular	GO:0015	sodium ion transmembrane transporter activity	0.7911	0.86279	0	0	2	157
GO:0006140 regulation of nucleotide metabolic process	1	0.79146	0.31	0.012	Biological	GO:0006	regulation of nucleotide metabolic process	0.79146	0.86225	0	0	1	85
GO:0007492 endoderm development	1	0.79146	0.31	0.012	Biological	GO:0007	endoderm development	0.79146	0.86225	0	0	1	85
GO:0010921 regulation of phosphatase activity	1	0.79146	0.31	0.012	Biological	GO:0010	regulation of phosphatase activity	0.79146	0.86225	0	0	1	85
GO:0046889 positive regulation of lipid biosynthetic process	1	0.79146	0.31	0.012	Biological	GO:0046	positive regulation of lipid biosynthetic process	0.79146	0.86225	0	0	1	85
GO:1903351 cellular response to dopamine	1	0.79146	0.31	0.012	Biological	GO:1903	cellular response to dopamine	0.79146	0.86225	0	0	1	85
GO:0016052 carbohydrate catabolic process	2	0.79221	0.619	0.013	Biological	GO:0016	carbohydrate catabolic process	0.79221	0.86288	0	0	2	160
GO:0043235 receptor complex	6	0.79237	1.78	0.014	Cellular C	GO:0043	receptor complex	0.79237	0.91362	0	0	6	431
GO:0016772 transferase activity, transferring phosphorus-containing gr	15	0.79253	4.386	0.016	Molecular	GO:0016	transferase activity, transferring phosphorus-containing groups	0.79253	0.86279	0	0	15	964
GO:0016791 phosphatase activity	4	0.79305	1.17	0.014	Molecular	GO:0016	phosphatase activity	0.79305	0.86279	0	0	4	292
GO:0030163 protein catabolic process	16	0.79398	4.954	0.015	Biological	GO:0030	protein catabolic process	0.79398	0.86462	0	0	16	1041
GO:0051100 negative regulation of binding	2	0.79507	0.619	0.012	Biological	GO:0051	negative regulation of binding	0.79507	0.86562	0	0	2	161
GO:1903350 response to dopamine	1	0.79528	0.31	0.012	Biological	GO:1903	response to dopamine	0.79528	0.86565	0	0	1	86
GO:0070820 tertiary granule	2	0.79546	0.593	0.012	Cellular C	GO:0070	tertiary granule	0.79546	0.91521	0	0	2	163
GO:0060348 bone development	3	0.7958	0.929	0.013	Biological	GO:0060	bone development	0.7958	0.86594	0	0	3	231
GO:0030098 lymphocyte differentiation	6	0.79588	1.858	0.014	Biological	GO:0030	lymphocyte differentiation	0.79588	0.86594	0	0	6	428
GO:1901566 organonitrogen compound biosynthetic process	28	0.79616	8.669	0.016	Biological	GO:1901	organonitrogen compound biosynthetic process	0.79616	0.86604	0	0	28	1752
GO:0005546 phosphatidylinositol-4,5-bisphosphate binding	1	0.79687	0.292	0.012	Molecular	GO:0005	phosphatidylinositol-4,5-bisphosphate binding	0.79687	0.86585	0	0	1	85
GO:0005819 spindle	6	0.79773	1.78	0.014	Cellular C	GO:0005	spindle	0.79773	0.91585	0	0	6	434
GO:0032787 monocarboxylic acid metabolic process	9	0.79802	2.786	0.015	Biological	GO:0032	monocarboxylic acid metabolic process	0.79802	0.86765	0	0	9	618
GO:0008306 associative learning	1	0.79903	0.31	0.011	Biological	GO:0008	associative learning	0.79903	0.86765	0	0	1	87
GO:0008344 adult locomotory behavior	1	0.79903	0.31	0.011	Biological	GO:0008	adult locomotory behavior	0.79903	0.86765	0	0	1	87
GO:0010507 negative regulation of autophagy	1	0.79903	0.31	0.011	Biological	GO:0010	negative regulation of autophagy	0.79903	0.86765	0	0	1	87
GO:0019319 hexose biosynthetic process	1	0.79903	0.31	0.011	Biological	GO:0019	hexose biosynthetic process	0.79903	0.86765	0	0	1	87
GO:0021766 hippocampus development	1	0.79903	0.31	0.011	Biological	GO:0021	hippocampus development	0.79903	0.86765	0	0	1	87
GO:0030433 ubiquitin-dependent ERAD pathway	1	0.79903	0.31	0.011	Biological	GO:0030	ubiquitin-dependent ERAD pathway	0.79903	0.86765	0	0	1	87
GO:0033077 T cell differentiation in thymus	1	0.79903	0.31	0.011	Biological	GO:0033	T cell differentiation in thymus	0.79903	0.86765	0	0	1	87
GO:0003823 antigen binding	2	0.79974	0.585	0.013	Molecular	GO:0003	antigen binding	0.79974	0.86789	0	0	2	160
GO:0045055 regulated exocytosis	3	0.80055	0.929	0.013	Biological	GO:0045	regulated exocytosis	0.80055	0.86889	0	0	3	233
GO:0001539 cilium or flagellum-dependent cell motility	2	0.80068	0.619	0.012	Biological	GO:0001	cilium or flagellum-dependent cell motility	0.80068	0.86889	0	0	2	163
GO:0060285 cilium-dependent cell motility	2	0.80068	0.619	0.012	Biological	GO:0060	cilium-dependent cell motility	0.80068	0.86889	0	0	2	163
GO:0009059 macromolecule biosynthetic process	83	0.80112	25.697	0.017	Biological	GO:0009	macromolecule biosynthetic process	0.80112	0.86917	0	0	83	4896
GO:0007346 regulation of mitotic cell cycle	7	0.80175	2.167	0.014	Biological	GO:0007	regulation of mitotic cell cycle	0.80175	0.86966	0	0	7	495
GO:0016050 vesicle organization	5	0.80232	1.548	0.014	Biological	GO:0016	vesicle organization	0.80232	0.86976	0	0	5	367
GO:0008757 S-adenosylmethionine-dependent methyltransferase activit	2	0.80255	0.585	0.012	Molecular	GO:0008	S-adenosylmethionine-dependent methyltransferase activity	0.80255	0.86985	0	0	2	161
GO:0000123 histone acetyltransferase complex	1	0.80264	0.297	0.011	Cellular C	GO:0000	histone acetyltransferase complex	0.80264	0.91682	0	0	1	89
GO:0002312 B cell activation involved in immune response	1	0.80271	0.31	0.011	Biological	GO:0002	B cell activation involved in immune response	0.80271	0.86976	0	0	1	88
GO:0008589 regulation of smoothened signaling pathway	1	0.80271	0.31	0.011	Biological	GO:0008	regulation of smoothened signaling pathway	0.80271	0.86976	0	0	1	88
GO:0046785 microtubule polymerization	1	0.80271	0.31	0.011	Biological	GO:0046	microtubule polymerization	0.80271	0.86976	0	0	1	88
GO:0061053 somite development	1	0.80271	0.31	0.011	Biological	GO:0061	somite development	0.80271	0.86976	0	0	1	88
GO:0050817 coagulation	3	0.80289	0.929	0.013	Biological	GO:0050	coagulation	0.80289	0.86977	0	0	3	234
GO:0036064 ciliary basal body	2	0.80372	0.593	0.012	Cellular C	GO:0036	ciliary basal body	0.80372	0.91682	0	0	2	166
GO:0097729 9+2 motile cilium	2	0.80372	0.593	0.012	Cellular C	GO:0097	9+2 motile cilium	0.80372	0.91682	0	0	2	166
GO:0018130 heterocycle biosynthetic process	69	0.80417	21.362	0.017	Biological	GO:0018	heterocycle biosynthetic process	0.80417	0.87096	0	0	69	4112
GO:0022804 active transmembrane transporter activity	6	0.8048	1.754	0.014	Molecular	GO:0022	active transmembrane transporter activity	0.8048	0.87068	0	0	6	426
GO:0002703 regulation of leukocyte mediated immunity	3	0.8052	0.929	0.013	Biological	GO:0002	regulation of leukocyte mediated immunity	0.8052	0.87141	0	0	3	235
GO:0007599 hemostasis	3	0.8052	0.929	0.013	Biological	GO:0007	hemostasis	0.8052	0.87141	0	0	3	235
GO:0015079 potassium ion transmembrane transporter activity	2	0.80533	0.585	0.012	Molecular	GO:0015	potassium ion transmembrane transporter activity	0.80533	0.87068	0	0	2	162
GO:0002250 adaptive immune response	9	0.80548	2.786	0.014	Biological	GO:0002	adaptive immune response	0.80548	0.87141	0	0	9	623
GO:2001233 regulation of apoptotic signaling pathway	5	0.80608	1.548	0.014	Biological	GO:2001	regulation of apoptotic signaling pathway	0.80608	0.87141	0	0	5	369
GO:0031110 regulation of microtubule polymerization or depolymerizatic	1	0.80632	0.31	0.011	Biological	GO:0031	regulation of microtubule polymerization or depolymerization	0.80632	0.87141	0	0	1	89
GO:0046620 regulation of organ growth	1	0.80632	0.31	0.011	Biological	GO:0046	regulation of organ growth	0.80632	0.87141	0	0	1	89
GO:0048145 regulation of fibroblast proliferation	1	0.80632	0.31	0.011	Biological	GO:0048	regulation of fibroblast proliferation	0.80632	0.87141	0	0	1	89
GO:1900182 positive regulation of protein localization to nucleus	1	0.80632	0.31	0.011	Biological	GO:1900	positive regulation of protein localization to nucleus	0.80632	0.87141	0	0	1	89
GO:1903035 negative regulation of response to wounding	1	0.80632	0.31	0.011	Biological	GO:1903	negative regulation of response to wounding	0.80632	0.87141	0	0	1	89
GO:1903578 regulation of ATP metabolic process	1	0.80632	0.31	0.011	Biological	GO:1903	regulation of ATP metabolic process	0.80632	0.87141	0	0	1	89
GO:0099503 secretory vesicle	16	0.80664	4.748	0.015	Cellular C	GO:0099	secretory vesicle	0.80664	0.91819	0	0	16	1064
GO:0035591 signaling adaptor activity	1	0.808	0.292	0.011	Molecular	GO:0035	signaling adaptor activity	0.808	0.87179	0	0	1	88
GO:0004888 transmembrane signaling receptor activity	20	0.80837	5.848	0.016	Molecular	GO:0004	transmembrane signaling receptor activity	0.80837	0.87179	0	0	20	1272
GO:0007034 vacuolar transport	2	0.80884	0.619	0.012	Biological	GO:0007	vacuolar transport	0.80884	0.87357	0	0	2	166
GO:0016579 protein deubiquitination	2	0.80884	0.619	0.012	Biological	GO:0016	protein deubiquitination	0.80884	0.87357	0	0	2	166
GO:0035637 multicellular organismal signaling	2	0.80884	0.619	0.012	Biological	GO:0035	multicellular organismal signaling	0.80884	0.87357	0	0	2	166
GO:0035578 azurophil granule lumen	1	0.80973	0.297	0.011	Cellular C	GO:0035	azurophil granule lumen	0.80973	0.91975	0	0	1	91
GO:0008037 cell recognition	3	0.80977	0.929	0.013	Biological	GO:0008	cell recognition	0.80977	0.87392	0	0	3	237
GO:0045088 regulation of innate immune response	3	0.80977	0.929	0.013	Biological	GO:0045	regulation of innate immune response	0.80977	0.87392	0	0	3	237
GO:0045104 intermediate filament cytoskeleton organization	1	0.80987	0.31	0.011	Biological	GO:0045	intermediate filament cytoskeleton organization	0.80987	0.87392	0	0	1	90
GO:0051303 establishment of chromosome localization	1	0.80987	0.31	0.011	Biological	GO:0051	establishment of chromosome localization	0.80987	0.87392	0	0	1	90
GO:0019438 aromatic compound biosynthetic process	69	0.81138	21.362	0.017	Biological	GO:0019	aromatic compound biosynthetic process	0.81138	0.87511	0	0	69	4123
GO:0006606 protein import into nucleus	2	0.8115	0.619	0.012	Biological	GO:0006	protein import into nucleus	0.8115	0.87511	0	0	2	167
GO:0043488 regulation of mRNA stability	2	0.8115	0.619	0.012	Biological	GO:0043	regulation of mRNA stability	0.8115	0.87511	0	0	2	167
GO:0019217 regulation of fatty acid metabolic process	1	0.81336	0.31	0.011	Biological	GO:0019	regulation of fatty acid metabolic process	0.81336	0.87616	0	0	1	91
GO:0036473 cell death in response to oxidative stress	1	0.81336	0.31	0.011	Biological	GO:0036	cell death in response to oxidative stress	0.81336	0.87616	0	0	1	91
GO:0045103 intermediate filament-based process	1	0.81336	0.31	0.011	Biological	GO:0045	intermediate filament-based process	0.81336	0.87616	0	0	1	91
GO:0046364 monosaccharide biosynthetic process	1	0.81336	0.31	0.011	Biological	GO:0046	monosaccharide biosynthetic process	0.81336	0.87616	0	0	1	91
GO:1901992 positive regulation of mitotic cell cycle phase transition	1	0.81336	0.31	0.011	Biological	GO:1901	positive regulation of mitotic cell cycle phase transition	0.81336	0.87616	0	0	1	91
GO:0050670 regulation of lymphocyte proliferation	3	0.81425	0.929	0.013	Biological	GO:0050	regulation of lymphocyte proliferation	0.81425	0.87675	0	0	3	239
GO:1901617 organic hydroxy compound biosynthetic process	3	0.81425	0.929	0.013	Biological	GO:1901	organic hydroxy compound biosynthetic process	0.81425	0.87675	0	0	3	239
GO:0032555 purine ribonucleotide binding	31	0.81487	9.064	0.016	Molecular	GO:0032	purine ribonucleotide binding	0.81487	0.87771	0	0	31	1917
GO:0060271 cilium assembly	5	0.81524	1.548	0.013	Biological	GO:0060	cilium assembly	0.81524	0.87763	0	0	5	374
GO:0005319 lipid transporter activity	2	0.81609	0.585	0.012	Molecular	GO:0005	lipid transporter activity	0.81609	0.87793	0	0	2	166
GO:0002252 immune effector process	10	0.81636	3.096	0.014	Biological	GO:0002	immune effector process	0.81636	0.87814	0	0	10	693
GO:0051048 negative regulation of secretion	2	0.81671	0.619	0.012	Biological	GO:0051	negative regulation of secretion	0.81671	0.87814	0	0	2	169
GO:0007029 endoplasmic reticulum organization	1	0.81678	0.31	0.011	Biological	GO:0007	endoplasmic reticulum organization	0.81678	0.87814	0	0	1	92
GO:0019646 aerobic electron transport chain	1	0.81678	0.31	0.011	Biological	GO:0019	aerobic electron transport chain	0.81678	0.87814	0	0	1	92
GO:0050000 chromosome localization	1	0.81678	0.31	0.011	Biological	GO:0050	chromosome localization	0.81678	0.87814	0	0	1	92
GO:0061912 selective autophagy	1	0.81678	0.31	0.011	Biological	GO:0061	selective autophagy	0.81678	0.87814	0	0	1	92
GO:0043087 regulation of GTPase activity	5	0.81703	1.548	0.013	Biological	GO:0043	regulation of GTPase activity	0.81703	0.87823	0	0	5	375
GO:0006813 potassium ion transport	3	0.81864	0.929	0.012	Biological	GO:0006	potassium ion transport	0.81864	0.87958	0	0	3	241
GO:0032271 regulation of protein polymerization	3	0.81864	0.929	0.012	Biological	GO:0032	regulation of protein polymerization	0.81864	0.87958	0	0	3	241
GO:2001242 regulation of intrinsic apoptotic signaling pathway	2	0.81927	0.619	0.012	Biological	GO:2001	regulation of intrinsic apoptotic signaling pathway	0.81927	0.88006	0	0	2	170
GO:0009898 cytoplasmic side of plasma membrane	2	0.81935	0.593	0.012	Cellular C	GO:0009	cytoplasmic side of plasma membrane	0.81935	0.92781	0	0	2	172

GO:0046651 lymphocyte proliferation	4	0.81989	1.238	0.013	Biological	GO:00466	lymphocyte proliferation	0.81989	0.88024	0	0	4	310
GO:0030641 regulation of cellular pH	1	0.82013	0.31	0.011	Biological	GO:00306	regulation of cellular pH	0.82013	0.88024	0	0	1	93
GO:0060996 dendritic spine development	1	0.82013	0.31	0.011	Biological	GO:00609	dendritic spine development	0.82013	0.88024	0	0	1	93
GO:2000106 regulation of leukocyte apoptotic process	1	0.82013	0.31	0.011	Biological	GO:20001	regulation of leukocyte apoptotic process	0.82013	0.88024	0	0	1	93
GO:0031966 mitochondrial membrane	11	0.82042	3.264	0.014	Cellular C	GO:00319	mitochondrial membrane	0.82042	0.92781	0	0	11	767
GO:0044249 cellular biosynthetic process	100	0.82083	30.96	0.017	Biological	GO:00442	cellular biosynthetic process	0.82083	0.8808	0	0	100	5881
GO:0030141 secretory granule	13	0.82202	3.858	0.015	Cellular C	GO:00301	secretory granule	0.82202	0.92781	0	0	13	893
GO:0016358 dendrite development	3	0.82293	0.929	0.012	Biological	GO:00163	dendrite development	0.82293	0.88264	0	0	3	243
GO:0000422 autophagy of mitochondrion	1	0.82343	0.31	0.011	Biological	GO:00004	autophagy of mitochondrion	0.82343	0.88264	0	0	1	94
GO:0043648 dicarboxylic acid metabolic process	1	0.82343	0.31	0.011	Biological	GO:00436	dicarboxylic acid metabolic process	0.82343	0.88264	0	0	1	94
GO:0061726 mitochondrion disassembly	1	0.82343	0.31	0.011	Biological	GO:00617	mitochondrion disassembly	0.82343	0.88264	0	0	1	94
GO:0098869 cellular oxidant detoxification	1	0.82343	0.31	0.011	Biological	GO:00988	cellular oxidant detoxification	0.82343	0.88264	0	0	1	94
GO:0051170 import into nucleus	2	0.82428	0.619	0.012	Biological	GO:00511	import into nucleus	0.82428	0.88317	0	0	2	172
GO:1901136 carbohydrate derivative catabolic process	2	0.82428	0.619	0.012	Biological	GO:19011	carbohydrate derivative catabolic process	0.82428	0.88317	0	0	2	172
GO:0017076 purine nucleotide binding	31	0.82508	9.064	0.016	Molecular	GO:00170	purine nucleotide binding	0.82508	0.88553	0	0	31	1929
GO:0051219 phosphoprotein binding	1	0.82523	0.292	0.011	Molecular	GO:00512	phosphoprotein binding	0.82523	0.88553	0	0	1	93
GO:0016853 isomerase activity	2	0.82632	0.585	0.012	Molecular	GO:00168	isomerase activity	0.82632	0.88553	0	0	2	170
GO:0035082 axoneme assembly	1	0.82667	0.31	0.011	Biological	GO:00350	axoneme assembly	0.82667	0.88502	0	0	1	95
GO:0044070 regulation of anion transport	1	0.82667	0.31	0.011	Biological	GO:00440	regulation of anion transport	0.82667	0.88502	0	0	1	95
GO:0050776 regulation of immune response	14	0.8267	4.334	0.015	Biological	GO:00507	regulation of immune response	0.8267	0.88502	0	0	14	948
GO:0002706 regulation of lymphocyte mediated immunity	2	0.82674	0.619	0.012	Biological	GO:00027	regulation of lymphocyte mediated immunity	0.82674	0.88502	0	0	2	173
GO:0006091 generation of precursor metabolites and energy	7	0.82689	2.167	0.014	Biological	GO:00060	generation of precursor metabolites and energy	0.82689	0.88502	0	0	7	511
GO:0030072 peptide hormone secretion	3	0.82715	0.929	0.012	Biological	GO:00300	peptide hormone secretion	0.82715	0.8851	0	0	3	245
GO:0032838 plasma membrane bounded cell projection cytoplasm	3	0.82748	0.89	0.012	Cellular C	GO:00328	plasma membrane bounded cell projection cytoplasm	0.82748	0.93039	0	0	3	248
GO:0016887 ATP hydrolysis activity	5	0.82781	1.462	0.013	Molecular	GO:00168	ATP hydrolysis activity	0.82781	0.88553	0	0	5	375
GO:0032553 ribonucleotide binding	31	0.82921	9.064	0.016	Molecular	GO:00325	ribonucleotide binding	0.82921	0.88553	0	0	31	1934
GO:1905369 endopeptidase complex	1	0.82949	0.297	0.01	Cellular C	GO:19053	endopeptidase complex	0.82949	0.93039	0	0	1	97
GO:0036464 cytoplasmic ribonucleoprotein granule	3	0.82953	0.89	0.012	Cellular C	GO:00364	cytoplasmic ribonucleoprotein granule	0.82953	0.93039	0	0	3	249
GO:0022412 cellular process involved in reproduction in multicellular org	6	0.82955	1.858	0.013	Biological	GO:00224	cellular process involved in reproduction in multicellular organism	0.82955	0.88742	0	0	6	448
GO:0061337 cardiac conduction	1	0.82984	0.31	0.01	Biological	GO:00613	cardiac conduction	0.82984	0.88742	0	0	1	96
GO:1903312 negative regulation of mRNA metabolic process	1	0.82984	0.31	0.01	Biological	GO:19033	negative regulation of mRNA metabolic process	0.82984	0.88742	0	0	1	96
GO:0030695 GTPase regulator activity	7	0.83031	2.047	0.014	Molecular	GO:00306	GTPase regulator activity	0.83031	0.88553	0	0	7	505
GO:0060589 nucleoside-triphosphatase regulator activity	7	0.83031	2.047	0.014	Molecular	GO:00605	nucleoside-triphosphatase regulator activity	0.83031	0.88553	0	0	7	505
GO:0016197 endosomal transport	3	0.83127	0.929	0.012	Biological	GO:00161	endosomal transport	0.83127	0.88876	0	0	3	247
GO:0016407 acetyltransferase activity	1	0.83168	0.292	0.011	Molecular	GO:00164	acetyltransferase activity	0.83168	0.8859	0	0	1	95
GO:0022900 electron transport chain	2	0.83394	0.619	0.011	Biological	GO:00229	electron transport chain	0.83394	0.89142	0	0	2	176
GO:0010467 gene expression	101	0.83466	31.269	0.017	Biological	GO:00104	gene expression	0.83466	0.892	0	0	101	5962
GO:0098657 import into cell	3	0.83531	0.929	0.012	Biological	GO:00986	import into cell	0.83531	0.8925	0	0	3	249
GO:0031248 protein acetyltransferase complex	1	0.83562	0.297	0.01	Cellular C	GO:00312	protein acetyltransferase complex	0.83562	0.93469	0	0	1	99
GO:0006029 proteoglycan metabolic process	1	0.83602	0.31	0.01	Biological	GO:00060	proteoglycan metabolic process	0.83602	0.89269	0	0	1	98
GO:0044728 DNA methylation or demethylation	1	0.83602	0.31	0.01	Biological	GO:00447	DNA methylation or demethylation	0.83602	0.89269	0	0	1	98
GO:0051952 regulation of amine transport	1	0.83602	0.31	0.01	Biological	GO:00519	regulation of amine transport	0.83602	0.89269	0	0	1	98
GO:0019827 stem cell population maintenance	2	0.83628	0.619	0.011	Biological	GO:00198	stem cell population maintenance	0.83628	0.89274	0	0	2	177
GO:0044238 primary metabolic process	179	0.83643	55.418	0.017	Biological	GO:00442	primary metabolic process	0.83643	0.89274	0	0	179	10264
GO:0042742 defense response to bacterium	5	0.83745	1.548	0.013	Biological	GO:00427	defense response to bacterium	0.83745	0.89363	0	0	5	387
GO:0043202 lysosomal lumen	1	0.8386	0.297	0.01	Cellular C	GO:00432	lysosomal lumen	0.8386	0.93469	0	0	1	100
GO:1902493 acetyltransferase complex	1	0.8386	0.297	0.01	Cellular C	GO:19024	acetyltransferase complex	0.8386	0.93469	0	0	1	100
GO:0034620 cellular response to unfolded protein	1	0.83903	0.31	0.01	Biological	GO:00346	cellular response to unfolded protein	0.83903	0.89504	0	0	1	99
GO:0009056 catabolic process	42	0.83912	13.003	0.016	Biological	GO:00090	catabolic process	0.83912	0.89504	0	0	42	2620
GO:0140030 modification-dependent protein binding	2	0.8407	0.585	0.011	Molecular	GO:01400	modification-dependent protein binding	0.8407	0.89441	0	0	2	176
GO:0030833 regulation of actin filament polymerization	2	0.84087	0.619	0.011	Biological	GO:00308	regulation of actin filament polymerization	0.84087	0.89656	0	0	2	179
GO:0009314 response to radiation	6	0.84172	1.858	0.013	Biological	GO:00093	response to radiation	0.84172	0.89656	0	0	6	456
GO:0031647 regulation of protein stability	4	0.84181	1.238	0.012	Biological	GO:00316	regulation of protein stability	0.84181	0.89656	0	0	4	322
GO:0042773 ATP synthesis coupled electron transport	1	0.84198	0.31	0.01	Biological	GO:00427	ATP synthesis coupled electron transport	0.84198	0.89656	0	0	1	100
GO:0042775 mitochondrial ATP synthesis coupled electron transport	1	0.84198	0.31	0.01	Biological	GO:00427	mitochondrial ATP synthesis coupled electron transport	0.84198	0.89656	0	0	1	100
GO:0071868 cellular response to monoamine stimulus	1	0.84198	0.31	0.01	Biological	GO:00718	cellular response to monoamine stimulus	0.84198	0.89656	0	0	1	100
GO:0071870 cellular response to catecholamine stimulus	1	0.84198	0.31	0.01	Biological	GO:00718	cellular response to catecholamine stimulus	0.84198	0.89656	0	0	1	100
GO:0110020 regulation of actomyosin structure organization	1	0.84198	0.31	0.01	Biological	GO:01100	regulation of actomyosin structure organization	0.84198	0.89656	0	0	1	100
GO:0043487 regulation of RNA stability	2	0.84312	0.619	0.011	Biological	GO:00434	regulation of RNA stability	0.84312	0.89723	0	0	2	180
GO:0045580 regulation of T cell differentiation	2	0.84312	0.619	0.011	Biological	GO:00455	regulation of T cell differentiation	0.84312	0.89723	0	0	2	180
GO:0050851 antigen receptor-mediated signaling pathway	3	0.84315	0.929	0.012	Biological	GO:00508	antigen receptor-mediated signaling pathway	0.84315	0.89723	0	0	3	253
GO:0000775 chromosome, centromeric region	3	0.84328	0.89	0.012	Cellular C	GO:00007	chromosome, centromeric region	0.84328	0.93795	0	0	3	256
GO:0008135 translation factor activity, RNA binding	1	0.84388	0.292	0.01	Molecular	GO:00081	translation factor activity, RNA binding	0.84388	0.89669	0	0	1	99
GO:0002275 myeloid cell activation involved in immune response	1	0.84488	0.31	0.01	Biological	GO:00022	myeloid cell activation involved in immune response	0.84488	0.89842	0	0	1	101
GO:0019080 viral gene expression	1	0.84488	0.31	0.01	Biological	GO:00190	viral gene expression	0.84488	0.89842	0	0	1	101
GO:0050764 regulation of phagocytosis	1	0.84488	0.31	0.01	Biological	GO:00507	regulation of phagocytosis	0.84488	0.89842	0	0	1	101
GO:0090068 positive regulation of cell cycle process	3	0.84506	0.929	0.012	Biological	GO:00900	positive regulation of cell cycle process	0.84506	0.89842	0	0	3	254
GO:1901565 organonitrogen compound catabolic process	21	0.84527	6.502	0.015	Biological	GO:19015	organonitrogen compound catabolic process	0.84527	0.89842	0	0	21	1391
GO:0098727 maintenance of cell number	2	0.84535	0.619	0.011	Biological	GO:00987	maintenance of cell number	0.84535	0.89842	0	0	2	181
GO:0000226 microtubule cytoskeleton organization	9	0.84593	2.786	0.014	Biological	GO:00002	microtubule cytoskeleton organization	0.84593	0.89885	0	0	9	653
GO:0004725 protein tyrosine phosphatase activity	1	0.84679	0.292	0.01	Molecular	GO:00047	protein tyrosine phosphatase activity	0.84679	0.89867	0	0	1	100
GO:0005796 Golgi lumen	1	0.84721	0.297	0.01	Cellular C	GO:00057	Golgi lumen	0.84721	0.93853	0	0	1	103
GO:0101002 ficolin-1-rich granule	2	0.84731	0.593	0.011	Cellular C	GO:01010	ficolin-1-rich granule	0.84731	0.93853	0	0	2	184
GO:0004867 serine-type endopeptidase inhibitor activity	1	0.84964	0.292	0.01	Molecular	GO:00048	serine-type endopeptidase inhibitor activity	0.84964	0.8995	0	0	1	101
GO:0019888 protein phosphatase regulator activity	1	0.84964	0.292	0.01	Molecular	GO:00198	protein phosphatase regulator activity	0.84964	0.8995	0	0	1	101
GO:0009416 response to light stimulus	4	0.85026	1.238	0.012	Biological	GO:00094	response to light stimulus	0.85026	0.90276	0	0	4	327
GO:0007040 lysosome organization	1	0.85051	0.31	0.01	Biological	GO:00070	lysosome organization	0.85051	0.90276	0	0	1	103
GO:0071867 response to monoamine	1	0.85051	0.31	0.01	Biological	GO:00718	response to monoamine	0.85051	0.90276	0	0	1	103
GO:0071869 response to catecholamine	1	0.85051	0.31	0.01	Biological	GO:00718	response to catecholamine	0.85051	0.90276	0	0	1	103
GO:0080171 lytic vacuole organization	1	0.85051	0.31	0.01	Biological	GO:00801	lytic vacuole organization	0.85051	0.90276	0	0	1	103
GO:0080134 regulation of response to stress	21	0.85133	6.502	0.015	Biological	GO:00801	regulation of response to stress	0.85133	0.90343	0	0	21	1398
GO:0061013 regulation of mRNA catabolic process	2	0.85184	0.619	0.011	Biological	GO:00610	regulation of mRNA catabolic process	0.85184	0.90378	0	0	2	184
GO:0005496 steroid binding	1	0.85245	0.292	0.01	Molecular	GO:00054	steroid binding	0.85245	0.90136	0	0	1	102
GO:0034708 methyltransferase complex	1	0.8527	0.297	0.01	Cellular C	GO:00347	methyltransferase complex	0.8527	0.94255	0	0	1	105
GO:0032231 regulation of actin filament bundle assembly	1	0.85325	0.31	0.01	Biological	GO:00322	regulation of actin filament bundle assembly	0.85325	0.9049	0	0	1	104
GO:0033209 tumor necrosis factor-mediated signaling pathway	1	0.85325	0.31	0.01	Biological	GO:00332	tumor necrosis factor-mediated signaling pathway	0.85325	0.9049	0	0	1	104
GO:0005525 GTP binding	5	0.85367	1.462	0.013	Molecular	GO:00055	GTP binding	0.85367	0.90156	0	0	5	391
GO:0070646 protein modification by small protein removal	2	0.85395	0.619	0.011	Biological	GO:00706	protein modification by small protein removal	0.85395	0.90544	0	0	2	185
GO:0007281 germ cell development	4	0.85514	1.238	0.012	Biological	GO:00072	germ cell development	0.85514	0.90592	0	0	4	330
GO:0140014 mitotic nuclear division	4	0.85514	1.238	0.012	Biological	GO:01400	mitotic nuclear division	0.85514	0.90592	0	0	4	330
GO:0002694 regulation of leukocyte activation	9	0.85553	2.786	0.014	Biological	GO:00026	regulation of leukocyte activation	0.85553	0.90592	0	0	9	661
GO:0002377 immunoglobulin production	1	0.85595	0.31	0.01	Biological	GO:00023	immunoglobulin production	0.85595	0.90592	0	0	1	105
GO:0006626 protein targeting to mitochondrion	1	0.85595	0.31	0.01	Biological	GO:00066	protein targeting to mitochondrion	0.85595	0.90592	0	0	1	105
GO:0030838 positive regulation of actin filament polymerization	1	0.85595	0.31	0.01	Biological	GO:00308	positive regulation of actin filament polymerization	0.85595	0.90592	0	0	1	105
GO:0033045 regulation of sister chromatid segregation	1	0.85595	0.31	0.01	Biological	GO:00330	regulation of sister chromatid segregation	0.85595	0.90592	0	0	1	105
GO:0043266 regulation of potassium ion transport	1	0.85595	0.31	0.01	Biological	GO:00432	regulation of potassium ion transport	0.85595	0.90592	0	0	1	105
GO:0015698 inorganic anion transport	2	0.85604	0.619	0.011	Biological	GO:00156	inorganic anion transport	0.85604	0.90592	0	0	2	186
GO:0044344 cellular response to fibroblast growth factor stimulus	1	0.85859	0.31	0.009	Biological	GO:00443	cellular response to fibroblast growth factor stimulus	0.85859	0.90785	0	0	1	106
GO:0048144 fibroblast proliferation	1	0.85859	0.31	0.009	Biological	GO:00481	fibroblast proliferation	0.85859	0.90785	0	0	1	106
GO:0050773 regulation of dendrite development	1	0.85859	0.31	0.009	Biological	GO:00507	regulation of dendrite development	0.85859	0.90785	0	0	1	106
GO:1904035 regulation of epithelial cell apoptotic process	1	0.85859	0.31	0.009	Biological	GO:19040	regulation of epithelial cell apoptotic process	0.85859	0.90785	0	0	1	106
GO:0061025 membrane fusion	2	0.86012	0.619	0.011	Biological	GO:00610	membrane fusion	0.86012	0.90928	0	0	2	188
GO:0090734 site of DNA damage	1	0.86057	0.297	0.009	Cellular C	GO:00907	site of DNA damage	0.86057	0.94929	0	0	1	108
GO:0016788 hydrolase activity, acting on ester bonds	11	0.86058	3.216	0.014	Molecular	GO:00167	hydrolase activity, acting on ester bonds	0.86058	0.90775	0	0	11	780
GO:0015837 amine transport	1	0.86118	0.31	0.009	Biological	GO:00158	amine transport	0.86118	0.90982	0	0	1	107
GO:0019882 antigen processing and presentation	1	0.86118	0.31	0.009	Biological	GO:00198	antigen processing and presentation	0.86118	0.90982	0	0	1	107
GO:1902106 negative regulation of leukocyte differentiation	1	0.86118	0.31	0.009	Biological	GO:19021	negative regulation of leukocyte differentiation	0.86118	0.90982	0	0	1	107
GO:0035770 ribonucleoprotein granule	3	0.86295	0.89	0.011	Cellular C	GO:00357	ribonucleoprotein granule	0.86295	0.94995	0	0	3	267
GO:0003730 mRNA 3'-UTR binding	1	0.86314	0.292	0.009	Molecular	GO:00037	mRNA 3'-UTR binding	0.86314	0.90824	0	0	1	106
GO:0030594 neurotransmitter receptor activity	1	0.86314	0.292	0.009	Molecular	GO:00305	neurotransmitter receptor activity	0.86314	0.90824	0	0	1	106
GO:0007631 feeding behavior	1	0.86373	0.31	0.009	Biological	GO:00076	feeding behavior	0.86373	0.91189	0	0	1	108
GO:0098586 cellular response to virus	1	0.86373	0.31	0.009	Biological	GO:00985	cellular response to virus	0.86373	0.91189	0	0	1	108
GO:1901796 regulation of signal transduction by p53 class mediator	1	0.86373	0.31	0.009	Biological	GO:19017	regulation of signal transduction by p53 class mediator	0.86373	0.91189	0	0	1	108
GO:1901362 organic cyclic compound biosynthetic process	70	0.86386	21.672	0.016	Biological	GO:19013	organic cyclic compound biosynthetic process	0.86386	0.91189	0	0	70	4270
GO:0043603 cellular amide metabolic process	17	0.86528	5.263	0.015	Biological	GO:00436	cellular amide metabolic process	0.86528	0.91319	0	0	17	1171
GO:0051087 chaperone binding	1	0.86569	0.292	0.009	Molecular	GO:00510	chaperone binding	0.86569	0.9092	0	0	1	107
GO:0006892 post-Golgi vesicle-mediated transport	1	0.86623	0.31	0.009	Biological	GO:00068	post-Golgi vesicle-mediated transport	0.86623	0.91399	0	0	1	109
GO:0000149 SNARE binding	1	0.8682	0.292	0.009	Molecular	GO:00001	SNARE binding	0.8682	0.9092	0	0	1	108
GO:0004721 phosphoprotein phosphatase activity	2	0.86823	0.585	0.011	Molecular	GO:00047	phosphoprotein phosphatase activity	0.86823	0.9092	0	0	2	189
GO:0003924 GTPase activity	4	0.86825	1.17	0.012	Molecular	GO:00039	GTPase activity	0.86825	0.9092	0	0	4	333
GO:0000045 autophagosome assembly	1	0.86868	0.31	0.009	Biological	GO:00000	autophagosome assembly	0.86868	0.9162	0	0	1	110
GO:1990748 cellular detoxification	1	0.86868	0.31	0.009	Biological	GO:19907	cellular detoxification	0.86868	0.9162	0	0	1	110
GO:0006958 complement activation, classical pathway	1	0.87109	0.31	0.009	Biological	GO:00069	complement activation, classical pathway	0.87109	0.91777	0	0	1	111
GO:0036503 ERAD pathway	1	0.87109	0.31	0.009	Biological	GO:00365	ERAD pathway	0.87109	0.91777	0	0	1	111
GO:0046822 regulation of nucleocytoplasmic transport	1	0.87109	0.31	0.009	Biological	GO:00468	regulation of nucleocytoplasmic transport	0.87109	0.91777	0	0	1	111
GO:1902749 regulation of cell cycle G2/M phase transition	1	0.87109	0.31	0.009	Biological	GO:19027	regulation of cell cycle G2/M phase transition	0.87109	0.91777	0	0	1	111
GO:1903707 negative regulation of hemopoiesis	1	0.87109	0.31	0.009	Biological	GO:19037	negative regulation of hemopoiesis	0.87109	0.91777	0	0	1	111
GO:0009058 biosynthetic process	101	0.87294	31.269	0.017	Biological	GO:00090	biosynthetic process	0.87294	0.91952	0	0	101	6040
GO:0019395 fatty acid oxidation	1	0.87345	0.31	0.009	Biological	GO:00193	fatty acid oxidation	0.87345	0.91987	0	0	1	112
GO:0008022 protein C-terminus binding	2	0.87393	0.585	0.01	Molecular	GO:00080	protein C-terminus binding	0.87393	0.91404	0	0	2	192
GO:0044853 plasma membrane raft	1	0.87507	0.297	0.009	Cellular C	GO:00448	plasma membrane raft	0.87507	0.96132	0	0	1	114
GO:0008064 regulation of actin polymerization or depolymerization	2	0.87542	0.619	0.01	Biological	GO:00080	regulation of actin polymerization or depolymerization	0.87542	0.92155	0	0	2	196
GO:0009755 hormone-mediated signaling pathway	2	0.87542	0.619	0.01	Biological	GO:00097	hormone-mediated signaling pathway	0.87542	0.92155	0	0	2	196
GO:0071774 response to fibroblast growth factor	1	0.87578	0.31	0.009	Biological	GO:00717	response to fibroblast growth factor	0.87578	0.92173	0	0	1	113
GO:0000278 mitotic cell cycle	13	0.87712	4.025	0.014	Biological	GO:00002	mitotic cell cycle	0.87712	0.92295	0	0	13	936
GO:0005740 mitochondrial envelope	11	0.87826	3.264	0.013	Cellular C	GO:00057	mitochondrial envelope	0.87826	0.96285	0	0	11	820
GO:0010638 positive regulation of organelle organization	7	0.87885	2.167	0.013	Biological	GO:00106	positive regulation of organelle organization	0.87885	0.92454	0	0	7	551
GO:0001906 cell killing	2	0.87899	0.619	0.01	Biological	GO:00019	cell killing	0.87899	0.92454	0	0	2	198
GO:0000166 nucleotide binding	34	0.8791	9.942	0.016	Molecular	GO:00001	nucleotide binding	0.8791	0.91693	0	0	34	2178
GO:1901265 nucleoside phosphate binding	34	0.87972	9.942	0.016	Molecular	GO:19012	nucleoside phosphate binding	0.87972	0.91693	0	0	34	2179
GO:0005096 GTPase activator activity	6	0.87987	1.754	0.013	Molecular	GO:00050	GTPase activator activity	0.87987	0.91693	0	0	6	477
GO:0002708 positive regulation of lymphocyte mediated immunity	1	0.88029	0.31	0.009	Biological	GO:00027	positive regulation of lymphocyte mediated immunity	0.88029	0.92551	0	0	1	115
GO:1901989 positive regulation of cell cycle phase transition	1	0.88029	0.31	0.009	Biological	GO:19019	positive regulation of cell cycle phase transition	0.88029	0.92551	0	0	1	115
GO:0098796 membrane protein complex	18	0.88053	5.341	0.014	Cellular C	GO:00987	membrane protein complex	0.88053	0.96289	0	0	18	1266
GO:0000793 condensed chromosome	3	0.8819	0.89	0.011	Cellular C	GO:00007	condensed chromosome	0.8819	0.96289	0	0	3	279
GO:0051082 unfolded protein binding	1	0.88227	0.292	0.009	Molecular	GO:00510	unfolded protein binding	0.88227	0.91781	0	0	1	114
GO:0034249 negative regulation of cellular amide metabolic process	2	0.88248	0.619	0.01	Biological	GO:00342	negative regulation of cellular amide metabolic process	0.88248	0.92761	0	0	2	200
GO:0019001 guanyl nucleotide binding	5	0.8839	1.462	0.012	Molecular	GO:00190	guanyl nucleotide binding	0.8839	0.91781	0	0	5	413
GO:0032561 guanyl ribonucleotide binding	5	0.8839	1.462	0.012	Molecular	GO:00325	guanyl ribonucleotide binding	0.8839	0.91781	0	0	5	413
GO:0007051 spindle organization	2	0.88418	0.619	0.01	Biological	GO:00070	spindle organization	0.88418	0.92872	0	0	2	201
GO:0030041 actin filament polymerization	2	0.88418	0.619	0.01	Biological	GO:00300	actin filament polymerization	0.88418	0.92872	0	0	2	201
GO:1901576 organic substance biosynthetic process	99	0.88429	30.65	0.017	Biological	GO:19015	organic substance biosynthetic process	0.88429	0.92872	0	0	99	5954

GO:0030004 cellular monovalent inorganic cation homeostasis	1	0.88464	0.31	0.009	Biological	GO:0030(	cellular monovalent inorganic cation homeostasis	0.88464	0.92872	0	0	1	117
GO:0030518 intracellular steroid hormone receptor signaling pathway	1	0.88464	0.31	0.009	Biological	GO:0030!	intracellular steroid hormone receptor signaling pathway	0.88464	0.92872	0	0	1	117
GO:1905037 autophagosome organization	1	0.88464	0.31	0.009	Biological	GO:1905(	autophagosome organization	0.88464	0.92872	0	0	1	117
GO:0001824 blastocyst development	1	0.88676	0.31	0.008	Biological	GO:0001{	blastocyst development	0.88676	0.93035	0	0	1	118
GO:0007088 regulation of mitotic nuclear division	1	0.88676	0.31	0.008	Biological	GO:0007(	regulation of mitotic nuclear division	0.88676	0.93035	0	0	1	118
GO:0034440 lipid oxidation	1	0.88676	0.31	0.008	Biological	GO:0034	lipid oxidation	0.88676	0.93035	0	0	1	118
GO:0016064 immunoglobulin mediated immune response	2	0.88753	0.619	0.01	Biological	GO:0016(	immunoglobulin mediated immune response	0.88753	0.93096	0	0	2	203
GO:0015850 organic hydroxy compound transport	3	0.88805	0.929	0.011	Biological	GO:0015{	organic hydroxy compound transport	0.88805	0.93131	0	0	3	280
GO:0005829 cytosol	89	0.88839	26.409	0.016	Cellular C	GO:0005{	cytosol	0.88839	0.968	0	0	89	5467
GO:0009055 electron transfer activity	1	0.88874	0.292	0.009	Molecular	GO:0009(	electron transfer activity	0.88874	0.92173	0	0	1	117
GO:0035967 cellular response to topologically incorrect protein	1	0.88884	0.31	0.008	Biological	GO:0035!	cellular response to topologically incorrect protein	0.88884	0.93175	0	0	1	119
GO:0061640 cytoskeleton-dependent cytokinesis	1	0.88884	0.31	0.008	Biological	GO:0061(	cytoskeleton-dependent cytokinesis	0.88884	0.93175	0	0	1	119
GO:0004843 thiol-dependent deubiquitinase	1	0.89081	0.292	0.008	Molecular	GO:0004{	thiol-dependent deubiquitinase	0.89081	0.92278	0	0	1	118
GO:0002698 negative regulation of immune effector process	1	0.89088	0.31	0.008	Biological	GO:0002(	negative regulation of immune effector process	0.89088	0.9335	0	0	1	120
GO:0042752 regulation of circadian rhythm	1	0.89088	0.31	0.008	Biological	GO:0042	regulation of circadian rhythm	0.89088	0.9335	0	0	1	120
GO:0099568 cytoplasmic region	3	0.89185	0.89	0.01	Cellular C	GO:0099!	cytoplasmic region	0.89185	0.96877	0	0	3	286
GO:0006511 ubiquitin-dependent protein catabolic process	9	0.89206	2.786	0.013	Biological	GO:0006!	ubiquitin-dependent protein catabolic process	0.89206	0.93454	0	0	9	696
GO:0019724 B cell mediated immunity	2	0.89237	0.619	0.01	Biological	GO:0019	B cell mediated immunity	0.89237	0.93467	0	0	2	206
GO:0006304 DNA modification	1	0.89288	0.31	0.008	Biological	GO:0006	DNA modification	0.89288	0.93482	0	0	1	121
GO:0044237 cellular metabolic process	180	0.89289	55.728	0.017	Biological	GO:0044	cellular metabolic process	0.89289	0.93482	0	0	180	10444
GO:0016604 nuclear body	11	0.89329	3.264	0.013	Cellular C	GO:0016(	nuclear body	0.89329	0.96877	0	0	11	837
GO:0048515 spermatid differentiation	2	0.89395	0.619	0.01	Biological	GO:0048!	spermatid differentiation	0.89395	0.93554	0	0	2	207
GO:0050777 negative regulation of immune response	2	0.89395	0.619	0.01	Biological	GO:0050	negative regulation of immune response	0.89395	0.93554	0	0	2	207
GO:0043227 membrane-bounded organelle	230	0.89453	68.249	0.017	Cellular C	GO:0043	membrane-bounded organelle	0.89453	0.96877	0	0	230	13306
GO:0002224 toll-like receptor signaling pathway	1	0.89485	0.31	0.008	Biological	GO:0002	toll-like receptor signaling pathway	0.89485	0.93563	0	0	1	122
GO:0022904 respiratory electron transport chain	1	0.89485	0.31	0.008	Biological	GO:0022!	respiratory electron transport chain	0.89485	0.93563	0	0	1	122
GO:0071887 leukocyte apoptotic process	1	0.89485	0.31	0.008	Biological	GO:0071	leukocyte apoptotic process	0.89485	0.93563	0	0	1	122
GO:0043547 positive regulation of GTPase activity	3	0.89498	0.929	0.011	Biological	GO:0043!	positive regulation of GTPase activity	0.89498	0.93563	0	0	3	285
GO:0110053 regulation of actin filament organization	3	0.89498	0.929	0.011	Biological	GO:0110(	regulation of actin filament organization	0.89498	0.93563	0	0	3	285
GO:0051053 negative regulation of DNA metabolic process	1	0.89678	0.31	0.008	Biological	GO:0051(	negative regulation of DNA metabolic process	0.89678	0.93712	0	0	1	123
GO:0051224 negative regulation of protein transport	1	0.89678	0.31	0.008	Biological	GO:0051	negative regulation of protein transport	0.89678	0.93712	0	0	1	123
GO:0022853 active ion transmembrane transporter activity	3	0.89719	0.877	0.011	Molecular	GO:0022	active ion transmembrane transporter activity	0.89719	0.92828	0	0	3	282
GO:0007162 negative regulation of cell adhesion	3	0.89764	0.929	0.01	Biological	GO:0007	negative regulation of cell adhesion	0.89764	0.93783	0	0	3	287
GO:0002455 humoral immune response mediated by circulating immun	1	0.89867	0.31	0.008	Biological	GO:0002	humoral immune response mediated by circulating immunoglobulin	0.89867	0.93852	0	0	1	124
GO:0018022 peptidyl-lysine methylation	1	0.89867	0.31	0.008	Biological	GO:0018(	peptidyl-lysine methylation	0.89867	0.93852	0	0	1	124
GO:0051321 meiotic cell cycle	3	0.89895	0.929	0.01	Biological	GO:0051	meiotic cell cycle	0.89895	0.93861	0	0	3	288
GO:0005813 centrosome	8	0.89904	2.374	0.012	Cellular C	GO:0005{	centrosome	0.89904	0.97169	0	0	8	645
GO:0009057 macromolecule catabolic process	20	0.89916	6.192	0.014	Biological	GO:0009(	macromolecule catabolic process	0.89916	0.93863	0	0	20	1401
GO:0044257 cellular protein catabolic process	11	0.89966	3.406	0.013	Biological	GO:0044	cellular protein catabolic process	0.89966	0.93896	0	0	11	835
GO:0001704 formation of primary germ layer	1	0.90053	0.31	0.008	Biological	GO:0001	formation of primary germ layer	0.90053	0.93948	0	0	1	125
GO:2000278 regulation of DNA biosynthetic process	1	0.90053	0.31	0.008	Biological	GO:2000	regulation of DNA biosynthetic process	0.90053	0.93948	0	0	1	125
GO:0090079 translation regulator activity, nucleic acid binding	1	0.90063	0.292	0.008	Molecular	GO:0090(	translation regulator activity, nucleic acid binding	0.90063	0.92961	0	0	1	123
GO:0101005 deubiquitinase activity	1	0.90063	0.292	0.008	Molecular	GO:0101(	deubiquitinase activity	0.90063	0.92961	0	0	1	123
GO:0019941 modification-dependent protein catabolic process	9	0.90098	2.786	0.013	Biological	GO:0019!	modification-dependent protein catabolic process	0.90098	0.93974	0	0	9	706
GO:0045619 regulation of lymphocyte differentiation	2	0.9015	0.619	0.009	Biological	GO:0045(	regulation of lymphocyte differentiation	0.9015	0.93989	0	0	2	212
GO:1902600 proton transmembrane transport	2	0.9015	0.619	0.009	Biological	GO:1902(	proton transmembrane transport	0.9015	0.93989	0	0	2	212
GO:0001578 microtubule bundle formation	1	0.90236	0.31	0.008	Biological	GO:0001!	microtubule bundle formation	0.90236	0.9402	0	0	1	126
GO:0008286 insulin receptor signaling pathway	1	0.90236	0.31	0.008	Biological	GO:0008	insulin receptor signaling pathway	0.90236	0.9402	0	0	1	126
GO:0031623 receptor internalization	1	0.90236	0.31	0.008	Biological	GO:0031(	receptor internalization	0.90236	0.9402	0	0	1	126
GO:0008047 enzyme activator activity	1	0.90248	0.292	0.008	Molecular	GO:0008(	enzyme activator activity	0.90248	0.93041	0	0	1	124
GO:0071383 cellular response to steroid hormone stimulus	2	0.90294	0.619	0.009	Biological	GO:0071	cellular response to steroid hormone stimulus	0.90294	0.94061	0	0	2	213
GO:0140534 endoplasmic reticulum protein-containing complex	1	0.90331	0.297	0.008	Cellular C	GO:0140!	endoplasmic reticulum protein-containing complex	0.90331	0.97434	0	0	1	128
GO:0050868 negative regulation of T cell activation	1	0.90415	0.31	0.008	Biological	GO:0050{	negative regulation of T cell activation	0.90415	0.94168	0	0	1	127
GO:0003697 single-stranded DNA binding	1	0.9043	0.292	0.008	Molecular	GO:0003(	single-stranded DNA binding	0.9043	0.93118	0	0	1	125
GO:0007623 circadian rhythm	2	0.90437	0.619	0.009	Biological	GO:0007(	circadian rhythm	0.90437	0.94171	0	0	2	214
GO:0010639 negative regulation of organelle organization	4	0.90505	1.238	0.011	Biological	GO:0010(	negative regulation of organelle organization	0.90505	0.94222	0	0	4	367
GO:0005815 microtubule organizing center	11	0.90525	3.264	0.013	Cellular C	GO:0005{	microtubule organizing center	0.90525	0.97447	0	0	11	852
GO:0051051 negative regulation of transport	5	0.90545	1.548	0.011	Biological	GO:0051(	negative regulation of transport	0.90545	0.94239	0	0	5	439
GO:0050866 negative regulation of cell activation	2	0.90578	0.619	0.009	Biological	GO:0050{	negative regulation of cell activation	0.90578	0.94239	0	0	2	215
GO:0071805 potassium ion transmembrane transport	2	0.90578	0.619	0.009	Biological	GO:0071{	potassium ion transmembrane transport	0.90578	0.94239	0	0	2	215
GO:0044262 cellular carbohydrate metabolic process	3	0.90649	0.929	0.01	Biological	GO:0044	cellular carbohydrate metabolic process	0.90649	0.94293	0	0	3	294
GO:0051028 mRNA transport	1	0.90764	0.31	0.008	Biological	GO:0051(	mRNA transport	0.90764	0.94393	0	0	1	129
GO:1903046 meiotic cell cycle process	2	0.90854	0.619	0.009	Biological	GO:1903(	meiotic cell cycle process	0.90854	0.94467	0	0	2	217
GO:0065003 protein-containing complex assembly	24	0.90891	7.43	0.014	Biological	GO:0065(	protein-containing complex assembly	0.90891	0.94471	0	0	24	1663
GO:0006334 nucleosome assembly	1	0.90934	0.31	0.008	Biological	GO:0006	nucleosome assembly	0.90934	0.94471	0	0	1	130
GO:0051983 regulation of chromosome segregation	1	0.90934	0.31	0.008	Biological	GO:0051!	regulation of chromosome segregation	0.90934	0.94471	0	0	1	130
GO:0072329 monocarboxylic acid catabolic process	1	0.90934	0.31	0.008	Biological	GO:0072	monocarboxylic acid catabolic process	0.90934	0.94471	0	0	1	130
GO:0004197 cysteine-type endopeptidase activity	1	0.90956	0.292	0.008	Molecular	GO:0004	cysteine-type endopeptidase activity	0.90956	0.93548	0	0	1	128
GO:0007276 gamete generation	10	0.91002	3.096	0.013	Biological	GO:0007	gamete generation	0.91002	0.94522	0	0	10	783
GO:1990204 oxidoreductase complex	1	0.91015	0.297	0.008	Cellular C	GO:1990	oxidoreductase complex	0.91015	0.97777	0	0	1	132
GO:0043632 modification-dependent macromolecule catabolic process	9	0.91086	2.786	0.013	Biological	GO:0043(	modification-dependent macromolecule catabolic process	0.91086	0.94565	0	0	9	718
GO:0000018 regulation of DNA recombination	1	0.911	0.31	0.008	Biological	GO:0000(	regulation of DNA recombination	0.911	0.94565	0	0	1	131
GO:0051928 positive regulation of calcium ion transport	1	0.911	0.31	0.008	Biological	GO:0051!	positive regulation of calcium ion transport	0.911	0.94565	0	0	1	131
GO:0017111 nucleoside-triphosphatase activity	9	0.91207	2.632	0.013	Molecular	GO:0017	nucleoside-triphosphatase activity	0.91207	0.93695	0	0	9	708
GO:0007033 vacuole organization	2	0.91254	0.619	0.009	Biological	GO:0007(	vacuole organization	0.91254	0.94705	0	0	2	220
GO:0004519 endonuclease activity	1	0.91453	0.292	0.008	Molecular	GO:0004!	endonuclease activity	0.91453	0.93837	0	0	1	131
GO:0097193 intrinsic apoptotic signaling pathway	3	0.91465	0.929	0.01	Biological	GO:0097	intrinsic apoptotic signaling pathway	0.91465	0.94904	0	0	3	301
GO:0072594 establishment of protein localization to organelle	5	0.91524	1.548	0.011	Biological	GO:0072!	establishment of protein localization to organelle	0.91524	0.94946	0	0	5	449
GO:0006956 complement activation	1	0.91582	0.31	0.007	Biological	GO:0006!	complement activation	0.91582	0.94986	0	0	1	134
GO:0006275 regulation of DNA replication	1	0.91736	0.31	0.007	Biological	GO:0006	regulation of DNA replication	0.91736	0.95107	0	0	1	135
GO:0007498 mesoderm development	1	0.91736	0.31	0.007	Biological	GO:0007	mesoderm development	0.91736	0.95107	0	0	1	135
GO:0008168 methyltransferase activity	2	0.91843	0.585	0.009	Molecular	GO:0008	methyltransferase activity	0.91843	0.94125	0	0	2	221
GO:0051301 cell division	8	0.91865	2.477	0.012	Biological	GO:0051	cell division	0.91865	0.95199	0	0	8	661
GO:0050852 T cell receptor signaling pathway	1	0.91888	0.31	0.007	Biological	GO:0050{	T cell receptor signaling pathway	0.91888	0.95199	0	0	1	136
GO:0098754 detoxification	1	0.91888	0.31	0.007	Biological	GO:0098	detoxification	0.91888	0.95199	0	0	1	136
GO:1903311 regulation of mRNA metabolic process	3	0.91901	0.929	0.01	Biological	GO:1903	regulation of mRNA metabolic process	0.91901	0.95199	0	0	3	305
GO:1902911 protein kinase complex	1	0.9195	0.297	0.007	Cellular C	GO:1902!	protein kinase complex	0.9195	0.98549	0	0	1	138
GO:0015931 nucleobase-containing compound transport	2	0.92005	0.619	0.009	Biological	GO:0015!	nucleobase-containing compound transport	0.92005	0.95287	0	0	2	226
GO:0007098 centrosome cycle	1	0.92037	0.31	0.007	Biological	GO:0007(	centrosome cycle	0.92037	0.953	0	0	1	137
GO:0002443 leukocyte mediated immunity	5	0.92067	1.548	0.011	Biological	GO:0002	leukocyte mediated immunity	0.92067	0.95312	0	0	5	455
GO:0098791 Golgi apparatus subcompartment	4	0.92102	1.187	0.01	Cellular C	GO:0098	Golgi apparatus subcompartment	0.92102	0.98549	0	0	4	387
GO:0006839 mitochondrial transport	3	0.92112	0.929	0.01	Biological	GO:0006{	mitochondrial transport	0.92112	0.95338	0	0	3	307
GO:0043401 steroid hormone mediated signaling pathway	1	0.92183	0.31	0.007	Biological	GO:0043	steroid hormone mediated signaling pathway	0.92183	0.95353	0	0	1	138
GO:0046165 alcohol biosynthetic process	1	0.92183	0.31	0.007	Biological	GO:0046	alcohol biosynthetic process	0.92183	0.95353	0	0	1	138
GO:1903038 negative regulation of leukocyte cell-cell adhesion	1	0.92183	0.31	0.007	Biological	GO:1903(	negative regulation of leukocyte cell-cell adhesion	0.92183	0.95353	0	0	1	138
GO:0044248 cellular catabolic process	33	0.92341	10.217	0.015	Biological	GO:0044	cellular catabolic process	0.92341	0.95494	0	0	33	2239
GO:0008154 actin polymerization or depolymerization	2	0.92358	0.619	0.009	Biological	GO:0008	actin polymerization or depolymerization	0.92358	0.95494	0	0	2	229
GO:0015078 proton transmembrane transporter activity	1	0.92367	0.292	0.007	Molecular	GO:0015(	proton transmembrane transporter activity	0.92367	0.9455	0	0	1	137
GO:0051603 proteolysis involved in cellular protein catabolic process	10	0.9249	3.096	0.012	Biological	GO:0051(	proteolysis involved in cellular protein catabolic process	0.9249	0.9561	0	0	10	804
GO:0000228 nuclear chromosome	2	0.92508	0.593	0.009	Cellular C	GO:0000	nuclear chromosome	0.92508	0.98647	0	0	2	233
GO:2001234 negative regulation of apoptotic signaling pathway	2	0.92584	0.619	0.009	Biological	GO:2001	negative regulation of apoptotic signaling pathway	0.92584	0.95688	0	0	2	231
GO:0019783 ubiquitin-like protein-specific protease activity	1	0.92649	0.292	0.007	Molecular	GO:0019	ubiquitin-like protein-specific protease activity	0.92649	0.94616	0	0	1	139
GO:0052689 carboxylic ester hydrolase activity	1	0.92649	0.292	0.007	Molecular	GO:0052(	carboxylic ester hydrolase activity	0.92649	0.94616	0	0	1	139
GO:0000151 ubiquitin ligase complex	3	0.92651	0.89	0.009	Cellular C	GO:0000	ubiquitin ligase complex	0.92651	0.98647	0	0	3	316
GO:0002429 immune response-activating cell surface receptor signaling	3	0.92714	0.929	0.01	Biological	GO:0002	immune response-activating cell surface receptor signaling pathway	0.92714	0.95782	0	0	3	313
GO:0002757 immune response-activating signal transduction	3	0.92714	0.929	0.01	Biological	GO:0002	immune response-activating signal transduction	0.92714	0.95782	0	0	3	313
GO:0045727 positive regulation of translation	1	0.92742	0.31	0.007	Biological	GO:0045	positive regulation of translation	0.92742	0.95792	0	0	1	142
GO:0030667 secretory granule membrane	3	0.92747	0.89	0.009	Cellular C	GO:0030(	secretory granule membrane	0.92747	0.98647	0	0	3	317
GO:0031497 chromatin assembly	1	0.92876	0.31	0.007	Biological	GO:0031	chromatin assembly	0.92876	0.9591	0	0	1	143
GO:0006338 chromatin remodeling	3	0.92905	0.929	0.01	Biological	GO:0006	chromatin remodeling	0.92905	0.9592	0	0	3	315
GO:0051258 protein polymerization	3	0.92999	0.929	0.009	Biological	GO:0051	protein polymerization	0.92999	0.95982	0	0	3	316
GO:0016741 transferase activity, transferring one-carbon groups	2	0.93	0.585	0.009	Molecular	GO:0016	transferase activity, transferring one-carbon groups	0.93	0.94859	0	0	2	231
GO:0050778 positive regulation of immune response	7	0.93003	2.167	0.012	Biological	GO:0050	positive regulation of immune response	0.93003	0.95982	0	0	7	608
GO:0072521 purine-containing compound metabolic process	5	0.93063	1.548	0.011	Biological	GO:0072!	purine-containing compound metabolic process	0.93063	0.96014	0	0	5	467
GO:0048285 organelle fission	6	0.93085	1.858	0.011	Biological	GO:0048	organelle fission	0.93085	0.96014	0	0	6	539
GO:2001020 regulation of response to DNA damage stimulus	3	0.93091	0.929	0.009	Biological	GO:2001(	regulation of response to DNA damage stimulus	0.93091	0.96014	0	0	3	317
GO:0005085 guanyl-nucleotide exchange factor activity	2	0.93107	0.585	0.009	Molecular	GO:0005(	guanyl-nucleotide exchange factor activity	0.93107	0.94859	0	0	2	232
GO:0016072 rRNA metabolic process	2	0.93329	0.619	0.008	Biological	GO:0016(	rRNA metabolic process	0.93329	0.96239	0	0	2	238
GO:0006119 oxidative phosphorylation	1	0.93385	0.31	0.007	Biological	GO:0006	oxidative phosphorylation	0.93385	0.96257	0	0	1	147
GO:0031056 regulation of histone modification	1	0.93385	0.31	0.007	Biological	GO:0031(	regulation of histone modification	0.93385	0.96257	0	0	1	147
GO:0051052 regulation of DNA metabolic process	5	0.93444	1.548	0.011	Biological	GO:0051(	regulation of DNA metabolic process	0.93444	0.96283	0	0	5	472
GO:0002253 activation of immune response	4	0.93449	1.238	0.01	Biological	GO:0002	activation of immune response	0.93449	0.96283	0	0	4	398
GO:0043231 intracellular membrane-bounded organelle	208	0.93542	61.721	0.017	Cellular C	GO:0043	intracellular membrane-bounded organelle	0.93542	0.99295	0	0	208	12243
GO:0043271 negative regulation of ion transport	1	0.93626	0.31	0.007	Biological	GO:0043	negative regulation of ion transport	0.93626	0.96446	0	0	1	149
GO:1901575 organic substance catabolic process	31	0.93676	9.598	0.014	Biological	GO:1901	organic substance catabolic process	0.93676	0.96477	0	0	31	2151
GO:0034728 nucleosome organization	1	0.93743	0.31	0.007	Biological	GO:0034	nucleosome organization	0.93743	0.96527	0	0	1	150
GO:0006402 mRNA catabolic process	2	0.93817	0.619	0.008	Biological	GO:0006	mRNA catabolic process	0.93817	0.96583	0	0	2	243
GO:0045333 cellular respiration	2	0.93911	0.619	0.008	Biological	GO:0045	cellular respiration	0.93911	0.96659	0	0	2	244
GO:0016070 RNA metabolic process	72	0.93956	22.291	0.016	Biological	GO:0016(	RNA metabolic process	0.93956	0.96685	0	0	72	4579
GO:0005766 primary lysosome	1	0.93997	0.297	0.006	Cellular C	GO:0005	primary lysosome	0.93997	0.99384	0	0	1	154
GO:0042582 azurophil granule	1	0.93997	0.297	0.006	Cellular C	GO:0042!	azurophil granule	0.93997	0.99384	0	0	1	154
GO:0006352 DNA-templated transcription, initiation	1	0.94191	0.31	0.006	Biological	GO:0006	DNA-templated transcription, initiation	0.94191	0.96908	0	0	1	154
GO:0072522 purine-containing compound biosynthetic process	2	0.94272	0.619	0.008	Biological	GO:0072!	purine-containing compound biosynthetic process	0.94272	0.96971	0	0	2	248
GO:0006913 nucleocytoplasmic transport	3	0.94429	0.929	0.009	Biological	GO:0006!	nucleocytoplasmic transport	0.94429	0.97093	0	0	3	333
GO:0051169 nuclear transport	3	0.94429	0.929	0.009	Biological	GO:0051	nuclear transport	0.94429	0.97093	0	0	3	333
GO:0006333 chromatin assembly or disassembly	1	0.94506	0.31	0.006	Biological	GO:0006	chromatin assembly or disassembly	0.94506	0.97131	0	0	1	157
GO:0030183 B cell differentiation	1	0.94506	0.31	0.006	Biological	GO:0030	B cell differentiation	0.94506	0.97131	0	0	1	157
GO:0045182 translation regulator activity	1	0.94564	0.292	0.006	Molecular	GO:0045	translation regulator activity	0.94564	0.96168	0	0	1	155
GO:0070507 regulation of microtubule cytoskeleton organization	1	0.94607	0.31	0.006	Biological	GO:0070!	regulation of microtubule cytoskeleton organization	0.94607	0.97215	0	0	1	158
GO:0016462 pyrophosphatase activity	9	0.94684	2.632	0.012	Molecular	GO:0016	pyrophosphatase activity	0.94684	0.96168	0	0	9	762
GO:0033044 regulation of chromosome organization	2	0.94694	0.619	0.008	Biological	GO:0033(	regulation of chromosome organization	0.94694	0.97277	0	0	2	253
GO:0044839 cell cycle G2/M phase transition	1	0.94706	0.31	0.006	Biological	GO:0044	cell cycle G2/M phase transition	0.94706	0.97277	0	0	1	159
GO:0000280 nuclear division	5	0.94787	1.548	0.01	Biological	GO:0000	nuclear division	0.94787	0.97337	0	0	5	492
GO:0016241 regulation of macroautophagy	1	0.94803	0.31	0.006	Biological	GO:0016	regulation of macroautophagy	0.94803	0.97337	0	0	1	160
GO:0016817 hydrolase activity, acting on acid anhydrides	9	0.94836	2.632	0.012	Molecular	GO:0016{	hydrolase activity, acting on acid anhydrides	0.94836	0.96168	0	0	9	765
GO:0016818 hydrolase activity, acting on acid anhydrides, in phosphoru	9	0.94836	2.632	0.012	Molecular	GO:0016{	hydrolase activity, acting on acid anhydrides, in phosphorus-containi	0.94836	0.96168	0	0	9	765
GO:0098800 inner mitochondrial membrane protein complex	1	0.9491	0.297	0.006	Cellular C	GO:0098	inner mitochondrial membrane protein complex	0.9491	0.99856	0	0	1	163
GO:0004386 helicase activity	1	0.94959	0.292	0.006	Molecular	GO:0004	helicase activity	0.94959	0.96181	0	0	1	159
GO:0005730 nucleolus	12	0.94965	3.561	0.012	Cellular C	GO:0005	nucleolus	0.94965	0.99856	0	0	12	995
GO:0050657 nucleic acid transport	1	0.94993	0.31	0.006	Biological	GO:0050(	nucleic acid transport	0.94993	0.97483	0	0	1	162
GO:0050658 RNA transport	1	0.94993	0.31	0.006	Biological	GO:0050(	RNA transport	0.94993	0.97483	0	0	1	162
GO:0002768 immune response-regulating cell surface receptor signalin	3	0.95004	0.929	0.009	Biological	GO:0002	immune response-regulating cell surface receptor signaling pathway	0.95004	0.97483	0	0	3	341

GO:0019866 organelle inner membrane	6	0.95066	1.78	0.01	Cellular C	GO:00198	organelle inner membrane	0.95066	0.99856	0	0	6	577
GO:0046434 organophosphate catabolic process	1	0.95085	0.31	0.006	Biological	GO:00464	organophosphate catabolic process	0.95085	0.97508	0	0	1	163
GO:0051250 negative regulation of lymphocyte activation	1	0.95085	0.31	0.006	Biological	GO:00512	negative regulation of lymphocyte activation	0.95085	0.97508	0	0	1	163
GO:0032886 regulation of microtubule-based process	2	0.95087	0.619	0.008	Biological	GO:00328	regulation of microtubule-based process	0.95087	0.97508	0	0	2	258
GO:0002764 immune response-regulating signaling pathway	5	0.95196	1.548	0.01	Biological	GO:00027	immune response-regulating signaling pathway	0.95196	0.97599	0	0	5	499
GO:0051236 establishment of RNA localization	1	0.95264	0.31	0.006	Biological	GO:00512	establishment of RNA localization	0.95264	0.97629	0	0	1	165
GO:2000045 regulation of G1/S transition of mitotic cell cycle	1	0.95264	0.31	0.006	Biological	GO:20000	regulation of G1/S transition of mitotic cell cycle	0.95264	0.97629	0	0	1	165
GO:0019897 extrinsic component of plasma membrane	1	0.95271	0.297	0.006	Cellular C	GO:00198	extrinsic component of plasma membrane	0.95271	0.99856	0	0	1	167
GO:0005802 trans-Golgi network	2	0.95376	0.593	0.008	Cellular C	GO:00058	trans-Golgi network	0.95376	0.99856	0	0	2	265
GO:0051168 nuclear export	1	0.95437	0.31	0.006	Biological	GO:00511	nuclear export	0.95437	0.97786	0	0	1	167
GO:0006261 DNA-dependent DNA replication	1	0.95521	0.31	0.006	Biological	GO:00062	DNA-dependent DNA replication	0.95521	0.97827	0	0	1	168
GO:0034250 positive regulation of cellular amide metabolic process	1	0.95521	0.31	0.006	Biological	GO:00342	positive regulation of cellular amide metabolic process	0.95521	0.97827	0	0	1	168
GO:0044271 cellular nitrogen compound biosynthetic process	75	0.95535	23.22	0.016	Biological	GO:00442	cellular nitrogen compound biosynthetic process	0.95535	0.97827	0	0	75	4817
GO:0033043 regulation of organelle organization	15	0.95602	4.644	0.013	Biological	GO:00330	regulation of organelle organization	0.95602	0.97876	0	0	15	1198
GO:0016482 cytosolic transport	1	0.95684	0.31	0.006	Biological	GO:00164	cytosolic transport	0.95684	0.97939	0	0	1	170
GO:0090305 nucleic acid phosphodiester bond hydrolysis	2	0.95725	0.619	0.007	Biological	GO:00903	nucleic acid phosphodiester bond hydrolysis	0.95725	0.97961	0	0	2	267
GO:0042113 B cell activation	3	0.95822	0.929	0.008	Biological	GO:00421	B cell activation	0.95822	0.9802	0	0	3	354
GO:1901361 organic cyclic compound catabolic process	5	0.95828	1.548	0.01	Biological	GO:19013	organic cyclic compound catabolic process	0.95828	0.9802	0	0	5	511
GO:2001022 positive regulation of response to DNA damage stimulus	1	0.95841	0.31	0.006	Biological	GO:20010	positive regulation of response to DNA damage stimulus	0.95841	0.9802	0	0	1	172
GO:0043933 protein-containing complex subunit organization	25	0.95927	7.74	0.014	Biological	GO:00439	protein-containing complex subunit organization	0.95927	0.98088	0	0	25	1849
GO:0043433 negative regulation of DNA-binding transcription factor act	1	0.96139	0.31	0.006	Biological	GO:00434	negative regulation of DNA-binding transcription factor activity	0.96139	0.98285	0	0	1	176
GO:0051054 positive regulation of DNA metabolic process	2	0.96225	0.619	0.007	Biological	GO:00510	positive regulation of DNA metabolic process	0.96225	0.98348	0	0	2	275
GO:0009451 RNA modification	1	0.9628	0.31	0.006	Biological	GO:00094	RNA modification	0.9628	0.98348	0	0	1	178
GO:0017148 negative regulation of translation	1	0.9628	0.31	0.006	Biological	GO:00171	negative regulation of translation	0.9628	0.98348	0	0	1	178
GO:0072331 signal transduction by p53 class mediator	1	0.9628	0.31	0.006	Biological	GO:00723	signal transduction by p53 class mediator	0.9628	0.98348	0	0	1	178
GO:0006163 purine nucleotide metabolic process	4	0.96411	1.238	0.009	Biological	GO:00061	purine nucleotide metabolic process	0.96411	0.98461	0	0	4	446
GO:0044265 cellular macromolecule catabolic process	14	0.96644	4.334	0.012	Biological	GO:00442	cellular macromolecule catabolic process	0.96644	0.98671	0	0	14	1164
GO:0006325 chromatin organization	6	0.96655	1.858	0.01	Biological	GO:00063	chromatin organization	0.96655	0.98671	0	0	6	605
GO:0031461 cullin-RING ubiquitin ligase complex	1	0.96664	0.297	0.005	Cellular C	GO:00314	cullin-RING ubiquitin ligase complex	0.96664	1	0	0	1	186
GO:0007283 spermatogenesis	6	0.96731	1.858	0.01	Biological	GO:00072	spermatogenesis	0.96731	0.98728	0	0	6	607
GO:0006479 protein methylation	1	0.96794	0.31	0.005	Biological	GO:00064	protein methylation	0.96794	0.98743	0	0	1	186
GO:0008213 protein alkylation	1	0.96794	0.31	0.005	Biological	GO:00082	protein alkylation	0.96794	0.98743	0	0	1	186
GO:0090407 organophosphate biosynthetic process	6	0.96805	1.858	0.01	Biological	GO:00904	organophosphate biosynthetic process	0.96805	0.98743	0	0	6	609
GO:0032259 methylation	3	0.97055	0.929	0.008	Biological	GO:00322	methylation	0.97055	0.98949	0	0	3	379
GO:0071466 cellular response to xenobiotic stimulus	1	0.97079	0.31	0.005	Biological	GO:00714	cellular response to xenobiotic stimulus	0.97079	0.98949	0	0	1	191
GO:0031974 membrane-enclosed lumen	87	0.97081	25.816	0.015	Cellular C	GO:00319	membrane-enclosed lumen	0.97081	1	0	0	87	5667
GO:0043233 organelle lumen	87	0.97081	25.816	0.015	Cellular C	GO:00432	organelle lumen	0.97081	1	0	0	87	5667
GO:0070013 intracellular organelle lumen	87	0.97081	25.816	0.015	Cellular C	GO:00700	intracellular organelle lumen	0.97081	1	0	0	87	5667
GO:0006401 RNA catabolic process	2	0.97108	0.619	0.007	Biological	GO:00064	RNA catabolic process	0.97108	0.98949	0	0	2	292
GO:0046700 heterocycle catabolic process	4	0.9712	1.238	0.009	Biological	GO:00467	heterocycle catabolic process	0.9712	0.98949	0	0	4	463
GO:0000910 cytokinesis	1	0.97133	0.31	0.005	Biological	GO:00009	cytokinesis	0.97133	0.98949	0	0	1	192
GO:0042770 signal transduction in response to DNA damage	1	0.97133	0.31	0.005	Biological	GO:00427	signal transduction in response to DNA damage	0.97133	0.98949	0	0	1	192
GO:0002695 negative regulation of leukocyte activation	1	0.97186	0.31	0.005	Biological	GO:00026	negative regulation of leukocyte activation	0.97186	0.98949	0	0	1	193
GO:0006323 DNA packaging	1	0.97186	0.31	0.005	Biological	GO:00063	DNA packaging	0.97186	0.98949	0	0	1	193
GO:1902806 regulation of cell cycle G1/S phase transition	1	0.97186	0.31	0.005	Biological	GO:19028	regulation of cell cycle G1/S phase transition	0.97186	0.98949	0	0	1	193
GO:0044270 cellular nitrogen compound catabolic process	4	0.97337	1.238	0.009	Biological	GO:00442	cellular nitrogen compound catabolic process	0.97337	0.99064	0	0	4	469
GO:0009060 aerobic respiration	1	0.97338	0.31	0.005	Biological	GO:00090	aerobic respiration	0.97338	0.99064	0	0	1	196
GO:0048232 male gamete generation	6	0.97374	1.858	0.01	Biological	GO:00482	male gamete generation	0.97374	0.9908	0	0	6	626
GO:0071897 DNA biosynthetic process	1	0.97436	0.31	0.005	Biological	GO:00718	DNA biosynthetic process	0.97436	0.99103	0	0	1	198
GO:0140013 meiotic nuclear division	1	0.97436	0.31	0.005	Biological	GO:01400	meiotic nuclear division	0.97436	0.99103	0	0	1	198
GO:0004930 G protein-coupled receptor activity	9	0.97446	2.632	0.011	Molecular	GO:00049	G protein-coupled receptor activity	0.97446	0.98584	0	0	9	835
GO:0098687 chromosomal region	3	0.97495	0.89	0.008	Cellular C	GO:00986	chromosomal region	0.97495	1	0	0	3	395
GO:0007286 spermatid development	1	0.97529	0.31	0.005	Biological	GO:00072	spermatid development	0.97529	0.99169	0	0	1	200
GO:1903047 mitotic cell cycle process	8	0.97541	2.477	0.01	Biological	GO:19030	mitotic cell cycle process	0.97541	0.99169	0	0	8	780
GO:0006403 RNA localization	1	0.97575	0.31	0.005	Biological	GO:00064	RNA localization	0.97575	0.99184	0	0	1	201
GO:0019637 organophosphate metabolic process	11	0.97672	3.406	0.011	Biological	GO:00196	organophosphate metabolic process	0.97672	0.99262	0	0	11	999
GO:0030496 midbody	1	0.97732	0.297	0.005	Cellular C	GO:00304	midbody	0.97732	1	0	0	1	207
GO:0000377 RNA splicing, via transesterification reactions with bulged	2	0.97755	0.619	0.006	Biological	GO:00003	RNA splicing, via transesterification reactions with bulged adenosine	0.97755	0.99303	0	0	2	308
GO:0000398 mRNA splicing, via spliceosome	2	0.97755	0.619	0.006	Biological	GO:00003	mRNA splicing, via spliceosome	0.97755	0.99303	0	0	2	308
GO:0050864 regulation of B cell activation	1	0.9779	0.31	0.005	Biological	GO:00508	regulation of B cell activation	0.9779	0.99303	0	0	1	206
GO:0019439 aromatic compound catabolic process	4	0.97815	1.238	0.008	Biological	GO:00194	aromatic compound catabolic process	0.97815	0.99303	0	0	4	484
GO:0048193 Golgi vesicle transport	2	0.97826	0.619	0.006	Biological	GO:00481	Golgi vesicle transport	0.97826	0.99303	0	0	2	310
GO:0000070 mitotic sister chromatid segregation	1	0.97831	0.31	0.005	Biological	GO:00000	mitotic sister chromatid segregation	0.97831	0.99303	0	0	1	207
GO:0000375 RNA splicing, via transesterification reactions	2	0.97894	0.619	0.006	Biological	GO:00003	RNA splicing, via transesterification reactions	0.97894	0.99346	0	0	2	312
GO:0032991 protein-containing complex	88	0.97986	26.113	0.015	Cellular C	GO:00329	protein-containing complex	0.97986	1	0	0	88	5800
GO:0006282 regulation of DNA repair	1	0.98096	0.31	0.005	Biological	GO:00062	regulation of DNA repair	0.98096	0.99531	0	0	1	214
GO:0006959 humoral immune response	2	0.98234	0.619	0.006	Biological	GO:00069	humoral immune response	0.98234	0.99622	0	0	2	323
GO:0016236 macroautophagy	2	0.98262	0.619	0.006	Biological	GO:00162	macroautophagy	0.98262	0.99622	0	0	2	324
GO:0043414 macromolecule methylation	2	0.98262	0.619	0.006	Biological	GO:00434	macromolecule methylation	0.98262	0.99622	0	0	2	324
GO:0009152 purine ribonucleotide biosynthetic process	1	0.98265	0.31	0.005	Biological	GO:00091	purine ribonucleotide biosynthetic process	0.98265	0.99622	0	0	1	219
GO:0031981 nuclear lumen	66	0.98275	19.585	0.015	Cellular C	GO:00319	nuclear lumen	0.98275	1	0	0	66	4531
GO:0004518 nuclease activity	1	0.98316	0.292	0.005	Molecular	GO:00045	nuclease activity	0.98316	0.99348	0	0	1	217
GO:0080135 regulation of cellular response to stress	7	0.98392	2.167	0.009	Biological	GO:00801	regulation of cellular response to stress	0.98392	0.99717	0	0	7	744
GO:0006518 peptide metabolic process	9	0.98399	2.786	0.01	Biological	GO:00065	peptide metabolic process	0.98399	0.99717	0	0	9	893
GO:0034655 nucleobase-containing compound catabolic process	3	0.98435	0.929	0.007	Biological	GO:00346	nucleobase-containing compound catabolic process	0.98435	0.99733	0	0	3	423
GO:0006364 rRNA processing	1	0.98506	0.31	0.004	Biological	GO:00063	rRNA processing	0.98506	0.99784	0	0	1	227
GO:0000082 G1/S transition of mitotic cell cycle	1	0.98587	0.31	0.004	Biological	GO:00000	G1/S transition of mitotic cell cycle	0.98587	0.99847	0	0	1	230
GO:0045930 negative regulation of mitotic cell cycle	1	0.98613	0.31	0.004	Biological	GO:00459	negative regulation of mitotic cell cycle	0.98613	0.99853	0	0	1	231
GO:0000819 sister chromatid segregation	1	0.98639	0.31	0.004	Biological	GO:00008	sister chromatid segregation	0.98639	0.99858	0	0	1	232
GO:0009260 ribonucleotide biosynthetic process	1	0.98664	0.31	0.004	Biological	GO:00092	ribonucleotide biosynthetic process	0.98664	0.99864	0	0	1	233
GO:0032993 protein-DNA complex	1	0.98695	0.297	0.004	Cellular C	GO:00329	protein-DNA complex	0.98695	1	0	0	1	237
GO:0005840 ribosome	1	0.98765	0.297	0.004	Cellular C	GO:00058	ribosome	0.98765	1	0	0	1	240
GO:0005654 nucleoplasm	59	0.98791	17.507	0.014	Cellular C	GO:00056	nucleoplasm	0.98791	1	0	0	59	4171
GO:0006164 purine nucleotide biosynthetic process	1	0.98805	0.31	0.004	Biological	GO:00061	purine nucleotide biosynthetic process	0.98805	0.99987	0	0	1	239
GO:0046390 ribose phosphate biosynthetic process	1	0.98827	0.31	0.004	Biological	GO:00463	ribose phosphate biosynthetic process	0.98827	0.99989	0	0	1	240
GO:0140097 catalytic activity, acting on DNA	1	0.98911	0.292	0.004	Molecular	GO:01400	catalytic activity, acting on DNA	0.98911	0.99833	0	0	1	240
GO:0002449 lymphocyte mediated immunity	2	0.98912	0.619	0.006	Biological	GO:00024	lymphocyte mediated immunity	0.98912	1	0	0	2	353
GO:0055086 nucleobase-containing small molecule metabolic process	5	0.98913	1.548	0.008	Biological	GO:00550	nucleobase-containing small molecule metabolic process	0.98913	1	0	0	5	619
GO:0010608 posttranscriptional regulation of gene expression	4	0.9892	1.238	0.007	Biological	GO:00100	posttranscriptional regulation of gene expression	0.9892	1	0	0	4	536
GO:0008380 RNA splicing	3	0.98933	0.929	0.007	Biological	GO:00083	RNA splicing	0.98933	1	0	0	3	449
GO:0009117 nucleotide metabolic process	4	0.98978	1.238	0.007	Biological	GO:00091	nucleotide metabolic process	0.98978	1	0	0	4	540
GO:0032984 protein-containing complex disassembly	1	0.99027	0.31	0.004	Biological	GO:00329	protein-containing complex disassembly	0.99027	1	0	0	1	250
GO:0065004 protein-DNA complex assembly	1	0.99027	0.31	0.004	Biological	GO:00650	protein-DNA complex assembly	0.99027	1	0	0	1	250
GO:0140535 intracellular protein-containing complex	7	0.9908	2.077	0.009	Cellular C	GO:01405	intracellular protein-containing complex	0.9908	1	0	0	7	801
GO:0043604 amide biosynthetic process	8	0.99087	2.477	0.009	Biological	GO:00436	amide biosynthetic process	0.99087	1	0	0	8	869
GO:0006753 nucleoside phosphate metabolic process	4	0.99123	1.238	0.007	Biological	GO:00067	nucleoside phosphate metabolic process	0.99123	1	0	0	4	551
GO:0006996 organelle organization	56	0.99133	17.337	0.014	Biological	GO:00069	organelle organization	0.99133	1	0	0	56	3991
GO:0010564 regulation of cell cycle process	6	0.9917	1.858	0.008	Biological	GO:00105	regulation of cell cycle process	0.9917	1	0	0	6	721
GO:0022402 cell cycle process	13	0.99175	4.025	0.01	Biological	GO:00224	cell cycle process	0.99175	1	0	0	13	1245
GO:0006725 cellular aromatic compound metabolic process	87	0.99187	26.935	0.015	Biological	GO:00067	cellular aromatic compound metabolic process	0.99187	1	0	0	87	5832
GO:0044843 cell cycle G1/S phase transition	1	0.99193	0.31	0.004	Biological	GO:00448	cell cycle G1/S phase transition	0.99193	1	0	0	1	260
GO:0140513 nuclear protein-containing complex	14	0.99209	4.154	0.01	Cellular C	GO:01405	nuclear protein-containing complex	0.99209	1	0	0	14	1336
GO:0007059 chromosome segregation	2	0.99253	0.619	0.005	Biological	GO:00070	chromosome segregation	0.99253	1	0	0	2	376
GO:0005634 nucleus	118	0.99262	35.015	0.015	Cellular C	GO:00056	nucleus	0.99262	1	0	0	118	7721
GO:0090304 nucleic acid metabolic process	74	0.99296	22.91	0.015	Biological	GO:00903	nucleic acid metabolic process	0.99296	1	0	0	74	5097
GO:0034248 regulation of cellular amide metabolic process	3	0.99298	0.929	0.006	Biological	GO:00342	regulation of cellular amide metabolic process	0.99298	1	0	0	3	477
GO:0071824 protein-DNA complex subunit organization	1	0.99343	0.31	0.004	Biological	GO:00718	protein-DNA complex subunit organization	0.99343	1	0	0	1	271
GO:0005739 mitochondrion	19	0.99364	5.638	0.011	Cellular C	GO:00057	mitochondrion	0.99364	1	0	0	19	1710
GO:0005759 mitochondrial matrix	3	0.9938	0.89	0.006	Cellular C	GO:00057	mitochondrial matrix	0.9938	1	0	0	3	491
GO:0071103 DNA conformation change	1	0.99475	0.31	0.004	Biological	GO:00711	DNA conformation change	0.99475	1	0	0	1	283
GO:0006260 DNA replication	1	0.99522	0.31	0.003	Biological	GO:00062	DNA replication	0.99522	1	0	0	1	288
GO:0046034 ATP metabolic process	1	0.99548	0.31	0.003	Biological	GO:00460	ATP metabolic process	0.99548	1	0	0	1	291
GO:0005743 mitochondrial inner membrane	3	0.99554	0.89	0.006	Cellular C	GO:00057	mitochondrial inner membrane	0.99554	1	0	0	3	513
GO:1990234 transferase complex	7	0.99606	2.077	0.008	Cellular C	GO:19902	transferase complex	0.99606	1	0	0	7	871
GO:0019898 extrinsic component of membrane	1	0.99621	0.297	0.003	Cellular C	GO:00198	extrinsic component of membrane	0.99621	1	0	0	1	304
GO:0006302 double-strand break repair	1	0.99625	0.31	0.003	Biological	GO:00063	double-strand break repair	0.99625	1	0	0	1	301
GO:0009165 nucleotide biosynthetic process	1	0.99625	0.31	0.003	Biological	GO:00091	nucleotide biosynthetic process	0.99625	1	0	0	1	301
GO:0006417 regulation of translation	2	0.99627	0.619	0.005	Biological	GO:00064	regulation of translation	0.99627	1	0	0	2	418
GO:0010948 negative regulation of cell cycle process	1	0.99639	0.31	0.003	Biological	GO:00109	negative regulation of cell cycle process	0.99639	1	0	0	1	303
GO:0009150 purine ribonucleotide metabolic process	2	0.99639	0.619	0.005	Biological	GO:00091	purine ribonucleotide metabolic process	0.99639	1	0	0	2	420
GO:1901293 nucleoside phosphate biosynthetic process	1	0.99645	0.31	0.003	Biological	GO:19012	nucleoside phosphate biosynthetic process	0.99645	1	0	0	1	304
GO:0061695 transferase complex, transferring phosphorus-containing g	1	0.99654	0.297	0.003	Cellular C	GO:00616	transferase complex, transferring phosphorus-containing groups	0.99654	1	0	0	1	309
GO:0098798 mitochondrial protein-containing complex	1	0.99654	0.297	0.003	Cellular C	GO:00987	mitochondrial protein-containing complex	0.99654	1	0	0	1	309
GO:0042254 ribosome biogenesis	1	0.99683	0.31	0.003	Biological	GO:00422	ribosome biogenesis	0.99683	1	0	0	1	310
GO:1901363 heterocyclic compound binding	89	0.99701	26.023	0.015	Molecular	GO:19013	heterocyclic compound binding	0.99701	1	0	0	89	6027
GO:1901987 regulation of cell cycle phase transition	2	0.99719	0.619	0.005	Biological	GO:19019	regulation of cell cycle phase transition	0.99719	1	0	0	2	435
GO:0098813 nuclear chromosome segregation	1	0.99722	0.31	0.003	Biological	GO:00988	nuclear chromosome segregation	0.99722	1	0	0	1	317
GO:1901360 organic cyclic compound metabolic process	88	0.99738	27.245	0.014	Biological	GO:19013	organic cyclic compound metabolic process	0.99738	1	0	0	88	6073
GO:0009259 ribonucleotide metabolic process	2	0.99742	0.619	0.005	Biological	GO:00092	ribonucleotide metabolic process	0.99742	1	0	0	2	440
GO:0034641 cellular nitrogen compound metabolic process	94	0.99756	29.102	0.015	Biological	GO:00346	cellular nitrogen compound metabolic process	0.99756	1	0	0	94	6432
GO:0006259 DNA metabolic process	8	0.99759	2.477	0.008	Biological	GO:00062	DNA metabolic process	0.99759	1	0	0	8	980
GO:0006310 DNA recombination	1	0.9977	0.31	0.003	Biological	GO:00063	DNA recombination	0.9977	1	0	0	1	327
GO:0043043 peptide biosynthetic process	5	0.99773	1.548	0.007	Biological	GO:00430	peptide biosynthetic process	0.99773	1	0	0	5	735
GO:0019693 ribose phosphate metabolic process	2	0.99782	0.619	0.004	Biological	GO:00196	ribose phosphate metabolic process	0.99782	1	0	0	2	450
GO:0016071 mRNA metabolic process	5	0.99783	1.548	0.007	Biological	GO:00160	mRNA metabolic process	0.99783	1	0	0	5	738
GO:1901990 regulation of mitotic cell cycle phase transition	1	0.99798	0.31	0.003	Biological	GO:19019	regulation of mitotic cell cycle phase transition	0.99798	1	0	0	1	334
GO:0003729 mRNA binding	1	0.99803	0.292	0.003	Molecular	GO:00037	mRNA binding	0.99803	1	0	0	1	330
GO:0097159 organic cyclic compound binding	89	0.99816	26.023	0.015	Molecular	GO:00971	organic cyclic compound binding	0.99816	1	0	0	89	6101
GO:0046483 heterocycle metabolic process	82	0.9982	25.387	0.014	Biological	GO:00464	heterocycle metabolic process	0.9982	1	0	0	82	5778
GO:0006139 nucleobase-containing compound metabolic process	79	0.99833	24.458	0.014	Biological	GO:00061	nucleobase-containing compound metabolic process	0.99833	1	0	0	79	5612
GO:0006281 DNA repair	3	0.9988	0.929	0.005	Biological	GO:00062	DNA repair	0.9988	1	0	0	3	591
GO:0003676 nucleic acid binding	53	0.99896	15.497	0.013	Molecular	GO:00036	nucleic acid binding	0.99896	1	0	0	53	4047
GO:0006397 mRNA processing	2	0.99896	0.619	0.004	Biological	GO:00063	mRNA processing	0.99896	1	0	0	2	494
GO:0006974 cellular response to DNA damage stimulus	6	0.99908	1.858	0.007	Biological	GO:00069	cellular response to DNA damage stimulus	0.99908	1	0	0	6	887
GO:0034660 ncRNA metabolic process	2	0.99918	0.619	0.004	Biological	GO:00346	ncRNA metabolic process	0.99918	1	0	0	2	508
GO:0140098 catalytic activity, acting on RNA	1	0.99936	0.292	0.003	Molecular	GO:01400	catalytic activity, acting on RNA	0.99936	1	0	0	1	389
GO:1902494 catalytic complex	14	0.9994	4.154	0.009	Cellular C	GO:19024	catalytic complex	0.9994	1	0	0	14	1580
GO:0034470 ncRNA processing	1	0.99948	0.31	0.002	Biological	GO:00344	ncRNA processing	0.99948	1	0	0	1	406
GO:0051606 detection of stimulus	3	0.99952	0.929	0.005	Biological	GO:00516	detection of stimulus	0.99952	1	0	0	3	649
GO:0044770 cell cycle phase transition	2	0.99967	0.619	0.004	Biological	GO:00447	cell cycle phase transition	0.99967	1	0	0	2	560
GO:0051276 chromosome organization	7	0.99967	2.167	0.007	Biological	GO:00512	chromosome organization	0.99967	1	0	0	7	1049
GO:0044772 mitotic cell cycle phase transition	1	0.99979	0.31	0.002	Biological	GO:00447	mitotic cell cycle phase transition	0.99979	1	0	0	1	455

GO:0006412 translation	3	0.99981	0.929	0.004	Biological	GO:00064	translation	0.99981	1	0	0	3	706
GO:0022613 ribonucleoprotein complex biogenesis	1	0.99982	0.31	0.002	Biological	GO:00226	ribonucleoprotein complex biogenesis	0.99982	1	0	0	1	462
GO:0140640 catalytic activity, acting on a nucleic acid	2	0.99987	0.585	0.003	Molecular	GO:01406	catalytic activity, acting on a nucleic acid	0.99987	1	0	0	2	605
GO:0009593 detection of chemical stimulus	1	0.99987	0.31	0.002	Biological	GO:00095	detection of chemical stimulus	0.99987	1	0	0	1	481
GO:0034622 cellular protein-containing complex assembly	7	0.99991	2.167	0.006	Biological	GO:00346	cellular protein-containing complex assembly	0.99991	1	0	0	7	1137
GO:0007606 sensory perception of chemical stimulus	1	0.99991	0.31	0.002	Biological	GO:00076	sensory perception of chemical stimulus	0.99991	1	0	0	1	501
GO:0050906 detection of stimulus involved in sensory perception	1	0.99993	0.31	0.002	Biological	GO:00509	detection of stimulus involved in sensory perception	0.99993	1	0	0	1	514
GO:0006396 RNA processing	4	0.99998	1.238	0.004	Biological	GO:00063	RNA processing	0.99998	1	0	0	4	957
GO:0003723 RNA binding	12	0.99999	3.509	0.007	Molecular	GO:00037	RNA binding	0.99999	1	0	0	12	1722
GO:1990904 ribonucleoprotein complex	1	1	0.297	0.001	Cellular C	GO:19909	ribonucleoprotein complex	1	1	0	0	1	729
GO:0003674 molecular_function	342	1	100	0.019	Molecular	GO:00036	molecular_function	1	1	0	0	342	18452
GO:0005575 cellular_component	337	1	100	0.018	Cellular C	GO:00055	cellular_component	1	1	0	0	337	18696
GO:0008150 biological_process	323	1	100	0.018	Biological	GO:0008	biological_process	1	1	0	0	323	17718

Supplementary Table 6: The result of KEGG analysis

id	num	pvalue	per	ratio	class	ID	Descrptio	Pvalue	Qvalue	Up	Down	fg_num	bg_num
Pathways in cancer	22	0.00016	15.172	0.04	Human D	ko05200	Pathways	0.00016	0.03992	0	0	22	546
AGE-RAGE signaling pathway in diabetic complications	8	0.00042	5.517	0.077	Human D	ko04933	AGE-RAG	0.00042	0.05164	0	0	8	104
Vascular smooth muscle contraction	9	0.00066	6.207	0.065	Organism	ko04270	Vascular	0.00066	0.05399	0	0	9	139
Glycosphingolipid biosynthesis - lacto and neolacto series	4	0.00128	2.759	0.143	Metabolis	ko00601	Glycosph	0.00128	0.07803	0	0	4	28
Protein digestion and absorption	7	0.00224	4.828	0.067	Organism	ko04974	Protein di	0.00224	0.08419	0	0	7	105
Choline metabolism in cancer	7	0.00236	4.828	0.066	Human D	ko05231	Choline m	0.00236	0.08419	0	0	7	106
Glycosphingolipid biosynthesis - globo and isoglobo series	3	0.00242	2.069	0.188	Metabolis	ko00603	Glycosph	0.00242	0.08419	0	0	3	16
GnRH signaling pathway	6	0.00598	4.138	0.063	Organism	ko04912	GnRH sig	0.00598	0.16892	0	0	6	95
MAPK signaling pathway	12	0.00623	8.276	0.04	Environm	ko04010	MAPK sig	0.00623	0.16892	0	0	12	303
Inflammatory mediator regulation of TRP channels	6	0.0084	4.138	0.059	Organism	ko04750	Inflammat	0.0084	0.17091	0	0	6	102
Renin secretion	5	0.0085	3.448	0.068	Organism	ko04924	Renin sec	0.0085	0.17091	0	0	5	73
Renal cell carcinoma	5	0.00951	3.448	0.067	Human D	ko05211	Renal cel	0.00951	0.17091	0	0	5	75
Focal adhesion	9	0.01061	6.207	0.043	Cellular P	ko04510	Focal adh	0.01061	0.17091	0	0	9	210
Gastric acid secretion	5	0.01116	3.448	0.064	Organism	ko04971	Gastric ac	0.01116	0.17091	0	0	5	78
Ether lipid metabolism	4	0.01149	2.759	0.078	Metabolis	ko00565	Ether lipid	0.01149	0.17091	0	0	4	51
Apelin signaling pathway	7	0.01154	4.828	0.049	Environm	ko04371	Apelin sig	0.01154	0.17091	0	0	7	142
Proteoglycans in cancer	9	0.01191	6.207	0.042	Human D	ko05205	Proteogly	0.01191	0.17091	0	0	9	214
Glutamatergic synapse	6	0.01582	4.138	0.051	Organism	ko04724	Glutamate	0.01582	0.20608	0	0	6	117
Phototransduction - fly	3	0.01605	2.069	0.097	Organism	ko04745	Phototran	0.01605	0.20608	0	0	3	31
ErbB signaling pathway	5	0.01807	3.448	0.057	Environm	ko04012	ErbB sign	0.01807	0.22043	0	0	5	88
Oxytocin signaling pathway	7	0.02034	4.828	0.044	Organism	ko04921	Oxytocin	0.02034	0.22836	0	0	7	159
ECM-receptor interaction	5	0.02059	3.448	0.055	Environm	ko04512	ECM-rece	0.02059	0.22836	0	0	5	91
Mucin type O-glycan biosynthesis	3	0.02395	2.069	0.083	Metabolis	ko00512	Mucin typ	0.02395	0.25403	0	0	3	36
Hepatitis B	7	0.02506	4.828	0.042	Human D	ko05161	Hepatitis	0.02506	0.25478	0	0	7	166
Relaxin signaling pathway	6	0.02777	4.138	0.045	Organism	ko04926	Relaxin si	0.02777	0.25835	0	0	6	133
Long-term potentiation	4	0.02993	2.759	0.059	Organism	ko04720	Long-term	0.02993	0.25835	0	0	4	68
Glycerophospholipid metabolism	5	0.03062	3.448	0.05	Metabolis	ko00564	Glyceroph	0.03062	0.25835	0	0	5	101
Melanogenesis	5	0.03062	3.448	0.05	Organism	ko04916	Melanoge	0.03062	0.25835	0	0	5	101
Ras signaling pathway	9	0.03076	6.207	0.036	Environm	ko04014	Ras signa	0.03076	0.25835	0	0	9	252
cGMP-PKG signaling pathway	7	0.03216	4.828	0.04	Environm	ko04022	cGMP-PK	0.03216	0.25835	0	0	7	175
Amphetamine addiction	4	0.03282	2.759	0.057	Human D	ko05031	Amphetar	0.03282	0.25835	0	0	4	70
Nitrogen metabolism	2	0.03418	1.379	0.118	Metabolis	ko00910	Nitrogen r	0.03418	0.26061	0	0	2	17
Non-small cell lung cancer	4	0.04076	2.759	0.053	Human D	ko05223	Non-smal	0.04076	0.30065	0	0	4	75
Parathyroid hormone synthesis, secretion and action	5	0.04189	3.448	0.045	Organism	ko04928	Parathyro	0.04189	0.30065	0	0	5	110
Leishmaniasis	6	0.04336	4.138	0.041	Human D	ko05140	Leishman	0.04336	0.30225	0	0	6	148
cAMP signaling pathway	8	0.04619	5.517	0.035	Environm	ko04024	cAMP sig	0.04619	0.30709	0	0	8	230
NOD-like receptor signaling pathway	7	0.04679	4.828	0.037	Organism	ko04621	NOD-like	0.04679	0.30709	0	0	7	190
Arrhythmogenic right ventricular cardiomyopathy	4	0.04782	2.759	0.051	Human D	ko05412	Arrhythm	0.04782	0.30709	0	0	4	79
Cell adhesion molecules	6	0.04957	4.138	0.039	Environm	ko04514	Cell adhe	0.04957	0.31011	0	0	6	153
Calcium signaling pathway	10	0.05176	6.897	0.031	Environm	ko04020	Calcium s	0.05176	0.31571	0	0	10	321
Pancreatic cancer	4	0.05354	2.759	0.049	Human D	ko05212	Pancreati	0.05354	0.31864	0	0	4	82
HIF-1 signaling pathway	5	0.05702	3.448	0.042	Environm	ko04066	HIF-1 sigr	0.05702	0.32913	0	0	5	120
Sphingolipid metabolism	3	0.058	2.069	0.059	Metabolis	ko00600	Sphingoli	0.058	0.32913	0	0	3	51
EGFR tyrosine kinase inhibitor resistance	4	0.05962	2.759	0.047	Human D	ko01521	EGFR tyr	0.05962	0.3306	0	0	4	85
Cysteine and methionine metabolism	3	0.06366	2.069	0.057	Metabolis	ko00270	Cysteine	0.06366	0.34519	0	0	3	53
Hypertrophic cardiomyopathy	4	0.06826	2.759	0.045	Human D	ko05410	Hypertrop	0.06826	0.36208	0	0	4	89
Thyroid hormone signaling pathway	5	0.07301	3.448	0.039	Organism	ko04919	Thyroid h	0.07301	0.37307	0	0	5	129
alpha-Linolenic acid metabolism	2	0.07388	1.379	0.077	Metabolis	ko00592	alpha-Lin	0.07388	0.37307	0	0	2	26
Platelet activation	5	0.07492	3.448	0.038	Organism	ko04611	Platelet a	0.07492	0.37307	0	0	5	130
Cytokine-cytokine receptor interaction	9	0.07988	6.207	0.03	Environm	ko04060	Cytokine-	0.07988	0.38196	0	0	9	303
Morphine addiction	4	0.07993	2.759	0.043	Human D	ko05032	Morphine	0.07993	0.38196	0	0	4	94
MicroRNAs in cancer	6	0.0814	4.138	0.034	Human D	ko05206	MicroRNA	0.0814	0.38196	0	0	6	174
TGF-beta signaling pathway	4	0.08737	2.759	0.041	Environm	ko04350	TGF-beta	0.08737	0.39514	0	0	4	97
Long-term depression	3	0.08869	2.069	0.049	Organism	ko04730	Long-term	0.08869	0.39514	0	0	3	61
Dopaminergic synapse	5	0.08907	3.448	0.036	Organism	ko04728	Dopamine	0.08907	0.39514	0	0	5	137
Glycerolipid metabolism	3	0.09899	2.069	0.047	Metabolis	ko00561	Glycerolip	0.09899	0.43133	0	0	3	64
Circadian entrainment	4	0.10323	2.759	0.039	Organism	ko04713	Circadian	0.10323	0.43887	0	0	4	103
Arachidonic acid metabolism	3	0.10612	2.069	0.045	Metabolis	ko00590	Arachidor	0.10612	0.43887	0	0	3	66
GnRH secretion	3	0.10612	2.069	0.045	Organism	ko04929	GnRH sec	0.10612	0.43887	0	0	3	66
Spinocerebellar ataxia	5	0.11151	3.448	0.034	Human D	ko05017	Spinocere	0.11151	0.45347	0	0	5	147
Inflammatory bowel disease	3	0.12477	2.069	0.042	Human D	ko05321	Inflammat	0.12477	0.49908	0	0	3	71
Th17 cell differentiation	4	0.13541	2.759	0.035	Organism	ko04659	Th17 cell	0.13541	0.52444	0	0	4	114
Toxoplasmosis	4	0.13541	2.759	0.035	Human D	ko05145	Toxoplasr	0.13541	0.52444	0	0	4	114
TNF signaling pathway	4	0.13852	2.759	0.035	Environm	ko04668	TNF signa	0.13852	0.52734	0	0	4	115
Thyroid hormone synthesis	3	0.14048	2.069	0.04	Organism	ko04918	Thyroid h	0.14048	0.52734	0	0	3	75
Glioma	3	0.15682	2.069	0.038	Human D	ko05214	Glioma	0.15682	0.57168	0	0	3	79
Bladder cancer	2	0.15816	1.379	0.049	Human D	ko05219	Bladder c	0.15816	0.57168	0	0	2	41
Aldosterone-regulated sodium reabsorption	2	0.16428	1.379	0.048	Organism	ko04960	Aldostero	0.16428	0.57168	0	0	2	42
Growth hormone synthesis, secretion and action	4	0.16439	2.759	0.033	Organism	ko04935	Growth h	0.16439	0.57168	0	0	4	123
Hepatocellular carcinoma	5	0.16924	3.448	0.03	Human D	ko05225	Hepatoce	0.16924	0.57168	0	0	5	169
Ferroptosis	2	0.17044	1.379	0.047	Cellular P	ko04216	Ferroptos	0.17044	0.57168	0	0	2	43
Fat digestion and absorption	2	0.17044	1.379	0.047	Organism	ko04975	Fat digest	0.17044	0.57168	0	0	2	43
Sphingolipid signaling pathway	4	0.17112	2.759	0.032	Environm	ko04071	Sphingoli	0.17112	0.57168	0	0	4	125
Coronavirus disease - COVID-19	8	0.17338	5.517	0.026	Human D	ko05171	Coronavir	0.17338	0.57168	0	0	8	312
Neurotrophin signaling pathway	4	0.18139	2.759	0.031	Organism	ko04722	Neurotrop	0.18139	0.58232	0	0	4	128
Wnt signaling pathway	5	0.18377	3.448	0.029	Environm	ko04310	Wnt signa	0.18377	0.58232	0	0	5	174
Fc gamma R-mediated phagocytosis	5	0.18377	3.448	0.029	Organism	ko04666	Fc gamma	0.18377	0.58232	0	0	5	174
Osteoclast differentiation	4	0.18834	2.759	0.031	Organism	ko04380	Osteoclas	0.18834	0.58918	0	0	4	130
Amoebiasis	5	0.1927	3.448	0.028	Human D	ko05146	Amoebias	0.1927	0.59516	0	0	5	177
Other types of O-glycan biosynthesis	2	0.19545	1.379	0.043	Metabolis	ko00514	Other typ	0.19545	0.59612	0	0	2	47
Purine metabolism	4	0.20251	2.759	0.03	Metabolis	ko00230	Purine me	0.20251	0.61004	0	0	4	134
Phospholipase D signaling pathway	6	0.20591	4.138	0.026	Environm	ko04072	Phosphol	0.20591	0.61182	0	0	6	229
Cocaine addiction	2	0.20812	1.379	0.041	Human D	ko05030	Cocaine a	0.20812	0.61182	0	0	2	49
Gap junction	3	0.21793	2.069	0.032	Cellular P	ko04540	Gap junct	0.21793	0.62321	0	0	3	93
Colorectal cancer	3	0.21793	2.069	0.032	Human D	ko05210	Colorecta	0.21793	0.62321	0	0	3	93
Human cytomegalovirus infection	6	0.21965	4.138	0.026	Human D	ko05163	Human cy	0.21965	0.62321	0	0	6	234
Apoptosis	4	0.22433	2.759	0.029	Cellular P	ko04210	Apoptosis	0.22433	0.62916	0	0	4	140
Cholesterol metabolism	2	0.22728	1.379	0.038	Organism	ko04979	Cholester	0.22728	0.63016	0	0	2	52
Axon guidance	5	0.22985	3.448	0.026	Organism	ko04360	Axon guid	0.22985	0.63016	0	0	5	189
N-Glycan biosynthesis	2	0.2337	1.379	0.038	Metabolis	ko00510	N-Glycan	0.2337	0.63193	0	0	2	53
PI3K-Akt signaling pathway	10	0.23787	6.897	0.023	Environm	ko04151	PI3K-Akt	0.23787	0.63193	0	0	10	443
Endocrine and other factor-regulated calcium reabsorption	2	0.24012	1.379	0.037	Organism	ko04961	Endocrine	0.24012	0.63193	0	0	2	54
Th1 and Th2 cell differentiation	3	0.24086	2.069	0.031	Organism	ko04658	Th1 and T	0.24086	0.63193	0	0	3	98
Salivary secretion	3	0.24549	2.069	0.03	Organism	ko04970	Salivary s	0.24549	0.63372	0	0	3	99
Signaling pathways regulating pluripotency of stem cells	4	0.24673	2.759	0.027	Cellular P	ko04550	Signaling	0.24673	0.63372	0	0	4	146
Aldosterone synthesis and secretion	3	0.25014	2.069	0.03	Organism	ko04925	Aldostero	0.25014	0.63578	0	0	3	100
Phosphatidylinositol signaling system	3	0.25481	2.069	0.03	Environm	ko04070	Phosphat	0.25481	0.64095	0	0	3	101
Kaposi sarcoma-associated herpesvirus infection	5	0.26229	3.448	0.025	Human D	ko05167	Kaposi sa	0.26229	0.64879	0	0	5	199
Viral protein interaction with cytokine and cytokine receptor	3	0.26417	2.069	0.029	Environm	ko04061	Viral prote	0.26417	0.64879	0	0	3	103
Legionellosis	2	0.2659	1.379	0.034	Human D	ko05134	Legionello	0.2659	0.64879	0	0	2	58
Glycosphingolipid biosynthesis - ganglio series	1	0.27007	0.69	0.056	Metabolis	ko00604	Glycosph	0.27007	0.65245	0	0	1	18
Pancreatic secretion	3	0.27358	2.069	0.029	Organism	ko04972	Pancreati	0.27358	0.65444	0	0	3	105
Taurine and hypotaurine metabolism	1	0.28274	0.69	0.053	Metabolis	ko00430	Taurine a	0.28274	0.66126	0	0	1	19
African trypanosomiasis	3	0.28775	2.069	0.028	Human D	ko05143	African try	0.28775	0.66126	0	0	3	108
VEGF signaling pathway	2	0.29168	1.379	0.032	Environm	ko04370	VEGF sig	0.29168	0.66126	0	0	2	62
Neuroactive ligand-receptor interaction	8	0.29565	5.517	0.022	Environm	ko04080	Neuroacti	0.29565	0.66126	0	0	8	364
C-type lectin receptor signaling pathway	3	0.29723	2.069	0.027	Organism	ko04625	C-type lec	0.29723	0.66126	0	0	3	110
Chagas disease	3	0.29723	2.069	0.027	Human D	ko05142	Chagas d	0.29723	0.66126	0	0	3	110
Notch signaling pathway	2	0.29811	1.379	0.032	Environm	ko04330	Notch sig	0.29811	0.66126	0	0	2	63
Regulation of lipolysis in adipocytes	2	0.29811	1.379	0.032	Organism	ko04923	Regulatio	0.29811	0.66126	0	0	2	63
Shigellosis	6	0.30149	4.138	0.023	Human D	ko05131	Shigellosi	0.30149	0.66273	0	0	6	262
Basal cell carcinoma	2	0.30453	1.379	0.031	Human D	ko05217	Basal cell	0.30453	0.66345	0	0	2	64
Steroid hormone biosynthesis	2	0.31095	1.379	0.031	Metabolis	ko00140	Steroid ho	0.31095	0.66542	0	0	2	65

Salmonella infection	6	0.31368	4.138	0.023	Human D	ko05132	Salmonell	0.31368	0.66542	0	0	6	266
mTOR signaling pathway	4	0.31635	2.759	0.024	Environm	ko04150	mTOR sig	0.31635	0.66542	0	0	4	164
Dilated cardiomyopathy	4	0.31635	2.759	0.024	Human D	ko05414	Dilated ca	0.31635	0.66542	0	0	4	164
Serotonergic synapse	3	0.32101	2.069	0.026	Organism	ko04726	Serotoner	0.32101	0.66946	0	0	3	115
Rheumatoid arthritis	4	0.32816	2.759	0.024	Human D	ko05323	Rheumato	0.32816	0.67358	0	0	4	167
Cholinergic synapse	3	0.33054	2.069	0.026	Organism	ko04725	Cholinerg	0.33054	0.67358	0	0	3	117
Mannose type O-glycan biosynthesis	1	0.33127	0.69	0.043	Metabolis	ko00515	Mannose	0.33127	0.67358	0	0	1	23
Human immunodeficiency virus 1 infection	5	0.34361	3.448	0.022	Human D	ko05170	Human im	0.34361	0.68915	0	0	5	223
RIG-I-like receptor signaling pathway	2	0.34912	1.379	0.028	Organism	ko04622	RIG-I-like	0.34912	0.68915	0	0	2	71
Leukocyte transendothelial migration	3	0.34957	2.069	0.025	Organism	ko04670	Leukocyte	0.34957	0.68915	0	0	3	121
Hematopoietic cell lineage	4	0.35187	2.759	0.023	Organism	ko04640	Hematopo	0.35187	0.68915	0	0	4	173
Vitamin digestion and absorption	1	0.3543	0.69	0.04	Organism	ko04977	Vitamin di	0.3543	0.68915	0	0	1	25
Epstein-Barr virus infection	6	0.35689	4.138	0.021	Human D	ko05169	Epstein-B	0.35689	0.68915	0	0	6	280
Mitophagy - animal	2	0.3617	1.379	0.027	Cellular P	ko04137	Mitophagy	0.3617	0.68915	0	0	2	73
Pathways of neurodegeneration - multiple diseases	10	0.3636	6.897	0.02	Human D	ko05022	Pathways	0.3636	0.68915	0	0	10	499
Rap1 signaling pathway	5	0.36434	3.448	0.022	Environm	ko04015	Rap1 sign	0.36434	0.68915	0	0	5	229
Influenza A	4	0.36769	2.759	0.023	Human D	ko05164	Influenza	0.36769	0.69012	0	0	4	177
NF-kappa B signaling pathway	4	0.37164	2.759	0.022	Environm	ko04064	NF-kappa	0.37164	0.69222	0	0	4	178
Biosynthesis of unsaturated fatty acids	1	0.37654	0.69	0.037	Metabolis	ko01040	Biosynthe	0.37654	0.69602	0	0	1	27
Biosynthesis of amino acids	2	0.38658	1.379	0.026	Metabolis	ko01230	Biosynthe	0.38658	0.70014	0	0	2	77
Folate biosynthesis	1	0.38737	0.69	0.036	Metabolis	ko00790	Folate bio	0.38737	0.70014	0	0	1	28
Maturity onset diabetes of the young	1	0.38737	0.69	0.036	Human D	ko04950	Maturity o	0.38737	0.70014	0	0	1	28
PPAR signaling pathway	2	0.39273	1.379	0.026	Organism	ko03320	PPAR sig	0.39273	0.70461	0	0	2	78
Hippo signaling pathway - multiple species	1	0.39802	0.69	0.034	Environm	ko04392	Hippo sig	0.39802	0.7082	0	0	1	29
Linoleic acid metabolism	1	0.40848	0.69	0.033	Metabolis	ko00591	Linoleic a	0.40848	0.7082	0	0	1	30
Antifolate resistance	1	0.40848	0.69	0.033	Human D	ko01523	Antifolate	0.40848	0.7082	0	0	1	30
Phototransduction	1	0.40848	0.69	0.033	Organism	ko04744	Phototran	0.40848	0.7082	0	0	1	30
Pertussis	2	0.41103	1.379	0.025	Human D	ko05133	Pertussis	0.41103	0.7082	0	0	2	81
Metabolic pathways	29	0.41215	20	0.018	Metabolis	ko01100	Metabolic	0.41215	0.7082	0	0	29	1591
Glyoxylate and dicarboxylate metabolism	1	0.42887	0.69	0.031	Metabolis	ko00630	Glyoxylate	0.42887	0.72669	0	0	1	32
Apoptosis - multiple species	1	0.42887	0.69	0.031	Cellular P	ko04215	Apoptosis	0.42887	0.72669	0	0	1	32
Estrogen signaling pathway	3	0.44319	2.069	0.021	Organism	ko04915	Estrogen	0.44319	0.74578	0	0	3	141
Propanoate metabolism	1	0.44855	0.69	0.029	Metabolis	ko00640	Propanoa	0.44855	0.74964	0	0	1	34
Insulin secretion	2	0.46422	1.379	0.022	Organism	ko04911	Insulin se	0.46422	0.75861	0	0	2	90
Tuberculosis	5	0.46741	3.448	0.019	Human D	ko05152	Tuberculo	0.46741	0.75861	0	0	5	259
GABAergic synapse	2	0.46995	1.379	0.022	Organism	ko04727	GABAerg	0.46995	0.75861	0	0	2	91
Chemical carcinogenesis - receptor activation	4	0.47289	2.759	0.02	Human D	ko05207	Chemical	0.47289	0.75861	0	0	4	204
Measles	3	0.47926	2.069	0.02	Human D	ko05162	Measles	0.47926	0.75861	0	0	3	149
Small cell lung cancer	2	0.48695	1.379	0.021	Human D	ko05222	Small cell	0.48695	0.75861	0	0	2	94
Gastric cancer	3	0.49251	2.069	0.02	Human D	ko05226	Gastric ca	0.49251	0.75861	0	0	3	152
Complement and coagulation cascades	2	0.49254	1.379	0.021	Organism	ko04610	Complem	0.49254	0.75861	0	0	2	95
Viral carcinogenesis	4	0.4955	2.759	0.019	Human D	ko05203	Viral carc	0.4955	0.75861	0	0	4	210
Autophagy - animal	3	0.49689	2.069	0.02	Cellular P	ko04140	Autophag	0.49689	0.75861	0	0	3	153
B cell receptor signaling pathway	3	0.49689	2.069	0.02	Organism	ko04662	B cell rec	0.49689	0.75861	0	0	3	153
IL-17 signaling pathway	2	0.49809	1.379	0.021	Organism	ko04657	IL-17 sign	0.49809	0.75861	0	0	2	96
Retrograde endocannabinoid signaling	3	0.50126	2.069	0.019	Organism	ko04723	Retrograd	0.50126	0.75861	0	0	3	154
PD-L1 expression and PD-1 checkpoint pathway in cancer	2	0.5036	1.379	0.021	Human D	ko05235	PD-L1 ex	0.5036	0.75861	0	0	2	97
Alanine, aspartate and glutamate metabolism	1	0.50366	0.69	0.025	Metabolis	ko00250	Alanine, a	0.50366	0.75861	0	0	1	40
Toll and Imd signaling pathway	1	0.50366	0.69	0.025	Organism	ko04624	Toll and Ir	0.50366	0.75861	0	0	1	40
Nicotine addiction	1	0.5123	0.69	0.024	Human D	ko05033	Nicotine a	0.5123	0.76688	0	0	1	41
Various types of N-glycan biosynthesis	1	0.52079	0.69	0.024	Metabolis	ko00513	Various ty	0.52079	0.77308	0	0	1	42
Hippo signaling pathway	3	0.52278	2.069	0.019	Environm	ko04390	Hippo sig	0.52278	0.77308	0	0	3	159
Prostate cancer	2	0.53058	1.379	0.02	Human D	ko05215	Prostate c	0.53058	0.77365	0	0	2	102
Diabetic cardiomyopathy	4	0.53225	2.759	0.018	Human D	ko05415	Diabetic c	0.53225	0.77365	0	0	4	220
Hepatitis C	3	0.53545	2.069	0.019	Human D	ko05160	Hepatitis	0.53545	0.77365	0	0	3	162
Lipid and atherosclerosis	4	0.53585	2.759	0.018	Human D	ko05417	Lipid and	0.53585	0.77365	0	0	4	221
Tryptophan metabolism	1	0.54538	0.69	0.022	Metabolis	ko00380	Tryptopha	0.54538	0.78038	0	0	1	45
Human papillomavirus infection	6	0.54924	4.138	0.017	Human D	ko05165	Human pa	0.54924	0.78038	0	0	6	343
Toll-like receptor signaling pathway	2	0.55143	1.379	0.019	Organism	ko04620	Toll-like re	0.55143	0.78038	0	0	2	106
ABC transporters	1	0.5533	0.69	0.022	Environm	ko02010	ABC trans	0.5533	0.78038	0	0	1	46
Apoptosis - fly	1	0.56873	0.69	0.021	Cellular P	ko04214	Apoptosis	0.56873	0.7899	0	0	1	48
Protein processing in endoplasmic reticulum	3	0.5723	2.069	0.018	Genetic Ir	ko04141	Protein pr	0.5723	0.7899	0	0	3	171
Alzheimer disease	7	0.57331	4.828	0.017	Human D	ko05010	Alzheimer	0.57331	0.7899	0	0	7	411
Human T-cell leukemia virus 1 infection	4	0.57456	2.759	0.017	Human D	ko05166	Human T-	0.57456	0.7899	0	0	4	232
Pyruvate metabolism	1	0.57624	0.69	0.02	Metabolis	ko00620	Pyruvate	0.57624	0.7899	0	0	1	49
Carbohydrate digestion and absorption	1	0.58362	0.69	0.02	Organism	ko04973	Carbohyd	0.58362	0.79555	0	0	1	50
Chemical carcinogenesis - reactive oxygen species	4	0.58819	2.759	0.017	Human D	ko05208	Chemical	0.58819	0.79654	0	0	4	236
Vibrio cholerae infection	1	0.59088	0.69	0.02	Human D	ko05110	Vibrio cho	0.59088	0.79654	0	0	1	51
Tight junction	3	0.60352	2.069	0.017	Cellular P	ko04530	Tight junc	0.60352	0.80912	0	0	3	179
Ovarian steroidogenesis	1	0.61866	0.69	0.018	Organism	ko04913	Ovarian s	0.61866	0.8204	0	0	1	55
Malaria	1	0.61866	0.69	0.018	Human D	ko05144	Malaria	0.61866	0.8204	0	0	1	55
Hedgehog signaling pathway	1	0.63184	0.69	0.018	Environm	ko04340	Hedgehog	0.63184	0.83335	0	0	1	57
Fatty acid metabolism	1	0.64457	0.69	0.017	Metabolis	ko01212	Fatty acid	0.64457	0.84557	0	0	1	59
Endocytosis	4	0.67326	2.759	0.015	Cellular P	ko04144	Endocyto	0.67326	0.8708	0	0	4	263
Hippo signaling pathway - fly	1	0.67451	0.69	0.016	Environm	ko04391	Hippo sig	0.67451	0.8708	0	0	1	64
Cytosolic DNA-sensing pathway	1	0.67451	0.69	0.016	Organism	ko04623	Cytosolic	0.67451	0.8708	0	0	1	64
Cortisol synthesis and secretion	1	0.68578	0.69	0.015	Organism	ko04927	Cortisol s	0.68578	0.87448	0	0	1	66
Lysine degradation	1	0.69126	0.69	0.015	Metabolis	ko00310	Lysine de	0.69126	0.87448	0	0	1	67
FoxO signaling pathway	2	0.69457	1.379	0.014	Environm	ko04068	FoxO sign	0.69457	0.87448	0	0	2	138
Longevity regulating pathway - multiple species	1	0.69665	0.69	0.015	Organism	ko04213	Longevity	0.69665	0.87448	0	0	1	68
Neutrophil extracellular trap formation	4	0.70154	2.759	0.015	Organism	ko04613	Neutrophi	0.70154	0.87448	0	0	4	273
Chemical carcinogenesis - DNA adducts	1	0.70195	0.69	0.014	Human D	ko05204	Chemical	0.70195	0.87448	0	0	1	69
Glycolysis / Gluconeogenesis	1	0.70715	0.69	0.014	Metabolis	ko00010	Glycolysis	0.70715	0.87448	0	0	1	70
Acute myeloid leukemia	1	0.70715	0.69	0.014	Human D	ko05221	Acute my	0.70715	0.87448	0	0	1	70
Epithelial cell signaling in Helicobacter pylori infection	1	0.71227	0.69	0.014	Human D	ko05120	Epithelial	0.71227	0.87448	0	0	1	71
Fluid shear stress and atherosclerosis	2	0.7132	1.379	0.014	Human D	ko05418	Fluid shea	0.7132	0.87448	0	0	2	143
Oocyte meiosis	2	0.71681	1.379	0.014	Cellular P	ko04114	Oocyte m	0.71681	0.87451	0	0	2	144
p53 signaling pathway	1	0.72223	0.69	0.014	Cellular P	ko04115	p53 signa	0.72223	0.87604	0	0	1	73
Adherens junction	1	0.72709	0.69	0.014	Cellular P	ko04520	Adherens	0.72709	0.87604	0	0	1	74
Adrenergic signaling in cardiomyocytes	2	0.73088	1.379	0.014	Organism	ko04261	Adrenergi	0.73088	0.87604	0	0	2	148
Antigen processing and presentation	1	0.74115	0.69	0.013	Organism	ko04612	Antigen p	0.74115	0.87604	0	0	1	77
Prolactin signaling pathway	1	0.74115	0.69	0.013	Organism	ko04917	Prolactin	0.74115	0.87604	0	0	1	77
Synaptic vesicle cycle	1	0.74567	0.69	0.013	Organism	ko04721	Synaptic	0.74567	0.87604	0	0	1	78
Chronic myeloid leukemia	1	0.74567	0.69	0.013	Human D	ko05220	Chronic m	0.74567	0.87604	0	0	1	78
Breast cancer	2	0.74763	1.379	0.013	Human D	ko05224	Breast ca	0.74763	0.87604	0	0	2	153
Prion disease	4	0.75038	2.759	0.014	Human D	ko05020	Prion dise	0.75038	0.87604	0	0	4	292
Phagosome	3	0.76608	2.069	0.013	Cellular P	ko04145	Phagoson	0.76608	0.8859	0	0	3	230
Regulation of actin cytoskeleton	3	0.76608	2.069	0.013	Cellular P	ko04810	Regulatio	0.76608	0.8859	0	0	3	230
Cushing syndrome	2	0.77258	1.379	0.012	Human D	ko04934	Cushing s	0.77258	0.88814	0	0	2	161
Cellular senescence	2	0.77847	1.379	0.012	Cellular P	ko04218	Cellular s	0.77847	0.88814	0	0	2	163
Cardiac muscle contraction	1	0.77915	0.69	0.012	Organism	ko04260	Cardiac m	0.77915	0.88814	0	0	1	86
MAPK signaling pathway - fly	1	0.78302	0.69	0.011	Environm	ko04013	MAPK sig	0.78302	0.88814	0	0	1	87
Herpes simplex virus 1 infection	8	0.78634	5.517	0.014	Human D	ko05168	Herpes si	0.78634	0.88814	0	0	8	574
Necroptosis	2	0.78987	1.379	0.012	Cellular P	ko04217	Necroptos	0.78987	0.88814	0	0	2	167
JAK-STAT signaling pathway	2	0.80074	1.379	0.012	Environm	ko04630	JAK-STAT	0.80074	0.89215	0	0	2	171
Non-alcoholic fatty liver disease	2	0.80074	1.379	0.012	Human D	ko04932	Non-alcoh	0.80074	0.89215	0	0	2	171
Endocrine resistance	1	0.83057	0.69	0.01	Human D	ko01522	Endocrine	0.83057	0.92118	0	0	1	101
Glucagon signaling pathway	1	0.85031	0.69	0.009	Organism	ko04922	Glucagon	0.85031	0.93321	0	0	1	108
Natural killer cell mediated cytotoxicity	2	0.85212	1.379	0.01	Organism	ko04650	Natural ki	0.85212	0.93321	0	0	2	193
T cell receptor signaling pathway	1	0.85805	0.69	0.009	Organism	ko04660	T cell rece	0.85805	0.93321	0	0	1	111
Alcoholism	2	0.85811	1.379	0.01	Human D	ko05034	Alcoholism	0.85811	0.93321	0	0	2	196
Nucleocytoplasmic transport	1	0.86054	0.69	0.009	Genetic Ir	ko03013	Nucleocyt	0.86054	0.93321	0	0	1	112
Insulin resistance	1	0.8654	0.69	0.009	Human D	ko04931	Insulin res	0.8654	0.93432	0	0	1	114
Chemokine signaling pathway	2	0.86944	1.379	0.01	Organism	ko04062	Chemokin	0.86944	0.93455	0	0	2	202
Parkinson disease	3	0.87797	2.069	0.01	Human D	ko05012	Parkinson	0.87797	0.93655	0	0	3	286

Carbon metabolism	1	0.87897	0.69	0.008	Metabolis	ko01200	Carbon m	0.87897	0.93655	0	0	1	120
Primary immunodeficiency	1	0.88924	0.69	0.008	Human D	ko05340	Primary ir	0.88924	0.94337	0	0	1	125
Systemic lupus erythematosus	2	0.89573	1.379	0.009	Human D	ko05322	Systemic	0.89573	0.94524	0	0	2	218
Cell cycle	1	0.90043	0.69	0.008	Cellular P	ko04110	Cell cycle	0.90043	0.94524	0	0	1	131
Viral myocarditis	1	0.9039	0.69	0.008	Human D	ko05416	Viral myo	0.9039	0.94524	0	0	1	133
Amyotrophic lateral sclerosis	4	0.9065	2.759	0.01	Human D	ko05014	Amyotrop	0.9065	0.94524	0	0	4	385
Fc epsilon RI signaling pathway	1	0.91514	0.69	0.007	Organism	ko04664	Fc epsilor	0.91514	0.94737	0	0	1	140
Lysosome	1	0.9181	0.69	0.007	Cellular P	ko04142	Lysosome	0.9181	0.94737	0	0	1	142
Alcoholic liver disease	1	0.92372	0.69	0.007	Human D	ko04936	Alcoholic	0.92372	0.94737	0	0	1	146
Huntington disease	3	0.92408	2.069	0.009	Human D	ko05016	Huntingto	0.92408	0.94737	0	0	3	324
Ubiquitin mediated proteolysis	1	0.93021	0.69	0.007	Genetic Ir	ko04120	Ubiquitin	0.93021	0.94967	0	0	1	151
Staphylococcus aureus infection	1	0.95368	0.69	0.006	Human D	ko05150	Staphyloc	0.95368	0.9664	0	0	1	174
Transcriptional misregulation in cancer	2	0.95657	1.379	0.007	Human D	ko05202	Transcrip	0.95657	0.9664	0	0	2	278
Pathogenic Escherichia coli infection	2	0.95848	1.379	0.007	Human D	ko05130	Pathogen	0.95848	0.9664	0	0	2	281
Yersinia infection	1	0.97854	0.69	0.005	Human D	ko05135	Yersinia i	0.97854	0.98257	0	0	1	217
Thermogenesis	1	0.98726	0.69	0.004	Organism	ko04714	Thermoge	0.98726	0.98726	0	0	1	246

Supplementary Table 7: Overlap genes in DEGREE, EPC, MCC and MNC

DEGREE	EPC	MCC	MNC	Overlap gene
JUN	JUN	TIMP1	JUN	JUN
STAT1	STAT1	JUN	SOX9	STAT1
SOX9	TIMP1	TGFB2	STAT1	SOX9
COL1A2	SOX9	MMP1	COL1A2	COL1A2
APOE	COL1A2	COL1A2	APOE	APOE
TIMP1	TIMP3	TIMP3	TIMP1	TIMP1
TIMP3	APOE	CSF1	TIMP3	TIMP3
AR	CSF1	TGFA	CSF1	PRKCA
PRKCA	TGFB2	STAT1	PRKCA	CSF1
CSF1	MMP1	SOX9	TGFB2	ACTA2
ACTA2	EGR1	APOE	MMP1	TGFB2
TGFB2	ACTA2	ACTA2	GRIN2B	TAGLN
TAGLN	PRKCA	GRIN2B	TAGLN	MMP1
MMP1	TGFA	TAGLN	DDX58	FBLN2
FLNC	FBLN2	MMP10	ACTA2	TGFA
GRIN2B	AR	FBLN2	FBLN2	EGR1
ERBB4	KITLG	COL14A1	COL12A1	
FBLN2	TAGLN	COL12A1	EGR1	
DDX58	ERBB4	PRKCA	TGFA	
KITLG	EPAS1	EGR1	KITLG	
TGFA				
EGR1				
MAPT				

*multiple nodes may be included within the same ranking

Supplementary Table 8: Demographic characteristics of platinum resistant cases in TCGA OC cohort

Characteristics		Number	Proportion (%)
All cases	/	90	100
Age	＜60 years old	43	47.78
	⩾ 60 years old	47	52.22
Stage	IIC	1	1.11
	IIIB	1	1.11
	IIIC	75	83.33
	IV	13	14.44
Grade	G2	8	8.89
	G3	81	90.00
	NA	1	1.11
Race	White	82	91.11
	non-White	8	8.89

Supplementary Table 9

ID	Age	Race	TUMOR_	GRADE
TCGA.04.1338	<60	White	IIIC	G3
TCGA.04.1349	<60	White	IV	G3
TCGA.04.1362	⩾60	White	IIC	G3
TCGA.04.1364	<60	White	IIIC	G3
TCGA.04.1525	⩾60	White	IIIC	G3
TCGA.04.1638	⩾60	Non-white	IV	G3
TCGA.09.0366	⩾60	White	IIIC	G3
TCGA.09.0369	⩾60	White	IIIC	G3
TCGA.10.0926	<60	White	IIIC	G3
TCGA.10.0931	⩾60	White	IIIC	G3
TCGA.10.0934	⩾60	White	IIIC	G3
TCGA.10.0937	⩾60	White	IIIC	G3
TCGA.10.0938	<60	White	IIIC	G3
TCGA.13.0717	⩾60	White	IIIC	G3
TCGA.13.0720	⩾60	White	IIIC	G3
TCGA.13.0723	<60	White	IIIC	G3
TCGA.13.0724	<60	White	IV	G3
TCGA.13.0725	⩾60	White	IIIC	G3
TCGA.13.0727	<60	White	IIIC	G3
TCGA.13.0755	<60	White	IV	G3
TCGA.13.0757	<60	White	IIIC	G3
TCGA.13.0764	<60	White	IV	G3
TCGA.13.0791	⩾60	White	IIIC	G3
TCGA.13.0795	<60	White	IIIC	G3
TCGA.13.0803	⩾60	White	IIIC	G3
TCGA.13.0804	⩾60	White	IIIC	G3
TCGA.13.0805	⩾60	White	IIIC	G3
TCGA.13.0893	⩾60	Non-white	IIIC	G3
TCGA.13.0904	<60	White	IIIC	G3
TCGA.13.0911	⩾60	White	IV	G3
TCGA.13.0920	<60	White	IIIC	G3
TCGA.13.1477	⩾60	White	IV	G2
TCGA.13.1483	⩾60	White	IIIC	G3
TCGA.13.1485	⩾60	White	IV	G2
TCGA.13.1500	<60	White	IIIC	G3
TCGA.23.1027	⩾60	White	IIIC	G3
TCGA.23.1028	⩾60	White	IIIC	G3
TCGA.23.1117	⩾60	White	IIIC	G3
TCGA.23.2079	⩾60	White	IIIC	G3
TCGA.24.0970	<60	White	IIIC	G3
TCGA.24.0980	⩾60	White	IIIC	G3
TCGA.24.0982	<60	White	IIIC	G3
TCGA.24.1431	<60	White	IIIC	G3
TCGA.24.1434	⩾60	White	IIIC	G3
TCGA.24.1464	<60	White	IIIC	G3
TCGA.24.1548	⩾60	White	IIIC	G3
TCGA.24.1552	<60	White	IIIC	G3
TCGA.24.1557	⩾60	White	IIIC	G3
TCGA.24.1558	<60	White	IIIC	G3
TCGA.24.1560	⩾60	White	IIIC	G3
TCGA.24.1562	<60	White	IIIC	G3
TCGA.24.1564	<60	White	IIIC	G3
TCGA.24.1565	<60	White	IIIC	G3
TCGA.24.1567	⩾60	White	IIIB	G3
TCGA.24.1923	⩾60	White	IIIC	G3
TCGA.24.1924	<60	White	IIIC	G3

TCGA.24.1928	＜60	White	IIIC	G3
TCGA.24.2033	＜60	White	IIIC	G3
TCGA.25.1315	⩾60	White	IIIC	G3
TCGA.25.1316	⩾60	White	IIIC	G3
TCGA.25.1318	⩾60	White	IIIC	G3
TCGA.25.1328	⩾60	White	IIIC	G3
TCGA.25.1626	＜60	White	IIIC	G3
TCGA.25.1628	＜60	White	IIIC	G3
TCGA.25.1634	＜60	White	IIIC	G3
TCGA.25.2393	＜60	White	IIIC	G3
TCGA.25.2399	＜60	White	IIIC	G3
TCGA.25.2409	＜60	White	IV	G3
TCGA.29.1696	⩾60	White	IIIC	G2
TCGA.29.1698	⩾60	Non-white	IIIC	G3
TCGA.29.1701	⩾60	White	IIIC	G3
TCGA.29.1702	＜60	White	IIIC	G3
TCGA.29.1703	⩾60	Non-white	IIIC	G2
TCGA.29.1705	⩾60	White	IIIC	G2
TCGA.29.1775	⩾60	White	IIIC	G2
TCGA.30.1714	＜60	White	IV	G3
TCGA.30.1856	⩾60	White	IIIC	G3
TCGA.30.1866	＜60	White	IV	G2
TCGA.30.1891	＜60	White	IIIC	G2
TCGA.30.1892	⩾60	White	IIIC	G3
TCGA.31.1953	⩾60	Non-white	IIIC	G3
TCGA.36.1578	＜60	Non-white	IV	G3
TCGA.36.1580	＜60	Non-white	IIIC	G3
TCGA.57.1586	＜60	White	IIIC	G3
TCGA.61.1733	＜60	White	IIIC	G3
TCGA.61.1738	⩾60	Non-white	IIIC	G3
TCGA.61.1901	＜60	White	IV	G3
TCGA.61.1906	⩾60	White	IIIC	G3
TCGA.61.2000	＜60	White	IIIC	G3
TCGA.61.2110	⩾60	White	IIIC	NA

Supplementary Table 10: Related Compounds

Compound ID	Pref Name	CAS	Pubchem ID	Chembl ID	Formula	Smiles	Target ID	Protein Name	Symbol	Uniprot
C0004	Delphinidin	NA	CID:128853	CHEMBL276780	C15H10O	Oc1cc(O)c2c(c1)[o+]c(c(c2)O)c1cc(O)c(c(c1)O)O	T80OJF	Interstitial collagenase	MMP1	P03956
C0038	Eckol	CAS:88798-74-7	CID:145937	CHEMBL471187	C18H12O	C1=C(C=C(C=C1O)OC2=C(C=C(C3=C2OC4=C(C=C	T80OJF	Interstitial collagenase	MMP1	P03956
C0041	Emodin	CAS:518-82-1	CID:3220	CHEMBL289277	C15H10O	CC1=CC2=C(C(=C1)O)C(=O)C3=C(C2=O)C=C(C=C	T80OJF	Interstitial collagenase	MMP1	P03956
C0053	Esculetin	CAS:305-01-1	CID:5281416	CHEMBL244743	C9H6O4	C1=CC(=O)OC2=CC(=C(C=C21)O)O	T80OJF	Interstitial collagenase	MMP1	P03956
C0164	Kaempferol	CAS:520-18-3	CID:5280863	CHEMBL150	C15H10O	C1=CC(=CC=C1C2=C(C(=O)C3=C(C=C(C=C3O2)O)	T80OJF	Interstitial collagenase	MMP1	P03956
C0199	Luteolin	CAS:491-70-3	CID:5280445	CHEMBL151	C15H10O	C1=CC(=C(C=C1C2=CC(=O)C3=C(C=C(C=C3O2)O)	T80OJF	Interstitial collagenase	MMP1	P03956
C0305	Phorbol Myristate	CAS:16561-29-8	CID:27924	CHEMBL279115	C36H56O	CCCCCCCCCCCCCC(=O)O[C@@H]1[C@@H](C)[C	T80OJF	Interstitial collagenase	MMP1	P03956
C0352	Quercetin	CAS:117-39-5	CID:5280343	CHEMBL50	C15H10O	C1=CC(=C(C=C1C2=C(C(=O)C3=C(C=C(C=C3O2)O	T80OJF	Interstitial collagenase	MMP1	P03956
C0476	Ursolic Acid	CAS:77-52-1	CID:64945	CHEMBL169	C30H48O	CC1CCC2(CCC3(C(=CCC4C3(CCC5C4(CCC(C5(C)	T80OJF	Interstitial collagenase	MMP1	P03956
C0485	Wogonin	CAS:632-85-9	CID:5281703	CHEMBL16171	C16H12O	COC1=C(C=C(C2=C1OC(=CC2=O)C3=CC=CC=C3)	T80OJF	Interstitial collagenase	MMP1	P03956
C0639	(+/-)Nicotine	NA	CID:942	CHEMBL440464	C10H14N	CN1CCCC1C2=CN=CC=C2	T80OJF	Interstitial collagenase	MMP1	P03956
C0739	(1E,6E)-1,7-Bis(	NA	CID:969516	CHEMBL140	C21H20O	COC1=C(C=CC(=C1)C=CC(=O)CC(=O)C=CC2=CC(	T80OJF	Interstitial collagenase	MMP1	P03956
C0805	Bornyl Acetate	CAS:76-49-3	CID:6950274	NA	C12H20O	CC(=O)O[C@H]1C[C@@H]2C([C@@]1(C)CC2)(C)C	T80OJF	Interstitial collagenase	MMP1	P03956
C0914	Apigenin	CAS:520-36-5	CID:5280443	CHEMBL28	C15H10O	C1=CC(=CC=C1C2=CC(=O)C3=C(C=C(C=C3O2)O)	T80OJF	Interstitial collagenase	MMP1	P03956
C0930	Astragaloside Iv	CAS:84687-43-4	CID:124761776	NA	C41H68O	OC[C@H]1O[C@@H](O[C@H]2C[C@@H]3[C@]4([C	T80OJF	Interstitial collagenase	MMP1	P03956
C0989	hypericin	CAS:548-04-9	CID:5281051	NA	C30H16O	CC1=CC(=C2C3=C4C5=C(C(=CC(=C5C2=O)O)O)C6	T80OJF	Interstitial collagenase	MMP1	P03956
C1110	lutein	CAS:127-40-2	CID:6433159	NA	C40H56O	CC1=C(C(CC(C1)O)(C)C)C=CC(=CC=CC(=CC=CC=	T80OJF	Interstitial collagenase	MMP1	P03956
C1171	Berberine	CAS:2086-83-1	CID:2353	CHEMBL295124	C20H18N	COc1c(OC)ccc2c1c[n+]1CCc3c(c1c2)cc1c(c3)OCO1	T80OJF	Interstitial collagenase	MMP1	P03956
C1177	Beta Carotene	NA	CID:5280489	CHEMBL1293	C40H56	CC1=C(C(CCC1)(C)C)C=CC(=CC=CC(=CC=CC=C(	T80OJF	Interstitial collagenase	MMP1	P03956
C1214	Capsaicin	CAS:404-86-4	CID:1548943	CHEMBL294199	C18H27N	CC(C)C=CCCCCC(=O)NCC1=CC(=C(C=C1)O)OC	T80OJF	Interstitial collagenase	MMP1	P03956
C1222	Celastrol	NA	CID:122724	CHEMBL301982	C29H38O	CC1=C(C(=O)C=C2C1=CC=C3C2(CCC4(C3(CCC5(	T80OJF	Interstitial collagenase	MMP1	P03956
C1250	Colchicine	CAS:64-86-8	CID:6167	CHEMBL107	C22H25N	CC(=O)NC1CCC2=CC(=C(C(=C2C3=CC=C(C(=O)C	T80OJF	Interstitial collagenase	MMP1	P03956

Table 11: IC50 of A2780DDP and Ovcar4 to cisplatin and Quercetin

Cell	Compound	IC50 (μM)	Resistance Indices
A2780	cisplatin	1.69	3.46
A2780DDP	cisplatin	5.85	
Ovcar4	cisplatin	2.29	3.03
Ovcar4DDP	cisplatin	6.94	
A2780	Quercetin	16.82	/
A2780DDP	Quercetin	44.09	
Ovcar4	Quercetin	20.24	/
Ovcar4DDP	Quercetin	45.45	
